# A molecular communication channel consisting of a single reversible chain of hydrogen bonds in a conformationally flexible oligomer

**DOI:** 10.1016/j.chempr.2021.06.022

**Published:** 2021-09-09

**Authors:** David T.J. Morris, Steven M. Wales, David P. Tilly, Elliot H.E. Farrar, Matthew N. Grayson, John W. Ward, Jonathan Clayden

**Affiliations:** 1 School of Chemistry, University of Bristol, Cantock’s Close, Bristol BS8 1TS, UK; 2 Department of Chemistry, University of Bath, Claverton Down, Bath BA2 7AY, UK

**Keywords:** dynamic foldamer, urea, conformation, hydrogen bonding, NMR, communication, information theory, oligomer, binding, stimulus responsive

## Abstract

Communication of information through the global switching of conformation in synthetic molecules has hitherto entailed the inversion of chirality. Here, we report a class of oligomer through which information may be communicated through a global reversal of polarity. Ethylene-bridged oligoureas are constitutionally symmetrical, conformationally flexible molecules organized by a single chain of hydrogen bonds running the full length of the oligomer. NMR reveals that this hydrogen-bonded chain may undergo a coherent reversal of directionality. The directional uniformity of the hydrogen-bond chain allows it to act as a channel for the spatial communication of information on a molecular scale. A binding site at the terminus of an oligomer detects local information about changes in pH or anion concentration and transmits that information—in the form of a directionality switch in the hydrogen-bond chain—to a remote polarity-sensitive fluorophore. This propagation of polarity-encoded information provides a new mechanism for molecular communication.

## Highlights

• Simple urea oligomers organize themselves with a single coherent hydrogen-bond chain
• Reversing the polarity of the hydrogen bonding sends a message through the molecule
• Conformational messages can be transmitted by changes in pH or by anion binding
• Receipt of the conformational message is indicated by a remote fluorescent response

## The bigger picture

Despite the progress made by synthetic chemistry in building the molecular structures of nature, there are still domains of molecular science where biology is immensely more sophisticated than even the most advanced artificial chemical systems. One of these is the ability to use molecular structures to encode and communicate information. Nature stores information in the hydrogen-bond polarity of the base pairs and translates it into molecular function using the polarity matching of these bonds. Here, we outline an early step toward replicating this ability in the design, synthesis, and operation of a series of synthetic molecular devices that employ a structurally simple family of oligomeric molecules that can communicate information in the form of hydrogen-bond polarity. This study marks a significant step in the design of molecular systems that manipulate information, which will form the basis of the many compartmentalization-based nanotechnologies of the future.

## Graphical abstract

**Figure:**
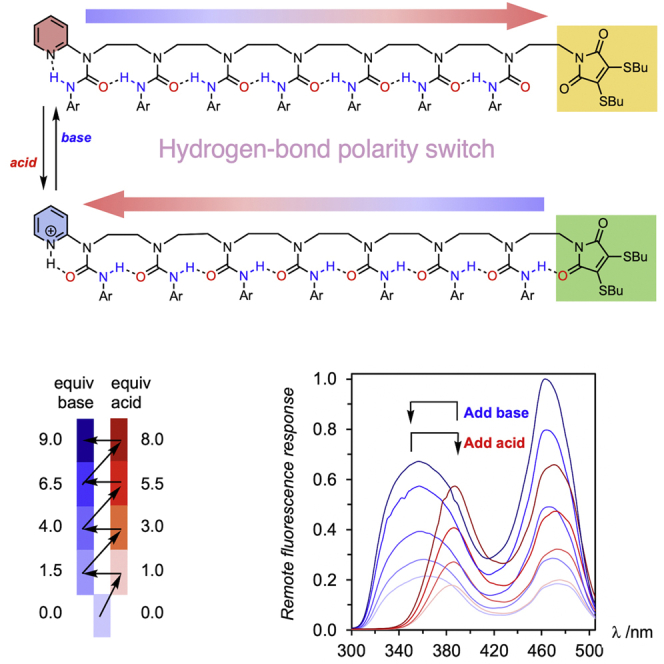

## Teaser

Information in nature is stored, communicated, and amplified by matching hydrogen-bond polarity in base pairs. Here, we show that structurally simple synthetic molecules can also use hydrogen-bond polarity to communicate information. Molecules with coherent chains of hydrogen bonds respond to changes in their environment by reversing the polarity of the chain, allowing messages to be communicated from one end of the molecule to the other. This work reveals a new mechanism for information handling in synthetic molecular systems.

## Introduction

**Figure 1:**
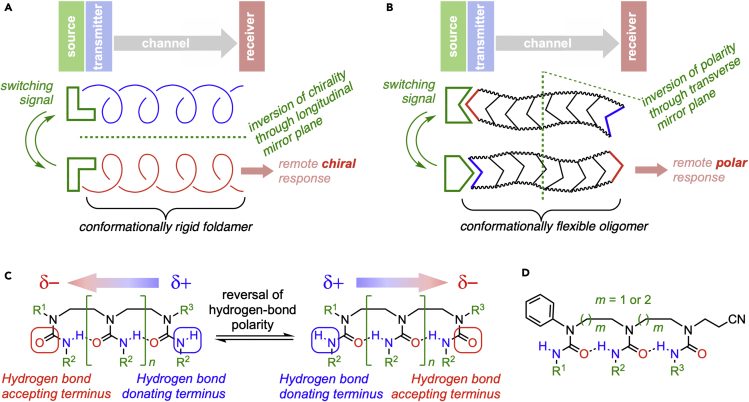
Strategies and structures for molecular communication of information (A) A previous model for the molecular communication of information using chirality switching in rigid foldamers. (B) This work: a mechanism for communicating information using a communication channel that involves polarity switching in a conformationally flexible oligomer. (C) Reversible directionality in hydrogen-bonded ethylene-bridged oligoureas. (D) Beta-sheet mimics reported by Nowick.

The general communication device theorized by Claude Shannon in the 1940s comprises an information source (input), a transmitter (which translates the information into a communicable form), a communication channel (the medium through which information is communicated), and a receiver (output).^1^ All macroscopic machinery can be reduced to informational inputs resulting in functional outputs, with the perturbation of a physical field (for example, a force exerted on a solid or the oscillation of an electromagnetic field) providing a communication channel. In artificial and biological molecular machines and devices, a chemical input is translated into molecular function. Chemical inputs and outputs are numerous (examples include the modulation of chromophores by pH, in indicators, or fluorescent responses to ligand binding), but molecular manifestations of communication channels remain sparse. Nature has mastered the manipulation of information at the molecular level, commonly using conformational changes mediated by supramolecular interactions as the communication channels,^2^^,^^3^ and synthetic information-processing mechanisms have been designed, which exploit competitive interactions and constitutional changes in chemical systems.^4^, ^5^, ^6^, ^7^ Progress toward synthetic molecular communication channels that allow spatial separation between input and output has likewise exploited conformational change, but has so far been limited to the use of a small number of rigid helical foldamers that may be induced to undergo a global conformational switch between two states: a left- and a right-handed screw sense (Figure 1A).^8^ This chiral switch has limited such devices to receivers that induce stereochemically mediated outputs (for example, control of diastereo- or enantioselective reactions,^9^^,^^10^ or modulation of fluorescence in a chiral and enantioenriched fluorophore^11^).

We now report the implementation of a more general design for a molecular communication channel that does not rely on the chirality of a conformationally rigid foldamer but instead exploits a global reversal of directionality in a chain of hydrogen bonds within a conformationally flexible oligomer (Figure 1B). We show that even in extended oligomers and in polar and hydrogen-bonding solvents, this continuous chain of hydrogen bonds is robust, meaning that an induced change in hydrogen-bond directionality at one point in the molecule leads to a global conformational response, communicating that response through the molecule regardless of the molecule’s overall conformational flexibility.

The structures in question are the ethylene-bridged oligoureas^12^^,^^13^ shown in Figure 1C. These linear *N*-linked ethylenediamine oligomers carry conformationally mobile side chains, each with a single NH and C=O group, connected through a single chain of hydrogen bonds running the full length of the oligomeric structure. Many oligomeric structures, including natural peptides and many foldamers (synthetic analogs of biopolymers with well-defined conformations), maintain a secondary structure through a network of intramolecular hydrogen bonds.^14^^,^^15^ In most cases, the directionality of these hydrogen bonds arises constitutionally from the structure of the foldamer. For example, amide-based foldamers, in common with the peptides they mimic,^16^ have a defined N terminus that acts as a hydrogen-bond donor and a defined C terminus that acts as a hydrogen-bond acceptor. However, in oligomers built from symmetrical monomers and linked through constitutionally symmetrical functional groups, hydrogen-bond directionality is potentially reversible. Such oligomers are limited to a single example from our laboratory: 2.5-helical foldamers, which are formed by connecting symmetrical (*meso*) cyclohexanediamines through carbonyl groups to form *N*,*N*′-disubstituted ureas.^17^ We previously showed that the hydrogen bonds that rigidify these foldamers select between two alternative helix screw senses with opposite hydrogen-bond directionalities according to the hydrogen-bonding capabilities of the monomer units, and that their directionality can be reversed by anionic ligands, leading to a global change in screw sense, detectable by circular dichroism.^18^ We now show, using the structurally distinct oligomeric scaffolds shown in Figure 1C, that global hydrogen-bond directionality switching is a more general phenomenon that may be decoupled from chirality, and that extended molecules containing a single, reversible chain of hydrogen bonds may be used as channels for the spatial communication of polarity-encoded information.

These *N*-linked ethylenediamine oligomers are constitutionally symmetrical and conformationally flexible structures with trisubstituted urea side chains. They are devoid of chirality, but each urea side chain is able to adopt either an *E* or a *Z* conformation. A unique structural feature allows this conformational feature to function as a communication channel: a single chain of hydrogen bonds links every urea side chain to its neighbors and runs the full length of the oligomer, meaning that the oligomer is characterized by a global, but reversible, coherent hydrogen-bond directionality. Ethylene-bridged oligoureas were explored by Nowick et al.^19^, ^20^, ^21^, ^22^ as geometric mimics of β-sheets (Figure 1D), who found that the population of non-hydrogen-bonded conformations of trimeric structures was undetectable by FTIR. Two global conformations are thus available, in which either terminal urea acts as a hydrogen-bond acceptor, but the established preference for an aryl group to lie *trans* to the carbonyl group in an *N*,*N*-disubstituted urea or amide^23^, ^24^, ^25^, ^26^ meant that in Nowick's oligomers only the hydrogen-bond directionality illustrated in Figure 1D was populated.

## Results and discussion

### Coherent and reversible hydrogen-bond polarity in ethylene-bridged oligoureas

**Figure 2:**
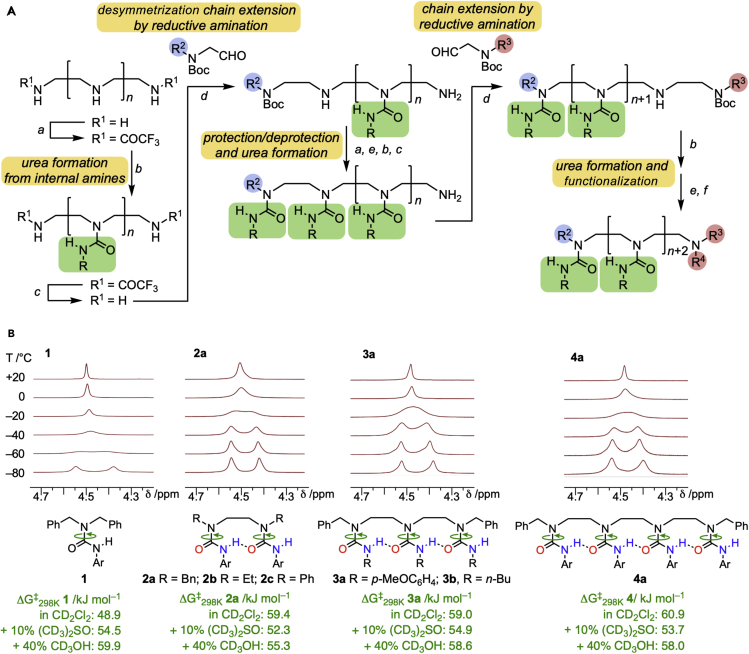
Symmetrical ethylene-bridged oligoureas: synthesis and reversible hydrogen bonding (A) An overview of the typical synthetic approach to ethylene-bridged oligoureas. Reagents and conditions: *a* EtOCOCF_3_, CH_2_Cl_2_, 0°C - rt; *b* ArNCO, CH_2_Cl_2_, rt; *c* NaOH, H_2_O, MeOH or EtOH, rt; *d* 1. RCHO, EtOH, rt; 2. NaBH_4_, EtOH, 0°C - rt; *e* CF_3_CO_2_H, CH_2_Cl_2_, rt; *f* E^+^, CH_2_Cl_2_, rt. (B) Structures, dynamic ^1^H NMR spectra, and barriers to directionality inversion of a homologous series of constitutionally symmetrical oligomers **1**–**4**. NMR spectra shown were acquired at 300 MHz in CD_2_Cl_2_ (for **1**) or 90:10 CD_2_Cl_2_ + (CD_3_)_2_SO (for **2**–**4**) at a concentration of 10 mg mL^−1^. Ar = *p*-MeOC_6_H_4_.

Ethylene-bridged oligoureas were made straightforwardly from available precursors as outlined in Figure 2A (and in detail in Schemes S1–S9). Reversible hydrogen-bond directionality in these systems was investigated initially in a homologous series of ureas **1**–**4** (Figure 2). Although **1**–**4** are constitutionally symmetrical, their NMR spectra at sub-ambient temperatures reveal a break of symmetry that results in two methylene signals of equal intensity (Figure 2B)—a result that can be explained only if the multiple urea functions are connected by an unbroken but reversible chain of hydrogen bonds.

Coalescence between the benzylic methylene signals of the monourea **1** (Figure 2B) indicated a barrier to rotation about the indicated urea C–N bond ΔG^‡^_298 K_ = 48.9 kJ mol^−1^ (from the line-shape and Eyring analysis: Table S1), and at −80 °C, the ^1^H NMR spectrum of the urea lies in the slow-exchange regime. The ^1^H NMR spectrum of diurea **2a**, consisting of two equally populated singlets at low temperature (Figure 2B), is most reasonably explained by a hydrogen bond that correlates the directionality of the two urea functions and breaks the constitutional symmetry of the molecule on the ^1^H NMR timescale. The two degenerate conformers interconvert through a barrier ΔG^‡^_298 K_ = 59.4 kJ mol^−1^ in CD_2_Cl_2_. No other conformer is evident for either **2a** or **2b**, indicating that no symmetrical structure is populated. Such a structure, lacking an intramolecular hydrogen bond, is however evident to some extent (about 40% at −80 °C) in the NMR spectrum of **2c** (Figure S9), in which the terminal phenyl groups repel the adjacent carbonyl groups.

More extended hydrogen-bond chains are evident in the ^1^H NMR spectra of triurea **3a** and tetraurea **4a**, where the terminal benzylic methylenes occupy only two identically populated environments (Figure 2B). The only simple explanation for this spectroscopic feature is an unbroken chain of hydrogen bonds that correlates the conformations of the two terminal urea functions and allows a population of only two isoenergetic (and degenerate) structures. The chemical shifts of the urea NH protons in CD_2_Cl_2_ further support this interpretation: at slow exchange (−60 °C) **3a** has one upfield (non-hydrogen-bonded) signal at 6.65 ppm and two downfield (hydrogen-bonded) NH signals at 9.28 ppm (Figure S10). At fast exchange, the central NH proton remains close to 9 ppm (it is always hydrogen-bonded), whereas the terminal NH groups coalesce to an exchange-averaged 8.94 ppm. Tetraurea **4a** behaves in a similar way (Figure S18).

The barriers to conformational interconversion in the non-hydrogen-bonding solvent CD_2_Cl_2_ **2**–**4** are all similar (Figure 2B) and are about 10 kJ mol^−1^ (at 298 K) higher than in **1**, which lacks an intramolecular hydrogen bond, with no evidence of concentration dependence (Figure S10). This suggests that the inversion of directionality in **2**–**4** occurs through a non-concerted mechanism in which only one hydrogen bond is broken at any one time. The 10 kJ mol^−1^ difference in energy between the barrier to rotation of **1** and that of **2**–**4** gives an estimate of the energetic penalty for breaking this hydrogen bond.

The situation changes in the presence of 10% *d*_6_-DMSO or 40% *d*_3_-MeOH (v/v) (Figure 2B): the barrier to rotation of **1** increases, as is typical for amide-like C–N bonds in more polar solvents,^27^ but that of **2**–**4** decreases. Presumably, hydrogen bonding to these solvents mitigates the enthalpic cost of breaking an intramolecular hydrogen bond during the directionality switch. Nonetheless, the hydrogen bond train itself proved remarkably resistant to the effect of these polar solvents and additives. Neither CD_3_OH nor (CD_3_)_2_SO (up to 50% v/v) interfered with the appearance of two coherent hydrogen-bonded conformers of **2**–**4** in CD_2_Cl_2_ (Figures S2, S3, S5, S6, S13–S15, S19, and S20). The only significant change in the NMR spectra was a shift downfield of the terminal, non-intramolecularly hydrogen-bonded proton in the presence of these hydrogen-bonding additives. Likewise, adding 2.5 equivalents of *N*,*N*′-di[3,5-bis(trifluoromethyl)phenyl]thiourea as a potent hydrogen-bond donor^28^ resulted in no changes to the intramolecularly hydrogen-bonded chain of **3a** (Figure S16).

### Control and switching of hydrogen-bond polarity

**Figure 3:**
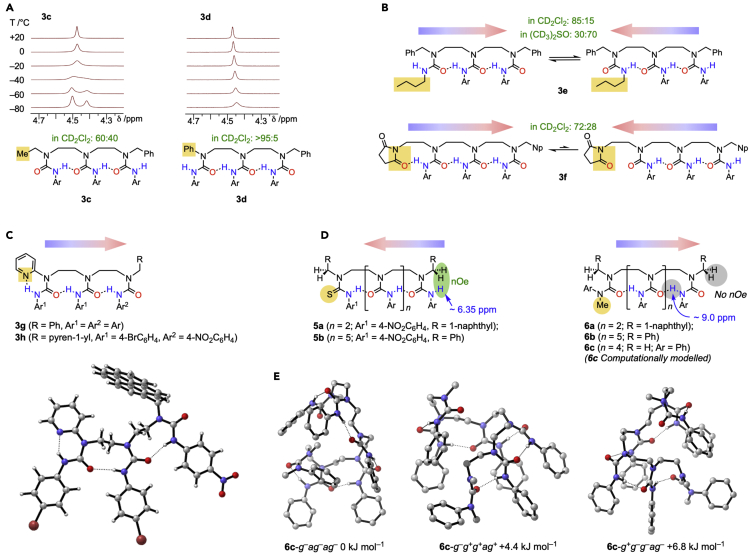
Modifications of terminal substituents induce a conformational response (A) Structures, dynamic NMR spectra, and ratios of conformers differing in hydrogen-bond directionality for constitutionally unsymmetrical triureas **3c** and **3d**. NMR spectra acquired at 300 MHz in solution in CD_2_Cl_2_ at a concentration of 10 mg mL^−1^. (B) Conformer populations in constitutionally unsymmetrical triureas **3e** and **3f**. The corresponding variable temperature NMR spectra are shown in Figures S21–S23. (C) X-ray crystal structure of oligomer **3h** showing three intramolecular hydrogen bonds (CCDC: 1993164). (D) Global conformational control from a terminal donor or acceptor in oligomers **5** and **6** containing up to seven urea units: the chemical shift of the highlighted proton is affected by structural features many bonds away. (E) Three lowest energy conformers of **6c** with corresponding backbone conformations (*g*^+^, *g*^–^ = gauche, *a*, anti, starting from NH terminus) and their relative energies (B3LYP-D3(BJ)/6–311G(d,p)/IEF-PCM(dichloromethane)//B3LYP-D3(BJ)/6-31G(d,p)).

Given the coherent hydrogen-bonded chains of **2**–**4**, changing just one terminus will break the constitutional symmetry of the oligomer, and must lead to differential populations of two conformers—both fully hydrogen-bonded, but differing in directionality.^18^ Oligomers **3c**–**3h** were made to elucidate the effect of modifying one of the terminal substituents (Figures 3A–3C). With one terminal ethyl group, the conformers are populated in a 60:40 ratio at −60°C in CD_2_Cl_2_ (**3c**), while with a terminal phenyl group (**3d**), the preference for the aryl substituent to lie *trans* to C=O leads to a single set of signals in the ^1^H NMR spectrum at all temperatures, with the Ph group at the hydrogen-bond-donating terminus.^19^, ^20^, ^21^, ^22^ Modifying the pendent nitrogen substituents also affects the conformer populations (Figure 3B). Oligomer **3e**, in which one terminal nitrogen carries an alkyl group, preferentially (85:15) adopts a conformation in which this (less acidic) NH proton does not participate in an intramolecular hydrogen bond, while in 10% (CD_3_)_2_SO (v/v) the preference inverts to 30:70, allowing the (more acidic) ArNH proton to participate in a stronger intermolecular hydrogen bond. In **3f**, a weakly hydrogen-bond-accepting succinimide provides an anchor for an adjacent hydrogen-bond-donating urea, favoring (in a ratio of 72:28) the conformer containing three hydrogen bonds.

With the much stronger hydrogen-bond-accepting pyridyl group^29^ of **3g** (Figure 3C) only a single directionality is shown by variable temperature (VT) NMR (Figure S26). Crystallographic evidence for the conformation of the hydrogen-bonded chain was obtained with the more crystalline derivative **3h** (Figure 3C), whose X-ray crystal structure shows that the oligomer adopts a structure with the pyridyl ring at the hydrogen-bond-donating terminus. As noted previously by Nowick, two nine-membered hydrogen-bonded rings link the urea functions, each of which adopts an anti conformation about the N–C–C–N bond in the oligourea backbone.^21^

The propagation of a conformational consequence from one terminus offers the prospect of using these simple urea chains as molecular communication channels. Compounds **5** and **6** (Figure 3D) were made to explore the way in which the information about structural details at one terminus can be communicated to a remote site. The *p*-nitrophenylthiourea terminus of **5a** and **5b** is a strong hydrogen-bond donor,^30^, ^31^, ^32^ enforcing a preferred orientation on the neighboring urea, which propagates through the full length of the oligomer. The induced conformation of the most distant urea in the chain is evident in the chemical shift of its NH proton (ca. 6.35 ppm in CD_2_Cl_2,_ Figures S27 and S28), characteristic of a non-intramolecularly hydrogen-bonded environment, and in the reciprocal nOe between this proton and the adjacent CH_2_ group (Figures S38–S40), even though this proton is 14 bonds away from the S atom in **5a** and 26 bonds away in **5b**. Likewise, the alkylated urea of **6a** and **6b**, which can act only as a hydrogen-bond acceptor, induces a remote conformational preference in the orientation of the terminal urea, shifting its proton downfield to ca. 9.00 ppm in CD_2_Cl_2_ (Figures S29 and S30, an intramolecularly hydrogen-bonded environment) and removing any nOe with the adjacent CH_2_ group (Figures S41 and S42). This proton is 15 bonds away from the controlling *N*-methyl group in **6a** and 27 bonds away in **6b**.

Unlike previous molecular communication mechanisms, defined three-dimensional conformational states are irrelevant to the function of these oligomers as communication channels. Nonetheless, the conformation of truncated analog **6c** was modeled computationally by DFT^33^^,^^34^ (Gaussian16 Revision A.03,^35^ B3LYP-D3(BJ)/6–311G(d,p)/IEF-PCM(dichloromethane)//B3LYP-D3(BJ)/6-31G(d,p): see computational details in the supplemental information) to gain insight into their conformational preferences. All conformations placed the tetrasubstituted urea at the hydrogen-bond-donating terminus, but a considerable degree of conformational inhomogeneity was evident (Figure 3E).^36^ Minima were found for each nine-membered hydrogen-bonded ring in which the N–C–C–N bond in the oligourea backbone adopted either of two alternative conformations: anti or gauche. The gauche conformation was found to be slightly more prevalent among the conformers of **6c**. Indeed, despite both the X-ray crystal structure of **3h** and previous observations by Nowick,^20^ modeling of a simple system with two *N*′-arylureas revealed the gauche conformation to be 2.1 kJ mol^−1^ lower in energy than the anti-conformation (Figure S202). The various permutations of these alternatives for each ring led to an ensemble of conformers, all of them nonetheless characterized by the same hydrogen-bond directionality. Figure 3E shows the three lowest energy conformers of **6c** and the corresponding pattern of gauche and anti conformations along the backbone. Similar features were evident in the modeled conformers of a truncated analog of **4a** (Figure S203). Ethylene-bridged oligoureas thus seem to form an intriguing new class of information-encoding dynamic molecular structure. They populate conformational space much more broadly than the classical definition of a foldamer would allow but are still characterized by one crucial conformational parameter—hydrogen-bond directionality—that is well controlled. This simultaneous conformational mobility and informational coherence raises the prospect of diverse applications for these oligomers, as they can adopt a range of shapes while still robustly maintaining the fidelity with which they transmit information. For example, their flexible conformation is indicative of a broader and more accommodating solubility profile than more rigid structures.

Conformational populations that are modulated by environmental signals are characteristic of a number of classes of biomolecules, such as allosteric enzymes, G-protein-coupled receptors, hemoglobin, and the opsin vision proteins.^37^, ^38^, ^39^ These molecules are characterized not only by inducible conformational change but also by their ability to translate a local chemical influence into a conformational response that has a spatially remote chemical consequence—in Shannon’s terminology, they use conformational switching on a molecular level as a channel to communicate information from a transmitter to a receiver. Attempts to mimic this relay of information using artificial structures have used screw-sense switching of rigid helices,^11^^,^^40^ but this family of switchable, conformationally dynamic oligomers opens the possibility of using polarity switching—with chemical consequences that reach beyond modulation of stereochemistry—as a form of communication channel.

**Figure 4:**
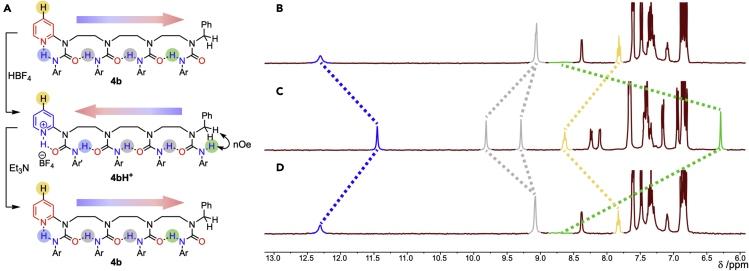
Protonation induces polarity inversion (A) NMR indicates polarity inversions mediated by reversible protonation of the terminal pyridyl group of **4b**. (B) ^1^H NMR spectrum of **4b** in CD_2_Cl_2_ at 3 mM (298 K). (C) Addition of HBF_4_ (1 equiv) at 298 K. A downfield shift of the yellow 4-pyridyl proton indicates protonation, which leads to a remote upfield shift of the remote (green) proton as it moves out of hydrogen bonding, and of the adjacent (blue) proton as it reorientates toward a weaker hydrogen-bond acceptor. (D) Recovery of the original conformation on further addition of Et_3_N (2 equiv) to the same sample (298 K). Ar, *p*-MeOC_6_H_4_; Np, 1-naphthyl.

Pyridine-terminated urea **4b** (Figure 4A) was made in order to test the responsiveness of the oligourea oligomer to a pH signal. As with its shorter homolog **3g**, VT NMR showed that the hydrogen-bond chain in **4b** adopts a preferred directionality in CD_2_Cl_2_ (Figure S31), with the diagnostic signal at 12.31 ppm (Figure 4B; the urea NH labeled in blue, hydrogen-bonded to the pyridine N) indicating that the pyridine lies at the oligomer’s hydrogen-bond-donating terminus. Protonation of the pyridyl group (shown by a downfield shift in its C4 proton, colored yellow) by the addition of tetrafluoroboric acid (Figure 4C) shifted this blue-coded proton upfield to 11.45 ppm, consistent with a conformational reorganization in which the terminal urea NH finds itself hydrogen bonded to another urea.^41^ The two central urea protons (at 9.06 and 9.07 ppm in **4b**) remain hydrogen-bonded to neighboring urea carbonyls after directionality reversal, but their signals shifted downfield to 9.29 and 9.81 ppm, consistent with the inductive effect of the pyridinium ion. At the same time, the terminal urea NH at ca. 8.8 ppm (a broad resonance more clearly resolved at 15 °C; Figure S32) moved out of hydrogen bonding and consequently shifted upfield to 6.29 ppm. This, along with an nOe to the benzylic methylene group (Figures S43 and S44), indicates a global reversal of urea directionality, with the urea oligomer acting as a communication channel, mediating the transmission of a pH signal. VT NMR of **4bH**^**+**^ (Figure S33) shows that a single conformer is populated in which the pyridinium ion acts as a hydrogen-bond donor, inverting the hydrogen-bond directionality so that it lies at the hydrogen-bond-accepting terminus of the oligomer. The addition of triethylamine (Figure 4D) returned the oligomer to its neutral conformation and restored the peaks to their original positions.

### Reversible hydrogen-bond chains as channels for the long-range communication of information

**Figure 5:**
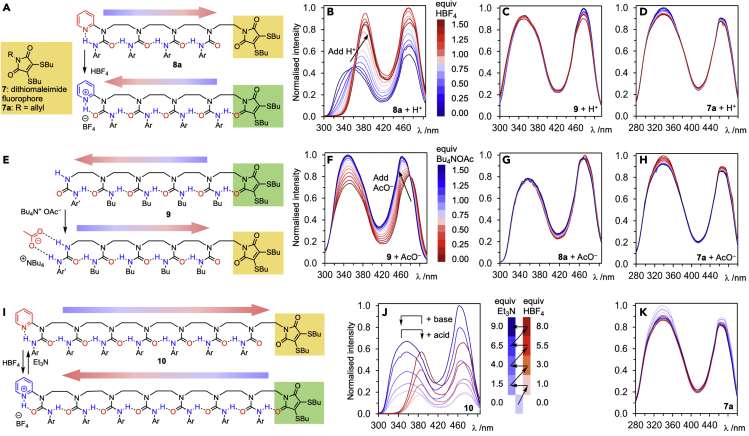
Oligoureas communicate information from a transmitter to a receiver by global switching of polarity (A) The hydrogen-bond-responsive dithiomaleimide fluorophore **7** incorporated into a switchable oligomer **8a**. (B–D) Effect of HBF_4_ (0–1.5 equiv), in CH_2_Cl_2_ solution, on the excitation spectrum (at 1 mM) of (B) **8a** (emission at 523 nm), (C) **9** (emission at 530 nm), and (D) **7a** (emission at 522 nm). (E) Anion-responsive oligomer **9** and its polarity switching on binding acetate. (F–H) Effect of Bu_4_N.OAc (0–1.5 equiv), in CH_2_Cl_2_ solution, on the excitation spectrum (at 1 mM) of (F) **9** (emission at 530 nm), (G) **8a** (emission at 523 nm), and (H) **7a** (emission at 522 nm). (I and J) (I) Reversible switching of the fluorophore-capped heptaurea **10** and its excitation spectra in CH_2_Cl_2_ at 1 mM (emission at 525 nm) on sequential addition of (J) HBF_4_ (aliquots of 1, 2, 2.5, 2.5 equiv) and Et_3_N (aliquots of 0, 1.5, 2.5, 2.5, 2.5 equiv). (K) The effect on its excitation spectrum (emission at 522 nm) of treating **7a** in CH_2_Cl_2_ at 1 mM with an equivalent sequence of aliquots. Ar = *p*-MeOC_6_H_4_; Ar′ = [3,5-(CF_3_)_2_]C_6_H_3_.

As a demonstration of the wider potential of signal transmission mediated by hydrogen-bond directionality switching, we designed a molecular device in which information about the presence of an added cation or anion is detected and transmitted through an oligourea communication channel to a remote receiver that is sensitive to local bond polarity. The dithiomaleimide fluorophore **7** (Figure 5A) is a weakly hydrogen-bond-accepting structure that is sensitive to its environment,^42^, ^43^, ^44^ and we hoped that it would respond to a local switch between a hydrogen-bond-donating and hydrogen-bond-accepting function. In contrast to typical receptors, such a device allows the functional and spatial decoupling of detector and response, raising the prospect of building modular devices for use in spatially compartmentalized systems such as on surfaces or in artificial cells.

Oligomers **8**–**10** (Figures 5A, 5E, and 5I) were designed and synthesized: all are terminated with a dithiomaleimide fluorophore, but each carries a responsive function remote from the fluorophore that can transmit chemical information as a conformational response. Oligomers **8a** and **10**, similar to **4b**, are acid-sensitive: we expect protonation of the pyridine function to lead to a change in conformation that will alter the environment of the remote fluorophore. Oligomer **9** carries an electron-deficient terminal *N*,*N*′-disubstituted urea that we expect to display binding selectivity toward anions.^34^^,^^45^ NMR experiments (Figures S34, S35, S46, and S52) indicate that these compounds adopt a preferred hydrogen-bond directionality shown in the upper parts of Figures 5A, 5E, and 5I.

The response of the fluorescence excitation spectrum (emission at 523 nm) of **8a** in CH_2_Cl_2_ was monitored as the oligomer was titrated with tetrafluoroboric acid (Figure 5B). As acid was added, the S_0_-S_2_ excitation wavelength maximum λ_max_ at 358 nm gradually red-shifted to 381 nm after 1 equiv, with a concomitant global hyperchromic shift. Minimal spectral changes were observed beyond 1 equiv of acid (λ_max_ S_0_-S_2_ with 1.5 equiv HBF_4_ = 384 nm). A similar red shift in the S_0_-S_2_ excitation band (Δλ_max_ S_0_-S_2_ = 21 nm) was seen for the longer homolog **10** on adding 1 equiv HBF_4_ (Figure S47). Under the same conditions, neither **9** nor **7a** underwent any significant changes in fluorescence excitation (Figures 5C and 5D). Additionally, a fluorescent control 8b with a tethered pyridyl group separated from the maleimide by the same number of bonds as in **8a** but without a linking chain of urea hydrogen bonds showed no λ_max_ shifts upon addition of HBF_4_ (Figure S48). This confirms that protonation of the pyridine leads to fluorescence changes only when a mechanism for intramolecular communication is available and provides evidence that the dithiomaleimide can be used as a probe of the local hydrogen-bonding environment, and hence a receiver for use with the oligourea communication channel. The response of **8a** is consistent with protonation of the pyridine function leading to a change in conformation (Figure 5A) that transmits information about its ionization state to the dithiomaleimide. As a result, the dithiomaleimide receiver is induced to act as a hydrogen-bond acceptor (indicated by the chemical shift of the adjacent NH: Figure S51), its fluorescence maximum consequently undergoing a red shift.

Next, the fluorescence excitation spectrum of **9** (emission at 530 nm) was monitored as tetrabutylammonium acetate was added (Figure 5E). The chain of hydrogen bonds in **9** involves alkyl ureas, which are weaker hydrogen-bond donors and stronger hydrogen-bond acceptors than aryl ureas (cf. Figure 3B). As tetrabutylammonium acetate was added, the S_0_-S_1_ excitation maximum at 478 nm gradually blue-shifted to 464 nm after 1 equiv (Figure 5F), consistent with the fluorophore receiver moving from a hydrogen-bonded to a non-hydrogen-bonded environment. Minimal spectral changes were observed beyond 1 equiv of acetate (λ_max_ S_0_-S_1_ with 1.5 equiv Bu_4_NOAc = 462 nm). The same ligand gave minimal response in **8a** or **7a** (Figures 5G and 5H). Oligomer **9** thus acts as an acetate-responsive device in which binding information is transmitted to a remote receiver, which responds with a modulation of its fluorescence properties.

The potential for reversible pH-controlled switching between the two polarity states was explored with both **8a** (Figure S55) and with its longer homolog **10** (Figure 5I). Starting from the neutral oligomer **10**, repeated sequential addition of acid (HBF_4_) and base (Et_3_N) successfully induced four cycles of the oscillation of the fluorophore excitation response between the blue-shifted, non-hydrogen-bonded “neutral” state (λ_max_ S_0_-S_2_ = 359 ± 4 nm) and the red-shifted, hydrogen-bonded “protonated” state (λ_max_ S_0_-S_2_ = 386 ± 2 nm) (Figure 5J). Under the same conditions, no significant changes in the excitation wavelength maxima were observed with the fluorophore (**7a**) alone (Figure 5K), showing that this oscillation in fluorescence is a consequence of the communication of information through a channel that entails inversion of the polarity of a single intramolecular chain of seven hydrogen bonds, which causes concerted rotation about seven consecutive urea C–N bonds.

### Conclusions

In summary, the structurally simple and synthetically accessible class of ethylenediamine oligomers offers a mechanism for the spatial communication of chemical information. Despite their three-dimensional conformational fluxionality, ethylene-bridged oligureas exhibit uniformity in one global conformational feature: the directionality of their single linear chain of hydrogen bonds. This hydrogen-bond chain acts as a communication channel that can carry information between spatially remote sites encoded in its polarity, and we demonstrate that it enables a signal (the presence of acid or base, or the binding of ligands) to induce a relayed response, such as a change in optical properties, that is reversible and repeatable through multiple communication cycles. A useful macromolecular analogy is a hydraulic communication channel in which a change in pressure is used to communicate information (for example, from a brake pedal to a brake pad) through a conformationally flexible tube.

The lack of a requirement for chirality in this mechanism offers several advantages over earlier examples of artificial communication devices based on helical foldamers. Their “minimal” design means that their synthesis is much simpler and their potential for functionalization is much greater than previous structures. Inputs and outputs of information are no longer stereochemically encoded, which frees potential future interfaces with biology from the complications that result from the use of single enantiomers in the enantiopure environment of a biological system. Even more importantly, the receiver of information is no longer constrained to translate an enantiomeric switch into a more general chemical response. A switch in hydrogen-bond polarity offers much greater chemical versatility in, for example, the potential to control the selective binding of ligands or metals, to alter metal coordination geometry, or to change the structure and activity at a catalytic site.

This spatial communication mechanism, which requires the use of constitutionally symmetrical dynamic structures, has hitherto remained unexploited in synthetic molecular devices. Localized hydrogen-bond directionality switching is nonetheless evident in nature, for example, during the catalytic cycle of proteases and lipases, where coupled active site histidine and aspartate residues switch from hydrogen-bond acceptors to hydrogen-bond donors during the hydrolysis mechanism. Nature is prevented from exploiting hydrogen-bond directionality switching as a general mechanism for spatial communication by the constitutional asymmetry of its α-amino acid building blocks. However, it is intriguing to note that although nature does not communicate information spatially through hydrogen-bonded chains, it does so temporally during the transcription of the genetic code, in which a series of sequential hydrogen-bond polarity matching events mediate the communication of coded information from DNA to mRNA to tRNA. Further exploration of the potential of hydrogen-bond chains as communication channels in more complex supramolecular networks and in non-homogeneous states is under way.

## Experimental procedures

### Resource availability

#### Lead contact

Further information and requests for resources should be directed to and will be fulfilled by the lead contact, Jonathan Clayden (j.clayden@bristol.ac.uk).

#### Materials availability

All materials generated in this study are available from the lead contact without restriction.

#### Data and code availability

Crystallographic data for the structure reported in this article have been deposited at the Cambridge Crystallographic Data Centre, under deposition number CCDC: 1993164. Copies of the data can be obtained free of charge from https://www.ccdc.cam.ac.uk/structures/.

## Supplemental information

Document S1. Supplemental experimental procedures, Figures S1–S203, Tables S1–S18, Schemes S1–S9, and supplemental references

Document S2. Article plus supplemental information

## Footnotes

Supplemental information can be found online at https://doi.org/10.1016/j.chempr.2021.06.022.
