# Supplementary material for: A molecular communication channel consisting of a single reversible chain of hydrogen bonds in a conformationally flexible oligomer
Source: Chem. 2021 Sep 9;7(9):2460–72. doi: 10.1016/j.chempr.2021.06.022 (PMC8442760; doi:10.1016/j.chempr.2021.06.022)
Supplement: Document S1. Supplemental experimental procedures, Figures S1–S203, Tables S1–S18, Schemes S1–S9, and supplemental references [file mmc1.pdf]

Chem, Volume *7*

## Supplemental information

# A molecular communication channel consisting of a single reversible chain of hydrogen bonds in a conformationally flexible oligomer

**David T.J. Morris, Steven M. Wales, David P. Tilly, Elliot H.E. Farrar, Matthew N. Grayson, John W. Ward, and Jonathan Clayden**

# Contents

Supplementary Methods ........ 2
- General Information ........ 2
- Experimental Procedures ........ 10
- VT NMR Experiments ........ 60
- Eyring Analyses ........ 91
- nOe Experiments ........ 101
- Fluorimetry ........ 105
  - Supplemental Experimental Procedures ........ 105
- NMR Characterisation ........ 114

Computational Details ........ 187

References ........ 205

## Supplementary Methods

## General Information

Where specified, procedures were performed using dried solvents and reagents under an atmosphere of nitrogen with glassware dried by flame-drying. Air and moisture-sensitive liquids/solutions were transferred to reaction vessels by syringe under an atmosphere of nitrogen. Anhydrous DCM, THF, MeCN, $Et_2O$ and toluene were dried using an anhydrous Engineering Grubbs-type solvent system. All other solvents and reagents were purchased from commercial suppliers and were used without further purification unless otherwise specified. Agitation was achieved using Teflon coated stirrer bars by magnetic induction. All thin layer chromatography (TLC) experiments were conducted on pre-coated plastic plates (Macherey-Nagel polygram SIL $G/UV_{254}$) and visualised using ultraviolet light (254 nm) or staining. In all instances where molecular sieves were used, 1.6 mm diameter pellets (4 Å pore size) bought from Sigma-Aldrich were activated by oven-drying. Flash chromatography was performed on an automated Biotage Isolera™ Spektra Four using gradient elution on pre-packed silica gel Biotage® SNAP Ultra, Sfär Duo or ZIP Sphere columns or manually using Fluorochem 60 silica (40-60 μm particle size). Solvent systems for TLC and flash chromatography are reported in solvent:solvent ratios. All melting point experiments were performed using a Stuart SMP10 Melting Point Apparatus upon recrystallisation from the specified solvent(s). All low-temperature VT NMR experiments were conducted using a JEOL ECS 300 spectrometer (300 MHz) or a Bruker AVANCE III HD 500 MHz NMR Spectrometer with 5 mm DCH $^{13}C$–$^{1}H$/D Cryo Probe (500 MHz). All high-temperature VT NMR experiments were conducted using a Varian VNMRS 500 MHz Direct Drive Spectrometer with Agilent OneNMR probe (500 MHz). All room temperature NMR experiments were conducted using a Bruker Nano 400 Spectrometer (400 MHz) or a Bruker AVANCE III HD 500 MHz NMR Spectrometer with 5 mm DCH $^{13}C$–$^{1}H$/D Cryo Probe (500 MHz), with chemical shifts reported (δ in ppm) relative to the specified deuterated solvent. All $^{19}F$ NMR spectra are referenced relative to an internal standard. All NMR characterisation experiments were performed at 25 °C and 1 atm unless otherwise specified. Multiplicity is reported as follows – s = singlet, d = doublet, t = triplet, q = quartet, m = multiplet. All spin-spin coupling constants (J) are reported in hertz (Hz) to the nearest 0.1 Hz. High resolution mass spectrometry experiments (HR-MS) were performed on a Bruker micrOTOF Spectrometer using electrospray ionisation, positive ion mode or a Bruker Ultraflex using MALDI with only molecular ion ($[M+H]^+$ or $[M+Na]^+$) peaks being reported. Infrared spectroscopy (IR) experiments were performed using a PerkinElmer Spectrum Two FTIR Spectrometer. All absorbances are reported in $cm^{-1}$. Fluorescence spectra were recorded using a PerkinElmer LS45 fluorimeter.

<u>Synthetic Schemes</u>

Ureas **1** and **2** were made from their corresponding disubstituted amines. Ureas **3** and **4** were synthesised in two steps from their corresponding oligoethylenediamines by reductive amination and reaction with the appropriate isocyanate.

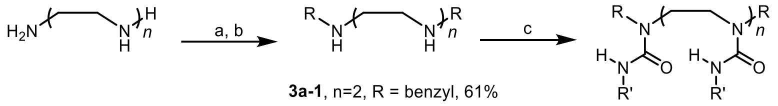


**1**, n=0, R = benzyl, R' = 4-MeO-Ph, >99% (from dibenzylamine)
**2a**, n=1, R = benzyl, R' = 4-MeO-Ph, 67% (from *N*,*N*'-dibenzylethylenediamine)
**2b**, n=1, R = ethyl, R' = 4-MeO-Ph, 82% (from *N*,*N*'-diethylethylenediamine)
**2c**, n=1, R = phenyl, R' = Ph, >99% (from *N*,*N*'-diphenylethylenediamine)
**3a**, n=2, R = benzyl, R' = 4-MeO-Ph, 54% (from **3a-1**)
**3b**, n=2, R = benzyl, R' = *n*-butyl, 54% (from **3a-1**)
**4a**, n=3, R = benzyl, R' = 4-MeO-Ph, 16% (2 steps from triethylenetetramine)

**Scheme S1.** Synthesis of symmetrical oligoureas **1-4**. Reagents and conditions: (a) benzaldehyde (2.0 equiv), EtOH, RT, 16 h; (b) $NaBH_4$ (3.0 equiv), EtOH, 0 °C to RT, 3 h; (c) R'NCO (1.5 equiv per amine), DCM, RT, 16 h.

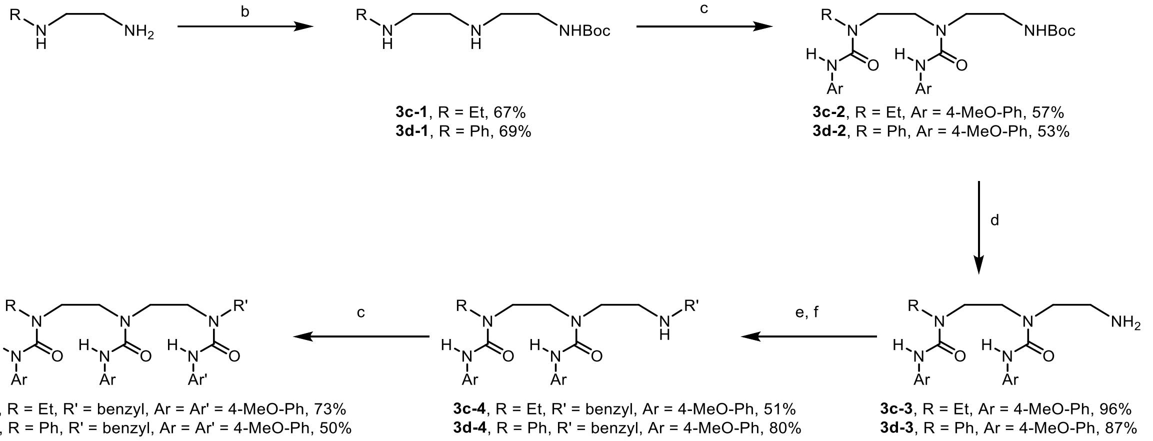


**Scheme S2.** Synthesis of mixed triureas **3c** and **3d**. Reagents and conditions: (a) Ethylenediamine (6.4 equiv), neat, 120 °C, 16 h; (b) $H_2$, Pd/C, *N*-Boc-aminoacetaldehyde, (1.1 equiv), MeOH, RT, 16 h; (c) ArNCO (1.5 equiv per amine), DCM, RT, 16 h; (d) TFA, DCM, RT, 16 h; (e) aldehyde (2.0 equiv), EtOH, RT, 16 h; (f) $NaBH_4$ (3.0 equiv) EtOH, 0 °C to RT, 3 h.

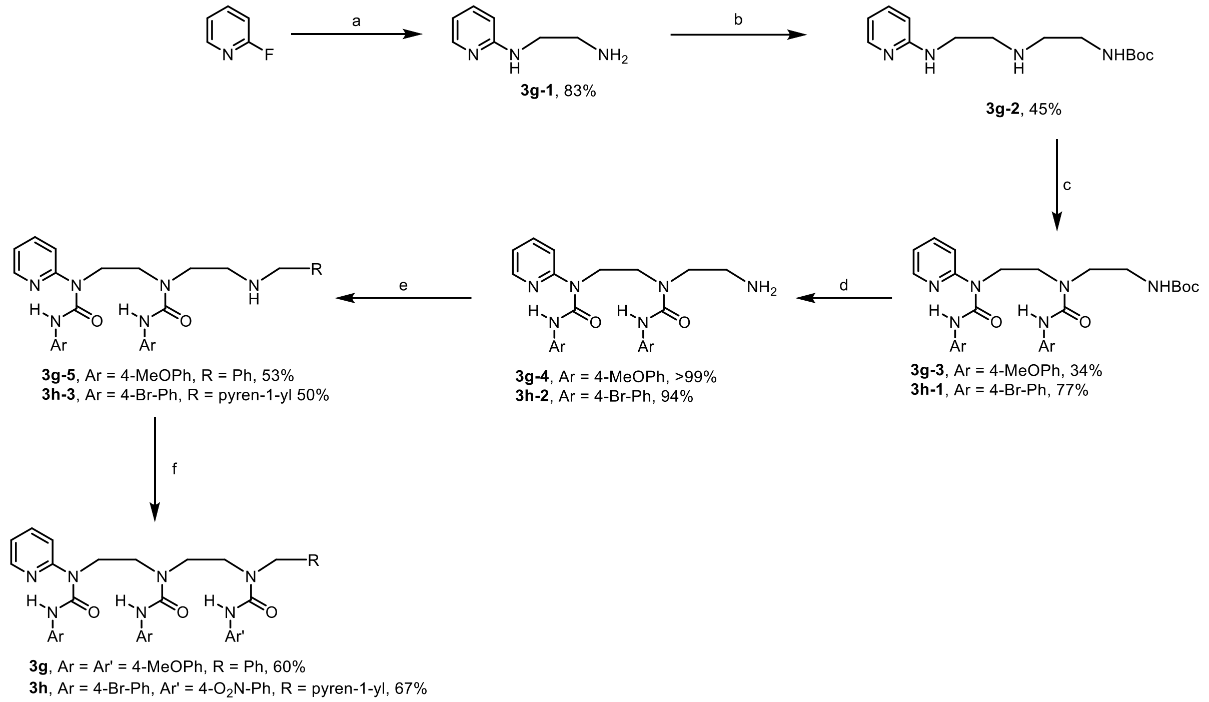


**Scheme S3.** Synthesis of triureas **3g** and **3h**. Reagents and conditions: (a) Ethylenediamine (10.0 equiv), neat, 120 °C, 18.5 h; (b) *N*-Boc-aminoacetaldehyde, (1.0 equiv), $NaB(OAc)_3H$ (1.4 equiv), DCE, RT, 20 h; (c) ArNCO (1.0 equiv per amine), DCM, RT, 16 h (for **3g-3**) or DCE, RT to 40 °C, 64 h (for **3h-1**); (d) TFA, DCM, RT, 16 h (for **3g-4**) or 4 h (for **3h-2**); (e) benzaldehyde (1.5 equiv), MeOH, RT, 16 h *then* $NaBH_4$, 0 °C to RT, 3 h (for **3g-5**) or 1-pyrenecarboxaldehyde (1.0 equiv), $NaB(OAc)_3H$, DCE, RT, 5 h (for **3h-3**); (f) Ar'NCO (1.0 equiv), DCM, RT, 16 h (for **3g**) or 4.5 h (for **3h**).

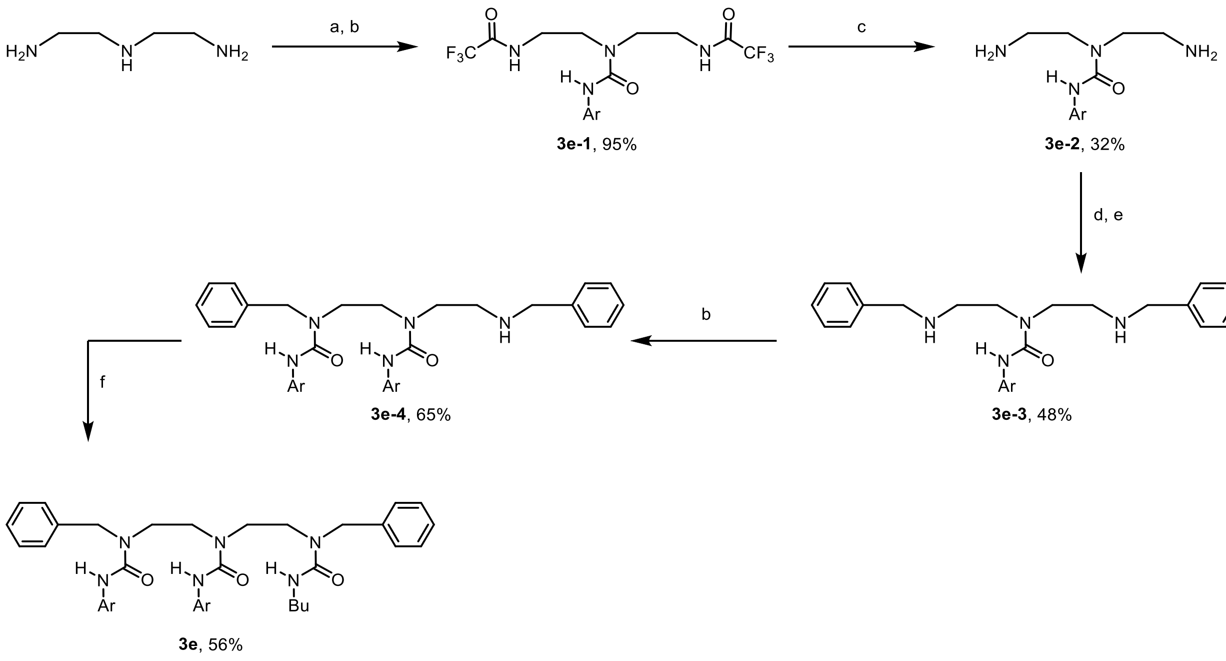


**Scheme S4.** Synthesis of **3e**. Reagents and conditions: (a) EtOC(O)$CF_3$ (2.2 equiv), DCM, 0 °C to RT, 2 h; (b) 4-MeO-PhNCO (1.5 equiv), DCM, RT, 16 h; (c) NaOH, MeOH, $H_2O$, RT, 5 h; (d) benzaldehyde (2.0 equiv), EtOH, RT, 16 h; (e) $NaBH_4$ (3.0 equiv), EtOH, 0 °C to RT, 3 h; (f) $^{n}$BuNCO (1.5 equiv), DCM, RT, 16 h. For all compounds, Ar = 4-MeO-Ph.

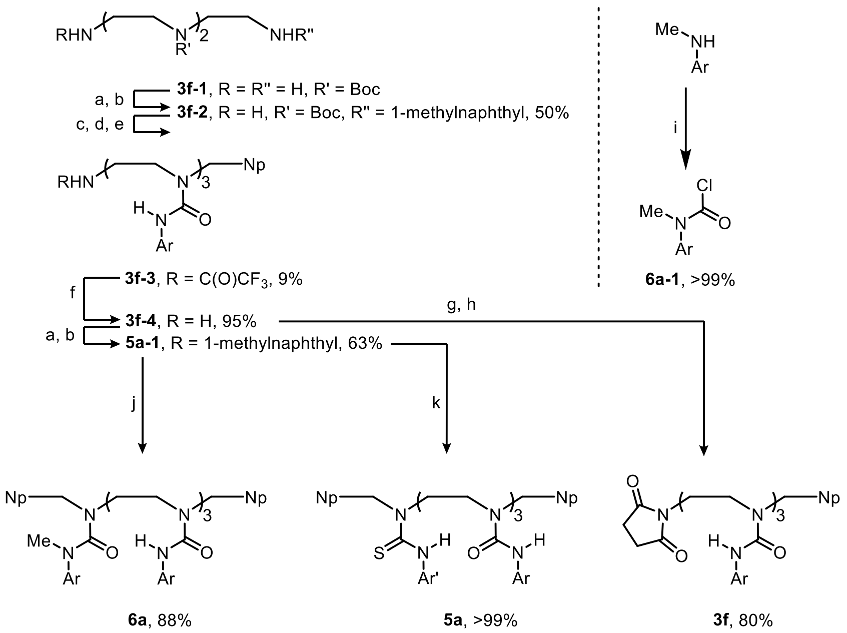


**Scheme S5.** Synthesis of **3f**, **5a** and **6a**. Reagents and conditions: (a) 1-naphthaldehyde (1.0 equiv), EtOH, RT, 16 h; (b) $NaBH_4$, EtOH, 0 °C to RT, 3 h; (c) Ethyl trifluoroacetate (1.1 equiv per primary amine), DCM, 0 °C to RT, 1 h; (d) HCl/$Et_2O$, EtOH, RT, 16 h; (e) 4-methoxyphenyl isocyanate (1.5 equiv per amine), TEA (2.0 equiv per amine), DCM, RT, 16 h; (f) NaOH (7.3 equiv), MeOH/$H_2O$, RT,

5 h; (g) succinic anhydride, DCM, reflux, 16 h; (h) Acetyl chloride, DCM, 0 °C to RT, 3 h; (i) triphosgene (0.5 equiv, DCM, 0 °C to RT, 1 h; (j) *N*-methyl-*N*-(4-methoxyphenyl)carbamoyl chloride (1.5 equiv), TEA (1.5 equiv), DCM, RT, 16 h; (k) 4-nitrophenyl isothiocyanate, DCM, RT, 16 h. For all compounds, Ar = 4-MeO-Ph and Ar' = 4-$O_2$N-Ph.

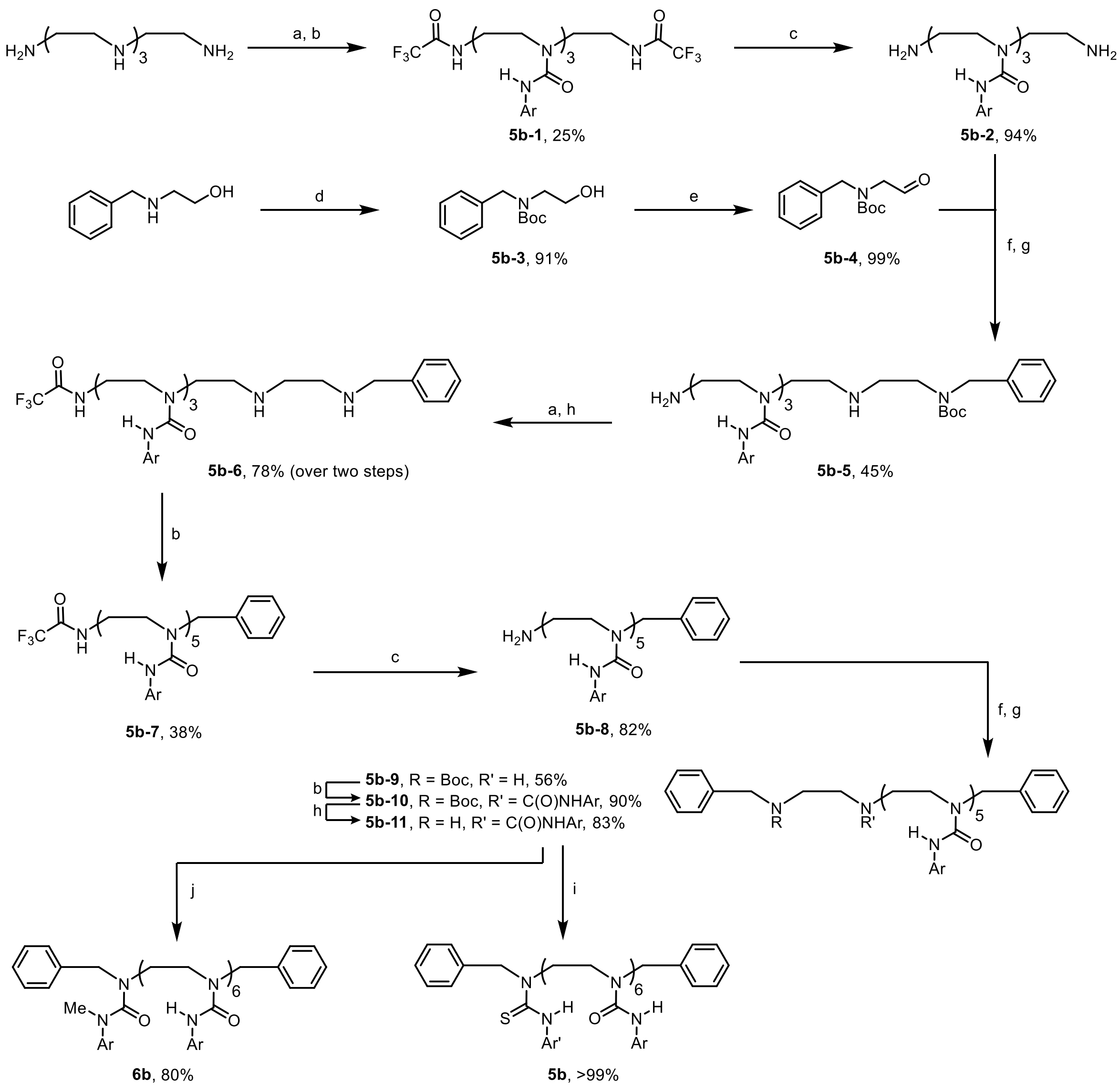


**Scheme S6.** Synthesis of extended oligoureas **5b** and **6b**. Reagents and conditions: (a) Ethyl trifluoroacetate (1.1 equiv per primary amine), DCM, 0 °C to RT, 1 h; (b) 4-methoxyphenyl isocyanate (1.5 equiv per amine), DCM, RT, 16 h; (c) NaOH (7.3 equiv), MeOH/$H_2O$, RT, 5 h; (d) $Boc_2O$ (1.0 equiv), aq. NaOH (1 M), DCM, RT, 16 h; (e) oxalyl chloride (1.5 equiv), DMSO (3.0 equiv), TEA (9.0 equiv), DCM, −78 °C to 0 °C, 3 h; (f) **5b-4** (1.0 equiv), EtOH, RT, 16 h; (g) $NaBH_4$, EtOH, 0 °C to RT, 3 h; (h) TFA, DCM, RT, 16 h; (i) 4-nitrophenyl isothiocyanate (1.5 equiv), DCM, RT, 16 h; (j) *N*-methyl-*N*-(4-methoxyphenyl)carbamoyl chloride (1.5 equiv), TEA (1.5 equiv), DCM, RT, 16 h. For all compounds, Ar = 4-MeO-Ph and Ar' = 4-$O_2$N-Ph.

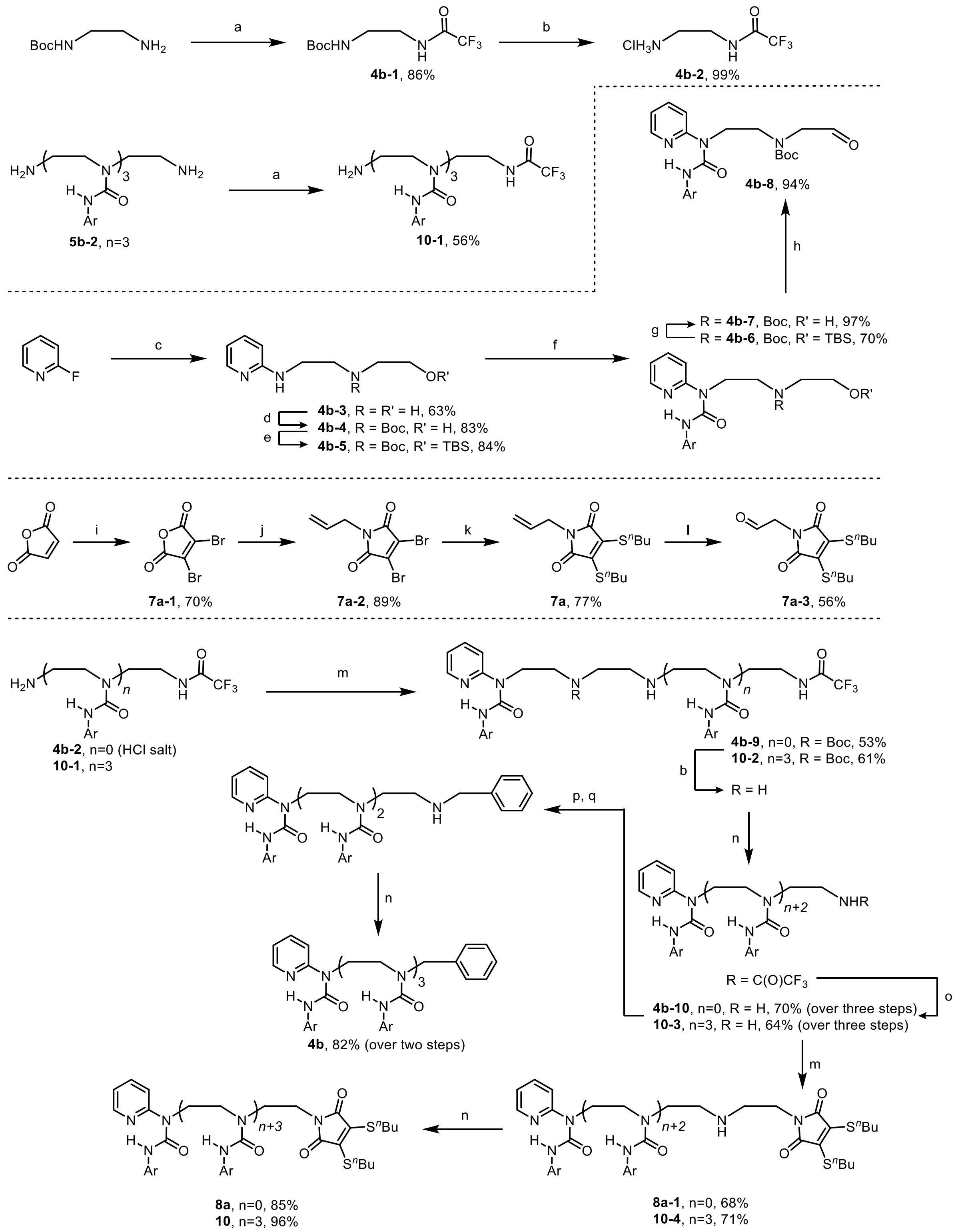


**Scheme S7.** Synthesis of pyridine-terminated oligoureas **4b**, **8a** and **10** and dithiomaleimide control compound **7a**. Reagents and conditions: (a) Ethyl trifluoroacetate (1.0–1.6 equiv per amine), DCM, 0 °C to RT, 4 h (for **10-1**) or 96 h (for **4b-1**); (b) TFA, DCM, RT, 15–24 h (*then* HCl in MeOH for **4b-2**); (c) *N*-(2-hydroxyethyl)ethylenediamine (3.0 equiv), neat, 120 °C, 17.5 h; (d) $Boc_2O$ (1.0 equiv), THF, 0 °C to RT, 20 h; (e) TBSCl, TEA, DMF, 0 °C to RT, 4 h; (f) 4-methoxyphenyl isocyanate, DCE,

40 °C, 20 h; (g) TBAF, THF, RT, 40 min; (h) DMP, DCM, 0 °C to RT, 6 h; (i) $Br_2$, $AlCl_3$, neat, 120 °C, 16 h; (j) Allylamine, AcOH, reflux, 5 h; (k) *n*-butanethiol, TEA, $Et_2O$, RT, 16 h; (l) $OsO_4$ (10 mol%), $NaIO_4$, 2,6-lutidine, Dioxane/$H_2O$, RT, 16 h; (m) Aldehyde (1.0 equiv), $NaB(OAc)_3H$, TEA (for **4b-9**), DCE, RT, 2–5 h; (n) 4-methoxyphenyl isocyanate (1.0 equiv per amine), DCM, RT, 30 min–2 h; (o) NaOH (8 equiv), EtOH/THF/$H_2O$ (2:1:1), RT, 16–18 h; (p) Benzaldehyde, MeOH/THF (1:1), RT, 17 h; (q) $NaBH_4$, MeOH/THF (1:1), 0 °C to RT, 3 h. For all compounds, Ar = 4-MeO-Ph.

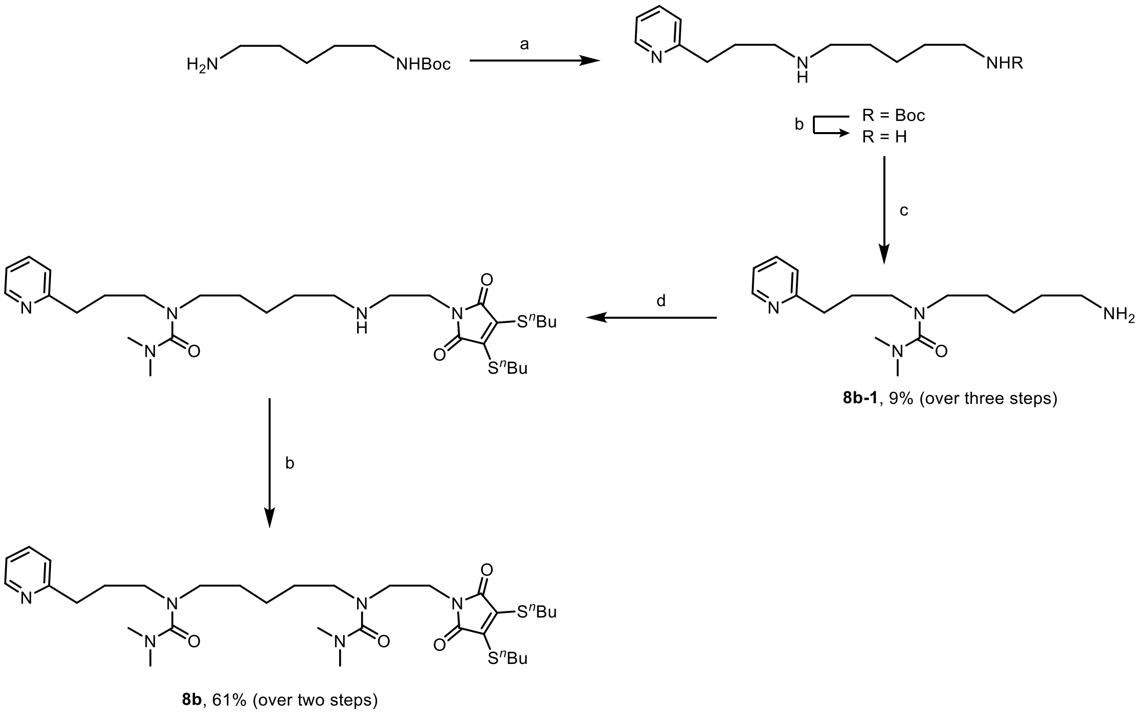


**Scheme S8.** Synthesis of fluorescent control compound **8b**. Reagents and conditions: (a) 3-(2-pyridyl)propionaldehyde, $NaB(OAc)_3H$, DCE, RT, 2 h; (b) *N*,*N*-dimethylcarbamoyl chloride, TEA, DCE, 45 °C, 16 h; (c) TFA, DCM, RT, 2 h; (d) **7a-3** (1.0 equiv), $NaB(OAc)_3H$, DCE, RT, 2 h.

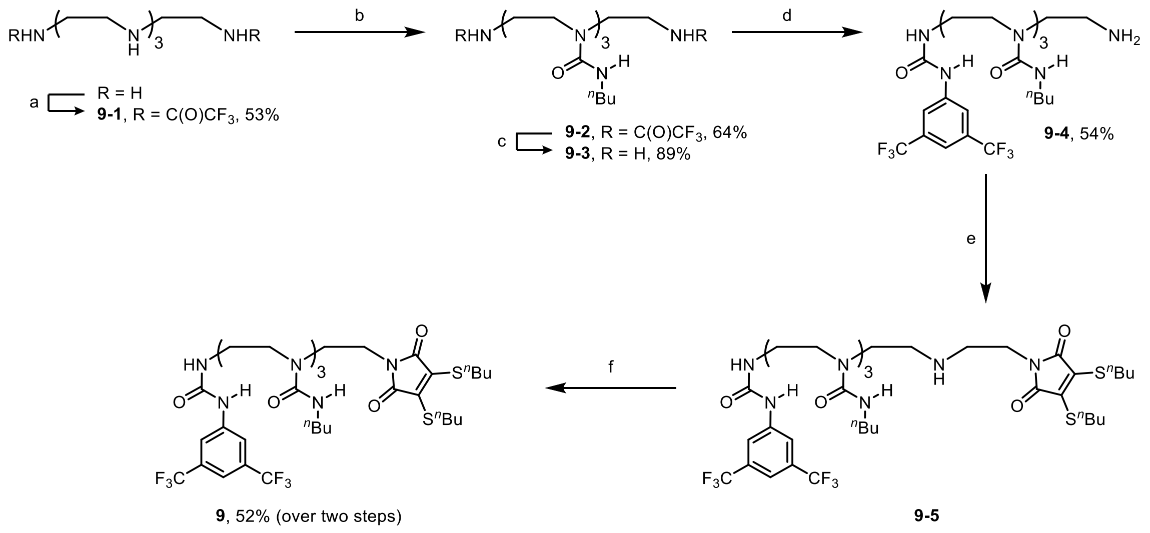


**Scheme S9.** Synthesis of anion-binding foldamer **9**. Reagents and conditions: (a) Ethyl trifluoroacetate (2.0 equiv), DCM, 0 °C to RT, 24 h; (b) $^n$BuNCO (3.0 equiv), DCE, 40 °C, 16 h; (c) NaOH (8 equiv), EtOH/THF/$H_2O$ (2:1:1), RT, 18 h; (d) 3,5-bis(trifluoromethyl)phenyl isocyanate (0.7 equiv), DCM, 0 °C to RT, 4 h; (e) **7a-3** (1.0 equiv), NaB(OAc)$_3$H, DCE, RT, 2 h; (f) $^n$BuNCO (1.0 equiv), DCM, RT, 2 h.

General Procedures

Experimental Procedures

**General procedure A – (thio)urea formation from amines and iso(thio)cyanates**

Under a dry, inert atmosphere, a solution of secondary amine (1.00 equiv, 0.10 M) in anhydrous DCM was cooled to 0 °C. To this solution was added iso(thio)cyanate (1.50 equiv per amine) dropwise/over three portions. The resulting solution was stirred for 16 h and then concentrated *in vacuo* to give the crude (thio)urea.

**General procedure B – reductive amination by $NaBH_4$ reduction**

Under a dry, inert atmosphere, to a solution of primary amine (1.00 equiv, 0.10 M) in anhydrous MeOH was added aldehyde (1.50 equiv per amine). The resulting solution was stirred for 16 h. The solution was then cooled to 0 °C and sodium borohydride (3.00 equiv per amine) was added over three portions. The resulting white suspension was warmed to room temperature, stirred for 3 h and concentrated *in vacuo*. The residue was then diluted with MeOH and concentrated *in vacuo*. The crude product was diluted with distilled water and the aqueous solution extracted with DCM three times. The combined organic extracts were washed with brine, dried ($MgSO_4$), filtered and concentrated *in vacuo* to give the crude secondary amine.

**General procedure C – trifluoroacetylation and urea formation on oligoethylenediamines**

Under a dry, inert atmosphere, a solution of amine (1.00 equiv, 1.6 M) in anhydrous DCM was cooled to 0 °C. Ethyl trifluoroacetate (1.10 equiv per primary amine) was added dropwise over 30 minutes and the resultant solution was stirred for 30 minutes at 0 °C. The solution was then warmed to room temperature and stirred for a further hour. Isocyanate (1.50 equiv per secondary amine) and the resultant solution was stirred for 16 h.

**General procedure D – hydrolysis of trifluoroacetamides**

To a solution of trifluoroacetamide (1.00 equiv, 0.10 M) was added aqueous sodium hydroxide (15% w/w). The resultant colourless solution was stirred for 5 hours and concentrated *in vacuo*. The crude product was then dissolved in distilled water and washed with DCM three times. The combined organic

extracts were washed with brine, dried ($MgSO_4$), filtered and concentrated *in vacuo* to give the crude primary amine.

**General procedure E – succinimidation of amines by *in situ* activation with acetyl chloride**

Under a dry inert, atmosphere, succinic anhydride (1.00 equiv per amine) was added in one portion to a solution of amine (1.00 equiv, 0.10 M) in anhydrous DCM. TEA (1.00 equiv per amine) was added and the solution was refluxed for 16 h. The solution was cooled to 0 °C and acetyl chloride (2.00 equiv per amine) was added dropwise and the solution was stirred at room temperature for a further 3 hours. The resulting solution was concentrated *in vacuo* and the crude product diluted in saturated aqueous $NaHCO_3$. The aqueous solution was extracted with DCM three times and the combined organic extracts were washed with brine, dried ($MgSO_4$), filtered and concentrated *in vacuo* to give the crude succinimide.

**General procedure F – chemoselective Boc-protection of secondary amines by trifluoroacetylation, carboxylation and hydrolysis.**

Under a dry, inert atmosphere, a solution of amine (1.00 equiv, 1.60 M) in anhydrous DCM was cooled to 0 °C. To the solution was added ethyl trifluoroacetate (1.10 equiv) dropwise over 30 minutes and the resultant solution was stirred for 30 minutes. The solution was warmed to room temperature and stirred for a further hour. The solution was then diluted with anhydrous DCM (10 mL $mmol^{-1}$) and cooled to 0 °C. A solution of di-*tert*-butyl dicarbonate (1.20 equiv per amine, 2.00 M) in anhydrous DCM was added over three portions followed by anhydrous TEA (1.00 equiv per amine) in one portion. The resulting solution was stirred for 16 h and diluted with saturated aqueous $NaHCO_3$. The organic phase was washed with deionised water, dried ($MgSO_4$), filtered and concentrated *in vacuo*. The crude yellow oil (0.20 M) was dissolved in methanol and a solution of aqueous NaOH (7.30 equiv, 4.20 M) was added. The resulting colourless solution was stirred for 5 h and concentrated *in vacuo*. The crude product was dissolved in deionised water and the aqueous phase was extracted with DCM four times. The combined organic extracts were dried ($MgSO_4$), filtered and concentrated *in vacuo* to give the title carbamate.

**General procedure G – urea formation from amines and carbamoyl chlorides**

Under a dry, inert atmosphere, a solution of secondary amine (1.00 equiv, 0.10 M) in anhydrous DCM was cooled to 0 °C. To this solution was added carbamoyl chloride (1.50 equiv per secondary amine) dropwise/over three portions and anhydrous triethylamine (1.50 equiv per secondary amine). The resulting solution was stirred for 16 h and then diluted with distilled water. The aqueous solution was

extracted with DCM three times and the combined organic extracts were washed with brine, dried ($MgSO_4$), filtered and concentrated *in vacuo* to give the crude urea.

**General procedure H – Boc deprotection of amines using TFA**

Under a dry, inert atmosphere, TFA (20% by volume) was added dropwise to a solution of Boc-protected amine (1.00 equiv, 0.10 M) in anhydrous DCM (80% by volume). The solution was stirred for 16 h and concentrated *in vacuo*. The crude product was diluted with saturated aqueous $NaHCO_3$ and then washed with DCM three times. The combined organic extracts were washed with brine, dried ($MgSO_4$), filtered and concentrated *in vacuo* to give the crude amine.

**General procedure I – palladium-catalysed hydrogenative reductive amination**

Under a dry, inert atmosphere, a round-bottomed flask was charged with a solution of amine (1.00 equiv, 0.10 M) in anhydrous MeOH. To this solution was added aldehyde (1.08 equiv) and palladium on carbon (10% wt., 10 mol%). The flask was then equipped with a three-way tap with a balloon filled with hydrogen attached. The flask was evacuated and refilled with nitrogen three times, and then evacuated and refilled with hydrogen. The black suspension was then left to stir for 16 h. The black suspension was then filtered through celite and washed with MeOH. The filtrate was the concentrated *in vacuo* to give the crude secondary amine.

**General procedure J – monoalkylation of diamines by reductive amination**

Under a dry, inert atmosphere, a solution of amine (1.00 equiv, 0.10 M) in anhydrous MeOH was prepared. To this solution, aldehyde (1.10 equiv) was added dropwise over 30 minutes. The resultant solution was stirred for 16 h. The solution was then cooled to 0 °C and sodium borohydride (3.00 equiv) was added over three portions. The resulting white suspension was warmed to room temperature, stirred for 3 h and concentrated *in vacuo*. The residue was then diluted with MeOH and concentrated *in vacuo*. The crude product was then diluted with distilled water and the aqueous solution extracted with DCM three times. The combined organic extracts were washed with brine, dried ($MgSO_4$), filtered and concentrated *in vacuo* to give the crude secondary amine.

**General procedure K – Swern oxidation of primary alcohols**

Under a dry, inert atmosphere, a solution of anhydrous DMSO (3.00 equiv, 0.40 M) in anhydrous DCM was cooled to −78 °C. To this solution was added a solution of oxalyl chloride (1.50 equiv, 2.00 M) in anhydrous DCM dropwise. The resultant solution was stirred for 20 minutes, and a solution of alcohol (1.00 equiv, 0.80 M) in anhydrous DCM was added dropwise. The resulting solution was stirred for 2 h at −78 °C and anhydrous TEA (9.00 equiv) was added dropwise to give a white suspension. The suspension was warmed to 0 °C and stirred for 30 minutes, after which the reaction was quenched with aqueous citric acid (10% w/w.). The mixture was stirred for 15 minutes at 0 °C and warmed to room temperature. The aqueous phase was then extracted with DCM three times, and the combined organic extracts washed with aqueous citric acid (10% w/w), distilled water, saturated aqueous $Na_2CO_3$ and brine. The combined organic extracts were then dried ($MgSO_4$), filtered and concentrated *in vacuo* to give the crude aldehyde.

**General procedure L – Johnson-Lemieux oxidation of *N*-allyl carbamates/maleimides**

Under an inert atmosphere, a solution of allyl carbamate/maleimide (1.00 equiv, 0.10 M) in dioxane:water (3:1) was prepared. To this solution was added sodium periodate (4.00 equiv), 2,6-lutidine (2.00 equiv) and osmium tetroxide (4% wt. in water, 0.10 equiv). The solution was stirred for 4 h and then quenched with saturated aqueous $Na_2S_2O_3$. After stirring for 30 minutes, the solution was diluted with EtOAc and the organic phase washed with saturated aqueous $Na_2S_2O_3$ and brine. The organic phase was then dried ($MgSO_4$), filtered and concentrated *in vacuo* to give the crude aldehyde.

## 6.3 Experimental Procedures

**1,1-Dibenzyl-3-(4-methoxyphenyl)urea, 1**

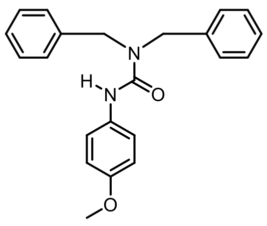

Synthesised according to general procedure A using *N*,*N*-dibenzylamine (198 mg, 1.00 mmol) and 4-methoxyphenyl isocyanate (0.10 mL, 0.75 mmol). The crude product was diluted with aqueous HCl (1.00 M) and extracted with DCM (3 x 10 mL). The combined organic extracts were dried ($MgSO_4$), filtered and concentrated *in vacuo* to yield the title amine as a white solid (296 mg, 1.00 mmol, >99%). **M.P.** 179-180 °C ($CHCl_3$). **TLC** – $R_f$ = 0.23 ($SiO_2$, 1:99 MeOH:DCM). **$^1$H NMR** (400 MHz, $CDCl_3$) $\delta_H$ 3.66 (s, 3H, OC*H*$_3$), 4.50 (s, 4H, 2 x C*H*$_2$Ar), 6.17 (s, 1H, N*H*), 6.69 (d, J = 9.0, 2H, 2 x Ar*H*), 7.03 (d, J = 9.0, 2H, 2 x Ar*H*), 7.19-7.31 (m, 10H, 10 x Ar*H*). **$^{13}$C NMR** (101 MHz, $CDCl_3$) $\delta_C$ 50.8 (2 x *C*H$_2$Ar), 55.6 (O*C*H$_3$), 114.2 (2 x Ar*C*), 122.3 (2 x Ar*C*), 127.5 (4 x Ar*C*), 127.8 (2 x Ar*C*),

129.0 (4 x Ar*C*), 132.1 (2 x Ar*C*), 137.4 (Ar*C*), 156.0 (Ar*C*), 156.5 (*C*O). **HR-MS** (ESI, positive ion mode) – *m/z* for $[C_{22}H_{22}N_2O_2+H]^+$ = 347.1760. Found 347.1727. **FTIR** (neat) – 3431, 2988, 2901, 1653.

**1,4-Dibenzyl-1,4-bis(4-methoxyanilinylcarbonyl)-1,4-diazabutane, 2a**

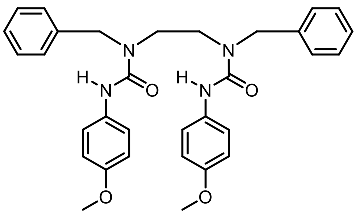

Synthesised according to general procedure A using *N,N'*-dibenzylethylenediamine (100 mg, 0.42 mmol) and 4-methoxyphenyl isocyanate (0.16 mL, 1.25 mmol). The crude product was purified by column chromatography (25-100% EtOAc:PE) to yield the title diurea as a foamy white solid (152 mg, 0.28 mmol, 67%). **M.P.** 136-137 °C ($CHCl_3$). **TLC** – $R_f$ = 0.23 ($SiO_2$, 5:95 MeOH:DCM). **$^1$H NMR** (400 MHz, $CDCl_3$) $\delta_H$ 3.36 (s, 4H, 2 x NC*H*$_2$), 3.76 (s, 6H, 2 x OC*H*$_3$), 4.45 (s, 4H, 2 x C*H*$_2$Ar), 6.80 (d, J = 9.0, 4H, 4 x Ar*H*), 7.19 (d, J = 9.0, 4H, 4 x Ar*H*, 7.29-7.39 (m, 10H, 10 x Ar*H*), 7.61 (s, 2H, 2 x N*H*). **$^{13}$C NMR** (101 MHz, $CDCl_3$) $\delta_C$ 46.7 (2 x *C*H$_2$), 51.9 (2 x *C*H$_2$Ar), 55.5 (2 x O*C*H$_3$), 114.0 (4 x Ar*C*), 121.9 (4 x Ar*C*), 127.3 (4 x Ar*C*), 127.8 (4 x Ar*C*), 129.0 (2 x Ar*C*), 132.2 (2 x Ar*C*), 137.5 (2 x Ar*C*), 155.5 (2 x Ar*C*), 156.4 (2 x *C*O). **HR-MS** (ESI, positive ion mode) – *m/z* for $[C_{32}H_{34}N_4O_4+Na]^+$ = 561.2478. Found 561.2467. **FTIR** (neat) – 3291, 2945, 2901, 1638.

**1,4-Diethyl-1,4-bis(4-methoxyanilinylcarbonyl)-1,4-diazabutane, 2b**

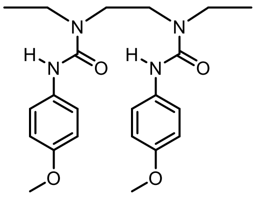

Synthesised according to general procedure A using *N,N'*-diethylethylenediamine (100 mg, 0.86 mmol) and 4-methoxyphenyl isocyanate (0.33 mL, 2.58 mmol). The crude product was purified by column chromatography (25-100% EtOAc:PE) to yield the title diurea as a foamy white solid (294 mg, 0.71 mmol, 82%). **M.P.** 219-220 °C ($CHCl_3$). **TLC** – $R_f$ = 0.40 ($SiO_2$, 90:10 EtOAc:PE). **$^1$H NMR** (400 MHz, $CDCl_3$) $\delta_H$ 1.23 (t, J = 7.1, 6H, 2 x C*H*$_3$), 3.40 (q, J = 7.1, 4H, 2 x C*H*$_2$), 3.47 (s, 4H, 2 x C*H*$_2$), 3.78 (s, 6H, 2 x OC*H*$_3$), 6.82 (d, J = 9.0, 4H, 4 x Ar*H*), 7.31 (s, 2H, 2 x N*H*), 7.41 (d, J = 9.0, 4H, 4 x Ar*H*). **$^{13}$C NMR** (101 MHz, $CDCl_3$) $\delta_C$ 14.4 (2 x *C*H$_3$), 43.5 (2 x N*C*H$_2$CH$_3$), 46.7 (2 x *C*H$_2$), 55.7 (2 x O*C*H$_3$), 114.1 (4 x Ar*C*), 121.9 (4 x Ar*C*), 132.7 (2 x Ar*C*), 155.7 (2 x Ar*C*), 155.9 (2 x *C*O). **HR-MS** (ESI, positive ion mode) – *m/z* for $[C_{22}H_{30}N_4O_4+Na]^+$ = 437.2165. Found 437.2143. **FTIR** (neat) – 3280, 2984, 2919, 1629.

**1,4-Diphenyl-1,4-bis(4anilinylcarbonyl)-1,4-diazabutane, 2c**

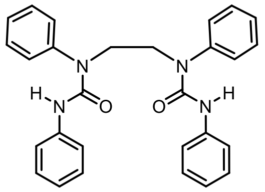

Synthesised according to general procedure A using *N,N'*-diphenylethylenediamine (50 mg, 0.24 mmol) and phenyl isocyanate (0.06 mL, 0.58 mmol). The crude product was purified by column chromatography (3% MeOH:DCM) to yield the title diurea as a white solid (108 mg, 0.24 mmol, >99%). **M.P.** 230-231 °C (DCM). **$^{1}$H NMR** (400 MHz, $CDCl_3$) $\delta_H$ 3.93 (s, 4H, 2 x C*H*$_2$), 6.49 (s, 2H, 2 x N*H*), 6.91 (t, J = 7.2, 2H, 2 x Ar*H*), 7.08-7.18 (m, 4H, 4 x Ar*H*), 7.20-7.26 (m, 8H, 8 x Ar*H*), 7.26-7.31 (m, 2H, 2 x Ar*H*), 7.31-7.43 (m, 4H, 4 x Ar*H*). **$^{13}$C NMR** (101 MHz, $CDCl_3$) $\delta_C$ 48.6 (2 x N*C*H$_2$), 119.7 (4 x Ar*C*), 123.1 (4 x Ar*C*), 128.1 (4 x Ar*C*), 128.3 (4 x Ar*C*), 128.9 (4 x Ar*C*), 130.4 (4 x Ar*C*), 138.9 (2 x Ar*C*), 141.9 (2 x Ar*C*), 154.6 (2 x *C*O). **HR-MS** (ESI, positive ion mode) – *m/z* for $[C_{28}H_{26}N_4O_2+Na]^+$ 473.1948, observed 473.1954. **FTIR** (neat) = 3417, 1668, 1593, 1518, 1492, 1488, 1198.

**1,7-Dibenzyl-1,4,7-triazaheptane, 3a-1**

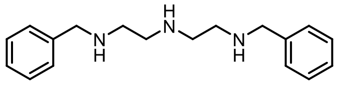

Synthesised according to general procedure B using diethylenetriamine (0.21 mL, 1.94 mmol) and benzaldehyde (0.39 mL, 3.88 mmol). The title triamine was isolated as a white powder (330 mg, 1.18 mmol, 61%). Spectroscopic data matched that previously reported.[1]

**1,7-Dibenzyl-1,4,7-tris(4-methoxyanilinylcarbonyl)-1,4,7-triazaheptane, 3a**

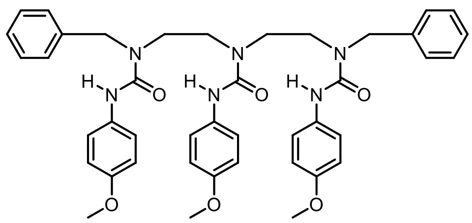

Synthesised according to general procedure A using **3a-1** (300 mg, 1.06 mmol) and 4-methoxyphenyl isocyanate (0.41 mL, 3.18 mmol). The crude product was purified by column chromatography (25-100% EtOAc:PE) to yield the title triurea as a white solid (418 mg, 0.57 mmol, 54%). **M.P.** 146-147 °C ($CHCl_3$). **TLC** – $R_f$ = 0.20 ($SiO_2$, 90:10 EtOAc:PE). **$^{1}$H NMR** (500 MHz, $CDCl_3$) $\delta_H$ 3.34 (t, J = 7.4, 4H, 2 x NC*H*$_2$), 3.48 (t, J = 7.4, 4H, 2 x NC*H*$_2$), 3.79 (s, 6H, 2 x OC*H*$_3$), 3.81 (s, 3H, OC*H*$_3$), 4.57 (s, 4H, 2 x C*H*$_2$Ar), 6.83 (d, J = 9.0, 4H, 4 x Ar*H*), 6.85 (d, J = 9.0, 2H, 2 x Ar*H*), 7.29 (d, J = 9.0, 4H, 4 x Ar*H*), 7.32-7.44 (m, 10H, 10 x Ar*H*), 7.59 (d, J = 9.0, 2H, 2 x Ar*H*), 8.84 (s, 1H, N*H*). **$^{13}$C NMR** (126 MHz, $CDCl_3$) $\delta_C$ 47.8 (2 x N*C*H$_2$), 47.8 (2 x N*C*H$_2$), 52.4 (2 x *C*H$_2$Ar), 55.6 (2 x O*C*H$_3$), 55.6 (O*C*H$_3$), 114.0 (2 x Ar*C*), 114.1 (4 x Ar*C*), 121.2 (2 x Ar*C*), 121.8 (4 x Ar*C*), 127.1 (4 x Ar*C*), 127.2 (2 x Ar*C*), 129.2 (4 x Ar*C*), 133.1 (2 x Ar*C*), 133.1 (Ar*C*), 155.4 (2 x Ar*C*), 155.4 (Ar*C*), 156.5 (2 x *C*O), 156.5 (*C*O). **HR-MS** (ESI, positive ion mode) – *m/z* for $[C_{42}H_{46}N_6O_6+Na]^+$ = 753.3377. Found 753.3362. **FTIR** (neat) – 3334, 2992, 2948, 1650, 1624.

**1,7-Dibenzyl-1,4,7-tris(butylaminocarbonyl)-1,4,7-triazaheptane, 3b**

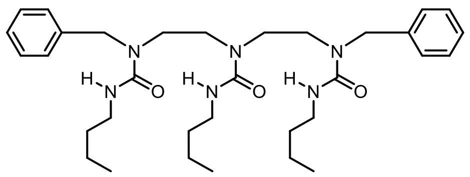

Synthesised according to general procedure A using **3a-1** (30 mg, 0.10 mmol) and *n*-butyl isocyanate (0.06 mL, 0.45 mmol). The crude product was purified by column chromatography (1-5% MeOH:DCM) to yield the title triurea as a white solid (31 mg, 0.05 mmol, 54%). **M.P.** 111-112 °C ($CHCl_3$). **TLC** – $R_f$ = 0.68 ($SiO_2$, 90:10 DCM:MeOH). **$^1$H NMR** (400 MHz, $CDCl_3$) $\delta_H$ 0.80 (t, J = 7.4, 6H, 2 x C*H*$_3$), 0.85 (t, J = 7.3, 3H, C*H*$_3$), 1.12-1.50 (m, 12H, 6 x C*H*$_2$), 3.04-3.26 (m, 14H, 7 x NC*H*$_2$), 4.34 (s, 4H, 2 x C*H*$_2$Ar), 5.06 (s, 2H, 2 x N*H*), 6.38 (t, J = 5.0, 1H, N*H*), 7.13 (d, J = 7.5, 4H, 4 x Ar*H*), 7.20 (t, J = 7.2, 2H, 2 x Ar*H*), 7.26 (t, J = 7.5, 4H, 4 x Ar*H*). **$^{13}$C NMR** (101 MHz, $CDCl_3$) $\delta_C$ 13.9 (2 x *C*H$_3$), 14.0 (*C*H$_3$), 20.1 (2 x *C*H$_2$), 20.3 (*C*H$_2$), 32.2 (*C*H$_2$), 32.3 (2 x *C*H$_2$), 47.3 (3 x N*C*H$_2$), 52.0 (2 x *C*H$_2$Ar), 127.0 (4 x Ar*C*), 127.7 (2 x Ar*C*), 129.0 (4 x Ar*C*), 138.0 (2 x Ar*C*), 158.7 (2 x *C*O), 158.8 (*C*O). **HR-MS** (ESI, positive ion mode) – *m/z* for $[C_{33}H_{52}N_6O_3+Na]^+$ = 603.3999. Found 603.4012. **FTIR** (neat) – 3326, 2963, 2910, 1640.

**1,10-Dibenzyl-1,4,7,10-tetrakis(4-methoxyanilinylcarbonyl)-1,4,7,10-tetraazadecane, 4a**

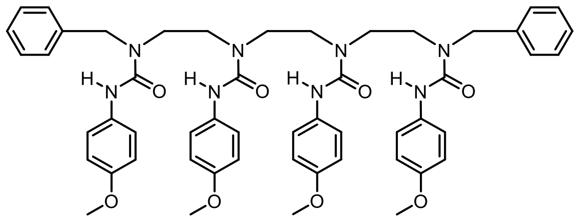

***Step 1*** (*reductive amination*): Under a dry, inert atmosphere, a solution of triethylenetetramine (0.31 mL, 2.05 mmol, 1.00 equiv, 0.10 M) in anhydrous MeOH (20 mL) was prepared. To this solution was added benzaldehyde (0.42 mL, 4.10 mmol, 2.00 equiv) and the resulting colourless solution was stirred for 16 h. The solution was cooled to 0 °C and $NaBH_4$ (388 mg, 10.26 mmol, 6.00 equiv) was added in one portion. The resulting white suspension was warmed to room temperature, stirred for 3 h and concentrated *in vacuo*. The residue was diluted with MeOH and concentrated *in vacuo*. The crude product was diluted with deionised water (20 mL) and the aqueous solution extracted with DCM (3 x 20 mL). The combined organic extracts were washed with brine, dried ($MgSO_4$), filtered and concentrated *in vacuo* to give the crude secondary amine. ***Step 2*** (*urea formation*): Under a dry, inert atmosphere, the crude brown oil (1.00 equiv, 0.10 M) was dissolved in anhydrous DCM (20 mL) and cooled to 0 °C. To the solution was added 4-methoxyphenyl isocyanate (1.30 mL, 12.30 mmol, 3.00 equiv) dropwise. The resulting solution was stirred for 16 h and concentrated *in vacuo*. The crude product was purified by column chromatography (1-2% MeOH:DCM) to yield the title tetraurea as a white solid (312 mg, 0.34 mmol, 16%). **M.P.** 158-159 °C (DCM). **TLC** – $R_f$ = 0.21 ($SiO_2$, 1:99 MeOH:DCM). **$^1$H NMR** (400 MHz, $CDCl_3$) $\delta_H$ 3.32 (s, 4H, 2 x NC*H*$_2$), 3.39-3.43 (m, 4H, 2 x NC*H*$_2$), 3.48 (t, J = 7.5, 4H, 2 x NC*H*$_2$), 3.76 (s, 6H, 2 x OC*H*$_3$), 3.78 (s, 6H, 2 x OC*H*$_3$), 6.80 (d, J = 9.1, 4H, 4 x Ar*H*), 6.82 (d, J = 9.1, 4H, 4 x Ar*H*), 7.27 (d, J = 9.1, 4H, 4 X Ar*H*), 7.29-7.40 (m, 10H, 10 x Ar*H*), 7.59 (d, J = 9.1, 4H, 4 x Ar*H*), 8.33 (s, 2H, 2 x N*H*), 8.93 (s, 2H,

2 x N*H*). **$^{13}$C NMR** (101 MHz, $CDCl_3$) $\delta_C$ 47.7 (6 x N*C*$H_2$), 52.2 (2 x *C*$H_2$Ar), 55.5 (2 x O*C*$H_3$), 55.5 (2 x O*C*$H_3$), 113.9 (4 x Ar*C*), 114.0 (4 x Ar*C*), 121.1 (4 x Ar*C*), 121.9 (4 x Ar*C*), 126.8 (4 x Ar*C*), 127.9 (2 x Ar*C*), 129.1 (4 x Ar*C*), 133.1 (4 x Ar*C*), 155.2 (4 x Ar*C*), 156.4 (4 x *C*O). **HR-MS** (ESI, positive ion mode) – *m/z* for $[C_{52}H_{58}N_8O_8+Na]^+$ = 945.4275. Found 945.4233. **FTIR** (neat) – 3330, 2992, 2948, 1643, 1604.

**1-Ethyl-7-(*tert*-butoxycarbonyl)-1,4,7-triazaheptane, 3c-1**

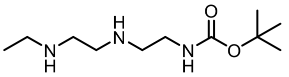

Synthesised according to general procedure I using *N*-ethylethylenediamine (0.60 mL, 5.67 mmol) and *N*-Boc-2-aminoacetaldehyde (910 mg, 6.12 mmol). The title carbamate was isolated as a colourless oil (879 mg, 3.80 mmol, 67%). **TLC** – $R_f$ = 0.23 ($SiO_2$, 10:90:1 MeOH:DCM:$NH_4OH$). **$^1$H NMR** (400 MHz, $CDCl_3$) $\delta_H$ 1.23 (t, J = 6.5, 3H, C*$H_3$*), 1.44 (s, 9H, C(C*$H_3$*)$_3$), 2.76 (t, J = 5.4, 2H, NC*$H_2$*), 2.91 (t, J = 6.3, 2H, NC*$H_2$*), 3.00 (t, J = 6.3, 2H, NC*$H_2$*), 3.23 ($C^2H_2$, 2H, m), 3.71 (q, J = 5.4, 2H, NC*$H_2$*), 5.45 (t, J = 5.4, 1H, N*H*). **$^{13}$C NMR** (101 MHz, $CDCl_3$) $\delta_C$ 18.4 (*C*$H_3$), 28.5 (C(*C*$H_3$), 40.1 (N*C*$H_2$), 46.0 (N*C*$H_2$), 47.1 (N*C*$H_2$), 49.1 (N*C*$H_2$), 58.4 (N*C*$H_2$), 81.4 (*C*($CH_3$)$_3$), 156.9 (*C*O). **HR-MS** (ESI, positive ion mode) – *m/z* for $[C_{11}H_{25}N_3O_2+H]^+$ = 232.2025 . Found 232.2019. **FTIR** (neat) – 3352, 2963, 1694.

**1-Ethyl-1,4-bis(4-methoxyanilinylcarbonyl)-7-(*tert*-butoxycarbonyl)-1,4,7-triazanonane, 3c-2**

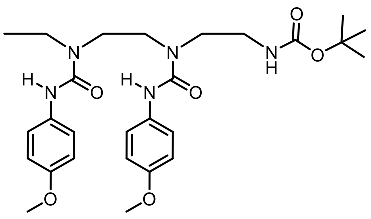

Synthesised according to general procedure A using **3c-1** (100 mg, 0.43 mmol) and 4-methoxyphenyl isocyanate (0.17 mL, 1.30 mmol). The crude product was purified by column chromatography (25-100% EtOAc:PE) to yield the title diurea as a foamy white solid (122 mg, 0.25 mmol, 57%). **M.P.** 158-159 °C ($CHCl_3$). **TLC** – $R_f$ = 0.31 ($SiO_2$, 90:10 EtOAc:PE). **$^1$H NMR** (400 MHz, $CDCl_3$) $\delta_H$ 1.15 (t, J = 7.1, 3H, C*$H_3$*), 1.43 (s, 9H, C(C*$H_3$*)$_3$), 3.15 (t, J = 6.5, 2H, NC*$H_2$*), 3.36-3.45 (m, 8H, 4 x NC*$H_2$*), 3.78 (s, 3H, OC*$H_3$*), 3.79 (s, 3H, OC*$H_3$*), 5.35 (s, 1H, N*H*), 6.82 (d, J = 9.1, 2H, 2 x Ar*H*), 6.84 (d, J = 9.1, 2H, 2 x Ar*H*), 7.4 (d, J = 9.1, 2H, 2 x Ar*H*), 7.54 (d, J = 9.1, 2H, 2 x Ar*H*), 8.51 (s, 1H, N*H*). **$^{13}$C NMR** (101 MHz, $CDCl_3$) $\delta_C$ 14.1 (*C*$H_3$), 28.2 (C(*C*$H_3$)$_3$), 40.1 (N*C*$H_2$), 43.0 (N*C*$H_2$), 45.9 (N*C*$H_2$), 47.8 (N*C*$H_2$), 47.9 (N*C*$H_2$), 55.5 (2 x O*C*$H_3$), 80.1 (*C*($CH_3$)$_3$), 114.0 (4 x Ar*C*), 121.3 (2 x Ar*C*), 121.8 (2 x Ar*C*), 133.0 (Ar*C*), 133.1 (Ar*C*), 155.3 (Ar*C*), 155.3 (Ar*C*), 155.9 (*C*O) 156.4 (*C*O), 157.1 (*C*O). **HR-MS** (ESI, positive ion mode) – *m/z* for $[C_{27}H_{39}N_5O_6+Na]^+$ = 552.2798. Found 552.2777. **FTIR** (neat) – 3311, 2917, 1645, 1628.

**1-Ethyl-1,4-bis(4-methoxyanilinylcarbonyl)-1,4,7-triazanonane, 3c-3**

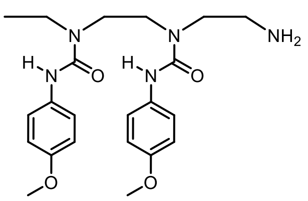

Synthesised according to general procedure H using **3c-2** (100 mg, 0.22 mmol). The title amine was isolated as a foamy white solid (76 mg, 0.21 mmol, 96%). **M.P.** 162-163 °C ($CHCl_3$). **TLC** – $R_f$ = 0.03 ($SiO_2$, 90:10 EtOAc:PE). **$^1$H NMR** (400 MHz, $CDCl_3$) $\delta_H$ 1.14 (t, J = 7.2, 3H, C$H_3$), 1.78 (s, 2H, N$H_2$), 2.89 (t, J = 4.2, 2H, NC$H_2$), 3.34-3.44 (m, 8H, 4 x NC$H_2$), 3.74 (s, 3H, OC$H_3$), 3.75 (s, 3H, OC$H_3$), 6.75 (d, J = 9.0, 2H, x Ar*H*), 6.77 (d, J = 9.0, 2H, x Ar*H*), 7.31 (d, J = 9.0, 2H, Ar*H*), 7.50 (d, J = 9.0, 2H, Ar*H*), 8.29 (s, 1H, N*H*), 10.0 (s, 1H, N*H*). **$^{13}$C NMR** (101 MHz, $CDCl_3$) $\delta_C$ 14.2 (*C*$H_3$), 41.7 (N*C*$H_2$), 42.7 (N*C*$H_2$), 44.8 (N*C*$H_2$), 48.1 (N*C*$H_2$), 52.8 (N*C*$H_2$), 55.5 (O*C*$H_3$), 55.6 (O*C*$H_3$), 113.9 (2 x Ar*C*), 114.0 (2 x Ar*C*), 120.8 (2 x Ar*C*), 121.5 (2 x Ar*C*), 133.3 (Ar*C*), 133.5 (Ar*C*), 155.1 (2 x Ar*C*), 155.9 (*C*O), 158.4 (*C*O). **HR-MS** (ESI, positive ion mode) – *m/z* for $[C_{22}H_{31}N_5O_4+H]^+$ = 430.2454. Found 430.2451. **FTIR** (neat) – 3444, 3354, 2966, 2902, 1671.

**1-Ethyl-1,4-bis(4-methoxyanilinylcarbonyl)-7-benzyl-1,4,7-triazanonane, 3c-4**

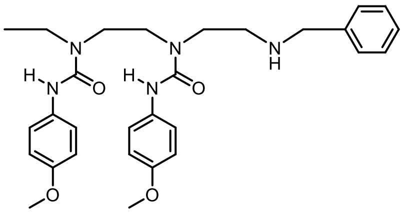

Synthesised according to general procedure B using **3c-3** (396 mg, 0.94 mmol) and benzaldehyde (0.15 mL, 1.41 mmol). The crude product was purified by column chromatography (2-10% MeOH:DCM) to yield the title amine as a white solid (249 mg, 0.48 mmol, 51%). **M.P.** 132-133 °C ($CHCl_3$). **TLC** – $R_f$ = 0.30 ($SiO_2$, 5:95 MeOH:DCM). **$^1$H NMR** (400 MHz, $CDCl_3$) $\delta_H$ 1.14 (t, J = 7.0, 3H, C$H_3$), 2.07 (s, 1H, N*H*), 2.85 (t, J = 4.3, NC$H_2$), 3.33-3.45 (m, 8H, 4 x NC$H_2$), 3.75 (s, 3H, OC$H_3$), 3.76 (s, 3H, OC$H_3$), 3.81 (s, 2H, C$H_2$Ar), 6.76 (d, J = 8.5, 2H, 2 x Ar*H*), 6.79 (d, J = 8.5, 2H, 2 x Ar*H*), 7.18 (d, J = 8.5, 2H, 2 x Ar*H*), 7.23-7.33 (m, 5H, 5 x Ar*H*), 7.51 (d, J = 8.5, 2H, 2 x Ar*H*), 8.27 (s, 1H, N*H*), 9.91 (s, 1H, N*H*). **$^{13}$C NMR** (101 MHz, $CDCl_3$) $\delta_C$ 14.1 (*C*$H_3$), 42.6 (N*C*$H_2$), 45.1 (N*C*$H_2$), 48.2 (N*C*$H_2$), 49.4 (N*C*$H_2$), 50.7 (N*C*$H_2$), 54.2 (*C*$H_2$Ar), 55.5 (O*C*$H_3$), 55.5 (O*C*$H_3$), 113.9 (2 x Ar*C*), 113.9 (2 x Ar*C*), 120.9 (2 x Ar*C*), 121.3 (2 x Ar*C*), 127.5 (2 x Ar*C*), 128.3 (2 x Ar*C*), 128.7 (Ar*C*), 133.1 (Ar*C*), 133.5 (Ar*C*), 138.9 (Ar*C*), 155.0 (2 x Ar*C*), 155.7 (*C*O), 158.2 (*C*O). **HR-MS** (ESI, positive ion mode) – *m/z* for $[C_{29}H_{37}N_5O_4+H]^+$ = 520.2924. Found 520.2893. **FTIR** (neat) – 3337, 3322, 2993, 2912, 1654.

**1-Ethyl-1,4,7-tris(4-methoxyanilinylcarbonyl)-7-benzyl-1,4,7-triazanonane, 3c**

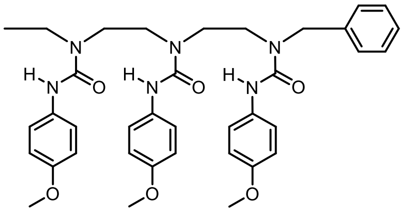

Synthesised according to general procedure A using **3c-4** (120 mg, 0.23 mmol) and 4-methoxyphenyl isocyanate (0.04 mL, 0.35 mmol). The crude product was purified by column chromatography (1-2% MeOH:DCM) to yield the title triurea as a foamy white solid (112 mg, 0.17 mmol, 73%). **M.P.** 110-111 °C ($CHCl_3$). **TLC** – $R_f$ = 0.15 ($SiO_2$, 1:99 MeOH:DCM). **$^1$H NMR** (500 MHz, $CDCl_3$) $\delta_H$ 1.16 (t, J = 7.1, 3H, C*H*3), 3.30 (q, J = 7.1, 2H, NC*H*2), 3.29-3.41 (m, 6H, 3 x NC*H*2), 3.49 (t, J = 7.4, 2H, NC*H*2), 3.76 (s, 3H, OC*H*3), 3.77 (s, 3H, OC*H*3), 3.77 (s, 3H, OC*H*3), 4.58 (s, 2H, C*H*2Ar), 6.79-6.83 (m, 6H, 6 x Ar*H*), 7.27-7.33 (m, 4H, 4 x Ar*H*), 7.34-7.39 (m, 5H, 5 x Ar*H*), 7.57 (d, J = 9.0, 2H, 2 x Ar*H*), 8.89 (s, 1H, N*H*). **$^{13}$C NMR** (126 MHz, $CDCl_3$) $\delta_C$ 14.3 (*C*H3), 43.7 (N*C*H2), 46.7 (N*C*H2), 47.9 (3 x N*C*H2), 51.9 (*C*H2Ar), 55.6 (3 x O*C*H3), 113.9 (2 x Ar*C*), 113.9 (2 x Ar*C*), 114.0 (2 x Ar*C*), 121.2 (2 x Ar*C*), 121.8 (2 x Ar*C*), 122.0 (2 x Ar*C*), 127.2 (2 x Ar*C*), 127.9 (Ar*C*), 129.0 (2 x Ar*C*), 133.3 (3 x Ar*C*), 137.8 (Ar*C*), 155.3 (*C*O), 155.6 (3 x Ar*C*), 156.0 (*C*O), 156.5 (*C*O). **HR-MS** (ESI, positive ion mode) – *m/z* for $[C_{37}H_{44}N_6O_6+H]^+$ = 669.3401. Found 669.3424. **FTIR** (neat) – 3312, 2978, 2923, 1650.

**1-Phenyl-7-(*tert*-butoxycarbonyl)-1,4,7-triazaheptane, 3d-1**

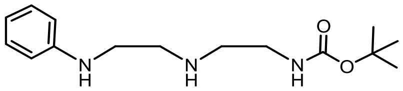

Synthesised according to general procedure I using *N*-phenylethylenediamine (0.50 mL, 3.80 mmol) and *N*-boc-2-aminoacetaldehyde (653 mg, 4.10 mmol). The title carbamate was isolated as a colourless oil (727 mg, 2.62 mmol, 69%). Spectroscopic data matched that previously reported.[2]

**1-Phenyl-1,4-bis(4-methoxyanilinylcarbonyl)-7-(*tert*-butoxycarbonyl)-1,4,7-triazanonane, 3d-2**

Synthesised according to general procedure A using **3d-1** (626 mg, 2.24 mmol) and 4-methoxyphenyl

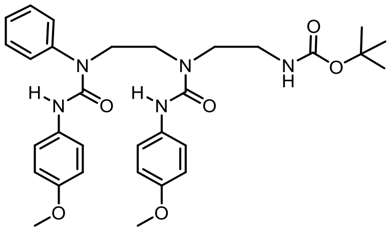

isocyanate (0.87 mL, 6.72 mmol). The crude product was purified by column chromatography (12-100% EtOAc:PE) to yield the title diurea as a foamy white solid (686 mg, 1.19 mmol, 53%). **M.P.** 80-81 °C ($CHCl_3$). **TLC** – $R_f$ = 0.19 ($SiO_2$, 50:50 EtOAc:PE). **$^1$H NMR** (400 MHz, $CDCl_3$) $\delta_H$ 1.40 (s, 9H, C(C*H*3)3), 3.26 (q, J = 5.7, NC*H*2), 3.44 (t, J = 6.1, 2H, NC*H*2), 3.65 (t, J = 5.7, 2H, NC*H*2), 3.77 (s, 3H, OC*H*3), 3.78 (s, 3H, OC*H*3), 3.82 (t, J = 6.1, 2H, NC*H*2), 5.14 (t, J = 5.7, 1H, N*H*), 6.15 (s, 1H, N*H*), 6.81 (d, J = 8.9, 2H, 2 x Ar*H*), 6.83 (d, J = 8.9, 2H, 2 x Ar*H*), 7.19 (d, J = 8.9, 2H, 2 x Ar*H*), 7.31 (d, J = 7.6, 2H, 2 x Ar*H*), 7.41 (t, J = 7.6, 1H, Ar*H*), 7.50 (t, J = 7.6, 2H, Ar*H*), 7.55 (d, J = 8.9, 2H, Ar*H*), 8.67 (s, 1H, N*H*). **$^{13}$C NMR** (101 MHz, $CDCl_3$) $\delta_C$ 28.6 (C(*C*H3)3), 40.2 (N*C*H2), 46.5 (N*C*H2), 47.3 (N*C*H2), 49.6 (N*C*H2), 55.6 (2 x O*C*H3),

79.5 (*C*(C$H_3$)$_3$), 114.0-114.2 (4 x Ar*C*), 121.5-121.8 (4 x Ar*C*), 128.1 (2 x Ar*C*), 128.7 (Ar*C*), 130.9 (2 x Ar*C*), 131.4 (Ar*C*), 133.5 (Ar*C*), 141.8 (Ar*C*), 155.3-155.5 (2 x Ar*C*), 156.0 (*C*O), 156.6 (2 x *C*O). **HR-MS** (ESI, positive ion mode) – *m/z* for $[C_{31}H_{39}N_5O_6+H]^+$ = 578.2979. Found 578.2954. **FTIR** (neat) – 3423, 3321, 2973, 2935, 1652, 1594.

**1-Phenyl-1,4-bis(4-methoxyanilinylcarbonyl)-1,4,7-triazaheptane, 3d-3**

Synthesised according to general procedure H using **3d-2** (200 mg, 0.34 mmol). The title amine was

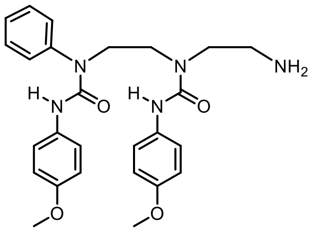


isolated as a foamy white solid (141 mg, 0.30 mmol, 87%). **M.P.** 123-124 °C (DCM). **TLC** – $R_f$ = 0.10 ($SiO_2$, 90:10 EtOAc:PE). **$^1$H NMR** (500 MHz, $CDCl_3$) $\delta_H$ 2.91 (t, J = 5.6, 2H, NC$H_2$), 3.41 (t, J = 5.6, 2H, NC$H_2$), 3.63 (t, J = 7.2, 2H, NC$H_2$), 3.76 (s, 3H, OC$H_3$), 3.77 (s, 3H, OC$H_3$), 3.86 (t, J = 7.2, 2H, NC$H_2$), 6.28 (s, 1H, N*H*), 6.79 (d, J = 9.0, 2H, 2 x Ar*H*), 6.81 (d, J = 9.0, 2H, 2 x Ar*H*), 7.20 (d, J = 9.0, 2H, 2 x Ar*H*), 7.33 (t, J = 8.2, 2H, 2 x Ar*H*), 7.38 (tt, J = 7.4, 1.2, 1H, Ar*H*), 7.42 (d, J = 9.0, 2H, 2 x Ar*H*), 7.48 (t, J = 8.2, 2H, 2 x Ar*H*), 9.23 (s, 1H, N*H*). **$^{13}$C NMR** (126 MHz, $CDCl_3$) $\delta_C$ 41.5 (N*C*$H_2$), 46.6 (N*C*$H_2$), 49.0 (N*C*$H_2$), 51.5 (N*C*$H_2$), 55.5 (2 x O*C*$H_3$), 113.9-114.0 (4 x Ar*C*), 121.1-121.8 (4 x Ar*C*), 128.0-128.2 (3 x Ar*C*), 130.5 (2 x Ar*C*), 131.4 (Ar*C*), 133.5 (Ar*C*), 141.8 (Ar*C*), 155.1-155.4 (2 x Ar*C*), 157.0 (2 x *C*O). **HR-MS** (ESI, positive ion mode) – *m/z* for $[C_{26}H_{31}N_5O_4+H]^+$ = 478.2454. Found 478.2446. **FTIR** (neat) – 3677, 3432, 2985, 2902, 1654.

**1-Phenyl-1,4-bis(4-methoxyanilinylcarbonyl)-7-benzyl-1,4,7-triazaheptane, 3d-4**

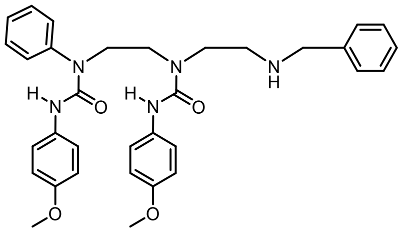


Synthesised according to general procedure B using **3d-3** (300 mg, 0.63 mmol) and benzaldehyde (0.13 mL, 1.26 mmol). The crude product was purified using column chromatography (1-5% MeOH:DCM, 1% TEA) to yield the title amine as a foamy white solid (285 mg, 0.50 mmol, 80%). **M.P.** 187-188 °C ($CHCl_3$). **TLC** – $R_f$ = 0.08 ($SiO_2$, 1:99 MeOH:DCM). **$^1$H NMR** (400 MHz, $CDCl_3$) $\delta_H$ 2.88 (t, J = 5.5, 2H, NC$H_2$), 3.48 (t, J = 5.5, 2H, NC$H_2$), 3.62 (t, J = 6.9, 2H, NC$H_2$), 3.76 (s, 3H, OC$H_3$), 3.77 (s, 3H, OC$H_3$), 3.81 (s, 2H, C$H_2$Ar), 3.85 (t, J = 6.9, 2H, NC$H_2$), 6.30 (s, 1H, N*H*), 6.78 (d, J = 9.0, 2H, 2 x Ar*H*), 6.79 (d, J = 9.0, 2H, 2 x Ar*H*), 7.20 (d, J = 9.0, 2H, 2 x Ar*H*), 7.26-7.39 (m, 10H, 10 x Ar*H*), 7.46 (d, J = 9.0, 2H, 2 x Ar*H*), 9.26 (s, 1H, N*H*). **$^{13}$C NMR** (101 MHz, $CDCl_3$) $\delta_C$ 46.8 (N*C*$H_2$), 48.9-49.3 (3 x N*C*$H_2$), 54.1 (*C*$H_2$Ar), 55.5 (2 x O*C*$H_3$), 113.9 (2 x Ar*C*), 114.1 (2 x Ar*C*), 121.2 (2 x Ar*C*), 121.8 (2 x Ar*C*), 127.0 (Ar*C*), 127.2 (2 x Ar*C*), 128.0 (2 x Ar*C*), 128.2 (Ar*C*), 128.3 (2 x Ar*C*), 128.5 (2 x Ar*C*), 131.5 (Ar*C*), 133.5 (Ar*C*), 141.9 (Ar*C*), 150.3 (Ar*C*), 155.0 (Ar*C*), 155.2 (Ar*C*), 155.9 (*C*O), 156.4 (*C*O). **HR-MS**

(ESI, positive ion mode) – *m/z* for $[C_{33}H_{37}N_5O_4+H]^+$ = 568.2924. Found 569.2897. **FTIR** (neat) – 3410, 2933, 2833, 1651.

**1-Phenyl-1,4,7-tris(4-methoxyanilinylcarbonyl)-7-benzyl-1,4,7-triazaheptane, 3d**

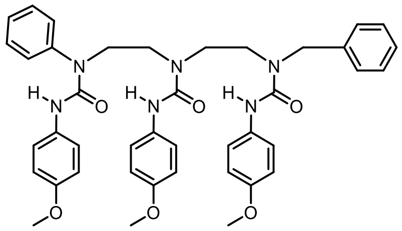

Synthesised according to general procedure A using **3d-4** (100 mg, 0.18 mmol) and 4-methoxyphenyl isocyanate (0.03 mL, 0.26 mmol). The crude product was purified using column chromatography (2-10% MeOH:DCM) to give the title triurea as a foamy white solid (65 mg, 0.09 mmol, 50%). **M.P.** 146-147 °C ($CHCl_3$). **TLC** – $R_f$ = 0.43 ($SiO_2$, 5:95 MeOH:DCM). **$^1$H NMR** (400 MHz, $CDCl_3$) $\delta_H$ 3.15 (t, J = 7.0, 2H, NC$H_2$), 3.35 (t, J = 7.0, 2H, NC$H_2$), 3.48 (t, J = 7.0, 2H, NC$H_2$), 3.64 (t, J = 7.0, 2H, NC$H_2$), 3.78 (s, 3H, OC$H_3$), 3.78 (s, 3H, OC$H_3$), 3.80 (s, 3H, OC$H_3$), 4.60 (s, 2H, C$H_2$Ar), 6.14 (s, 1H, N*H*), 6.73 (d, J = 9.1, 2H, 2 x Ar*H*), 6.74 (d, J = 9.1, 2H, 2 x Ar*H*), 6.75 (d, J = 9.1, 2H, 2 x Ar*H*), 7.10 (d, J = 9.1, 2H, 2 x Ar*H*), 7.20-7.36 (m, 10H, 10 x Ar*H*), 7.43 (d, J = 9.1, 2H, 2 x Ar*H*), 7.48 (d, J = 9.1, 2H, 2 x Ar*H*), 8.33 (s, 1H, N*H*), 8.72 (s, 1H, N*H*). **$^{13}$C NMR** (101 MHz, $CDCl_3$) $\delta_C$ 45.5 (N*C*$H_2$), 47.3 (N*C*$H_2$), 47.8 (N*C*$H_2$), 49.9 (N*C*$H_2$), 51.0 (*C*$H_2$Ar), 55.5 (3 x O*C*$H_3$), 113.9 (2 x Ar*C*), 113.9 (2 x Ar*C*), 114.1 (2 x Ar*C*), 121.2 (2 x Ar*C*), 121.4 (2 x Ar*C*), 121.9 (2 x Ar*C*), 127.4-128.7 (10 x Ar*C*), 130.7 (Ar*C*), 131.0 (Ar*C*), 133.0 (Ar*C*), 138.7 (Ar*C*), 141.7 (Ar*C*), 155.2 (Ar*C*), 155.3 (Ar*C*), 155.5 (Ar*C*), 156.1 (*C*O), 156.2 (*C*O), 156.4 (*C*O). **HR-MS** (ESI, positive ion mode) – *m/z* for $[C_{41}H_{44}N_6O_6+H]^+$ = 717.3401. Found 717.3391. **FTIR** (neat) – 3398, 2952, 1651.

**1,7-Bis(trifluoroacetyl)-4-(4-methoxyanilinylcarbonyl)-1,4,7-triazaheptane, 3e-1**

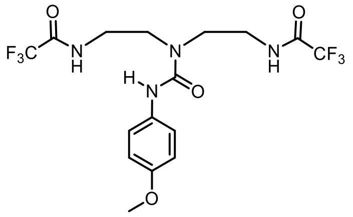

Synthesised according to general procedure C using diethylenetriamine (2.10 mL, 19.4 mmol) and 4-methoxyphenyl isocyanate (3.77 mL, 29.1 mmol). The resulting solution was concentrated *in vacuo* and the residue was recrystallised from DCM:$Et_2O$. The residue was then washed with $Et_2O$ and air-dried to yield the title urea as a white solid (8.20 g, 18.5 mmol, 95%). **M.P.** 134-135 °C ($CHCl_3$). **TLC** – $R_f$ = 0.25 ($SiO_2$, 5:95 MeOH:DCM). **$^1$H NMR** (500 MHz, $(CD)_3SO$) $\delta_H$ 3.38 (q, J = 5.9, 4H, 2 x NC$H_2$), 3.46 (t, J = 5.9, 4H, 2 x NC$H_2$), 6.84 (d, J = 8.9, 2H, 2 x Ar*H*), 7.32 (d, J = 8.9, 2H, 2 x Ar*H*), 8.08 (s, 1H, N*H*), 9.50 (t, J = 5.9, 2H, 2 x N*H*). **$^{13}$C NMR** (126 MHz, $(CD)_3SO$) $\delta_C$ 38.1 (2 x N*C*$H_2$), 45.7 (2 x N*C*$H_2$), 55.1 (O*C*$H_3$), 113.5 (2 x Ar*C*), 115.9 (q, J = 287.5, 2 x *C*$F_3$), 122.2 (2 x Ar*C*), 133.1 (Ar*C*), 154.7 (O*C*$H_3$), 155.3 (*C*O), 156.5 (q, J = 37.3, 2 x *C*O). **$^{19}$F NMR** (377 MHz, $CDCl_3$) $\delta_F$ −76.8 (2 x C*F*$_3$). **HR-MS** (ESI, positive ion mode) – *m/z* for $[C_{16}H_{18}F_6N_4O_4+H]^+$ = 445.1310. Found 445.1292. **FTIR** (neat) – 2960, 1731, 1706.

**4-(4-Methoxyanilinylcarbonyl)-1,4,7-triazaheptane, 3e-2**

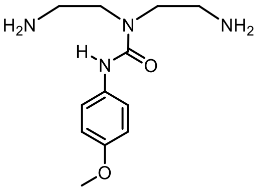

Synthesised according to general procedure D using **3e-1** (2.5 g, 5.63 mmol). The resulting solution was concentrated *in vacuo* and the residue was diluted with deionised water. The aqueous solution was extracted with DCM (3 x 50 mL). The combined organic extracts were washed with brine, dried ($MgSO_4$), filtered and concentrated *in vacuo* to give the title diamine as a colourless oil (450 mg, 1.78 mmol, 32%). **TLC** – $R_f$ = 0.04 ($SiO_2$, 10:90:1 DCM:MeOH:TEA).$^1$**H NMR** (400 MHz, $CDCl_3$) $\delta_H$ 1.49 (s, 4H, 2 x N*H*$_2$), 2.84 (t, J = 5.7, 4H, 2 x NC*H*$_2$), 3.29 (t, J = 5.7, 4H, 2 x NC*H*$_2$), 3.68 (s, 3H, OC*H*$_3$), 6.72 (d, J = 9.0, 2H, 2 x Ar*H*), 7.20 (d, J = 9.0, 2H, 2 x Ar*H*), 9.86 (s, 1H, N*H*). $^{13}$**C NMR** (101 MHz, $CDCl_3$) $\delta_C$ 41.2 (2 x N*C*H$_2$), 51.3 (2 x N*C*H$_2$), 55.5 (O*C*H$_3$), 114.0 (2 x Ar*C*), 120.8 (2 x Ar*C*), 133.8 (Ar*C*), 154.8 (Ar*C*), 158.3 (*C*O). **HR-MS** (ESI, positive ion mode) – *m/z* for $[C_{12}H_{20}N_4O_2+H]^+$ = 253.1665. Found 253.1653. **FTIR** (neat) – 2938, 2838, 1643, 1604.

**1,7-Dibenzyl-4-(4-methoxyanilinylcarbonyl)-1,4,7-triazaheptane, 3e-3**

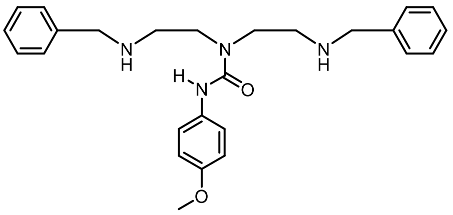

Synthesised according to general procedure B using **3e-2** (380 mg, 1.51 mmol) and benzaldehyde (0.46 mL, 4.52 mmol). The crude product was purified using column chromatography (2-10% MeOH:DCM) to yield the title diamine as a colourless oil (315 mg, 0.73 mmol, 48%). **TLC** – $R_f$ = 0.26 ($SiO_2$, 10:90 MeOH:DCM). $^1$**H NMR** (400 MHz, $CDCl_3$) $\delta_H$ 1.85 (s, 2H, 2 x N*H*), 2.86 (t, J = 5.6, 4H, 2 x NC*H*$_2$), 3.44 (t, J = 5.6, 4H, 2 x NC*H*$_2$), 3.76 (s, 3H, OC*H*$_3$), 3.81 (s, 4H, 2 x C*H*$_2$Ar), 6.76 (d, J = 9.0, 2H, 2 x Ar*H*), 7.14 (d, J = 9.0, 2H, 2 x Ar*H*), 7.26-7.34 (m, 10H, 10 x Ar*H*), 9.70 (s, 1H, N*H*). $^{13}$**C NMR** (101 MHz, $CDCl_3$) $\delta_C$ 48.7 (2 x N*C*H$_2$), 49.2 (2 x N*C*H$_2$), 54.2 (2 x *C*H$_2$Ar), 55.7 (O*C*H$_3$), 114.1 (2 x Ar*C*), 121.1 (2 x Ar*C*), 127.2 (2 x Ar*C*), 128.4 (4 x Ar*C*), 128.7 (4 x Ar*C*), 133.7 (Ar*C*), 139.7 (2 x Ar*C*), 155.0 (Ar*C*), 158.2 (*C*O). **HR-MS** (ESI, positive ion mode) – *m/z* for $[C_{26}H_{32}N_4O_2+H]^+$ = 433.2604. Found 4332.2586. **FTIR** (neat) – 2931, 2835, 1650.

**1,7-Dibenzyl-1,4-bis(4-methoxyanilinylcarbonyl)-1,4,7-triazaheptane, 3e-4**

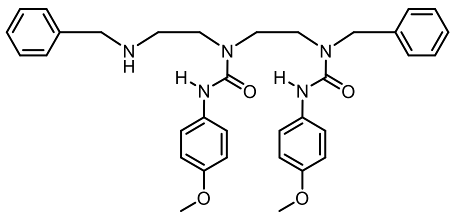

Synthesised according to general procedure A using **3e-3** (271 mg, 0.63 mmol) and 4-methoxyphenyl isocyanate (0.08 mL, 0.63 mmol). The crude product was purified using column chromatography (2-10% MeOH:DCM) to yield the title diamine as a foamy white solid (238 mg, 0.41 mmol, 65%). **M.P.** 140-141 °C ($CHCl_3$/PE). **TLC** – $R_f$ = 0.26 ($SiO_2$, 5:95 MeOH:DCM). **$^1$H NMR** (500 MHz, $CDCl_3$) $\delta_H$ 2.82 (t, J = 4.5, 2H, NC*H*$_2$), 3.25 (t, J = 7.8, 2H, NC*H*$_2$), 3.29 (t, J = 4.5, 2H, NC*H*$_2$), 3.47 (d, J = 7.8, 2H, NC*H*$_2$), 3.77 (s, 3H, OC*H*$_3$), 3.77 (s, 3H, OC*H*$_3$), 3.82 (s, 2H, C*H*$_2$Ar), 4.62 (s, 2H, C*H*$_2$Ar), 6.76 (d, J = 8.8, 2H, 2 x Ar*H*), 6.80 (d, J = 9.0, 2H, 2 x Ar*H*), 7.15 (d, J = 9.0, 2H, 2 x Ar*H*), 7.24-7.27 (m, 2H, 2 x Ar*H*), 7.27-7.35 (m, 8H, 8 x Ar*H*), 7.52 (d, J = 8.8, 2H, 2 x Ar*H*), 8.36 (s, 1H, N*H*), 9.81 (s, 1H, N*H*). **$^{13}$C NMR** (126 MHz, $CDCl_3$) $\delta_C$ 45.3 (N*C*H$_2$), 48.0 (N*C*H$_2$), 49.4 (N*C*H$_2$), 51.0 (N*C*H$_2$), 51.1 (*C*H$_2$Ar), 54.4 (*C*H$_2$Ar), 55.7 (2 x O*C*H$_3$), 114.0 (2 x Ar*C*), 114.1 (2 x Ar*C*), 121.0 (2 x Ar*C*), 121.5 (2 x Ar*C*), 127.6 (Ar*C*), 127.7 (Ar*C*), 127.9 (2 x Ar*C*), 128.5 (2 x Ar*C*), 128.8 (2 x Ar*C*), 128.9 (2 x Ar*C*), 133.1 (Ar*C*), 133.5 (Ar*C*), 138.6 (Ar*C*), 138.9 (Ar*C*), 155.3 (2 x Ar*C*), 156.3 (*C*O), 158.3 (*C*O). **HR-MS** (ESI, positive ion mode) – *m/z* for $[C_{34}H_{39}N_5O_4+H]^+$ = 582.3080. Found 582.3101. **FTIR** (neat) – 2968, 2905, 1653, 1648.

**1,7-Dibenzyl-1,4-bis(4-methoxyanilinylcarbonyl)-7-butylaminocarbonyl-1,4,7-triazaheptane, 3e**

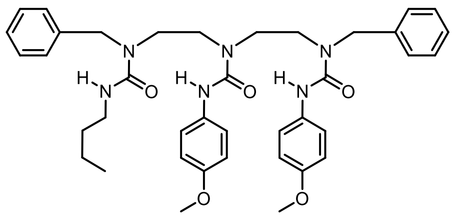

Synthesised according to general procedure A using **3e-4** (60 mg, 0.10 mmol) and *n*-butyl isocyanate (0.02 mL, 0.15 mmol). The crude product was purified using column chromatography (2-5% MeOH:DCM) to yield the title triurea as a foamy white solid (38 mg, 0.06 mmol, 56%). **M.P.** 171-172 °C ($Et_2O$/PE). **TLC** – $R_f$ = 0.45 ($SiO_2$, EtOAc). **$^1$H NMR** (400 MHz, $CDCl_3$) $\delta_H$ 0.85 (t, J = 7.8, 3H, C*H*$_3$), 1.20-1.22 (m, 2H, C*H*$_2$), 1.37-1.40 (m, 2H, C*H*$_2$), 3.16-3.47 (m, 10H, 5 x NC*H*$_2$), 3.76 (s, 3H, OC*H*$_3$), 3.78 (s, 3H, OC*H*$_3$), 4.38 (s, 2H, C*H*$_2$Ar), 4.57 (s, 2H, C*H*$_2$Ar), 6.81 (d, J = 9.0, 2H, 2 x Ar*H*), 6.84 (d, J = 9.0, 2H, 2 x Ar*H*), 7.12 (d, J = 7.5, 2H, 2 x Ar*H*), 7.18-7.33 (m, 10H, 10 x Ar*H*), 7.38 (s, 1H, N*H*), 7.53 (d, J = 8.9, 2H, 2 x Ar*H*), 8.84 (s, 1H, N*H*). **$^{13}$C NMR** (101 MHz, $CDCl_3$) $\delta_C$ 13.9 (*C*H$_3$), 20.0 (*C*H$_2$), 32.2 (*C*H$_2$), 40.7 (N*C*H$_2$), 46.7 (N*C*H$_2$), 47.6 (N*C*H$_2$), 48.4 (N*C*H$_2$), 49.9 (N*C*H$_2$), 51.9 (*C*H$_2$Ar), 52.7 (*C*H$_2$Ar), 55.7 (2 x O*C*H$_3$), 114.0 (4 x Ar*C*), 121.1 (2 x Ar*C*), 121.6 (2 x Ar*C*), 126.6 (2 x Ar*C*), 127.6 (2 x Ar*C*), 128.0 (2 x Ar*C*), 128.9 (2 x Ar*C*), 129.2 (2 x Ar*C*), 133.0 (Ar*C*), 133.4 (Ar*C*), 137.7 (Ar*C*), 138.2 (Ar*C*), 155.3 (Ar*C*), 155.4 (Ar*C*), 156.4 (*C*O), 156.6 (*C*O), 158.9 (*C*O). **HR-MS** (ESI, positive ion mode) – *m/z* for $[C_{39}H_{48}N_6O_5+Na]^+$ = 703.3584. Found 703.3575. **FTIR** (neat) – 2960, 2932, 1644, 1607.

**4,7-Bis(*tert*-butoxycarbonyl)-1,4,7,10-tetraazadecane, 3f-1**

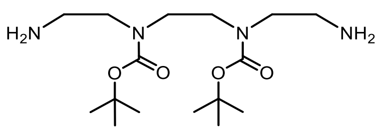

Synthesised according to general procedure F using triethylenetetramine (2.6 mL, 17.1 mmol). The crude product was then reacted according to general procedure D. The title carbamate was isolated as a pale yellow oil (4.1 g, 11.9 mmol, 67%). **TLC** – $R_f$ = 0.04 ($SiO_2$, 5:95:1 MeOH:DCM:$NH_3$). Spectroscopic data matched that previously reported.[3]

**1-(Naphthalen-1-ylmethyl)-4,7-bis(*tert*-butoxycarbonyl)-1,4,7,10-tetraazadecane, 3f-2**

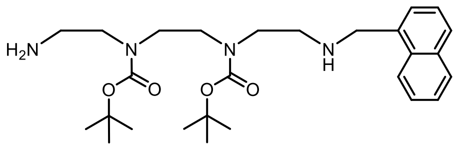

Synthesised according to general procedure J using **3f-1** (4.20 g, 12.10 mmol) and 1-naphthaldehyde (1.68 mL, 12.10 mmol, 1.00 equiv). The crude product was purified by column chromatography (1-10% MeOH:DCM, 1% $NH_4OH$) to yield the title amine as a foamy white solid (2.90 g, 6.0 mmol, 50%). **M.P.** 42-43 °C ($CHCl_3$). **TLC** – $R_f$ = 0.09 ($SiO_2$, 5:95:1 MeOH:DCM:$NH_4OH$). **$^1$H NMR** (400 MHz, $CDCl_3$) $\delta_H$ 1.43 (s, 9H, C(C$H_3$)$_3$), 1.45 (s, 9H, C(C$H_3$)$_3$), 2.78 (t, J = 6.2, 2H, NC$H_2$), 2.88 (t, J = 6.2, 2H, NC$H_2$), 3.17-3.46 (m, 8H, 4 x NC$H_2$), 4.24 (s, 2H, C$H_2$Ar), 7.37-7.53 (m, 4H, 4 x Ar*H*), 7.75 (d, J = 8.1, 1H, Ar*H*), 7.84 (d, J = 7.5, 1H, Ar*H*), 8.11-8.14 (m, 1H, Ar*H*). **$^{13}$C NMR** (101 MHz, $CDCl_3$) $\delta_C$ 28.5 (2 x C(*C*$H_3$)$_3$), 40.6 (N*C*$H_2$, rot.), 40.9 (N*C*$H_2$, rot.), 45.7 (N*C*$H_2$), 46.0 (N*C*$H_2$), 47.7 (N*C*$H_2$), 48.4 (N*C*$H_2$), 51.2 (*C*$H_2$Ar), 51.6 (N*C*$H_2$), 79.5 (*C*($CH_3$)$_3$), 79.9 (*C*($CH_3$)$_3$), 123.8 (Ar*C*), 125.3 (Ar*C*), 125.6 (Ar*C*), 126.0 (2 x Ar*C*), 127.7 (Ar*C*), 128.6 (Ar*C*), 131.8 (Ar*C*), 133.9 (Ar*C*), 135.9 (Ar*C*), 155.4 (*C*O), 155.7 (*C*O). **HR-MS** (ESI, positive ion mode) – *m/z* for $[C_{27}H_{43}N_4O_4+H]^+$ = 487.3284. Found 487.3279. **FTIR** (neat) – 3401, 2946, 1687.

**1-(Naphthalen-1-ylmethyl)-1,4,7-tris(4-methoxyanilinylcarbonyl)-10-trifluoroacetyl-1,4,7,10-tetraazadecane, 3f-3**

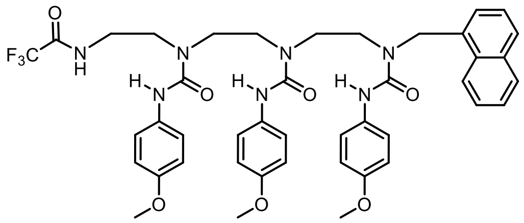

***Step 1*** (*trifluoroacetylation*): Under a dry, inert atmosphere, a solution of **3f-2** (1.5 g, 5.4 mmol, 1.00 equiv, 1.00 M) in anhydrous DCM (5.4 mL) was cooled to 0 °C. Ethyl trifluoroacetate (0.70 mL, 5.9 mmol, 1.10 equiv) was added dropwise over 30 minutes and the resulting solution was stirred for 30 minutes. The solution was warmed to room temperature and stirred for a further hour. The solution was then diluted with saturated aqueous $NaHCO_3$ (50 mL) and DCM (50 mL). The organic phase was washed with deionised water, dried ($MgSO_4$), filtered and concentrated *in vacuo* to give the crude trifluoroacetamide. ***Step 2*** (*Boc-deprotection*): Under a dry, inert atmosphere, the crude product was dissolved in anhydrous DCM (43 mL) and TFA (11 mL) was added dropwise.

The resulting solution was stirred for 16 h and concentrated *in vacuo*. The crude product was diluted with saturated aqueous $NaHCO_3$ (100 mL) and extracted with DCM (3 x 50 mL). The combined organic extracts were dried ($MgSO_4$), filtered and concentrated *in vacuo* to give the crude triamine. ***Step 3*** (*urea formation*): Under a dry, inert atmosphere, the residue was dissolved in anhydrous DCM (55 mL) and cooled to 0 °C. Anhydrous TEA (6.7 mL, 48.6 mmol, 9.00 equiv) was added in one portion and 4-methoxyphenyl isocyanate (3.2 mL, 24.3 mmol, 4.50 equiv) was added dropwise to give a colourless solution. The solution was stirred for 16 h then concentrated *in vacuo* and purified by column chromatography (1-10% MeOH:DCM) to yield the title triurea as a foamy white solid (403 mg, 0.5 mmol, 9%). **M.P.** 140-141 °C ($CHCl_3$). **TLC** – $R_f$ = 0.19 ($SiO_2$, 5:95 MeOH:DCM). **$^1$H NMR** (400 MHz, $CDCl_3$) $\delta_H$ 2.89-3.57 (m, 12H, 6 x NC*H*$_2$), 3.70 (s, 3H, OC*H*$_3$), 3.71 (s, 3H, OC*H*$_3$), 3.74 (s, 3H, OC*H*$_3$), 5.04 (s, 2H, C*H*$_2$Ar), 5.75 (s, 1H, N*H*), 6.66-6.86 (m, 6H, 6 x Ar*H*), 7.03 (d, J = 9.0, 2H, 2 x Ar*H*), 7.35-7.60 (m, 8H, 8 x Ar*H*), 7.76 (d, J = 7.7, 1H, Ar*H*), 7.81 (d, J = 7.7, 1H, Ar*H*), 8.00 (d, J = 7.7, 1H, Ar*H*), 8.57 (s, 1H, N*H*), 8.88 (s, 1H, N*H*). **$^{13}$C NMR** (101 MHz, $CDCl_3$) $\delta_C$ 39.7 (N*C*H$_2$), 45.8 (N*C*H$_2$), 46.3 (N*C*H$_2$), 47.5 (N*C*H$_2$), 47.8 (N*C*H$_2$), 48.4 (N*C*H$_2$), 49.7 (*C*H$_2$Ar), 55.5 (3 x O*C*H$_3$), 114.1 (2 x Ar*C*), 114.2 (2 x Ar*C*), 114.4 (2 x Ar*C*), 117.6 (q, J = 287.2, *C*F$_3$), 121.3 (2 x Ar*C*), 121.7 (2 x Ar*C*), 122.3 (2 x Ar*C*), 125.4 (Ar*C*), 126.3 (Ar*C*), 126.8 (Ar*C*), 128.3 (Ar*C*), 128.6 (Ar*C*), 128.9 (Ar*C*), 129.3 (Ar*C*), 131.3 (Ar*C*), 132.7 (Ar*C*), 132.9 (Ar*C*), 133.1 (Ar*C*), 133.2 (Ar*C*), 133.9 (Ar*C*), 155.3 (Ar*C*), 155.4 (Ar*C*), 155.6 (Ar*C*), 156.2 (q, J = 36.2, *C*O), 156.7 (*C*O), 157.0 (*C*O). **$^{19}$F NMR** (377 MHz, $CDCl_3$) $\delta_F$ −72.3 (C*F*$_3$). **HR-MS** (MALDI) – *m/z* for $[C_{43}H_{46}F_3N_7O_7+Na]^+$ = 852.3309. Found 852.3288. **FTIR** (neat) – 3309, 2976, 2923, 1648.

**1-(Naphthalen-1-ylmethyl)-1,4,7-tris(4-methoxyanilinylcarbonyl)-1,4,7,10-tetraazadecane, 3f-4**

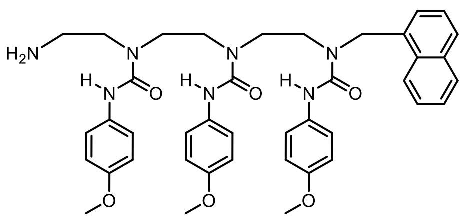


Synthesised according to general procedure D using **3f-3** (400 mg, 0.49 mmol). The title amine was isolated as a foamy white solid (342 mg, 0.47 mmol, 95%). **M.P.** 133-134 °C ($CHCl_3$). **TLC** – $R_f$ = 0.30 ($SiO_2$, 5:95 MeOH:DCM). **$^1$H NMR** (400 MHz, $CDCl_3$) $\delta_H$ 1.88 (s, 2H, N*H*$_2$), 2.80 (t, J = 7.0, 2H, NC*H*$_2$), 3.01-3.26 (m, 8H, 4 x NC*H*$_2$), 3.45 (t, J = 6.3, 2H, NC*H*$_2$), 3.70 (s, 3H, OC*H*$_3$), 3.73 (s, 3H, OC*H*$_3$), 3.75 (s, 3H, OC*H*$_3$), 5.05 (s, 2H, C*H*$_2$Ar), 5.97 (s, 1H, N*H*), 6.75 (d, J = 8.9, 2H, 2 x Ar*H*), 6.77 (d, J = 8.9, 2H, 2 x Ar*H*), 6.80 (d, J = 8.9, 2H, 2 x Ar*H*), 7.03 (d, J = 8.9, 2H, 2 x Ar*H*), 7.34-7.47 (m, 4H, 4 x Ar*H*), 7.50 (d, J = 8.9, 2H, 2 x Ar*H*), 7.59 (d, J = 8.9, 2H, 2 x Ar*H*), 7.75 (d, J = 7.6, 1H, Ar*H*), 7.80 (d, J = 7.6, 1H, Ar*H*), 8.00 (d, J = 7.6, 1H, Ar*H*), 8.67 (s, 1H, N*H*), 9.01 (s, 1H, N*H*). **$^{13}$C NMR** (101 MHz, $CDCl_3$) $\delta_C$ 39.3 (N*C*H$_2$), 46.9 (N*C*H$_2$), 47.3 (N*C*H$_2$), 47.0 (N*C*H$_2$), 48.1 (N*C*H$_2$), 49.2 (N*C*H$_2$), 49.3 (*C*H$_2$Ar), 55.4 (O*C*H$_3$), 55.5 (O*C*H$_3$), 55.5 (O*C*H$_3$), 113.9 (2 x Ar*C*), 113.9 (2 x Ar*C*), 114.0 (2 x Ar*C*), 121.1 (2 x Ar*C*), 121.5 (2 x Ar*C*), 121.6 (2 x Ar*C*), 125.4 (Ar*C*), 125.5 (Ar*C*), 126.2

(Ar*C*), 126.7 (Ar*C*), 128.3 (Ar*C*), 128.4 (Ar*C*), 128.8 (Ar*C*), 131.2 (Ar*C*), 131.6 (Ar*C*), 131.8 (Ar*C*), 132.9 (Ar*C*), 133.1 (Ar*C*), 133.8 (Ar*C*), 155.2 (Ar*C*), 155.3 (Ar*C*), 155.6 (Ar*C*), 156.6 (*C*O), 156.6 (*C*O), 157.4 (*C*O). **HR-MS** (ESI, positive ion mode) – *m/z* for $[C_{41}H_{47}N_7O_6+H]^+$ = 734.3666. Found 734.3659. **FTIR** (neat) – 3307, 3000, 2948, 1647.

**1-(Naphthalen-1-ylmethyl)-1,4,7-tris(4-methoxyanilinylcarbonyl)-7-(succinimidylethyl)-1,4,7-triazaheptane, 3f**

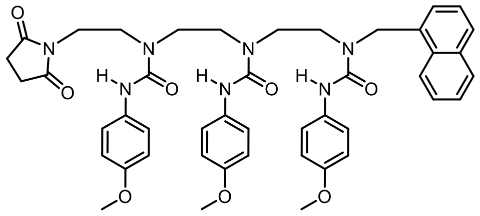

Synthesised according to general procedure E using **3f-4** (75 mg, 0.10 mmol). The crude product was purified by column chromatography (1-10% MeOH:DCM) to give the title succinimide as a foamy white solid (67 mg, 0.08 mmol, 80%). **M.P.** 152-153 °C ($CHCl_3$). **TLC** – $R_f$ = 0.50 ($SiO_2$, 5:95 MeOH:DCM). **$^1$H NMR** (500 MHz, $CDCl_3$) $\delta_H$ 2.74 (s, 4H, 2 x C(O)C*H*$_2$), 3.14-3.19 (m, 2H, NC*H*$_2$), 3.24-3.29 (m, 4H, 2 x NC*H*$_2$), 3.39 (t, J = 6.4, 2H, NC*H*$_2$), 3.53 (t, J = 7.3, 2H, NC*H*$_2$), 3.68 (t, J = 7.0, 2H, NC*H*$_2$), 3.80-3.81 (m, 9H, 3 x OC*H*$_3$), 5.13 (s, 2H, C*H*$_2$Ar), 6.82-6.86 (m, 6H, 6 x Ar*H*), 7.47-7.63 (m, 10H, 10 x Ar*H*), 7.90 (d, J = 8.3, 1H, Ar*H*), 7.95 (d, J = 8.0, 1H, Ar*H*), 8.11-8.13 (m, 1H, Ar*H*), 8.69 (s, 1H, N*H*). **$^{13}$C NMR** (126 MHz, $CDCl_3$) $\delta_C$ 28.2 (2 x C(O)*C*H$_2$), 37.0 (N*C*H$_2$), 45.2 (N*C*H$_2$), 47.1 (N*C*H$_2$), 47.3 (N*C*H$_2$), 47.7 (N*C*H$_2$), 47.8 (N*C*H$_2$), 49.4 (*C*H$_2$Ar), 55.5 (O*C*H$_3$), 55.5 (O*C*H$_3$), 55.5 (O*C*H$_3$), 113.9 (2 x Ar*C*), 113.9 (2 x Ar*C*), 114.0 (2 x Ar*C*), 121.0 (2 x Ar*C*), 121.1 (2 x Ar*C*), 121.7 (2 x Ar*C*), 125.4 (2 x Ar*C*), 126.3 (2 x Ar*C*), 126.9 (3 x Ar*C*), 128.6 (Ar*C*), 128.8 (Ar*C*), 128.9 (Ar*C*), 133.0 (Ar*C*), 133.1 (Ar*C*), 133.9 (Ar*C*), 155.2 (Ar*C*), 155.5 (Ar*C*), 155.5 (Ar*C*), 155.8 (*C*O), 156.2 (*C*O), 156.3 (*C*O), 177.6 (2 x *C*O). **HR-MS** (ESI, positive ion mode) – *m/z* for $[C_{45}H_{49}N_7O_8+Na]^+$ = 838.3540. Found 838.3533. **FTIR** (neat) – 3322, 2997, 2908, 1699, 1651.

***N*-(2-Pyridyl)ethylenediamine, 3g-1**

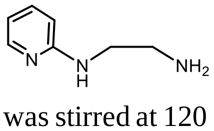

Under a dry, inert atmosphere, 2-fluoropyridine (2.0 mL, 23.4 mmol, 1.00 equiv, 1.0 M) was added to freshly distilled ethylenediamine (23.4 mL) and the solution was stirred at 120 °C for 16 h. Upon cooling, the solution was diluted with EtOAc (100 mL) and washed with distilled water (5 x 50 mL). The organic layer was washed with brine, dried ($MgSO_4$), filtered and concentrated *in vacuo* to give the title diamine as yellow powder (2664 mg, 19.4 mmol, 83%). Spectroscopic data matched that previously reported.[4]

**1-(2-Pyridyl)-7-(*tert*-butoxycarbonyl)-1,4,7-triazaheptane, 3g-2**

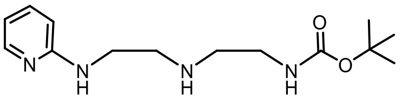

To a solution of **3g-1** (918.0 mg, 6.69 mmol, 1.0 equiv) in lab grade 1,2-dichloroethane (20.0 mL) at room temperature under air was added solid *N*-Boc-2-aminoacetaldehyde (1.065 g, 6.69 mmol, 1.0 equiv). A further aliquot of 1,2-dichloroethane (2.3 mL) was used to rinse residual *N*-Boc-2-aminoacetaldehyde into the reaction vessel. Solid $NaBH(OAc)_3$ (1.986 g, 9.37 mmol, 1.4 equiv) was added and the suspension was stirred at room temperature for 20 h. Aqueous NaOH (10 mL, 2.0 M), water (20 mL) and brine (10 mL) were added and the organic layer was separated. The product was further extracted with $CH_2Cl_2$ (2 × 25 mL) then the combined organic extracts were washed with saturated $NaHCO_3$/brine (1:1, 50 mL), dried ($Na_2SO_4$) and concentrated. Flash chromatography (manual, 35.8 g silica, MeOH/[35% aqueous $NH_3$]/EtOAc* gradient from 5:1:94 to 7.5:1:91.5) gave **3g-2** (837.9 mg, 45%) as a pale yellow gum that eventually solidified upon storage. [*This eluent combination should be prepared freshly before chromatography. Solutions stored for long periods (weeks/months) were found to contain non-volatile acetamide as an impurity.] **M.P.** 72-73 °C ($CHCl_3$). **TLC** – $R_f$ = 0.09 ($SiO_2$, 5:95 MeOH:DCM). **$^1$H NMR** (400 MHz, $CDCl_3$) $\delta_H$ 1.44 (s, 9H, C(C$H_3$)$_3$), 2.76 (t, J = 5.8, 2H, NC$H_2$), 2.88 (t, J = 5.8, 2H, NC$H_2$), 3.22 (q, J = 5.8, 2H, NC$H_2$), 3.34 (q, J = 5.8, 2H, NC$H_2$), 4.85 (s, 1H, N*H*), 4.92 (s, 1H, N*H*), 6.41 (d, J = 8.5, 1H, Ar*H*), 6.56 (t, J = 6.0, 1H, Ar*H*), 7.40 (t, J = 7.6, 1H, Ar*H*), 8.08 (d, J = 4.9, 1H, Ar*H*). **$^{13}$C NMR** (101 MHz, $CDCl_3$) $\delta_C$ 28.6 (C(*C*H$_3$)$_3$), 40.5 (N*C*H$_2$), 41.9 (N*C*H$_2$), 48.7 (N*C*H$_2$), 49.1 (N*C*H$_2$), 79.2 (*C*(CH$_3$)$_3$), 107.4 (Ar*C*), 113.0 (Ar*C*), 137.5 (Ar*C*), 148.3 (Ar*C*), 156.5 (*C*O), 158.5 (Ar*C*). **HR-MS** (ESI, positive ion mode) – *m/z* for $[C_{14}H_{26}N_4O+Na]^+$ = 303.1797. Found 303.1807. **FTIR** (neat) – 3356, 2991, 2894, 1695.

**1-(2-Pyridyl)-1,4-bis(4-methoxyanilinylcarbonyl)-7-(*tert*-butoxycarbonyl)-1,4,7-triazaheptane, 3g-3**

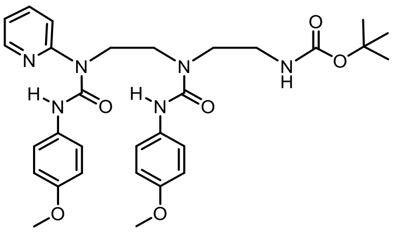

Synthesised according to general procedure A using **3g-2** (163 mg, 0.58 mmol) and 4-methoxyphenyl isocyanate (0.23 mL, 1.74 mmol). The crude product was purified by column chromatography (12-100% EtOAc:PE) to yield the title diurea as a foamy white solid (114 mg, 0.20 mmol, 34%). **M.P.** 119-120 °C ($CHCl_3$). **TLC** – $R_f$ = 0.48 ($SiO_2$, 5:95 MeOH:DCM). **$^1$H NMR** (500 MHz, $CD_2Cl_2$) $\delta_H$ 1.44 (s, 9H, C(C$H_3$)$_3$), 3.32 (q, J = 6.3, 2H, NC$H_2$), 3.54 (t, J = 6.6, 2H, NC$H_2$), 3.64 (t, J = 6.6, 2H, NC$H_2$), 3.78-3.79 (m, 6H, 2 x OC$H_3$), 4.10 (t, J = 6.3, 2H, NC$H_2$), 5.31 (t, J = 5.3, 1H, N*H*), 6.86 (d, J = 9.0, 2H, 2 x Ar*H*), 6.89 (d, J = 9.0, 2H, 2 x Ar*H*), 7.04 (dd, J = 5.1, 7.2, 1H, Ar*H*), 7.42 (s, 1H, Ar*H*), 7.50 (d, J = 9.0, 2H, 2 x Ar*H*), 7.56 (d, J = 9.0, 2H, 2 x Ar*H*), 7.82 (ddd, J = 8.0, 7.5, 1.9, 1H, Ar*H*), 8.34 (dd, J = 4.9, 1.5, 1H, Ar*H*), 8.68 (s, 1H, N*H*), 12.48 (s, 1H, N*H*). **$^{13}$C NMR** (126 MHz, $CD_2Cl_2$) $\delta_C$ 28.7 (C(*C*H$_3$)$_3$), 41.0 (N*C*H$_2$), 45.2

(N$CH_2$), 46.7 (N$CH_2$), 48.3 (N$CH_2$), 56.0 (2 x O$CH_3$), 80.0 ($C(CH_3)_3$), 113.0 (Ar*C*), 114.3-114.5 (4 x Ar*C*), 118.4 (Ar*C*), 121.8 (2 x Ar*C*), 122.6 (2 x Ar*C*), 132.5 (Ar*C*), 134.1 (Ar*C*), 140.0 (Ar*C*), 146.4 (Ar*C*), 154.9 (Ar*C*), 155.8 (Ar*C*), 155.9 (*C*O), 156.5 (*C*O), 156.8 (*C*O). **HR-MS** (ESI, positive ion mode) – *m/z* for $[C_{30}H_{38}N_6O_6+H]^+$ = 579.2931. Found 579.2895. **FTIR** (neat) – 3497, 2990, 2943, 1694, 1660.

**1-(2-Pyridyl)-1,4-bis(4-methoxyanilinylcarbonyl)-1,4,7-triazaheptane, 3g-4**

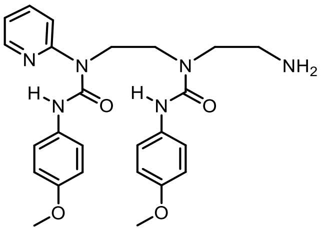


Synthesised according to general procedure H using **3g-3** (200 mg, 0.34 mmol). The title amine was isolated as a foamy white solid (163 mg, 0.34 mmol, >99%). **M.P.** 139-140 °C ($CHCl_3$). **TLC** – $R_f$ = 0.09 ($SiO_2$, 90:10 EtOAc:PE). **$^1$H NMR** (400 MHz, $CDCl_3$) $\delta_H$ 3.00 (t, J = 4.9, 2H, NC$H_2$), 3.51 (t, J = 4.9, 2H, NC$H_2$), 3.61 (t, J = 7.2, 2H, NC$H_2$), 3.77 (s, 3H, OC$H_3$), 3.79 (s, 3H, OC$H_3$), 4.17 (t, J = 7.2, 2H, NC$H_2$), 6.84 (d, J = 8.9, 2H, 2 x Ar*H*), 6.88 (d, J = 8.9, 2H, 2 x Ar*H*), 6.98 (dd, J = 5.0, 6.8, 1H, Ar*H*), 7.35 (d, J = 8.9, 2H, NC$H_2$), 7.45 (d, J = 8.9, 2H, NC$H_2$), 7.67 (d, J = 7.7, 1H, Ar*H*), 7.78 (ddd, J = 1.8, 5.8, 7.7, 1H, Ar*H*), 8.28 (d, J = 5.0, 1H, Ar*H*), 9.75 (s, 1H, N*H*), 12.61 (s, 1H, N*H*). **$^{13}$C NMR** (101 MHz, $CDCl_3$) $\delta_C$ 41.8 (N$CH_2$), 43.6 (N$CH_2$), 46.1 (N$CH_2$), 52.1 (N$CH_2$), 55.5 (2 x O$CH_3$), 112.7 (Ar*C*), 114.1 (4 x Ar*C*), 117.5 (Ar*C*), 121.3 (2 x Ar*C*), 122.5 (2 x Ar*C*), 131.9 (Ar*C*), 133.4 (Ar*C*), 139.6 (Ar*C*), 145.3 (Ar*C*), 154.4 (*C*O), 155.1 (Ar*C*), 155.4 (Ar*C*), 158.0 (Ar*C*). **HR-MS** (ESI, positive ion mode) – *m/z* for $[C_{25}H_{30}N_6O_4+H]^+$ = 479.2407. Found 479.2380. **FTIR** (neat) – 3329, 3130, 2992, 2947, 1659.

**1-(2-Pyridyl)-1,4-bis(4-methoxyanilinylcarbonyl)-7-benzyl-1,4,7-triazaheptane, 3g-5**

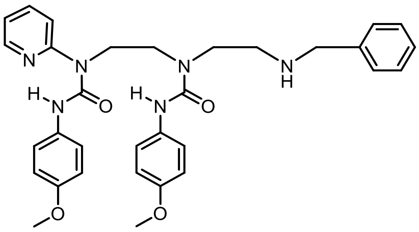


Synthesised according to general procedure B using **3g-4** (140 mg, 0.29 mmol) and benzaldehyde (0.05 mL, 0.44 mmol). The crude product was purified by column chromatography (3-10% MeOH:DCM) to yield the title triamine as a foamy white solid (87 mg, 0.15 mmol, 53%). **M.P.** 142-143 °C ($CHCl_3$). **TLC** – $R_f$ = 0.48 ($SiO_2$, 10:90 MeOH:DCM). **$^1$H NMR** (500 MHz, $CDCl_3$) $\delta_H$ 2.03 (s, 1H, N*H*), 2.95 (t, J = 4.9, 2H, NC$H_2$), 3.56 (t, J = 4.9, 2H, NC$H_2$), 3.60 (t, J = 8.2, 2H, NC$H_2$), 3.78 (s, 3H, OC$H_3$), 3.80 (s, 3H, OC$H_3$), 3.84 (s, 2H, C$H_2$Ar), 4.15 (t, J = 8.2, 2H, NC$H_2$), 6.80 (d, J = 9.0, 2H, 2 x Ar*H*), 6.88 (d, J = 9.0, 2H, 2 x Ar*H*), 6.98 (dd, J = 5.0, 7.2, 1H, Ar*H*), 7.22 (d, J = 9.0 2H, 2 x Ar*H*), 7.25-7.34 (m, 5H, 5 x Ar*H*), 7.46 (d, J = 9.0, 2H, 2 x Ar*H*), 7.66-7.70 (m, 1H, Ar*H*), 7.74 (ddd, J = 1.8, 7.4, 8.6, 1H, Ar*H*), 8.27 (d, J = 5.0, 1H, Ar*H*), 9.70 (s, 1H, N*H*), 12.61 (s, 1H, N*H*). **$^{13}$C NMR** (126 MHz, $CDCl_3$) $\delta_C$ 43.5 (N$CH_2$), 46.2 (N$CH_2$), 49.6 (N$CH_2$), 50.0 (N$CH_2$), 54.3 ($CH_2$Ar), 55.5 (2 x O$CH_3$), 112.8 (Ar*C*), 114.0

(2 x Ar*C*), 114.1 (2 x Ar*C*) 117.4 (Ar*C*), 121.5 (2 x Ar*C*), 122.4 (2 x Ar*C*), 127.5 (Ar*C*), 128.3 (2 x Ar*C*), 128.7 (2 x Ar*C*), 132.0 (Ar*C*), 133.3 (Ar*C*), 138.9 (Ar*C*), 139.6 (Ar*C*), 145.3 (Ar*C*), 154.3 (Ar*C*), 155.1 (Ar*C*), 155.4 (*C*O), 155.9 (Ar*C*), 158.0 (*C*O). **HR-MS** (ESI, positive ion mode) – *m/z* for $[C_{32}H_{36}N_6O_4+H]^+$ = 569.2876. Found 569.2875. **FTIR** (neat) – 3326, 2993, 2950, 1652.

**1-(2-Pyridyl)-1,4,7-tris(4-methoxyanilinylcarbonyl)-7-benzyl-1,4,7-triazaheptane, 3g**

Synthesised according to general procedure A using **3g-5** (62 mg, 0.11 mmol) and 4-methoxyphenyl isocyanate (0.02 mL, 0.17 mmol) dropwise. The crude product was purified by column chromatography (1-2% MeOH:DCM) to yield the title triurea as a foamy white solid (47 mg, 0.07 mmol, 60%). **M.P.** 200-201 °C (DCM). **TLC** – $R_f$ = 0.26 ($SiO_2$, 1:99 MeOH:DCM). **$^1$H NMR** (500 MHz, $CDCl_3$) $\delta_H$ 3.32-3.37 (m, 2H, NC*H*$_2$), 3.51 (t, J = 8.0, 2H, NC*H*$_2$), 3.54 (t, J = 8.2, 2H, NC*H*$_2$), 3.76 (s, 3H, OC*H*$_3$), 3.78 (s, 3H, OC*H*$_3$), 3.79 (s, 3H, OC*H*$_3$), 3.98 (t, J = 8.0, 2H, NC*H*$_2$), 4.63 (s, 2H, C*H*$_2$Ar), 6.82 (d, J = 9.0, 2H, 2 x Ar*H*), 6.86 (d, J = 9.0, 2H, 2 x Ar*H*), 6.88 (d, J = 9.0, 2H, 2 x Ar*H*), 7.08 (dd, J = 5.0, 7.2, 1H, Ar*H*), 7.30-7.40 (m, 6H, 6 x Ar*H*), 7.48 (d, J = 9.0, 2H, 2 x Ar*H*), 7.51-7.53 (m, 2H, 2 x Ar*H*), 7.61 (d, J = 9.0, 2H, 2 x Ar*H*), 7.82 (ddd, J = 1.9, 7.4, 8.7, 1H, Ar*H*), 8.36 (dd, J = 1.6, 5.0, 1H, Ar*H*), 8.95 (s, 1H, N*H*), 12.34 (s, 1H, N*H*). **$^{13}$C NMR** (126 MHz, $CDCl_3$) $\delta_C$ 45.2 (N*C*H$_2$), 47.0 (2 x N*C*H$_2$), 48.5 (N*C*H$_2$), 51.2 (*C*H$_2$Ar), 55.4 (3 x O*C*H$_3$), 112.0 (Ar*C*), 113.7 (2 x Ar*C*), 113.8 (2 x Ar*C*), 114.0 (2 x Ar*C*), 118.1 (Ar*C*), 121.1 (2 x Ar*C*), 121.3 (2 x Ar*C*), 122.0 (2 x Ar*C*), 127.3 (Ar*C*), 127.6 (2 x Ar*C*), 128.7 (2 x Ar*C*), 131.7 (Ar*C*), 133.4 (2 x Ar*C*), 139.5 (2 x Ar*C*), 146.1 (Ar*C*), 154.4 (*C*O), 155.1 (Ar*C*), 155.2 (Ar*C*), 155.3 (Ar*C*), 156.0 (*C*O), 156.3 (*C*O). **HR-MS** (ESI, positive ion mode) – *m/z* for $[C_{40}H_{43}N_7O_6+H]^+$ = 718.3353. Found 718.3348. **FTIR** (neat) – 3307, 3185, 3051, 2981, 2875, 1660, 1607.

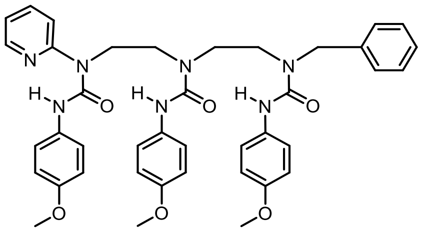


**1-(2-Pyridyl)-1,4-bis(4-bromoanilinylcarbonyl)-7-(*tert*-butoxycarbonyl)-1,4,7-triazanonane, 3h-1**

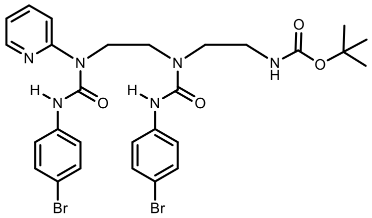


To a solution of **3g-2** (146.9 mg, 0.52 mmol, 1.0 equiv) in lab grade 1,2-dichloroethane (3.5 mL) at room temperature under air was added solid 4-bromophenyl isocyanate (212.7 mg, 1.07 mmol, 2.05 equiv). The mixture was heated to 40 °C (closed system: 7 mL vial with cap) and maintained at this temperature for 64 h. While still at 40 °C, MeOH (~2.5 mL) was added and the mixture was stirred for a further 20 min to quench any unreacted isocyanate. The mixture was then concentrated *in vacuo*. Flash chromatography (Biotage, 10 g SNAP Ultra column, MeOH/$CH_2Cl_2$ gradient from 0.2:99.8 to 2.5:97.5) gave **3h-1** (274.1 mg, 77%) as a white

solid. **TLC** – $R_f$ = 0.48 (2.5:97.5 MeOH/$CH_2Cl_2$). **$^1$H NMR** (400 MHz, $CDCl_3$) $\delta_H$ 1.45 (s, 9H), 3.32 – 3.38 (m, 2H), 3.56 (t, $J$ = 7.1 Hz, 2H), 3.62 – 3.69 (m, 2H), 4.07 – 4.12 (m, 2H), 5.07 (d, $J$ = 5.1 Hz, 1H), 7.05 (dd, $J$ = 7.4, 5.0 Hz, 1H), 7.38 – 7.49 (m, 7H), 7.61 (d, $J$ = 8.9 Hz, 2H), 7.83 (ddd, $J$ = 9.0, 7.3, 2.0 Hz, 1H), 8.32 (dd, $J$ = 5.1, 1.9 Hz, 1H), 8.85 (s, 1H), 12.90 (s, 1H). **$^{13}$C NMR** (101 MHz, $CDCl_3$) $\delta_C$ 28.5, 40.4, 44.5, 46.3, 47.8, 80.4, 112.6, 115.0, 116.1, 118.2, 121.2, 122.1, 131.7, 132.0, 138.0, 139.4, 140.0, 145.7, 154.1, 155.1, 155.9, 157.2. **HR-MS** (ESI, positive ion mode) – *m/z* for $[C_{28}H_{32}(^{79}Br)_2N_6O_4+H]^+$ = 675.0925. Found 675.0940.

**1-(2-Pyridyl)-1,4-bis(4-bromoanilinylcarbonyl)-1,4,7-triazanonane, 3h-2**

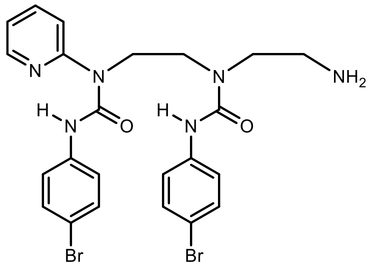


To a solution of **3h-1** (268.1 mg, 0.40 mmol, 1.0 equiv) in lab grade $CH_2Cl_2$ (2.0 mL) at room temperature under air was added TFA (0.46 mL, 5.95 mmol, 15.0 equiv) and the mixture was stirred at room temperature for 4 h. The mixture was diluted with $CH_2Cl_2$ (20 mL) and washed with aqueous $K_2CO_3$ (20 mL, 1.0 M). The aqueous phase was back-extracted with $CH_2Cl_2$ (15 mL). The combined organic extracts were dried ($Na_2SO_4$) and concentrated to give **3h-2** (214.6 mg, 94%) as a yellow solid. **TLC** – $R_f$ = 0.20 (10:90 MeOH/$CH_2Cl_2$). **$^1$H NMR** (400 MHz, $CDCl_3$) $\delta_H$ 1.69 (bs, 2H), 2.95 – 3.08 (m, 2H), 3.45 – 3.66 (m, 4H), 4.16 (t, $J$ = 7.9 Hz, 2H), 7.02 (t, $J$ = 6.3 Hz, 1H), 7.28 – 7.53 (m, 8H), 7.61 – 7.87 (m, 2H), 8.27 – 8.32 (m, 1H), 10.31 (bs, 1H), 12.94 (s, 1H). **$^{13}$C NMR** (101 MHz, $CDCl_3$) $\delta_C$ 41.9, 43.5, 46.2, 52.3, 112.9, 114.5, 115.8, 117.9, 120.9, 122.1, 131.7, 131.9, 138.2, 139.6, 139.9, 145.4, 154.0, 155.2, 157.6. **HR-MS** (ESI, positive ion mode) – *m/z* calcd for $[C_{23}H_{24}(^{79}Br)_2N_6O_2+H]^+$ = 575.0400. Found 575.0414.

**1-(2-Pyridyl)-1,4-bis(4-bromoanilinylcarbonyl)-7-(pyren-1-ylmethyl)-1,4,7-triazanonane, 3h-3**

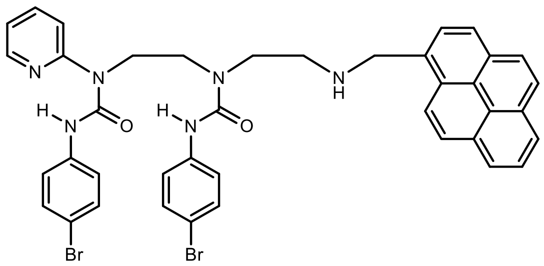


To a round-bottomed flask was added **3h-2** (39.9 mg, 0.069 mmol, 1.0 equiv) and 1-pyrenecarboxaldehyde (15.9 mg, 0.069 mmol, 1.0 equiv). After purging with $N_2$, lab grade 1,2-dichloroethane (1.4 mL) was added and the mixture was stirred at room temperature for 2 min until most solids had dissolved. The septum was briefly removed and solid $NaBH(OAc)_3$ (20.5 mg, 0.097 mmol, 1.4 equiv) was added. The mixture was stirred at room temperature for 5 h. Aqueous $K_2CO_3$ (10 mL, 1.0 M) was added and the product was extracted with $CH_2Cl_2$ (25 mL + 10 mL) then the combined organic extracts were dried ($Na_2SO_4$) and concentrated. Flash chromatography (Biotage, 5 g Zip Sphere column, MeOH/[35% aqueous

$NH_3$]/EtOAc* gradient from 0.6:0.12:99.28 to 5:1:94) gave **3h-3** (27.2 mg, 50%) as a white solid. [*This eluent combination should be prepared freshly before chromatography. Solutions stored for long periods (weeks/months) were found to contain non-volatile acetamide as an impurity.] **TLC** – $R_f$ = 0.33 (4:0.8:95.2 MeOH/[35% aqueous $NH_3$]/EtOAc). **$^1$H NMR** (400 MHz, $CDCl_3$) $\delta_H$ 3.13 (t, *J* = 4.5 Hz, 2H), 3.52 – 3.64 (m, 4H), 4.03 – 4.13 (m, 2H), 4.54 (s, 2H), 6.81 – 7.09 (m, 4H), 7.35 – 7.70 (broad m, 2H), 7.41 – 7.48 (m, 4H), 7.92 (d, *J* = 7.8 Hz, 1H), 7.98 – 8.29 (m, 10H), 9.89 (bs, 1H), 12.89 (s, 1H). **$^{13}$C NMR** (101 MHz, $CDCl_3$) δ (33 resolved resonances observed out of 36 theoretical) 43.7, 46.4, 50.4, 52.4, 112.7, 114.5, 115.9, 117.8, 121.0, 122.1, 122.7, 124.9, 125.2, 125.4, 125.6, 126.3, 127.4, 127.5, 127.7, 128.4, 129.1, 130.8, 131.2, 131.3, 131.4, 131.9, 132.4, 138.2, 139.2, 139.7, 145.3, 154.0, 155.1. **HR-MS** (MALDI, positive ion mode) – *m/z* for $[C_{40}H_{34}(^{79}Br)_2N_6O_2+H]^+$ = 789.1183. Found 789.1199.

**1-(2-Pyridyl)-1,4-bis(4-bromoanilinylcarbonyl)-7-(4-nitroanilinylcarbonyl)-7-(pyren-1-ylmethyl)-1,4,7-triazanonane, 3h**

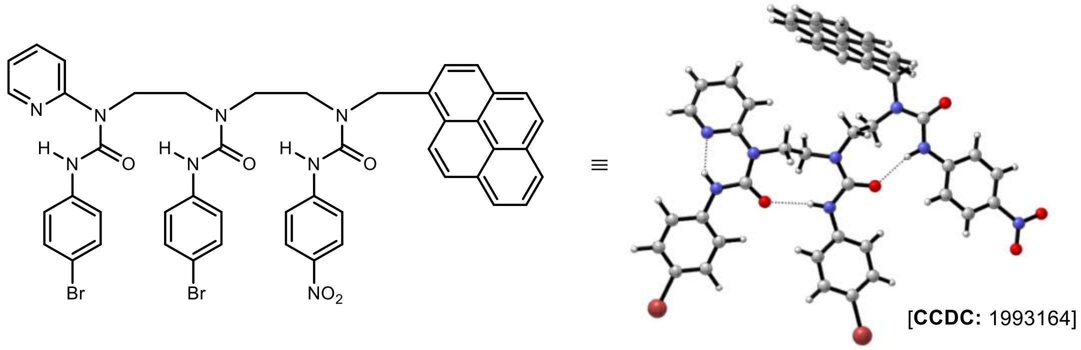


To a solution of **3h-3** (59.2 mg, 0.075 mmol, 1.0 equiv) in lab grade $CH_2Cl_2$ (0.5 mL) at room temperature under air was added a solution/suspension of 4-nitrophenyl isocyanate (12.3 mg, 0.075 mmol, 1.0 equiv) in $CH_2Cl_2$ (1.0 mL). The suspension was stirred at room temperature for 4.5 h, then stood in a freezer arbitrarily for 10 days. The precipitated product, which is sparingly soluble in $CH_2Cl_2$, was collected by filtration and the reaction vial and precipitate were rinsed/washed with $CH_2Cl_2$ (2 × 6 mL). The precipitate was air-dried in a fume hood to give **3h** (48.1 mg, 67%) as a pale yellow solid. **TLC** – $R_f$ = 0.31 ($CH_2Cl_2$) with tailing due to poor solubility. **$^1$H NMR** (400 MHz, DMF-$d_7$) $\delta_H$ 3.68 – 3.85 (m, 6H), 4.17 (t, *J* = 7.4 Hz, 2H), 5.56 (s, 2H), 7.19 (dd, *J* = 7.3, 5.0 Hz, 1H), 7.49 – 7.54 (m, 3H), 7.58 (d, *J* = 8.8 Hz, 2H), 7.64 (d, *J* = 8.9 Hz, 2H), 7.77 (d, *J* = 8.9 Hz, 2H), 7.85 – 7.90 (m, 1H), 8.04 – 8.15 (m, 4H), 8.20 – 8.39 (m, 8H), 8.43 – 8.49 (m, 2H), 9.25 (s, 1H), 9.87 (s, 1H), 12.06 (s, 1H). **$^{13}$C NMR** (126 MHz, DMF-$d_7$) $\delta_C$ (44 resolved resonances observed out of 41 theoretical; a minor unidentified aromatic impurity was present) 45.1, 45.9, 46.9, 47.5, 48.9, 114.2, 115.0, 115.0, 118.5, 119.0, 119.2, 121.6, 122.1, 123.2, 124.7, 125.0, 125.0, 125.3, 125.3, 125.7, 125.8, 126.7, 127.5, 127.9, 128.1, 128.8, 131.0, 131.1, 131.6, 131.8, 131.9, 139.2, 139.8, 140.4, 141.9, 142.3, 146.6, 147.0, 148.0, 152.5, 154.4, 155.2, 155.7, 156.3. **HR-MS** (MALDI, positive ion mode) – *m/z* for $[C_{47}H_{38}(^{79}Br)_2N_8O_5+Na]^+$ = 975.1224. Found 975.1239. Crystals of **3h** suitable for X-ray

crystallography were obtained by diffusion of toluene into a solution of **3h** in DMF/1,2-DCE (CCDC: 1993164).

**1,10-Bis(naphthalen-1-ylmethyl)-1,4,7-tris(4-methoxyanilinylcarbonyl)-1,4,7,10-tetraazadecane, 5a-1**

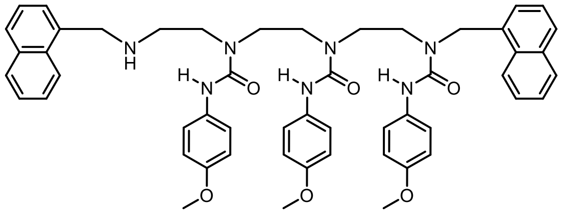

Synthesised according to general procedure B using **3f-4** (175 mg, 0.24 mmol) and 1-naphthaldehyde (0.05 mL, 0.36 mmol). The crude product was purified by column chromatography (2-10% MeOH:DCM) to give the title amine as a foamy white solid (132 mg, 0.15 mmol, 63%). **M.P.** 149-150 °C ($CHCl_3$). **TLC** – $R_f$ = 0.44 ($SiO_2$, 5:95 MeOH:DCM). **$^1$H NMR** (400 MHz, $CDCl_3$) $\delta_H$ 2.90 (t, J = 4.6, 2H, NC$H_2$), 2.94-2.98 (m, 2H, NC$H_2$), 3.08-3.17 (m, 4H, 2 x NC$H_2$), 3.26-3.29 (m, 2H, NC$H_2$), 3.45 (t, J = 7.6, 2H, NC$H_2$), 3.74 (s, 3H, OC$H_3$), 3.76 (s, 3H, OC$H_3$), 3.77 (s, 3H, OC$H_3$), 4.28 (s, 2H, C$H_2$Ar), 5.10 (s, 2H, C$H_2$Ar), 6.64 (d, J = 9.0, 2H, 2 x Ar*H*), 6.77 (d, J = 9.0, 2H, 2 x Ar*H*), 6.83 (d, J = 9.0, 2H, 2 x Ar*H*), 6.91 (d, J = 8.0, 2H, 2 x Ar*H*), 7.36-7.60 (m, 8H, 8 x Ar*H*), 7.77-7.83 (m, 2H, 2 x Ar*H*), 7.86-7.89 (m, 2H, 2 x Ar*H*), 8.00 (d, J = 8.0, 1H, Ar*H*), 8.13 (d, J = 8.0, 1H, Ar*H*), 8.48 (s, 1H, N*H*), 8.71 (s, 1H, N*H*), 9.59 (s, 1H, N*H*). **$^{13}$C NMR** (101 MHz, $CDCl_3$) $\delta_C$ 45.4 (N*C*$H_2$), 46.8 (N*C*$H_2$), 46.9 (N*C*$H_2$), 47.8 (N*C*$H_2$), 49.7 (N*C*$H_2$), 51.1 (N*C*$H_2$), 51.8 (*C*$H_2$Ar), 53.3 (*C*$H_2$Ar), 55.5 (3 x O*C*$H_3$), 113.8 (2 x Ar*C*), 113.9 (2 x Ar*C*), 114.0 (2 x Ar*C*), 120.9 (2 x Ar*C*), 121.0 (2 x Ar*C*), 121.5 (2 x Ar*C*), 123.1 (Ar*C*), 123.7 (Ar*C*), 125.3 (Ar*C*), 125.4 (Ar*C*), 126.0 (Ar*C*), 126.2 (Ar*C*), 126.6 (Ar*C*), 126.7 (Ar*C*), 128.4 (Ar*C*), 128.5 (Ar*C*), 128.6 (Ar*C*), 129.0 (Ar*C*), 131.5 (Ar*C*), 131.7 (Ar*C*), 132.6 (Ar*C*), 132.8 (Ar*C*), 133.1 (Ar*C*), 133.8 (Ar*C*), 134.0 (Ar*C*), 134.3 (Ar*C*), 155.1 (Ar*C*), 155.3 (Ar*C*), 156.0 (Ar*C*), 156.1 (*C*O), 156.4 (*C*O), 158.2 (*C*O). **HR-MS** (ESI, positive ion mode) – *m/z* for $[C_{52}H_{55}N_7O_6+Na]^+$ = 896.4112. Found 896.4092. **FTIR** (neat) – 3312, 2965, 2902, 1637.

**1,10-Bis(naphthalen-1-ylmethyl)-1,4,7-tris(4-methoxyanilinylcarbonyl)-10-(4-nitroanilinylthiocarbonyl)-1,4,7,10-tetraazadecane, 5a**

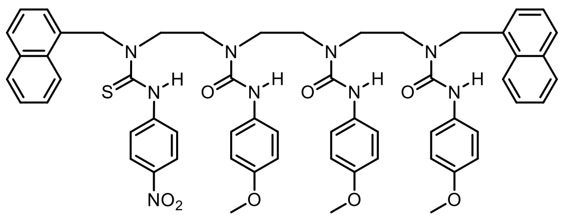

Synthesised according to general procedure A using **5a-1** (100 mg, 0.11 mmol) and 4-nitrophenyl isothiocyanate (31 mg, 0.17 mmol). The crude product was purified by column chromatography (1-2% MeOH:DCM) to yield the title thiourea as a yellow solid (116 mg, 0.11 mmol, >99%). **M.P.** 174-175 °C (DCM). **TLC** – $R_f$ = 0.32 ($SiO_2$, 1:99 MeOH:DCM). **$^1$H NMR** (500 MHz, $CDCl_3$) $\delta_H$ 2.89-3.71 (m, 12H, 2 x NC*H*$_2$), 3.79 (s, 3H, OC*H*$_3$), 3.79 (s, 3H, OC*H*$_3$), 3.81 (s, 3H, OC*H*$_3$), 5.04 (s, 2H, C*H*$_2$Ar), 5.67 (s, 2H, C*H*$_2$Ar), 6.38 (s, 1H, N*H*), 6.83 (d, J = 9.0, 2H, 2 x Ar*H*), 6.83 (d, J = 9.0, 2H, 2 x Ar*H*), 6.86 (d, J = 9.0, 2H, 2 x Ar*H*), 7.17-7.48 (m, 8H, 8 x Ar*H*), 7.51 (d, J = 9.0, 2H, 2 x Ar*H*), 7.60 (d, J = 9.0, 2H, 2 x Ar*H*), 7.64-8.18 (m, 12H, 12 x Ar*H*), 8.96 (s, 2H, 2 x N*H*), 10.44 (s, 1H, N*H*). **$^{13}$C NMR** (126 MHz, $CDCl_3$) $\delta_C$ 47.0-47.9 (4 x N*C*H$_2$), 48.6 (N*C*H$_2$), 48.9 (N*C*H$_2$), 51.1 (*C*H$_2$Ar), 54.1 (*C*H$_2$Ar), 55.5 (3 x O*C*H$_3$), 113.9 (2 x Ar*C*), 113.9 (2 x Ar*C*), 114.0 (2 x Ar*C*), 121.0 (6 x Ar*C*), 122.4 (Ar*C*), 123.2 (Ar*C*), 123.4 (Ar*C*), 123.8 (Ar*C*), 124.0 (Ar*C*), 125.3 (Ar*C*), 125.6 (Ar*C*), 126.4 (Ar*C*), 126.7 (Ar*C*), 127.0 (2 x Ar*C*), 127.2 (2 x Ar*C*), 128.5 (Ar*C*), 128.6 (Ar*C*), 128.8 (Ar*C*), 129.1 (Ar*C*), 129.3 (Ar*C*), 130.6 (Ar*C*), 131.0 (Ar*C*), 131.5 (Ar*C*), 132.6 (Ar*C*), 132.8 (Ar*C*), 133.7 (Ar*C*), 134.0 (Ar*C*), 143.7 (Ar*C*), 147.0 (Ar*C*), 155.4 (Ar*C*), 155.5 (Ar*C*), 155.5 (Ar*C*), 156.4 (*C*O), 156.5 (*C*O), 156.9 (*C*O), 180.1 (*C*S). **HR-MS** (ESI, positive ion mode) – *m/z* for $[C_{59}H_{59}N_9O_8S+Na]^+$ = 1076.4105. Found 1076.4112. **FTIR** (neat) – 3698, 3304, 2996, 2901, 1651.

**1,13-Bis(trifluoroacetyl)-4,7,10-tris(4-methoxyanilinylcarbonyl)-1,4,7,10,13-pentaazatridecane, 5b-1**

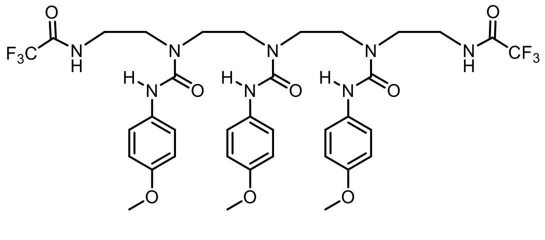

Synthesised according to general procedure C using tetraethylenepentamine (2.51 mL, 13.20 mmol) and 4-methoxyphenyl isocyanate (6.84 mL, 52.80 mmol). After 16 h, a white precipitate formed and was filtered. The residue was washed with DCM (3 x 10 mL) and air-dried to afford the title triurea as a white solid (2.74 g, 3.32 mmol, 25%). **M.P.** 177-178 °C (DCM). **TLC** – $R_f$ = 0.26 (5:95 MeOH:DCM). **$^1$H NMR** (400 MHz, $(CD_3)_2SO$) $\delta_H$ 3.36-3.54 (m, 16H, 8 x NC*H*$_2$), 3.71 (s, 6H, 2 x OC*H*$_3$), 3.72 (3H, s, OC*H*$_3$), 6.83 (d, J = 9.0, 4H, 4 x Ar*H*), 6.85 (d, J = 9.0, 2H, 2 x Ar*H*), 7.38 (d, J = 9.0, 4H, 4 x Ar*H*), 7.44 (d, J = 9.0, 2H, 2 x Ar*H*), 8.47 (s, 2H, 2 x N*H*), 8.81 (s, 1H, N*H*), 9.50 (t, J = 5.7, 2H, 2 x N*H*). **$^{13}$C NMR** (101 MHz, $(CD_3)_2SO$) $\delta_C$ 38.3 (2 x N*C*H$_2$), 46.5 (2 x N*C*H$_2$), 46.7 (2 x N*C*H$_2$), 47.0 (2 x N*C*H$_2$), 55.1 (2 x O*C*H$_3$), 55.2 (O*C*H$_3$), 113.5 (4 x Ar*C*), 113.6 (2 x

Ar*C*), 116.3 (q, J = 290.7, 2 x $CF_3$), 121.4 (2 x Ar*C*), 121.8 (4 x Ar*C*), 133.2 (2 x Ar*C*), 133.3 (Ar*C*), 154.7 (2 x *C*O), 155.6 (Ar*C*), 155.9 (q, J = 35.6, 2 x *C*O). **$^{19}$F NMR** (377 MHz, $(CD_3)_2SO$) $\delta_F$ −74.5 (2 x C*F*$_3$). **HR-MS** (MALDI) – *m/z* for $[C_{36}H_{42}F_6N_8O_8+Na]^+$ = 851.2927. Found 851.2888. **FTIR** (neat) – 3296, 2976, 2945, 1719, 1645.

**4,7,10-Tris(4-methoxyanilinylcarbonyl)-1,4,7,10,13-pentaazatridecane, 5b-2**

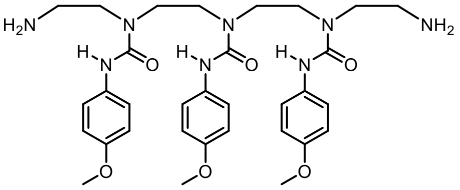


Synthesised according to general procedure D using **5b-1** (4.30 g, 5.19 mmol). After 5 h, a white precipitate formed and was filtered. The residue was washed with MeOH (3 x 10 mL) and air-dried to afford the title diamine as a white solid (3.11 g, 4.88 mmol, 94%). **M.P.** 163-164 °C (DCM). **TLC** – $R_f$ = 0.05 (5:95:1 MeOH:DCM:TEA). **$^1$H NMR** (500 MHz, $(CD_3)_2SO$) $\delta_H$ 2.78 (t, J = 5.7, 4H, 2 x NC*H*$_2$), 3.37 (t, J = 6.0, 4H, 2 x NC*H*$_2$), 3.42 (t, J = 6.6, 4H, 2 x NC*H*$_2$), 3.48 (t, J = 6.6, 4H, 2 x NC*H*$_2$), 3.70 (s, 6H, 2 x OC*H*$_3$), 3.71 (s, 3H, OC*H*$_3$), 6.83 (d, J = 9.0, 4H, 4 x Ar*H*), 6.85 (d, J = 9.0, 2H, 2 x Ar*H*), 7.37 (d, J = 9.0, 4H, 4 x Ar*H*), 7.49 (d, J = 9.0, 2H, 2 x Ar*H*), 8.98 (s, 1H, N*H*), 9.51 (s, 2H, 2 x N*H*). **$^{13}$C NMR** (126 MHz, $(CD_3)_2SO$) $\delta_C$ 40.8 (2 x N*C*H$_2$), 46.3 (2 x N*C*H$_2$), 46.8 (2 x N*C*H$_2$), 47.0 (2 x N*C*H$_2$), 55.6 (2 x O*C*H$_3$), 55.7 (O*C*H$_3$), 114.1 (4 x Ar*C*), 114.1 (2 x Ar*C*), 121.1 (2 x Ar*C*), 121.2 (4 x Ar*C*), 134.0 (Ar*C*), 134.1 (2 x Ar*C*), 154.8 (2 x *C*O), 154.9 (2 x Ar*C*), 156.1 (Ar*C*), 157.0 (*C*O). **HR-MS** (MALDI) – *m/z* for $[C_{32}H_{44}N_8O_6+Na]^+$ = 659.3282. Found 659.3329. **FTIR** (neat) – 3237, 2987, 2930, 1647.

***tert*-Butyl benzyl(2-hydroxyethyl)carbamate, 5b-3**

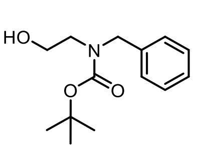


To a solution of amine *N*-benzylethanolamine (3.55 mL, 25.0 mmol, 1.0 equiv, 0.25 M) in DCM (100 mL) was added a solution of sodium hydroxide (1.0 M) in distilled water. The mixture was cooled to 0 °C and a solution of di-*tert*-butyl dicarbonate (5.47 g, 25.0 mmol, 1.0 equiv, 0.25 M) in DCM (100 mL) was added over three portions. The resultant mixture was stirred for 16 h and the aqueous phase was washed with DCM three times. The combined organic extracts were washed with distilled water twice, dried ($MgSO_4$), filtered and concentrated *in vacuo*. The crude product was purified by column chromatography (10-30% EtOAc:PE) to give the title carbamate as a colourless oil (5.72 g, 22.8 mmol, 91%). **TLC** – $R_f$ = 0.05 (10:90 EtOAc:PE). Spectroscopic data matched that previously reported.[5]

***tert*-Butyl benzyl(2-oxoethyl)carbamate, 5b-4**

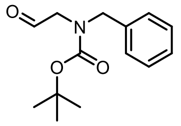

Synthesised according to general procedure K using **5b-3** (1000 mg, 3.98 mmol). The crude product was purified by column chromatography (10-20% EtOAc:PE) to give the title aldehyde as a colourless oil (982 mg, 3.94 mmol, 99%). **TLC** – $R_f$ = 0.13 (10:90 EtOAc:PE). Spectroscopic data matched that previously reported.[5]

**1-Benzyl-1-(*tert*-butoxycarbonyl)-7,10,13-tris(4-methoxyanilinylcarbonyl)-1,4,7,10,13,16-hexaazahexadecane, 5b-5**

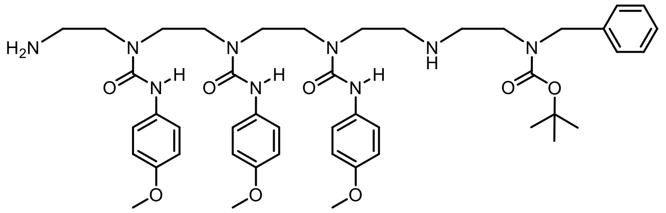

Synthesised according to general procedure J using **5b-2** (200 mg, 0.31 mmol) and **5b-4** (86 mg, 0.35 mmol). The crude product was purified by column chromatography (1-10% MeOH:DCM, 1% TEA) to give the title amine as a foamy white solid (122 mg, 0.14 mmol, 45%). **M.P.** 150-151 °C ($CHCl_3$). **TLC** – $R_f$ = 0.38 (10:90:1 MeOH:DCM:TEA). **$^1$H NMR** (400 MHz, $CDCl_3$) $\delta_H$ 1.45 (s, 9H, C(C*H*$_3$)$_3$), 2.70-2.83 (m, 4H, 2 x NC*H*$_2$), 2.96 (t, J = 4.9, NC*H*$_2$), 3.26-3.57 (m, 14H, 7 x NC*H*$_2$), 3.76 (s, 3H, OC*H*$_3$), 3.76 (s, 3H, OC*H*$_3$), 3.77 (s, 3H, OC*H*$_3$), 4.41 (s, 2H, C*H*$_2$Ar), 6.79 (d, J = 9.1, 2H, 2 x Ar*H*), 6.80 (d, J = 9.1, 2H, 2 x Ar*H*), 6.82 (d, J = 9.1, 2H, 2 x Ar*H*), 7.18 (d, J = 9.1, 2H, 2 x Ar*H*), 7.27-7.37 (m, 7H, 7 x Ar*H*), 7.57 (d, J = 9.1, 2H, 2 x Ar*H*), 8.96 (s, 1H, N*H*), 9.73 (s, 1H, N*H*), 9.83 (s, 1H, N*H*). **$^{13}$C NMR** (101 MHz, $CDCl_3$) $\delta_C$ 28.5 (C(*C*H$_3$)$_3$), 46.1 (7 x N*C*H$_2$), 46.4 (N*C*H$_2$), 47.2 (N*C*H$_2$), 48.1 (*C*H$_2$Ar), 49.6 (N*C*H$_2$), 55.6 (3 x O*C*H$_3$), 80.4 (*C*(CH$_3$)$_3$), 113.8-114.3 (6 x Ar*C*), 120.0-121.4 (6 x Ar*C*), 127.3 (2 x Ar*C*), 127.5 (Ar*C*), 128.6 (2 x Ar*C*), 133.3-133.7 (3 x Ar*C*), 138.3 (Ar*C*), 155.1 (3 x Ar*C*), 156.0 (3 x *C*O), 156.4 (*C*O). **HR-MS** (ESI, positive ion mode) – *m/z* for $[C_{46}H_{63}N_9O_8+H]^+$ = 870.4878. Found 870.4899. **FTIR** (neat) – 3290, 2994, 2934, 1649.

**1-Benzyl-7,10,13-tris(4-methoxyanilinylcarbonyl)-16-trifluoroacetyl-1,4,7,10,13,16-hexaazahexadecane, 5b-6**

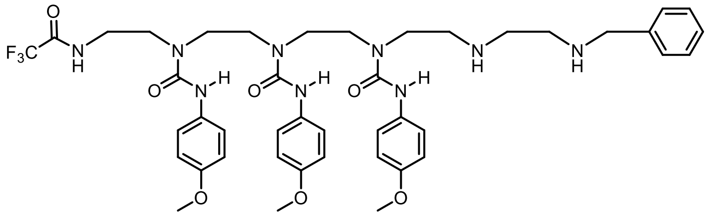

Under a dry, inert atmosphere, a solution of **5b-5** (700 mg, 0.80 mmol, 1.00 equiv, 1.0 M) in anhydrous DCM (0.8 mL) was cooled to 0 °C. Ethyl trifluoroacetate (0.11 mL, 0.88 mmol, 1.10 equiv) was added dropwise over 30 minutes and the resulting solution was stirred for 30 minutes at 0 °C. The solution was then warmed to room temperature and stirred for a further hour. The solution was then

diluted with anhydrous DCM (6 mL) and TFA (2 mL) was added dropwise. The solution was stirred for 16 h and concentrated *in vacuo*. The crude product was diluted with saturated aqueous $NaHCO_3$ and then extracted with DCM (3 x 25 mL). The combined organic extracts were dried ($MgSO_4$), filtered and concentrated *in vacuo* to give the title amine as a foamy white solid (540 mg, 0.62 mmol, 78%). **M.P.** 156-157 °C ($CHCl_3$). **TLC** – $R_f$ = 0.21 (5:95:1 MeOH:DCM:TEA). **$^1$H NMR** (400 MHz, $CDCl_3$) $\delta_H$ 2.69-2.85 (m, 8H, 4 x NC$H_2$), 3.32-3.55 (m, 14H, C$H_2$Ar, 6 x NC$H_2$), 3.74 (s, 3H, OC$H_3$), 3.76 (s, 6H, 2 x OC$H_3$), 6.74-6.84 (m, 6H, 6 x Ar*H*), 7.21-7.32 (m, 5H, 5 x Ar*H*), 7.52 (d, J = 8.9, 2H, 2 x Ar*H*), 7.59 (d, J = 8.9, 2H, 2 x Ar*H*), 7.60 (d, J = 8.9, 2H, 2 x Ar*H*), 8.82 (s, 1H, N*H*), 9.00 (s, 1H, N*H*), 9.13 (s, 1H, N*H*). **$^{13}$C NMR** (101 MHz, $CDCl_3$) $\delta_C$ 46.4 (N$CH_2$), 47.1 (N$CH_2$), 48.3 (N$CH_2$), 48.4 (N$CH_2$), 48.4 (N$CH_2$), 48.5 (N$CH_2$), 49.2 ($CH_2$Ar), 49.6 (N$CH_2$), 49.7 (N$CH_2$), 50.0 (N$CH_2$), 50.1 (N$CH_2$), 55.5 (3 x O$CH_3$), 113.9 (2 x Ar*C*), 114.0 (2 x Ar*C*), 114.0 (2 x Ar*C*), 115.9 (q, J = 287.8, $CF_3$), 120.9 (2 x Ar*C*), 121.1 (2 x Ar*C*), 121.5 (2 x Ar*C*), 127.1 (Ar*C*), 128.0 (2 x Ar*C*), 128.5 (2 x Ar*C*), 132.9-133.6 (3 x Ar*C*), 140.1 (Ar*C*), 155.0 (Ar*C*), 155.2 (Ar*C*), 155.3 (Ar*C*), 155.3 (*C*O), 155.5 (*C*O), 156.4 (q, J = 36.4, *C*O), 156.6 (*C*O). **$^{19}$F NMR** (377 MHz, $CDCl_3$) $\delta_F$ −74.3 (C$F_3$). **HR-MS** (ESI, positive ion mode) – *m/z* for $[C_{43}H_{54}F_3N_9O_7+Na]^+$ = 888.3996. Found 888.3981. **FTIR** (neat) – 3324, 2990, 2945, 1647.

**1-Benzyl-1,4,7,10,13-pentakis(4-methoxyanilinylcarbonyl)-16-trifluoroacetyl-1,4,7,10,13,16-hexaazahexadecane, 5b-7**

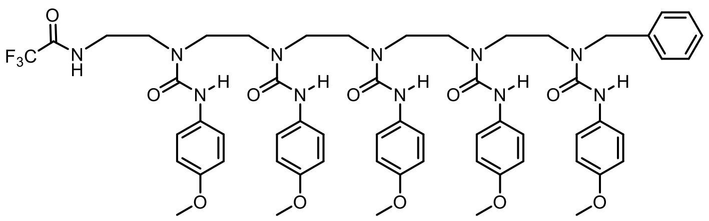


Synthesised according to general procedure A using **5b-6** (1.0 g, 1.15 mmol) and 4-methoxyphenyl isocyanate (0.45 mL, 3.46 mmol). The crude product was purified by column chromatography (2-10% MeOH:DCM) to give the title pentaurea as a foamy white solid (503 mg, 0.43 mmol, 38%). **M.P.** 186-187 °C (DCM/$Et_2O$). **TLC** – $R_f$ = 0.25 (5:95 MeOH:DCM). **$^1$H NMR** (400 MHz, $CDCl_3$) $\delta_H$ 3.32-3.64 (m, 20H, 10 x NC$H_2$), 3.71-3.81 (m, 15H, 5 x OC$H_3$), 4.49 (s, 2H, C$H_2$Ar), 5.88 (s, 1H, N*H*), 6.78-6.85 (m, 10H, 10 x Ar*H*), 7.07 (d, J = 9.0, 2H, 2 x Ar*H*), 7.20 (d, J = 9.0, 2H, 2 x Ar*H*), 7.26-7.36 (m, 5H, 5 x Ar*H*), 7.49 (d, J = 9.0, 2H, 2 x Ar*H*), 7.56 (d, J = 9.0, 2H, 2 x Ar*H*), 7.57 (d, J = 9.0, 2H, 2 x Ar*H*), 8.34 (s, 1H, N*H*), 8.87-9.18 (m, 5H, 5 x N*H*). **$^{13}$C NMR** (101 MHz, $CDCl_3$) $\delta_C$ 47.0-48.0 (10 x N$CH_2$), 48.2 ($CH_2$Ar), 55.5-55.6 (5 x O$CH_3$), 114.0 (6 x Ar*C*), 114.1 (2 x Ar*C*), 114.2 (2 x Ar*C*), 116.5 (q, J = 284.9, $CF_3$), 121.1-121.3 (10 x Ar*C*), 126.6 (2 x Ar*C*), 127.9 (Ar*C*), 129.1 (2 x Ar*C*), 132.8 (Ar*C*), 132.9 (Ar*C*), 133.0 (3 x Ar*C*), 140.6 (Ar*C*), 155.3 (4 x Ar*C*), 155.9 (Ar*C*), 156.6 (4 x *C*O), 156.7 (q, J = 36.1, *C*O), 157.3 (*C*O). **$^{19}$F NMR** (377 MHz, $CDCl_3$) $\delta_F$ −74.9 (C$F_3$). **HR-MS** (MALDI) – *m/z* for $[C_{59}H_{68}F_3N_{11}O_{11}+Na]^+$ = 1186.4950. Found 1186.4893. **FTIR** (neat) – 3320, 2924, 2898, 1648.

**1-Benzyl-1,4,7,10,13-pentakis(4-methoxyanilinylcarbonyl)-1,4,7,10,13,16-hexaazahexadecane, 5b-8**

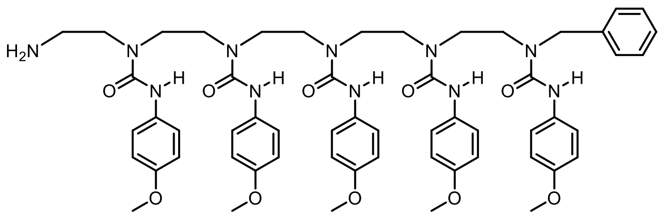

Synthesised according to general procedure D using **5b-7** (450 mg, 0.39 mmol). The title amine was isolated as a foamy white solid (340 mg, 0.32 mmol, 82%). **M.P.** 150-151 °C (DCM/$Et_2O$). **TLC** – $R_f$ = 0.18 (8:92 MeOH:DCM). **$^1$H NMR** (500 MHz, $CDCl_3$) $\delta_H$ 2.97 (t, J = 4.0, 2H, NC*H*2), 3.35-3.63 (m, 18H, 9 x NC*H*2), 3.79-3.82 (m, 15H, 5 x OC*H*3), 4.63 (s, 2H, C*H*2Ar), 6.80-6.88 (m, 10H, 10 x Ar*H*), 7.26-7.36 (m, 9H, 9 x Ar*H*), 7.60 (d, J = 8.8, 2H, 2 x Ar*H*), 7.63 (d, J = 8.8, 4H, 4 x Ar*H*), 8.97 (s, 1H, N*H*), 9.08-9.13 (m, 3H, 3 x N*H*), 10.1 (s, 1H, N*H*). **$^{13}$C NMR** (126 MHz, $CDCl_3$) $\delta_C$ 42.1 (N*C*H2), 46.8-48.6 (9 x N*C*H2), 51.0 (*C*H2Ar), 55.5-55.6 (5 x O*C*H3), 113.9 (6 x Ar*C*), 114.0 (2 x Ar*C*), 114.1 (2 x Ar*C*), 120.6 (2 x Ar*C*), 121.1 (6 x Ar*C*), 121.7 (2 x Ar*C*), 126.9 (2 x Ar*C*), 127.7 (Ar*C*), 129.0 (2 x Ar*C*), 133.2-133.5 (5 x Ar*C*), 140.0 (Ar*C*), 155.2 (5 x Ar*C*), 156.7 (5 x *C*O). **HR-MS** (ESI, positive ion mode) – *m/z* for $[C_{57}H_{69}N_{11}O_{10}+Na]^+$ = 1090.5127. Found 1090.5095. **FTIR** (neat) – 3315, 3031, 2950, 1640.

**1,19-Dibenzyl-1,4,7,10,13-pentakis(4-methoxyanilinylcarbonyl)-19-(*tert*-butoxycarbonyl)-1,4,7,10,13,16,19-heptaazanonadecane, 5b-9**

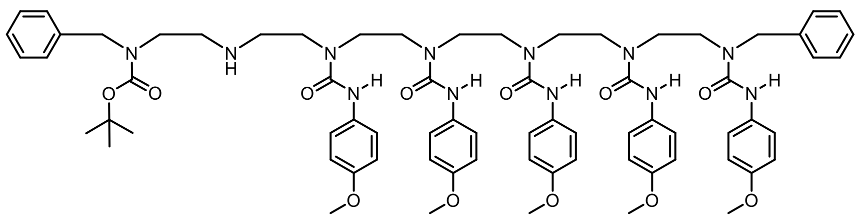

Synthesised according to general procedure B using **5b-8** (340 mg, 0.32 mmol) and **5b-4** (159 mg, 0.64 mmol). The crude product was purified by column chromatography (2-10% MeOH:DCM) to give the title amine as a foamy white solid (233 mg, 0.18 mmol, 56%). **M.P.** 155-156 °C (DCM/$Et_2O$). **TLC** – $R_f$ = 0.33 (8:92 MeOH:DCM). **$^1$H NMR** (400 MHz, $CDCl_3$) $\delta_H$ 1.46 (s, 9H, C(C*H*3)3), 1.91 (s, 1H, N*H*), 2.71-2.79 (m, 4H, 2 x NC*H*2), 3.24-3.58 (m, 20H, 10 x NC*H*2), 3.74-3.79 (m, 15H, 5 x OC*H*3), 4.40 (s, 2H, C*H*2Ar), 4.58 (s, 2H, C*H*2Ar), 6.77-6.87 (m, 10H, 10 x Ar*H*), 7.15 (d, J = 9.0, 2H, 2 x Ar*H*), 7.25-7.39 (m, 14H, 14 x Ar*H*), 7.53-7.68 (m, 6H, 6 x Ar*H*), 8.98-9.15 (m, 4H, 4 x N*H*), 9.91 (s, 1H, N*H*). **$^{13}$C NMR** (101 MHz, $CDCl_3$) $\delta_C$ 28.5 (*C*(CH3)3), 42.4 (N*C*H2), 43.2 (N*C*H2), 46.4-49.0 (10 x N*C*H2), 50.2 (*C*H2Ar), 51.3 (*C*H2Ar), 55.4-55.6 (5 x O*C*H3), 80.1 (*C*(CH3)3), 113.8-114.1 (10 x Ar*C*), 121.0-121.3 (10 x Ar*C*), 126.9-128.8 (10 x Ar*C*), 132.8-133.9 (5 x Ar*C*), 140.2 (Ar*C*), 140.8 (Ar*C*), 155.1-155.4 (5 x Ar*C*) 156.0-156.5 (6 x *C*O). **HR-MS** (ESI, positive ion mode) – *m/z* for $[C_{71}H_{88}N_{12}O_{12}+H]^+$ = 1301.6723. Found 1301.6700. **FTIR** (neat) – 3287, 2933, 1650.

**1,19-Dibenzyl-1,4,7,10,13,16-hexakis(4-methoxyanilinylcarbonyl)-19-(*tert*-butoxycarbonyl)-1,4,7,10,13,16,19-heptaazanonadecane, 5b-10**

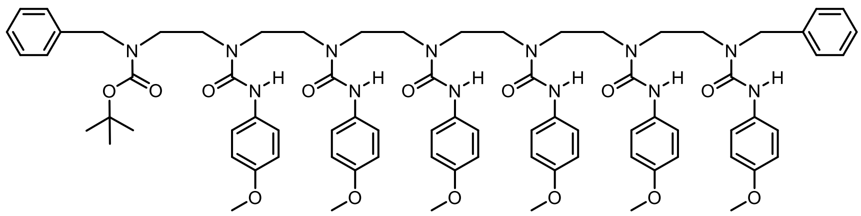

Synthesised according to general procedure A using **5b-9** (135 mg, 0.10 mmol) and 4-methoxphenyl isocyanate (0.02 mL, 0.16 mmol). The crude product was purified by column chromatography (2-10% MeOH:DCM) to give the title hexaurea as a foamy white solid (130 mg, 0.09 mmol, 90%). **M.P.** 190-191 °C (DCM/$Et_2O$). **TLC** – $R_f$ = 0.23 (3:97 MeOH:DCM). **$^1$H NMR** (400 MHz, $CDCl_3$) $\delta_H$ 1.50 (s, 9H, C(C*H*$_3$)$_3$), 3.28-3.60 (m, 24H, 12 x NC*H*$_2$), 3.74-3.81 (m, 18H, 6 x OC*H*$_3$), 4.41 (s, 2H, C*H*$_2$Ar), 4.57 (s, 2H, C*H*$_2$Ar), 6.76-6.87 (m, 12H, 12 x Ar*H*), 7.19 (d, J = 9.0, 2H, 2 x Ar*H*), 7.28-7.41 (m, 10H, 10 x Ar*H*), 7.57-7.65 (m, 10H, 10 x Ar*H*), 8.83 (s, 1H, N*H*), 8.97 (s, 1H, N*H*), 9.02 (s, 1H, N*H*), 9.13 (s, 1H, N*H*), 9.17 (s, 1H, N*H*). **$^{13}$C NMR** (101 MHz, $CDCl_3$) $\delta_C$ 28.5 (C(*C*H$_3$)$_3$), 45.8-49.0 (12 x N*C*H$_2$), 50.2-51.5 (2 x *C*H$_2$Ar), 55.5-55.6 (6 x O*C*H$_3$), 80.0 (*C*(CH$_3$)$_3$), 113.7-114.1 (12 x Ar*C*), 120.7-121.7 (12 x Ar*C*), 127.4-128.8 (10 x Ar*C*), 132.8-133.6 (6 x Ar*C*), 139.9-140.6 (2 x Ar*C*), 155.0-155.4 (6 x Ar*C*), 156.7-157.1 (7 x *C*O). **HR-MS** (MALDI) – *m/z* for $[C_{79}H_{95}N_{13}O_{14}+Na]^+$ = 1472.7019. Found 1472.6984. **FTIR** (neat) – 3321, 2924, 2853, 1713.

**1,19-Dibenzyl-1,4,7,10,13,16-hexakis(4-methoxyanilinylcarbonyl)-1,4,7,10,13,16,19-heptaazanonadecane, 5b-11**

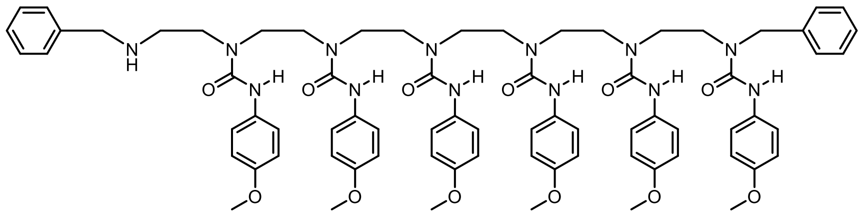

Synthesised according to general procedure H using **5b-10** (130 mg, 0.09 mmol). The title amine was isolated as a foamy white solid (101 mg, 0.07 mmol, 83%). **M.P.** 204-205 °C (DCM/$Et_2O$). **TLC** – $R_f$ = 0.13 (3:97 MeOH:DCM). **$^1$H NMR** (400 MHz, $CDCl_3$) $\delta_H$ 2.87 (s, 2H, NC*H*$_2$), 3.30-3.62 (m, 22H, 11 x NC*H*$_2$), 3.75-3.78 (m, 18H, 6 x OC*H*$_3$), 3.82 (s, 2H, C*H*$_2$Ar), 4.57 (C*H*$_2$Ar), 6.74-6.86 (m, 12H, 12 x Ar*H*), 7.22 (d, J = 9.0, 2H, 2 x Ar*H*), 7.28-7.41 (m, 12H, 12 x Ar*H*), 7.55-7.64 (m, 8H, 8 x Ar*H*), 8.95 (s, 1H, N*H*), 9.06 (s, 1H, N*H*), 9.08 (s, 1H, N*H*), 9.15 (s, 1H, N*H*), 10.04 (s, 1H, N*H*). **$^{13}$C NMR** (101 MHz, $CDCl_3$) $\delta_C$ 47.0-49.2 (12 x N*C*H$_2$), 51.0-52.2 (2 x *C*H$_2$Ar), 55.6-55.7 (6 x O*C*H$_3$), 113.8-114.3 (12 x Ar*C*), 121.1-122.6 (12 x Ar*C*), 127.6-129.0 (10 x Ar*C*), 133.0-133.8 (6 x Ar*C*), 140.3-140.6 (2 x Ar*C*), 155.2-156.8 (6 x *C*O, 6 x Ar*C*). **HR-MS** (ESI, positive ion mode) – *m/z* for $[C_{74}H_{87}N_{13}O_{12}+Na]^+$ = 1372.6495. Found 1372.6494. **FTIR** (neat) – 3338, 2950, 1645.

**1,19-Dibenzyl-1,4,7,10,13,16-hexakis(4-methoxyanilinylcarbonyl)-19-(4-nitroanilinylthiocarbonyl)-1,4,7,10,13,16,19-heptaazanonadecane, 5b**

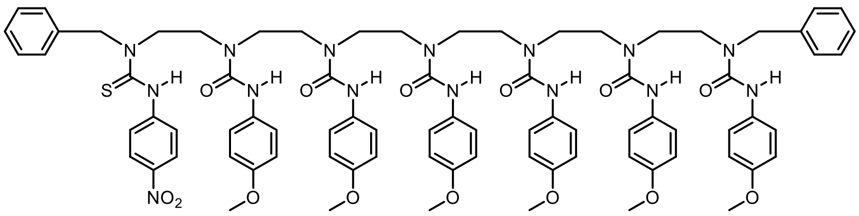

Synthesised according to general procedure A using **5b-11** (40 mg, 0.03 mmol) and 4-nitrophenyl isothiocyanate (8 mg, 0.04 mmol). The crude product was purified by column chromatography (2-10% MeOH:DCM) to give the title thiourea as a yellow solid (46 mg, 0.03 mmol, >99%). **M.P.** 159-160 °C (DCM/$Et_2O$). **TLC** – $R_f$ = 0.28 (3:97 MeOH:DCM). **$^1$H NMR** (500 MHz, $CD_2Cl_2$) $\delta_H$ 3.38-3.68 (m, 24H, 12 x NC*H*$_2$), 3.76-3.85 (m, 18H, 6 x OC*H*$_3$), 4.61 (s, 2H, C*H*$_2$Ar), 5.32 (s, 2H, C*H*$_2$Ar), 6.31 (s, 1H, N*H*), 6.84-6.93 (m, 12H, 12 x Ar*H*), 7.17-7.67 (m, 22H, 22 x Ar*H*), 8.06 (d, J = 9.0, 2H, 2 x Ar*H*), 8.19 (d, J = 8.8, 2H, 2 x Ar*H*), 9.03 (s, 1H, N*H*), 9.17 (s, 1H, N*H*), 9.19 (s, 1H, N*H*), 9.23 (s, 1H, N*H*), 9.29 (s, 1H, N*H*), 10.48 (s, 1H, N*H*). **$^{13}$C NMR** (126 MHz, $CD_2Cl_2$) $\delta_C$ 47.3-49.0 (12 x N*C*H$_2$), 53.3 (*C*H$_2$Ar), 55.9-56.2 (6 x O*C*H$_3$), 56.8 (*C*H$_2$Ar), 114.3-114.7 (12 x Ar*C*), 121.6-121.9 (12 x Ar*C*), 123.0 (2 x Ar*C*), 124.4 (2 x Ar*C*), 126.6-130.2 (10 x Ar*C*), 133.4-134.0 (6 x Ar*C*), 138.9-139.3 (2 x Ar*C*), 143.7 (Ar*C*), 147.4 (Ar*C*), 155.4-155.8 (6 x Ar*C*), 156.7-157.2 (6 x *C*O), 182.3 (*C*S). **HR-MS** (MALDI) – *m/z* for $[C_{81}H_{91}N_{15}O_{14}S+Na]^+$ = 1552.6488. Found 1552.6491. **FTIR** (neat) – 3675, 3345, 2971, 2914, 1648, 1606, 1551.

***N*-(4-methoxyphenyl)-*N*-methylcarbamoyl chloride, 6a-1**

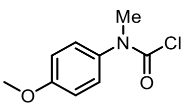

Under a dry, inert atmosphere, a solution of triphosgene (500 mg, 1.68 mmol, 0.46 equiv) in anhydrous DCM (5 mL) was cooled to 0 °C. To this solution was added anhydrous pyridine (0.30 mL, 3.66 mmol, 1.00 equiv) dropwise and the solution was stirred for 5 minutes. 4-methoxy-*N*-methylaniline (502 mg, 3.66 mmol, 1.00 equiv, 0.70 M) was added, the solution was warmed to room temperature and stirred for 1 h. Hydrochloric acid (1 M, 10 mL) was added and the aqueous phase extracted with DCM (3 x 5 mL). The combined organic extracts were washed with saturated aqueous $NaHCO_3$, dried ($MgSO_4$), filtered and concentrated *in vacuo*. The crude product was purified by column chromatography (50% EtOAc:PE) to give the title carbamoyl chloride as a yellow oil (731 mg, 3.66 mmol, >99%). **TLC** – $R_f$ = 0.63 ($SiO_2$, 1:1 EtOAc:PE). Spectroscopic data matched that previously reported.[6]

**1,10-Bis(naphthalen-1-ylmethyl)-1,4,7-tris(4-methoxyanilinylcarbonyl)-10-((*N*-methyl-4-methoxyanilinyl)carbonyl)-1,4,7,10-tetraazadecane, 6a**

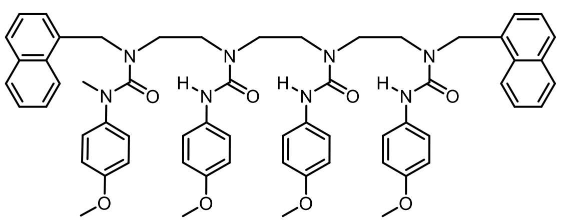

Synthesised according to general procedure G using **5a-1** (30 mg, 0.03 mmol) and **6a-1** (10 mg, 0.05 mmol). The crude product was purified by column chromatography (1-2% MeOH:DCM) to yield the title tetraurea as a white solid (27 mg, 0.03 mmol, 88%). **M.P.** 153-154 °C ($CHCl_3$). **TLC** – $R_f$ = 0.14 ($SiO_2$, 1:99 MeOH:DCM). **$^1$H NMR** (500 MHz, $CD_2Cl_2$) $\delta_H$ 2.86-2.96 (m, 4H, 2 x NC*H*$_2$), 3.05 (NC*H*$_2$), 3.27-3.30 (m, 4H, 2 x NC*H*$_2$), 3.36 (t, J = 7.0, 2H, NC*H*$_2$), 3.66 (s, 3H, NC*H*$_3$), 3.76 (s, 3H, OC*H*$_3$), 3.78 (s, 3H, OC*H*$_3$), 3.78 (s, 3H, OC*H*$_3$), 4.50 (s, 2H, C*H*$_2$Ar), 5.08 (s, 2H, C*H*$_2$Ar), 6.71 (d, J = 8.9, 2H, 2 x Ar*H*), 6.79 (d, J = 9.0, 2H, 2 x Ar*H*), 6.85 (d, J = 9.0, 2H, 2 x Ar*H*), 6.86 (d, J = 9.0, 2H, 2 x Ar*H*), 7.05 (d, J = 8.9, 2H, 2 x Ar*H*), 7.12 (d, J = 6.7, 1H, Ar*H*), 7.27 (t, J = 7.5, 1H, Ar*H*), 7.36-7.73 (m, 13H, 13 x Ar*H*), 7.79 (d, J = 8.0, 1H, Ar*H*), 7.83 (d, J = 8.0, 1H, Ar*H*), 8.15 (d, J = 8.0, 1H, Ar*H*), 8.73 (s, 1H, N*H*), 8.85 (s, 1H, N*H*). 9.09 (s, 1H, N*H*). **$^{13}$C NMR** (126 MHz, $CD_2Cl_2$) $\delta_C$ 40.9 (N*C*H$_3$), 44.9 (N*C*H$_2$), 47.0 (N*C*H$_2$), 47.1 (N*C*H$_2$), 47.4 (N*C*H$_2$), 47.8 (N*C*H$_2$), 48.5 (N*C*H$_2$), 48.7 (*C*H$_2$Ar), 51.9 (*C*H$_2$Ar), 55.4 (4 x O*C*H$_3$), 113.7 (2 x Ar*C*), 113.7 (2 x Ar*C*), 113.8 (2 x Ar*C*), 114.8 (2 x Ar*C*), 121.0 (2 x Ar*C*), 121.1 (2 x Ar*C*), 122.5 (2 x Ar*C*), 123.5 (2 x Ar*C*), 125.0 (Ar*C*), 125.2 (Ar*C*), 125.3 (Ar*C*), 125.8 (Ar*C*), 126.0 (Ar*C*), 126.1 (Ar*C*), 126.4 (Ar*C*), 126.5 (Ar*C*), 126.7 (Ar*C*), 128.0 (Ar*C*), 128.4 (Ar*C*), 128.3 (Ar*C*), 128.5 (Ar*C*), 128.6 (Ar*C*), 128.9 (Ar*C*), 131.1 (Ar*C*), 131.6 (Ar*C*), 132.3 (Ar*C*), 133.2 (Ar*C*), 133.3 (Ar*C*), 133.6 (Ar*C*), 133.8 (Ar*C*), 138.9 (Ar*C*), 155.0 (Ar*C*), 155.1 (Ar*C*), 155.3 (Ar*C*), 156.4 (*C*O), 156.5 (*C*O), 157.4 (*C*O), 162.8 (*C*O). **HR-MS** (ESI, positive ion mode) – *m/z* for $[C_{61}H_{64}N_8O_8+Na]^+$ = 1059.4745. Found 1059.4725. **FTIR** (neat) – 3315, 2990, 2905, 1652, 1609.

**1,19-Dibenzyl-1,4,7,10,13,16-hexakis(4-methoxyanilinylcarbonyl)-19-((*N*-methyl-4-methoxyanilinyl)carbonyl)-1,4,7,10,13,16,19-heptaazanonadecane, 6b**

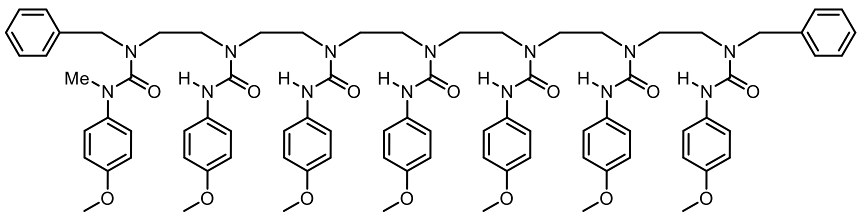

Synthesised according to general procedure G using **5b-11** (40 mg, 0.03 mmol) and **6a-1** (9 mg, 0.04 mmol). The crude product was purified by column chromatography (2-10% MeOH:DCM) to give the title heptaurea as a foamy white solid (37 mg, 0.02 mmol, 80%). **M.P.** 170-171 °C (DCM/$Et_2O$). **TLC** – $R_f$ = 0.15 (3:97 MeOH:DCM). **$^1$H NMR** (500 MHz, $CD_2Cl_2$) $\delta_H$ 3.30-3.60 (m, 12H, 6 x NC$H_2$), 3.76 (s, 3H, NC$H_3$), 3.80-3.84 (m, 18H, 6 x OC$H_3$), 4.07 (s, 2H, C$H_2$Ar), 4.59 (s, 2H, C$H_2$Ar), 6.77-6.89 (m, 14H, 14 x Ar*H*), 6.95 (d, J = 7.0, 2H, 2 x Ar*H*), 7.02 (d, J = 8.8, 2H, 2 x Ar*H*), 7.17-7.40 (m, 10H, 10 x Ar*H*), 7.56-7.67 (m, 10H, 10 x Ar*H*), 8.99-9.23 (m, 6H, 6 x N*H*). **$^{13}$C NMR** (126 MHz, $CD_2Cl_2$) $\delta_C$ 40.4 (N*C*$H_3$), 46.3-49.1 (12 x N*C*$H_2$), 53.0 (*C*$H_2$Ar), 53.8 (*C*$H_2$Ar), 55.3-55.5 (7 x O*C*$H_3$), 113.7-114.1 (14 x Ar*C*), 121.0-121.5 (14 x Ar*C*), 126.2-128.7 (10 x Ar*C*), 133.2-133.6 (6 x Ar*C*), 137.7 (Ar*C*), 138.6-139.2 (2 x Ar*C*), 155.2-155.7 (7 x Ar*C*), 156.5-157.0 (6 x *C*O), 163.1 (*C*O). **HR-MS** (MALDI) – *m/z* for $[C_{83}H_{96}N_{14}O_{14}+Na]^+$ = 1535.7128. Found 1535.7096. **FTIR** (neat) – 3349, 2968, 2931, 1649.

***tert*-Butyl (2-trifluoroacetamidoethyl)carbamate, 4b-1**

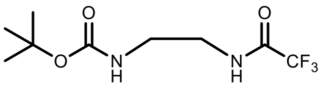

To a solution of *N*-Boc-ethylenediamine (1.602 g, 10.00 mmol, 1.0 equiv) in lab grade $CH_2Cl_2$ (10 mL) at room temperature under air was added over 1 min a solution of ethyl trifluoroacetate (1.19 mL, 10.00 mmol, 1.0 equiv) in $CH_2Cl_2$ (10 mL). The mixture was stirred at room temperature for 19 h. Additional ethyl trifluoroacetate (0.71 mL, 6.00 mmol, 0.6 equiv) was added and the mixture was stirred for a further 75.5 h. The mixture was diluted with $CH_2Cl_2$ (40 mL) and washed with aqueous HCl (40 mL, 1.0 M) to remove unreacted *N*-Boc-ethylenediamine. The organic phase was dried ($Na_2SO_4$) and concentrated to give **4b-1** (2.206 g, 86%) as a white solid. **TLC** – $R_f$ = 0.65 (5:95 MeOH/$CH_2Cl_2$, ninhydrin stain). **$^1$H NMR** (400 MHz, $CDCl_3$) $\delta_H$ 1.42 (s, 9H), 3.31 – 3.47 (m, 4H), 5.02 – 5.11 (broad m, 1H), 7.91 (bs, 1H). **$^{13}$C NMR** (101 MHz, $CDCl_3$) $\delta_C$ 28.3, 39.2, 42.2, 80.6, 116.0 (q, *J* = 287.6 Hz), 157.9, 158.0 (q, *J* = 37.3 Hz). **$^{19}$F NMR** (376 MHz, $CDCl_3$) $\delta_F$ –76.0 (s, 3F). Spectroscopic data matches that previously reported.[7]

**2-Trifluoroacetamidoethylammonium chloride, 4b-2**

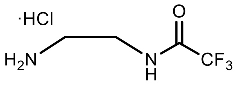


To a solution of **4b-1** (2.154 g, 8.41 mmol, 1.0 equiv in lab grade $CH_2Cl_2$ (42 mL) at room temperature under air was added TFA (9.66 mL, 126.1 mmol, 15.0 equiv and the mixture was stirred at room temperature for 15 h. The mixture was concentrated on a rotary evaporator and then under high vacuum to remove most of the excess TFA. The resulting colourless gum (3.177 g, 140% of the theoretical mass of the product amine·TFA salt) was dissolved in lab grade MeOH (10 mL) and HCl (1.25 M in MeOH, 20 mL, 25 mmol) was added. The mixture was concentrated *in vacuo* to give the HCl salt **4b-2** (1.604 g, 99%) as a white solid. **$^1$H NMR** (400 MHz, $CD_3OD$) $\delta_H$ 3.15 (t, $J$ = 6.2 Hz, 2H), 3.61 (t, $J$ = 6.1 Hz, 2H). **$^{13}$C NMR** (101 MHz, $CD_3OD$) $\delta_C$ 38.3, 39.9, 117.3 (q, $J$ = 286.5 Hz), 159.9 (q, $J$ = 37.6 Hz). **$^{19}$F NMR** (376 MHz, $CD_3OD$) $\delta_F$ –77.2 (s, 3F). Spectroscopic data matches that previously reported.[8]

***N*-(2-Pyridyl)-*N*'-(2-hydroxyethyl)-1,2-diaminoethane, 4b-3**

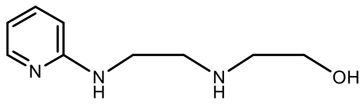


A mixture of 2-fluoropyridine (1.72 mL, 20.0 mmol, 1.0 equiv) and aminoethylethanolamine (6.07 mL, 60.0 mmol, 3.0 equiv) under $N_2$ was heated at 120 °C (flask with condenser but no water flow necessary) for 17.5 h. After cooling to room temperature, aqueous $K_2CO_3$ (25 mL, 1.0 M) and water (25 mL) were added. The water-soluble product was extracted with $CH_2Cl_2$ (6 × 50 mL) then the combined organic extracts were concentrated. Flash chromatography (Biotage, 100 g SNAP Ultra column, MeOH/[35% aqueous $NH_3$]/$CH_2Cl_2$ gradient from 2.5:0.5:97 to 10:2:88) gave two fractions containing **4b-3**: the first fraction (1.053 g) contained an unknown higher $R_F$ impurity and showed ~80 mol % purity of **4b-3** by $^1$H/$^{13}$C NMR; the second fraction contained pure **4b-3** (1.229 g) as a colourless gum. Total yield of **4b-3** (>90 mol% combined purity) = 2.282 g, 63%. **TLC** – $R_f$ = 0.28 (10:2:88 MeOH/[35% aqueous $NH_3$]/$CH_2Cl_2$). **$^1$H NMR** (400 MHz, $CDCl_3$) $\delta_H$ 2.74 – 2.77 (m, 2H), 2.85 (t, $J$ = 5.8 Hz, 2H), 3.08 (bs, 2H), 3.38 (q, $J$ = 5.7 Hz, 2H), 3.65 (t, $J$ = 5.2 Hz, 2H), 5.21 (t, $J$ = 5.7 Hz, 1H), 6.38 (d, $J$ = 8.4 Hz, 1H), 6.52 (ddd, $J$ = 7.2, 5.2, 1.0 Hz, 1H), 7.37 (ddd, $J$ = 8.7, 7.1, 1.9 Hz, 1H), 8.01 (dd, $J$ = 5.2, 1.9 Hz, 1H). **$^{13}$C NMR** (101 MHz, $CDCl_3$) $\delta_C$ 41.8, 48.5, 51.2, 61.0, 107.3, 112.9, 137.6, 147.9, 158.9. **HR-MS** (ESI, positive ion mode) – *m/z* for $[C_9H_{15}N_3O+H]^+$ = 182.1288. Found 182.1293.

**1-(2-Pyridyl)-4-(*tert*-butoxycarbonyl)-6-hydroxy-1,4-diazahexane, 4b-4**

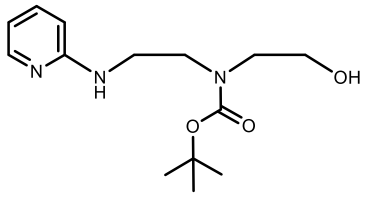


To a solution of **4b-3** (1.203 g, 6.64 mmol, 1.0 equiv) in lab grade THF (12.1 mL) at 0 °C opened to the air was added over 2 min a solution of $Boc_2O$ (1.449 g, 6.64 mmol, 1.0 equiv) in THF (10.0 mL). The flask was fitted with a septum which was pierced with a needle (to vent $CO_2$). The mixture was allowed to warm to room temperature and stirred for 20 h before being concentrated *in vacuo*. Flash chromatography (Biotage, 45 g Zip Sphere column, MeOH/$CH_2Cl_2$ gradient from 1:99 to 6:94) gave **4b-4** (1.544 g, 83%) as a colourless gum. **TLC** – $R_f$ = 0.13 (5:95 MeOH/$CH_2Cl_2$). **$^1$H NMR** (400 MHz, $CDCl_3$) $\delta_H$ (~1:1 mixture of Boc rotamers; *indicates resolved rotamers) [1.32* (s) & 1.37* (s), 9H in total], 3.30 – 3.81 (m, 8H), [4.47* (bs) & 5.32* (bs), 1H in total], 4.91 – 5.09 (m, 1H), 6.37 (d, *J* = 8.4 Hz, 1H), 6.46 – 6.56 (m, 1H), 7.28 – 7.39 (m, 1H), 7.92 – 8.03 (m, 1H). **$^{13}$C NMR** (101 MHz, $CDCl_3$) $\delta_C$ (~1:1 mixture of Boc rotamers; *indicates resolved rotamers:) 28.4, 40.9*, 41.2*, 48.8*, 49.5*, 51.8*, 53.3*, 61.3*, 61.9*, 79.9*, 80.3*, 107.8*, 109.0*, 112.5*, 112.9*, 137.5, 147.0*, 147.8*, 156.4*, 156.8,* 158.5*, 158.8*. **HR-MS** (ESI, positive ion mode) – *m/z* for $[C_{14}H_{23}N_3O_3+H]^+$ = 282.1812. Found 282.1802.

**1-(2-Pyridyl)-4-(*tert*-butoxycarbonyl)-6-(*tert*-butyldimethylsilylhydroxy)-1,4-diazahexane, 4b-5**

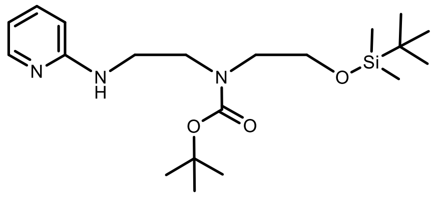


To a solution of **4b-4** (1.425 g, 5.06 mmol, 1.0 equiv) and $Et_3N$ (0.85 mL, 6.08 mmol, 1.2 equiv) in anhydrous DMF (6.1 mL) at 0 °C under $N_2$ was added a solution of TBSCl (763.4 mg, 5.06 mmol, 1.0 equiv) in DMF (4.0 mL). The ice bath was removed and the mixture was stirred at room temperature for 2 h. Additional $Et_3N$ (0.14 mL, 1.01 mmol, 0.2 equiv) and a solution of TBSCl (76.3 mg, 0.51 mmol, 0.1 equiv) in DMF (0.5 mL) were added and stirring continued for a further 1.5 h. Water (75 mL) was added and the product was extracted with $Et_2O$ (50 mL + 70 mL). The combined organic extracts were dried ($Na_2SO_4$) and concentrated. Flash chromatography (Biotage, 30 g Zip Sphere column, EtOAc/petroleum ether gradient from 0:100 to 40:60) gave **4b-5** (1.685 g, 84%) as a colourless gum. **TLC** – $R_f$ = 0.48 (25:75 EtOAc/petroleum ether). **$^1$H NMR** (400 MHz, $CDCl_3$) $\delta_H$ (~1:1 mixture of Boc rotamers; *indicates resolved rotamers) 0.03 (s, 6H), 0.87 (s, 9H), [1.41* (s) & 1.44* (s), 9H in total], 3.25 – 3.36 (m, 2H), 3.42 – 3.55 (m, 4H), 3.64 – 3.78 (m, 2H), [4.80 – 4.88* (m) & 5.08 – 5.16* (m), 1H in total], 6.38 (d, *J* = 8.4 Hz, 1H), 6.47 – 6.57 (m, 1H), 7.32 – 7.39 (m, 1H), 8.03 – 8.07 (m, 1H). **$^{13}$C NMR** (101 MHz, $CDCl_3$) $\delta_C$ (~1:1 mixture of Boc rotamers; *indicates resolved rotamers) –5.3, 18.4, 26.0, 28.5, 40.8*, 41.4*, 47.9*, 48.1*, 50.28*, 50.34*, 61.8, 79.9, 107.2*, 107.5*, 112.6*, 112.9*, 137.26*, 137.33*, 148.1*, 148.2*, 155.7*, 156.6*, 158.6*, 158.8*. **HR-MS** (ESI, positive ion mode) – *m/z* for $[C_{20}H_{37}N_3O_3Si+H]^+$ = 396.2677. Found 396.2669.

**1-(2-Pyridyl)-1-(4-methoxyanilinylcarbonyl)-4-(*tert*-butoxycarbonyl)-6-(*tert*-butyldimethylsilylhydroxy)-1,4-diazahexane, 4b-6**

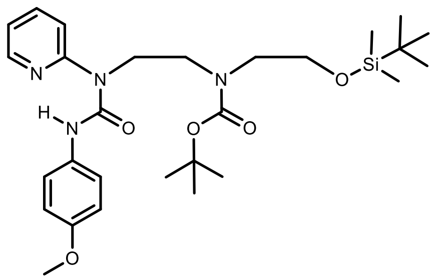

To a solution of **4b-5** (1.054 g, 2.66 mmol, 1.0 equiv) in lab grade 1,2-dichloroethane (3.0 mL) at room temperature under air was added a solution of 4-methoxyphenyl isocyanate (397.4 mg, 2.66 mmol, 1.0 equiv) in 1,2-dichloroethane (2.3 mL). The mixture was heated to 40 °C (closed system: round-bottom flask with septum) and maintained at this temperature for 20 h. After cooling to room temperature, MeOH (~5 mL) was added and the mixture was stirred for 30 min to quench any unreacted isocyanate. The mixture was concentrated *in vacuo*. Flash chromatography (Biotage, 30 g Zip Sphere column, EtOAc/petroleum ether gradient from 0:100 to 40:60) gave **4b-6** (1.013 g, 70%) as a yellow gum. **TLC** – $R_f$ = 0.34 (15:85 EtOAc/petroleum ether). **$^1$H NMR** (400 MHz, $CDCl_3$) $\delta_H$ (1.6:1 mixture of Boc rotamers; **indicates resolved *major* rotamer; *indicates resolved *minor* rotamer) 0.04 (s, 6H), 0.87 (s, 9H), 1.48 (s, 9H), 3.40 (dt, *J* = 12.8, 5.9 Hz, 2H), 3.53 – 3.63 (m, 2H), [3.70** (t, *J* = 6.3 Hz) & 3.75 – 3.79* (m), 2H in total], 3.79 (s, 3H), 4.07 – 4.12 (m, 2H), 6.87 (d, *J* = 9.1 Hz, 2H), 6.95 – 7.00 (m, 1H), [7.24 – 7.27* (m) & 7.63** (d, *J* = 8.6 Hz), 1H in total], 7.42 – 7.49 (m, 2H), [7.68* (ddd, *J* = 8.9, 7.3, 2.0 Hz) & 7.77** (ddd, *J* = 9.1, 7.3, 2.0 Hz), 1H in total], [8.27** (dd, *J* = 5.0, 2.0 Hz) & 8.31* (dd, *J* = 5.0, 2.0 Hz), 1H in total]; *Note:* For this compound, the spectral width of the $^1$H NMR experiment was insufficient to observe the NH proton, which typically resonates at ~12.5 ppm in $CDCl_3$. **$^{13}$C NMR** (101 MHz, $CDCl_3$) $\delta_C$ (1.6:1 mixture of Boc rotamers; **indicates resolved *major* rotamer; *indicates resolved *minor* rotamer) –5.2, 18.4, 26.0, 28.6, 43.3**, 43.7*, 46.4, 50.4*, 50.8**, 55.6, 61.4, 79.8**, 80.0*, 111.8*, 113.0**, 114.2, 117.3*, 117.4**, 122.37**, 122.43*, 132.2*, 132.4**, 139.0*, 139.4**, 145.5**, 145.8*, 153.9*, 154.2**, 155.4*, 155.7, 155.8**, 155.9*, 156.1**. **HR-MS** (ESI, positive ion mode) – *m/z* for $[C_{28}H_{44}N_4O_5Si+H]^+$ = 545.3154. Found 545.3181.

Unreacted starting material **4b-5** (315.2 mg, 30%) was recovered from a subsequent column fraction.

**1-(2-Pyridyl)-1-(4-methoxyanilinylcarbonyl)-4-(*tert*-butoxycarbonyl)-6-hydroxy-1,4-diazahexane, 4b-7**

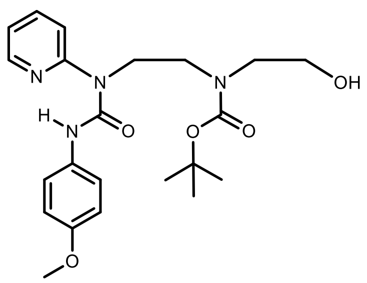

To a solution of **4b-6** (974.9 mg, 1.79 mmol, 1.0 equiv) in lab grade THF (1.5 mL) at room temperature under air was added a commercial solution of TBAF (1.0 M in THF, 4.5 mL, 4.5 mmol, 2.5 equiv). The flask was fitted with a septum and the mixture was stirred at room temperature under $N_2$ for 40 min. The reaction mixture (including solvent) was directly loaded on to a silica column (21.5 g silica) and elution was performed with THF (~150 mL) to remove most of the ammonium salts. The eluent was concentrated *in vacuo*. Flash chromatography (Biotage, 10 g SNAP Ultra column, EtOAc/petroleum ether gradient from 40:60 to 100:0) gave **4b-7** (746.7 mg, 97%) as a colourless gum. **TLC** – $R_f$ = 0.21 (60:40 EtOAc/petroleum ether). **$^1$H NMR** (400 MHz, $CDCl_3$) $\delta_H$ (1.2:1 mixture of Boc rotamers; **indicates resolved *major* rotamer; *indicates resolved *minor* rotamer) [1.47* (s) & 1.48** (s), 9H in total], 2.77 (bs, 1H), 3.44 – 3.61 (m, 4H), 3.73 – 3.83 (m, 2H), 3.78 (s, 3H), 4.08 – 4.16 (m, 2H), 6.86 (d, *J* = 9.0 Hz, 2H), 6.96 – 7.03 (m, 1H), [7.24** (d, *J* = 9.0 Hz) & 7.57* (d, *J* = 8.6 Hz), 1H in total], 7.40 – 7.46 (m, 2H), [7.70** (t, *J* = 7.3 Hz) & 7.78* (t, *J* = 8.1 Hz), 1H in total], [8.28* (d, *J* = 5.5 Hz) & 8.31** (d, *J* = 5.2 Hz), 1H in total]; *Note:* For this compound, the spectral width of the $^1$H NMR experiment was insufficient to observe the NH proton, which typically resonates at ~12.5 ppm in $CDCl_3$. **$^{13}$C NMR** (101 MHz, $CDCl_3$) $\delta_C$ (1.2:1 mixture of Boc rotamers; **indicates resolved *major* rotamer; *indicates resolved *minor* rotamer) 28.6, 43.6*, 43.9**, 46.3, 51.5, 55.6, 61.8*, 62.5**, 80.3*, 80.8**, 111.7**, 112.8*, 114.2, 117.5, 122.5*, 122.6**, 131.9**, 132.1*, 139.0**, 139.6*, 145.5*, 145.9**, 154.1**, 154.4*, 155.5, 155.9*, 156.0, 157.0**. **HR-MS** (ESI, positive ion mode) – *m/z* for $[C_{22}H_{30}N_4O_5+H]^+$ = 431.2289. Found 431.2282.

**1-(2-Pyridyl)-1-(4-methoxyanilinylcarbonyl)-4-(*tert*-butoxycarbonyl)-6-oxo-1,4-diazahexane, 4b-8**

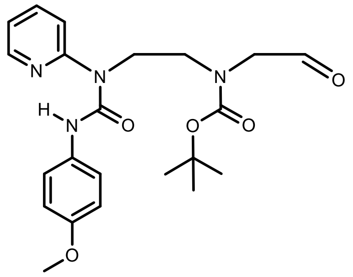

To a solution of **4b-7** (662.6 mg, 1.54 mmol, 1.0 equiv) in lab grade $CH_2Cl_2$ (15.4 mL) at 0 °C under air was added solid Dess-Martin periodinane (783.4 mg, 1.85 mmol, 1.2 equiv). The mixture was maintained at 0–5 °C for 3 h, then allowed to warm to room temperature over a further 3 h. The mixture was re-cooled to 0 °C and $Et_2O$ (50 mL) was added. The suspension was gravity filtered and the reaction flask and filter cake were rinsed/washed with $Et_2O$ (25 mL). The filtrate was washed with saturated $NaHCO_3$ (30 mL), dried ($Na_2SO_4$) and concentrated. The crude product was passed through a silica plug (4 mL of silica in a 10 mL syringe cartridge) with $Et_2O$ (~30 mL) to give **4b-8** (619.1 mg, 94%) as a pale yellow foam, containing minor

impurities by NMR and used without further purification. **TLC** – $R_f$ = 0.29–0.64 [streaks] (60:40 EtOAc/petroleum ether). **$^1$H NMR** (400 MHz, $CDCl_3$) $\delta_H$ (1.8:1 mixture of Boc rotamers; **indicates resolved *major* rotamer; *indicates resolved *minor* rotamer) [1.42** (s) & 1.48* (s), 9H in total], 3.56 – 3.64 (m, 2H), 3.78 (s, 3H), 4.05 – 4.13 (m, 4H), 6.86 (d, *J* = 9.0 Hz, 2H), 6.97 – 7.03 (m, 1H), [7.22* (d, *J* = 8.6 Hz) & 7.53** (d, *J* = 8.6 Hz), 1H in total], 7.38 – 7.44 (m, 2H), [7.68 – 7.73* (m) & 7.79** (ddd, *J* = 9.0, 7.3, 2.0 Hz), 1H in total], [8.28** (dd, *J* = 5.1, 1.9 Hz) & 8.32* (dd, *J* = 5.0, 2.0 Hz), 1H in total], [9.56** (s) & 9.57* (s), 1H in total], [12.49* (s) & 12.52** (s), 1H in total]. **$^{13}$C NMR** (101 MHz, $CDCl_3$) $\delta_C$ (1.8:1 mixture of Boc rotamers; **indicates resolved *major* rotamer; *indicates resolved *minor* rotamer) 28.3**, 28.5*, 43.5**, 43.7*, 46.4*, 47.0**, 55.6, 58.2*, 59.0**, 81.1**, 81.2*, 111.7*, 112.5**, 114.2, 117.6, 122.56**, 122.63*, 131.8*, 132.0**, 139.1*, 139.6**, 145.6**, 146.0*, 154.0*, 154.2**, (155.4, 155.49, 155.55; unable to assign major/minor rotamers in this region due to overlapping signal/s), 156.0**, 156.1*, 198.2. **HR-MS** (ESI, positive ion mode) – *m/z* for $[C_{22}H_{28}N_4O_5+H]^+$ = 429.2132. Found 429.2132.

**1-(2-Pyridyl)-1-(4-methoxyanilinylcarbonyl)-4-(*tert*-butoxycarbonyl)-10-trifluoroacetyl-1,4,7,10-tetraazadecane, 4b-9**

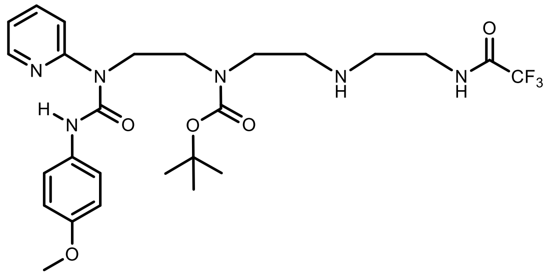


To a reaction vial containing neat aldehyde **4b-8** (230.7 mg, 0.54 mmol, 1.0 equiv) was added sequentially amine·HCl salt **4b-2** (155.5 mg, 0.81 mmol, 1.5 equiv), lab grade 1,2-dichloroethane (1.8 mL), $Et_3N$ (0.11 mL, 0.81 mmol, 1.5 equiv) and solid $NaBH(OAc)_3$ (159.8 mg, 0.75 mmol, 1.4 equiv). The mixture was stirred at room temperature under air for 4 h. Saturated $NaHCO_3$ (15 mL) was added and the product was extracted with $CH_2Cl_2$ (30 mL + 20 mL) then the combined organic extracts were dried ($Na_2SO_4$) and concentrated. Flash chromatography (Biotage, 10 g Sfär Duo column, MeOH/$CH_2Cl_2$ gradient from 0:100 to 10:90) gave **4b-9** (162.0 mg, 53%) as a colourless gum. **TLC** – $R_f$ = 0.38 (7.5:92.5 MeOH/$CH_2Cl_2$). **$^1$H NMR** (400 MHz, $CDCl_3$) $\delta_H$ (~1:1 mixture of Boc rotamers; *indicates resolved rotamers) [1.49* (s) & 1.53* (s), 9H in total], 2.79 – 2.88 (m, 4H), 3.38 – 3.55 (m, 6H), 3.80 (s, 3H), 4.11 – 4.20 (m, 2H), 6.87 (d, *J* = 9.0 Hz, 2H), 6.97 – 7.04 (m, 1H), 7.41 (d, *J* = 8.6 Hz, 2H), 7.62 – 7.74 (m, 2H), 7.79 – 7.85* (m, 0.5H), 8.27 – 8.34 (m, 1H), [12.59* (s) & 12.72* (s), 1H in total]; *Note:* An ArH signal of one rotamer (of integration 0.5H) is likely obscured by the $CHCl_3$ resonance. **$^{19}$F NMR** (376 MHz, $CDCl_3$) $\delta_F$ (~1:1 mixture of resolved Boc rotamers) [–75.7 (s) & –75.6 (s), 3F in total].

**1-(2-Pyridyl)-1,4,7-tris(4-methoxyanilinylcarbonyl)-1,4,7,10-tetraazadecane, 4b-10**

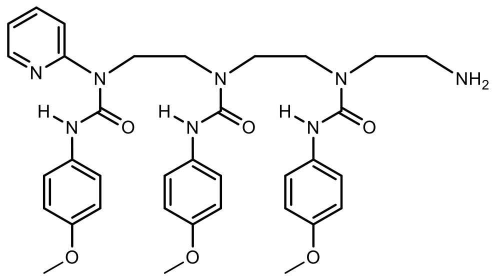


***Step 1** (Boc deprotection):* To a solution of **4b-9** (162.0 mg, 0.28 mmol, 1.0 equiv) in lab grade $CH_2Cl_2$ (2.85 mL) at room temperature under air was added TFA (0.65 mL, 8.55 mmol, 30.0 equiv) and the mixture was stirred at room temperature for 16 h. After cooling to 0 °C, saturated $NaHCO_3$ (20 mL) was slowly added to the open flask over 5 min with stirring. The product was extracted with $CH_2Cl_2$ (2 × 25 mL + 15 mL) and the combined organic extracts were dried ($Na_2SO_4$) and concentrated to give the crude product (151.0 mg, 113% of theoretical) as a brown oil. **TLC** – $R_f$ = 0.40 (10:2:88 MeOH/[35% aqueous $NH_3$]/$CH_2Cl_2$). ***Step 2** (bis-urea formation):* The crude product from *Step 1* (≤0.28 mmol, 1.0 equiv) was taken up in lab grade $CH_2Cl_2$ (1.0 mL) and a solution of 4-methoxyphenyl isocyanate (89.2 mg, 0.60 mmol, 2.1 equiv) in $CH_2Cl_2$ (1.8 mL) was added. The mixture was stirred at room temperature under air for 2.5 h. MeOH (3 mL) was added and the mixture was stirred for 30 min to quench any unreacted isocyanate (potentially producing the corresponding ‘non-polar’ methyl carbamate as a by-product). The mixture was concentrated *in vacuo*. Flash chromatography (Biotage, 10 g Sfär Duo column, MeOH/$CH_2Cl_2$ gradient from 0:100 to 6.5:93.5) gave the desired product (174.8 mg, 80% over two steps) as a white solid. **TLC** – $R_f$ = 0.35 (5:95 MeOH/$CH_2Cl_2$). ***Step 3** (trifluoroacetamide hydrolysis):* The product from *Step 2* (174.8 mg, 0.23 mmol, 1.0 equiv) was dissolved in lab grade THF (1.14 mL) containing a small amount of DMF for solubility (8 drops from a Pasteur pipette). To this was added EtOH (2.3 mL) and a solution of NaOH (62.0 mg, 1.82 mmol, 8.0 equiv in water (1.14 mL). The mixture was stirred at room temperature under air for 24 h. Most of the solvents were removed *in vacuo*, then water (20 mL) was added. The product was extracted with $CH_2Cl_2$ (40 mL + 20 mL) then the combined organic extracts were dried ($Na_2SO_4$) and concentrated. Flash chromatography (Biotage, 10 g Sfär Duo column, MeOH/[35% aqueous $NH_3$]/$CH_2Cl_2$ gradient from 0:0:100 to 10:2:88) gave **4b-10** (133.4 mg, 70% over three steps) as a white solid. **TLC** – $R_f$ = 0.50 (10:2:88 MeOH/[35% aqueous $NH_3$]/$CH_2Cl_2$). **$^1$H NMR** (400 MHz, $CDCl_3$) $\delta_H$ 2.86 – 2.94 (m, 2H), 3.32 – 3.50 (m, 4H), 3.57 – 3.70 (m, 4H), 3.75 (s, 3H), 3.77 (s, 3H), 3.78 (s, 3H), 4.06 – 4.14 (m, 2H), 6.77 – 6.91 (m, 6H), 6.97 (t, *J* = 6.1 Hz, 1H), 7.32 (d, *J* = 8.5 Hz, 2H), 7.47 (d, *J* = 8.4 Hz, 2H), 7.61 (d, *J* = 8.6 Hz, 2H), 7.77 (t, *J* = 8.0 Hz, 1H), 8.27 (d, *J* = 4.9 Hz, 1H), 9.03 (s, 1H), 10.10 (bs, 1H), 12.62 (bs, 1H); *Note:* One signal of the pyridine ring (ArH3) is too broad to be identified. **$^{13}$C NMR** (101 MHz, $CDCl_3$) $\delta_C$ 41.8, 44.3, 46.5, 47.0, 48.0, 52.7, 55.5 (3 × unresolved O$CH_3$), 112.8, 113.9, 114.0, 114.1, 117.6, 120.6, 121.4, 122.4, 131.9, 133.4, 133.5, 139.7, 145.4, 154.4, 155.0, 155.1, 155.3, 155.9, 156.4, 158.4. **HR-MS** (ESI, positive ion mode) – *m/z* for $[C_{35}H_{42}N_8O_6+H]^+$ = 671.3300. Found 671.3271.

**1-(2-Pyridyl)-1,4,7,10-tetrakis(4-methoxyanilinylcarbonyl)-10-benzyl-1,4,7,10-tetraazadecane, 4b**

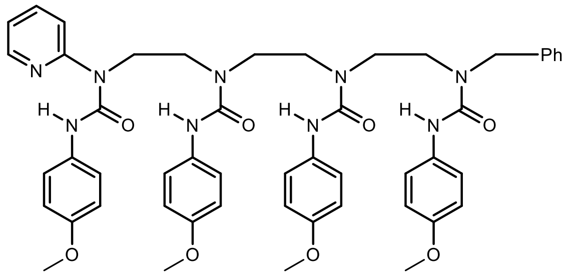

***Step 1*** *(reductive amination):* To a solution of **4b-10** (33.5 mg, 0.050 mmol, 1.0 equiv) in lab grade MeOH (0.5 mL) was added a solution of benzaldehyde (5.3 mg weighed, 0.050 mmol, 1.0 equiv) in lab grade THF (0.5 mL) and the mixture was stirred under air at room temperature for 16.5 h. The mixture was cooled to 0 °C and solid $NaBH_4$ (5.0 mg, 0.13 mmol, 2.6 equiv) was added. After 2 min at 0 °C, the ice bath was removed and the mixture was stirred at room temperature for 3 h. 1 M $K_2CO_3$ (5 mL) and water (15 mL) were added. The product was extracted with $CH_2Cl_2$ (25 mL + 15 mL) then the combined organic extracts were dried ($Na_2SO_4$) and concentrated. Flash chromatography (Biotage, 5 g Sfär Duo column, MeOH/$CH_2Cl_2$ gradient from 0:100 to 8:92) gave the product (32.1 mg, 84%) as a white solid. **TLC** – $R_f$ = 0.50 (10:90 MeOH/$CH_2Cl_2$). ***Step 2*** *(urea formation):* The product from *Step 1* (32.1 mg, 0.042 mmol, 1.0 equiv) was dissolved in lab grade $CH_2Cl_2$ (0.34 mL) and a solution of 4-methoxyphenyl isocyanate (6.3 mg, 0.042 mmol, 1.0 equiv) in $CH_2Cl_2$ (0.5 mL) was added. The mixture was stirred at room temperature under air for 30 min. MeOH (5 mL) was added and the mixture was stirred for 5 min to quench any unreacted isocyanate. The mixture was concentrated *in vacuo*. Flash chromatography (Biotage, 5 g Sfär Duo column, MeOH/$CH_2Cl_2$ gradient from 0:100 to 6:94) gave **4b** (37.5 mg, 82% over two steps) as a white solid. **TLC** – $R_f$ = 0.49 (5:95 MeOH/$CH_2Cl_2$). **$^1$H NMR** (400 MHz, $CD_2Cl_2$) $\delta_H$ 3.41 – 3.56 (m, 8H), 3.62 – 3.68 (m, 2H), 3.77 (s, 3H), 3.79 (s, 3H), 3.796 (s, 3H), 3.801 (s, 3H), 4.04 – 4.10 (m, 2H), 4.62 (s, 2H), 6.80 – 6.92 (m, 8H), 7.09 (dd, *J* = 7.4, 5.0 Hz, 1H), 7.26 – 7.41 (m, 5H), 7.48 – 7.52 (m, 4H), 7.61 – 7.65 (m, 4H), 7.81 (ddd, *J* = 8.5, 7.3, 2.0 Hz, 1H), 8.38 (dd, *J* = 5.3, 1.9 Hz, 1H), 8.66 (very bs, 1H), 9.07 (overlapping singlets, 2H, 2 × N*H*), 12.31 (bs, 1H) ; *Note:* One signal of the pyridine ring (ArH3) is too broad to be identified. **$^{13}$C NMR** (126 MHz, $CD_2Cl_2$) δ 45.5 – 52.5 (7 × broad semi-resolved signals from N*C*$H_2$), 55.91, 55.92, 55.94, 55.95, 112.9, 114.3, 114.4, 114.5, 118.8, 121.6, 121.7, 122.0, 122.6, 127.7, 127.9, 129.3, 132.2, 133.8, 134.0, 140.1, 146.7, 155.0, 155.70, 155.74, 155.9, 156.6, 156.8, 157.0, 157.1. **HR-MS** (ESI, positive ion mode) – *m/z* for $[C_{50}H_{55}N_9O_8+H]^+$ = 910.4246. Found 910.4268.

**Dibromomaleic anhydride, 7a-1**

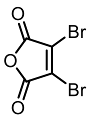

Under a dry, inert atmosphere, bromine (1.57 mL, 30.6 mmol, 2.00 equiv) was added to maleic anhydride (1500 mg, 15.3 mmol, 1.00 equiv) in a microwave vial. To this solution was added aluminium trichloride (300 mg, 2.3 mmol, 0.15 equiv) and the vial was sealed and heated to 160 °C for 20 h behind a blast shield. The suspension was cooled to room temperature and opened to air. The suspension was diluted with EtOAc (25 mL) and filtered, and the residue was washed

with EtOAc (10 mL). The filtrate was then concentrated *in vacuo* to give the desired anhydride as a white solid (2732 mg, 10.7 mmol, 70%). a Spectroscopic data matched that previously reported.[9]

**Allyl dibromomaleimide, 7a-2**

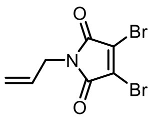


Under a dry, inert atmosphere, allylamine (0.72 mL, 9.58 mmol, 1.00 equiv, 0.10 M) was added to a solution of **7a-1** (2.45 g, 9.58 mmol, 1.00 equiv) in AcOH (96 mL). The solution was heated to reflux for 5 h, after which time the AcOH was removed by distillation. The crude product was purified by column chromatography (5-20% EtOAc:PE) to give the title maleimide as a white powder (2.51 g, 8.51 mmol, 89%). **M.P. –** 96-97 °C (DCM). **TLC** – $R_f$ = 0.65 ($SiO_2$, 20:80 EtOAc:PE). **$^1$H NMR** (400 MHz, $CDCl_3$) – 4.20 (dt, J = 1.4, 5.9, 2H, NC*H*$_2$), 5.20-5.28 (m, 2H, C=C*H*$_2$), 5.79 (ddq, J = 5.9, 10.2, 17.0, 1H, C=C*H*). **$^{13}$C NMR** (101 MHz, $CDCl_3$) – 41.8 (*C*H$_2$), 119.1 (C=*C*H$_2$), 129.5 (2 x C=*C*Br), 130.6 (C=*C*H), 163.6 (2 x *C*O). **HR-MS** (ESI, positive ion mode) – *m/z* for $[C_7H_6Br_2NO_2+H]^+$ = 293.8765. Found 293.8760. **FTIR** (neat) – 2932, 1783, 1713.

**Allyl di(*n*-butylmercapto)maleimide, 7a**

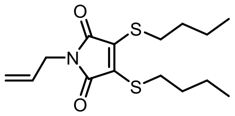


Under a dry, inert atmosphere, triethylamine (0.99 mL, 7.12 mmol, 2.10 equiv) was added dropwise to a solution of **7a-2** (1.0 g, 3.39 mmol, 1.00 equiv, 0.10 M) in anhydrous $Et_2O$ (34 mL). To this solution was added 1-butanethiol (0.76 mL, 7.12 mmol, 2.10 equiv) dropwise and a yellow precipitate formed. The suspension was stirred at room temperature for 16 h. The suspension was then concentrated *in vacuo* and the crude product was purified by column chromatography (5-20% $Et_2O$:PE) to give the title bis(sulfide) as a yellow oil (814 mg, 2.60 mmol, 77%). **TLC** – $R_f$ = 0.60 ($SiO_2$, 20:80 $Et_2O$:PE). **$^1$H NMR** (400 MHz, $CDCl_3$) $\delta_H$ 0.92 (t, J = 7.3, 6H, 2 x C*H*$_3$), 1.43-1.45 (m, 4H, 2 x C*H*$_2$), 1.60-1.63 (m, 4H, 2 x C*H*$_2$), 3.29 (t, J = 7.5, 4H, 2 x SC*H*$_2$), 4.10 (dd, J = 1.3, 5.7, 2H, NC*H*$_2$), 5.14-5.22 (m, 2H, C=C*H*$_2$), 5.79 (ddt, J = 5.9, 10.2, 15.9, 1H, C=C*H*). **$^{13}$C NMR** (101 MHz, $CDCl_3$) $\delta_C$ 13.7 (2 x *C*H$_3$), 21.7 (2 x *C*H$_2$), 31.6 (2 x S*C*H$_2$), 32.6 (2 x *C*H$_2$), 40.7 (N*C*H$_2$), 117.8 (C=*C*H$_2$), 131.7 (C=*C*H), 135.8 (2 x C=*C*S), 166.3 (2 x *C*O). **HR-MS** (ESI, positive ion mode) – *m/z* for $[C_{15}H_{23}NO_2S_2+H]^+$ = 314.1248. Found 314.1231. **FTIR** (neat) – 2958, 2930, 2872, 1764, 1701.

**2-Oxoethyl-di(*n*-butylmercapto)maleimide, 7a-3**

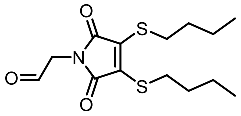


Synthesised according to general procedure L using **7a** (250 mg, 0.70 mmol). The crude product was purified by column chromatography (20-100% $Et_2O$:PE) to give the title aldehyde as a yellow oil (123 mg, 0.39 mmol, 56%). **TLC** – $R_f$ = 0.08 ($SiO_2$, 20:80 $Et_2O$:PE). **$^1$H NMR** (500 MHz, $CDCl_3$) $\delta_H$ 0.92 (t, J = 7.3, 6H, 2 x C*H3*), 1.41-1.44 (m, 4H, 2 x C*H2*), 1.62-1.64 (m, 4H, 2 x C*H2*), 3.28 (t, J = 7.4, 2 x SC*H2*), 4.36 (s, 2H, NC*H2*), 9.56 (s, 1H, C*H*O). **$^{13}$C NMR** (126 MHz, $CDCl_3$) $\delta_C$ 13.5 (2 x *C*$H_3$), 21.6 (2 x *C*$H_2$), 31.6 (2 x S*C*$H_2$), 32.5 (2 x *C*$H_2$), 47.7 (N*C*$H_2$), 136.1 (C=*C*S), 165.8 (2 x *C*O), 193.6 (*C*HO). **HR-MS** (ESI, positive ion mode) – *m/z* for $[C_{14}H_{22}NO_3S_2+H]^+$ = 316.1041. Found 316.1016. **FTIR** (neat) – 2957, 2930, 1701.

**1-(2-Pyridyl)-10-((di(*n*-butylmercapto)maleimido)ethyl)-1,4,7,10-tetrakis(4-methoxyanilinylcarbonyl)-1,4,7,10-tetraazadecane, 8a**

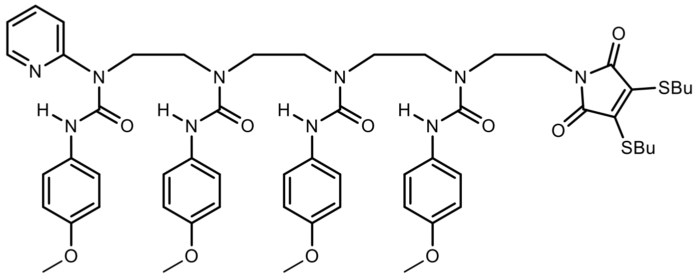


***Step 1*** *(reductive amination):* To a solution of amine **4b-10** (95.9 mg, 0.14 mmol, 1.0 equiv) in lab grade 1,2-dichloroethane (0.4 mL) at room temperature was added a solution of aldehyde **7a-3** (45.1 mg, 0.14 mmol, 1.0 equiv) in 1,2-dichloroethane (1.0 mL). Solid $NaBH(OAc)_3$ (42.4 mg, 0.20 mmol, 1.4 equiv) was added and the mixture was stirred at room temperature under air for 5 h. Saturated $NaHCO_3$ (8 mL) and water (15 mL) were added. The product was extracted with $CH_2Cl_2$ (30 mL + 10 mL) then the combined organic extracts were dried ($Na_2SO_4$) and concentrated. Flash chromatography (Biotage, 10 g Sfär Duo column, MeOH/$CH_2Cl_2$ gradient from 0:100 to 7:93) gave **8a-1** (94.0 mg, 68%) as a yellow semi-solid. **TLC** – $R_f$ = 0.35 (5:95 MeOH/$CH_2Cl_2$). ***Step 2*** *(urea formation):* The product from *Step 1* (56.5 mg, 0.058 mmol, 1.0 equiv) was dissolved in lab grade $CH_2Cl_2$ (0.5 mL) and a solution of 4-methoxyphenyl isocyanate (8.7 mg, 0.058 mmol, 1.0 equiv) in $CH_2Cl_2$ (0.7 mL) was added. The mixture was stirred at room temperature under air for 40 min. MeOH (1 mL) was added and the mixture was stirred for 5 min to quench any unreacted isocyanate. The mixture was concentrated *in vacuo*. Flash chromatography (Biotage, 5 g Sfär Duo column, MeOH/$CH_2Cl_2$ gradient from 0:100 to 4:96) gave **8a** (55.5 mg, 85%) as a yellow solid. **TLC** – $R_f$ = 0.49 (5:95 MeOH/$CH_2Cl_2$). **$^1$H NMR** (400 MHz, $CD_2Cl_2$) $\delta_H$ 0.90 (t, *J* = 7.3 Hz, 6H), 1.40 (h, *J* = 7.3 Hz, 4H), 1.60 (p, *J* = 7.7 Hz, 4H), 3.25 (t, *J* = 7.4 Hz, 4H), 3.44 – 3.60 (m, 10H), 3.68 – 3.74 (m, 4H), 3.77 (s, 3H), 3.79 (s, 3H), 3.801 (s, 3H), 3.803 (s, 3H), 4.08 – 4.14 (m, 2H), 6.79 – 6.93 (m, 8H), 7.08 (dd, *J* = 7.3, 5.0 Hz, 1H), 7.19 – 7.40 (broad m, 1H), 7.49 – 7.55 (m, 4H), 7.62 – 7.67 (m, 4H), 7.83 (ddd, *J* = 9.1, 7.3, 2.0 Hz, 1H), 8.38 (dd, *J* = 5.2, 1.9 Hz, 1H), 8.61 (very bs,

1H), 9.09 (bs, 1H), 9.12 (bs, 1H), 12.38 (bs, 1H). **$^{13}$C NMR** (126 MHz, $CD_2Cl_2$) $\delta_C$ 13.9, 22.2, 32.2, 33.0, 37.4, 45.5 – 49.8 (7 × broad semi-resolved signals from N*C*$H_2$), 55.90, 55.92, 55.94, 56.0, 113.0, 114.26, 114.30, 114.4, 114.5, 118.7, 121.6, 121.67, 121.71, 122.6, 132.3, 133.9, 134.0, 136.8, 140.2, 146.6, 155.0, 155.7, 155.8, 156.2, 156.6, 157.1, 167.4. **HR-MS** (ESI, positive ion mode) – *m/z* for $[C_{57}H_{70}N_{10}O_{10}S_2+H]^+$ = 1119.4791. Found 1119.4750.

**1-(2-Pyridyl)-4-(dimethylaminocarbonyl)-4,10-diazadecane, 8b-1**

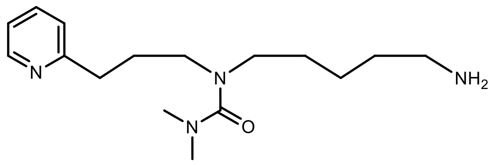


*Preparation of aldehyde for reductive amination*: To a solution of 3-(2-pyridyl)-1-propanol (214.1 mg, 1.56 mmol, 1.0 equiv) in lab grade $CH_2Cl_2$ (15.6 mL) at 0 °C under air was added solid Dess-Martin periodinane (728.2 mg, 1.72 mmol, 1.1 equiv) and the mixture was allowed to warm to room temperature over 3 h. The mixture was re-cooled to 0 °C and $Et_2O$ (50 mL) was added. The suspension was gravity filtered and the reaction flask and filter cake were rinsed/washed with $Et_2O$ (15 mL). The filtrate was washed with saturated $NaHCO_3$ (20 mL), dried ($Na_2SO_4$) and concentrated. Flash chromatography (Biotage, 5 g Sfär Duo column, MeCN/$CH_2Cl_2$ gradient from 0:100 to 100:0) gave 3-(2-pyridyl)propanal (41.0 mg, 19%) as a light brown oil. **TLC** – $R_f$ = 0.11 (MeCN). **$^1$H NMR** (400 MHz, $CDCl_3$) $\delta_H$ 2.95 (tdd, *J* = 7.1, 1.3, 0.7 Hz, 2H), 3.14 (t, *J* = 7.1 Hz, 2H), 7.09 – 7.15 (m, 1H), 7.19 (d, *J* = 7.8 Hz, 1H), 7.59 (td, *J* = 7.7, 1.8 Hz, 1H), 8.51 (ddd, *J* = 4.9, 1.9, 0.9 Hz, 1H), 9.87 (t, *J* = 1.3 Hz, 1H). Spectroscopic data matches that previously reported.[10] ***Step 1** (reductive amination):* To neat 3-(2-pyridyl)propanal (41.0 mg, 0.30 mmol, 1.0 equiv) was added a solution of *N*-Boc-cadaverine (61.4 mg, 0.30 mmol, 1.0 equiv) in lab grade 1,2-dichloroethane (3 mL). Solid $NaBH(OAc)_3$ (90.0 mg, 0.42 mmol, 1.4 equiv) was added and the mixture was stirred at room temperature under air for 2 h. Aqueous $K_2CO_3$ (8 mL, 1.0 M) and water (15 mL) were added. The product was extracted with $CH_2Cl_2$ (25 mL + 15 mL) then the combined organic extracts were dried ($Na_2SO_4$) and concentrated. Flash chromatography (Biotage, 5 g Sfär Duo column, MeOH/[35% aqueous $NH_3$]/$CH_2Cl_2$ gradient from 0:0:100 to 8:1.6:90.4) gave a colourless gum (61.7 mg, 63% if pure) containing the desired product with a significant higher $R_F$ impurity, presumably from di-alkylation. **TLC** – $R_f$ (desired product) = 0.20 (10:2:88 MeOH/[35% aqueous $NH_3$]/$CH_2Cl_2$, UV or ninhydrin stain). ***Step 2** (urea formation):* The impure product from *Step 1* (61.7 mg, 0.19 mmol if pure, 1.0 equiv) was dissolved in lab grade 1,2-dichloroethane (0.96 mL) and $Et_3N$ (54 µL, 0.38 mmol, 2.0 equiv) and dimethylcarbamoyl chloride (27 µL, 0.29 mmol, 1.5 equiv) were sequentially added. The mixture was heated at 45 °C under air (closed system: flask with septum) for 16 h. The mixture was concentrated *in vacuo*. Flash chromatography (Biotage, 5 g Sfär Duo column, MeOH/$CH_2Cl_2$ gradient from 0:100 to 5:95) gave the product (~9 mg, ~8% over two steps) as a colourless gum. **TLC** – $R_f$ = 0.53 (7.5:92.5 MeOH/$CH_2Cl_2$). ***Step 3** (Boc deprotection):* The product from *Step 2* was dissolved in lab grade $CH_2Cl_2$ (0.47 mL) and TFA (54 µL, 0.70 mmol, ~30 equiv) was

added. The mixture was stirred under air at room temperature for 2 h. 1 M $K_2CO_3$ (5 mL) was added and the product was extracted with $CH_2Cl_2$ (25 mL + 15 mL). The combined organic extracts were dried ($Na_2SO_4$) and concentrated to give **8b-1** (7.8 mg, 9% over three steps) as a pale yellow oil. **TLC** – $R_f$ = 0.23 (10:2:88 MeOH/[35% aqueous $NH_3$]/$CH_2Cl_2$). **$^1$H NMR** (400 MHz, $CDCl_3$) $\delta_H$ 1.22 – 1.31 (m, 2H), 1.38 – 1.55 (m, 4H), 1.92 – 2.00 (m, 2H), 2.63 – 2.70 (m, 2H), 2.73 – 2.78 (m, 2H), 2.75 (s, 6H), 3.09 – 3.20 (m, 4H), 7.10 (ddd, $J$ = 7.5, 4.9, 1.2 Hz, 1H), 7.14 (dt, $J$ = 7.8, 1.1 Hz, 1H), 7.58 (td, $J$ = 7.6, 1.8 Hz, 1H), 8.51 (ddd, $J$ = 4.9, 1.9, 1.0 Hz, 1H). **$^{13}$C NMR** (101 MHz, $CDCl_3$) $\delta_C$ 24.4, 28.0, 28.1, 33.6, 35.7, 38.8, 42.2, 47.7, 48.3, 121.2, 123.0, 136.5, 149.4, 161.5, 165.6. **HR-MS** (ESI, positive ion mode) – *m/z* for $[C_{16}H_{28}N_4O+H]^+$ = 293.2336. Found 293.2344.

**1-(2-Pyridyl)-10-((di(*n*-butylmercapto)maleimido)ethyl)-4,10-bis(dimethylaminocarbonyl)-4,10-diazadecane, 8b**

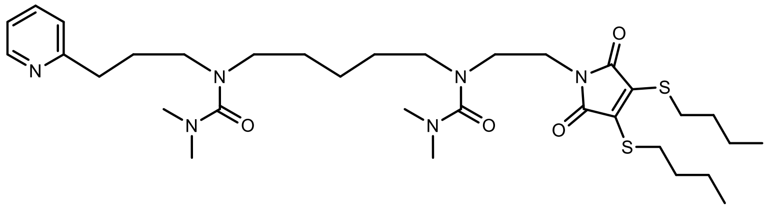


***Step 1** (reductive amination):* To a reaction vial containing amine **8b-1** (6.9 mg, 0.023 mmol, 1.0 equiv) was sequentially aldehyde **7a-3** (7.4 mg, 0.023 mmol, 1.0 equiv), lab grade 1,2-dichloroethane (0.47 mL) and solid $NaBH(OAc)_3$ (7.0 mg, 0.033 mmol, 1.4 equiv). The mixture was stirred under air at room temperature for 2 h. Saturated $NaHCO_3$ (8 mL) was added and the product was extracted with $CH_2Cl_2$ (25 mL + 10 mL). The combined organic extracts were dried ($Na_2SO_4$) and concentrated. Flash chromatography (Biotage, 5 g Sfär Duo column, MeOH/$CH_2Cl_2$ gradient from 0:100 to 8.5:91.5) gave the product (8.4 mg, 60%) as a yellow gum. **TLC** – $R_f$ = 0.44 (10:90 MeOH/$CH_2Cl_2$). ***Step 2** (urea formation):* To the product from *Step 1* (8.4 mg, 0.014 mmol, 1.0 equiv) was added a solution containing $Et_3N$ (2.6 mg, 0.026 mmol, 1.8 equiv) and dimethylcarbamoyl chloride (2.3 mg, 0.021 mmol, 1.5 equiv) in lab grade 1,2-dichloroethane (0.28 mL). The mixture was heated at 45 °C under air (closed system: flask with septum) for 15.5 h. The mixture was concentrated *in vacuo*. Flash chromatography (Biotage, 5 g Sfär Duo column, MeOH/$CH_2Cl_2$ gradient from 0:100 to 7:93) gave **8b** (9.6 mg, quantitative, or 61% over two steps) as a yellow gum. **TLC** – $R_f$ = 0.22 (5:95 MeOH/$CH_2Cl_2$). **$^1$H NMR** (400 MHz, $CDCl_3$) $\delta_H$ 0.92 (t, $J$ = 7.3 Hz, 6H), 1.13 – 1.22 (m, 2H), 1.38 – 1.52 (m, 8H), 1.58 – 1.66 (m, 4H), 1.92 – 2.00 (m, 2H), 2.73 – 2.77 (m, 14H, including 2 × overlapping singlets), 3.03 – 3.18 (m, 6H), 3.25 – 3.32 (m, 6H), 3.62 (t, $J$ = 6.1 Hz, 2H), 7.10 (ddd, $J$ = 7.5, 4.9, 1.1 Hz, 1H), 7.14 (dt, $J$ = 7.8, 1.1 Hz, 1H), 7.58 (td, $J$ = 7.7, 1.9 Hz, 1H), 8.51 (ddd, $J$ = 4.9, 1.9, 1.0 Hz, 1H). **$^{13}$C NMR** (101 MHz, $CDCl_3$) $\delta_C$ 13.7, 21.8, 24.4, 27.9, 28.0, 28.1, 31.7, 32.6, 35.7, 36.8, 38.6, 38.8, 45.9, 47.8, 48.2, 49.7, 121.3, 123.0, 135.9, 136.5, 149.3, 161.5, 164.9, 165.5, 166.6. **HR-MS** (ESI, positive ion mode) – *m/z* for $[C_{33}H_{54}N_6O_4S_2+H]^+$ = 663.3721. Found 663.3704.

**1,13-Bis(trifluoroacetyl)-4,7,10-tris(*n*-butylcarbonyl)-1,4,7,10,13-pentaazatridecane, 9-2**

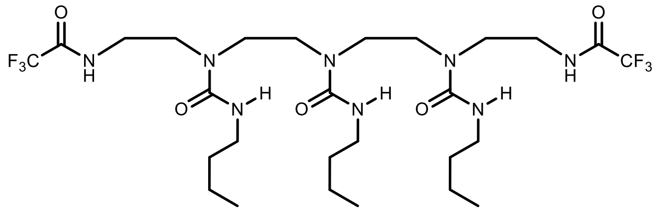


***Step 1*** *(bis-trifluoroacetylation):* To a solution of tetraethylenepentamine (849.4 mg, 4.49 mmol, 1.0 equiv) in lab grade $CH_2Cl_2$ (4.5 mL) under air at 0 °C was added slowly over 30 min a solution of ethyl trifluoroacetate (1.275 g, 8.97 mmol, 2.0 equiv) in $CH_2Cl_2$ (4.5 mL). After complete addition, the solution was allowed to warm to room temperature with stirring over a further 23.5 h. The mixture was concentrated *in vacuo*. Flash chromatography (manual, 25 g silica, MeOH/[35% aqueous $NH_3$]/$CH_2Cl_2$ gradient from 5:1:94 to 15:3:82) gave **9-1** (904.0 mg, 53% if pure) containing unidentified impurities by NMR. **TLC** – $R_f$ = 0.41 (20:4:76 MeOH/[35% aqueous $NH_3$]/$CH_2Cl_2$, ninhydrin stain). ***Step 2*** *(tris-urea formation):* The impure product from *Step 1* (809.0 mg, 2.12 mmol if pure, 1.0 equiv) was dissolved in lab grade 1,2-dichloroethane (6.4 mL) and butyl isocyanate (0.72 mL, 6.36 mmol, 3.0 equiv) was added. The mixture was heated at 40 °C under air (closed system: flask with septum) for 16.5 h. The mixture was concentrated *in vacuo*. Flash chromatography (Biotage, 25 g Sfär Duo column, MeOH/$CH_2Cl_2$ gradient from 0:100 to 8:92) gave **9-2** (920.0 mg, 64%, uncorrected for impurities in the starting material) as a white solid. **MP** 156-157 °C. **TLC** – $R_f$ = 0.27 (5:95 MeOH/$CH_2Cl_2$, ninhydrin stain). **$^1$H NMR** (400 MHz, $CDCl_3$) $\delta_H$ 8.55 (s, 2H, 2 × N*H*), 6.36 (t, *J* = 5.2 Hz, 1H, N*H*), 6.09 (s, 2H, 2 × N*H*), 3.39 (m, 8H, 4×C*H*2N), 3.28 (m, 8H, 4 × C*H*2N), 3.18 (m, 6H, 3 × $CH_{2\ butyl}$), 1.55 – 1.45 (m, 6H, 3 × $CH_{2\ butyl}$), 1.41 – 1.24 (m, 6H, 3 × $CH_{2\ butyl}$), 0.91 (m, 9H, 3 × $CH_{3\ butyl}$). **$^{13}$C NMR** (101 MHz, $CDCl_3$) $\delta_C$ 159.2 – 158.8 (3C, 3 × *C*=O), 158.1 (q, *J* = 37.4 Hz, 2C, 2 × *C*=O$CF_3$), 115.8 (q, *J* = 287.1 Hz, 2C, 2 × *C*$F_3$), 47.7 (2C, 2 × *C*$H_2$N), 47.4 (2C, 2 × *C*$H_2$N), 46.5 (2C, 2 × *C*$H_2$N), 41.0. – 40.8 (3 × $CH_{2\ butyl}$), 40.0 (2C, 2 × *C*$H_2$N), 32.1 – 32.0 (3 × $CH_{2\ butyl}$), 20.3 – 20.2 (3 × $CH_{2\ butyl}$), 13.9 – 13.8 (3 × $CH_{3\ butyl}$). **HR-MS** (ESI, positive ion mode) – *m/z* for $[C_{27}H_{48}F_6N_8O_5+Na]^+$ = 701.3544. Found 701.3519. **FTIR** (neat) = 3269, 3211, 3075, 2869, 2834, 1708, 1633, 1548, 1208, 1155.

**4,7,10-Tris(*n*-butylcarbonyl)-1,4,7,10,13-pentaazatridecane, 9-3**

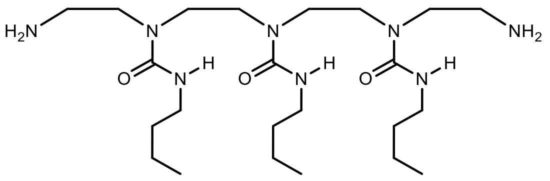


To a solution of **9-2** (920.0 mg, 1.36 mmol, 1.0 equiv) in lab grade THF (6.8 mL) and EtOH (13.6 mL) was added a solution of NaOH (433.7 mg, 10.84 mmol, 8.0 equiv) in water (6.8 mL) and the mixture was stirred at room temperature under air for 18 h. Most of the solvents were removed *in vacuo*, then water (30 mL) was added. The product was extracted with $CH_2Cl_2$ (2 × 30 mL + 15 mL) then the combined organic extracts were dried ($Na_2SO_4$) and concentrated to give **9-3** (584.1 mg, 89%) as a colourless gum. **TLC** – $R_f$ = 0.11 (10:2:88 MeOH/[35% aqueous $NH_3$]/$CH_2Cl_2$, ninhydrin stain). **$^1$H NMR** (400 MHz, $CDCl_3$) $\delta_H$ 6.66 (3H, 3 × N$H_{urea}$), 3.33 – 3.08 (m, 18H, 9 × C$H_2$), 2.83 (t, *J* = 5.6 Hz, 4H, 2 × C$H_2$$NH_2$), 1.56 – 2.03 (m, 4H, 4 × N*H*), 1.55 – 1.41 (m, 6H, 3 × C$H_{2\ butyl}$), 1.39 – 1.26 (m, 6H, 3 × C$H_{2\ butyl}$), 0.89 (m, 9H, 3 × C$H_{3\ butyl}$). **$^{13}$C NMR** (101 MHz, $CDCl_3$) $\delta_C$ 160.0 (2 × *C*=O), 158.8 (*C*=O), 51.9 (2C, 2 × *C*$H_2$N), 47.0 (4C, 4 × *C*$H_2$N), 41.5 (2C, 2 × *C*$H_2$N), 40.8 – 40.6 (3 × *C*$H_{2\ butyl}$), 32.3 – 32.2 (3 × *C*$H_{2\ butyl}$), 20.3 (3 × *C*$H_{2\ butyl}$), 14.0 – 13.9 (3 × *C*$H_{3\ butyl}$). **HR-MS** (ESI, positive ion mode) – *m/z* for $[C_{23}H_{50}N_8O_3+H]^+$ = 487.4079. Found 487.4054. *m/z* for $[C_{23}H_{50}N_8O_3+Na]^+$ = 509.3898. Found 509.3909. **FTIR** (neat) = 3287, 2966, 2930, 2871, 1622, 1537, 1271, 1218.

**1-(3,5-Bis(trifluoromethyl)anilinylcarbonyl)-4,7,10-Tris(*n*-butylcarbonyl)-1,4,7,10,13-pentaazatridecane, 9-4**

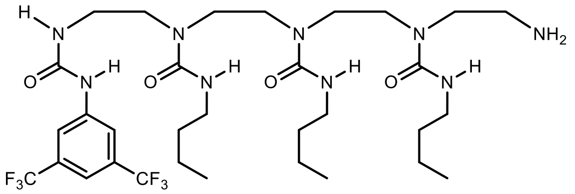


To a solution of **9-3** (340.7 mg, 0.70 mmol, 1.4 equiv) in lab grade $CH_2Cl_2$ (14 mL) at 0 °C under air was added dropwise over 5 min a solution of 3,5-bis(trifluoromethyl)phenyl isocyanate (127.6 mg, 0.50 mmol, 1.0 equiv) in $CH_2Cl_2$ (2.7 mL) and the mixture was allowed to warm to room temperature with stirring over 3.5 h. The mixture was concentrated *in vacuo*. Flash chromatography (Biotage, 10 g Sfär Duo column, MeOH/[35% aqueous $NH_3$]/$CH_2Cl_2$ gradient from 0:0:100 to 10:2:88) gave **9-4** (199.8 mg, 54%, based on the isocyanate) as a white solid. **TLC** – $R_f$ = 0.21 (10:2:88 MeOH/[35% aqueous $NH_3$]/$CH_2Cl_2$). **$^1$H NMR** (400 MHz, $CDCl_3$) $\delta_H$ 8.96 (s, 1H, N*H*), 7.84 (s, 2H, 2 × C$H_{Ar}$), 7.32 (s, 1H, C$H_{Ar}$), 6.81 (s, 3H, N*H*), 6.55 (s, 1H, N*H*), 3.37– 3.20 (m, 10 H, 5 × C$H_2$), 3.20 – 3.10 (m, 8H, 4 × C$H_2$), 3.06 (td, *J* = 7.0, 5.3 Hz, 2H, C$H_2$NH), 2.77 (t, *J* = 5.0 Hz, 2H, C$H_2$$NH_2$), 1.75 – 1.0 (m, 2H, $NH_2$), 1.54 – 1.31 (m, 6H, 3 × C$H_2$), 1.31 – 1.12 (m, 6H, 3 × C$H_2$), 0.93 – 0.64 (m, 9H, 3 × C$H_3$). **$^{13}$C NMR** (101 MHz, $CDCl_3$) $\delta_C$ 160.7 (C=O), 159.1 (C=O), 159.0 (C=O), 156.3 (C=O), 141.8 ($C_{Ar}$), 132.07 (q, *J* = 33.0 Hz, 2C, 2 × *C*$CF_3$), 123.5 (q, *J* = 272.7 Hz, 2C, 2 × *C*$F_3$), 119.45 (2C, 2 × *C*$H_{Ar}$), 114.9 (*C*$H_{Ar}$), 52.6 (*C*$H_2$), 48.6 (*C*$H_2$), 47.9 (*C*$H_2$), 47.5 (*C*$H_2$), 47.4 (*C*$H_2$), 47.0 (*C*$H_2$), 41.6 (*C*$H_2$), 41.2 (*C*$H_2$),

40.9 ($CH_2$), 40.5 ($CH_2$), 39.4 ($CH_2$), 32.3 ($CH_2$), 32.1 ($CH_2$), 31.9 ($CH_2$), 20.3 – 20.2 (2C, 2 × $CH_2$), 13.9 – 13.8 (2C, 2 × $CH_3$). **HR-MS** (ESI, positive ion mode) – *m/z* for $[C_{32}H_{53}F_6N_9O_4+H]^+$ = 742.4197. Found 742.4182. **FTIR** (neat) = 3296, 2959, 2932, 2873, 1625, 1556, 1387, 1276, 1132.

**1-(3,5-Bis(trifluoromethyl)anilinylcarbonyl)-4,7,10,13-tetrakis(butylaminocarbonyl)-13-((di(butylmercapto)maleimido)ethyl)-1,4,7,10,13-pentaazatridecane, 9**

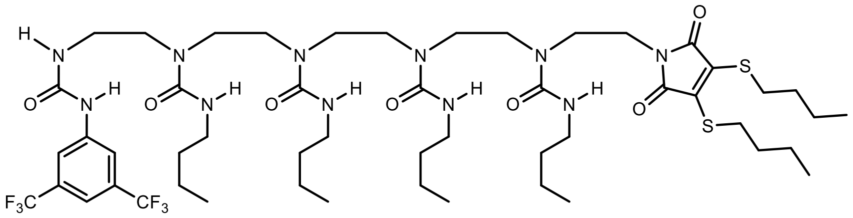

***Step 1** (reductive amination):* To a solution of amine **9-4** (74.2 mg, 0.10 mmol, 1.0 equiv) in lab grade 1,2-dichloroethane (0.2 mL) at room temperature was added a solution of aldehyde **7a-3** (31.5 mg, 0.10 mmol, 1.0 equiv) in 1,2-dichloroethane (0.8 mL). Solid $NaBH(OAc)_3$ (29.7 mg, 0.14 mmol, 1.4 equiv) was added and the mixture was stirred at room temperature under air for 2 h. Saturated $NaHCO_3$ (8 mL) and water (15 mL) were added. The product was extracted with $CH_2Cl_2$ (30 mL + 10 mL) then the combined organic extracts were dried ($Na_2SO_4$) and concentrated. Flash chromatography (Biotage, 10 g Sfär Duo column, MeOH/$CH_2Cl_2$ gradient from 0:100 to 7.5:92.5) gave **9-5** (55.8 mg, 54%) as a yellow semi-solid. **TLC** – $R_f$ = 0.14 (5:95 MeOH/$CH_2Cl_2$). ***Step 2** (urea formation):* The product from *Step 1* (55.8 mg, 0.054 mmol, 1.0 equiv) was dissolved in lab grade $CH_2Cl_2$ (0.3 mL) and a solution of butyl isocyanate (5.3 mg, 0.054 mmol, 1.0 equiv) in $CH_2Cl_2$ (0.8 mL) was added. The mixture was stirred at room temperature under air for 2 h. The solvent was removed *in vacuo* to give **9** (59.2 mg, 97%, or 52% over two steps) as a yellow solid. **M.P.** 165-166 °C ($CHCl_3$/PE). **TLC** – $R_f$ = 0.36 (5:95 MeOH/$CH_2Cl_2$). **$^1$H NMR** (500 MHz, $CD_2Cl_2$) $\delta_H$ 0.86 – 0.94 (m, 18H), 1.31 – 1.46 (m, 12H), 1.47 – 1.56 (m, 8H), 1.60 (p, *J* = 7.4 Hz, 4H), 3.15 – 3.43 (m, 30H), 3.63 (t, *J* = 6.8 Hz, 2H), 6.40 – 7.02 (overlapping broad singlets, 4H, 4 × N*H*), 7.43 (s, 1H), 8.00 (s, 2H), 9.05 (bs, 1H). *Note:* One NH resonance, presumably the NH at the N terminus proximal to the maleimide, was not observed. **$^{13}$C NMR** (126 MHz, $CD_2Cl_2$) $\delta_C$ 13.9, 14.06, 14.15, 14.2, 20.675, 20.685, 20.71, 22.2, 32.1, 32.5, 32.56, 32.63, 32.65, 33.0, 37.3, 40.0, 41.2, 41.3, 41.38, 41.44, 46.6, 47.7, 48.0, 48.2, 48.3, 48.9, 115.0, 118.3, 124.1 (q, *J* = 272.5 Hz), 132.2 (q, *J* = 32.9 Hz), 136.6, 142.5, 156.7, 158.8, 159.36, 159.40, 159.5, 167.4. **$^{19}$F NMR** (377 MHz, $CDCl_3$) $\delta_F$ −64.6 (s, 6F, 2 × C*F*$_3$). **HR-MS** (ESI, positive ion mode) – *m/z* for $[C_{51}H_{83}F_6N_{11}O_7S_2+Na]^+$ = 1162.5720. Found 1162.5765. **FTIR** (neat) – 3294, 3211, 2995, 1761, 1633.

**1-Trifluoroacetyl-4,7,10-tris(*n*-butylcarbonyl)-1,4,7,10,13-pentaazatridecane, 10-1**

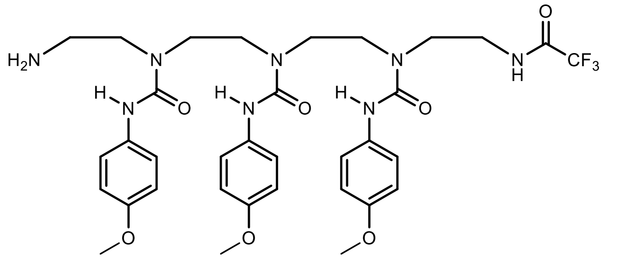

To a solution of **5b-2** (636.8 mg, 1.00 mmol, 1.0 equiv) in lab grade $CH_2Cl_2$ (1.0 mL) at 0 °C under $N_2$ was added over 75 min a solution of ethyl trifluoroacetate (142.1 mg, 1.00 mmol, 1.0 equiv) in $CH_2Cl_2$ (1.0 mL), while maintaining the temperature at 0–5 °C. After complete addition, the mixture was allowed to warm to room temperature and stirred for a further 2 h and 15 min. The reaction mixture (including solvent) was directly subjected to flash chromatography (Biotage, 10 g Sfär Duo column, MeOH/$CH_2Cl_2$ gradient from 1:99 to 10:90) to give **10-1** (412.7 mg, 56%) as a white solid. **TLC** – $R_f$ = 0.32 (10:2:88 MeOH/[35% aqueous $NH_3$]/$CH_2Cl_2$). **$^1$H NMR** (400 MHz, DMSO-$d_6$) $\delta_H$ 2.80 (t, $J$ = 5.8 Hz, 2H), 3.31 – 3.51 (m, 14H), 3.70 (overlapping singlets, 6H, 2 × OC$H_3$), 3.71 (s, 3H, 1 × OC$H_3$), 6.80 – 6.87 (m, 6H), 7.34 (d, $J$ = 9.0 Hz, 2H), 7.39 (d, $J$ = 8.8 Hz, 2H), 7.46 (d, $J$ = 9.0 Hz, 2H), 8.56 (bs, 1H), 8.94 (bs, 1H), 9.50 (bs, 1H). **$^{13}$C NMR** (101 MHz, DMSO-$D_6$) $\delta_C$ 38.4, 40.6, 45.9 – 46.8 (6 × broad semi-resolved signals from N$C$H$_2$), 55.16 (2 × unresolved O$C$H$_3$), 55.20 (1 × O$C$H$_3$), 113.56, 113.64, 113.7, 116.0 (q, $J$ = 288.1 Hz), 121.2, 121.3, 121.6, 133.4, 133.5, 154.5, 154.6, 155.6, 155.9, 156.7, 156.8; *Note:* the signal to noise ratio was too low to clearly observe the CO resonance (quartet). **$^{19}$F NMR** (376 MHz, DMSO-$D_6$) $\delta_F$ –74.4 (s, 3F). **HR-MS** (ESI, positive ion mode) – *m/z* for $[C_{34}H_{43}F_3N_8O_7+H]^+$ = 733.3280. Found 733.3277.

**1-(2-Pyridyl)-1,10,13,16-tetrakis(4-methoxyanilinylcarbonyl)-4-(*tert*-butoxycarbonyl)-19-trifluoroacetyl-1,4,7,10,13,16,19-heptaazanonadecane, 10-2**

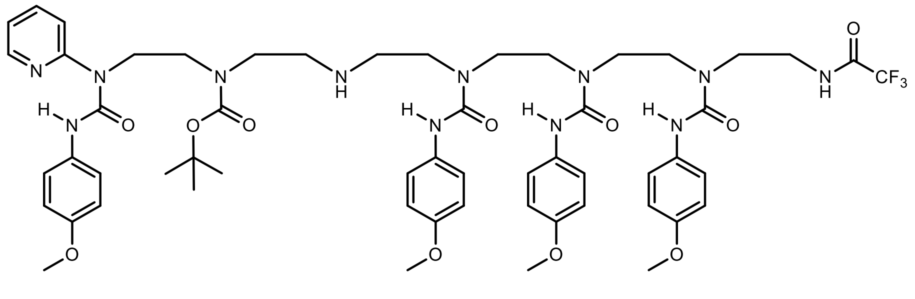

To a solution/suspension of amine **10-1** (638.6 mg, 0.87 mmol, 1.0 equiv) in lab grade 1,2-dichloroethane (5.0 mL) at room temperature under air was added a solution of aldehyde **4b-8** (373.4 mg, 0.87 mmol, 1.0 equiv) in 1,2-dichloroethane (3.7 mL). After stirring for 10 min, solid $NaBH(OAc)_3$ (258.6 mg, 1.22 mmol, 1.4 equiv) was added and the mixture was stirred under $N_2$ at room temperature for 5 h. Saturated $NaHCO_3$ (25 mL) and water (15 mL) were added. The product was extracted with $CH_2Cl_2$ (40 mL + 2 × 20 mL) then the combined organic extracts were washed with saturated $NaHCO_3$/brine (1:1, 30 mL), dried ($Na_2SO_4$) and concentrated. Flash chromatography (Biotage, 25 g Sfär Duo column, MeOH/$CH_2Cl_2$ gradient from 1:99 to 10:90) gave **10-2** (609.9 mg, 61%) as an off-

white amorphous solid. **TLC** – $R_f$ = 0.31 (7.5:92.5 MeOH/$CH_2Cl_2$). **$^1$H NMR** (400 MHz, $CDCl_3$) $\delta_H$ (~1:1 mixture of Boc rotamers; *indicates resolved rotamers) 1.47 (s, 9H), 2.83 – 2.98 (m, 4H), 3.34 – 3.56 (m, 18H), 3.72 – 3.79 (overlapping singlets, 12H, 4 × OC*H*$_3$), 4.02 – 4.13 (m, 2H), 6.77 – 6.88 (m, 8H), 6.95 – 7.03 (m, 1H), 7.15 – 7.82 (m, 10H), 8.24 – 8.33 (m, 1H), 8.67 – 8.79 (broad m, 1H), 9.12 (bs, 2H), [9.77* (bs) & 9.87* (bs), 1H in total], [12.56* (bs) & 12.62* (bs), 1H in total]. **$^{19}$F NMR** (376 MHz, $CDCl_3$) $\delta_F$ –75.8 (s, 3F). **HR-MS** (ESI, positive ion mode) – *m/z* for $[C_{56}H_{71}F_3N_{12}O_{11}+Na]^+$ = 1167.5210. Found 1167.5184.

**1-(2-Pyridyl)-1,4,7,10,13,16-hexakis(4-methoxyanilinylcarbonyl)-1,4,7,10,13,16,19-heptaazanonadecane**, **10-3**

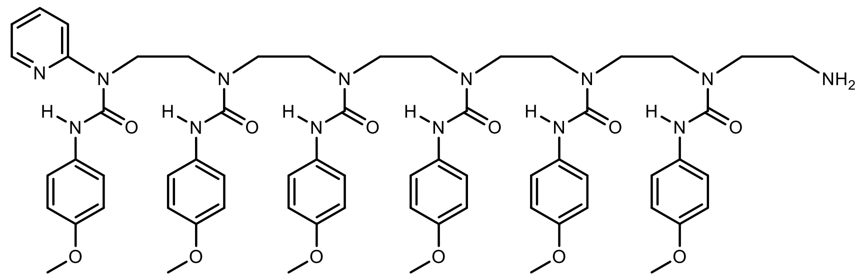


***Step 1** (Boc deprotection):* To a solution of **10-2** (609.9 mg, 0.53 mmol, 1.0 equiv) in lab grade $CH_2Cl_2$ (5.3 mL) at room temperature under air was added TFA (1.22 mL, 15.98 mmol, 30.0 equiv) and the mixture was stirred at room temperature for 24 h. After cooling to 0 °C, saturated $NaHCO_3$ (25 mL) was slowly added to the open flask over 10 min with stirring. The product was extracted with $CH_2Cl_2$ (2 × 20 mL) and the combined organic extracts were dried ($Na_2SO_4$) and concentrated. Flash chromatography (Biotage, 10 g Sfär Duo column, MeOH/[35% aqueous $NH_3$]/EtOAc gradient from 1:0.2:98.8 to 10:2:88) gave the product (484.8 mg, 87% if pure) as an off-white semi-solid contaminated with ~20 wt % acetamide, presumably arising from the chromatography eluent mixture. **TLC** – $R_f$ = 0.19 (10:2:88 MeOH/[35% aqueous $NH_3$]/EtOAc). ***Step 2** (bis-urea formation):* The impure product from *Step 1* (484.8 mg, 0.46 mmol if pure, 1.0 equiv) was taken up in lab grade $CH_2Cl_2$ (3.0 mL) and a solution of 4-methoxyphenyl isocyanate (138.4 mg, 0.93 mmol, 2.0 equiv) in $CH_2Cl_2$ (1.6 mL) was added. The mixture was stirred at room temperature under air for 2 h. MeOH (5 mL) was added and the mixture was stirred for 2 h to quench any unreacted isocyanate (potentially producing the corresponding 'non-polar' methyl carbamate as a by-product). The mixture was concentrated *in vacuo*. Flash chromatography (Biotage, 10 g Sfär Duo column, MeOH/$CH_2Cl_2$ gradient from 1:99 to 10:90) gave the product (514.7 mg, 72% over two steps) as an off-white solid. **TLC** – $R_f$ = 0.42 (7.5:92.5 MeOH/$CH_2Cl_2$). ***Step 3** (trifluoroacetamide hydrolysis):* The product from *Step 2* (514.7 mg, 0.38 mmol, 1.0 equiv) was dissolved in a mixture of lab grade THF (1.9 mL) and EtOH (3.9 mL). A solution of NaOH (104.2 mg, 3.06 mmol, 8.0 equiv) in water (1.9 mL) was added and the mixture was stirred at room temperature under air for 16 h. Most of the solvents were removed *in vacuo*, then water (30 mL) was added. The product was extracted with $CH_2Cl_2$ (60 mL + 20 mL) then the combined organic extracts were dried

($Na_2SO_4$) and concentrated. Flash chromatography (Biotage, 10 g Sfär Duo column, MeOH/$CH_2Cl_2$ gradient from 0:100 to 10:90 then MeOH/[35% aqueous $NH_3$]/$CH_2Cl_2$ 10:2:88) gave **10-3** (424.5 mg, 64% over three steps) as a white solid. **TLC** – $R_f$ = 0.32 (10:90 MeOH/$CH_2Cl_2$). **$^1$H NMR** (400 MHz, $CDCl_3$) $\delta_H$ 2.85 – 2.92 (m, 2H), 3.28 – 3.72 (m, 20H), 3.75 – 3.81 (overlapping singlets, 18H, 6 × OC*H*$_3$), 4.01 – 4.14 (m, 2H), 6.79 – 6.91 (m, 12H), 7.00 (dd, *J* = 7.5, 4.9 Hz, 1H), 7.31 (d, *J* = 8.7 Hz, 2H), 7.48 (d, *J* = 8.6 Hz, 2H), 7.60 – 7.68 (m, 8H), 7.76 (t, *J* = 7.9 Hz, 1H), 8.30 (d, *J* = 3.9 Hz, 1H), 9.10 – 9.26 (overlapping broad singlets, 4H, 4 × N*H*), 10.23 (bs, 1H), 12.65 (bs, 1H); *Note:* One signal of the pyridine ring (ArH3) is too broad to be identified. **$^{13}$C NMR** (101 MHz, $CDCl_3$) $\delta_C$ 41.9, 47.0, 47.3 – 49.0 (9 × broad semi-resolved signals from N*C*H$_2$), 53.0, 55.6 (6 × unresolved O*C*H$_3$), 112.6, 113.9, 114.0, 114.1, 114.2, 118.1, 120.7, 121.0, 121.2, 121.5, 122.4, 131.8, 133.2, 133.4, 133.5, 133.6, 140.0, 145.6, 154.7, 155.2, 155.3, 156.1, 156.56, 156.62, 156.7, 158.7. **HR-MS** (ESI, positive ion mode) – *m/z* for $[C_{65}H_{78}N_{14}O_{12}+H]^+$ = 1247.5996. Found 1247.5962.

**1-(2-Pyridyl)-19-((di(*n*-butylmercapto)maleimido)ethyl)-1,4,7,10,13,16,19-heptakis(4-methoxyanilinylcarbonyl)-1,4,7,10,13,16,19-heptaazanonadecane, 10**

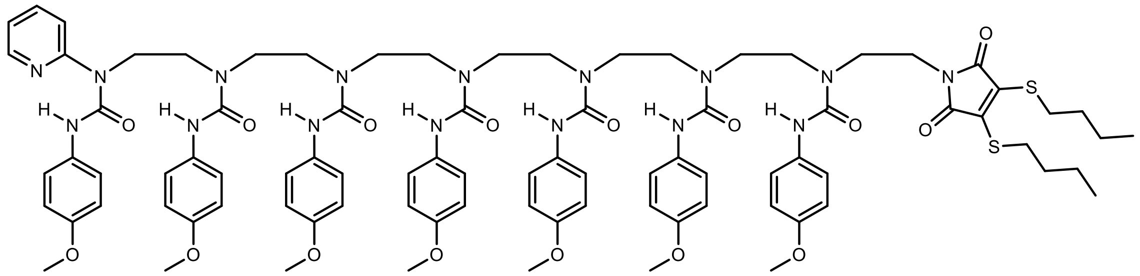

***Step 1*** *(reductive amination):* To a solution of amine **10-3** (187.1 mg, 0.15 mmol, 1.0 equiv) in lab grade 1,2-dichloroethane (0.5 mL) at room temperature was added a solution of aldehyde **7a-3** (47.3 mg, 0.15 mmol, 1.0 equiv) in 1,2-dichloroethane (1.0 mL). Solid $NaBH(OAc)_3$ (44.5 mg, 0.21 mmol, 1.4 equiv) was added and the mixture was stirred at room temperature under air for 2 h. Saturated $NaHCO_3$ (5 mL) and water (15 mL) were added. The product was extracted with $CH_2Cl_2$ (25 mL + 15 mL) then the combined organic extracts were dried ($Na_2SO_4$) and concentrated. Flash chromatography (Biotage, 10 g SNAP Ultra column, MeOH/$CH_2Cl_2$ gradient from 0:100 to 7.5:92.5) gave **10-4** (163.9 mg, 71%) as a yellow solid. **TLC** – $R_f$ = 0.32 (7.5:92.5 MeOH/$CH_2Cl_2$). ***Step 2*** *(urea formation):* The product from *Step 1* (163.9 mg, 0.11 mmol, 1.0 equiv) was dissolved in lab grade $CH_2Cl_2$ (0.5 mL) and a solution of 4-methoxyphenyl isocyanate (15.8 mg, 0.11 mmol, 1.0 equiv) in $CH_2Cl_2$ (0.6 mL) was added. The mixture was stirred at room temperature under air for 1 h. MeOH (5 mL) was added and the mixture was stirred for 15 min to quench any unreacted isocyanate. The mixture was concentrated *in vacuo*. Flash chromatography (Biotage, 5 g Sfär Duo column, MeOH/$CH_2Cl_2$ gradient from 0:100 to 7:93) gave **10** (172.5 mg, 96%) as a yellow solid. *Note:* For this longer oligomer, slow/medium exchange between two principal conformers was observed at room temperature by $^1$H NMR, as indicated by general peak broadening and partial splitting of the NH resonance at ~12.5 ppm (proximal

to the pyridine). Coalescence was observed by $^{1}$H NMR upon heating to 55 °C (see the VT NMR section). **TLC** – $R_f$ = 0.38 (5:95 MeOH/$CH_2Cl_2$). **$^{1}$H NMR** (400 MHz, $CDCl_3$, 25 °C) $\delta_H$ 0.89 (t, *J* = 7.3 Hz, 6H), 1.39 (h, *J* = 7.4 Hz, 4H), 1.58 (p, *J* = 7.5 Hz, 4H), 3.22 (t, *J* = 7.4 Hz, 4H), 3.32 – 3.69 (m, 26H), 3.74 – 3.82 (overlapping singlets, 21H, 7 × OC$H_3$), 3.98 – 4.14 (m, 2H), 6.77 – 6.92 (m, 14H), 7.00 (t, *J* = 6.2 Hz, 1H), 7.45 – 7.51 (m, 4H), 7.58 – 7.75 (m, 11H), 8.26 – 8.32 (m, 1H), 8.94 – 9.29 (overlapping broad singlets, 5H, 5 × N*H*), 12.10 – 12.90 (two overlapping broad singlets from partially decoalesced rotamers, 1H in total, N*H* proximal to pyridine). *Note:* Two resonances could not be identified, presumably due to extensive peak broadening/chemical exchange as observed previously for analogous protons in smaller homologues: 1) ArH3 of the pyridine ring and 2) the NH at the C-terminus, proximal to the maleimide. **$^{13}$C NMR** (101 MHz, $CDCl_3$) $\delta_C$ 13.6, 21.7, 31.7, 32.4, 36.7, 46.1 – 49.3 (13 × broad semi-resolved signals from N*C*$H_2$), 55.5 (7 × unresolved O*C*$H_3$), 112.5, 113.9, 113.98, 114.04, 114.1, 118.1, 121.1, 121.4, 122.1, 131.7, 131.9, 133.2, 133.36, 133.44, 135.9, 139.9, 145.7, 154.5, 155.1, 155.17, 155.24, 155.8, 156.0, 156.5, 156.6, 166.9. **HR-MS** (Nanospray, positive ion mode) – *m/z* for $[C_{87}H_{106}N_{16}O_{16}S_2+H]^+$ = 1695.7492. Found 1695.7474.

Supplementary Data

VT NMR Experiments

All low-temperature VT NMR experiments were conducted using a JEOL ECS 300 spectrometer (300 MHz) or a Bruker AVANCE III HD 500 MHz NMR Spectrometer with 5 mm DCH $^{13}C$–$^{1}H$/D Cryo Probe (500 MHz).

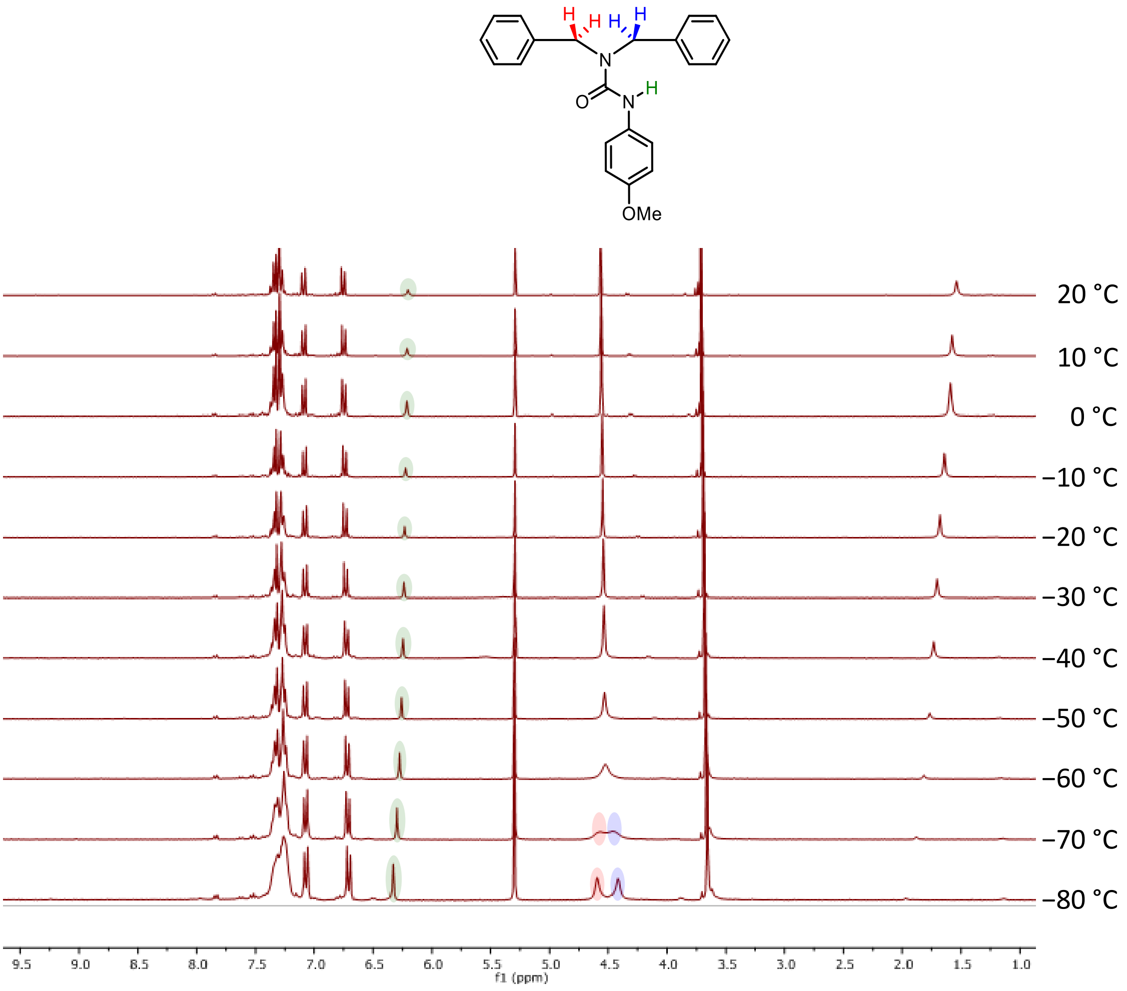


**Figure S1 – VT NMR data for 1 (300 MHz, $CD_2Cl_2$, 29 mM).** Signals at 20 °C: 4.56 (benzylic methylenes) and 6.20 ppm (ureido proton) at 20 °C. Upon cooling, the signal at 4.56 ppm is unaffected by decreasing temperature until −50 °C, where it broadens and eventually decoalesces into two singlets in a 50:50 ratio at −70 °C, which sharpen further at −80 °C. The signal at 6.20 ppm sharpens with decreasing temperature and moves downfield. These data are consistent with one populated conformation where rotation of the $Bn_2N$–CO bond is fast on the NMR timescale and becomes slow between −60 °C and −70 °C. The signals corresponding to the benzylic methylenes decoalesce because at slow exchange, one of the benzylic methylenes is proximate to a ureido proton, and the other is proximate to a ureido carbonyl, making them chemically inequivalent. Additionally, the downfield shift of the ureido proton with decreasing temperature is attributed to intermolecular hydrogen bonding. The assignment of the decoalesced benzylic methylene signals is arbitrary – it is unknown which methylene is proximate to the ureido carbonyl/proton.

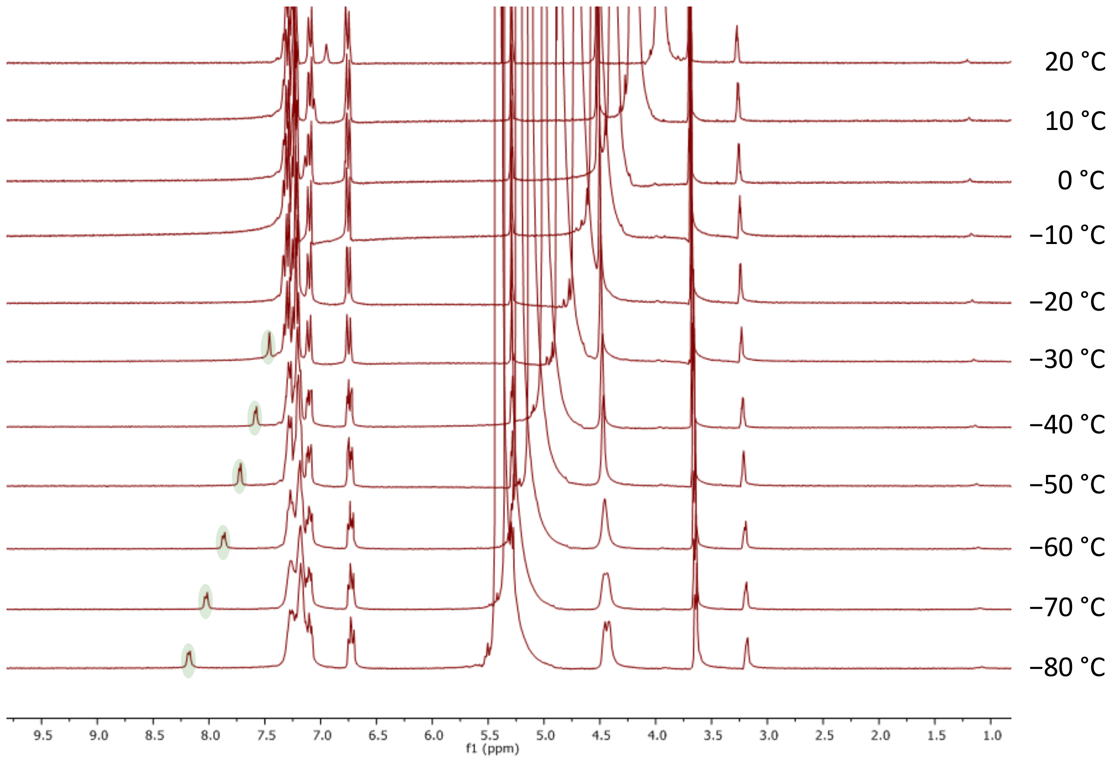


**Figure S2 – VT NMR data for 1 (300 MHz, 40% $CD_3OH$ in $CD_2Cl_2$, 29 mM).** Signals at 20 °C: 4.52 (benzylic methylenes) and 6.95 ppm (ureido proton). Upon cooling, the signal at 4.52 ppm broadens until eventually decoalescing at −70 °C. The signal at 6.95 ppm shifts downfield with decreasing temperature. In comparison to that observed in neat $CD_2Cl_2$ (Figure S1), a decoalescence event is observed at the same temperature but the anisochronicity was much less pronounced in the presence of $CD_3OH$. The ureido proton signal is also much more downfield in the presence of $CD_3OH$ due to intermolecular hydrogen bonding with the solvent.

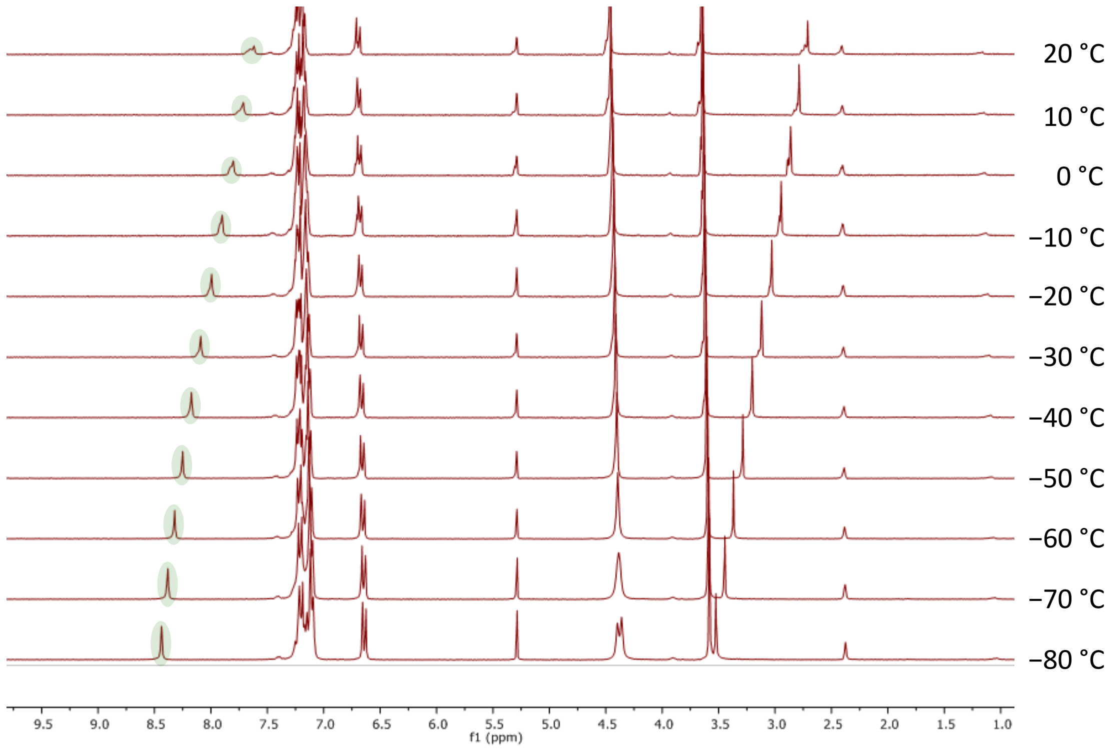


**Figure S3 – VT NMR data for 1 (300 MHz, 10% $(CD_3)_2SO$ in $CD_2Cl_2$, 29 mM).** Signals at 20 °C: 4.46 (benzylic methylenes) and 7.63 ppm (ureido proton). The signal at 4.46 ppm broadens with decreasing temperature and eventually decoalesces at −80 °C. The signal at 7.63 ppm shifts downfield and sharpens upon cooling. This shows that **1** exists as one conformation, and rotation about the $Bn_2N$–CO bond is slow on the NMR timescale below −80 °C. Additionally, $d_6$-DMSO hydrogen bonds to the ureido proton, as is demonstrated by its downfield shift with decreasing temperature.

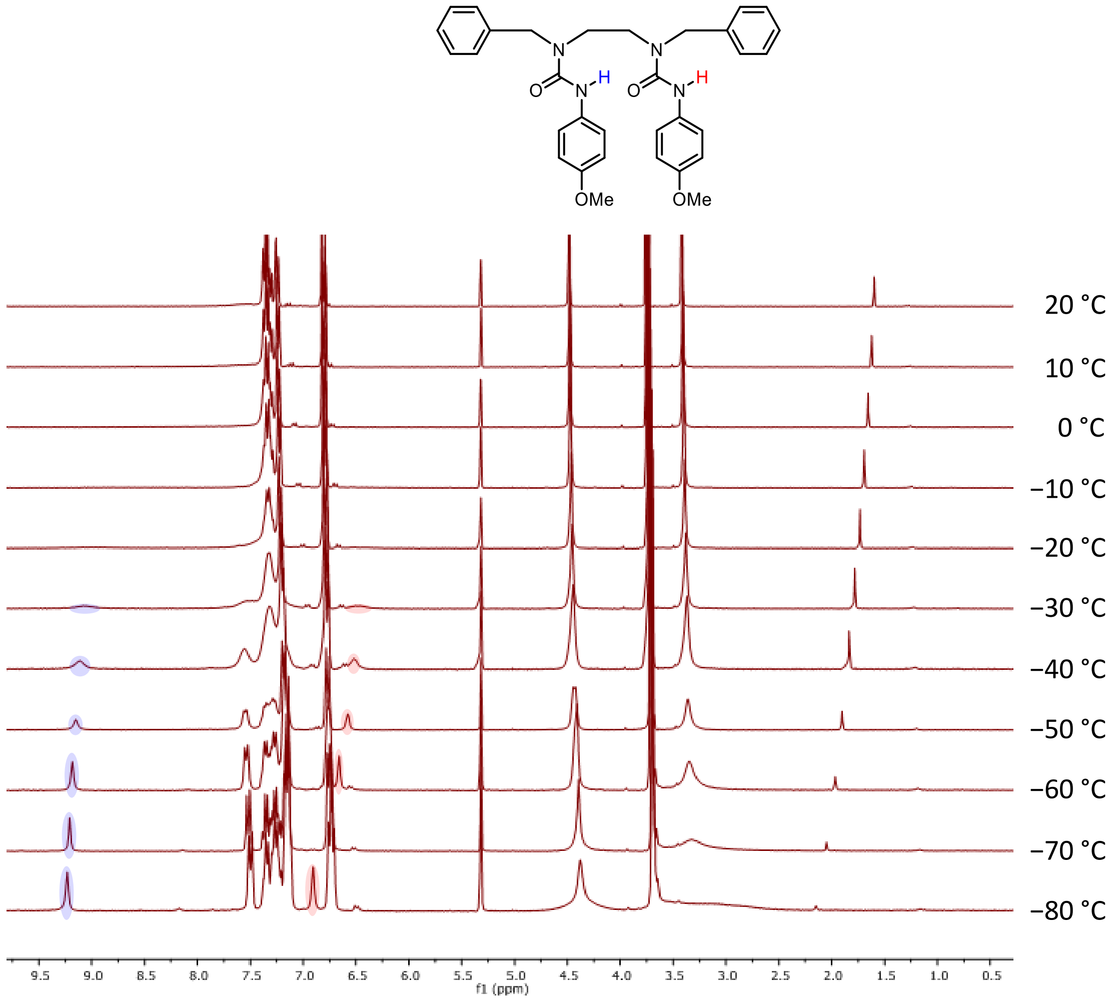


**Figure S4 – VT NMR data for 2a (300 MHz, $CD_2Cl_2$, 19 mM).** Signals at 20 °C: 4.48 ppm (benzylic methylenes). Upon cooling, the signal at 4.48 ppm broadens and decoalesces at −50 °C. The decoalesced signals broaden further with decreasing temperature. Signals appear at 6.47 (1 H) and 9.06 ppm (1 H) at −30 °C, which sharpen and shift downfield with decreasing temperature. These data are consistent with one populated conformer of uniform directionality where the two benzylic methylenes are chemically inequivalent at slow exchange. At slow exchange, one ureido proton is in hydrogen bonding (9.06 ppm at −40 °C) and the other is not (6.47 ppm at −40 °C). Triurea **3a** and tetraurea **4a** were also analysed by VT NMR (Figure S10, S16), and they were found to behave analogously to diurea **2a**, but with increasing numbers of hydrogen-bonding ureido protons.

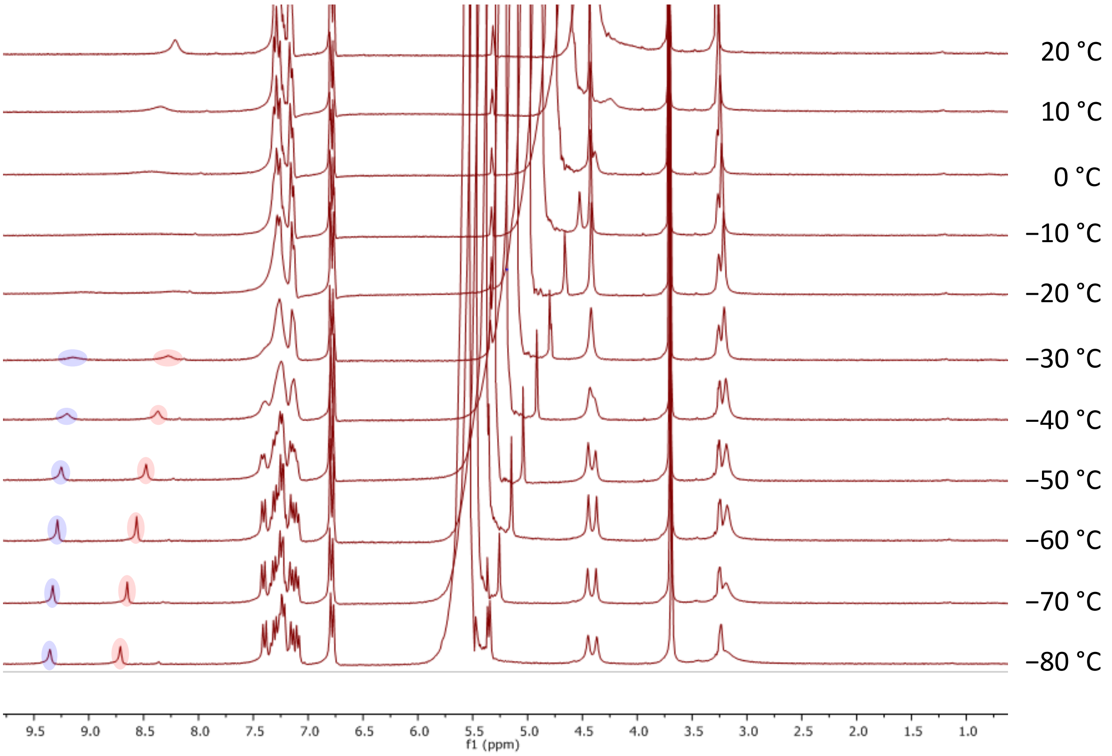


**Figure S5 – VT NMR data for 2a (300 MHz, 40% $CD_3OH$ in $CD_2Cl_2$, 19 mM).** Signals at 10 °C (the benzylic methylene signal was obscured by the alcohol peak at 20 °C): 4.43 (benzylic methylenes) and 8.21 ppm (ureido protons). Upon cooling, the signal at 4.43 ppm broadens with decreasing temperature and decoalesces at −40 °C into two singlets in a 50:50 ratio, which sharpen upon further cooling. The signal at 8.21 ppm shifts downfield and broadens with decreasing temperature, until it decoalesces into two broad singlets (1 H each) at −30 °C. These singlets sharpen and shift further downfield with further cooling. If the $CD_3OH$ interrupted the intramolecular hydrogen bonding, the resultant intermolecular hydrogen bond with $CD_3OH$ would give rise to conformations where both ureas are chemically identical at slow exchange. However, decoalescence of the benzylic methylenes was still observed, indicating directional uniformity. The $CD_3OH$ did not interrupt the intramolecular hydrogen bonding, it simply complemented it by hydrogen bonding to both ends; one through the hydroxyl oxygen and the other through the hydroxyl proton. This was further validated by the chemical shifts of the ureido protons at low temperature. At −60 °C, there is a signal at 9.29 ppm corresponding to an intramolecularly hydrogen-bonded ureido proton, which is very similar to **2a** in $CD_2Cl_2$ (9.18 ppm). The other ureido proton shows a signal at 8.56 ppm, which is very different to that observed in $CD_2Cl_2$ (6.66 ppm) due to the formation of a hydrogen bond with $CD_3OH$ (Figure S4). Triurea **3a** and tetraurea **4a** were also analysed by VT NMR (Figure S13, S19), and they were found to behave analogously to diurea **2a**, but with increasing numbers of hydrogen-bonding ureido protons.

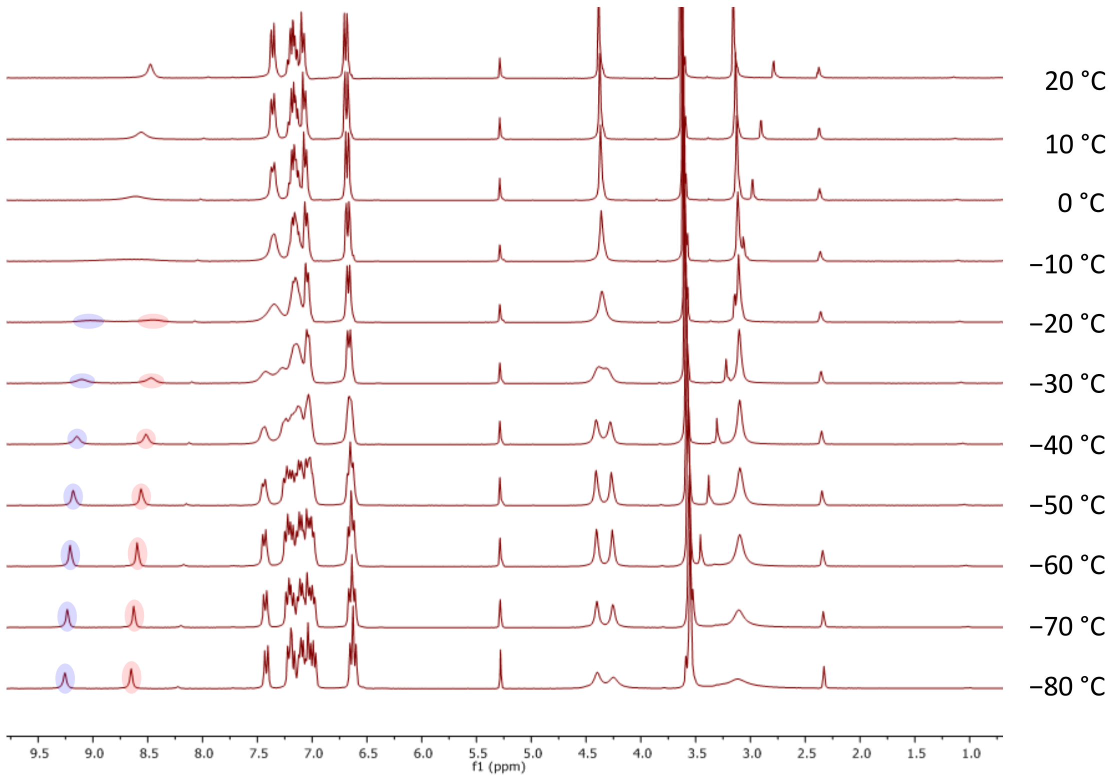


**Figure S6 – VT NMR data for 2a (300 MHz, 10% $(CD_3)_2SO$ in $CD_2Cl_2$, 19 mM).** Signals at 20 °C: 4.38 (benzylic methylenes) and 8.47 ppm (ureido protons). The signal at 4.38 ppm broadens and decoalesces at −30 °C into two singlets in a 50:50 ratio, which sharpen further upon cooling. The signal at 8.47 ppm shifts downfield and broadens with decreasing temperature until it decoalesces into two singlets (1 H each) at −20 °C, which then sharpen and shift further downfield with decreasing temperature. These data show that that $d_6$-DMSO does not interrupt the intramolecular hydrogen bonding in **2a**, it instead complements it by hydrogen bonding to the exposed terminal ureido proton. This is further validated by the chemical shifts of the ureido protons at low temperature. At −60 °C, there is a signal at 9.21 ppm corresponding to a ureido proton intramolecularly hydrogen bonded to the other urea. The other ureido proton shows a signal at 8.59 ppm, which is much more downfield than that observed in $CD_2Cl_2$ (6.66 ppm at −60 °C) as it is hydrogen bonding with $d_6$-DMSO. Triurea **3a** and tetraurea **4a** were also analysed by VT NMR (Figure S14, S20), and they were found to behave analogously to diurea **2a**, but with increasing numbers of hydrogen-bonding ureido protons.

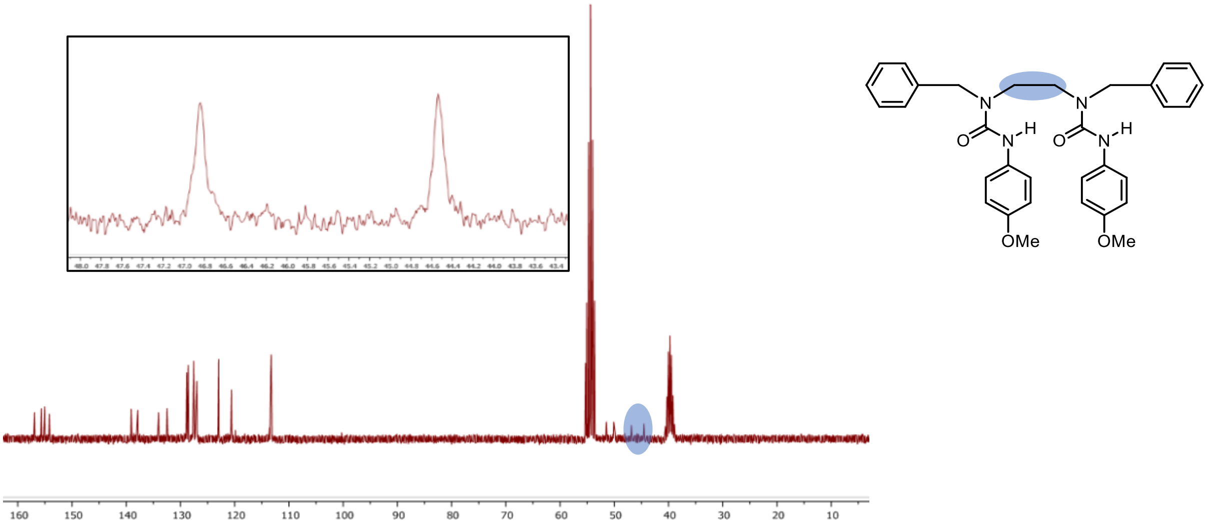


**Figure S7 – $^{13}$C spectrum of 2a (76 MHz, $CD_2Cl_2$, 19 mM) at −60 °C.** There are four methylene environments at slow exchange, ruling out a $C_2$-symmetrical conformation in favour of the $C_1$ conformation depicted above.

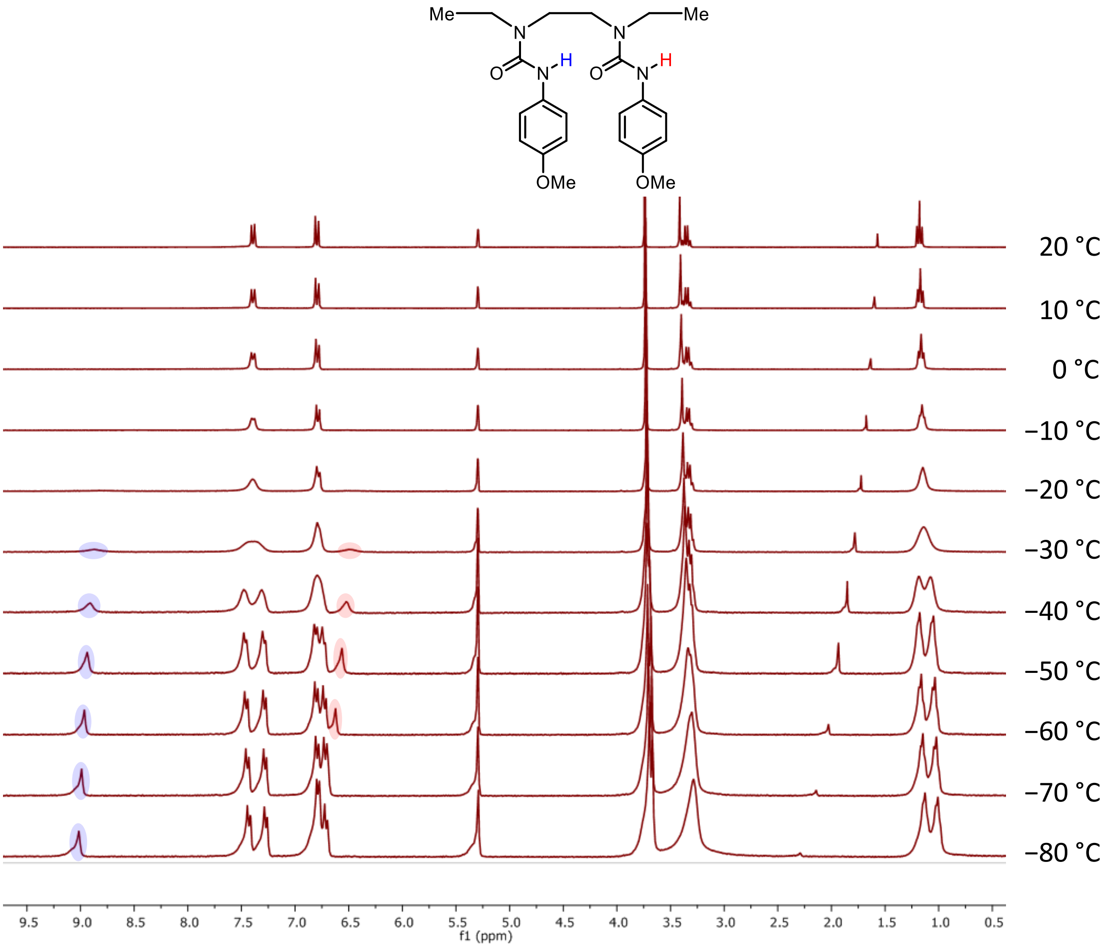


**Figure S8 – VT NMR data for 2b (300 MHz, $CD_2Cl_2$, 24 mM).**

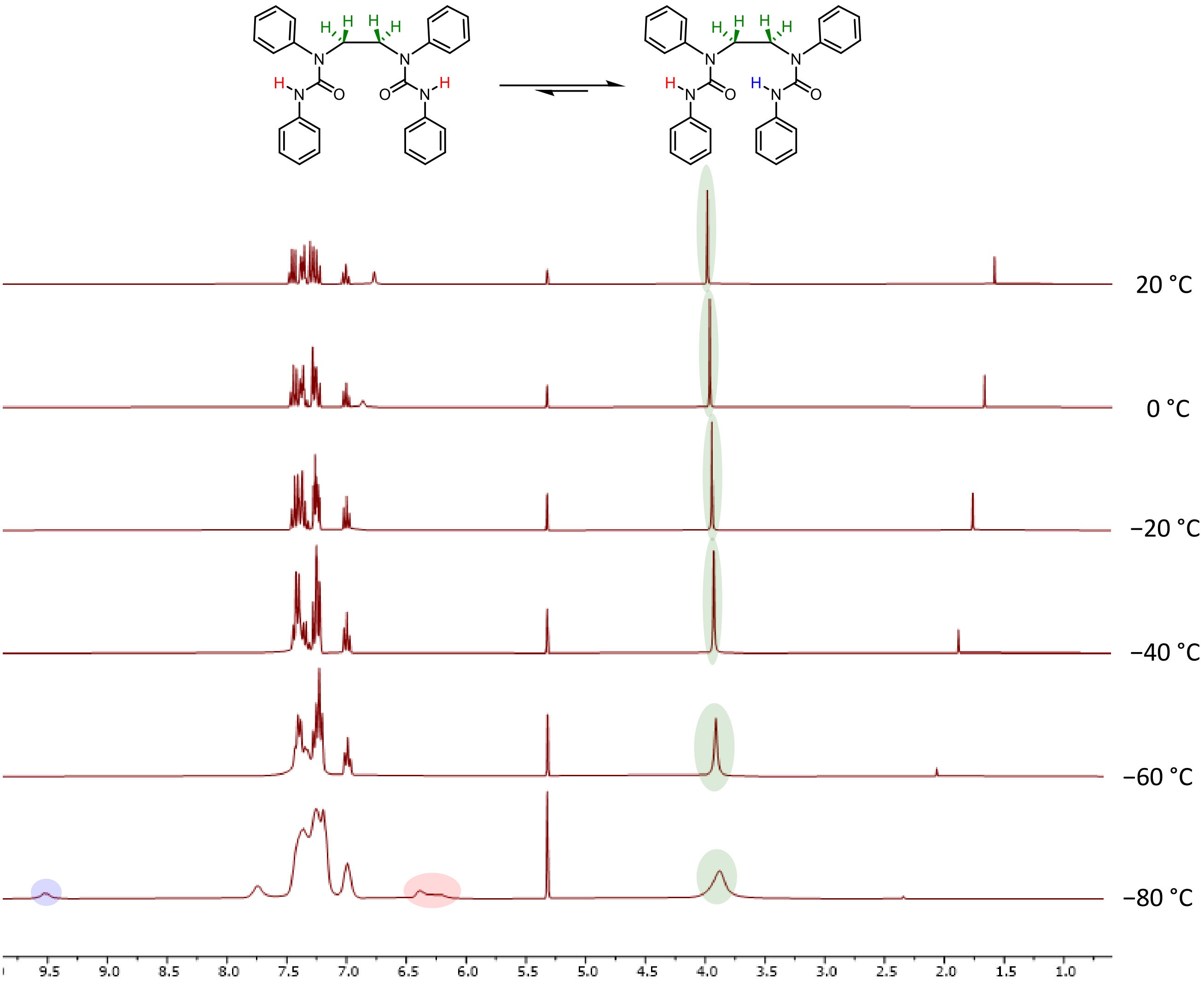


**Figure S9 – VT NMR data for 2c (300 MHz, $CD_2Cl_2$, 36 mM).** Signals at 20 °C: 3.93 (methylenes) and 6.49 ppm (ureido protons). The signal at 3.93 ppm remains sharp until it broadens at −60 °C. The signal at 6.49 ppm shifts downfield and broadens with decreasing temperature. Broadening continues into the baseline, where decoalescence into three singlets at 6.23 (0.6 H), 6.40 (0.8 H) and 9.52 ppm (0.6 H) at −80 °C occurs. These data conclude that at −80 °C, two conformers are present in a 40:60 ratio. One unsymmetric conformation, where one ureido proton is intramolecularly hydrogen bonded and the other is not (6.23 and 9.52 ppm) is present as the major conformer. Another symmetric conformer, where the ureido protons are identical and not in hydrogen bonding (6.40 ppm) is the minor conformer.

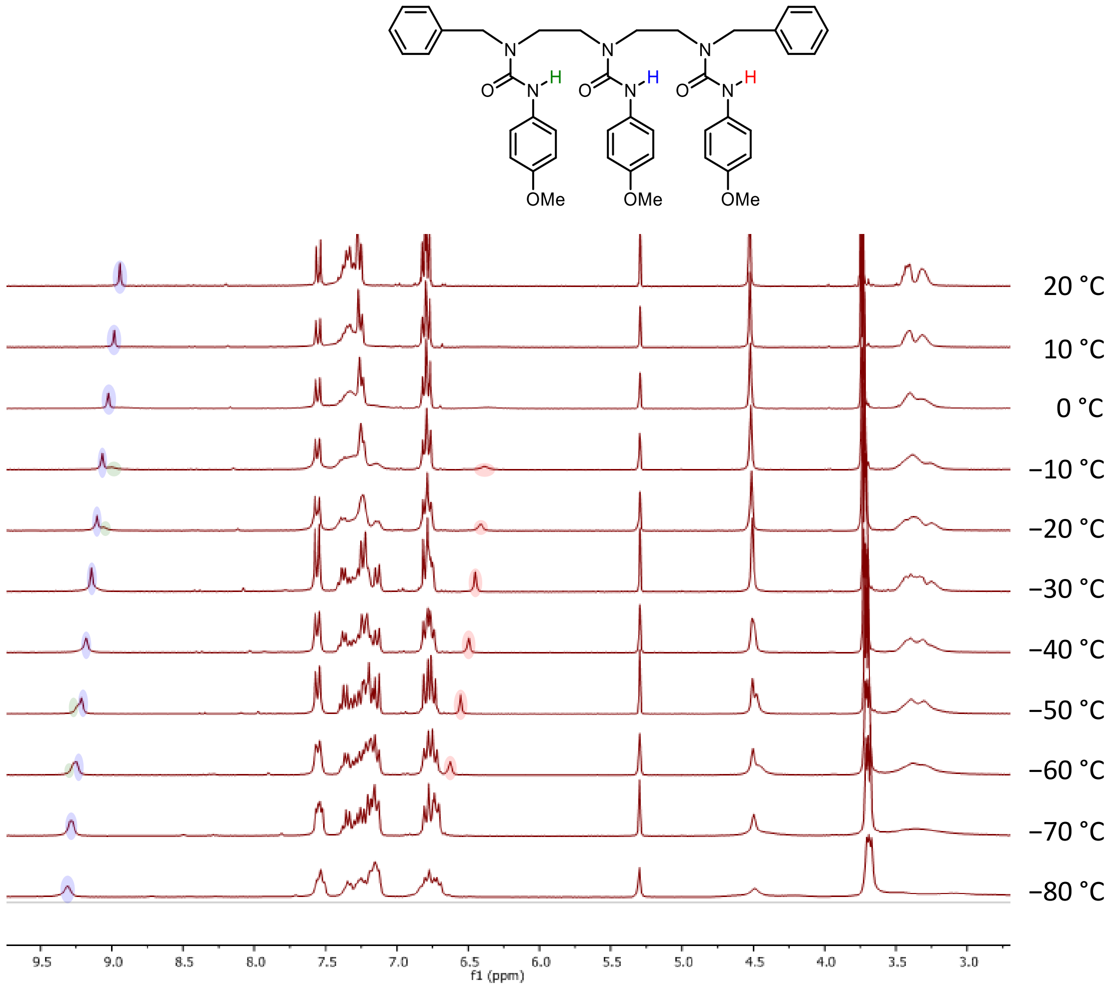


**Figure S10 – VT NMR data for 3a (300 MHz, $CD_2Cl_2$, 14 mM).**

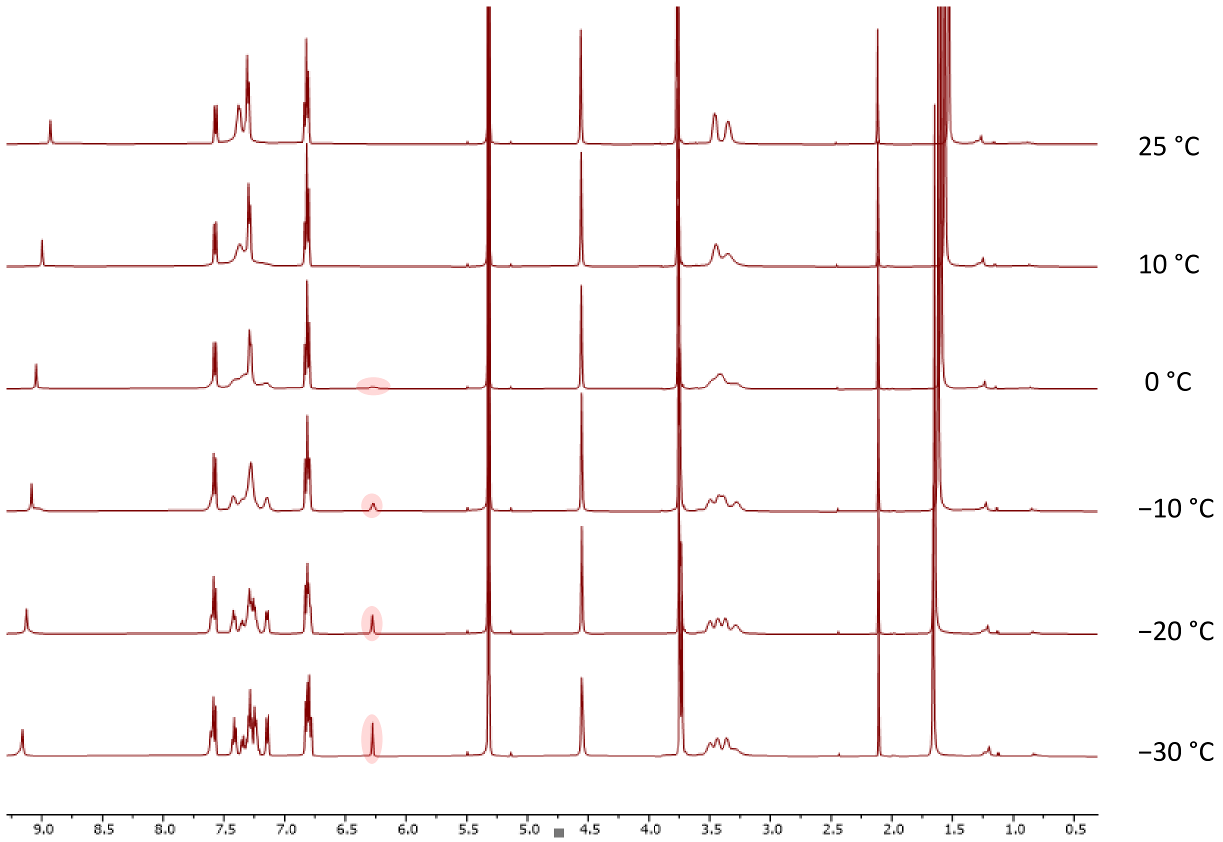


**Figure S11 – VT NMR data for 3a (500 MHz, $CD_2Cl_2$, 2.5 mM).** At slow exchange (< 0 °C), a signal appears at 6.28 ppm corresponding to the non-hydrogen bonding ureido proton, which remains at the same chemical shift through to −30 °C.

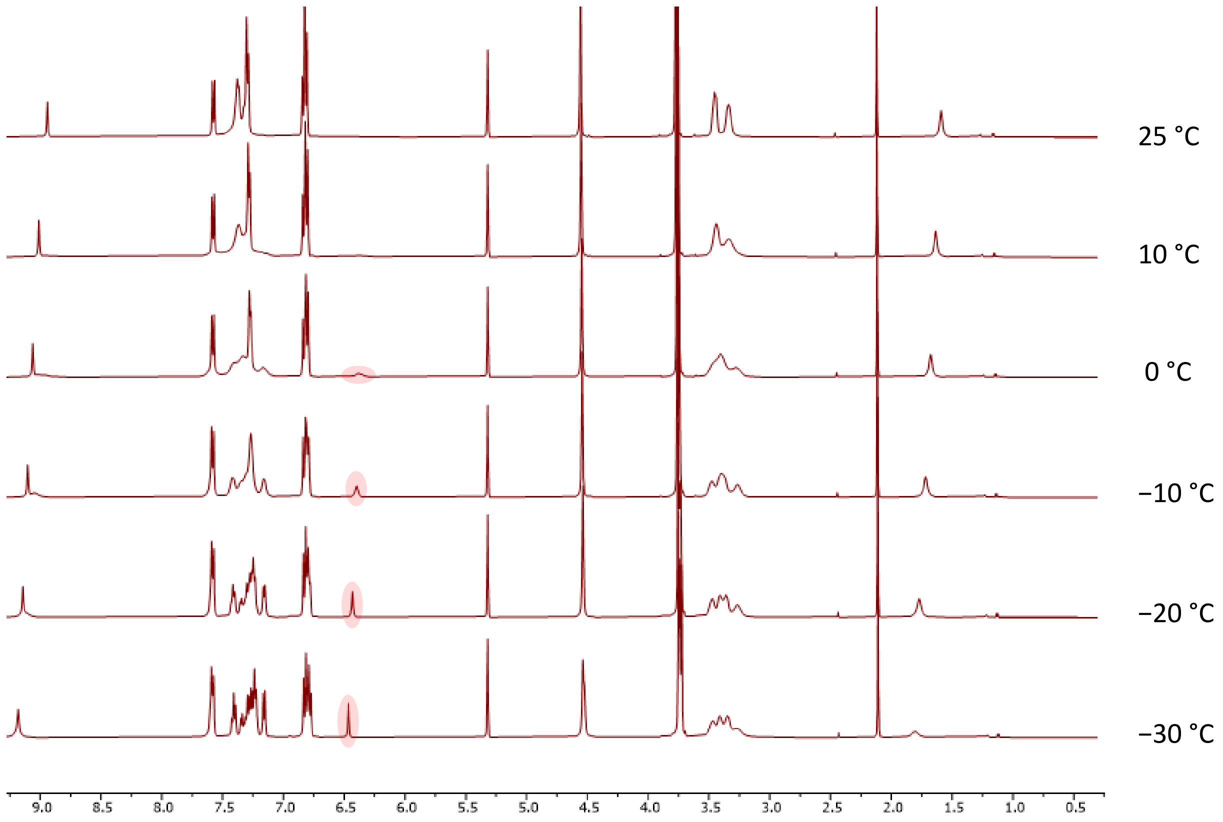


**Figure S12 – VT NMR data for 3a (500 MHz, $CD_2Cl_2$, 25 mM).** At slow exchange (< 0 °C), a signal appears at at 6.37 ppm corresponding to the non-hydrogen bonding ureido proton. The signal moves downfield with decreasing temperature to 6.48 ppm at −30 °C. The concentration and temperature dependence of the non-intramolecularly hydrogen-bonding ureido proton is attributed to intermolecular hydrogen bonding with the carbonyl of the urea at the other terminus. It is assumed that this intermolecular hydrogen bonding translates to other compounds at this concentration when terminal hydrogen-bond acceptors are available.

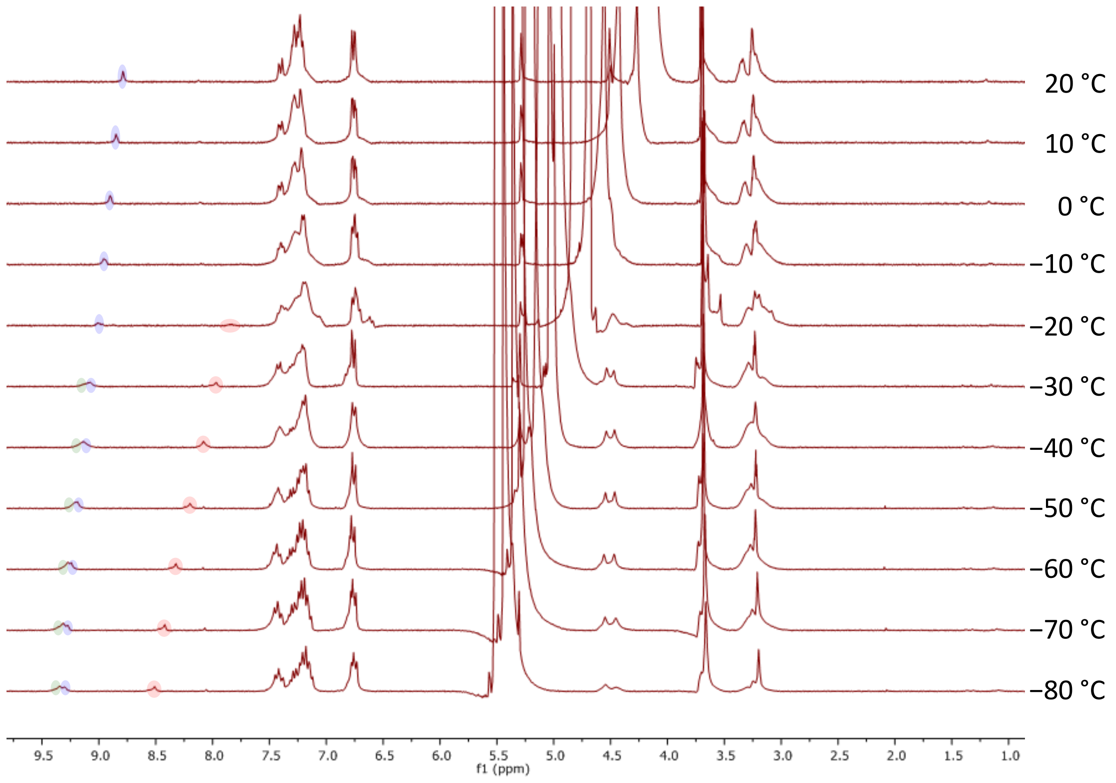


**Figure S13 – VT NMR data for 3a (300 MHz, 40% $CD_3OH$ in $CD_2Cl_2$, 14 mM).**

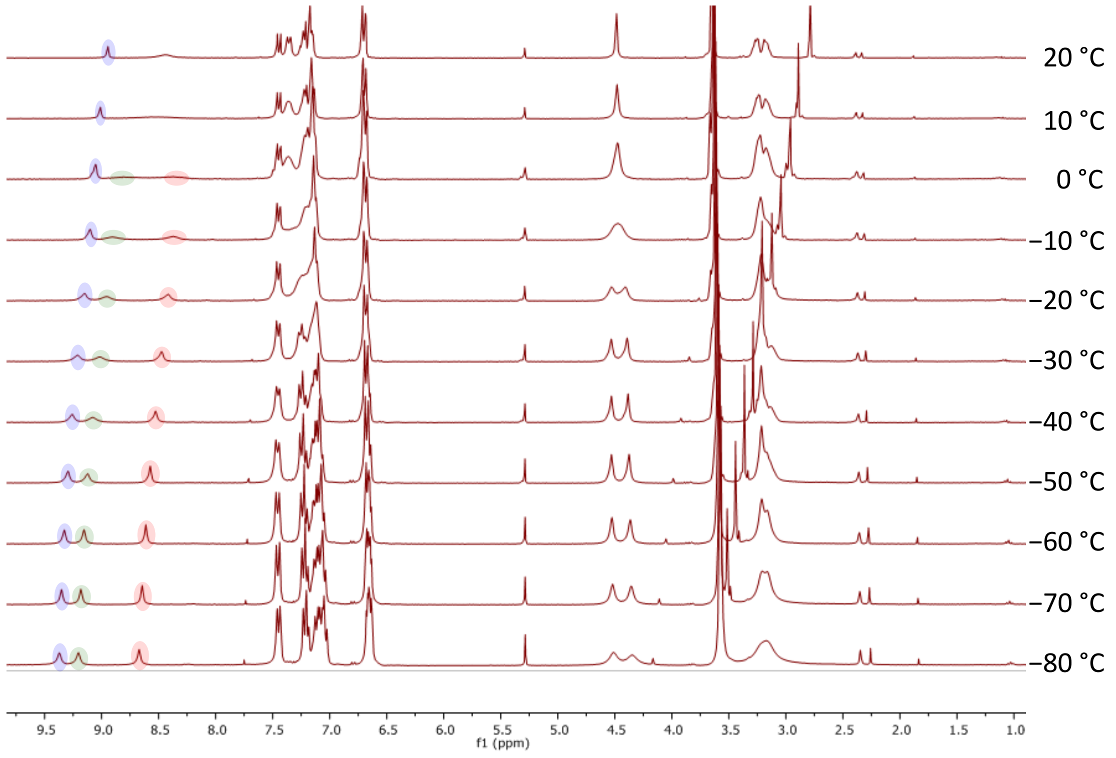


**Figure S14 – VT NMR data for 3a (300 MHz, 10% $(CD_3)_2SO$ in $CD_2Cl_2$, 14 mM).**

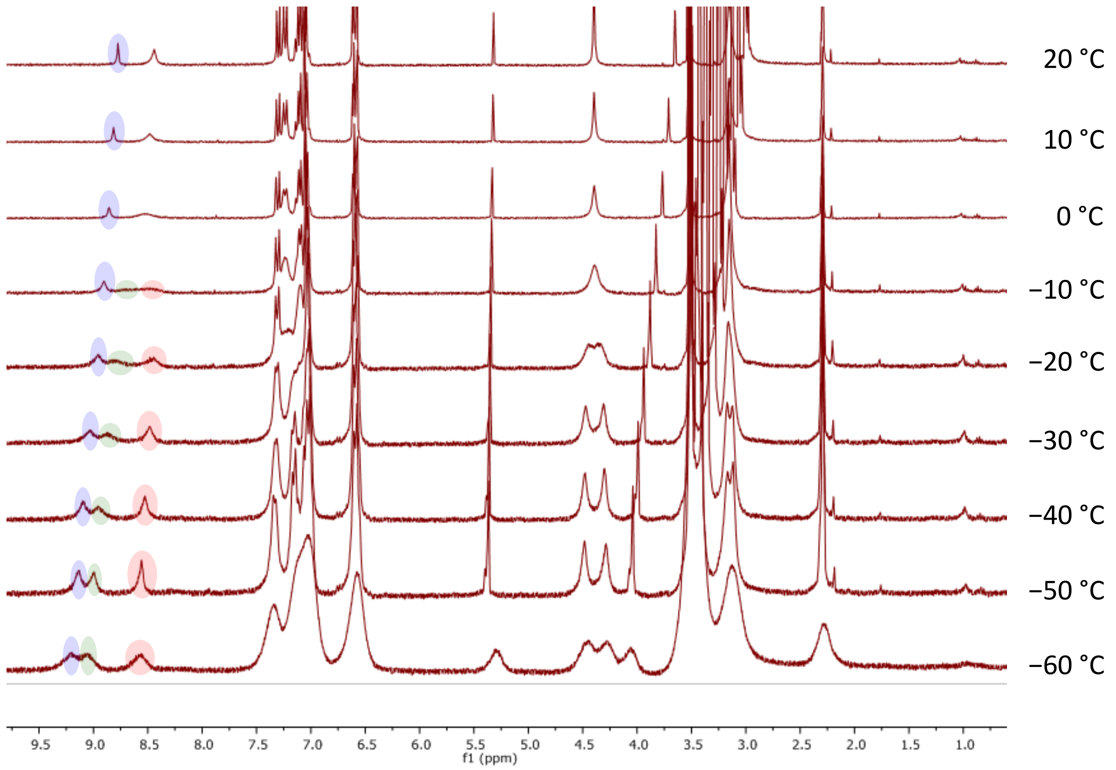


**Figure S15 – VT NMR data for 3a (300 MHz, 50% $(CD_3)_2SO$ in $CD_2Cl_2$, 14 mM).**

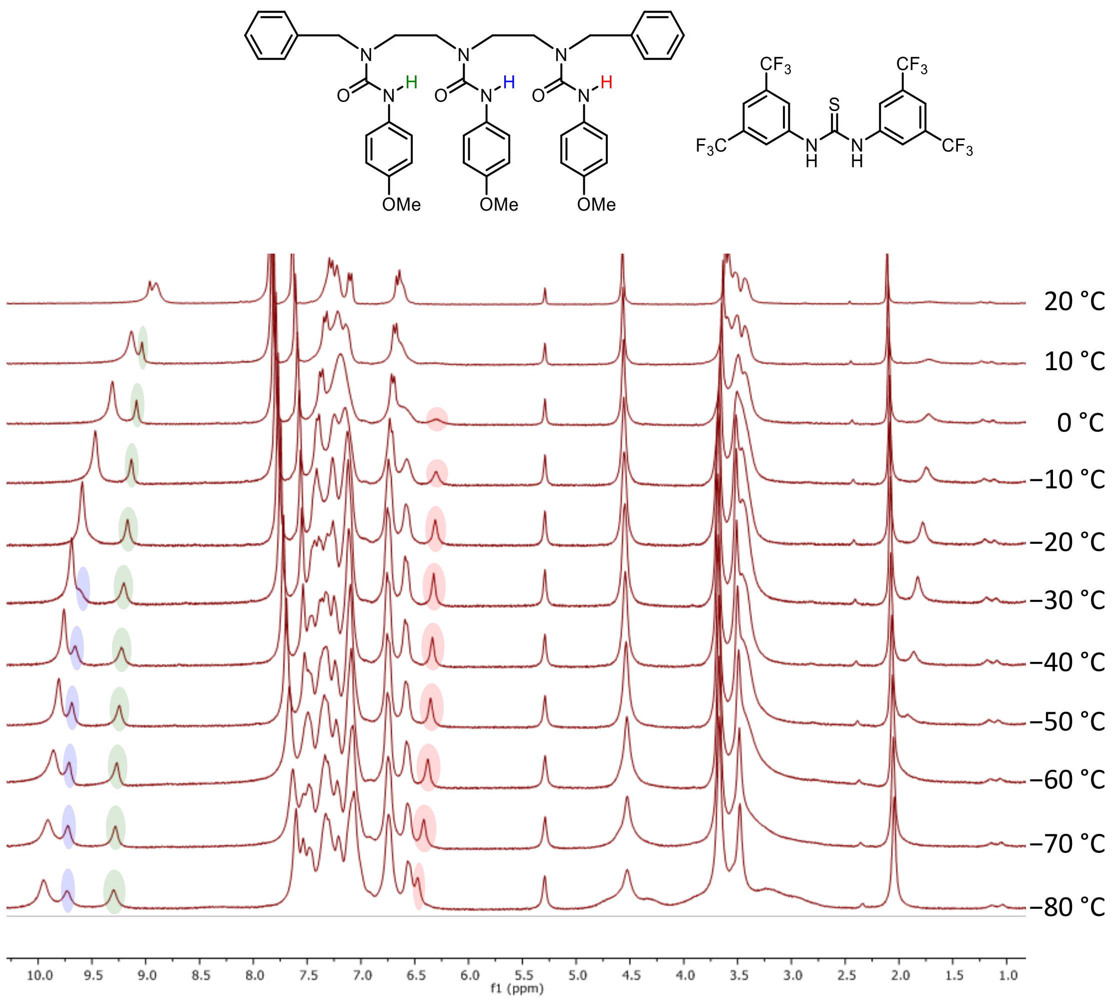


**Figure S16 – VT NMR data for 3a (300 MHz, 2.5 equiv of *N*,*N*'-bis(trifluoromethyl)phenyl thiourea in $CD_2Cl_2$, 14 mM).**

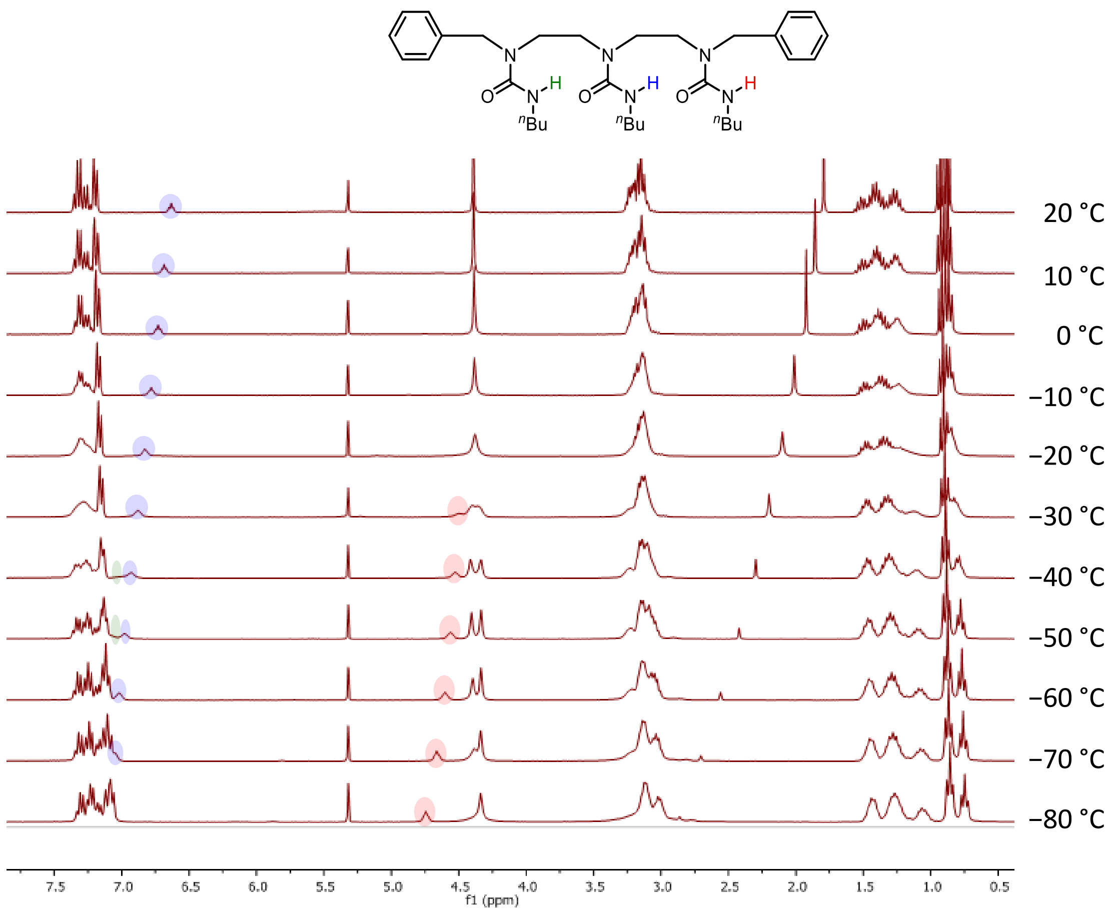


**Figure S17 – VT NMR data for 3b (300 MHz, $CD_2Cl_2$, 17 mM).**

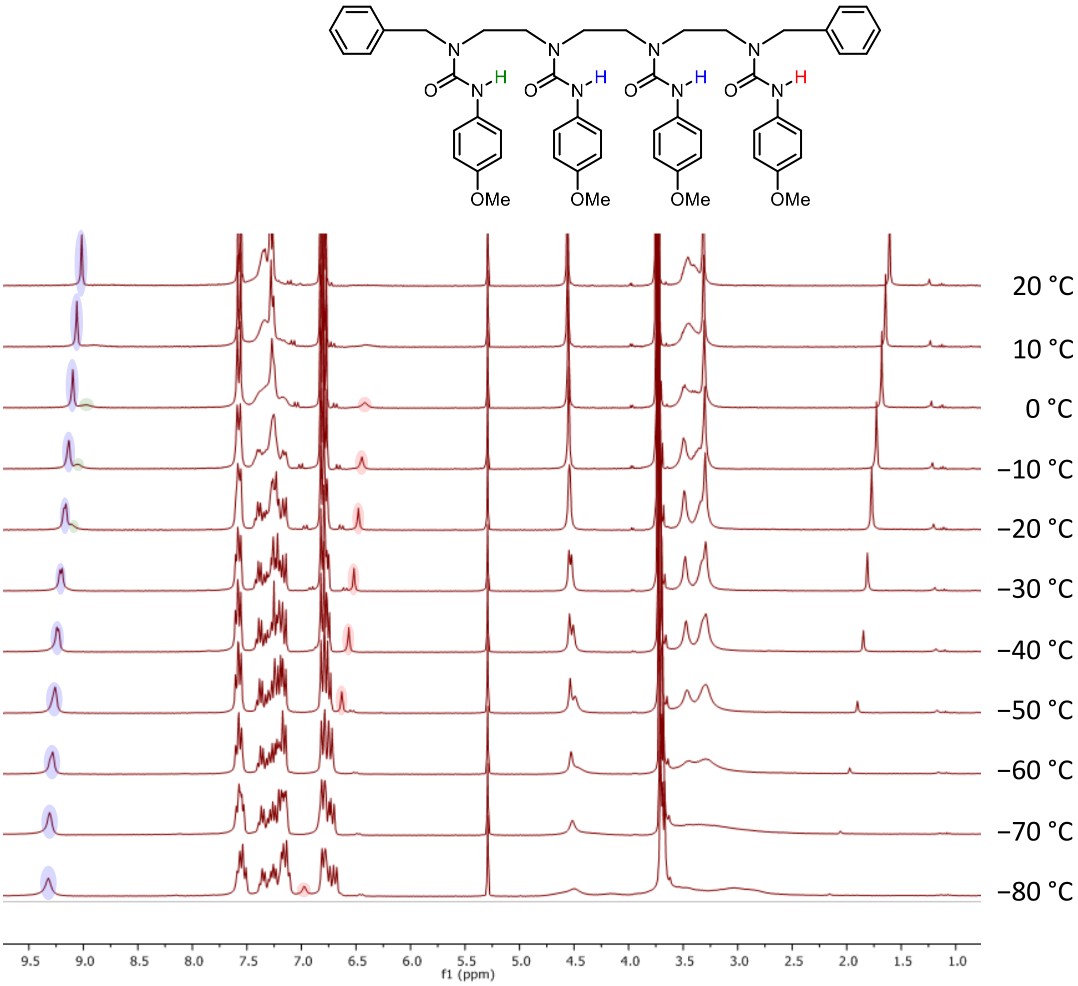


**Figure S18 – VT NMR data for 4a (300 MHz, $CD_2Cl_2$, 11 mM).**

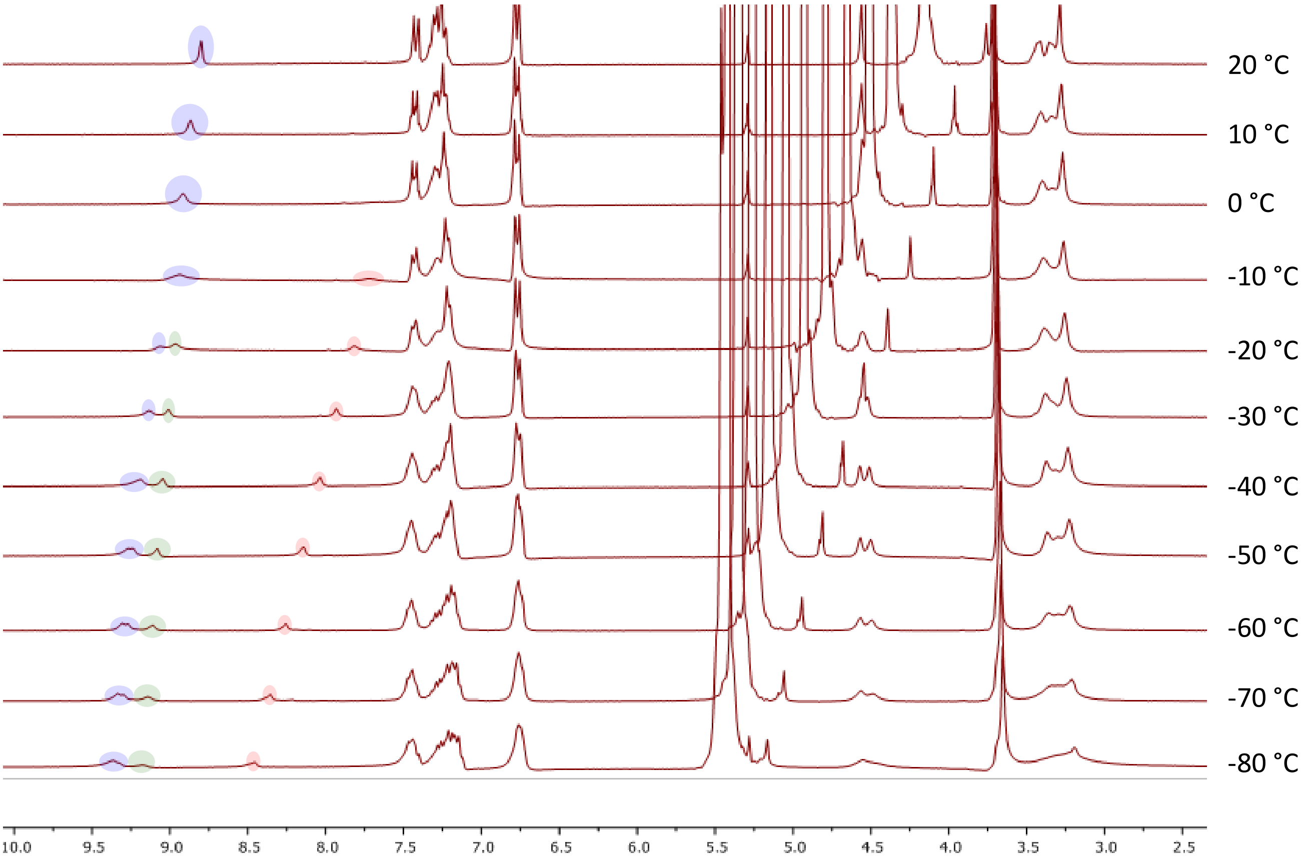


**Figure S19 – VT NMR data for 4a (300 MHz, 40% $CD_3OH$ in $CD_2Cl_2$, 11 mM).**

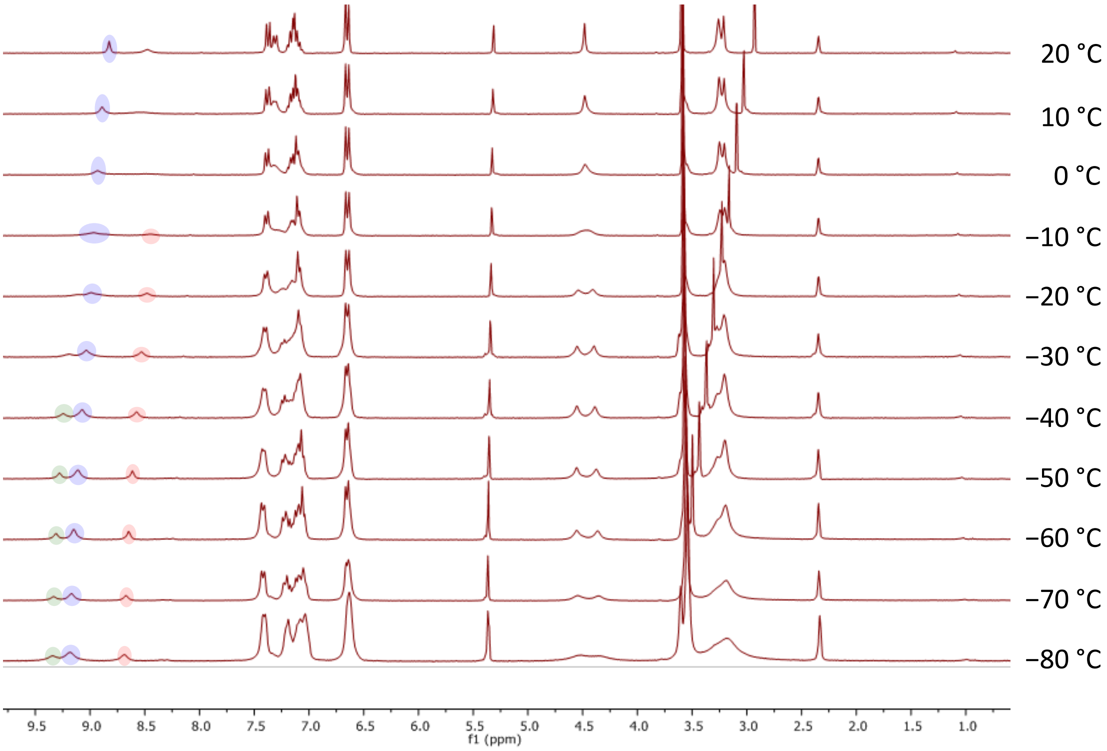


**Figure S20 – VT NMR data for 4a (300 MHz, 10% $(CD_3)_2SO$ in $CD_2Cl_2$, 11 mM).**

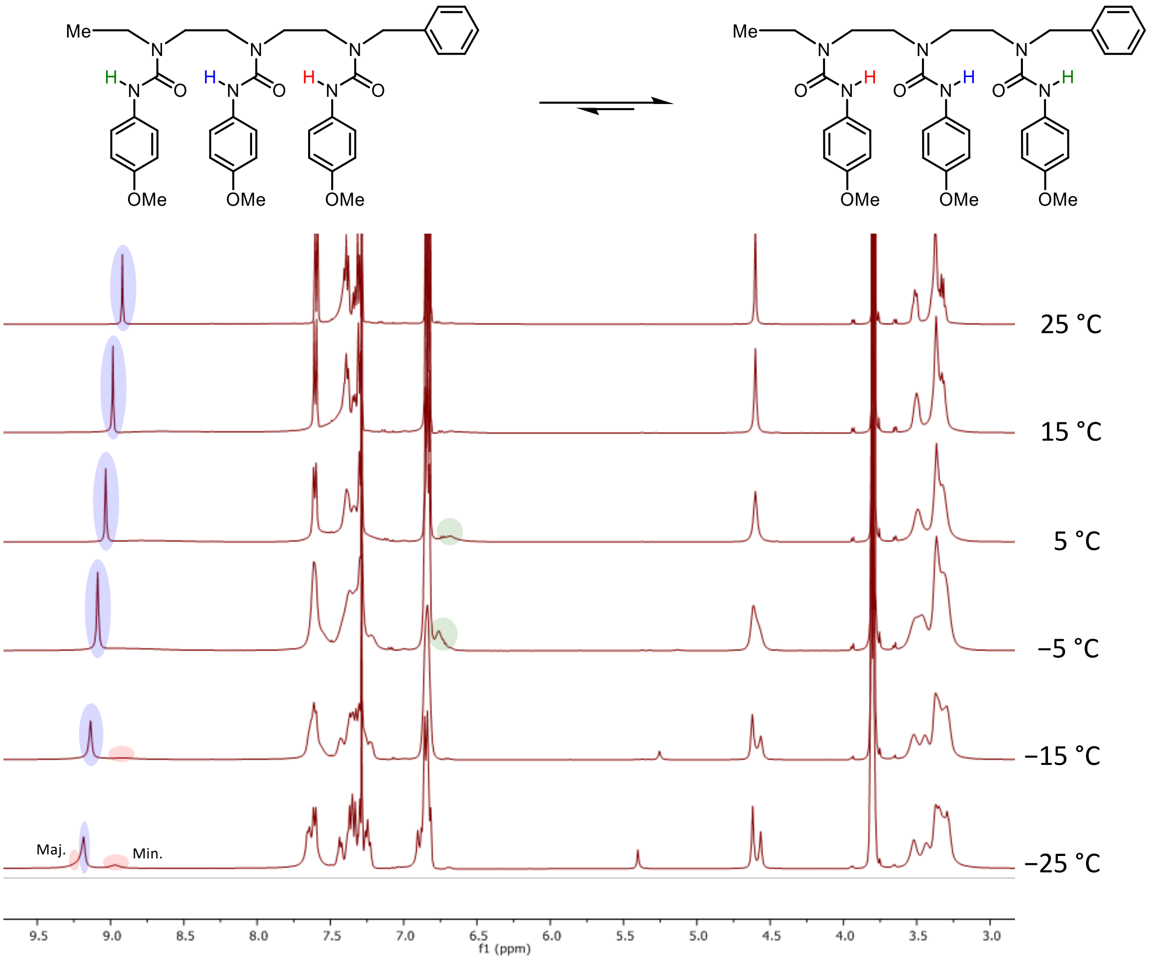


**Figure S21 – VT NMR data for 3c (500 MHz, $CD_2Cl_2$, 15 mM).** Signals at 25 °C: 4.60 (benzylic methylene) and 8.92 ppm (central urea ureido proton). Upon cooling, the signal at 4.60 ppm broadens and decoalesces into two distinct singlets at −15 °C. These decoalesced signals sharpen at −25 °C, where they were present in a ratio of 60:40. The signal at 8.92 ppm (1 H) shifts downfield with decreasing temperature to reach a value of 9.19 ppm at −25 °C. At 5 °C, a new signal appears at 6.76 ppm (1 H), which sharpens and shifts downfield upon further decreasing the temperature. Similarly, new signals emerge at 8.90 (0.40 H) and 9.23 ppm (0.60 H, overlapping) at −15 °C, which behave the same. These data show that there are two conformations in fast exchange on the NMR timescale at 20 °C, which enter the slow-exchange region between −5 °C and −15 °C. These two conformations are the two different directionalities, populated in a 40:60 ratio at −25 °C. Each of the directionalities have two hydrogen-bonded ureido protons, and a non-hydrogen-bonded ureido proton. The signals at 6.76 and 9.19 ppm at −25 °C correspond to the non-hydrogen-bonded and central hydrogen-bonded ureido protons, respectively of both conformers. The signals at 8.90 (0.40 H) and 9.23 ppm (0.60 H) at −25 °C correspond to the peripheral hydrogen-bonded ureido protons in the minor and major conformers, respectively.

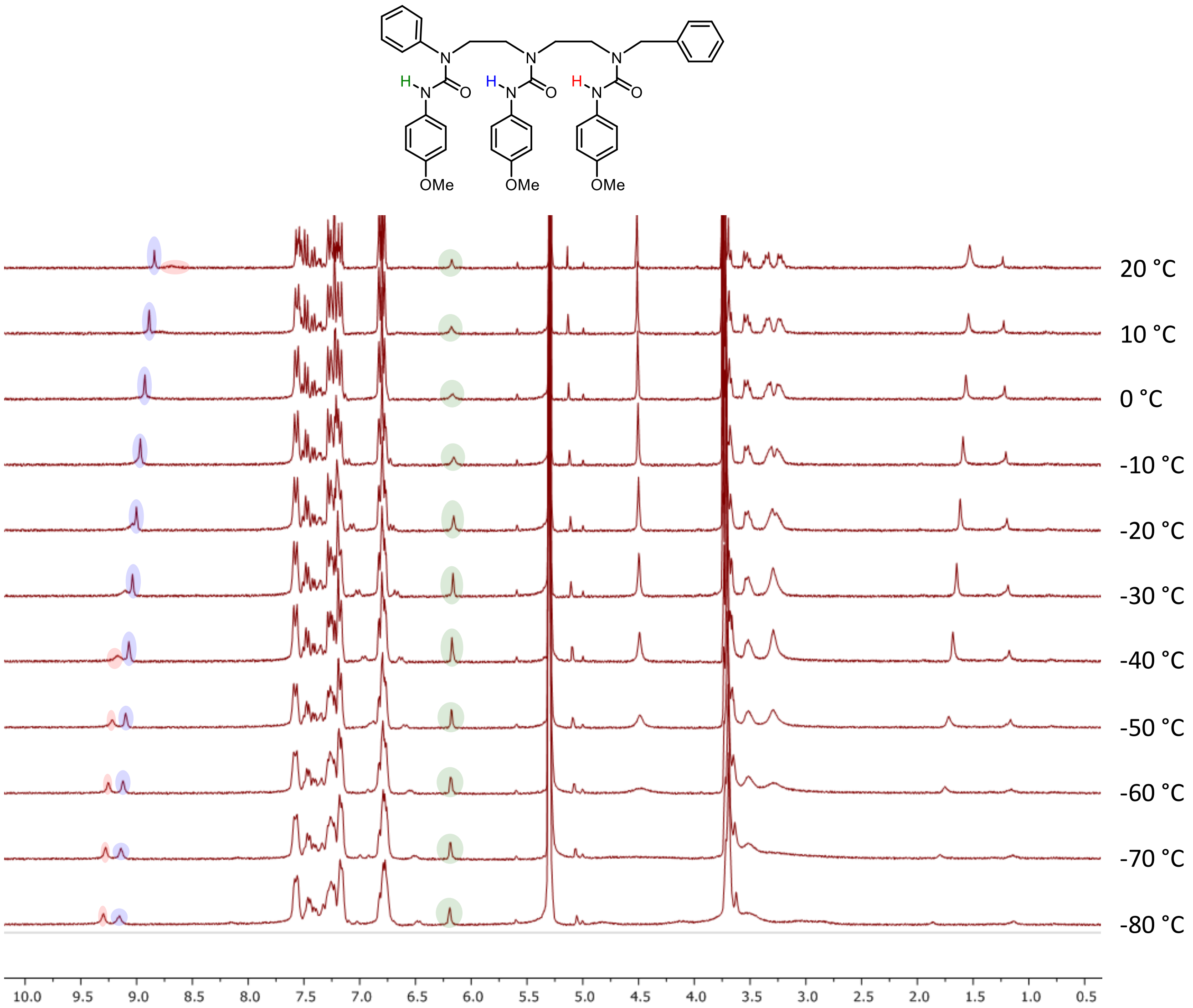


**Figure S22 – VT NMR data for 3d (300 MHz, $CD_2Cl_2$, 14 mM).**

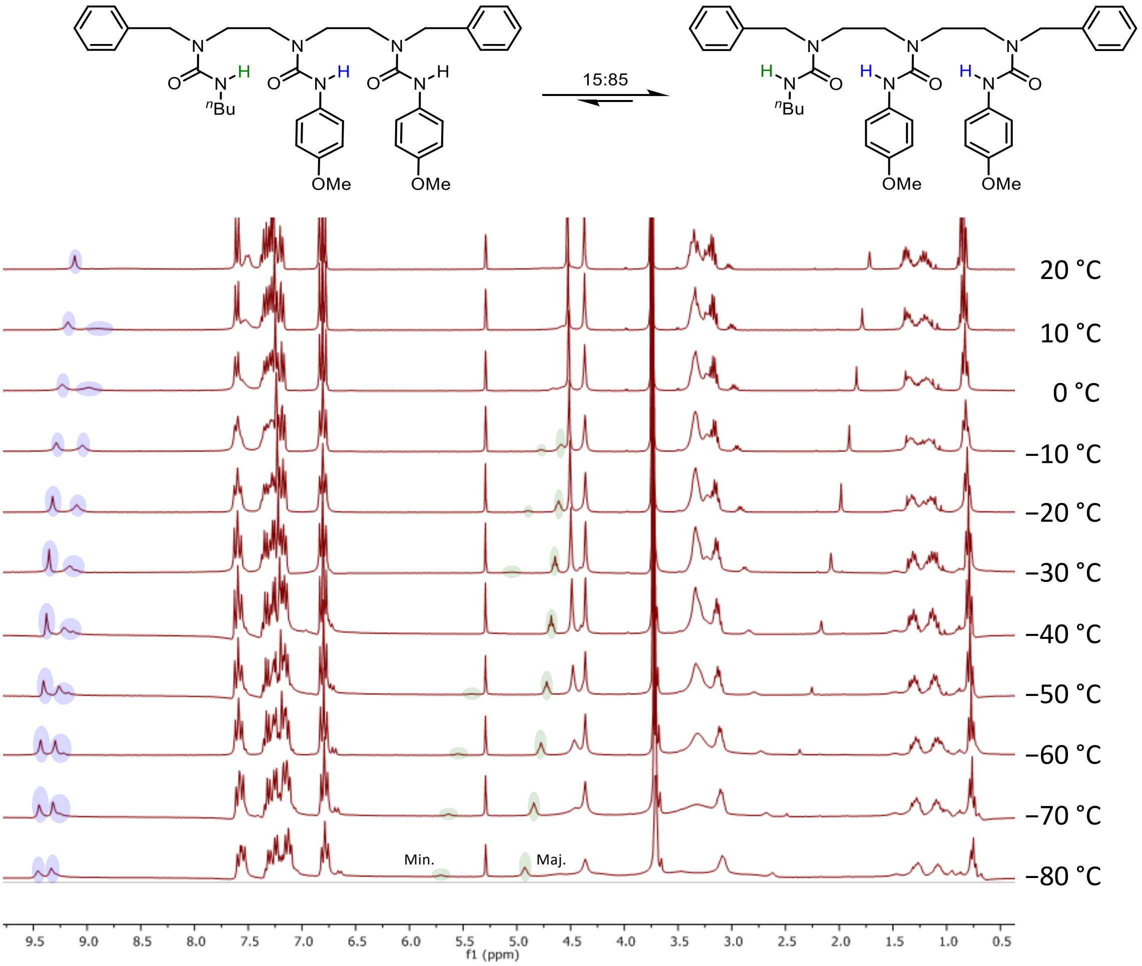


**Figure S23 – VT NMR data for 3e (300 MHz, $CD_2Cl_2$, 15 mM).** Signals at 20 °C: 4.37 (alkyl urea benzylic methylene), 4.53 (aryl urea benzylic methylene), 8.65 (peripheral aryl urea ureido proton) and 9.11 ppm (central ureido proton) at 20 °C. Upon cooling, the signal at 4.37 ppm stays approximately constant until it broadens at −20 °C and decoalesces into two singlets (0.30 H and 1.70 H) at −30 °C. The signal at 4.53 ppm broadens until it cannot be observed at −80 °C. The signals at 8.65 (very broad) and 9.11 ppm sharpen and shift downfield with decreasing temperature. At −10 °C, signals appear at 4.59 (0.85 H) and 4.77 ppm (0.15 H), which sharpen and shift downfield upon further decreasing the temperature. Another signal appears at 9.10 ppm (0.15 H) at −30 °C, which shifts downfield with further cooling. These data indicate that **3e** exists as two conformations populated in a 85:15 ratio at −60 °C. These conformations are the two directionalities, and the major conformer is the directionality where the *n*-butyl urea is at the N-terminus. This is evidenced by the observable decoalescence of the alkyl urea benzylic methylene into two signals in a 85:15 ratio. At −60 °C, the two conformers are in slow exchange on the NMR timescale, and so three ureido protons can be observed for each. For the major conformer, there is a non-hydrogen bonded alkyl ureido proton (4.78 ppm, 0.85 H), and two aryl ureido protons that are (9.30 and 9.43 ppm, 0.85 H each). For the minor conformer, there is an alkyl ureido proton in hydrogen bonding (5.55 ppm, 0.15 H) and an aryl ureido proton in hydrogen bonding (9.21 ppm, 0.15 H).

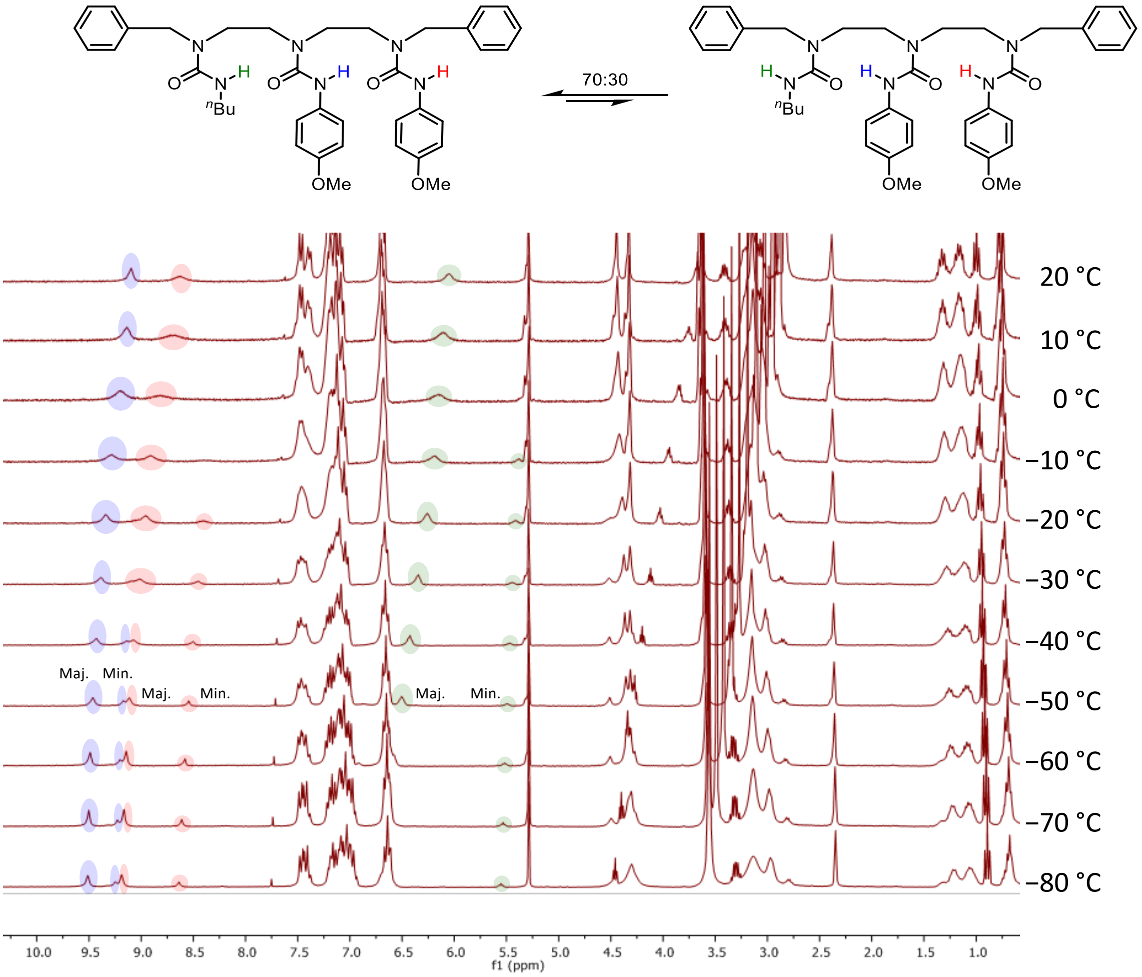


**Figure S24 – VT NMR data for 3e (300 MHz, 10% $(CD_3)_2SO$ in $CD_2Cl_2$, 15 mM).** Signals at 20 °C: 4.33 (alkyl urea benzylic methylene), 4.44 (aryl urea benzylic methylene), 6.05 (alkyl urea ureido proton), 8.62 (peripheral aryl urea ureido proton) and 9.09 ppm (central ureido proton) at 20 °C. Upon cooling, the signal at 4.33 ppm broadens throughout the experiment. The same is observed for the signal at 4.44 ppm but it also moved upfield. The signals at 6.05, 8.62 and 9.09 ppm shift downfield with decreasing temperature. Additional signals appear at 5.38 (0.30 H), 8.38 (0.30 H) and 9.07 ppm (0.30 H) at −10 °C, which sharpen and shift downfield upon further cooling. These observations can be explained by the population of two conformers in a 70:30 ratio. This is not elucidated by an observable decoalescence of the benzylic methylene signals but can be inferred from the chemical shifts and relative integrations of the ureido protons. The two conformers correspond to the two directionalities and in each case, $d_6$-DMSO hydrogen bonds to the exposed ureido proton. At slow exchange, each of the two conformers contain three ureido protons. In one directionality, the *n*-butyl urea acts as an intramolecular HBA, forcing the other two aryl ureido protons into intramolecular hydrogen bonding and leaving its own ureido proton to hydrogen bond to $d_6$-DMSO. This is shown by the signals at 5.52 (alkyl urea ureido proton, 0.30 H), 8.58 (peripheral aryl urea ureido proton, 0.30 H) and 9.20 ppm (central ureido proton, 0.30 H) at −60 °C. This also identifies this directionality as the minor conformer. In the other directionality (the major conformer), where the *n*-butyl urea acts as an intramolecular HBD, one of the aryl ureas is in an intramolecular hydrogen bond and the other an intermolecular hydrogen bond with $d_6$-DMSO. This shows signals at 6.58 (alkyl urea ureido proton, 0.70 H), 9.18 (peripheral aryl urea ureido proton) and 9.49 ppm (central ureido proton) at −60 °C.

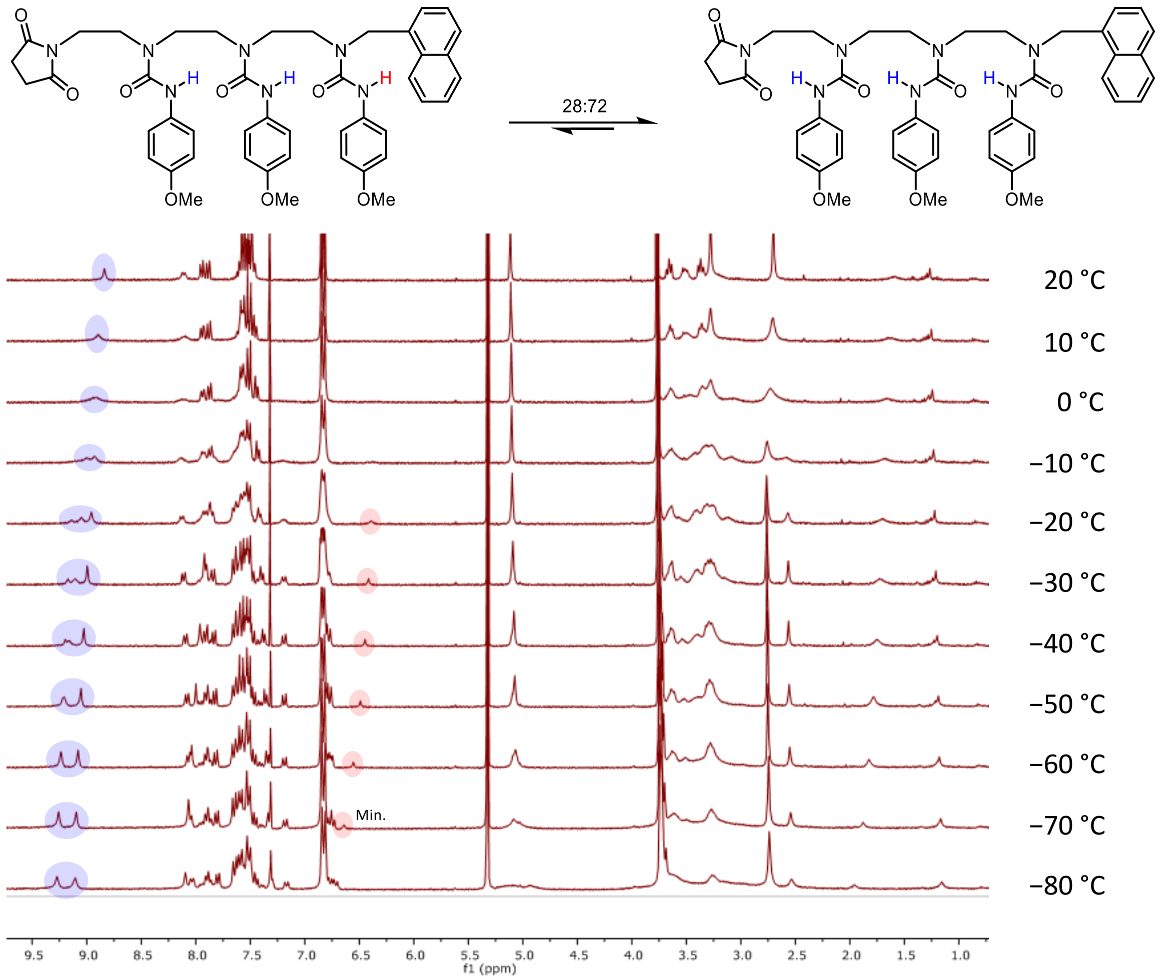


**Figure S25 – VT NMR data for 3f (300 MHz, $CD_2Cl_2$, 12 mM).** Signals at 20 °C: 2.70 (succinimidyl methylenes), 5.11 (benzylic methylene) and 8.84 ppm (one ureido proton) at 20 °C. Upon cooling, the signal at 2.70 ppm broadens and decoalesces at −10 °C into two singlets, which then sharpen upon further decreasing the temperature (2.87 H and 1.13 H at −60 °C). The signal at 5.11 ppm also broadens and decoalesces into two closely overlapping singlets at −40 °C. The signal at 8.84 ppm moves downfield with decreasing temperature before decoalescing into three signals at −20 °C, which then shift further downfield. Additionally, a new signal appears at 6.39 ppm (0.28 H) at −10 °C, which sharpens and moves downfield upon further decreasing the temperature. These data are consistent with the presence of two conformations populated in a 28:72 ratio at −60 °C, which are the two different directionalities. This is indicated by the two different environments of the succinimide at slow exchange – one where it is hydrogen bonding to the adjacent urea, and one where it is not. Finally, the two different conformations give rise to six different environments for ureido protons. Five of these ureido protons hydrogen bond either to other ureas or to the succinimide, but the sixth ureido proton is not hydrogen bonded, giving rise to a signal at 6.39 ppm at −10 °C. This signal also integrates for 0.28 H at −60 °C, and the other ureido proton integrations account for the remaining 2.72 H, indicating that the conformer where the succinimide is at the C-terminus is the minor conformer.

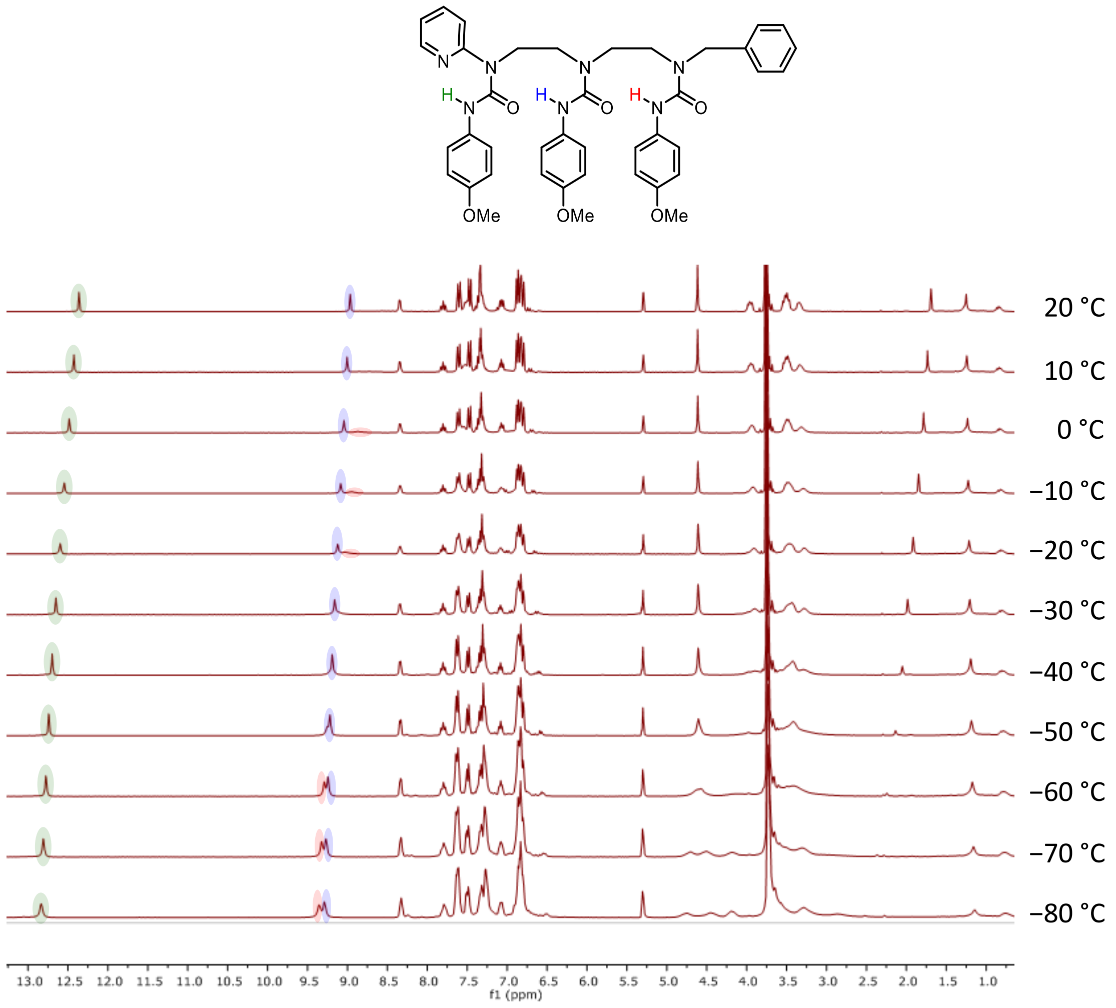


**Figure S26 – VT NMR data for 3g (300 MHz, $CD_2Cl_2$, 14 mM).**

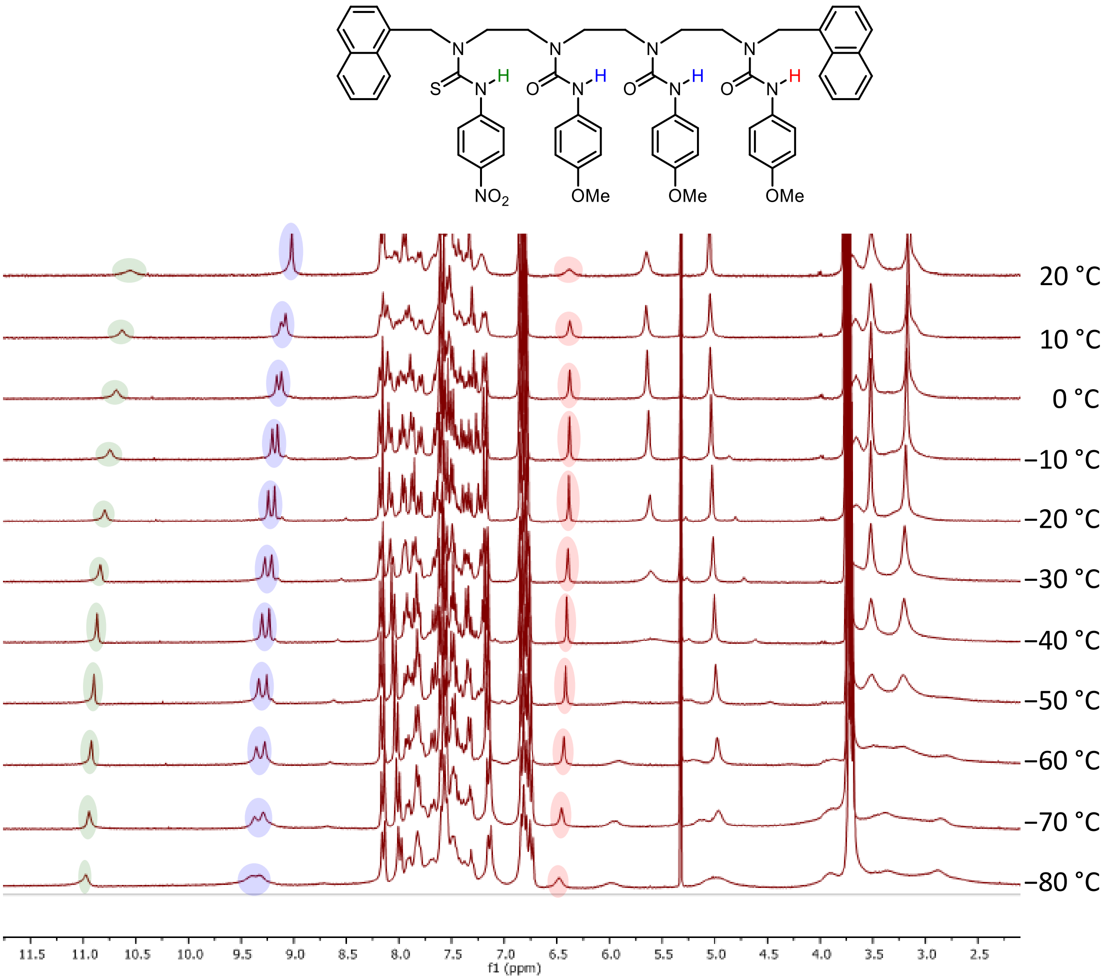


**Figure S27 – VT NMR data for 5a (300 MHz, $CD_2Cl_2$, 19 mM).**

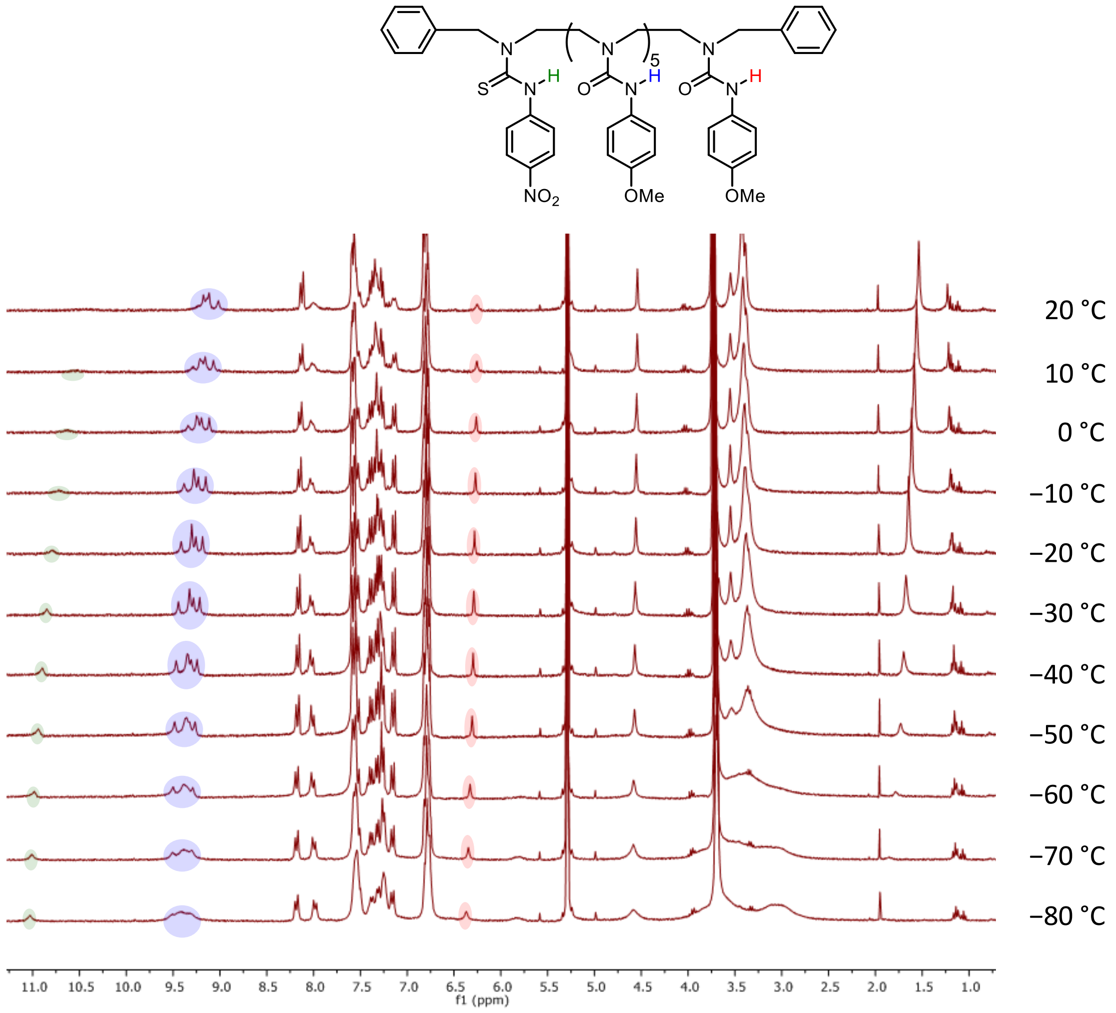


**Figure S28 – VT NMR data for 5b (300 MHz, $CD_2Cl_2$, 61 mM).**

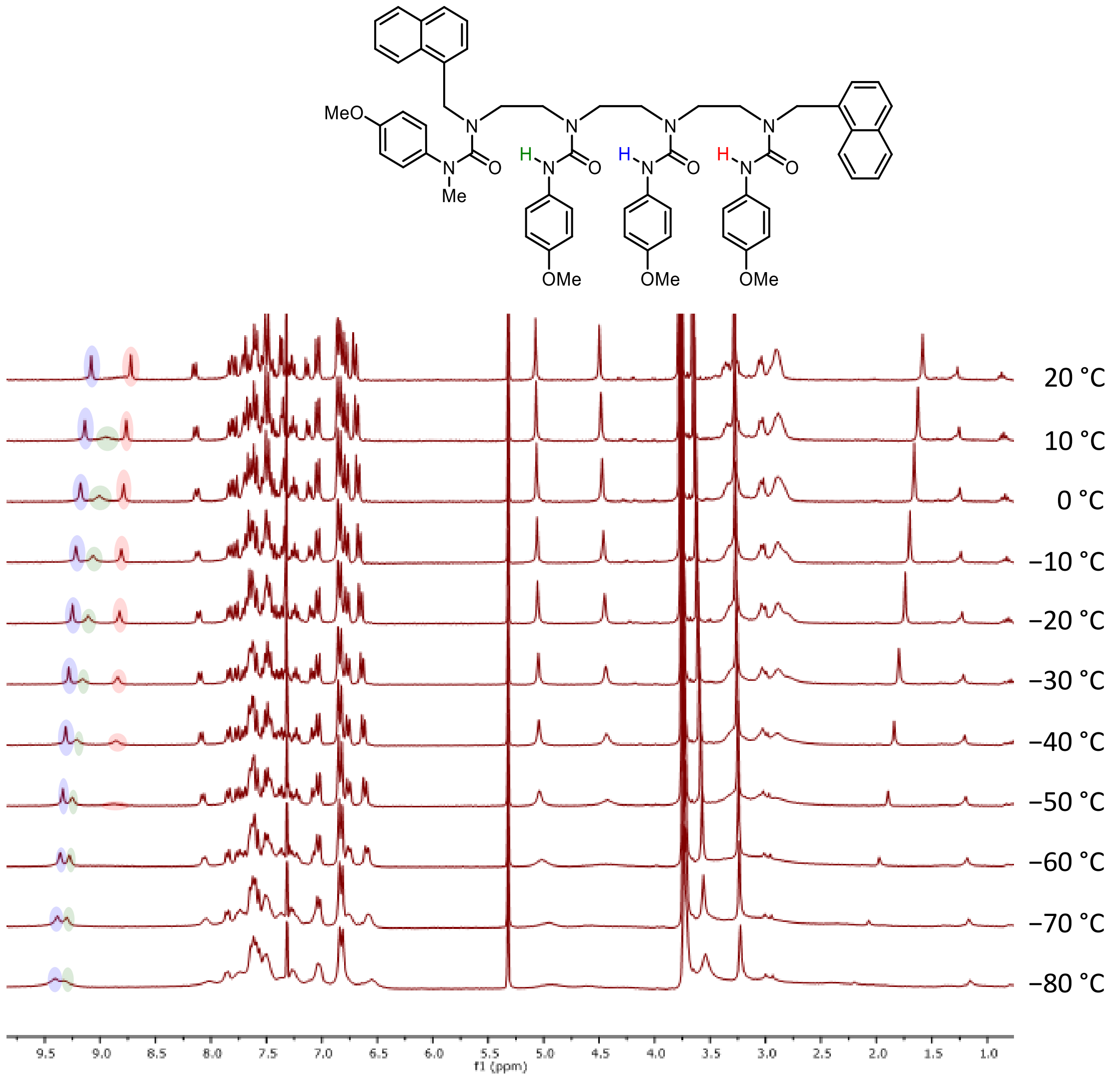


**Figure S29 – VT NMR data for 6a (300 MHz, $CD_2Cl_2$, 19 mM).**

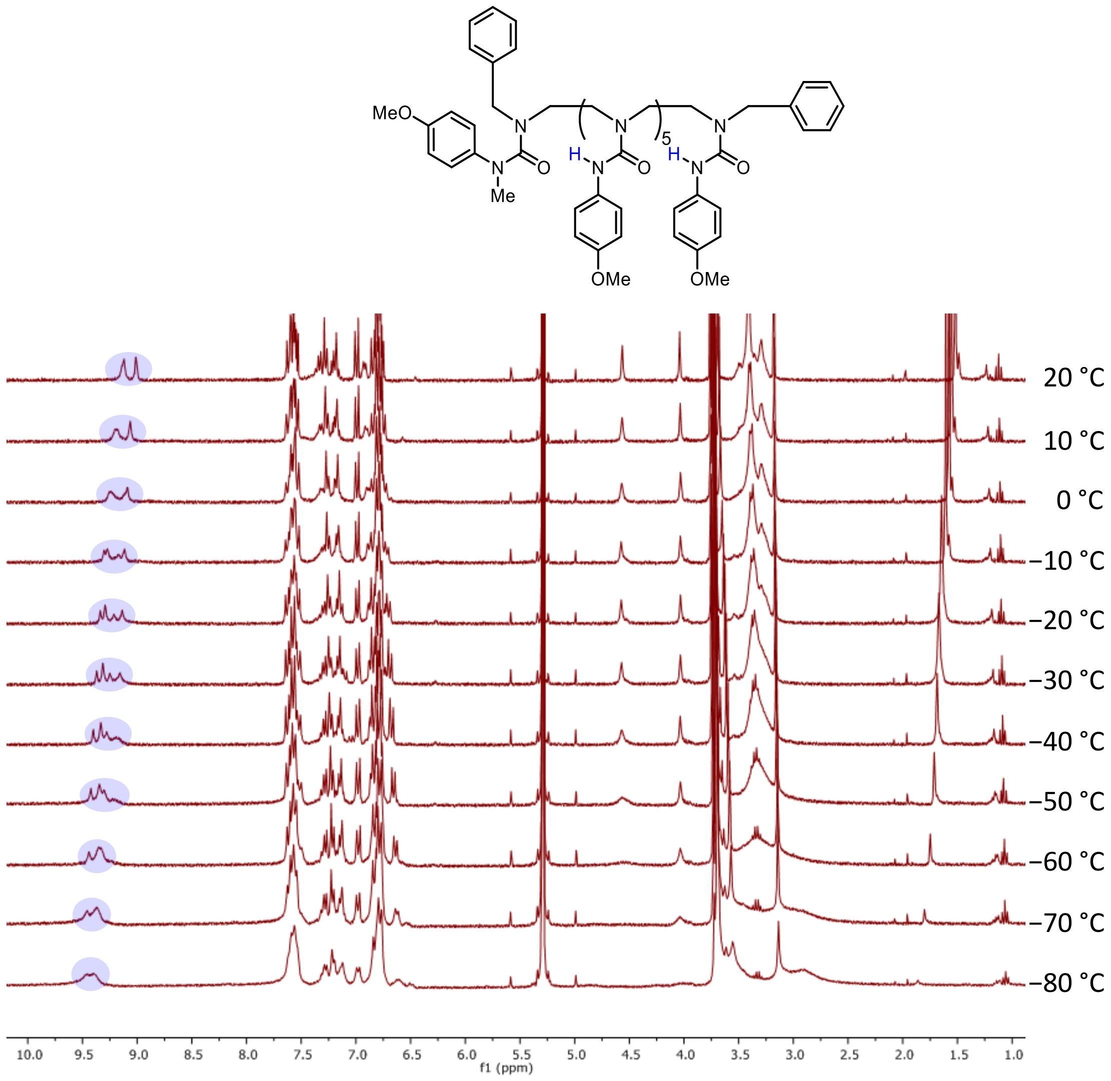


**Figure S30 – VT NMR data for 6b (300 MHz, $CD_2Cl_2$, 49 mM).**

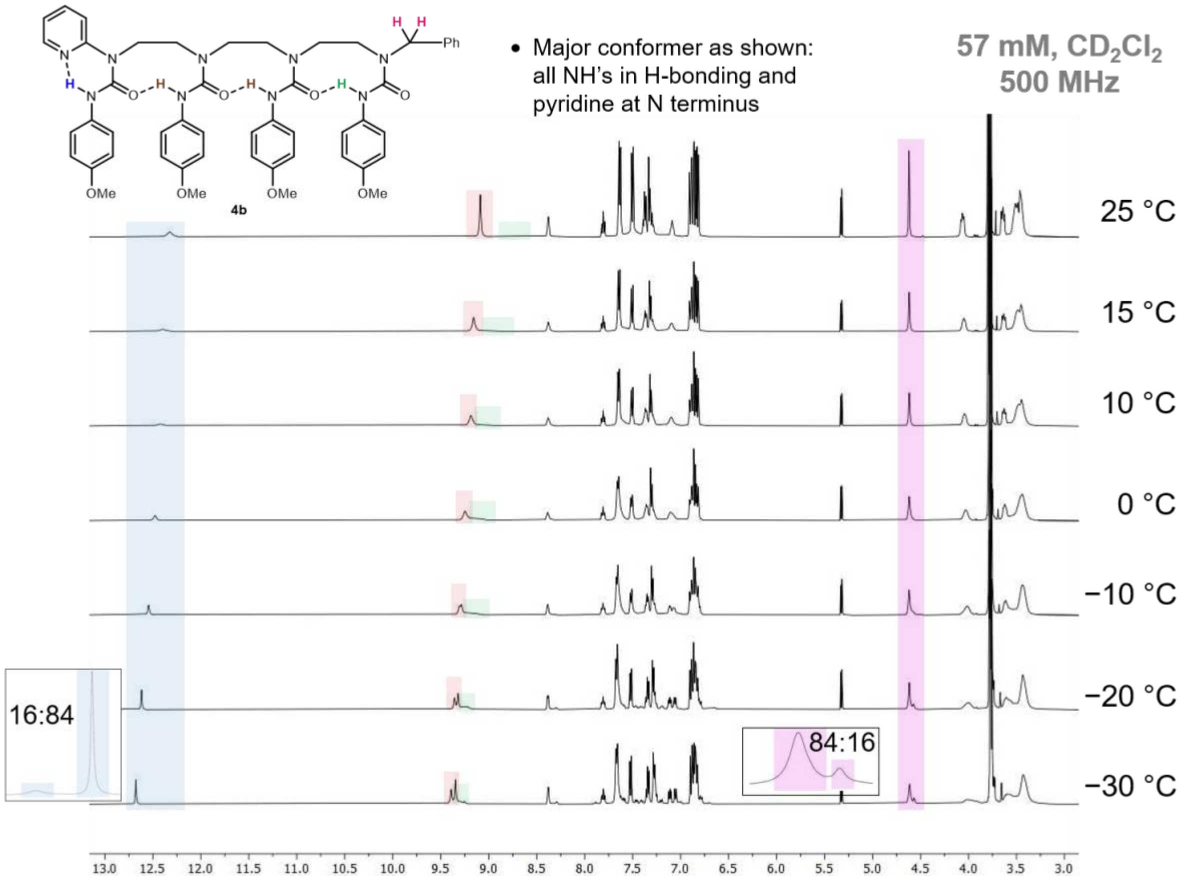


**Figure S31 – VT NMR data for 4b (500 MHz, $CD_2Cl_2$, 57 mM).**

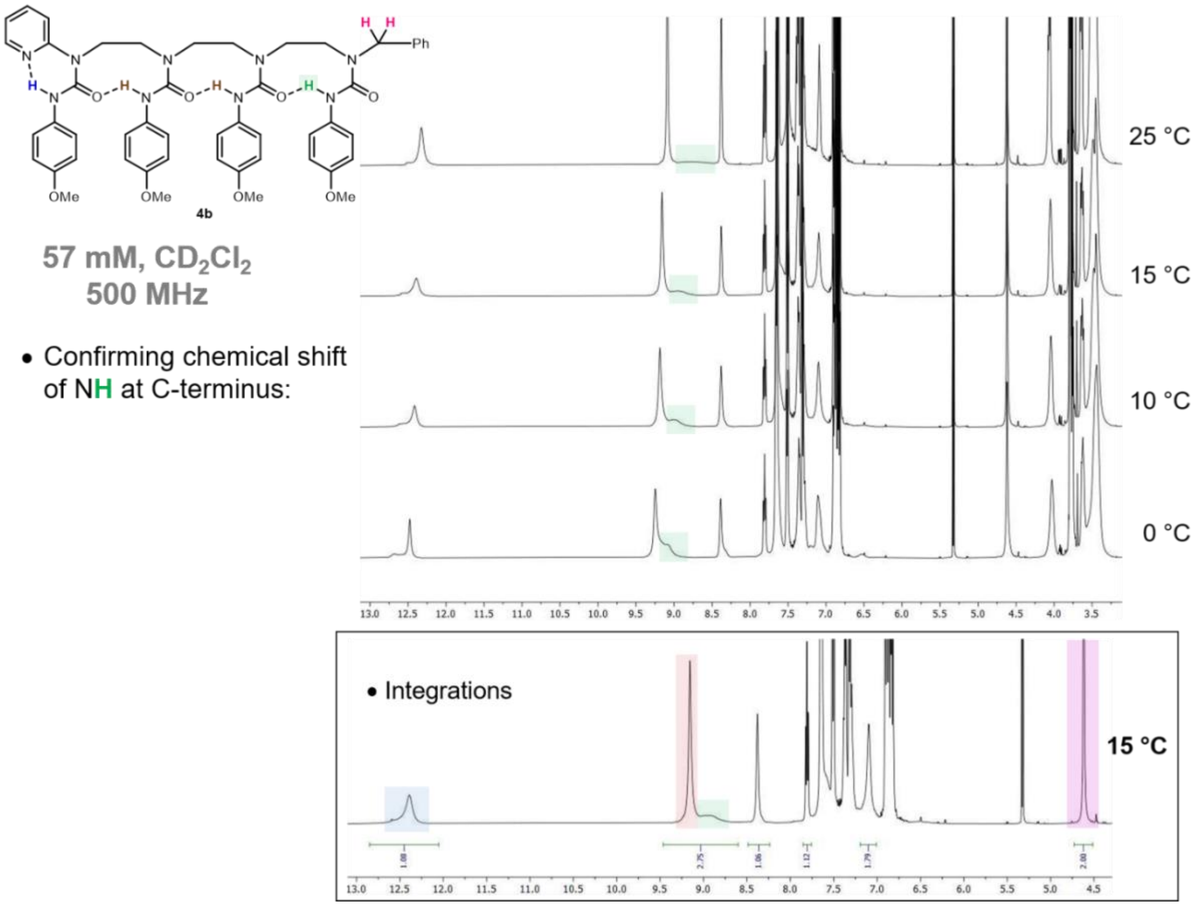


**Figure S32 – VT NMR data for monitoring the C-terminal N–H of 4b (500 MHz, $CD_2Cl_2$, 57 mM).**

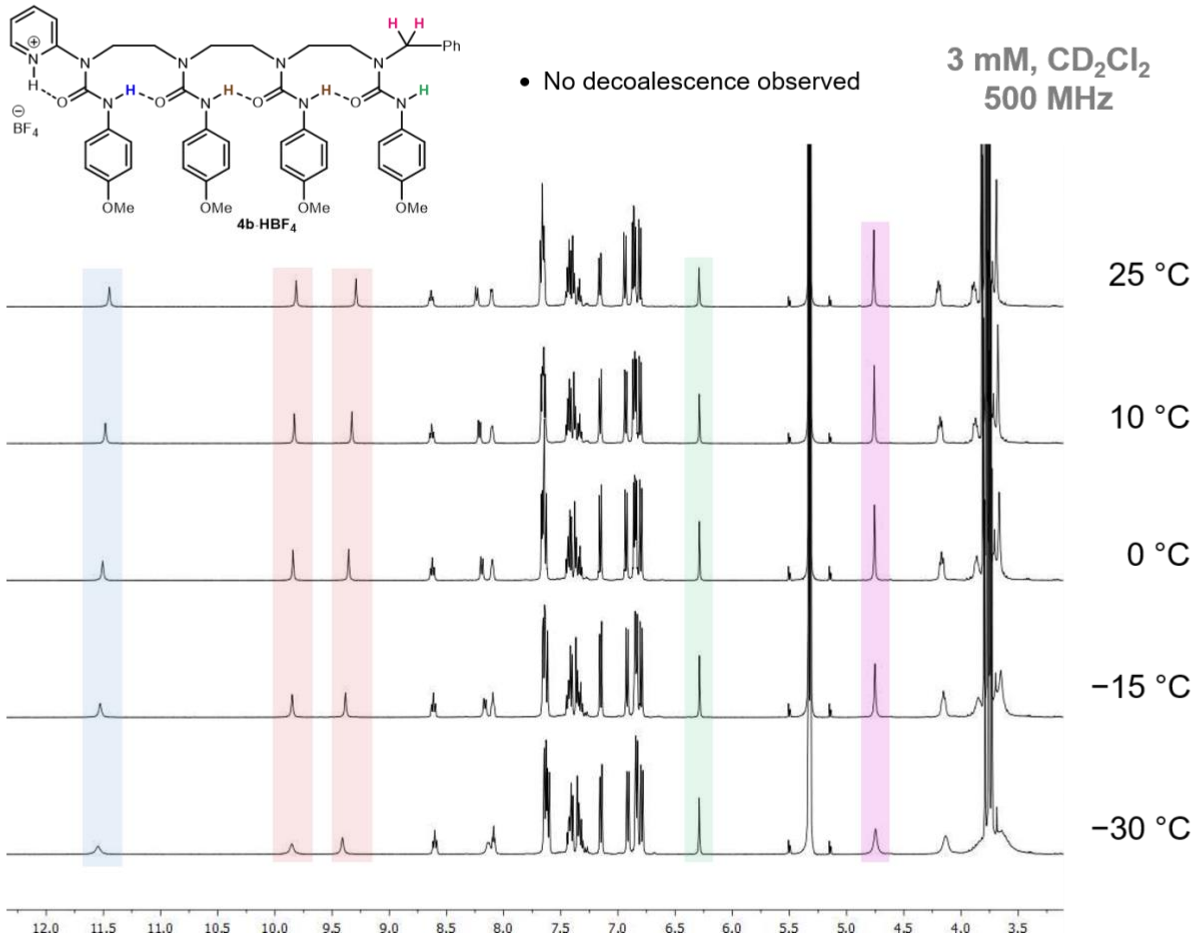


**Figure S33 – VT NMR data for 4b.HBF$_4$ (500 MHz, CD$_2$Cl$_2$, 3 mM).**

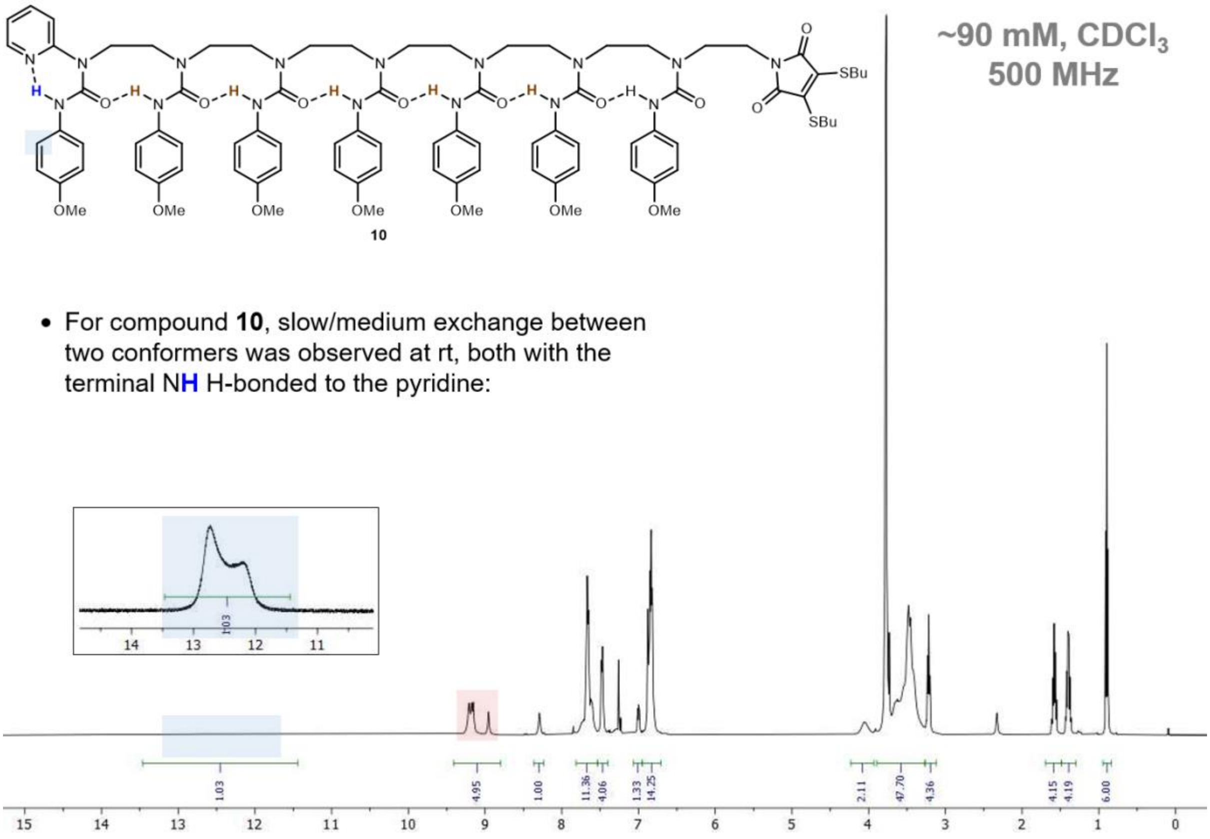


**Figure S34 – $^{1}$H NMR spectrum of 10 (500 MHz, $CDCl_3$, 90 mM).**

• Coalescence was observed upon heating:

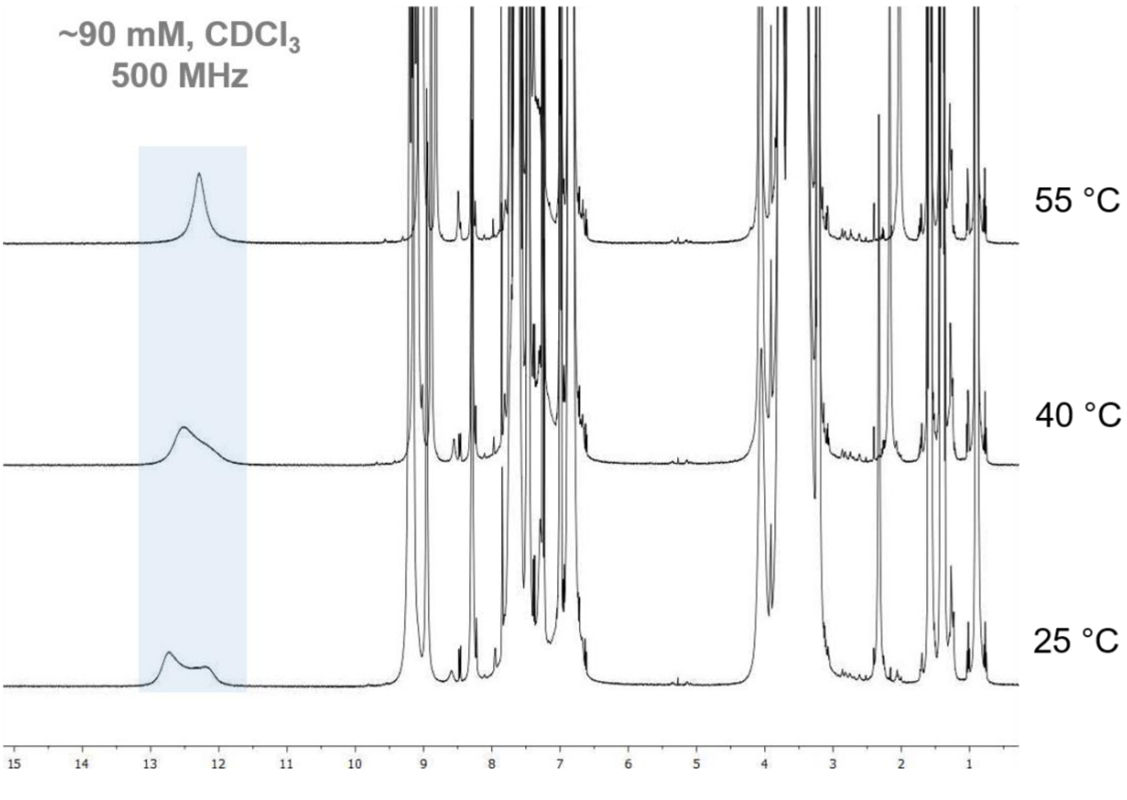


**Figure S35 – VT NMR data for 10 (500 MHz, $CDCl_3$, 90 mM).**

<u>Eyring Analyses</u>

All Eyring plots were constructed using rate constants that were attained by line shape fitting of experimental spectra at different temperatures. Spectra were simulated using SpinWorks 4 NMR processing software (available at ftp://davinci.chem.umanitoba.ca/pub/marat/SpinWorks/) with appropriate exchange vectors. For each temperature, the corresponding rate of hydrogen-bond-directionality reversal was estimated using the dynamic NMR simulation module DNMR3. The Eyring-Polanyi equation was rearranged to give an equation of the form y = mx + c such that a plot of *1/T* against *ln(k/T)* gives a straight line of gradient $\frac{-\Delta H^{\ddagger}}{R}$ and intercept $ln\frac{k_B}{h} + \frac{\Delta S^{\ddagger}}{R}$. These data were used with the Gibbs free energy equation to extrapolate $\Delta G^{\ddagger}$ at 298 K. Examples of simulation of systems in mutual exchange and non-mutual exchange are shown below.

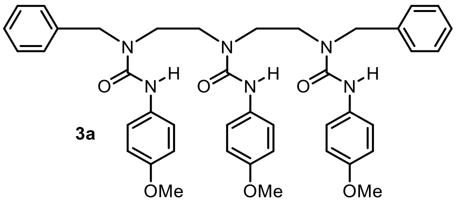

3a

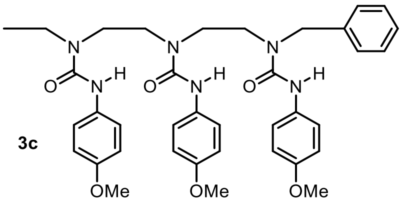

3c

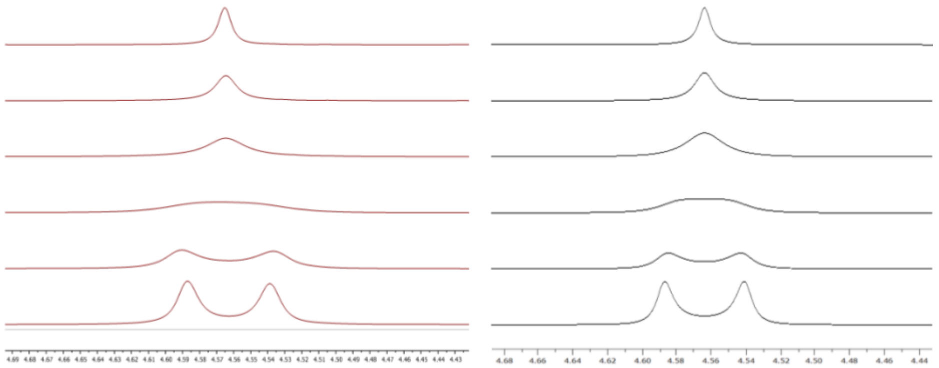

| *T* / K | *k* / s$^{-1}$ |
|---|---|
| 298 | 250 |
| 288 | 150 |
| 278 | 90 |
| 268 | 51 |
| 258 | 27 |
| 248 | 17 |

**Figure S36 – Experimental (left) and simulated (right) spectra for 3a.**

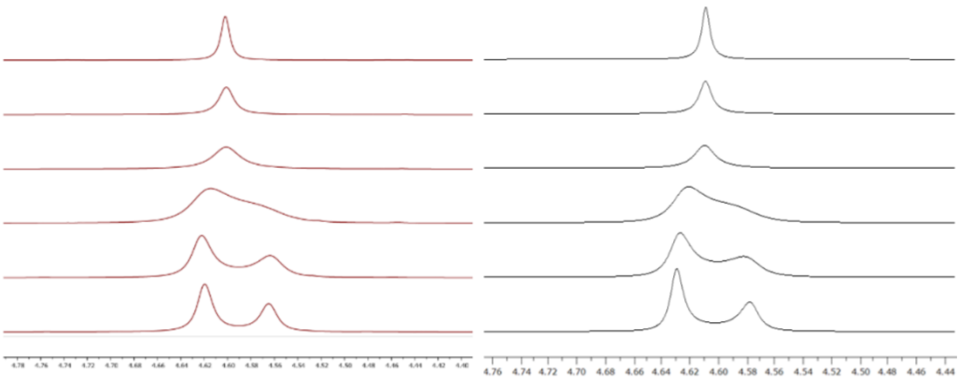

| *T* / K | *k* / s$^{-1}$ |
|---|---|
| 298 | 330 |
| 288 | 200 |
| 278 | 120 |
| 268 | 44 |
| 258 | 27 |
| 248 | 15 |

**Figure S37 – Experimental (left) and simulated (right) spectra for 3c.**

**Table S1 – Eyring plot for 1.**

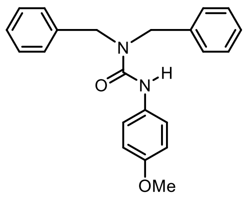


$\Delta G^{\ddagger}_{298\ K}$ = 48.9 kJ mol$^{-1}$

($CD_2Cl_2$)

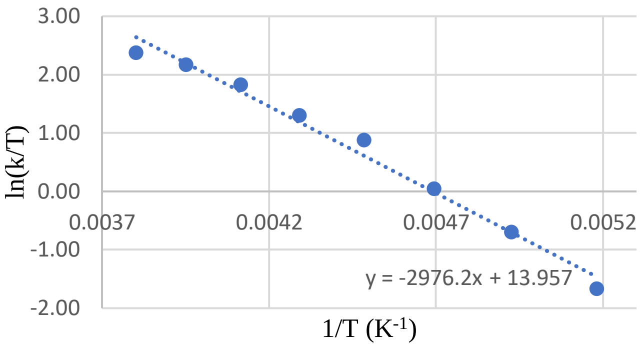


| **Temperature (K)** | **Rate Constant (s$^{-1}$)** | **1/T (K$^{-1}$)** | **ln(k/T)** | **ΔG$^{\ddagger}$ (kJ mol$^{-1}$)** | **t½ (s)** |
|---|---|---|---|---|---|
| 193 | 36 | 0.0052 | -1.6792 | 40.4763 | 0.0096 |
| 203 | 100 | 0.0049 | -0.7080 | 41.2913 | 0.0035 |
| 213 | 220 | 0.0047 | 0.0323 | 42.1064 | 0.0016 |
| 223 | 530 | 0.0045 | 0.8657 | 42.9215 | 0.0007 |
| 233 | 850 | 0.0043 | 1.2942 | 43.7365 | 0.0004 |
| 243 | 1500 | 0.0041 | 1.8202 | 44.5516 | 0.0002 |
| 253 | 2200 | 0.0040 | 2.1628 | 45.3667 | 0.0002 |
| 263 | 2800 | 0.0038 | 2.3652 | 46.1817 | 0.0001 |

**Table S2 – Eyring plot for 1 (40% $CD_3OH$ in $CD_2Cl_2$).**

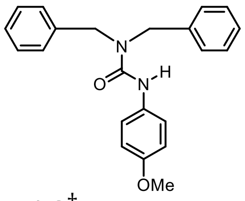


$\Delta G^{\ddagger}_{298\ K}$ = 59.9 kJ mol$^{-1}$

(40% $CD_3OD$ in $CD_2Cl_2$)

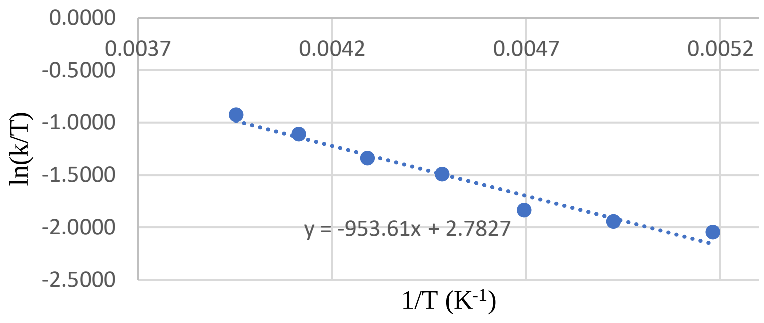


| Temperature (K) | Rate Constant ($s^{-1}$) | 1/T ($K^{-1}$) | ln(k/T) | ΔG‡ (kJ mol$^{-1}$) | $t_{½}$ (s) |
|---|---|---|---|---|---|
| 193 | 25 | 0.0052 | -2.0438 | 41.5908 | 0.0139 |
| 203 | 29 | 0.0049 | -1.9459 | 43.3350 | 0.0120 |
| 213 | 34 | 0.0047 | -1.8349 | 45.0791 | 0.0102 |
| 223 | 50 | 0.0045 | -1.4951 | 46.8233 | 0.0069 |
| 233 | 61 | 0.0043 | -1.3402 | 48.5674 | 0.0057 |
| 243 | 80 | 0.0041 | -1.1110 | 50.3116 | 0.0043 |
| 253 | 100 | 0.0040 | -0.9282 | 52.0557 | 0.0035 |

**Table S3 – Eyring plot for 1 (10% $(CD_3)_2SO$ in $CD_2Cl_2$).**

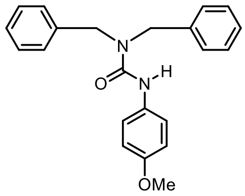


$\Delta G^{\ddagger}_{298\ K}$ = 54.5 kJ mol$^{-1}$

(10% $(CD_3)_2SO$ in $CD_2Cl_2$)

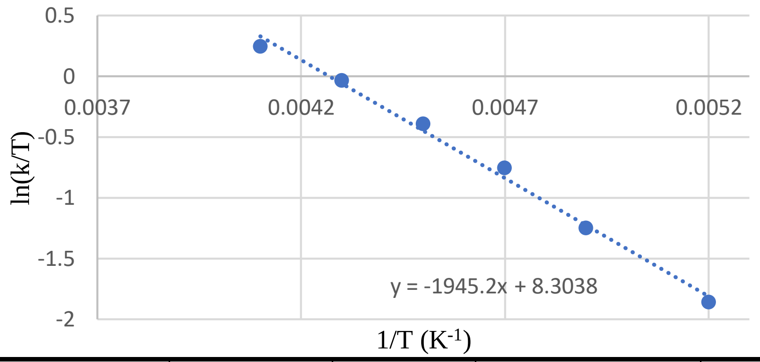


| Temperature (K) | Rate Constant ($s^{-1}$) | 1/T ($K^{-1}$) | ln(k/T) | ΔG‡ (kJ mol$^{-1}$) | $t_{½}$ (s) |
|---|---|---|---|---|---|
| 193 | 30 | 0.0052 | -1.8615 | 40.9945 | 0.0116 |
| 203 | 58 | 0.0049 | -1.2528 | 42.2732 | 0.0060 |
| 213 | 100 | 0.0047 | -0.7561 | 43.5518 | 0.0035 |
| 223 | 150 | 0.0045 | -0.3965 | 44.8305 | 0.0023 |
| 233 | 225 | 0.0043 | -0.0349 | 46.1091 | 0.0015 |
| 243 | 310 | 0.0041 | 0.2435 | 47.3878 | 0.0011 |

**Table S4 – Eyring plot for 2a.**

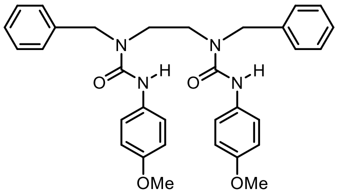


$\Delta G^{\ddagger}_{298\,K}$ = 59.4 kJ mol$^{-1}$

($CD_2Cl_2$)

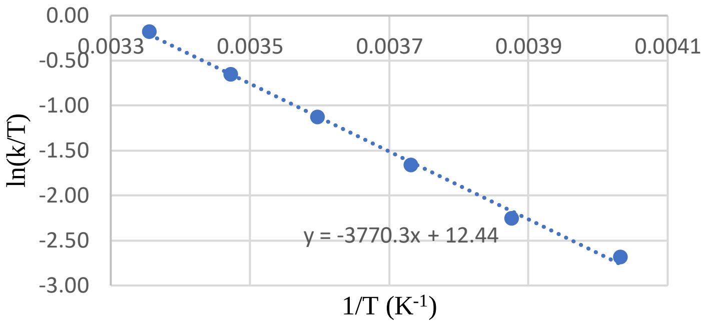


| Temperature (K) | Rate Constant ($s^{-1}$) | 1/T ($K^{-1}$) | ln(k/T) | ΔG‡ (kJ $mol^{-1}$) | t½ (s) |
|---|---|---|---|---|---|
| 248 | 17 | 0.0040 | -2.6802 | 54.6897 | 0.0204 |
| 258 | 27 | 0.0039 | -2.2571 | 55.6309 | 0.0128 |
| 268 | 51 | 0.0037 | -1.6592 | 56.5721 | 0.0068 |
| 278 | 90 | 0.0036 | -1.1278 | 57.5133 | 0.0039 |
| 288 | 150 | 0.0035 | -0.6523 | 58.4544 | 0.0023 |
| 298 | 250 | 0.0034 | -0.1756 | 59.3956 | 0.0014 |

**Table S5 – Eyring plot for 2a (40% $CD_3OH$ in $CD_2Cl_2$).**

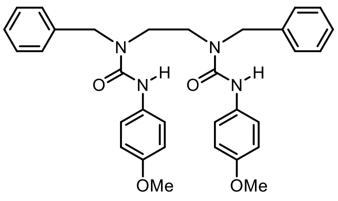


$\Delta G^{\ddagger}_{298\,K}$ = 55.3 kJ mol$^{-1}$

(40% $CD_3OD$ in $CD_2Cl_2$)

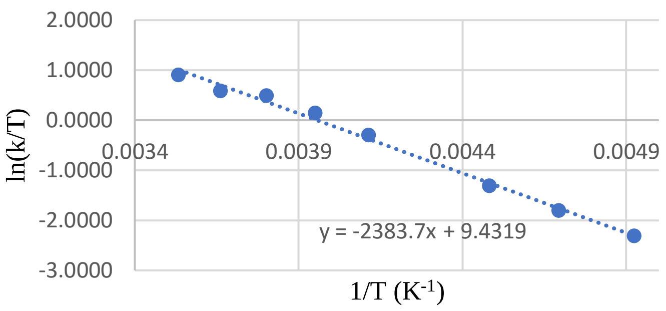


| Temperature (K) | Rate Constant ($s^{-1}$) | 1/T ($K^{-1}$) | ln(k/T) | ΔG‡ (kJ $mol^{-1}$) | t½ (s) |
|---|---|---|---|---|---|
| 203 | 20 | 0.0049 | -2.3175 | 44.0026 | 0.0173 |
| 213 | 35 | 0.0047 | -1.8059 | 45.1939 | 0.0099 |
| 223 | 60 | 0.0045 | -1.3128 | 46.3852 | 0.0058 |
| 243 | 180 | 0.0041 | -0.3001 | 48.7678 | 0.0019 |
| 253 | 290 | 0.0040 | 0.1365 | 49.9591 | 0.0012 |
| 263 | 430 | 0.0038 | 0.4916 | 51.1504 | 0.0008 |
| 273 | 490 | 0.0037 | 0.5849 | 52.3418 | 0.0007 |
| 283 | 700 | 0.0035 | 0.9056 | 53.5331 | 0.0005 |

**Table S6 – Eyring plot for 2a (10% $(CD_3)_2SO$ in $CD_2Cl_2$).**

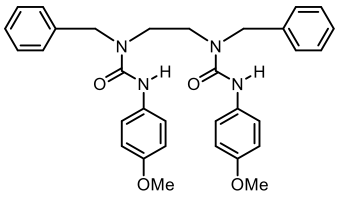


$\Delta G^{\ddagger}_{298\ K}$ = 52.3 kJ mol$^{-1}$

(10% $(CD_3)_2SO$ in $CD_2Cl_2$)

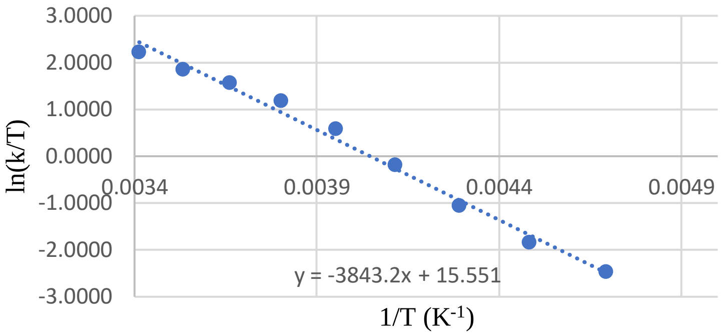


| Temperature (K) | Rate Constant (s$^{-1}$) | 1/T (K$^{-1}$) | ln(k/T) | ΔG$^{‡}$ (kJ mol$^{-1}$) | $t_{½}$ (s) |
|---|---|---|---|---|---|
| 213 | 18 | 0.0047 | -2.4709 | 46.4921 | 0.0193 |
| 223 | 35 | 0.0045 | -1.8518 | 47.1746 | 0.0099 |
| 233 | 80 | 0.0043 | -1.0690 | 47.8572 | 0.0043 |
| 243 | 200 | 0.0041 | -0.1947 | 48.5397 | 0.0017 |
| 253 | 450 | 0.0040 | 0.5759 | 49.2222 | 0.0008 |
| 263 | 850 | 0.0038 | 1.1731 | 49.9048 | 0.0004 |
| 273 | 1300 | 0.0037 | 1.5606 | 50.5873 | 0.0003 |
| 283 | 1800 | 0.0035 | 1.8501 | 51.2698 | 0.0002 |

## Table S7 – Eyring plot for 2b.

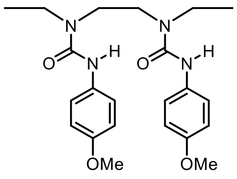


$\Delta G^{\ddagger}_{298\,K}$ = 51.5 kJ mol$^{-1}$

($CD_2Cl_2$)

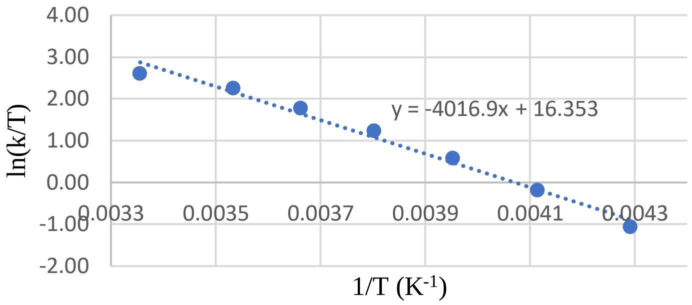


| Temperature (K) | Rate Constant (s$^{-1}$) | 1/T (K$^{-1}$) | ln(k/T) | ΔG$^{\ddagger}$ (kJ mol$^{-1}$) | $t_{½}$ (s) |
|---|---|---|---|---|---|
| 193 | 55 | 0.0052 | -1.2554 | 45.5790 | 0.0063 |
| 233 | 80 | 0.0043 | -1.0690 | 47.8309 | 0.0043 |
| 243 | 200 | 0.0041 | -0.1947 | 48.3939 | 0.0017 |
| 253 | 450 | 0.0040 | 0.5759 | 48.9569 | 0.0008 |
| 263 | 900 | 0.0038 | 1.2302 | 49.5198 | 0.0004 |
| 273 | 1600 | 0.0037 | 1.7683 | 50.0828 | 0.0002 |
| 283 | 2700 | 0.0035 | 2.2556 | 50.6458 | 0.0001 |
| 298 | 4000 | 0.0034 | 2.5970 | 51.4902 | 0.0001 |

## Table S8 – Eyring plot for 3a.

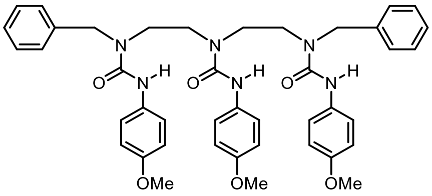


$\Delta G^{\ddagger}_{298\,K}$ = 59.0 kJ mol$^{-1}$

($CD_2Cl_2$)

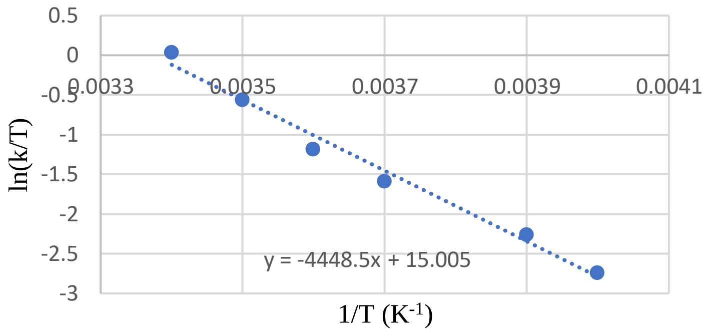


| Temperature (K) | Rate Constant (s$^{-1}$) | 1/T (K$^{-1}$) | ln(k/T) | ΔG$^{\ddagger}$ (kJ mol$^{-1}$) | $t_{½}$ (s) |
|---|---|---|---|---|---|
| 248 | 16 | 0.0040 | -2.7408 | 54.8299 | 0.0217 |
| 258 | 27 | 0.0039 | -2.2571 | 55.6705 | 0.0128 |
| 268 | 55 | 0.0037 | -1.5837 | 56.5110 | 0.0063 |
| 278 | 85 | 0.0036 | -1.1850 | 57.3515 | 0.0041 |
| 288 | 165 | 0.0035 | -0.5570 | 58.1920 | 0.0021 |
| 298 | 310 | 0.0034 | 0.0395 | 59.0325 | 0.0011 |

**Table S9 – Eyring plot for 3a (40% $CD_3OH$ in $CD_2Cl_2$).**

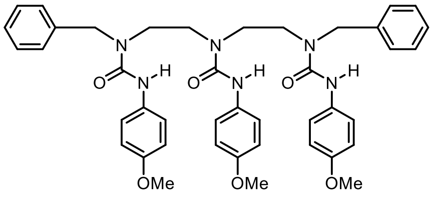

$\Delta G^{\ddagger}_{298\,K}$ = 58.6 kJ mol$^{-1}$

(40% $CD_3OD$ in $CD_2Cl_2$)

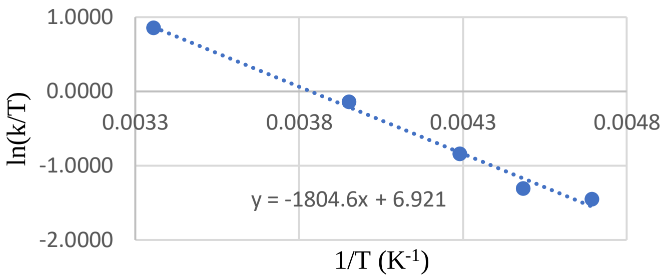


| Temperature (K) | Rate Constant ($s^{-1}$) | 1/T ($K^{-1}$) | ln(k/T) | ΔG‡ (kJ mol$^{-1}$) | $t_{½}$ (s) |
|---|---|---|---|---|---|
| 213 | 50 | 0.0047 | -1.4493 | 52.4842 | 0.0069 |
| 223 | 60 | 0.0045 | -1.3128 | 53.2037 | 0.0058 |
| 233 | 100 | 0.0043 | -0.8459 | 53.9233 | 0.0035 |
| 253 | 220 | 0.0040 | -0.1398 | 55.3623 | 0.0016 |
| 298 | 700 | 0.0034 | 0.8540 | 58.6002 | 0.0005 |

**Table S10 – Eyring plot for 3a (10% $(CD_3)_2SO$ in $CD_2Cl_2$).**

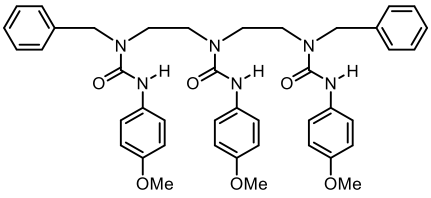

$\Delta G^{\ddagger}_{298\,K}$ = 54.9 kJ mol$^{-1}$

(10% $(CD_3)_2SO$ in $CD_2Cl_2$)

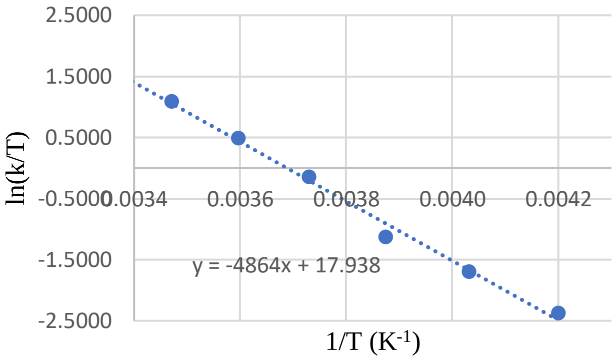


| Temperature (K) | Rate Constant ($s^{-1}$) | 1/T ($K^{-1}$) | ln(k/T) | ΔG‡ (kJ mol$^{-1}$) | $t_{½}$ (s) |
|---|---|---|---|---|---|
| 238 | 22 | 0.0042 | -2.3812 | 51.9623 | 0.0158 |
| 248 | 45 | 0.0040 | -1.7068 | 52.4464 | 0.0077 |
| 258 | 83 | 0.0039 | -1.1341 | 52.9305 | 0.0042 |
| 268 | 230 | 0.0037 | -0.1529 | 53.4145 | 0.0015 |
| 278 | 450 | 0.0036 | 0.4816 | 53.8986 | 0.0008 |
| 288 | 850 | 0.0035 | 1.0823 | 54.3827 | 0.0004 |
| 298 | 1500 | 0.0034 | 1.6161 | 54.8667 | 0.0002 |

**Table S11 – Eyring plot for 3b.**

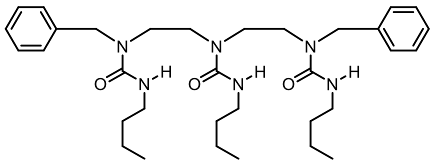

$\Delta G^{\ddagger}_{298\,K}$ = 57.9 kJ mol$^{-1}$

($CD_2Cl_2$)

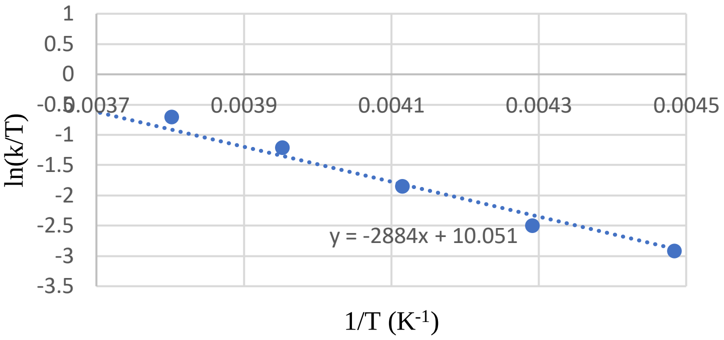


| Temperature (K) | Rate Constant ($s^{-1}$) | 1/T ($K^{-1}$) | ln(k/T) | ΔG‡ (kJ mol$^{-1}$) | $t_{½}$ (s) |
|---|---|---|---|---|---|
| 223 | 12 | 0.0045 | -2.9223 | 49.3977 | 0.0289 |
| 233 | 19 | 0.0043 | -2.5066 | 50.5376 | 0.0182 |
| 243 | 38 | 0.0041 | -1.8555 | 51.6775 | 0.0091 |
| 253 | 75 | 0.0040 | -1.2159 | 52.8173 | 0.0046 |
| 263 | 130 | 0.0038 | -0.7046 | 53.9572 | 0.0027 |
| 273 | 198 | 0.0037 | -0.3212 | 55.0970 | 0.0018 |
| 283 | 260 | 0.0035 | -0.0848 | 56.2369 | 0.0013 |
| 298 | 310 | 0.0034 | 0.0395 | 57.9467 | 0.0011 |

**Table S12 – Eyring plot for 4a.**

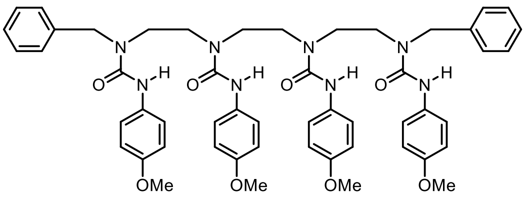

$\Delta G^{\ddagger}_{298\,K}$ = 60.9 kJ mol$^{-1}$

($CD_2Cl_2$)

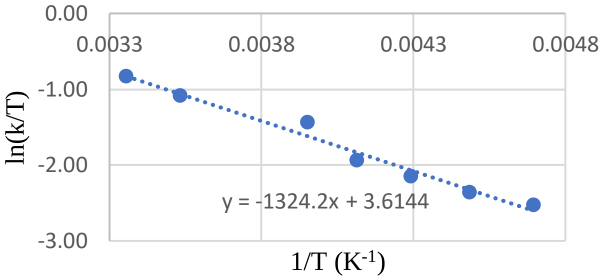


| Temperature (K) | Rate Constant ($s^{-1}$) | 1/T ($K^{-1}$) | ln(k/T) | ΔG‡ (kJ mol$^{-1}$) | $t_{½}$ (s) |
|---|---|---|---|---|---|
| 213 | 17 | 0.0047 | -2.5281 | 46.6874 | 0.0204 |
| 223 | 21 | 0.0045 | -2.3626 | 48.3624 | 0.0165 |
| 233 | 27 | 0.0043 | -2.1552 | 50.0374 | 0.0128 |
| 243 | 35 | 0.0041 | -1.9377 | 51.7124 | 0.0099 |
| 253 | 60 | 0.0040 | -1.4390 | 53.3874 | 0.0058 |
| 283 | 95 | 0.0035 | -1.0916 | 58.4124 | 0.0036 |
| 298 | 130 | 0.0034 | -0.8296 | 60.9249 | 0.0027 |

## Table S13 – Eyring plot for 4a.

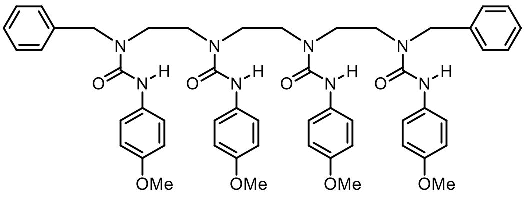


$\Delta G^{\ddagger}_{298\,K}$ = 58.0 kJ mol$^{-1}$

(40% $CD_3OH$ in $CD_2Cl_2$)

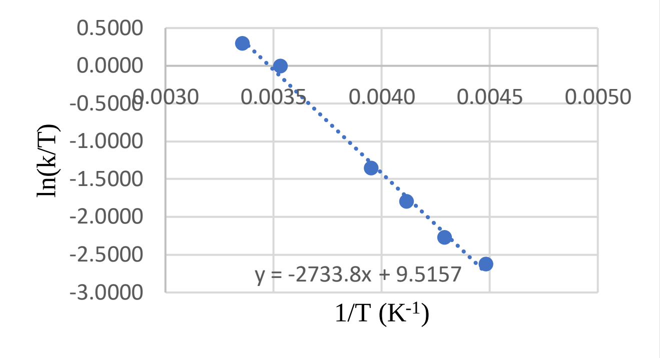


| Temperature (K) | Rate Constant (s$^{-1}$) | 1/T (K$^{-1}$) | ln(k/T) | ΔG$^{‡}$ (kJ mol$^{-1}$) | $t_{½}$ (s) |
|---|---|---|---|---|---|
| 223 | 16 | 0.0045 | -2.6346 | 49.1408 | 0.0217 |
| 233 | 24 | 0.0043 | -2.2730 | 50.3251 | 0.0144 |
| 243 | 40 | 0.0041 | -1.8042 | 51.5094 | 0.0087 |
| 253 | 65 | 0.0040 | -1.3590 | 52.6938 | 0.0053 |
| 283 | 280 | 0.0035 | -0.0107 | 56.2468 | 0.0012 |
| 298 | 400 | 0.0034 | 0.2944 | 58.0233 | 0.0009 |

## Table S14 – Eyring plot for 4a (10% $(CD_3)_2SO$ in $CD_2Cl_2$).

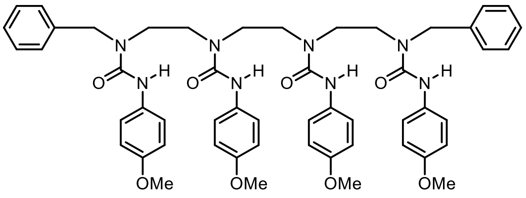


$\Delta G^{\ddagger}_{298\,K}$ = 53.7 kJ mol$^{-1}$

(10% $(CD_3)_2SO$ in $CD_2Cl_2$)

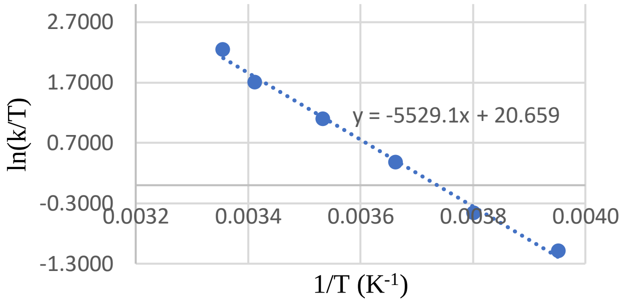


| Temperature (K) | Rate Constant (s$^{-1}$) | 1/T (K$^{-1}$) | ln(k/T) | ΔG$^{‡}$ (kJ mol$^{-1}$) | $t_{½}$ (s) |
|---|---|---|---|---|---|
| 253 | 85 | 0.0040 | -1.0907 | 52.4946 | 0.0041 |
| 263 | 165 | 0.0038 | -0.4662 | 52.7524 | 0.0021 |
| 273 | 400 | 0.0037 | 0.3820 | 53.0102 | 0.0009 |
| 283 | 850 | 0.0035 | 1.0998 | 53.2681 | 0.0004 |
| 293 | 1600 | 0.0034 | 1.6976 | 53.5259 | 0.0002 |
| 298 | 2800 | 0.0034 | 2.2403 | 53.6548 | 0.0001 |

**Table S15 – Eyring plot for 3c (minor conformer to major conformer).**

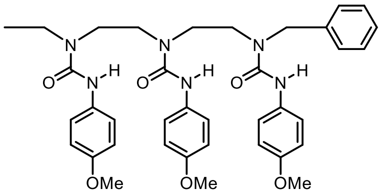


$\Delta G^{\ddagger}_{298\,K}$ = 58.1 kJ mol$^{-1}$
(minor conformer to major conformer)

$\Delta G^{\ddagger}_{298\,K}$ = 59.2 kJ mol$^{-1}$

(major conformer to minor conformer)

($CD_2Cl_2$)

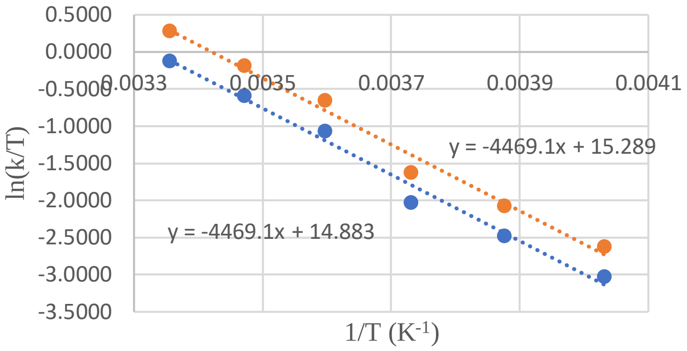


| Temperature (K) | Rate Constant ($s^{-1}$) | 1/T ($K^{-1}$) | ln(k/T) | ΔG‡ (kJ mol$^{-1}$) | $t_{½}$ (s) |
|---|---|---|---|---|---|
| 248 | 18 | 0.0040 | -2.6231 | 54.6252 | 0.0193 |
| 258 | 32 | 0.0039 | -2.0748 | 55.3295 | 0.0107 |
| 268 | 52 | 0.0037 | -1.6245 | 56.0338 | 0.0066 |
| 278 | 144 | 0.0036 | -0.6578 | 56.7382 | 0.0024 |
| 288 | 240 | 0.0035 | -0.1823 | 57.4425 | 0.0014 |
| 298 | 396 | 0.0034 | 0.2843 | 58.1468 | 0.0009 |

**Table S16 – Eyring plot for 3c (major conformer to minor conformer).**

| Temperature (K) | Rate Constant ($s^{-1}$) | 1/T ($K^{-1}$) | ln(k/T) | ΔG‡ (kJ mol$^{-1}$) | $t_{½}$ (s) |
|---|---|---|---|---|---|
| 248 | 12 | 0.0040 | -3.0285 | 55.4624 | 0.0289 |
| 258 | 22 | 0.0039 | -2.4803 | 56.2005 | 0.0160 |
| 268 | 35 | 0.0037 | -2.0299 | 56.9385 | 0.0098 |
| 278 | 96 | 0.0036 | -1.0633 | 57.6766 | 0.0036 |
| 288 | 160 | 0.0035 | -0.5878 | 58.4147 | 0.0022 |
| 298 | 264 | 0.0034 | -0.1211 | 59.1527 | 0.0013 |

<u>nOe Experiments</u>

All 1D nOe studies were conducted on a Bruker AVANCE III HD 500 MHz NMR Spectrometer with 5 mm DCH $^{13}C$–$^{1}H$/D Cryo Probe (500 MHz) at 0 °C in $CD_2Cl_2$.

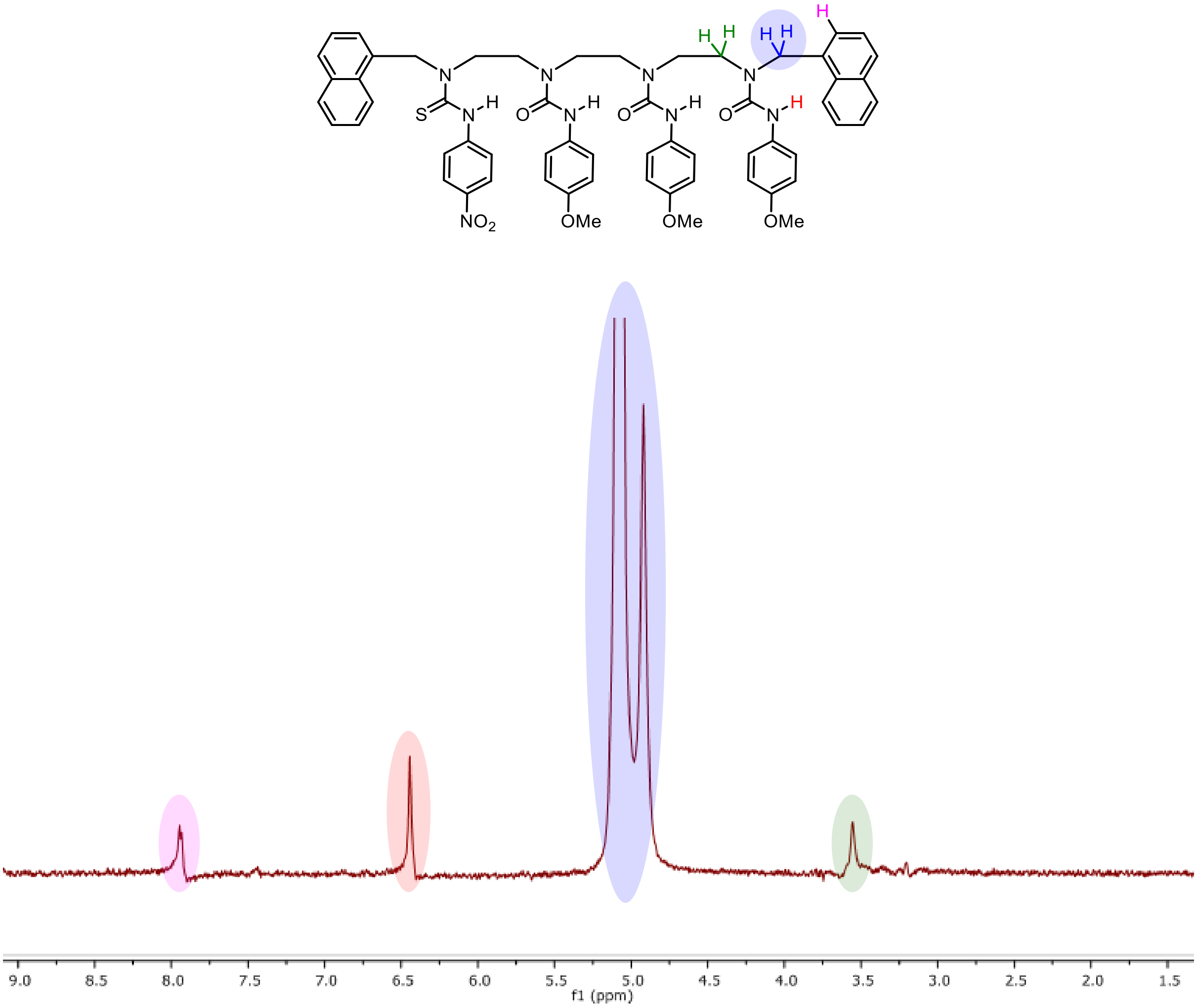


**Figure S38 – nOe studies of 5a (irradiation of blue).**

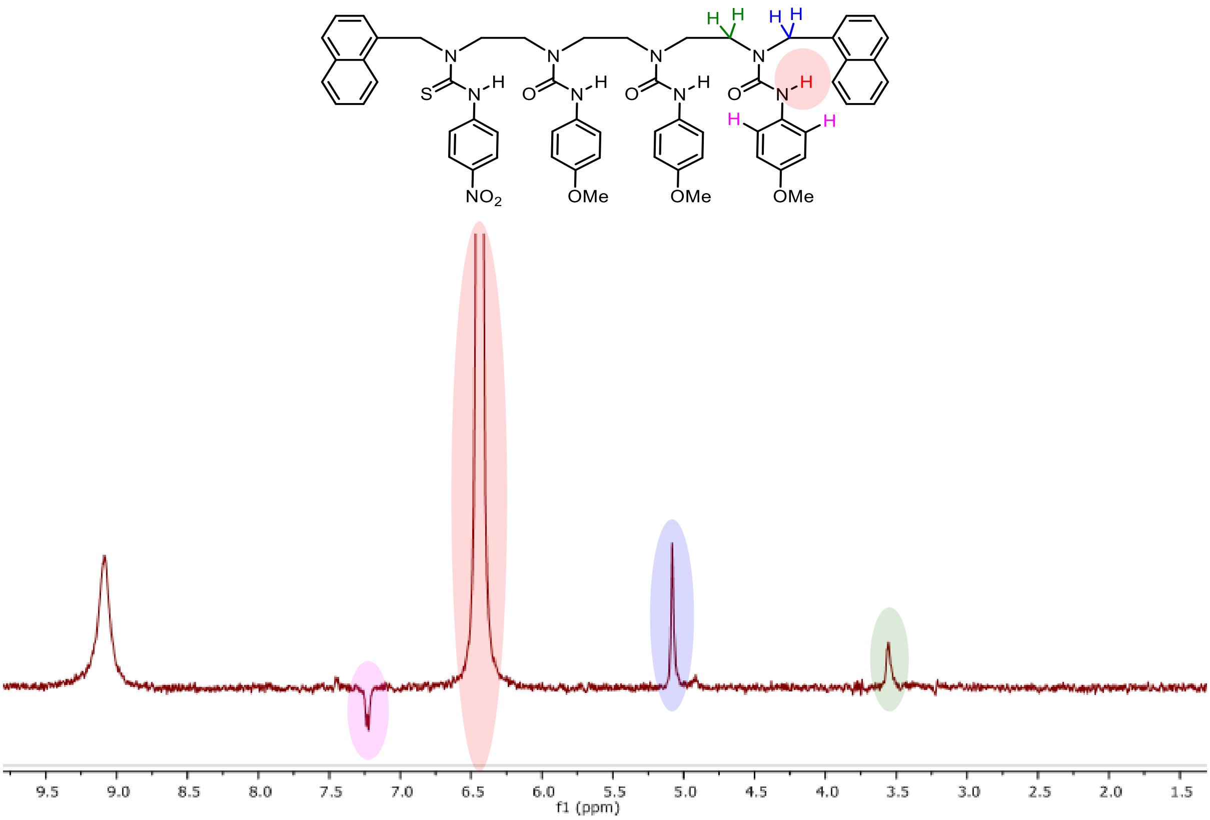


**Figure S39 – nOe studies of 5a (irradiation of red).**

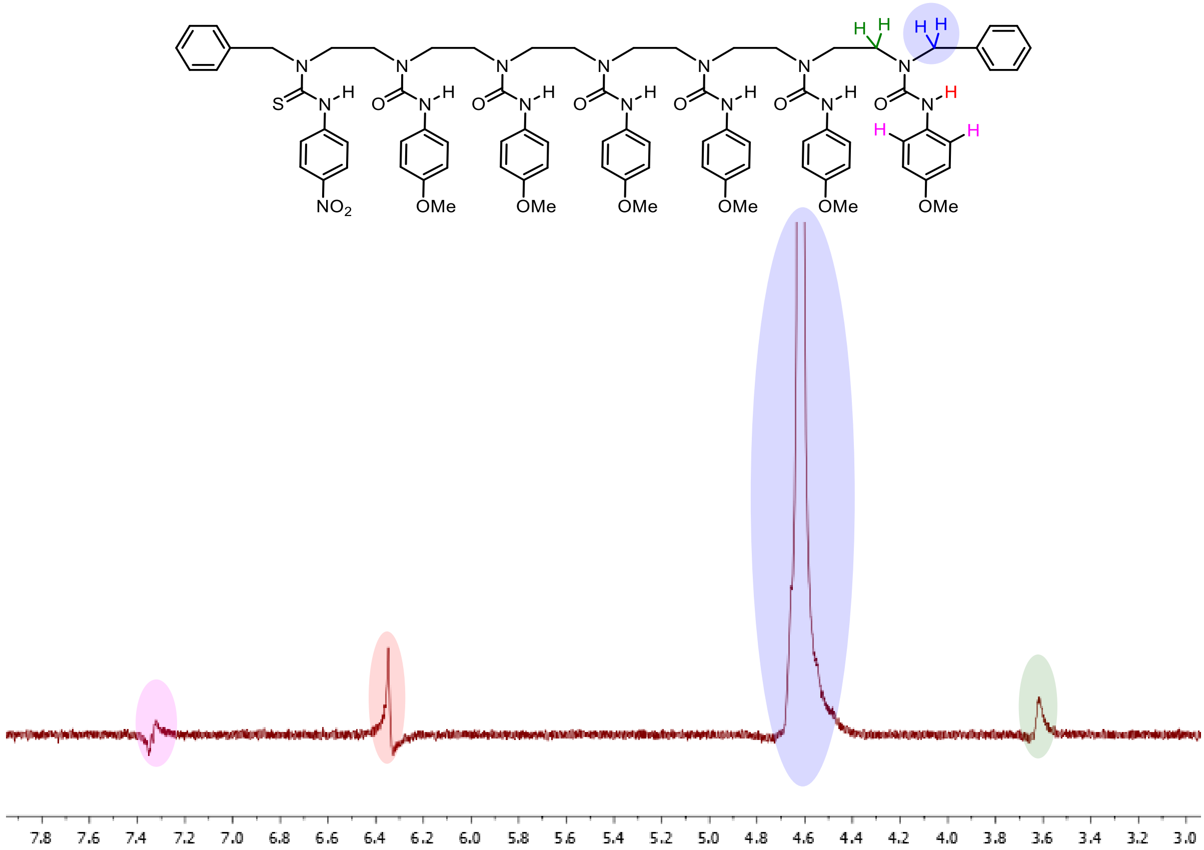


**Figure S40 – nOe studies of 5b (irradiation of blue).**

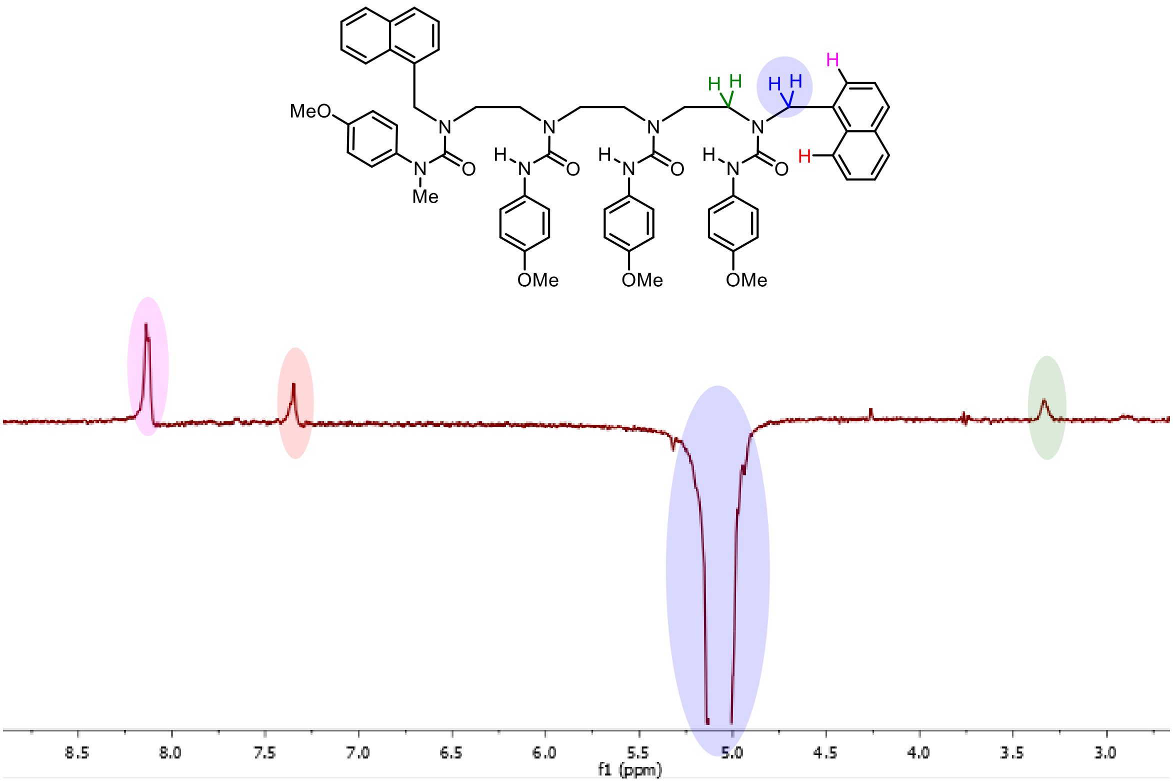


**Figure S41 – nOe studies of 6a (irradiation of blue).**

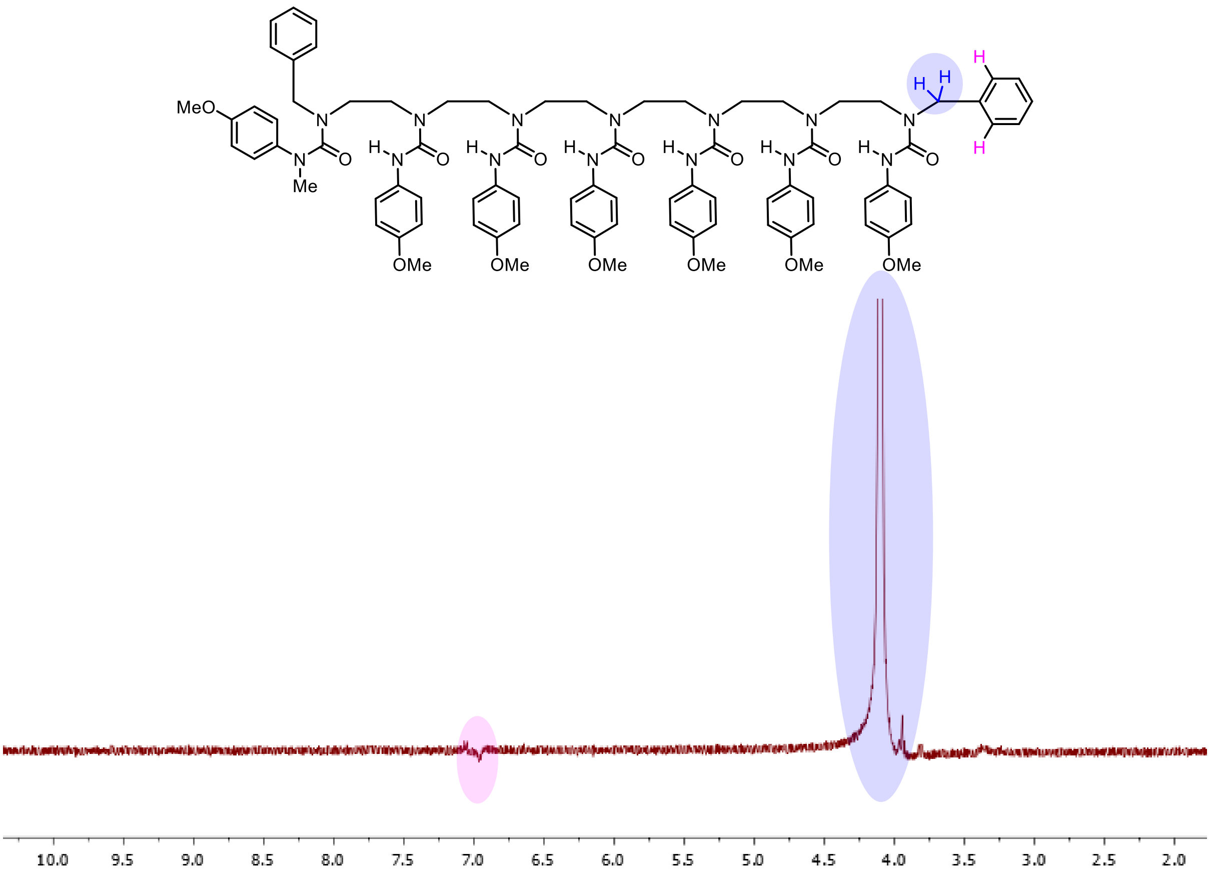


**Figure S42 – nOe studies of 6b (irradiation of blue).**

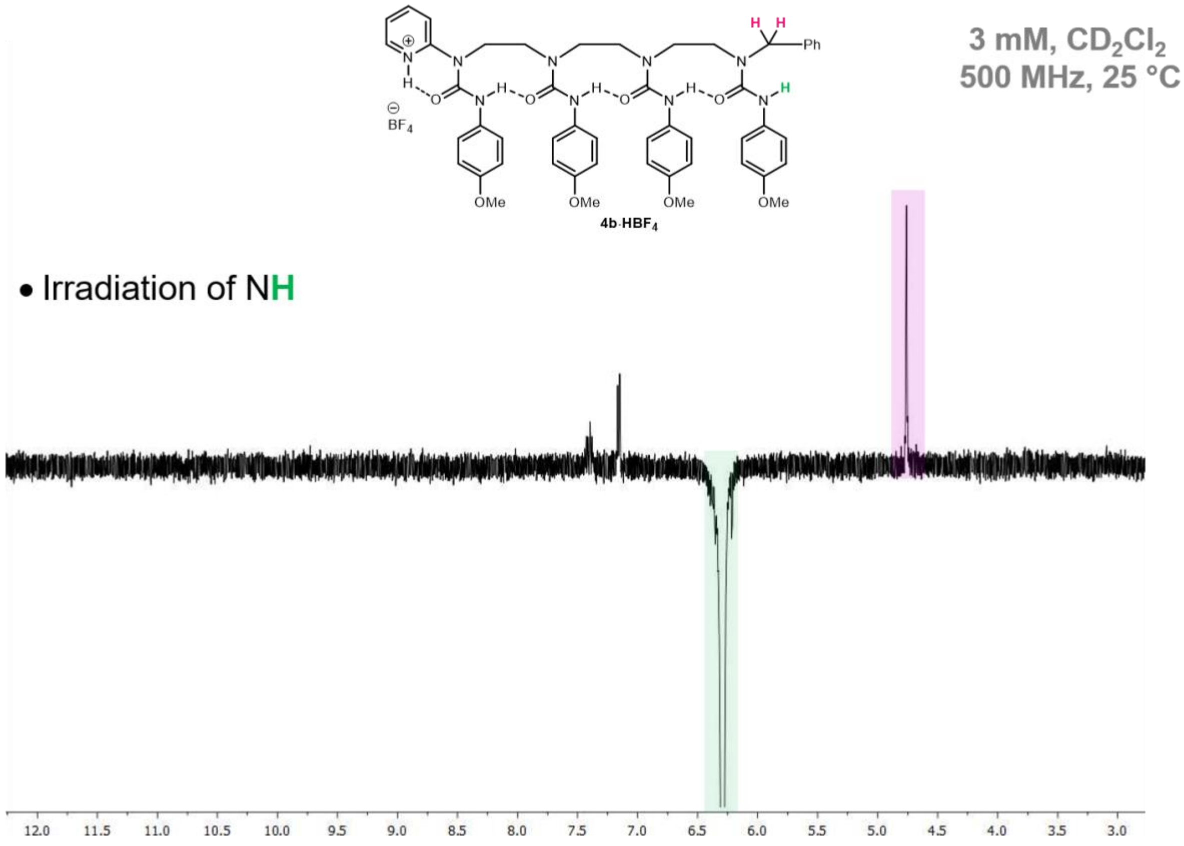


**Figure S43 – nOe studies of 4b.HBF$_4$ (irradiation of green).**

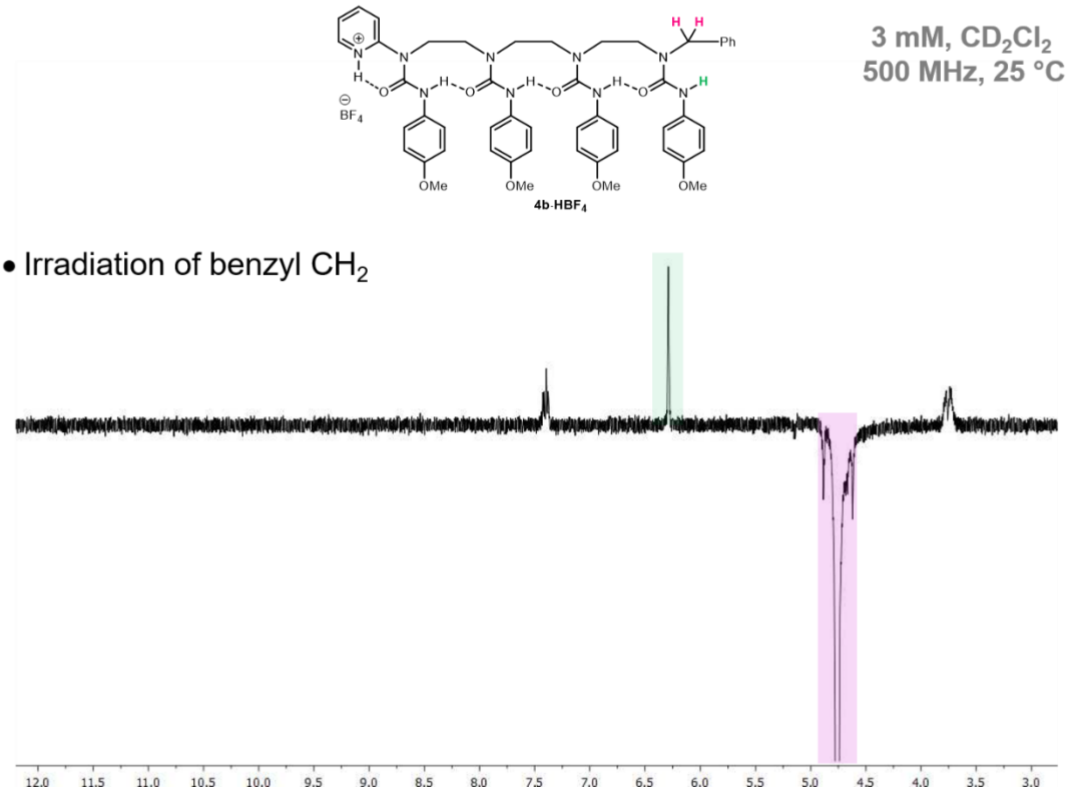


**Figure S44 – nOe studies of 4b.HBF$_4$ (irradiation of pink).**

Fluorimetry

Supplemental Experimental Procedures

**Summary of Fluorescence Wavelength Maxima**

**Table S17 – Fluorescence wavelength maxima for compounds 7-10.**

| **Compound** | **$CH_2Cl_2$ at 1 mM** | |
|---|---|---|
| | **Excitation $\lambda_{max}$ ($S_0 \rightarrow S_2$)** | **Emission $\lambda_{max}$** |
| 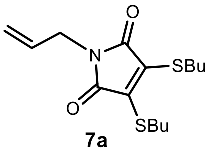 **7a** | 339 nm | 522 nm |
| 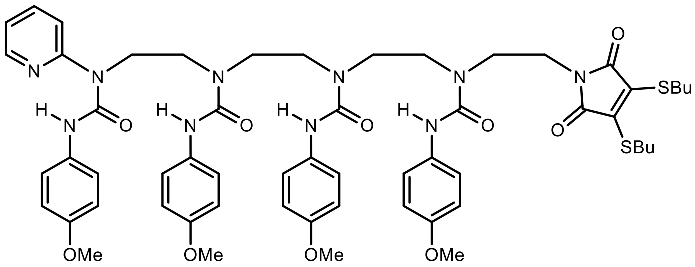 **8a** | 356 nm | 523 nm |
| 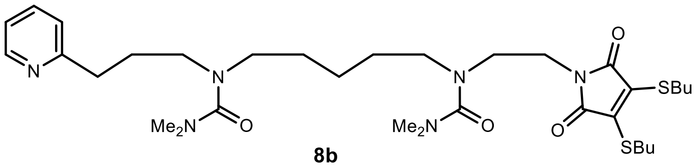 **8b** | 344 nm | 523 nm |
| 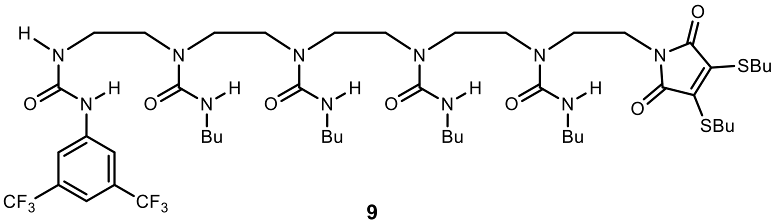 **9** | 352 nm | 530 nm |
| 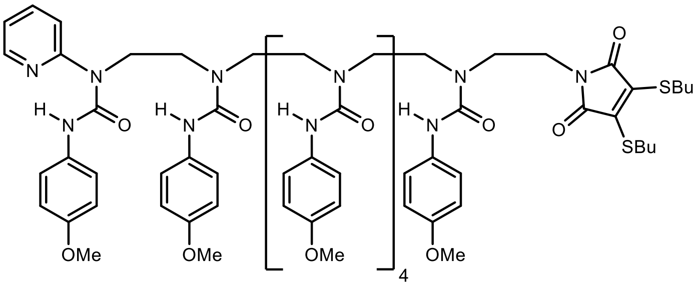 **10** | 364 nm | 525 nm |

## General Points

• All fluorescence experiments were carried out on a PerkinElmer LS45 fluorimeter at room temperature at a concentration of 1.0 mM in lab grade dichloromethane. Excitation spectra were recorded at the maximum emission wavelength for each substrate (see Table S17 above)

• All samples were prepared by directly weighing 2.0 μmol of the substrate into a glass fluorescence cuvette followed by the addition of 2.0 mL of dichloromethane

• All fluorescence spectral measurements represent the average of 2 accumulation scans

• Magnetic stirring of all samples was performed (using a micro stir bar inside the cuvette) with the stirring power set to 'high'. In all titrations, a homogenous solution was maintained throughout the entire experiments.

• The photomultiplier voltage used was dependent on the substrate: experiments with **7a**, **8b** and **9** were performed at 700 V; experiments with **8a** and **10** were performed at 900 V. The requirement for an increased photomultiplier voltage for compounds **8a** and **10** was due to their fluorescence intensities being approximately two orders of magnitude lower than the other compounds (at constant concentration and voltage) - see excitation spectra below:

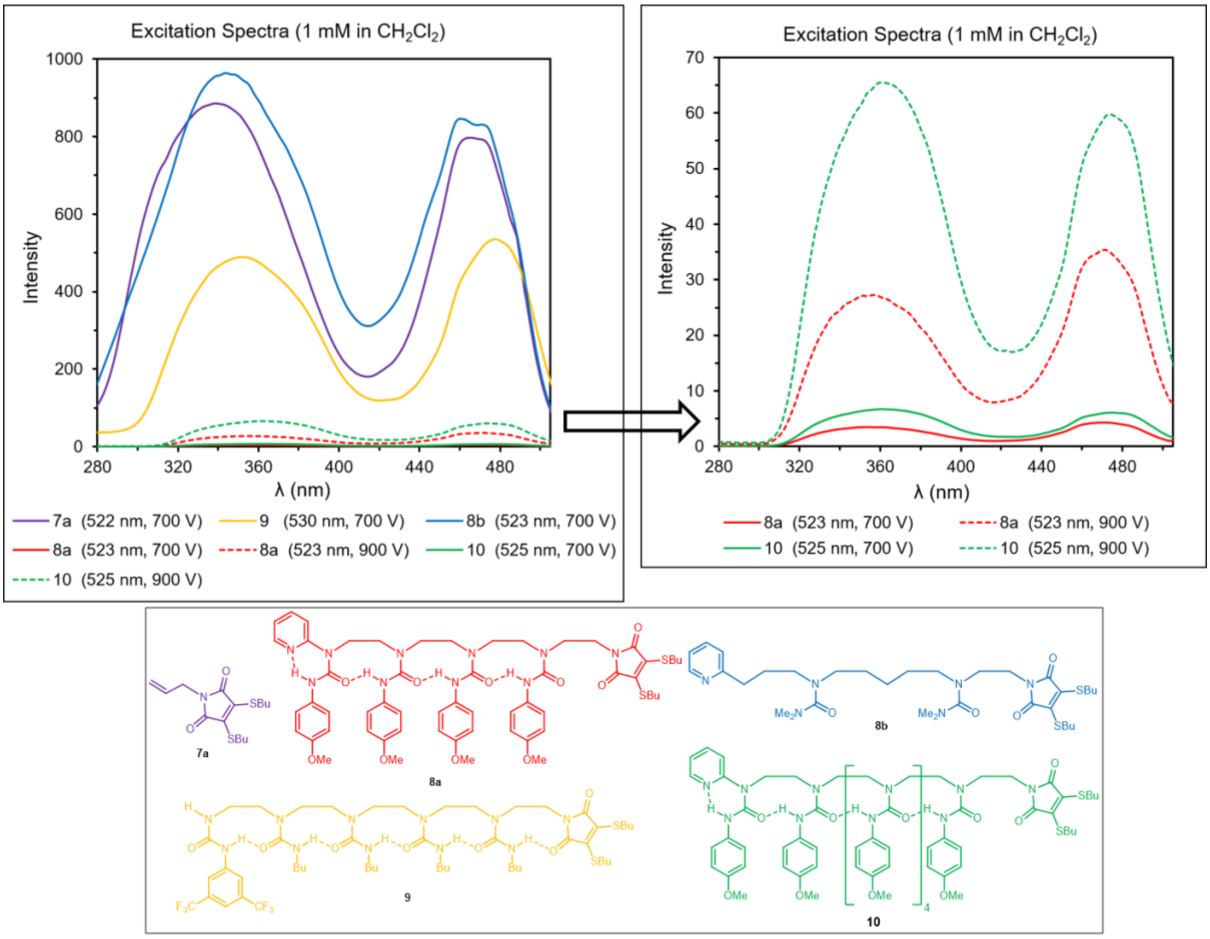


**Figure S45 – Fluorescence traces for compounds 7-10.**

**Fluorescence Titrations with Acid**

• ***General titration procedure with acid***: To a solution of the substrate (2.0 µmol) in lab grade dichloromethane (2.0 mL) in a glass cuvette at room temperature with magnetic stirring was sequentially added aliquots of a solution of $HBF_4{\cdot}Et_2O$ (0.0125 M in $CH_2Cl_2$, 20 µL each time = 0.25 µmol, 0.125 equiv) until 1.5 equiv of acid had been added (12 aliquots in total). A fluorescence spectrum was recorded after the addition of each aliquot.

• Stock solutions of $HBF_4$ for the titration experiments were prepared using the commercially available 1:1 complex with diethyl ether ($HBF_4{\cdot}Et_2O$: Aldrich 400068). Stock solutions were prepared in plastic bottles to prevent glass etching which was otherwise observed. $HBF_4{\cdot}Et_2O$ is an oil that is miscible with dichloromethane. Note, however, that over several hours/days, some droplets of $HBF_4{\cdot}Et_2O$ may be observed to precipitate out. Gentle shaking quickly restores the homogeneity. [During titrations, gentle shaking of the stock solution was always carried out before each new aliquot was added].

• Fluorescence intensities shown in acid titration spectra in the manuscript and below are not corrected for changes in concentration - the substrate concentrations decreased from 1.0 to 0.9 mM at the completion of the titrations.

• After completion of the fluorescence titration of foldamer **8a** with acid (see Fig 3b in manuscript), further evidence of protonation of the pyridine nitrogen and directionality switching was provided by $^1$H NMR spectroscopy:

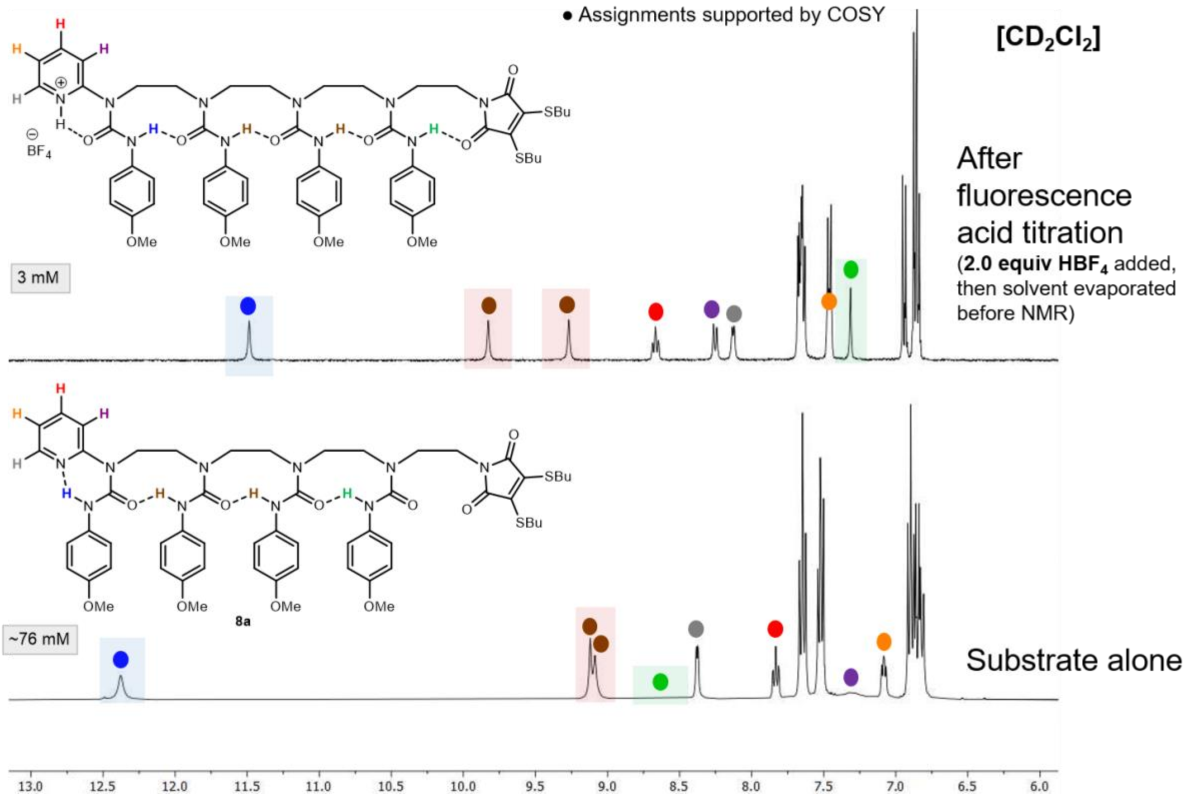


**Figure S46 – NMR studies of 8a upon treatment with $HBF_4$.**

• <u>Additional titrations with $HBF_4$</u>: The fluorescence spectra below show the effect of $HBF_4$ (0–1.5 equiv) in $CH_2Cl_2$ on the excitation spectrum (at 1 mM) of longer foldamer **10** (emission at 525 nm) and control compound **8b** (emission at 523 nm). As substoichiometric amounts of acid were sequentially added to **10**, the $S_0$–$S_2$ excitation maximum ($\lambda_{max}$) at 364 nm gradually red-shifted to 385 nm after 1 equiv. Minimal spectral changes were observed beyond 1 equiv of acid. Under the same conditions, **8b**

(without a linking chain of urea hydrogen bonds between the pyridine and the fluorophore) did not undergo any significant $\lambda_{max}$ shifting during the titration.

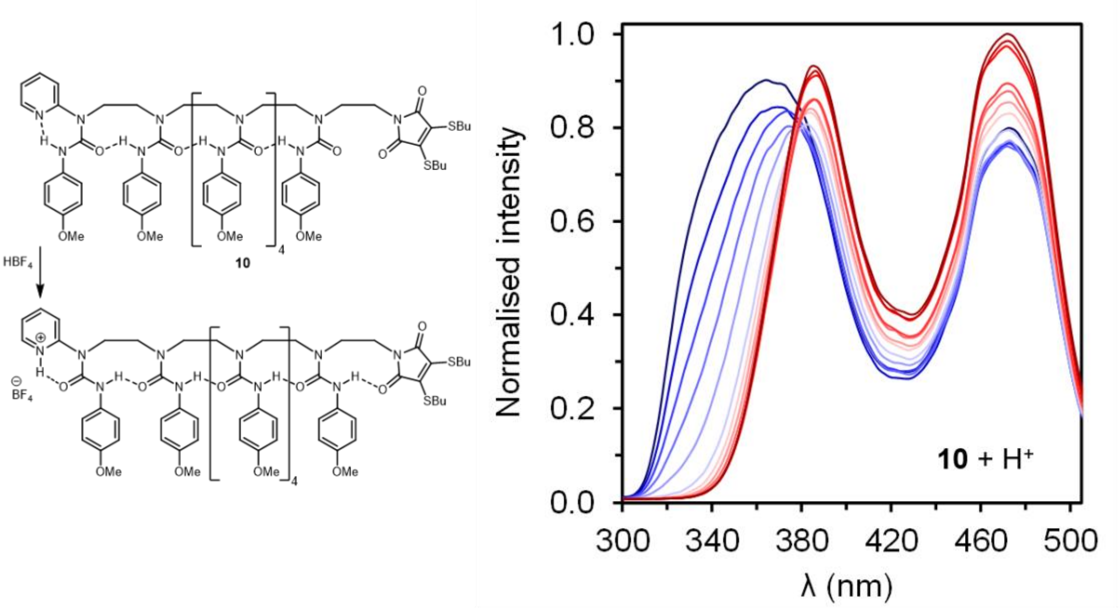


**Figure S47 – Fluorescence titrations for compound 10.**

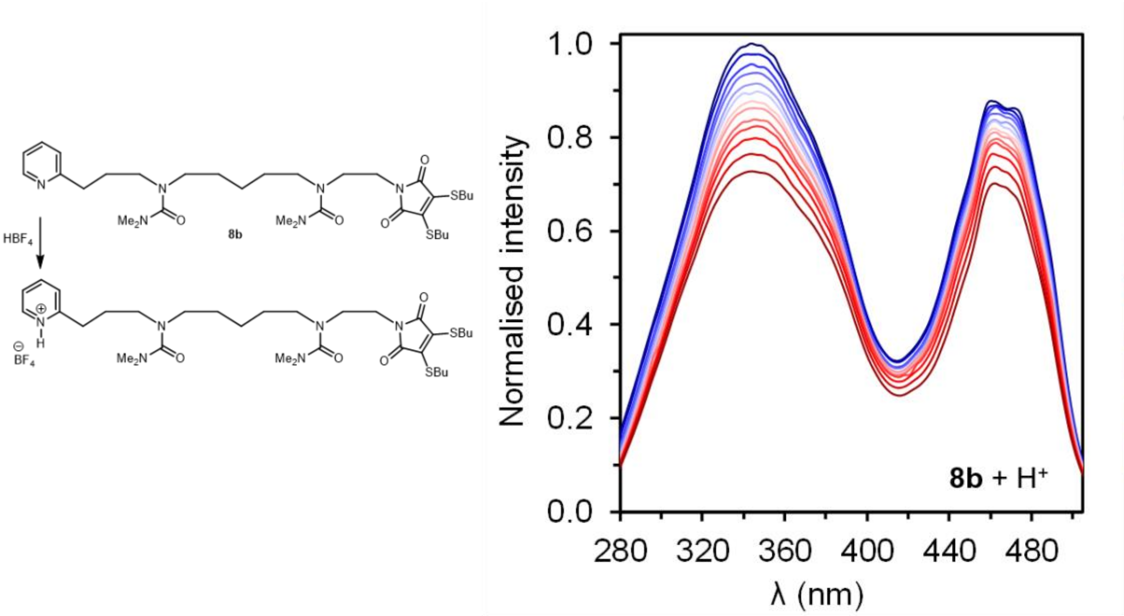


**Figure S48 – Fluorescence titrations for compound 8b.**

• After completion of the fluorescence titration of control compound **8b** with acid, evidence of pyridine N-protonation was provided by $^{1}$H NMR spectroscopy:

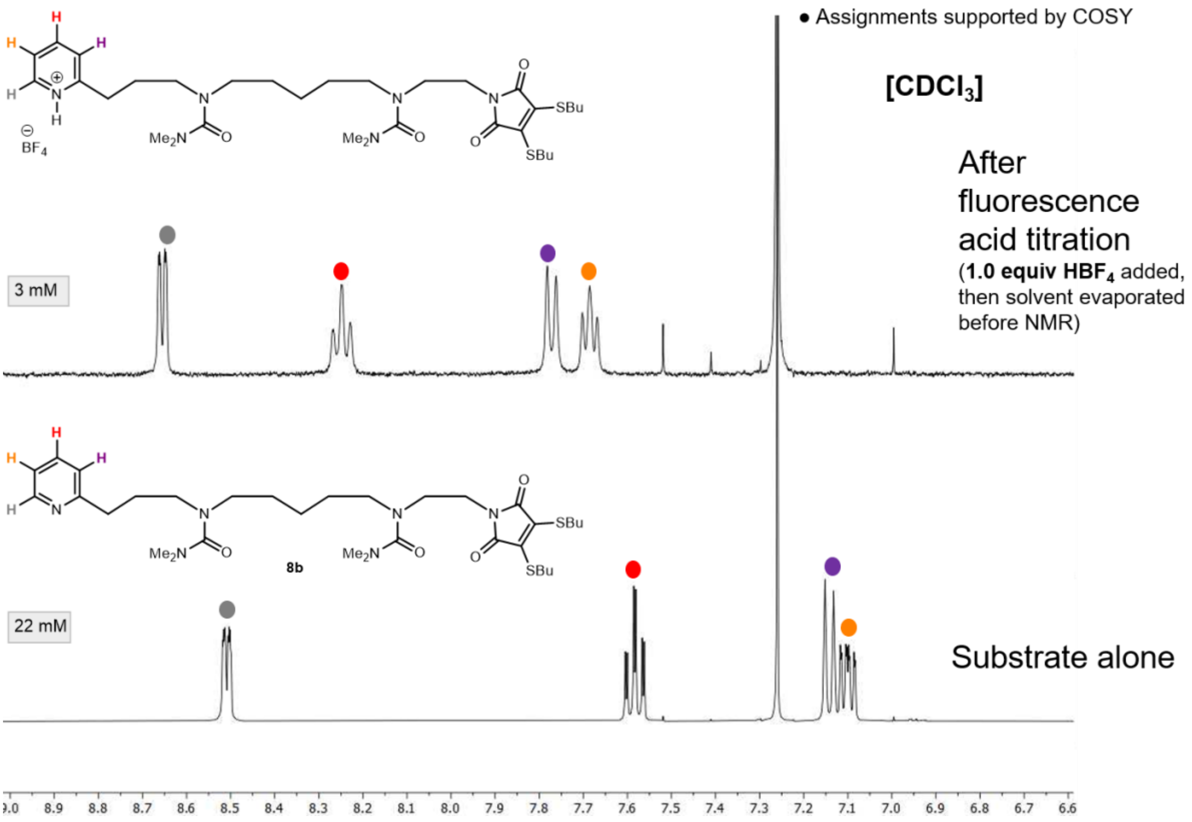


**Figure S49 – NMR studies of 8b upon treatment with $HBF_4$.**

• The plots below show the change in the fluorescence excitation wavelength maxima (relative to the initial wavelength maxima) for the $S_0$–$S_2$ transition band during the titrations with $HBF_4$. Only compounds **8a** and **10** containing a tethered pyridyl group and a linking chain of urea H-bonds to the fluorophore underwent significant $\lambda_{max}$ changes (red shifting).

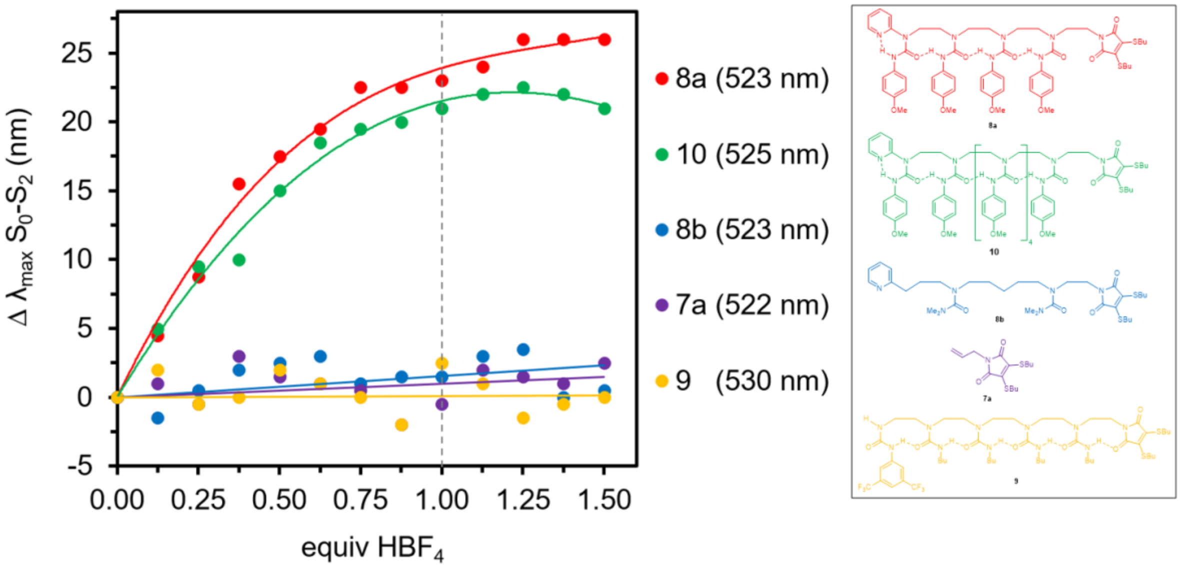


**Figure S50 – Fluorescence excitation plots upon titration of $HBF_4$ into compounds 7-10.**

**Further Evidence of the Maleimide Fluorophore as a Hydrogen-Bond Acceptor**

• Further evidence of the proposed fluorophore hydrogen-bonding interaction was provided by comparing the $^{1}$H NMR spectra of **4b**·$HBF_4$ and **8a**·$HBF_4$ (obtained at constant concentration) in which sharp signals for all NH resonances were observed; these pyridinium salts differ only by the identity of their terminal group (benzyl versus *N*-ethylmaleimide). The chemical shift of the urea NH proximal to the maleimide in **8a**·$HBF_4$ was 7.3 ppm ($CD_2Cl_2$) which is significantly more downfield than that of the NH proximal to the benzyl group in **4b**·$HBF_4$ (6.3 ppm, $CD_2Cl_2$), indicative of the maleimide acting as a hydrogen-bond acceptor; all other pairs of analogous NH protons in **4b**·$HBF_4$ and 8**a**·$HBF_4$ had essentially identical chemical shifts (≤0.04 ppm differences).

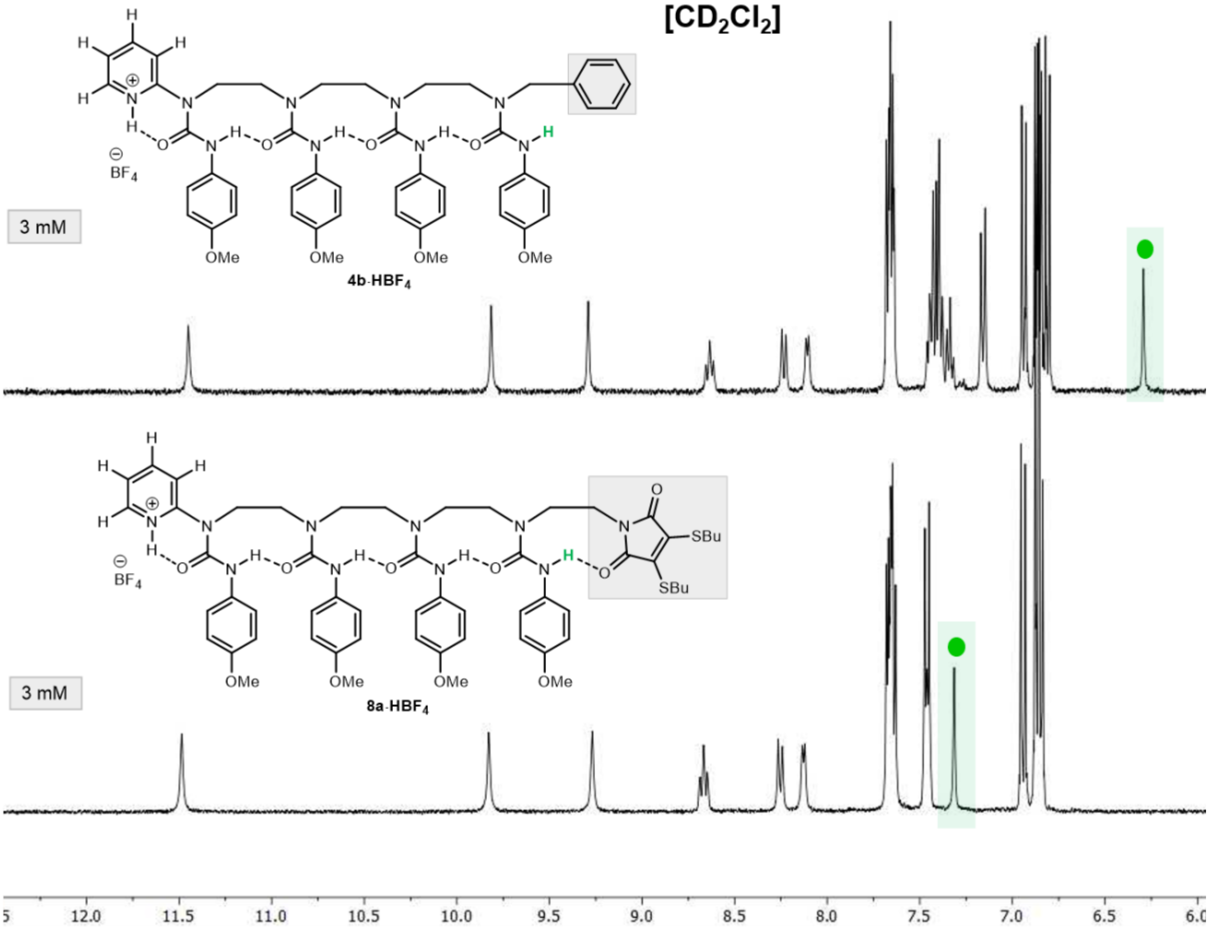


**Figure S51 – Comparison of NMR spectra of 4b and 8a upon addition of $HBF_4$.**

**Fluorescence Titrations with Acetate**

• ***General titration procedure with acetate:*** To a solution of the substrate (2.0 μmol) in lab grade dichloromethane (2.0 mL) in a glass cuvette at room temperature with magnetic stirring was sequentially added aliquots of a solution of $Bu_4N$·OAc (0.0125 M in $CH_2Cl_2$, 20 μL each time = 0.25 μmol, 0.125 equiv) until 1.5 equiv of acetate had been added (12 aliquots in total). A fluorescence spectrum was recorded after the addition of each aliquot.

• Fluorescence intensities shown in acetate titration spectra in the manuscript are not corrected for changes in concentration - the substrate concentrations decreased from 1.0 to 0.9 mM at the completion of the titrations.

• After completion of the fluorescence titration of foldamer **9** with acetate (see Fig 4f in manuscript), further evidence of acetate binding was provided by $^1$H NMR and NOE spectroscopy:

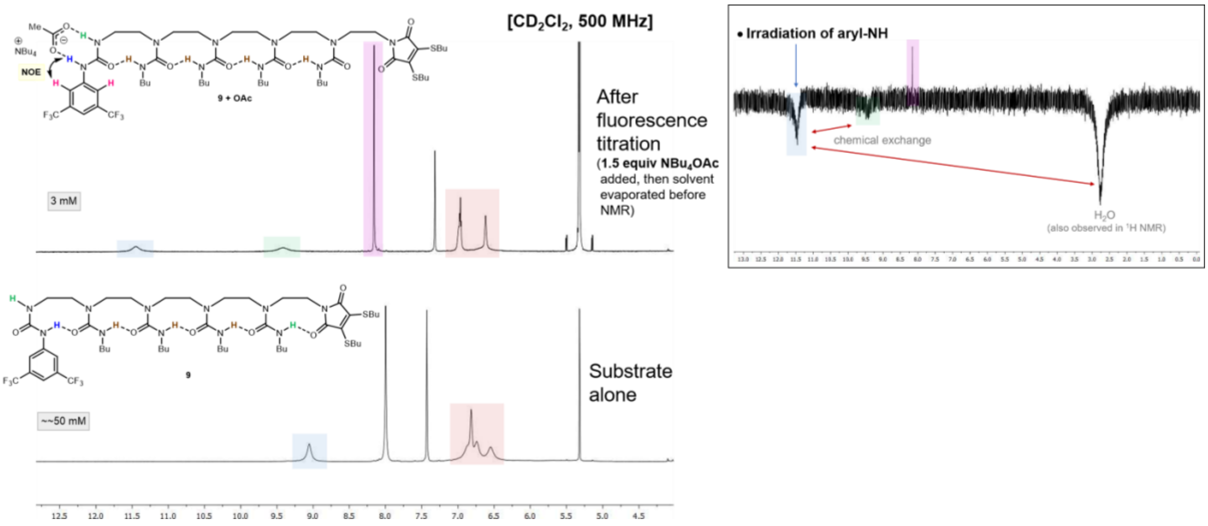


**Figure S52 – NMR studies of 9 upon treatment with $NBu_4OAc$.**

• $^1$H NMR spectroscopy was also used to confirm a lack of binding interaction/s of **8a** with acetate (see Fig 4g in manuscript), as indicated by minimal/no changes in chemical shifts:

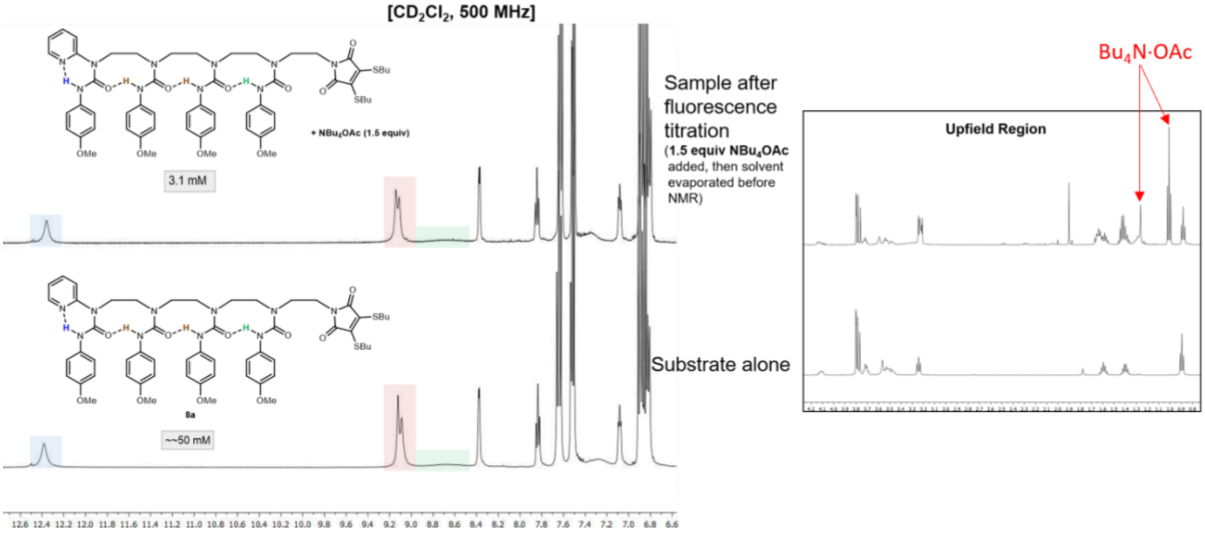


**Figure S53 – NMR studies of 8a upon treatment with $NBu_4OAc$.**

• The plots below show the change in the fluorescence excitation wavelength maxima (relative to the initial wavelength maxima) for the $S_0$–$S_1$ transition band during the titrations with $Bu_4N{\cdot}OAc$. Only compound **9** containing an anion binding site (electron-deficient disubstituted urea) and a linking chain of urea H-bonds to the fluorophore underwent a significant $\lambda_{max}$ change with 1 equiv of acetate (blue shift).

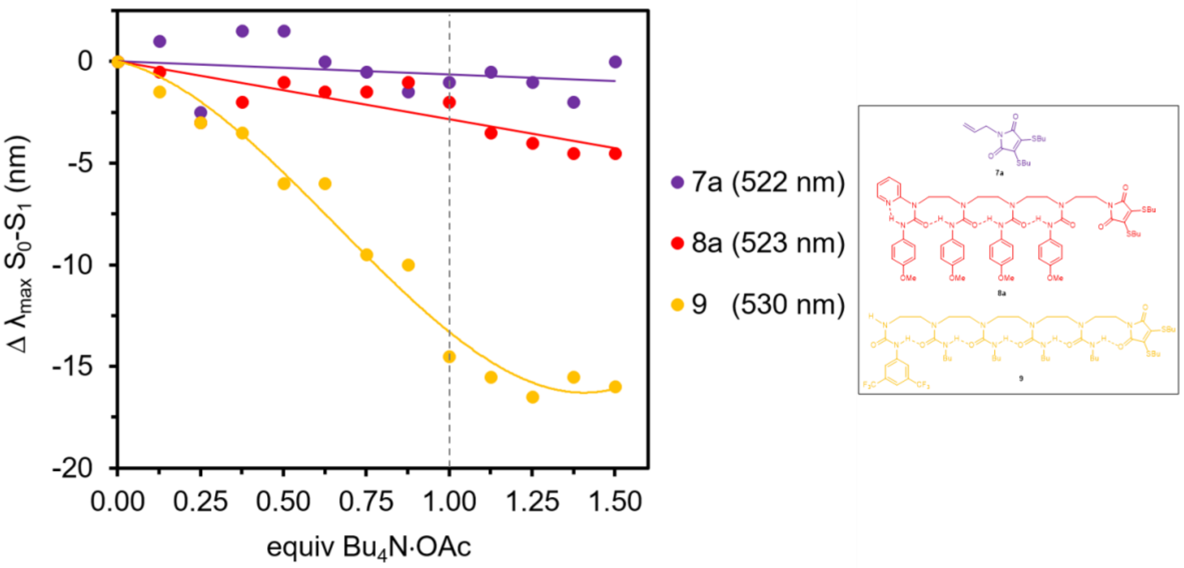


**Figure S54 – Fluorescence excitation plots upon titration of $NBu_4OAc$ into compounds 7-9.**

**Fluorescence Experiments with Acid/Base Switching**

• ***General titration procedure for acid/base switching cycles:*** To a solution of the substrate (2.0 µmol) in lab grade dichloromethane (2.0 mL) in a glass cuvette at room temperature with magnetic stirring was sequentially added 20–50 µL aliquots (specified below) of a solution of $HBF_4{\cdot}Et_2O$ (0.10 M in $CH_2Cl_2$) and a solution of $Et_3N$ (0.10 M in $CH_2Cl_2$). A fluorescence spectrum was recorded at each of the following titration points:

**Table S18 – Acid and base cycles for remote fluorescence switching of 8a.**

| Titration Point | Additive | Total equiv of $HBF_4$ added | Total equiv of $Et_3N$ added |
|---|---|---|---|
| 'Neutral' | - | 0.0 | 0.0 |
| 'Acidic' | 20 µL of $HBF_4$ solution | 1.0 | 0.0 |
| 'Basic' | 30 µL of $Et_3N$ solution | 1.0 | 1.5 |
| 'Acidic' | 40 µL of $HBF_4$ solution | 3.0 | 1.5 |
| 'Basic' | 50 µL of $Et_3N$ solution | 3.0 | 4.0 |
| 'Acidic' | 50 µL of $HBF_4$ solution | 5.5 | 4.0 |
| 'Basic' | 50 µL of $Et_3N$ solution | 5.5 | 6.5 |
| 'Acidic' | 50 µL of $HBF_4$ solution | 8.0 | 6.5 |
| 'Basic' | 50 µL of $Et_3N$ solution | 8.0 | 9.0 |

• Comments regarding the preparation of stock solutions of $HBF_4{\cdot}Et_2O$ are given previously in the 'fluorescence titrations with acid' section.

• Fluorescence intensities shown in acid/base switching titration spectra in the manuscript and below are not corrected for changes in concentration - the substrate concentrations decreased from 1.0 to 0.85 mM at the completion of the titrations.

• <u>Additional results with acid/base switching cycles</u>: Reversible pH-controlled switching between the two polarity states was also demonstrated with the shorter foldamer **8a**. Starting in the absence of additives, followed by the repeated sequential addition of acid ($HBF_4$) and base ($Et_3N$), four cycles of the fluorophore excitation response between the blue-shifted, non-hydrogen bonded 'neutral' state and the red-shifted, hydrogen bonded 'protonated' state were successfully induced.

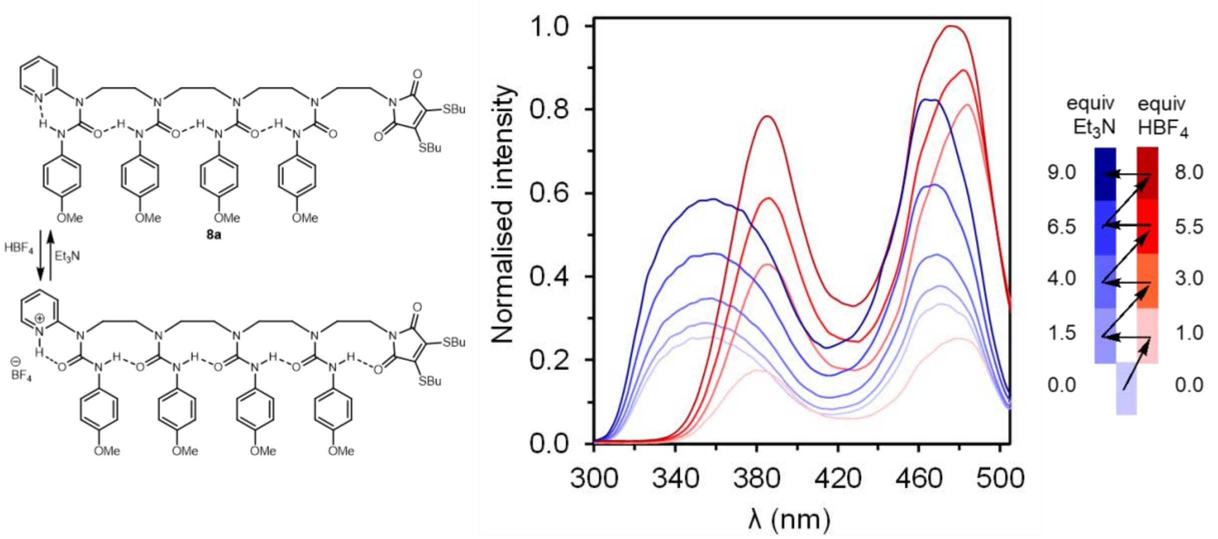


**Figure S55 – Acid/base switching by sequential addition of $HBF_4$ and $Et_3N$ to 8a.**

NMR Characterisation

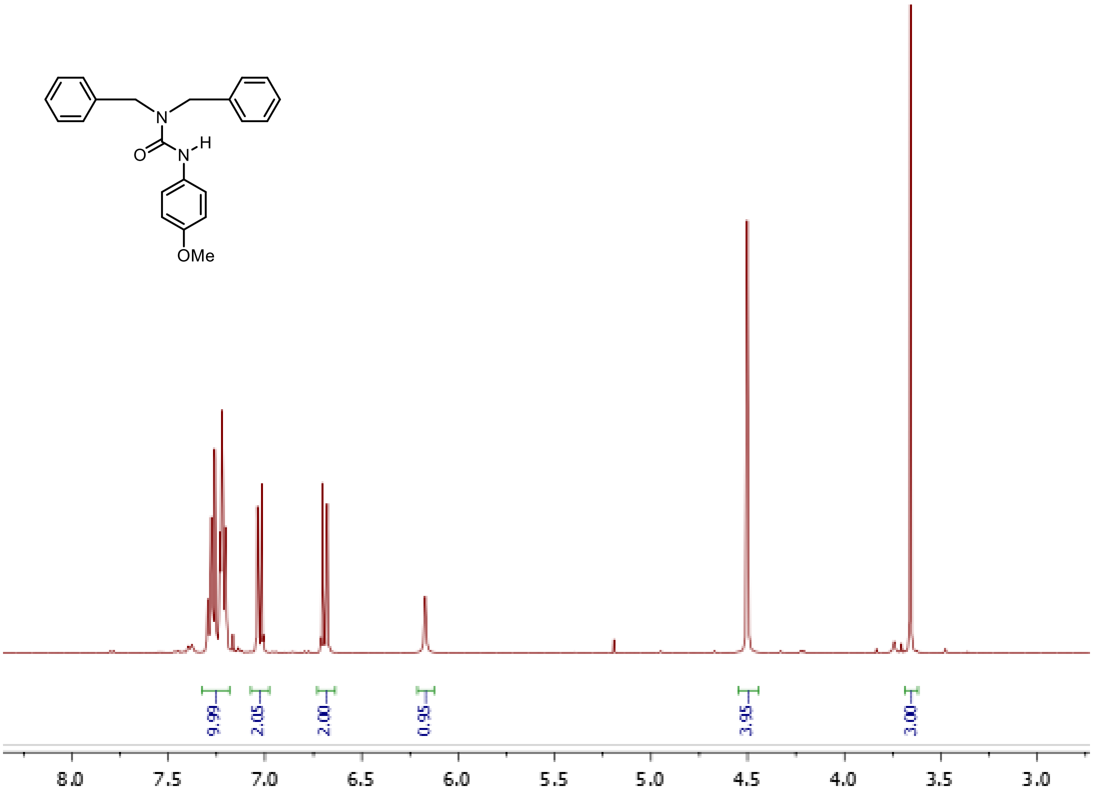


**Figure S56 – $^{1}$H NMR spectrum of 1 (400 MHz, $CDCl_3$).**

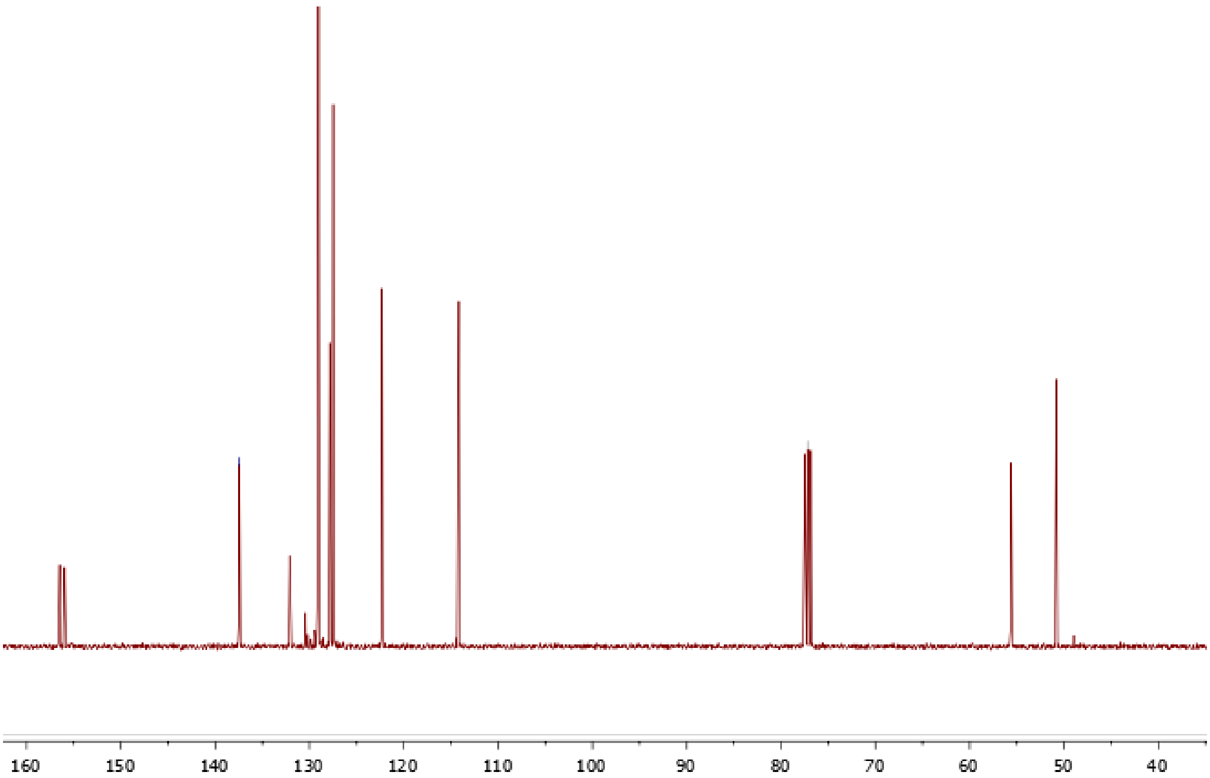


**Figure S57 – $^{13}$C NMR spectrum of 1 (101 MHz, $CDCl_3$).**

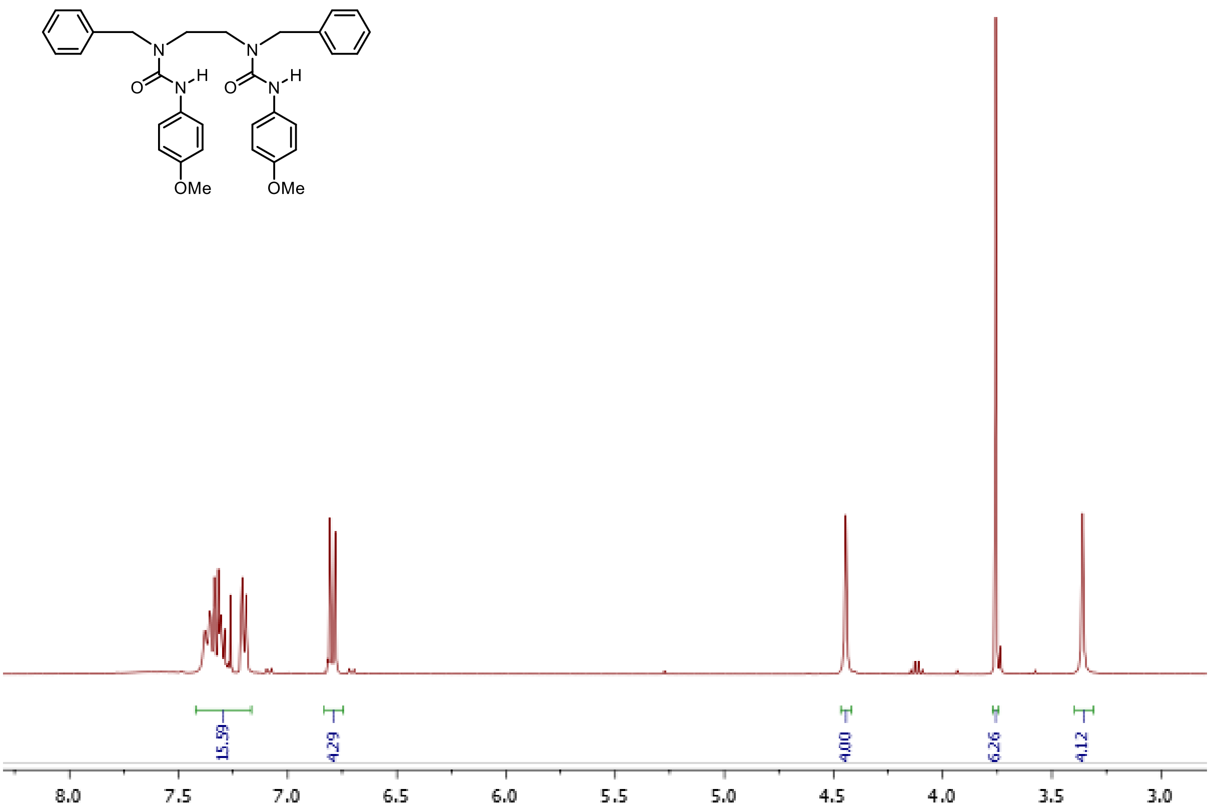


**Figure S58 – $^{1}$H NMR spectrum of 2a (400 MHz, $CDCl_3$).**

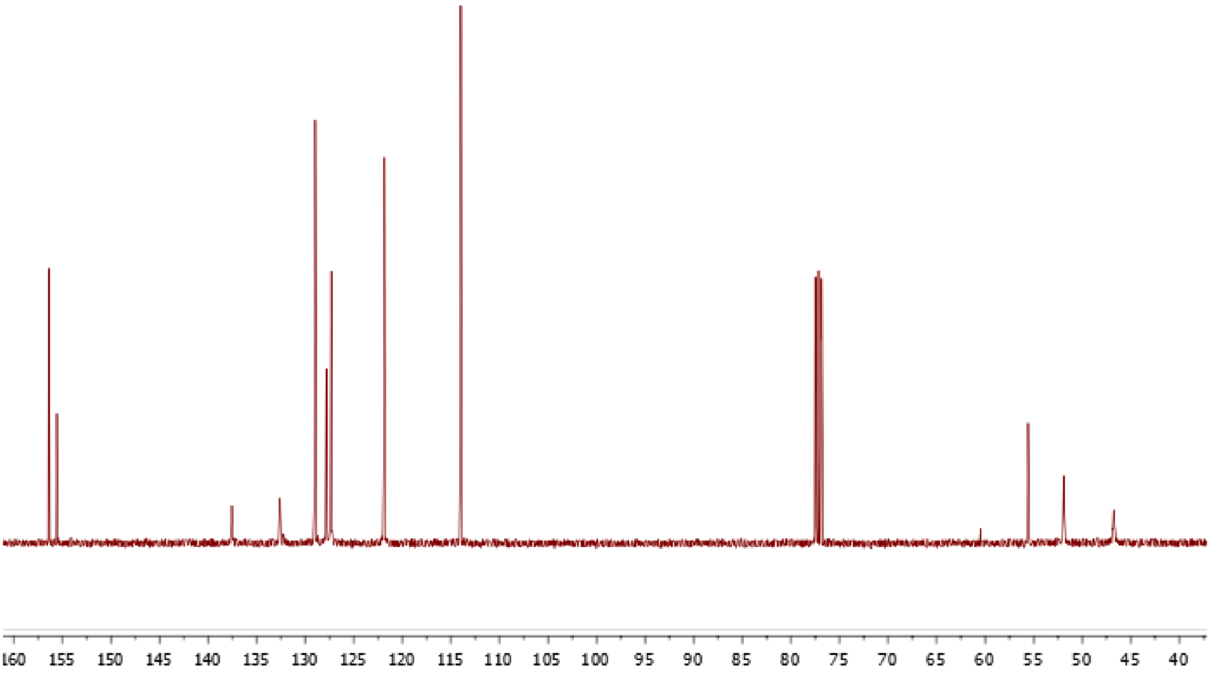


**Figure S59 – $^{13}$C NMR spectrum of 2a (101 MHz, $CDCl_3$).**

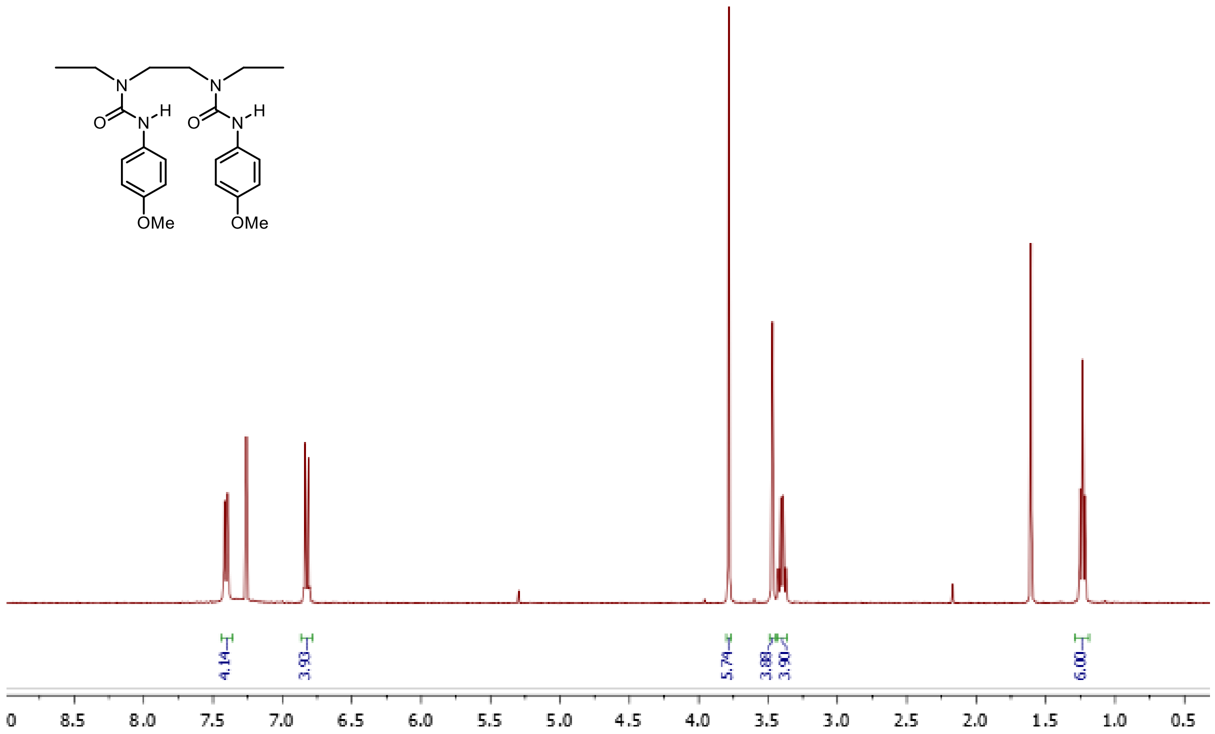


**Figure S60 – $^{1}$H NMR spectrum of 2b (400 MHz, $CDCl_3$).**

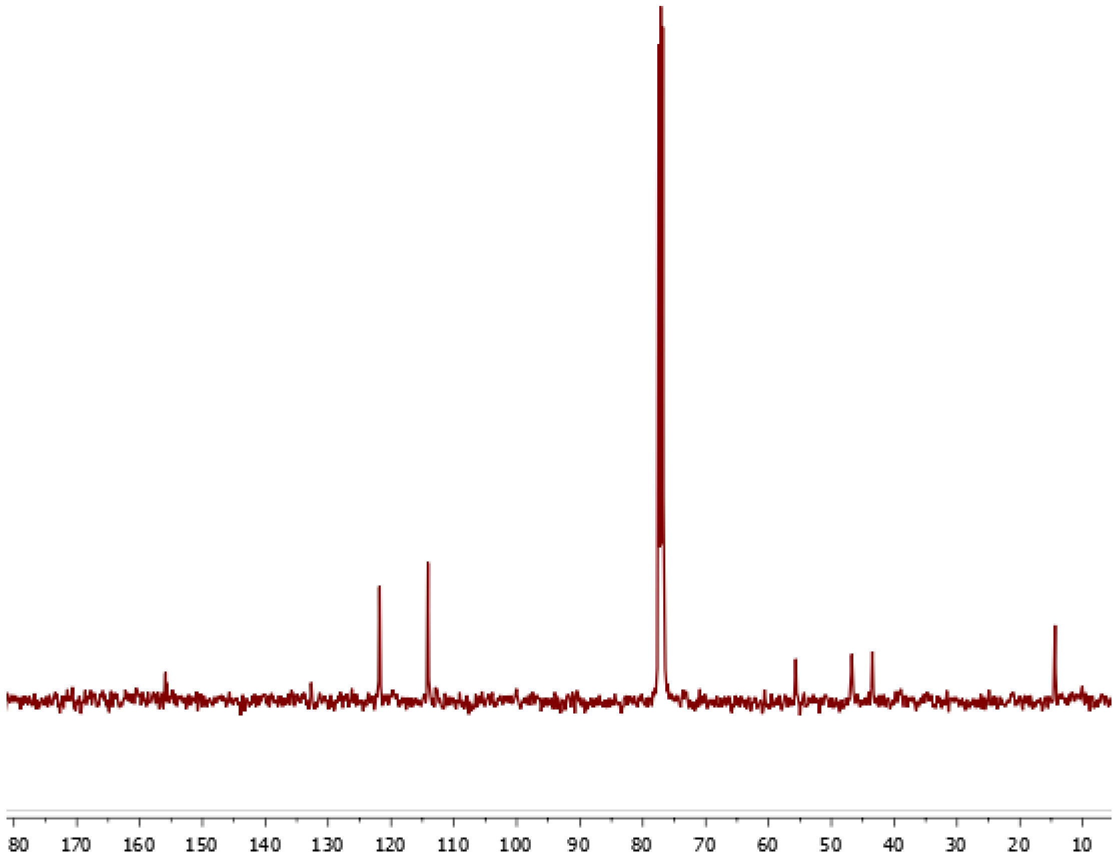


**Figure S61 – $^{13}$C NMR spectrum of 2b (101 MHz, $CDCl_3$).**

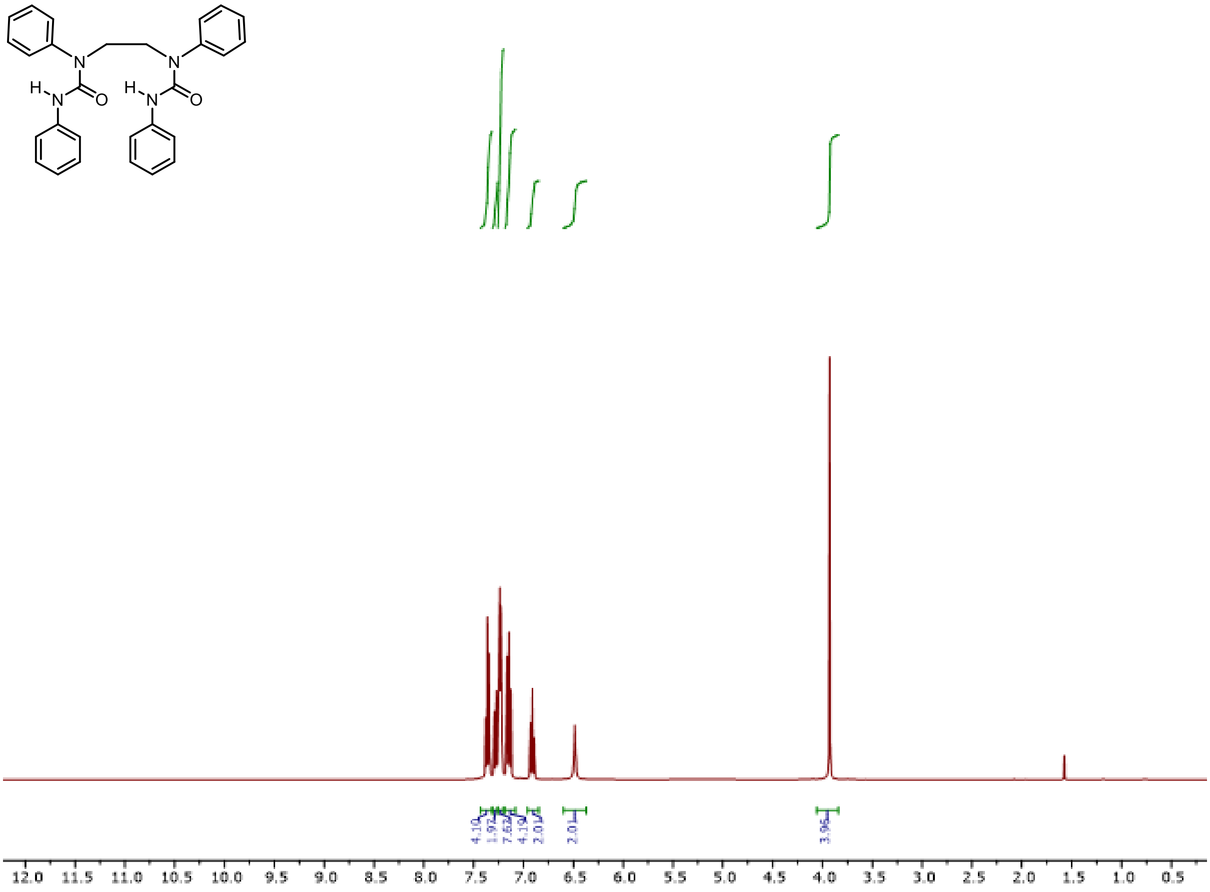


**Figure S62 – $^{1}$H NMR spectrum of 2c (400 MHz, $CDCl_3$).**

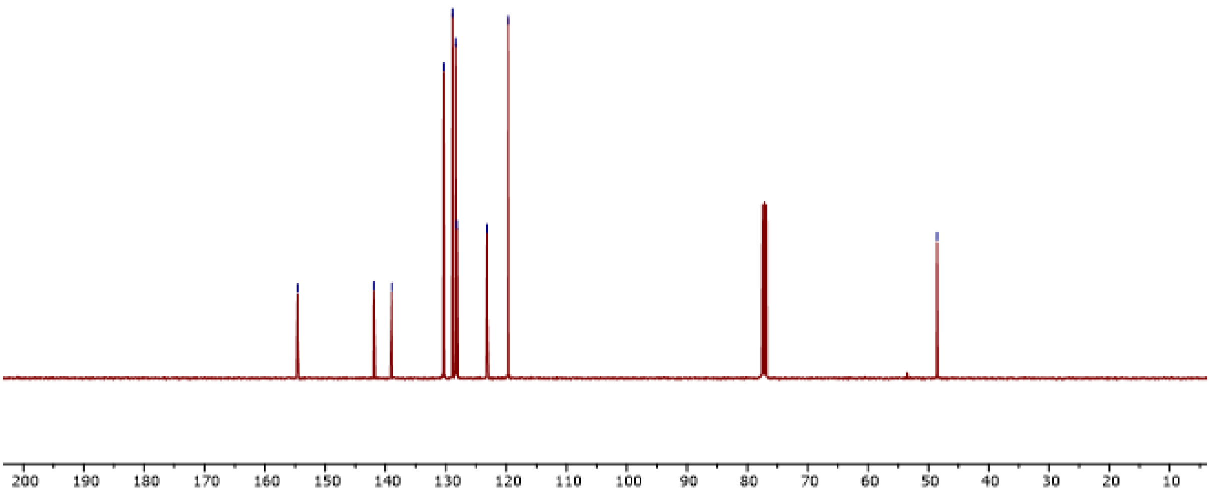


**Figure S63 – $^{13}$C NMR spectrum of 2c (101 MHz, $CDCl_3$).**

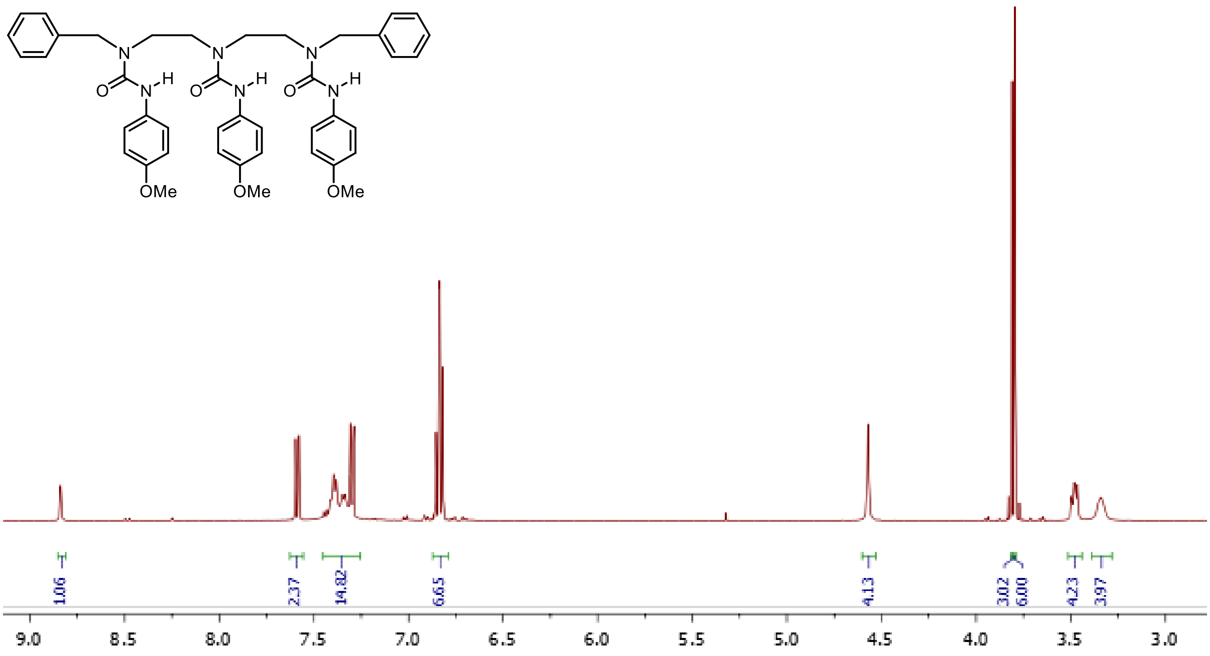


**Figure S64 – $^{1}$H NMR spectrum of 3a (400 MHz, $CDCl_3$).**

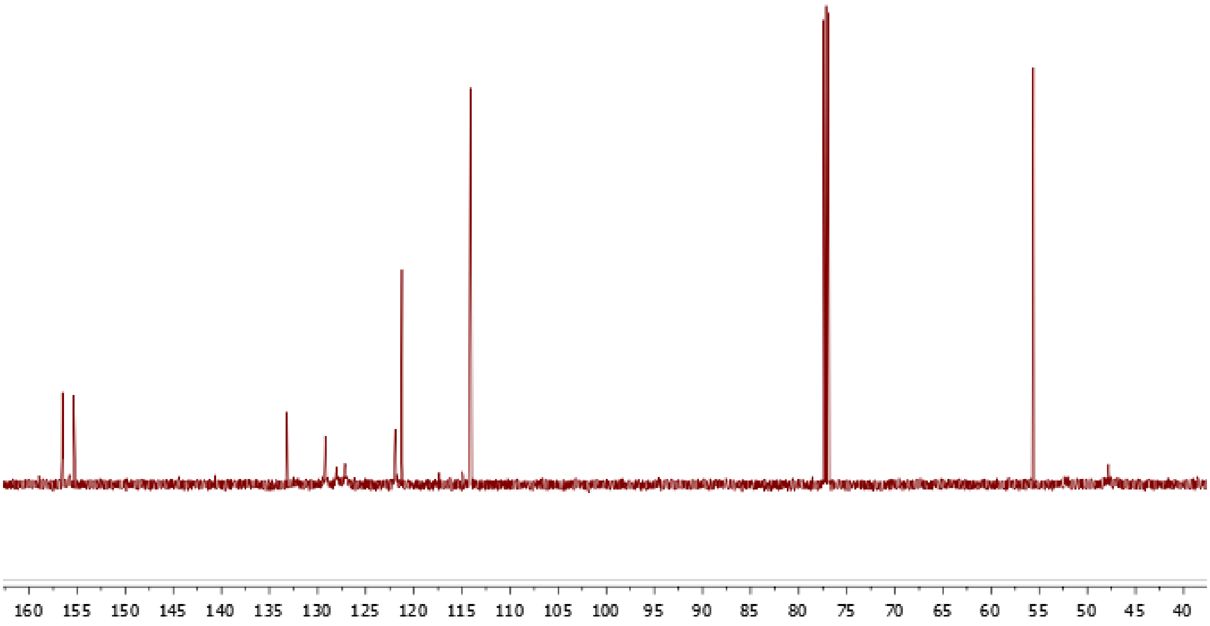


**Figure S65 – $^{13}$C NMR spectrum of 3a (101 MHz, $CDCl_3$).**

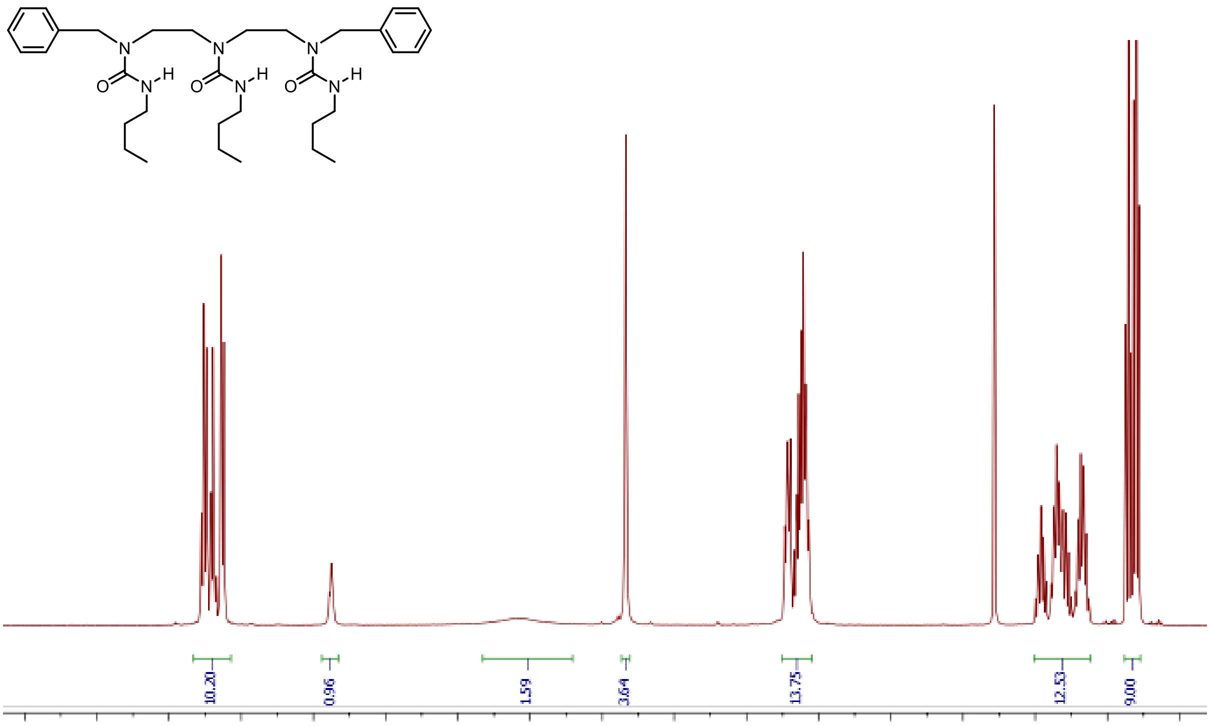


**Figure S66 – $^{1}$H NMR spectrum of 3b (400 MHz, $CDCl_3$).**

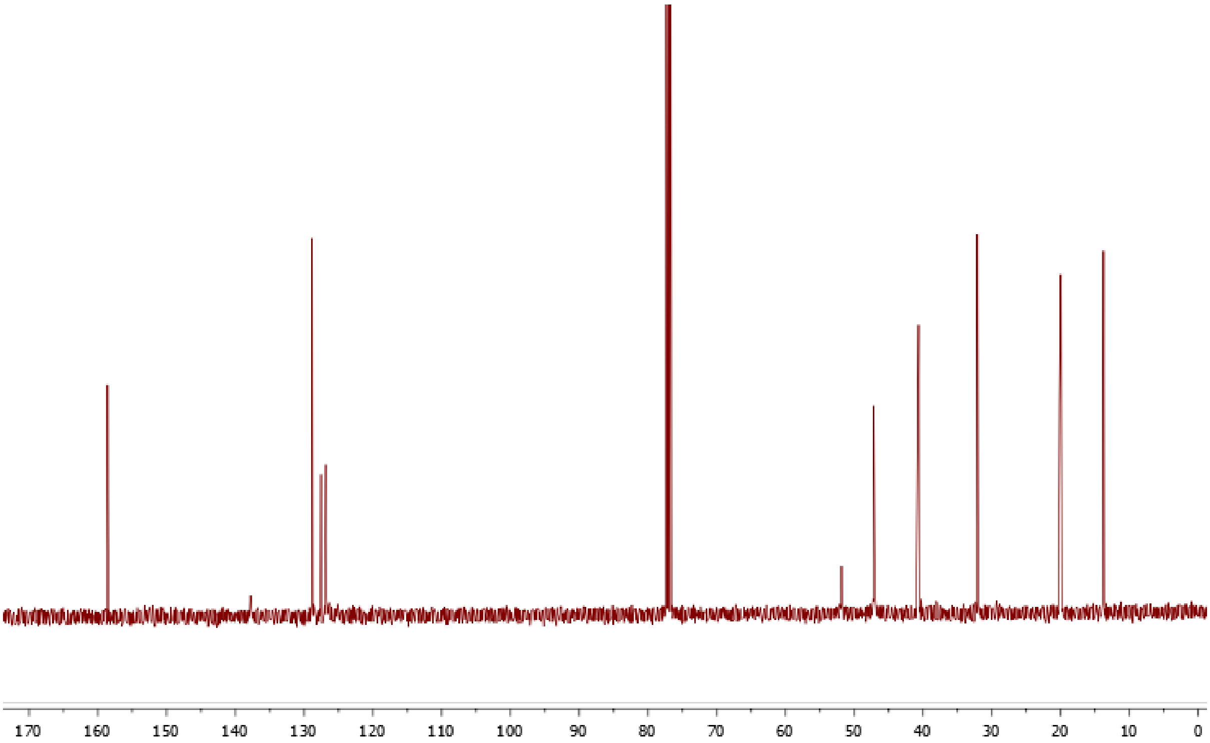


**Figure S67 – $^{13}$C NMR spectrum of 3b (101 MHz, $CDCl_3$).**

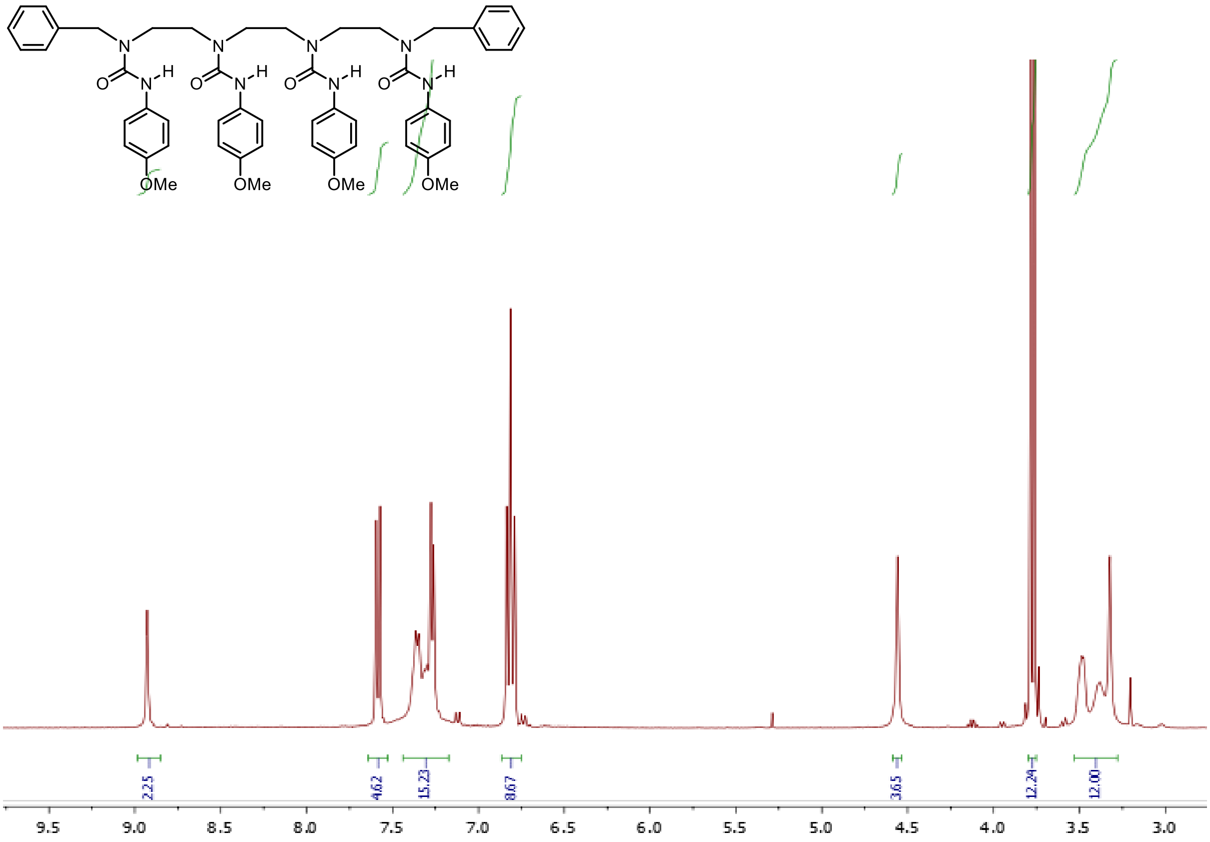


**Figure S68 – $^{1}H$ NMR spectrum of 4a (400 MHz, $CDCl_3$).**

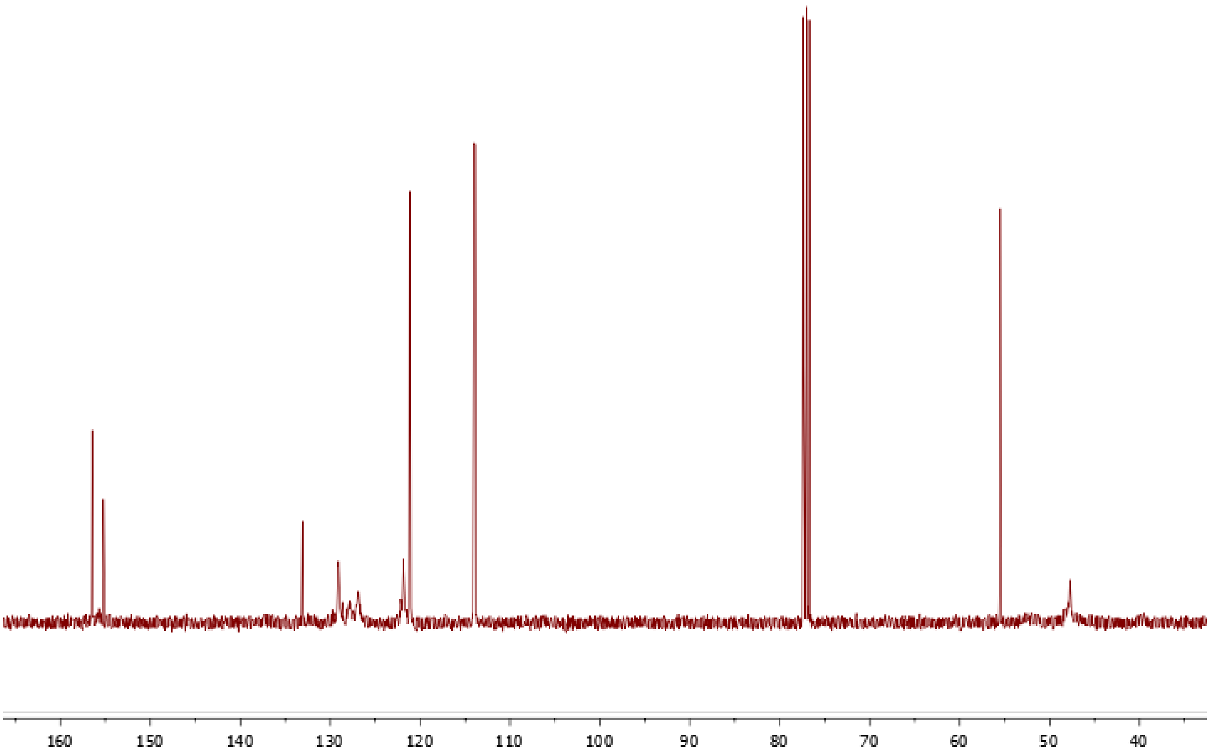


**Figure S69 – $^{13}C$ NMR spectrum of 4a (101 MHz, $CDCl_3$).**

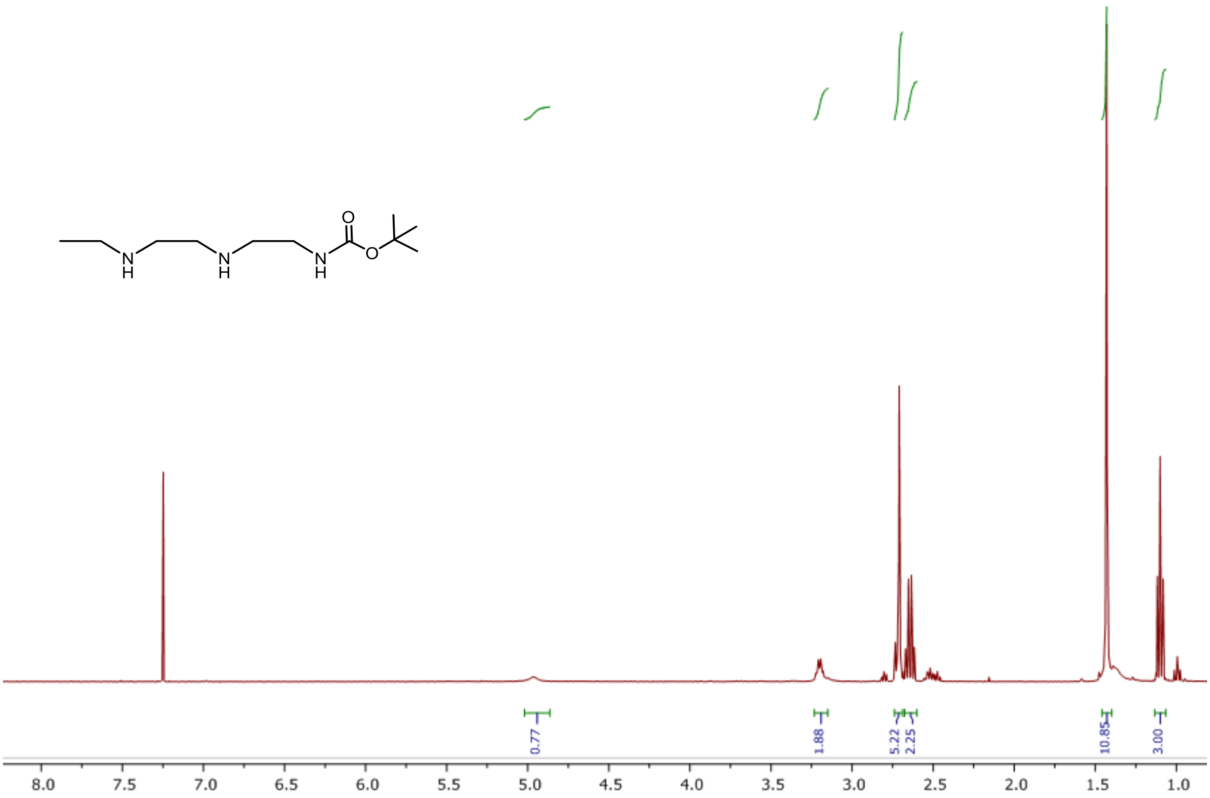


**Figure S70 – $^{1}$H NMR spectrum of 3c-1 (400 MHz, $CDCl_3$).**

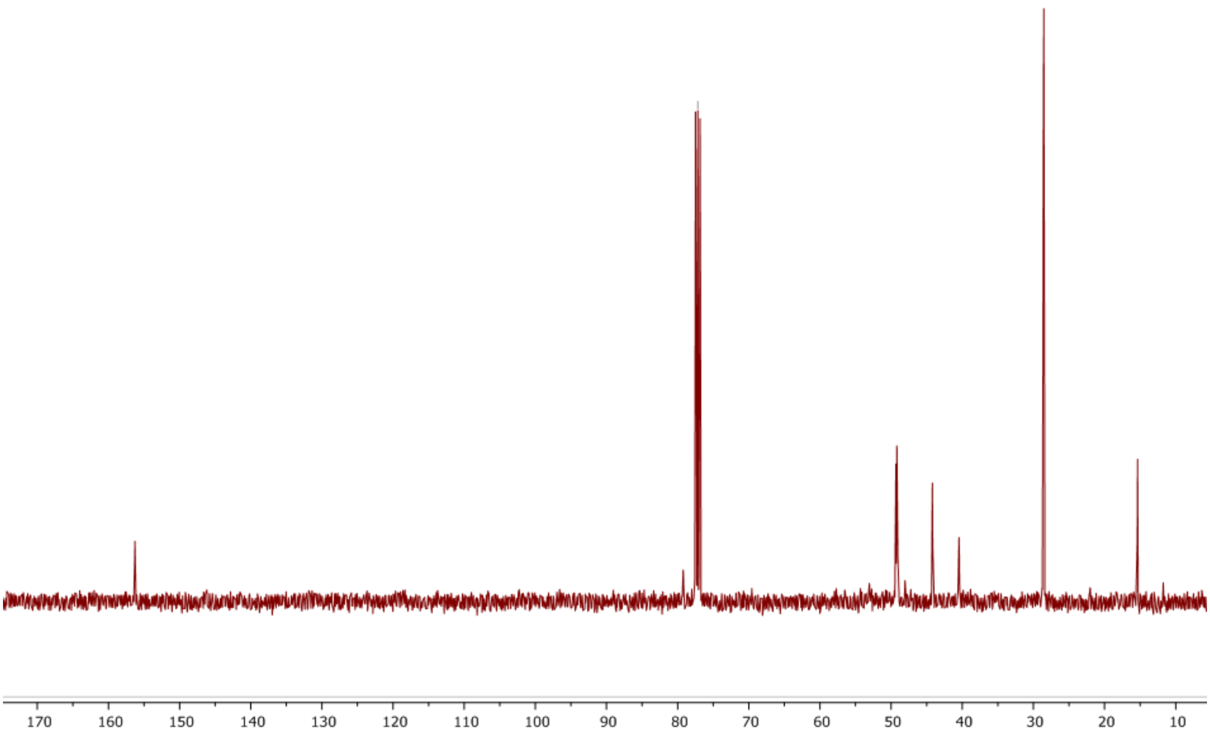


**Figure S71 – $^{13}$C NMR spectrum of 3c-1 (101 MHz, $CDCl_3$).**

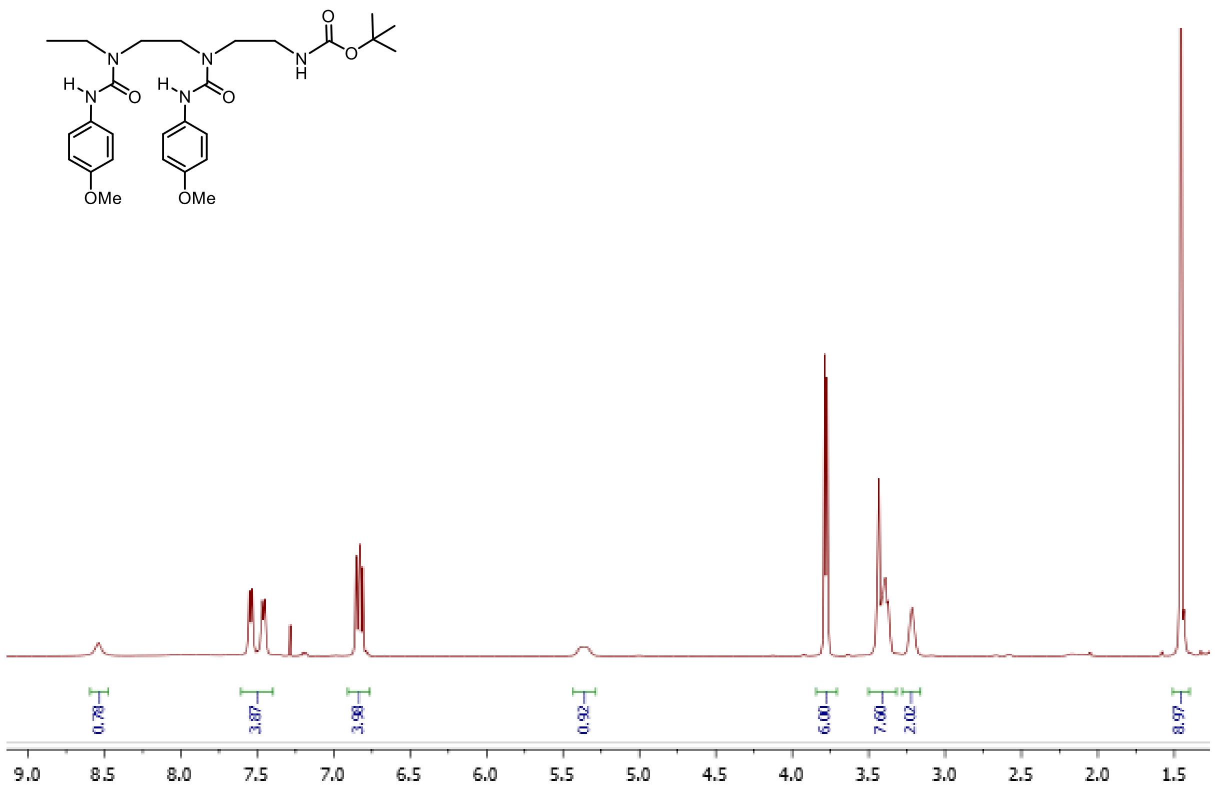


**Figure S72 – $^{1}$H NMR spectrum of 3c-2 (500 MHz, $CDCl_3$).**

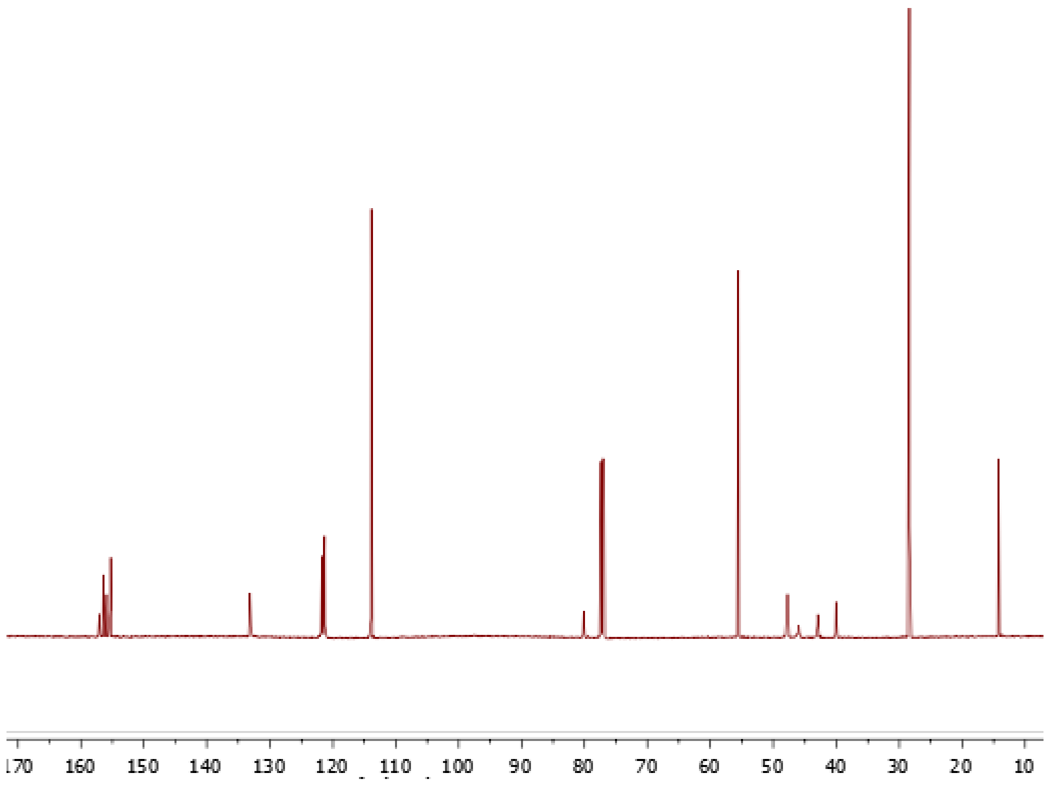


**Figure S73 – $^{13}$C NMR spectrum of 3c-2 (126 MHz, $CDCl_3$).**

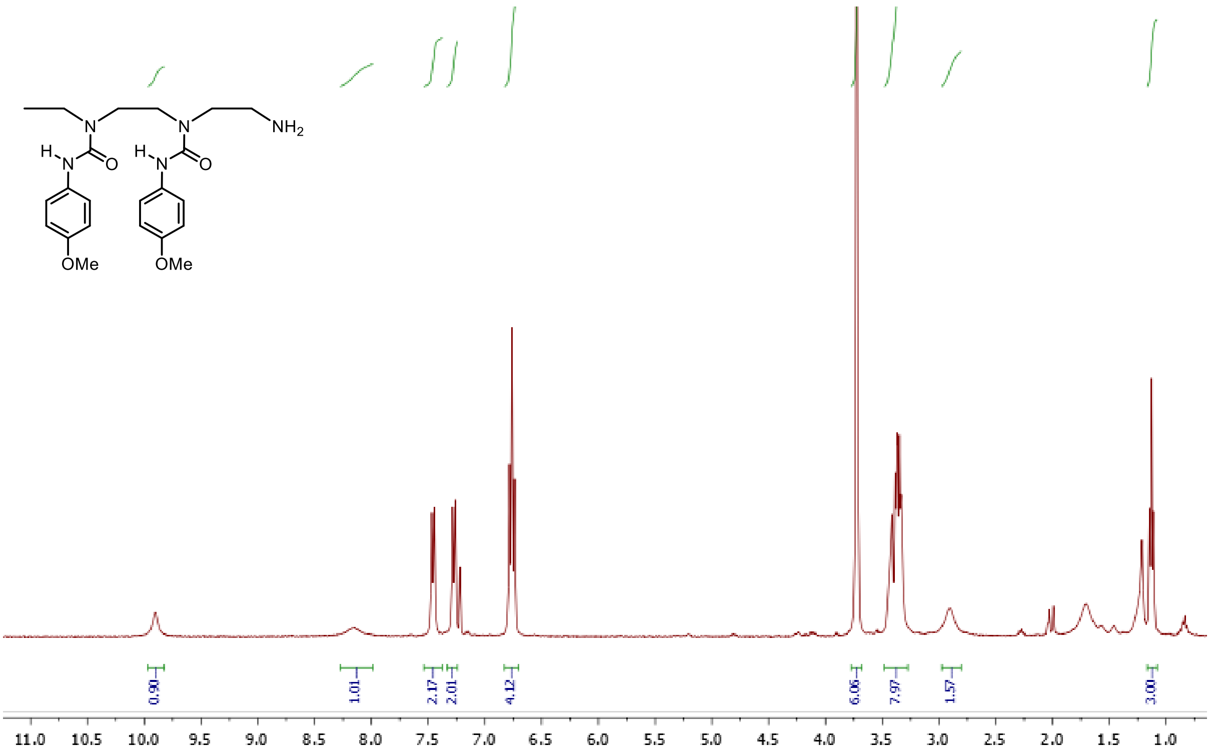


**Figure S74 – $^{1}$H NMR spectrum of 3c-3 (400 MHz, $CDCl_3$).**

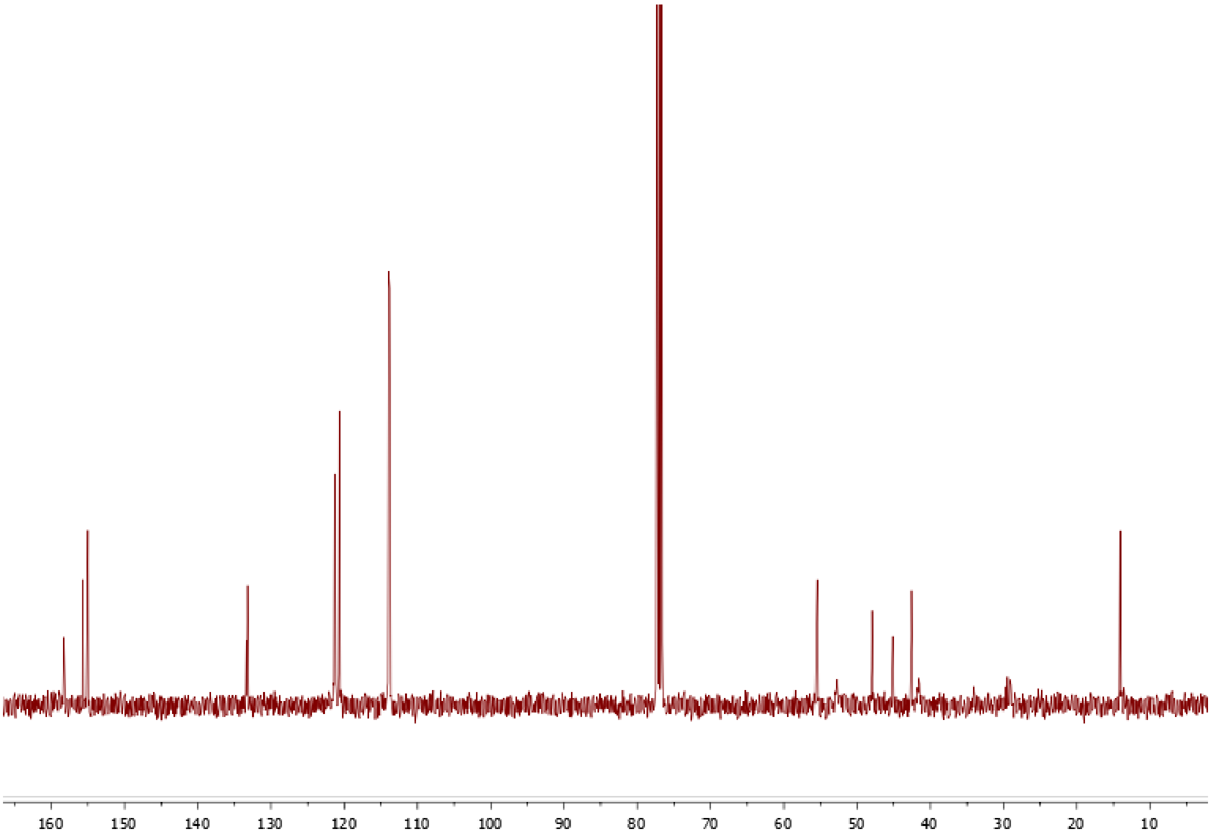


**Figure S75 – $^{13}$C NMR spectrum of 3c-3 (101 MHz, $CDCl_3$).**

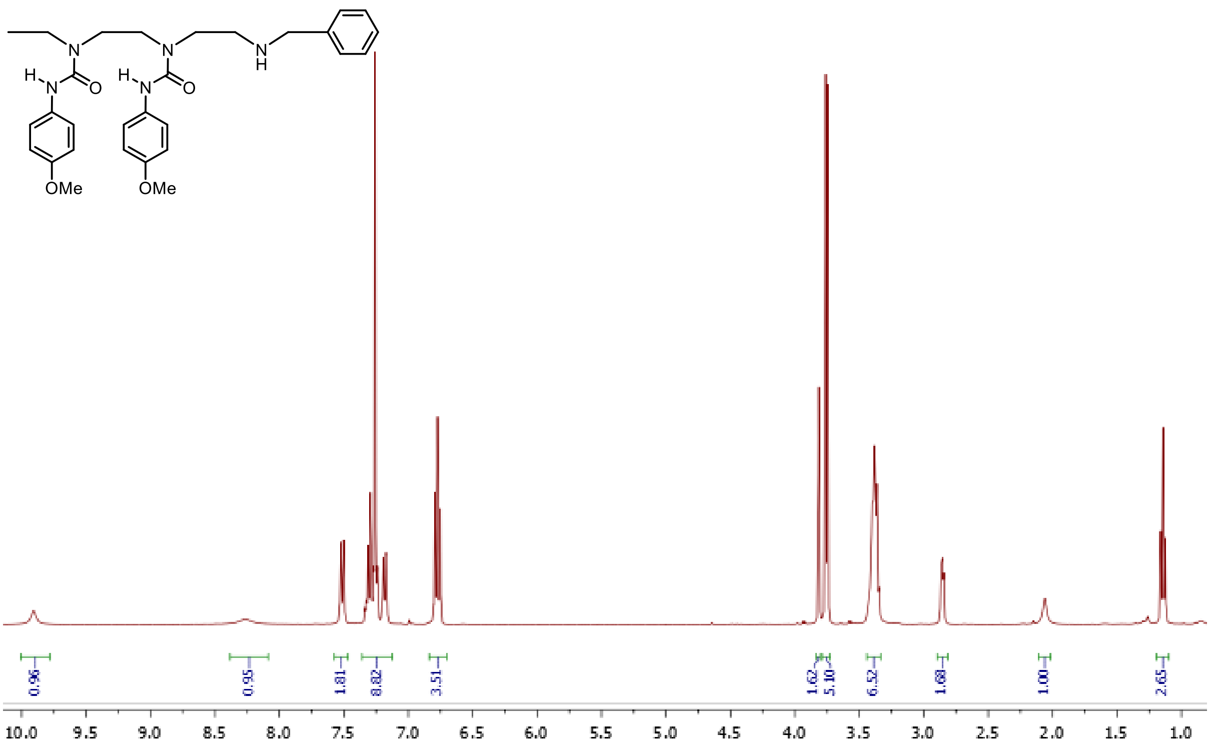


**Figure S76 – $^{1}$H NMR spectrum of 3c-4 (400 MHz, $CDCl_3$).**

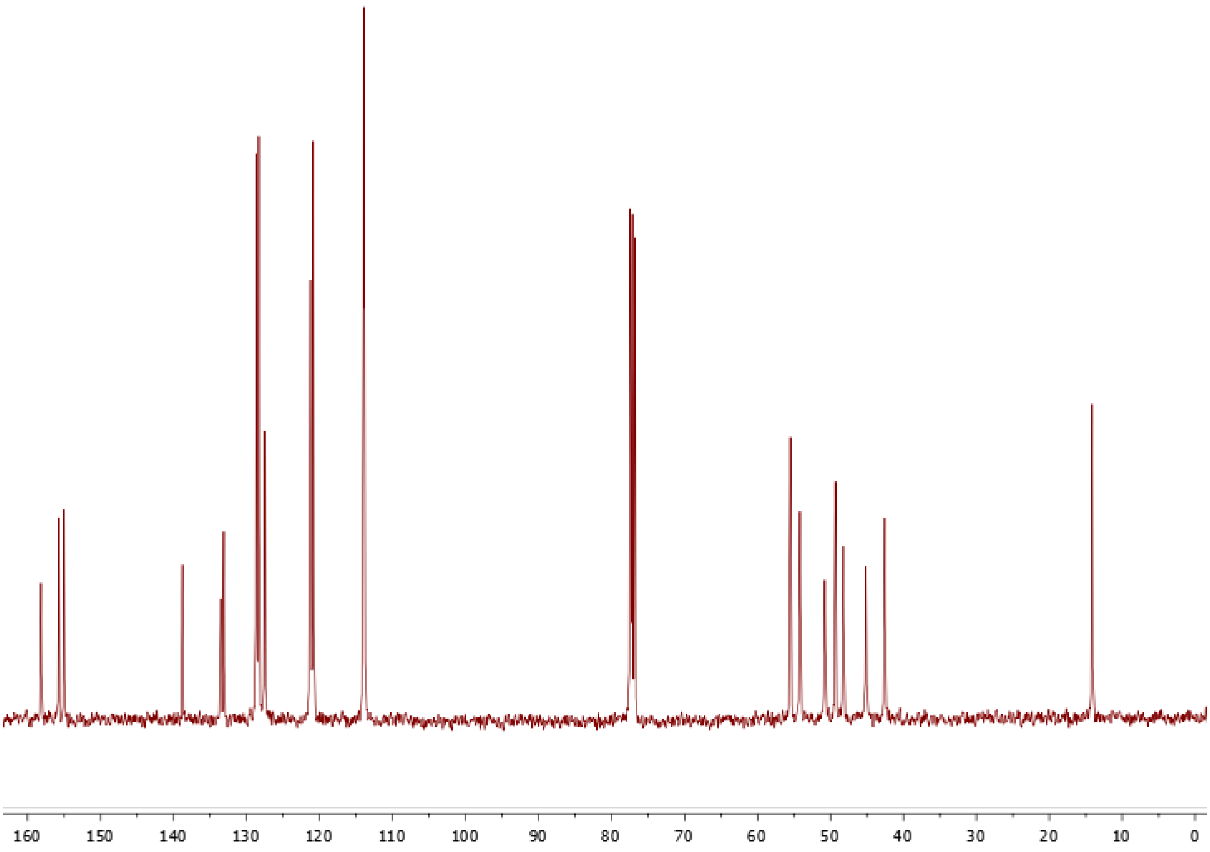


**Figure S77 – $^{13}$C NMR spectrum of 3c-4 (101 MHz, $CDCl_3$).**

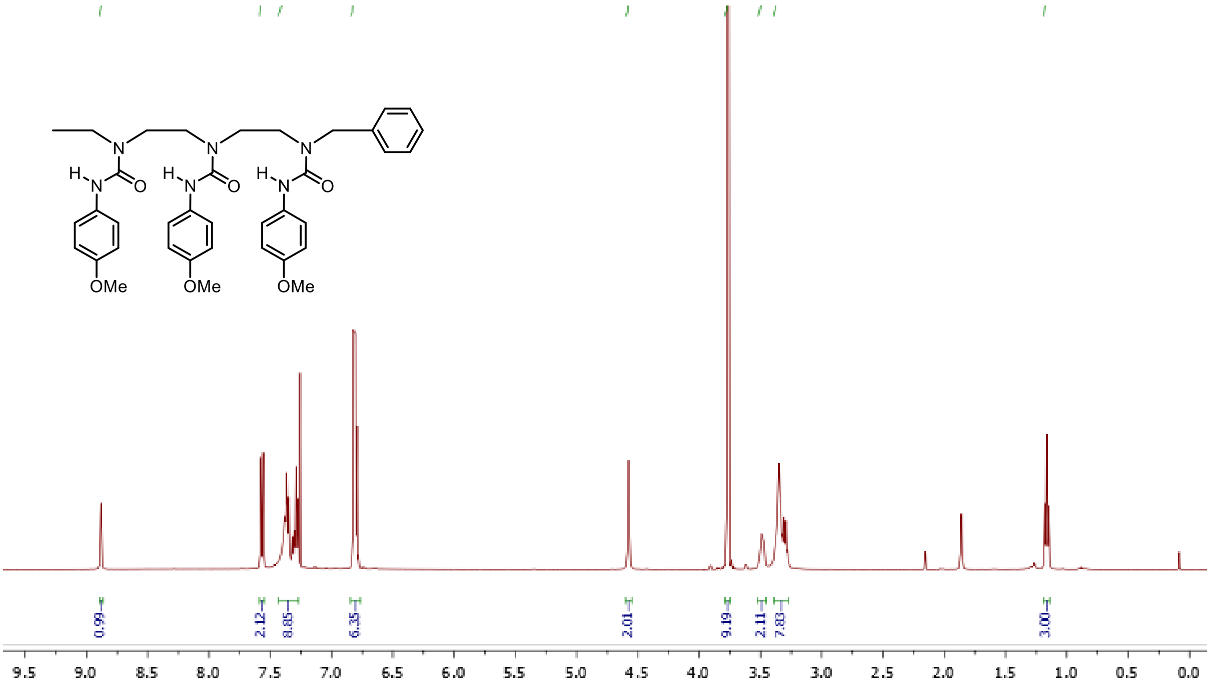


**Figure S78 – $^{1}$H NMR spectrum of 3c (500 MHz, $CDCl_3$).**

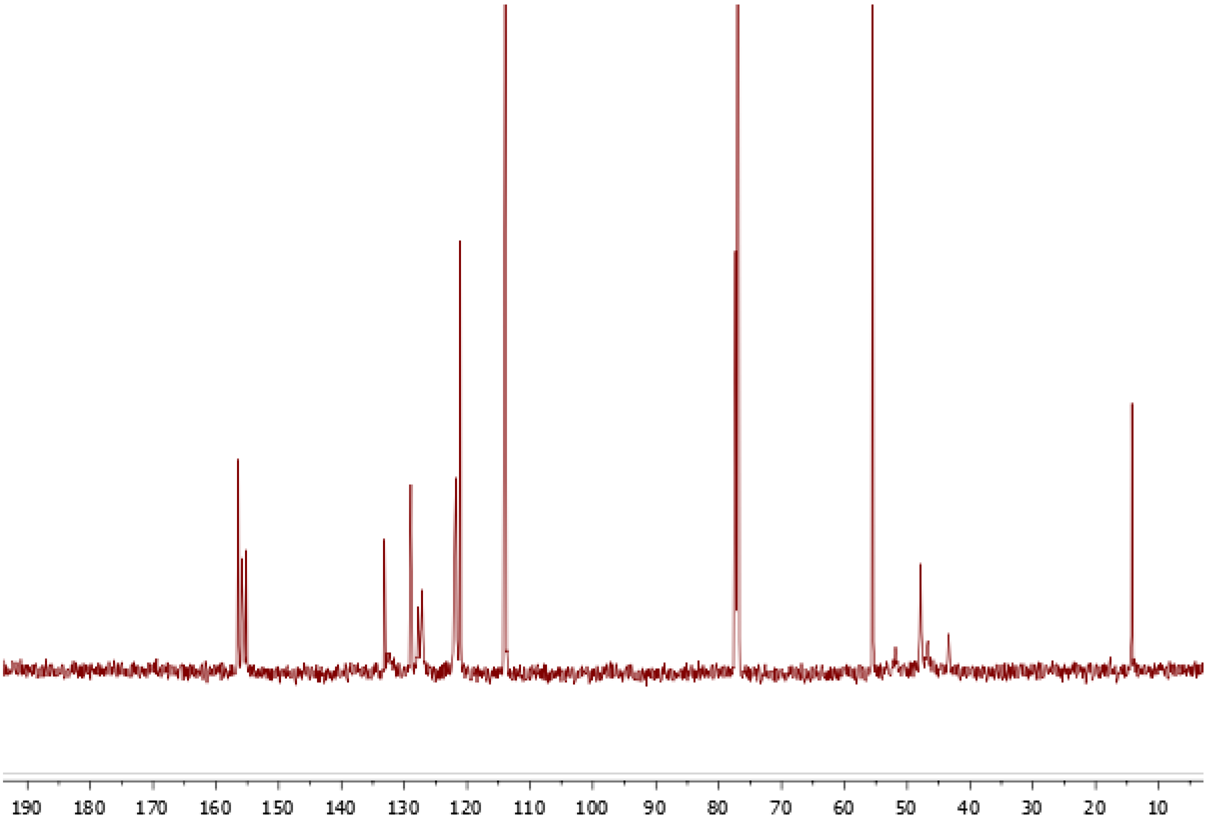


**Figure S79 – $^{13}$C NMR spectrum of 3c (126 MHz, $CDCl_3$).**

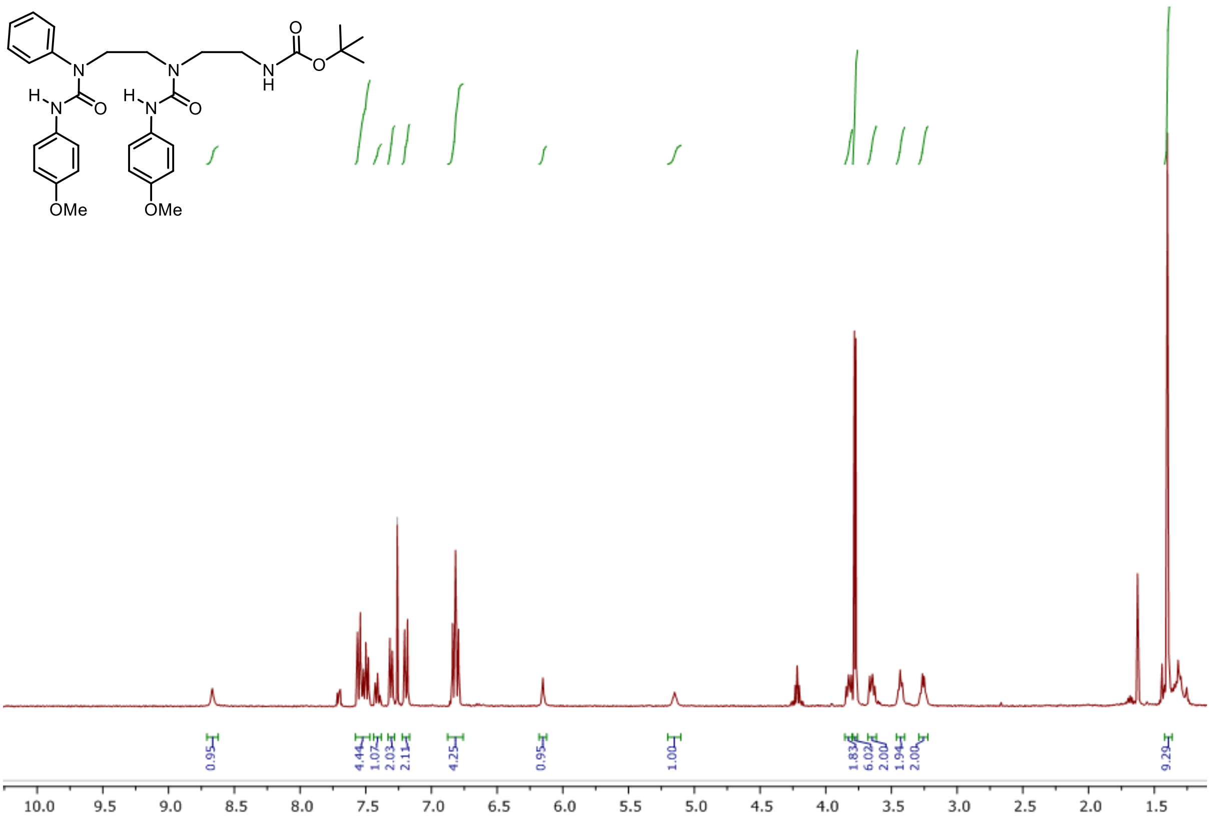

Figure S80 – $^{1}$H NMR spectrum of 3d-2 (400 MHz, $CDCl_3$).

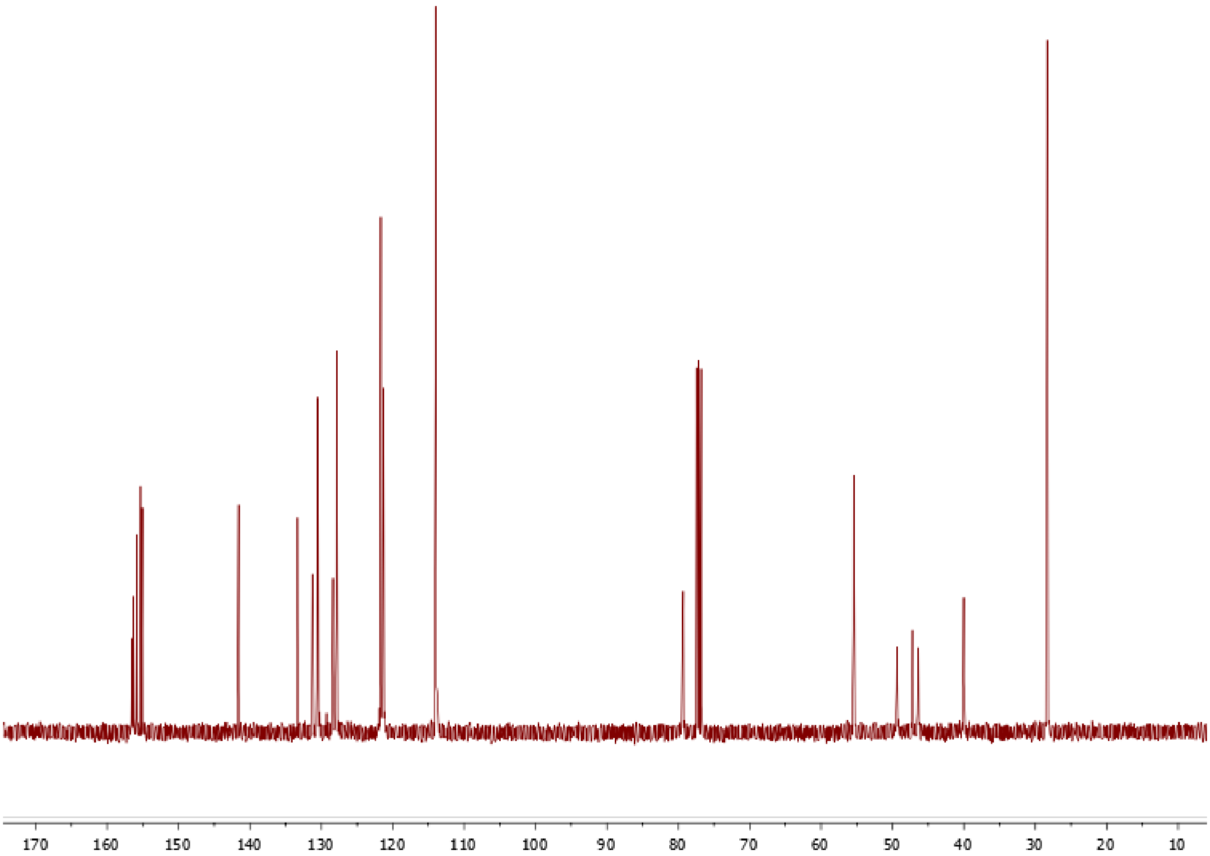

Figure S81 – $^{13}$C NMR spectrum of 3d-2 (101 MHz, $CDCl_3$).

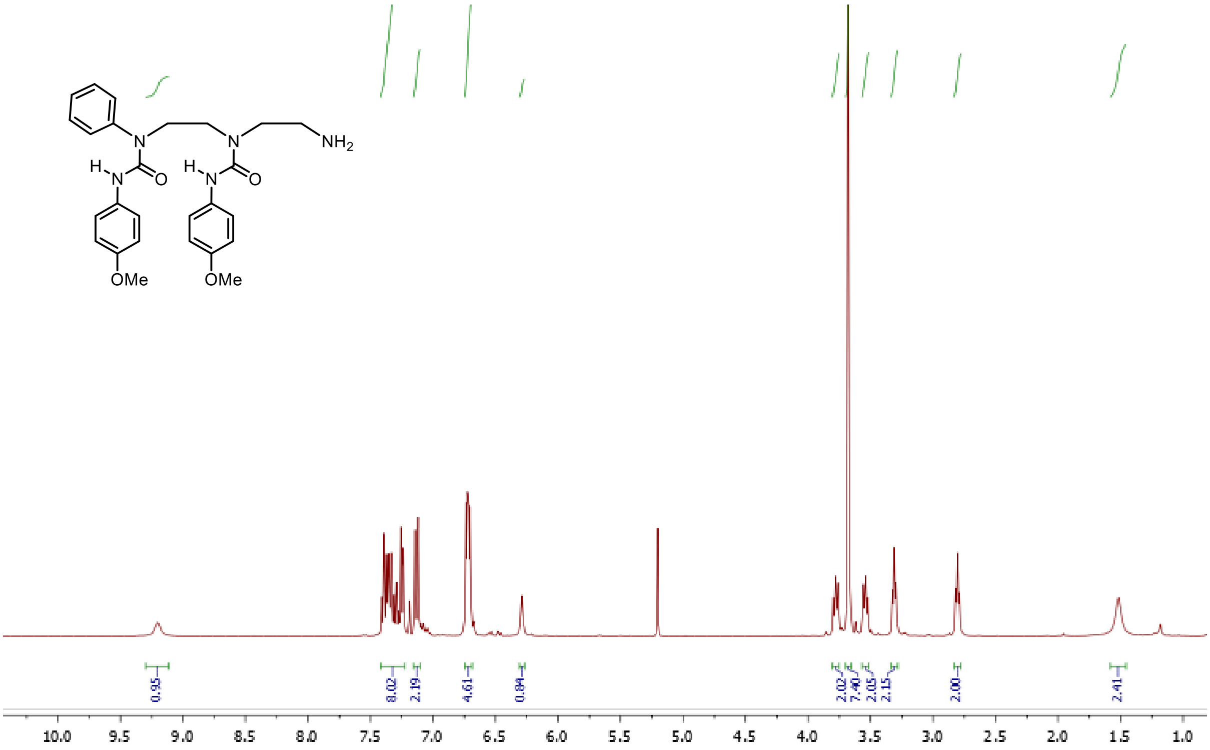


**Figure S82 – $^{1}$H NMR spectrum of 3d-3 (400 MHz, $CDCl_3$).**

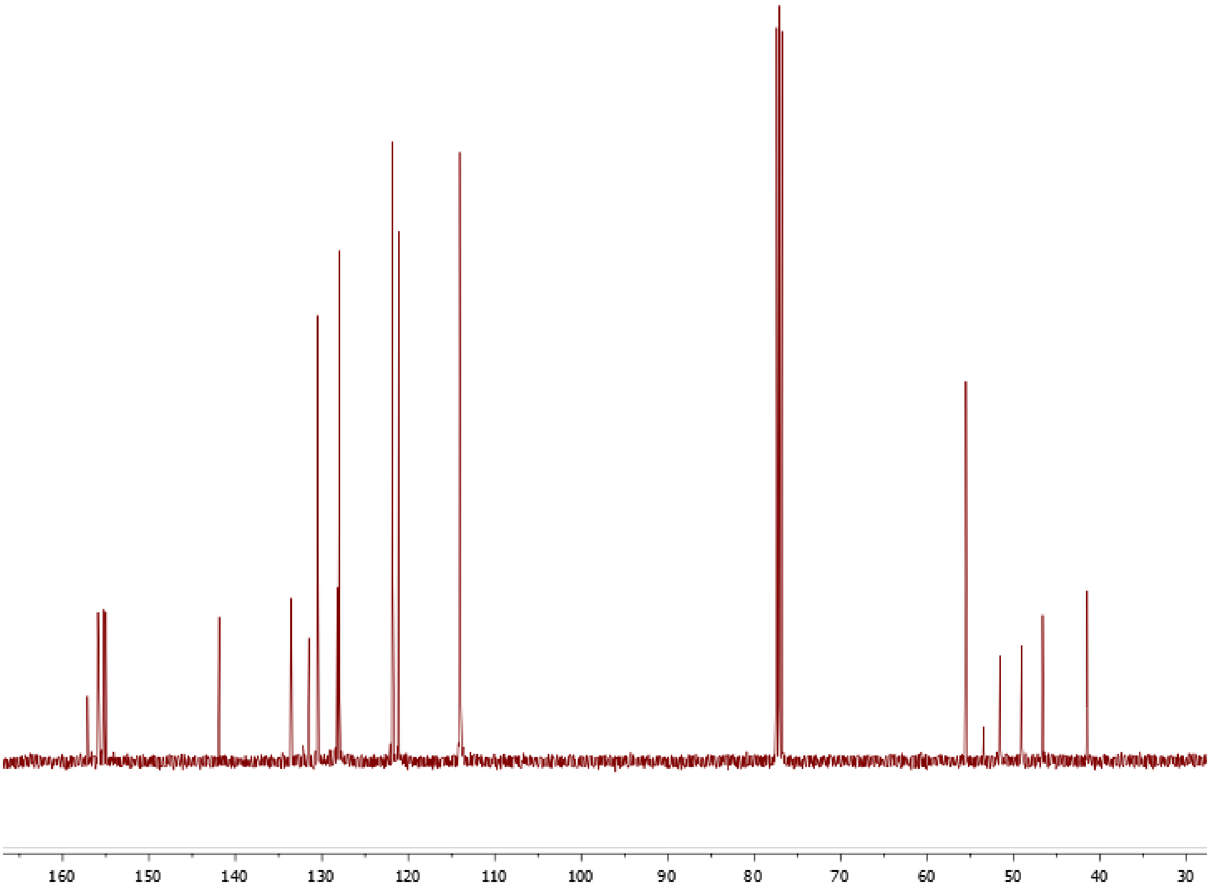


**Figure S83 – $^{13}$C NMR spectrum of 3d-3 (101 MHz, $CDCl_3$).**

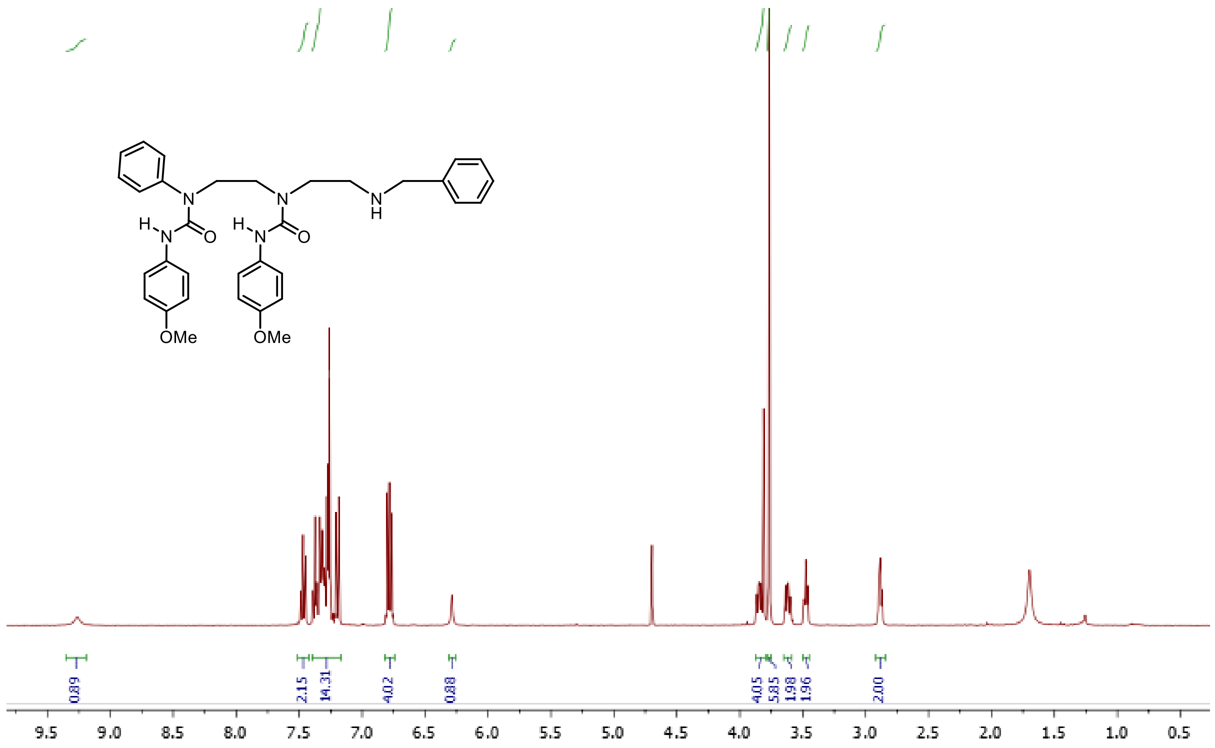


**Figure S84 – $^{1}$H NMR spectrum of 3d-4 (400 MHz, $CDCl_3$).**

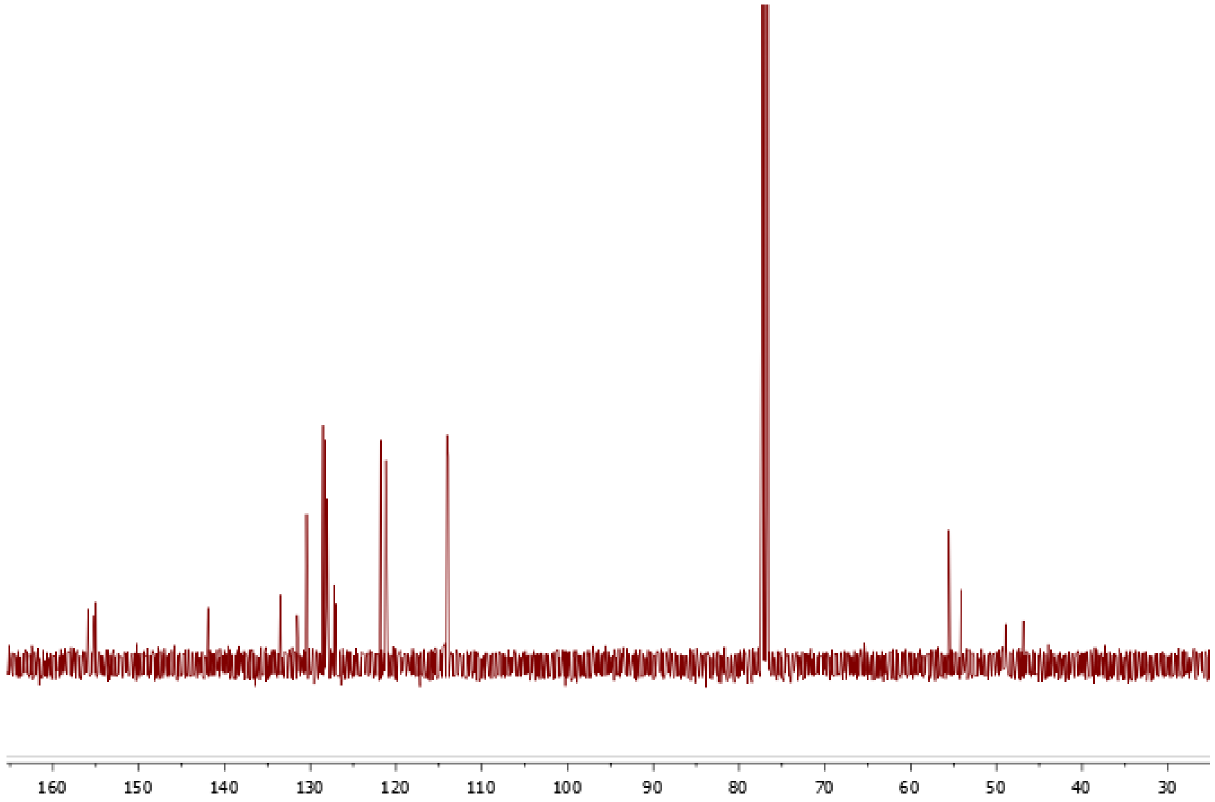


**Figure S85 – $^{13}$C NMR spectrum of 3d-4 (101 MHz, $CDCl_3$).**

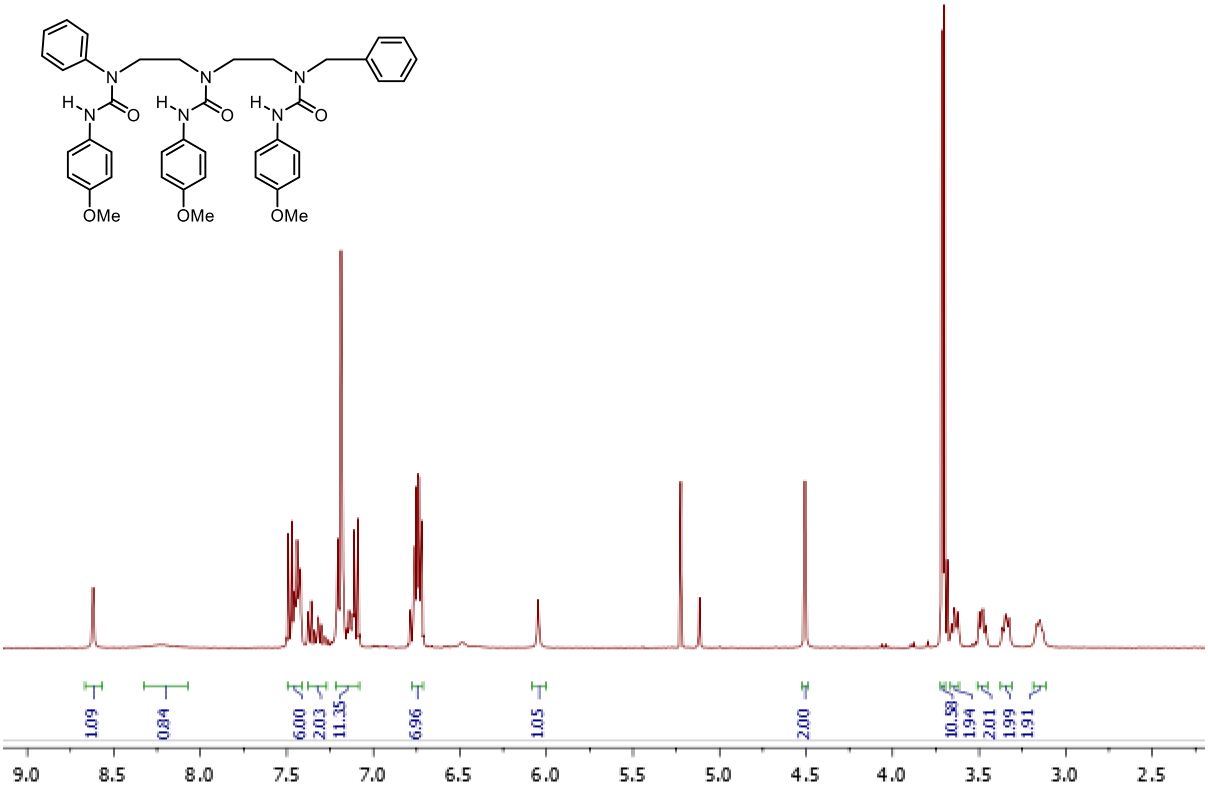


**Figure S86 – $^{1}$H NMR spectrum of 3d (400 MHz, $CDCl_3$).**

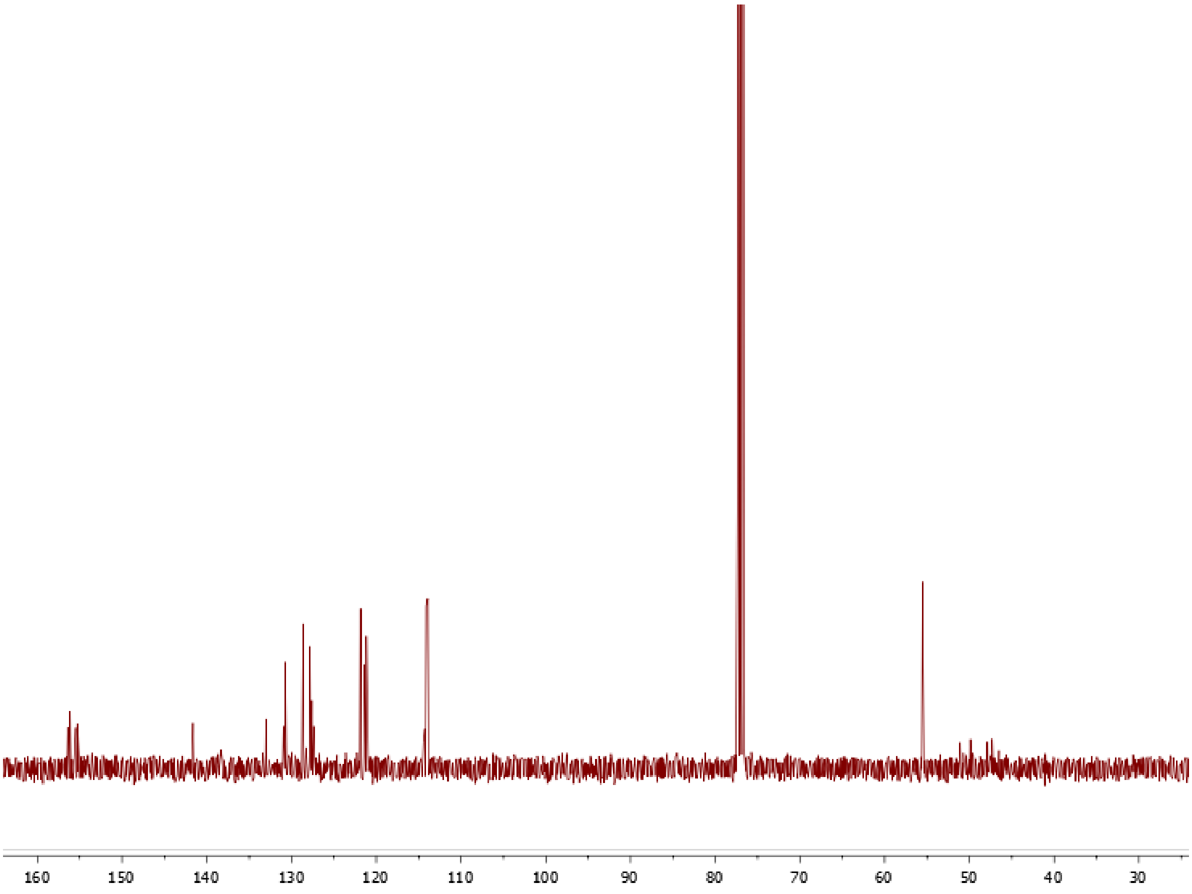


**Figure S87 – $^{13}$C NMR spectrum of 3d (101 MHz, $CDCl_3$).**

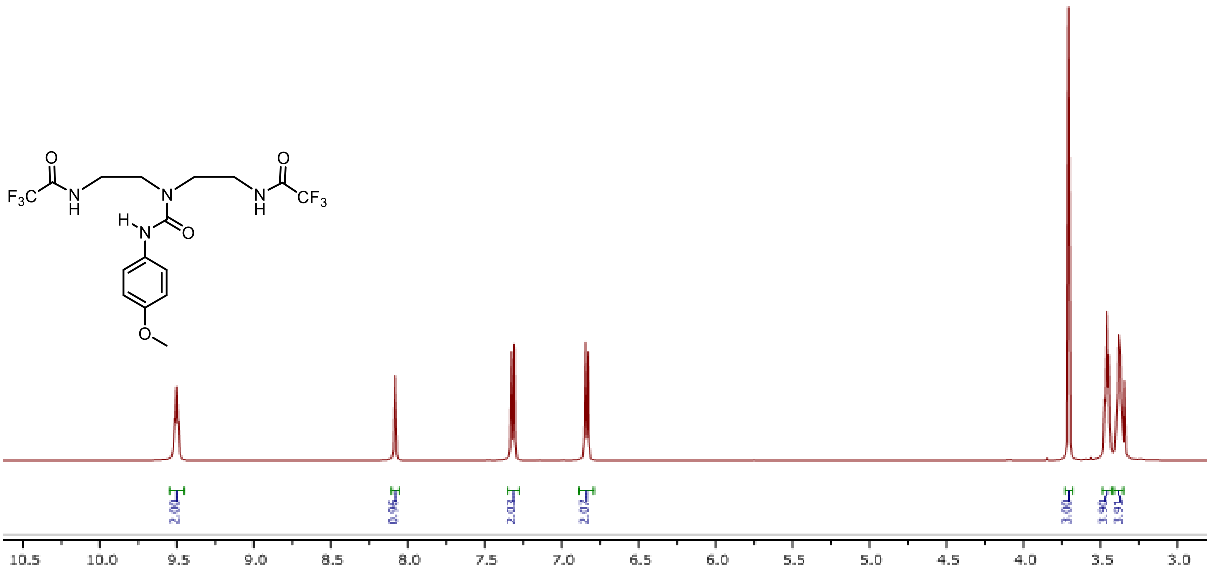


**Figure S88 – $^{1}H$ NMR spectrum of 3e-1 (500 MHz, $(CD_3)_2SO$).**

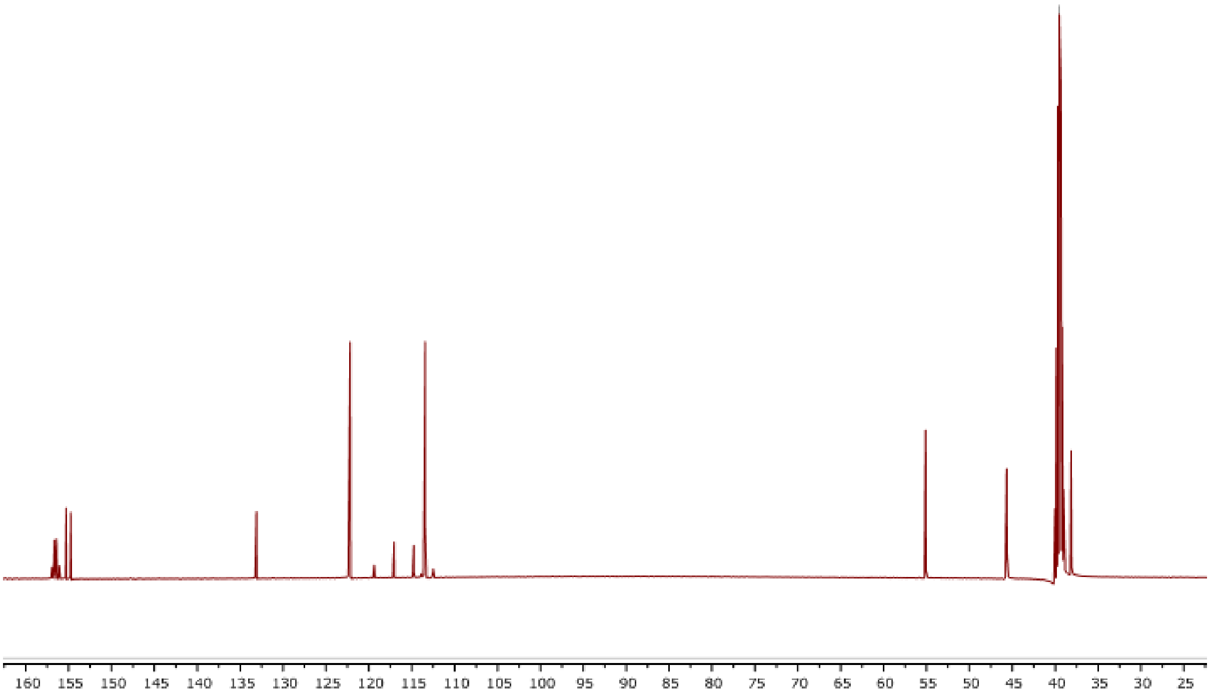


**Figure S89 – $^{13}C$ NMR spectrum of 3e-1 (126 MHz, $(CD_3)_2SO$).**

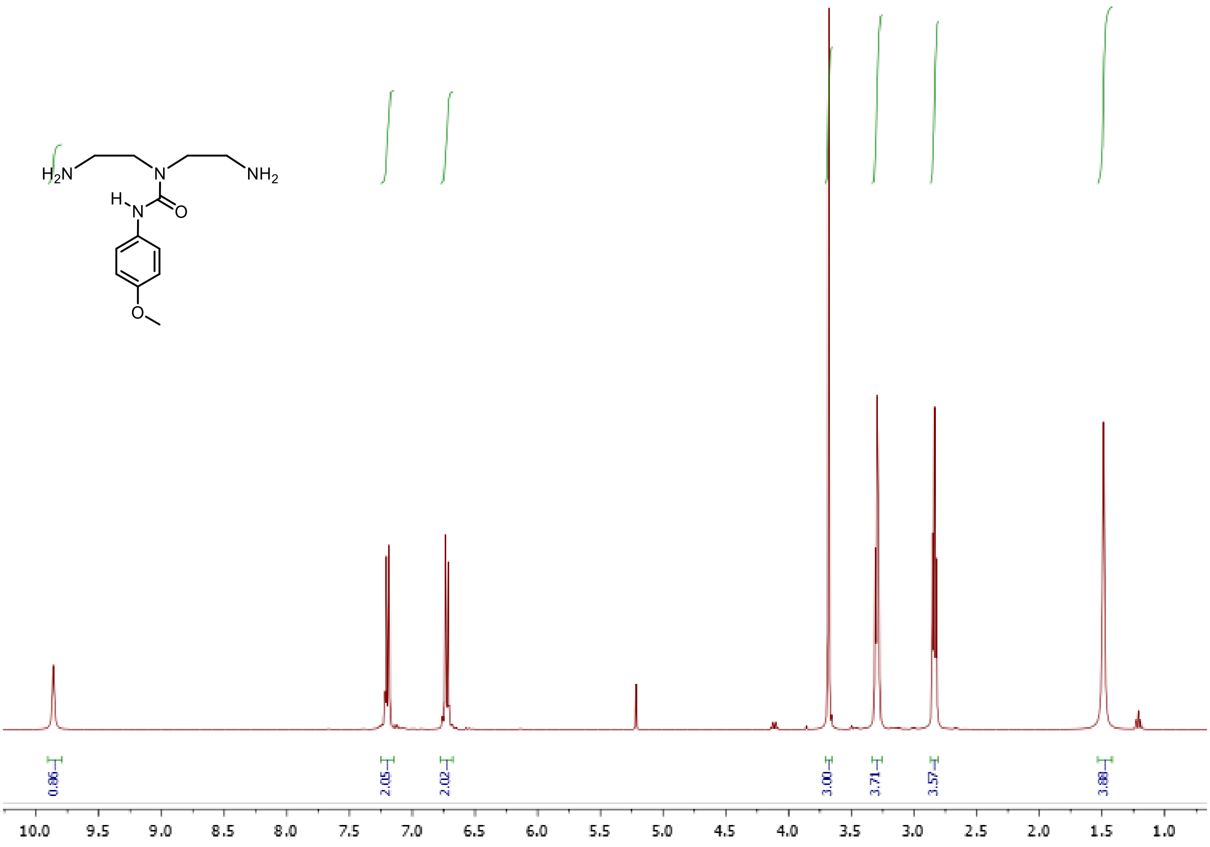


**Figure S90 – $^{1}$H NMR spectrum of 3e-2 (400 MHz, $CDCl_3$).**

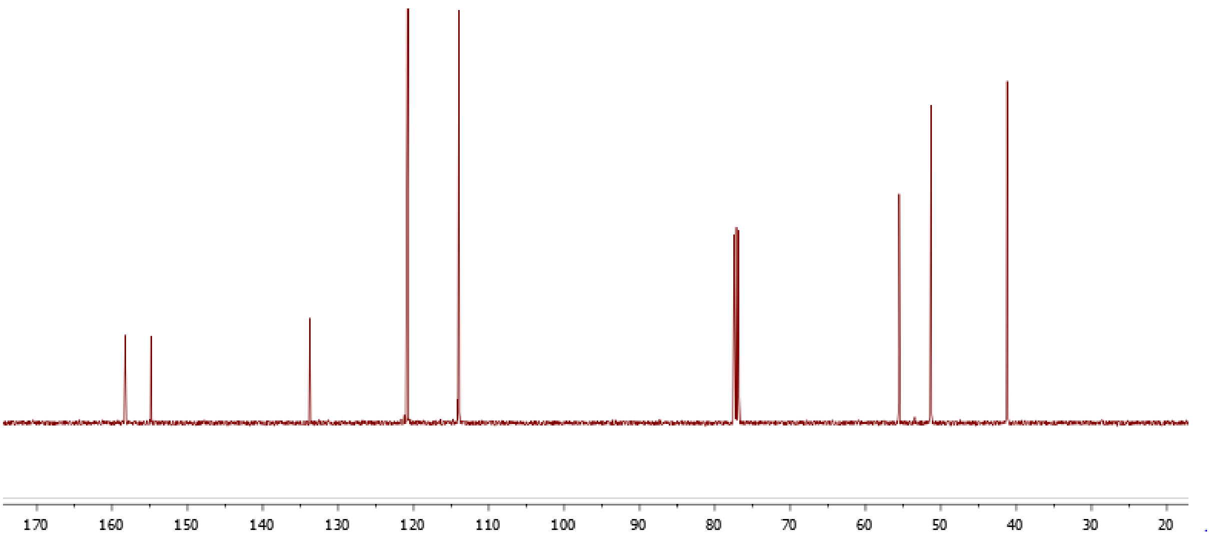


**Figure S91 – $^{13}$C NMR spectrum of 3e-2 (101 MHz, $CDCl_3$).**

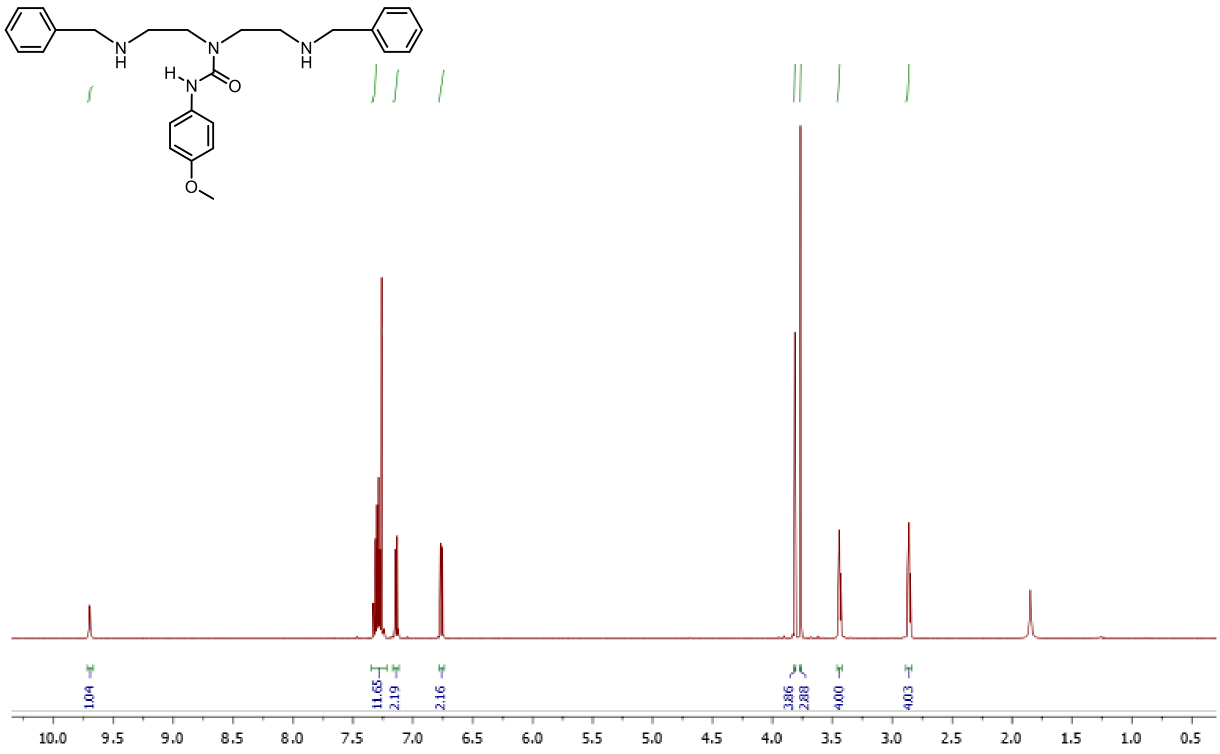


**Figure S92 – $^{1}$H NMR spectrum of 3e-3 (500 MHz, $CDCl_3$).**

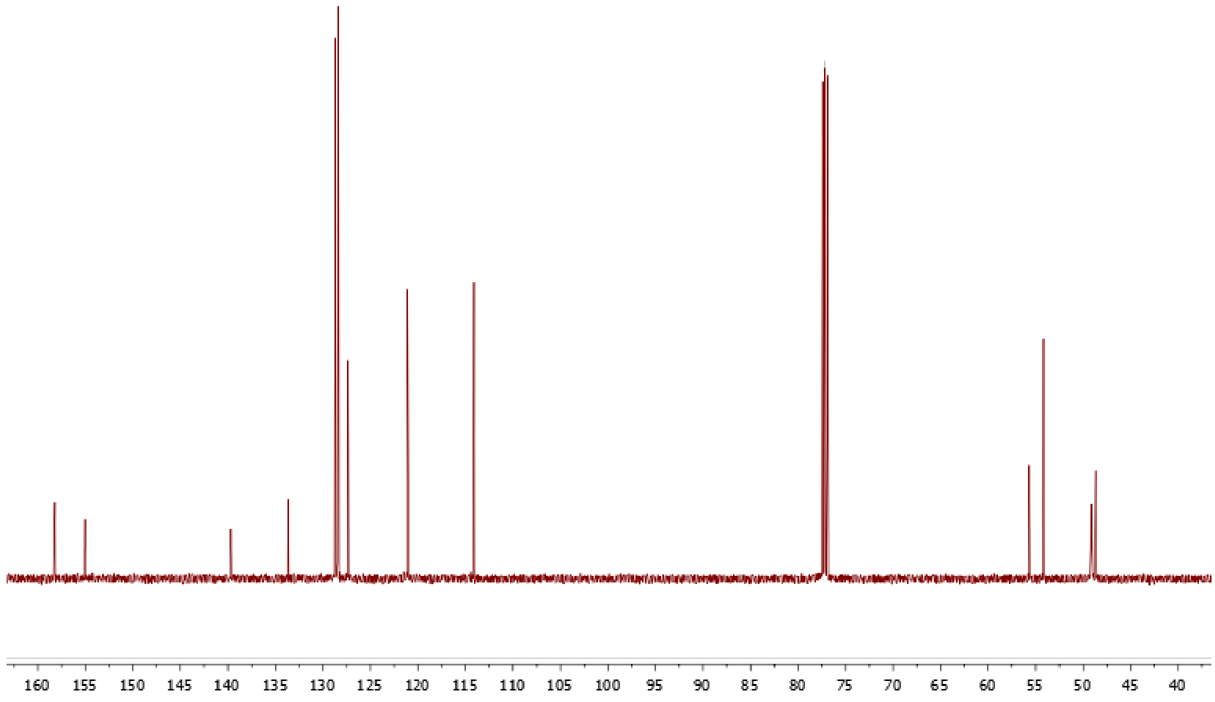


**Figure S93 – $^{13}$C NMR spectrum of 3e-3 (126 MHz, $CDCl_3$).**

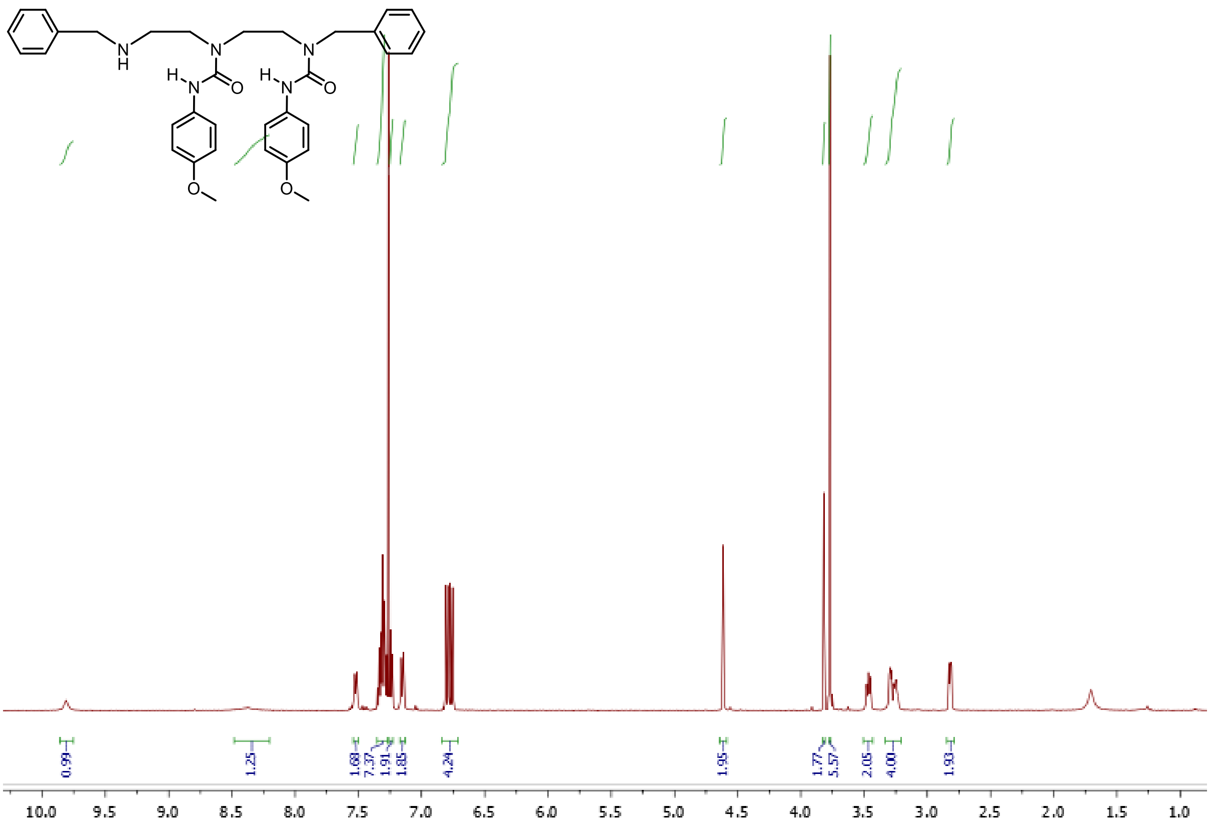

**Figure S94 – $^{1}H$ NMR spectrum of 3e-4 (500 MHz, $CDCl_3$).**

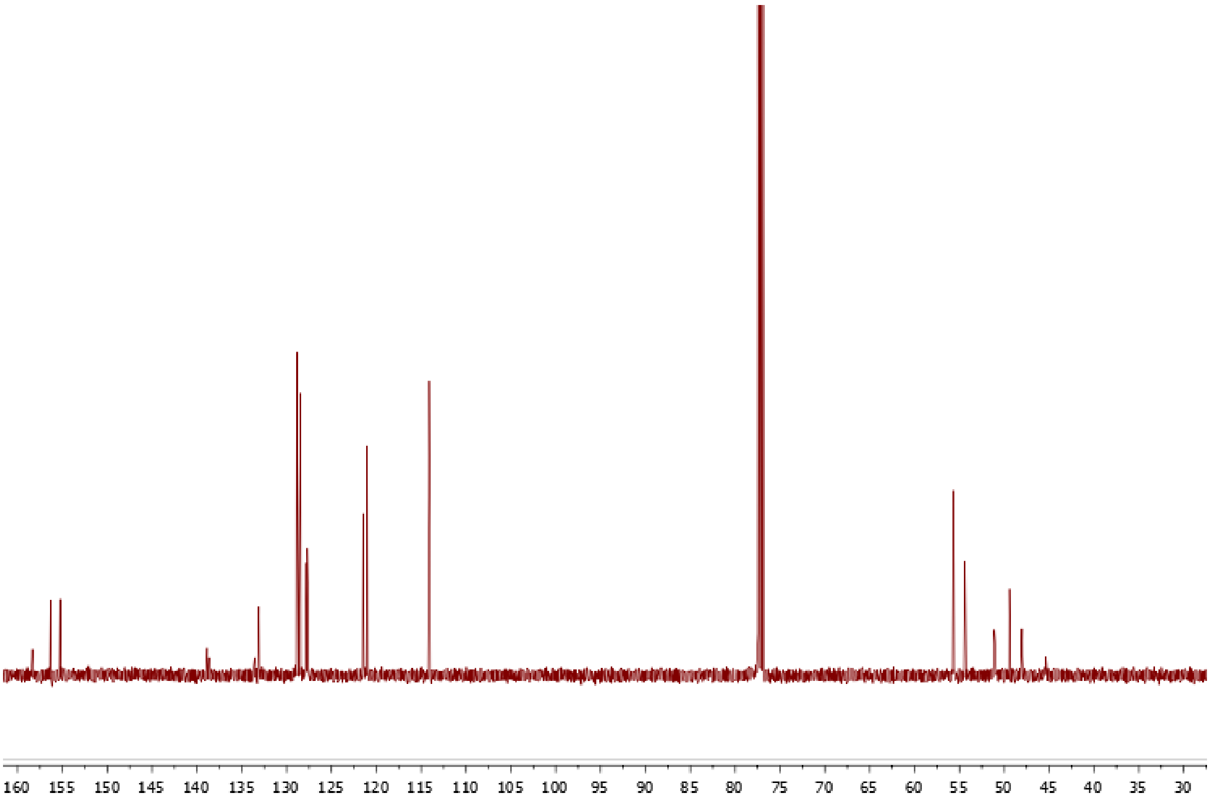

**Figure S95 – $^{13}C$ NMR spectrum of 3e-4 (126 MHz, $CDCl_3$).**

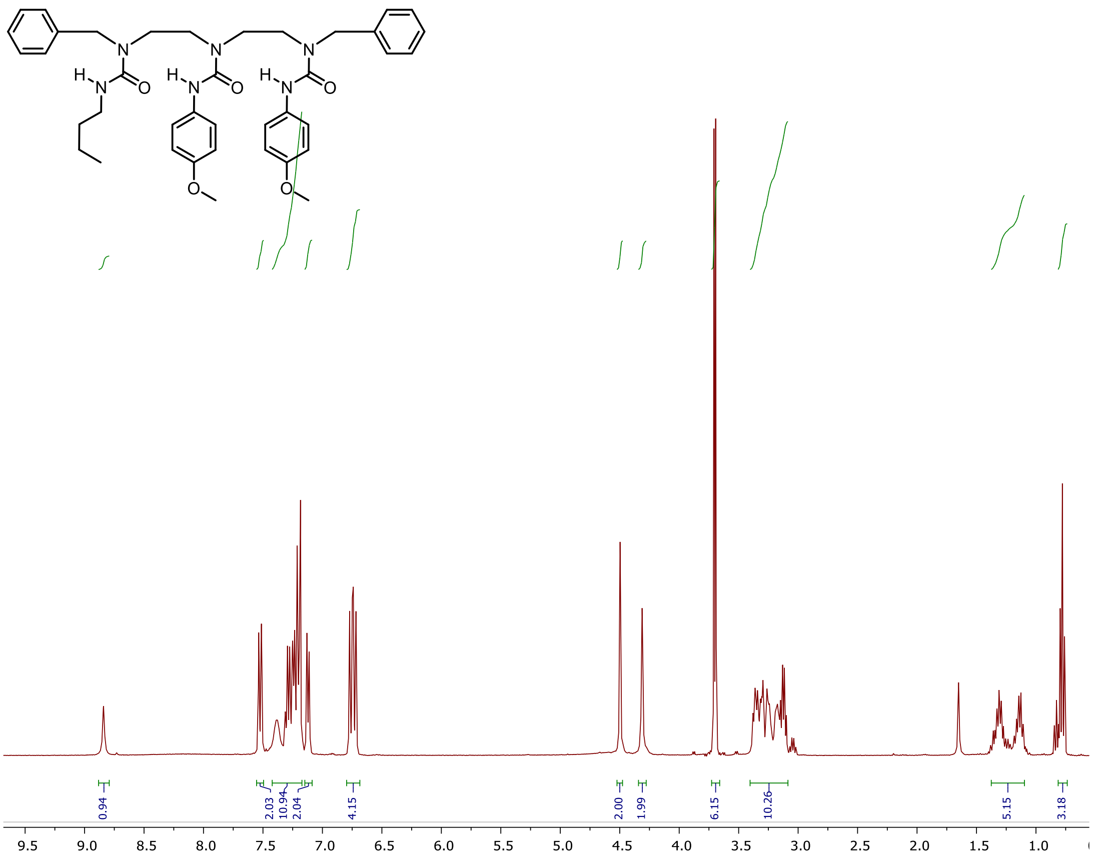


**Figure S96 – $^{1}$H NMR spectrum of 3e (400 MHz, $CDCl_3$).**

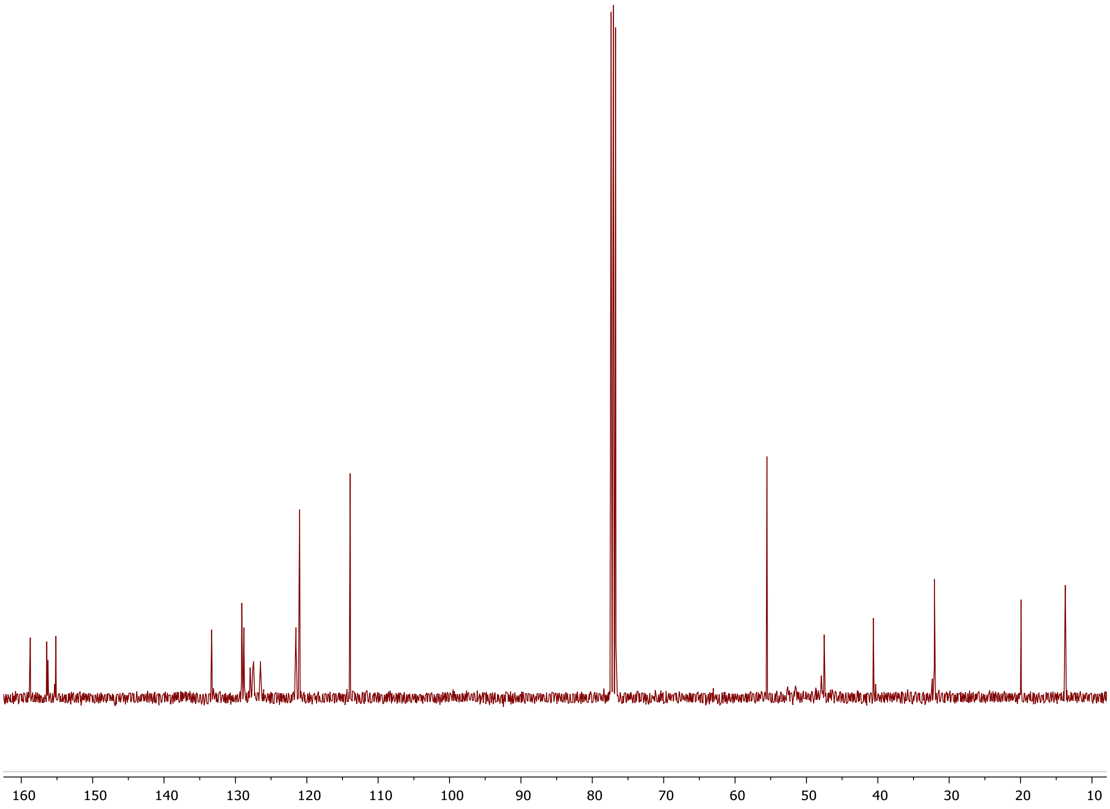


**Figure S97 – $^{13}$C NMR spectrum of 3e (101 MHz, $CDCl_3$).**

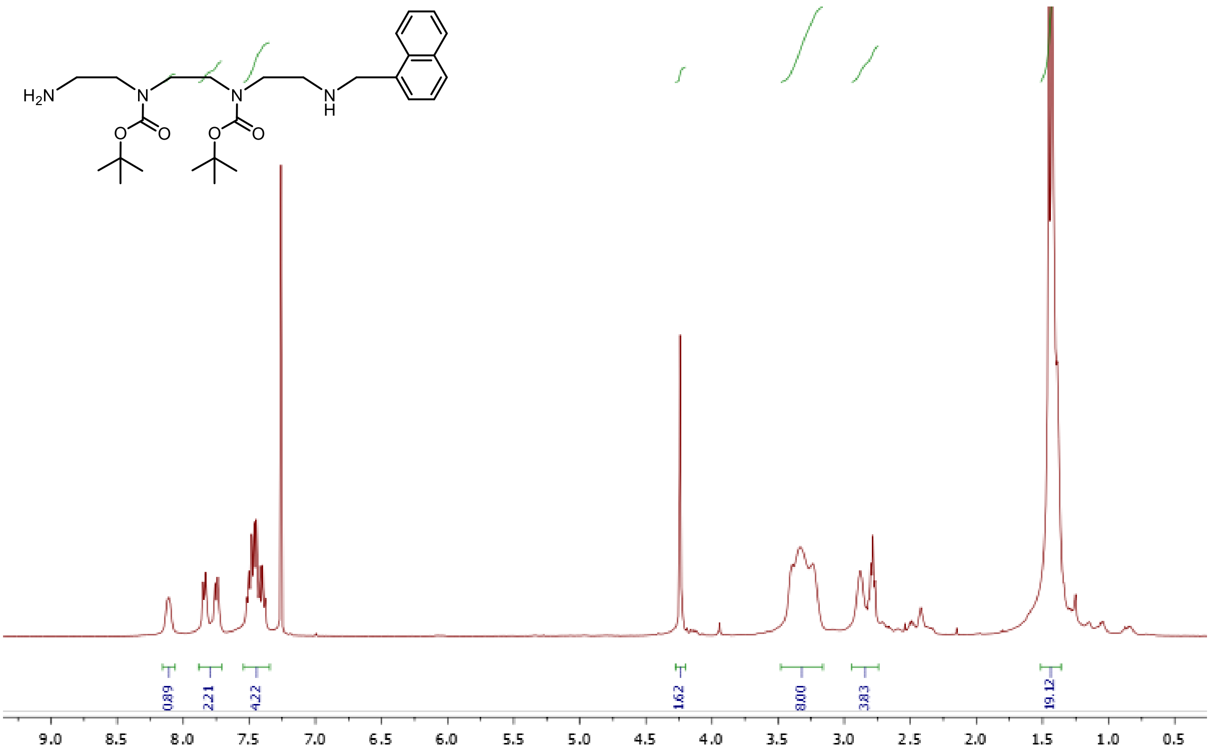


**Figure S98 – $^{1}$H NMR spectrum of 3f-2 (400 MHz, CDCl$_3$).**

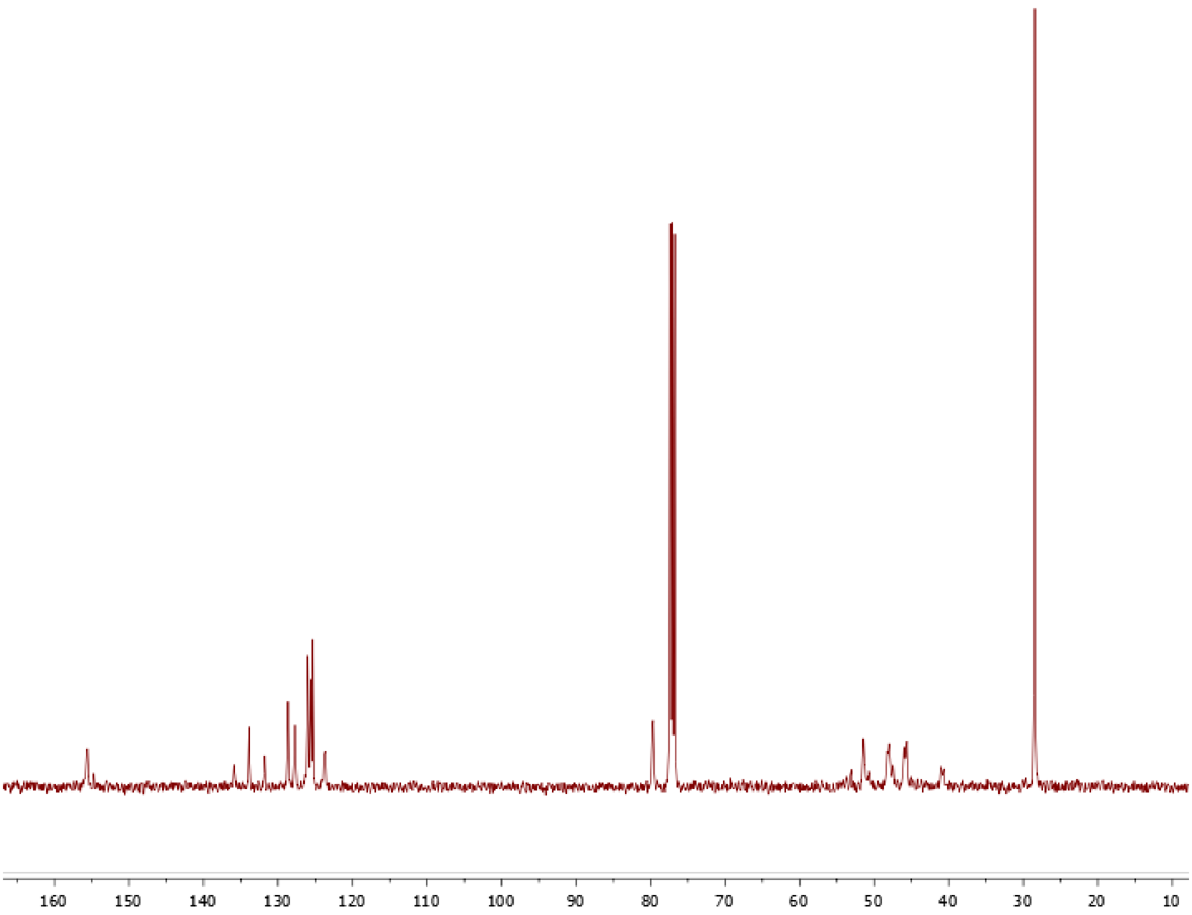


**Figure S99 – $^{13}$C NMR spectrum of 3f-2 (101 MHz, CDCl$_3$).**

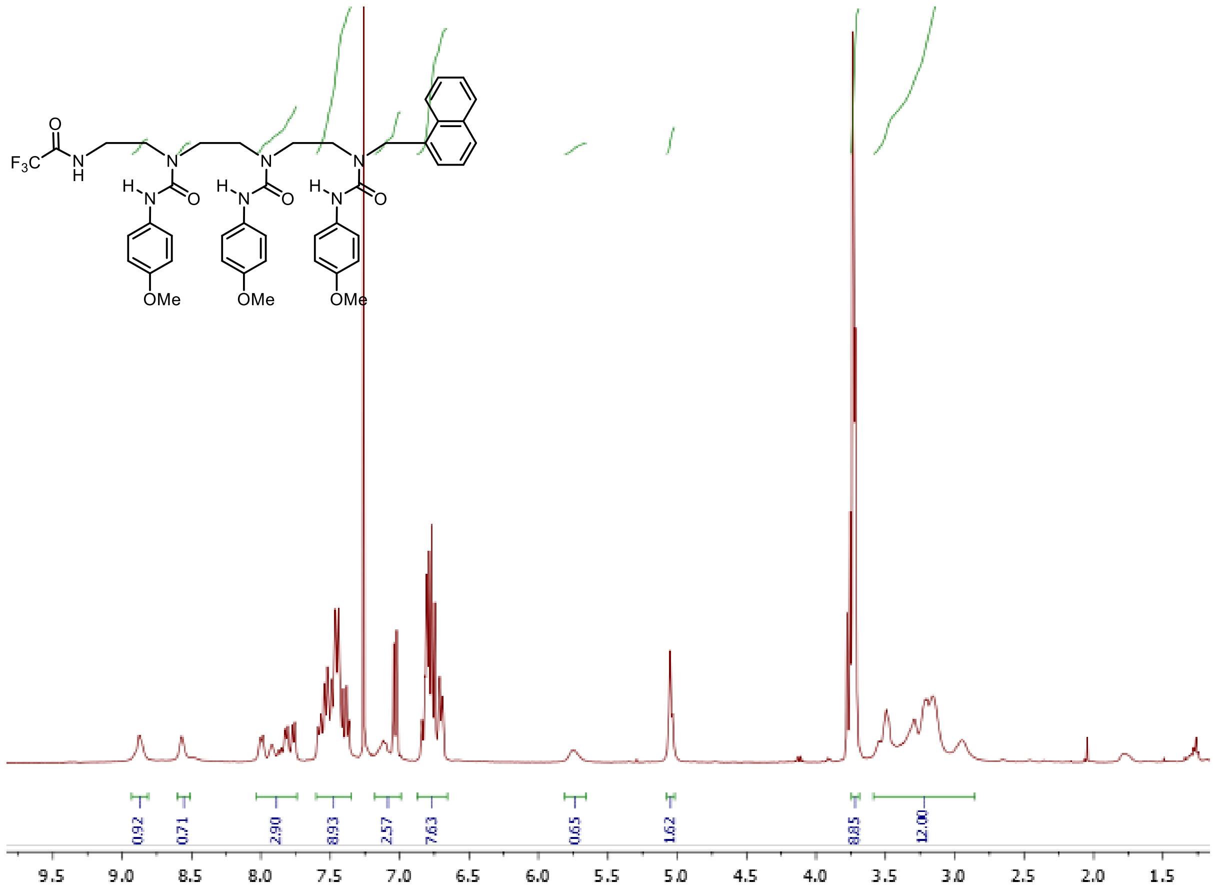

**Figure S100 – $^{1}$H NMR spectrum of 3f-3 (400 MHz, $CDCl_3$).**

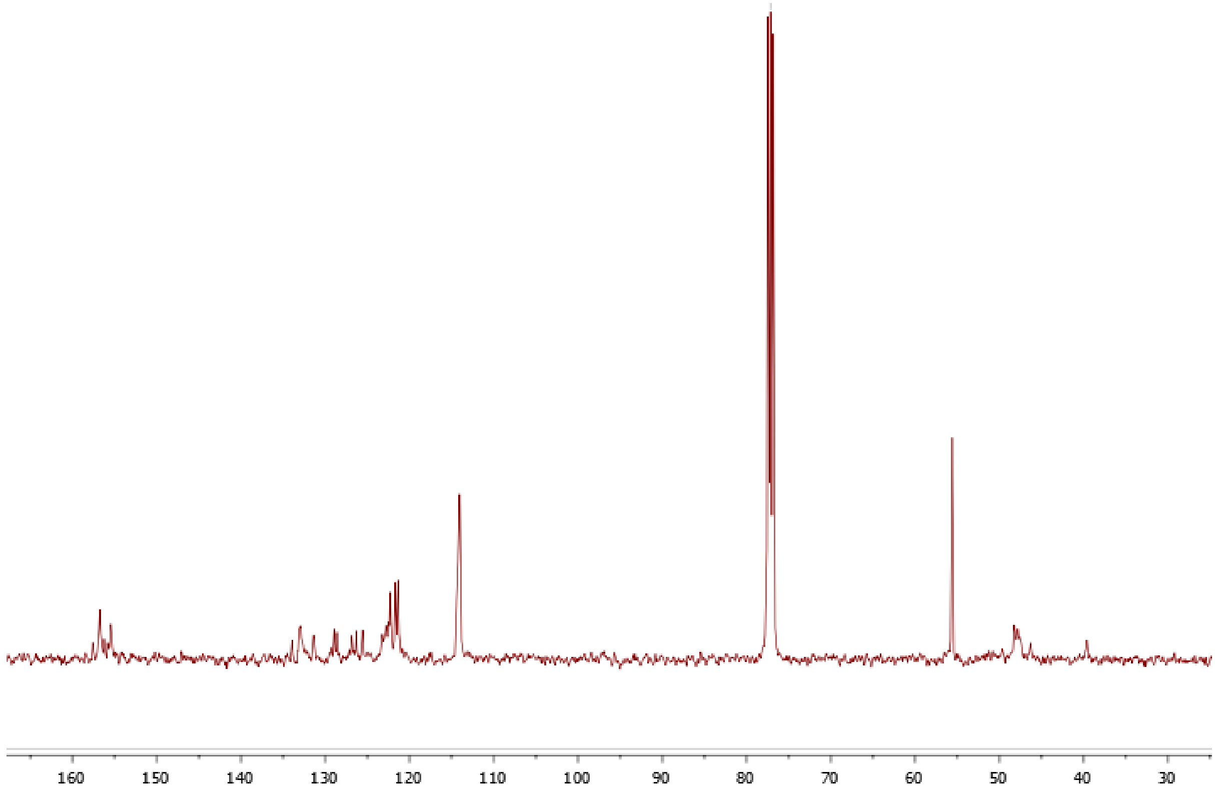

**Figure S101 – $^{13}$C NMR spectrum of 3f-3 (101 MHz, $CDCl_3$).**

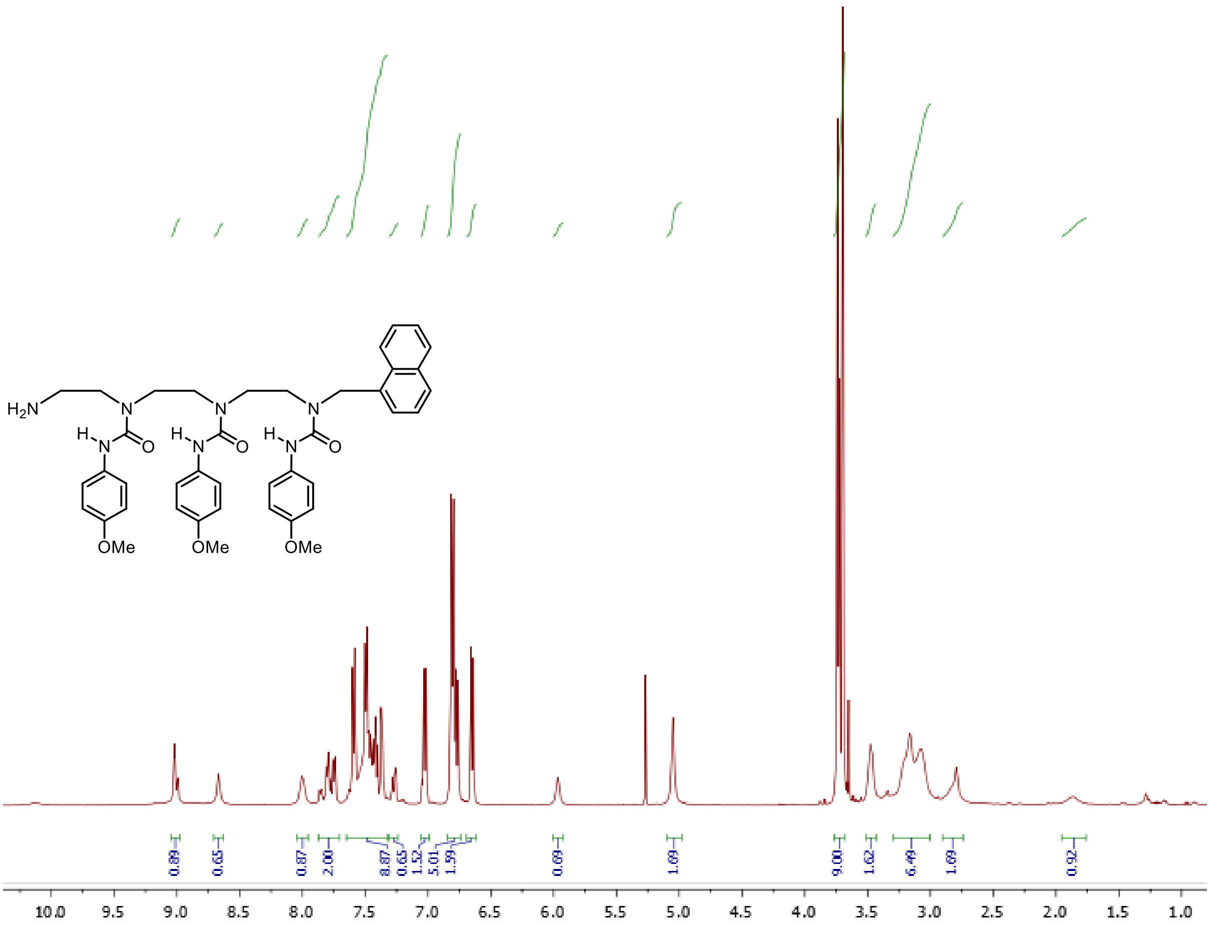


**Figure S102 – $^{1}$H NMR spectrum of 3f-4 (500 MHz, $CDCl_3$).**

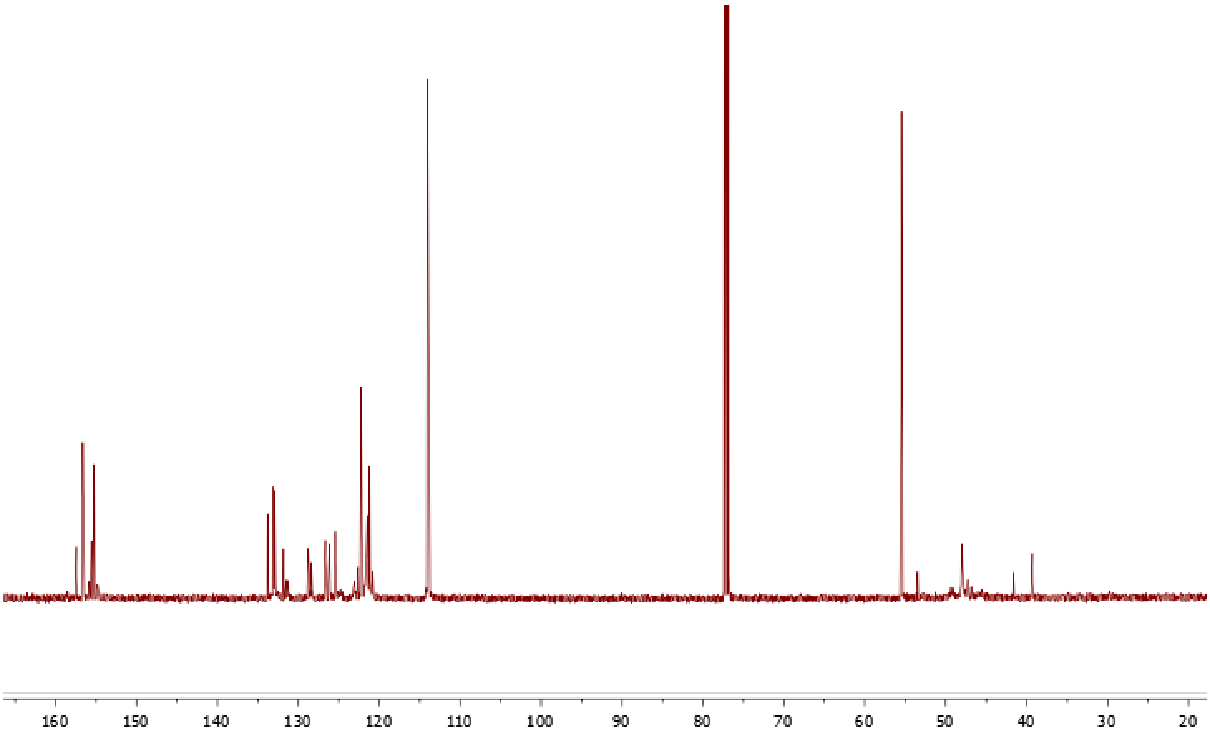


**Figure S103 – $^{13}$C NMR spectrum of 3f-4 (126 MHz, $CDCl_3$).**

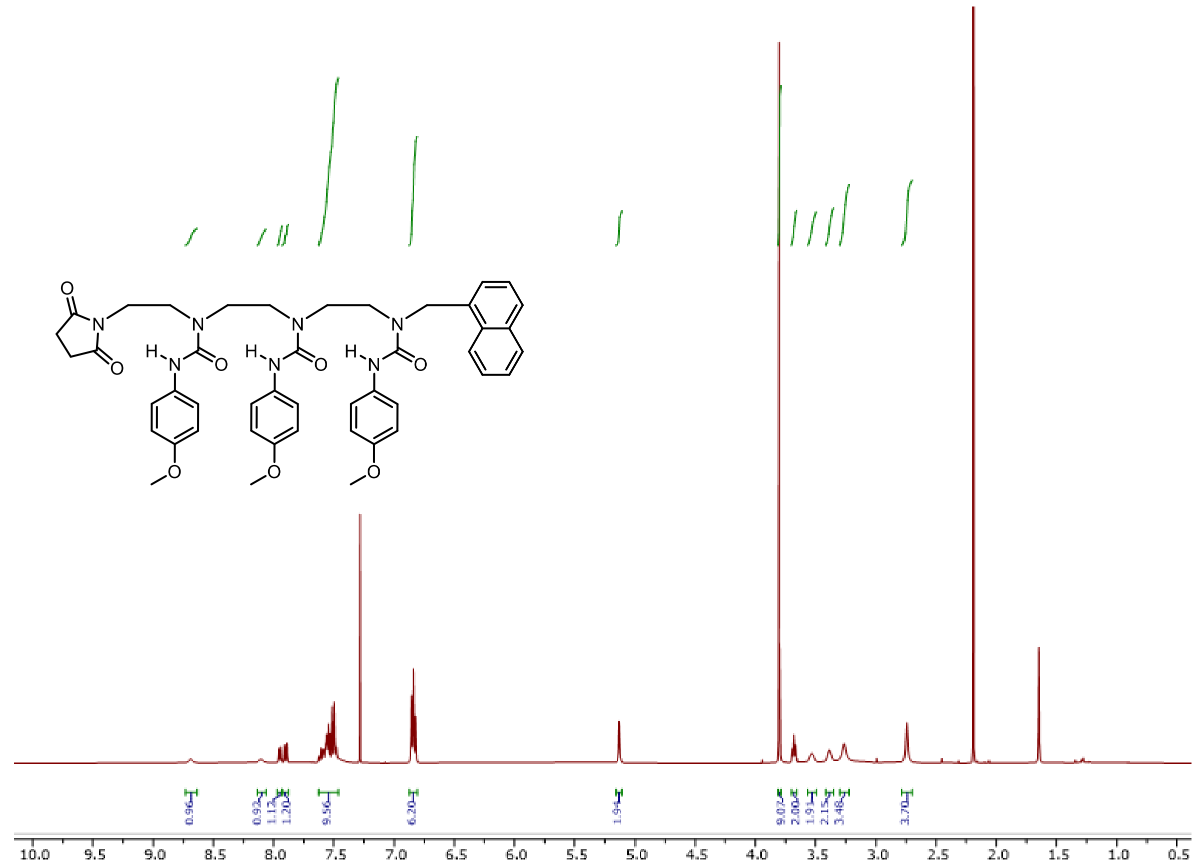


**Figure S104 – $^{1}$H NMR spectrum of 3f (500 MHz, $CDCl_3$).**

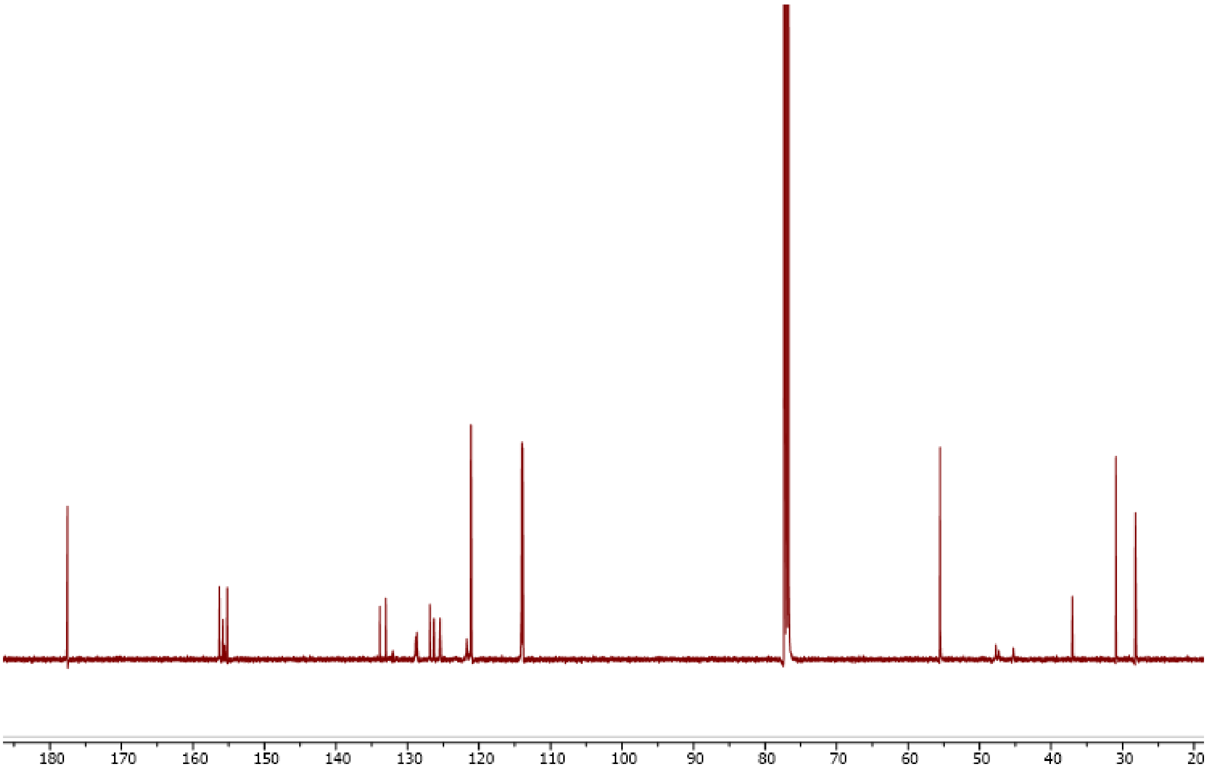


**Figure S105 – $^{13}$C NMR spectrum of 3f (126 MHz, $CDCl_3$).**

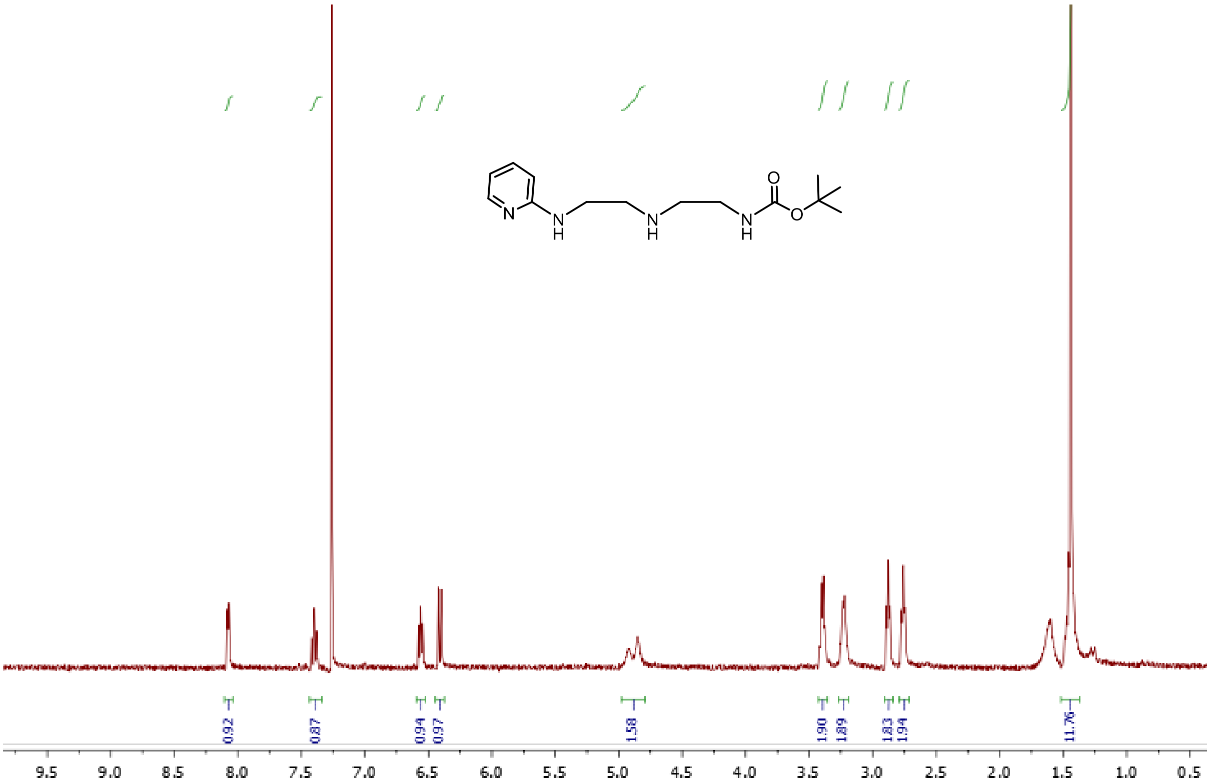


**Figure S106 – $^{1}$H NMR spectrum of 3g-2 (400 MHz, $CDCl_3$).**

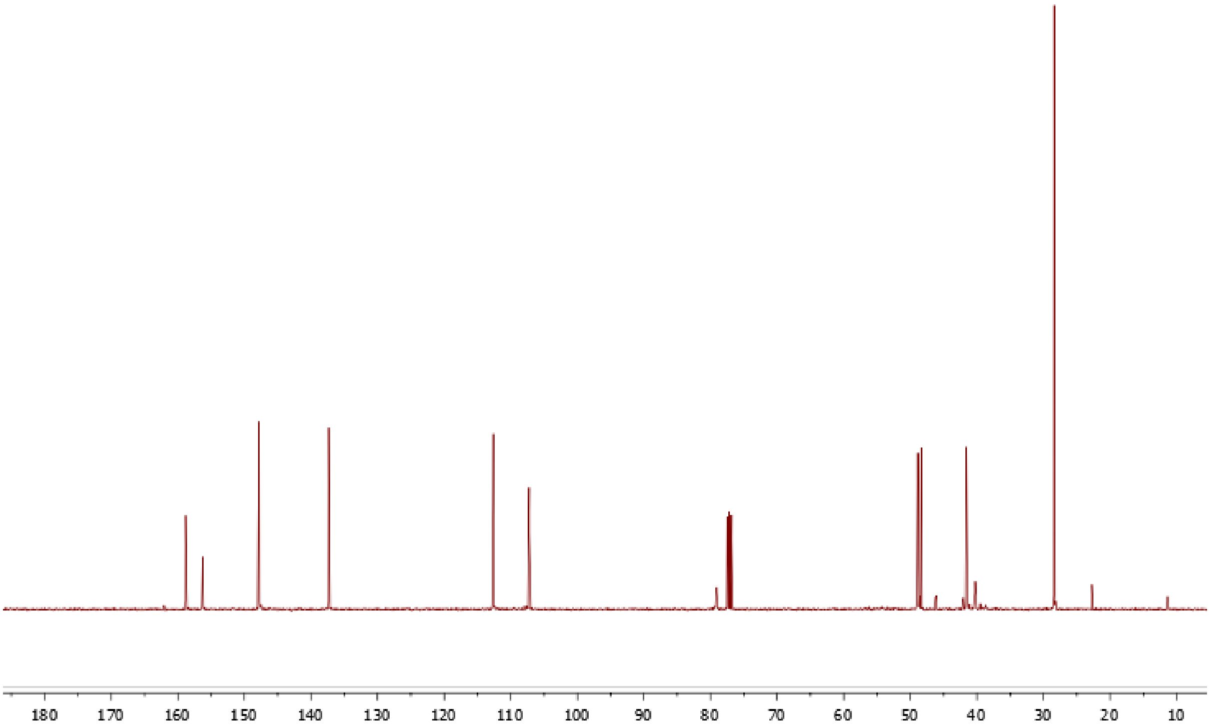


**Figure S107 – $^{13}$C NMR spectrum of 3g-2 (101 MHz, $CDCl_3$).**

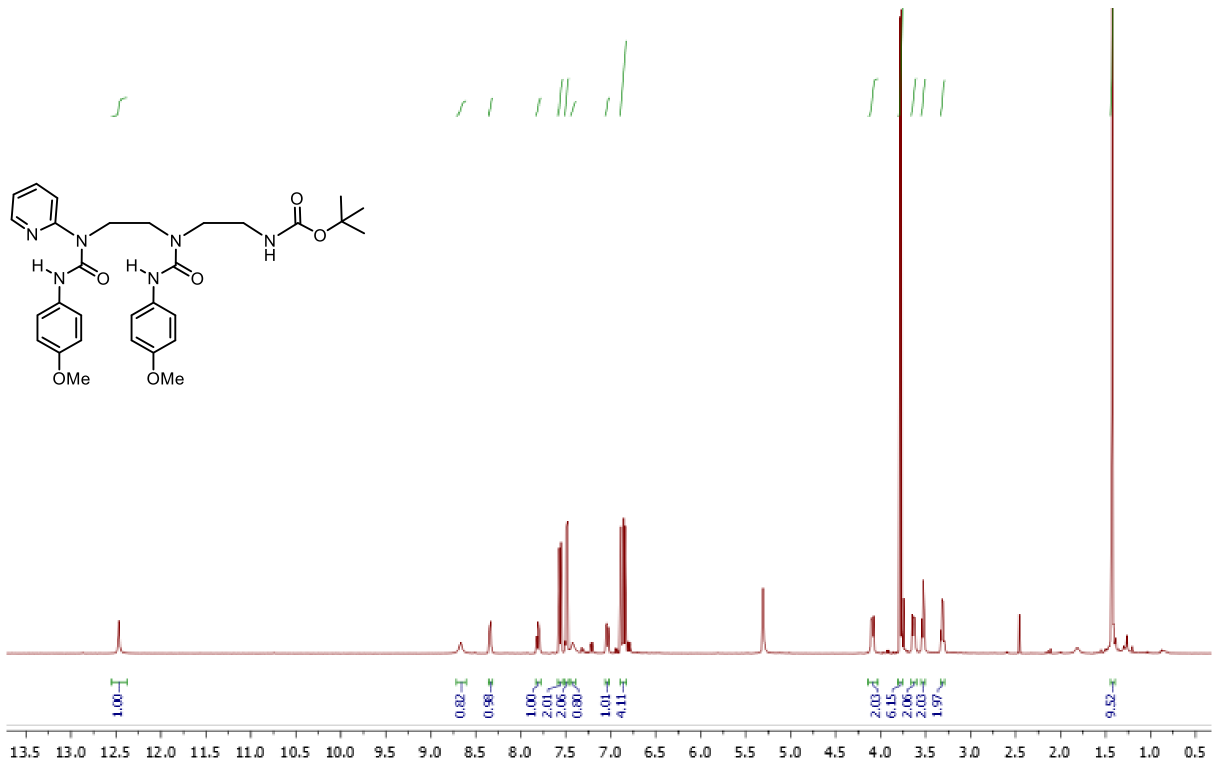

**Figure S108 – $^{1}$H NMR spectrum of 3g-3 (400 MHz, $CD_2Cl_2$).**

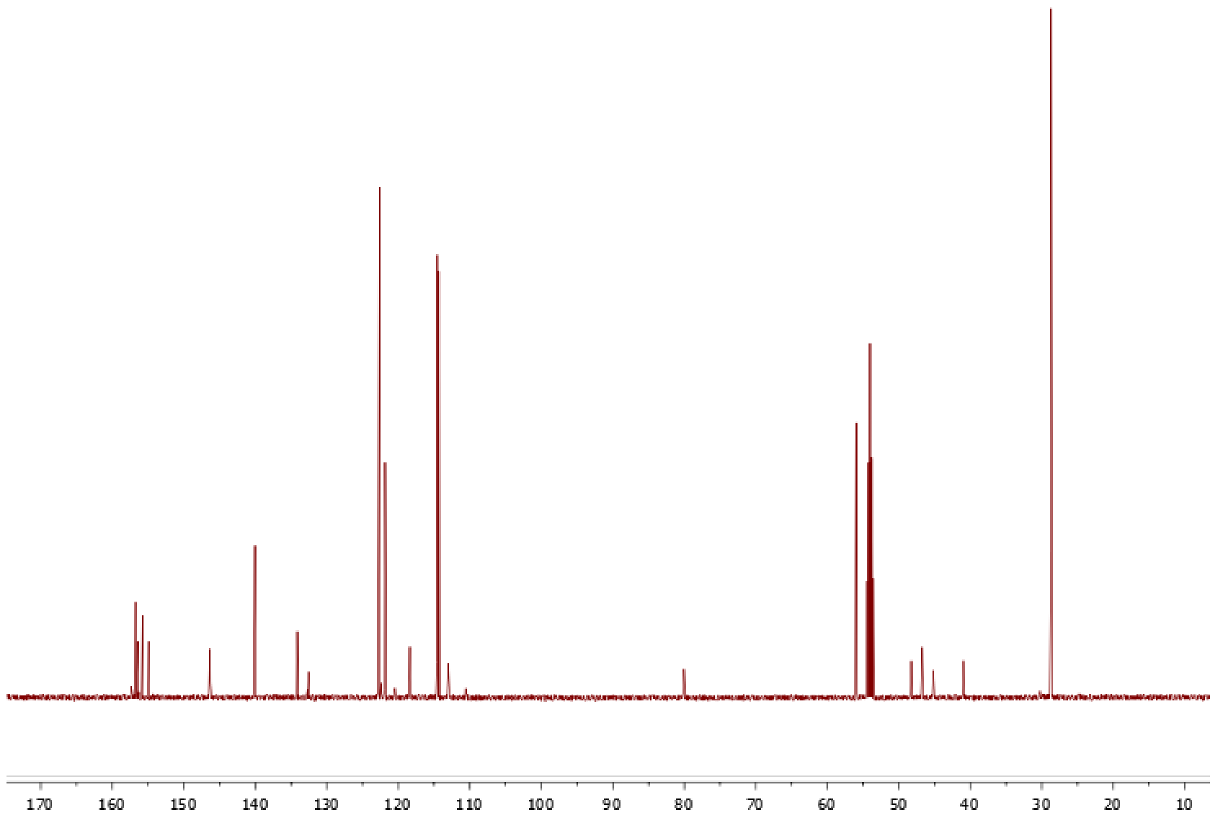

**Figure S109 – $^{13}$C NMR spectrum of 3g-3 (101 MHz, $CD_2Cl_2$).**

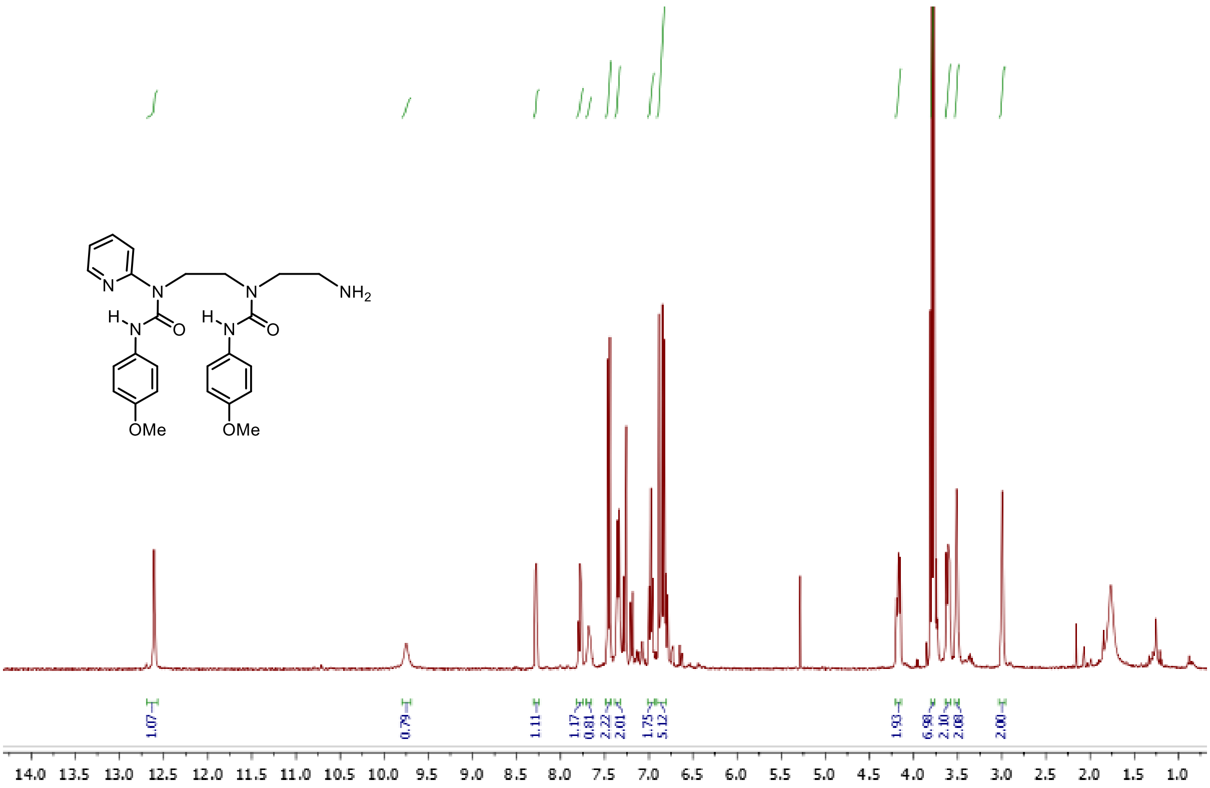


**Figure S110 – $^{1}$H NMR spectrum of 3g-4 (400 MHz, $CD_2Cl_2$).**

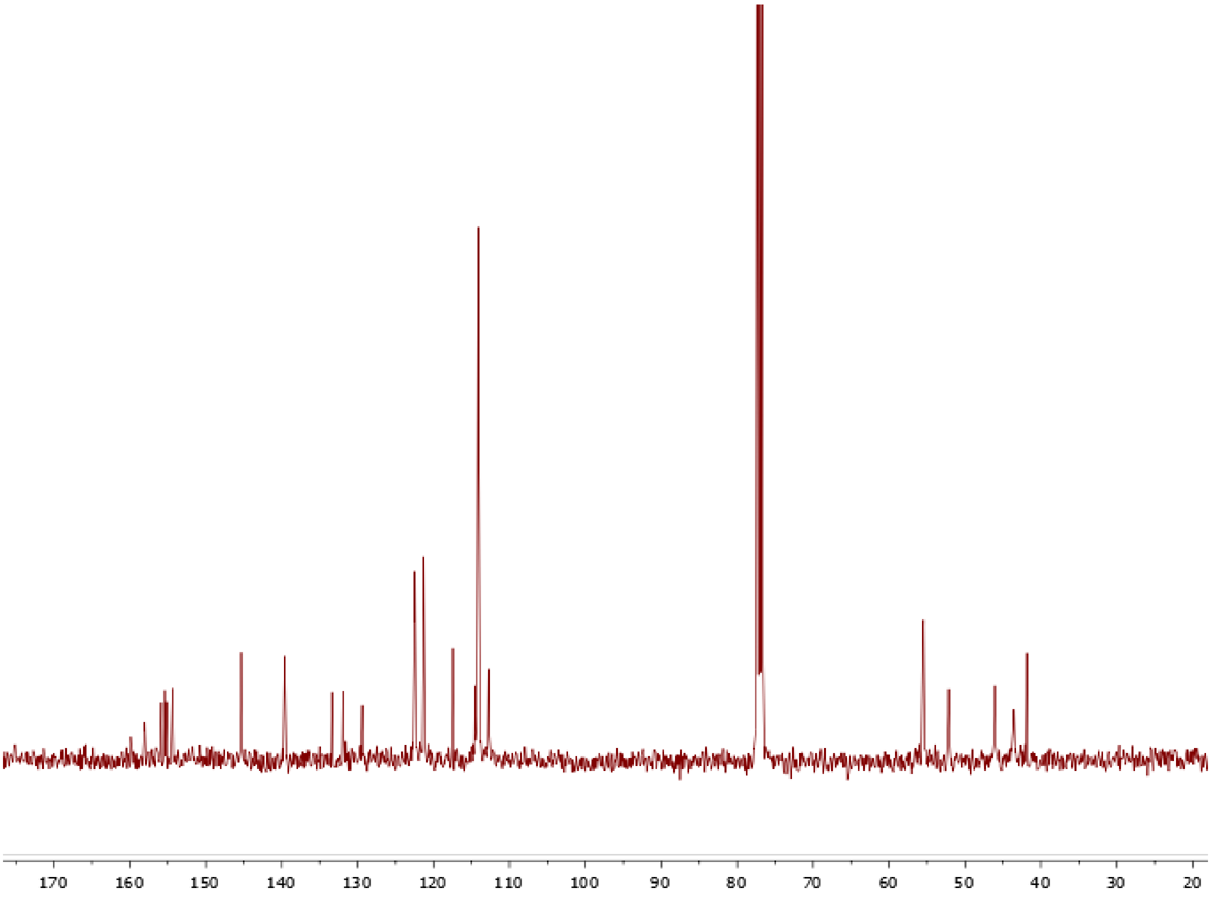


**Figure S111 – $^{13}$C NMR spectrum of 3g-4 (101 MHz, $CDCl_3$).**

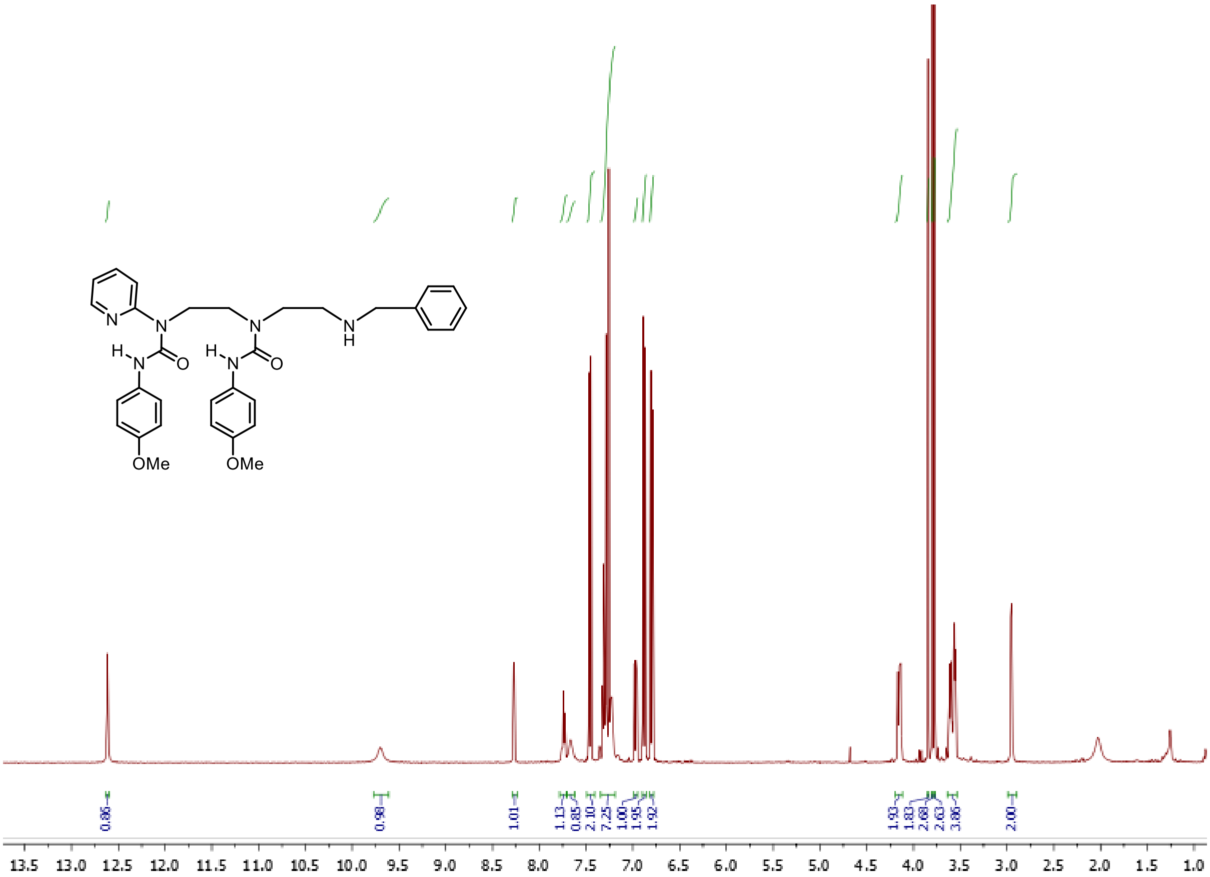


**Figure S112 – $^{1}$H NMR spectrum of 3g-5 (500 MHz, $CDCl_3$).**

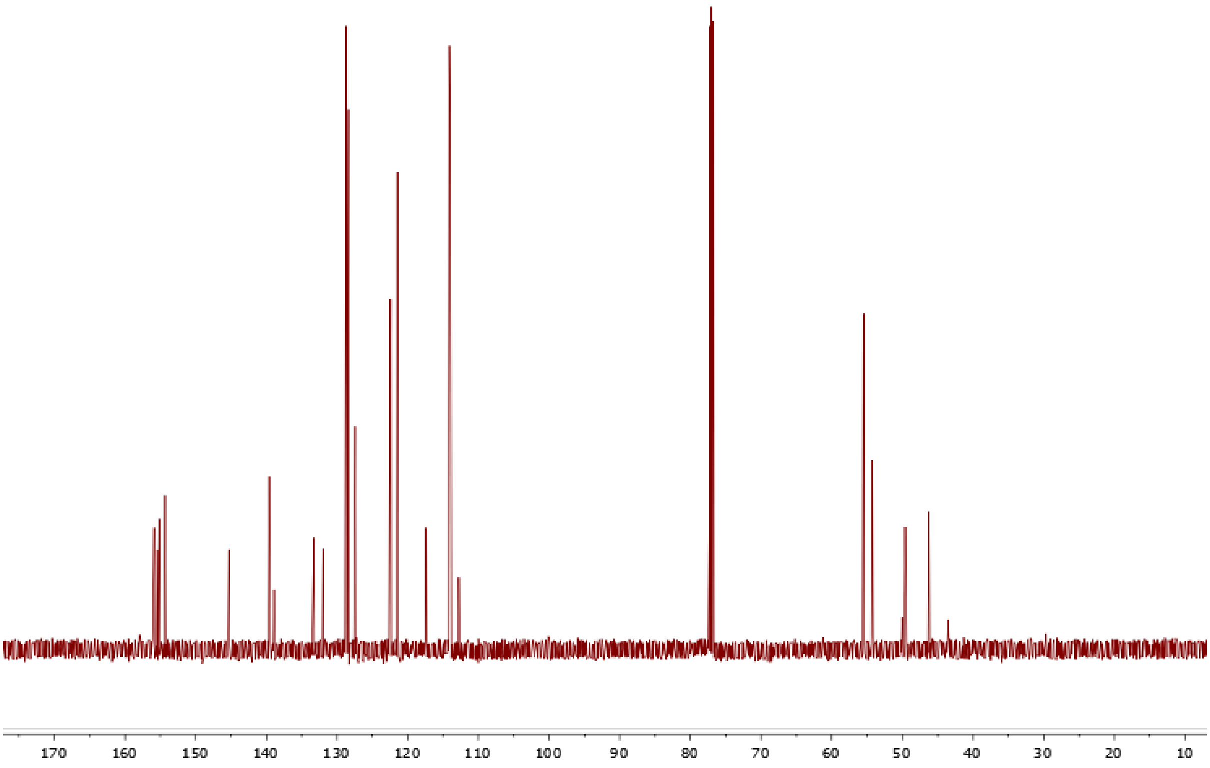


**Figure S113 – $^{13}$C NMR spectrum of 3g-5 (126 MHz, $CDCl_3$).**

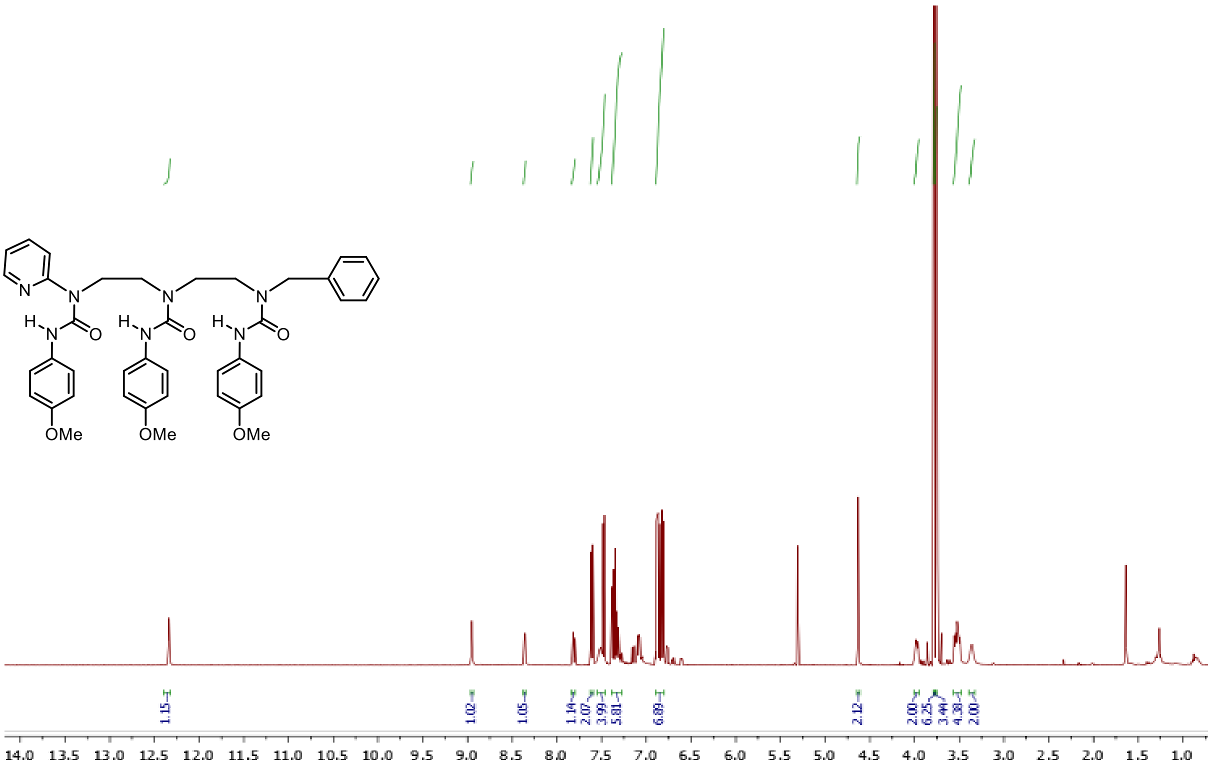


**Figure S114 – $^{1}$H NMR spectrum of 3g (500 MHz, $CD_2Cl_2$).**

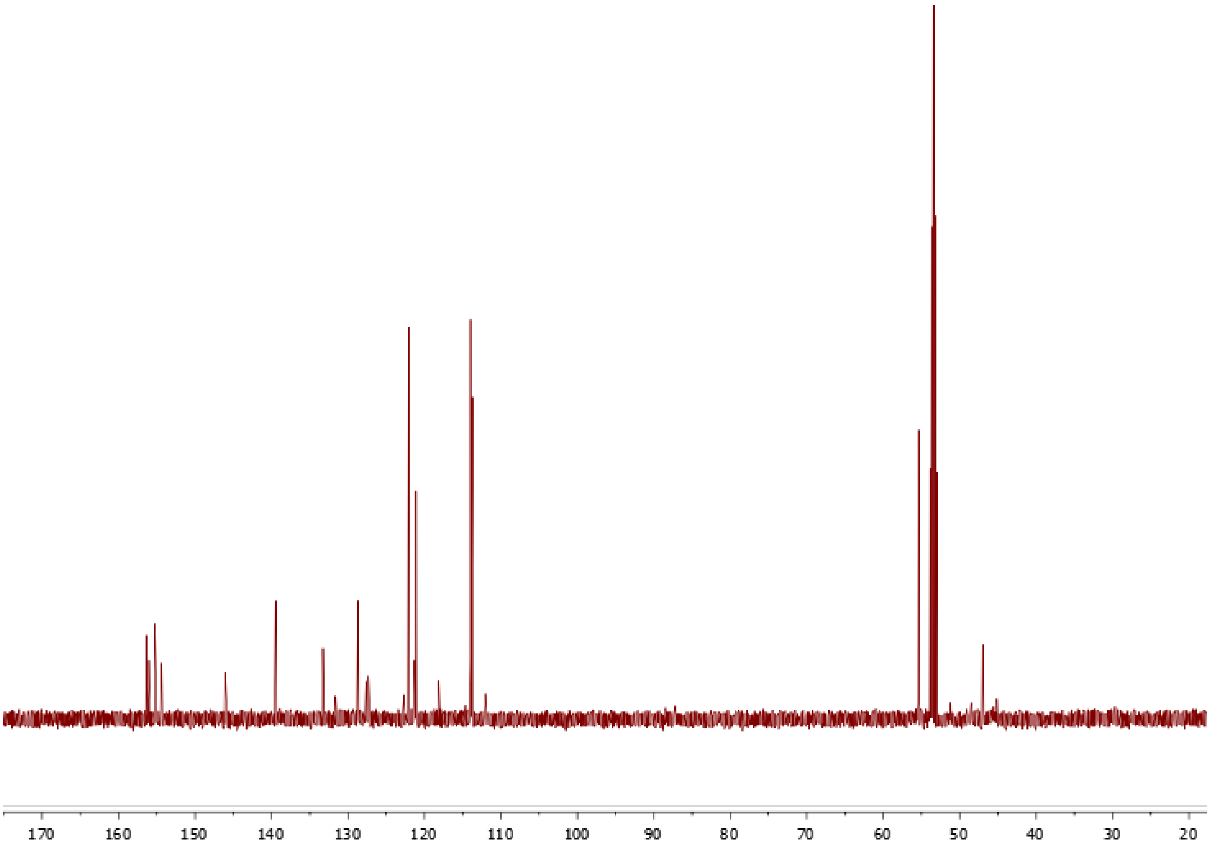


**Figure S115 – $^{13}$C NMR spectrum of 3g (126 MHz, $CD_2Cl_2$).**

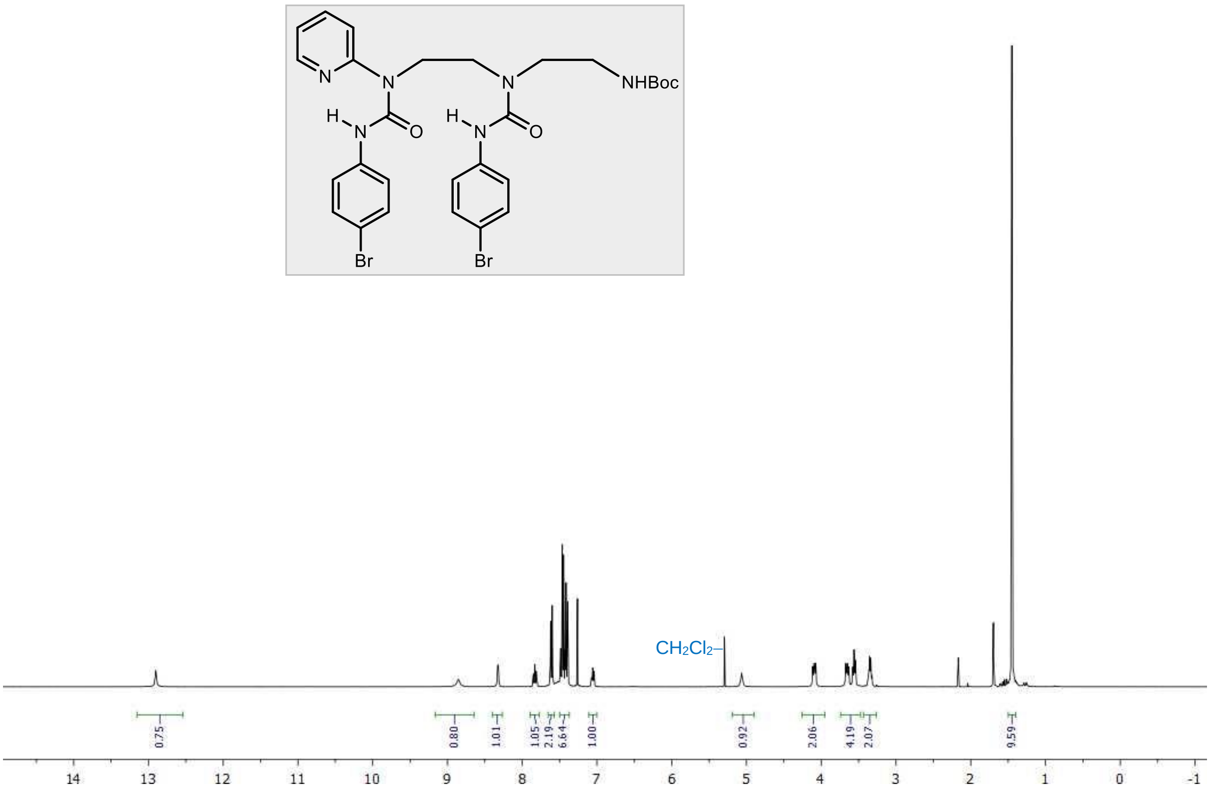

Figure S116 – $^{1}H$ NMR spectrum of 3h-1 (400 MHz, $CDCl_3$).

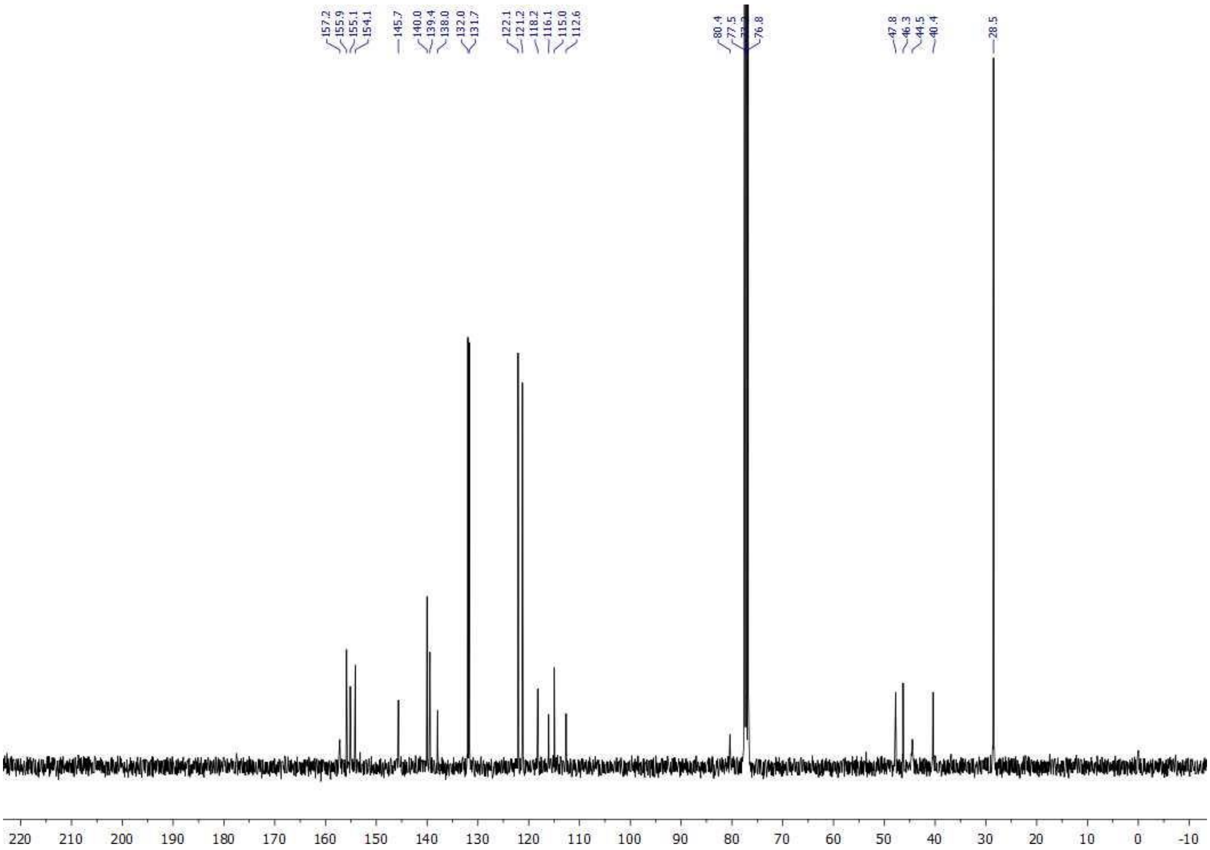

Figure S117 – $^{13}C$ NMR spectrum of 3h-1 (101 MHz, $CDCl_3$).

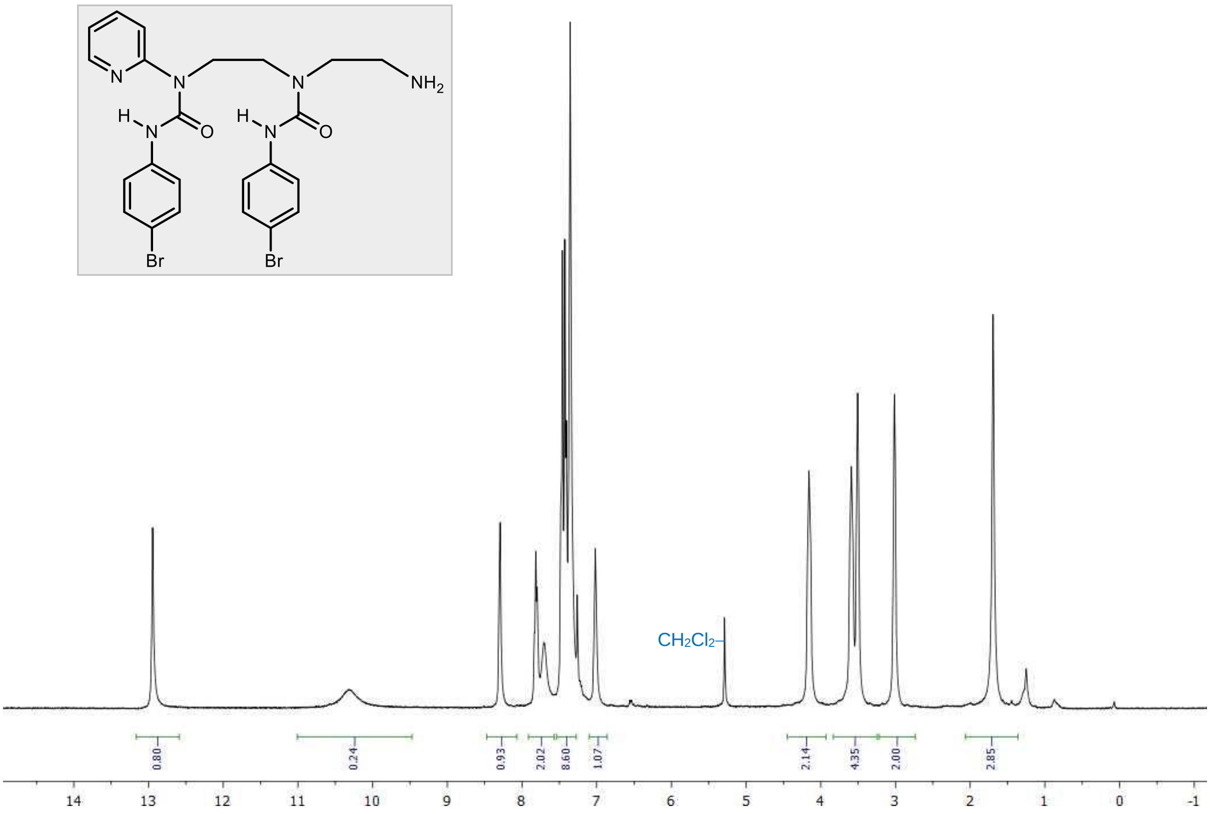


**Figure S118 – ${}^{1}$H NMR spectrum of 3h-2 (400 MHz, $CDCl_3$).**

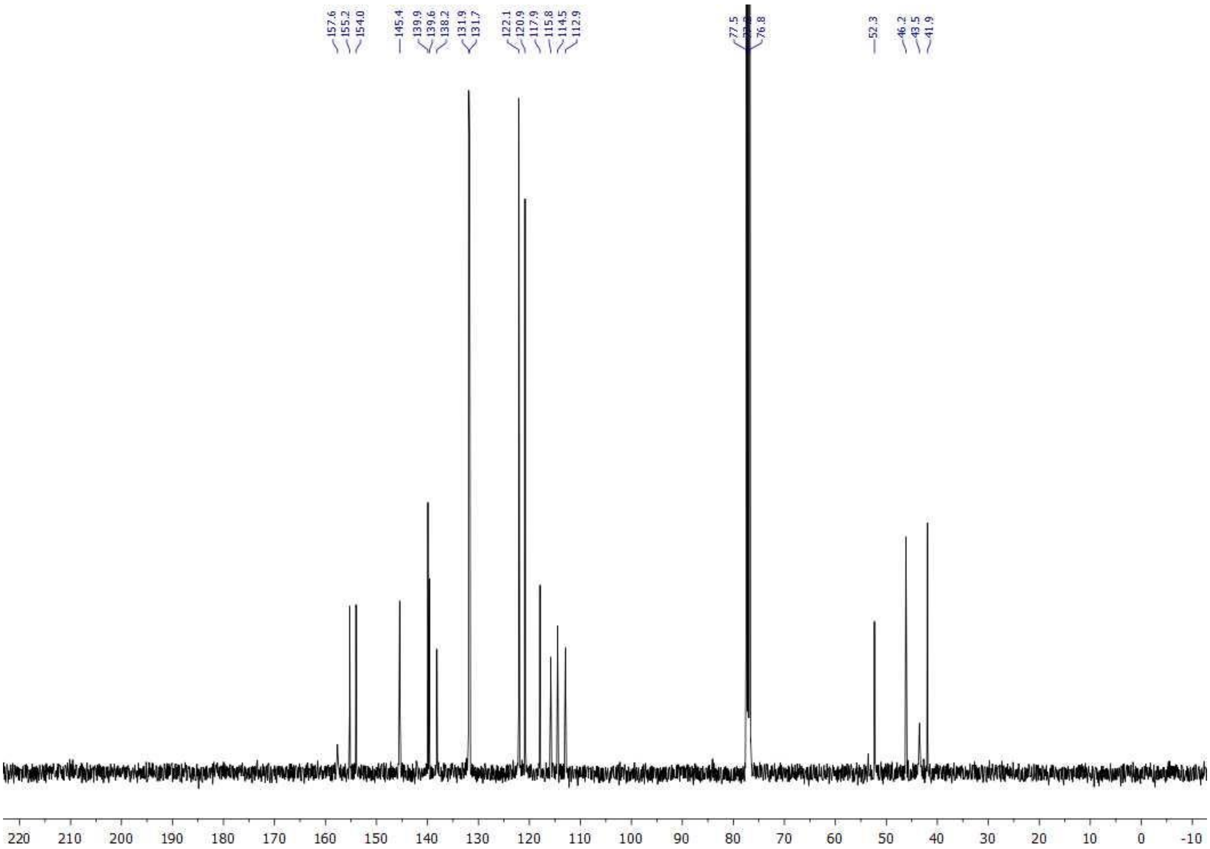


**Figure S119 – ${}^{13}$C NMR spectrum of 3h-2 (101 MHz, $CDCl_3$).**

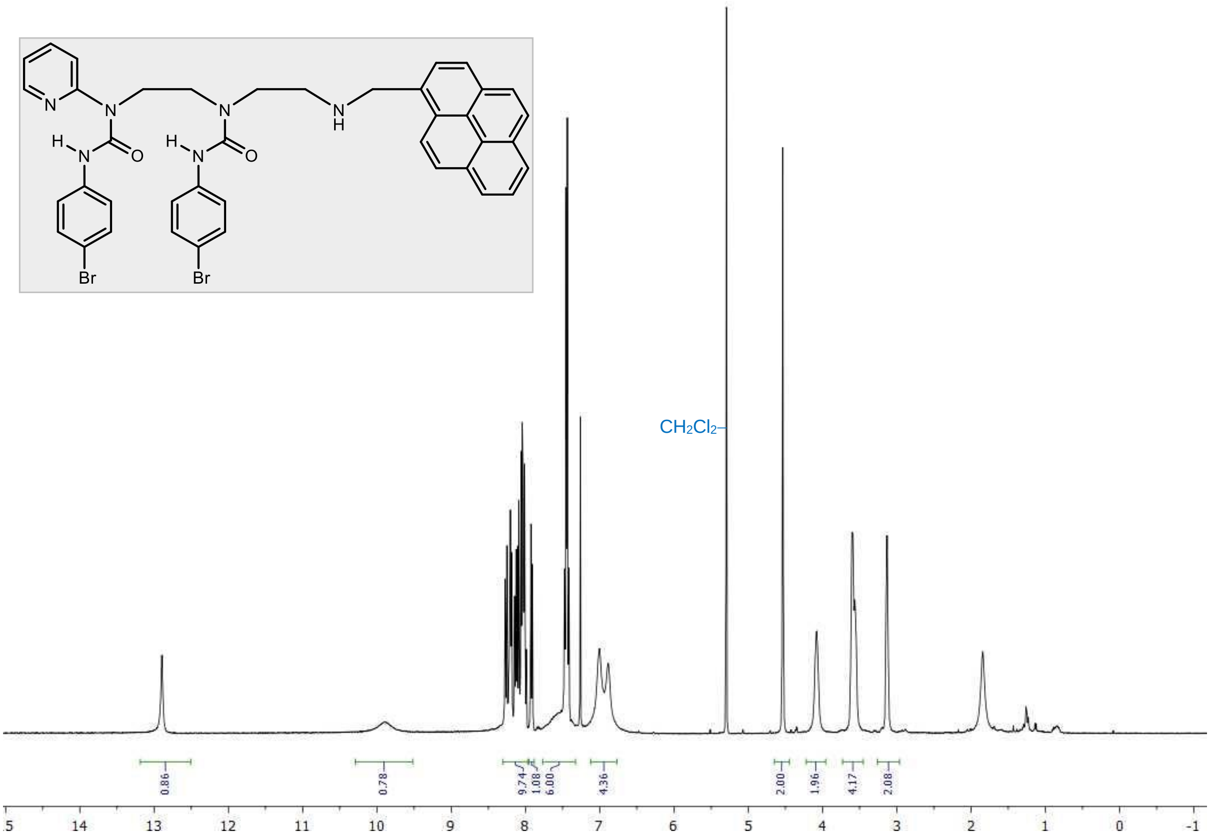


Figure S120 – $^{1}$H NMR spectrum of 3h-3 (400 MHz, $CDCl_3$).

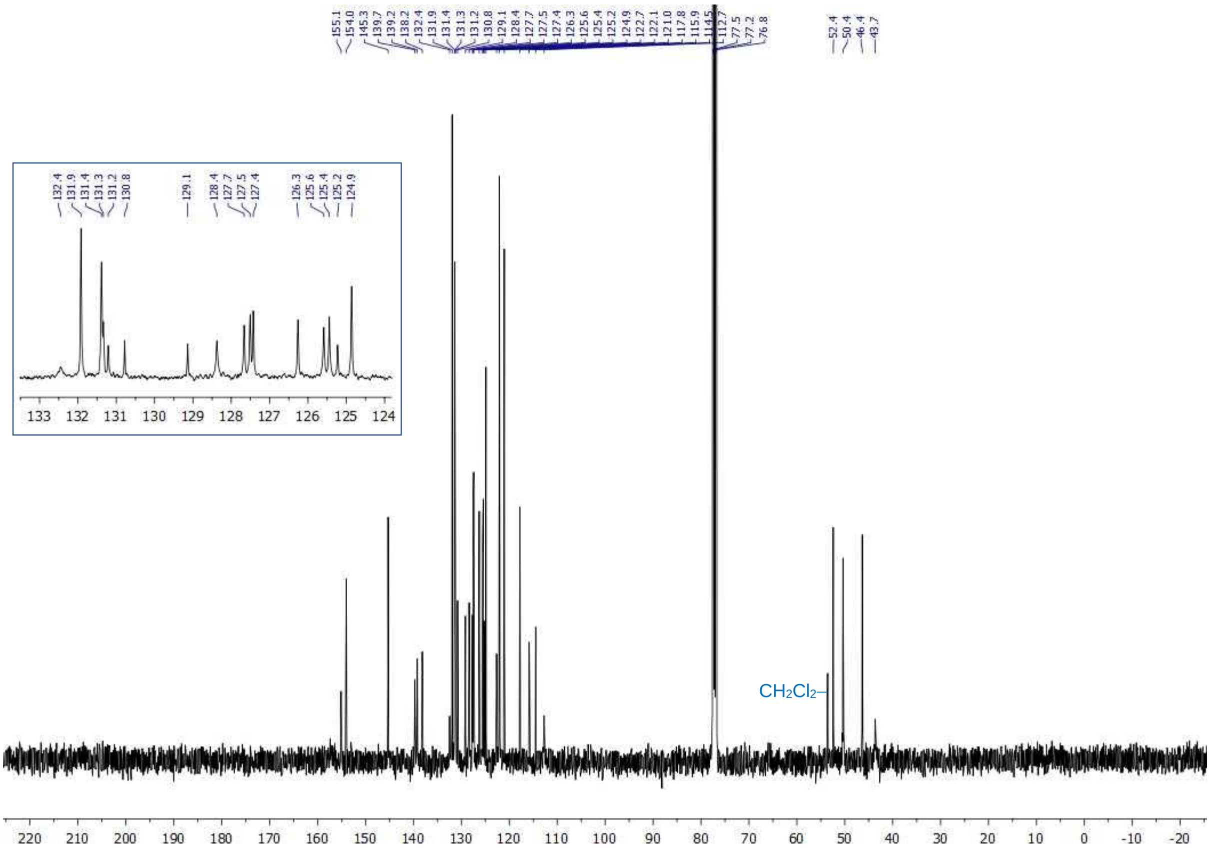


Figure S121 – $^{13}$C NMR spectrum of 3h-3 (101 MHz, $CDCl_3$).

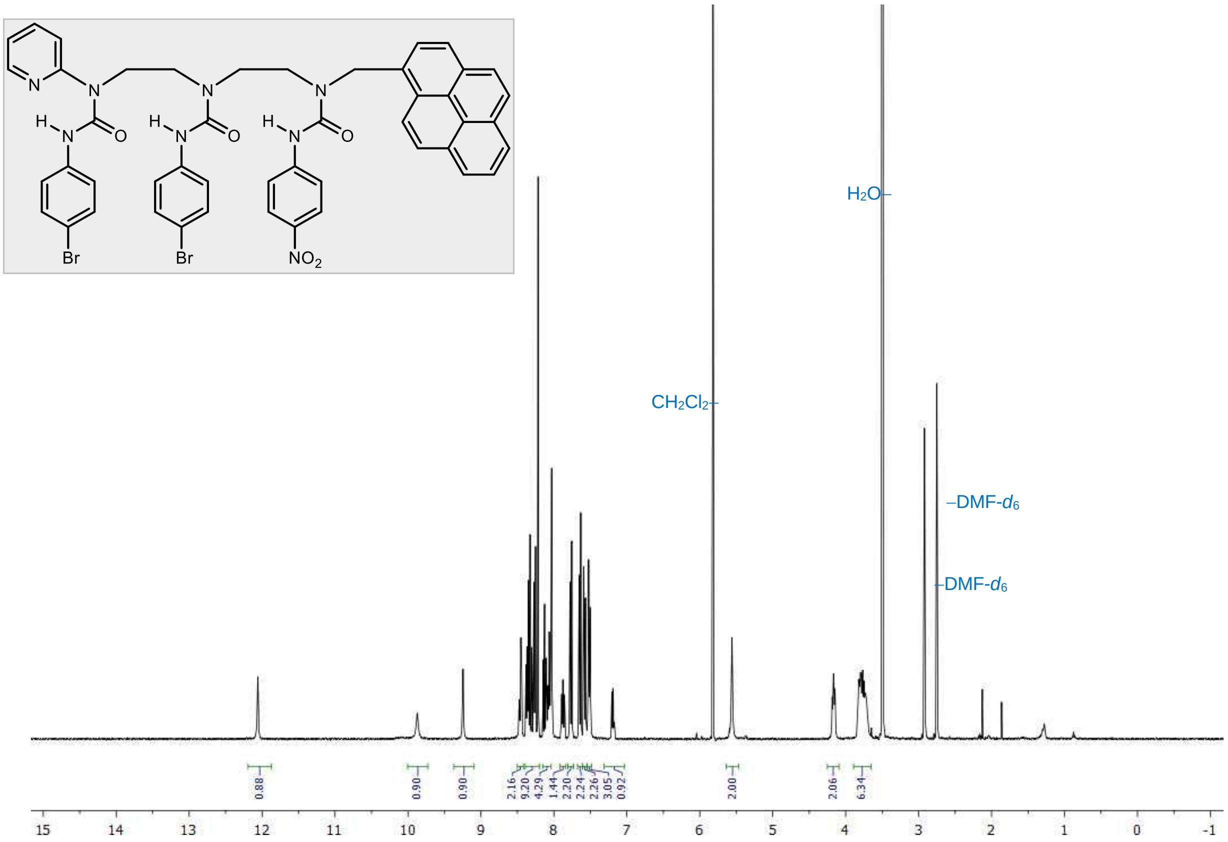


**Figure S122 – $^{1}$H NMR spectrum of 3h (400 MHz, DMF-$d_7$).**

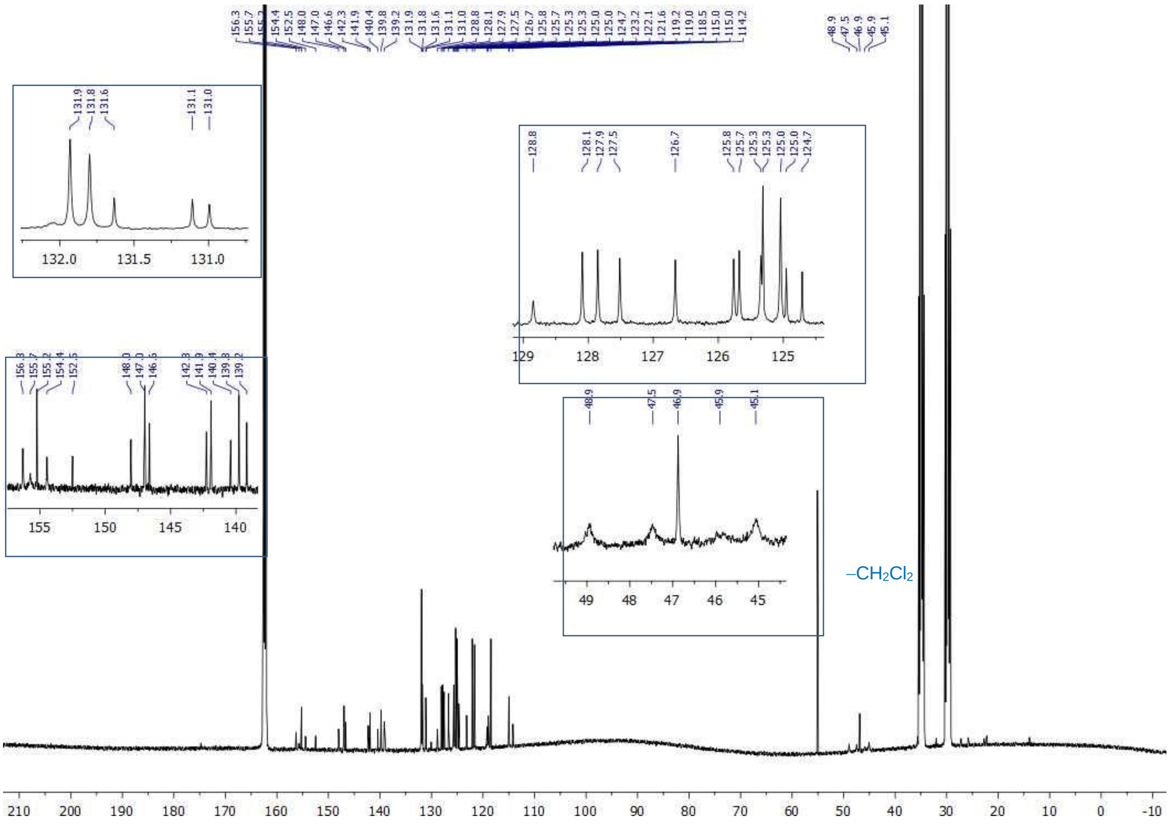


**Figure S123 – $^{13}$C NMR spectrum of 3h (101 MHz, DMF-$d_7$).**

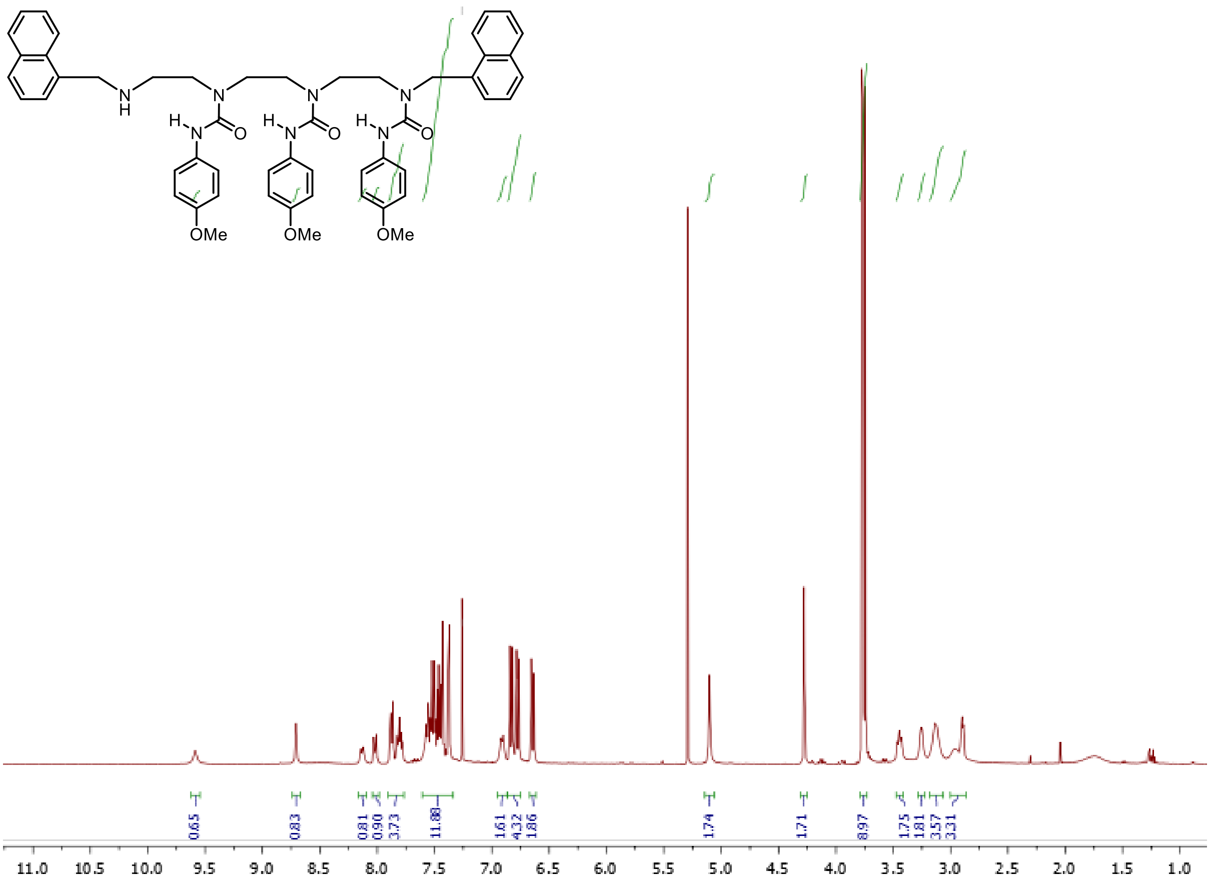


**Figure S124 – $^{1}$H NMR spectrum of 5a-1 (400 MHz, $CDCl_3$).**

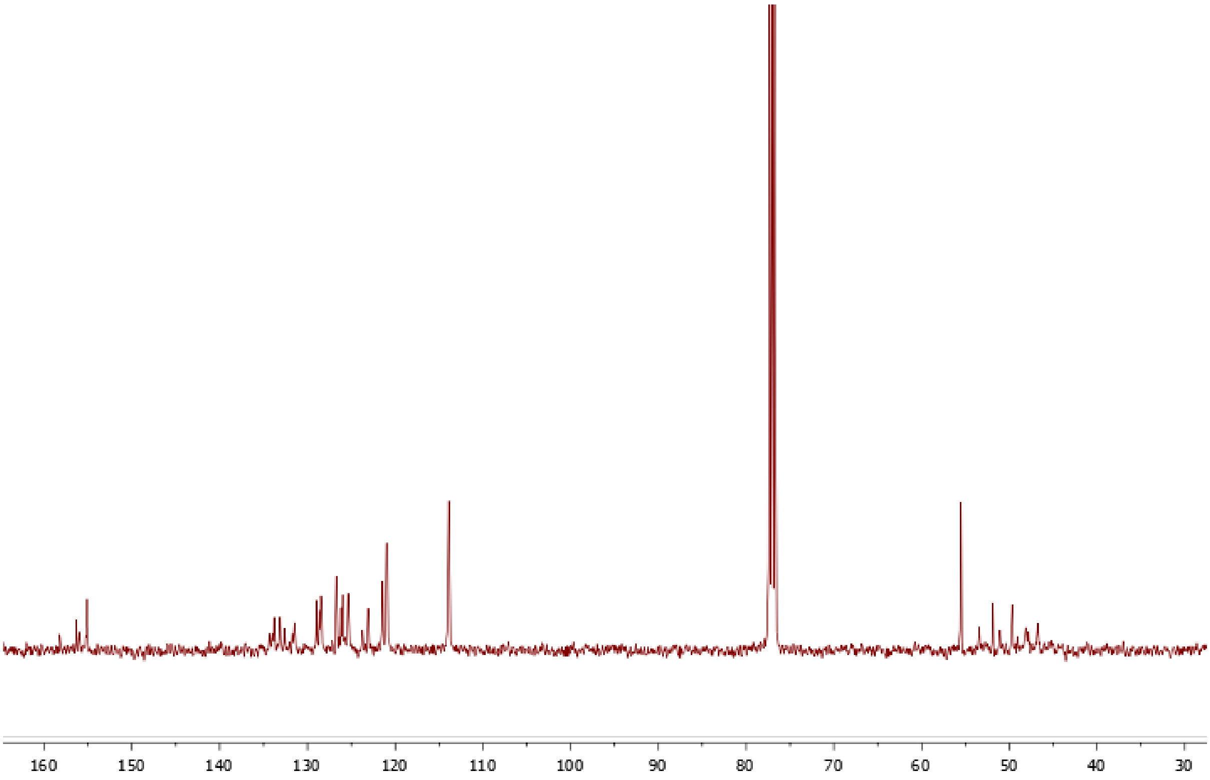


**Figure S125 – $^{13}$C NMR spectrum of 5a-1 (101 MHz, $CDCl_3$).**

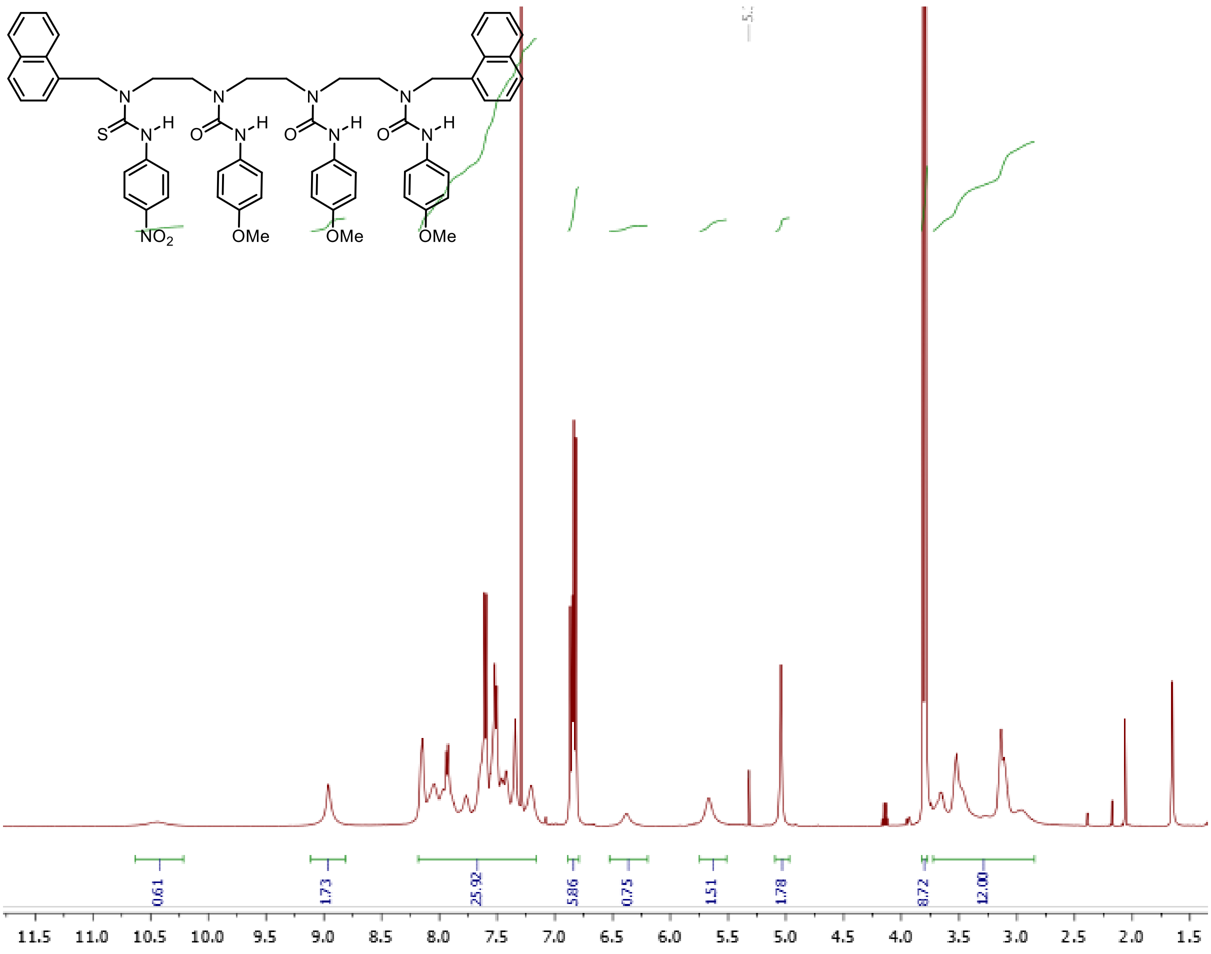


**Figure S126 – $^{1}$H NMR spectrum of 5a (500 MHz, $CDCl_3$).**

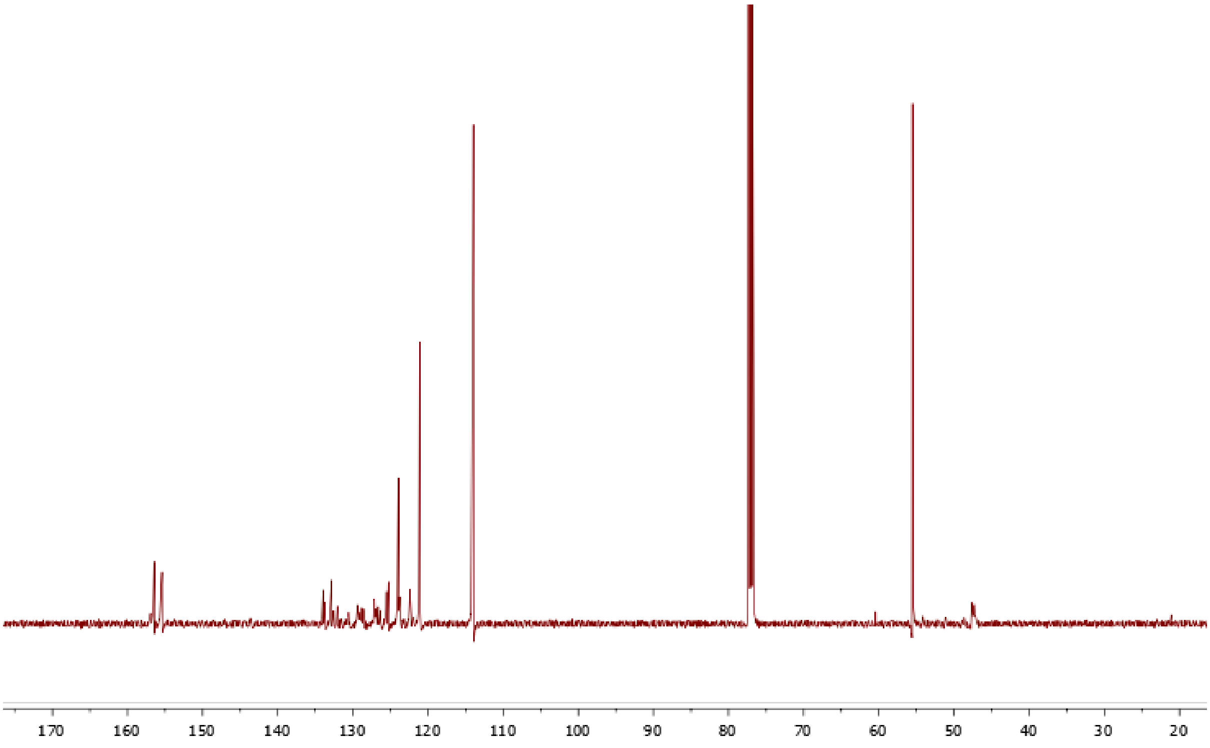


**Figure S127 – $^{13}$C NMR spectrum of 5a (126 MHz, $CDCl_3$).**

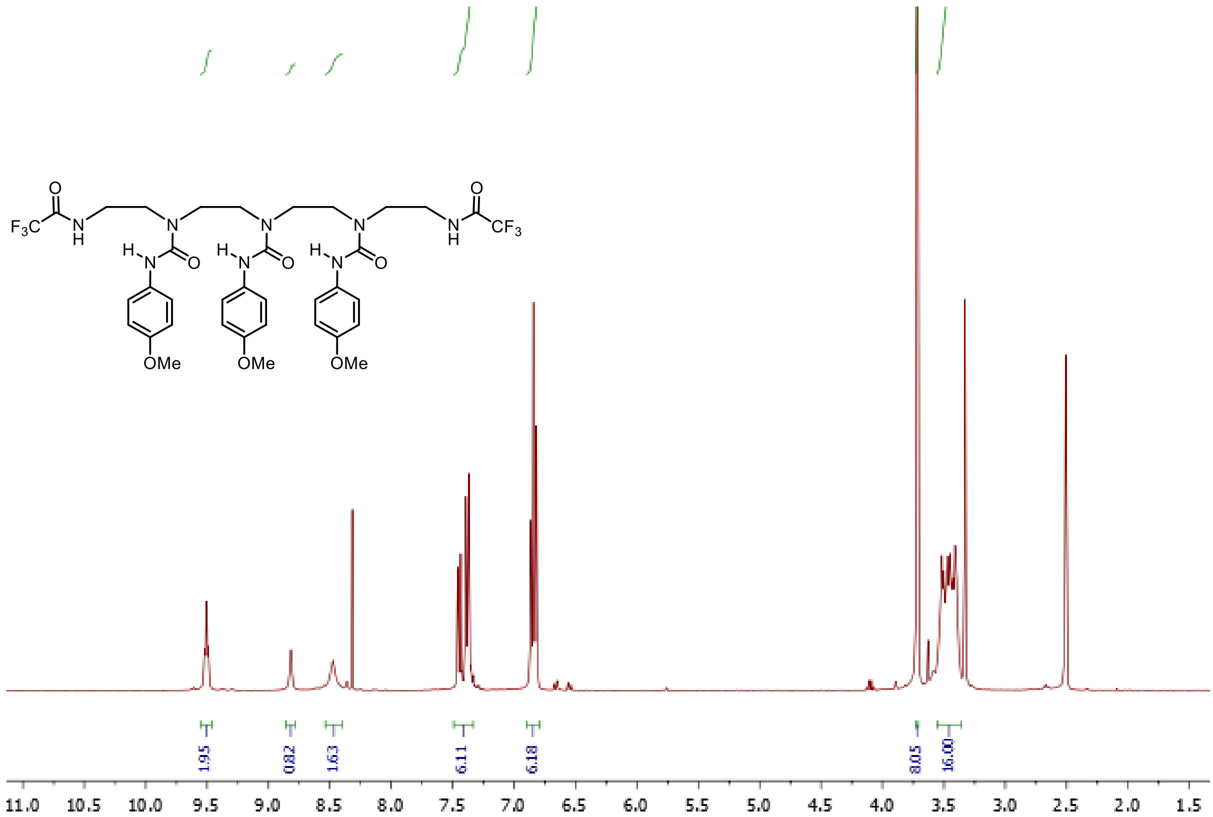


**Figure S128 – $^{1}H$ NMR spectrum of 5b-1 (400 MHz, $CDCl_3$).**

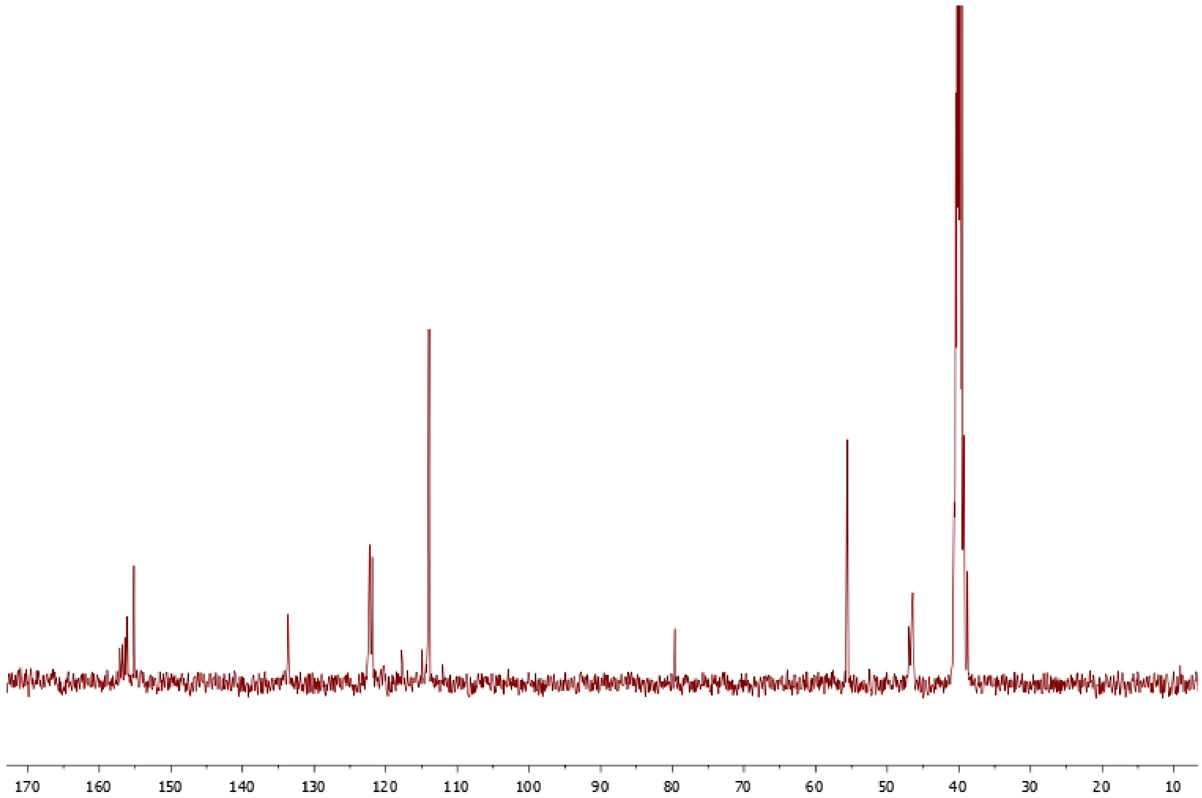


**Figure S129 – $^{13}C$ NMR spectrum of 5b-1 (101 MHz, $CDCl_3$).**

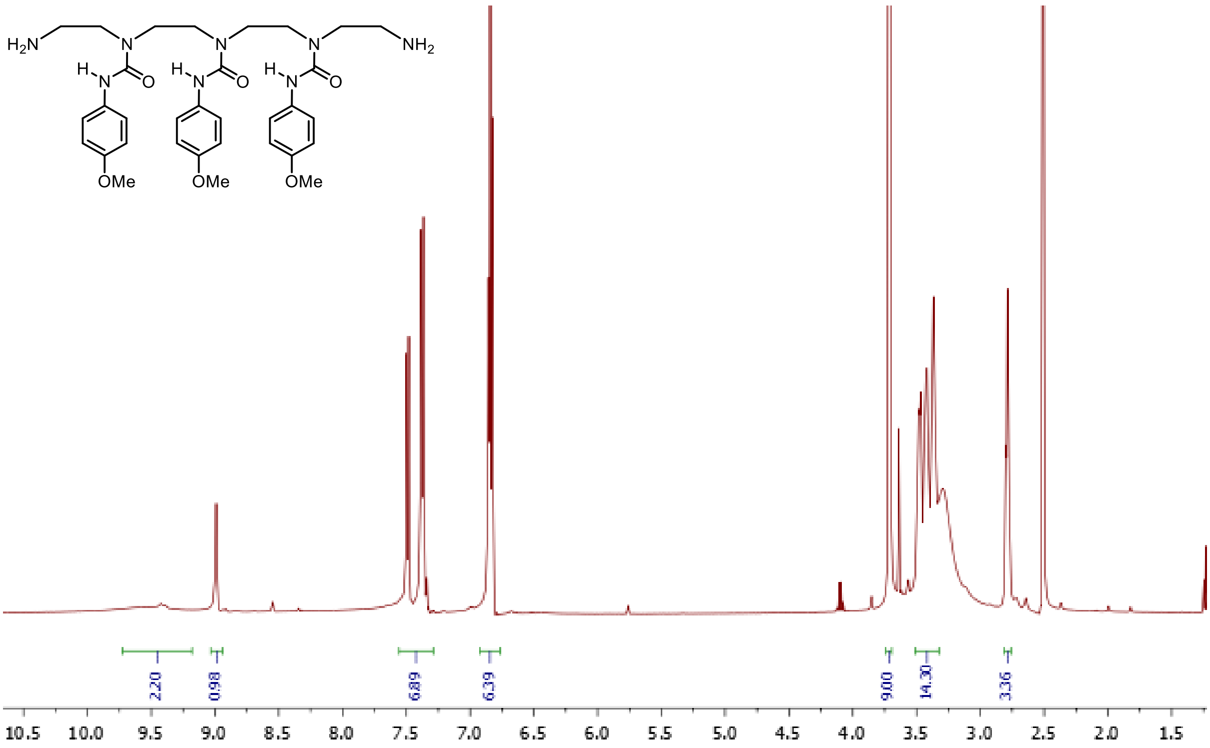


**Figure S130 – $^{1}$H NMR spectrum of 5b-2 (500 MHz, $(CD_3)_2SO$).**

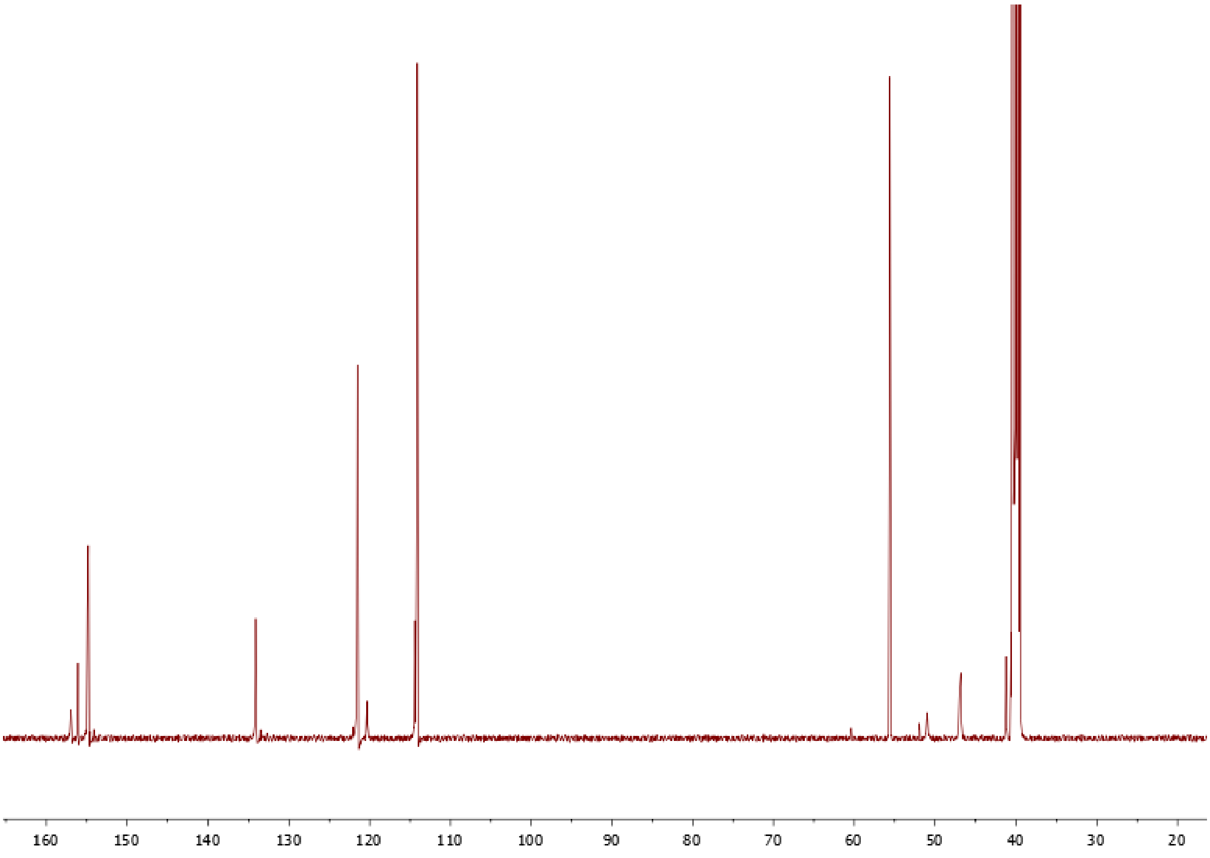


**Figure S131 – $^{13}$C NMR spectrum of 5b-2 (126 MHz, $(CD_3)_2SO$).**

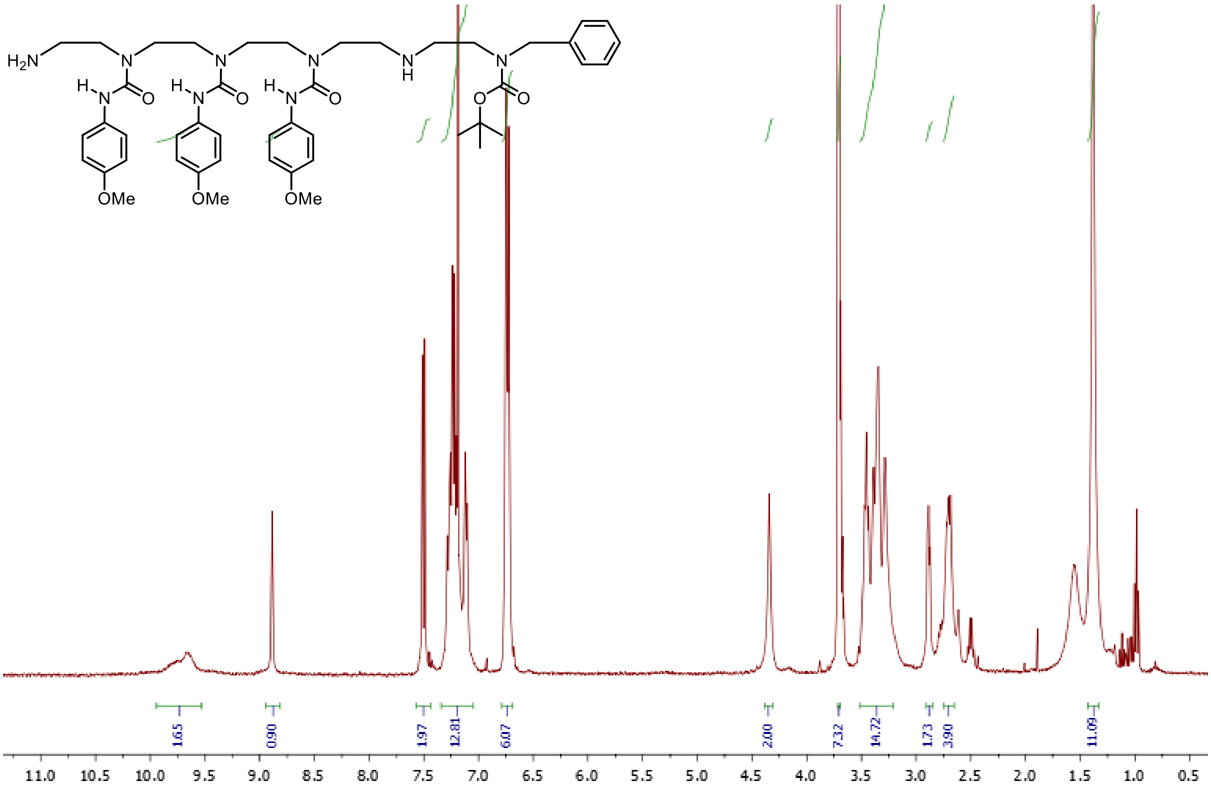


**Figure S132 – $^{1}$H NMR spectrum of 5b-5 (400 MHz, $CDCl_3$).**

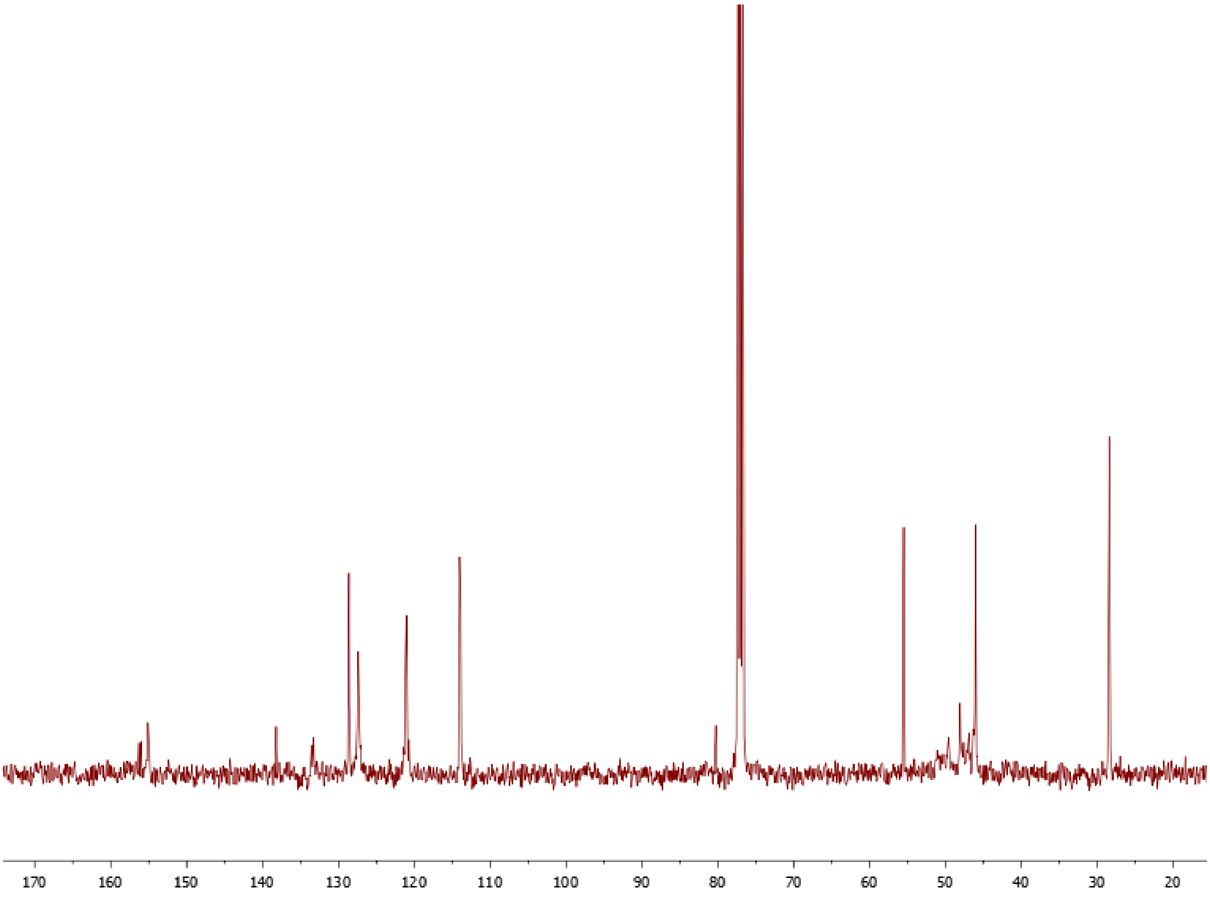


**Figure S133 – $^{13}$C NMR spectrum of 5b-5 (101 MHz, $CDCl_3$).**

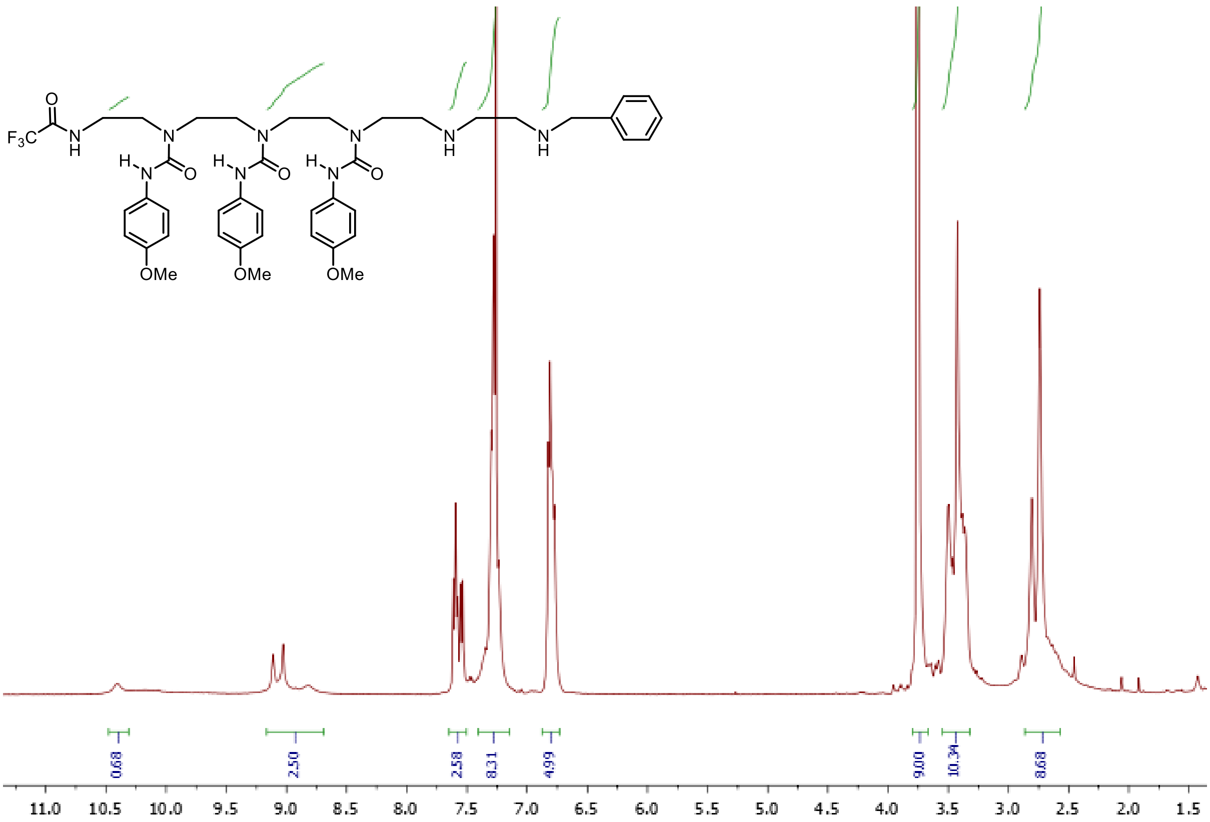

Figure S134 – $^{1}$H NMR spectrum of 5b-6 (400 MHz, $CDCl_3$).

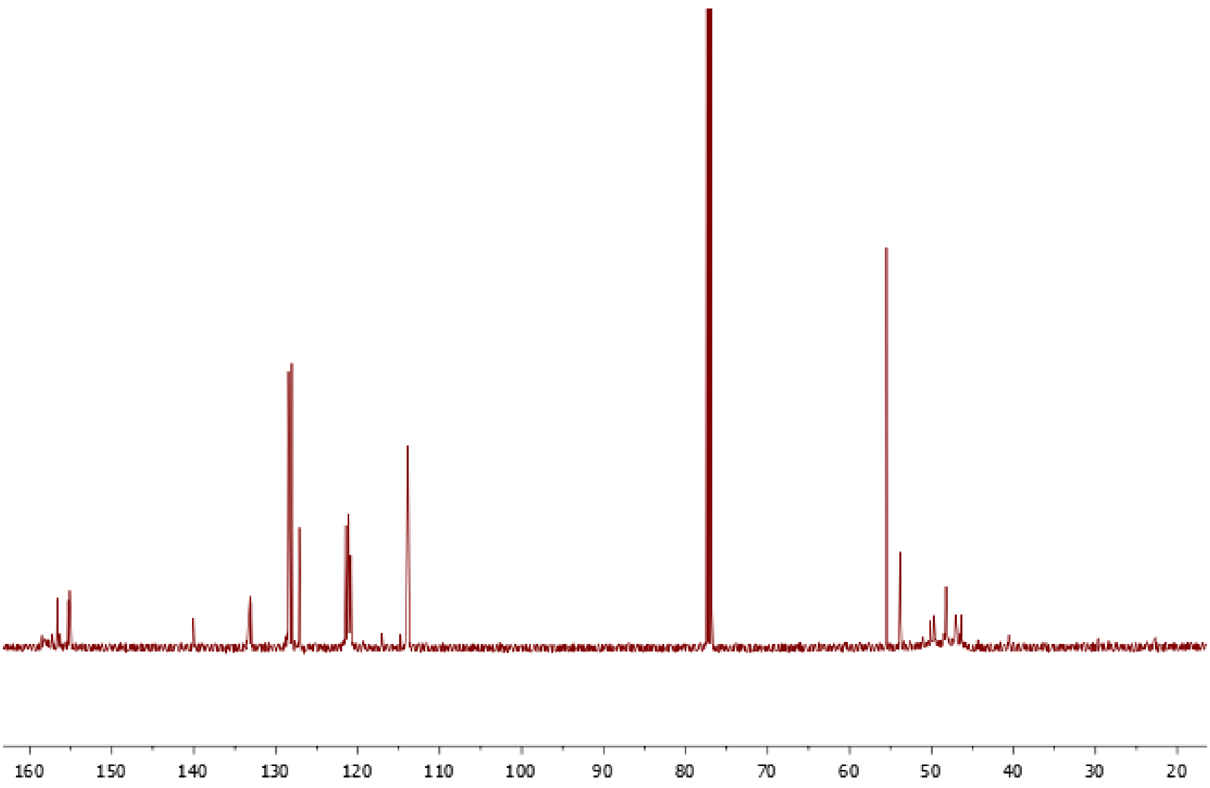

Figure S135 – $^{13}$C NMR spectrum of 5b-6 (101 MHz, $CDCl_3$).

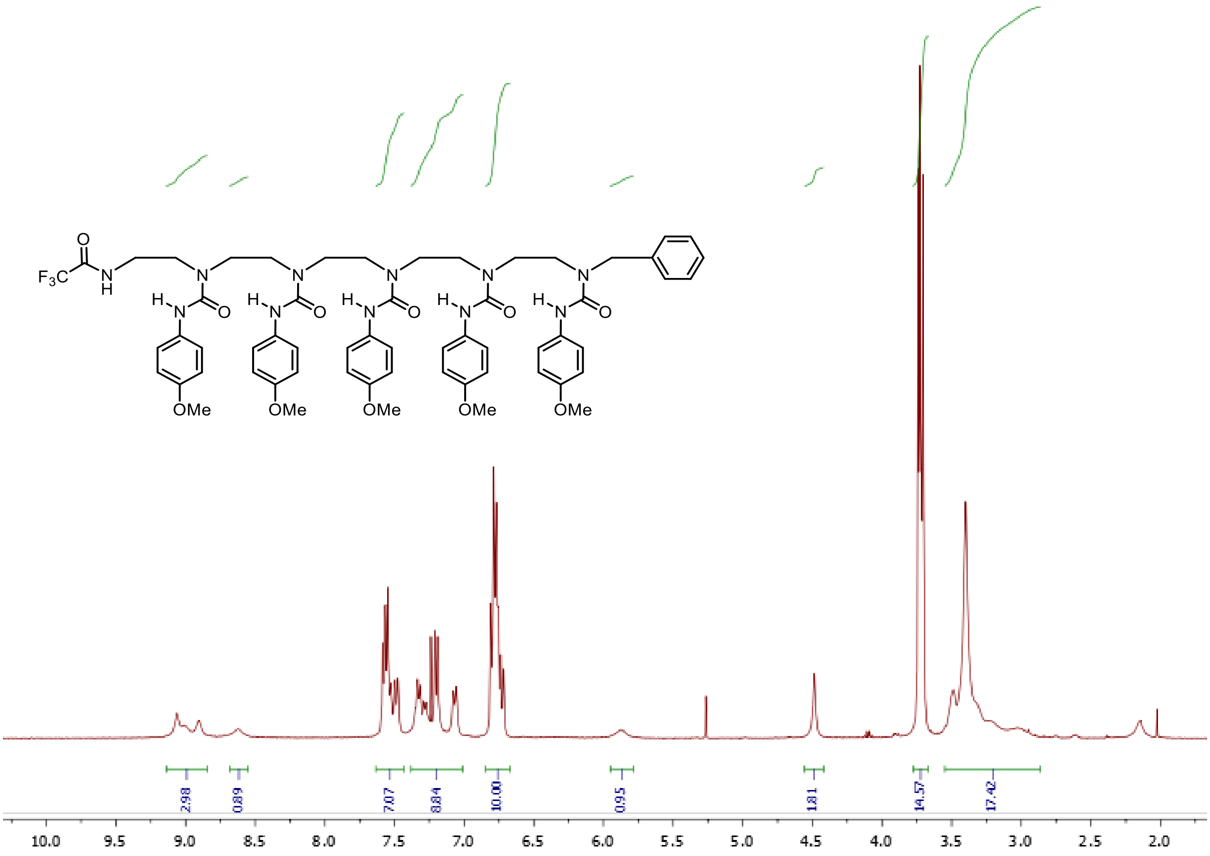


**Figure S136 – $^{1}$H NMR spectrum of 5b-7 (400 MHz, $CDCl_3$).**

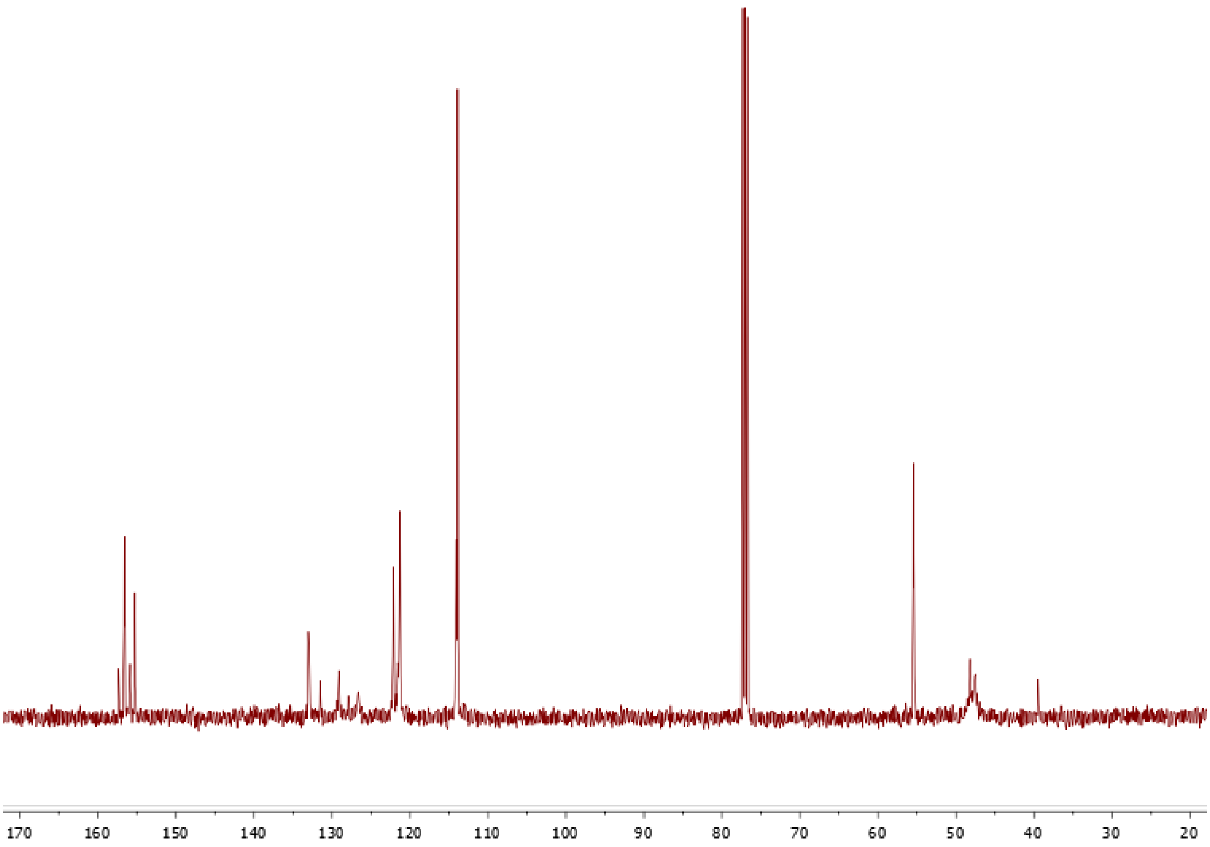


**Figure S137 – $^{13}$C NMR spectrum of 5b-7 (101 MHz, $CDCl_3$).**

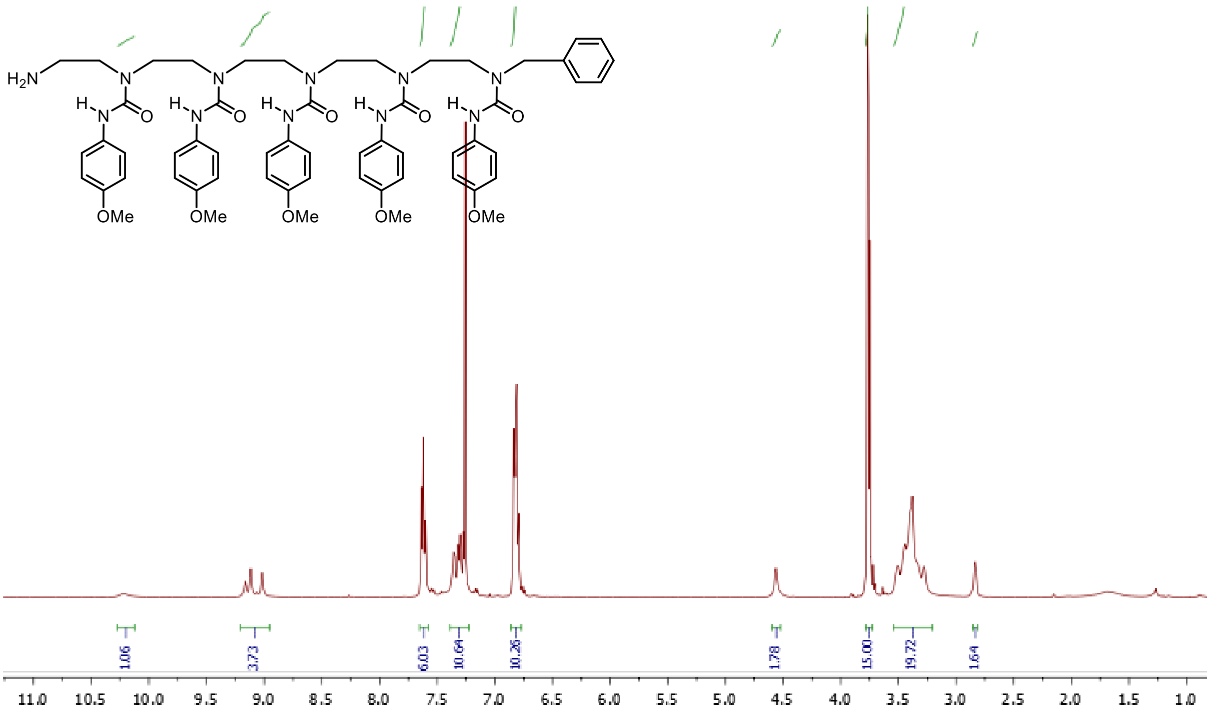


**Figure S138 – $^{1}$H NMR spectrum of 5b-8 (500 MHz, CDCl$_3$).**

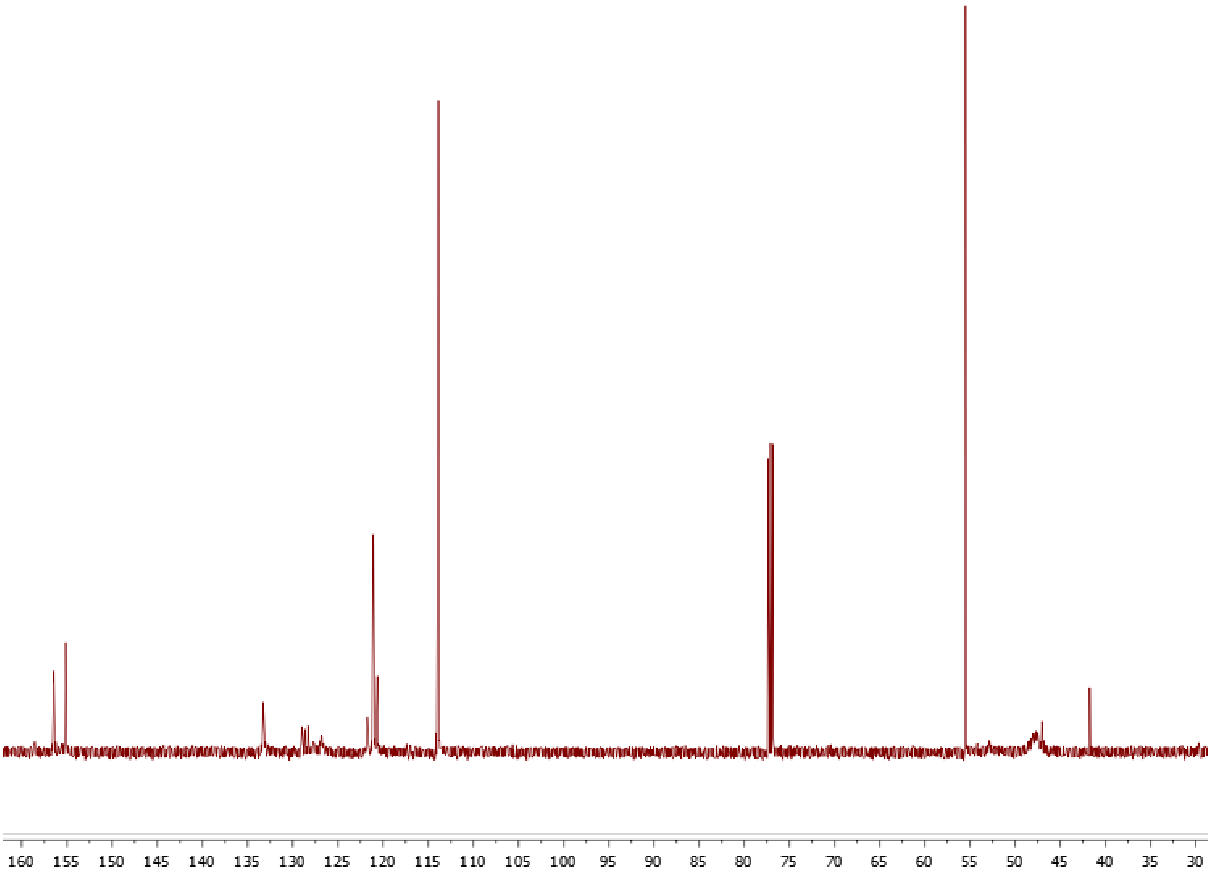


**Figure S139 – $^{13}$C NMR spectrum of 5b-8 (126 MHz, CDCl$_3$).**

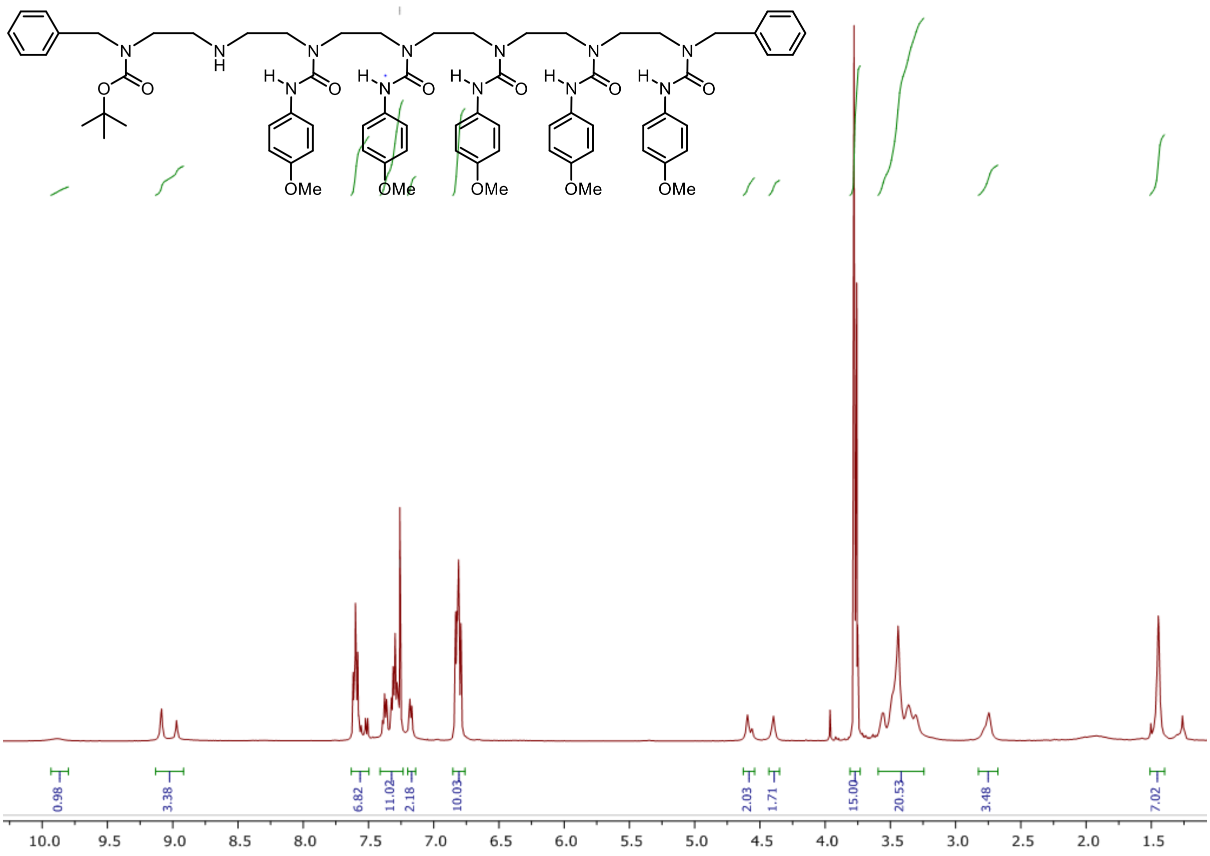


**Figure S140 – $^{1}$H NMR spectrum of 5b-9 (500 MHz, $CDCl_3$).**

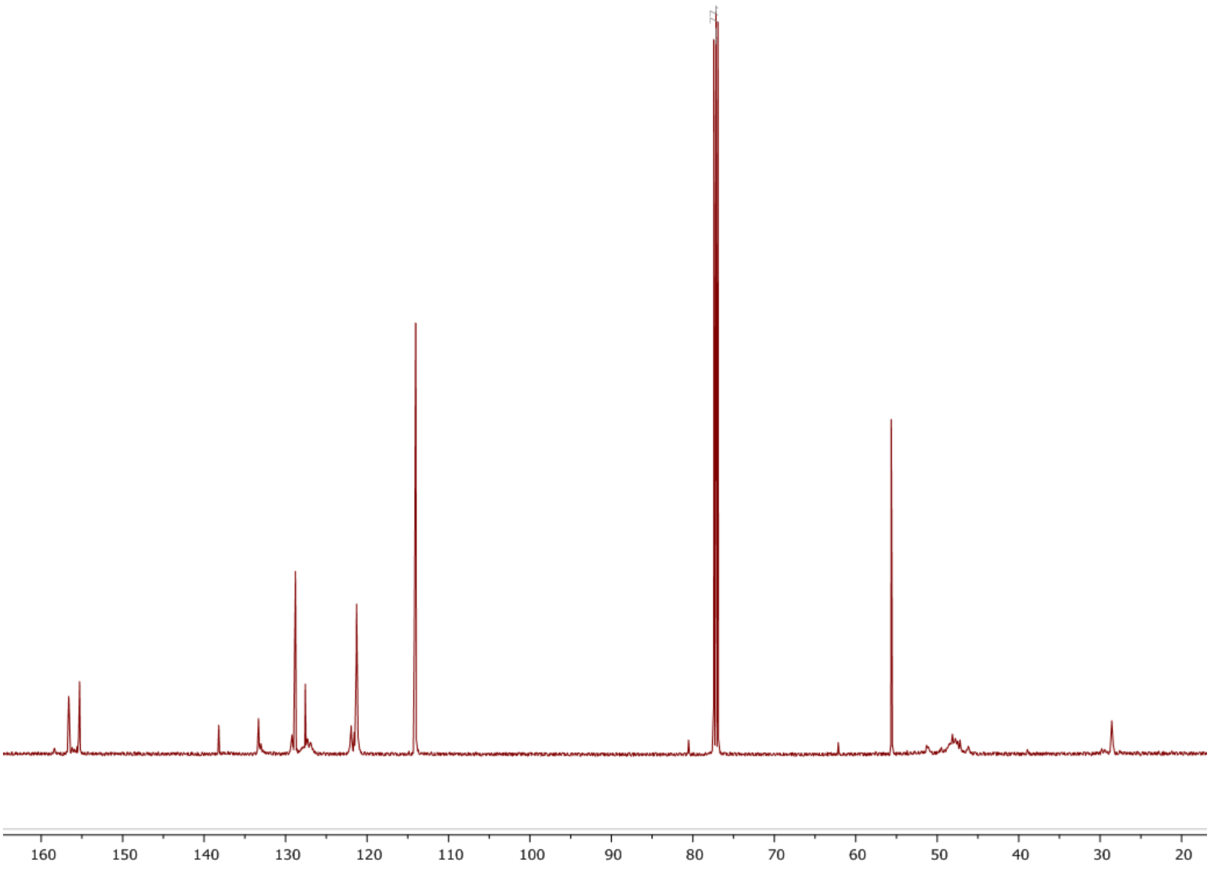


**Figure S141 – $^{13}$C NMR spectrum of 5b-9 (126 MHz, $CDCl_3$).**

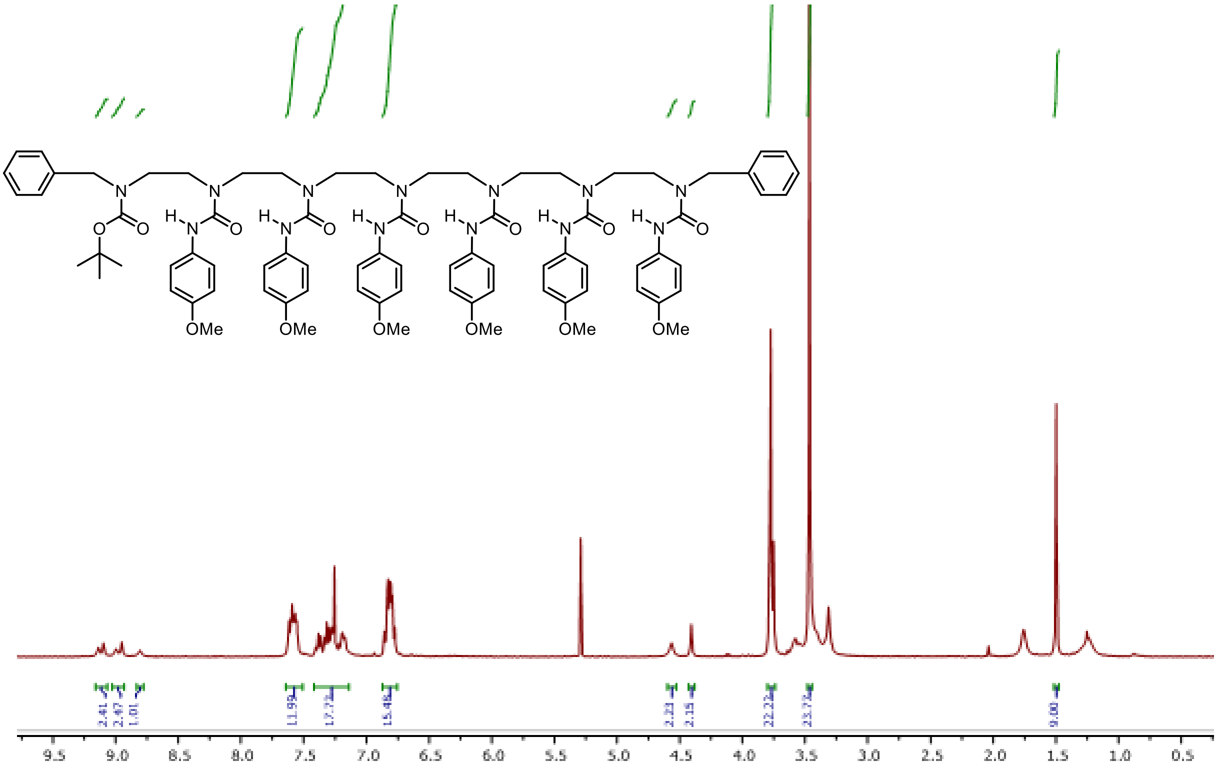

Figure S142 – $^{1}$H NMR spectrum of 5b-10 (500 MHz, $CDCl_3$).

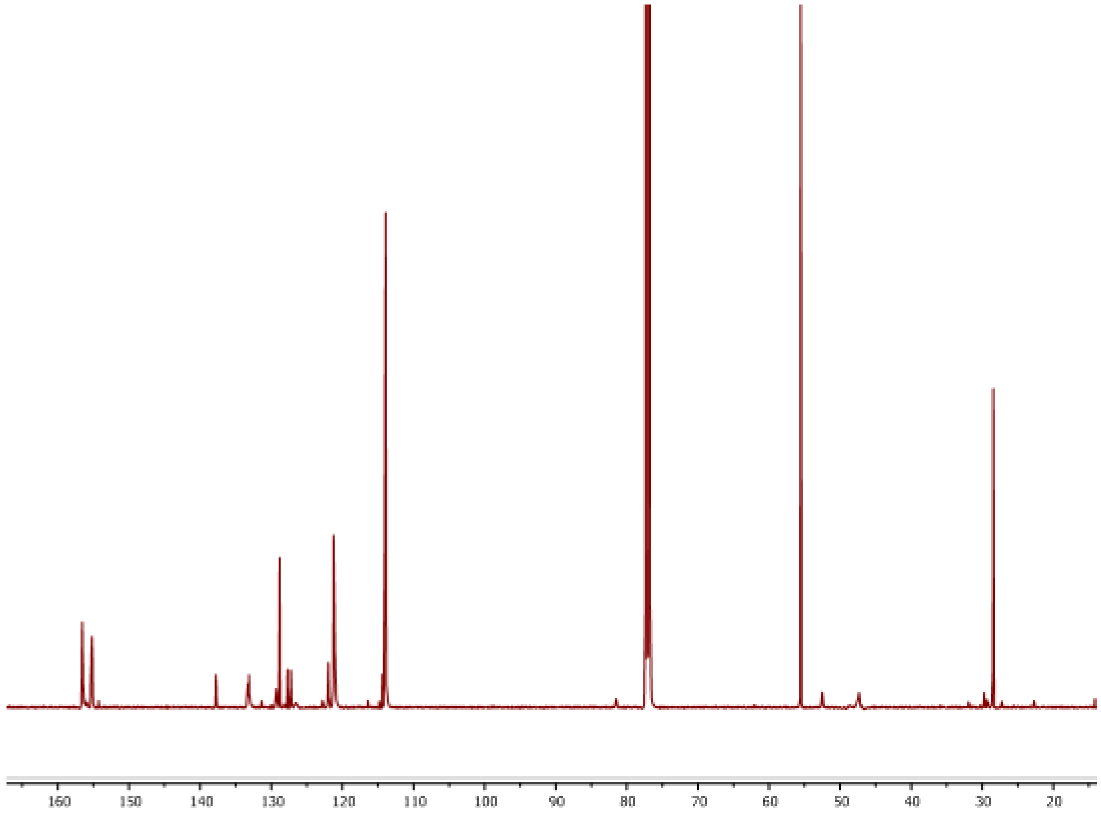

Figure S143 – $^{13}$C NMR spectrum of 5b-10 (126 MHz, $CDCl_3$).

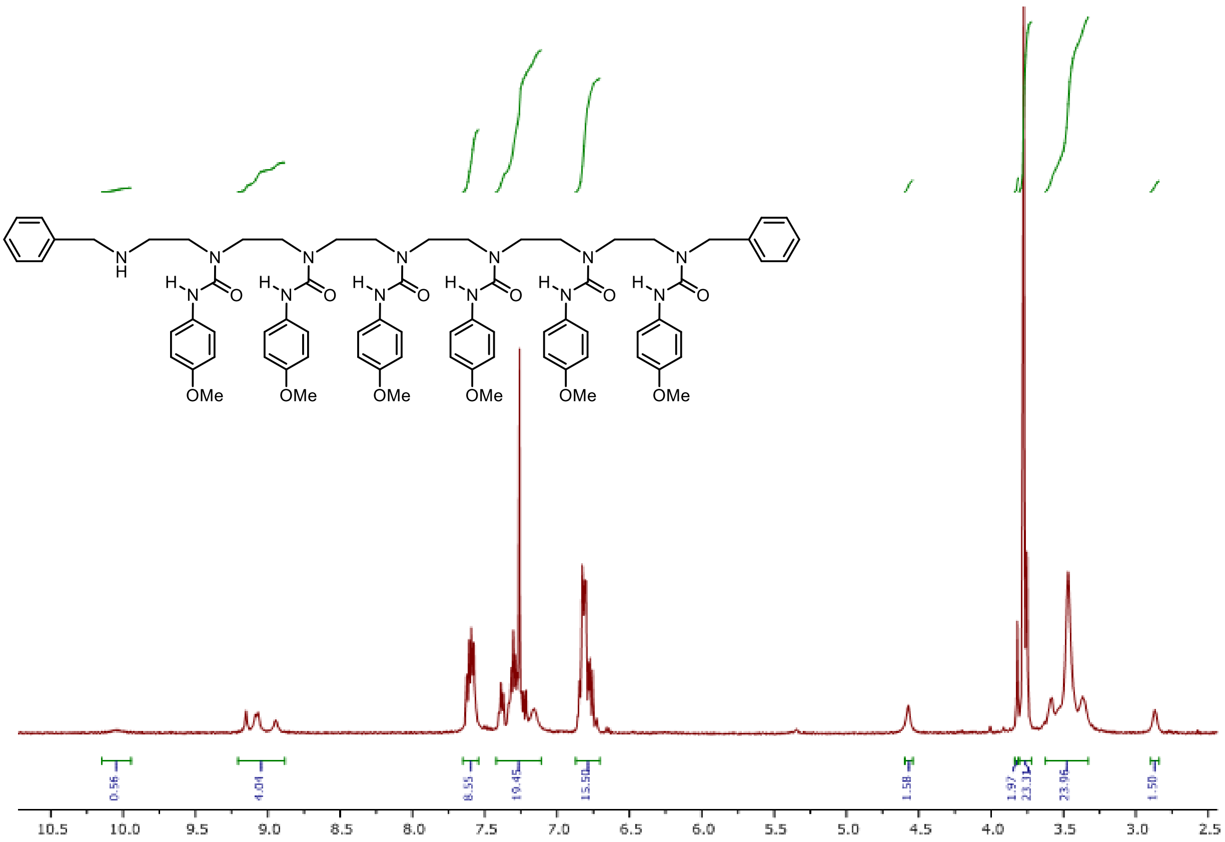


**Figure S144 – $^{1}H$ NMR spectrum of 5b-11 (400 MHz, $CDCl_3$).**

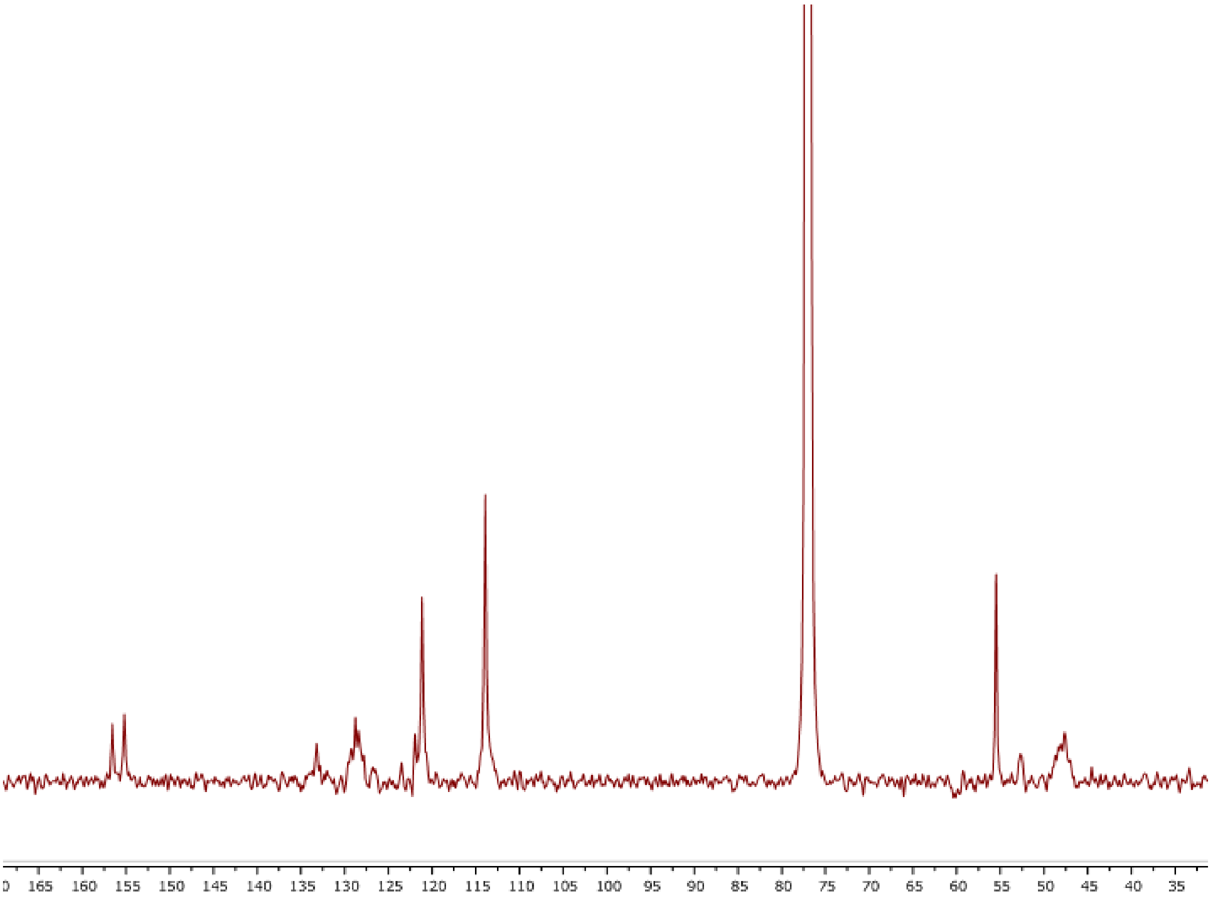


**Figure S145 – $^{13}C$ NMR spectrum of 5b-11 (101 MHz, $CDCl_3$).**

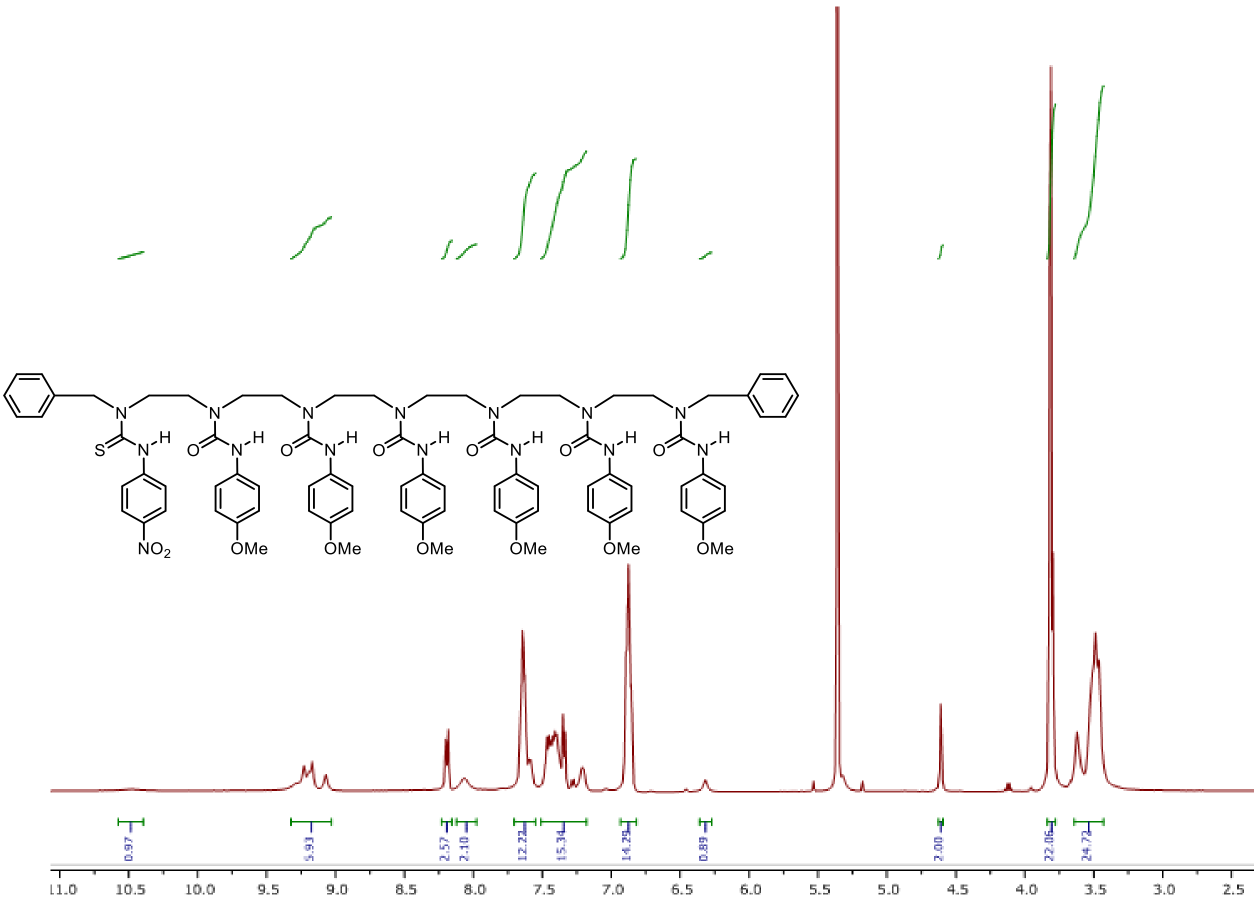


**Figure S146 – $^{1}H$ NMR spectrum of 5b (500 MHz, $CD_2Cl_2$).**

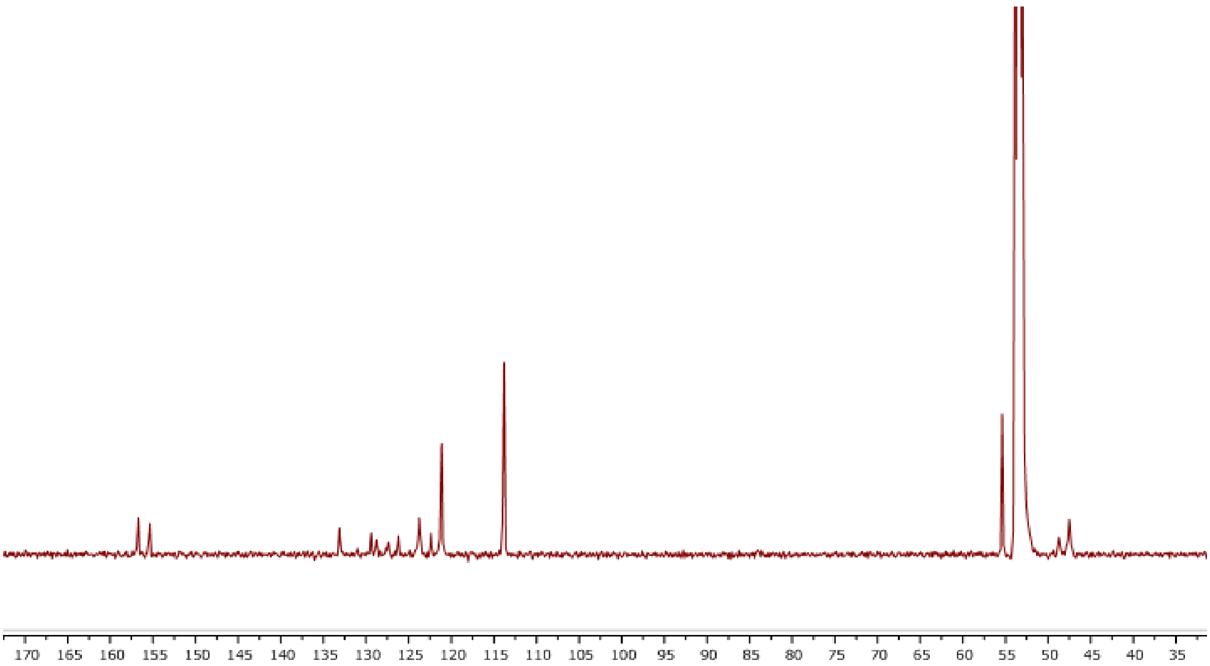


**Figure S147 – $^{13}C$ NMR spectrum of 5b (126 MHz, $CD_2Cl_2$).**

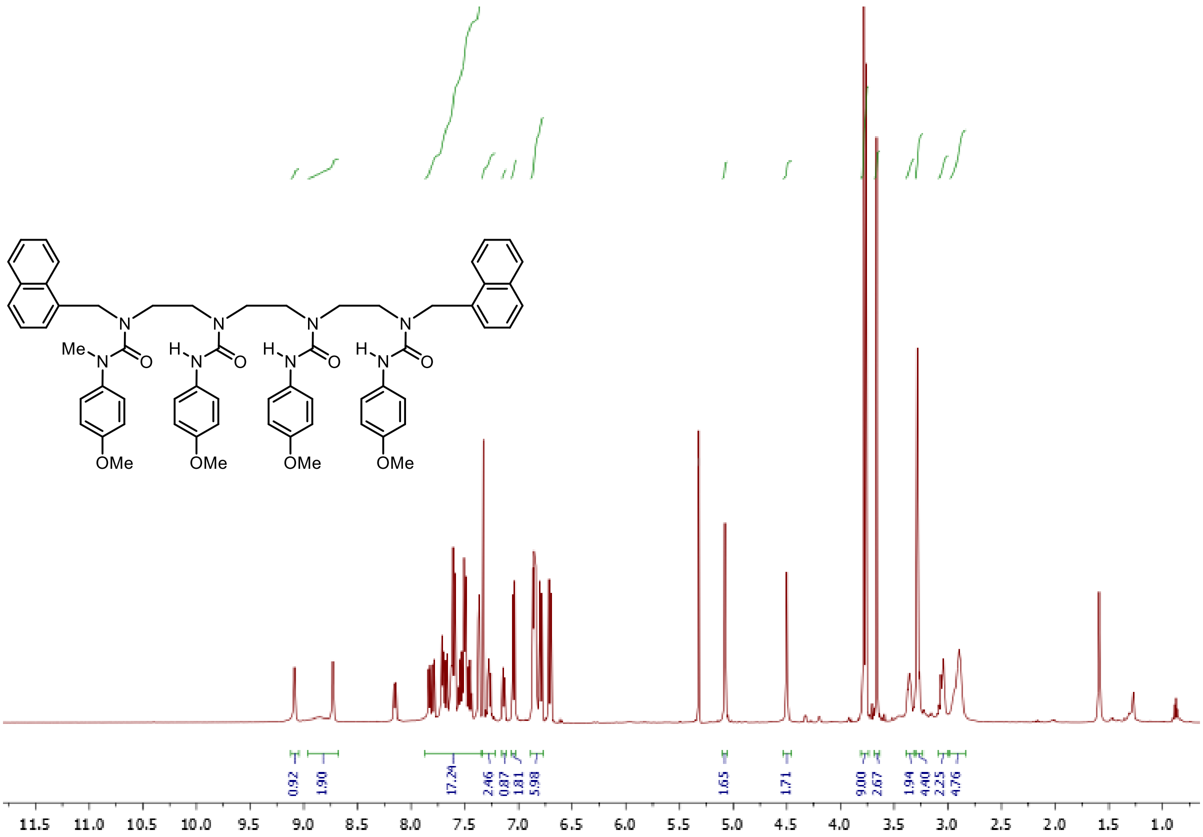


**Figure S148 – $^{1}$H NMR spectrum of 6a (500 MHz, $CD_2Cl_2$).**

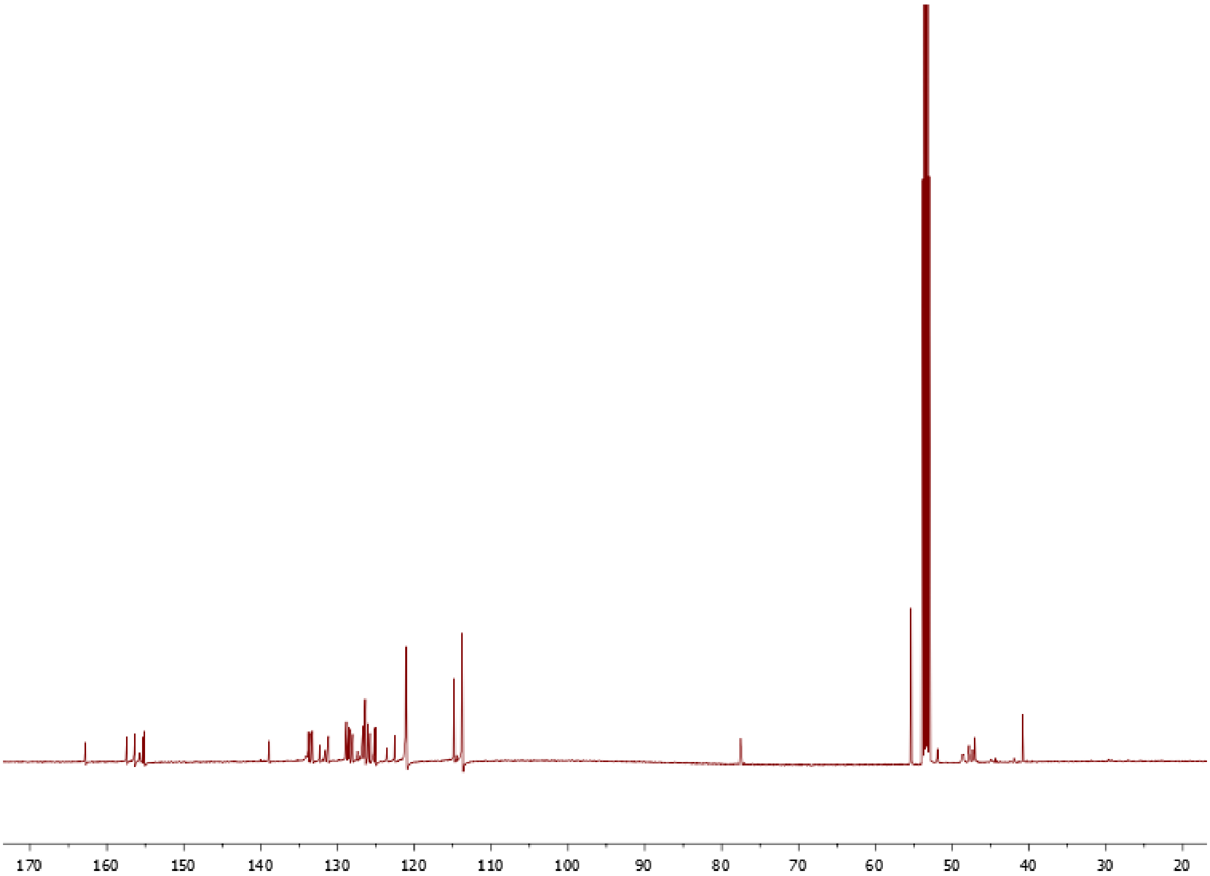


**Figure S149 – $^{13}$C NMR spectrum of 6a (126 MHz, $CD_2Cl_2$).**

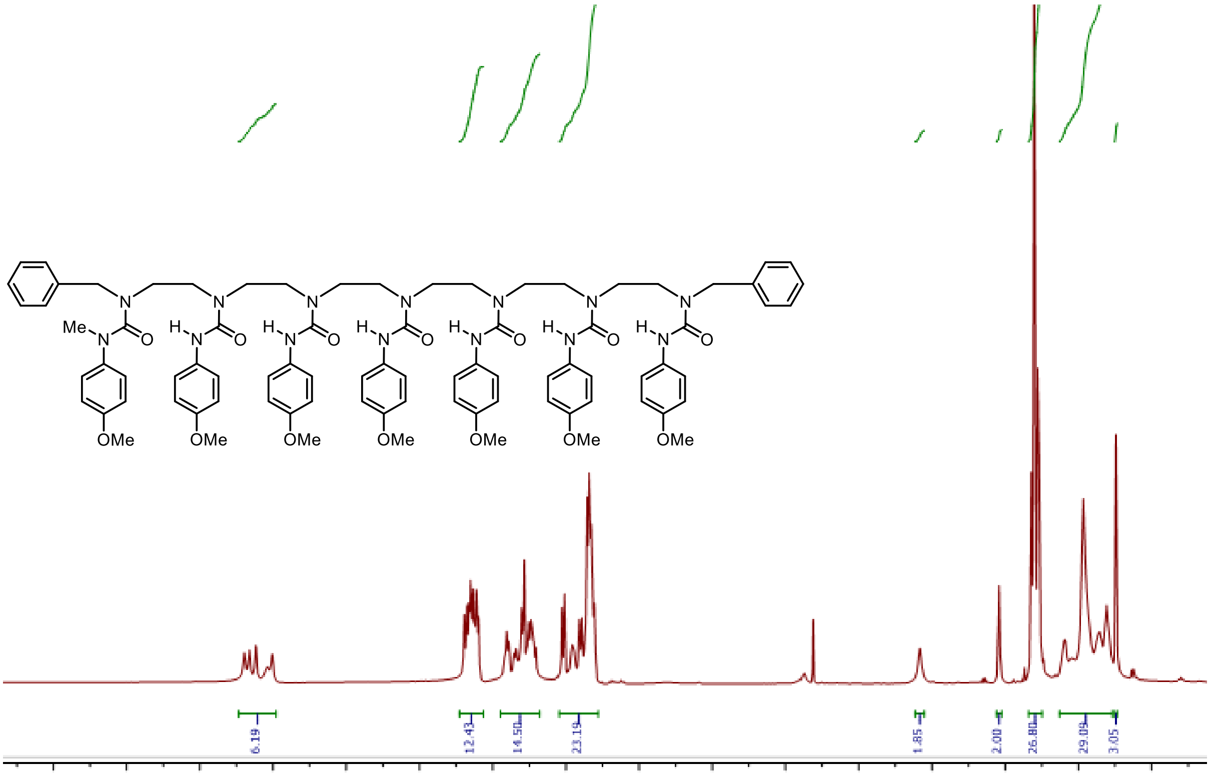


**Figure S150 – $^{1}$H NMR spectrum of 6b (500 MHz, $CDCl_3$).**

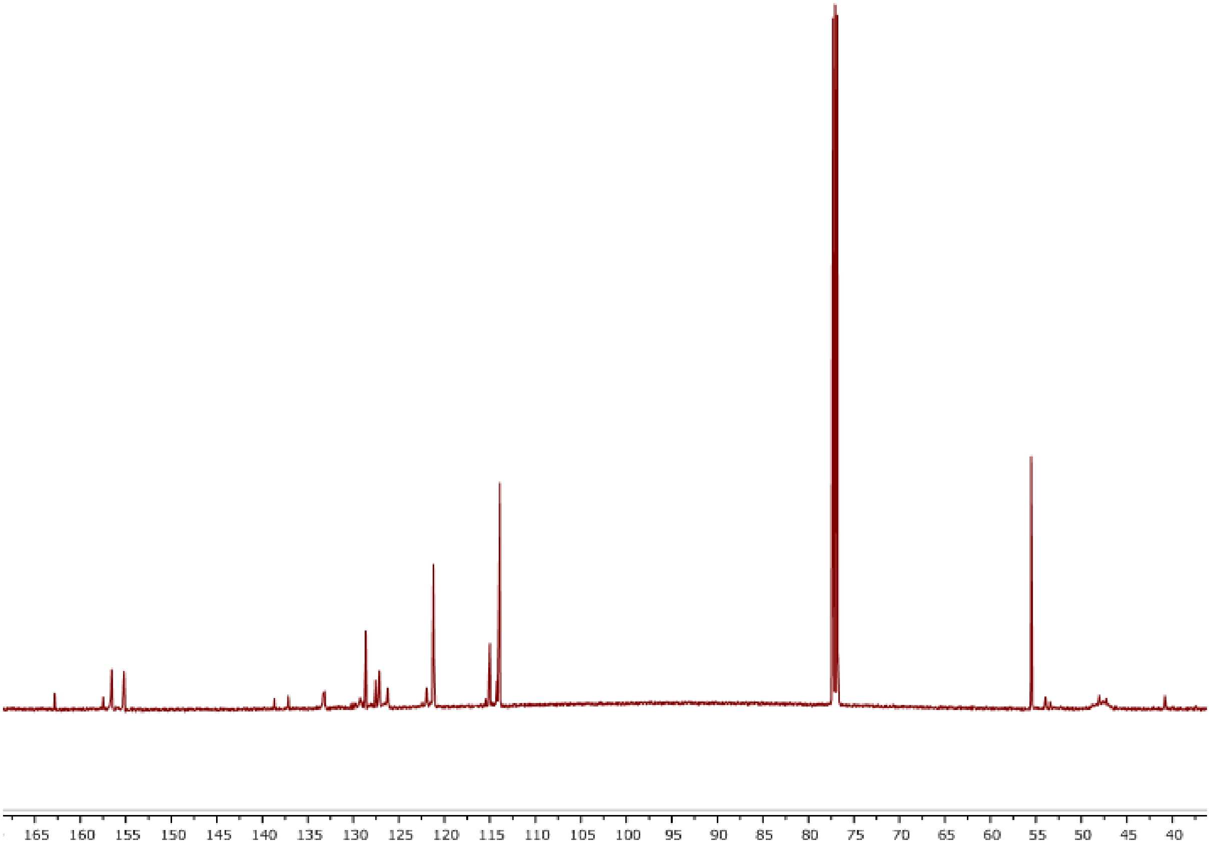


**Figure S151 – $^{13}$C NMR spectrum of 6b (126 MHz, $CDCl_3$).**

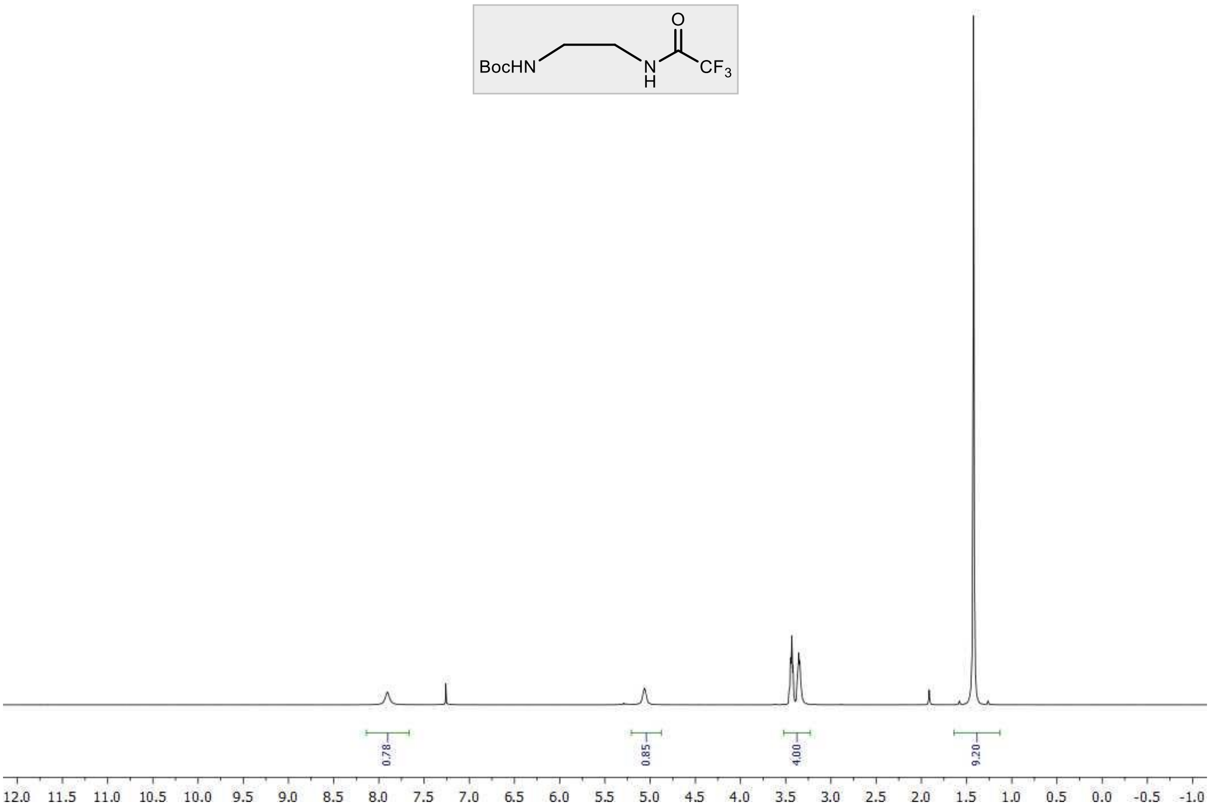


**Figure S152 – $^{1}$H NMR spectrum of 4b-1 (400 MHz, $CDCl_3$).**

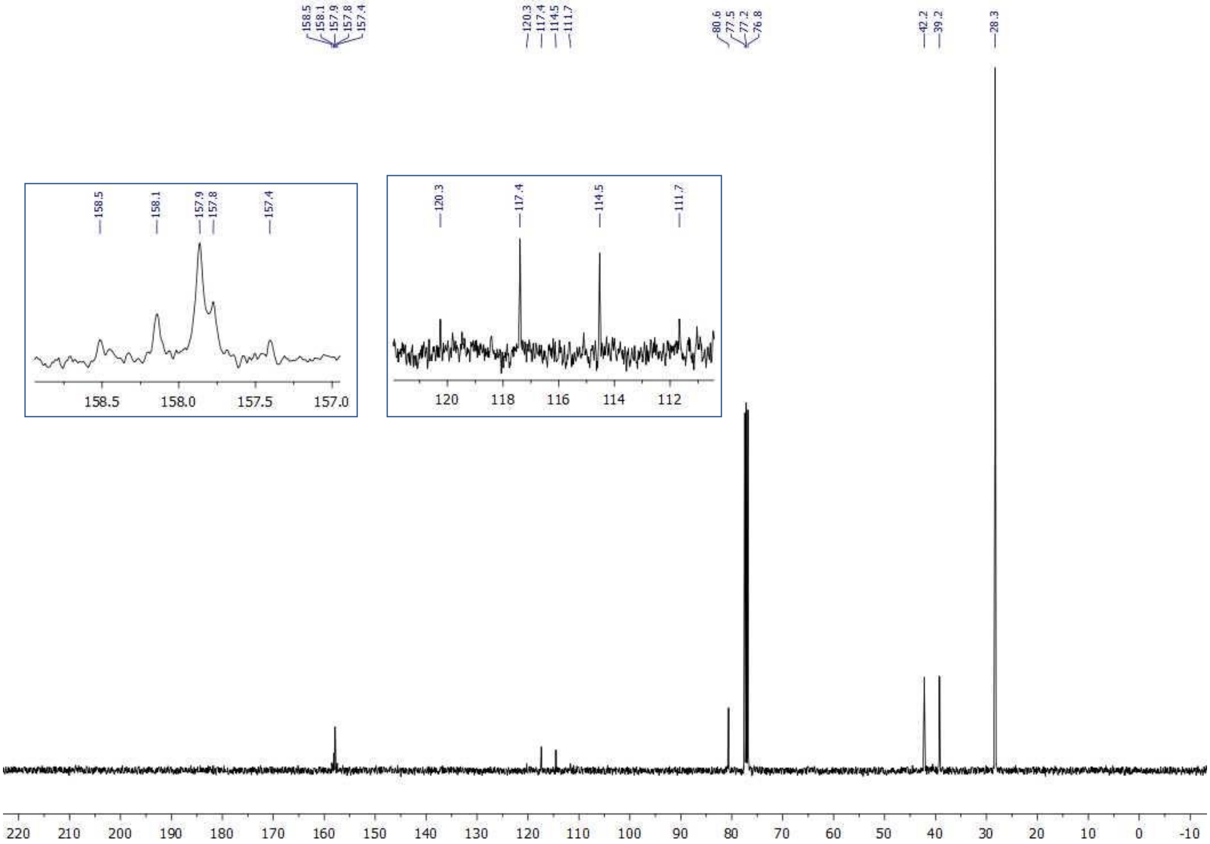


**Figure S153 – $^{13}$C NMR spectrum of 4b-1 (101 MHz, $CDCl_3$).**

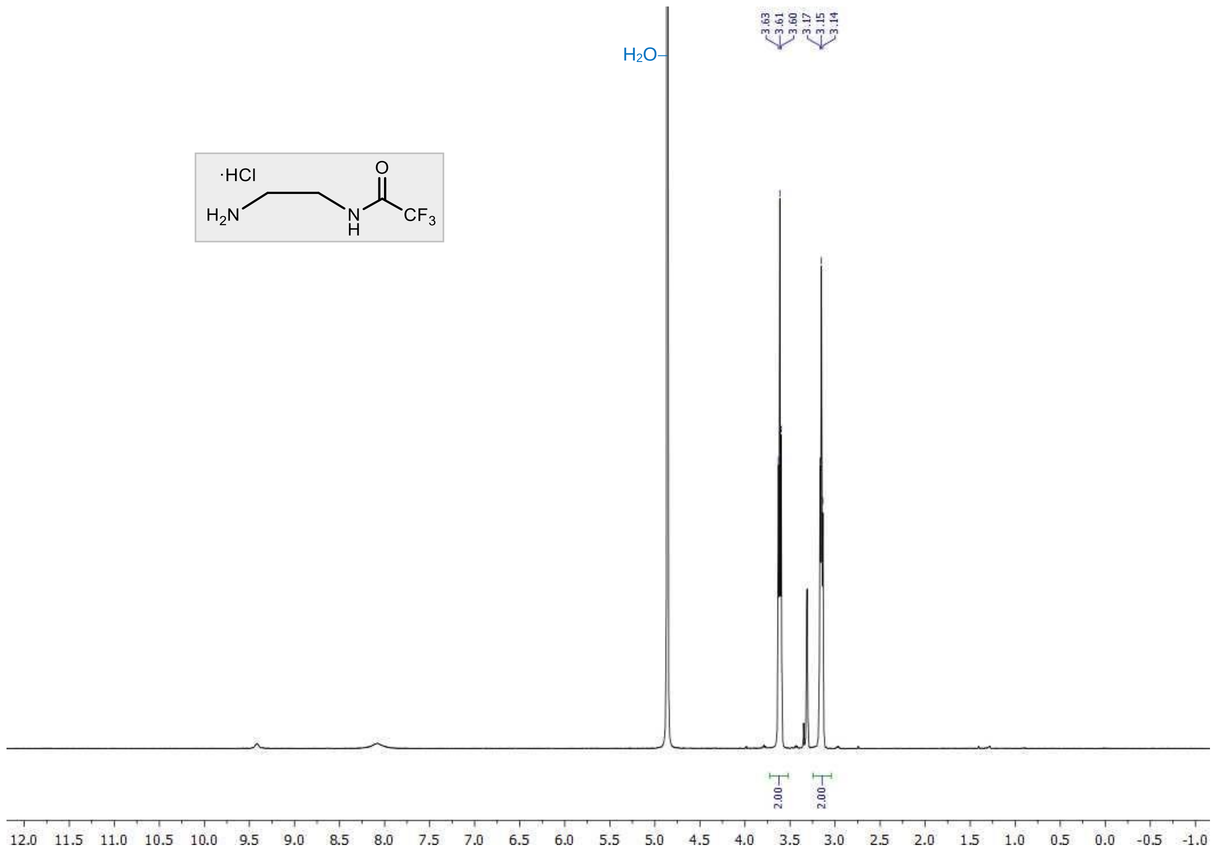


**Figure S154 – $^{1}$H NMR spectrum of 4b-2 (400 MHz, $CD_3OD$).**

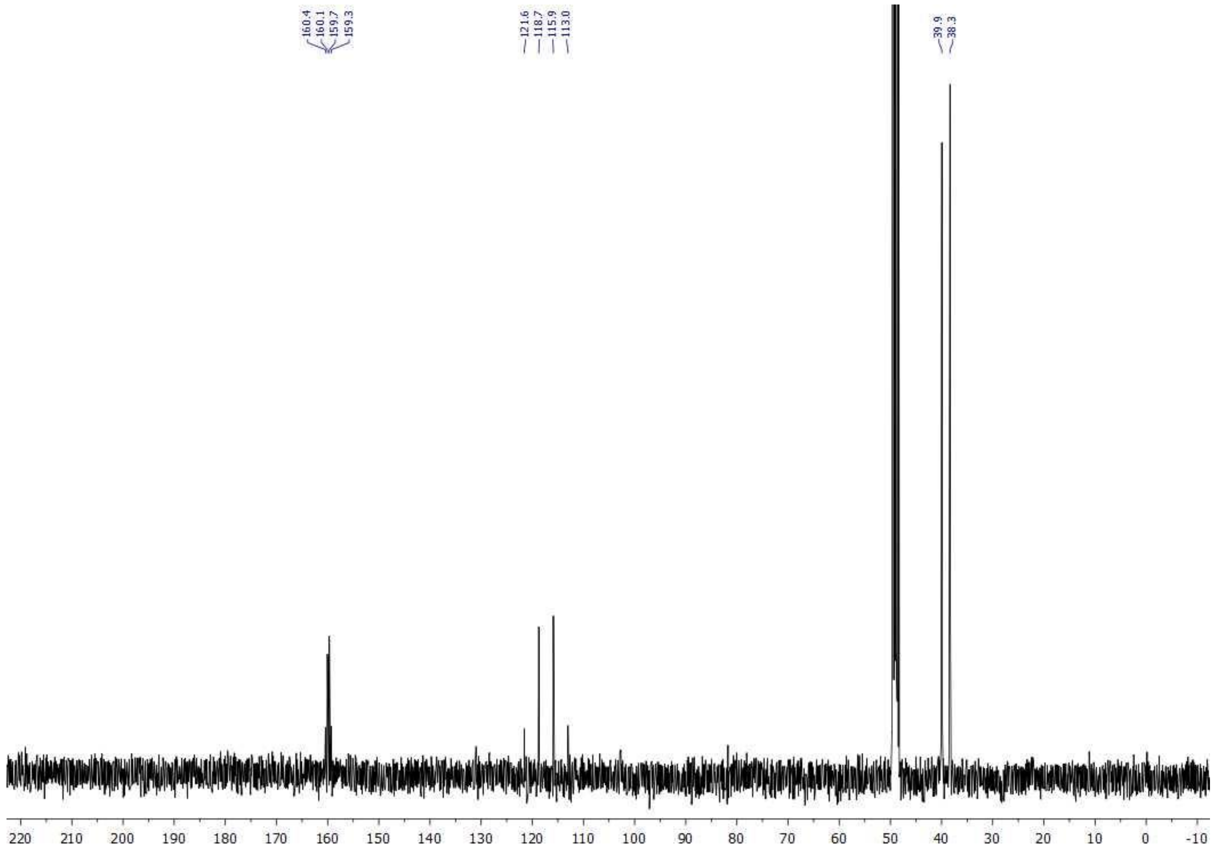


**Figure S155 – $^{13}$C NMR spectrum of 4b-2 (101 MHz, $CD_3OD$).**

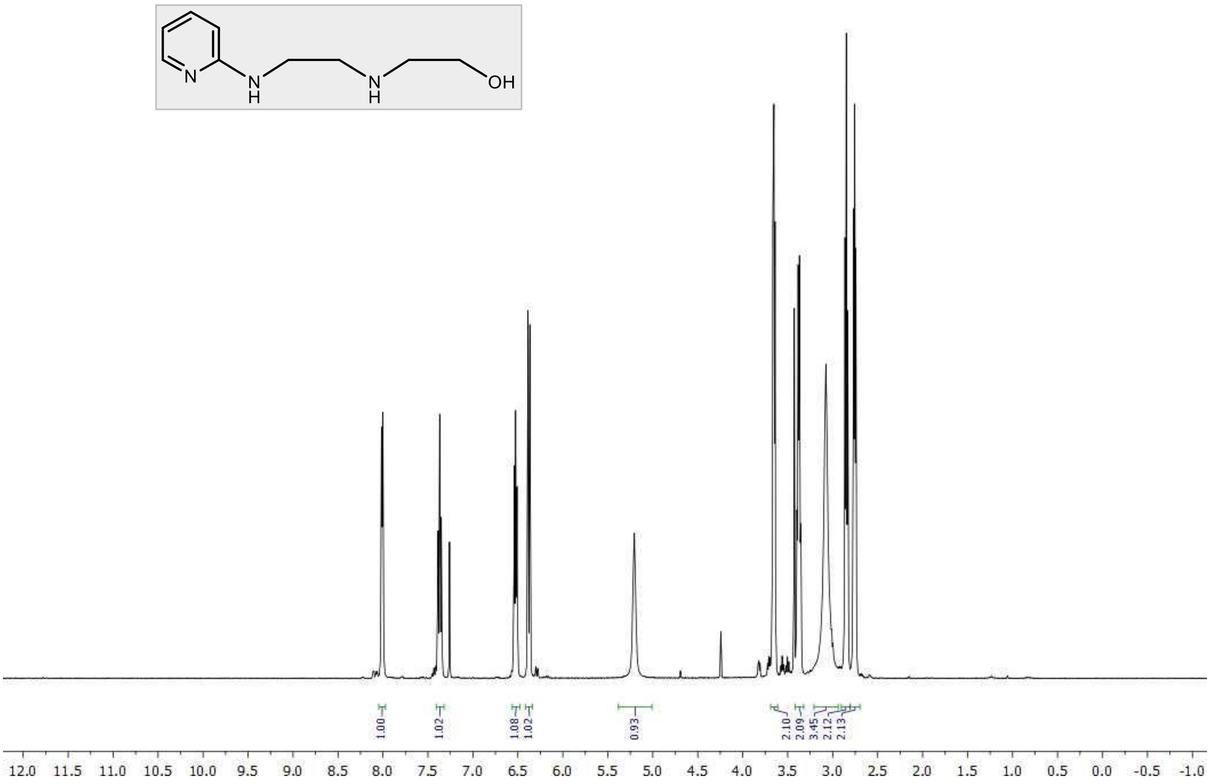

**Figure S156 – $^{1}$H NMR spectrum of 4b-3 (400 MHz, $CDCl_3$).**

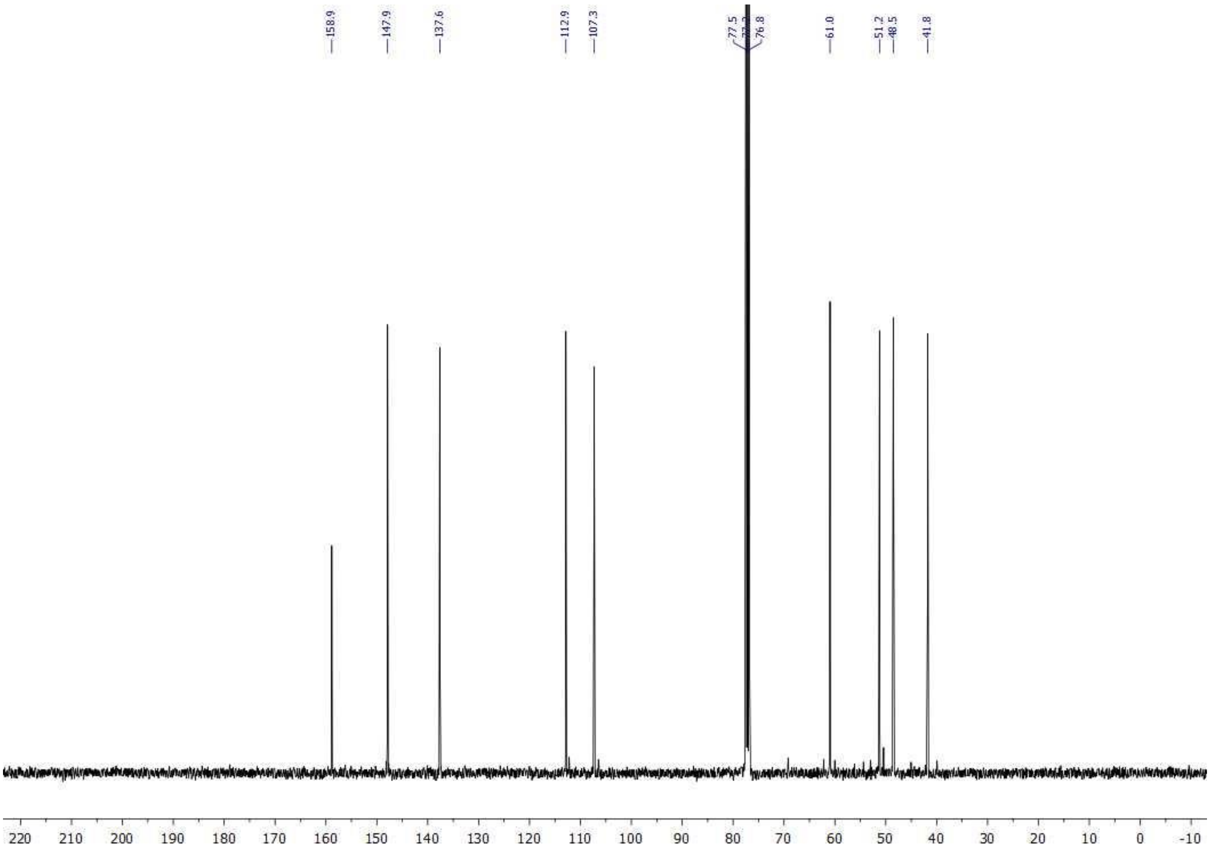

**Figure S157 – $^{13}$C NMR spectrum of 4b-3 (101 MHz, $CDCl_3$).**

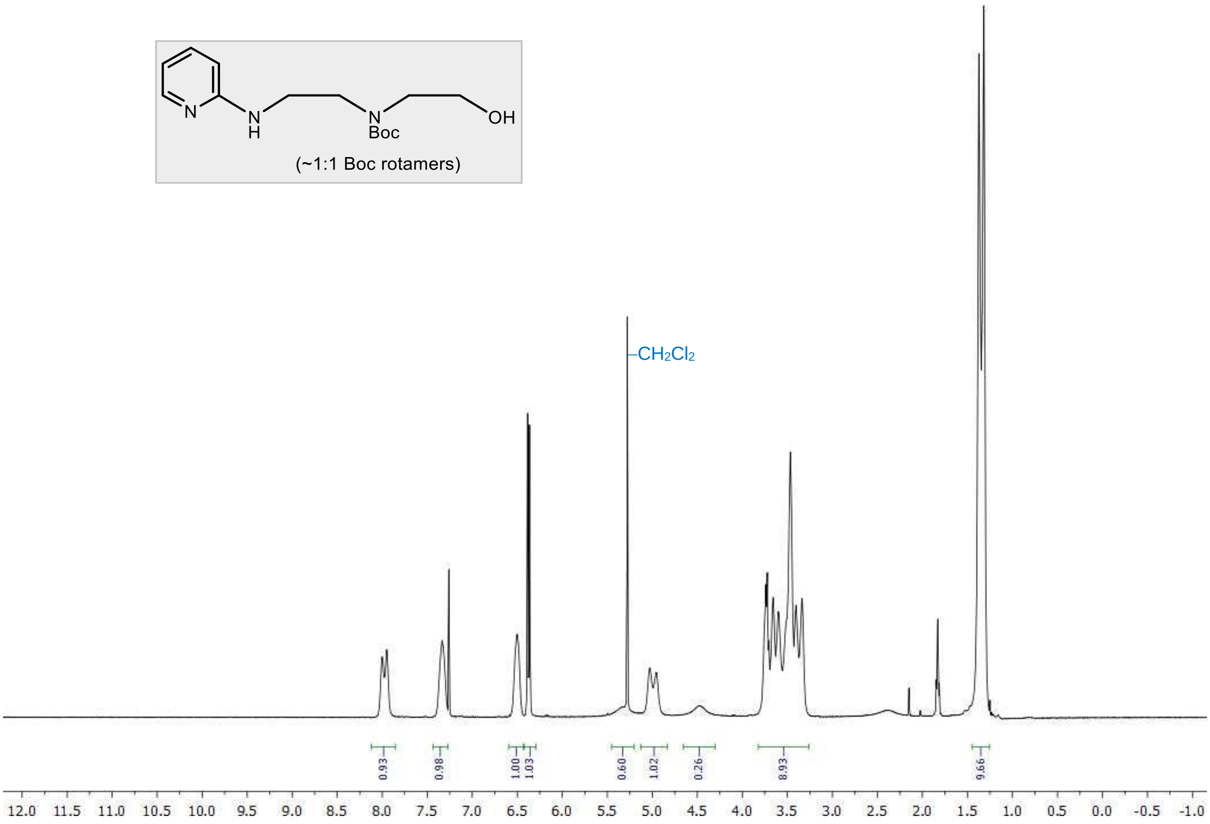


**Figure S158 – $^{1}$H NMR spectrum of 4b-4 (400 MHz, $CDCl_3$).**

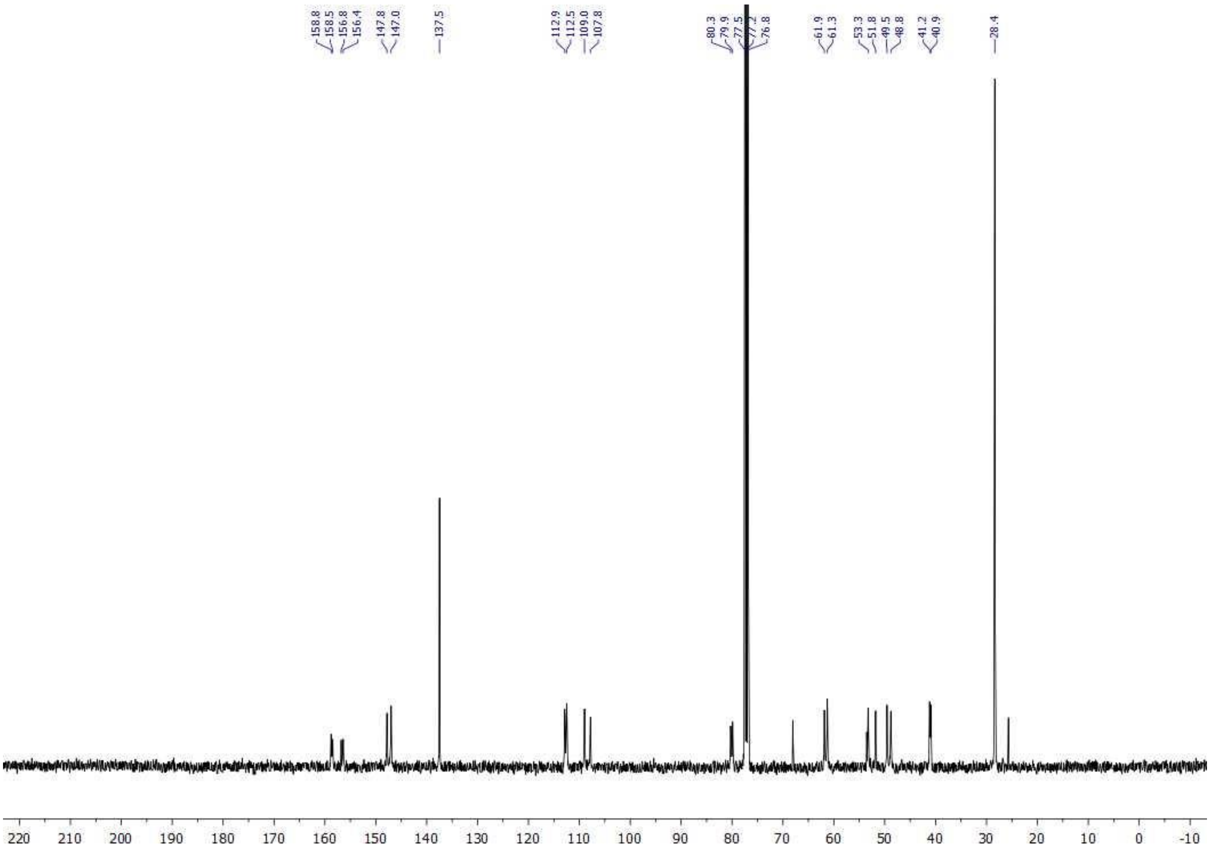


**Figure S159 – $^{13}$C NMR spectrum of 4b-4 (101 MHz, $CDCl_3$).**

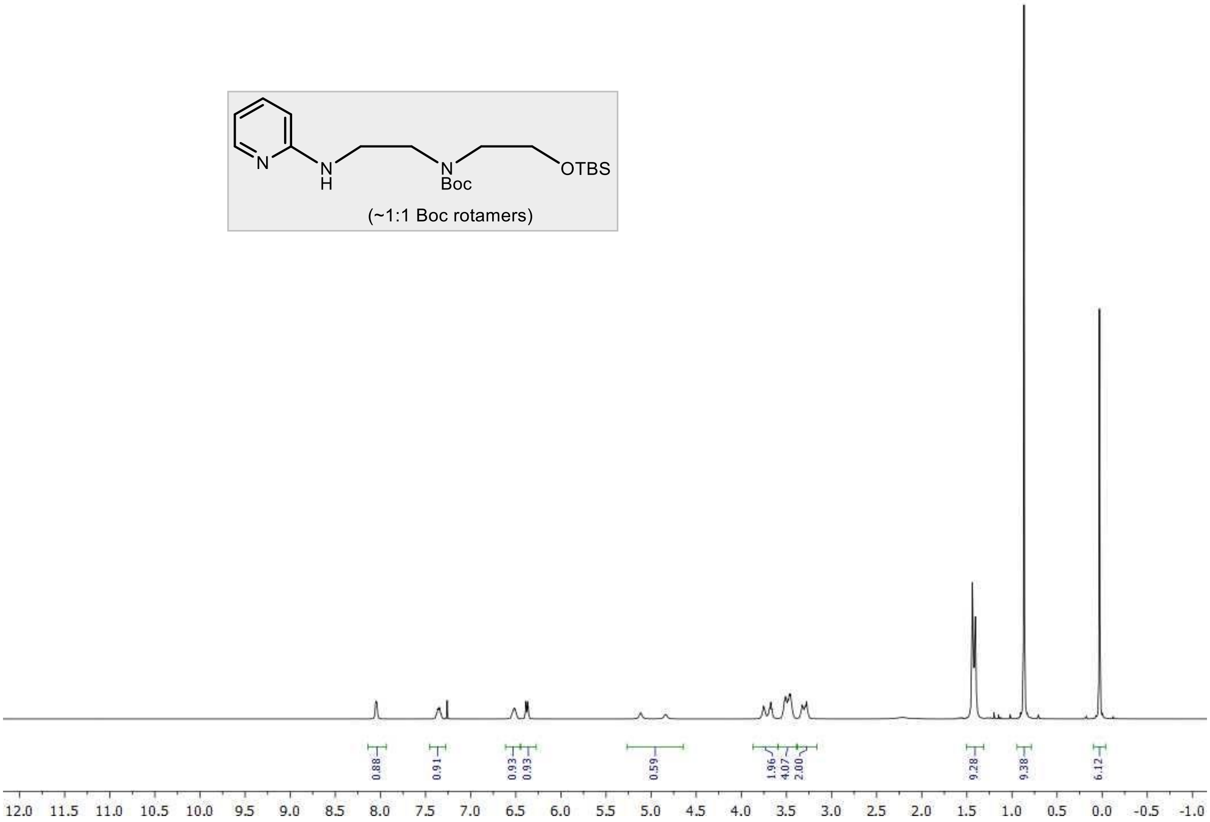


**Figure S160 – $^{1}$H NMR spectrum of 4b-5 (400 MHz, $CDCl_3$).**

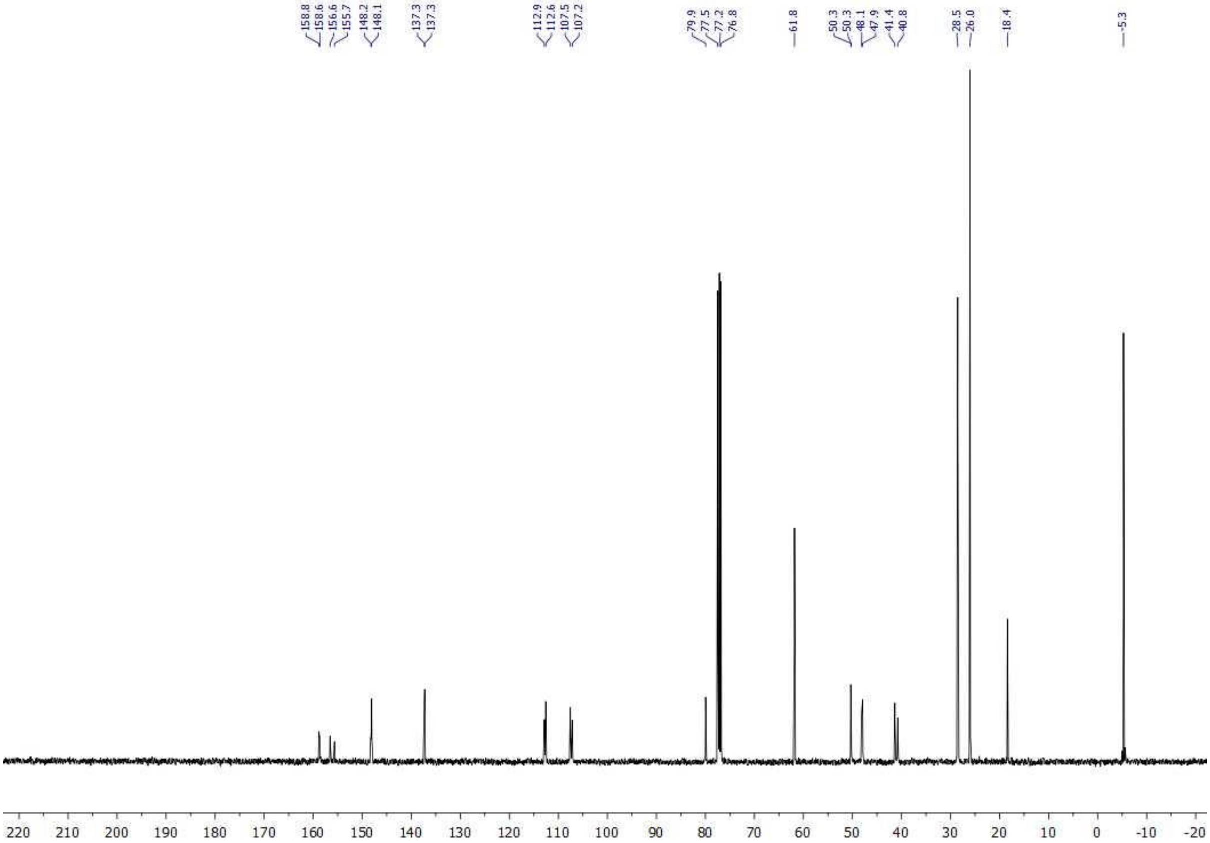


**Figure S161 – $^{13}$C NMR spectrum of 4b-5 (101 MHz, $CDCl_3$).**

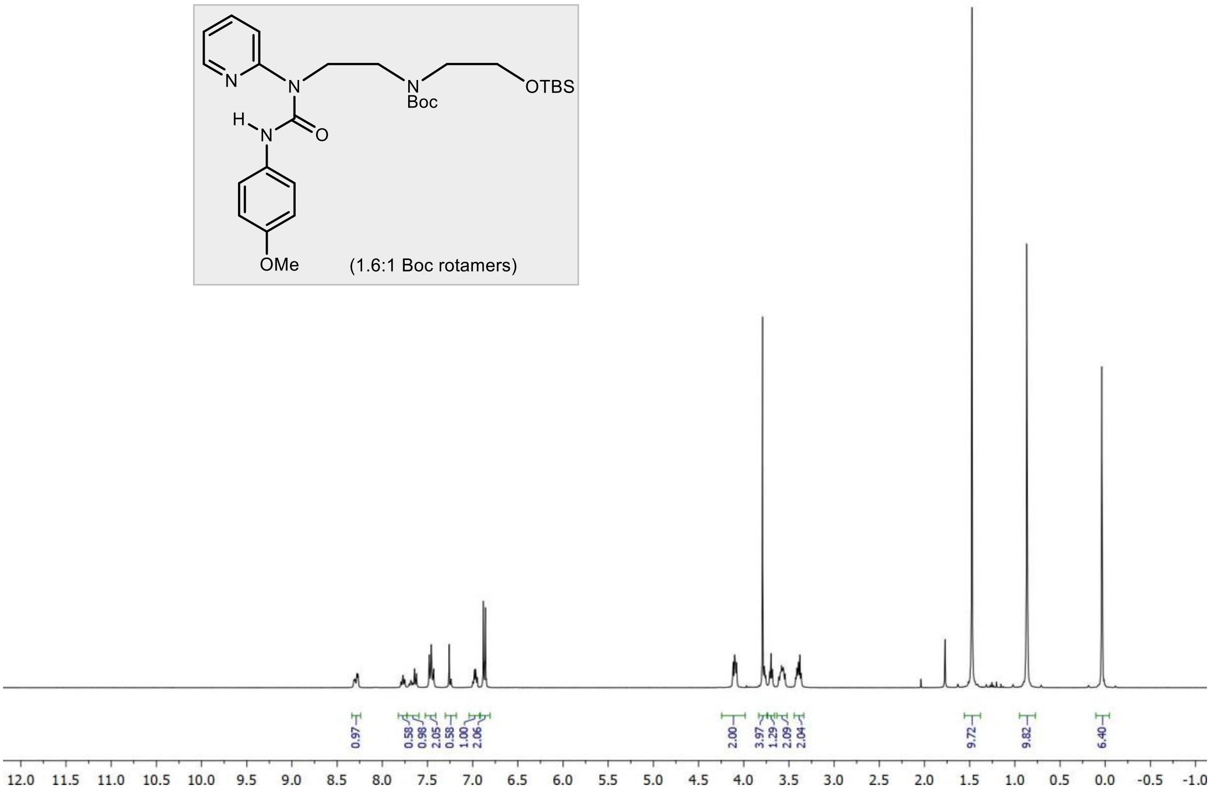


Figure S162 – $^{1}$H NMR spectrum of 4b-6 (400 MHz, $CDCl_3$).

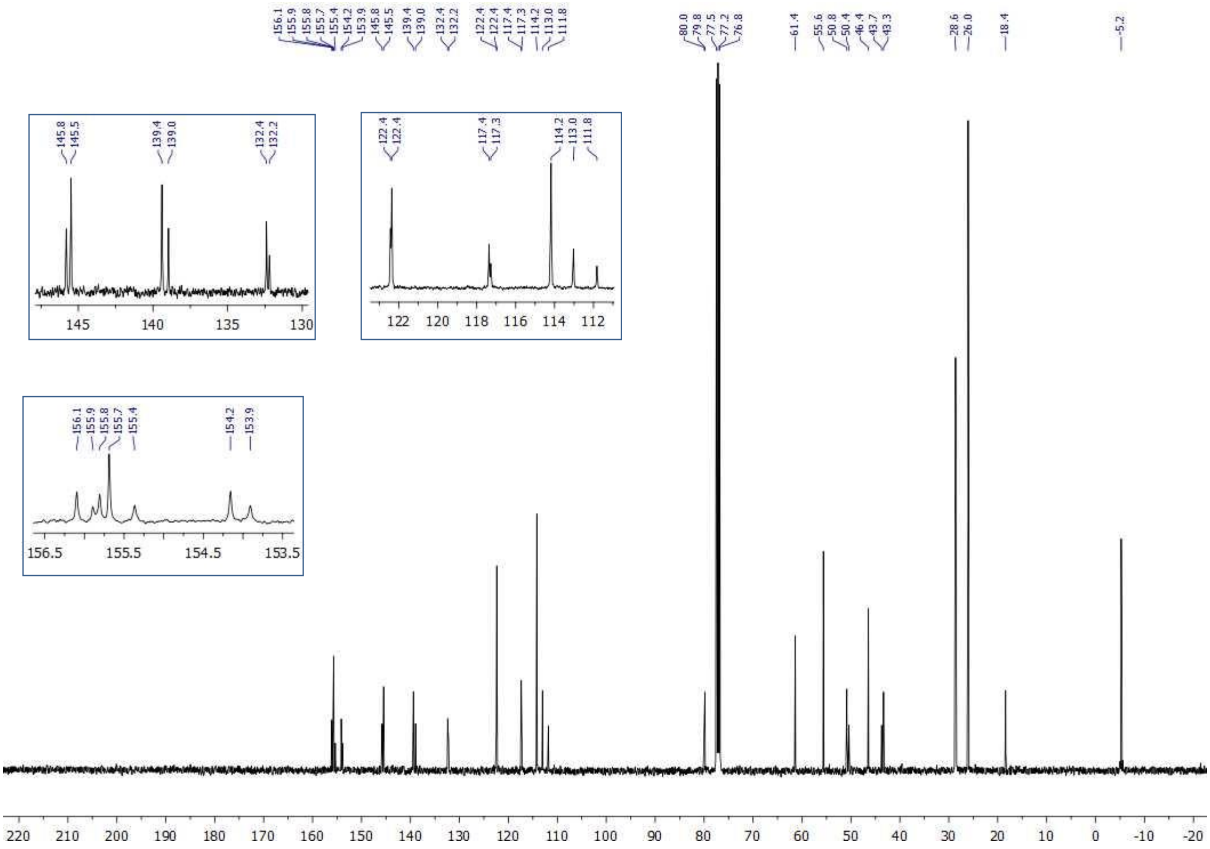

Figure S163 – $^{13}$C NMR spectrum of 4b-6 (101 MHz, $CDCl_3$).

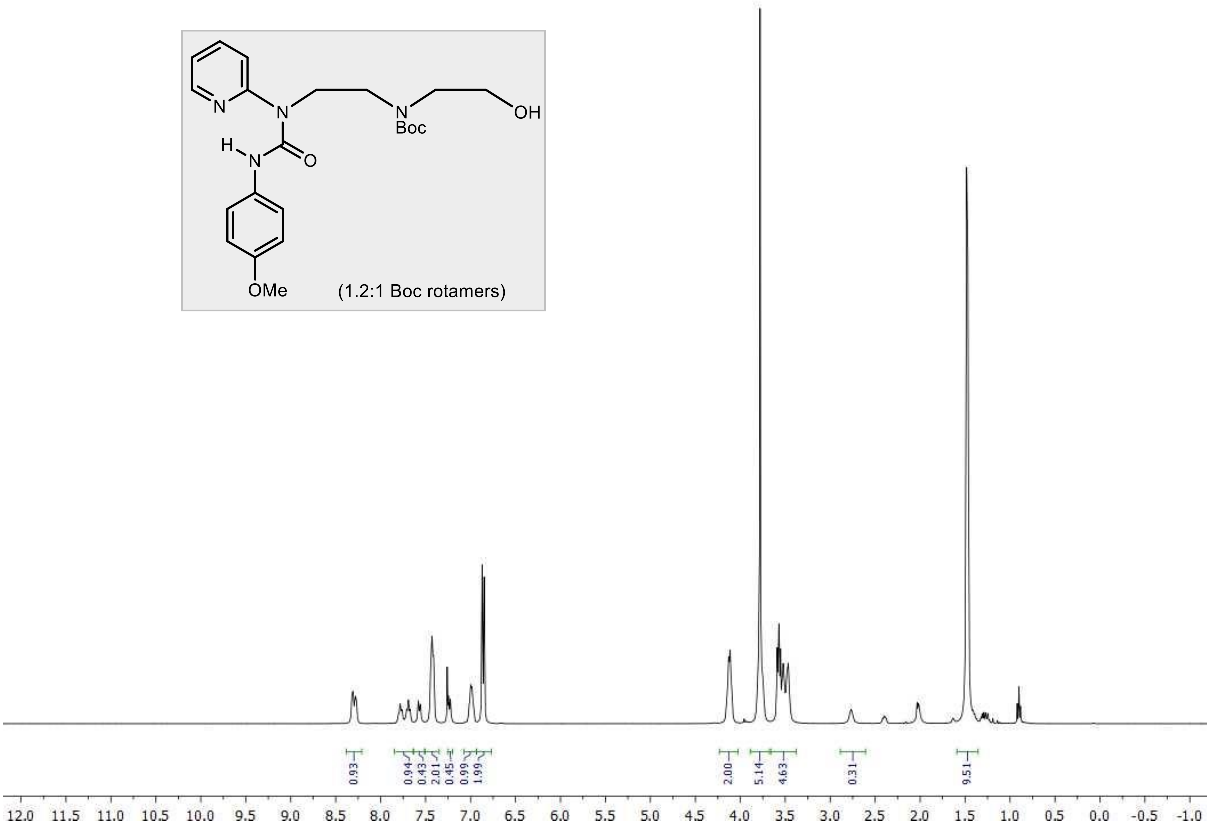


**Figure S164 – $^{1}$H NMR spectrum of 4b-7 (400 MHz, $CDCl_3$).**

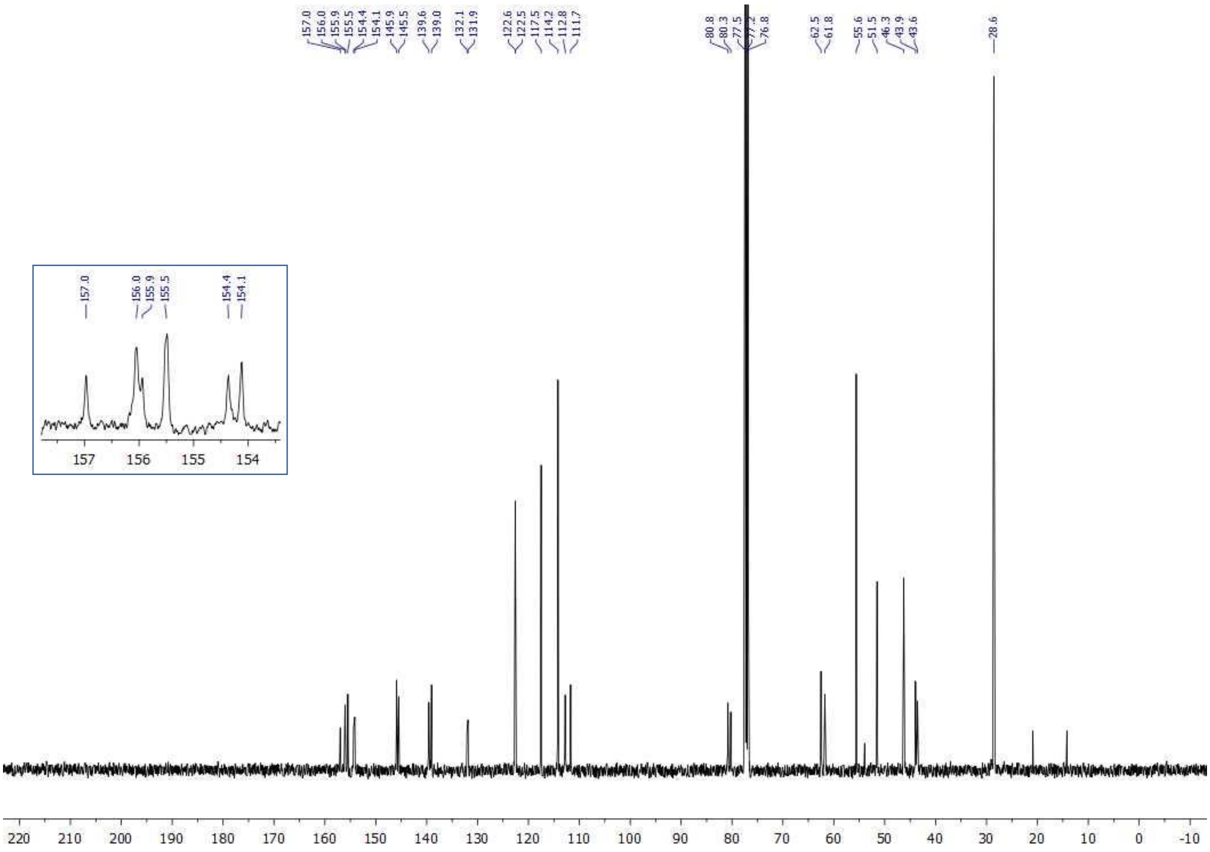


**Figure S165 – $^{13}$C NMR spectrum of 4b-7 (101 MHz, $CDCl_3$).**

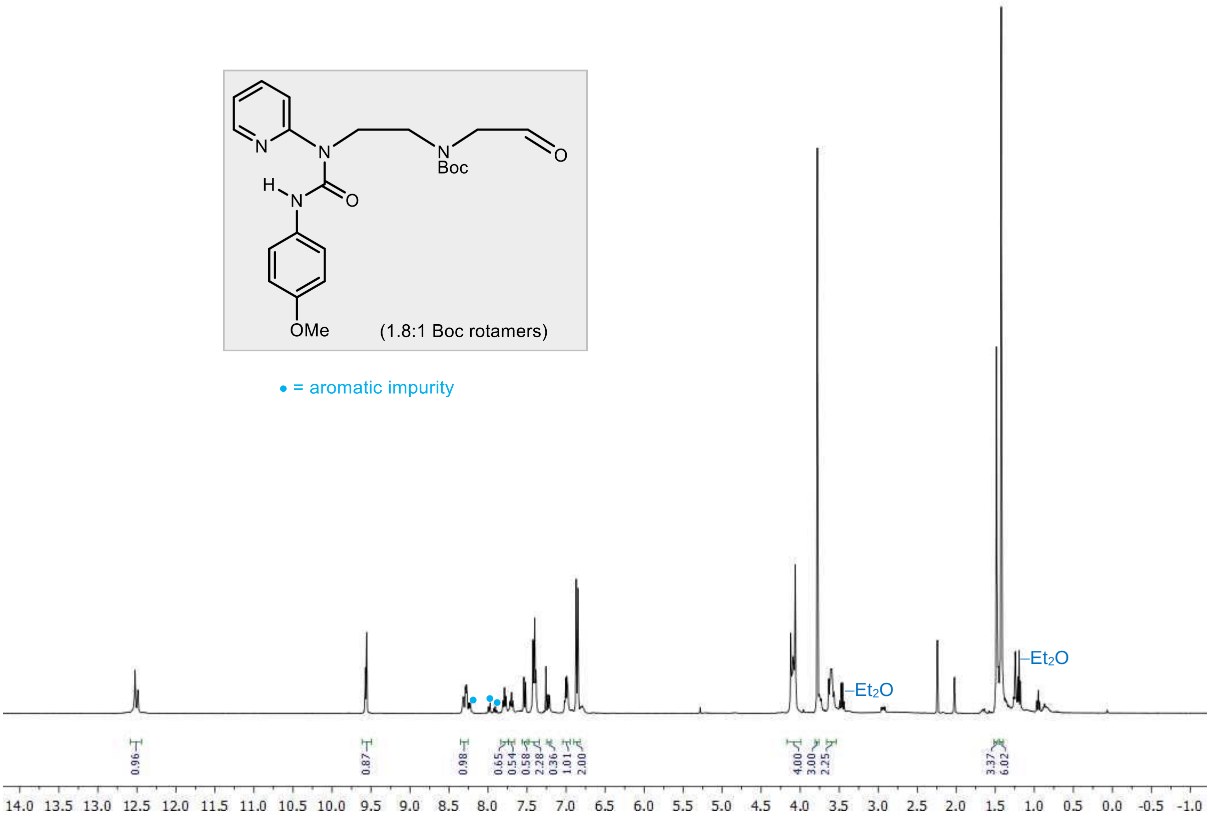


**Figure S166 – $^{1}$H NMR spectrum of 4b-8 (400 MHz, $CDCl_3$).**

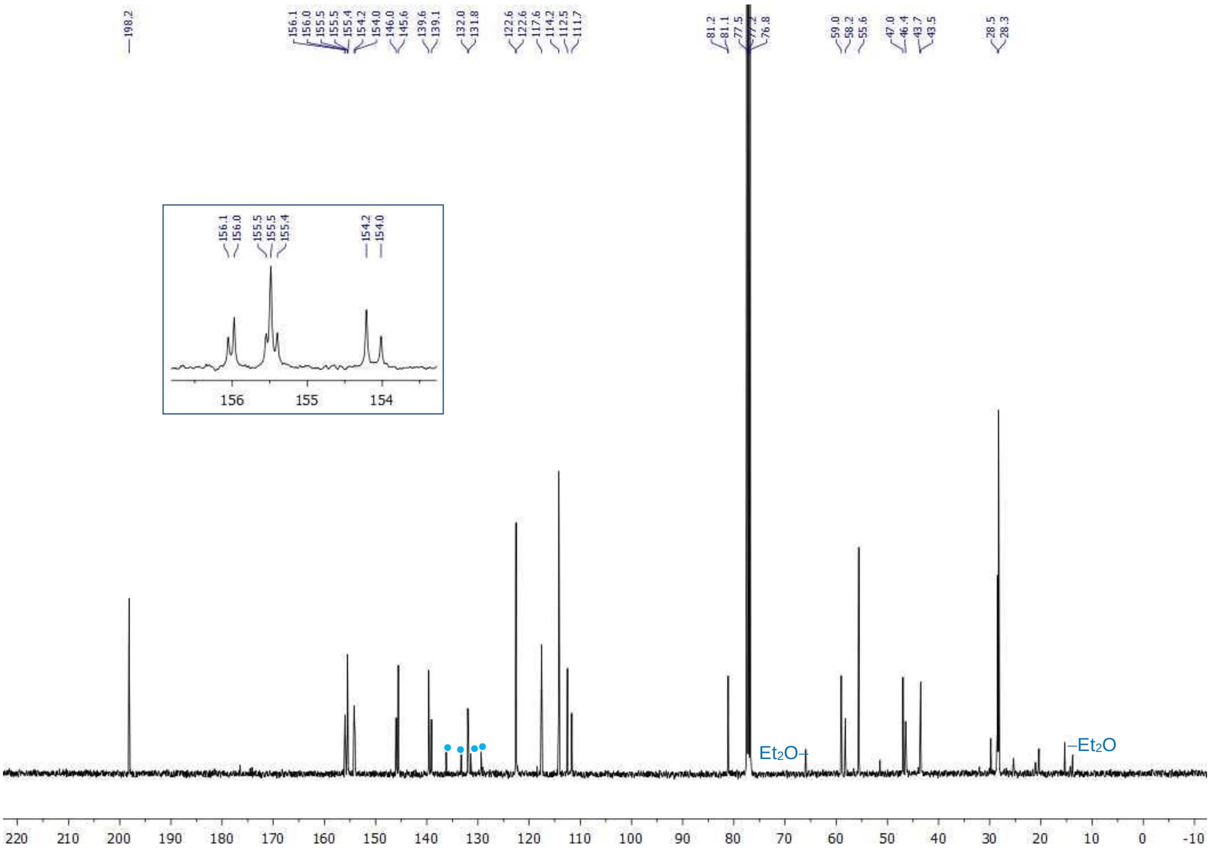


**Figure S167 – $^{13}$C NMR spectrum of 4b-8 (101 MHz, $CDCl_3$).**

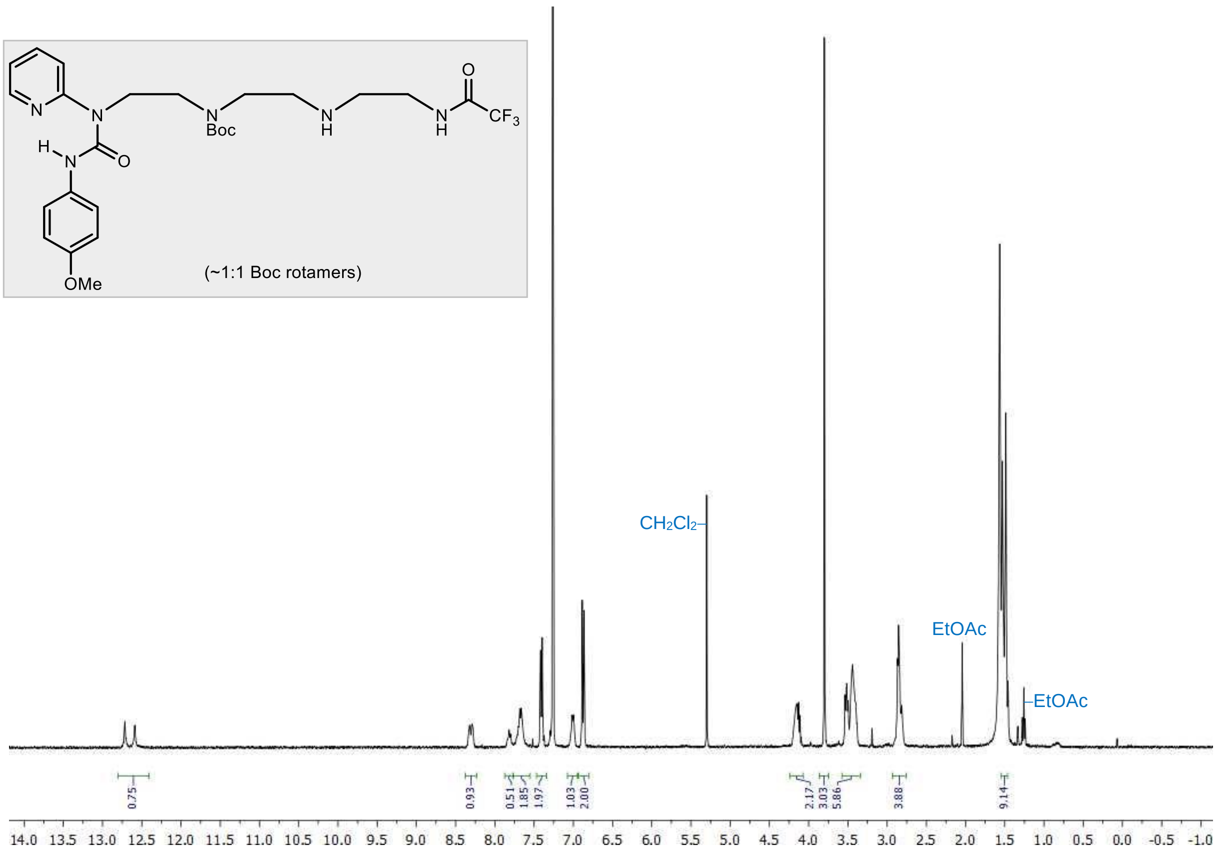


**Figure S168 – ¹H NMR spectrum of 4b-9 (400 MHz, $CDCl_3$).**

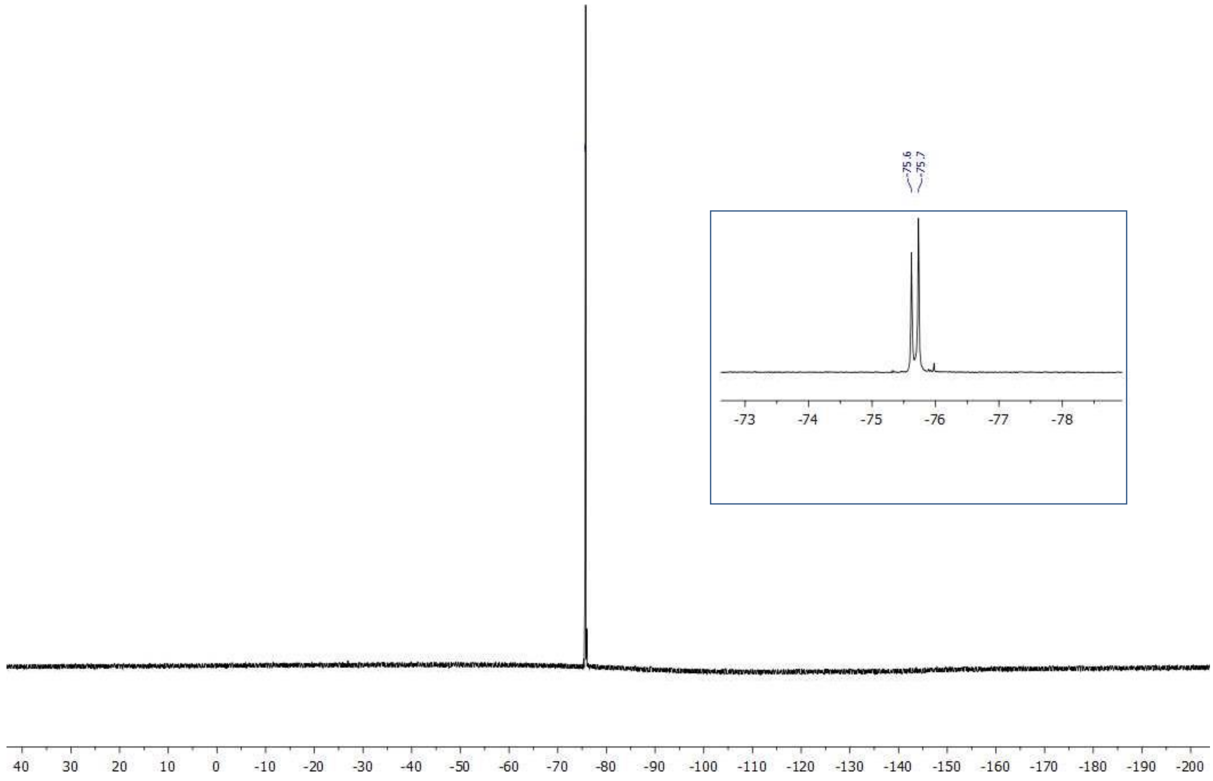


**Figure S169 – ¹⁹F NMR spectrum of 4b-9 (376 MHz, $CDCl_3$).**

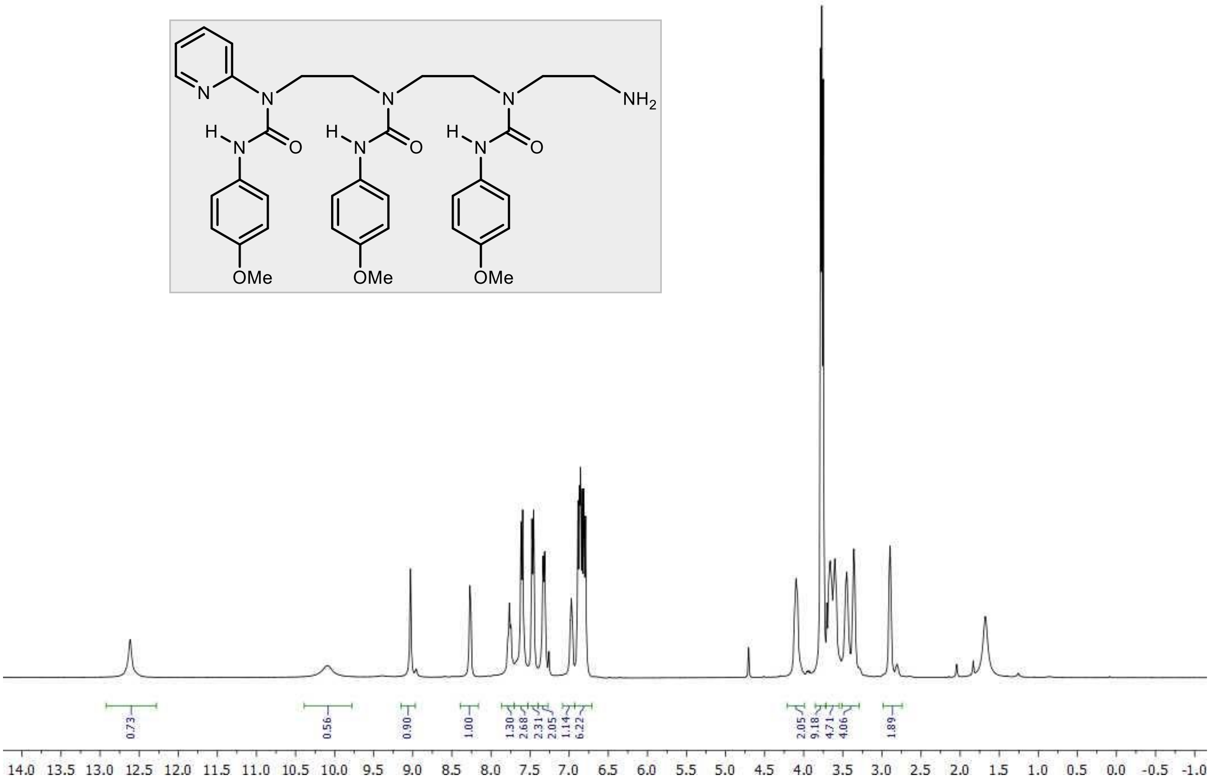

**Figure S170 – $^{1}$H NMR spectrum of 4b-10 (400 MHz, $CDCl_3$).**

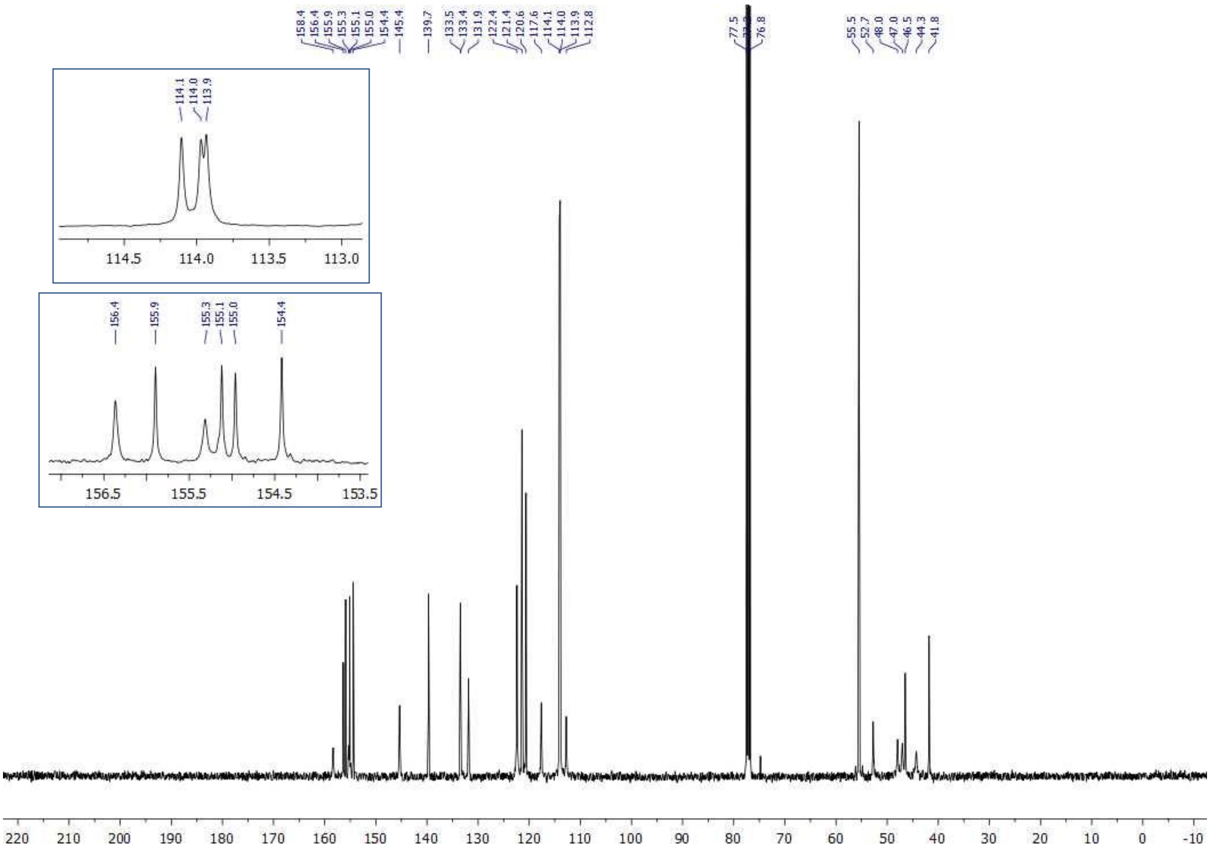

**Figure S171 – $^{13}$C NMR spectrum of 4b-10 (101 MHz, $CDCl_3$).**

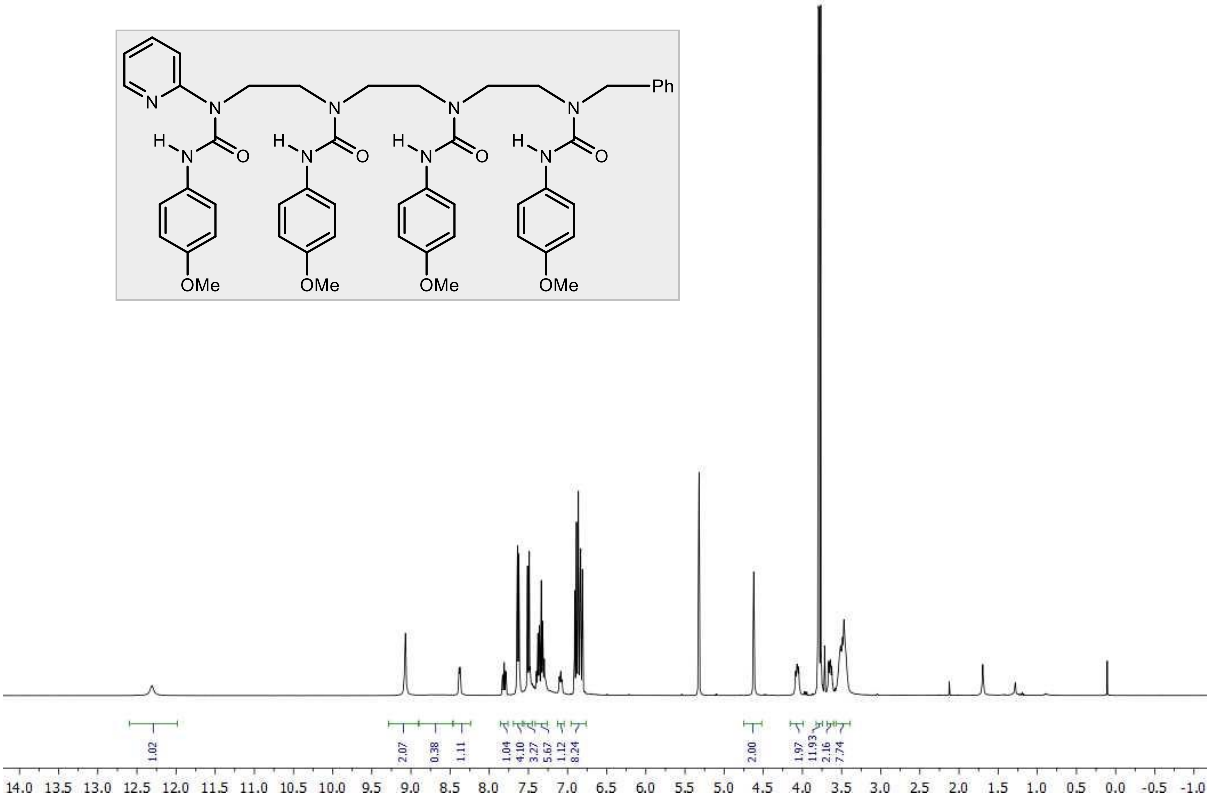


**Figure S172 – $^{1}$H NMR spectrum of 4b (400 MHz, $CD_2Cl_2$).**

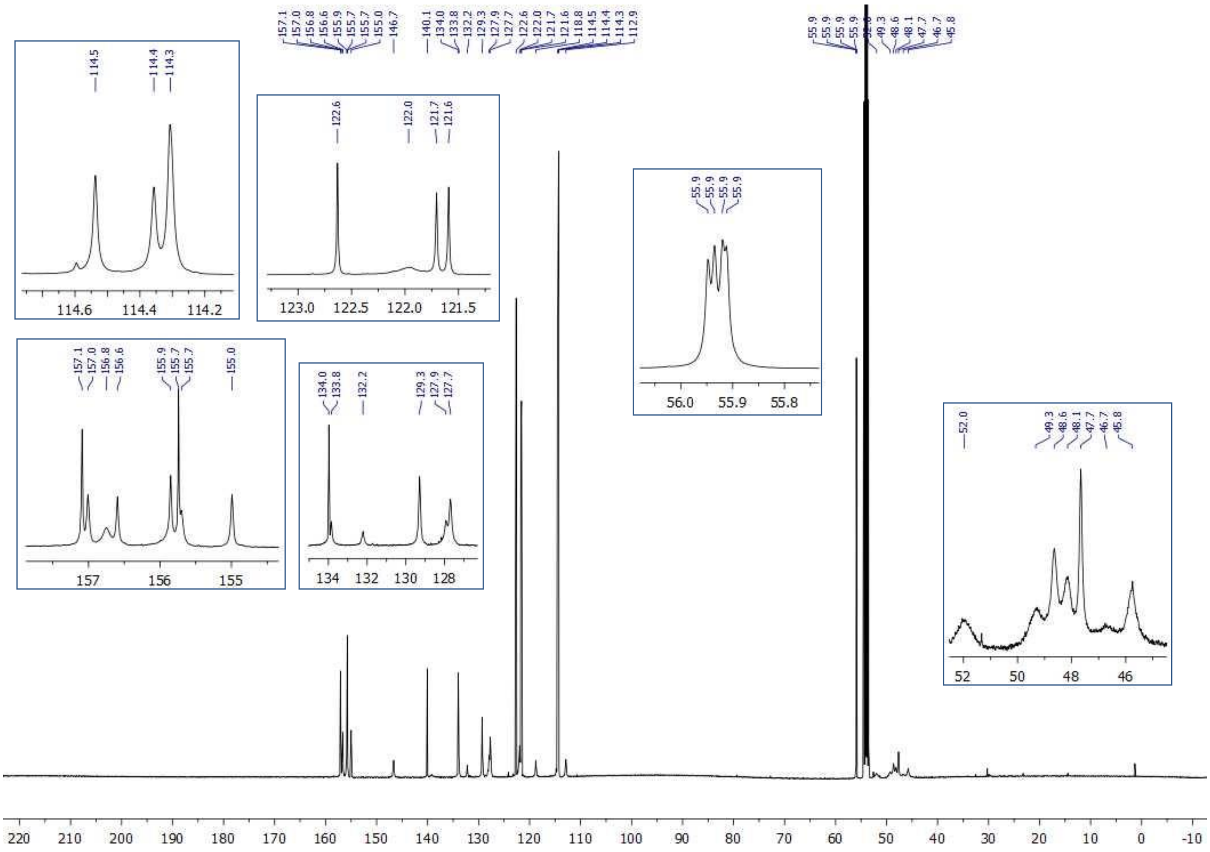


**Figure S173 – $^{13}$C NMR spectrum of 4b (101 MHz, $CD_2Cl_2$).**

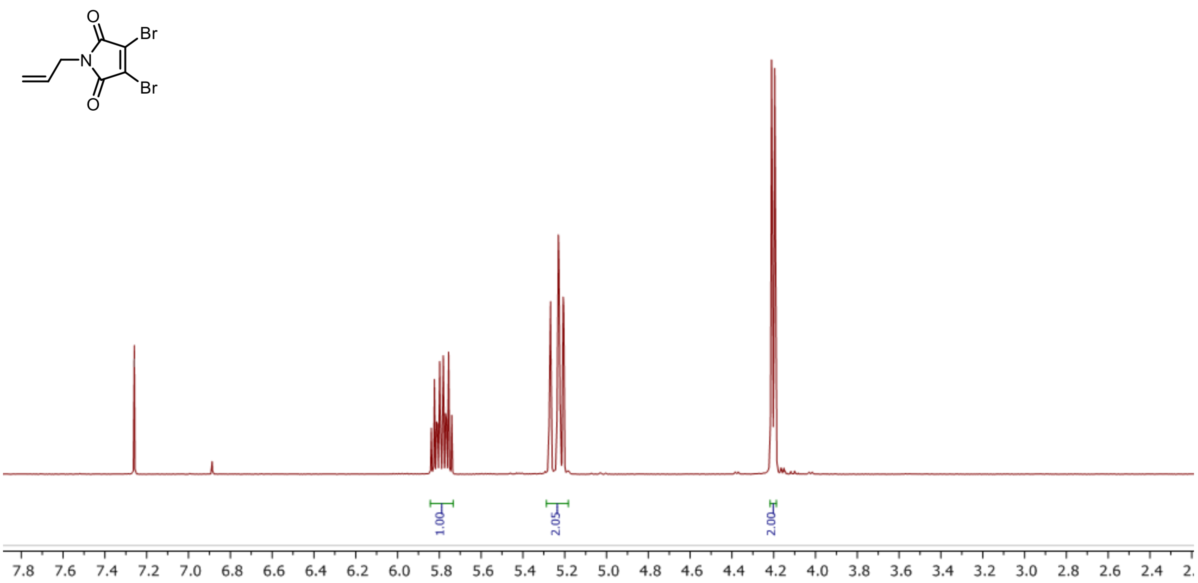


**Figure S174 – $^1$H NMR spectrum of 7a-2 (400 MHz, $CDCl_3$).**

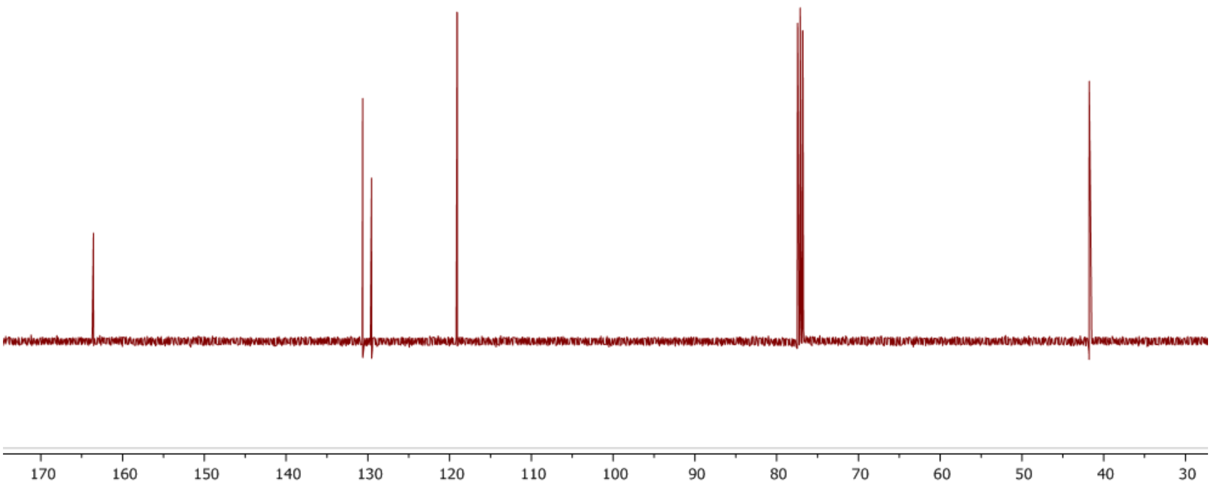


**Figure S175 – $^{13}$C NMR spectrum of 7a-2 (101 MHz, $CDCl_3$).**

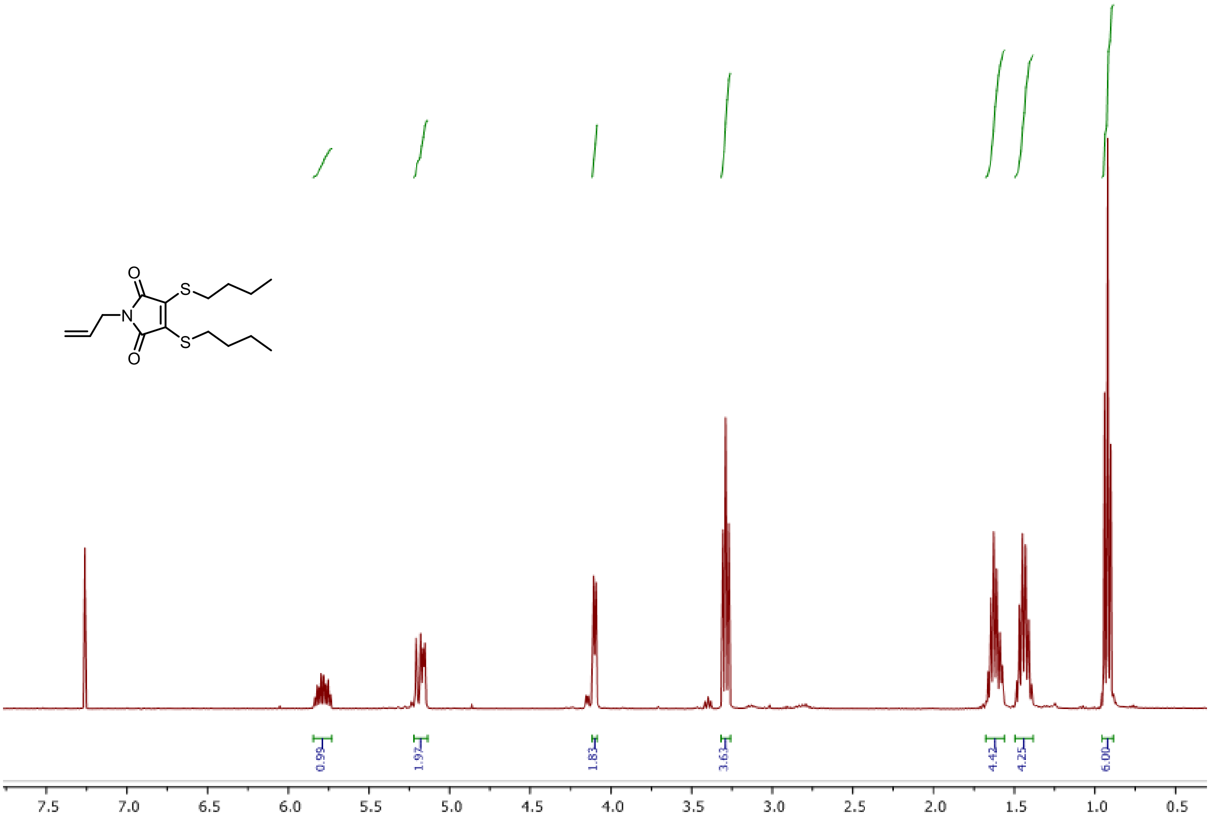


**Figure S176 – $^{1}$H NMR spectrum of 7a (400 MHz, $CDCl_3$).**

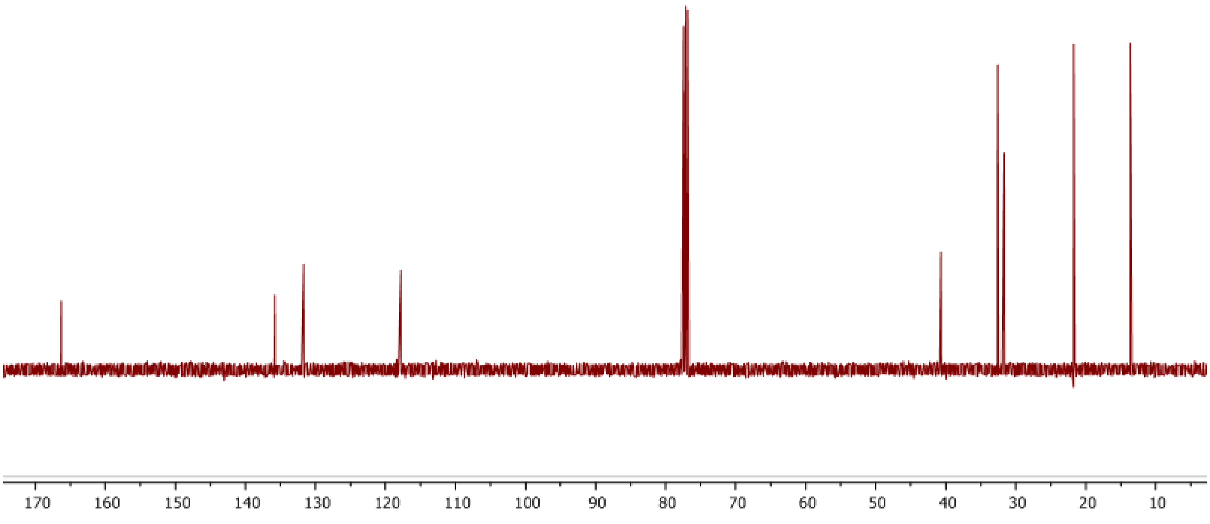


**Figure S177 – $^{13}$C NMR spectrum of 7a (101 MHz, $CDCl_3$).**

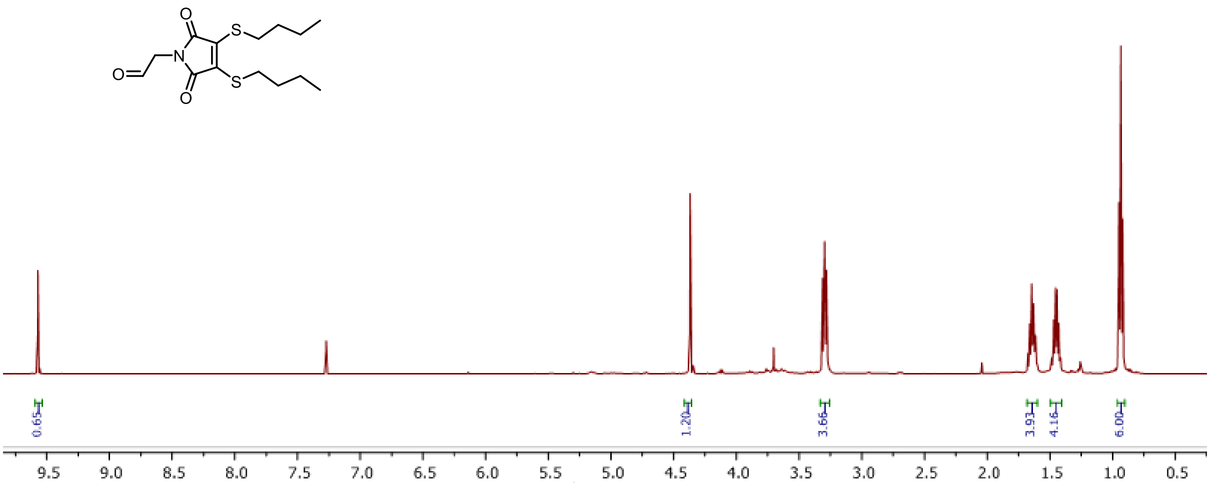


**Figure S178 – $^{1}$H NMR spectrum of 7a-3 (400 MHz, $CDCl_3$).**

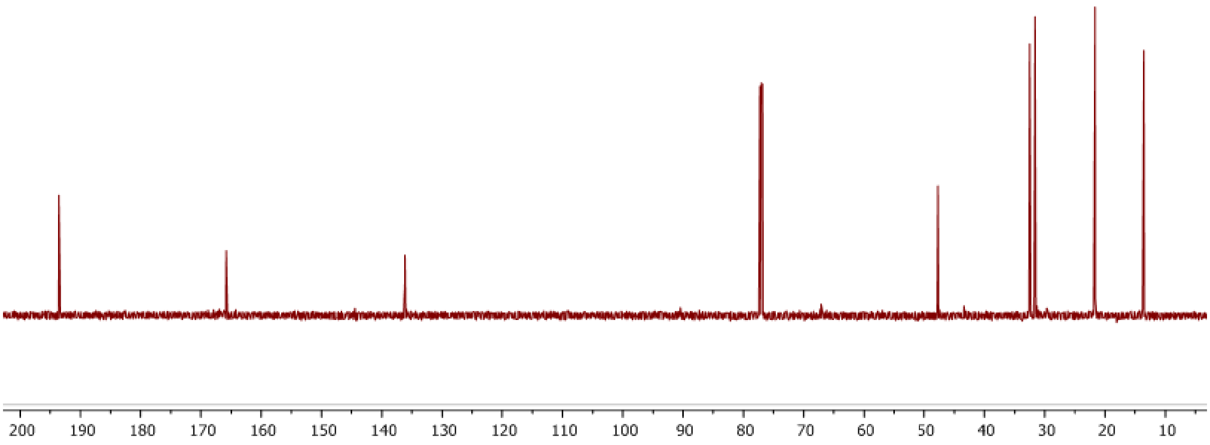


**Figure S179 – $^{13}$C NMR spectrum of 7a-3 (101 MHz, $CDCl_3$).**

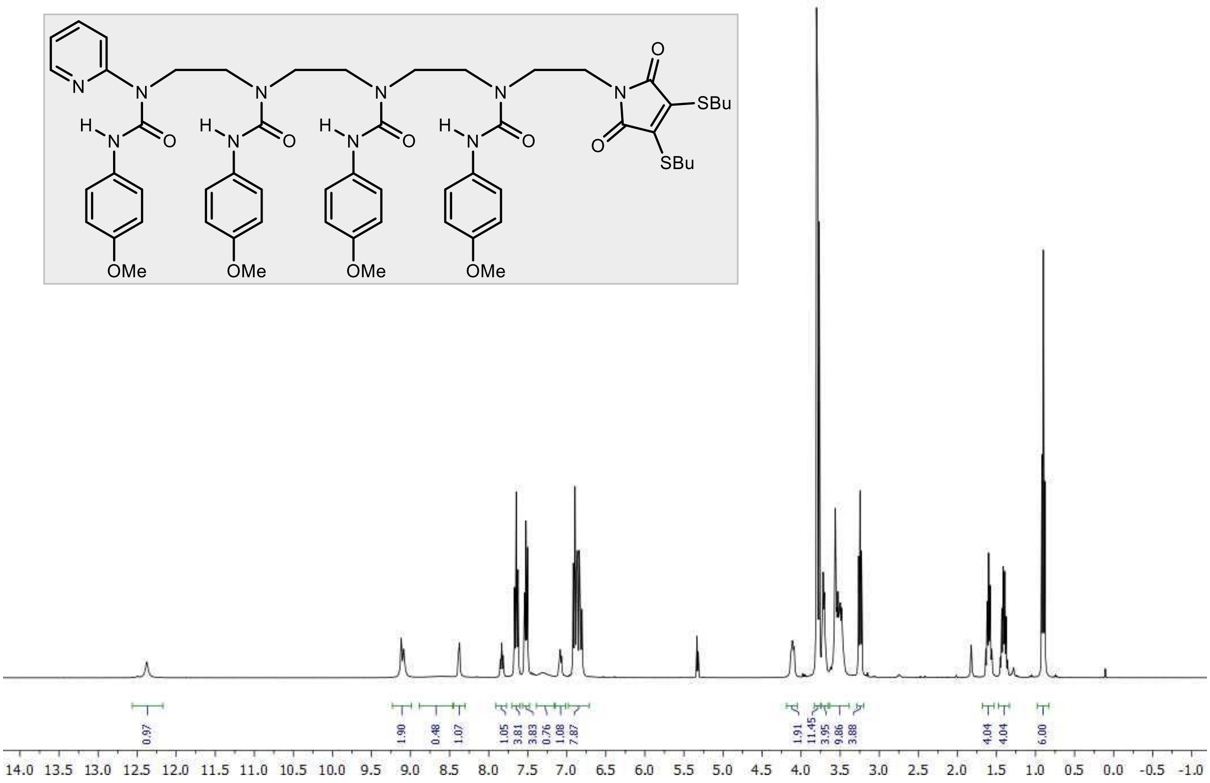

**Figure S180 – $^1$H NMR spectrum of 8a (400 MHz, $CD_2Cl_2$).**

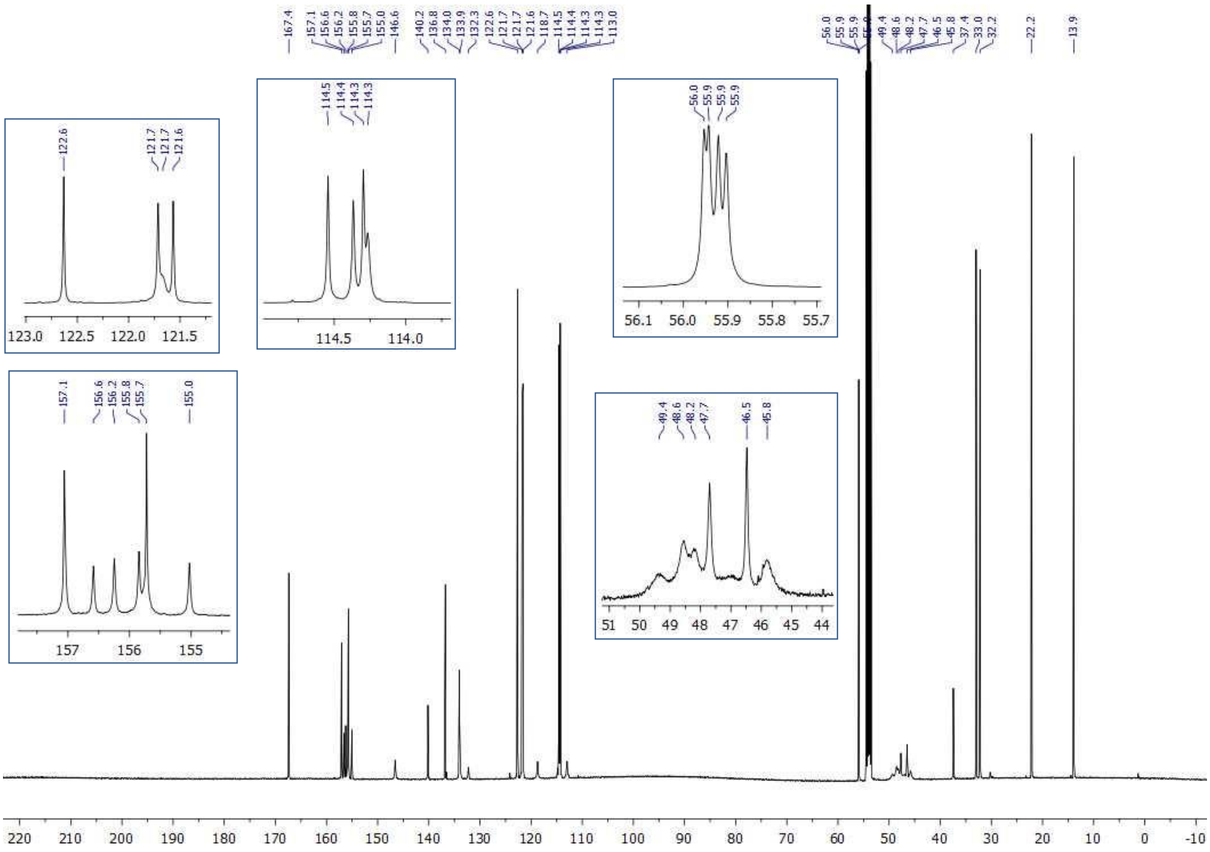

**Figure S181 – $^{13}$C NMR spectrum of 8a (126 MHz, $CD_2Cl_2$).**

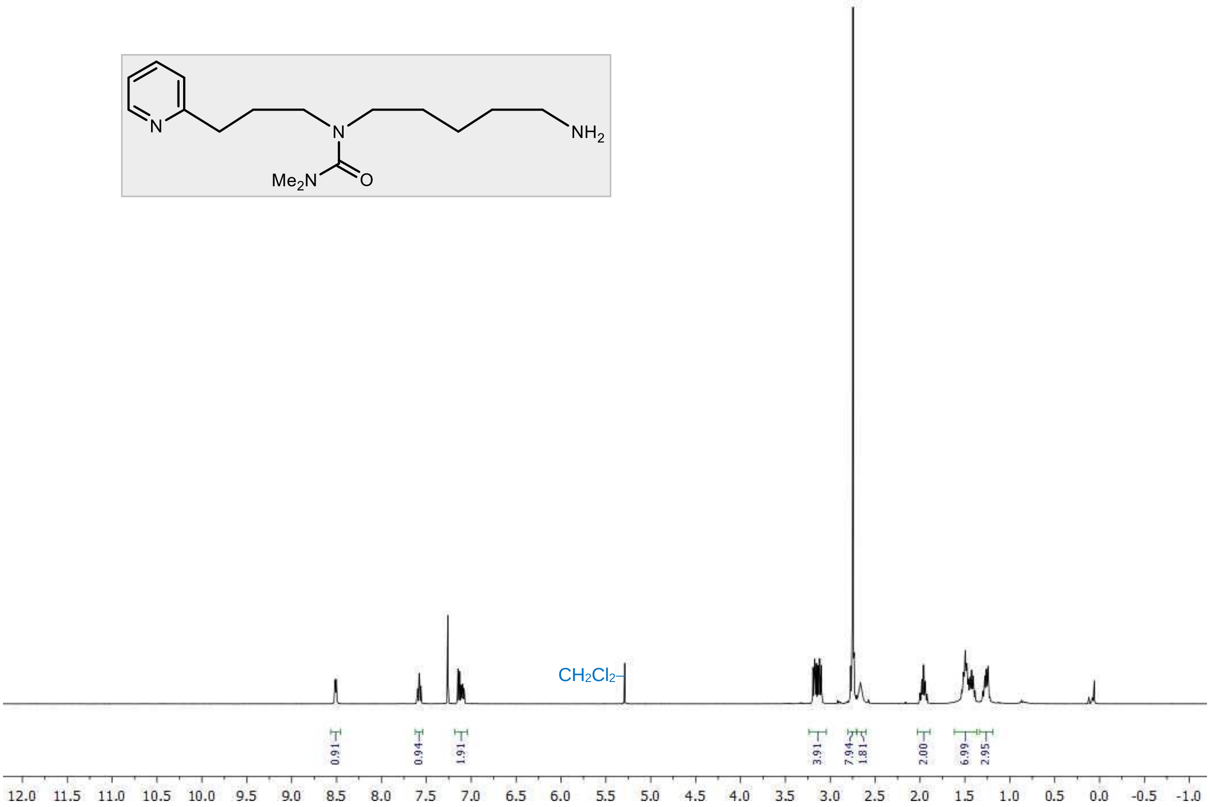


**Figure S182 – $^{1}$H NMR spectrum of 8b-1 (400 MHz, $CDCl_3$).**

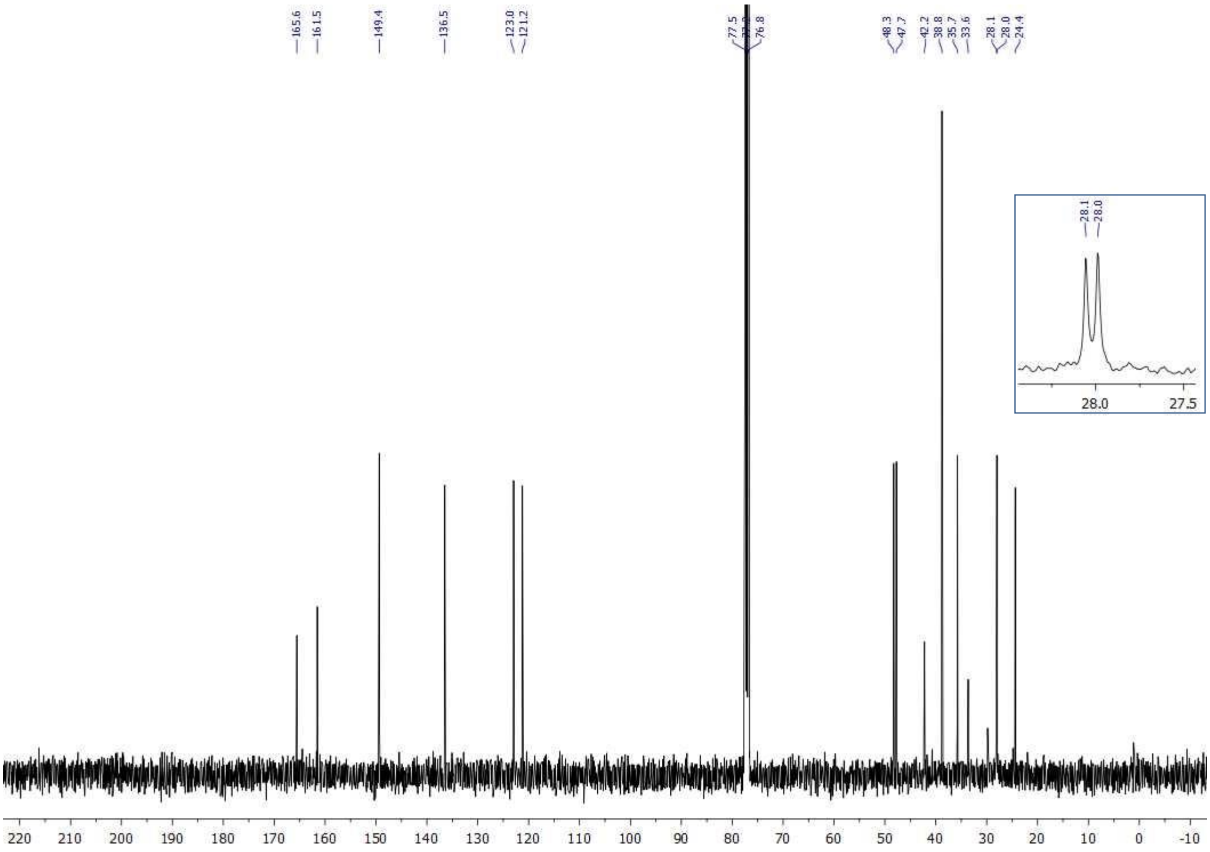


**Figure S183 – $^{13}$C NMR spectrum of 8b-1 (101 MHz, $CDCl_3$).**

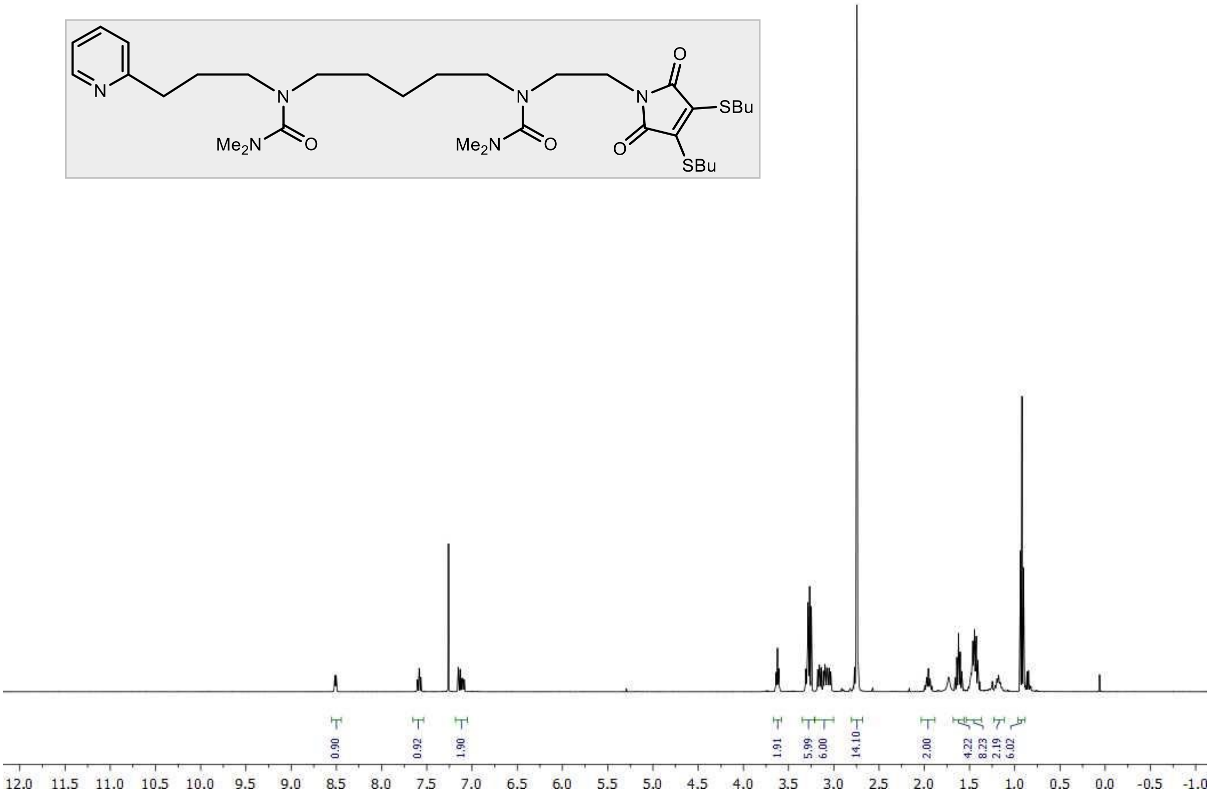

**Figure S184 – $^{1}$H NMR spectrum of 8b (400 MHz, $CDCl_3$).**

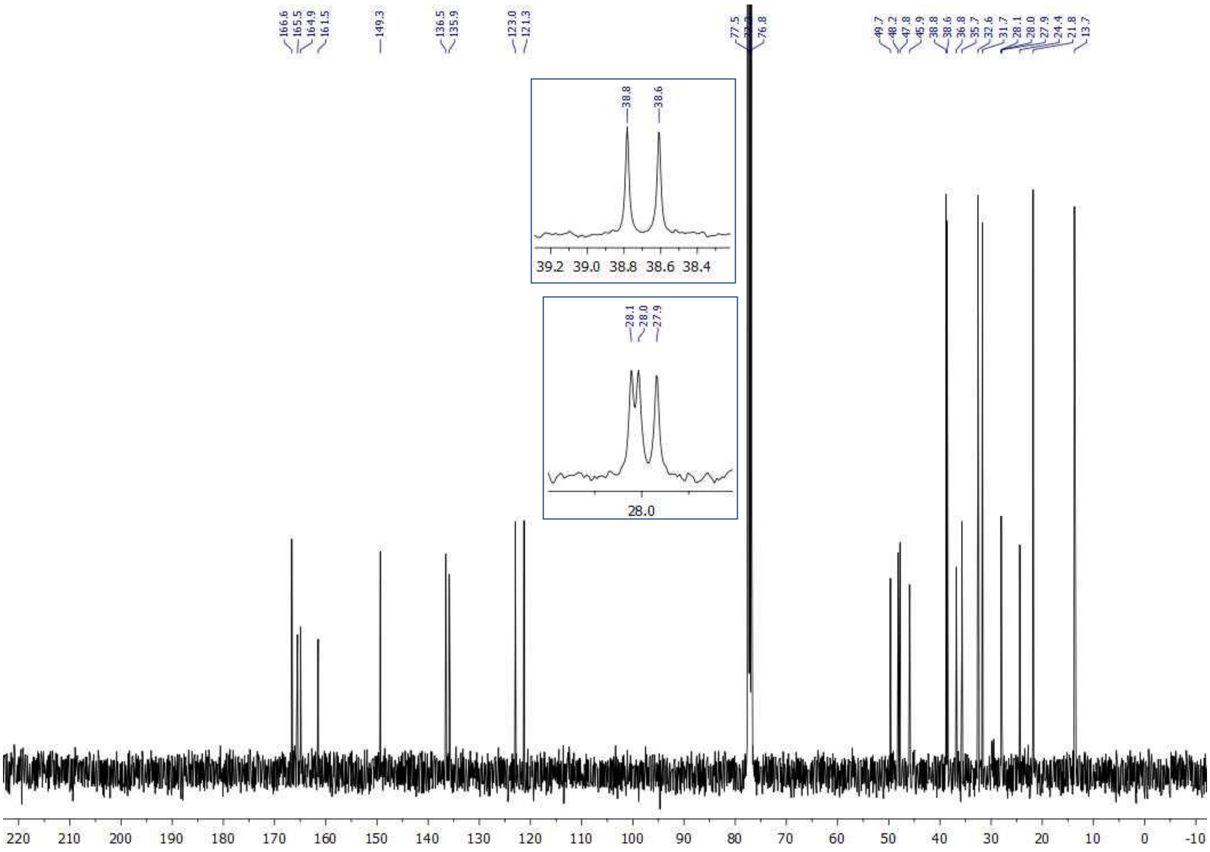

**Figure S185 – $^{13}$C NMR spectrum of 8b (101 MHz, $CDCl_3$).**

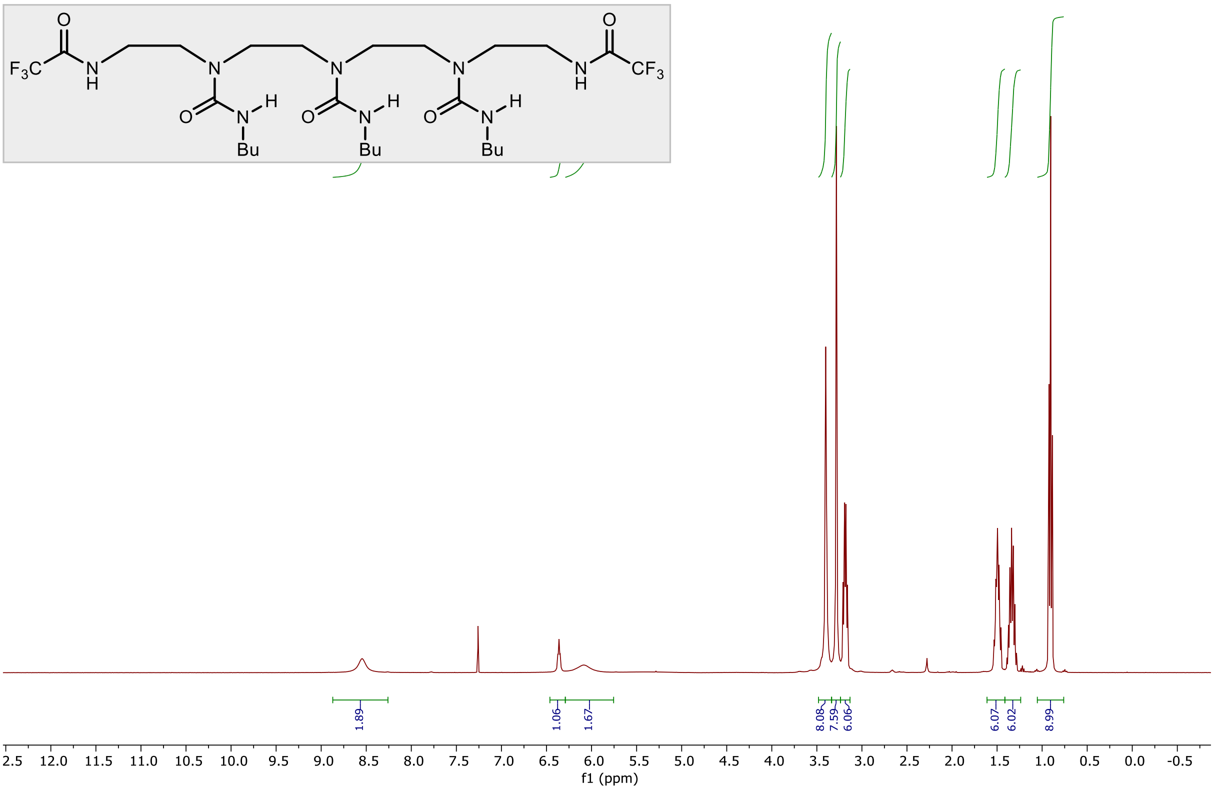


**Figure S186 – $^{1}$H NMR spectrum of 9-2 (400 MHz, $CDCl_3$).**

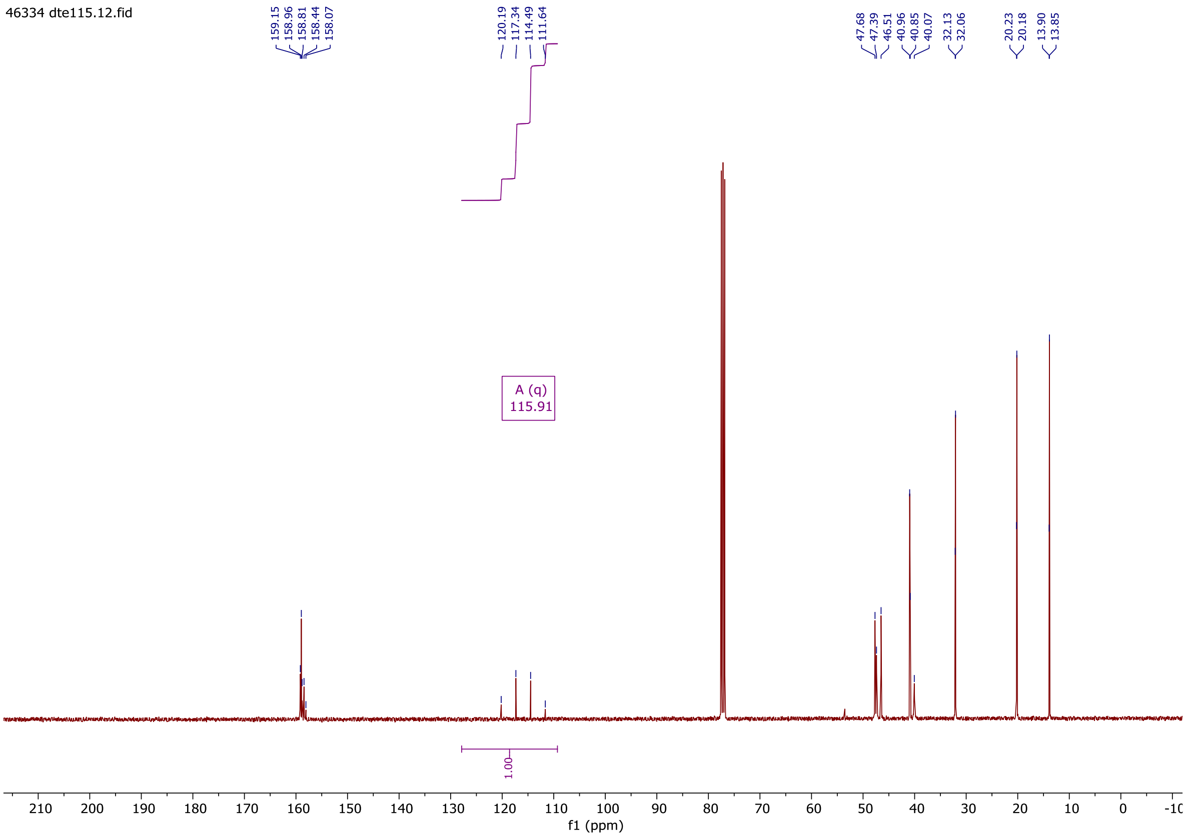


**Figure S187 – $^{13}$C NMR spectrum of 9-2 (101 MHz, $CDCl_3$).**

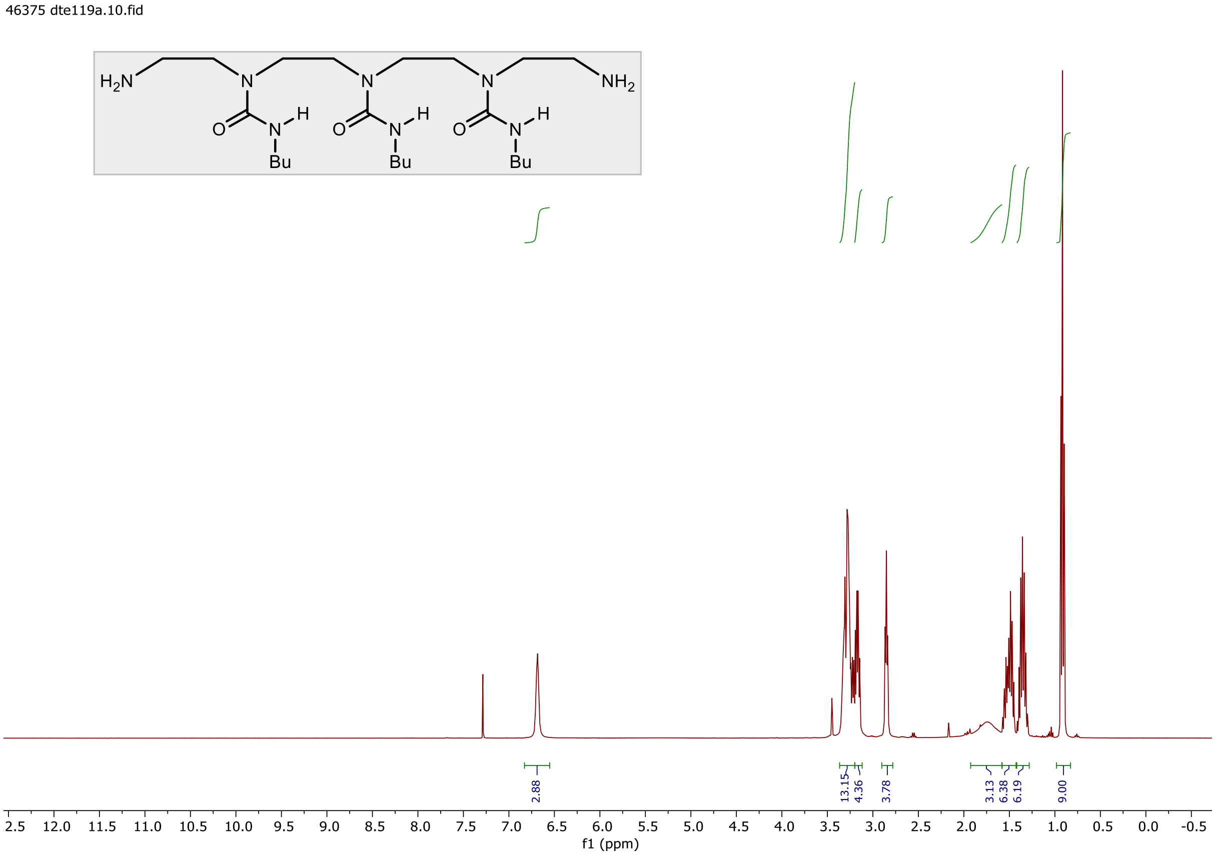


**Figure S188 – $^{1}$H NMR spectrum of 9-3 (400 MHz, $CDCl_3$).**

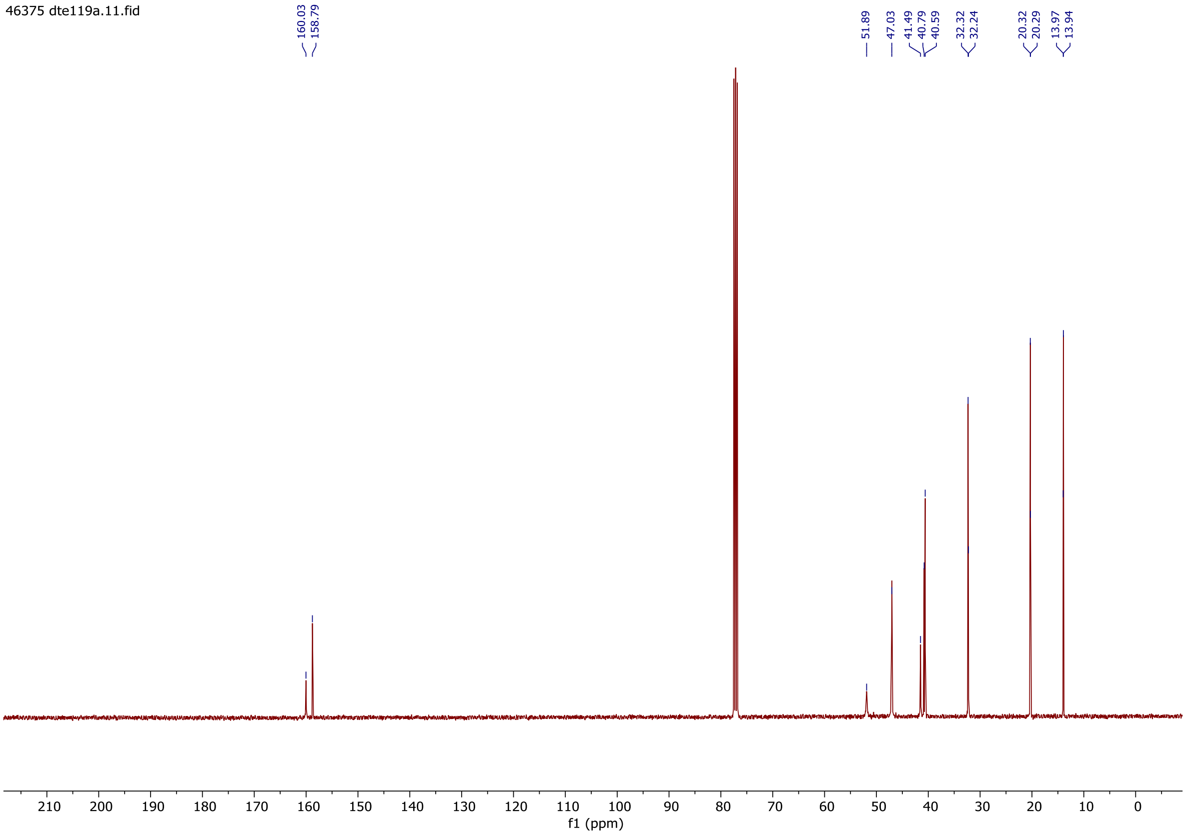


**Figure S189 – $^{13}$C NMR spectrum of 9-3 (101 MHz, $CDCl_3$).**

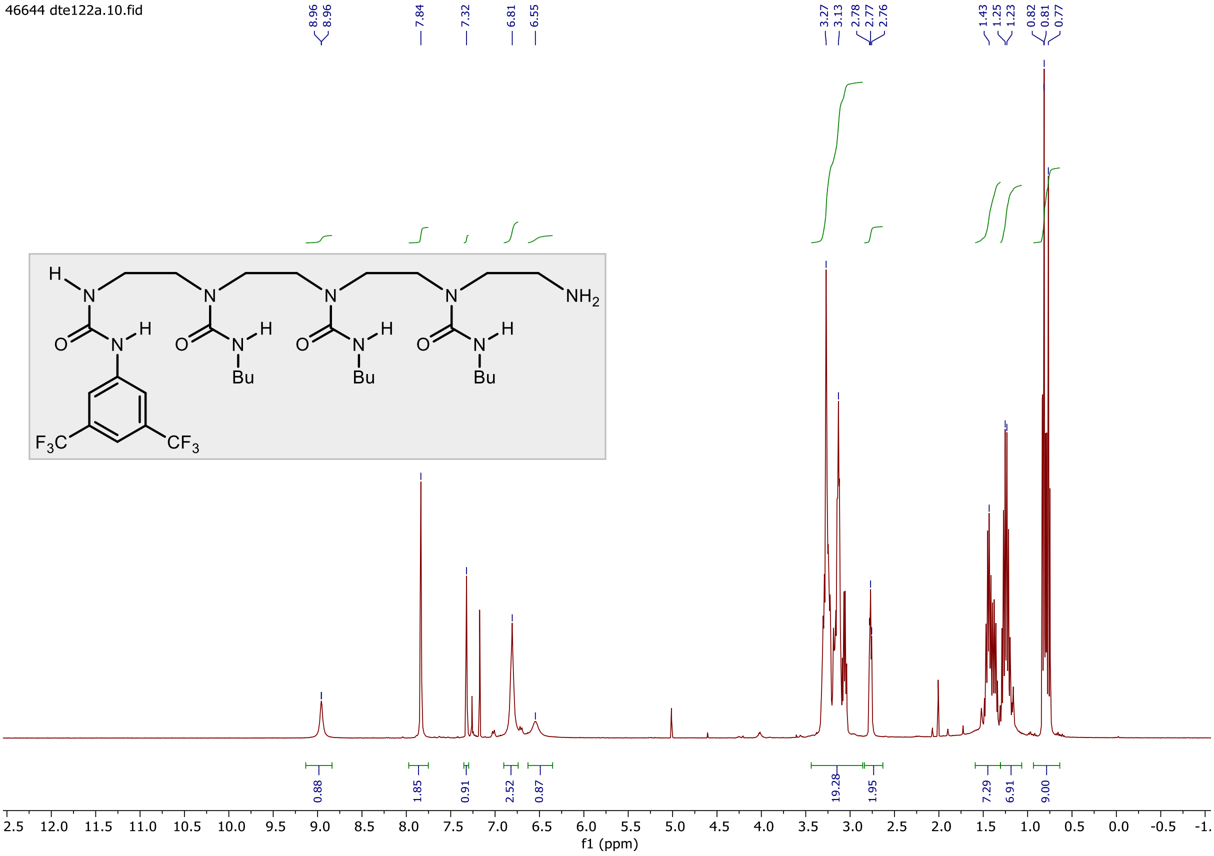


**Figure S190 – $^{1}$H NMR spectrum of 9-4 (400 MHz, CDCl$_3$).**

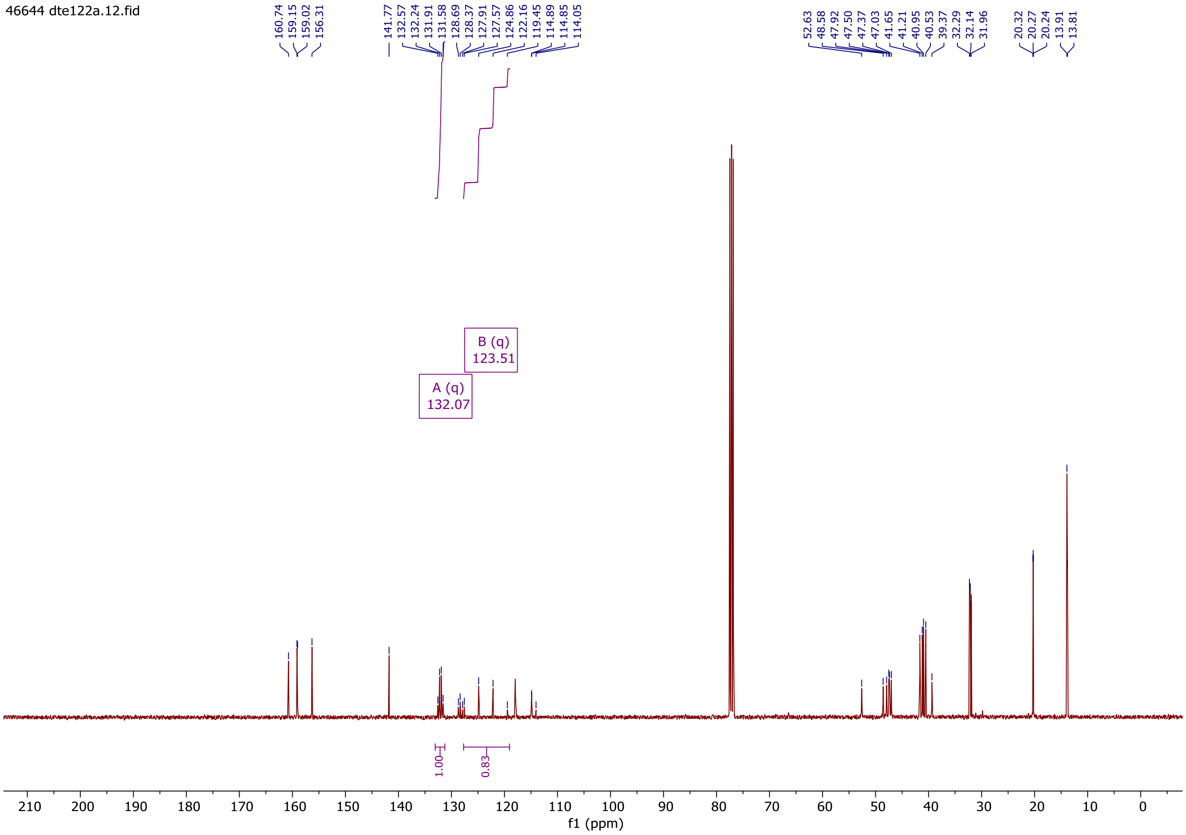


**Figure S191 – $^{13}$C NMR spectrum of 9-4 (101 MHz, CDCl$_3$).**

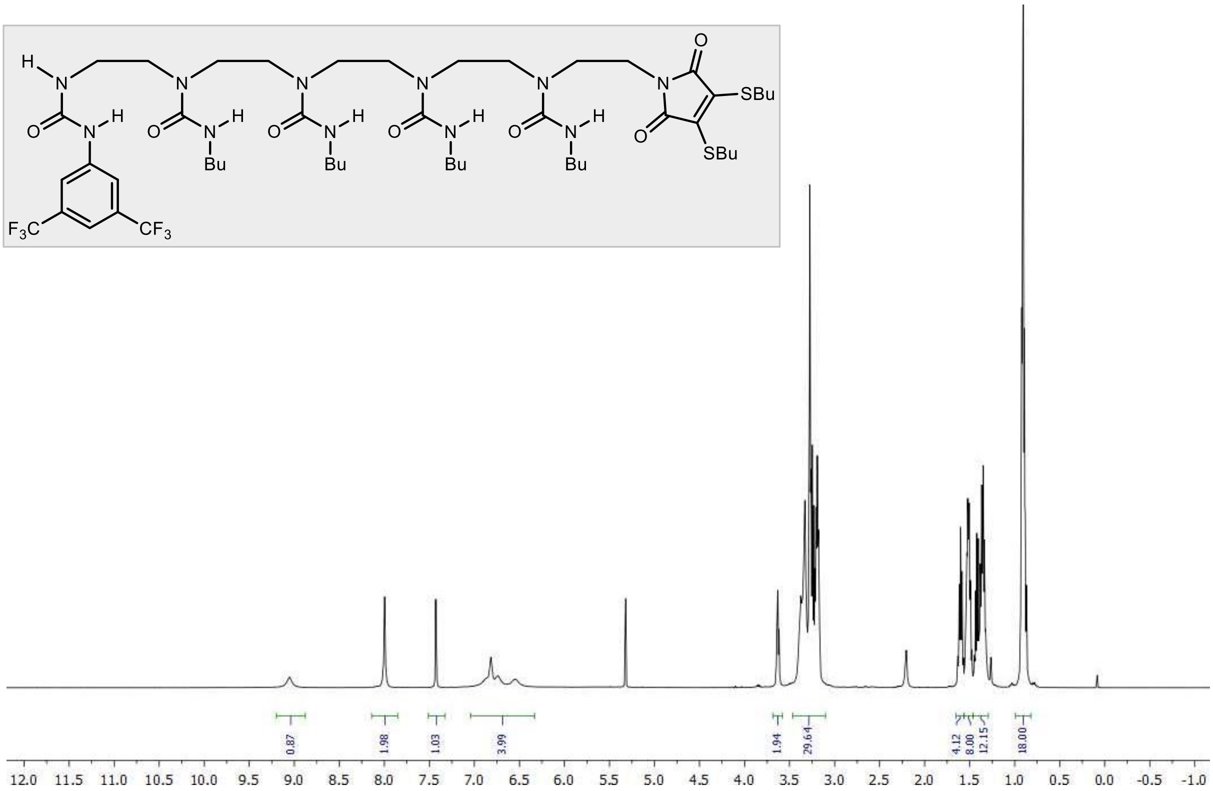

**Figure S192 – $^{1}$H NMR spectrum of 9 (500 MHz, $CD_2Cl_2$).**

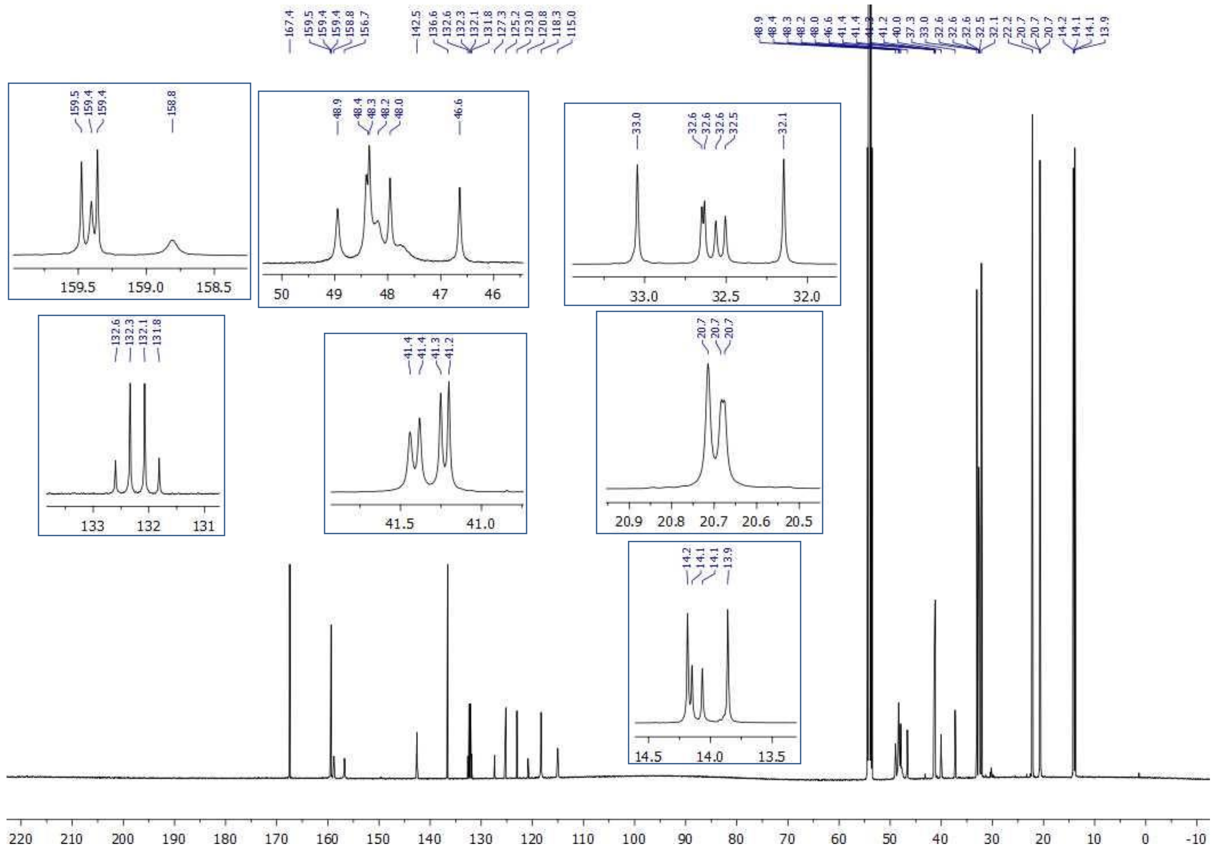

**Figure S193 – $^{13}$C NMR spectrum of 9 (126 MHz, $CD_2Cl_2$).**

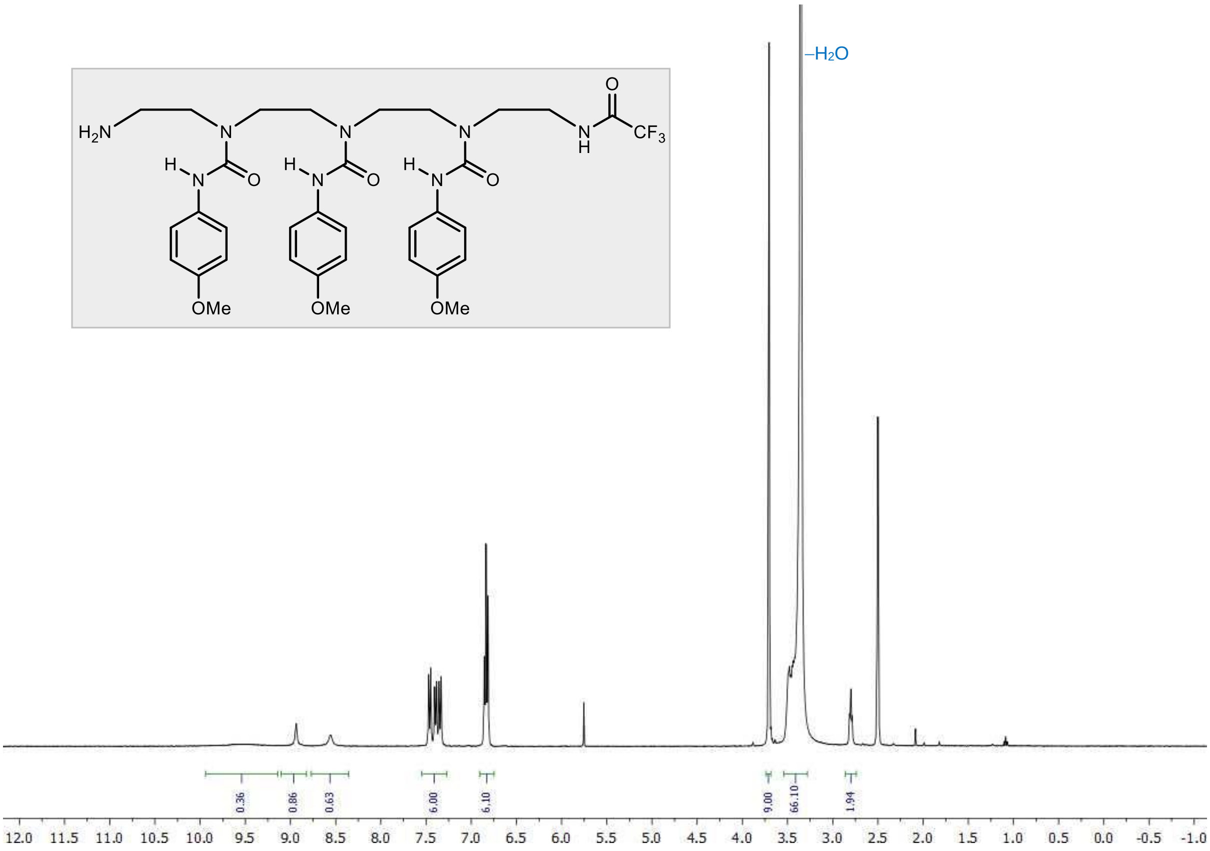


**Figure S194 – $^{1}$H NMR spectrum of 10-1 (400 MHz, $(CD_3)_2SO$).**

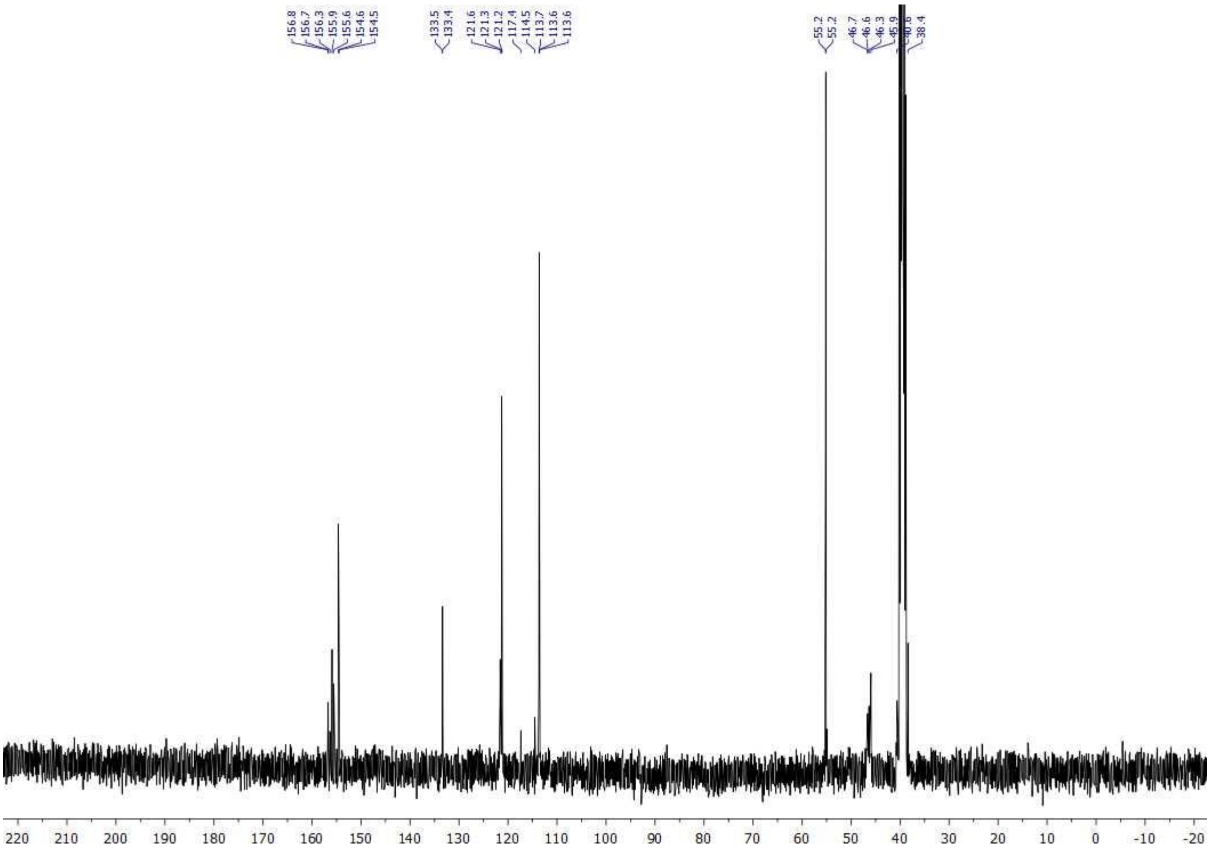


**Figure S195 – $^{13}$C NMR spectrum of 10-1 (101 MHz, $(CD_3)_2SO$).**

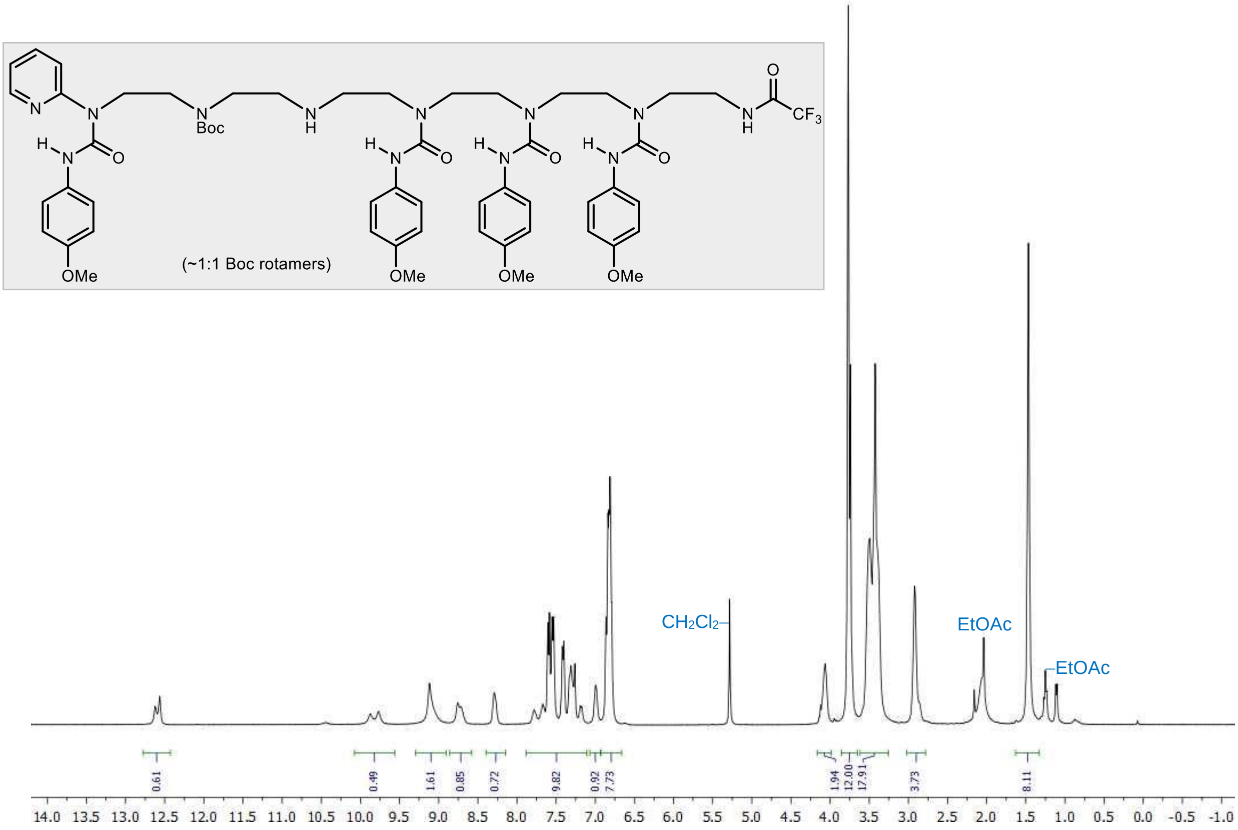


**Figure S196 – $^{1}$H NMR spectrum of 10-2 (400 MHz, $CDCl_3$).**

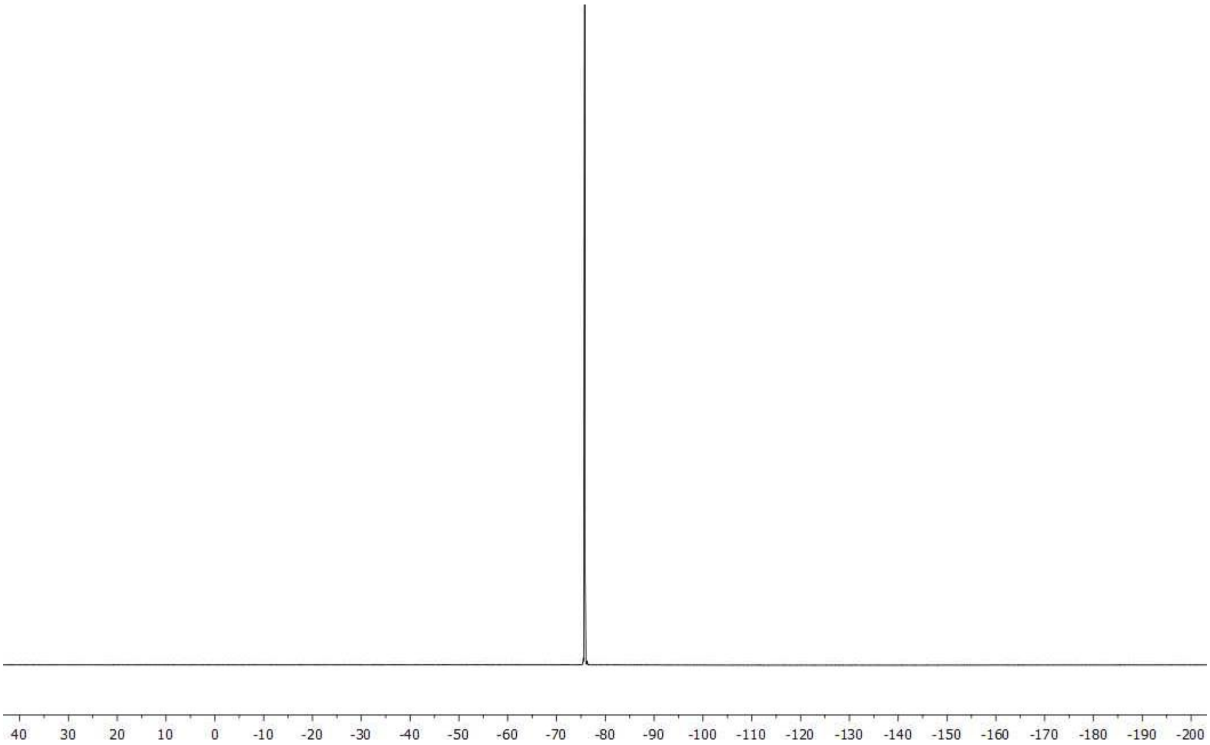


**Figure S197 – $^{19}$F NMR spectrum of 10-2 (376 MHz, $CDCl_3$).**

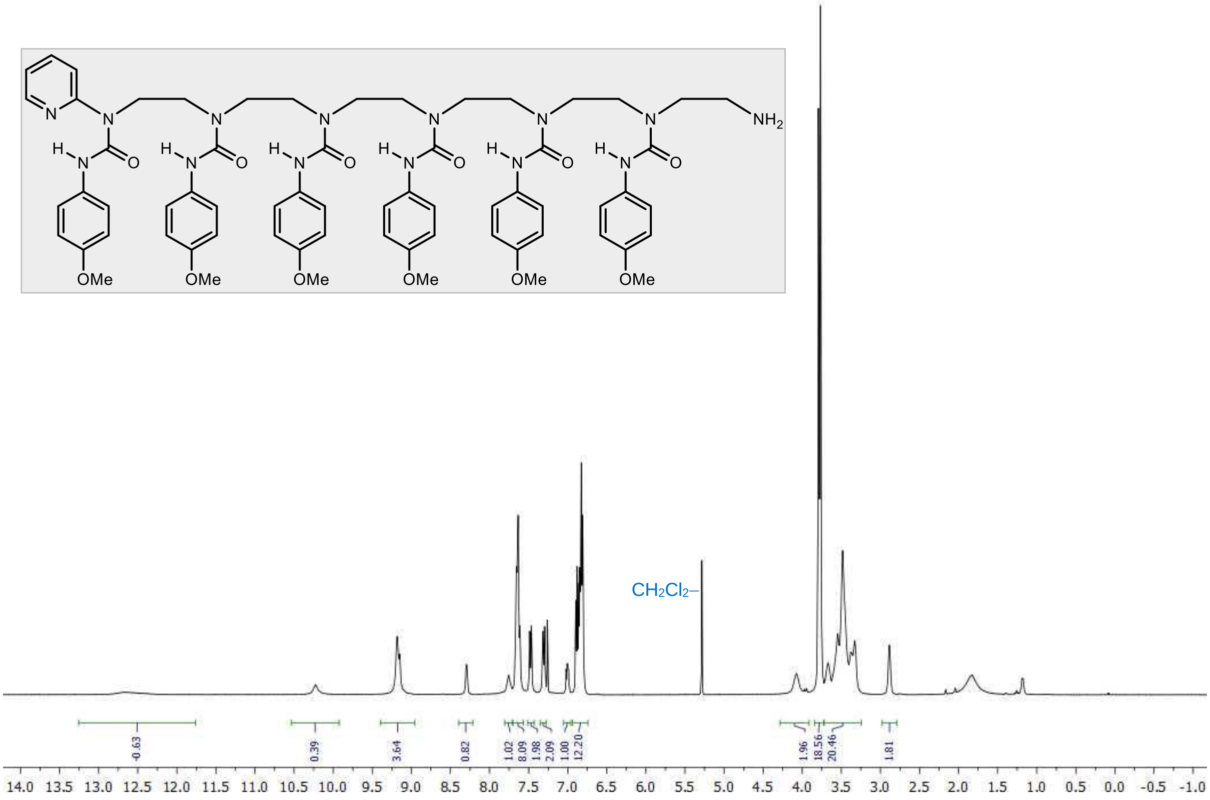


**Figure S198 – $^{1}$H NMR spectrum of 10-3 (400 MHz, $CDCl_3$).**

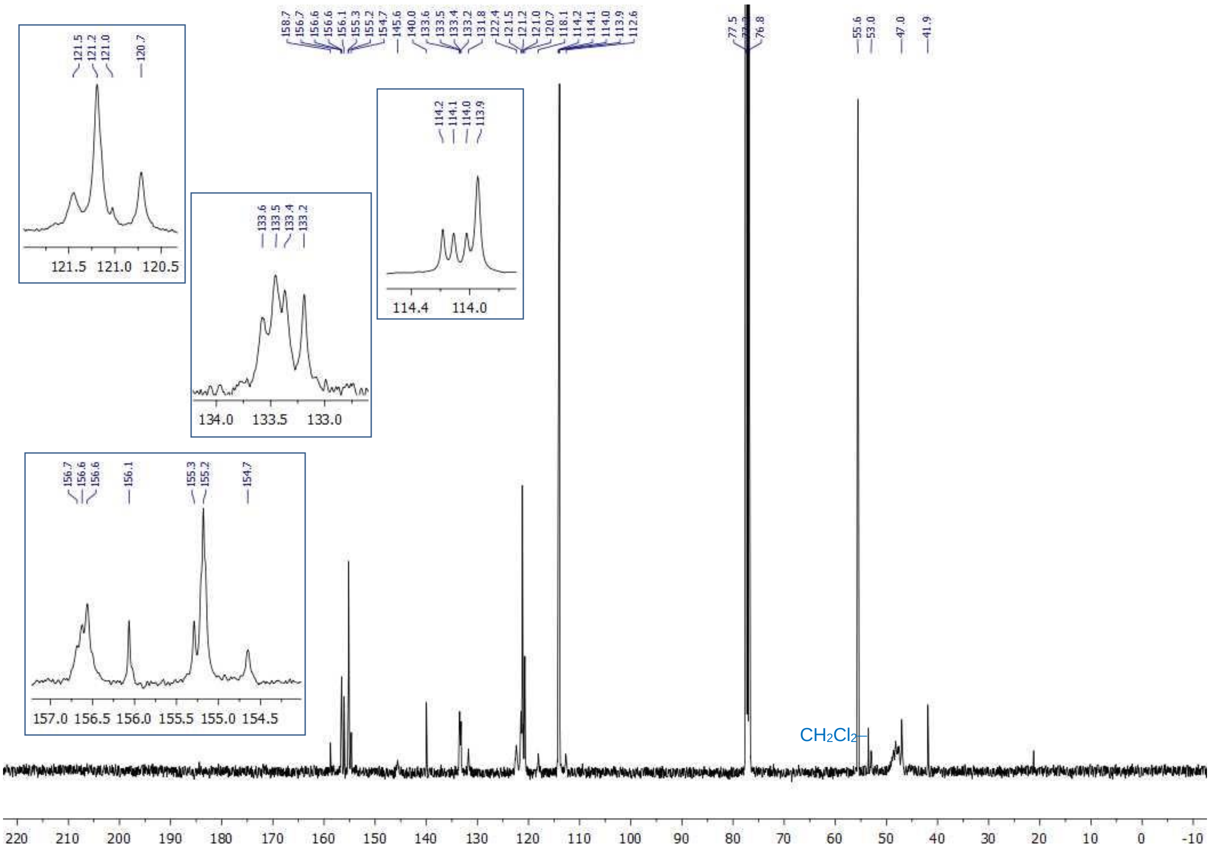


**Figure S199 – $^{13}$C NMR spectrum of 10-3 (101 MHz, $CDCl_3$).**

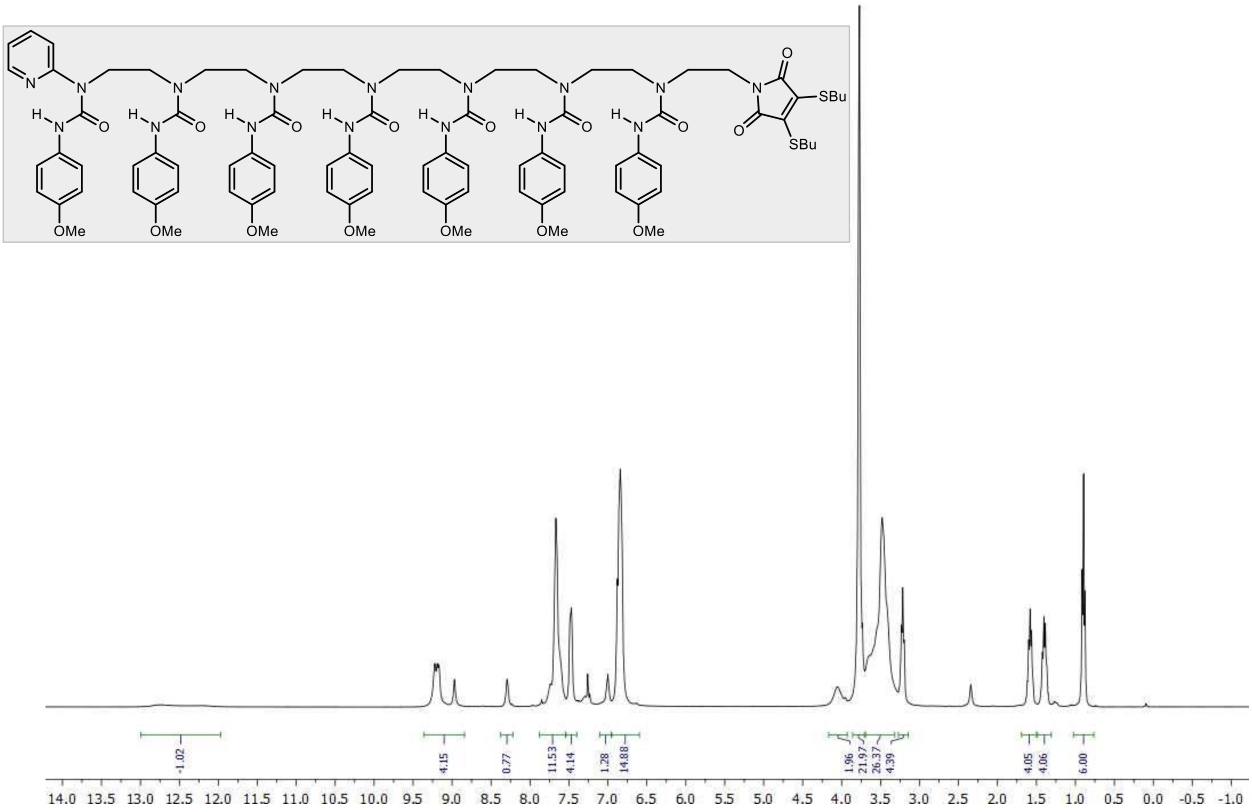

**Figure S200 – $^{1}$H NMR spectrum of 10 (400 MHz, $CDCl_3$).**

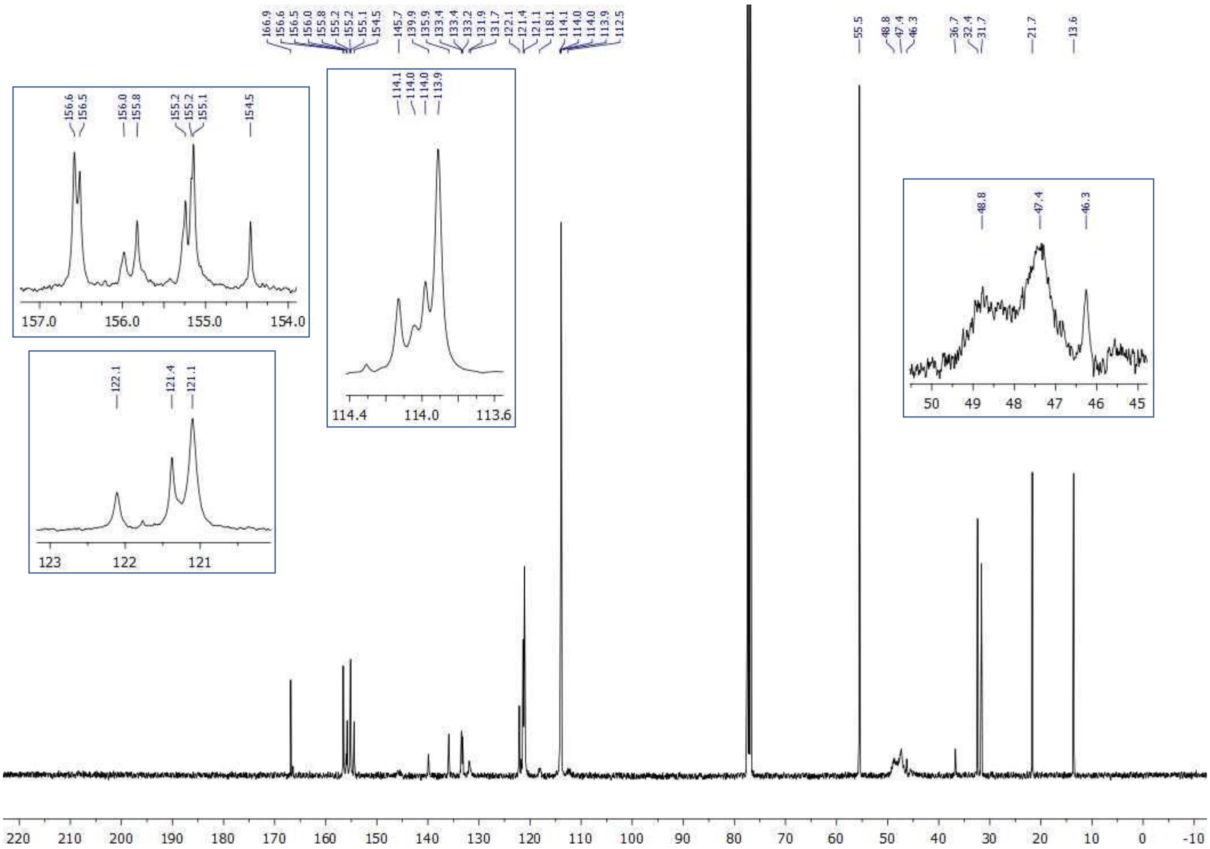

**Figure S201 – $^{13}$C NMR spectrum of 10 (101 MHz, $CDCl_3$).**

Computational Details

Conformational searches were carried out for all computed structures using the conformational search tool within Schrödinger's MacroModel (version 11.3)[11,12] with the OPLS3e force field.[13] A Monte Carlo Multiple Minimum (MCMM)[14] / low-mode sampling approach[15] was used to explore the possible conformations of each species. Conformations provided by these searches were subsequently optimised by DFT calculations carried out using Gaussian16 (Revision A.03)[16] with the B3LYP density functional,[17,18] dispersion-corrected with the D3 version of Grimme's dispersion[19] with Becke-Johnson damping (D3(BJ)),[20-22] and a split-valence polarised 6-31G(d,p) basis set.[23]

Single point energy (SPE) calculations were used to correct the Gibbs free energy derived from the original B3LYP calculations.[24] These were performed with an ultrafine integration grid using the B3LYP-D3(BJ) density functional and the larger split-valence double polarised 6-311G(d,p) basis set.[25] The integral equation formalism version of the polarisable continuum model (IEF-PCM)[26] (dichloromethane (DCM)) was used to incorporate the effect of solvent. All temperature (298.15 K, except where specified) and concentration-corrected (1 mol/l) quasiharmonic (Grimme approximation[27]) free energies were calculated with GoodVibes[28] with a vibrational scaling factor of 0.977.[29]

Similar methods have previously been used for the successful modelling of ureas.[30,31]

**Anti vs. gauche 9-membered ring conformation**

A simple system **S** (Figure S202a) containing two truncated urea units was modelled in order to compare the relative free energies of the gauche and anti conformations of the 9-membered hydrogen bonded ring. The gauche conformation was found to be 2.1 kJ mol$^{-1}$ lower in energy than the anti conformation (Figure S202b).

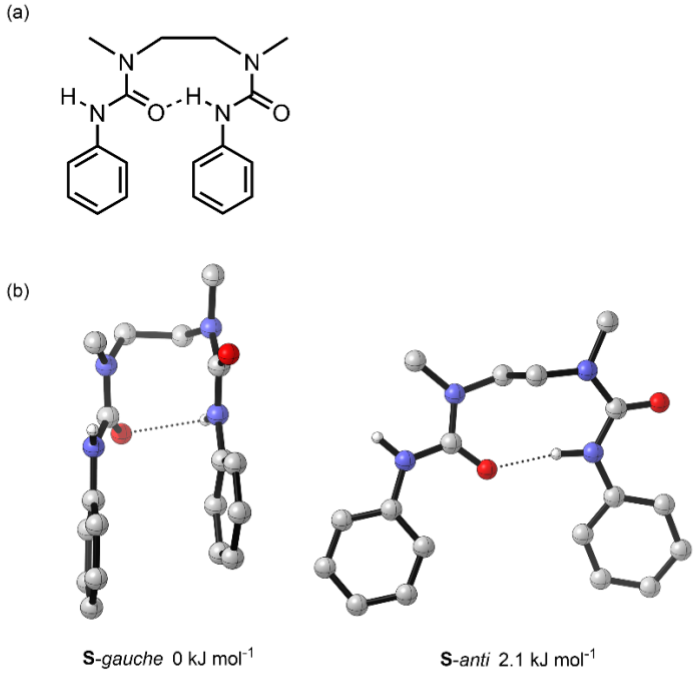


**Figure S202** – Representation and simulations of diurea **S**. (a) Two-urea system **S**; (b) Computed anti and gauche conformations of **S** (B3LYP-D3(BJ)/6-311G(d,p)/IEF-PCM(DCM)//B3LYP-D3(BJ)/6-31G(d,p)).

**Conformations of 4'**

Modelling of **4'** (a truncated version of **4**, with Ar = Ph and benzyl substituents truncated to methyl, Figure S203) revealed a slightly greater prevalence of the gauche backbone conformation, as observed for **7c**.

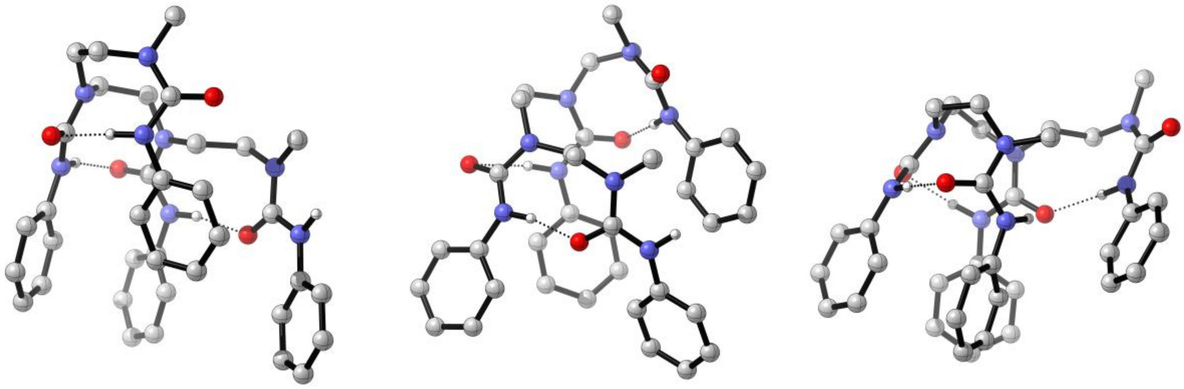

**4'**-*gag* 0 kJ mol$^{-1}$ **4'**-*agg* 1.6 kJ mol$^{-1}$ **4'**-*gga* 5.7 kJ mol$^{-1}$

**Figure S203** – Three lowest energy conformers of **4'** with corresponding backbone conformations (*g* = gauche, *a* = anti) starting from N terminus (B3LYP-D3(BJ)/6-311G(d,p)/IEF-PCM(DCM)//B3LYP-D3(BJ)/6-31G(d,p)).

**Energies and molecular geometries of included computed structures**

All energies in Hartrees, coordinates in Å. Cartesian coordinates generated by ESIgen software.[32] All quasiharmonic energies were temperature-corrected at 298.15 K.

The energies and molecular geometries for all other computed structures are available upon request.

<u>S-anti</u>

B3LYP-D3(BJ)/6-31G(d,p) Energy = -1068.796201
B3LYP-D3(BJ)/6-311G(d,p)/IEF-PCM(DCM) Energy = -1069.057396
B3LYP-D3(BJ)/6-311G(d,p)/IEF-PCM(DCM)//B3LYP-D3(BJ)/6-31G(d,p) Quasiharmonic Free Energy = -1068.728710

Frequencies (Top 3 out of 132)

1. 14.4753 cm$^{-1}$
2. 19.0664 cm$^{-1}$
3. 28.3748 cm$^{-1}$

B3LYP-D3(BJ)/6-31G(d,p) Molecular Geometry in Cartesian Coordinates

| | | | |
|---|---|---|---|
| C | 3.58070 | -3.28547 | -0.47419 |
| H | 3.34865 | -4.00632 | 0.32071 |
| H | 3.53984 | -3.80885 | -1.43783 |
| N | 2.65827 | -2.16343 | -0.45736 |
| C | 3.17398 | -0.88736 | -0.23495 |
| O | 4.38585 | -0.69493 | -0.16247 |

| | | | |
|---|---|---|---|
| N | 2.22601 | 0.10938 | -0.10866 |
| H | 1.24448 | -0.13691 | -0.02261 |
| C | 1.24951 | -2.44157 | -0.64320 |
| H | 1.15532 | -3.35677 | -1.23736 |
| H | 0.78206 | -1.64327 | -1.22466 |
| C | 0.48613 | -2.59768 | 0.68993 |
| H | 0.80600 | -1.81155 | 1.37654 |
| H | 0.71513 | -3.55671 | 1.16054 |
| N | -0.96235 | -2.50461 | 0.51636 |
| C | -1.77169 | -3.70708 | 0.39525 |
| H | -1.11239 | -4.57499 | 0.37398 |
| H | -2.45688 | -3.83705 | 1.24508 |
| H | -2.35511 | -3.70905 | -0.53274 |
| C | -1.46181 | -1.23895 | 0.31568 |
| O | -0.71347 | -0.25591 | 0.24856 |
| N | -2.83091 | -1.15187 | 0.19344 |
| H | -3.37120 | -1.96511 | 0.44333 |
| C | 2.47399 | 1.48659 | 0.06310 |
| C | 3.72998 | 2.08565 | -0.11583 |
| C | 1.37151 | 2.28793 | 0.40787 |
| C | 3.86229 | 3.46375 | 0.05021 |
| H | 4.57949 | 1.46938 | -0.36818 |
| C | 1.52397 | 3.66151 | 0.56660 |
| H | 0.40240 | 1.82299 | 0.55583 |
| C | 2.77087 | 4.26263 | 0.38904 |
| H | 4.84059 | 3.91487 | -0.08993 |
| H | 0.66000 | 4.26309 | 0.83453 |
| C | -3.59658 | 0.01224 | -0.03908 |
| C | -4.97254 | -0.07104 | 0.21596 |
| C | -3.05662 | 1.20536 | -0.53853 |
| C | -5.80112 | 1.02141 | -0.02082 |
| H | -5.39261 | -0.99566 | 0.60546 |
| C | -3.89942 | 2.29220 | -0.76511 |
| H | -1.99758 | 1.27363 | -0.73585 |
| C | -5.26851 | 2.21355 | -0.51140 |
| H | -6.86409 | 0.93824 | 0.18325 |
| H | -3.47141 | 3.21293 | -1.14957 |
| H | -5.91160 | 3.06836 | -0.69283 |
| H | 2.88948 | 5.33426 | 0.51503 |
| H | 4.58561 | -2.89711 | -0.32113 |

<u>S-gauche</u>

B3LYP-D3(BJ)/6-31G(d,p) Energy = -1068.804266
B3LYP-D3(BJ)/6-311G(d,p)/IEF-PCM(DCM) Energy = -1069.060786
B3LYP-D3(BJ)/6-311G(d,p)/IEF-PCM(DCM)//B3LYP-D3(BJ)/6-31G(d,p) Quasiharmonic Free Energy = -1068.729494

Frequencies (Top 3 out of 132)

1. 27.1193 $cm^{-1}$
2. 38.6956 $cm^{-1}$
3. 43.9169 $cm^{-1}$

B3LYP-D3(BJ)/6-31G(d,p) Molecular Geometry in Cartesian Coordinates

| | | | |
|---|---|---|---|
| C | -4.57791 | 0.83436 | 0.23840 |
| H | -5.43496 | 0.62352 | -0.40730 |
| H | -4.64988 | 0.19988 | 1.13283 |
| N | -3.35429 | 0.61070 | -0.52023 |
| C | -2.23176 | 1.30606 | -0.07587 |
| O | -2.30204 | 2.07182 | 0.88643 |
| N | -1.07831 | 1.10932 | -0.80558 |
| H | -1.07780 | 0.38356 | -1.50574 |
| C | -3.28184 | -0.63813 | -1.26369 |
| H | -2.51311 | -0.58863 | -2.03473 |
| H | -4.23102 | -0.77632 | -1.79307 |
| C | -3.02336 | -1.87433 | -0.37783 |
| H | -2.83182 | -2.73143 | -1.02829 |
| H | -3.90751 | -2.09547 | 0.22465 |
| N | -1.90213 | -1.71357 | 0.54340 |
| C | -2.15179 | -1.32957 | 1.92781 |
| H | -3.18877 | -1.56008 | 2.17362 |
| H | -1.52327 | -1.91179 | 2.61024 |
| H | -1.99750 | -0.25683 | 2.10505 |
| C | -0.64249 | -1.71348 | -0.01784 |
| O | -0.47989 | -1.93438 | -1.22238 |
| N | 0.39062 | -1.45166 | 0.85933 |
| H | 0.14616 | -1.03546 | 1.74370 |
| C | 0.20156 | 1.64595 | -0.55793 |
| C | 0.48420 | 2.57856 | 0.44984 |
| C | 1.23694 | 1.19799 | -1.39391 |
| C | 1.79096 | 3.04526 | 0.59945 |
| H | -0.31314 | 2.92543 | 1.08947 |
| C | 2.53076 | 1.67664 | -1.23270 |
| H | 1.02553 | 0.44616 | -2.14810 |
| C | 2.81851 | 2.60695 | -0.23360 |
| H | 1.99967 | 3.76872 | 1.38251 |
| H | 3.31960 | 1.29839 | -1.87398 |
| C | 1.76043 | -1.35634 | 0.53774 |
| C | 2.57014 | -0.60380 | 1.39694 |
| C | 2.33206 | -2.00251 | -0.56591 |
| C | 3.93372 | -0.48613 | 1.15331 |
| H | 2.12100 | -0.07913 | 2.23581 |
| C | 3.69910 | -1.86958 | -0.80100 |
| H | 1.70343 | -2.57495 | -1.23189 |
| C | 4.50859 | -1.11653 | 0.04976 |
| H | 4.54347 | 0.11400 | 1.82123 |
| H | 4.13414 | -2.36795 | -1.66230 |
| H | 5.57187 | -1.02057 | -0.14509 |
| H | 3.83049 | 2.97605 | -0.10209 |
| H | -4.60930 | 1.87166 | 0.56483 |

**4’** Conformation 1

B3LYP-D3(BJ)/6-31G(d,p) Energy = -2136.447874
B3LYP-D3(BJ)/6-311G(d,p)/IEF-PCM(DCM) Energy = -2136.947469

B3LYP-D3(BJ)/6-311G(d,p)/IEF-PCM(DCM)//B3LYP-D3(BJ)/6-31G(d,p) Quasiharmonic Free Energy = -2136.275887

Frequencies (Top 3 out of 264)

1. 12.0903 $cm^{-1}$
2. 16.4682 $cm^{-1}$
3. 25.5427 $cm^{-1}$

B3LYP-D3(BJ)/6-31G(d,p) Molecular Geometry in Cartesian Coordinates

| | | | |
|---|---|---|---|
| C | 2.970479 | -0.319753 | -2.452636 |
| H | 3.034539 | -1.406484 | -2.551550 |
| H | 3.146801 | 0.119826 | -3.440884 |
| C | 4.100294 | 0.169544 | -1.529381 |
| H | 5.046061 | -0.189462 | -1.947254 |
| H | 4.149588 | 1.257036 | -1.571766 |
| N | 1.611738 | -0.018525 | -2.011201 |
| N | 4.007042 | -0.294713 | -0.144687 |
| C | 1.201330 | 1.298923 | -2.045095 |
| O | 2.033997 | 2.203176 | -2.252113 |
| C | 3.456770 | 0.471813 | 0.870578 |
| O | 3.468268 | 0.039646 | 2.037122 |
| N | -0.123017 | 1.527191 | -1.803277 |
| N | 2.905343 | 1.674748 | 0.529944 |
| H | -0.770452 | 0.745365 | -1.684997 |
| H | 2.842335 | 1.928466 | -0.452657 |
| C | 0.742911 | -1.103004 | -1.573830 |
| H | 0.165033 | -0.776415 | -0.707615 |
| H | 1.368500 | -1.922240 | -1.234220 |
| C | 4.921349 | -1.365121 | 0.280343 |
| H | 5.381398 | -1.753889 | -0.630801 |
| H | 5.724137 | -0.946127 | 0.901375 |
| C | -0.223276 | -1.617299 | -2.661504 |
| H | 0.286770 | -2.306047 | -3.339776 |
| H | -0.592318 | -0.780272 | -3.256846 |
| N | -1.370206 | -2.314188 | -2.086084 |
| C | -1.300608 | -3.748290 | -1.824472 |
| H | -1.223464 | -3.962111 | -0.754915 |
| H | -2.158034 | -4.270769 | -2.267475 |
| H | -0.394712 | -4.142327 | -2.281369 |
| C | -2.348569 | -1.512713 | -1.556607 |
| O | -2.280211 | -0.276091 | -1.625367 |
| N | -3.423631 | -2.163474 | -0.993826 |
| H | -3.341801 | -3.160963 | -0.879049 |
| C | 2.166040 | 2.540890 | 1.373474 |
| C | 1.511869 | 3.604634 | 0.735205 |
| C | 2.044210 | 2.388269 | 2.762417 |
| C | 0.709090 | 4.476423 | 1.461026 |
| H | 1.614321 | 3.724700 | -0.335958 |
| C | 1.237986 | 3.275758 | 3.476791 |
| H | 2.560110 | 1.582536 | 3.260730 |
| C | 0.560649 | 4.314223 | 2.839383 |
| H | 0.187158 | 5.269027 | 0.935625 |
| H | 1.141773 | 3.143021 | 4.550406 |

| | | | |
|---|---|---|---|
| C | -0.728593 | 2.785977 | -1.595731 |
| C | -0.270515 | 3.968988 | -2.188761 |
| C | -1.858613 | 2.814617 | -0.765189 |
| C | -0.929757 | 5.168985 | -1.922784 |
| H | 0.600271 | 3.939896 | -2.828216 |
| C | -2.513709 | 4.017212 | -0.521110 |
| H | -2.204255 | 1.891377 | -0.313361 |
| C | -2.050464 | 5.204142 | -1.092156 |
| H | -0.564328 | 6.083931 | -2.379856 |
| H | -3.384029 | 4.026856 | 0.128345 |
| C | -4.483133 | -1.578136 | -0.274186 |
| C | -5.243530 | -2.428558 | 0.541830 |
| C | -4.809839 | -0.216228 | -0.341124 |
| C | -6.294012 | -1.926465 | 1.303055 |
| H | -4.998121 | -3.486896 | 0.589009 |
| C | -5.862703 | 0.272393 | 0.430955 |
| H | -4.234365 | 0.439184 | -0.976217 |
| C | -6.606999 | -0.567271 | 1.259446 |
| H | -6.864325 | -2.600665 | 1.934512 |
| H | -6.101306 | 1.330442 | 0.377240 |
| H | -0.071006 | 4.989279 | 3.408154 |
| H | -2.558166 | 6.143159 | -0.895291 |
| H | -7.421732 | -0.171671 | 1.856894 |
| C | 4.319470 | -2.543244 | 1.064831 |
| H | 5.141808 | -3.248725 | 1.245990 |
| H | 3.983846 | -2.201795 | 2.039533 |
| N | 3.258315 | -3.261661 | 0.383056 |
| C | 3.668535 | -4.248252 | -0.601132 |
| H | 4.322524 | -5.003763 | -0.147113 |
| H | 2.777717 | -4.734390 | -0.992432 |
| H | 4.215803 | -3.775373 | -1.426400 |
| C | 1.911884 | -3.161219 | 0.686360 |
| N | 1.565935 | -2.169521 | 1.580836 |
| H | 2.254960 | -1.455536 | 1.810648 |
| O | 1.079598 | -3.900933 | 0.149245 |
| C | 0.228125 | -1.762774 | 1.796698 |
| C | -0.026958 | -0.389905 | 1.914952 |
| C | -0.831404 | -2.672549 | 1.906401 |
| C | -1.325031 | 0.071925 | 2.120501 |
| H | 0.790027 | 0.316075 | 1.846793 |
| C | -2.125822 | -2.196584 | 2.108651 |
| H | -0.631949 | -3.732272 | 1.828705 |
| C | -2.386248 | -0.828857 | 2.210439 |
| H | -1.494339 | 1.141354 | 2.200689 |
| H | -2.945670 | -2.902973 | 2.191638 |
| H | -3.402182 | -0.476708 | 2.349471 |

<u>**4’** Conformation 2</u>

B3LYP-D3(BJ)/6-31G(d,p) Energy = -2136.448287
B3LYP-D3(BJ)/6-311G(d,p)/IEF-PCM(DCM) Energy = -2136.947461
B3LYP-D3(BJ)/6-311G(d,p)/IEF-PCM(DCM)//B3LYP-D3(BJ)/6-31G(d,p) Quasiharmonic Free Energy = -2136.275264

Frequencies (Top 3 out of 264)

1. 16.7922 $cm^{-1}$
2. 21.4112 $cm^{-1}$
3. 24.8879 $cm^{-1}$

B3LYP-D3(BJ)/6-31G(d,p) Molecular Geometry in Cartesian Coordinates

| | | | |
|---|---|---|---|
| C | 2.232213 | -2.825778 | -1.311286 |
| H | 2.165400 | -3.679448 | -1.992279 |
| H | 2.980672 | -2.132363 | -1.702252 |
| C | 2.703366 | -3.370567 | 0.047873 |
| H | 1.970772 | -4.093927 | 0.412077 |
| H | 3.620824 | -3.939176 | -0.123768 |
| N | 0.951970 | -2.125953 | -1.312459 |
| N | 3.005968 | -2.350626 | 1.055597 |
| C | -0.185237 | -2.906449 | -1.163355 |
| O | -0.073675 | -4.104089 | -0.857006 |
| C | 2.046433 | -1.720128 | 1.819034 |
| O | 2.349066 | -0.734907 | 2.515593 |
| N | -1.382092 | -2.271695 | -1.364609 |
| N | 0.778739 | -2.236888 | 1.770944 |
| H | -1.386630 | -1.264509 | -1.536660 |
| H | 0.624590 | -3.056867 | 1.199470 |
| C | 0.928082 | -0.671408 | -1.227326 |
| H | 1.887003 | -0.341386 | -0.837819 |
| H | 0.172987 | -0.364644 | -0.501689 |
| C | 4.404880 | -2.105276 | 1.419230 |
| H | 4.547938 | -2.298673 | 2.488931 |
| H | 4.997186 | -2.843274 | 0.874803 |
| C | 0.650340 | 0.030421 | -2.573568 |
| H | 1.559289 | 0.068114 | -3.175853 |
| H | -0.101560 | -0.529746 | -3.136015 |
| N | 0.193333 | 1.402094 | -2.359007 |
| C | 1.064076 | 2.520685 | -2.702735 |
| H | 0.997180 | 3.311561 | -1.954174 |
| H | 2.100379 | 2.185221 | -2.673137 |
| H | 0.829275 | 2.929257 | -3.694468 |
| C | -1.058402 | 1.532244 | -1.826889 |
| O | -1.760283 | 0.535092 | -1.585569 |
| N | -1.502160 | 2.807992 | -1.570181 |
| H | -0.883756 | 3.567525 | -1.806339 |
| C | -0.417137 | -1.619720 | 2.205528 |
| C | -0.469946 | -0.422704 | 2.934642 |
| C | -1.609384 | -2.264385 | 1.848645 |
| C | -1.712521 | 0.135636 | 3.238618 |
| H | 0.446191 | 0.046997 | 3.257634 |
| C | -2.839873 | -1.699623 | 2.164162 |
| H | -1.571802 | -3.198654 | 1.299597 |
| C | -2.899415 | -0.487246 | 2.853775 |
| H | -1.744006 | 1.070583 | 3.790022 |
| H | -3.746113 | -2.205690 | 1.849832 |
| C | -2.669837 | -2.808400 | -1.157109 |
| C | -2.925516 | -4.166789 | -0.915859 |
| C | -3.742327 | -1.899869 | -1.189670 |

| | | | |
|---|---|---|---|
| C | -4.235534 | -4.589207 | -0.686812 |
| H | -2.103959 | -4.866630 | -0.901083 |
| C | -5.041823 | -2.342752 | -0.968719 |
| H | -3.543649 | -0.850701 | -1.375189 |
| C | -5.300458 | -3.689941 | -0.707343 |
| H | -4.418787 | -5.642338 | -0.493932 |
| H | -5.856300 | -1.624739 | -0.994680 |
| C | -2.545219 | 3.161637 | -0.686873 |
| C | -3.135425 | 2.265779 | 0.216799 |
| C | -2.937446 | 4.507365 | -0.679233 |
| C | -4.103535 | 2.730858 | 1.104842 |
| H | -2.830979 | 1.230089 | 0.231521 |
| C | -3.901020 | 4.957395 | 0.218755 |
| H | -2.478508 | 5.200465 | -1.379407 |
| C | -4.494082 | 4.070060 | 1.117242 |
| H | -4.546937 | 2.027635 | 1.802934 |
| H | -4.188489 | 6.004346 | 0.211934 |
| H | -3.858055 | -0.042407 | 3.100569 |
| H | -6.314835 | -4.032398 | -0.528777 |
| H | -5.247474 | 4.417921 | 1.816445 |
| C | 4.962034 | -0.697708 | 1.138055 |
| H | 4.587784 | -0.003909 | 1.884694 |
| H | 6.052849 | -0.750564 | 1.268724 |
| N | 4.676921 | -0.185567 | -0.190731 |
| C | 5.499174 | -0.695233 | -1.274840 |
| H | 5.170373 | -0.238125 | -2.205789 |
| H | 6.558886 | -0.453545 | -1.117269 |
| H | 5.403748 | -1.784398 | -1.347156 |
| C | 3.911870 | 0.942928 | -0.431095 |
| N | 3.107462 | 1.340566 | 0.621024 |
| H | 2.856861 | 0.650853 | 1.331518 |
| O | 3.932371 | 1.519271 | -1.522008 |
| C | 2.283376 | 2.484292 | 0.578770 |
| C | 0.993438 | 2.395687 | 1.117506 |
| C | 2.736051 | 3.706678 | 0.064972 |
| C | 0.166639 | 3.515702 | 1.149263 |
| H | 0.639810 | 1.447392 | 1.504784 |
| C | 1.894197 | 4.817552 | 0.085163 |
| H | 3.728995 | 3.770630 | -0.360643 |
| C | 0.607424 | 4.731643 | 0.624461 |
| H | -0.832592 | 3.427237 | 1.561139 |
| H | 2.252277 | 5.760257 | -0.318374 |
| H | -0.044387 | 5.599359 | 0.640268 |

**4’** Conformation 3

B3LYP-D3(BJ)/6-31G(d,p) Energy = -2136.447515
B3LYP-D3(BJ)/6-311G(d,p)/IEF-PCM(DCM) Energy = -2136.946462
B3LYP-D3(BJ)/6-311G(d,p)/IEF-PCM(DCM)//B3LYP-D3(BJ)/6-31G(d,p) Quasiharmonic Free Energy = -2136.273715

Frequencies (Top 3 out of 264)

1. 16.1269 $cm^{-1}$

2. 22.8771 $cm^{-1}$
3. 33.5560 $cm^{-1}$

B3LYP-D3(BJ)/6-31G(d,p) Molecular Geometry in Cartesian Coordinates

| | | | |
|---|---|---|---|
| C | 0.074350 | -4.054180 | -0.448270 |
| H | -0.522280 | -4.974650 | -0.412300 |
| H | 0.993810 | -4.278060 | -0.994530 |
| C | 0.437330 | -3.679760 | 1.003690 |
| H | -0.448890 | -3.719550 | 1.629870 |
| H | 1.126050 | -4.446420 | 1.378290 |
| N | -0.615710 | -3.019280 | -1.219950 |
| N | 1.078940 | -2.383010 | 1.140110 |
| C | -1.874040 | -2.661040 | -0.762770 |
| O | -2.312390 | -3.159750 | 0.288860 |
| C | 0.447860 | -1.221040 | 1.509680 |
| O | 1.078140 | -0.140780 | 1.499300 |
| N | -2.558360 | -1.721140 | -1.486780 |
| N | -0.855500 | -1.301330 | 1.922680 |
| H | -2.018230 | -1.136020 | -2.125610 |
| H | -1.434230 | -2.055480 | 1.553490 |
| C | -0.191570 | -2.860590 | -2.613860 |
| H | -0.995070 | -2.400940 | -3.178940 |
| H | -0.034920 | -3.857570 | -3.048110 |
| C | 2.444400 | -2.239320 | 0.648700 |
| H | 2.715770 | -3.157571 | 0.122740 |
| H | 2.471780 | -1.427090 | -0.073420 |
| C | 1.112630 | -2.075820 | -2.842510 |
| H | 1.215040 | -1.944250 | -3.931220 |
| H | 1.964750 | -2.684110 | -2.518100 |
| N | 1.248910 | -0.804940 | -2.135340 |
| C | 2.578130 | -0.196371 | -2.297350 |
| H | 2.620811 | 0.766609 | -1.793690 |
| H | 3.347390 | -0.843111 | -1.868470 |
| H | 2.811740 | -0.044251 | -3.360830 |
| C | 0.149440 | 0.055350 | -2.167270 |
| O | -0.787100 | -0.095260 | -2.965770 |
| N | 0.180961 | 1.027060 | -1.209510 |
| H | 0.747931 | 0.835230 | -0.388130 |
| C | -1.575560 | -0.218320 | 2.481250 |
| C | -0.972169 | 0.728200 | 3.320420 |
| C | -2.946350 | -0.136140 | 2.214270 |
| C | -1.737669 | 1.766490 | 3.847870 |
| H | 0.085061 | 0.660200 | 3.536660 |
| C | -3.701129 | 0.902230 | 2.752480 |
| H | -3.414410 | -0.874680 | 1.575690 |
| C | -3.101669 | 1.864390 | 3.565370 |
| H | -1.260719 | 2.500930 | 4.490000 |
| H | -4.757959 | 0.958270 | 2.514530 |
| C | -3.763550 | -1.121110 | -1.046180 |
| C | -4.779490 | -1.845940 | -0.411360 |
| C | -3.922750 | 0.251030 | -1.275580 |
| C | -5.925410 | -1.180600 | 0.023390 |
| H | -4.654280 | -2.905260 | -0.235910 |
| C | -5.074279 | 0.900910 | -0.842710 |

| | | | |
|---|---|---|---|
| H | -3.134069 | 0.806970 | -1.767810 |
| C | -6.079160 | 0.191600 | -0.182990 |
| H | -6.705170 | -1.744920 | 0.526050 |
| H | -5.167909 | 1.969250 | -1.008360 |
| C | -0.779939 | 2.054860 | -1.081080 |
| C | -1.157659 | 2.451190 | 0.205360 |
| C | -1.339769 | 2.683150 | -2.199880 |
| C | -2.106919 | 3.455810 | 0.373120 |
| H | -0.730819 | 1.952920 | 1.065960 |
| C | -2.296499 | 3.680070 | -2.019220 |
| H | -1.045039 | 2.375320 | -3.194320 |
| C | -2.687499 | 4.070530 | -0.736740 |
| H | -2.402639 | 3.734680 | 1.378510 |
| H | -2.734339 | 4.157620 | -2.890530 |
| H | -3.689409 | 2.677740 | 3.979300 |
| H | -6.974219 | 0.699730 | 0.162120 |
| H | -3.433919 | 4.847610 | -0.605580 |
| C | 3.465580 | -1.956161 | 1.769490 |
| H | 3.039240 | -1.240481 | 2.475580 |
| H | 3.668480 | -2.866701 | 2.339770 |
| N | 4.728430 | -1.465321 | 1.241830 |
| C | 5.752600 | -2.453431 | 0.938410 |
| H | 5.443720 | -3.141411 | 0.138020 |
| H | 6.648580 | -1.929411 | 0.612880 |
| H | 5.974380 | -3.041991 | 1.834350 |
| C | 4.863960 | -0.206691 | 0.658310 |
| N | 3.808971 | 0.655829 | 0.884110 |
| H | 2.967800 | 0.299059 | 1.327880 |
| O | 5.865720 | 0.092629 | 0.009870 |
| C | 3.629461 | 1.936899 | 0.325600 |
| C | 2.452591 | 2.617960 | 0.686500 |
| C | 4.516961 | 2.538099 | -0.581020 |
| C | 2.151191 | 3.856120 | 0.128840 |
| H | 1.771581 | 2.160800 | 1.395940 |
| C | 4.202221 | 3.785029 | -1.121920 |
| H | 5.427621 | 2.023719 | -0.847880 |
| C | 3.023931 | 4.449719 | -0.783610 |
| H | 1.223061 | 4.346700 | 0.404000 |
| H | 4.895561 | 4.237499 | -1.825190 |
| H | 2.789551 | 5.414549 | -1.221570 |

**6c** Conformation 1

B3LYP-D3(BJ)/6-31G(d,p) Energy = -3243.412998
B3LYP-D3(BJ)/6-311G(d,p)/IEF-PCM(DCM) Energy = -3244.160209
B3LYP-D3(BJ)/6-311G(d,p)/IEF-PCM(DCM)//B3LYP-D3(BJ)/6-31G(d,p) Quasiharmonic Free Energy = -3243.117092

Frequencies (Top 3 out of 405)

1. 21.0484 $cm^{-1}$
2. 24.1099 $cm^{-1}$

3. 27.5837 $cm^{-1}$

B3LYP-D3(BJ)/6-31G(d,p) Molecular Geometry in Cartesian Coordinates

| | | | |
|---|---|---|---|
| C | -0.279648 | -2.355445 | -3.451955 |
| H | -0.740615 | -2.976082 | -4.226443 |
| H | 0.311125 | -1.583836 | -3.954094 |
| C | 0.649272 | -3.255118 | -2.616084 |
| H | 0.110688 | -4.154345 | -2.322762 |
| H | 1.480565 | -3.579274 | -3.254206 |
| N | -1.315836 | -1.663909 | -2.694587 |
| N | 1.198147 | -2.602278 | -1.435774 |
| C | -2.386345 | -2.407254 | -2.238084 |
| O | -2.316207 | -3.651504 | -2.234387 |
| C | 0.752530 | -2.807076 | -0.145832 |
| O | 1.397860 | -2.344677 | 0.812009 |
| N | -3.465857 | -1.700699 | -1.790991 |
| N | -0.393835 | -3.534471 | 0.011903 |
| H | -3.484187 | -0.683852 | -1.904236 |
| H | -0.992339 | -3.696350 | -0.796931 |
| C | -1.114210 | -0.263510 | -2.348175 |
| H | -0.042623 | -0.100049 | -2.235577 |
| H | -1.557994 | -0.072228 | -1.370064 |
| C | 2.439164 | -1.848250 | -1.565936 |
| H | 2.510077 | -1.463632 | -2.584372 |
| H | 2.404541 | -0.984522 | -0.903819 |
| C | -1.702273 | 0.723900 | -3.380868 |
| H | -2.627662 | 0.311646 | -3.786931 |
| H | -1.008196 | 0.871368 | -4.212817 |
| N | -1.984362 | 2.030394 | -2.798292 |
| C | -0.966541 | 3.066553 | -2.891380 |
| H | -0.650750 | 3.138309 | -3.938895 |
| H | -1.407424 | 4.027983 | -2.639504 |
| C | -3.084417 | 2.086252 | -1.977512 |
| O | -3.851619 | 1.108813 | -1.892900 |
| N | -3.297204 | 3.252417 | -1.285352 |
| H | -2.496955 | 3.864389 | -1.140821 |
| C | -0.988165 | -3.870358 | 1.249658 |
| C | -0.271193 | -3.986685 | 2.447933 |
| C | -2.364668 | -4.129957 | 1.236640 |
| C | -0.945925 | -4.339853 | 3.615853 |
| H | 0.788238 | -3.779079 | 2.464407 |
| C | -3.025436 | -4.478501 | 2.410073 |
| H | -2.915513 | -4.046364 | 0.308230 |
| C | -2.320784 | -4.582473 | 3.611105 |
| H | -0.384197 | -4.422131 | 4.541665 |
| H | -4.096076 | -4.655481 | 2.376973 |
| C | -4.583865 | -2.217394 | -1.099023 |
| C | -4.953188 | -3.570160 | -1.111170 |
| C | -5.356155 | -1.295734 | -0.372257 |
| C | -6.055029 | -3.987533 | -0.363517 |
| H | -4.373948 | -4.273936 | -1.690231 |
| C | -6.457803 | -1.728898 | 0.358716 |
| H | -5.083731 | -0.246405 | -0.387947 |
| C | -6.809824 | -3.079530 | 0.378413 |

| | | | |
|---|---|---|---|
| H | -6.326762 | -5.039030 | -0.373487 |
| H | -7.037303 | -1.002864 | 0.920953 |
| C | -4.345292 | 3.485935 | -0.367630 |
| C | -4.133650 | 4.504286 | 0.574973 |
| C | -5.562695 | 2.790897 | -0.373799 |
| C | -5.116101 | 4.806482 | 1.512410 |
| H | -3.190498 | 5.040944 | 0.565328 |
| C | -6.534083 | 3.103970 | 0.576934 |
| H | -5.737285 | 2.020407 | -1.109181 |
| C | -6.321931 | 4.103350 | 1.525523 |
| H | -4.934916 | 5.595489 | 2.236232 |
| H | -7.472226 | 2.556827 | 0.565935 |
| H | -2.834415 | -4.848192 | 4.529477 |
| H | -7.664874 | -3.416975 | 0.955377 |
| H | -7.085913 | 4.335475 | 2.260412 |
| C | 3.667175 | -2.713575 | -1.210203 |
| H | 3.416157 | -3.336710 | -0.347227 |
| H | 3.897212 | -3.397411 | -2.030708 |
| N | 4.861225 | -1.930480 | -0.922973 |
| C | 5.960662 | -1.880583 | -1.884387 |
| H | 5.734411 | -2.618386 | -2.657517 |
| H | 6.893637 | -2.180760 | -1.396874 |
| C | 5.063519 | -1.296993 | 0.288644 |
| N | 4.077990 | -1.435994 | 1.230437 |
| H | 3.174528 | -1.837347 | 0.970435 |
| O | 6.095882 | -0.625397 | 0.462295 |
| C | 4.083065 | -0.845353 | 2.514840 |
| C | 5.258842 | -0.495846 | 3.193928 |
| C | 2.837742 | -0.629062 | 3.124575 |
| C | 5.175656 | 0.094475 | 4.454380 |
| H | 6.216676 | -0.662475 | 2.723705 |
| C | 2.776072 | -0.053558 | 4.390453 |
| H | 1.930392 | -0.908541 | 2.601237 |
| C | 3.940835 | 0.322188 | 5.061480 |
| H | 6.092290 | 0.371895 | 4.966297 |
| H | 1.808255 | 0.107262 | 4.855109 |
| H | 3.886807 | 0.778089 | 6.044939 |
| C | 6.192756 | -0.502914 | -2.539053 |
| N | 4.982134 | 0.117174 | -3.044084 |
| C | 4.463574 | -0.396453 | -4.299357 |
| H | 4.134723 | -1.441706 | -4.212342 |
| H | 3.615101 | 0.215230 | -4.597880 |
| H | 5.240618 | -0.350437 | -5.070527 |
| C | 4.221270 | 1.044029 | -2.352559 |
| O | 3.144112 | 1.447845 | -2.807949 |
| N | 4.753033 | 1.494602 | -1.160074 |
| H | 5.432088 | 0.906402 | -0.686415 |
| C | 4.105822 | 2.407564 | -0.293858 |
| C | 4.276644 | 2.223806 | 1.083975 |
| C | 3.331357 | 3.477115 | -0.757919 |
| C | 3.621276 | 3.053746 | 1.989390 |
| H | 4.905756 | 1.418585 | 1.440635 |
| C | 2.674326 | 4.299151 | 0.156970 |
| H | 3.218698 | 3.629883 | -1.821572 |
| C | 2.799050 | 4.084930 | 1.530724 |

| | | | |
|---|---|---|---|
| H | 3.745454 | 2.871601 | 3.052233 |
| H | 2.037039 | 5.098239 | -0.208571 |
| H | 2.268730 | 4.720529 | 2.232558 |
| C | 0.293983 | 2.817108 | -2.046636 |
| N | 0.055237 | 2.375199 | -0.668526 |
| C | 0.916894 | 1.304061 | -0.143872 |
| H | 0.356309 | 0.397016 | 0.091272 |
| H | 1.431362 | 1.636653 | 0.757207 |
| H | 1.670421 | 1.086834 | -0.897235 |
| C | -0.514370 | 3.301572 | 0.168723 |
| O | -0.835715 | 4.431650 | -0.252395 |
| N | -0.704123 | 2.941810 | 1.490060 |
| C | -1.079937 | 1.616644 | 1.888527 |
| C | -2.161325 | 0.970704 | 1.281929 |
| C | -0.382903 | 0.987740 | 2.923107 |
| C | -2.525684 | -0.310806 | 1.696203 |
| H | -2.720613 | 1.469770 | 0.502493 |
| C | -0.767712 | -0.282426 | 3.346997 |
| H | 0.475023 | 1.486498 | 3.361436 |
| C | -1.838119 | -0.935780 | 2.735337 |
| H | -3.361428 | -0.813870 | 1.222952 |
| H | -0.220562 | -0.778412 | 4.142420 |
| H | -2.132468 | -1.923912 | 3.063684 |
| H | 0.915641 | 2.058950 | -2.518707 |
| H | 0.872043 | 3.746339 | -2.053907 |
| H | 6.689988 | 0.158459 | -1.833458 |
| H | 6.886083 | -0.639899 | -3.378300 |
| C | -1.053078 | 4.008121 | 2.430342 |
| H | -0.625160 | 3.770340 | 3.406652 |
| H | -0.640563 | 4.947565 | 2.066385 |
| H | -2.138650 | 4.110321 | 2.535193 |

**6c** Conformation 2

B3LYP-D3(BJ)/6-31G(d,p) Energy = -3243.411078
B3LYP-D3(BJ)/6-311G(d,p)/IEF-PCM(DCM) Energy = -3244.156867
B3LYP-D3(BJ)/6-311G(d,p)/IEF-PCM(DCM)//B3LYP-D3(BJ)/6-31G(d,p) Quasiharmonic Free Energy = -3243.115423

Frequencies (Top 3 out of 405)

1. 14.9547 $cm^{-1}$
2. 21.4245 $cm^{-1}$
3. 22.8086 $cm^{-1}$

B3LYP-D3(BJ)/6-31G(d,p) Molecular Geometry in Cartesian Coordinates

| | | | |
|---|---|---|---|
| C | 0.293292 | -4.270385 | -1.384771 |
| H | -0.614429 | -3.958620 | -1.908593 |
| H | 0.594235 | -5.246412 | -1.792484 |
| C | -0.063526 | -4.505460 | 0.093804 |
| H | -0.791751 | -5.326059 | 0.101728 |
| H | 0.806779 | -4.859611 | 0.637150 |
| N | 1.310960 | -3.260206 | -1.703646 |

| | | | |
|---|---|---|---|
| N | -0.672483 | -3.378301 | 0.786957 |
| C | 2.563952 | -3.408476 | -1.138853 |
| O | 2.744037 | -4.237257 | -0.226637 |
| C | -0.037426 | -2.507756 | 1.649313 |
| O | -0.708520 | -1.660518 | 2.268913 |
| N | 3.566913 | -2.595759 | -1.599538 |
| N | 1.318145 | -2.611769 | 1.748354 |
| H | 3.317857 | -1.788550 | -2.169097 |
| H | 1.795867 | -3.277641 | 1.142632 |
| C | 1.083057 | -2.483706 | -2.925565 |
| H | 0.482976 | -3.086771 | -3.615918 |
| H | 2.032197 | -2.302350 | -3.425476 |
| C | -2.078048 | -3.113587 | 0.530450 |
| H | -2.354828 | -3.647641 | -0.374287 |
| H | -2.222535 | -2.052907 | 0.318415 |
| C | 0.353005 | -1.146481 | -2.700460 |
| H | -0.707675 | -1.330092 | -2.514909 |
| H | 0.432161 | -0.566690 | -3.626349 |
| N | 0.863387 | -0.374957 | -1.570719 |
| C | -0.037161 | -0.143909 | -0.446997 |
| H | -0.709473 | -0.997482 | -0.399556 |
| H | 0.508390 | -0.132381 | 0.493766 |
| C | 2.108456 | 0.183373 | -1.677431 |
| O | 2.800398 | -0.013096 | -2.697307 |
| N | 2.551549 | 0.905494 | -0.600379 |
| H | 1.956789 | 0.989965 | 0.219028 |
| C | 2.198125 | -1.727744 | 2.416859 |
| C | 3.567309 | -1.952888 | 2.202938 |
| C | 1.791555 | -0.664752 | 3.235192 |
| C | 4.517860 | -1.126195 | 2.788510 |
| H | 3.880196 | -2.771368 | 1.566552 |
| C | 2.759945 | 0.150556 | 3.823721 |
| H | 0.742537 | -0.485988 | 3.406697 |
| C | 4.120090 | -0.069406 | 3.610629 |
| H | 5.567757 | -1.309061 | 2.584998 |
| H | 2.432863 | 0.974196 | 4.450978 |
| C | 4.849826 | -2.479869 | -1.008500 |
| C | 5.486692 | -1.237898 | -1.128939 |
| C | 5.496169 | -3.527613 | -0.339206 |
| C | 6.729278 | -1.024204 | -0.542961 |
| H | 4.992875 | -0.439723 | -1.667169 |
| C | 6.743175 | -3.299332 | 0.243629 |
| H | 5.013837 | -4.490027 | -0.254292 |
| C | 7.363400 | -2.052072 | 0.157145 |
| H | 7.186581 | -0.043096 | -0.625562 |
| H | 7.232317 | -4.112839 | 0.771400 |
| C | 3.740893 | 1.652826 | -0.516720 |
| C | 4.456347 | 2.104902 | -1.634289 |
| C | 4.192639 | 1.971431 | 0.771991 |
| C | 5.625791 | 2.841555 | -1.446727 |
| H | 4.107957 | 1.855849 | -2.626406 |
| C | 5.355095 | 2.716467 | 0.941205 |
| H | 3.639648 | 1.609371 | 1.630643 |
| C | 6.085169 | 3.152306 | -0.166336 |
| H | 6.179166 | 3.179136 | -2.318196 |

| | | | |
|---|---|---|---|
| H | 5.696825 | 2.943472 | 1.946759 |
| H | 4.860432 | 0.578956 | 4.068937 |
| H | 8.330387 | -1.887119 | 0.622079 |
| H | 6.997292 | 3.725403 | -0.033647 |
| C | -3.012812 | -3.504891 | 1.688328 |
| H | -3.147080 | -4.588342 | 1.727717 |
| H | -2.562864 | -3.209913 | 2.638707 |
| N | -4.325976 | -2.887405 | 1.536270 |
| C | -5.441356 | -3.666024 | 1.003695 |
| H | -6.331945 | -3.487836 | 1.613710 |
| H | -5.167177 | -4.717940 | 1.113915 |
| C | -4.539950 | -1.538619 | 1.742285 |
| N | -3.502121 | -0.823944 | 2.285122 |
| H | -2.579172 | -1.254956 | 2.355515 |
| O | -5.629715 | -1.033605 | 1.416108 |
| C | -3.489258 | 0.570552 | 2.519820 |
| C | -2.231253 | 1.180037 | 2.648321 |
| C | -4.648791 | 1.351558 | 2.641006 |
| C | -2.132994 | 2.552761 | 2.857902 |
| H | -1.331259 | 0.583073 | 2.560940 |
| C | -4.532443 | 2.727103 | 2.841805 |
| H | -5.618609 | 0.884488 | 2.556445 |
| C | -3.282686 | 3.339562 | 2.945609 |
| H | -1.143829 | 2.991645 | 2.930075 |
| H | -5.437363 | 3.322246 | 2.923648 |
| H | -3.205420 | 4.411414 | 3.099109 |
| C | -5.822874 | -3.380944 | -0.465639 |
| N | -4.704864 | -3.390682 | -1.394553 |
| C | -4.241499 | -4.691567 | -1.845382 |
| H | -5.078106 | -5.263404 | -2.262452 |
| H | -3.486426 | -4.542222 | -2.614265 |
| H | -3.805844 | -5.278220 | -1.024617 |
| C | -4.037826 | -2.262360 | -1.843917 |
| O | -3.073462 | -2.347283 | -2.614049 |
| N | -4.526376 | -1.061887 | -1.373458 |
| H | -5.105341 | -1.059159 | -0.539124 |
| C | -4.116906 | 0.222797 | -1.780090 |
| C | -3.519965 | 0.493338 | -3.020245 |
| C | -4.387326 | 1.278582 | -0.896550 |
| C | -3.199050 | 1.810948 | -3.350890 |
| H | -3.307339 | -0.319532 | -3.699784 |
| C | -4.052258 | 2.583704 | -1.238054 |
| H | -4.849889 | 1.066609 | 0.060063 |
| C | -3.456707 | 2.862287 | -2.469193 |
| H | -2.736907 | 2.012777 | -4.312813 |
| H | -4.253761 | 3.379385 | -0.528177 |
| H | -3.193270 | 3.881615 | -2.730623 |
| C | -0.888306 | 1.117737 | -0.587994 |
| N | -0.140305 | 2.367843 | -0.710048 |
| C | 0.061558 | 2.931704 | -2.040222 |
| H | 0.998534 | 3.489074 | -2.061899 |
| H | 0.133015 | 2.110989 | -2.756988 |
| H | -0.754927 | 3.592635 | -2.344311 |
| C | 0.457255 | 2.875456 | 0.410795 |
| O | 0.758984 | 2.158893 | 1.380728 |

| | | | |
|---|---|---|---|
| N | 0.762594 | 4.229001 | 0.392630 |
| C | -0.153600 | 5.172734 | -0.165629 |
| C | -1.533812 | 5.018978 | 0.011148 |
| C | 0.333311 | 6.264508 | -0.891541 |
| C | -2.413208 | 5.940655 | -0.554061 |
| H | -1.914108 | 4.177640 | 0.578588 |
| C | -0.550983 | 7.193412 | -1.436577 |
| H | 1.403348 | 6.367910 | -1.041189 |
| C | -1.928216 | 7.032067 | -1.276433 |
| H | -3.482350 | 5.811235 | -0.414841 |
| H | -0.162910 | 8.035771 | -2.000856 |
| H | -2.616491 | 7.750857 | -1.709436 |
| H | -1.515967 | 1.036766 | -1.473518 |
| H | -1.552759 | 1.182405 | 0.274141 |
| H | -6.535045 | -4.153846 | -0.780571 |
| H | -6.352857 | -2.433005 | -0.525029 |
| C | 1.660860 | 4.700487 | 1.450609 |
| H | 1.150757 | 4.752827 | 2.419645 |
| H | 2.507499 | 4.020126 | 1.533227 |
| H | 2.021711 | 5.694430 | 1.184991 |

**6c** Conformation 3

B3LYP-D3(BJ)/6-31G(d,p) Energy = -3243.410674
B3LYP-D3(BJ)/6-311G(d,p)/IEF-PCM(DCM) Energy = -3244.156034
B3LYP-D3(BJ)/6-311G(d,p)/IEF-PCM(DCM)//B3LYP-D3(BJ)/6-31G(d,p) Quasiharmonic Free Energy = -3243.114514

Frequencies (Top 3 out of 405)

1. 15.8495 $cm^{-1}$
2. 19.2611 $cm^{-1}$
3. 20.6169 $cm^{-1}$

B3LYP-D3(BJ)/6-31G(d,p) Molecular Geometry in Cartesian Coordinates

| | | | |
|---|---|---|---|
| C | -0.059102 | -4.322943 | -1.304323 |
| H | -0.313361 | -5.321104 | -1.689716 |
| H | 0.836521 | -3.983265 | -1.831490 |
| C | 0.300404 | -4.508113 | 0.181105 |
| H | -0.555916 | -4.888108 | 0.729101 |
| H | 1.063563 | -5.295954 | 0.209366 |
| N | -1.119131 | -3.367753 | -1.654040 |
| N | 0.856710 | -3.341239 | 0.852601 |
| C | -2.366933 | -3.552858 | -1.089172 |
| O | -2.516568 | -4.361100 | -0.153472 |
| C | 0.181364 | -2.482638 | 1.696500 |
| O | 0.812176 | -1.592430 | 2.297574 |
| N | -3.401994 | -2.798144 | -1.578151 |
| N | -1.168308 | -2.647463 | 1.797633 |
| H | -3.185472 | -2.001567 | -2.174195 |
| H | -1.614544 | -3.342722 | 1.200776 |

| | | | |
|---|---|---|---|
| C | -0.919414 | -2.614116 | -2.895422 |
| H | -1.872517 | -2.492846 | -3.406342 |
| H | -0.286314 | -3.206016 | -3.565422 |
| C | 2.246500 | -3.012412 | 0.584882 |
| H | 2.548081 | -3.555609 | -0.306349 |
| H | 2.336189 | -1.951631 | 0.345052 |
| C | -0.255748 | -1.237954 | -2.700489 |
| H | -0.364624 | -0.680986 | -3.637008 |
| H | 0.812391 | -1.365200 | -2.512522 |
| N | -0.805268 | -0.471936 | -1.585533 |
| C | 0.076179 | -0.182311 | -0.460158 |
| H | -0.466123 | -0.214320 | 0.481566 |
| H | 0.808481 | -0.985101 | -0.419687 |
| C | -2.075528 | 0.021742 | -1.703970 |
| O | -2.743998 | -0.201024 | -2.733815 |
| N | -2.568891 | 0.709868 | -0.625734 |
| H | -1.996619 | 0.796906 | 0.209235 |
| C | -2.088711 | -1.791348 | 2.448253 |
| C | -1.731374 | -0.703408 | 3.256136 |
| C | -3.446204 | -2.073637 | 2.226903 |
| C | -2.735799 | 0.080987 | 3.825574 |
| H | -0.691807 | -0.478725 | 3.430221 |
| C | -4.433508 | -1.278023 | 2.794492 |
| H | -3.721204 | -2.911766 | 1.598704 |
| C | -4.084460 | -0.195190 | 3.605040 |
| H | -2.446263 | 0.924823 | 4.444449 |
| H | -5.473683 | -1.505389 | 2.585989 |
| C | -4.688642 | -2.711411 | -0.989831 |
| C | -5.296502 | -3.763107 | -0.291567 |
| C | -5.370001 | -1.497018 | -1.144506 |
| C | -6.551206 | -3.564559 | 0.285567 |
| H | -4.779190 | -4.704544 | -0.180641 |
| C | -6.619829 | -1.313000 | -0.563812 |
| H | -4.905454 | -0.696804 | -1.705739 |
| C | -7.216351 | -2.343575 | 0.164768 |
| H | -7.010663 | -4.380415 | 0.835874 |
| H | -7.113211 | -0.352180 | -0.673179 |
| C | -3.758485 | 1.459921 | -0.577316 |
| C | -4.440585 | 1.910765 | -1.716540 |
| C | -4.242502 | 1.788696 | 0.696561 |
| C | -5.605914 | 2.661759 | -1.564713 |
| H | -4.069151 | 1.651479 | -2.697643 |
| C | -5.400939 | 2.546592 | 0.830248 |
| H | -3.714050 | 1.429891 | 1.571746 |
| C | -6.095525 | 2.985694 | -0.298776 |
| H | -6.132240 | 2.999689 | -2.452624 |
| H | -5.766605 | 2.783646 | 1.825056 |
| H | -4.853629 | 0.428989 | 4.049349 |
| H | -8.188998 | -2.201155 | 0.625306 |
| H | -7.004386 | 3.569625 | -0.193747 |
| C | 3.206168 | -3.321958 | 1.747039 |
| H | 2.747103 | -3.024081 | 2.691957 |
| H | 3.398559 | -4.395147 | 1.815938 |
| N | 4.483600 | -2.639795 | 1.566906 |
| C | 5.631703 | -3.370333 | 1.035388 |

| | | | |
|---|---|---|---|
| H | 5.413012 | -4.432594 | 1.168021 |
| H | 6.518562 | -3.135562 | 1.631612 |
| C | 4.628217 | -1.276464 | 1.735594 |
| N | 3.561921 | -0.602256 | 2.274731 |
| H | 2.663521 | -1.078368 | 2.366990 |
| O | 5.685976 | -0.724220 | 1.381386 |
| C | 3.479121 | 0.796472 | 2.469939 |
| C | 4.596932 | 1.641751 | 2.541445 |
| C | 2.192940 | 1.341981 | 2.607464 |
| C | 4.410480 | 3.015703 | 2.696095 |
| H | 5.588814 | 1.224533 | 2.453193 |
| C | 2.025324 | 2.714481 | 2.770256 |
| H | 1.324109 | 0.696290 | 2.559066 |
| C | 3.131928 | 3.564396 | 2.803101 |
| H | 5.283540 | 3.660556 | 2.737584 |
| H | 1.016685 | 3.104226 | 2.851491 |
| H | 2.999138 | 4.635661 | 2.916469 |
| C | 5.983804 | -3.094360 | -0.442972 |
| N | 4.860664 | -3.186310 | -1.361398 |
| C | 4.469090 | -4.520792 | -1.781530 |
| H | 4.071545 | -5.113196 | -0.945733 |
| H | 3.702981 | -4.431328 | -2.548738 |
| H | 5.334744 | -5.053158 | -2.191407 |
| C | 4.128189 | -2.107800 | -1.829794 |
| O | 3.164278 | -2.264814 | -2.589525 |
| N | 4.550532 | -0.870997 | -1.390525 |
| H | 5.137847 | -0.816482 | -0.563696 |
| C | 4.071465 | 0.379495 | -1.826617 |
| C | 3.440519 | 0.584474 | -3.062662 |
| C | 4.304467 | 1.472959 | -0.978663 |
| C | 3.048897 | 1.874498 | -3.424465 |
| H | 3.257297 | -0.257066 | -3.715236 |
| C | 3.900367 | 2.749938 | -1.351048 |
| H | 4.792500 | 1.311965 | -0.024766 |
| C | 3.270186 | 2.962749 | -2.578336 |
| H | 2.560431 | 2.025008 | -4.382727 |
| H | 4.076776 | 3.575118 | -0.668145 |
| H | 2.952649 | 3.959647 | -2.865249 |
| C | 0.836171 | 1.136725 | -0.591174 |
| N | 0.007686 | 2.340407 | -0.664600 |
| C | -0.262197 | 2.907217 | -1.982015 |
| H | 0.540650 | 3.566689 | -2.322868 |
| H | -1.197105 | 3.466631 | -1.957414 |
| H | -0.373044 | 2.088516 | -2.696732 |
| C | -0.607774 | 2.770679 | 0.478606 |
| O | -0.856795 | 1.994273 | 1.417019 |
| N | -0.973947 | 4.109914 | 0.527990 |
| C | -0.205832 | 5.135511 | -0.082525 |
| C | -0.842693 | 6.263811 | -0.612112 |
| C | 1.193983 | 5.051392 | -0.126566 |
| C | -0.091352 | 7.286249 | -1.190735 |
| H | -1.924758 | 6.332554 | -0.582263 |
| C | 1.933128 | 6.068306 | -0.723625 |
| H | 1.694989 | 4.192704 | 0.302887 |
| C | 1.298147 | 7.191727 | -1.258291 |

| | | | |
|---|---|---|---|
| H | -0.599187 | 8.154406 | -1.599641 |
| H | 3.015956 | 5.989221 | -0.752666 |
| H | 1.880237 | 7.985311 | -1.715425 |
| H | 1.515212 | 1.225788 | 0.257030 |
| H | 1.445364 | 1.118244 | -1.492779 |
| H | 6.459104 | -2.119675 | -0.526031 |
| H | 6.735646 | -3.832511 | -0.748917 |
| C | -2.046140 | 4.492491 | 1.449168 |
| H | -2.984475 | 4.679622 | 0.916853 |
| H | -2.196968 | 3.671257 | 2.146802 |
| H | -1.754727 | 5.390999 | 1.998169 |

**Full Gaussian Reference**

Frisch, M. J.; Trucks, G. W.; Schlegel, H. B.; Scuseria, G. E.; Robb, M. A.; Cheeseman, J. R.; Scalmani, G.; Barone, V.; Mennucci, B.; Petersson, G. A.; Nakatsuji, H.; Caricato, M.; Li, X.; Hratchian, H. P.; Izmaylov, A. F.; Bloino, J.; Zheng, J.; Sonnenberg, J. L.; Hada, M.; Ehara, M.; Toyota, K.; Fukuda, R.; Hasegawa, J.; Ishida, M.; Nakajima, T.; Honda, Y.; Kitao, O.; Nakai, H.; Vreven, T.; Montgomery, J. A.; Peralta, J. E.; Ogliaro, F.; Bearpark, M.; Heyd, J. J.; Brothers, E.; Kudin, K. N.; Staroverov, V. N.; Kobayashi, R.; Normand, J.; Raghavachari, K.; A. Rendell, J. C.; Burant, S.; Iyengar, S.; Tomasi, J.; Cossi, M.; Rega, N.; Millam, J. M.; Klene, M.; Knox, J. E.; Cross, J. B.; Bakken, V.; Adamo, C.; Jaramillo, J.; Gomperts, R.; Stratmann, R. E.; Yazyev, O.; Austin, A. J.; Cammi, R.; Pomelli, C.; Ochterski, J. W.; Martin, R. L.; Morokuma, K.; Zakrzewski, V. G.; Voth, G. A.; Salvador, P.; Dannenberg, J. J.; Dapprich, S.; Daniels, A. D.; Farkas, O.; Foresman, J. B.; Ortiz, J. V.; Cioslowski, J.; Fox, D. J. Gaussian 16, Revision A.03. Gaussian, Inc.: Wallingford CT 2016.

## References


1. Galaup, C., Couchet, J.-M., Bedel, S., Tisnès, P., and Picard, C. (2005). Direct Access to Terpyridine-Containing Polyazamacrocycles as Photosensitizing Ligands for Eu(III) Luminescence in Aqueous Media. J. Org. Chem. *70*, 2274–2284.

2. Nowick, J.S., Mahrus, S., Smith, E.M., and Ziller, J.W. (1996). Triurea Derivatives of Diethylenetriamine as Potential Templates for the Formation of Artificial β-Sheets1. J. Am. Chem. Soc. *118*, 1066–1072.

3. Campaña, A.G., Leigh, D.A., and Lewandowska, U. (2013). One-Dimensional Random Walk of a Synthetic Small Molecule Toward a Thermodynamic Sink. J. Am. Chem. Soc. *135*, 8639–8645.

4. Robinson, A., Thomas, G.L., Spandl, R.J., Welch, M., and Spring, D.R. (2008). Gemmacin B: bringing diversity back into focus. Org. Biomol. Chem. *6*, 2978–2981.

5. Si, C., Fales, K.R., Torrado, A., Frimpong, K., Kaoudi, T., Vandeveer, H.G., and Njoroge, F.G. (2016). Enantioselective Synthesis of 3,3-Difluoropyrrolidin-4-ol, a Valuable Building Block in Medicinal Chemistry. J. Org. Chem. *81*, 4359–4363.

6. Fujihara, T., Katafuchi, Y., Iwai, T., Terao, J., and Tsuji, Y. (2010). Palladium-Catalyzed Intermolecular Addition of Formamides to Alkynes. J. Am. Chem. Soc. *132*, 2094–2098.

7. Hamilton, S.K., and Harth, E. (2009). Molecular Dendritic Transporter Nanoparticle Vectors Provide Efficient Intracellular Delivery of Peptides. ACS Nano *3*, 402–410.

8. Zhang, G.-D., Nishiyama, N., Harada, A., Jiang, D.-L., Aida, T., and Kataoka, K. (2003). pH-

sensitive Assembly of Light-Harvesting Dendrimer Zinc Porphyrin Bearing Peripheral Groups of Primary Amine with Poly(ethylene glycol)-b-poly(aspartic acid) in Aqueous Solution. Macromolecules *36*, 1304–1309.

9. Castañeda, L., Maruani, A., Schumacher, F.F., Miranda, E., Chudasama, V., Chester, K.A., Baker, J.R., Smith, M.E.B., and Caddick, S. (2013). Acid-cleavable thiomaleamic acid linker for homogeneous antibody–drug conjugation. Chem. Commun. *49*, 8187–8189.

10. Huang, H., Yu, C., Zhang, Y., Zhang, Y., Mariano, P.S., and Wang, W. (2017). Chemo- and Regioselective Organo-Photoredox Catalyzed Hydroformylation of Styrenes via a Radical Pathway. J. Am. Chem. Soc. *139*, 9799–9802.

11. Mohamadi, F., Richards, N.G.J., Guida, W.C., Liskamp, R., Lipton, M., Caufield, C., Chang, G., Hendrickson, T., and Still, W.C. (1990). Macromodel—an integrated software system for modeling organic and bioorganic molecules using molecular mechanics. J. Comput. Chem. *11*, 440–467.

12. Macromodel, version 11.3. (2016).

13. Harder, E., Damm, W., Maple, J., Wu, C., Reboul, M., Xiang, J.Y., Wang, L., Lupyan, D., Dahlgren, M.K., Knight, J.L., et al. (2016). OPLS3: A Force Field Providing Broad Coverage of Drug-like Small Molecules and Proteins. J. Chem. Theory Comput. *12*, 281–296.

14. Chang, G., Guida, W.C., and Still, W.C. (1989). An internal-coordinate Monte Carlo method for searching conformational space. J. Am. Chem. Soc. *111*, 4379–4386.

15. Kolossváry, I., and Guida, W.C. (1999). Low-mode conformational search elucidated: Application to C39H80 and flexible docking of 9-deazaguanine inhibitors into PNP. J. Comput. Chem. *20*, 1671–1684.

16. Frisch, M.J., Trucks, G.W., Schlegel, H.B., and Scuseria, G. (2016). Gaussian16 Revision A. 03 (Wallingford, CT: Gaussian Inc.).

17. Becke, A.D. (1993). Density-functional thermochemistry. III. The role of exact exchange. J. Chem. Phys. *98*, 5648–5652.

18. Stephens, P.J., Devlin, F.J., Chabalowski, C.F., and Frisch, M.J. (1994). Ab Initio Calculation of Vibrational Absorption and Circular Dichroism Spectra Using Density Functional Force Fields. J. Phys. Chem. *98*, 11623–11627.

19. Grimme, S., Antony, J., Ehrlich, S., and Krieg, H. (2010). A consistent and accurate ab initio parametrization of density functional dispersion correction (DFT-D) for the 94 elements H-Pu. J. Chem. Phys. *132*, 154104.

20. Becke, A.D., and Johnson, E.R. (2005). A density-functional model of the dispersion interaction. J. Chem. Phys. *123*, 154101.

21. Johnson, E.R., and Becke, A.D. (2006). A post-Hartree-Fock model of intermolecular interactions: Inclusion of higher-order corrections. J. Chem. Phys. *124*, 174104.

22. Grimme, S., Ehrlich, S., and Goerigk, L. (2011). Effect of the damping function in dispersion corrected density functional theory. J. Comput. Chem. *32*, 1456–1465.

23. Hehre, W.J., Ditchfield, R., and Pople, J.A. (1972). Self—Consistent Molecular Orbital Methods. XII. Further Extensions of Gaussian—Type Basis Sets for Use in Molecular Orbital Studies of Organic Molecules. J. Chem. Phys. *56*, 2257–2261.

24. Simón, L., and Goodman, J.M. (2011). How reliable are DFT transition structures? Comparison of GGA, hybrid-meta-GGA and meta-GGA functionals. Org. Biomol. Chem. *9*, 689–700.

25. Krishnan, R., Binkley, J.S., Seeger, R., and Pople, J.A. (1980). Self-consistent molecular orbital methods. XX. A basis set for correlated wave functions. J. Chem. Phys. *72*, 650–654.

26. Mennucci, B., Cammi, R., and Tomasi, J. (1998). Excited states and solvatochromic shifts within a nonequilibrium solvation approach: A new formulation of the integral equation formalism method at the self-consistent field, configuration interaction, and multiconfiguration self-consistent field level. J. Chem. Phys. *109*, 2798–2807.

27. Grimme, S. (2012). Supramolecular Binding Thermodynamics by Dispersion-Corrected Density Functional Theory. Chem. – A Eur. J. *18*, 9955–9964.

28. Funes-Ardoiz, I., Paton, R.S. (2018) Goodvibes: version 2.0.3. doi:10.5281/zenodo.595246.

29. Alecu, I.M., Zheng, J., Zhao, Y., and Truhlar, D.G. (2010). Computational Thermochemistry: Scale Factor Databases and Scale Factors for Vibrational Frequencies Obtained from Electronic Model Chemistries. J. Chem. Theory Comput. *6*, 2872–2887.

30. Grayson, M.N., and Houk, K.N. (2016). Cinchona Urea-Catalyzed Asymmetric Sulfa-Michael Reactions: The Brønsted Acid−Hydrogen Bonding Model. J. Am. Chem. Soc. *138*, 9041–9044.

31. Grayson, M.N. (2017). Mechanism and Origins of Stereoselectivity in the Cinchona Thiourea- and Squaramide-Catalyzed Asymmetric Michael Addition of Nitroalkanes to Enones. J. Org. Chem. *82*, 4396–4401.

32. Rodríguez-Guerra Pedregal, J., Gómez-Orellana, P., and Maréchal, J.-D. (2018). ESIgen: Electronic Supporting Information Generator for Computational Chemistry Publications. J. Chem. Inf. Model. *58*, 561–564.
