# Supplementary material for: A molecular communication channel consisting of a single reversible chain of hydrogen bonds in a conformationally flexible oligomer
Source: Chem. 2021 Sep 9;7(9):2460–72. doi: 10.1016/j.chempr.2021.06.022 (PMC8442760; doi:10.1016/j.chempr.2021.06.022)
Supplement: Document S2. Article plus supplemental information [file mmc2.pdf]

Chem

CellPress
OPEN ACCESS

Article

A molecular communication channel consisting of a single reversible chain of hydrogen bonds in a conformationally flexible oligomer

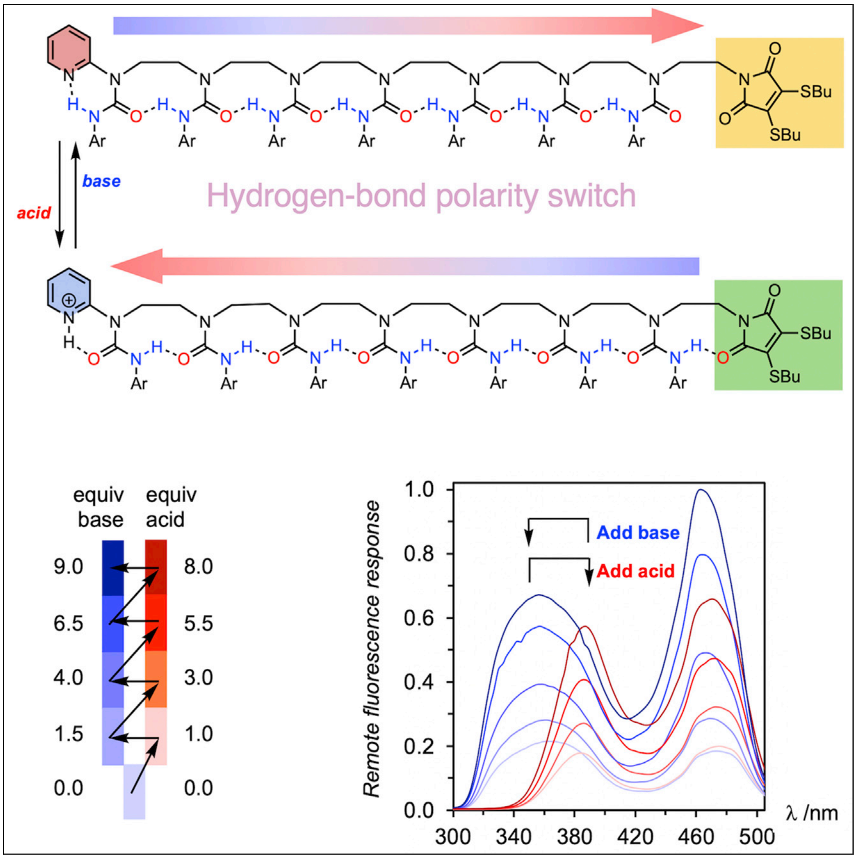

Information in nature is stored, communicated, and amplified by matching hydrogen-bond polarity in base pairs. Here, we show that structurally simple synthetic molecules can also use hydrogen-bond polarity to communicate information. Molecules with coherent chains of hydrogen bonds respond to changes in their environment by reversing the polarity of the chain, allowing messages to be communicated from one end of the molecule to the other. This work reveals a new mechanism for information handling in synthetic molecular systems.

3 GOOD HEALTH AND WELL-BEING

9 INDUSTRY, INNOVATION AND INFRASTRUCTURE

David T.J. Morris, Steven M. Wales, David P. Tilly, Elliot H.E. Farrar, Matthew N. Grayson, John W. Ward, Jonathan Clayden

j.clayden@bristol.ac.uk

Highlights

Simple urea oligomers organize themselves with a single coherent hydrogen-bond chain

Reversing the polarity of the hydrogen bonding sends a message through the molecule

Conformational messages can be transmitted by changes in pH or by anion binding

Receipt of the conformational message is indicated by a remote fluorescent response

Morris et al., Chem 7, 2460–2472
September 9, 2021 © 2021 The Authors.
Published by Elsevier Inc.
https://doi.org/10.1016/j.chempr.2021.06.022

CellPress
OPEN ACCESS

Chem

Article

A molecular communication channel consisting of a single reversible chain of hydrogen bonds in a conformationally flexible oligomer

David T.J. Morris,[1] Steven M. Wales,[1] David P. Tilly,[1] Elliot H.E. Farrar,[2] Matthew N. Grayson,[2] John W. Ward,[1] and Jonathan Clayden[1,3,*]

SUMMARY

Communication of information through the global switching of conformation in synthetic molecules has hitherto entailed the inversion of chirality. Here, we report a class of oligomer through which information may be communicated through a global reversal of polarity. Ethylene-bridged oligoureas are constitutionally symmetrical, conformationally flexible molecules organized by a single chain of hydrogen bonds running the full length of the oligomer. NMR reveals that this hydrogen-bonded chain may undergo a coherent reversal of directionality. The directional uniformity of the hydrogen-bond chain allows it to act as a channel for the spatial communication of information on a molecular scale. A binding site at the terminus of an oligomer detects local information about changes in pH or anion concentration and transmits that information—in the form of a directionality switch in the hydrogen-bond chain—to a remote polarity-sensitive fluorophore. This propagation of polarity-encoded information provides a new mechanism for molecular communication.

The bigger picture

Despite the progress made by synthetic chemistry in building the molecular structures of nature, there are still domains of molecular science where biology is immensely more sophisticated than even the most advanced artificial chemical systems. One of these is the ability to use molecular structures to encode and communicate information. Nature stores information in the hydrogen-bond polarity of the base pairs and translates it into molecular function using the polarity matching of these bonds. Here, we outline an early step toward replicating this ability in the design, synthesis, and operation of a series of synthetic molecular devices that employ a structurally simple family of oligomeric molecules that can communicate information in the form of hydrogen-bond polarity. This study marks a significant step in the design of molecular systems that manipulate information, which will form the basis of the many compartmentalization-based nanotechnologies of the future.

INTRODUCTION

The general communication device theorized by Claude Shannon in the 1940s comprises an information source (input), a transmitter (which translates the information into a communicable form), a communication channel (the medium through which information is communicated), and a receiver (output).[1] All macroscopic machinery can be reduced to informational inputs resulting in functional outputs, with the perturbation of a physical field (for example, a force exerted on a solid or the oscillation of an electromagnetic field) providing a communication channel. In artificial and biological molecular machines and devices, a chemical input is translated into molecular function. Chemical inputs and outputs are numerous (examples include the modulation of chromophores by pH, in indicators, or fluorescent responses to ligand binding), but molecular manifestations of communication channels remain sparse. Nature has mastered the manipulation of information at the molecular level, commonly using conformational changes mediated by supramolecular interactions as the communication channels,[2,3] and synthetic information-processing mechanisms have been designed, which exploit competitive interactions and constitutional changes in chemical systems.[4–7] Progress toward synthetic molecular communication channels that allow spatial separation between input and output has likewise exploited conformational change, but has so far been limited to the use of a small number of rigid helical foldamers that may be induced to undergo a global conformational switch between two states: a left- and a right-handed screw sense (Figure 1A).[8] This chiral switch has limited such devices to receivers that induce

 © 2021 The Authors. Published by Elsevier Inc.
This is an open access article under the CC BY license (http://creativecommons.org/licenses/by/4.0/).

CellPress
OPEN ACCESS

stereochemically mediated outputs (for example, control of diastereo- or enantioselective reactions,[9,10] or modulation of fluorescence in a chiral and enantioenriched fluorophore[11]).

We now report the implementation of a more general design for a molecular communication channel that does not rely on the chirality of a conformationally rigid foldamer but instead exploits a global reversal of directionality in a chain of hydrogen bonds within a conformationally flexible oligomer (Figure 1B). We show that even in extended oligomers and in polar and hydrogen-bonding solvents, this continuous chain of hydrogen bonds is robust, meaning that an induced change in hydrogen-bond directionality at one point in the molecule leads to a global conformational response, communicating that response through the molecule regardless of the molecule's overall conformational flexibility.

The structures in question are the ethylene-bridged oligoureas[12,13] shown in Figure 1C. These linear *N*-linked ethylenediamine oligomers carry conformationally mobile side chains, each with a single NH and C=O group, connected through a single chain of hydrogen bonds running the full length of the oligomeric structure. Many oligomeric structures, including natural peptides and many foldamers (synthetic analogs of biopolymers with well-defined conformations), maintain a secondary structure through a network of intramolecular hydrogen bonds.[14,15] In most cases, the directionality of these hydrogen bonds arises constitutionally from the structure of the foldamer. For example, amide-based foldamers, in common with the peptides they mimic,[16] have a defined N terminus that acts as a hydrogen-bond donor and a defined C terminus that acts as a hydrogen-bond acceptor. However, in oligomers built from symmetrical monomers and linked through constitutionally symmetrical functional groups, hydrogen-bond directionality is potentially reversible. Such oligomers are limited to a single example from our laboratory: 2.5-helical foldamers, which are formed by connecting symmetrical (*meso*) cyclohexanediamines through carbonyl groups to form *N*,*N*′-disubstituted ureas.[17] We previously showed that the hydrogen bonds that rigidify these foldamers select between two alternative helix screw senses with opposite hydrogen-bond directionalities according to the hydrogen-bonding capabilities of the monomer units, and that their directionality can be reversed by anionic ligands, leading to a global change in screw sense, detectable by circular dichroism.[18] We now show, using the structurally distinct oligomeric scaffolds shown in Figure 1C, that global hydrogen-bond directionality switching is a more general phenomenon that may be decoupled from chirality, and that extended molecules containing a single, reversible chain of hydrogen bonds may be used as channels for the spatial communication of polarity-encoded information.

These *N*-linked ethylenediamine oligomers are constitutionally symmetrical and conformationally flexible structures with trisubstituted urea side chains. They are devoid of chirality, but each urea side chain is able to adopt either an *E* or a *Z* conformation. A unique structural feature allows this conformational feature to function as a communication channel: a single chain of hydrogen bonds links every urea side chain to its neighbors and runs the full length of the oligomer, meaning that the oligomer is characterized by a global, but reversible, coherent hydrogen-bond directionality. Ethylene-bridged oligoureas were explored by Nowick et al.[19–22] as geometric mimics of β-sheets (Figure 1D), who found that the population of non-hydrogen-bonded conformations of trimeric structures was undetectable by FTIR. Two global conformations are thus available, in which either terminal urea acts as a hydrogen-bond acceptor, but the established preference for an aryl group to lie *trans* to the

[1]School of Chemistry, University of Bristol, Cantock's Close, Bristol BS8 1TS, UK
[2]Department of Chemistry, University of Bath, Claverton Down, Bath BA2 7AY, UK
[3]Lead contact
*Correspondence: j.clayden@bristol.ac.uk
https://doi.org/10.1016/j.chempr.2021.06.022

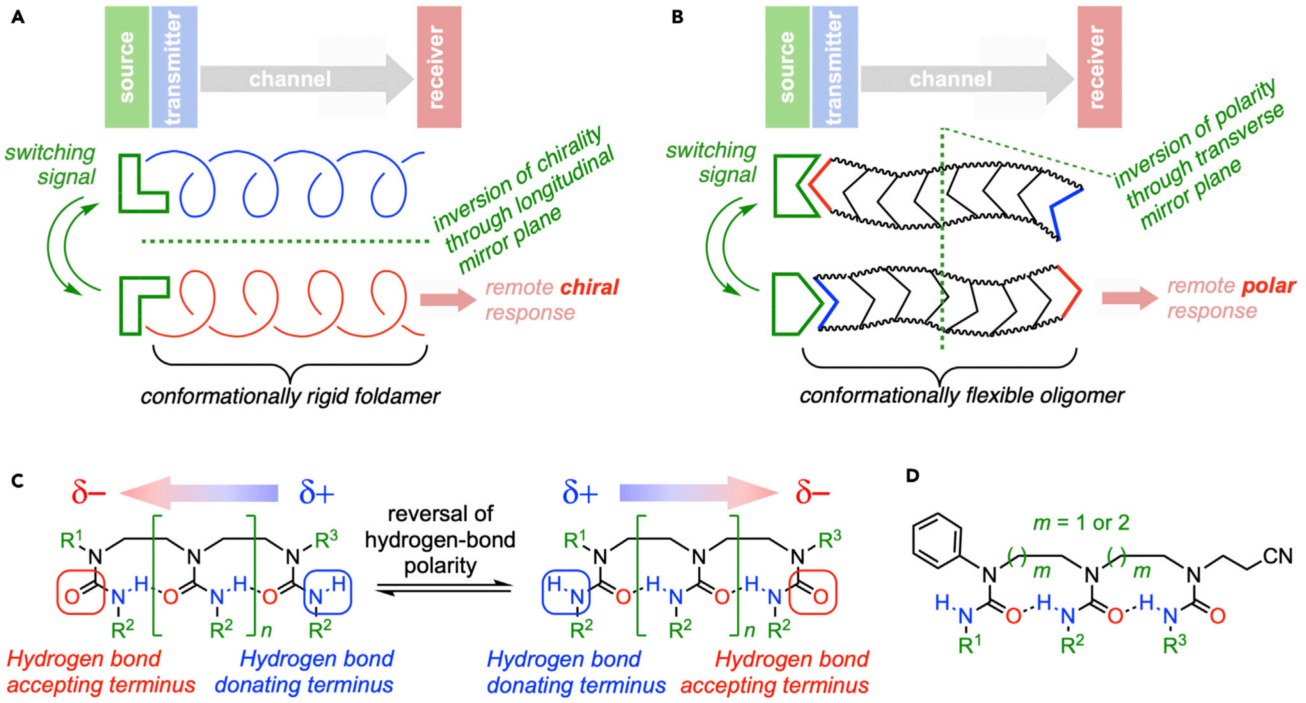

Figure 1. Strategies and structures for molecular communication of information

(A) A previous model for the molecular communication of information using chirality switching in rigid foldamers.

(B) This work: a mechanism for communicating information using a communication channel that involves polarity switching in a conformationally flexible oligomer.

(C) Reversible directionality in hydrogen-bonded ethylene-bridged oligoureas.

(D) Beta-sheet mimics reported by Nowick.

carbonyl group in an *N*,*N*-disubstituted urea or amide[23–26] meant that in Nowick's oligomers only the hydrogen-bond directionality illustrated in Figure 1D was populated.

RESULTS AND DISCUSSION

Coherent and reversible hydrogen-bond polarity in ethylene-bridged oligoureas

Ethylene-bridged oligoureas were made straightforwardly from available precursors as outlined in Figure 2A (and in detail in Schemes S1–S9). Reversible hydrogen-bond directionality in these systems was investigated initially in a homologous series of ureas **1**–**4** (Figure 2). Although **1**–**4** are constitutionally symmetrical, their NMR spectra at sub-ambient temperatures reveal a break of symmetry that results in two methylene signals of equal intensity (Figure 2B)—a result that can be explained only if the multiple urea functions are connected by an unbroken but reversible chain of hydrogen bonds.

Coalescence between the benzylic methylene signals of the monourea **1** (Figure 2B) indicated a barrier to rotation about the indicated urea C–N bond $\Delta G^{\ddagger}_{298\ K}$ = 48.9 kJ mol^{-1} (from the line-shape and Eyring analysis: Table S1), and at −80 °C, the ^{1}H NMR spectrum of the urea lies in the slow-exchange regime. The ^{1}H NMR spectrum of di-urea **2a**, consisting of two equally populated singlets at low temperature (Figure 2B), is most reasonably explained by a hydrogen bond that correlates the directionality of the two urea functions and breaks the constitutional symmetry of the molecule on the ^{1}H NMR timescale. The two degenerate conformers interconvert through a

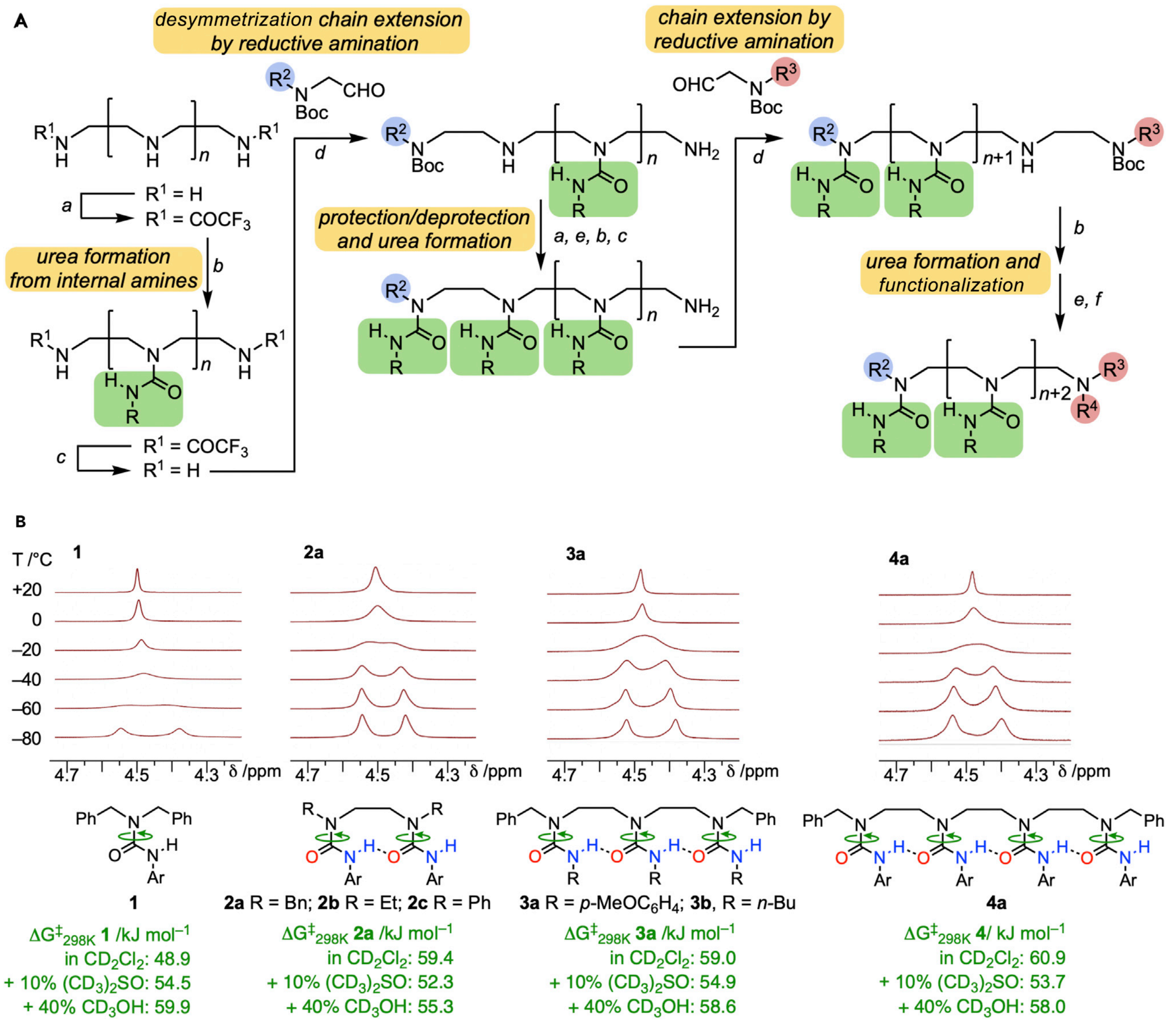

Figure 2. Symmetrical ethylene-bridged oligoureas: synthesis and reversible hydrogen bonding

(A) An overview of the typical synthetic approach to ethylene-bridged oligoureas. Reagents and conditions: *a* $EtOCOCF_3$, CH_2Cl_2, 0°C - rt; *b* ArNCO, CH_2Cl_2, rt; *c* NaOH, H_2O, MeOH or EtOH, rt; *d* 1. RCHO, EtOH, rt; 2. $NaBH_4$, EtOH, 0°C - rt; *e* CF_3CO_2H, CH_2Cl_2, rt; *f* E^+, CH_2Cl_2, rt.

(B) Structures, dynamic ^1H NMR spectra, and barriers to directionality inversion of a homologous series of constitutionally symmetrical oligomers **1–4**. NMR spectra shown were acquired at 300 MHz in CD_2Cl_2 (for **1**) or 90:10 CD_2Cl_2 + $(CD_3)_2SO$ (for **2–4**) at a concentration of 10 mg mL^{-1}. Ar = *p*-$MeOC_6H_4$.

barrier $\Delta G^{\ddagger}_{298\ K}$ = 59.4 kJ mol^{-1} in CD_2Cl_2. No other conformer is evident for either 2a or 2b, indicating that no symmetrical structure is populated. Such a structure, lacking an intramolecular hydrogen bond, is however evident to some extent (about 40% at −80 °C) in the NMR spectrum of 2c (Figure S9), in which the terminal phenyl groups repel the adjacent carbonyl groups.

More extended hydrogen-bond chains are evident in the ^1H NMR spectra of triurea 3a and tetraurea 4a, where the terminal benzylic methylenes occupy only two identically populated environments (Figure 2B). The only simple explanation for this spectroscopic feature is an unbroken chain of hydrogen bonds that correlates the conformations of the two terminal urea functions and allows a population of only

two isoenergetic (and degenerate) structures. The chemical shifts of the urea NH protons in CD_2Cl_2 further support this interpretation: at slow exchange (−60 °C) **3a** has one upfield (non-hydrogen-bonded) signal at 6.65 ppm and two downfield (hydrogen-bonded) NH signals at 9.28 ppm (Figure S10). At fast exchange, the central NH proton remains close to 9 ppm (it is always hydrogen-bonded), whereas the terminal NH groups coalesce to an exchange-averaged 8.94 ppm. Tetraurea **4a** behaves in a similar way (Figure S18).

The barriers to conformational interconversion in the non-hydrogen-bonding solvent CD_2Cl_2 **2**–**4** are all similar (Figure 2B) and are about 10 kJ mol^{-1} (at 298 K) higher than in **1**, which lacks an intramolecular hydrogen bond, with no evidence of concentration dependence (Figure S10). This suggests that the inversion of directionality in **2**–**4** occurs through a non-concerted mechanism in which only one hydrogen bond is broken at any one time. The 10 kJ mol^{-1} difference in energy between the barrier to rotation of **1** and that of **2**–**4** gives an estimate of the energetic penalty for breaking this hydrogen bond.

The situation changes in the presence of 10% d_6-DMSO or 40% d_3-MeOH (v/v) (Figure 2B): the barrier to rotation of **1** increases, as is typical for amide-like C–N bonds in more polar solvents,[27] but that of **2**–**4** decreases. Presumably, hydrogen bonding to these solvents mitigates the enthalpic cost of breaking an intramolecular hydrogen bond during the directionality switch. Nonetheless, the hydrogen bond train itself proved remarkably resistant to the effect of these polar solvents and additives. Neither CD_3OH nor $(CD_3)_2SO$ (up to 50% v/v) interfered with the appearance of two coherent hydrogen-bonded conformers of **2**–**4** in CD_2Cl_2 (Figures S2, S3, S5, S6, S13–S15, S19, and S20). The only significant change in the NMR spectra was a shift downfield of the terminal, non-intramolecularly hydrogen-bonded proton in the presence of these hydrogen-bonding additives. Likewise, adding 2.5 equivalents of *N*,*N*′-di[3,5-bis(trifluoromethyl)phenyl]thiourea as a potent hydrogen-bond donor[28] resulted in no changes to the intramolecularly hydrogen-bonded chain of **3a** (Figure S16).

Control and switching of hydrogen-bond polarity

Given the coherent hydrogen-bonded chains of **2**–**4**, changing just one terminus will break the constitutional symmetry of the oligomer, and must lead to differential populations of two conformers—both fully hydrogen-bonded, but differing in directionality.[18] Oligomers **3c**–**3h** were made to elucidate the effect of modifying one of the terminal substituents (Figures 3A–3C). With one terminal ethyl group, the conformers are populated in a 60:40 ratio at −60°C in CD_2Cl_2 (**3c**), while with a terminal phenyl group (**3d**), the preference for the aryl substituent to lie *trans* to C=O leads to a single set of signals in the ^{1}H NMR spectrum at all temperatures, with the Ph group at the hydrogen-bond-donating terminus.[19–22] Modifying the pendent nitrogen substituents also affects the conformer populations (Figure 3B). Oligomer **3e**, in which one terminal nitrogen carries an alkyl group, preferentially (85:15) adopts a conformation in which this (less acidic) NH proton does not participate in an intramolecular hydrogen bond, while in 10% $(CD_3)_2SO$ (v/v) the preference inverts to 30:70, allowing the (more acidic) ArNH proton to participate in a stronger intermolecular hydrogen bond. In **3f**, a weakly hydrogen-bond-accepting succinimide provides an anchor for an adjacent hydrogen-bond-donating urea, favoring (in a ratio of 72:28) the conformer containing three hydrogen bonds.

With the much stronger hydrogen-bond-accepting pyridyl group[29] of **3g** (Figure 3C) only a single directionality is shown by variable temperature (VT) NMR (Figure S26).

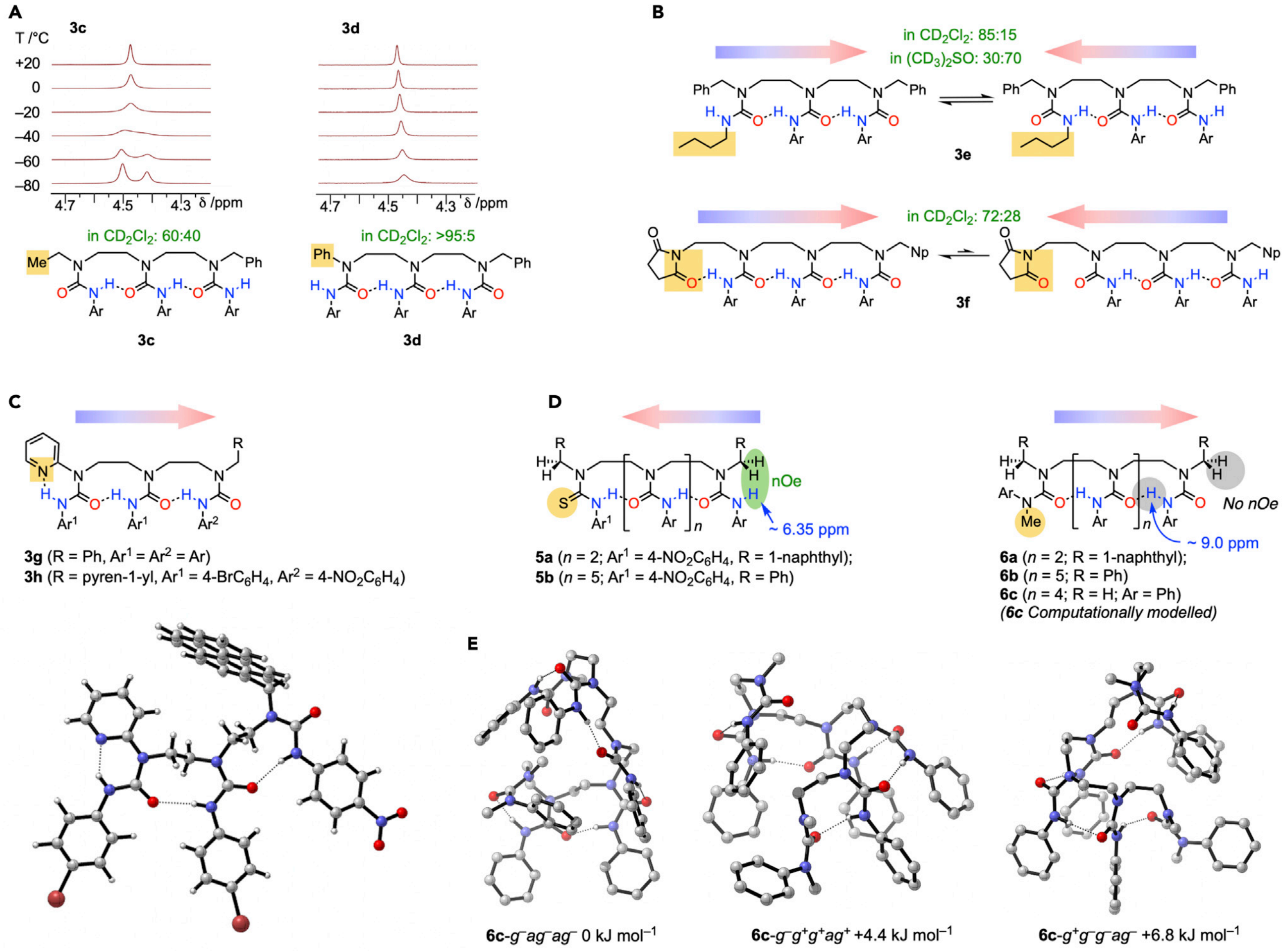

Figure 3. Modifications of terminal substituents induce a conformational response

(A) Structures, dynamic NMR spectra, and ratios of conformers differing in hydrogen-bond directionality for constitutionally unsymmetrical triureas **3c** and **3d**. NMR spectra acquired at 300 MHz in solution in CD_2Cl_2 at a concentration of 10 mg mL^{-1}.

(B) Conformer populations in constitutionally unsymmetrical triureas **3e** and **3f**. The corresponding variable temperature NMR spectra are shown in Figures S21–S23.

(C) X-ray crystal structure of oligomer **3h** showing three intramolecular hydrogen bonds (CCDC: 1993164).

(D) Global conformational control from a terminal donor or acceptor in oligomers **5** and **6** containing up to seven urea units: the chemical shift of the highlighted proton is affected by structural features many bonds away.

(E) Three lowest energy conformers of **6c** with corresponding backbone conformations (g^+, g^- = gauche, *a*, anti, starting from NH terminus) and their relative energies (B3LYP-D3(BJ)/6–311G(d,p)/IEF-PCM(dichloromethane)//B3LYP-D3(BJ)/6-31G(d,p)).

Crystallographic evidence for the conformation of the hydrogen-bonded chain was obtained with the more crystalline derivative **3h** (Figure 3C), whose X-ray crystal structure shows that the oligomer adopts a structure with the pyridyl ring at the hydrogen-bond-donating terminus. As noted previously by Nowick, two nine-membered hydrogen-bonded rings link the urea functions, each of which adopts an anti conformation about the N–C–C–N bond in the oligourea backbone.[21]

The propagation of a conformational consequence from one terminus offers the prospect of using these simple urea chains as molecular communication channels. Compounds **5** and **6** (Figure 3D) were made to explore the way in which the information about structural details at one terminus can be communicated to a remote site. The *p*-nitrophenylthiourea terminus of **5a** and **5b** is a strong hydrogen-bond

donor,[30–32] enforcing a preferred orientation on the neighboring urea, which propagates through the full length of the oligomer. The induced conformation of the most distant urea in the chain is evident in the chemical shift of its NH proton (ca. 6.35 ppm in CD_2Cl_2, Figures S27 and S28), characteristic of a non-intramolecularly hydrogen-bonded environment, and in the reciprocal nOe between this proton and the adjacent CH_2 group (Figures S38–S40), even though this proton is 14 bonds away from the S atom in **5a** and 26 bonds away in **5b**. Likewise, the alkylated urea of **6a** and **6b**, which can act only as a hydrogen-bond acceptor, induces a remote conformational preference in the orientation of the terminal urea, shifting its proton downfield to ca. 9.00 ppm in CD_2Cl_2 (Figures S29 and S30, an intramolecularly hydrogen-bonded environment) and removing any nOe with the adjacent CH_2 group (Figures S41 and S42). This proton is 15 bonds away from the controlling *N*-methyl group in **6a** and 27 bonds away in **6b**.

Unlike previous molecular communication mechanisms, defined three-dimensional conformational states are irrelevant to the function of these oligomers as communication channels. Nonetheless, the conformation of truncated analog **6c** was modeled computationally by DFT[33,34] (Gaussian16 Revision A.03,[35] B3LYP-D3(BJ)/6–311G(d,p)/IEF-PCM(dichloromethane)//B3LYP-D3(BJ)/6-31G(d,p): see computational details in the supplemental information) to gain insight into their conformational preferences. All conformations placed the tetrasubstituted urea at the hydrogen-bond-donating terminus, but a considerable degree of conformational inhomogeneity was evident (Figure 3E).[36] Minima were found for each nine-membered hydrogen-bonded ring in which the N–C–C–N bond in the oligourea backbone adopted either of two alternative conformations: anti or gauche. The gauche conformation was found to be slightly more prevalent among the conformers of **6c**. Indeed, despite both the X-ray crystal structure of **3h** and previous observations by Nowick,[20] modeling of a simple system with two *N*′-arylureas revealed the gauche conformation to be 2.1 kJ mol^{-1} lower in energy than the anti-conformation (Figure S202). The various permutations of these alternatives for each ring led to an ensemble of conformers, all of them nonetheless characterized by the same hydrogen-bond directionality. Figure 3E shows the three lowest energy conformers of **6c** and the corresponding pattern of gauche and anti conformations along the backbone. Similar features were evident in the modeled conformers of a truncated analog of **4a** (Figure S203). Ethylene-bridged oligoureas thus seem to form an intriguing new class of information-encoding dynamic molecular structure. They populate conformational space much more broadly than the classical definition of a foldamer would allow but are still characterized by one crucial conformational parameter—hydrogen-bond directionality—that is well controlled. This simultaneous conformational mobility and informational coherence raises the prospect of diverse applications for these oligomers, as they can adopt a range of shapes while still robustly maintaining the fidelity with which they transmit information. For example, their flexible conformation is indicative of a broader and more accommodating solubility profile than more rigid structures.

Conformational populations that are modulated by environmental signals are characteristic of a number of classes of biomolecules, such as allosteric enzymes, G-protein-coupled receptors, hemoglobin, and the opsin vision proteins.[37–39] These molecules are characterized not only by inducible conformational change but also by their ability to translate a local chemical influence into a conformational response that has a spatially remote chemical consequence—in Shannon's terminology, they use conformational switching on a molecular level as a channel to communicate information from a transmitter to a receiver. Attempts to mimic this

CellPress
OPEN ACCESS

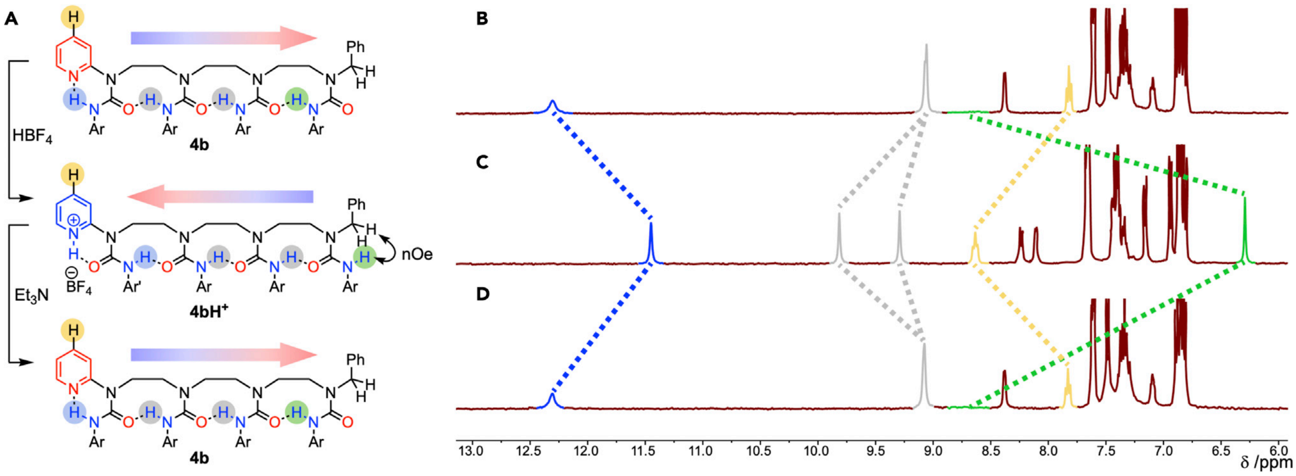

Figure 4. Protonation induces polarity inversion

(A) NMR indicates polarity inversions mediated by reversible protonation of the terminal pyridyl group of **4b**.

(B) ^{1}H NMR spectrum of **4b** in CD_2Cl_2 at 3 mM (298 K).

(C) Addition of HBF_4 (1 equiv) at 298 K. A downfield shift of the yellow 4-pyridyl proton indicates protonation, which leads to a remote upfield shift of the remote (green) proton as it moves out of hydrogen bonding, and of the adjacent (blue) proton as it reorientates toward a weaker hydrogen-bond acceptor.

(D) Recovery of the original conformation on further addition of Et_3N (2 equiv) to the same sample (298 K). Ar, *p*-$MeOC_6H_4$; Np, 1-naphthyl.

relay of information using artificial structures have used screw-sense switching of rigid helices,[11,40] but this family of switchable, conformationally dynamic oligomers opens the possibility of using polarity switching—with chemical consequences that reach beyond modulation of stereochemistry—as a form of communication channel.

Pyridine-terminated urea **4b** (Figure 4A) was made in order to test the responsiveness of the oligourea oligomer to a pH signal. As with its shorter homolog **3g**, VT NMR showed that the hydrogen-bond chain in **4b** adopts a preferred directionality in CD_2Cl_2 (Figure S31), with the diagnostic signal at 12.31 ppm (Figure 4B; the urea NH labeled in blue, hydrogen-bonded to the pyridine N) indicating that the pyridine lies at the oligomer's hydrogen-bond-donating terminus. Protonation of the pyridyl group (shown by a downfield shift in its C4 proton, colored yellow) by the addition of tetrafluoroboric acid (Figure 4C) shifted this blue-coded proton upfield to 11.45 ppm, consistent with a conformational reorganization in which the terminal urea NH finds itself hydrogen bonded to another urea.[41] The two central urea protons (at 9.06 and 9.07 ppm in **4b**) remain hydrogen-bonded to neighboring urea carbonyls after directionality reversal, but their signals shifted downfield to 9.29 and 9.81 ppm, consistent with the inductive effect of the pyridinium ion. At the same time, the terminal urea NH at ca. 8.8 ppm (a broad resonance more clearly resolved at 15 °C; Figure S32) moved out of hydrogen bonding and consequently shifted upfield to 6.29 ppm. This, along with an nOe to the benzylic methylene group (Figures S43 and S44), indicates a global reversal of urea directionality, with the urea oligomer acting as a communication channel, mediating the transmission of a pH signal. VT NMR of $\mathbf{4bH^{+}}$ (Figure S33) shows that a single conformer is populated in which the pyridinium ion acts as a hydrogen-bond donor, inverting the hydrogen-bond directionality so that it lies at the hydrogen-bond-accepting terminus of the oligomer. The addition of triethylamine (Figure 4D) returned the oligomer to its neutral conformation and restored the peaks to their original positions.

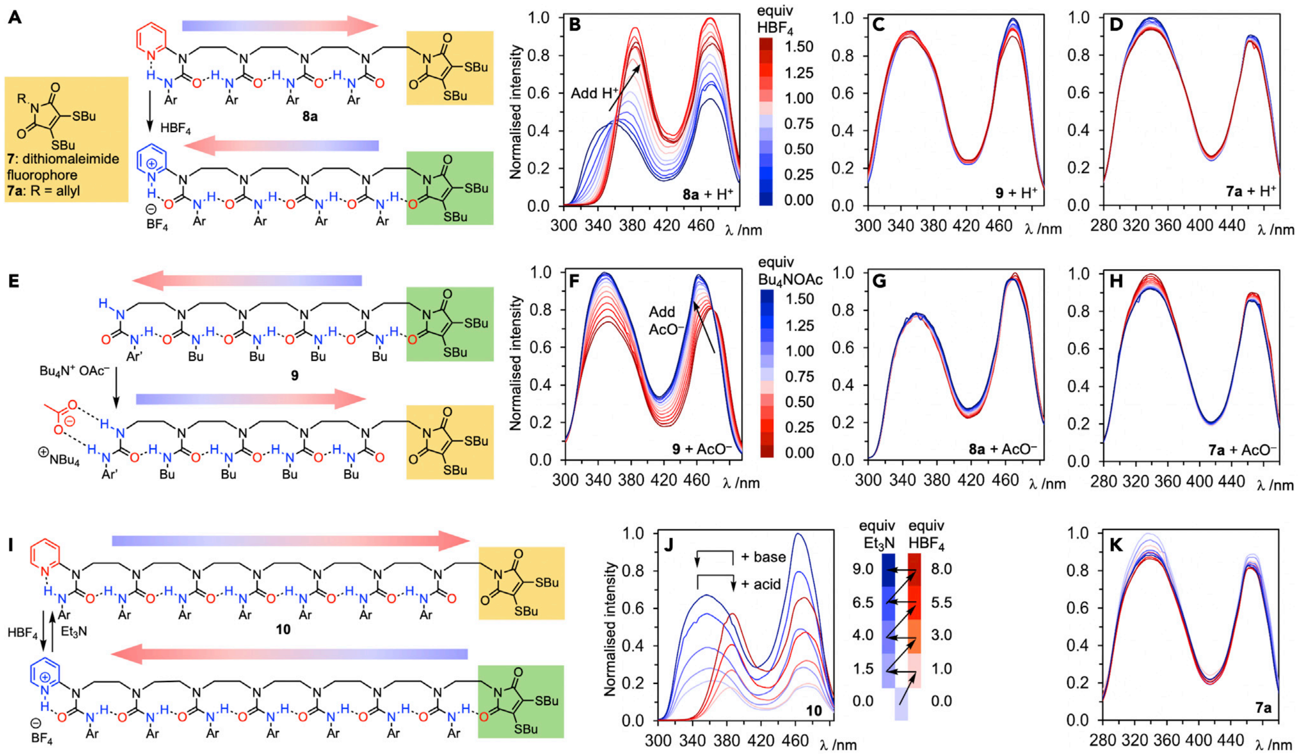

Figure 5. Oligoureas communicate information from a transmitter to a receiver by global switching of polarity

(A) The hydrogen-bond-responsive dithiomaleimide fluorophore **7** incorporated into a switchable oligomer **8a**.

(B–D) Effect of HBF_4 (0–1.5 equiv), in CH_2Cl_2 solution, on the excitation spectrum (at 1 mM) of (B) **8a** (emission at 523 nm), (C) **9** (emission at 530 nm), and (D) **7a** (emission at 522 nm).

(E) Anion-responsive oligomer **9** and its polarity switching on binding acetate.

(F–H) Effect of $Bu_4N.OAc$ (0–1.5 equiv), in CH_2Cl_2 solution, on the excitation spectrum (at 1 mM) of (F) **9** (emission at 530 nm), (G) **8a** (emission at 523 nm), and (H) **7a** (emission at 522 nm).

(I and J) (I) Reversible switching of the fluorophore-capped heptaurea **10** and its excitation spectra in CH_2Cl_2 at 1 mM (emission at 525 nm) on sequential addition of (J) HBF_4 (aliquots of 1, 2, 2.5, 2.5 equiv) and Et_3N (aliquots of 0, 1.5, 2.5, 2.5, 2.5 equiv).

(K) The effect on its excitation spectrum (emission at 522 nm) of treating **7a** in CH_2Cl_2 at 1 mM with an equivalent sequence of aliquots. Ar = *p*-$MeOC_6H_4$; Ar′ = $[3,5\text{-}(CF_3)_2]C_6H_3$.

Reversible hydrogen-bond chains as channels for the long-range communication of information

As a demonstration of the wider potential of signal transmission mediated by hydrogen-bond directionality switching, we designed a molecular device in which information about the presence of an added cation or anion is detected and transmitted through an oligourea communication channel to a remote receiver that is sensitive to local bond polarity. The dithiomaleimide fluorophore **7** (Figure 5A) is a weakly hydrogen-bond-accepting structure that is sensitive to its environment,[42–44] and we hoped that it would respond to a local switch between a hydrogen-bond-donating and hydrogen-bond-accepting function. In contrast to typical receptors, such a device allows the functional and spatial decoupling of detector and response, raising the prospect of building modular devices for use in spatially compartmentalized systems such as on surfaces or in artificial cells.

Oligomers **8–10** (Figures 5A, 5E, and 5I) were designed and synthesized: all are terminated with a dithiomaleimide fluorophore, but each carries a responsive function remote from the fluorophore that can transmit chemical information as a conformational response. Oligomers **8a** and **10**, similar to **4b**, are acid-sensitive: we expect

protonation of the pyridine function to lead to a change in conformation that will alter the environment of the remote fluorophore. Oligomer **9** carries an electron-deficient terminal *N*,*N*′-disubstituted urea that we expect to display binding selectivity toward anions.[34,45] NMR experiments (Figures S34, S35, S46, and S52) indicate that these compounds adopt a preferred hydrogen-bond directionality shown in the upper parts of Figures 5A, 5E, and 5I.

The response of the fluorescence excitation spectrum (emission at 523 nm) of **8a** in CH_2Cl_2 was monitored as the oligomer was titrated with tetrafluoroboric acid (Figure 5B). As acid was added, the S_0-S_2 excitation wavelength maximum λ_{max} at 358 nm gradually red-shifted to 381 nm after 1 equiv, with a concomitant global hyperchromic shift. Minimal spectral changes were observed beyond 1 equiv of acid (λ_{max} S_0-S_2 with 1.5 equiv HBF_4 = 384 nm). A similar red shift in the S_0-S_2 excitation band ($\Delta\lambda_{max}$ S_0-S_2 = 21 nm) was seen for the longer homolog **10** on adding 1 equiv HBF_4 (Figure S47). Under the same conditions, neither **9** nor **7a** underwent any significant changes in fluorescence excitation (Figures 5C and 5D). Additionally, a fluorescent control 8b with a tethered pyridyl group separated from the maleimide by the same number of bonds as in **8a** but without a linking chain of urea hydrogen bonds showed no λ_{max} shifts upon addition of HBF_4 (Figure S48). This confirms that protonation of the pyridine leads to fluorescence changes only when a mechanism for intramolecular communication is available and provides evidence that the dithiomaleimide can be used as a probe of the local hydrogen-bonding environment, and hence a receiver for use with the oligourea communication channel. The response of **8a** is consistent with protonation of the pyridine function leading to a change in conformation (Figure 5A) that transmits information about its ionization state to the dithiomaleimide. As a result, the dithiomaleimide receiver is induced to act as a hydrogen-bond acceptor (indicated by the chemical shift of the adjacent NH: Figure S51), its fluorescence maximum consequently undergoing a red shift.

Next, the fluorescence excitation spectrum of **9** (emission at 530 nm) was monitored as tetrabutylammonium acetate was added (Figure 5E). The chain of hydrogen bonds in **9** involves alkyl ureas, which are weaker hydrogen-bond donors and stronger hydrogen-bond acceptors than aryl ureas (cf. Figure 3B). As tetrabutylammonium acetate was added, the S_0-S_1 excitation maximum at 478 nm gradually blue-shifted to 464 nm after 1 equiv (Figure 5F), consistent with the fluorophore receiver moving from a hydrogen-bonded to a non-hydrogen-bonded environment. Minimal spectral changes were observed beyond 1 equiv of acetate (λ_{max} S_0-S_1 with 1.5 equiv Bu_4NOAc = 462 nm). The same ligand gave minimal response in **8a** or **7a** (Figures 5G and 5H). Oligomer **9** thus acts as an acetate-responsive device in which binding information is transmitted to a remote receiver, which responds with a modulation of its fluorescence properties.

The potential for reversible pH-controlled switching between the two polarity states was explored with both **8a** (Figure S55) and with its longer homolog **10** (Figure 5I). Starting from the neutral oligomer **10**, repeated sequential addition of acid (HBF_4) and base (Et_3N) successfully induced four cycles of the oscillation of the fluorophore excitation response between the blue-shifted, non-hydrogen-bonded "neutral" state (λ_{max} S_0-S_2 = 359 $\pm$ 4 nm) and the red-shifted, hydrogen-bonded "protonated" state (λ_{max} S_0-S_2 = 386 $\pm$ 2 nm) (Figure 5J). Under the same conditions, no significant changes in the excitation wavelength maxima were observed with the fluorophore (**7a**) alone (Figure 5K), showing that this oscillation in fluorescence is a consequence of the communication of information through a channel that entails

inversion of the polarity of a single intramolecular chain of seven hydrogen bonds, which causes concerted rotation about seven consecutive urea C–N bonds.

Conclusions

In summary, the structurally simple and synthetically accessible class of ethylenediamine oligomers offers a mechanism for the spatial communication of chemical information. Despite their three-dimensional conformational fluxionality, ethylene-bridged oligureas exhibit uniformity in one global conformational feature: the directionality of their single linear chain of hydrogen bonds. This hydrogen-bond chain acts as a communication channel that can carry information between spatially remote sites encoded in its polarity, and we demonstrate that it enables a signal (the presence of acid or base, or the binding of ligands) to induce a relayed response, such as a change in optical properties, that is reversible and repeatable through multiple communication cycles. A useful macromolecular analogy is a hydraulic communication channel in which a change in pressure is used to communicate information (for example, from a brake pedal to a brake pad) through a conformationally flexible tube.

The lack of a requirement for chirality in this mechanism offers several advantages over earlier examples of artificial communication devices based on helical foldamers. Their "minimal" design means that their synthesis is much simpler and their potential for functionalization is much greater than previous structures. Inputs and outputs of information are no longer stereochemically encoded, which frees potential future interfaces with biology from the complications that result from the use of single enantiomers in the enantiopure environment of a biological system. Even more importantly, the receiver of information is no longer constrained to translate an enantiomeric switch into a more general chemical response. A switch in hydrogen-bond polarity offers much greater chemical versatility in, for example, the potential to control the selective binding of ligands or metals, to alter metal coordination geometry, or to change the structure and activity at a catalytic site.

This spatial communication mechanism, which requires the use of constitutionally symmetrical dynamic structures, has hitherto remained unexploited in synthetic molecular devices. Localized hydrogen-bond directionality switching is nonetheless evident in nature, for example, during the catalytic cycle of proteases and lipases, where coupled active site histidine and aspartate residues switch from hydrogen-bond acceptors to hydrogen-bond donors during the hydrolysis mechanism. Nature is prevented from exploiting hydrogen-bond directionality switching as a general mechanism for spatial communication by the constitutional asymmetry of its α-amino acid building blocks. However, it is intriguing to note that although nature does not communicate information spatially through hydrogen-bonded chains, it does so temporally during the transcription of the genetic code, in which a series of sequential hydrogen-bond polarity matching events mediate the communication of coded information from DNA to mRNA to tRNA. Further exploration of the potential of hydrogen-bond chains as communication channels in more complex supramolecular networks and in non-homogeneous states is under way.

EXPERIMENTAL PROCEDURES

Resource availability

Lead contact

Further information and requests for resources should be directed to and will be fulfilled by the lead contact, Jonathan Clayden (j.clayden@bristol.ac.uk).

Materials availability

All materials generated in this study are available from the lead contact without restriction.

Data and code availability

Crystallographic data for the structure reported in this article have been deposited at the Cambridge Crystallographic Data Centre, under deposition number CCDC: 1993164. Copies of the data can be obtained free of charge from https://www.ccdc.cam.ac.uk/structures/.

SUPPLEMENTAL INFORMATION

Supplemental information can be found online at https://doi.org/10.1016/j.chempr.2021.06.022.

ACKNOWLEDGMENTS

We acknowledge the support of the EPSRC through the Bristol Chemical Synthesis Centre for Doctoral Training (studentship to D.T.J.M.), Programme grant EP/P027067/1 (*Molecular Robotics*), and DTP funding (studentship to E.H.E.F.); the ERC through advanced grant 883786 (DOGMATRON); the Europen Commission through a Marie Sklodowska Curie fellowship to D.T.; the Leverhulme Trust; and Dr Hazel Sparkes for X-ray crystallographic analysis. This research made use of the Balena High Performance Computing (HPC) Service at the University of Bath.

AUTHOR CONTRIBUTIONS

J.C., J.W.W., and S.M.W. devised the project. D.T.J.M., S.M.W., D.P.T., and J.W.W. designed and synthesized the molecular structures, carried out the experimental work, and analyzed the data. J.W.W. contributed to the supervision of the project. E.H.E.F. and M.N.G. carried out the computational work. D.T.J.M., S.M.W., E.H.E.F., M.N.G., and J.C. wrote the manuscript.

DECLARATION OF INTERESTS

The authors declare no competing interests.

Received: March 26, 2021
Revised: May 11, 2021
Accepted: June 22, 2021
Published: July 28, 2021

REFERENCES

1. Shannon, C.E. (1948). A mathematical theory of communication. Bell Syst. Tech. J. *27*, 623–656.
2. Uda, S., Saito, T.H., Kudo, T., Kokaji, T., Tsuchiya, T., Kubota, H., Komori, Y., Ozaki, Y., and Kuroda, S. (2013). Robustness and compensation of information transmission of signaling pathways. Science *341*, 558–561.
3. Keshelava, A., Solis, G.P., Hersch, M., Koval, A., Kryuchkov, M., Bergmann, S., and Katanaev, V.L. (2018). High capacity in G protein-coupled receptor signaling. Nat. Commun. *9*, 876.
4. Lehn, J.M. (2007). From supramolecular chemistry towards constitutional dynamic chemistry and adaptive chemistry. Chem. Soc. Rev. *36*, 151–160.
5. Meng, W., Clegg, J.K., Thoburn, J.D., and Nitschke, J.R. (2011). Controlling the transmission of stereochemical information through space in terphenyl-edged Fe4L6 cages. J. Am. Chem. Soc. *133*, 13652–13660.
6. Salles, A.G., Zarra, S., Turner, R.M., and Nitschke, J.R. (2013). A self-organizing chemical assembly line. J. Am. Chem. Soc. *135*, 19143–19146.
7. Brioche, J., Pike, S.J., Tshepelevitsh, S., Leito, I., Morris, G.A., Webb, S.J., and Clayden, J. (2015). Conformational switching of a foldamer in a multicomponent system by pH-filtered selection between competing noncovalent interactions. J. Am. Chem. Soc. *137*, 6680–6691.
8. Le Bailly, B.A.F.F., and Clayden, J. (2016). Dynamic foldamer chemistry. Chem. Commun. (Camb) *52*, 4852–4863.
9. Mazzier, D., Crisma, M., De Poli, M., Marafon, G., Peggion, C., Clayden, J., and Moretto, A. (2016). Helical foldamers incorporating photoswitchable residues for light-mediated modulation of conformational preference. J. Am. Chem. Soc. *138*, 8007–8018.
10. Le Bailly, B.A.F., Byrne, L., and Clayden, J. (2016). Refoldable foldamers: global conformational switching by deletion or insertion of a single hydrogen bond. Angew. Chem. Int. Ed. Engl. *55*, 2132–2136.
11. Lister, F.G.A.A., Le Bailly, B.A.F.F., Webb, S.J., and Clayden, J. (2017). Ligand-modulated

conformational switching in a fully synthetic membrane-bound receptor. Nature Chem *9*, 420–425.

12. Araki, T., Nogami, F., Tsukube, H., Nagata, K., and Iyoshi, S. (1978). Nonclassical urea oligomers. I. Selective reactivity of 1-(N-substituted-carbamyl)aziridine for ring-transformation reactions and ring-opening polymerization. J. Polym. Sci. Polym. Chem. Ed. *16*, 1037–1057.

13. Araki, T., Kubo, Y., and Yasuda, Y. (1989). Presence of intramolecular hydrogen bonds in Hexakis(N-phenylcarbamoyl) pentaethylenehexamine. Chem. Express *4*, 605–608.

14. Huc, I., and Hecht, S. (2007). Foldamers: Structure, Properties, and Applications (John Wiley & Sons).

15. Guichard, G., and Huc, I. (2011). Synthetic foldamers. Chem. Commun. (Camb) *47*, 5933–5941.

16. Girvin, Z.C., Andrews, M.K., Liu, X., and Gellman, S.H. (2019). Foldamer-templated catalysis of macrocycle formation. Science *366*, 1528–1531.

17. Wechsel, R., Raftery, J., Cavagnat, D., Guichard, G., and Clayden, J. (2016). The meso helix: symmetry and symmetry-breaking in dynamic oligourea foldamers with reversible hydrogen-bond polarity. Angew. Chem. Int. Ed. Engl. *55*, 9657–9661.

18. Wechsel, R., Žabka, M., Ward, J.W., and Clayden, J. (2018). Competing hydrogen-bond polarities in a dynamic oligourea foldamer: a molecular spring torsion balance. J. Am. Chem. Soc. *140*, 3528–3531.

19. Nowick, J.S., Powell, N.A., Martinez, E.J., Smith, E.M., and Noronha, G. (1992). Molecular scaffolds. I. Intramolecular hydrogen bonding in a family of di- and triureas. J. Org. Chem. *57*, 3763–3765.

20. Nowick, J.S., Abdi, M., Bellamo, K.A., Love, J.A., Martinez, E.J., Noronha, G., Smith, E.M., and Ziller, J.W. (1995). Molecular scaffolds. 2. Intramolecular hydrogen bonding in 1,2-diaminoethane Diureas. J. Am. Chem. Soc. *117*, 89–99.

21. Nowick, J.S., Mahrus, S., Smith, E.M., and Ziller, J.W. (1996). Triurea Derivatives of diethylenetriamine as potential templates for the formation of artificial β-sheets 1. J. Am. Chem. Soc. *118*, 1066–1072.

22. Nowick, J.S. (2006). What I have learned by using chemical model systems to study biomolecular structure and interactions. Org. Biomol. Chem. *4*, 3869–3885.

23. Yamaguchi, K., Matsumura, G., Kagechika, H., Azumaya, I., Ito, Y., Itai, A., and Shudo, K. (1991). Aromatic architecture. Use of the N-methylamide structure as a molecular splint. J. Am. Chem. Soc. *113*, 5474–5475.

24. Itai, A., Toriumi, Y., Saito, S., Kagechika, H., and Shudo, K. (1992). Preference for cis-amide structure in N-acyl-N-methylanilines. J. Am. Chem. Soc. *114*, 10649–10650.

25. Tanatani, A., Kagechika, H., Azumaya, I., Fukutomi, R., Ito, Y., Yamaguchi, K., and Shudo, K. (1997). Helical aromatic urea and guanidine. Tetrahedron Lett *38*, 4425–4428.

26. Clayden, J., Lemiègre, L., Pickworth, M., and Jones, L. (2008). Conformation and stereodynamics of 2,2′-disubstituted N,N′-diaryl ureas. Org. Biomol. Chem. *6*, 2908–2913.

27. Cox, C., and Lectka, T. (1998). Solvent effects on the barrier to rotation in carbamates. J. Org. Chem. *63*, 2426–2427.

28. Hunter, C.A. (2004). Quantifying intermolecular interactions: guidelines for the molecular recognition toolbox. Angew. Chem. Int. Ed. Engl. *43*, 5310–5324.

29. Corbin, P.S., Zimmerman, S.C., Thiessen, P.A., Hawryluk, N.A., and Murray, T.J. (2001). Complexation-induced unfolding of heterocyclic ureas. Simple foldamers equilibrate with multiply hydrogen-bonded sheetlike structures. J. Am. Chem. Soc. *123*, 10475–10488.

30. Takemoto, Y. (2005). Recognition and activation by ureas and thioureas: stereoselective reactions using ureas and thioureas as hydrogen-bonding donors. Org. Biomol. Chem. *3*, 4299–4306.

31. Li, A.F., Wang, J.H., Wang, F., and Jiang, Y.B. (2010). Anion complexation and sensing using modified urea and thiourea-based receptors. Chem. Soc. Rev. *39*, 3729–3745.

32. Zhang, Z., and Schreiner, P.R. (2009). (Thio)urea organocatalysis—what can be learnt from anion recognition? Chem. Soc. Rev. *38*, 1187–1198.

33. Grayson, M.N., and Houk, K.N. (2016). Cinchona urea-catalyzed asymmetric sulfa-Michael reactions: the Brønsted acid−hydrogen bonding model. J. Am. Chem. Soc. *138*, 9041–9044.

34. Grayson, M.N. (2017). Mechanism and origins of stereoselectivity in the cinchona thiourea- and Squaramide-catalyzed asymmetric Michael addition of nitroalkanes to enones. J. Org. Chem. *82*, 4396–4401.

35. Frisch, M.J., Trucks, G.W., Schlegel, H.B., Scuseria, G., et al. (2016). Gaussian 16, Revision C.01(Gaussian). https://gaussian.com/.

36. Legault, C.Y. (2009). CYLview, 1.0b (University Sherbrooke). http://www.cylview.org.

37. Wei, G., Xi, W., Nussinov, R., and Ma, B. (2016). Protein ensembles: how does nature harness thermodynamic fluctuations for life? The diverse functional roles of conformational ensembles in the cell. Chem. Rev. *116*, 6516–6551.

38. Nussinov, R. (2016). Introduction to protein ensembles and allostery. Chem. Rev. *116*, 6263–6266.

39. Nussinov, R., Tsai, C.J., and Liu, J. (2014). Principles of allosteric interactions in cell signaling. J. Am. Chem. Soc. *136*, 17692–17701.

40. Brown, R.A., Diemer, V., Webb, S.J., and Clayden, J. (2013). End-to-end conformational communication through a synthetic purinergic receptor by ligand-induced helicity switching. Nat. Chem. *5*, 853–860.

41. Jordan, L.M., Boyle, P.D., Sargent, A.L., and Allen, W.E. (2010). Binding of carboxylic acids by fluorescent pyridyl ureas. J. Org. Chem. *75*, 8450–8456.

42. Robin, M.P., Mabire, A.B., Damborsky, J.C., Thom, E.S., Winzer-Serhan, U.H., Raymond, J.E., and O'Reilly, R.K. (2013). New functional handle for use as a self-reporting contrast and delivery agent in nanomedicine. J. Am. Chem. Soc. *135*, 9518–9524.

43. Mabire, A.B., Robin, M.P., Quan, W.D., Willcock, H., Stavros, V.G., and O'Reilly, R.K. (2015). Aminomaleimide fluorophores: a simple functional group with bright, solvent dependent emission. Chem. Commun. (Camb) *51*, 9733–9736.

44. Robin, M.P., Wilson, P., Mabire, A.B., Kiviaho, J.K., Raymond, J.E., Haddleton, D.M., and O'Reilly, R.K. (2013). Conjugation-induced fluorescent labeling of proteins and polymers using dithiomaleimides. J. Am. Chem. Soc. *135*, 2875–2878.

45. Gale, P.A., and Caltagirone, C. (2015). Anion sensing by small molecules and molecular ensembles. Chem. Soc. Rev. *44*, 4212–4227.

Chem, Volume *7*

Supplemental information

A molecular communication channel consisting of a single reversible chain of hydrogen bonds in a conformationally flexible oligomer

David T.J. Morris, Steven M. Wales, David P. Tilly, Elliot H.E. Farrar, Matthew N. Grayson, John W. Ward, and Jonathan Clayden

Contents

Supplementary Methods 2
General Information 2
Experimental Procedures 10
VT NMR Experiments 60
Eyring Analyses 91
nOe Experiments 101
Fluorimetry 105
Supplemental Experimental Procedures 105
NMR Characterisation 114
Computational Details 187
References 205

Supplementary Methods

General Information

Where specified, procedures were performed using dried solvents and reagents under an atmosphere of nitrogen with glassware dried by flame-drying. Air and moisture-sensitive liquids/solutions were transferred to reaction vessels by syringe under an atmosphere of nitrogen. Anhydrous DCM, THF, MeCN, Et_2O and toluene were dried using an anhydrous Engineering Grubbs-type solvent system. All other solvents and reagents were purchased from commercial suppliers and were used without further purification unless otherwise specified. Agitation was achieved using Teflon coated stirrer bars by magnetic induction. All thin layer chromatography (TLC) experiments were conducted on pre-coated plastic plates (Macherey-Nagel polygram SIL G/UV_{254}) and visualised using ultraviolet light (254 nm) or staining. In all instances where molecular sieves were used, 1.6 mm diameter pellets (4 Å pore size) bought from Sigma-Aldrich were activated by oven-drying. Flash chromatography was performed on an automated Biotage Isolera™ Spektra Four using gradient elution on pre-packed silica gel Biotage® SNAP Ultra, Sfär Duo or ZIP Sphere columns or manually using Fluorochem 60 silica (40-60 μm particle size). Solvent systems for TLC and flash chromatography are reported in solvent:solvent ratios. All melting point experiments were performed using a Stuart SMP10 Melting Point Apparatus upon recrystallisation from the specified solvent(s). All low-temperature VT NMR experiments were conducted using a JEOL ECS 300 spectrometer (300 MHz) or a Bruker AVANCE III HD 500 MHz NMR Spectrometer with 5 mm DCH ^{13}C–^{1}H/D Cryo Probe (500 MHz). All high-temperature VT NMR experiments were conducted using a Varian VNMRS 500 MHz Direct Drive Spectrometer with Agilent OneNMR probe (500 MHz). All room temperature NMR experiments were conducted using a Bruker Nano 400 Spectrometer (400 MHz) or a Bruker AVANCE III HD 500 MHz NMR Spectrometer with 5 mm DCH ^{13}C–^{1}H/D Cryo Probe (500 MHz), with chemical shifts reported (δ in ppm) relative to the specified deuterated solvent. All ^{19}F NMR spectra are referenced relative to an internal standard. All NMR characterisation experiments were performed at 25 °C and 1 atm unless otherwise specified. Multiplicity is reported as follows – s = singlet, d = doublet, t = triplet, q = quartet, m = multiplet. All spin-spin coupling constants (J) are reported in hertz (Hz) to the nearest 0.1 Hz. High resolution mass spectrometry experiments (HR-MS) were performed on a Bruker micrOTOF Spectrometer using electrospray ionisation, positive ion mode or a Bruker Ultraflex using MALDI with only molecular ion ($[M+H]^+$ or $[M+Na]^+$) peaks being reported. Infrared spectroscopy (IR) experiments were performed using a PerkinElmer Spectrum Two FTIR Spectrometer. All absorbances are reported in cm^{-1}. Fluorescence spectra were recorded using a PerkinElmer LS45 fluorimeter.

Synthetic Schemes

Ureas **1** and **2** were made from their corresponding disubstituted amines. Ureas **3** and **4** were synthesised in two steps from their corresponding oligoethylenediamines by reductive amination and reaction with the appropriate isocyanate.

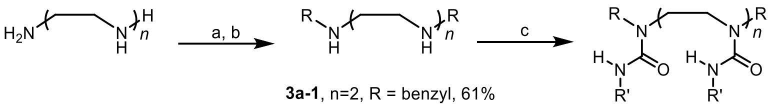

3a-1, n=2, R = benzyl, 61%

1, n=0, R = benzyl, R' = 4-MeO-Ph, >99% (from dibenzylamine)
2a, n=1, R = benzyl, R' = 4-MeO-Ph, 67% (from *N,N'*-dibenzylethylenediamine)
2b, n=1, R = ethyl, R' = 4-MeO-Ph, 82% (from *N,N'*-diethylethylenediamine)
2c, n=1, R = phenyl, R' = Ph, >99% (from *N,N'*-diphenylethylenediamine)
3a, n=2, R = benzyl, R' = 4-MeO-Ph, 54% (from **3a-1**)
3b, n=2, R = benzyl, R' = *n*-butyl, 54% (from **3a-1**)
4a, n=3, R = benzyl, R' = 4-MeO-Ph, 16% (2 steps from triethylenetetramine)

Scheme S1. Synthesis of symmetrical oligoureas **1-4**. Reagents and conditions: (a) benzaldehyde (2.0 equiv), EtOH, RT, 16 h; (b) $NaBH_4$ (3.0 equiv), EtOH, 0 °C to RT, 3 h; (c) R'NCO (1.5 equiv per amine), DCM, RT, 16 h.

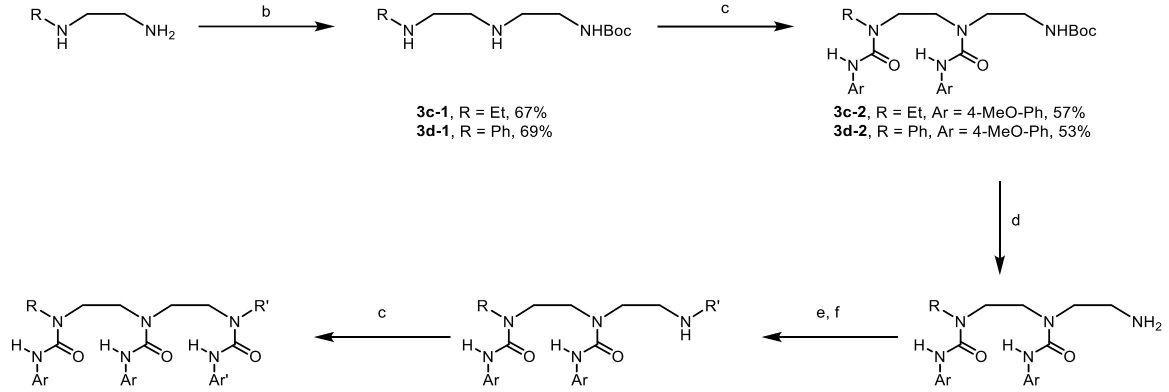

3c-1, R = Et, 67%
3d-1, R = Ph, 69%

3c-2, R = Et, Ar = 4-MeO-Ph, 57%
3d-2, R = Ph, Ar = 4-MeO-Ph, 53%

3c, R = Et, R' = benzyl, Ar = Ar' = 4-MeO-Ph, 73%
3d, R = Ph, R' = benzyl, Ar = Ar' = 4-MeO-Ph, 50%

3c-4, R = Et, R' = benzyl, Ar = 4-MeO-Ph, 51%
3d-4, R = Ph, R' = benzyl, Ar = 4-MeO-Ph, 80%

3c-3, R = Et, Ar = 4-MeO-Ph, 96%
3d-3, R = Ph, Ar = 4-MeO-Ph, 87%

Scheme S2. Synthesis of mixed triureas **3c** and **3d**. Reagents and conditions: (a) Ethylenediamine (6.4 equiv), neat, 120 °C, 16 h; (b) H_2, Pd/C, *N*-Boc-aminoacetaldehyde, (1.1 equiv), MeOH, RT, 16 h; (c) ArNCO (1.5 equiv per amine), DCM, RT, 16 h; (d) TFA, DCM, RT, 16 h; (e) aldehyde (2.0 equiv), EtOH, RT, 16 h; (f) $NaBH_4$ (3.0 equiv) EtOH, 0 °C to RT, 3 h.

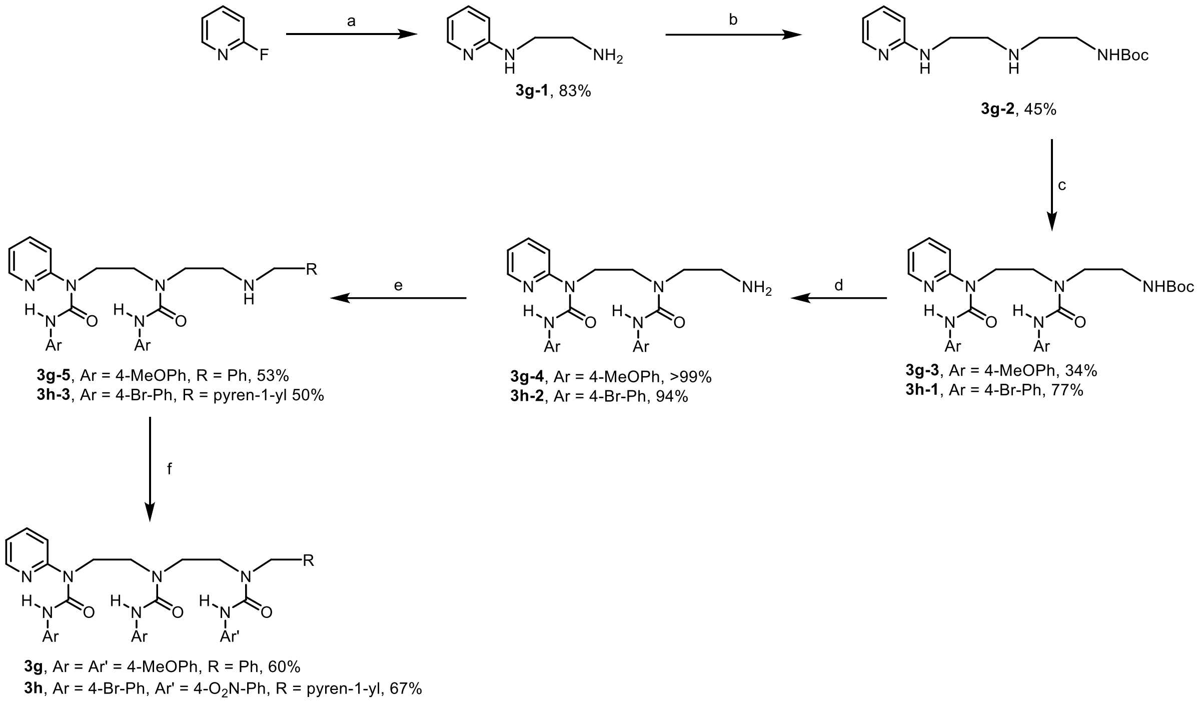

Scheme S3. Synthesis of triureas **3g** and **3h**. Reagents and conditions: (a) Ethylenediamine (10.0 equiv), neat, 120 °C, 18.5 h; (b) *N*-Boc-aminoacetaldehyde, (1.0 equiv), $NaB(OAc)_3H$ (1.4 equiv), DCE, RT, 20 h; (c) ArNCO (1.0 equiv per amine), DCM, RT, 16 h (for **3g-3**) or DCE, RT to 40 °C, 64 h (for **3h-1**); (d) TFA, DCM, RT, 16 h (for **3g-4**) or 4 h (for **3h-2**); (e) benzaldehyde (1.5 equiv), MeOH, RT, 16 h *then* $NaBH_4$, 0 °C to RT, 3 h (for **3g-5**) or 1-pyrenecarboxaldehyde (1.0 equiv), $NaB(OAc)_3H$, DCE, RT, 5 h (for **3h-3**); (f) Ar'NCO (1.0 equiv), DCM, RT, 16 h (for **3g**) or 4.5 h (for **3h**).

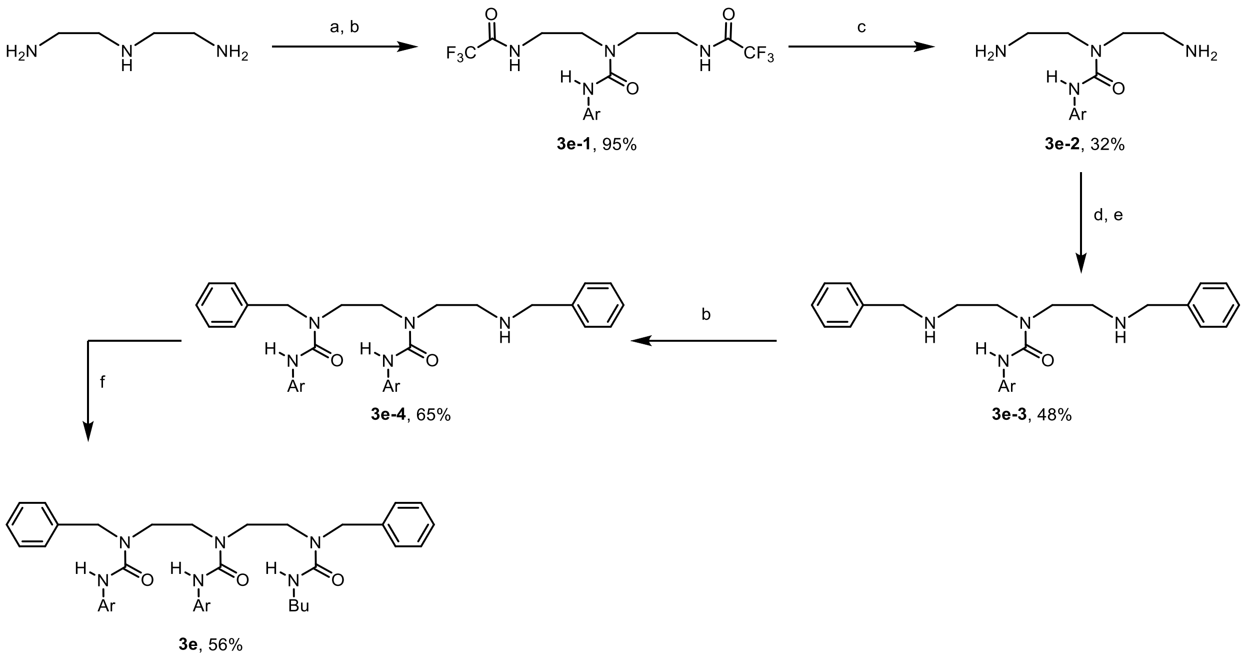

Scheme S4. Synthesis of **3e**. Reagents and conditions: (a) EtOC(O)CF_3 (2.2 equiv), DCM, 0 °C to RT, 2 h; (b) 4-MeO-PhNCO (1.5 equiv), DCM, RT, 16 h; (c) NaOH, MeOH, H_2O, RT, 5 h; (d) benzaldehyde (2.0 equiv), EtOH, RT, 16 h; (e) $NaBH_4$ (3.0 equiv), EtOH, 0 °C to RT, 3 h; (f) ^{n}BuNCO (1.5 equiv), DCM, RT, 16 h. For all compounds, Ar = 4-MeO-Ph.

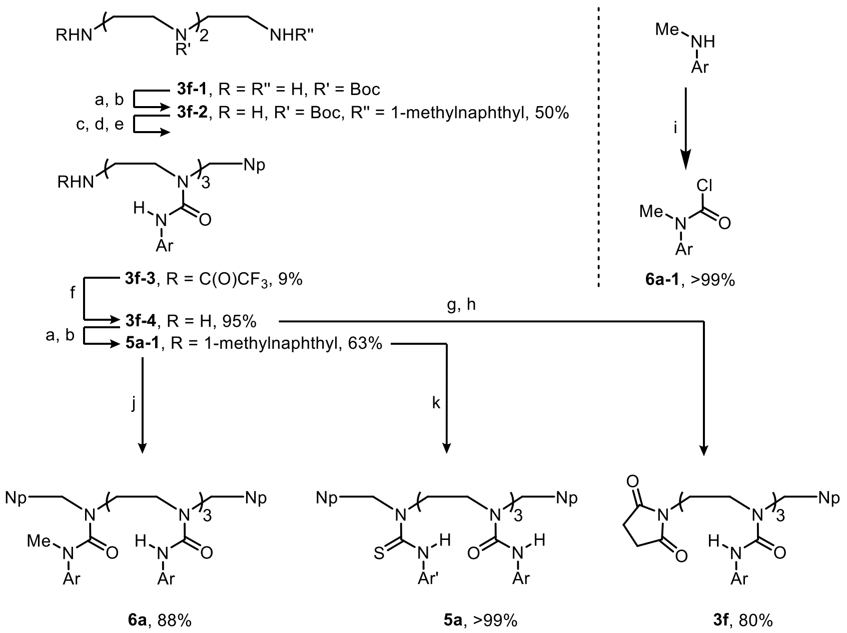

Scheme S5. Synthesis of **3f**, **5a** and **6a**. Reagents and conditions: (a) 1-naphthaldehyde (1.0 equiv), EtOH, RT, 16 h; (b) $NaBH_4$, EtOH, 0 °C to RT, 3 h; (c) Ethyl trifluoroacetate (1.1 equiv per primary amine), DCM, 0 °C to RT, 1 h; (d) HCl/Et_2O, EtOH, RT, 16 h; (e) 4-methoxyphenyl isocyanate (1.5 equiv per amine), TEA (2.0 equiv per amine), DCM, RT, 16 h; (f) NaOH (7.3 equiv), MeOH/H_2O, RT,

5 h; (g) succinic anhydride, DCM, reflux, 16 h; (h) Acetyl chloride, DCM, 0 °C to RT, 3 h; (i) triphosgene (0.5 equiv, DCM, 0 °C to RT, 1 h; (j) *N*-methyl-*N*-(4-methoxyphenyl)carbamoyl chloride (1.5 equiv), TEA (1.5 equiv), DCM, RT, 16 h; (k) 4-nitrophenyl isothiocyanate, DCM, RT, 16 h. For all compounds, Ar = 4-MeO-Ph and Ar' = 4-O_2N-Ph.

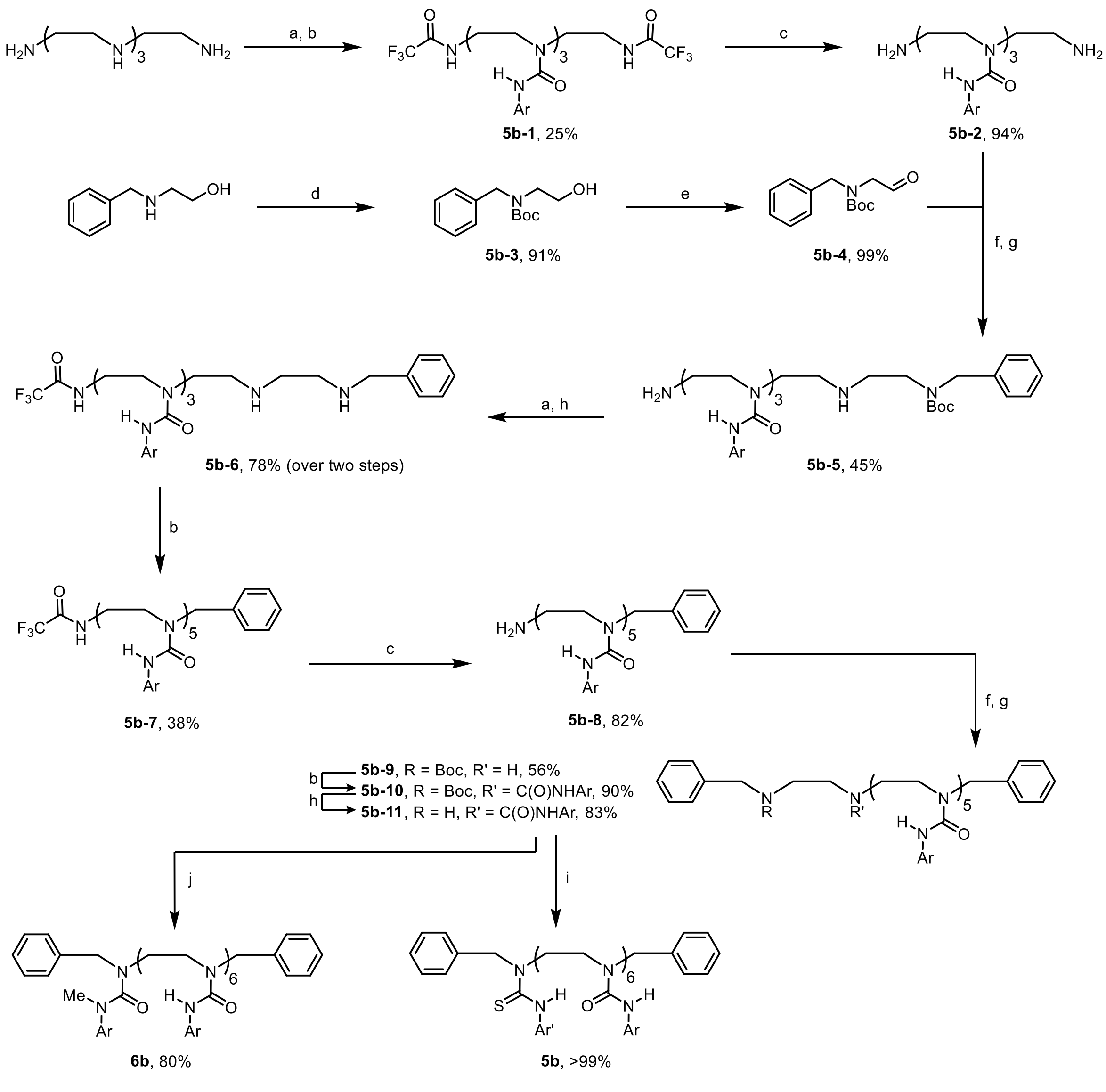

Scheme S6. Synthesis of extended oligoureas **5b** and **6b**. Reagents and conditions: (a) Ethyl trifluoroacetate (1.1 equiv per primary amine), DCM, 0 °C to RT, 1 h; (b) 4-methoxyphenyl isocyanate (1.5 equiv per amine), DCM, RT, 16 h; (c) NaOH (7.3 equiv), MeOH/H_2O, RT, 5 h; (d) Boc_2O (1.0 equiv), aq. NaOH (1 M), DCM, RT, 16 h; (e) oxalyl chloride (1.5 equiv), DMSO (3.0 equiv), TEA (9.0 equiv), DCM, −78 °C to 0 °C, 3 h; (f) **5b-4** (1.0 equiv), EtOH, RT, 16 h; (g) $NaBH_4$, EtOH, 0 °C to RT, 3 h; (h) TFA, DCM, RT, 16 h; (i) 4-nitrophenyl isothiocyanate (1.5 equiv), DCM, RT, 16 h; (j) *N*-methyl-*N*-(4-methoxyphenyl)carbamoyl chloride (1.5 equiv), TEA (1.5 equiv), DCM, RT, 16 h. For all compounds, Ar = 4-MeO-Ph and Ar' = 4-O_2N-Ph.

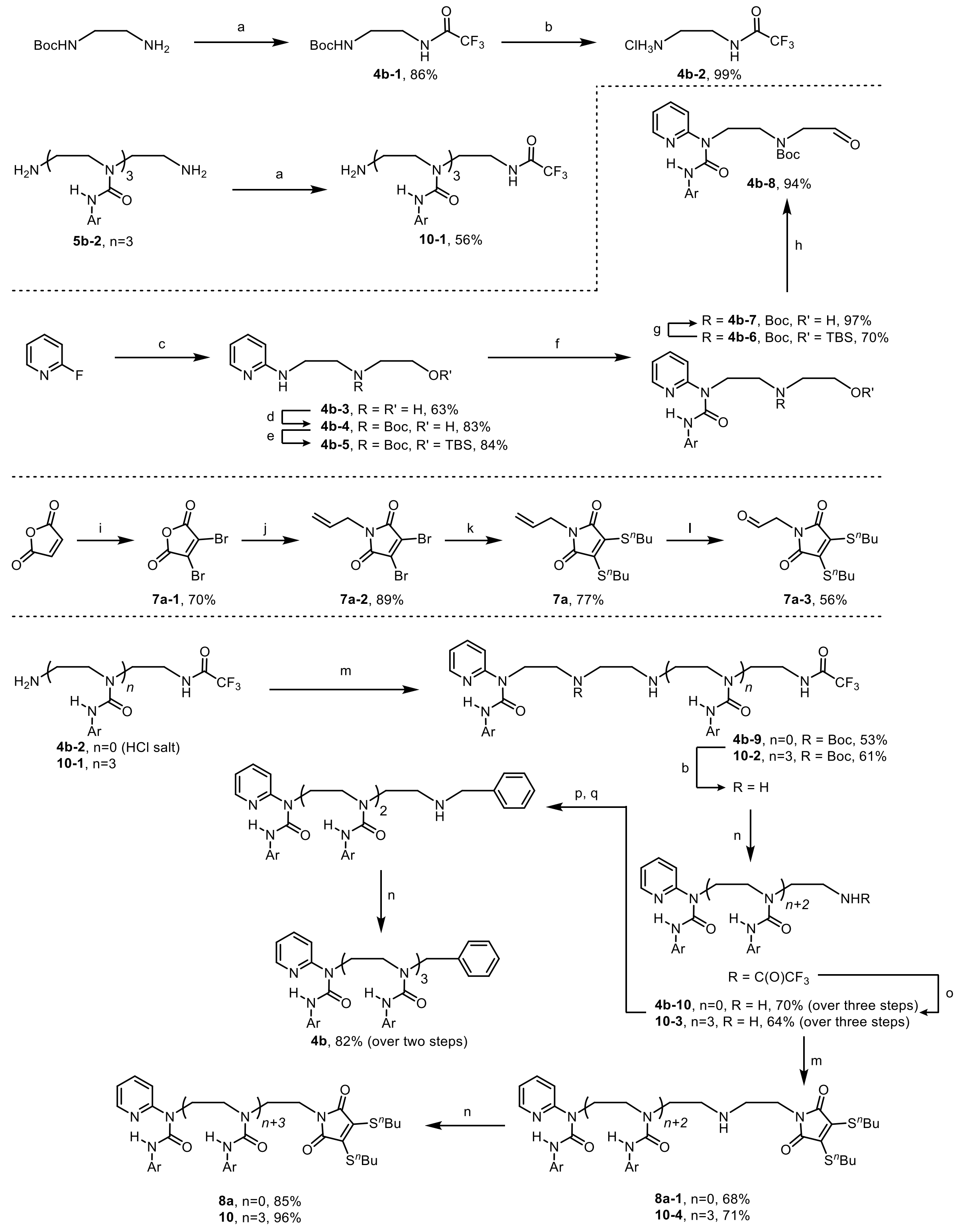

Scheme S7. Synthesis of pyridine-terminated oligoureas **4b**, **8a** and **10** and dithiomaleimide control compound **7a**. Reagents and conditions: (a) Ethyl trifluoroacetate (1.0–1.6 equiv per amine), DCM, 0 °C to RT, 4 h (for **10-1**) or 96 h (for **4b-1**); (b) TFA, DCM, RT, 15–24 h (*then* HCl in MeOH for **4b-2**); (c) *N*-(2-hydroxyethyl)ethylenediamine (3.0 equiv), neat, 120 °C, 17.5 h; (d) Boc_2O (1.0 equiv), THF, 0 °C to RT, 20 h; (e) TBSCl, TEA, DMF, 0 °C to RT, 4 h; (f) 4-methoxyphenyl isocyanate, DCE,

40 °C, 20 h; (g) TBAF, THF, RT, 40 min; (h) DMP, DCM, 0 °C to RT, 6 h; (i) Br_2, $AlCl_3$, neat, 120 °C, 16 h; (j) Allylamine, AcOH, reflux, 5 h; (k) *n*-butanethiol, TEA, Et_2O, RT, 16 h; (l) OsO_4 (10 mol%), $NaIO_4$, 2,6-lutidine, Dioxane/H_2O, RT, 16 h; (m) Aldehyde (1.0 equiv), $NaB(OAc)_3H$, TEA (for **4b-9**), DCE, RT, 2–5 h; (n) 4-methoxyphenyl isocyanate (1.0 equiv per amine), DCM, RT, 30 min–2 h; (o) NaOH (8 equiv), EtOH/THF/H_2O (2:1:1), RT, 16–18 h; (p) Benzaldehyde, MeOH/THF (1:1), RT, 17 h; (q) $NaBH_4$, MeOH/THF (1:1), 0 °C to RT, 3 h. For all compounds, Ar = 4-MeO-Ph.

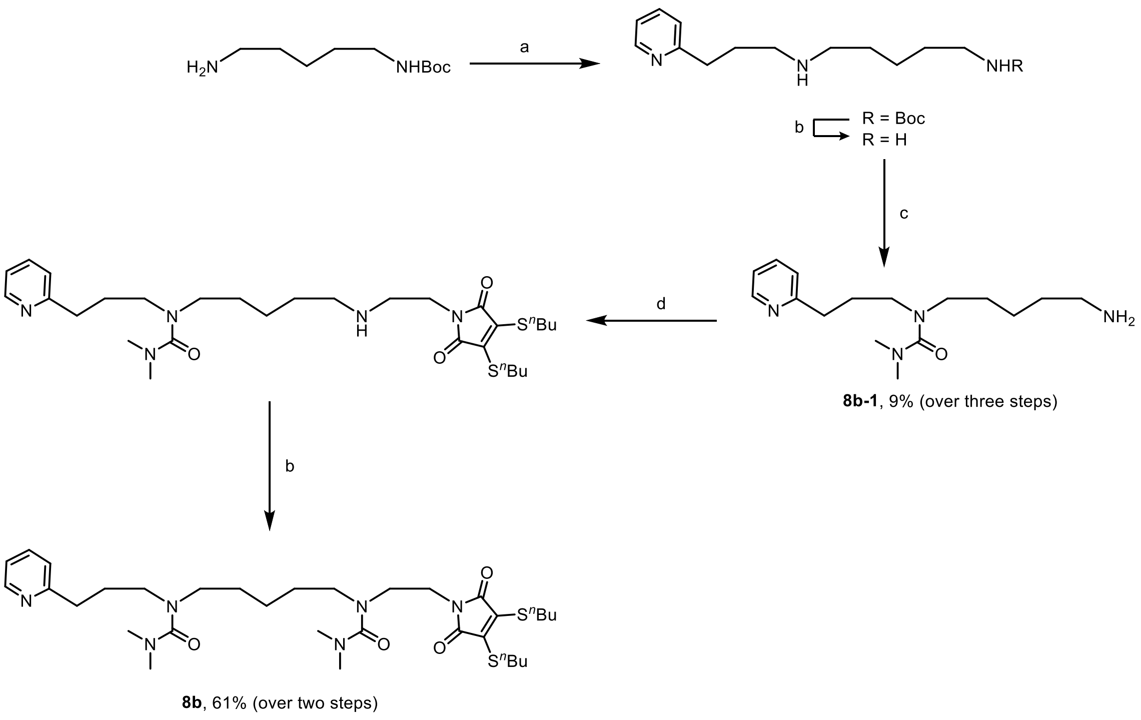

Scheme S8. Synthesis of fluorescent control compound **8b**. Reagents and conditions: (a) 3-(2-pyridyl)propionaldehyde, $NaB(OAc)_3H$, DCE, RT, 2 h; (b) *N*,*N*-dimethylcarbamoyl chloride, TEA, DCE, 45 °C, 16 h; (c) TFA, DCM, RT, 2 h; (d) **7a-3** (1.0 equiv), $NaB(OAc)_3H$, DCE, RT, 2 h.

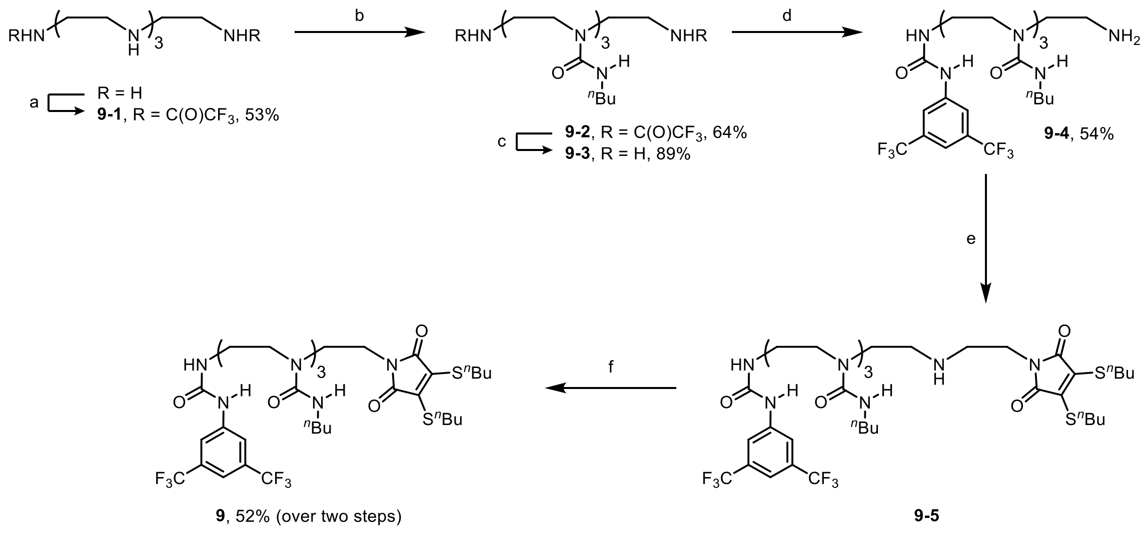

Scheme S9. Synthesis of anion-binding foldamer **9**. Reagents and conditions: (a) Ethyl trifluoroacetate (2.0 equiv), DCM, 0 °C to RT, 24 h; (b) ^{n}BuNCO (3.0 equiv), DCE, 40 °C, 16 h; (c) NaOH (8 equiv), EtOH/THF/H_2O (2:1:1), RT, 18 h; (d) 3,5-bis(trifluoromethyl)phenyl isocyanate (0.7 equiv), DCM, 0 °C to RT, 4 h; (e) **7a-3** (1.0 equiv), NaB(OAc)$_3$H, DCE, RT, 2 h; (f) ^{n}BuNCO (1.0 equiv), DCM, RT, 2 h.

General Procedures

Experimental Procedures

General procedure A – (thio)urea formation from amines and iso(thio)cyanates

Under a dry, inert atmosphere, a solution of secondary amine (1.00 equiv, 0.10 M) in anhydrous DCM was cooled to 0 °C. To this solution was added iso(thio)cyanate (1.50 equiv per amine) dropwise/over three portions. The resulting solution was stirred for 16 h and then concentrated *in vacuo* to give the crude (thio)urea.

General procedure B – reductive amination by $NaBH_4$ reduction

Under a dry, inert atmosphere, to a solution of primary amine (1.00 equiv, 0.10 M) in anhydrous MeOH was added aldehyde (1.50 equiv per amine). The resulting solution was stirred for 16 h. The solution was then cooled to 0 °C and sodium borohydride (3.00 equiv per amine) was added over three portions. The resulting white suspension was warmed to room temperature, stirred for 3 h and concentrated *in vacuo*. The residue was then diluted with MeOH and concentrated *in vacuo*. The crude product was diluted with distilled water and the aqueous solution extracted with DCM three times. The combined organic extracts were washed with brine, dried ($MgSO_4$), filtered and concentrated *in vacuo* to give the crude secondary amine.

General procedure C – trifluoroacetylation and urea formation on oligoethylenediamines

Under a dry, inert atmosphere, a solution of amine (1.00 equiv, 1.6 M) in anhydrous DCM was cooled to 0 °C. Ethyl trifluoroacetate (1.10 equiv per primary amine) was added dropwise over 30 minutes and the resultant solution was stirred for 30 minutes at 0 °C. The solution was then warmed to room temperature and stirred for a further hour. Isocyanate (1.50 equiv per secondary amine) and the resultant solution was stirred for 16 h.

General procedure D – hydrolysis of trifluoroacetamides

To a solution of trifluoroacetamide (1.00 equiv, 0.10 M) was added aqueous sodium hydroxide (15% w/w). The resultant colourless solution was stirred for 5 hours and concentrated *in vacuo*. The crude product was then dissolved in distilled water and washed with DCM three times. The combined organic

extracts were washed with brine, dried ($MgSO_4$), filtered and concentrated *in vacuo* to give the crude primary amine.

General procedure E – succinimidation of amines by *in situ* activation with acetyl chloride

Under a dry inert, atmosphere, succinic anhydride (1.00 equiv per amine) was added in one portion to a solution of amine (1.00 equiv, 0.10 M) in anhydrous DCM. TEA (1.00 equiv per amine) was added and the solution was refluxed for 16 h. The solution was cooled to 0 °C and acetyl chloride (2.00 equiv per amine) was added dropwise and the solution was stirred at room temperature for a further 3 hours. The resulting solution was concentrated *in vacuo* and the crude product diluted in saturated aqueous $NaHCO_3$. The aqueous solution was extracted with DCM three times and the combined organic extracts were washed with brine, dried ($MgSO_4$), filtered and concentrated *in vacuo* to give the crude succinimide.

General procedure F – chemoselective Boc-protection of secondary amines by trifluoroacetylation, carboxylation and hydrolysis.

Under a dry, inert atmosphere, a solution of amine (1.00 equiv, 1.60 M) in anhydrous DCM was cooled to 0 °C. To the solution was added ethyl trifluoroacetate (1.10 equiv) dropwise over 30 minutes and the resultant solution was stirred for 30 minutes. The solution was warmed to room temperature and stirred for a further hour. The solution was then diluted with anhydrous DCM (10 mL $mmol^{-1}$) and cooled to 0 °C. A solution of di-*tert*-butyl dicarbonate (1.20 equiv per amine, 2.00 M) in anhydrous DCM was added over three portions followed by anhydrous TEA (1.00 equiv per amine) in one portion. The resulting solution was stirred for 16 h and diluted with saturated aqueous $NaHCO_3$. The organic phase was washed with deionised water, dried ($MgSO_4$), filtered and concentrated *in vacuo*. The crude yellow oil (0.20 M) was dissolved in methanol and a solution of aqueous NaOH (7.30 equiv, 4.20 M) was added. The resulting colourless solution was stirred for 5 h and concentrated *in vacuo*. The crude product was dissolved in deionised water and the aqueous phase was extracted with DCM four times. The combined organic extracts were dried ($MgSO_4$), filtered and concentrated *in vacuo* to give the title carbamate.

General procedure G – urea formation from amines and carbamoyl chlorides

Under a dry, inert atmosphere, a solution of secondary amine (1.00 equiv, 0.10 M) in anhydrous DCM was cooled to 0 °C. To this solution was added carbamoyl chloride (1.50 equiv per secondary amine) dropwise/over three portions and anhydrous triethylamine (1.50 equiv per secondary amine). The resulting solution was stirred for 16 h and then diluted with distilled water. The aqueous solution was

extracted with DCM three times and the combined organic extracts were washed with brine, dried ($MgSO_4$), filtered and concentrated *in vacuo* to give the crude urea.

General procedure H – Boc deprotection of amines using TFA

Under a dry, inert atmosphere, TFA (20% by volume) was added dropwise to a solution of Boc-protected amine (1.00 equiv, 0.10 M) in anhydrous DCM (80% by volume). The solution was stirred for 16 h and concentrated *in vacuo*. The crude product was diluted with saturated aqueous $NaHCO_3$ and then washed with DCM three times. The combined organic extracts were washed with brine, dried ($MgSO_4$), filtered and concentrated *in vacuo* to give the crude amine.

General procedure I – palladium-catalysed hydrogenative reductive amination

Under a dry, inert atmosphere, a round-bottomed flask was charged with a solution of amine (1.00 equiv, 0.10 M) in anhydrous MeOH. To this solution was added aldehyde (1.08 equiv) and palladium on carbon (10% wt., 10 mol%). The flask was then equipped with a three-way tap with a balloon filled with hydrogen attached. The flask was evacuated and refilled with nitrogen three times, and then evacuated and refilled with hydrogen. The black suspension was then left to stir for 16 h. The black suspension was then filtered through celite and washed with MeOH. The filtrate was the concentrated *in vacuo* to give the crude secondary amine.

General procedure J – monoalkylation of diamines by reductive amination

Under a dry, inert atmosphere, a solution of amine (1.00 equiv, 0.10 M) in anhydrous MeOH was prepared. To this solution, aldehyde (1.10 equiv) was added dropwise over 30 minutes. The resultant solution was stirred for 16 h. The solution was then cooled to 0 °C and sodium borohydride (3.00 equiv) was added over three portions. The resulting white suspension was warmed to room temperature, stirred for 3 h and concentrated *in vacuo*. The residue was then diluted with MeOH and concentrated *in vacuo*. The crude product was then diluted with distilled water and the aqueous solution extracted with DCM three times. The combined organic extracts were washed with brine, dried ($MgSO_4$), filtered and concentrated *in vacuo* to give the crude secondary amine.

General procedure K – Swern oxidation of primary alcohols

Under a dry, inert atmosphere, a solution of anhydrous DMSO (3.00 equiv, 0.40 M) in anhydrous DCM was cooled to −78 °C. To this solution was added a solution of oxalyl chloride (1.50 equiv, 2.00 M) in anhydrous DCM dropwise. The resultant solution was stirred for 20 minutes, and a solution of alcohol (1.00 equiv, 0.80 M) in anhydrous DCM was added dropwise. The resulting solution was stirred for 2 h at −78 °C and anhydrous TEA (9.00 equiv) was added dropwise to give a white suspension. The suspension was warmed to 0 °C and stirred for 30 minutes, after which the reaction was quenched with aqueous citric acid (10% w/w.). The mixture was stirred for 15 minutes at 0 °C and warmed to room temperature. The aqueous phase was then extracted with DCM three times, and the combined organic extracts washed with aqueous citric acid (10% w/w), distilled water, saturated aqueous Na_2CO_3 and brine. The combined organic extracts were then dried ($MgSO_4$), filtered and concentrated *in vacuo* to give the crude aldehyde.

General procedure L – Johnson-Lemieux oxidation of *N*-allyl carbamates/maleimides

Under an inert atmosphere, a solution of allyl carbamate/maleimide (1.00 equiv, 0.10 M) in dioxane:water (3:1) was prepared. To this solution was added sodium periodate (4.00 equiv), 2,6-lutidine (2.00 equiv) and osmium tetroxide (4% wt. in water, 0.10 equiv). The solution was stirred for 4 h and then quenched with saturated aqueous $Na_2S_2O_3$. After stirring for 30 minutes, the solution was diluted with EtOAc and the organic phase washed with saturated aqueous $Na_2S_2O_3$ and brine. The organic phase was then dried ($MgSO_4$), filtered and concentrated *in vacuo* to give the crude aldehyde.

6.3 Experimental Procedures

1,1-Dibenzyl-3-(4-methoxyphenyl)urea, 1

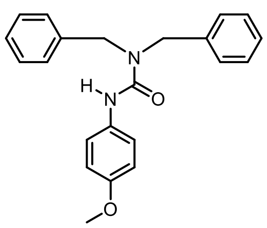

Synthesised according to general procedure A using *N*,*N*-dibenzylamine (198 mg, 1.00 mmol) and 4-methoxyphenyl isocyanate (0.10 mL, 0.75 mmol). The crude product was diluted with aqueous HCl (1.00 M) and extracted with DCM (3 x 10 mL). The combined organic extracts were dried ($MgSO_4$), filtered and concentrated *in vacuo* to yield the title amine as a white solid (296 mg, 1.00 mmol, >99%). **M.P.** 179-180 °C ($CHCl_3$). **TLC** – R_f = 0.23 (SiO_2, 1:99 MeOH:DCM). **^{1}H NMR** (400 MHz, $CDCl_3$) δ_H 3.66 (s, 3H, OC*H*$_3$), 4.50 (s, 4H, 2 x C*H*$_2$Ar), 6.17 (s, 1H, N*H*), 6.69 (d, J = 9.0, 2H, 2 x Ar*H*), 7.03 (d, J = 9.0, 2H, 2 x Ar*H*), 7.19-7.31 (m, 10H, 10 x Ar*H*). **^{13}C NMR** (101 MHz, $CDCl_3$) δ_C 50.8 (2 x *C*H$_2$Ar), 55.6 (O*C*H$_3$), 114.2 (2 x Ar*C*), 122.3 (2 x Ar*C*), 127.5 (4 x Ar*C*), 127.8 (2 x Ar*C*),

129.0 (4 x Ar*C*), 132.1 (2 x Ar*C*), 137.4 (Ar*C*), 156.0 (Ar*C*), 156.5 (*C*O). **HR-MS** (ESI, positive ion mode) – *m/z* for $[C_{22}H_{22}N_2O_2+H]^+$ = 347.1760. Found 347.1727. **FTIR** (neat) – 3431, 2988, 2901, 1653.

1,4-Dibenzyl-1,4-bis(4-methoxyanilinylcarbonyl)-1,4-diazabutane, 2a

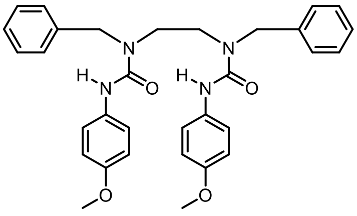

Synthesised according to general procedure A using *N,N'*-dibenzylethylenediamine (100 mg, 0.42 mmol) and 4-methoxyphenyl isocyanate (0.16 mL, 1.25 mmol). The crude product was purified by column chromatography (25-100% EtOAc:PE) to yield the title diurea as a foamy white solid (152 mg, 0.28 mmol, 67%). **M.P.** 136-137 °C ($CHCl_3$). **TLC** – R_f = 0.23 (SiO_2, 5:95 MeOH:DCM). **^{1}H NMR** (400 MHz, $CDCl_3$) δ_H 3.36 (s, 4H, 2 x NC*H*$_2$), 3.76 (s, 6H, 2 x OC*H*$_3$), 4.45 (s, 4H, 2 x C*H*$_2$Ar), 6.80 (d, J = 9.0, 4H, 4 x Ar*H*), 7.19 (d, J = 9.0, 4H, 4 x Ar*H*, 7.29-7.39 (m, 10H, 10 x Ar*H*), 7.61 (s, 2H, 2 x N*H*). **^{13}C NMR** (101 MHz, $CDCl_3$) δ_C 46.7 (2 x *C*H$_2$), 51.9 (2 x *C*H$_2$Ar), 55.5 (2 x O*C*H$_3$), 114.0 (4 x Ar*C*), 121.9 (4 x Ar*C*), 127.3 (4 x Ar*C*), 127.8 (4 x Ar*C*), 129.0 (2 x Ar*C*), 132.2 (2 x Ar*C*), 137.5 (2 x Ar*C*), 155.5 (2 x Ar*C*), 156.4 (2 x *C*O). **HR-MS** (ESI, positive ion mode) – *m/z* for $[C_{32}H_{34}N_4O_4+Na]^+$ = 561.2478. Found 561.2467. **FTIR** (neat) – 3291, 2945, 2901, 1638.

1,4-Diethyl-1,4-bis(4-methoxyanilinylcarbonyl)-1,4-diazabutane, 2b

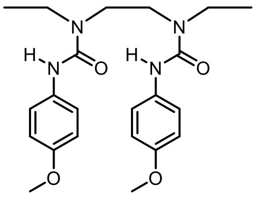

Synthesised according to general procedure A using *N,N'*-diethylethylenediamine (100 mg, 0.86 mmol) and 4-methoxyphenyl isocyanate (0.33 mL, 2.58 mmol). The crude product was purified by column chromatography (25-100% EtOAc:PE) to yield the title diurea as a foamy white solid (294 mg, 0.71 mmol, 82%). **M.P.** 219-220 °C ($CHCl_3$). **TLC** – R_f = 0.40 (SiO_2, 90:10 EtOAc:PE). **^{1}H NMR** (400 MHz, $CDCl_3$) δ_H 1.23 (t, J = 7.1, 6H, 2 x C*H*$_3$), 3.40 (q, J = 7.1, 4H, 2 x C*H*$_2$), 3.47 (s, 4H, 2 x C*H*$_2$), 3.78 (s, 6H, 2 x OC*H*$_3$), 6.82 (d, J = 9.0, 4H, 4 x Ar*H*), 7.31 (s, 2H, 2 x N*H*), 7.41 (d, J = 9.0, 4H, 4 x Ar*H*). **^{13}C NMR** (101 MHz, $CDCl_3$) δ_C 14.4 (2 x *C*H$_3$), 43.5 (2 x N*C*H$_2$CH$_3$), 46.7 (2 x *C*H$_2$), 55.7 (2 x O*C*H$_3$), 114.1 (4 x Ar*C*), 121.9 (4 x Ar*C*), 132.7 (2 x Ar*C*), 155.7 (2 x Ar*C*), 155.9 (2 x *C*O). **HR-MS** (ESI, positive ion mode) – *m/z* for $[C_{22}H_{30}N_4O_4+Na]^+$ = 437.2165. Found 437.2143. **FTIR** (neat) – 3280, 2984, 2919, 1629.

1,4-Diphenyl-1,4-bis(4anilinylcarbonyl)-1,4-diazabutane, 2c

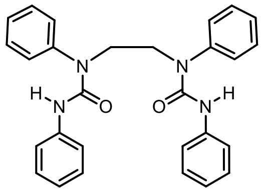

Synthesised according to general procedure A using *N,N'*-diphenylethylenediamine (50 mg, 0.24 mmol) and phenyl isocyanate (0.06 mL, 0.58 mmol). The crude product was purified by column chromatography (3% MeOH:DCM) to yield the title diurea as a white solid (108 mg, 0.24 mmol, >99%). **M.P.** 230-231 °C (DCM). **^{1}H NMR** (400 MHz, $CDCl_3$) δ_H 3.93 (s, 4H, 2 x C*H*$_2$), 6.49 (s, 2H, 2 x N*H*), 6.91 (t, J = 7.2, 2H, 2 x Ar*H*), 7.08-7.18 (m, 4H, 4 x Ar*H*), 7.20-7.26 (m, 8H, 8 x Ar*H*), 7.26-7.31 (m, 2H, 2 x Ar*H*), 7.31-7.43 (m, 4H, 4 x Ar*H*). **^{13}C NMR** (101 MHz, $CDCl_3$) δ_C 48.6 (2 x N*C*H$_2$), 119.7 (4 x Ar*C*), 123.1 (4 x Ar*C*), 128.1 (4 x Ar*C*), 128.3 (4 x Ar*C*), 128.9 (4 x Ar*C*), 130.4 (4 x Ar*C*), 138.9 (2 x Ar*C*), 141.9 (2 x Ar*C*), 154.6 (2 x *C*O). **HR-MS** (ESI, positive ion mode) – *m/z* for $[C_{28}H_{26}N_4O_2+Na]^+$ 473.1948, observed 473.1954. **FTIR** (neat) = 3417, 1668, 1593, 1518, 1492, 1488, 1198.

1,7-Dibenzyl-1,4,7-triazaheptane, 3a-1

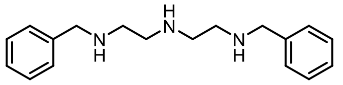

Synthesised according to general procedure B using diethylenetriamine (0.21 mL, 1.94 mmol) and benzaldehyde (0.39 mL, 3.88 mmol). The title triamine was isolated as a white powder (330 mg, 1.18 mmol, 61%). Spectroscopic data matched that previously reported.[1]

1,7-Dibenzyl-1,4,7-tris(4-methoxyanilinylcarbonyl)-1,4,7-triazaheptane, 3a

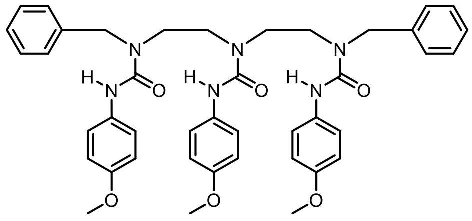

Synthesised according to general procedure A using **3a-1** (300 mg, 1.06 mmol) and 4-methoxyphenyl isocyanate (0.41 mL, 3.18 mmol). The crude product was purified by column chromatography (25-100% EtOAc:PE) to yield the title triurea as a white solid (418 mg, 0.57 mmol, 54%). **M.P.** 146-147 °C ($CHCl_3$). **TLC** – R_f = 0.20 (SiO_2, 90:10 EtOAc:PE). **^{1}H NMR** (500 MHz, $CDCl_3$) δ_H 3.34 (t, J = 7.4, 4H, 2 x NC*H*$_2$), 3.48 (t, J = 7.4, 4H, 2 x NC*H*$_2$), 3.79 (s, 6H, 2 x OC*H*$_3$), 3.81 (s, 3H, OC*H*$_3$), 4.57 (s, 4H, 2 x C*H*$_2$Ar), 6.83 (d, J = 9.0, 4H, 4 x Ar*H*), 6.85 (d, J = 9.0, 2H, 2 x Ar*H*), 7.29 (d, J = 9.0, 4H, 4 x Ar*H*), 7.32-7.44 (m, 10H, 10 x Ar*H*), 7.59 (d, J = 9.0, 2H, 2 x Ar*H*), 8.84 (s, 1H, N*H*). **^{13}C NMR** (126 MHz, $CDCl_3$) δ_C 47.8 (2 x N*C*H$_2$), 47.8 (2 x N*C*H$_2$), 52.4 (2 x *C*H$_2$Ar), 55.6 (2 x O*C*H$_3$), 55.6 (O*C*H$_3$), 114.0 (2 x Ar*C*), 114.1 (4 x Ar*C*), 121.2 (2 x Ar*C*), 121.8 (4 x Ar*C*), 127.1 (4 x Ar*C*), 127.2 (2 x Ar*C*), 129.2 (4 x Ar*C*), 133.1 (2 x Ar*C*), 133.1 (Ar*C*), 155.4 (2 x Ar*C*), 155.4 (Ar*C*), 156.5 (2 x *C*O), 156.5 (*C*O). **HR-MS** (ESI, positive ion mode) – *m/z* for $[C_{42}H_{46}N_6O_6+Na]^+$ = 753.3377. Found 753.3362. **FTIR** (neat) – 3334, 2992, 2948, 1650, 1624.

1,7-Dibenzyl-1,4,7-tris(butylaminocarbonyl)-1,4,7-triazaheptane, 3b

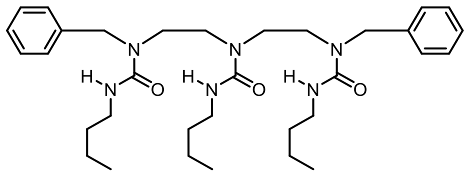

Synthesised according to general procedure A using **3a-1** (30 mg, 0.10 mmol) and *n*-butyl isocyanate (0.06 mL, 0.45 mmol). The crude product was purified by column chromatography (1-5% MeOH:DCM) to yield the title triurea as a white solid (31 mg, 0.05 mmol, 54%). **M.P.** 111-112 °C ($CHCl_3$). **TLC** – R_f = 0.68 (SiO_2, 90:10 DCM:MeOH). **^{1}H NMR** (400 MHz, $CDCl_3$) δ_H 0.80 (t, J = 7.4, 6H, 2 x C*H*$_3$), 0.85 (t, J = 7.3, 3H, C*H*$_3$), 1.12-1.50 (m, 12H, 6 x C*H*$_2$), 3.04-3.26 (m, 14H, 7 x NC*H*$_2$), 4.34 (s, 4H, 2 x C*H*$_2$Ar), 5.06 (s, 2H, 2 x N*H*), 6.38 (t, J = 5.0, 1H, N*H*), 7.13 (d, J = 7.5, 4H, 4 x Ar*H*), 7.20 (t, J = 7.2, 2H, 2 x Ar*H*), 7.26 (t, J = 7.5, 4H, 4 x Ar*H*). **^{13}C NMR** (101 MHz, $CDCl_3$) δ_C 13.9 (2 x *C*H$_3$), 14.0 (*C*H$_3$), 20.1 (2 x *C*H$_2$), 20.3 (*C*H$_2$), 32.2 (*C*H$_2$), 32.3 (2 x *C*H$_2$), 47.3 (3 x N*C*H$_2$), 52.0 (2 x *C*H$_2$Ar), 127.0 (4 x Ar*C*), 127.7 (2 x Ar*C*), 129.0 (4 x Ar*C*), 138.0 (2 x Ar*C*), 158.7 (2 x *C*O), 158.8 (*C*O). **HR-MS** (ESI, positive ion mode) – *m/z* for $[C_{33}H_{52}N_6O_3+Na]^+$ = 603.3999. Found 603.4012. **FTIR** (neat) – 3326, 2963, 2910, 1640.

1,10-Dibenzyl-1,4,7,10-tetrakis(4-methoxyanilinylcarbonyl)-1,4,7,10-tetraazadecane, 4a

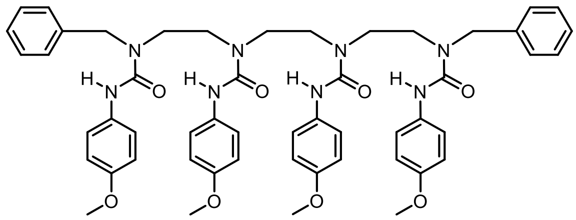

Step 1 (*reductive amination*): Under a dry, inert atmosphere, a solution of triethylenetetramine (0.31 mL, 2.05 mmol, 1.00 equiv, 0.10 M) in anhydrous MeOH (20 mL) was prepared. To this solution was added benzaldehyde (0.42 mL, 4.10 mmol, 2.00 equiv) and the resulting colourless solution was stirred for 16 h. The solution was cooled to 0 °C and $NaBH_4$ (388 mg, 10.26 mmol, 6.00 equiv) was added in one portion. The resulting white suspension was warmed to room temperature, stirred for 3 h and concentrated *in vacuo*. The residue was diluted with MeOH and concentrated *in vacuo*. The crude product was diluted with deionised water (20 mL) and the aqueous solution extracted with DCM (3 x 20 mL). The combined organic extracts were washed with brine, dried ($MgSO_4$), filtered and concentrated *in vacuo* to give the crude secondary amine. ***Step 2*** (*urea formation*): Under a dry, inert atmosphere, the crude brown oil (1.00 equiv, 0.10 M) was dissolved in anhydrous DCM (20 mL) and cooled to 0 °C. To the solution was added 4-methoxyphenyl isocyanate (1.30 mL, 12.30 mmol, 3.00 equiv) dropwise. The resulting solution was stirred for 16 h and concentrated *in vacuo*. The crude product was purified by column chromatography (1-2% MeOH:DCM) to yield the title tetraurea as a white solid (312 mg, 0.34 mmol, 16%). **M.P.** 158-159 °C (DCM). **TLC** – R_f = 0.21 (SiO_2, 1:99 MeOH:DCM). **^{1}H NMR** (400 MHz, $CDCl_3$) δ_H 3.32 (s, 4H, 2 x NC*H*$_2$), 3.39-3.43 (m, 4H, 2 x NC*H*$_2$), 3.48 (t, J = 7.5, 4H, 2 x NC*H*$_2$), 3.76 (s, 6H, 2 x OC*H*$_3$), 3.78 (s, 6H, 2 x OC*H*$_3$), 6.80 (d, J = 9.1, 4H, 4 x Ar*H*), 6.82 (d, J = 9.1, 4H, 4 x Ar*H*), 7.27 (d, J = 9.1, 4H, 4 X Ar*H*), 7.29-7.40 (m, 10H, 10 x Ar*H*), 7.59 (d, J = 9.1, 4H, 4 x Ar*H*), 8.33 (s, 2H, 2 x N*H*), 8.93 (s, 2H,

2 x NH). **^{13}C NMR** (101 MHz, $CDCl_3$) δ_C 47.7 (6 x NCH_2), 52.2 (2 x CH_2Ar), 55.5 (2 x OCH_3), 55.5 (2 x OCH_3), 113.9 (4 x Ar*C*), 114.0 (4 x Ar*C*), 121.1 (4 x Ar*C*), 121.9 (4 x Ar*C*), 126.8 (4 x Ar*C*), 127.9 (2 x Ar*C*), 129.1 (4 x Ar*C*), 133.1 (4 x Ar*C*), 155.2 (4 x Ar*C*), 156.4 (4 x *C*O). **HR-MS** (ESI, positive ion mode) – *m/z* for $[C_{52}H_{58}N_8O_8+Na]^+$ = 945.4275. Found 945.4233. **FTIR** (neat) – 3330, 2992, 2948, 1643, 1604.

1-Ethyl-7-(*tert*-butoxycarbonyl)-1,4,7-triazaheptane, 3c-1

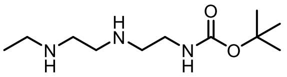

Synthesised according to general procedure I using *N*-ethylethylenediamine (0.60 mL, 5.67 mmol) and *N*-Boc-2-aminoacetaldehyde (910 mg, 6.12 mmol). The title carbamate was isolated as a colourless oil (879 mg, 3.80 mmol, 67%). **TLC** – R_f = 0.23 (SiO_2, 10:90:1 MeOH:DCM:NH_4OH). **^{1}H NMR** (400 MHz, $CDCl_3$) δ_H 1.23 (t, J = 6.5, 3H, CH_3), 1.44 (s, 9H, C(CH_3)$_3$), 2.76 (t, J = 5.4, 2H, NCH_2), 2.91 (t, J = 6.3, 2H, NCH_2), 3.00 (t, J = 6.3, 2H, NCH_2), 3.23 (C^2H_2, 2H, m), 3.71 (q, J = 5.4, 2H, NCH_2), 5.45 (t, J = 5.4, 1H, NH). **^{13}C NMR** (101 MHz, $CDCl_3$) δ_C 18.4 (CH_3), 28.5 (C(CH_3), 40.1 (NCH_2), 46.0 (NCH_2), 47.1 (NCH_2), 49.1 (NCH_2), 58.4 (NCH_2), 81.4 (*C*(CH_3)$_3$), 156.9 (*C*O). **HR-MS** (ESI, positive ion mode) – *m/z* for $[C_{11}H_{25}N_3O_2+H]^+$ = 232.2025 . Found 232.2019. **FTIR** (neat) – 3352, 2963, 1694.

1-Ethyl-1,4-bis(4-methoxyanilinylcarbonyl)-7-(*tert*-butoxycarbonyl)-1,4,7-triazanonane, 3c-2

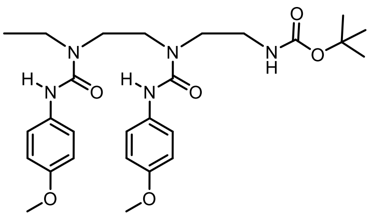

Synthesised according to general procedure A using **3c-1** (100 mg, 0.43 mmol) and 4-methoxyphenyl isocyanate (0.17 mL, 1.30 mmol). The crude product was purified by column chromatography (25-100% EtOAc:PE) to yield the title diurea as a foamy white solid (122 mg, 0.25 mmol, 57%). **M.P.** 158-159 °C ($CHCl_3$). **TLC** – R_f = 0.31 (SiO_2, 90:10 EtOAc:PE). **^{1}H NMR** (400 MHz, $CDCl_3$) δ_H 1.15 (t, J = 7.1, 3H, CH_3), 1.43 (s, 9H, C(CH_3)$_3$), 3.15 (t, J = 6.5, 2H, NCH_2), 3.36-3.45 (m, 8H, 4 x NCH_2), 3.78 (s, 3H, OCH_3), 3.79 (s, 3H, OCH_3), 5.35 (s, 1H, NH), 6.82 (d, J = 9.1, 2H, 2 x ArH), 6.84 (d, J = 9.1, 2H, 2 x ArH), 7.4 (d, J = 9.1, 2H, 2 x ArH), 7.54 (d, J = 9.1, 2H, 2 x ArH), 8.51 (s, 1H, NH). **^{13}C NMR** (101 MHz, $CDCl_3$) δ_C 14.1 (CH_3), 28.2 (C(CH_3)$_3$), 40.1 (NCH_2), 43.0 (NCH_2), 45.9 (NCH_2), 47.8 (NCH_2), 47.9 (NCH_2), 55.5 (2 x OCH_3), 80.1 (*C*(CH_3)$_3$), 114.0 (4 x Ar*C*), 121.3 (2 x Ar*C*), 121.8 (2 x Ar*C*), 133.0 (Ar*C*), 133.1 (Ar*C*), 155.3 (Ar*C*), 155.3 (Ar*C*), 155.9 (*C*O) 156.4 (*C*O), 157.1 (*C*O). **HR-MS** (ESI, positive ion mode) – *m/z* for $[C_{27}H_{39}N_5O_6+Na]^+$ = 552.2798. Found 552.2777. **FTIR** (neat) – 3311, 2917, 1645, 1628.

1-Ethyl-1,4-bis(4-methoxyanilinylcarbonyl)-1,4,7-triazanonane, 3c-3

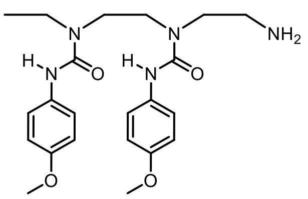

Synthesised according to general procedure H using **3c-2** (100 mg, 0.22 mmol). The title amine was isolated as a foamy white solid (76 mg, 0.21 mmol, 96%). **M.P.** 162-163 °C ($CHCl_3$). **TLC** – R_f = 0.03 (SiO_2, 90:10 EtOAc:PE). **^{1}H NMR** (400 MHz, $CDCl_3$) δ_H 1.14 (t, J = 7.2, 3H, C*H*$_3$), 1.78 (s, 2H, N*H*$_2$), 2.89 (t, J = 4.2, 2H, NC*H*$_2$), 3.34-3.44 (m, 8H, 4 x NC*H*$_2$), 3.74 (s, 3H, OC*H*$_3$), 3.75 (s, 3H, OC*H*$_3$), 6.75 (d, J = 9.0, 2H, x Ar*H*), 6.77 (d, J = 9.0, 2H, x Ar*H*), 7.31 (d, J = 9.0, 2H, Ar*H*), 7.50 (d, J = 9.0, 2H, Ar*H*), 8.29 (s, 1H, N*H*), 10.0 (s, 1H, N*H*). **^{13}C NMR** (101 MHz, $CDCl_3$) δ_C 14.2 (*C*H$_3$), 41.7 (N*C*H$_2$), 42.7 (N*C*H$_2$), 44.8 (N*C*H$_2$), 48.1 (N*C*H$_2$), 52.8 (N*C*H$_2$), 55.5 (O*C*H$_3$), 55.6 (O*C*H$_3$), 113.9 (2 x Ar*C*), 114.0 (2 x Ar*C*), 120.8 (2 x Ar*C*), 121.5 (2 x Ar*C*), 133.3 (Ar*C*), 133.5 (Ar*C*), 155.1 (2 x Ar*C*), 155.9 (*C*O), 158.4 (*C*O). **HR-MS** (ESI, positive ion mode) – *m/z* for $[C_{22}H_{31}N_5O_4+H]^+$ = 430.2454. Found 430.2451. **FTIR** (neat) – 3444, 3354, 2966, 2902, 1671.

1-Ethyl-1,4-bis(4-methoxyanilinylcarbonyl)-7-benzyl-1,4,7-triazanonane, 3c-4

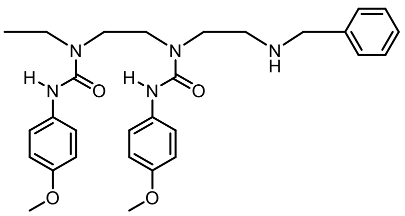

Synthesised according to general procedure B using **3c-3** (396 mg, 0.94 mmol) and benzaldehyde (0.15 mL, 1.41 mmol). The crude product was purified by column chromatography (2-10% MeOH:DCM) to yield the title amine as a white solid (249 mg, 0.48 mmol, 51%). **M.P.** 132-133 °C ($CHCl_3$). **TLC** – R_f = 0.30 (SiO_2, 5:95 MeOH:DCM). **^{1}H NMR** (400 MHz, $CDCl_3$) δ_H 1.14 (t, J = 7.0, 3H, C*H*$_3$), 2.07 (s, 1H, N*H*), 2.85 (t, J = 4.3, NC*H*$_2$), 3.33-3.45 (m, 8H, 4 x NC*H*$_2$), 3.75 (s, 3H, OC*H*$_3$), 3.76 (s, 3H, OC*H*$_3$), 3.81 (s, 2H, C*H*$_2$Ar), 6.76 (d, J = 8.5, 2H, 2 x Ar*H*), 6.79 (d, J = 8.5, 2H, 2 x Ar*H*), 7.18 (d, J = 8.5, 2H, 2 x Ar*H*), 7.23-7.33 (m, 5H, 5 x Ar*H*), 7.51 (d, J = 8.5, 2H, 2 x Ar*H*), 8.27 (s, 1H, N*H*), 9.91 (s, 1H, N*H*). **^{13}C NMR** (101 MHz, $CDCl_3$) δ_C 14.1 (*C*H$_3$), 42.6 (N*C*H$_2$), 45.1 (N*C*H$_2$), 48.2 (N*C*H$_2$), 49.4 (N*C*H$_2$), 50.7 (N*C*H$_2$), 54.2 (*C*H$_2$Ar), 55.5 (O*C*H$_3$), 55.5 (O*C*H$_3$), 113.9 (2 x Ar*C*), 113.9 (2 x Ar*C*), 120.9 (2 x Ar*C*), 121.3 (2 x Ar*C*), 127.5 (2 x Ar*C*), 128.3 (2 x Ar*C*), 128.7 (Ar*C*), 133.1 (Ar*C*), 133.5 (Ar*C*), 138.9 (Ar*C*), 155.0 (2 x Ar*C*), 155.7 (*C*O), 158.2 (*C*O). **HR-MS** (ESI, positive ion mode) – *m/z* for $[C_{29}H_{37}N_5O_4+H]^+$ = 520.2924. Found 520.2893. **FTIR** (neat) – 3337, 3322, 2993, 2912, 1654.

1-Ethyl-1,4,7-tris(4-methoxyanilinylcarbonyl)-7-benzyl-1,4,7-triazanonane, 3c

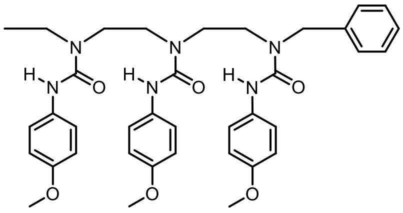

Synthesised according to general procedure A using **3c-4** (120 mg, 0.23 mmol) and 4-methoxyphenyl isocyanate (0.04 mL, 0.35 mmol). The crude product was purified by column chromatography (1-2% MeOH:DCM) to yield the title triurea as a foamy white solid (112 mg, 0.17 mmol, 73%). **M.P.** 110-111 °C ($CHCl_3$). **TLC** – R_f = 0.15 (SiO_2, 1:99 MeOH:DCM). **^{1}H NMR** (500 MHz, $CDCl_3$) δ_H 1.16 (t, J = 7.1, 3H, C*H*$_3$), 3.30 (q, J = 7.1, 2H, NC*H*$_2$), 3.29-3.41 (m, 6H, 3 x NC*H*$_2$), 3.49 (t, J = 7.4, 2H, NC*H*$_2$), 3.76 (s, 3H, OC*H*$_3$), 3.77 (s, 3H, OC*H*$_3$), 3.77 (s, 3H, OC*H*$_3$), 4.58 (s, 2H, C*H*$_2$Ar), 6.79-6.83 (m, 6H, 6 x Ar*H*), 7.27-7.33 (m, 4H, 4 x Ar*H*), 7.34-7.39 (m, 5H, 5 x Ar*H*), 7.57 (d, J = 9.0, 2H, 2 x Ar*H*), 8.89 (s, 1H, N*H*). **^{13}C NMR** (126 MHz, $CDCl_3$) δ_C 14.3 (*C*H$_3$), 43.7 (N*C*H$_2$), 46.7 (N*C*H$_2$), 47.9 (3 x N*C*H$_2$), 51.9 (*C*H$_2$Ar), 55.6 (3 x O*C*H$_3$), 113.9 (2 x Ar*C*), 113.9 (2 x Ar*C*), 114.0 (2 x Ar*C*), 121.2 (2 x Ar*C*), 121.8 (2 x Ar*C*), 122.0 (2 x Ar*C*), 127.2 (2 x Ar*C*), 127.9 (Ar*C*), 129.0 (2 x Ar*C*), 133.3 (3 x Ar*C*), 137.8 (Ar*C*), 155.3 (*C*O), 155.6 (3 x Ar*C*), 156.0 (*C*O), 156.5 (*C*O). **HR-MS** (ESI, positive ion mode) – *m/z* for $[C_{37}H_{44}N_6O_6+H]^+$ = 669.3401. Found 669.3424. **FTIR** (neat) – 3312, 2978, 2923, 1650.

1-Phenyl-7-(*tert*-butoxycarbonyl)-1,4,7-triazaheptane, 3d-1

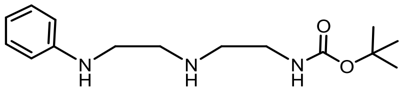

Synthesised according to general procedure I using *N*-phenylethylenediamine (0.50 mL, 3.80 mmol) and *N*-boc-2-aminoacetaldehyde (653 mg, 4.10 mmol). The title carbamate was isolated as a colourless oil (727 mg, 2.62 mmol, 69%). Spectroscopic data matched that previously reported.[2]

1-Phenyl-1,4-bis(4-methoxyanilinylcarbonyl)-7-(*tert*-butoxycarbonyl)-1,4,7-triazanonane, 3d-2

Synthesised according to general procedure A using **3d-1** (626 mg, 2.24 mmol) and 4-methoxyphenyl

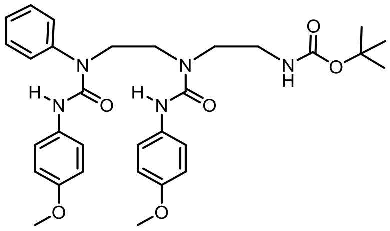

isocyanate (0.87 mL, 6.72 mmol). The crude product was purified by column chromatography (12-100% EtOAc:PE) to yield the title diurea as a foamy white solid (686 mg, 1.19 mmol, 53%). **M.P.** 80-81 °C ($CHCl_3$). **TLC** – R_f = 0.19 (SiO_2, 50:50 EtOAc:PE). **^{1}H NMR** (400 MHz, $CDCl_3$) δ_H 1.40 (s, 9H, C(C*H*$_3$)$_3$), 3.26 (q, J = 5.7, NC*H*$_2$), 3.44 (t, J = 6.1, 2H, NC*H*$_2$), 3.65 (t, J = 5.7, 2H, NC*H*$_2$), 3.77 (s, 3H, OC*H*$_3$), 3.78 (s, 3H, OC*H*$_3$), 3.82 (t, J = 6.1, 2H, NC*H*$_2$), 5.14 (t, J = 5.7, 1H, N*H*), 6.15 (s, 1H, N*H*), 6.81 (d, J = 8.9, 2H, 2 x Ar*H*), 6.83 (d, J = 8.9, 2H, 2 x Ar*H*), 7.19 (d, J = 8.9, 2H, 2 x Ar*H*), 7.31 (d, J = 7.6, 2H, 2 x Ar*H*), 7.41 (t, J = 7.6, 1H, Ar*H*), 7.50 (t, J = 7.6, 2H, Ar*H*), 7.55 (d, J = 8.9, 2H, Ar*H*), 8.67 (s, 1H, N*H*). **^{13}C NMR** (101 MHz, $CDCl_3$) δ_C 28.6 (C(*C*H$_3$)$_3$), 40.2 (N*C*H$_2$), 46.5 (N*C*H$_2$), 47.3 (N*C*H$_2$), 49.6 (N*C*H$_2$), 55.6 (2 x O*C*H$_3$),

79.5 ($C(CH_3)_3$), 114.0-114.2 (4 x Ar*C*), 121.5-121.8 (4 x Ar*C*), 128.1 (2 x Ar*C*), 128.7 (Ar*C*), 130.9 (2 x Ar*C*), 131.4 (Ar*C*), 133.5 (Ar*C*), 141.8 (Ar*C*), 155.3-155.5 (2 x Ar*C*), 156.0 (*C*O), 156.6 (2 x *C*O). **HR-MS** (ESI, positive ion mode) – *m/z* for $[C_{31}H_{39}N_5O_6+H]^+$ = 578.2979. Found 578.2954. **FTIR** (neat) – 3423, 3321, 2973, 2935, 1652, 1594.

1-Phenyl-1,4-bis(4-methoxyanilinylcarbonyl)-1,4,7-triazaheptane, 3d-3

Synthesised according to general procedure H using **3d-2** (200 mg, 0.34 mmol). The title amine was isolated as a foamy white solid (141 mg, 0.30 mmol, 87%). **M.P.** 123-124 °C (DCM). **TLC** – R_f = 0.10 (SiO_2, 90:10 EtOAc:PE). **^{1}H NMR** (500 MHz, $CDCl_3$) δ_H 2.91 (t, J = 5.6, 2H, NCH_2), 3.41 (t, J = 5.6, 2H, NCH_2), 3.63 (t, J = 7.2, 2H, NCH_2), 3.76 (s, 3H, OCH_3), 3.77 (s, 3H, OCH_3), 3.86 (t, J = 7.2, 2H, NCH_2), 6.28 (s, 1H, N*H*), 6.79 (d, J = 9.0, 2H, 2 x Ar*H*), 6.81 (d, J = 9.0, 2H, 2 x Ar*H*), 7.20 (d, J = 9.0, 2H, 2 x Ar*H*), 7.33 (t, J = 8.2, 2H, 2 x Ar*H*), 7.38 (tt, J = 7.4, 1.2, 1H, Ar*H*), 7.42 (d, J = 9.0, 2H, 2 x Ar*H*), 7.48 (t, J = 8.2, 2H, 2 x Ar*H*), 9.23 (s, 1H, N*H*). **^{13}C NMR** (126 MHz, $CDCl_3$) δ_C 41.5 (NCH_2), 46.6 (NCH_2), 49.0 (NCH_2), 51.5 (NCH_2), 55.5 (2 x OCH_3), 113.9-114.0 (4 x Ar*C*), 121.1-121.8 (4 x Ar*C*), 128.0-128.2 (3 x Ar*C*), 130.5 (2 x Ar*C*), 131.4 (Ar*C*), 133.5 (Ar*C*), 141.8 (Ar*C*), 155.1-155.4 (2 x Ar*C*), 157.0 (2 x *C*O). **HR-MS** (ESI, positive ion mode) – *m/z* for $[C_{26}H_{31}N_5O_4+H]^+$ = 478.2454. Found 478.2446. **FTIR** (neat) – 3677, 3432, 2985, 2902, 1654.

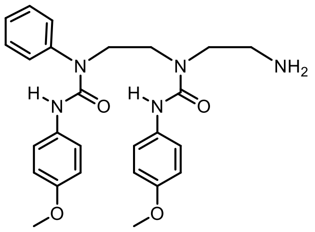

1-Phenyl-1,4-bis(4-methoxyanilinylcarbonyl)-7-benzyl-1,4,7-triazaheptane, 3d-4

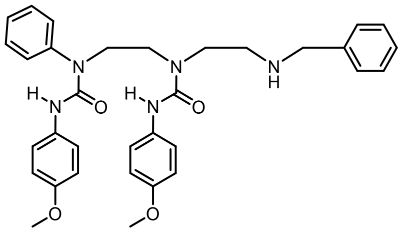

Synthesised according to general procedure B using **3d-3** (300 mg, 0.63 mmol) and benzaldehyde (0.13 mL, 1.26 mmol). The crude product was purified using column chromatography (1-5% MeOH:DCM, 1% TEA) to yield the title amine as a foamy white solid (285 mg, 0.50 mmol, 80%). **M.P.** 187-188 °C ($CHCl_3$). **TLC** – R_f = 0.08 (SiO_2, 1:99 MeOH:DCM). **^{1}H NMR** (400 MHz, $CDCl_3$) δ_H 2.88 (t, J = 5.5, 2H, NCH_2), 3.48 (t, J = 5.5, 2H, NCH_2), 3.62 (t, J = 6.9, 2H, NCH_2), 3.76 (s, 3H, OCH_3), 3.77 (s, 3H, OCH_3), 3.81 (s, 2H, CH_2Ar), 3.85 (t, J = 6.9, 2H, NCH_2), 6.30 (s, 1H, N*H*), 6.78 (d, J = 9.0, 2H, 2 x Ar*H*), 6.79 (d, J = 9.0, 2H, 2 x Ar*H*), 7.20 (d, J = 9.0, 2H, 2 x Ar*H*), 7.26-7.39 (m, 10H, 10 x Ar*H*), 7.46 (d, J = 9.0, 2H, 2 x Ar*H*), 9.26 (s, 1H, N*H*). **^{13}C NMR** (101 MHz, $CDCl_3$) δ_C 46.8 (NCH_2), 48.9-49.3 (3 x NCH_2), 54.1 (CH_2Ar), 55.5 (2 x OCH_3), 113.9 (2 x Ar*C*), 114.1 (2 x Ar*C*), 121.2 (2 x Ar*C*), 121.8 (2 x Ar*C*), 127.0 (Ar*C*), 127.2 (2 x Ar*C*), 128.0 (2 x Ar*C*), 128.2 (Ar*C*), 128.3 (2 x Ar*C*), 128.5 (2 x Ar*C*), 131.5 (Ar*C*), 133.5 (Ar*C*), 141.9 (Ar*C*), 150.3 (Ar*C*), 155.0 (Ar*C*), 155.2 (Ar*C*), 155.9 (*C*O), 156.4 (*C*O). **HR-MS**

(ESI, positive ion mode) – *m/z* for $[C_{33}H_{37}N_5O_4+H]^+$ = 568.2924. Found 569.2897. **FTIR** (neat) – 3410, 2933, 2833, 1651.

1-Phenyl-1,4,7-tris(4-methoxyanilinylcarbonyl)-7-benzyl-1,4,7-triazaheptane, 3d

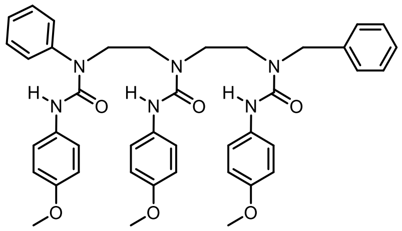

Synthesised according to general procedure A using **3d-4** (100 mg, 0.18 mmol) and 4-methoxyphenyl isocyanate (0.03 mL, 0.26 mmol). The crude product was purified using column chromatography (2-10% MeOH:DCM) to give the title triurea as a foamy white solid (65 mg, 0.09 mmol, 50%). **M.P.** 146-147 °C ($CHCl_3$). **TLC** – R_f = 0.43 (SiO_2, 5:95 MeOH:DCM). **^{1}H NMR** (400 MHz, $CDCl_3$) δ_H 3.15 (t, J = 7.0, 2H, NCH_2), 3.35 (t, J = 7.0, 2H, NCH_2), 3.48 (t, J = 7.0, 2H, NCH_2), 3.64 (t, J = 7.0, 2H, NCH_2), 3.78 (s, 3H, OCH_3), 3.78 (s, 3H, OCH_3), 3.80 (s, 3H, OCH_3), 4.60 (s, 2H, CH_2Ar), 6.14 (s, 1H, N*H*), 6.73 (d, J = 9.1, 2H, 2 x Ar*H*), 6.74 (d, J = 9.1, 2H, 2 x Ar*H*), 6.75 (d, J = 9.1, 2H, 2 x Ar*H*), 7.10 (d, J = 9.1, 2H, 2 x Ar*H*), 7.20-7.36 (m, 10H, 10 x Ar*H*), 7.43 (d, J = 9.1, 2H, 2 x Ar*H*), 7.48 (d, J = 9.1, 2H, 2 x Ar*H*), 8.33 (s, 1H, N*H*), 8.72 (s, 1H, N*H*). **^{13}C NMR** (101 MHz, $CDCl_3$) δ_C 45.5 (N*C*H_2), 47.3 (N*C*H_2), 47.8 (N*C*H_2), 49.9 (N*C*H_2), 51.0 (*C*H_2Ar), 55.5 (3 x O*C*H_3), 113.9 (2 x Ar*C*), 113.9 (2 x Ar*C*), 114.1 (2 x Ar*C*), 121.2 (2 x Ar*C*), 121.4 (2 x Ar*C*), 121.9 (2 x Ar*C*), 127.4-128.7 (10 x Ar*C*), 130.7 (Ar*C*), 131.0 (Ar*C*), 133.0 (Ar*C*), 138.7 (Ar*C*), 141.7 (Ar*C*), 155.2 (Ar*C*), 155.3 (Ar*C*), 155.5 (Ar*C*), 156.1 (*C*O), 156.2 (*C*O), 156.4 (*C*O). **HR-MS** (ESI, positive ion mode) – *m/z* for $[C_{41}H_{44}N_6O_6+H]^+$ = 717.3401. Found 717.3391. **FTIR** (neat) – 3398, 2952, 1651.

1,7-Bis(trifluoroacetyl)-4-(4-methoxyanilinylcarbonyl)-1,4,7-triazaheptane, 3e-1

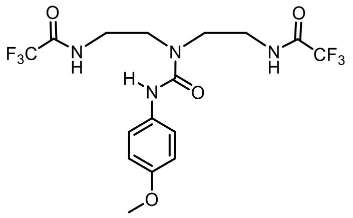

Synthesised according to general procedure C using diethylenetriamine (2.10 mL, 19.4 mmol) and 4-methoxyphenyl isocyanate (3.77 mL, 29.1 mmol). The resulting solution was concentrated *in vacuo* and the residue was recrystallised from DCM:Et_2O. The residue was then washed with Et_2O and air-dried to yield the title urea as a white solid (8.20 g, 18.5 mmol, 95%). **M.P.** 134-135 °C ($CHCl_3$). **TLC** – R_f = 0.25 (SiO_2, 5:95 MeOH:DCM). **^{1}H NMR** (500 MHz, $(CD)_3SO$) δ_H 3.38 (q, J = 5.9, 4H, 2 x NCH_2), 3.46 (t, J = 5.9, 4H, 2 x NCH_2), 6.84 (d, J = 8.9, 2H, 2 x Ar*H*), 7.32 (d, J = 8.9, 2H, 2 x Ar*H*), 8.08 (s, 1H, N*H*), 9.50 (t, J = 5.9, 2H, 2 x N*H*). **^{13}C NMR** (126 MHz, $(CD)_3SO$) δ_C 38.1 (2 x N*C*H_2), 45.7 (2 x N*C*H_2), 55.1 (O*C*H_3), 113.5 (2 x Ar*C*), 115.9 (q, J = 287.5, 2 x *C*F_3), 122.2 (2 x Ar*C*), 133.1 (Ar*C*), 154.7 (O*C*H_3), 155.3 (*C*O), 156.5 (q, J = 37.3, 2 x *C*O). **^{19}F NMR** (377 MHz, $CDCl_3$) δ_F −76.8 (2 x C*F*$_3$). **HR-MS** (ESI, positive ion mode) – *m/z* for $[C_{16}H_{18}F_6N_4O_4+H]^+$ = 445.1310. Found 445.1292. **FTIR** (neat) – 2960, 1731, 1706.

4-(4-Methoxyanilinylcarbonyl)-1,4,7-triazaheptane, 3e-2

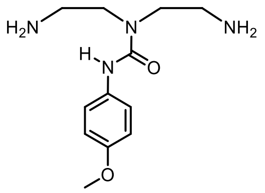

Synthesised according to general procedure D using **3e-1** (2.5 g, 5.63 mmol). The resulting solution was concentrated *in vacuo* and the residue was diluted with deionised water. The aqueous solution was extracted with DCM (3 x 50 mL). The combined organic extracts were washed with brine, dried ($MgSO_4$), filtered and concentrated *in vacuo* to give the title diamine as a colourless oil (450 mg, 1.78 mmol, 32%). **TLC** – R_f = 0.04 (SiO_2, 10:90:1 DCM:MeOH:TEA).**^{1}H NMR** (400 MHz, $CDCl_3$) δ_H 1.49 (s, 4H, 2 x N*H*$_2$), 2.84 (t, J = 5.7, 4H, 2 x NC*H*$_2$), 3.29 (t, J = 5.7, 4H, 2 x NC*H*$_2$), 3.68 (s, 3H, OC*H*$_3$), 6.72 (d, J = 9.0, 2H, 2 x Ar*H*), 7.20 (d, J = 9.0, 2H, 2 x Ar*H*), 9.86 (s, 1H, N*H*). **^{13}C NMR** (101 MHz, $CDCl_3$) δ_C 41.2 (2 x N*C*H$_2$), 51.3 (2 x N*C*H$_2$), 55.5 (O*C*H$_3$), 114.0 (2 x Ar*C*), 120.8 (2 x Ar*C*), 133.8 (Ar*C*), 154.8 (Ar*C*), 158.3 (*C*O). **HR-MS** (ESI, positive ion mode) – *m/z* for $[C_{12}H_{20}N_4O_2+H]^+$ = 253.1665. Found 253.1653. **FTIR** (neat) – 2938, 2838, 1643, 1604.

1,7-Dibenzyl-4-(4-methoxyanilinylcarbonyl)-1,4,7-triazaheptane, 3e-3

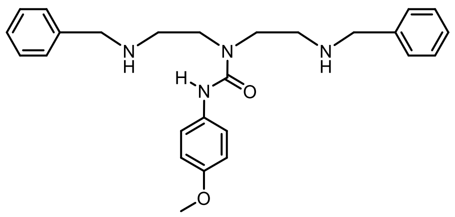

Synthesised according to general procedure B using **3e-2** (380 mg, 1.51 mmol) and benzaldehyde (0.46 mL, 4.52 mmol). The crude product was purified using column chromatography (2-10% MeOH:DCM) to yield the title diamine as a colourless oil (315 mg, 0.73 mmol, 48%). **TLC** – R_f = 0.26 (SiO_2, 10:90 MeOH:DCM). **^{1}H NMR** (400 MHz, $CDCl_3$) δ_H 1.85 (s, 2H, 2 x N*H*), 2.86 (t, J = 5.6, 4H, 2 x NC*H*$_2$), 3.44 (t, J = 5.6, 4H, 2 x NC*H*$_2$), 3.76 (s, 3H, OC*H*$_3$), 3.81 (s, 4H, 2 x C*H*$_2$Ar), 6.76 (d, J = 9.0, 2H, 2 x Ar*H*), 7.14 (d, J = 9.0, 2H, 2 x Ar*H*), 7.26-7.34 (m, 10H, 10 x Ar*H*), 9.70 (s, 1H, N*H*). **^{13}C NMR** (101 MHz, $CDCl_3$) δ_C 48.7 (2 x N*C*H$_2$), 49.2 (2 x N*C*H$_2$), 54.2 (2 x *C*H$_2$Ar), 55.7 (O*C*H$_3$), 114.1 (2 x Ar*C*), 121.1 (2 x Ar*C*), 127.2 (2 x Ar*C*), 128.4 (4 x Ar*C*), 128.7 (4 x Ar*C*), 133.7 (Ar*C*), 139.7 (2 x Ar*C*), 155.0 (Ar*C*), 158.2 (*C*O). **HR-MS** (ESI, positive ion mode) – *m/z* for $[C_{26}H_{32}N_4O_2+H]^+$ = 433.2604. Found 4332.2586. **FTIR** (neat) – 2931, 2835, 1650.

1,7-Dibenzyl-1,4-bis(4-methoxyanilinylcarbonyl)-1,4,7-triazaheptane, 3e-4

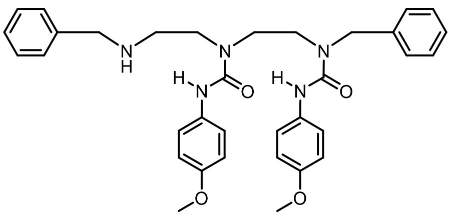

Synthesised according to general procedure A using **3e-3** (271 mg, 0.63 mmol) and 4-methoxyphenyl isocyanate (0.08 mL, 0.63 mmol). The crude product was purified using column chromatography (2-10% MeOH:DCM) to yield the title diamine as a foamy white solid (238 mg, 0.41 mmol, 65%). **M.P.** 140-141 °C ($CHCl_3$/PE). **TLC** – R_f = 0.26 (SiO_2, 5:95 MeOH:DCM). **^{1}H NMR** (500 MHz, $CDCl_3$) δ_H 2.82 (t, J = 4.5, 2H, NC*H*$_2$), 3.25 (t, J = 7.8, 2H, NC*H*$_2$), 3.29 (t, J = 4.5, 2H, NC*H*$_2$), 3.47 (d, J = 7.8, 2H, NC*H*$_2$), 3.77 (s, 3H, OC*H*$_3$), 3.77 (s, 3H, OC*H*$_3$), 3.82 (s, 2H, C*H*$_2$Ar), 4.62 (s, 2H, C*H*$_2$Ar), 6.76 (d, J = 8.8, 2H, 2 x Ar*H*), 6.80 (d, J = 9.0, 2H, 2 x Ar*H*), 7.15 (d, J = 9.0, 2H, 2 x Ar*H*), 7.24-7.27 (m, 2H, 2 x Ar*H*), 7.27-7.35 (m, 8H, 8 x Ar*H*), 7.52 (d, J = 8.8, 2H, 2 x Ar*H*), 8.36 (s, 1H, N*H*), 9.81 (s, 1H, N*H*). **^{13}C NMR** (126 MHz, $CDCl_3$) δ_C 45.3 (N*C*H$_2$), 48.0 (N*C*H$_2$), 49.4 (N*C*H$_2$), 51.0 (N*C*H$_2$), 51.1 (*C*H$_2$Ar), 54.4 (*C*H$_2$Ar), 55.7 (2 x O*C*H$_3$), 114.0 (2 x Ar*C*), 114.1 (2 x Ar*C*), 121.0 (2 x Ar*C*), 121.5 (2 x Ar*C*), 127.6 (Ar*C*), 127.7 (Ar*C*), 127.9 (2 x Ar*C*), 128.5 (2 x Ar*C*), 128.8 (2 x Ar*C*), 128.9 (2 x Ar*C*), 133.1 (Ar*C*), 133.5 (Ar*C*), 138.6 (Ar*C*), 138.9 (Ar*C*), 155.3 (2 x Ar*C*), 156.3 (*C*O), 158.3 (*C*O). **HR-MS** (ESI, positive ion mode) – *m/z* for $[C_{34}H_{39}N_5O_4+H]^+$ = 582.3080. Found 582.3101. **FTIR** (neat) – 2968, 2905, 1653, 1648.

1,7-Dibenzyl-1,4-bis(4-methoxyanilinylcarbonyl)-7-butylaminocarbonyl-1,4,7-triazaheptane, 3e

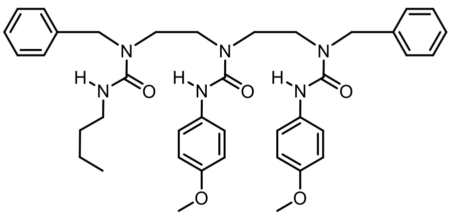

Synthesised according to general procedure A using **3e-4** (60 mg, 0.10 mmol) and *n*-butyl isocyanate (0.02 mL, 0.15 mmol). The crude product was purified using column chromatography (2-5% MeOH:DCM) to yield the title triurea as a foamy white solid (38 mg, 0.06 mmol, 56%). **M.P.** 171-172 °C (Et_2O/PE). **TLC** – R_f = 0.45 (SiO_2, EtOAc). **^{1}H NMR** (400 MHz, $CDCl_3$) δ_H 0.85 (t, J = 7.8, 3H, C*H*$_3$), 1.20-1.22 (m, 2H, C*H*$_2$), 1.37-1.40 (m, 2H, C*H*$_2$), 3.16-3.47 (m, 10H, 5 x NC*H*$_2$), 3.76 (s, 3H, OC*H*$_3$), 3.78 (s, 3H, OC*H*$_3$), 4.38 (s, 2H, C*H*$_2$Ar), 4.57 (s, 2H, C*H*$_2$Ar), 6.81 (d, J = 9.0, 2H, 2 x Ar*H*), 6.84 (d, J = 9.0, 2H, 2 x Ar*H*), 7.12 (d, J = 7.5, 2H, 2 x Ar*H*), 7.18-7.33 (m, 10H, 10 x Ar*H*), 7.38 (s, 1H, N*H*), 7.53 (d, J = 8.9, 2H, 2 x Ar*H*), 8.84 (s, 1H, N*H*). **^{13}C NMR** (101 MHz, $CDCl_3$) δ_C 13.9 (*C*H$_3$), 20.0 (*C*H$_2$), 32.2 (*C*H$_2$), 40.7 (N*C*H$_2$), 46.7 (N*C*H$_2$), 47.6 (N*C*H$_2$), 48.4 (N*C*H$_2$), 49.9 (N*C*H$_2$), 51.9 (*C*H$_2$Ar), 52.7 (*C*H$_2$Ar), 55.7 (2 x O*C*H$_3$), 114.0 (4 x Ar*C*), 121.1 (2 x Ar*C*), 121.6 (2 x Ar*C*), 126.6 (2 x Ar*C*), 127.6 (2 x Ar*C*), 128.0 (2 x Ar*C*), 128.9 (2 x Ar*C*), 129.2 (2 x Ar*C*), 133.0 (Ar*C*), 133.4 (Ar*C*), 137.7 (Ar*C*), 138.2 (Ar*C*), 155.3 (Ar*C*), 155.4 (Ar*C*), 156.4 (*C*O), 156.6 (*C*O), 158.9 (*C*O). **HR-MS** (ESI, positive ion mode) – *m/z* for $[C_{39}H_{48}N_6O_5+Na]^+$ = 703.3584. Found 703.3575. **FTIR** (neat) – 2960, 2932, 1644, 1607.

4,7-Bis(*tert*-butoxycarbonyl)-1,4,7,10-tetraazadecane, 3f-1

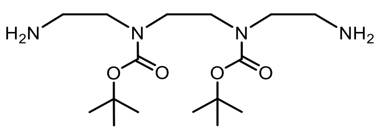

Synthesised according to general procedure F using triethylenetetramine (2.6 mL, 17.1 mmol). The crude product was then reacted according to general procedure D. The title carbamate was isolated as a pale yellow oil (4.1 g, 11.9 mmol, 67%). **TLC** – R_f = 0.04 (SiO_2, 5:95:1 MeOH:DCM:NH_3). Spectroscopic data matched that previously reported.[3]

1-(Naphthalen-1-ylmethyl)-4,7-bis(*tert*-butoxycarbonyl)-1,4,7,10-tetraazadecane, 3f-2

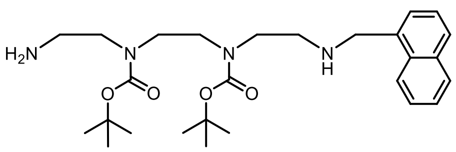

Synthesised according to general procedure J using **3f-1** (4.20 g, 12.10 mmol) and 1-naphthaldehyde (1.68 mL, 12.10 mmol, 1.00 equiv). The crude product was purified by column chromatography (1-10% MeOH:DCM, 1% NH_4OH) to yield the title amine as a foamy white solid (2.90 g, 6.0 mmol, 50%). **M.P.** 42-43 °C ($CHCl_3$). **TLC** – R_f = 0.09 (SiO_2, 5:95:1 MeOH:DCM:NH_4OH). **^{1}H NMR** (400 MHz, $CDCl_3$) δ_H 1.43 (s, 9H, C(C*H*$_3$)$_3$), 1.45 (s, 9H, C(C*H*$_3$)$_3$), 2.78 (t, J = 6.2, 2H, NC*H*$_2$), 2.88 (t, J = 6.2, 2H, NC*H*$_2$), 3.17-3.46 (m, 8H, 4 x NC*H*$_2$), 4.24 (s, 2H, C*H*$_2$Ar), 7.37-7.53 (m, 4H, 4 x Ar*H*), 7.75 (d, J = 8.1, 1H, Ar*H*), 7.84 (d, J = 7.5, 1H, Ar*H*), 8.11-8.14 (m, 1H, Ar*H*). **^{13}C NMR** (101 MHz, $CDCl_3$) δ_C 28.5 (2 x C(*C*H$_3$)$_3$), 40.6 (N*C*H$_2$, rot.), 40.9 (N*C*H$_2$, rot.), 45.7 (N*C*H$_2$), 46.0 (N*C*H$_2$), 47.7 (N*C*H$_2$), 48.4 (N*C*H$_2$), 51.2 (*C*H$_2$Ar), 51.6 (N*C*H$_2$), 79.5 (*C*(CH$_3$)$_3$), 79.9 (*C*(CH$_3$)$_3$), 123.8 (Ar*C*), 125.3 (Ar*C*), 125.6 (Ar*C*), 126.0 (2 x Ar*C*), 127.7 (Ar*C*), 128.6 (Ar*C*), 131.8 (Ar*C*), 133.9 (Ar*C*), 135.9 (Ar*C*), 155.4 (*C*O), 155.7 (*C*O). **HR-MS** (ESI, positive ion mode) – *m/z* for $[C_{27}H_{43}N_4O_4+H]^+$ = 487.3284. Found 487.3279. **FTIR** (neat) – 3401, 2946, 1687.

1-(Naphthalen-1-ylmethyl)-1,4,7-tris(4-methoxyanilinylcarbonyl)-10-trifluoroacetyl-1,4,7,10-tetraazadecane, 3f-3

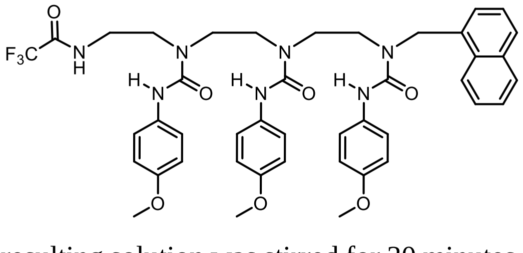

Step 1 (*trifluoroacetylation*): Under a dry, inert atmosphere, a solution of **3f-2** (1.5 g, 5.4 mmol, 1.00 equiv, 1.00 M) in anhydrous DCM (5.4 mL) was cooled to 0 °C. Ethyl trifluoroacetate (0.70 mL, 5.9 mmol, 1.10 equiv) was added dropwise over 30 minutes and the resulting solution was stirred for 30 minutes. The solution was warmed to room temperature and stirred for a further hour. The solution was then diluted with saturated aqueous $NaHCO_3$ (50 mL) and DCM (50 mL). The organic phase was washed with deionised water, dried ($MgSO_4$), filtered and concentrated *in vacuo* to give the crude trifluoroacetamide. ***Step 2*** (*Boc-deprotection*): Under a dry, inert atmosphere, the crude product was dissolved in anhydrous DCM (43 mL) and TFA (11 mL) was added dropwise.

The resulting solution was stirred for 16 h and concentrated *in vacuo*. The crude product was diluted with saturated aqueous $NaHCO_3$ (100 mL) and extracted with DCM (3 x 50 mL). The combined organic extracts were dried ($MgSO_4$), filtered and concentrated *in vacuo* to give the crude triamine. ***Step 3*** (*urea formation*): Under a dry, inert atmosphere, the residue was dissolved in anhydrous DCM (55 mL) and cooled to 0 °C. Anhydrous TEA (6.7 mL, 48.6 mmol, 9.00 equiv) was added in one portion and 4-methoxyphenyl isocyanate (3.2 mL, 24.3 mmol, 4.50 equiv) was added dropwise to give a colourless solution. The solution was stirred for 16 h then concentrated *in vacuo* and purified by column chromatography (1-10% MeOH:DCM) to yield the title triurea as a foamy white solid (403 mg, 0.5 mmol, 9%). **M.P.** 140-141 °C ($CHCl_3$). **TLC** – R_f = 0.19 (SiO_2, 5:95 MeOH:DCM). **^{1}H NMR** (400 MHz, $CDCl_3$) δ_H 2.89-3.57 (m, 12H, 6 x NCH_2), 3.70 (s, 3H, OCH_3), 3.71 (s, 3H, OCH_3), 3.74 (s, 3H, OCH_3), 5.04 (s, 2H, CH_2Ar), 5.75 (s, 1H, N*H*), 6.66-6.86 (m, 6H, 6 x Ar*H*), 7.03 (d, J = 9.0, 2H, 2 x Ar*H*), 7.35-7.60 (m, 8H, 8 x Ar*H*), 7.76 (d, J = 7.7, 1H, Ar*H*), 7.81 (d, J = 7.7, 1H, Ar*H*), 8.00 (d, J = 7.7, 1H, Ar*H*), 8.57 (s, 1H, N*H*), 8.88 (s, 1H, N*H*). **^{13}C NMR** (101 MHz, $CDCl_3$) δ_C 39.7 (N*C*H_2), 45.8 (N*C*H_2), 46.3 (N*C*H_2), 47.5 (N*C*H_2), 47.8 (N*C*H_2), 48.4 (N*C*H_2), 49.7 (*C*H_2Ar), 55.5 (3 x O*C*H_3), 114.1 (2 x Ar*C*), 114.2 (2 x Ar*C*), 114.4 (2 x Ar*C*), 117.6 (q, J = 287.2, *C*F_3), 121.3 (2 x Ar*C*), 121.7 (2 x Ar*C*), 122.3 (2 x Ar*C*), 125.4 (Ar*C*), 126.3 (Ar*C*), 126.8 (Ar*C*), 128.3 (Ar*C*), 128.6 (Ar*C*), 128.9 (Ar*C*), 129.3 (Ar*C*), 131.3 (Ar*C*), 132.7 (Ar*C*), 132.9 (Ar*C*), 133.1 (Ar*C*), 133.2 (Ar*C*), 133.9 (Ar*C*), 155.3 (Ar*C*), 155.4 (Ar*C*), 155.6 (Ar*C*), 156.2 (q, J = 36.2, *C*O), 156.7 (*C*O), 157.0 (*C*O). **^{19}F NMR** (377 MHz, $CDCl_3$) δ_F −72.3 (C*F*$_3$). **HR-MS** (MALDI) – *m/z* for $[C_{43}H_{46}F_3N_7O_7{+}Na]^+$ = 852.3309. Found 852.3288. **FTIR** (neat) – 3309, 2976, 2923, 1648.

1-(Naphthalen-1-ylmethyl)-1,4,7-tris(4-methoxyanilinylcarbonyl)-1,4,7,10-tetraazadecane, 3f-4

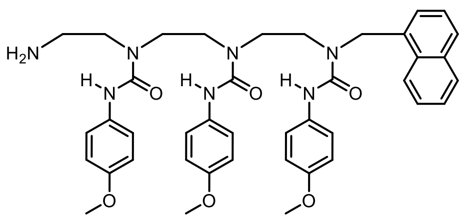

Synthesised according to general procedure D using **3f-3** (400 mg, 0.49 mmol). The title amine was isolated as a foamy white solid (342 mg, 0.47 mmol, 95%). **M.P.** 133-134 °C ($CHCl_3$). **TLC** – R_f = 0.30 (SiO_2, 5:95 MeOH:DCM). **^{1}H NMR** (400 MHz, $CDCl_3$) δ_H 1.88 (s, 2H, NH_2), 2.80 (t, J = 7.0, 2H, NCH_2), 3.01-3.26 (m, 8H, 4 x NCH_2), 3.45 (t, J = 6.3, 2H, NCH_2), 3.70 (s, 3H, OCH_3), 3.73 (s, 3H, OCH_3), 3.75 (s, 3H, OCH_3), 5.05 (s, 2H, CH_2Ar), 5.97 (s, 1H, N*H*), 6.75 (d, J = 8.9, 2H, 2 x Ar*H*), 6.77 (d, J = 8.9, 2H, 2 x Ar*H*), 6.80 (d, J = 8.9, 2H, 2 x Ar*H*), 7.03 (d, J = 8.9, 2H, 2 x Ar*H*), 7.34-7.47 (m, 4H, 4 x Ar*H*), 7.50 (d, J = 8.9, 2H, 2 x Ar*H*), 7.59 (d, J = 8.9, 2H, 2 x Ar*H*), 7.75 (d, J = 7.6, 1H, Ar*H*), 7.80 (d, J = 7.6, 1H, Ar*H*), 8.00 (d, J = 7.6, 1H, Ar*H*), 8.67 (s, 1H, N*H*), 9.01 (s, 1H, N*H*). **^{13}C NMR** (101 MHz, $CDCl_3$) δ_C 39.3 (N*C*H_2), 46.9 (N*C*H_2), 47.3 (N*C*H_2), 47.0 (N*C*H_2), 48.1 (N*C*H_2), 49.2 (N*C*H_2), 49.3 (*C*H_2Ar), 55.4 (O*C*H_3), 55.5 (O*C*H_3), 55.5 (O*C*H_3), 113.9 (2 x Ar*C*), 113.9 (2 x Ar*C*), 114.0 (2 x Ar*C*), 121.1 (2 x Ar*C*), 121.5 (2 x Ar*C*), 121.6 (2 x Ar*C*), 125.4 (Ar*C*), 125.5 (Ar*C*), 126.2

(Ar*C*), 126.7 (Ar*C*), 128.3 (Ar*C*), 128.4 (Ar*C*), 128.8 (Ar*C*), 131.2 (Ar*C*), 131.6 (Ar*C*), 131.8 (Ar*C*), 132.9 (Ar*C*), 133.1 (Ar*C*), 133.8 (Ar*C*), 155.2 (Ar*C*), 155.3 (Ar*C*), 155.6 (Ar*C*), 156.6 (*C*O), 156.6 (*C*O), 157.4 (*C*O). **HR-MS** (ESI, positive ion mode) – *m/z* for $[C_{41}H_{47}N_7O_6+H]^+$ = 734.3666. Found 734.3659. **FTIR** (neat) – 3307, 3000, 2948, 1647.

1-(Naphthalen-1-ylmethyl)-1,4,7-tris(4-methoxyanilinylcarbonyl)-7-(succinimidylethyl)-1,4,7-triazaheptane, 3f

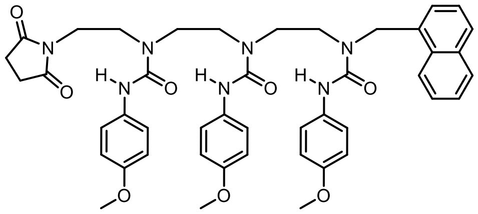

Synthesised according to general procedure E using **3f-4** (75 mg, 0.10 mmol). The crude product was purified by column chromatography (1-10% MeOH:DCM) to give the title succinimide as a foamy white solid (67 mg, 0.08 mmol, 80%). **M.P.** 152-153 °C ($CHCl_3$). **TLC** – R_f = 0.50 (SiO_2, 5:95 MeOH:DCM). **^{1}H NMR** (500 MHz, $CDCl_3$) δ_H 2.74 (s, 4H, 2 x C(O)CH_2), 3.14-3.19 (m, 2H, NCH_2), 3.24-3.29 (m, 4H, 2 x NCH_2), 3.39 (t, J = 6.4, 2H, NCH_2), 3.53 (t, J = 7.3, 2H, NCH_2), 3.68 (t, J = 7.0, 2H, NCH_2), 3.80-3.81 (m, 9H, 3 x OCH_3), 5.13 (s, 2H, CH_2Ar), 6.82-6.86 (m, 6H, 6 x Ar*H*), 7.47-7.63 (m, 10H, 10 x Ar*H*), 7.90 (d, J = 8.3, 1H, Ar*H*), 7.95 (d, J = 8.0, 1H, Ar*H*), 8.11-8.13 (m, 1H, Ar*H*), 8.69 (s, 1H, N*H*). **^{13}C NMR** (126 MHz, $CDCl_3$) δ_C 28.2 (2 x C(O)*C*H_2), 37.0 (N*C*H_2), 45.2 (N*C*H_2), 47.1 (N*C*H_2), 47.3 (N*C*H_2), 47.7 (N*C*H_2), 47.8 (N*C*H_2), 49.4 (*C*H_2Ar), 55.5 (O*C*H_3), 55.5 (O*C*H_3), 55.5 (O*C*H_3), 113.9 (2 x Ar*C*), 113.9 (2 x Ar*C*), 114.0 (2 x Ar*C*), 121.0 (2 x Ar*C*), 121.1 (2 x Ar*C*), 121.7 (2 x Ar*C*), 125.4 (2 x Ar*C*), 126.3 (2 x Ar*C*), 126.9 (3 x Ar*C*), 128.6 (Ar*C*), 128.8 (Ar*C*), 128.9 (Ar*C*), 133.0 (Ar*C*), 133.1 (Ar*C*), 133.9 (Ar*C*), 155.2 (Ar*C*), 155.5 (Ar*C*), 155.5 (Ar*C*), 155.8 (*C*O), 156.2 (*C*O), 156.3 (*C*O), 177.6 (2 x *C*O). **HR-MS** (ESI, positive ion mode) – *m/z* for $[C_{45}H_{49}N_7O_8+Na]^+$ = 838.3540. Found 838.3533. **FTIR** (neat) – 3322, 2997, 2908, 1699, 1651.

***N*-(2-Pyridyl)ethylenediamine, 3g-1**

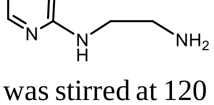

Under a dry, inert atmosphere, 2-fluoropyridine (2.0 mL, 23.4 mmol, 1.00 equiv, 1.0 M) was added to freshly distilled ethylenediamine (23.4 mL) and the solution was stirred at 120 °C for 16 h. Upon cooling, the solution was diluted with EtOAc (100 mL) and washed with distilled water (5 x 50 mL). The organic layer was washed with brine, dried ($MgSO_4$), filtered and concentrated *in vacuo* to give the title diamine as yellow powder (2664 mg, 19.4 mmol, 83%). Spectroscopic data matched that previously reported.[4]

1-(2-Pyridyl)-7-(*tert*-butoxycarbonyl)-1,4,7-triazaheptane, 3g-2

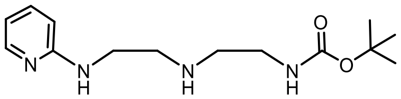

To a solution of **3g-1** (918.0 mg, 6.69 mmol, 1.0 equiv) in lab grade 1,2-dichloroethane (20.0 mL) at room temperature under air was added solid *N*-Boc-2-aminoacetaldehyde (1.065 g, 6.69 mmol, 1.0 equiv). A further aliquot of 1,2-dichloroethane (2.3 mL) was used to rinse residual *N*-Boc-2-aminoacetaldehyde into the reaction vessel. Solid $NaBH(OAc)_3$ (1.986 g, 9.37 mmol, 1.4 equiv) was added and the suspension was stirred at room temperature for 20 h. Aqueous NaOH (10 mL, 2.0 M), water (20 mL) and brine (10 mL) were added and the organic layer was separated. The product was further extracted with CH_2Cl_2 (2 × 25 mL) then the combined organic extracts were washed with saturated $NaHCO_3$/brine (1:1, 50 mL), dried (Na_2SO_4) and concentrated. Flash chromatography (manual, 35.8 g silica, MeOH/[35% aqueous NH_3]/EtOAc* gradient from 5:1:94 to 7.5:1:91.5) gave **3g-2** (837.9 mg, 45%) as a pale yellow gum that eventually solidified upon storage. [*This eluent combination should be prepared freshly before chromatography. Solutions stored for long periods (weeks/months) were found to contain non-volatile acetamide as an impurity.] **M.P.** 72-73 °C ($CHCl_3$). **TLC** – R_f = 0.09 (SiO_2, 5:95 MeOH:DCM). **^{1}H NMR** (400 MHz, $CDCl_3$) δ_H 1.44 (s, 9H, C(C*H*$_3$)$_3$), 2.76 (t, J = 5.8, 2H, NC*H*$_2$), 2.88 (t, J = 5.8, 2H, NC*H*$_2$), 3.22 (q, J = 5.8, 2H, NC*H*$_2$), 3.34 (q, J = 5.8, 2H, NC*H*$_2$), 4.85 (s, 1H, N*H*), 4.92 (s, 1H, N*H*), 6.41 (d, J = 8.5, 1H, Ar*H*), 6.56 (t, J = 6.0, 1H, Ar*H*), 7.40 (t, J = 7.6, 1H, Ar*H*), 8.08 (d, J = 4.9, 1H, Ar*H*). **^{13}C NMR** (101 MHz, $CDCl_3$) δ_C 28.6 (C(*C*H$_3$)$_3$), 40.5 (N*C*H$_2$), 41.9 (N*C*H$_2$), 48.7 (N*C*H$_2$), 49.1 (N*C*H$_2$), 79.2 (*C*(CH$_3$)$_3$), 107.4 (Ar*C*), 113.0 (Ar*C*), 137.5 (Ar*C*), 148.3 (Ar*C*), 156.5 (*C*O), 158.5 (Ar*C*). **HR-MS** (ESI, positive ion mode) – *m/z* for $[C_{14}H_{26}N_4O+Na]^+$ = 303.1797. Found 303.1807. **FTIR** (neat) – 3356, 2991, 2894, 1695.

1-(2-Pyridyl)-1,4-bis(4-methoxyanilinylcarbonyl)-7-(*tert*-butoxycarbonyl)-1,4,7-triazaheptane, 3g-3

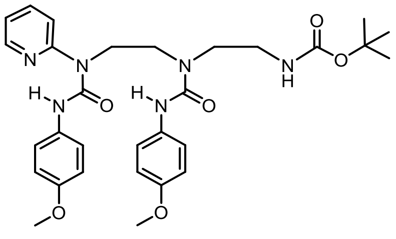

Synthesised according to general procedure A using **3g-2** (163 mg, 0.58 mmol) and 4-methoxyphenyl isocyanate (0.23 mL, 1.74 mmol). The crude product was purified by column chromatography (12-100% EtOAc:PE) to yield the title diurea as a foamy white solid (114 mg, 0.20 mmol, 34%). **M.P.** 119-120 °C ($CHCl_3$). **TLC** – R_f = 0.48 (SiO_2, 5:95 MeOH:DCM). **^{1}H NMR** (500 MHz, CD_2Cl_2) δ_H 1.44 (s, 9H, C(C*H*$_3$)$_3$), 3.32 (q, J = 6.3, 2H, NC*H*$_2$), 3.54 (t, J = 6.6, 2H, NC*H*$_2$), 3.64 (t, J = 6.6, 2H, NC*H*$_2$), 3.78-3.79 (m, 6H, 2 x OC*H*$_3$), 4.10 (t, J = 6.3, 2H, NC*H*$_2$), 5.31 (t, J = 5.3, 1H, N*H*), 6.86 (d, J = 9.0, 2H, 2 x Ar*H*), 6.89 (d, J = 9.0, 2H, 2 x Ar*H*), 7.04 (dd, J = 5.1, 7.2, 1H, Ar*H*), 7.42 (s, 1H, Ar*H*), 7.50 (d, J = 9.0, 2H, 2 x Ar*H*), 7.56 (d, J = 9.0, 2H, 2 x Ar*H*), 7.82 (ddd, J = 8.0, 7.5, 1.9, 1H, Ar*H*), 8.34 (dd, J = 4.9, 1.5, 1H, Ar*H*), 8.68 (s, 1H, N*H*), 12.48 (s, 1H, N*H*). **^{13}C NMR** (126 MHz, CD_2Cl_2) δ_C 28.7 (C(*C*H$_3$)$_3$), 41.0 (N*C*H$_2$), 45.2

(NCH_2), 46.7 (NCH_2), 48.3 (NCH_2), 56.0 (2 x OCH_3), 80.0 (C(CH_3)$_3$), 113.0 (Ar*C*), 114.3-114.5 (4 x Ar*C*), 118.4 (Ar*C*), 121.8 (2 x Ar*C*), 122.6 (2 x Ar*C*), 132.5 (Ar*C*), 134.1 (Ar*C*), 140.0 (Ar*C*), 146.4 (Ar*C*), 154.9 (Ar*C*), 155.8 (Ar*C*), 155.9 (*C*O), 156.5 (*C*O), 156.8 (*C*O). **HR-MS** (ESI, positive ion mode) – *m/z* for $[C_{30}H_{38}N_6O_6+H]^+$ = 579.2931. Found 579.2895. **FTIR** (neat) – 3497, 2990, 2943, 1694, 1660.

1-(2-Pyridyl)-1,4-bis(4-methoxyanilinylcarbonyl)-1,4,7-triazaheptane, 3g-4

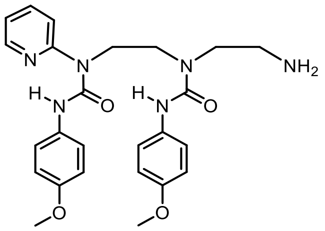

Synthesised according to general procedure H using **3g-3** (200 mg, 0.34 mmol). The title amine was isolated as a foamy white solid (163 mg, 0.34 mmol, >99%). **M.P.** 139-140 °C ($CHCl_3$). **TLC** – R_f = 0.09 (SiO_2, 90:10 EtOAc:PE). **^{1}H NMR** (400 MHz, $CDCl_3$) δ_H 3.00 (t, J = 4.9, 2H, NCH_2), 3.51 (t, J = 4.9, 2H, NCH_2), 3.61 (t, J = 7.2, 2H, NCH_2), 3.77 (s, 3H, OCH_3), 3.79 (s, 3H, OCH_3), 4.17 (t, J = 7.2, 2H, NCH_2), 6.84 (d, J = 8.9, 2H, 2 x Ar*H*), 6.88 (d, J = 8.9, 2H, 2 x Ar*H*), 6.98 (dd, J = 5.0, 6.8, 1H, Ar*H*), 7.35 (d, J = 8.9, 2H, NCH_2), 7.45 (d, J = 8.9, 2H, NCH_2), 7.67 (d, J = 7.7, 1H, Ar*H*), 7.78 (ddd, J = 1.8, 5.8, 7.7, 1H, Ar*H*), 8.28 (d, J = 5.0, 1H, Ar*H*), 9.75 (s, 1H, N*H*), 12.61 (s, 1H, N*H*). **^{13}C NMR** (101 MHz, $CDCl_3$) δ_C 41.8 (NCH_2), 43.6 (NCH_2), 46.1 (NCH_2), 52.1 (NCH_2), 55.5 (2 x OCH_3), 112.7 (Ar*C*), 114.1 (4 x Ar*C*), 117.5 (Ar*C*), 121.3 (2 x Ar*C*), 122.5 (2 x Ar*C*), 131.9 (Ar*C*), 133.4 (Ar*C*), 139.6 (Ar*C*), 145.3 (Ar*C*), 154.4 (*C*O), 155.1 (Ar*C*), 155.4 (Ar*C*), 158.0 (Ar*C*). **HR-MS** (ESI, positive ion mode) – *m/z* for $[C_{25}H_{30}N_6O_4+H]^+$ = 479.2407. Found 479.2380. **FTIR** (neat) – 3329, 3130, 2992, 2947, 1659.

1-(2-Pyridyl)-1,4-bis(4-methoxyanilinylcarbonyl)-7-benzyl-1,4,7-triazaheptane, 3g-5

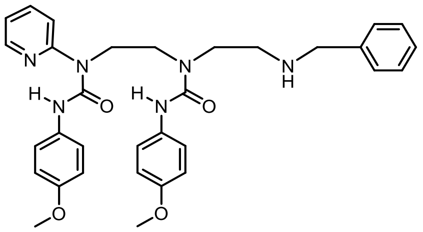

Synthesised according to general procedure B using **3g-4** (140 mg, 0.29 mmol) and benzaldehyde (0.05 mL, 0.44 mmol). The crude product was purified by column chromatography (3-10% MeOH:DCM) to yield the title triamine as a foamy white solid (87 mg, 0.15 mmol, 53%). **M.P.** 142-143 °C ($CHCl_3$). **TLC** – R_f = 0.48 (SiO_2, 10:90 MeOH:DCM). **^{1}H NMR** (500 MHz, $CDCl_3$) δ_H 2.03 (s, 1H, N*H*), 2.95 (t, J = 4.9, 2H, NCH_2), 3.56 (t, J = 4.9, 2H, NCH_2), 3.60 (t, J = 8.2, 2H, NCH_2), 3.78 (s, 3H, OCH_3), 3.80 (s, 3H, OCH_3), 3.84 (s, 2H, CH_2Ar), 4.15 (t, J = 8.2, 2H, NCH_2), 6.80 (d, J = 9.0, 2H, 2 x Ar*H*), 6.88 (d, J = 9.0, 2H, 2 x Ar*H*), 6.98 (dd, J = 5.0, 7.2, 1H, Ar*H*), 7.22 (d, J = 9.0 2H, 2 x Ar*H*), 7.25-7.34 (m, 5H, 5 x Ar*H*), 7.46 (d, J = 9.0, 2H, 2 x Ar*H*), 7.66-7.70 (m, 1H, Ar*H*), 7.74 (ddd, J = 1.8, 7.4, 8.6, 1H, Ar*H*), 8.27 (d, J = 5.0, 1H, Ar*H*), 9.70 (s, 1H, N*H*), 12.61 (s, 1H, N*H*). **^{13}C NMR** (126 MHz, $CDCl_3$) δ_C 43.5 (NCH_2), 46.2 (NCH_2), 49.6 (NCH_2), 50.0 (NCH_2), 54.3 (CH_2Ar), 55.5 (2 x OCH_3), 112.8 (Ar*C*), 114.0

(2 x Ar*C*), 114.1 (2 x Ar*C*) 117.4 (Ar*C*), 121.5 (2 x Ar*C*), 122.4 (2 x Ar*C*), 127.5 (Ar*C*), 128.3 (2 x Ar*C*), 128.7 (2 x Ar*C*), 132.0 (Ar*C*), 133.3 (Ar*C*), 138.9 (Ar*C*), 139.6 (Ar*C*), 145.3 (Ar*C*), 154.3 (Ar*C*), 155.1 (Ar*C*), 155.4 (*C*O), 155.9 (Ar*C*), 158.0 (*C*O). **HR-MS** (ESI, positive ion mode) – *m/z* for $[C_{32}H_{36}N_6O_4+H]^+$ = 569.2876. Found 569.2875. **FTIR** (neat) – 3326, 2993, 2950, 1652.

1-(2-Pyridyl)-1,4,7-tris(4-methoxyanilinylcarbonyl)-7-benzyl-1,4,7-triazaheptane, 3g

Synthesised according to general procedure A using **3g-5** (62 mg, 0.11 mmol) and 4-methoxyphenyl

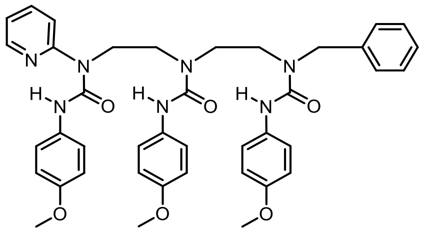

isocyanate (0.02 mL, 0.17 mmol) dropwise. The crude product was purified by column chromatography (1-2% MeOH:DCM) to yield the title triurea as a foamy white solid (47 mg, 0.07 mmol, 60%). **M.P.** 200-201 °C (DCM). **TLC** – R_f = 0.26 (SiO_2, 1:99 MeOH:DCM). **^{1}H NMR** (500 MHz, $CDCl_3$) δ_H 3.32-3.37 (m, 2H, NC*H*$_2$), 3.51 (t, J = 8.0, 2H, NC*H*$_2$), 3.54 (t, J = 8.2, 2H, NC*H*$_2$), 3.76 (s, 3H, OC*H*$_3$), 3.78 (s, 3H, OC*H*$_3$), 3.79 (s, 3H, OC*H*$_3$), 3.98 (t, J = 8.0, 2H, NC*H*$_2$), 4.63 (s, 2H, C*H*$_2$Ar), 6.82 (d, J = 9.0, 2H, 2 x Ar*H*), 6.86 (d, J = 9.0, 2H, 2 x Ar*H*), 6.88 (d, J = 9.0, 2H, 2 x Ar*H*), 7.08 (dd, J = 5.0, 7.2, 1H, Ar*H*), 7.30-7.40 (m, 6H, 6 x Ar*H*), 7.48 (d, J = 9.0, 2H, 2 x Ar*H*), 7.51-7.53 (m, 2H, 2 x Ar*H*), 7.61 (d, J = 9.0, 2H, 2 x Ar*H*), 7.82 (ddd, J = 1.9, 7.4, 8.7, 1H, Ar*H*), 8.36 (dd, J = 1.6, 5.0, 1H, Ar*H*), 8.95 (s, 1H, N*H*), 12.34 (s, 1H, N*H*). **^{13}C NMR** (126 MHz, $CDCl_3$) δ_C 45.2 (N*C*H$_2$), 47.0 (2 x N*C*H$_2$), 48.5 (N*C*H$_2$), 51.2 (*C*H$_2$Ar), 55.4 (3 x O*C*H$_3$), 112.0 (Ar*C*), 113.7 (2 x Ar*C*), 113.8 (2 x Ar*C*), 114.0 (2 x Ar*C*), 118.1 (Ar*C*), 121.1 (2 x Ar*C*), 121.3 (2 x Ar*C*), 122.0 (2 x Ar*C*), 127.3 (Ar*C*), 127.6 (2 x Ar*C*), 128.7 (2 x Ar*C*), 131.7 (Ar*C*), 133.4 (2 x Ar*C*), 139.5 (2 x Ar*C*), 146.1 (Ar*C*), 154.4 (*C*O), 155.1 (Ar*C*), 155.2 (Ar*C*), 155.3 (Ar*C*), 156.0 (*C*O), 156.3 (*C*O). **HR-MS** (ESI, positive ion mode) – *m/z* for $[C_{40}H_{43}N_7O_6+H]^+$ = 718.3353. Found 718.3348. **FTIR** (neat) – 3307, 3185, 3051, 2981, 2875, 1660, 1607.

1-(2-Pyridyl)-1,4-bis(4-bromoanilinylcarbonyl)-7-(*tert*-butoxycarbonyl)-1,4,7-triazanonane, 3h-1

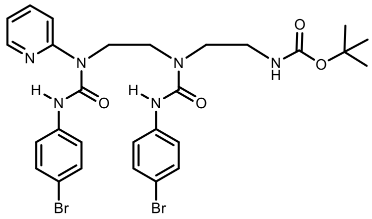

To a solution of **3g-2** (146.9 mg, 0.52 mmol, 1.0 equiv) in lab grade 1,2-dichloroethane (3.5 mL) at room temperature under air was added solid 4-bromophenyl isocyanate (212.7 mg, 1.07 mmol, 2.05 equiv). The mixture was heated to 40 °C (closed system: 7 mL vial with cap) and maintained at this temperature for 64 h. While still at 40 °C, MeOH (~2.5 mL) was added and the mixture was stirred for a further 20 min to quench any unreacted isocyanate. The mixture was then concentrated *in vacuo*. Flash chromatography (Biotage, 10 g SNAP Ultra column, MeOH/CH_2Cl_2 gradient from 0.2:99.8 to 2.5:97.5) gave **3h-1** (274.1 mg, 77%) as a white

solid. **TLC** – R_f = 0.48 (2.5:97.5 MeOH/CH_2Cl_2). **^{1}H NMR** (400 MHz, $CDCl_3$) δ_H 1.45 (s, 9H), 3.32 – 3.38 (m, 2H), 3.56 (t, *J* = 7.1 Hz, 2H), 3.62 – 3.69 (m, 2H), 4.07 – 4.12 (m, 2H), 5.07 (d, *J* = 5.1 Hz, 1H), 7.05 (dd, *J* = 7.4, 5.0 Hz, 1H), 7.38 – 7.49 (m, 7H), 7.61 (d, *J* = 8.9 Hz, 2H), 7.83 (ddd, *J* = 9.0, 7.3, 2.0 Hz, 1H), 8.32 (dd, *J* = 5.1, 1.9 Hz, 1H), 8.85 (s, 1H), 12.90 (s, 1H). **^{13}C NMR** (101 MHz, $CDCl_3$) δ_C 28.5, 40.4, 44.5, 46.3, 47.8, 80.4, 112.6, 115.0, 116.1, 118.2, 121.2, 122.1, 131.7, 132.0, 138.0, 139.4, 140.0, 145.7, 154.1, 155.1, 155.9, 157.2. **HR-MS** (ESI, positive ion mode) – *m/z* for $[C_{28}H_{32}(^{79}Br)_2N_6O_4+H]^+$ = 675.0925. Found 675.0940.

1-(2-Pyridyl)-1,4-bis(4-bromoanilinylcarbonyl)-1,4,7-triazanonane, 3h-2

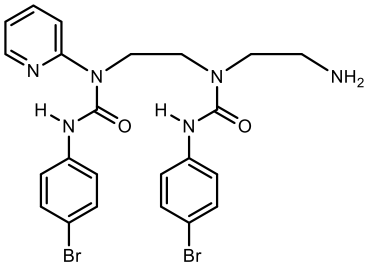

To a solution of **3h-1** (268.1 mg, 0.40 mmol, 1.0 equiv) in lab grade CH_2Cl_2 (2.0 mL) at room temperature under air was added TFA (0.46 mL, 5.95 mmol, 15.0 equiv) and the mixture was stirred at room temperature for 4 h. The mixture was diluted with CH_2Cl_2 (20 mL) and washed with aqueous K_2CO_3 (20 mL, 1.0 M). The aqueous phase was back-extracted with CH_2Cl_2 (15 mL). The combined organic extracts were dried (Na_2SO_4) and concentrated to give **3h-2** (214.6 mg, 94%) as a yellow solid. **TLC** – R_f = 0.20 (10:90 MeOH/CH_2Cl_2). **^{1}H NMR** (400 MHz, $CDCl_3$) δ_H 1.69 (bs, 2H), 2.95 – 3.08 (m, 2H), 3.45 – 3.66 (m, 4H), 4.16 (t, *J* = 7.9 Hz, 2H), 7.02 (t, *J* = 6.3 Hz, 1H), 7.28 – 7.53 (m, 8H), 7.61 – 7.87 (m, 2H), 8.27 – 8.32 (m, 1H), 10.31 (bs, 1H), 12.94 (s, 1H). **^{13}C NMR** (101 MHz, $CDCl_3$) δ_C 41.9, 43.5, 46.2, 52.3, 112.9, 114.5, 115.8, 117.9, 120.9, 122.1, 131.7, 131.9, 138.2, 139.6, 139.9, 145.4, 154.0, 155.2, 157.6. **HR-MS** (ESI, positive ion mode) – *m/z* calcd for $[C_{23}H_{24}(^{79}Br)_2N_6O_2+H]^+$ = 575.0400. Found 575.0414.

1-(2-Pyridyl)-1,4-bis(4-bromoanilinylcarbonyl)-7-(pyren-1-ylmethyl)-1,4,7-triazanonane, 3h-3

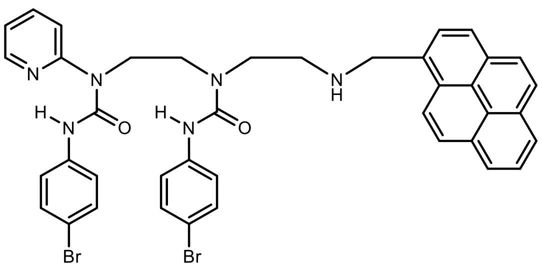

To a round-bottomed flask was added **3h-2** (39.9 mg, 0.069 mmol, 1.0 equiv) and 1-pyrenecarboxaldehyde (15.9 mg, 0.069 mmol, 1.0 equiv). After purging with N_2, lab grade 1,2-dichloroethane (1.4 mL) was added and the mixture was stirred at room temperature for 2 min until most solids had dissolved. The septum was briefly removed and solid $NaBH(OAc)_3$ (20.5 mg, 0.097 mmol, 1.4 equiv) was added. The mixture was stirred at room temperature for 5 h. Aqueous K_2CO_3 (10 mL, 1.0 M) was added and the product was extracted with CH_2Cl_2 (25 mL + 10 mL) then the combined organic extracts were dried (Na_2SO_4) and concentrated. Flash chromatography (Biotage, 5 g Zip Sphere column, MeOH/[35% aqueous

NH_3]/EtOAc* gradient from 0.6:0.12:99.28 to 5:1:94) gave **3h-3** (27.2 mg, 50%) as a white solid. [*This eluent combination should be prepared freshly before chromatography. Solutions stored for long periods (weeks/months) were found to contain non-volatile acetamide as an impurity.] **TLC** – R_f = 0.33 (4:0.8:95.2 MeOH/[35% aqueous NH_3]/EtOAc). **^{1}H NMR** (400 MHz, $CDCl_3$) δ_H 3.13 (t, *J* = 4.5 Hz, 2H), 3.52 – 3.64 (m, 4H), 4.03 – 4.13 (m, 2H), 4.54 (s, 2H), 6.81 – 7.09 (m, 4H), 7.35 – 7.70 (broad m, 2H), 7.41 – 7.48 (m, 4H), 7.92 (d, *J* = 7.8 Hz, 1H), 7.98 – 8.29 (m, 10H), 9.89 (bs, 1H), 12.89 (s, 1H). **^{13}C NMR** (101 MHz, $CDCl_3$) δ (33 resolved resonances observed out of 36 theoretical) 43.7, 46.4, 50.4, 52.4, 112.7, 114.5, 115.9, 117.8, 121.0, 122.1, 122.7, 124.9, 125.2, 125.4, 125.6, 126.3, 127.4, 127.5, 127.7, 128.4, 129.1, 130.8, 131.2, 131.3, 131.4, 131.9, 132.4, 138.2, 139.2, 139.7, 145.3, 154.0, 155.1. **HR-MS** (MALDI, positive ion mode) – *m/z* for $[C_{40}H_{34}(^{79}Br)_2N_6O_2+H]^+$ = 789.1183. Found 789.1199.

1-(2-Pyridyl)-1,4-bis(4-bromoanilinylcarbonyl)-7-(4-nitroanilinylcarbonyl)-7-(pyren-1-ylmethyl)-1,4,7-triazanonane, 3h

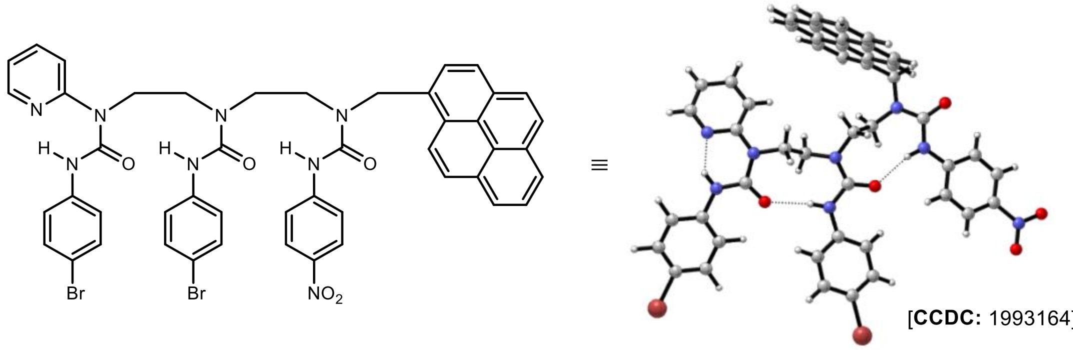

[**CCDC**: 1993164]

To a solution of **3h-3** (59.2 mg, 0.075 mmol, 1.0 equiv) in lab grade CH_2Cl_2 (0.5 mL) at room temperature under air was added a solution/suspension of 4-nitrophenyl isocyanate (12.3 mg, 0.075 mmol, 1.0 equiv) in CH_2Cl_2 (1.0 mL). The suspension was stirred at room temperature for 4.5 h, then stood in a freezer arbitrarily for 10 days. The precipitated product, which is sparingly soluble in CH_2Cl_2, was collected by filtration and the reaction vial and precipitate were rinsed/washed with CH_2Cl_2 (2 × 6 mL). The precipitate was air-dried in a fume hood to give **3h** (48.1 mg, 67%) as a pale yellow solid. **TLC** – R_f = 0.31 (CH_2Cl_2) with tailing due to poor solubility. **^{1}H NMR** (400 MHz, DMF-d_7) δ_H 3.68 – 3.85 (m, 6H), 4.17 (t, *J* = 7.4 Hz, 2H), 5.56 (s, 2H), 7.19 (dd, *J* = 7.3, 5.0 Hz, 1H), 7.49 – 7.54 (m, 3H), 7.58 (d, *J* = 8.8 Hz, 2H), 7.64 (d, *J* = 8.9 Hz, 2H), 7.77 (d, *J* = 8.9 Hz, 2H), 7.85 – 7.90 (m, 1H), 8.04 – 8.15 (m, 4H), 8.20 – 8.39 (m, 8H), 8.43 – 8.49 (m, 2H), 9.25 (s, 1H), 9.87 (s, 1H), 12.06 (s, 1H). **^{13}C NMR** (126 MHz, DMF-d_7) δ_C (44 resolved resonances observed out of 41 theoretical; a minor unidentified aromatic impurity was present) 45.1, 45.9, 46.9, 47.5, 48.9, 114.2, 115.0, 115.0, 118.5, 119.0, 119.2, 121.6, 122.1, 123.2, 124.7, 125.0, 125.0, 125.3, 125.3, 125.7, 125.8, 126.7, 127.5, 127.9, 128.1, 128.8, 131.0, 131.1, 131.6, 131.8, 131.9, 139.2, 139.8, 140.4, 141.9, 142.3, 146.6, 147.0, 148.0, 152.5, 154.4, 155.2, 155.7, 156.3. **HR-MS** (MALDI, positive ion mode) – *m/z* for $[C_{47}H_{38}(^{79}Br)_2N_8O_5+Na]^+$ = 975.1224. Found 975.1239. Crystals of **3h** suitable for X-ray

crystallography were obtained by diffusion of toluene into a solution of **3h** in DMF/1,2-DCE (CCDC: 1993164).

1,10-Bis(naphthalen-1-ylmethyl)-1,4,7-tris(4-methoxyanilinylcarbonyl)-1,4,7,10-tetraazadecane, 5a-1

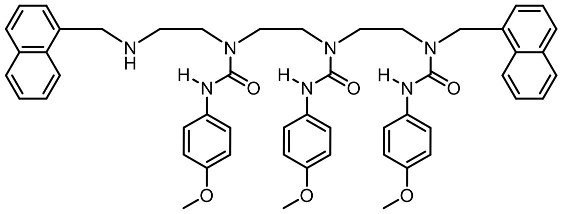

Synthesised according to general procedure B using **3f-4** (175 mg, 0.24 mmol) and 1-naphthaldehyde (0.05 mL, 0.36 mmol). The crude product was purified by column chromatography (2-10% MeOH:DCM) to give the title amine as a foamy white solid (132 mg, 0.15 mmol, 63%). **M.P.** 149-150 °C ($CHCl_3$). **TLC** – R_f = 0.44 (SiO_2, 5:95 MeOH:DCM). **^{1}H NMR** (400 MHz, $CDCl_3$) δ_H 2.90 (t, J = 4.6, 2H, NCH_2), 2.94-2.98 (m, 2H, NCH_2), 3.08-3.17 (m, 4H, 2 x NCH_2), 3.26-3.29 (m, 2H, NCH_2), 3.45 (t, J = 7.6, 2H, NCH_2), 3.74 (s, 3H, OCH_3), 3.76 (s, 3H, OCH_3), 3.77 (s, 3H, OCH_3), 4.28 (s, 2H, CH_2Ar), 5.10 (s, 2H, CH_2Ar), 6.64 (d, J = 9.0, 2H, 2 x Ar*H*), 6.77 (d, J = 9.0, 2H, 2 x Ar*H*), 6.83 (d, J = 9.0, 2H, 2 x Ar*H*), 6.91 (d, J = 8.0, 2H, 2 x Ar*H*), 7.36-7.60 (m, 8H, 8 x Ar*H*), 7.77-7.83 (m, 2H, 2 x Ar*H*), 7.86-7.89 (m, 2H, 2 x Ar*H*), 8.00 (d, J = 8.0, 1H, Ar*H*), 8.13 (d, J = 8.0, 1H, Ar*H*), 8.48 (s, 1H, N*H*), 8.71 (s, 1H, N*H*), 9.59 (s, 1H, N*H*). **^{13}C NMR** (101 MHz, $CDCl_3$) δ_C 45.4 (N*C*H_2), 46.8 (N*C*H_2), 46.9 (N*C*H_2), 47.8 (N*C*H_2), 49.7 (N*C*H_2), 51.1 (N*C*H_2), 51.8 (*C*H_2Ar), 53.3 (*C*H_2Ar), 55.5 (3 x O*C*H_3), 113.8 (2 x Ar*C*), 113.9 (2 x Ar*C*), 114.0 (2 x Ar*C*), 120.9 (2 x Ar*C*), 121.0 (2 x Ar*C*), 121.5 (2 x Ar*C*), 123.1 (Ar*C*), 123.7 (Ar*C*), 125.3 (Ar*C*), 125.4 (Ar*C*), 126.0 (Ar*C*), 126.2 (Ar*C*), 126.6 (Ar*C*), 126.7 (Ar*C*), 128.4 (Ar*C*), 128.5 (Ar*C*), 128.6 (Ar*C*), 129.0 (Ar*C*), 131.5 (Ar*C*), 131.7 (Ar*C*), 132.6 (Ar*C*), 132.8 (Ar*C*), 133.1 (Ar*C*), 133.8 (Ar*C*), 134.0 (Ar*C*), 134.3 (Ar*C*), 155.1 (Ar*C*), 155.3 (Ar*C*), 156.0 (Ar*C*), 156.1 (*C*O), 156.4 (*C*O), 158.2 (*C*O). **HR-MS** (ESI, positive ion mode) – *m/z* for $[C_{52}H_{55}N_7O_6+Na]^+$ = 896.4112. Found 896.4092. **FTIR** (neat) – 3312, 2965, 2902, 1637.

1,10-Bis(naphthalen-1-ylmethyl)-1,4,7-tris(4-methoxyanilinylcarbonyl)-10-(4-nitroanilinylthiocarbonyl)-1,4,7,10-tetraazadecane, 5a

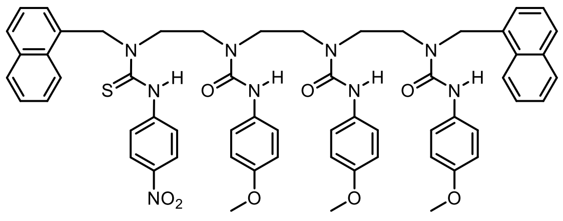

Synthesised according to general procedure A using **5a-1** (100 mg, 0.11 mmol) and 4-nitrophenyl isothiocyanate (31 mg, 0.17 mmol). The crude product was purified by column chromatography (1-2% MeOH:DCM) to yield the title thiourea as a yellow solid (116 mg, 0.11 mmol, >99%). **M.P.** 174-175 °C (DCM). **TLC** – R_f = 0.32 (SiO_2, 1:99 MeOH:DCM). **^{1}H NMR** (500 MHz, $CDCl_3$) δ_H 2.89-3.71 (m, 12H, 2 x NC*H*$_2$), 3.79 (s, 3H, OC*H*$_3$), 3.79 (s, 3H, OC*H*$_3$), 3.81 (s, 3H, OC*H*$_3$), 5.04 (s, 2H, C*H*$_2$Ar), 5.67 (s, 2H, C*H*$_2$Ar), 6.38 (s, 1H, N*H*), 6.83 (d, J = 9.0, 2H, 2 x Ar*H*), 6.83 (d, J = 9.0, 2H, 2 x Ar*H*), 6.86 (d, J = 9.0, 2H, 2 x Ar*H*), 7.17-7.48 (m, 8H, 8 x Ar*H*), 7.51 (d, J = 9.0, 2H, 2 x Ar*H*), 7.60 (d, J = 9.0, 2H, 2 x Ar*H*), 7.64-8.18 (m, 12H, 12 x Ar*H*), 8.96 (s, 2H, 2 x N*H*), 10.44 (s, 1H, N*H*). **^{13}C NMR** (126 MHz, $CDCl_3$) δ_C 47.0-47.9 (4 x N*C*H$_2$), 48.6 (N*C*H$_2$), 48.9 (N*C*H$_2$), 51.1 (*C*H$_2$Ar), 54.1 (*C*H$_2$Ar), 55.5 (3 x O*C*H$_3$), 113.9 (2 x Ar*C*), 113.9 (2 x Ar*C*), 114.0 (2 x Ar*C*), 121.0 (6 x Ar*C*), 122.4 (Ar*C*), 123.2 (Ar*C*), 123.4 (Ar*C*), 123.8 (Ar*C*), 124.0 (Ar*C*), 125.3 (Ar*C*), 125.6 (Ar*C*), 126.4 (Ar*C*), 126.7 (Ar*C*), 127.0 (2 x Ar*C*), 127.2 (2 x Ar*C*), 128.5 (Ar*C*), 128.6 (Ar*C*), 128.8 (Ar*C*), 129.1 (Ar*C*), 129.3 (Ar*C*), 130.6 (Ar*C*), 131.0 (Ar*C*), 131.5 (Ar*C*), 132.6 (Ar*C*), 132.8 (Ar*C*), 133.7 (Ar*C*), 134.0 (Ar*C*), 143.7 (Ar*C*), 147.0 (Ar*C*), 155.4 (Ar*C*), 155.5 (Ar*C*), 155.5 (Ar*C*), 156.4 (*C*O), 156.5 (*C*O), 156.9 (*C*O), 180.1 (*C*S). **HR-MS** (ESI, positive ion mode) – *m/z* for $[C_{59}H_{59}N_9O_8S+Na]^+$ = 1076.4105. Found 1076.4112. **FTIR** (neat) – 3698, 3304, 2996, 2901, 1651.

1,13-Bis(trifluoroacetyl)-4,7,10-tris(4-methoxyanilinylcarbonyl)-1,4,7,10,13-pentaazatridecane, 5b-1

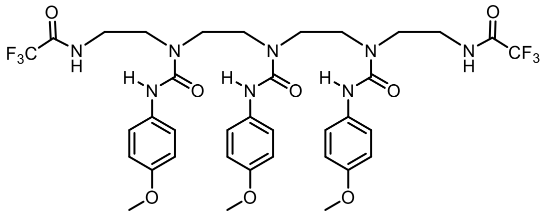

Synthesised according to general procedure C using tetraethylenepentamine (2.51 mL, 13.20 mmol) and 4-methoxyphenyl isocyanate (6.84 mL, 52.80 mmol). After 16 h, a white precipitate formed and was filtered. The residue was washed with DCM (3 x 10 mL) and air-dried to afford the title triurea as a white solid (2.74 g, 3.32 mmol, 25%). **M.P.** 177-178 °C (DCM). **TLC** – R_f = 0.26 (5:95 MeOH:DCM). **^{1}H NMR** (400 MHz, $(CD_3)_2SO$) δ_H 3.36-3.54 (m, 16H, 8 x NC*H*$_2$), 3.71 (s, 6H, 2 x OC*H*$_3$), 3.72 (3H, s, OC*H*$_3$), 6.83 (d, J = 9.0, 4H, 4 x Ar*H*), 6.85 (d, J = 9.0, 2H, 2 x Ar*H*), 7.38 (d, J = 9.0, 4H, 4 x Ar*H*), 7.44 (d, J = 9.0, 2H, 2 x Ar*H*), 8.47 (s, 2H, 2 x N*H*), 8.81 (s, 1H, N*H*), 9.50 (t, J = 5.7, 2H, 2 x N*H*). **^{13}C NMR** (101 MHz, $(CD_3)_2SO$) δ_C 38.3 (2 x N*C*H$_2$), 46.5 (2 x N*C*H$_2$), 46.7 (2 x N*C*H$_2$), 47.0 (2 x N*C*H$_2$), 55.1 (2 x O*C*H$_3$), 55.2 (O*C*H$_3$), 113.5 (4 x Ar*C*), 113.6 (2 x

Ar*C*), 116.3 (q, J = 290.7, 2 x *C*F_3), 121.4 (2 x Ar*C*), 121.8 (4 x Ar*C*), 133.2 (2 x Ar*C*), 133.3 (Ar*C*), 154.7 (2 x *C*O), 155.6 (Ar*C*), 155.9 (q, J = 35.6, 2 x *C*O). **^{19}F NMR** (377 MHz, $(CD_3)_2SO$) δ_F −74.5 (2 x CF_3). **HR-MS** (MALDI) – *m/z* for $[C_{36}H_{42}F_6N_8O_8+Na]^+$ = 851.2927. Found 851.2888. **FTIR** (neat) – 3296, 2976, 2945, 1719, 1645.

4,7,10-Tris(4-methoxyanilinylcarbonyl)-1,4,7,10,13-pentaazatridecane, 5b-2

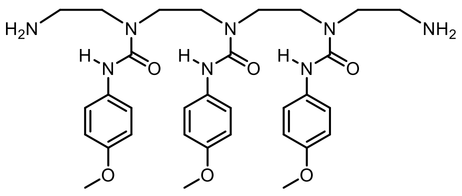

Synthesised according to general procedure D using **5b-1** (4.30 g, 5.19 mmol). After 5 h, a white precipitate formed and was filtered. The residue was washed with MeOH (3 x 10 mL) and air-dried to afford the title diamine as a white solid (3.11 g, 4.88 mmol, 94%). **M.P.** 163-164 °C (DCM). **TLC** – R_f = 0.05 (5:95:1 MeOH:DCM:TEA). **^{1}H NMR** (500 MHz, $(CD_3)_2SO$) δ_H 2.78 (t, J = 5.7, 4H, 2 x N*C*H_2), 3.37 (t, J = 6.0, 4H, 2 x NC*H_2*), 3.42 (t, J = 6.6, 4H, 2 x NC*H_2*), 3.48 (t, J = 6.6, 4H, 2 x NC*H_2*), 3.70 (s, 6H, 2 x OC*H_3*), 3.71 (s, 3H, OC*H_3*), 6.83 (d, J = 9.0, 4H, 4 x Ar*H*), 6.85 (d, J = 9.0, 2H, 2 x Ar*H*), 7.37 (d, J = 9.0, 4H, 4 x Ar*H*), 7.49 (d, J = 9.0, 2H, 2 x Ar*H*), 8.98 (s, 1H, N*H*), 9.51 (s, 2H, 2 x N*H*). **^{13}C NMR** (126 MHz, $(CD_3)_2SO$) δ_C 40.8 (2 x N*C*H_2), 46.3 (2 x N*C*H_2), 46.8 (2 x N*C*H_2), 47.0 (2 x N*C*H_2), 55.6 (2 x O*C*H_3), 55.7 (O*C*H_3), 114.1 (4 x Ar*C*), 114.1 (2 x Ar*C*), 121.1 (2 x Ar*C*), 121.2 (4 x Ar*C*), 134.0 (Ar*C*), 134.1 (2 x Ar*C*), 154.8 (2 x *C*O), 154.9 (2 x Ar*C*), 156.1 (Ar*C*), 157.0 (*C*O). **HR-MS** (MALDI) – *m/z* for $[C_{32}H_{44}N_8O_6+Na]^+$ = 659.3282. Found 659.3329. **FTIR** (neat) – 3237, 2987, 2930, 1647.

***tert*-Butyl benzyl(2-hydroxyethyl)carbamate, 5b-3**

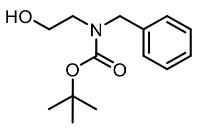

To a solution of amine *N*-benzylethanolamine (3.55 mL, 25.0 mmol, 1.0 equiv, 0.25 M) in DCM (100 mL) was added a solution of sodium hydroxide (1.0 M) in distilled water. The mixture was cooled to 0 °C and a solution of di-*tert*-butyl dicarbonate (5.47 g, 25.0 mmol, 1.0 equiv, 0.25 M) in DCM (100 mL) was added over three portions. The resultant mixture was stirred for 16 h and the aqueous phase was washed with DCM three times. The combined organic extracts were washed with distilled water twice, dried ($MgSO_4$), filtered and concentrated *in vacuo*. The crude product was purified by column chromatography (10-30% EtOAc:PE) to give the title carbamate as a colourless oil (5.72 g, 22.8 mmol, 91%). **TLC** – R_f = 0.05 (10:90 EtOAc:PE). Spectroscopic data matched that previously reported.[5]

***tert*-Butyl benzyl(2-oxoethyl)carbamate, 5b-4**

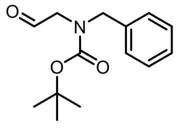

Synthesised according to general procedure K using **5b-3** (1000 mg, 3.98 mmol). The crude product was purified by column chromatography (10-20% EtOAc:PE) to give the title aldehyde as a colourless oil (982 mg, 3.94 mmol, 99%). **TLC** – R_f = 0.13 (10:90 EtOAc:PE). Spectroscopic data matched that previously reported.[5]

1-Benzyl-1-(*tert*-butoxycarbonyl)-7,10,13-tris(4-methoxyanilinylcarbonyl)-1,4,7,10,13,16-hexaazahexadecane, 5b-5

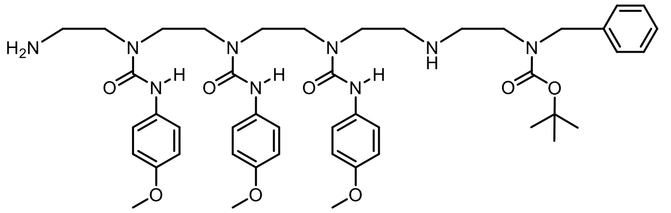

Synthesised according to general procedure J using **5b-2** (200 mg, 0.31 mmol) and **5b-4** (86 mg, 0.35 mmol). The crude product was purified by column chromatography (1-10% MeOH:DCM, 1% TEA) to give the title amine as a foamy white solid (122 mg, 0.14 mmol, 45%). **M.P.** 150-151 °C ($CHCl_3$). **TLC** – R_f = 0.38 (10:90:1 MeOH:DCM:TEA). **^{1}H NMR** (400 MHz, $CDCl_3$) δ_H 1.45 (s, 9H, C(C*H*$_3$)$_3$), 2.70-2.83 (m, 4H, 2 x NC*H*$_2$), 2.96 (t, J = 4.9, NC*H*$_2$), 3.26-3.57 (m, 14H, 7 x NC*H*$_2$), 3.76 (s, 3H, OC*H*$_3$), 3.76 (s, 3H, OC*H*$_3$), 3.77 (s, 3H, OC*H*$_3$), 4.41 (s, 2H, C*H*$_2$Ar), 6.79 (d, J = 9.1, 2H, 2 x Ar*H*), 6.80 (d, J = 9.1, 2H, 2 x Ar*H*), 6.82 (d, J = 9.1, 2H, 2 x Ar*H*), 7.18 (d, J = 9.1, 2H, 2 x Ar*H*), 7.27-7.37 (m, 7H, 7 x Ar*H*), 7.57 (d, J = 9.1, 2H, 2 x Ar*H*), 8.96 (s, 1H, N*H*), 9.73 (s, 1H, N*H*), 9.83 (s, 1H, N*H*). **^{13}C NMR** (101 MHz, $CDCl_3$) δ_C 28.5 (C(*C*H$_3$)$_3$), 46.1 (7 x N*C*H$_2$), 46.4 (N*C*H$_2$), 47.2 (N*C*H$_2$), 48.1 (*C*H$_2$Ar), 49.6 (N*C*H$_2$), 55.6 (3 x O*C*H$_3$), 80.4 (*C*(CH$_3$)$_3$), 113.8-114.3 (6 x Ar*C*), 120.0-121.4 (6 x Ar*C*), 127.3 (2 x Ar*C*), 127.5 (Ar*C*), 128.6 (2 x Ar*C*), 133.3-133.7 (3 x Ar*C*), 138.3 (Ar*C*), 155.1 (3 x Ar*C*), 156.0 (3 x *C*O), 156.4 (*C*O). **HR-MS** (ESI, positive ion mode) – *m/z* for $[C_{46}H_{63}N_9O_8+H]^+$ = 870.4878. Found 870.4899. **FTIR** (neat) – 3290, 2994, 2934, 1649.

1-Benzyl-7,10,13-tris(4-methoxyanilinylcarbonyl)-16-trifluoroacetyl-1,4,7,10,13,16-hexaazahexadecane, 5b-6

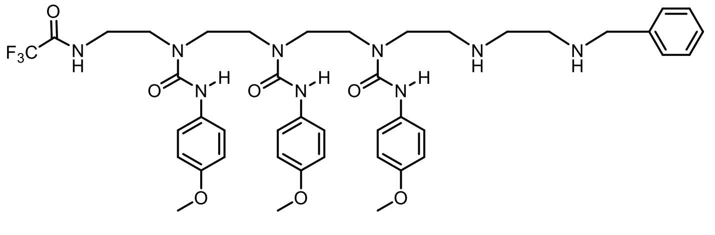

Under a dry, inert atmosphere, a solution of **5b-5** (700 mg, 0.80 mmol, 1.00 equiv, 1.0 M) in anhydrous DCM (0.8 mL) was cooled to 0 °C. Ethyl trifluoroacetate (0.11 mL, 0.88 mmol, 1.10 equiv) was added dropwise over 30 minutes and the resulting solution was stirred for 30 minutes at 0 °C. The solution was then warmed to room temperature and stirred for a further hour. The solution was then

diluted with anhydrous DCM (6 mL) and TFA (2 mL) was added dropwise. The solution was stirred for 16 h and concentrated *in vacuo*. The crude product was diluted with saturated aqueous $NaHCO_3$ and then extracted with DCM (3 x 25 mL). The combined organic extracts were dried ($MgSO_4$), filtered and concentrated *in vacuo* to give the title amine as a foamy white solid (540 mg, 0.62 mmol, 78%). **M.P.** 156-157 °C ($CHCl_3$). **TLC** – R_f = 0.21 (5:95:1 MeOH:DCM:TEA). **^{1}H NMR** (400 MHz, $CDCl_3$) δ_H 2.69-2.85 (m, 8H, 4 x NC*H*$_2$), 3.32-3.55 (m, 14H, C*H*$_2$Ar, 6 x NC*H*$_2$), 3.74 (s, 3H, OC*H*$_3$), 3.76 (s, 6H, 2 x OC*H*$_3$), 6.74-6.84 (m, 6H, 6 x Ar*H*), 7.21-7.32 (m, 5H, 5 x Ar*H*), 7.52 (d, J = 8.9, 2H, 2 x Ar*H*), 7.59 (d, J = 8.9, 2H, 2 x Ar*H*), 7.60 (d, J = 8.9, 2H, 2 x Ar*H*), 8.82 (s, 1H, N*H*), 9.00 (s, 1H, N*H*), 9.13 (s, 1H, N*H*). **^{13}C NMR** (101 MHz, $CDCl_3$) δ_C 46.4 (N*C*H$_2$), 47.1 (N*C*H$_2$), 48.3 (N*C*H$_2$), 48.4 (N*C*H$_2$), 48.4 (N*C*H$_2$), 48.5 (N*C*H$_2$), 49.2 (*C*H$_2$Ar), 49.6 (N*C*H$_2$), 49.7 (N*C*H$_2$), 50.0 (N*C*H$_2$), 50.1 (N*C*H$_2$), 55.5 (3 x O*C*H$_3$), 113.9 (2 x Ar*C*), 114.0 (2 x Ar*C*), 114.0 (2 x Ar*C*), 115.9 (q, J = 287.8, *C*F$_3$), 120.9 (2 x Ar*C*), 121.1 (2 x Ar*C*), 121.5 (2 x Ar*C*), 127.1 (Ar*C*), 128.0 (2 x Ar*C*), 128.5 (2 x Ar*C*), 132.9-133.6 (3 x Ar*C*), 140.1 (Ar*C*), 155.0 (Ar*C*), 155.2 (Ar*C*), 155.3 (Ar*C*), 155.3 (*C*O), 155.5 (*C*O), 156.4 (q, J = 36.4, *C*O), 156.6 (*C*O). **^{19}F NMR** (377 MHz, $CDCl_3$) δ_F −74.3 (C*F*$_3$). **HR-MS** (ESI, positive ion mode) – *m/z* for $[C_{43}H_{54}F_3N_9O_7+Na]^+$ = 888.3996. Found 888.3981. **FTIR** (neat) – 3324, 2990, 2945, 1647.

1-Benzyl-1,4,7,10,13-pentakis(4-methoxyanilinylcarbonyl)-16-trifluoroacetyl-1,4,7,10,13,16-hexaazahexadecane, 5b-7

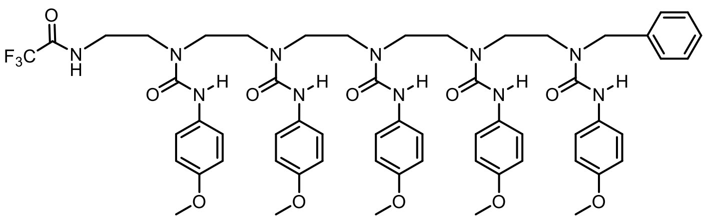

Synthesised according to general procedure A using **5b-6** (1.0 g, 1.15 mmol) and 4-methoxyphenyl isocyanate (0.45 mL, 3.46 mmol). The crude product was purified by column chromatography (2-10% MeOH:DCM) to give the title pentaurea as a foamy white solid (503 mg, 0.43 mmol, 38%). **M.P.** 186-187 °C (DCM/Et_2O). **TLC** – R_f = 0.25 (5:95 MeOH:DCM). **^{1}H NMR** (400 MHz, $CDCl_3$) δ_H 3.32-3.64 (m, 20H, 10 x NC*H*$_2$), 3.71-3.81 (m, 15H, 5 x OC*H*$_3$), 4.49 (s, 2H, C*H*$_2$Ar), 5.88 (s, 1H, N*H*), 6.78-6.85 (m, 10H, 10 x Ar*H*), 7.07 (d, J = 9.0, 2H, 2 x Ar*H*), 7.20 (d, J = 9.0, 2H, 2 x Ar*H*), 7.26-7.36 (m, 5H, 5 x Ar*H*), 7.49 (d, J = 9.0, 2H, 2 x Ar*H*), 7.56 (d, J = 9.0, 2H, 2 x Ar*H*), 7.57 (d, J = 9.0, 2H, 2 x Ar*H*), 8.34 (s, 1H, N*H*), 8.87-9.18 (m, 5H, 5 x N*H*). **^{13}C NMR** (101 MHz, $CDCl_3$) δ_C 47.0-48.0 (10 x N*C*H$_2$), 48.2 (*C*H$_2$Ar), 55.5-55.6 (5 x O*C*H$_3$), 114.0 (6 x Ar*C*), 114.1 (2 x Ar*C*), 114.2 (2 x Ar*C*), 116.5 (q, J = 284.9, *C*F$_3$), 121.1-121.3 (10 x Ar*C*), 126.6 (2 x Ar*C*), 127.9 (Ar*C*), 129.1 (2 x Ar*C*), 132.8 (Ar*C*), 132.9 (Ar*C*), 133.0 (3 x Ar*C*), 140.6 (Ar*C*), 155.3 (4 x Ar*C*), 155.9 (Ar*C*), 156.6 (4 x *C*O), 156.7 (q, J = 36.1, *C*O), 157.3 (*C*O). **^{19}F NMR** (377 MHz, $CDCl_3$) δ_F −74.9 (C*F*$_3$). **HR-MS** (MALDI) – *m/z* for $[C_{59}H_{68}F_3N_{11}O_{11}+Na]^+$ = 1186.4950. Found 1186.4893. **FTIR** (neat) – 3320, 2924, 2898, 1648.

1-Benzyl-1,4,7,10,13-pentakis(4-methoxyanilinylcarbonyl)-1,4,7,10,13,16-hexaazahexadecane, 5b-8

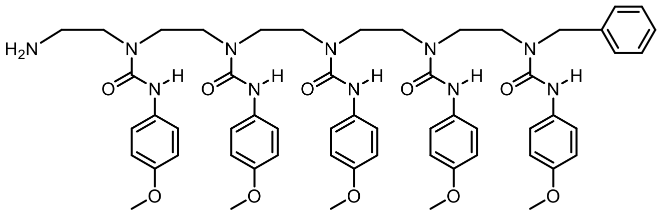

Synthesised according to general procedure D using **5b-7** (450 mg, 0.39 mmol). The title amine was isolated as a foamy white solid (340 mg, 0.32 mmol, 82%). **M.P.** 150-151 °C (DCM/Et_2O). **TLC** – R_f = 0.18 (8:92 MeOH:DCM). **^{1}H NMR** (500 MHz, $CDCl_3$) δ_H 2.97 (t, J = 4.0, 2H, NC*H*$_2$), 3.35-3.63 (m, 18H, 9 x NC*H*$_2$), 3.79-3.82 (m, 15H, 5 x OC*H*$_3$), 4.63 (s, 2H, C*H*$_2$Ar), 6.80-6.88 (m, 10H, 10 x Ar*H*), 7.26-7.36 (m, 9H, 9 x Ar*H*), 7.60 (d, J = 8.8, 2H, 2 x Ar*H*), 7.63 (d, J = 8.8, 4H, 4 x Ar*H*), 8.97 (s, 1H, N*H*), 9.08-9.13 (m, 3H, 3 x N*H*), 10.1 (s, 1H, N*H*). **^{13}C NMR** (126 MHz, $CDCl_3$) δ_C 42.1 (N*C*H$_2$), 46.8-48.6 (9 x N*C*H$_2$), 51.0 (*C*H$_2$Ar), 55.5-55.6 (5 x O*C*H$_3$), 113.9 (6 x Ar*C*), 114.0 (2 x Ar*C*), 114.1 (2 x Ar*C*), 120.6 (2 x Ar*C*), 121.1 (6 x Ar*C*), 121.7 (2 x Ar*C*), 126.9 (2 x Ar*C*), 127.7 (Ar*C*), 129.0 (2 x Ar*C*), 133.2-133.5 (5 x Ar*C*), 140.0 (Ar*C*), 155.2 (5 x Ar*C*), 156.7 (5 x *C*O). **HR-MS** (ESI, positive ion mode) – *m/z* for $[C_{57}H_{69}N_{11}O_{10}+Na]^+$ = 1090.5127. Found 1090.5095. **FTIR** (neat) – 3315, 3031, 2950, 1640.

1,19-Dibenzyl-1,4,7,10,13-pentakis(4-methoxyanilinylcarbonyl)-19-(*tert*-butoxycarbonyl)-1,4,7,10,13,16,19-heptaazanonadecane, 5b-9

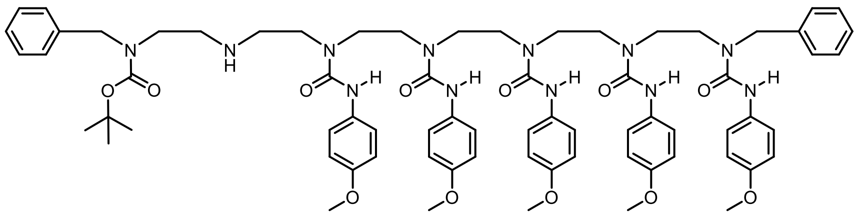

Synthesised according to general procedure B using **5b-8** (340 mg, 0.32 mmol) and **5b-4** (159 mg, 0.64 mmol). The crude product was purified by column chromatography (2-10% MeOH:DCM) to give the title amine as a foamy white solid (233 mg, 0.18 mmol, 56%). **M.P.** 155-156 °C (DCM/Et_2O). **TLC** – R_f = 0.33 (8:92 MeOH:DCM). **^{1}H NMR** (400 MHz, $CDCl_3$) δ_H 1.46 (s, 9H, C(C*H*$_3$)$_3$), 1.91 (s, 1H, N*H*), 2.71-2.79 (m, 4H, 2 x NC*H*$_2$), 3.24-3.58 (m, 20H, 10 x NC*H*$_2$), 3.74-3.79 (m, 15H, 5 x OC*H*$_3$), 4.40 (s, 2H, C*H*$_2$Ar), 4.58 (s, 2H, C*H*$_2$Ar), 6.77-6.87 (m, 10H, 10 x Ar*H*), 7.15 (d, J = 9.0, 2H, 2 x Ar*H*), 7.25-7.39 (m, 14H, 14 x Ar*H*), 7.53-7.68 (m, 6H, 6 x Ar*H*), 8.98-9.15 (m, 4H, 4 x N*H*), 9.91 (s, 1H, N*H*). **^{13}C NMR** (101 MHz, $CDCl_3$) δ_C 28.5 (*C*(CH$_3$)$_3$), 42.4 (N*C*H$_2$), 43.2 (N*C*H$_2$), 46.4-49.0 (10 x N*C*H$_2$), 50.2 (*C*H$_2$Ar), 51.3 (*C*H$_2$Ar), 55.4-55.6 (5 x O*C*H$_3$), 80.1 (*C*(CH$_3$)$_3$), 113.8-114.1 (10 x Ar*C*), 121.0-121.3 (10 x Ar*C*), 126.9-128.8 (10 x Ar*C*), 132.8-133.9 (5 x Ar*C*), 140.2 (Ar*C*), 140.8 (Ar*C*), 155.1-155.4 (5 x Ar*C*) 156.0-156.5 (6 x *C*O). **HR-MS** (ESI, positive ion mode) – *m/z* for $[C_{71}H_{88}N_{12}O_{12}+H]^+$ = 1301.6723. Found 1301.6700. **FTIR** (neat) – 3287, 2933, 1650.

1,19-Dibenzyl-1,4,7,10,13,16-hexakis(4-methoxyanilinylcarbonyl)-19-(*tert*-butoxycarbonyl)-1,4,7,10,13,16,19-heptaazanonadecane, 5b-10

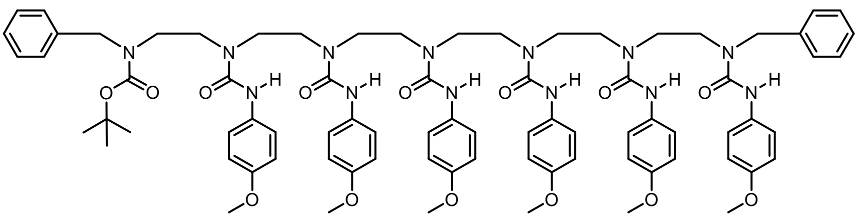

Synthesised according to general procedure A using **5b-9** (135 mg, 0.10 mmol) and 4-methoxphenyl isocyanate (0.02 mL, 0.16 mmol). The crude product was purified by column chromatography (2-10% MeOH:DCM) to give the title hexaurea as a foamy white solid (130 mg, 0.09 mmol, 90%). **M.P.** 190-191 °C (DCM/Et_2O). **TLC** – R_f = 0.23 (3:97 MeOH:DCM). **^{1}H NMR** (400 MHz, $CDCl_3$) δ_H 1.50 (s, 9H, C(CH_3)$_3$), 3.28-3.60 (m, 24H, 12 x NCH_2), 3.74-3.81 (m, 18H, 6 x OCH_3), 4.41 (s, 2H, CH_2Ar), 4.57 (s, 2H, CH_2Ar), 6.76-6.87 (m, 12H, 12 x Ar*H*), 7.19 (d, J = 9.0, 2H, 2 x Ar*H*), 7.28-7.41 (m, 10H, 10 x Ar*H*), 7.57-7.65 (m, 10H, 10 x Ar*H*), 8.83 (s, 1H, N*H*), 8.97 (s, 1H, N*H*), 9.02 (s, 1H, N*H*), 9.13 (s, 1H, N*H*), 9.17 (s, 1H, N*H*). **^{13}C NMR** (101 MHz, $CDCl_3$) δ_C 28.5 (C(*C*H_3)$_3$), 45.8-49.0 (12 x N*C*H_2), 50.2-51.5 (2 x *C*H_2Ar), 55.5-55.6 (6 x O*C*H_3), 80.0 (*C*(CH_3)$_3$), 113.7-114.1 (12 x Ar*C*), 120.7-121.7 (12 x Ar*C*), 127.4-128.8 (10 x Ar*C*), 132.8-133.6 (6 x Ar*C*), 139.9-140.6 (2 x Ar*C*), 155.0-155.4 (6 x Ar*C*), 156.7-157.1 (7 x *C*O). **HR-MS** (MALDI) – *m/z* for $[C_{79}H_{95}N_{13}O_{14}+Na]^+$ = 1472.7019. Found 1472.6984. **FTIR** (neat) – 3321, 2924, 2853, 1713.

1,19-Dibenzyl-1,4,7,10,13,16-hexakis(4-methoxyanilinylcarbonyl)-1,4,7,10,13,16,19-heptaazanonadecane, 5b-11

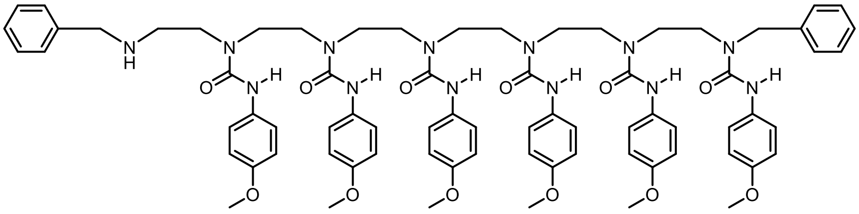

Synthesised according to general procedure H using **5b-10** (130 mg, 0.09 mmol). The title amine was isolated as a foamy white solid (101 mg, 0.07 mmol, 83%). **M.P.** 204-205 °C (DCM/Et_2O). **TLC** – R_f = 0.13 (3:97 MeOH:DCM). **^{1}H NMR** (400 MHz, $CDCl_3$) δ_H 2.87 (s, 2H, NCH_2), 3.30-3.62 (m, 22H, 11 x NCH_2), 3.75-3.78 (m, 18H, 6 x OCH_3), 3.82 (s, 2H, CH_2Ar), 4.57 (CH_2Ar), 6.74-6.86 (m, 12H, 12 x Ar*H*), 7.22 (d, J = 9.0, 2H, 2 x Ar*H*), 7.28-7.41 (m, 12H, 12 x Ar*H*), 7.55-7.64 (m, 8H, 8 x Ar*H*), 8.95 (s, 1H, N*H*), 9.06 (s, 1H, N*H*), 9.08 (s, 1H, N*H*), 9.15 (s, 1H, N*H*), 10.04 (s, 1H, N*H*). **^{13}C NMR** (101 MHz, $CDCl_3$) δ_C 47.0-49.2 (12 x N*C*H_2), 51.0-52.2 (2 x *C*H_2Ar), 55.6-55.7 (6 x O*C*H_3), 113.8-114.3 (12 x Ar*C*), 121.1-122.6 (12 x Ar*C*), 127.6-129.0 (10 x Ar*C*), 133.0-133.8 (6 x Ar*C*), 140.3-140.6 (2 x Ar*C*), 155.2-156.8 (6 x *C*O, 6 x Ar*C*). **HR-MS** (ESI, positive ion mode) – *m/z* for $[C_{74}H_{87}N_{13}O_{12}+Na]^+$ = 1372.6495. Found 1372.6494. **FTIR** (neat) – 3338, 2950, 1645.

1,19-Dibenzyl-1,4,7,10,13,16-hexakis(4-methoxyanilinylcarbonyl)-19-(4-nitroanilinylthiocarbonyl)-1,4,7,10,13,16,19-heptaazanonadecane, 5b

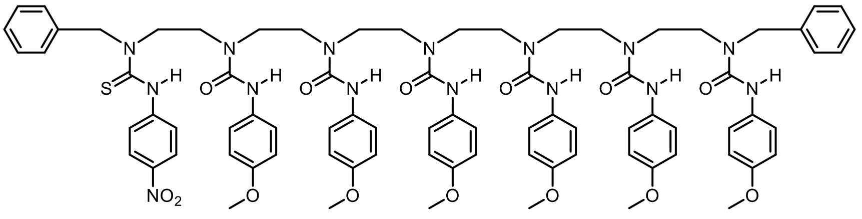

Synthesised according to general procedure A using **5b-11** (40 mg, 0.03 mmol) and 4-nitrophenyl isothiocyanate (8 mg, 0.04 mmol). The crude product was purified by column chromatography (2-10% MeOH:DCM) to give the title thiourea as a yellow solid (46 mg, 0.03 mmol, >99%). **M.P.** 159-160 °C (DCM/Et_2O). **TLC** – R_f = 0.28 (3:97 MeOH:DCM). **^{1}H NMR** (500 MHz, CD_2Cl_2) δ_H 3.38-3.68 (m, 24H, 12 x NC*H*$_2$), 3.76-3.85 (m, 18H, 6 x OC*H*$_3$), 4.61 (s, 2H, C*H*$_2$Ar), 5.32 (s, 2H, C*H*$_2$Ar), 6.31 (s, 1H, N*H*), 6.84-6.93 (m, 12H, 12 x Ar*H*), 7.17-7.67 (m, 22H, 22 x Ar*H*), 8.06 (d, J = 9.0, 2H, 2 x Ar*H*), 8.19 (d, J = 8.8, 2H, 2 x Ar*H*), 9.03 (s, 1H, N*H*), 9.17 (s, 1H, N*H*), 9.19 (s, 1H, N*H*), 9.23 (s, 1H, N*H*), 9.29 (s, 1H, N*H*), 10.48 (s, 1H, N*H*). **^{13}C NMR** (126 MHz, CD_2Cl_2) δ_C 47.3-49.0 (12 x N*C*H$_2$), 53.3 (*C*H$_2$Ar), 55.9-56.2 (6 x O*C*H$_3$), 56.8 (*C*H$_2$Ar), 114.3-114.7 (12 x Ar*C*), 121.6-121.9 (12 x Ar*C*), 123.0 (2 x Ar*C*), 124.4 (2 x Ar*C*), 126.6-130.2 (10 x Ar*C*), 133.4-134.0 (6 x Ar*C*), 138.9-139.3 (2 x Ar*C*), 143.7 (Ar*C*), 147.4 (Ar*C*), 155.4-155.8 (6 x Ar*C*), 156.7-157.2 (6 x *C*O), 182.3 (*C*S). **HR-MS** (MALDI) – *m/z* for $[C_{81}H_{91}N_{15}O_{14}S+Na]^+$ = 1552.6488. Found 1552.6491. **FTIR** (neat) – 3675, 3345, 2971, 2914, 1648, 1606, 1551.

***N*-(4-methoxyphenyl)-*N*-methylcarbamoyl chloride, 6a-1**

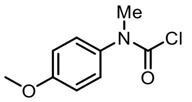

Under a dry, inert atmosphere, a solution of triphosgene (500 mg, 1.68 mmol, 0.46 equiv) in anhydrous DCM (5 mL) was cooled to 0 °C. To this solution was added anhydrous pyridine (0.30 mL, 3.66 mmol, 1.00 equiv) dropwise and the solution was stirred for 5 minutes. 4-methoxy-*N*-methylaniline (502 mg, 3.66 mmol, 1.00 equiv, 0.70 M) was added, the solution was warmed to room temperature and stirred for 1 h. Hydrochloric acid (1 M, 10 mL) was added and the aqueous phase extracted with DCM (3 x 5 mL). The combined organic extracts were washed with saturated aqueous $NaHCO_3$, dried ($MgSO_4$), filtered and concentrated *in vacuo*. The crude product was purified by column chromatography (50% EtOAc:PE) to give the title carbamoyl chloride as a yellow oil (731 mg, 3.66 mmol, >99%). **TLC** – R_f = 0.63 (SiO_2, 1:1 EtOAc:PE). Spectroscopic data matched that previously reported.[6]

1,10-Bis(naphthalen-1-ylmethyl)-1,4,7-tris(4-methoxyanilinylcarbonyl)-10-((*N*-methyl-4-methoxyanilinyl)carbonyl)-1,4,7,10-tetraazadecane, 6a

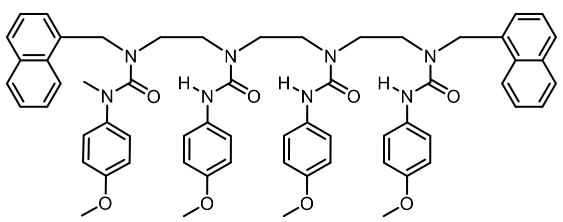

Synthesised according to general procedure G using **5a-1** (30 mg, 0.03 mmol) and **6a-1** (10 mg, 0.05 mmol). The crude product was purified by column chromatography (1-2% MeOH:DCM) to yield the title tetraurea as a white solid (27 mg, 0.03 mmol, 88%). **M.P.** 153-154 °C ($CHCl_3$). **TLC** – R_f = 0.14 (SiO_2, 1:99 MeOH:DCM). **^{1}H NMR** (500 MHz, CD_2Cl_2) δ_H 2.86-2.96 (m, 4H, 2 x NC*H*$_2$), 3.05 (NC*H*$_2$), 3.27-3.30 (m, 4H, 2 x NC*H*$_2$), 3.36 (t, J = 7.0, 2H, NC*H*$_2$), 3.66 (s, 3H, NC*H*$_3$), 3.76 (s, 3H, OC*H*$_3$), 3.78 (s, 3H, OC*H*$_3$), 3.78 (s, 3H, OC*H*$_3$), 4.50 (s, 2H, C*H*$_2$Ar), 5.08 (s, 2H, C*H*$_2$Ar), 6.71 (d, J = 8.9, 2H, 2 x Ar*H*), 6.79 (d, J = 9.0, 2H, 2 x Ar*H*), 6.85 (d, J = 9.0, 2H, 2 x Ar*H*), 6.86 (d, J = 9.0, 2H, 2 x Ar*H*), 7.05 (d, J = 8.9, 2H, 2 x Ar*H*), 7.12 (d, J = 6.7, 1H, Ar*H*), 7.27 (t, J = 7.5, 1H, Ar*H*), 7.36-7.73 (m, 13H, 13 x Ar*H*), 7.79 (d, J = 8.0, 1H, Ar*H*), 7.83 (d, J = 8.0, 1H, Ar*H*), 8.15 (d, J = 8.0, 1H, Ar*H*), 8.73 (s, 1H, N*H*), 8.85 (s, 1H, N*H*). 9.09 (s, 1H, N*H*). **^{13}C NMR** (126 MHz, CD_2Cl_2) δ_C 40.9 (N*C*H$_3$), 44.9 (N*C*H$_2$), 47.0 (N*C*H$_2$), 47.1 (N*C*H$_2$), 47.4 (N*C*H$_2$), 47.8 (N*C*H$_2$), 48.5 (N*C*H$_2$), 48.7 (*C*H$_2$Ar), 51.9 (*C*H$_2$Ar), 55.4 (4 x O*C*H$_3$), 113.7 (2 x Ar*C*), 113.7 (2 x Ar*C*), 113.8 (2 x Ar*C*), 114.8 (2 x Ar*C*), 121.0 (2 x Ar*C*), 121.1 (2 x Ar*C*), 122.5 (2 x Ar*C*), 123.5 (2 x Ar*C*), 125.0 (Ar*C*), 125.2 (Ar*C*), 125.3 (Ar*C*), 125.8 (Ar*C*), 126.0 (Ar*C*), 126.1 (Ar*C*), 126.4 (Ar*C*), 126.5 (Ar*C*), 126.7 (Ar*C*), 128.0 (Ar*C*), 128.4 (Ar*C*), 128.3 (Ar*C*), 128.5 (Ar*C*), 128.6 (Ar*C*), 128.9 (Ar*C*), 131.1 (Ar*C*), 131.6 (Ar*C*), 132.3 (Ar*C*), 133.2 (Ar*C*), 133.3 (Ar*C*), 133.6 (Ar*C*), 133.8 (Ar*C*), 138.9 (Ar*C*), 155.0 (Ar*C*), 155.1 (Ar*C*), 155.3 (Ar*C*), 156.4 (*C*O), 156.5 (*C*O), 157.4 (*C*O), 162.8 (*C*O). **HR-MS** (ESI, positive ion mode) – *m/z* for $[C_{61}H_{64}N_8O_8+Na]^+$ = 1059.4745. Found 1059.4725. **FTIR** (neat) – 3315, 2990, 2905, 1652, 1609.

1,19-Dibenzyl-1,4,7,10,13,16-hexakis(4-methoxyanilinylcarbonyl)-19-((*N*-methyl-4-methoxyanilinyl)carbonyl)-1,4,7,10,13,16,19-heptaazanonadecane, 6b

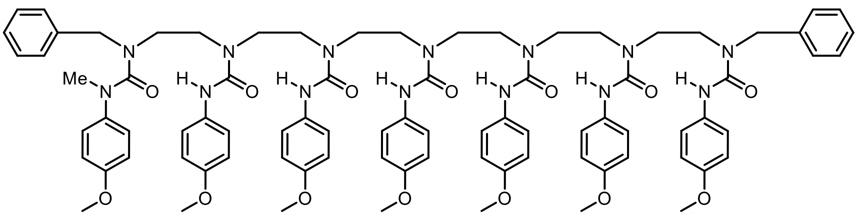

Synthesised according to general procedure G using **5b-11** (40 mg, 0.03 mmol) and **6a-1** (9 mg, 0.04 mmol). The crude product was purified by column chromatography (2-10% MeOH:DCM) to give the title heptaurea as a foamy white solid (37 mg, 0.02 mmol, 80%). **M.P.** 170-171 °C (DCM/Et_2O). **TLC** – R_f = 0.15 (3:97 MeOH:DCM). **^{1}H NMR** (500 MHz, CD_2Cl_2) δ_H 3.30-3.60 (m, 12H, 6 x NCH_2), 3.76 (s, 3H, NCH_3), 3.80-3.84 (m, 18H, 6 x OCH_3), 4.07 (s, 2H, CH_2Ar), 4.59 (s, 2H, CH_2Ar), 6.77-6.89 (m, 14H, 14 x Ar*H*), 6.95 (d, J = 7.0, 2H, 2 x Ar*H*), 7.02 (d, J = 8.8, 2H, 2 x Ar*H*), 7.17-7.40 (m, 10H, 10 x Ar*H*), 7.56-7.67 (m, 10H, 10 x Ar*H*), 8.99-9.23 (m, 6H, 6 x N*H*). **^{13}C NMR** (126 MHz, CD_2Cl_2) δ_C 40.4 (N*C*H_3), 46.3-49.1 (12 x N*C*H_2), 53.0 (*C*H_2Ar), 53.8 (*C*H_2Ar), 55.3-55.5 (7 x O*C*H_3), 113.7-114.1 (14 x Ar*C*), 121.0-121.5 (14 x Ar*C*), 126.2-128.7 (10 x Ar*C*), 133.2-133.6 (6 x Ar*C*), 137.7 (Ar*C*), 138.6-139.2 (2 x Ar*C*), 155.2-155.7 (7 x Ar*C*), 156.5-157.0 (6 x *C*O), 163.1 (*C*O). **HR-MS** (MALDI) – *m/z* for $[C_{83}H_{96}N_{14}O_{14}+Na]^+$ = 1535.7128. Found 1535.7096. **FTIR** (neat) – 3349, 2968, 2931, 1649.

***tert*-Butyl (2-trifluoroacetamidoethyl)carbamate, 4b-1**

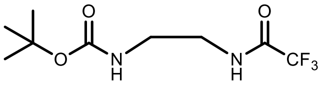

To a solution of *N*-Boc-ethylenediamine (1.602 g, 10.00 mmol, 1.0 equiv) in lab grade CH_2Cl_2 (10 mL) at room temperature under air was added over 1 min a solution of ethyl trifluoroacetate (1.19 mL, 10.00 mmol, 1.0 equiv) in CH_2Cl_2 (10 mL). The mixture was stirred at room temperature for 19 h. Additional ethyl trifluoroacetate (0.71 mL, 6.00 mmol, 0.6 equiv) was added and the mixture was stirred for a further 75.5 h. The mixture was diluted with CH_2Cl_2 (40 mL) and washed with aqueous HCl (40 mL, 1.0 M) to remove unreacted *N*-Boc-ethylenediamine. The organic phase was dried (Na_2SO_4) and concentrated to give **4b-1** (2.206 g, 86%) as a white solid. **TLC** – R_f = 0.65 (5:95 MeOH/CH_2Cl_2, ninhydrin stain). **^{1}H NMR** (400 MHz, $CDCl_3$) δ_H 1.42 (s, 9H), 3.31 – 3.47 (m, 4H), 5.02 – 5.11 (broad m, 1H), 7.91 (bs, 1H). **^{13}C NMR** (101 MHz, $CDCl_3$) δ_C 28.3, 39.2, 42.2, 80.6, 116.0 (q, *J* = 287.6 Hz), 157.9, 158.0 (q, *J* = 37.3 Hz). **^{19}F NMR** (376 MHz, $CDCl_3$) δ_F –76.0 (s, 3F). Spectroscopic data matches that previously reported.[7]

2-Trifluoroacetamidoethylammonium chloride, 4b-2

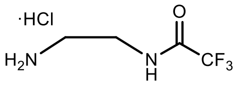

To a solution of **4b-1** (2.154 g, 8.41 mmol, 1.0 equiv in lab grade CH_2Cl_2 (42 mL) at room temperature under air was added TFA (9.66 mL, 126.1 mmol, 15.0 equiv and the mixture was stirred at room temperature for 15 h. The mixture was concentrated on a rotary evaporator and then under high vacuum to remove most of the excess TFA. The resulting colourless gum (3.177 g, 140% of the theoretical mass of the product amine·TFA salt) was dissolved in lab grade MeOH (10 mL) and HCl (1.25 M in MeOH, 20 mL, 25 mmol) was added. The mixture was concentrated *in vacuo* to give the HCl salt **4b-2** (1.604 g, 99%) as a white solid. **^{1}H NMR** (400 MHz, CD_3OD) δ_H 3.15 (t, J = 6.2 Hz, 2H), 3.61 (t, J = 6.1 Hz, 2H). **^{13}C NMR** (101 MHz, CD_3OD) δ_C 38.3, 39.9, 117.3 (q, J = 286.5 Hz), 159.9 (q, J = 37.6 Hz). **^{19}F NMR** (376 MHz, CD_3OD) δ_F –77.2 (s, 3F). Spectroscopic data matches that previously reported.[8]

***N*-(2-Pyridyl)-*N*'-(2-hydroxyethyl)-1,2-diaminoethane, 4b-3**

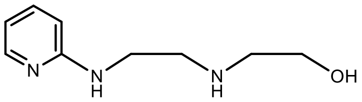

A mixture of 2-fluoropyridine (1.72 mL, 20.0 mmol, 1.0 equiv) and aminoethylethanolamine (6.07 mL, 60.0 mmol, 3.0 equiv) under N_2 was heated at 120 °C (flask with condenser but no water flow necessary) for 17.5 h. After cooling to room temperature, aqueous K_2CO_3 (25 mL, 1.0 M) and water (25 mL) were added. The water-soluble product was extracted with CH_2Cl_2 (6 × 50 mL) then the combined organic extracts were concentrated. Flash chromatography (Biotage, 100 g SNAP Ultra column, MeOH/[35% aqueous NH_3]/CH_2Cl_2 gradient from 2.5:0.5:97 to 10:2:88) gave two fractions containing **4b-3**: the first fraction (1.053 g) contained an unknown higher R_F impurity and showed ~80 mol % purity of **4b-3** by ^{1}H/^{13}C NMR; the second fraction contained pure **4b-3** (1.229 g) as a colourless gum. Total yield of **4b-3** (>90 mol% combined purity) = 2.282 g, 63%. **TLC** – R_f = 0.28 (10:2:88 MeOH/[35% aqueous NH_3]/CH_2Cl_2). **^{1}H NMR** (400 MHz, $CDCl_3$) δ_H 2.74 – 2.77 (m, 2H), 2.85 (t, J = 5.8 Hz, 2H), 3.08 (bs, 2H), 3.38 (q, J = 5.7 Hz, 2H), 3.65 (t, J = 5.2 Hz, 2H), 5.21 (t, J = 5.7 Hz, 1H), 6.38 (d, J = 8.4 Hz, 1H), 6.52 (ddd, J = 7.2, 5.2, 1.0 Hz, 1H), 7.37 (ddd, J = 8.7, 7.1, 1.9 Hz, 1H), 8.01 (dd, J = 5.2, 1.9 Hz, 1H). **^{13}C NMR** (101 MHz, $CDCl_3$) δ_C 41.8, 48.5, 51.2, 61.0, 107.3, 112.9, 137.6, 147.9, 158.9. **HR-MS** (ESI, positive ion mode) – m/z for $[C_9H_{15}N_3O+H]^+$ = 182.1288. Found 182.1293.

1-(2-Pyridyl)-4-(*tert*-butoxycarbonyl)-6-hydroxy-1,4-diazahexane, 4b-4

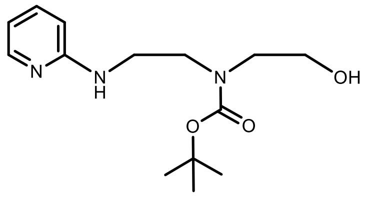

To a solution of **4b-3** (1.203 g, 6.64 mmol, 1.0 equiv) in lab grade THF (12.1 mL) at 0 °C opened to the air was added over 2 min a solution of Boc_2O (1.449 g, 6.64 mmol, 1.0 equiv) in THF (10.0 mL). The flask was fitted with a septum which was pierced with a needle (to vent CO_2). The mixture was allowed to warm to room temperature and stirred for 20 h before being concentrated *in vacuo*. Flash chromatography (Biotage, 45 g Zip Sphere column, MeOH/CH_2Cl_2 gradient from 1:99 to 6:94) gave **4b-4** (1.544 g, 83%) as a colourless gum. **TLC** – R_f = 0.13 (5:95 MeOH/CH_2Cl_2). **^{1}H NMR** (400 MHz, $CDCl_3$) δ_H (~1:1 mixture of Boc rotamers; *indicates resolved rotamers) [1.32* (s) & 1.37* (s), 9H in total], 3.30 – 3.81 (m, 8H), [4.47* (bs) & 5.32* (bs), 1H in total], 4.91 – 5.09 (m, 1H), 6.37 (d, *J* = 8.4 Hz, 1H), 6.46 – 6.56 (m, 1H), 7.28 – 7.39 (m, 1H), 7.92 – 8.03 (m, 1H). **^{13}C NMR** (101 MHz, $CDCl_3$) δ_C (~1:1 mixture of Boc rotamers; *indicates resolved rotamers:) 28.4, 40.9*, 41.2*, 48.8*, 49.5*, 51.8*, 53.3*, 61.3*, 61.9*, 79.9*, 80.3*, 107.8*, 109.0*, 112.5*, 112.9*, 137.5, 147.0*, 147.8*, 156.4*, 156.8,* 158.5*, 158.8*. **HR-MS** (ESI, positive ion mode) – *m/z* for $[C_{14}H_{23}N_3O_3+H]^+$ = 282.1812. Found 282.1802.

1-(2-Pyridyl)-4-(*tert*-butoxycarbonyl)-6-(*tert*-butyldimethylsilylhydroxy)-1,4-diazahexane, 4b-5

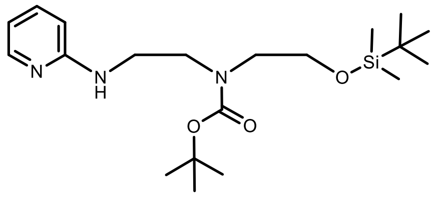

To a solution of **4b-4** (1.425 g, 5.06 mmol, 1.0 equiv) and Et_3N (0.85 mL, 6.08 mmol, 1.2 equiv) in anhydrous DMF (6.1 mL) at 0 °C under N_2 was added a solution of TBSCl (763.4 mg, 5.06 mmol, 1.0 equiv) in DMF (4.0 mL). The ice bath was removed and the mixture was stirred at room temperature for 2 h. Additional Et_3N (0.14 mL, 1.01 mmol, 0.2 equiv) and a solution of TBSCl (76.3 mg, 0.51 mmol, 0.1 equiv) in DMF (0.5 mL) were added and stirring continued for a further 1.5 h. Water (75 mL) was added and the product was extracted with Et_2O (50 mL + 70 mL). The combined organic extracts were dried (Na_2SO_4) and concentrated. Flash chromatography (Biotage, 30 g Zip Sphere column, EtOAc/petroleum ether gradient from 0:100 to 40:60) gave **4b-5** (1.685 g, 84%) as a colourless gum. **TLC** – R_f = 0.48 (25:75 EtOAc/petroleum ether). **^{1}H NMR** (400 MHz, $CDCl_3$) δ_H (~1:1 mixture of Boc rotamers; *indicates resolved rotamers) 0.03 (s, 6H), 0.87 (s, 9H), [1.41* (s) & 1.44* (s), 9H in total], 3.25 – 3.36 (m, 2H), 3.42 – 3.55 (m, 4H), 3.64 – 3.78 (m, 2H), [4.80 – 4.88* (m) & 5.08 – 5.16* (m), 1H in total], 6.38 (d, *J* = 8.4 Hz, 1H), 6.47 – 6.57 (m, 1H), 7.32 – 7.39 (m, 1H), 8.03 – 8.07 (m, 1H). **^{13}C NMR** (101 MHz, $CDCl_3$) δ_C (~1:1 mixture of Boc rotamers; *indicates resolved rotamers) –5.3, 18.4, 26.0, 28.5, 40.8*, 41.4*, 47.9*, 48.1*, 50.28*, 50.34*, 61.8, 79.9, 107.2*, 107.5*, 112.6*, 112.9*, 137.26*, 137.33*, 148.1*, 148.2*, 155.7*, 156.6*, 158.6*, 158.8*. **HR-MS** (ESI, positive ion mode) – *m/z* for $[C_{20}H_{37}N_3O_3Si+H]^+$ = 396.2677. Found 396.2669.

1-(2-Pyridyl)-1-(4-methoxyanilinylcarbonyl)-4-(*tert*-butoxycarbonyl)-6-(*tert*-butyldimethylsilylhydroxy)-1,4-diazahexane, 4b-6

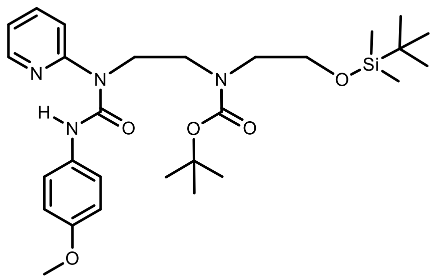

To a solution of **4b-5** (1.054 g, 2.66 mmol, 1.0 equiv) in lab grade 1,2-dichloroethane (3.0 mL) at room temperature under air was added a solution of 4-methoxyphenyl isocyanate (397.4 mg, 2.66 mmol, 1.0 equiv) in 1,2-dichloroethane (2.3 mL). The mixture was heated to 40 °C (closed system: round-bottom flask with septum) and maintained at this temperature for 20 h. After cooling to room temperature, MeOH (~5 mL) was added and the mixture was stirred for 30 min to quench any unreacted isocyanate. The mixture was concentrated *in vacuo*. Flash chromatography (Biotage, 30 g Zip Sphere column, EtOAc/petroleum ether gradient from 0:100 to 40:60) gave **4b-6** (1.013 g, 70%) as a yellow gum. **TLC** – R_f = 0.34 (15:85 EtOAc/petroleum ether). **^{1}H NMR** (400 MHz, $CDCl_3$) δ_H (1.6:1 mixture of Boc rotamers; **indicates resolved *major* rotamer; *indicates resolved *minor* rotamer) 0.04 (s, 6H), 0.87 (s, 9H), 1.48 (s, 9H), 3.40 (dt, *J* = 12.8, 5.9 Hz, 2H), 3.53 – 3.63 (m, 2H), [3.70** (t, *J* = 6.3 Hz) & 3.75 – 3.79* (m), 2H in total], 3.79 (s, 3H), 4.07 – 4.12 (m, 2H), 6.87 (d, *J* = 9.1 Hz, 2H), 6.95 – 7.00 (m, 1H), [7.24 – 7.27* (m) & 7.63** (d, *J* = 8.6 Hz), 1H in total], 7.42 – 7.49 (m, 2H), [7.68* (ddd, *J* = 8.9, 7.3, 2.0 Hz) & 7.77** (ddd, *J* = 9.1, 7.3, 2.0 Hz), 1H in total], [8.27** (dd, *J* = 5.0, 2.0 Hz) & 8.31* (dd, *J* = 5.0, 2.0 Hz), 1H in total]; *Note:* For this compound, the spectral width of the ^{1}H NMR experiment was insufficient to observe the NH proton, which typically resonates at ~12.5 ppm in $CDCl_3$. **^{13}C NMR** (101 MHz, $CDCl_3$) δ_C (1.6:1 mixture of Boc rotamers; **indicates resolved *major* rotamer; *indicates resolved *minor* rotamer) –5.2, 18.4, 26.0, 28.6, 43.3**, 43.7*, 46.4, 50.4*, 50.8**, 55.6, 61.4, 79.8**, 80.0*, 111.8*, 113.0**, 114.2, 117.3*, 117.4**, 122.37**, 122.43*, 132.2*, 132.4**, 139.0*, 139.4**, 145.5**, 145.8*, 153.9*, 154.2**, 155.4*, 155.7, 155.8**, 155.9*, 156.1**. **HR-MS** (ESI, positive ion mode) – *m/z* for $[C_{28}H_{44}N_4O_5Si+H]^+$ = 545.3154. Found 545.3181.

Unreacted starting material **4b-5** (315.2 mg, 30%) was recovered from a subsequent column fraction.

1-(2-Pyridyl)-1-(4-methoxyanilinylcarbonyl)-4-(*tert*-butoxycarbonyl)-6-hydroxy-1,4-diazahexane, 4b-7

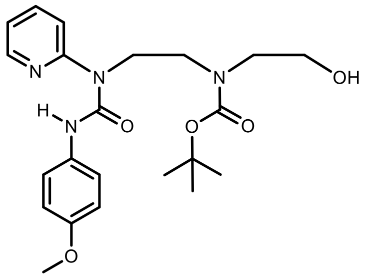

To a solution of **4b-6** (974.9 mg, 1.79 mmol, 1.0 equiv) in lab grade THF (1.5 mL) at room temperature under air was added a commercial solution of TBAF (1.0 M in THF, 4.5 mL, 4.5 mmol, 2.5 equiv). The flask was fitted with a septum and the mixture was stirred at room temperature under N_2 for 40 min. The reaction mixture (including solvent) was directly loaded on to a silica column (21.5 g silica) and elution was performed with THF (~150 mL) to remove most of the ammonium salts. The eluent was concentrated *in vacuo*. Flash chromatography (Biotage, 10 g SNAP Ultra column, EtOAc/petroleum ether gradient from 40:60 to 100:0) gave **4b-7** (746.7 mg, 97%) as a colourless gum. **TLC** – R_f = 0.21 (60:40 EtOAc/petroleum ether). **^{1}H NMR** (400 MHz, $CDCl_3$) δ_H (1.2:1 mixture of Boc rotamers; **indicates resolved *major* rotamer; *indicates resolved *minor* rotamer) [1.47* (s) & 1.48** (s), 9H in total], 2.77 (bs, 1H), 3.44 – 3.61 (m, 4H), 3.73 – 3.83 (m, 2H), 3.78 (s, 3H), 4.08 – 4.16 (m, 2H), 6.86 (d, *J* = 9.0 Hz, 2H), 6.96 – 7.03 (m, 1H), [7.24** (d, *J* = 9.0 Hz) & 7.57* (d, *J* = 8.6 Hz), 1H in total], 7.40 – 7.46 (m, 2H), [7.70** (t, *J* = 7.3 Hz) & 7.78* (t, *J* = 8.1 Hz), 1H in total], [8.28* (d, *J* = 5.5 Hz) & 8.31** (d, *J* = 5.2 Hz), 1H in total]; *Note:* For this compound, the spectral width of the ^{1}H NMR experiment was insufficient to observe the NH proton, which typically resonates at ~12.5 ppm in $CDCl_3$. **^{13}C NMR** (101 MHz, $CDCl_3$) δ_C (1.2:1 mixture of Boc rotamers; **indicates resolved *major* rotamer; *indicates resolved *minor* rotamer) 28.6, 43.6*, 43.9**, 46.3, 51.5, 55.6, 61.8*, 62.5**, 80.3*, 80.8**, 111.7**, 112.8*, 114.2, 117.5, 122.5*, 122.6**, 131.9**, 132.1*, 139.0**, 139.6*, 145.5*, 145.9**, 154.1**, 154.4*, 155.5, 155.9*, 156.0, 157.0**. **HR-MS** (ESI, positive ion mode) – *m/z* for $[C_{22}H_{30}N_4O_5+H]^+$ = 431.2289. Found 431.2282.

1-(2-Pyridyl)-1-(4-methoxyanilinylcarbonyl)-4-(*tert*-butoxycarbonyl)-6-oxo-1,4-diazahexane, 4b-8

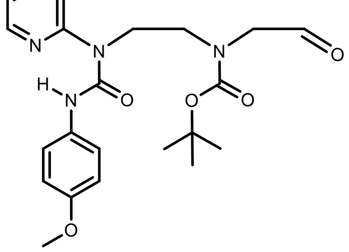

To a solution of **4b-7** (662.6 mg, 1.54 mmol, 1.0 equiv) in lab grade CH_2Cl_2 (15.4 mL) at 0 °C under air was added solid Dess-Martin periodinane (783.4 mg, 1.85 mmol, 1.2 equiv). The mixture was maintained at 0–5 °C for 3 h, then allowed to warm to room temperature over a further 3 h. The mixture was re-cooled to 0 °C and Et_2O (50 mL) was added. The suspension was gravity filtered and the reaction flask and filter cake were rinsed/washed with Et_2O (25 mL). The filtrate was washed with saturated $NaHCO_3$ (30 mL), dried (Na_2SO_4) and concentrated. The crude product was passed through a silica plug (4 mL of silica in a 10 mL syringe cartridge) with Et_2O (~30 mL) to give **4b-8** (619.1 mg, 94%) as a pale yellow foam, containing minor

impurities by NMR and used without further purification. **TLC** – R_f = 0.29–0.64 [streaks] (60:40 EtOAc/petroleum ether). **^{1}H NMR** (400 MHz, $CDCl_3$) δ_H (1.8:1 mixture of Boc rotamers; **indicates resolved *major* rotamer; *indicates resolved *minor* rotamer) [1.42** (s) & 1.48* (s), 9H in total], 3.56 – 3.64 (m, 2H), 3.78 (s, 3H), 4.05 – 4.13 (m, 4H), 6.86 (d, *J* = 9.0 Hz, 2H), 6.97 – 7.03 (m, 1H), [7.22* (d, *J* = 8.6 Hz) & 7.53** (d, *J* = 8.6 Hz), 1H in total], 7.38 – 7.44 (m, 2H), [7.68 – 7.73* (m) & 7.79** (ddd, *J* = 9.0, 7.3, 2.0 Hz), 1H in total], [8.28** (dd, *J* = 5.1, 1.9 Hz) & 8.32* (dd, *J* = 5.0, 2.0 Hz), 1H in total], [9.56** (s) & 9.57* (s), 1H in total], [12.49* (s) & 12.52** (s), 1H in total]. **^{13}C NMR** (101 MHz, $CDCl_3$) δ_C (1.8:1 mixture of Boc rotamers; **indicates resolved *major* rotamer; *indicates resolved *minor* rotamer) 28.3**, 28.5*, 43.5**, 43.7*, 46.4*, 47.0**, 55.6, 58.2*, 59.0**, 81.1**, 81.2*, 111.7*, 112.5**, 114.2, 117.6, 122.56**, 122.63*, 131.8*, 132.0**, 139.1*, 139.6**, 145.6**, 146.0*, 154.0*, 154.2**, (155.4, 155.49, 155.55; unable to assign major/minor rotamers in this region due to overlapping signal/s), 156.0**, 156.1*, 198.2. **HR-MS** (ESI, positive ion mode) – *m/z* for $[C_{22}H_{28}N_4O_5+H]^+$ = 429.2132. Found 429.2132.

1-(2-Pyridyl)-1-(4-methoxyanilinylcarbonyl)-4-(*tert*-butoxycarbonyl)-10-trifluoroacetyl-1,4,7,10-tetraazadecane, 4b-9

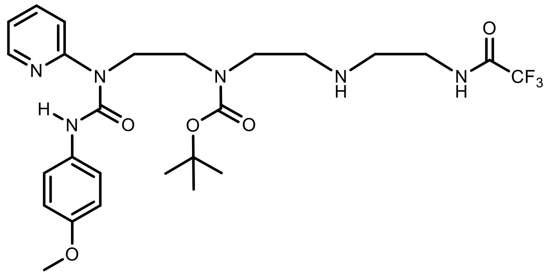

To a reaction vial containing neat aldehyde **4b-8** (230.7 mg, 0.54 mmol, 1.0 equiv) was added sequentially amine·HCl salt **4b-2** (155.5 mg, 0.81 mmol, 1.5 equiv), lab grade 1,2-dichloroethane (1.8 mL), Et_3N (0.11 mL, 0.81 mmol, 1.5 equiv) and solid $NaBH(OAc)_3$ (159.8 mg, 0.75 mmol, 1.4 equiv). The mixture was stirred at room temperature under air for 4 h. Saturated $NaHCO_3$ (15 mL) was added and the product was extracted with CH_2Cl_2 (30 mL + 20 mL) then the combined organic extracts were dried (Na_2SO_4) and concentrated. Flash chromatography (Biotage, 10 g Sfär Duo column, MeOH/CH_2Cl_2 gradient from 0:100 to 10:90) gave **4b-9** (162.0 mg, 53%) as a colourless gum. **TLC** – R_f = 0.38 (7.5:92.5 MeOH/CH_2Cl_2). **^{1}H NMR** (400 MHz, $CDCl_3$) δ_H (~1:1 mixture of Boc rotamers; *indicates resolved rotamers) [1.49* (s) & 1.53* (s), 9H in total], 2.79 – 2.88 (m, 4H), 3.38 – 3.55 (m, 6H), 3.80 (s, 3H), 4.11 – 4.20 (m, 2H), 6.87 (d, *J* = 9.0 Hz, 2H), 6.97 – 7.04 (m, 1H), 7.41 (d, *J* = 8.6 Hz, 2H), 7.62 – 7.74 (m, 2H), 7.79 – 7.85* (m, 0.5H), 8.27 – 8.34 (m, 1H), [12.59* (s) & 12.72* (s), 1H in total]; *Note:* An ArH signal of one rotamer (of integration 0.5H) is likely obscured by the $CHCl_3$ resonance. **^{19}F NMR** (376 MHz, $CDCl_3$) δ_F (~1:1 mixture of resolved Boc rotamers) [–75.7 (s) & –75.6 (s), 3F in total].

1-(2-Pyridyl)-1,4,7-tris(4-methoxyanilinylcarbonyl)-1,4,7,10-tetraazadecane, 4b-10

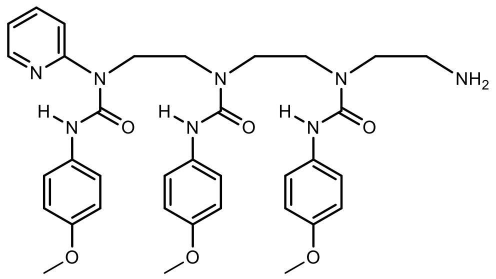

***Step 1** (Boc deprotection):* To a solution of **4b-9** (162.0 mg, 0.28 mmol, 1.0 equiv) in lab grade CH_2Cl_2 (2.85 mL) at room temperature under air was added TFA (0.65 mL, 8.55 mmol, 30.0 equiv) and the mixture was stirred at room temperature for 16 h. After cooling to 0 °C, saturated $NaHCO_3$ (20 mL) was slowly added to the open flask over 5 min with stirring. The product was extracted with CH_2Cl_2 (2 × 25 mL + 15 mL) and the combined organic extracts were dried (Na_2SO_4) and concentrated to give the crude product (151.0 mg, 113% of theoretical) as a brown oil. **TLC** – R_f = 0.40 (10:2:88 MeOH/[35% aqueous NH_3]/CH_2Cl_2). ***Step 2** (bis-urea formation):* The crude product from *Step 1* (≤0.28 mmol, 1.0 equiv) was taken up in lab grade CH_2Cl_2 (1.0 mL) and a solution of 4-methoxyphenyl isocyanate (89.2 mg, 0.60 mmol, 2.1 equiv) in CH_2Cl_2 (1.8 mL) was added. The mixture was stirred at room temperature under air for 2.5 h. MeOH (3 mL) was added and the mixture was stirred for 30 min to quench any unreacted isocyanate (potentially producing the corresponding 'non-polar' methyl carbamate as a by-product). The mixture was concentrated *in vacuo*. Flash chromatography (Biotage, 10 g Sfär Duo column, MeOH/CH_2Cl_2 gradient from 0:100 to 6.5:93.5) gave the desired product (174.8 mg, 80% over two steps) as a white solid. **TLC** – R_f = 0.35 (5:95 MeOH/CH_2Cl_2). ***Step 3** (trifluoroacetamide hydrolysis):* The product from *Step 2* (174.8 mg, 0.23 mmol, 1.0 equiv) was dissolved in lab grade THF (1.14 mL) containing a small amount of DMF for solubility (8 drops from a Pasteur pipette). To this was added EtOH (2.3 mL) and a solution of NaOH (62.0 mg, 1.82 mmol, 8.0 equiv in water (1.14 mL). The mixture was stirred at room temperature under air for 24 h. Most of the solvents were removed *in vacuo*, then water (20 mL) was added. The product was extracted with CH_2Cl_2 (40 mL + 20 mL) then the combined organic extracts were dried (Na_2SO_4) and concentrated. Flash chromatography (Biotage, 10 g Sfär Duo column, MeOH/[35% aqueous NH_3]/CH_2Cl_2 gradient from 0:0:100 to 10:2:88) gave **4b-10** (133.4 mg, 70% over three steps) as a white solid. **TLC** – R_f = 0.50 (10:2:88 MeOH/[35% aqueous NH_3]/CH_2Cl_2). **^{1}H NMR** (400 MHz, $CDCl_3$) δ_H 2.86 – 2.94 (m, 2H), 3.32 – 3.50 (m, 4H), 3.57 – 3.70 (m, 4H), 3.75 (s, 3H), 3.77 (s, 3H), 3.78 (s, 3H), 4.06 – 4.14 (m, 2H), 6.77 – 6.91 (m, 6H), 6.97 (t, *J* = 6.1 Hz, 1H), 7.32 (d, *J* = 8.5 Hz, 2H), 7.47 (d, *J* = 8.4 Hz, 2H), 7.61 (d, *J* = 8.6 Hz, 2H), 7.77 (t, *J* = 8.0 Hz, 1H), 8.27 (d, *J* = 4.9 Hz, 1H), 9.03 (s, 1H), 10.10 (bs, 1H), 12.62 (bs, 1H); *Note:* One signal of the pyridine ring (ArH3) is too broad to be identified. **^{13}C NMR** (101 MHz, $CDCl_3$) δ_C 41.8, 44.3, 46.5, 47.0, 48.0, 52.7, 55.5 (3 × unresolved O*C*H_3), 112.8, 113.9, 114.0, 114.1, 117.6, 120.6, 121.4, 122.4, 131.9, 133.4, 133.5, 139.7, 145.4, 154.4, 155.0, 155.1, 155.3, 155.9, 156.4, 158.4. **HR-MS** (ESI, positive ion mode) – *m/z* for $[C_{35}H_{42}N_8O_6+H]^+$ = 671.3300. Found 671.3271.

1-(2-Pyridyl)-1,4,7,10-tetrakis(4-methoxyanilinylcarbonyl)-10-benzyl-1,4,7,10-tetraazadecane, 4b

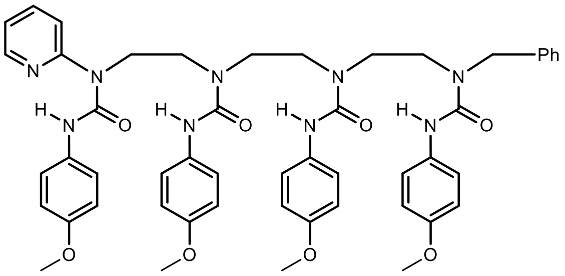

***Step 1** (reductive amination):* To a solution of **4b-10** (33.5 mg, 0.050 mmol, 1.0 equiv) in lab grade MeOH (0.5 mL) was added a solution of benzaldehyde (5.3 mg weighed, 0.050 mmol, 1.0 equiv) in lab grade THF (0.5 mL) and the mixture was stirred under air at room temperature for 16.5 h. The mixture was cooled to 0 °C and solid $NaBH_4$ (5.0 mg, 0.13 mmol, 2.6 equiv) was added. After 2 min at 0 °C, the ice bath was removed and the mixture was stirred at room temperature for 3 h. 1 M K_2CO_3 (5 mL) and water (15 mL) were added. The product was extracted with CH_2Cl_2 (25 mL + 15 mL) then the combined organic extracts were dried (Na_2SO_4) and concentrated. Flash chromatography (Biotage, 5 g Sfär Duo column, MeOH/CH_2Cl_2 gradient from 0:100 to 8:92) gave the product (32.1 mg, 84%) as a white solid. **TLC** – R_f = 0.50 (10:90 MeOH/CH_2Cl_2). ***Step 2** (urea formation):* The product from *Step 1* (32.1 mg, 0.042 mmol, 1.0 equiv) was dissolved in lab grade CH_2Cl_2 (0.34 mL) and a solution of 4-methoxyphenyl isocyanate (6.3 mg, 0.042 mmol, 1.0 equiv) in CH_2Cl_2 (0.5 mL) was added. The mixture was stirred at room temperature under air for 30 min. MeOH (5 mL) was added and the mixture was stirred for 5 min to quench any unreacted isocyanate. The mixture was concentrated *in vacuo*. Flash chromatography (Biotage, 5 g Sfär Duo column, MeOH/CH_2Cl_2 gradient from 0:100 to 6:94) gave **4b** (37.5 mg, 82% over two steps) as a white solid. **TLC** – R_f = 0.49 (5:95 MeOH/CH_2Cl_2). **^{1}H NMR** (400 MHz, CD_2Cl_2) δ_H 3.41 – 3.56 (m, 8H), 3.62 – 3.68 (m, 2H), 3.77 (s, 3H), 3.79 (s, 3H), 3.796 (s, 3H), 3.801 (s, 3H), 4.04 – 4.10 (m, 2H), 4.62 (s, 2H), 6.80 – 6.92 (m, 8H), 7.09 (dd, *J* = 7.4, 5.0 Hz, 1H), 7.26 – 7.41 (m, 5H), 7.48 – 7.52 (m, 4H), 7.61 – 7.65 (m, 4H), 7.81 (ddd, *J* = 8.5, 7.3, 2.0 Hz, 1H), 8.38 (dd, *J* = 5.3, 1.9 Hz, 1H), 8.66 (very bs, 1H), 9.07 (overlapping singlets, 2H, 2 × N*H*), 12.31 (bs, 1H) ; *Note:* One signal of the pyridine ring (ArH3) is too broad to be identified. **^{13}C NMR** (126 MHz, CD_2Cl_2) δ 45.5 – 52.5 (7 × broad semi-resolved signals from N*C*H_2), 55.91, 55.92, 55.94, 55.95, 112.9, 114.3, 114.4, 114.5, 118.8, 121.6, 121.7, 122.0, 122.6, 127.7, 127.9, 129.3, 132.2, 133.8, 134.0, 140.1, 146.7, 155.0, 155.70, 155.74, 155.9, 156.6, 156.8, 157.0, 157.1. **HR-MS** (ESI, positive ion mode) – *m/z* for $[C_{50}H_{55}N_9O_8+H]^+$ = 910.4246. Found 910.4268.

Dibromomaleic anhydride, 7a-1

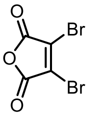

Under a dry, inert atmosphere, bromine (1.57 mL, 30.6 mmol, 2.00 equiv) was added to maleic anhydride (1500 mg, 15.3 mmol, 1.00 equiv) in a microwave vial. To this solution was added aluminium trichloride (300 mg, 2.3 mmol, 0.15 equiv) and the vial was sealed and heated to 160 °C for 20 h behind a blast shield. The suspension was cooled to room temperature and opened to air. The suspension was diluted with EtOAc (25 mL) and filtered, and the residue was washed

with EtOAc (10 mL). The filtrate was then concentrated *in vacuo* to give the desired anhydride as a white solid (2732 mg, 10.7 mmol, 70%). a Spectroscopic data matched that previously reported.[9]

Allyl dibromomaleimide, 7a-2

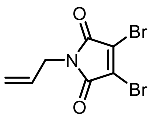

Under a dry, inert atmosphere, allylamine (0.72 mL, 9.58 mmol, 1.00 equiv, 0.10 M) was added to a solution of **7a-1** (2.45 g, 9.58 mmol, 1.00 equiv) in AcOH (96 mL). The solution was heated to reflux for 5 h, after which time the AcOH was removed by distillation. The crude product was purified by column chromatography (5-20% EtOAc:PE) to give the title maleimide as a white powder (2.51 g, 8.51 mmol, 89%). **M.P. –** 96-97 °C (DCM). **TLC** – R_f = 0.65 (SiO_2, 20:80 EtOAc:PE). **^{1}H NMR** (400 MHz, $CDCl_3$) – 4.20 (dt, J = 1.4, 5.9, 2H, NC*H*$_2$), 5.20-5.28 (m, 2H, C=C*H*$_2$), 5.79 (ddq, J = 5.9, 10.2, 17.0, 1H, C=C*H*). **^{13}C NMR** (101 MHz, $CDCl_3$) – 41.8 (*C*H$_2$), 119.1 (C=*C*H$_2$), 129.5 (2 x C=*C*Br), 130.6 (C=*C*H), 163.6 (2 x *C*O). **HR-MS** (ESI, positive ion mode) – *m/z* for $[C_7H_6Br_2NO_2+H]^+$ = 293.8765. Found 293.8760. **FTIR** (neat) – 2932, 1783, 1713.

Allyl di(*n*-butylmercapto)maleimide, 7a

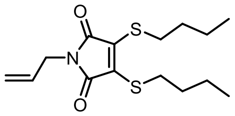

Under a dry, inert atmosphere, triethylamine (0.99 mL, 7.12 mmol, 2.10 equiv) was added dropwise to a solution of **7a-2** (1.0 g, 3.39 mmol, 1.00 equiv, 0.10 M) in anhydrous Et_2O (34 mL). To this solution was added 1-butanethiol (0.76 mL, 7.12 mmol, 2.10 equiv) dropwise and a yellow precipitate formed. The suspension was stirred at room temperature for 16 h. The suspension was then concentrated *in vacuo* and the crude product was purified by column chromatography (5-20% Et_2O:PE) to give the title bis(sulfide) as a yellow oil (814 mg, 2.60 mmol, 77%). **TLC** – R_f = 0.60 (SiO_2, 20:80 Et_2O:PE). **^{1}H NMR** (400 MHz, $CDCl_3$) δ_H 0.92 (t, J = 7.3, 6H, 2 x C*H*$_3$), 1.43-1.45 (m, 4H, 2 x C*H*$_2$), 1.60-1.63 (m, 4H, 2 x C*H*$_2$), 3.29 (t, J = 7.5, 4H, 2 x SC*H*$_2$), 4.10 (dd, J = 1.3, 5.7, 2H, NC*H*$_2$), 5.14-5.22 (m, 2H, C=C*H*$_2$), 5.79 (ddt, J = 5.9, 10.2, 15.9, 1H, C=C*H*). **^{13}C NMR** (101 MHz, $CDCl_3$) δ_C 13.7 (2 x *C*H$_3$), 21.7 (2 x *C*H$_2$), 31.6 (2 x S*C*H$_2$), 32.6 (2 x *C*H$_2$), 40.7 (N*C*H$_2$), 117.8 (C=*C*H$_2$), 131.7 (C=*C*H), 135.8 (2 x C=*C*S), 166.3 (2 x *C*O). **HR-MS** (ESI, positive ion mode) – *m/z* for $[C_{15}H_{23}NO_2S_2+H]^+$ = 314.1248. Found 314.1231. **FTIR** (neat) – 2958, 2930, 2872, 1764, 1701.

2-Oxoethyl-di(*n*-butylmercapto)maleimide, 7a-3

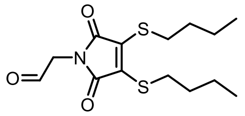

Synthesised according to general procedure L using **7a** (250 mg, 0.70 mmol). The crude product was purified by column chromatography (20-100% Et_2O:PE) to give the title aldehyde as a yellow oil (123 mg, 0.39 mmol, 56%). **TLC** – R_f = 0.08 (SiO_2, 20:80 Et_2O:PE). **^{1}H NMR** (500 MHz, $CDCl_3$) δ_H 0.92 (t, J = 7.3, 6H, 2 x C*H*$_3$), 1.41-1.44 (m, 4H, 2 x C*H*$_2$), 1.62-1.64 (m, 4H, 2 x C*H*$_2$), 3.28 (t, J = 7.4, 2 x SC*H*$_2$), 4.36 (s, 2H, NC*H*$_2$), 9.56 (s, 1H, C*H*O). **^{13}C NMR** (126 MHz, $CDCl_3$) δ_C 13.5 (2 x *C*H$_3$), 21.6 (2 x *C*H$_2$), 31.6 (2 x S*C*H$_2$), 32.5 (2 x *C*H$_2$), 47.7 (N*C*H$_2$), 136.1 (C=*C*S), 165.8 (2 x *C*O), 193.6 (*C*HO). **HR-MS** (ESI, positive ion mode) – *m/z* for $[C_{14}H_{22}NO_3S_2+H]^+$ = 316.1041. Found 316.1016. **FTIR** (neat) – 2957, 2930, 1701.

1-(2-Pyridyl)-10-((di(*n*-butylmercapto)maleimido)ethyl)-1,4,7,10-tetrakis(4-methoxyanilinylcarbonyl)-1,4,7,10-tetraazadecane, 8a

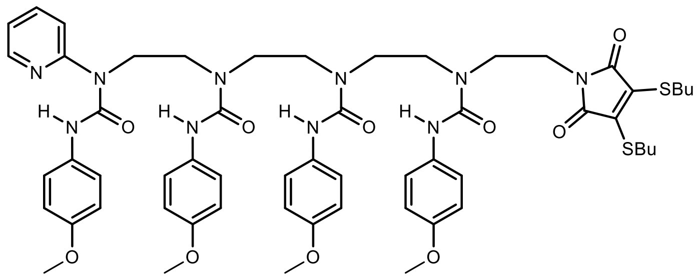

***Step 1** (reductive amination):* To a solution of amine **4b-10** (95.9 mg, 0.14 mmol, 1.0 equiv) in lab grade 1,2-dichloroethane (0.4 mL) at room temperature was added a solution of aldehyde **7a-3** (45.1 mg, 0.14 mmol, 1.0 equiv) in 1,2-dichloroethane (1.0 mL). Solid $NaBH(OAc)_3$ (42.4 mg, 0.20 mmol, 1.4 equiv) was added and the mixture was stirred at room temperature under air for 5 h. Saturated $NaHCO_3$ (8 mL) and water (15 mL) were added. The product was extracted with CH_2Cl_2 (30 mL + 10 mL) then the combined organic extracts were dried (Na_2SO_4) and concentrated. Flash chromatography (Biotage, 10 g Sfär Duo column, MeOH/CH_2Cl_2 gradient from 0:100 to 7:93) gave **8a-1** (94.0 mg, 68%) as a yellow semi-solid. **TLC** – R_f = 0.35 (5:95 MeOH/CH_2Cl_2). ***Step 2** (urea formation):* The product from *Step 1* (56.5 mg, 0.058 mmol, 1.0 equiv) was dissolved in lab grade CH_2Cl_2 (0.5 mL) and a solution of 4-methoxyphenyl isocyanate (8.7 mg, 0.058 mmol, 1.0 equiv) in CH_2Cl_2 (0.7 mL) was added. The mixture was stirred at room temperature under air for 40 min. MeOH (1 mL) was added and the mixture was stirred for 5 min to quench any unreacted isocyanate. The mixture was concentrated *in vacuo*. Flash chromatography (Biotage, 5 g Sfär Duo column, MeOH/CH_2Cl_2 gradient from 0:100 to 4:96) gave **8a** (55.5 mg, 85%) as a yellow solid. **TLC** – R_f = 0.49 (5:95 MeOH/CH_2Cl_2). **^{1}H NMR** (400 MHz, CD_2Cl_2) δ_H 0.90 (t, *J* = 7.3 Hz, 6H), 1.40 (h, *J* = 7.3 Hz, 4H), 1.60 (p, *J* = 7.7 Hz, 4H), 3.25 (t, *J* = 7.4 Hz, 4H), 3.44 – 3.60 (m, 10H), 3.68 – 3.74 (m, 4H), 3.77 (s, 3H), 3.79 (s, 3H), 3.801 (s, 3H), 3.803 (s, 3H), 4.08 – 4.14 (m, 2H), 6.79 – 6.93 (m, 8H), 7.08 (dd, *J* = 7.3, 5.0 Hz, 1H), 7.19 – 7.40 (broad m, 1H), 7.49 – 7.55 (m, 4H), 7.62 – 7.67 (m, 4H), 7.83 (ddd, *J* = 9.1, 7.3, 2.0 Hz, 1H), 8.38 (dd, *J* = 5.2, 1.9 Hz, 1H), 8.61 (very bs,

1H), 9.09 (bs, 1H), 9.12 (bs, 1H), 12.38 (bs, 1H). **^{13}C NMR** (126 MHz, CD_2Cl_2) δ_C 13.9, 22.2, 32.2, 33.0, 37.4, 45.5 – 49.8 (7 × broad semi-resolved signals from N*C*H$_2$), 55.90, 55.92, 55.94, 56.0, 113.0, 114.26, 114.30, 114.4, 114.5, 118.7, 121.6, 121.67, 121.71, 122.6, 132.3, 133.9, 134.0, 136.8, 140.2, 146.6, 155.0, 155.7, 155.8, 156.2, 156.6, 157.1, 167.4. **HR-MS** (ESI, positive ion mode) – *m/z* for $[C_{57}H_{70}N_{10}O_{10}S_2+H]^+$ = 1119.4791. Found 1119.4750.

1-(2-Pyridyl)-4-(dimethylaminocarbonyl)-4,10-diazadecane, 8b-1

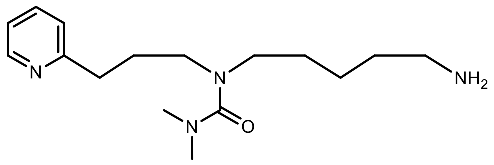

Preparation of aldehyde for reductive amination: To a solution of 3-(2-pyridyl)-1-propanol (214.1 mg, 1.56 mmol, 1.0 equiv) in lab grade CH_2Cl_2 (15.6 mL) at 0 °C under air was added solid Dess-Martin periodinane (728.2 mg, 1.72 mmol, 1.1 equiv) and the mixture was allowed to warm to room temperature over 3 h. The mixture was re-cooled to 0 °C and Et_2O (50 mL) was added. The suspension was gravity filtered and the reaction flask and filter cake were rinsed/washed with Et_2O (15 mL). The filtrate was washed with saturated $NaHCO_3$ (20 mL), dried (Na_2SO_4) and concentrated. Flash chromatography (Biotage, 5 g Sfär Duo column, MeCN/CH_2Cl_2 gradient from 0:100 to 100:0) gave 3-(2-pyridyl)propanal (41.0 mg, 19%) as a light brown oil. **TLC** – R_f = 0.11 (MeCN). **^{1}H NMR** (400 MHz, $CDCl_3$) δ_H 2.95 (tdd, *J* = 7.1, 1.3, 0.7 Hz, 2H), 3.14 (t, *J* = 7.1 Hz, 2H), 7.09 – 7.15 (m, 1H), 7.19 (d, *J* = 7.8 Hz, 1H), 7.59 (td, *J* = 7.7, 1.8 Hz, 1H), 8.51 (ddd, *J* = 4.9, 1.9, 0.9 Hz, 1H), 9.87 (t, *J* = 1.3 Hz, 1H). Spectroscopic data matches that previously reported.[10] ***Step 1** (reductive amination):* To neat 3-(2-pyridyl)propanal (41.0 mg, 0.30 mmol, 1.0 equiv) was added a solution of *N*-Boc-cadaverine (61.4 mg, 0.30 mmol, 1.0 equiv) in lab grade 1,2-dichloroethane (3 mL). Solid $NaBH(OAc)_3$ (90.0 mg, 0.42 mmol, 1.4 equiv) was added and the mixture was stirred at room temperature under air for 2 h. Aqueous K_2CO_3 (8 mL, 1.0 M) and water (15 mL) were added. The product was extracted with CH_2Cl_2 (25 mL + 15 mL) then the combined organic extracts were dried (Na_2SO_4) and concentrated. Flash chromatography (Biotage, 5 g Sfär Duo column, MeOH/[35% aqueous NH_3]/CH_2Cl_2 gradient from 0:0:100 to 8:1.6:90.4) gave a colourless gum (61.7 mg, 63% if pure) containing the desired product with a significant higher R_F impurity, presumably from di-alkylation. **TLC** – R_f (desired product) = 0.20 (10:2:88 MeOH/[35% aqueous NH_3]/CH_2Cl_2, UV or ninhydrin stain). ***Step 2** (urea formation):* The impure product from *Step 1* (61.7 mg, 0.19 mmol if pure, 1.0 equiv) was dissolved in lab grade 1,2-dichloroethane (0.96 mL) and Et_3N (54 µL, 0.38 mmol, 2.0 equiv) and dimethylcarbamoyl chloride (27 µL, 0.29 mmol, 1.5 equiv) were sequentially added. The mixture was heated at 45 °C under air (closed system: flask with septum) for 16 h. The mixture was concentrated *in vacuo*. Flash chromatography (Biotage, 5 g Sfär Duo column, MeOH/CH_2Cl_2 gradient from 0:100 to 5:95) gave the product (~9 mg, ~8% over two steps) as a colourless gum. **TLC** – R_f = 0.53 (7.5:92.5 MeOH/CH_2Cl_2). ***Step 3** (Boc deprotection):* The product from *Step 2* was dissolved in lab grade CH_2Cl_2 (0.47 mL) and TFA (54 µL, 0.70 mmol, ~30 equiv) was

added. The mixture was stirred under air at room temperature for 2 h. 1 M K_2CO_3 (5 mL) was added and the product was extracted with CH_2Cl_2 (25 mL + 15 mL). The combined organic extracts were dried (Na_2SO_4) and concentrated to give **8b-1** (7.8 mg, 9% over three steps) as a pale yellow oil. **TLC** – R_f = 0.23 (10:2:88 MeOH/[35% aqueous NH_3]/CH_2Cl_2). **^{1}H NMR** (400 MHz, $CDCl_3$) δ_H 1.22 – 1.31 (m, 2H), 1.38 – 1.55 (m, 4H), 1.92 – 2.00 (m, 2H), 2.63 – 2.70 (m, 2H), 2.73 – 2.78 (m, 2H), 2.75 (s, 6H), 3.09 – 3.20 (m, 4H), 7.10 (ddd, *J* = 7.5, 4.9, 1.2 Hz, 1H), 7.14 (dt, *J* = 7.8, 1.1 Hz, 1H), 7.58 (td, *J* = 7.6, 1.8 Hz, 1H), 8.51 (ddd, *J* = 4.9, 1.9, 1.0 Hz, 1H). **^{13}C NMR** (101 MHz, $CDCl_3$) δ_C 24.4, 28.0, 28.1, 33.6, 35.7, 38.8, 42.2, 47.7, 48.3, 121.2, 123.0, 136.5, 149.4, 161.5, 165.6. **HR-MS** (ESI, positive ion mode) – *m/z* for $[C_{16}H_{28}N_4O+H]^+$ = 293.2336. Found 293.2344.

1-(2-Pyridyl)-10-((di(*n*-butylmercapto)maleimido)ethyl)-4,10-bis(dimethylaminocarbonyl)-4,10-diazadecane, 8b

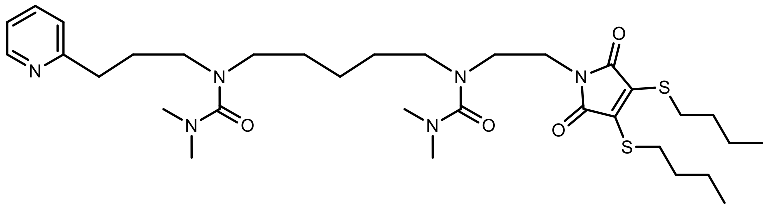

***Step 1** (reductive amination):* To a reaction vial containing amine **8b-1** (6.9 mg, 0.023 mmol, 1.0 equiv) was sequentially aldehyde **7a-3** (7.4 mg, 0.023 mmol, 1.0 equiv), lab grade 1,2-dichloroethane (0.47 mL) and solid $NaBH(OAc)_3$ (7.0 mg, 0.033 mmol, 1.4 equiv). The mixture was stirred under air at room temperature for 2 h. Saturated $NaHCO_3$ (8 mL) was added and the product was extracted with CH_2Cl_2 (25 mL + 10 mL). The combined organic extracts were dried (Na_2SO_4) and concentrated. Flash chromatography (Biotage, 5 g Sfär Duo column, MeOH/CH_2Cl_2 gradient from 0:100 to 8.5:91.5) gave the product (8.4 mg, 60%) as a yellow gum. **TLC** – R_f = 0.44 (10:90 MeOH/CH_2Cl_2). ***Step 2** (urea formation):* To the product from *Step 1* (8.4 mg, 0.014 mmol, 1.0 equiv) was added a solution containing Et_3N (2.6 mg, 0.026 mmol, 1.8 equiv) and dimethylcarbamoyl chloride (2.3 mg, 0.021 mmol, 1.5 equiv) in lab grade 1,2-dichloroethane (0.28 mL). The mixture was heated at 45 °C under air (closed system: flask with septum) for 15.5 h. The mixture was concentrated *in vacuo*. Flash chromatography (Biotage, 5 g Sfär Duo column, MeOH/CH_2Cl_2 gradient from 0:100 to 7:93) gave **8b** (9.6 mg, quantitative, or 61% over two steps) as a yellow gum. **TLC** – R_f = 0.22 (5:95 MeOH/CH_2Cl_2). **^{1}H NMR** (400 MHz, $CDCl_3$) δ_H 0.92 (t, *J* = 7.3 Hz, 6H), 1.13 – 1.22 (m, 2H), 1.38 – 1.52 (m, 8H), 1.58 – 1.66 (m, 4H), 1.92 – 2.00 (m, 2H), 2.73 – 2.77 (m, 14H, including 2 × overlapping singlets), 3.03 – 3.18 (m, 6H), 3.25 – 3.32 (m, 6H), 3.62 (t, *J* = 6.1 Hz, 2H), 7.10 (ddd, *J* = 7.5, 4.9, 1.1 Hz, 1H), 7.14 (dt, *J* = 7.8, 1.1 Hz, 1H), 7.58 (td, *J* = 7.7, 1.9 Hz, 1H), 8.51 (ddd, *J* = 4.9, 1.9, 1.0 Hz, 1H). **^{13}C NMR** (101 MHz, $CDCl_3$) δ_C 13.7, 21.8, 24.4, 27.9, 28.0, 28.1, 31.7, 32.6, 35.7, 36.8, 38.6, 38.8, 45.9, 47.8, 48.2, 49.7, 121.3, 123.0, 135.9, 136.5, 149.3, 161.5, 164.9, 165.5, 166.6. **HR-MS** (ESI, positive ion mode) – *m/z* for $[C_{33}H_{54}N_6O_4S_2+H]^+$ = 663.3721. Found 663.3704.

1,13-Bis(trifluoroacetyl)-4,7,10-tris(*n*-butylcarbonyl)-1,4,7,10,13-pentaazatridecane, 9-2

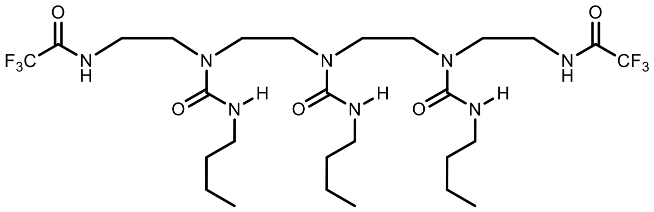

***Step 1** (bis-trifluoroacetylation):* To a solution of tetraethylenepentamine (849.4 mg, 4.49 mmol, 1.0 equiv) in lab grade CH_2Cl_2 (4.5 mL) under air at 0 °C was added slowly over 30 min a solution of ethyl trifluoroacetate (1.275 g, 8.97 mmol, 2.0 equiv) in CH_2Cl_2 (4.5 mL). After complete addition, the solution was allowed to warm to room temperature with stirring over a further 23.5 h. The mixture was concentrated *in vacuo*. Flash chromatography (manual, 25 g silica, MeOH/[35% aqueous NH_3]/CH_2Cl_2 gradient from 5:1:94 to 15:3:82) gave **9-1** (904.0 mg, 53% if pure) containing unidentified impurities by NMR. **TLC** – R_f = 0.41 (20:4:76 MeOH/[35% aqueous NH_3]/CH_2Cl_2, ninhydrin stain). ***Step 2** (tris-urea formation):* The impure product from *Step 1* (809.0 mg, 2.12 mmol if pure, 1.0 equiv) was dissolved in lab grade 1,2-dichloroethane (6.4 mL) and butyl isocyanate (0.72 mL, 6.36 mmol, 3.0 equiv) was added. The mixture was heated at 40 °C under air (closed system: flask with septum) for 16.5 h. The mixture was concentrated *in vacuo*. Flash chromatography (Biotage, 25 g Sfär Duo column, MeOH/CH_2Cl_2 gradient from 0:100 to 8:92) gave **9-2** (920.0 mg, 64%, uncorrected for impurities in the starting material) as a white solid. **MP** 156-157 °C. **TLC** – R_f = 0.27 (5:95 MeOH/CH_2Cl_2, ninhydrin stain). **^{1}H NMR** (400 MHz, $CDCl_3$) δ_H 8.55 (s, 2H, 2 × N*H*), 6.36 (t, *J* = 5.2 Hz, 1H, N*H*), 6.09 (s, 2H, 2 × N*H*), 3.39 (m, 8H, 4×C*H*$_2$N), 3.28 (m, 8H, 4 × C*H*$_2$N), 3.18 (m, 6H, 3 × C*H*$_{2\ \mathrm{butyl}}$), 1.55 – 1.45 (m, 6H, 3 × C*H*$_{2\ \mathrm{butyl}}$), 1.41 – 1.24 (m, 6H, 3 × C*H*$_{2\ \mathrm{butyl}}$), 0.91 (m, 9H, 3 × C*H*$_{3\ \mathrm{butyl}}$). **^{13}C NMR** (101 MHz, $CDCl_3$) δ_C 159.2 – 158.8 (3C, 3 × *C*=O), 158.1 (q, *J* = 37.4 Hz, 2C, 2 × *C*=OCF_3), 115.8 (q, *J* = 287.1 Hz, 2C, 2 × *C*F_3), 47.7 (2C, 2 × *C*H_2N), 47.4 (2C, 2 × *C*H_2N), 46.5 (2C, 2 × *C*H_2N), 41.0. – 40.8 (3 × *C*$H_{2\ \mathrm{butyl}}$), 40.0 (2C, 2 × *C*H_2N), 32.1 – 32.0 (3 × *C*$H_{2\ \mathrm{butyl}}$), 20.3 – 20.2 (3 × *C*$H_{2\ \mathrm{butyl}}$), 13.9 – 13.8 (3 × *C*$H_{3\ \mathrm{butyl}}$). **HR-MS** (ESI, positive ion mode) – *m/z* for $[C_{27}H_{48}F_6N_8O_5+Na]^+$ = 701.3544. Found 701.3519. **FTIR** (neat) = 3269, 3211, 3075, 2869, 2834, 1708, 1633, 1548, 1208, 1155.

4,7,10-Tris(*n*-butylcarbonyl)-1,4,7,10,13-pentaazatridecane, 9-3

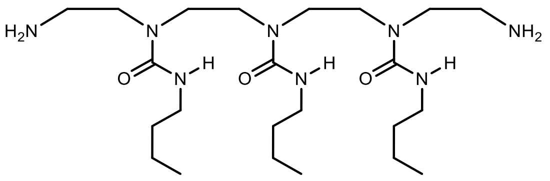

To a solution of **9-2** (920.0 mg, 1.36 mmol, 1.0 equiv) in lab grade THF (6.8 mL) and EtOH (13.6 mL) was added a solution of NaOH (433.7 mg, 10.84 mmol, 8.0 equiv) in water (6.8 mL) and the mixture was stirred at room temperature under air for 18 h. Most of the solvents were removed *in vacuo*, then water (30 mL) was added. The product was extracted with CH_2Cl_2 (2 × 30 mL + 15 mL) then the combined organic extracts were dried (Na_2SO_4) and concentrated to give **9-3** (584.1 mg, 89%) as a colourless gum. **TLC** – R_f = 0.11 (10:2:88 MeOH/[35% aqueous NH_3]/CH_2Cl_2, ninhydrin stain). **^{1}H NMR** (400 MHz, $CDCl_3$) δ_H 6.66 (3H, 3 × NH_{urea}), 3.33 – 3.08 (m, 18H, 9 × CH_2), 2.83 (t, *J* = 5.6 Hz, 4H, 2 × C$H_2$$NH_2$), 1.56 – 2.03 (m, 4H, 4 × N*H*), 1.55 – 1.41 (m, 6H, 3 × C$H_{2\ butyl}$), 1.39 – 1.26 (m, 6H, 3 × C$H_{2\ butyl}$), 0.89 (m, 9H, 3 × C$H_{3\ butyl}$). **^{13}C NMR** (101 MHz, $CDCl_3$) δ_C 160.0 (2 × *C*=O), 158.8 (*C*=O), 51.9 (2C, 2 × *C*H_2N), 47.0 (4C, 4 × *C*H_2N), 41.5 (2C, 2 × *C*H_2N), 40.8 – 40.6 (3 × *C*$H_{2\ butyl}$), 32.3 – 32.2 (3 × *C*$H_{2\ butyl}$), 20.3 (3 × *C*$H_{2\ butyl}$), 14.0 – 13.9 (3 × *C*$H_{3\ butyl}$). **HR-MS** (ESI, positive ion mode) – *m/z* for $[C_{23}H_{50}N_8O_3+H]^+$ = 487.4079. Found 487.4054. *m/z* for $[C_{23}H_{50}N_8O_3+Na]^+$ = 509.3898. Found 509.3909. **FTIR** (neat) = 3287, 2966, 2930, 2871, 1622, 1537, 1271, 1218.

1-(3,5-Bis(trifluoromethyl)anilinylcarbonyl)-4,7,10-Tris(*n*-butylcarbonyl)-1,4,7,10,13-pentaazatridecane, 9-4

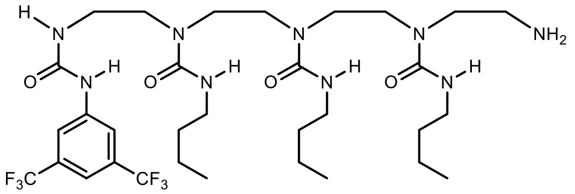

To a solution of **9-3** (340.7 mg, 0.70 mmol, 1.4 equiv) in lab grade CH_2Cl_2 (14 mL) at 0 °C under air was added dropwise over 5 min a solution of 3,5-bis(trifluoromethyl)phenyl isocyanate (127.6 mg, 0.50 mmol, 1.0 equiv) in CH_2Cl_2 (2.7 mL) and the mixture was allowed to warm to room temperature with stirring over 3.5 h. The mixture was concentrated *in vacuo*. Flash chromatography (Biotage, 10 g Sfär Duo column, MeOH/[35% aqueous NH_3]/CH_2Cl_2 gradient from 0:0:100 to 10:2:88) gave **9-4** (199.8 mg, 54%, based on the isocyanate) as a white solid. **TLC** – R_f = 0.21 (10:2:88 MeOH/[35% aqueous NH_3]/CH_2Cl_2). **^{1}H NMR** (400 MHz, $CDCl_3$) δ_H 8.96 (s, 1H, N*H*), 7.84 (s, 2H, 2 × CH_{Ar}), 7.32 (s, 1H, CH_{Ar}), 6.81 (s, 3H, N*H*), 6.55 (s, 1H, N*H*), 3.37– 3.20 (m, 10 H, 5 × CH_2), 3.20 – 3.10 (m, 8H, 4 × CH_2), 3.06 (td, *J* = 7.0, 5.3 Hz, 2H, CH_2NH), 2.77 (t, *J* = 5.0 Hz, 2H, C$H_2$$NH_2$), 1.75 – 1.0 (m, 2H, NH_2), 1.54 – 1.31 (m, 6H, 3 × CH_2), 1.31 – 1.12 (m, 6H, 3 × CH_2), 0.93 – 0.64 (m, 9H, 3 × CH_3). **^{13}C NMR** (101 MHz, $CDCl_3$) δ_C 160.7 (C=O), 159.1 (C=O), 159.0 (C=O), 156.3 (C=O), 141.8 (*C*$_{Ar}$), 132.07 (q, *J* = 33.0 Hz, 2C, 2 × *C*CF_3), 123.5 (q, *J* = 272.7 Hz, 2C, 2 × *C*F_3), 119.45 (2C, 2 × *C*H_{Ar}), 114.9 (*C*H_{Ar}), 52.6 (*C*H_2), 48.6 (*C*H_2), 47.9 (*C*H_2), 47.5 (*C*H_2), 47.4 (*C*H_2), 47.0 (*C*H_2), 41.6 (*C*H_2), 41.2 (*C*H_2),

40.9 (CH_2), 40.5 (CH_2), 39.4 (CH_2), 32.3 (CH_2), 32.1 (CH_2), 31.9 (CH_2), 20.3 – 20.2 (2C, 2 × CH_2), 13.9 – 13.8 (2C, 2 × CH_3). **HR-MS** (ESI, positive ion mode) – *m/z* for $[C_{32}H_{53}F_6N_9O_4+H]^+$ = 742.4197. Found 742.4182. **FTIR** (neat) = 3296, 2959, 2932, 2873, 1625, 1556, 1387, 1276, 1132.

1-(3,5-Bis(trifluoromethyl)anilinylcarbonyl)-4,7,10,13-tetrakis(butylaminocarbonyl)-13-((di(butylmercapto)maleimido)ethyl)-1,4,7,10,13-pentaazatridecane, 9

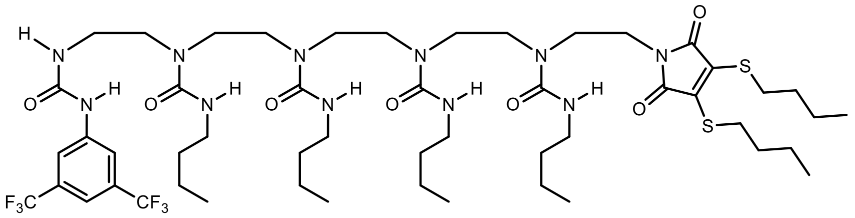

***Step 1** (reductive amination):* To a solution of amine **9-4** (74.2 mg, 0.10 mmol, 1.0 equiv) in lab grade 1,2-dichloroethane (0.2 mL) at room temperature was added a solution of aldehyde **7a-3** (31.5 mg, 0.10 mmol, 1.0 equiv) in 1,2-dichloroethane (0.8 mL). Solid $NaBH(OAc)_3$ (29.7 mg, 0.14 mmol, 1.4 equiv) was added and the mixture was stirred at room temperature under air for 2 h. Saturated $NaHCO_3$ (8 mL) and water (15 mL) were added. The product was extracted with CH_2Cl_2 (30 mL + 10 mL) then the combined organic extracts were dried (Na_2SO_4) and concentrated. Flash chromatography (Biotage, 10 g Sfär Duo column, MeOH/CH_2Cl_2 gradient from 0:100 to 7.5:92.5) gave **9-5** (55.8 mg, 54%) as a yellow semi-solid. **TLC** – R_f = 0.14 (5:95 MeOH/CH_2Cl_2). ***Step 2** (urea formation):* The product from *Step 1* (55.8 mg, 0.054 mmol, 1.0 equiv) was dissolved in lab grade CH_2Cl_2 (0.3 mL) and a solution of butyl isocyanate (5.3 mg, 0.054 mmol, 1.0 equiv) in CH_2Cl_2 (0.8 mL) was added. The mixture was stirred at room temperature under air for 2 h. The solvent was removed *in vacuo* to give **9** (59.2 mg, 97%, or 52% over two steps) as a yellow solid. **M.P.** 165-166 °C ($CHCl_3$/PE). **TLC** – R_f = 0.36 (5:95 MeOH/CH_2Cl_2). **^{1}H NMR** (500 MHz, CD_2Cl_2) δ_H 0.86 – 0.94 (m, 18H), 1.31 – 1.46 (m, 12H), 1.47 – 1.56 (m, 8H), 1.60 (p, *J* = 7.4 Hz, 4H), 3.15 – 3.43 (m, 30H), 3.63 (t, *J* = 6.8 Hz, 2H), 6.40 – 7.02 (overlapping broad singlets, 4H, 4 × N*H*), 7.43 (s, 1H), 8.00 (s, 2H), 9.05 (bs, 1H). *Note:* One NH resonance, presumably the NH at the N terminus proximal to the maleimide, was not observed. **^{13}C NMR** (126 MHz, CD_2Cl_2) δ_C 13.9, 14.06, 14.15, 14.2, 20.675, 20.685, 20.71, 22.2, 32.1, 32.5, 32.56, 32.63, 32.65, 33.0, 37.3, 40.0, 41.2, 41.3, 41.38, 41.44, 46.6, 47.7, 48.0, 48.2, 48.3, 48.9, 115.0, 118.3, 124.1 (q, *J* = 272.5 Hz), 132.2 (q, *J* = 32.9 Hz), 136.6, 142.5, 156.7, 158.8, 159.36, 159.40, 159.5, 167.4. **^{19}F NMR** (377 MHz, $CDCl_3$) δ_F −64.6 (s, 6F, 2 × C*F*$_3$). **HR-MS** (ESI, positive ion mode) – *m/z* for $[C_{51}H_{83}F_6N_{11}O_7S_2+Na]^+$ = 1162.5720. Found 1162.5765. **FTIR** (neat) – 3294, 3211, 2995, 1761, 1633.

1-Trifluoroacetyl-4,7,10-tris(*n*-butylcarbonyl)-1,4,7,10,13-pentaazatridecane, 10-1

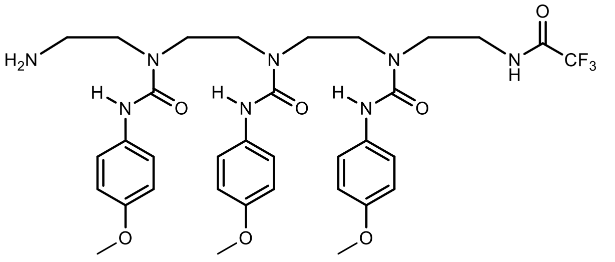

To a solution of **5b-2** (636.8 mg, 1.00 mmol, 1.0 equiv) in lab grade CH_2Cl_2 (1.0 mL) at 0 °C under N_2 was added over 75 min a solution of ethyl trifluoroacetate (142.1 mg, 1.00 mmol, 1.0 equiv) in CH_2Cl_2 (1.0 mL), while maintaining the temperature at 0–5 °C. After complete addition, the mixture was allowed to warm to room temperature and stirred for a further 2 h and 15 min. The reaction mixture (including solvent) was directly subjected to flash chromatography (Biotage, 10 g Sfär Duo column, MeOH/CH_2Cl_2 gradient from 1:99 to 10:90) to give **10-1** (412.7 mg, 56%) as a white solid. **TLC** – R_f = 0.32 (10:2:88 MeOH/[35% aqueous NH_3]/CH_2Cl_2). **^{1}H NMR** (400 MHz, DMSO-d_6) δ_H 2.80 (t, J = 5.8 Hz, 2H), 3.31 – 3.51 (m, 14H), 3.70 (overlapping singlets, 6H, 2 × OCH_3), 3.71 (s, 3H, 1 × OCH_3), 6.80 – 6.87 (m, 6H), 7.34 (d, J = 9.0 Hz, 2H), 7.39 (d, J = 8.8 Hz, 2H), 7.46 (d, J = 9.0 Hz, 2H), 8.56 (bs, 1H), 8.94 (bs, 1H), 9.50 (bs, 1H). **^{13}C NMR** (101 MHz, DMSO-D_6) δ_C 38.4, 40.6, 45.9 – 46.8 (6 × broad semi-resolved signals from NCH$_2$), 55.16 (2 × unresolved OCH$_3$), 55.20 (1 × OCH$_3$), 113.56, 113.64, 113.7, 116.0 (q, J = 288.1 Hz), 121.2, 121.3, 121.6, 133.4, 133.5, 154.5, 154.6, 155.6, 155.9, 156.7, 156.8; *Note:* the signal to noise ratio was too low to clearly observe the CO resonance (quartet). **^{19}F NMR** (376 MHz, DMSO-D_6) δ_F –74.4 (s, 3F). **HR-MS** (ESI, positive ion mode) – *m/z* for $[C_{34}H_{43}F_3N_8O_7+H]^+$ = 733.3280. Found 733.3277.

1-(2-Pyridyl)-1,10,13,16-tetrakis(4-methoxyanilinylcarbonyl)-4-(*tert*-butoxycarbonyl)-19-trifluoroacetyl-1,4,7,10,13,16,19-heptaazanonadecane, 10-2

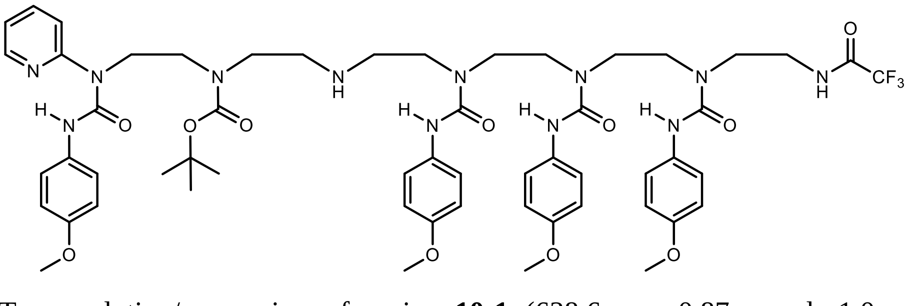

To a solution/suspension of amine **10-1** (638.6 mg, 0.87 mmol, 1.0 equiv) in lab grade 1,2-dichloroethane (5.0 mL) at room temperature under air was added a solution of aldehyde **4b-8** (373.4 mg, 0.87 mmol, 1.0 equiv) in 1,2-dichloroethane (3.7 mL). After stirring for 10 min, solid NaBH(OAc)$_3$ (258.6 mg, 1.22 mmol, 1.4 equiv) was added and the mixture was stirred under N_2 at room temperature for 5 h. Saturated $NaHCO_3$ (25 mL) and water (15 mL) were added. The product was extracted with CH_2Cl_2 (40 mL + 2 × 20 mL) then the combined organic extracts were washed with saturated $NaHCO_3$/brine (1:1, 30 mL), dried (Na_2SO_4) and concentrated. Flash chromatography (Biotage, 25 g Sfär Duo column, MeOH/CH_2Cl_2 gradient from 1:99 to 10:90) gave **10-2** (609.9 mg, 61%) as an off-

white amorphous solid. **TLC** – R_f = 0.31 (7.5:92.5 MeOH/CH_2Cl_2). **^{1}H NMR** (400 MHz, $CDCl_3$) δ_H (~1:1 mixture of Boc rotamers; *indicates resolved rotamers) 1.47 (s, 9H), 2.83 – 2.98 (m, 4H), 3.34 – 3.56 (m, 18H), 3.72 – 3.79 (overlapping singlets, 12H, 4 × OCH_3), 4.02 – 4.13 (m, 2H), 6.77 – 6.88 (m, 8H), 6.95 – 7.03 (m, 1H), 7.15 – 7.82 (m, 10H), 8.24 – 8.33 (m, 1H), 8.67 – 8.79 (broad m, 1H), 9.12 (bs, 2H), [9.77* (bs) & 9.87* (bs), 1H in total], [12.56* (bs) & 12.62* (bs), 1H in total]. **^{19}F NMR** (376 MHz, $CDCl_3$) δ_F –75.8 (s, 3F). **HR-MS** (ESI, positive ion mode) – *m/z* for $[C_{56}H_{71}F_3N_{12}O_{11}+Na]^+$ = 1167.5210. Found 1167.5184.

1-(2-Pyridyl)-1,4,7,10,13,16-hexakis(4-methoxyanilinylcarbonyl)-1,4,7,10,13,16,19-heptaazanonadecane, **10-3**

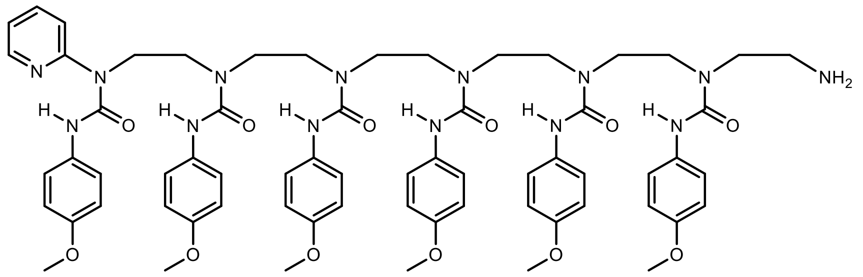

***Step 1** (Boc deprotection):* To a solution of **10-2** (609.9 mg, 0.53 mmol, 1.0 equiv) in lab grade CH_2Cl_2 (5.3 mL) at room temperature under air was added TFA (1.22 mL, 15.98 mmol, 30.0 equiv) and the mixture was stirred at room temperature for 24 h. After cooling to 0 °C, saturated $NaHCO_3$ (25 mL) was slowly added to the open flask over 10 min with stirring. The product was extracted with CH_2Cl_2 (2 × 20 mL) and the combined organic extracts were dried (Na_2SO_4) and concentrated. Flash chromatography (Biotage, 10 g Sfär Duo column, MeOH/[35% aqueous NH_3]/EtOAc gradient from 1:0.2:98.8 to 10:2:88) gave the product (484.8 mg, 87% if pure) as an off-white semi-solid contaminated with ~20 wt % acetamide, presumably arising from the chromatography eluent mixture. **TLC** – R_f = 0.19 (10:2:88 MeOH/[35% aqueous NH_3]/EtOAc). ***Step 2** (bis-urea formation):* The impure product from *Step 1* (484.8 mg, 0.46 mmol if pure, 1.0 equiv) was taken up in lab grade CH_2Cl_2 (3.0 mL) and a solution of 4-methoxyphenyl isocyanate (138.4 mg, 0.93 mmol, 2.0 equiv) in CH_2Cl_2 (1.6 mL) was added. The mixture was stirred at room temperature under air for 2 h. MeOH (5 mL) was added and the mixture was stirred for 2 h to quench any unreacted isocyanate (potentially producing the corresponding 'non-polar' methyl carbamate as a by-product). The mixture was concentrated *in vacuo*. Flash chromatography (Biotage, 10 g Sfär Duo column, MeOH/CH_2Cl_2 gradient from 1:99 to 10:90) gave the product (514.7 mg, 72% over two steps) as an off-white solid. **TLC** – R_f = 0.42 (7.5:92.5 MeOH/CH_2Cl_2). ***Step 3** (trifluoroacetamide hydrolysis):* The product from *Step 2* (514.7 mg, 0.38 mmol, 1.0 equiv) was dissolved in a mixture of lab grade THF (1.9 mL) and EtOH (3.9 mL). A solution of NaOH (104.2 mg, 3.06 mmol, 8.0 equiv) in water (1.9 mL) was added and the mixture was stirred at room temperature under air for 16 h. Most of the solvents were removed *in vacuo*, then water (30 mL) was added. The product was extracted with CH_2Cl_2 (60 mL + 20 mL) then the combined organic extracts were dried

(Na_2SO_4) and concentrated. Flash chromatography (Biotage, 10 g Sfär Duo column, MeOH/CH_2Cl_2 gradient from 0:100 to 10:90 then MeOH/[35% aqueous NH_3]/CH_2Cl_2 10:2:88) gave **10-3** (424.5 mg, 64% over three steps) as a white solid. **TLC** – R_f = 0.32 (10:90 MeOH/CH_2Cl_2). **^{1}H NMR** (400 MHz, $CDCl_3$) δ_H 2.85 – 2.92 (m, 2H), 3.28 – 3.72 (m, 20H), 3.75 – 3.81 (overlapping singlets, 18H, 6 × OC*H*$_3$), 4.01 – 4.14 (m, 2H), 6.79 – 6.91 (m, 12H), 7.00 (dd, *J* = 7.5, 4.9 Hz, 1H), 7.31 (d, *J* = 8.7 Hz, 2H), 7.48 (d, *J* = 8.6 Hz, 2H), 7.60 – 7.68 (m, 8H), 7.76 (t, *J* = 7.9 Hz, 1H), 8.30 (d, *J* = 3.9 Hz, 1H), 9.10 – 9.26 (overlapping broad singlets, 4H, 4 × N*H*), 10.23 (bs, 1H), 12.65 (bs, 1H); *Note:* One signal of the pyridine ring (ArH3) is too broad to be identified. **^{13}C NMR** (101 MHz, $CDCl_3$) δ_C 41.9, 47.0, 47.3 – 49.0 (9 × broad semi-resolved signals from N*C*H$_2$), 53.0, 55.6 (6 × unresolved O*C*H$_3$), 112.6, 113.9, 114.0, 114.1, 114.2, 118.1, 120.7, 121.0, 121.2, 121.5, 122.4, 131.8, 133.2, 133.4, 133.5, 133.6, 140.0, 145.6, 154.7, 155.2, 155.3, 156.1, 156.56, 156.62, 156.7, 158.7. **HR-MS** (ESI, positive ion mode) – *m/z* for $[C_{65}H_{78}N_{14}O_{12}+H]^+$ = 1247.5996. Found 1247.5962.

1-(2-Pyridyl)-19-((di(*n*-butylmercapto)maleimido)ethyl)-1,4,7,10,13,16,19-heptakis(4-methoxyanilinylcarbonyl)-1,4,7,10,13,16,19-heptaazanonadecane, 10

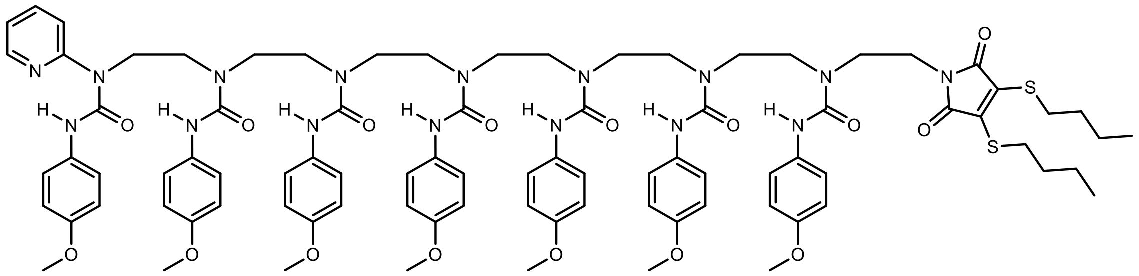

Step 1 *(reductive amination):* To a solution of amine **10-3** (187.1 mg, 0.15 mmol, 1.0 equiv) in lab grade 1,2-dichloroethane (0.5 mL) at room temperature was added a solution of aldehyde **7a-3** (47.3 mg, 0.15 mmol, 1.0 equiv) in 1,2-dichloroethane (1.0 mL). Solid $NaBH(OAc)_3$ (44.5 mg, 0.21 mmol, 1.4 equiv) was added and the mixture was stirred at room temperature under air for 2 h. Saturated $NaHCO_3$ (5 mL) and water (15 mL) were added. The product was extracted with CH_2Cl_2 (25 mL + 15 mL) then the combined organic extracts were dried (Na_2SO_4) and concentrated. Flash chromatography (Biotage, 10 g SNAP Ultra column, MeOH/CH_2Cl_2 gradient from 0:100 to 7.5:92.5) gave **10-4** (163.9 mg, 71%) as a yellow solid. **TLC** – R_f = 0.32 (7.5:92.5 MeOH/CH_2Cl_2). ***Step 2*** *(urea formation):* The product from *Step 1* (163.9 mg, 0.11 mmol, 1.0 equiv) was dissolved in lab grade CH_2Cl_2 (0.5 mL) and a solution of 4-methoxyphenyl isocyanate (15.8 mg, 0.11 mmol, 1.0 equiv) in CH_2Cl_2 (0.6 mL) was added. The mixture was stirred at room temperature under air for 1 h. MeOH (5 mL) was added and the mixture was stirred for 15 min to quench any unreacted isocyanate. The mixture was concentrated *in vacuo*. Flash chromatography (Biotage, 5 g Sfär Duo column, MeOH/CH_2Cl_2 gradient from 0:100 to 7:93) gave **10** (172.5 mg, 96%) as a yellow solid. *Note:* For this longer oligomer, slow/medium exchange between two principal conformers was observed at room temperature by ^{1}H NMR, as indicated by general peak broadening and partial splitting of the NH resonance at ~12.5 ppm (proximal

to the pyridine). Coalescence was observed by ^{1}H NMR upon heating to 55 °C (see the VT NMR section). **TLC** – R_f = 0.38 (5:95 MeOH/CH_2Cl_2). **^{1}H NMR** (400 MHz, $CDCl_3$, 25 °C) δ_H 0.89 (t, *J* = 7.3 Hz, 6H), 1.39 (h, *J* = 7.4 Hz, 4H), 1.58 (p, *J* = 7.5 Hz, 4H), 3.22 (t, *J* = 7.4 Hz, 4H), 3.32 – 3.69 (m, 26H), 3.74 – 3.82 (overlapping singlets, 21H, 7 × OCH_3), 3.98 – 4.14 (m, 2H), 6.77 – 6.92 (m, 14H), 7.00 (t, *J* = 6.2 Hz, 1H), 7.45 – 7.51 (m, 4H), 7.58 – 7.75 (m, 11H), 8.26 – 8.32 (m, 1H), 8.94 – 9.29 (overlapping broad singlets, 5H, 5 × N*H*), 12.10 – 12.90 (two overlapping broad singlets from partially decoalesced rotamers, 1H in total, N*H* proximal to pyridine). *Note:* Two resonances could not be identified, presumably due to extensive peak broadening/chemical exchange as observed previously for analogous protons in smaller homologues: 1) ArH3 of the pyridine ring and 2) the NH at the C-terminus, proximal to the maleimide. **^{13}C NMR** (101 MHz, $CDCl_3$) δ_C 13.6, 21.7, 31.7, 32.4, 36.7, 46.1 – 49.3 (13 × broad semi-resolved signals from NCH_2), 55.5 (7 × unresolved OCH_3), 112.5, 113.9, 113.98, 114.04, 114.1, 118.1, 121.1, 121.4, 122.1, 131.7, 131.9, 133.2, 133.36, 133.44, 135.9, 139.9, 145.7, 154.5, 155.1, 155.17, 155.24, 155.8, 156.0, 156.5, 156.6, 166.9. **HR-MS** (Nanospray, positive ion mode) – *m/z* for $[C_{87}H_{106}N_{16}O_{16}S_2+H]^+$ = 1695.7492. Found 1695.7474.

Supplementary Data

VT NMR Experiments

All low-temperature VT NMR experiments were conducted using a JEOL ECS 300 spectrometer (300 MHz) or a Bruker AVANCE III HD 500 MHz NMR Spectrometer with 5 mm DCH ^{13}C–^{1}H/D Cryo Probe (500 MHz).

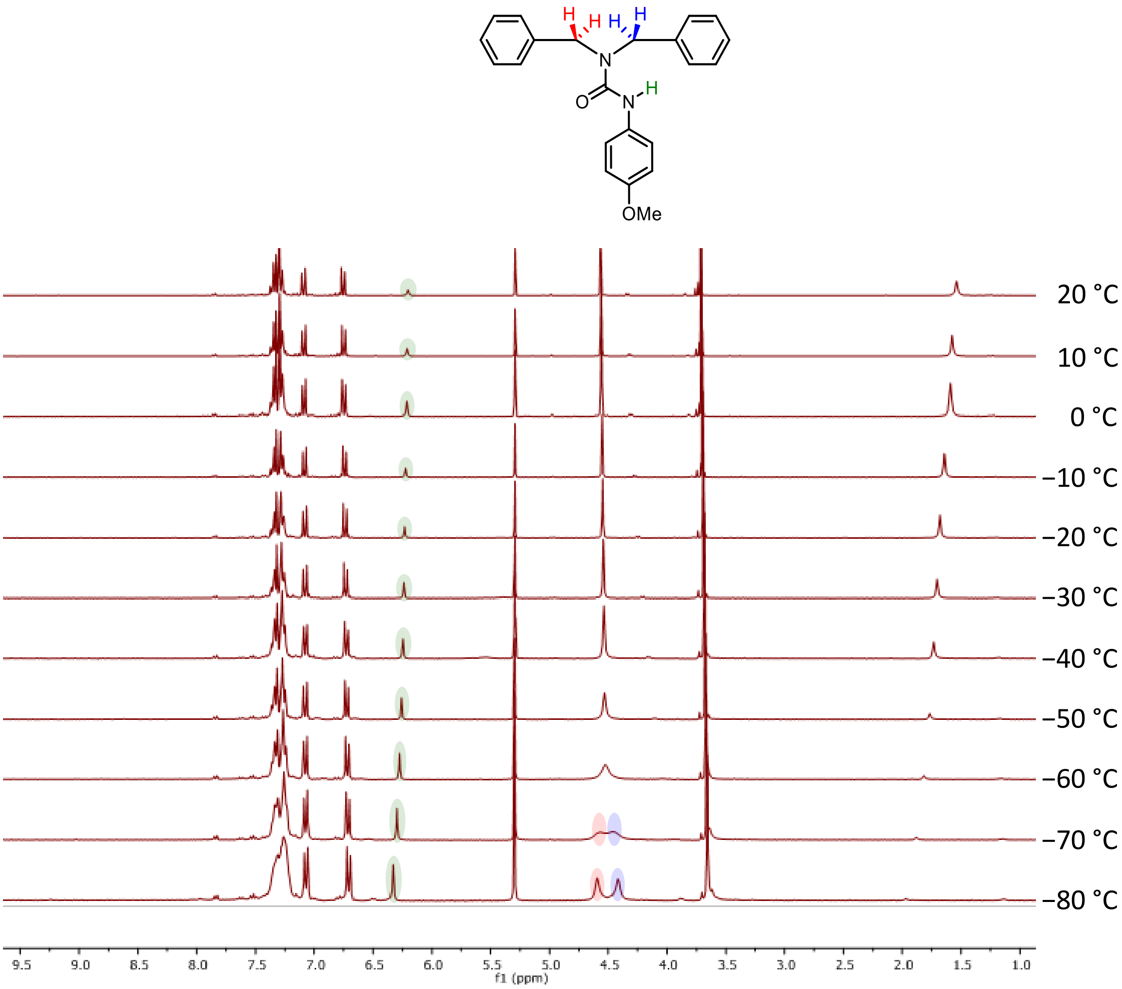

Figure S1 – VT NMR data for 1 (300 MHz, CD_2Cl_2, 29 mM). Signals at 20 °C: 4.56 (benzylic methylenes) and 6.20 ppm (ureido proton) at 20 °C. Upon cooling, the signal at 4.56 ppm is unaffected by decreasing temperature until −50 °C, where it broadens and eventually decoalesces into two singlets in a 50:50 ratio at −70 °C, which sharpen further at −80 °C. The signal at 6.20 ppm sharpens with decreasing temperature and moves downfield. These data are consistent with one populated conformation where rotation of the Bn_2N–CO bond is fast on the NMR timescale and becomes slow between −60 °C and −70 °C. The signals corresponding to the benzylic methylenes decoalesce because at slow exchange, one of the benzylic methylenes is proximate to a ureido proton, and the other is proximate to a ureido carbonyl, making them chemically inequivalent. Additionally, the downfield shift of the ureido proton with decreasing temperature is attributed to intermolecular hydrogen bonding. The assignment of the decoalesced benzylic methylene signals is arbitrary – it is unknown which methylene is proximate to the ureido carbonyl/proton.

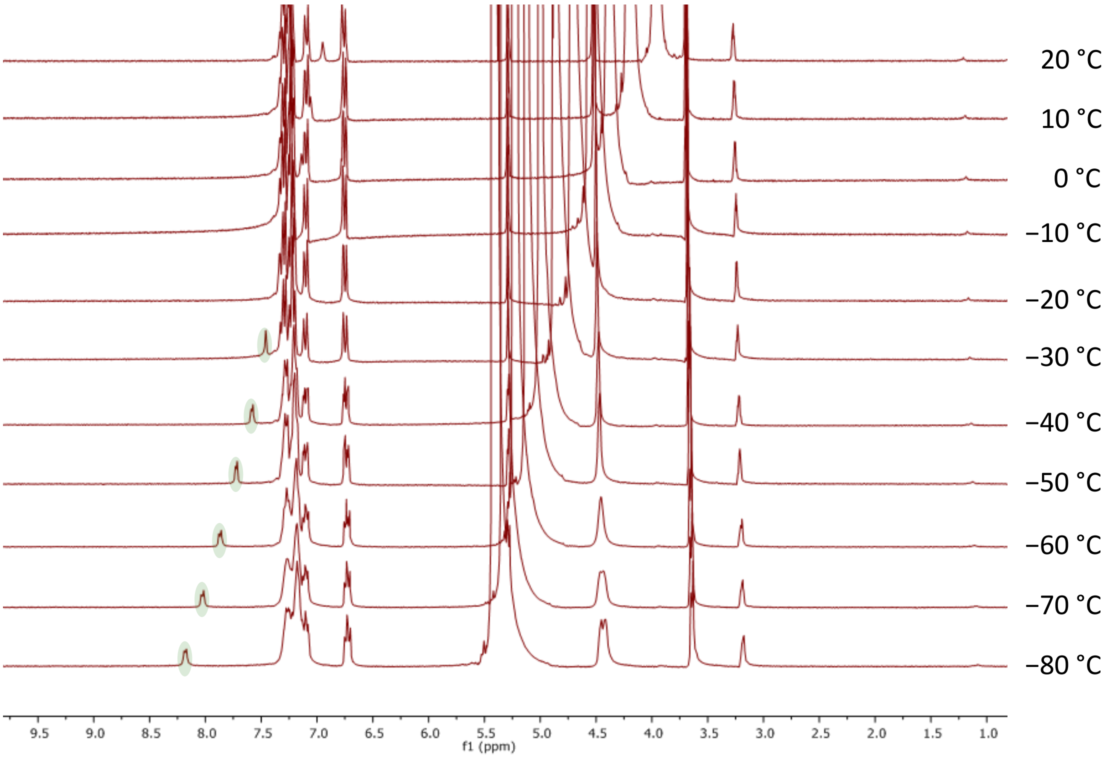

Figure S2 – VT NMR data for 1 (300 MHz, 40% CD_3OH in CD_2Cl_2, 29 mM). Signals at 20 °C: 4.52 (benzylic methylenes) and 6.95 ppm (ureido proton). Upon cooling, the signal at 4.52 ppm broadens until eventually decoalescing at −70 °C. The signal at 6.95 ppm shifts downfield with decreasing temperature. In comparison to that observed in neat CD_2Cl_2 (Figure S1), a decoalescence event is observed at the same temperature but the anisochronicity was much less pronounced in the presence of CD_3OH. The ureido proton signal is also much more downfield in the presence of CD_3OH due to intermolecular hydrogen bonding with the solvent.

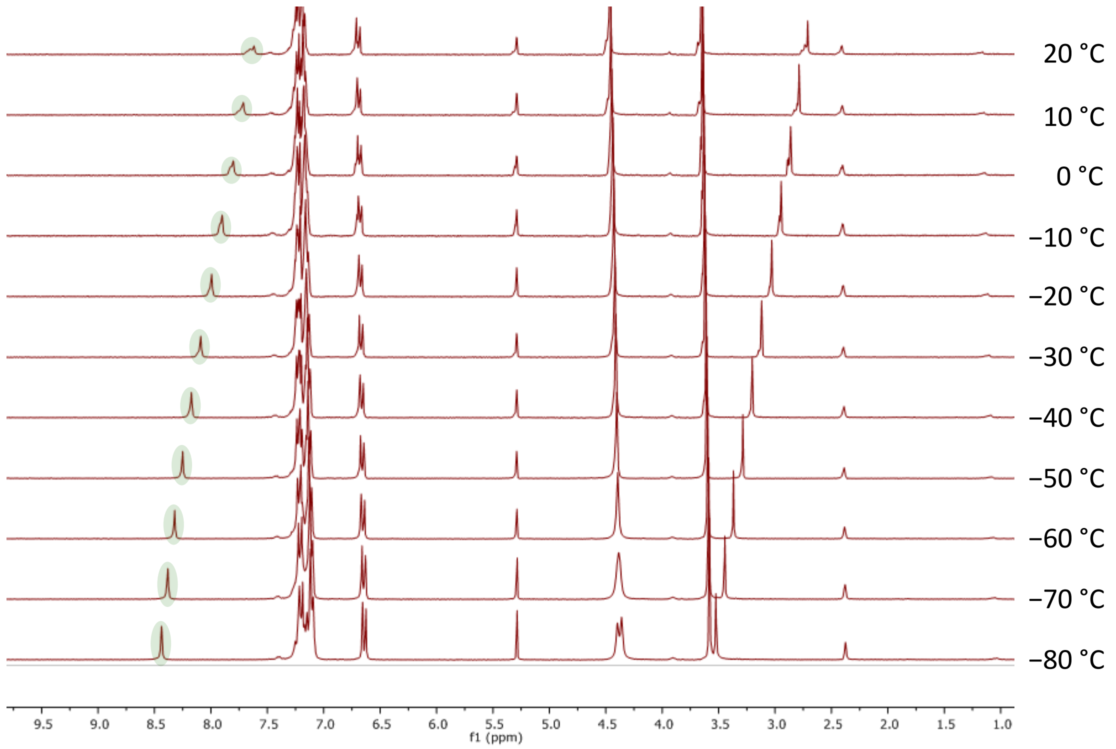

Figure S3 – VT NMR data for 1 (300 MHz, 10% $(CD_3)_2SO$ in CD_2Cl_2, 29 mM). Signals at 20 °C: 4.46 (benzylic methylenes) and 7.63 ppm (ureido proton). The signal at 4.46 ppm broadens with decreasing temperature and eventually decoalesces at −80 °C. The signal at 7.63 ppm shifts downfield and sharpens upon cooling. This shows that **1** exists as one conformation, and rotation about the Bn_2N–CO bond is slow on the NMR timescale below −80 °C. Additionally, d_6-DMSO hydrogen bonds to the ureido proton, as is demonstrated by its downfield shift with decreasing temperature.

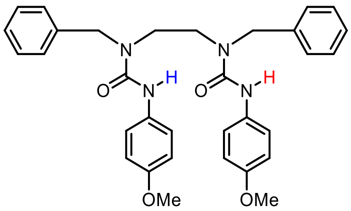

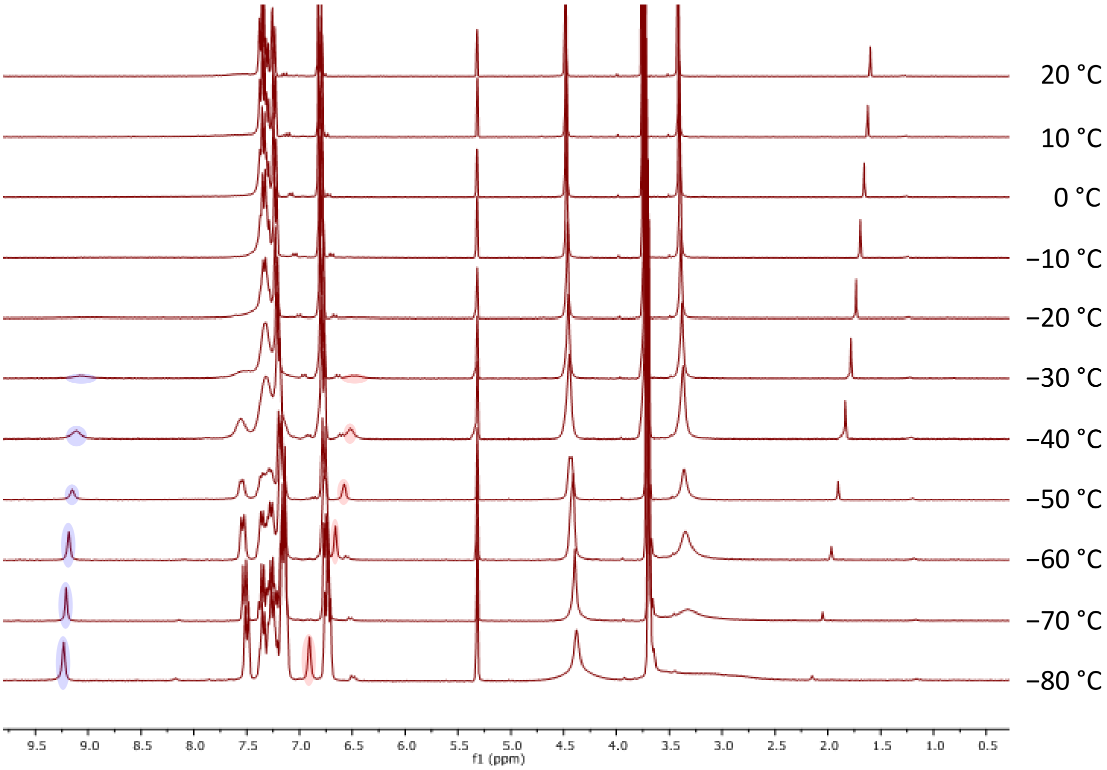

Figure S4 – VT NMR data for 2a (300 MHz, CD_2Cl_2, 19 mM). Signals at 20 °C: 4.48 ppm (benzylic methylenes). Upon cooling, the signal at 4.48 ppm broadens and decoalesces at −50 °C. The decoalesced signals broaden further with decreasing temperature. Signals appear at 6.47 (1 H) and 9.06 ppm (1 H) at −30 °C, which sharpen and shift downfield with decreasing temperature. These data are consistent with one populated conformer of uniform directionality where the two benzylic methylenes are chemically inequivalent at slow exchange. At slow exchange, one ureido proton is in hydrogen bonding (9.06 ppm at −40 °C) and the other is not (6.47 ppm at −40 °C). Triurea **3a** and tetraurea **4a** were also analysed by VT NMR (Figure S10, S16), and they were found to behave analogously to diurea **2a**, but with increasing numbers of hydrogen-bonding ureido protons.

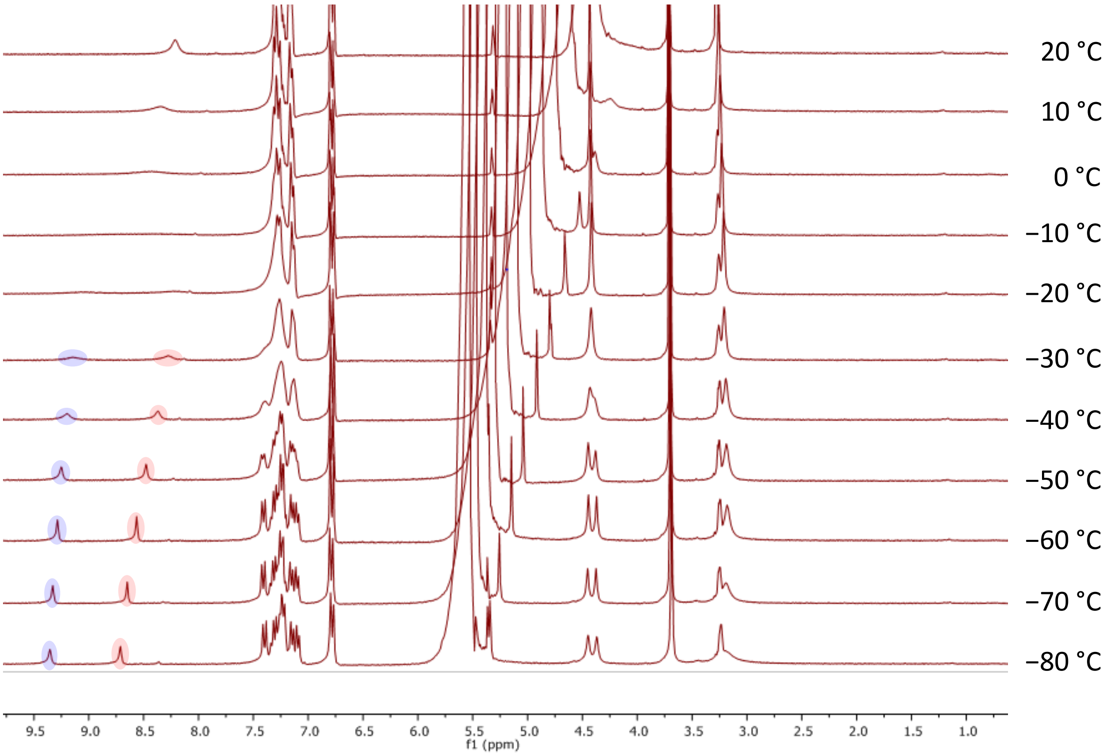

Figure S5 – VT NMR data for 2a (300 MHz, 40% CD_3OH in CD_2Cl_2, 19 mM). Signals at 10 °C (the benzylic methylene signal was obscured by the alcohol peak at 20 °C): 4.43 (benzylic methylenes) and 8.21 ppm (ureido protons). Upon cooling, the signal at 4.43 ppm broadens with decreasing temperature and decoalesces at −40 °C into two singlets in a 50:50 ratio, which sharpen upon further cooling. The signal at 8.21 ppm shifts downfield and broadens with decreasing temperature, until it decoalesces into two broad singlets (1 H each) at −30 °C. These singlets sharpen and shift further downfield with further cooling. If the CD_3OH interrupted the intramolecular hydrogen bonding, the resultant intermolecular hydrogen bond with CD_3OH would give rise to conformations where both ureas are chemically identical at slow exchange. However, decoalescence of the benzylic methylenes was still observed, indicating directional uniformity. The CD_3OH did not interrupt the intramolecular hydrogen bonding, it simply complemented it by hydrogen bonding to both ends; one through the hydroxyl oxygen and the other through the hydroxyl proton. This was further validated by the chemical shifts of the ureido protons at low temperature. At −60 °C, there is a signal at 9.29 ppm corresponding to an intramolecularly hydrogen-bonded ureido proton, which is very similar to **2a** in CD_2Cl_2 (9.18 ppm). The other ureido proton shows a signal at 8.56 ppm, which is very different to that observed in CD_2Cl_2 (6.66 ppm) due to the formation of a hydrogen bond with CD_3OH (Figure S4). Triurea **3a** and tetraurea **4a** were also analysed by VT NMR (Figure S13, S19), and they were found to behave analogously to diurea **2a**, but with increasing numbers of hydrogen-bonding ureido protons.

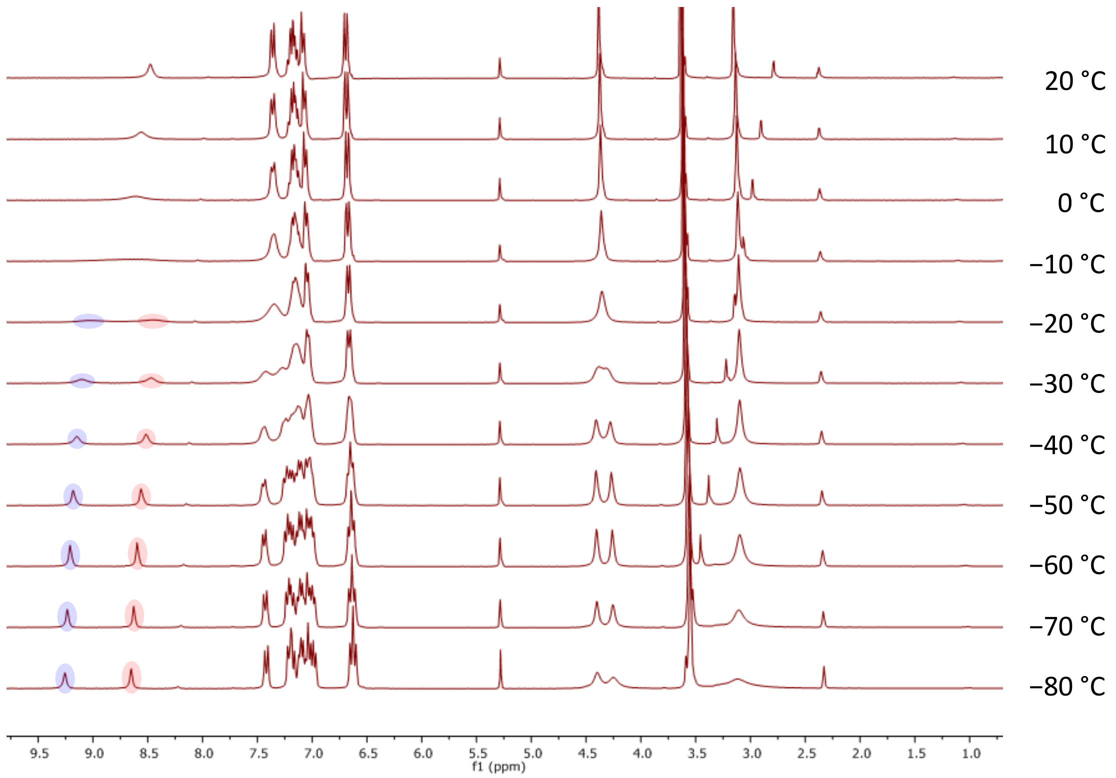

Figure S6 – VT NMR data for 2a (300 MHz, 10% $(CD_3)_2SO$ in CD_2Cl_2, 19 mM). Signals at 20 °C: 4.38 (benzylic methylenes) and 8.47 ppm (ureido protons). The signal at 4.38 ppm broadens and decoalesces at −30 °C into two singlets in a 50:50 ratio, which sharpen further upon cooling. The signal at 8.47 ppm shifts downfield and broadens with decreasing temperature until it decoalesces into two singlets (1 H each) at −20 °C, which then sharpen and shift further downfield with decreasing temperature. These data show that that d_6-DMSO does not interrupt the intramolecular hydrogen bonding in **2a**, it instead complements it by hydrogen bonding to the exposed terminal ureido proton. This is further validated by the chemical shifts of the ureido protons at low temperature. At −60 °C, there is a signal at 9.21 ppm corresponding to a ureido proton intramolecularly hydrogen bonded to the other urea. The other ureido proton shows a signal at 8.59 ppm, which is much more downfield than that observed in CD_2Cl_2 (6.66 ppm at −60 °C) as it is hydrogen bonding with d_6-DMSO. Triurea **3a** and tetraurea **4a** were also analysed by VT NMR (Figure S14, S20), and they were found to behave analogously to diurea **2a**, but with increasing numbers of hydrogen-bonding ureido protons.

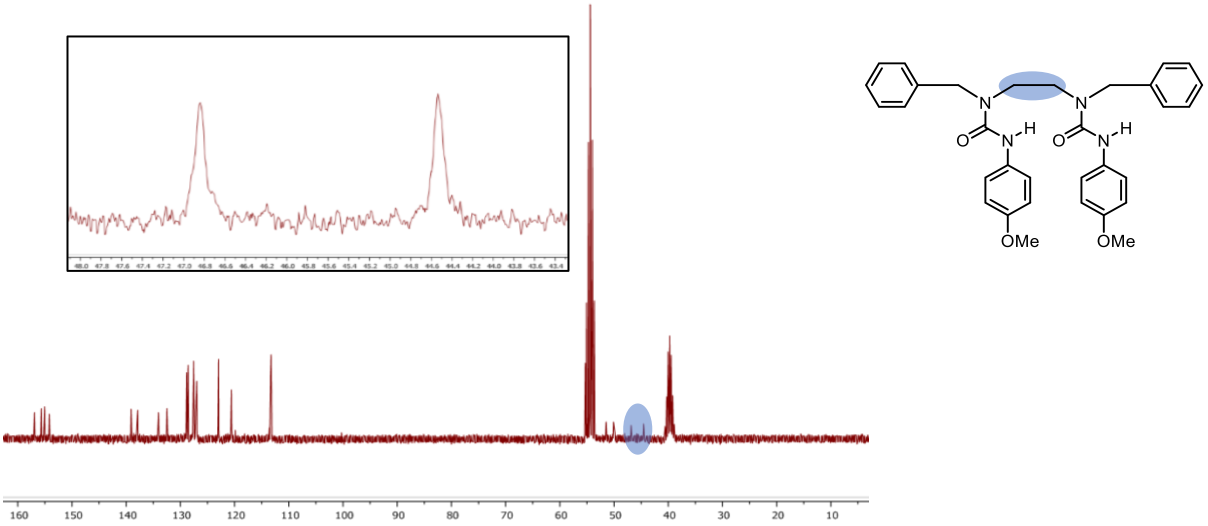

Figure S7 – ^{13}C spectrum of 2a (76 MHz, CD_2Cl_2, 19 mM) at −60 °C. There are four methylene environments at slow exchange, ruling out a C_2-symmetrical conformation in favour of the C_1 conformation depicted above.

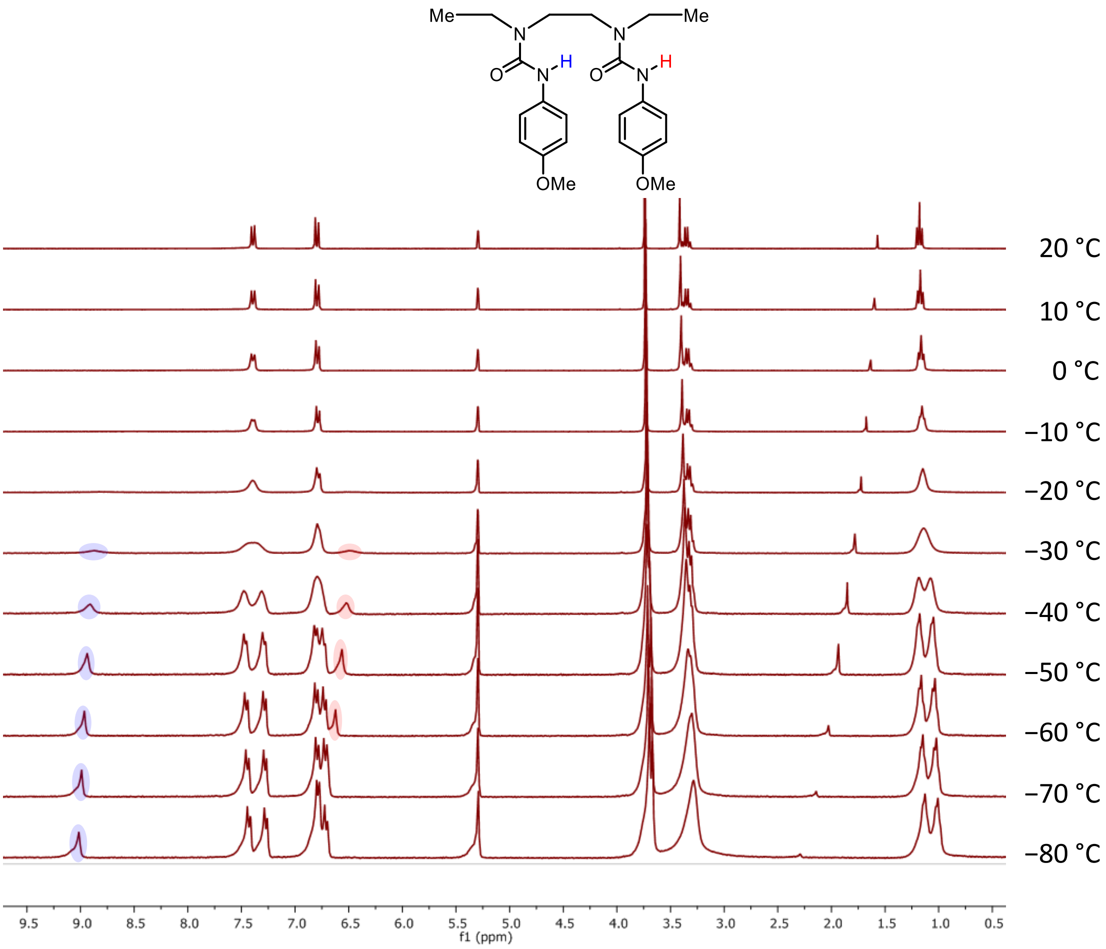

Figure S8 – VT NMR data for 2b (300 MHz, CD_2Cl_2, 24 mM).

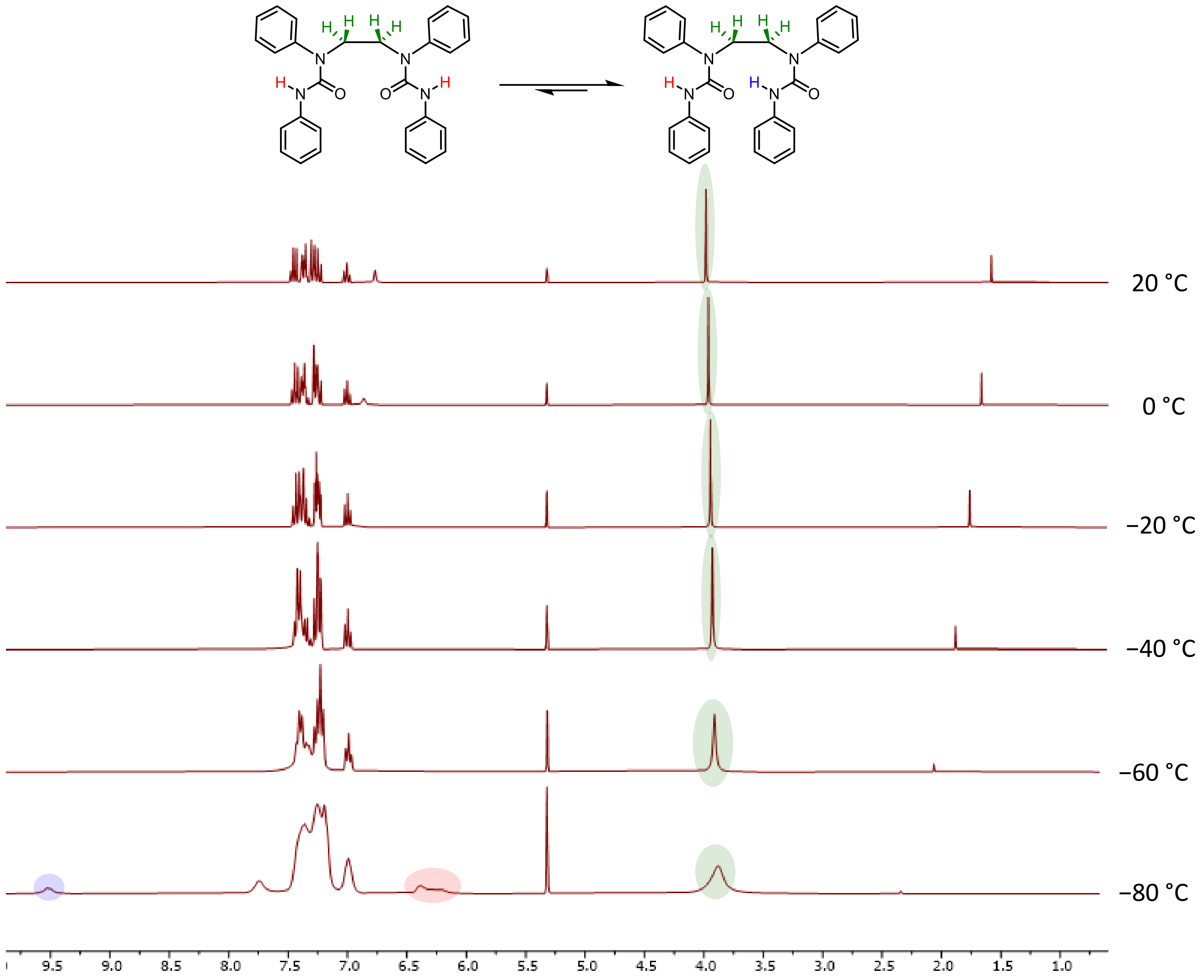

Figure S9 – VT NMR data for 2c (300 MHz, CD_2Cl_2, 36 mM). Signals at 20 °C: 3.93 (methylenes) and 6.49 ppm (ureido protons). The signal at 3.93 ppm remains sharp until it broadens at −60 °C. The signal at 6.49 ppm shifts downfield and broadens with decreasing temperature. Broadening continues into the baseline, where decoalescence into three singlets at 6.23 (0.6 H), 6.40 (0.8 H) and 9.52 ppm (0.6 H) at −80 °C occurs. These data conclude that at −80 °C, two conformers are present in a 40:60 ratio. One unsymmetric conformation, where one ureido proton is intramolecularly hydrogen bonded and the other is not (6.23 and 9.52 ppm) is present as the major conformer. Another symmetric conformer, where the ureido protons are identical and not in hydrogen bonding (6.40 ppm) is the minor conformer.

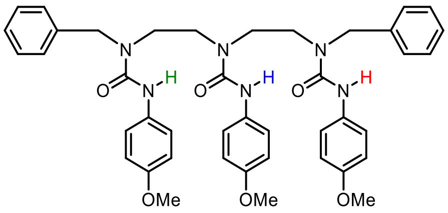

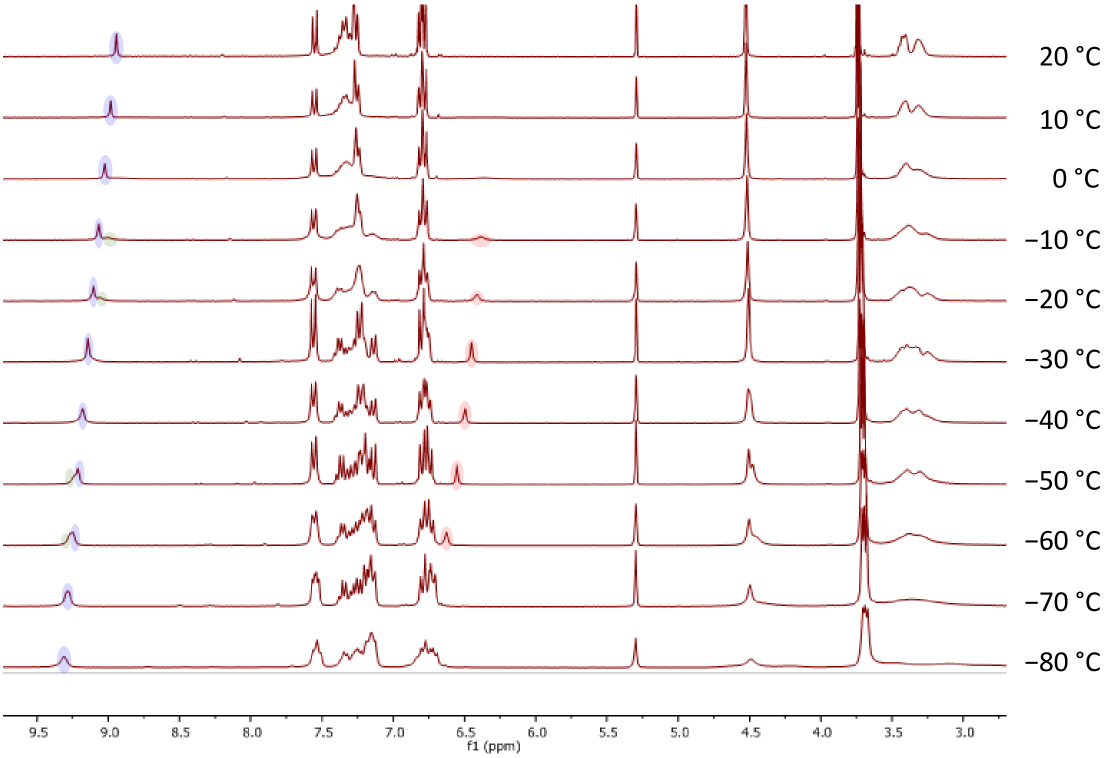

Figure S10 – VT NMR data for 3a (300 MHz, CD_2Cl_2, 14 mM).

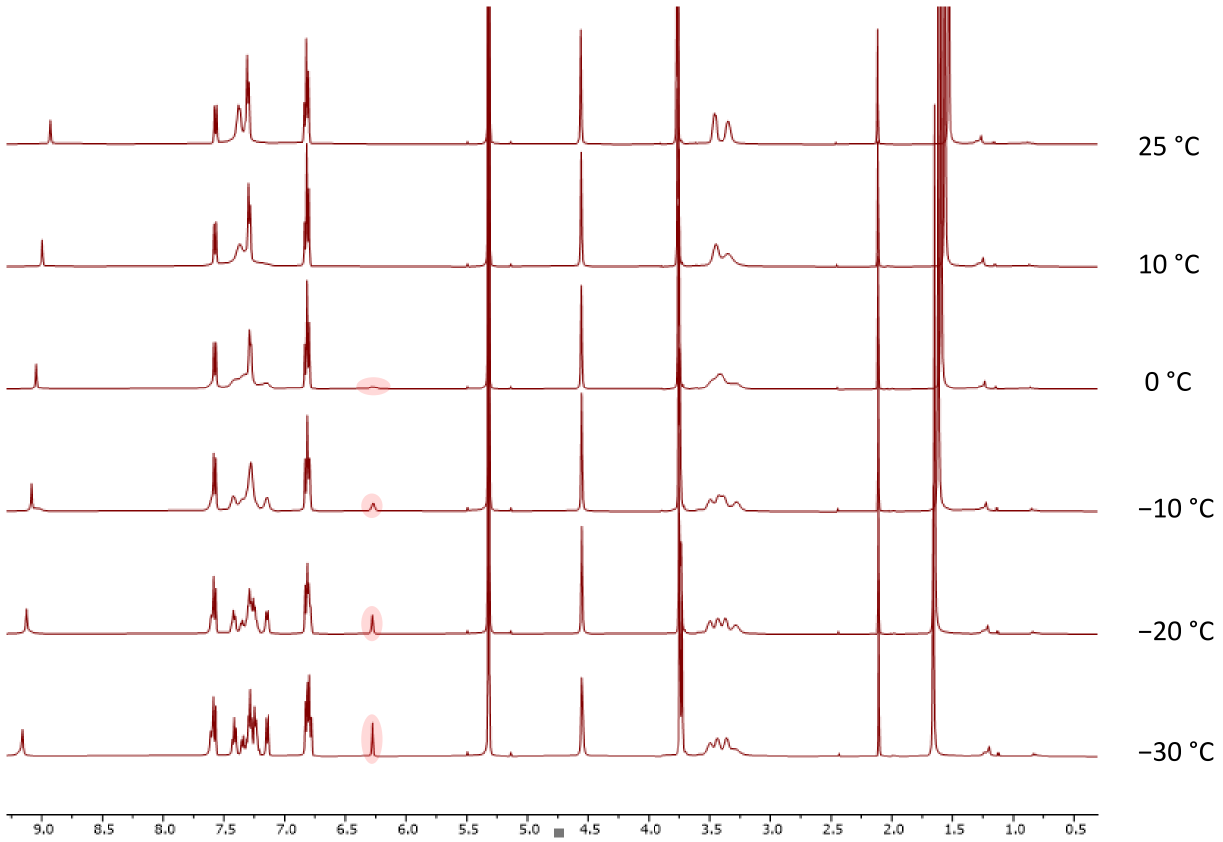

Figure S11 – VT NMR data for 3a (500 MHz, CD_2Cl_2, 2.5 mM). At slow exchange (< 0 °C), a signal appears at 6.28 ppm corresponding to the non-hydrogen bonding ureido proton, which remains at the same chemical shift through to −30 °C.

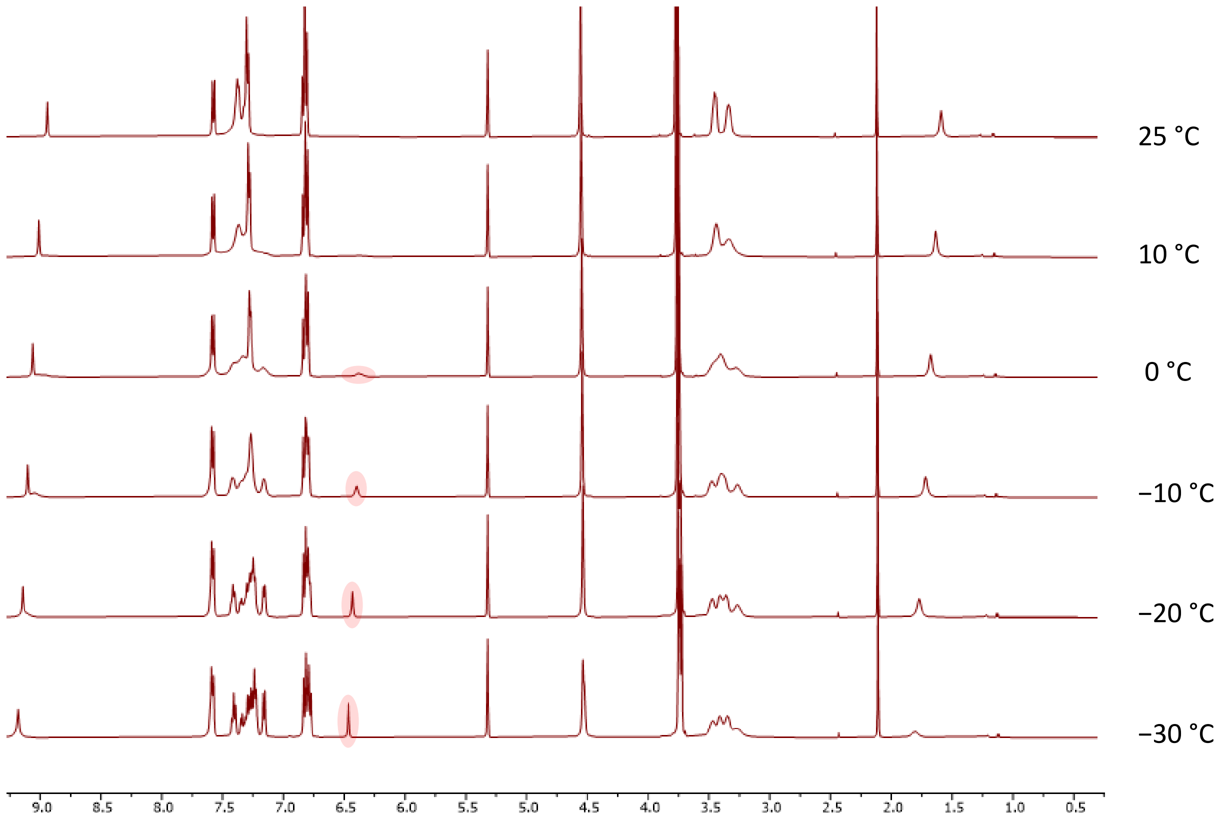

Figure S12 – VT NMR data for 3a (500 MHz, CD_2Cl_2, 25 mM). At slow exchange (< 0 °C), a signal appears at at 6.37 ppm corresponding to the non-hydrogen bonding ureido proton. The signal moves downfield with decreasing temperature to 6.48 ppm at −30 °C. The concentration and temperature dependence of the non-intramolecularly hydrogen-bonding ureido proton is attributed to intermolecular hydrogen bonding with the carbonyl of the urea at the other terminus. It is assumed that this intermolecular hydrogen bonding translates to other compounds at this concentration when terminal hydrogen-bond acceptors are available.

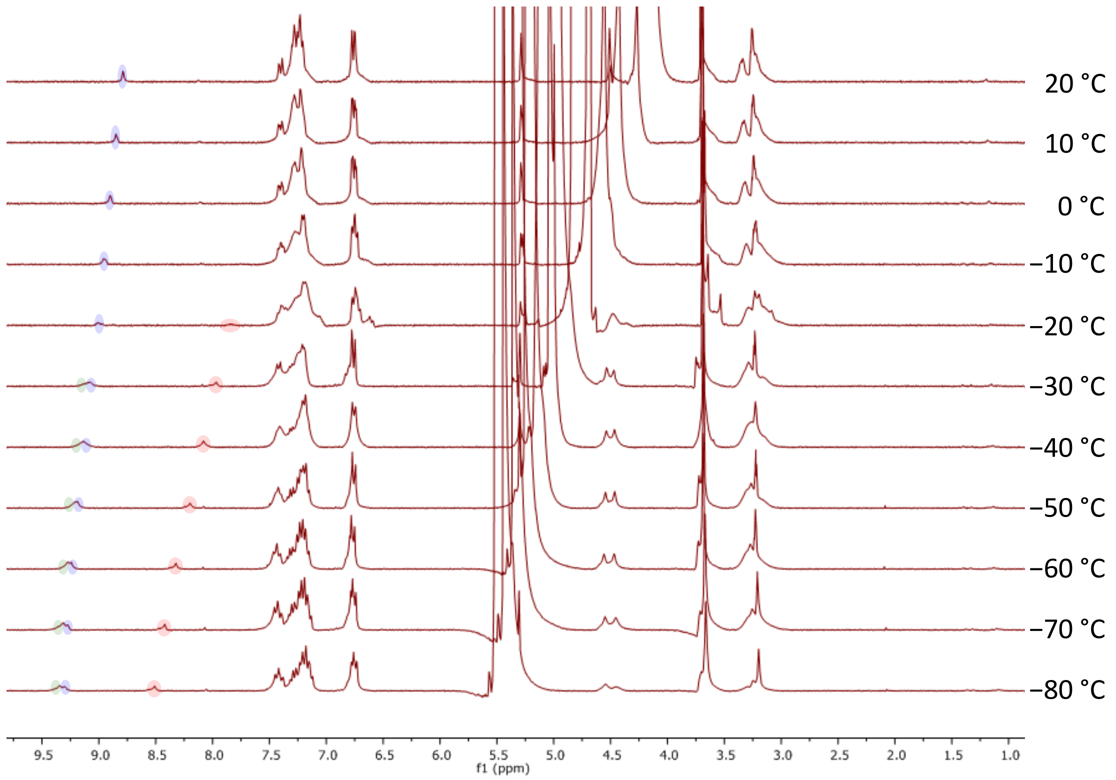

Figure S13 – VT NMR data for 3a (300 MHz, 40% CD_3OH in CD_2Cl_2, 14 mM).

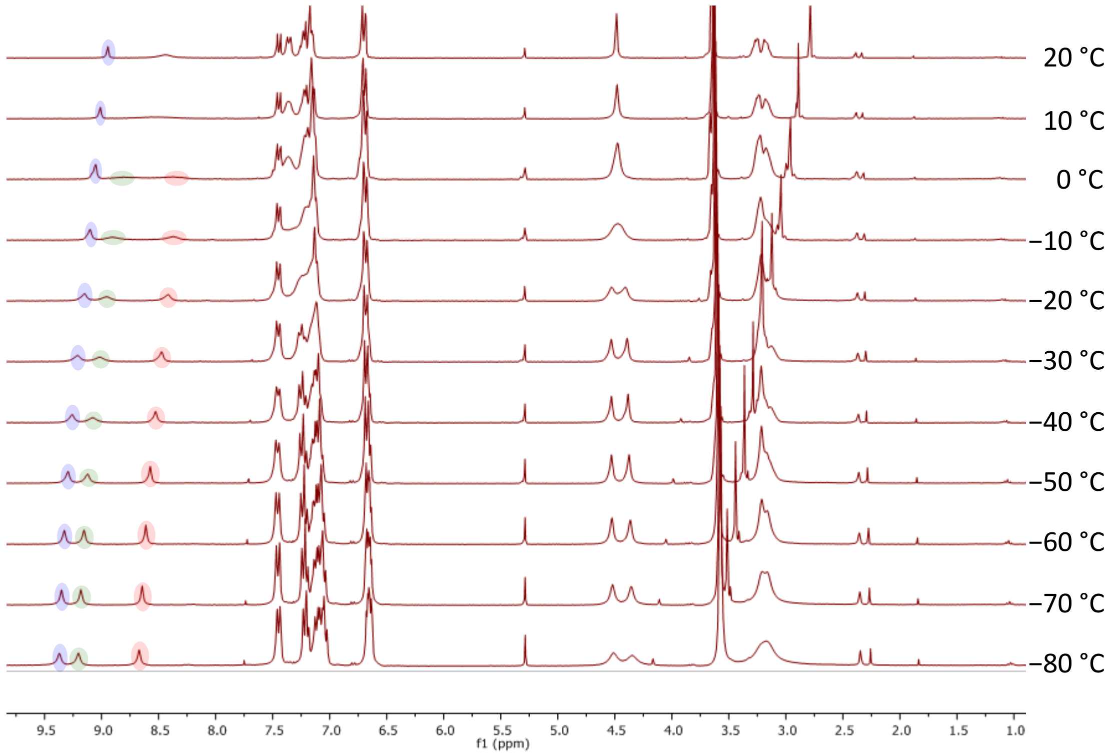

Figure S14 – VT NMR data for 3a (300 MHz, 10% $(CD_3)_2SO$ in CD_2Cl_2, 14 mM).

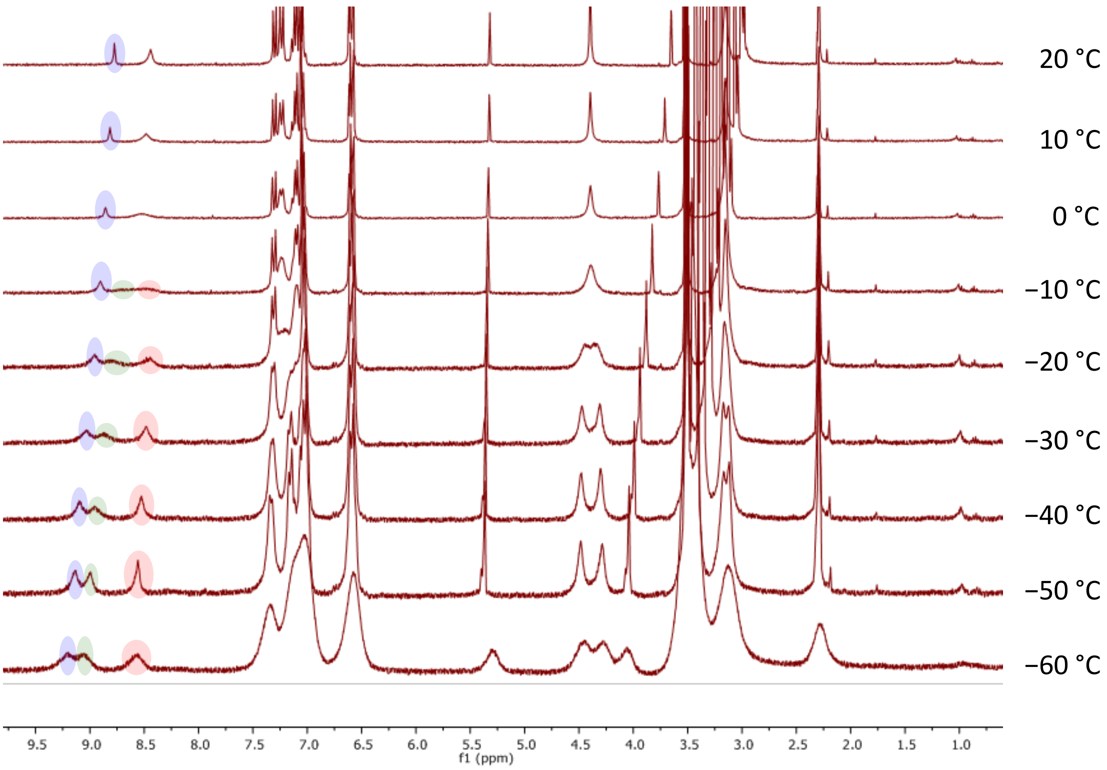

Figure S15 – VT NMR data for 3a (300 MHz, 50% $(CD_3)_2SO$ in CD_2Cl_2, 14 mM).

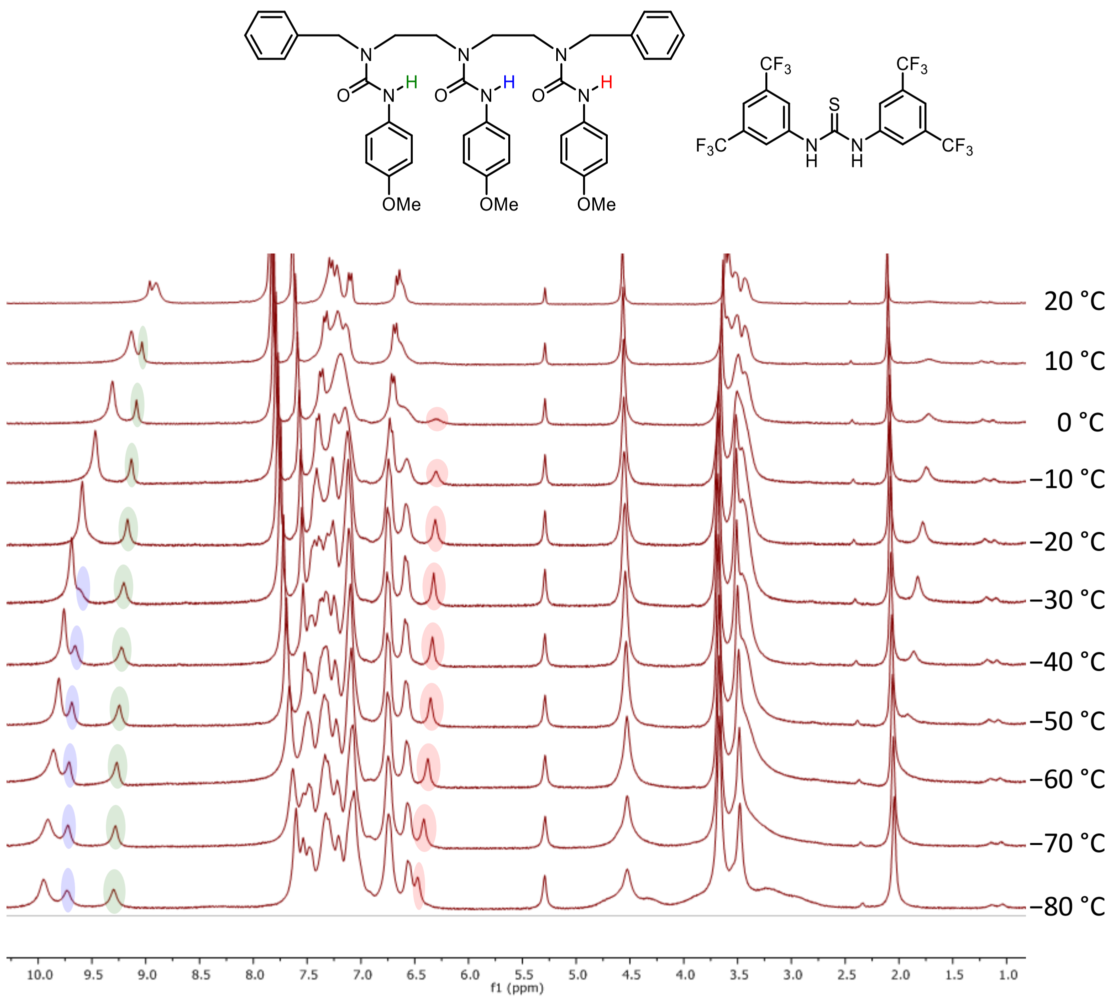

Figure S16 – VT NMR data for 3a (300 MHz, 2.5 equiv of *N*,*N*'-bis(trifluoromethyl)phenyl thiourea in CD_2Cl_2, 14 mM).

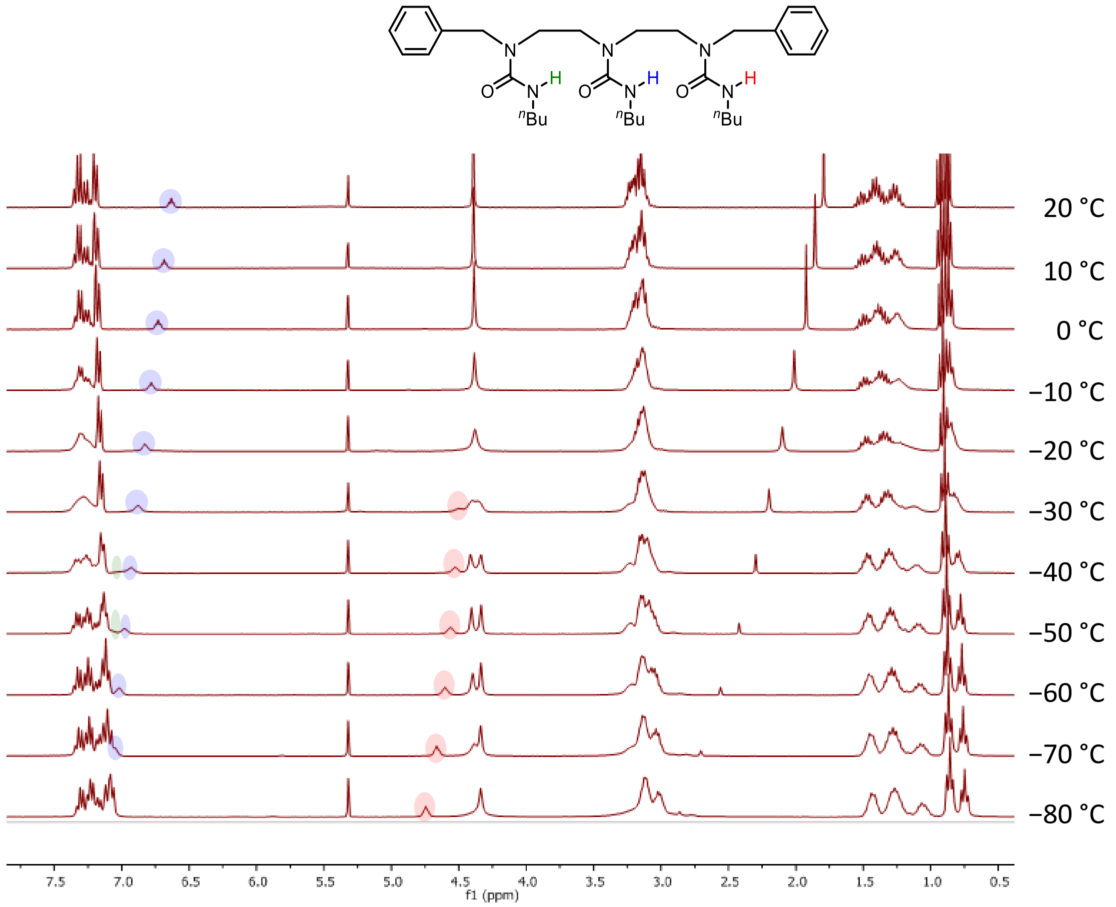

Figure S17 – VT NMR data for 3b (300 MHz, CD_2Cl_2, 17 mM).

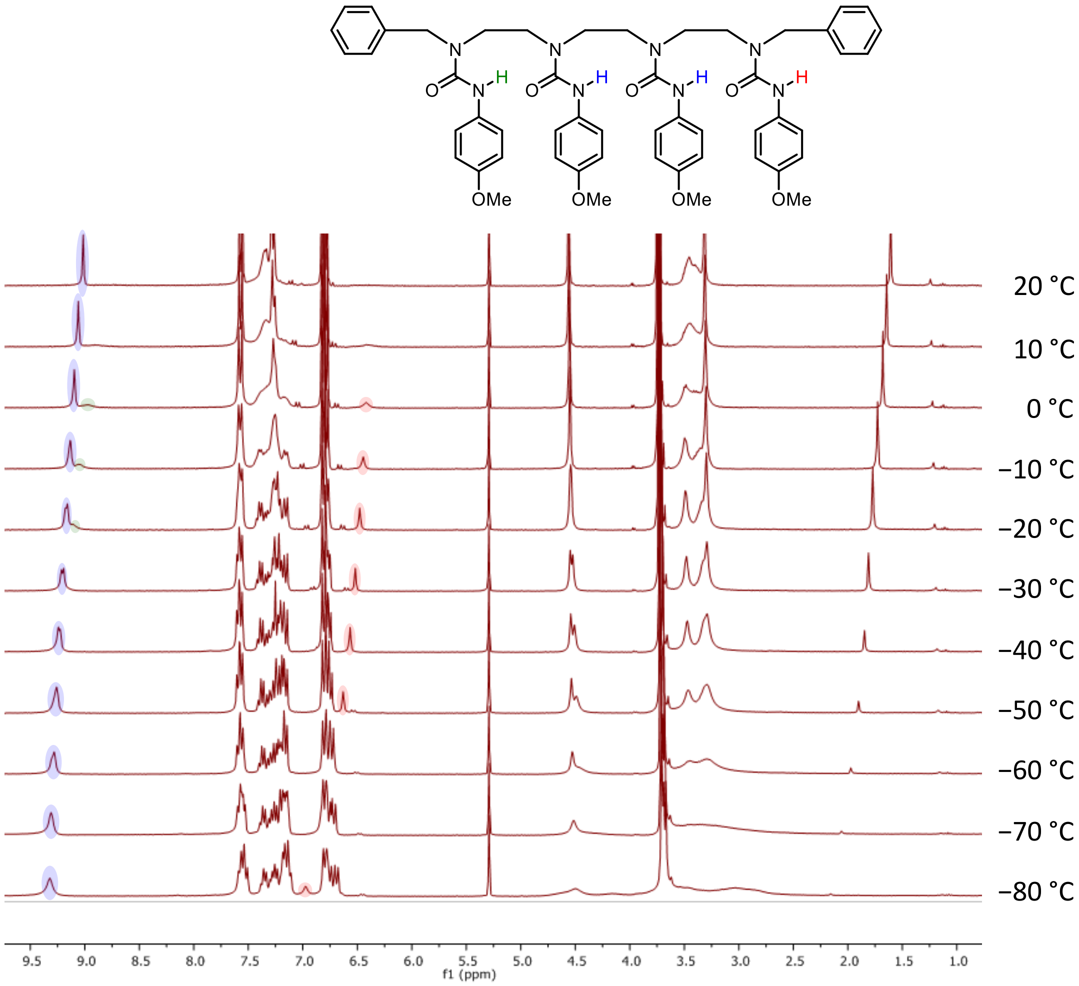

Figure S18 – VT NMR data for 4a (300 MHz, CD_2Cl_2, 11 mM).

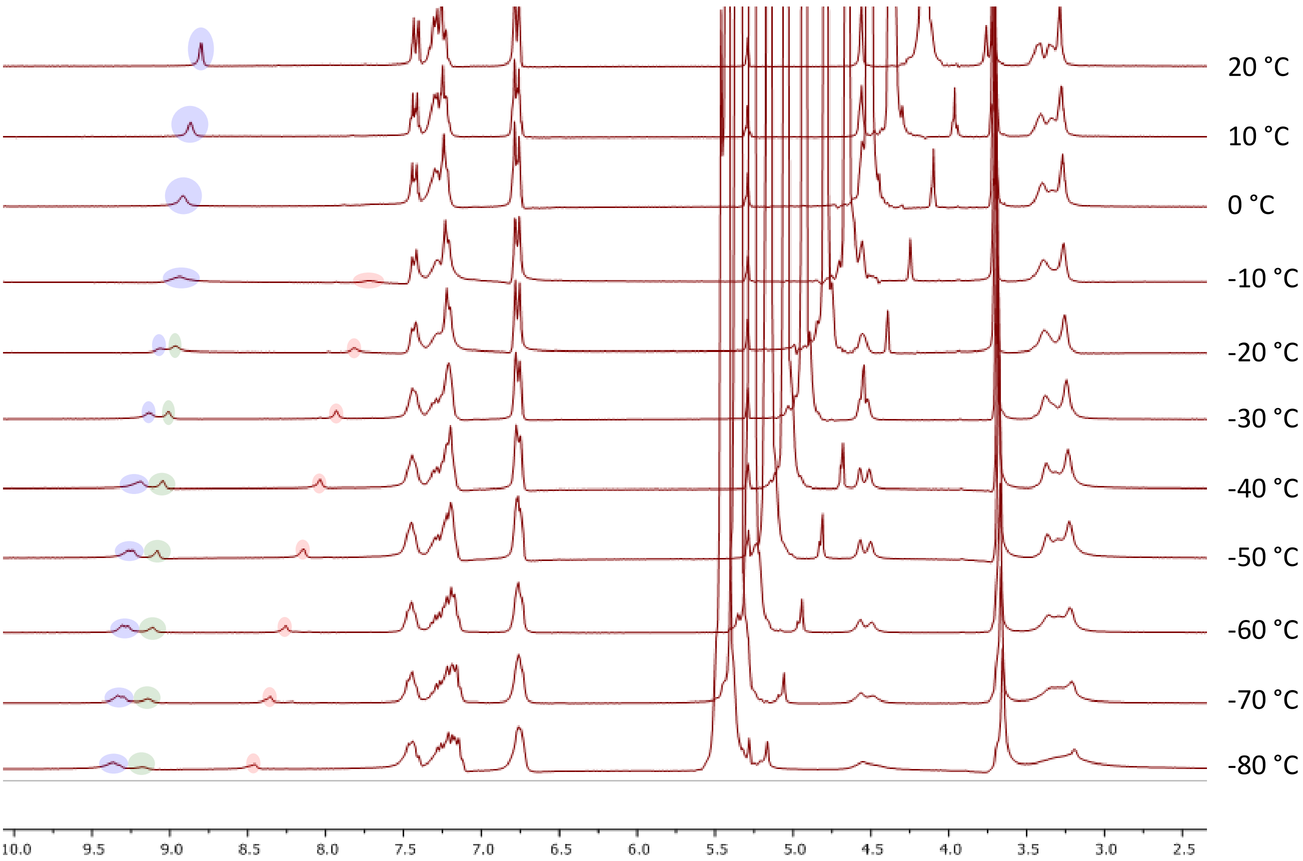

Figure S19 – VT NMR data for 4a (300 MHz, 40% CD_3OH in CD_2Cl_2, 11 mM).

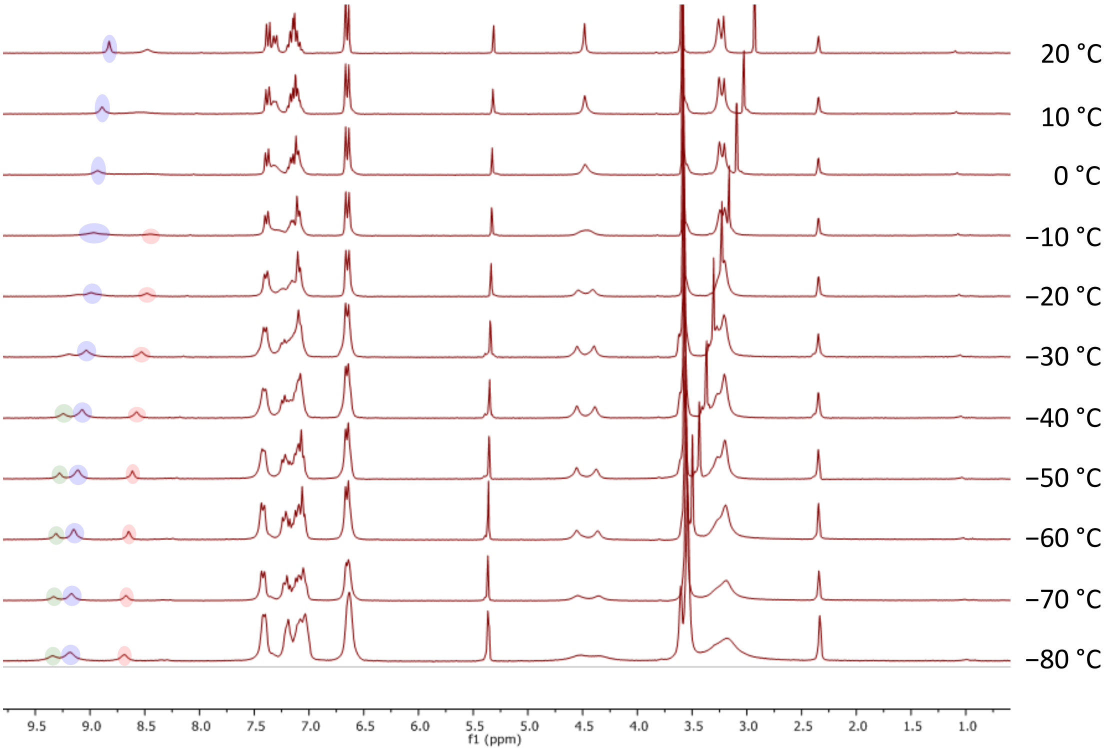

Figure S20 – VT NMR data for 4a (300 MHz, 10% $(CD_3)_2SO$ in CD_2Cl_2, 11 mM).

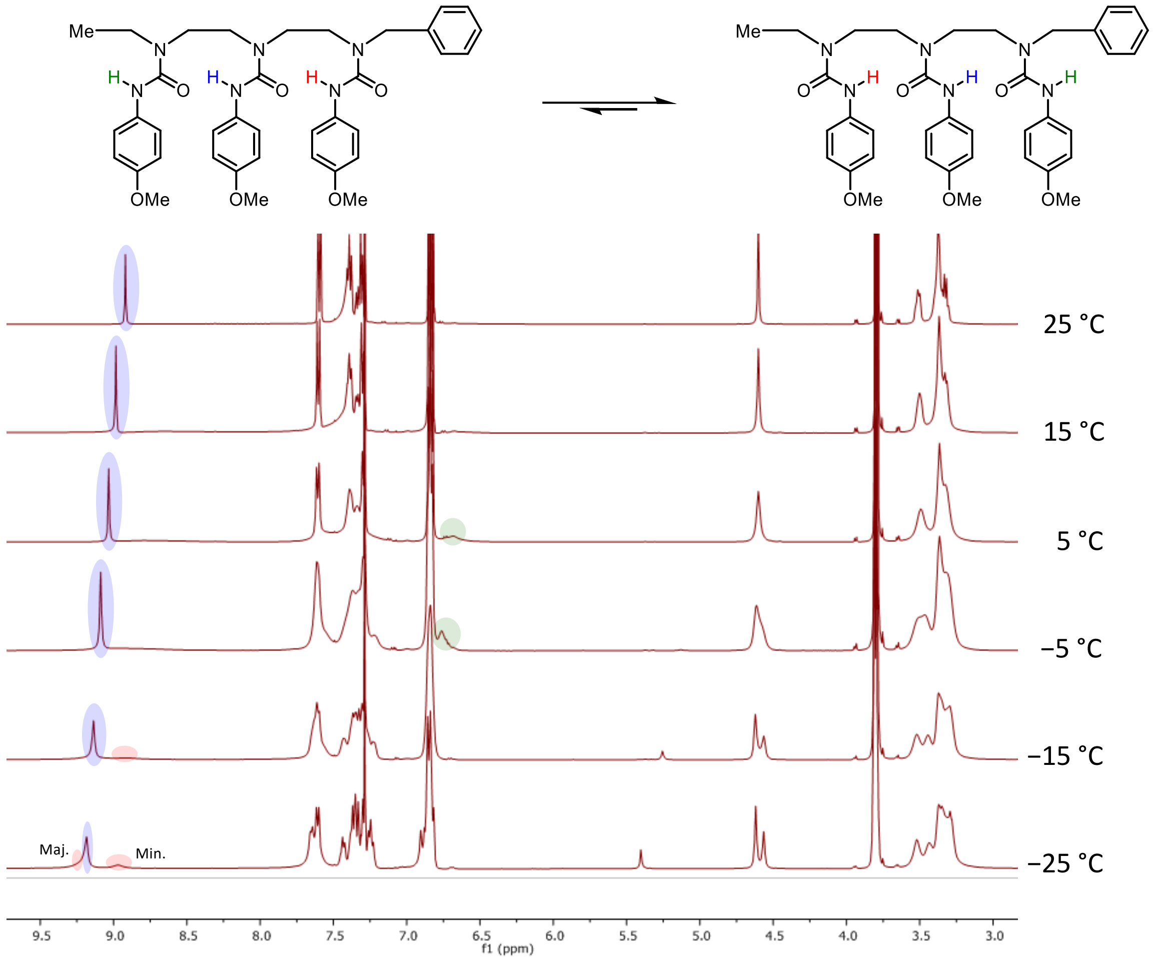

Figure S21 – VT NMR data for 3c (500 MHz, CD_2Cl_2, 15 mM). Signals at 25 °C: 4.60 (benzylic methylene) and 8.92 ppm (central urea ureido proton). Upon cooling, the signal at 4.60 ppm broadens and decoalesces into two distinct singlets at −15 °C. These decoalesced signals sharpen at −25 °C, where they were present in a ratio of 60:40. The signal at 8.92 ppm (1 H) shifts downfield with decreasing temperature to reach a value of 9.19 ppm at −25 °C. At 5 °C, a new signal appears at 6.76 ppm (1 H), which sharpens and shifts downfield upon further decreasing the temperature. Similarly, new signals emerge at 8.90 (0.40 H) and 9.23 ppm (0.60 H, overlapping) at −15 °C, which behave the same. These data show that there are two conformations in fast exchange on the NMR timescale at 20 °C, which enter the slow-exchange region between −5 °C and −15 °C. These two conformations are the two different directionalities, populated in a 40:60 ratio at −25 °C. Each of the directionalities have two hydrogen-bonded ureido protons, and a non-hydrogen-bonded ureido proton. The signals at 6.76 and 9.19 ppm at −25 °C correspond to the non-hydrogen-bonded and central hydrogen-bonded ureido protons, respectively of both conformers. The signals at 8.90 (0.40 H) and 9.23 ppm (0.60 H) at −25 °C correspond to the peripheral hydrogen-bonded ureido protons in the minor and major conformers, respectively.

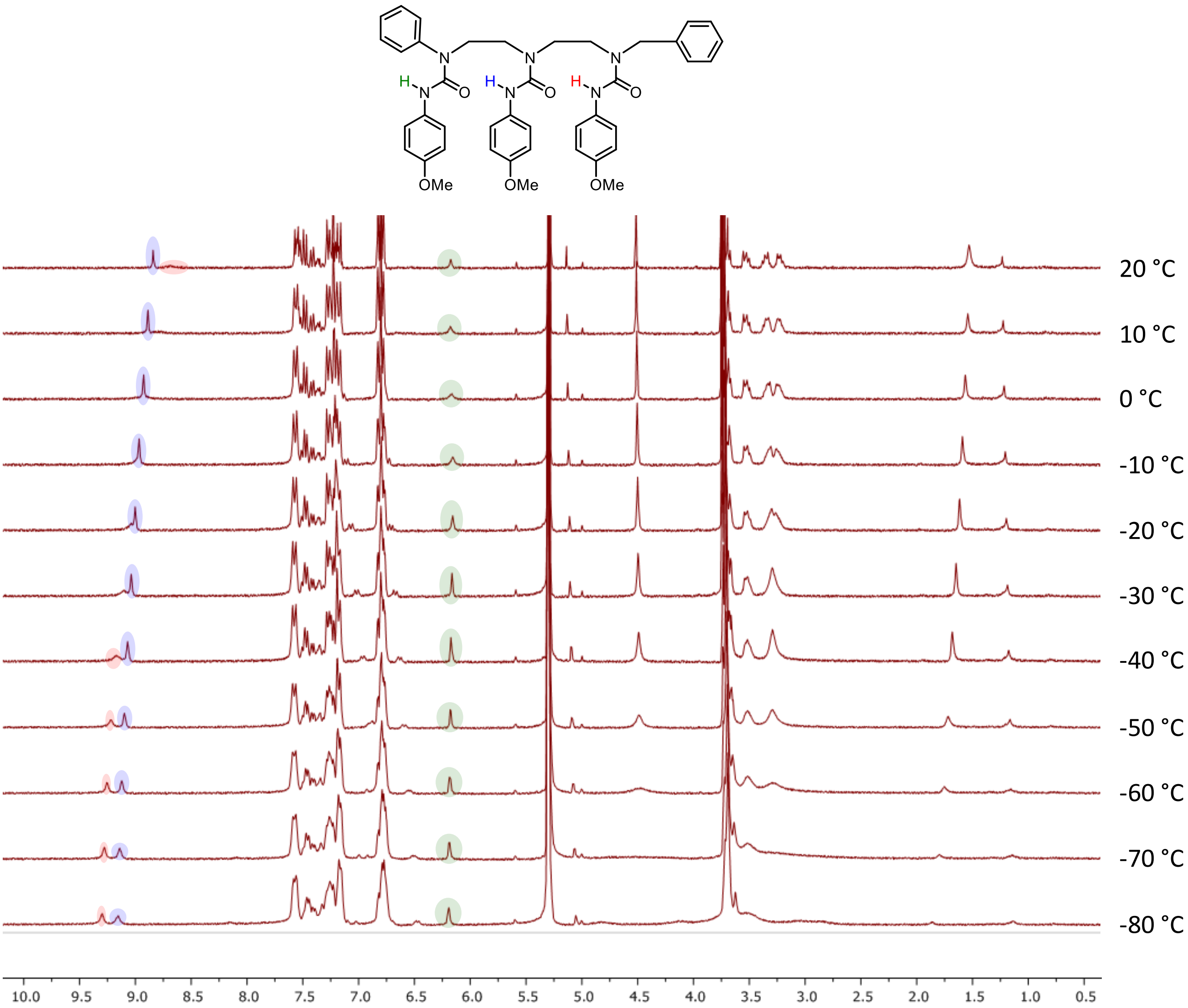

Figure S22 – VT NMR data for 3d (300 MHz, CD_2Cl_2, 14 mM).

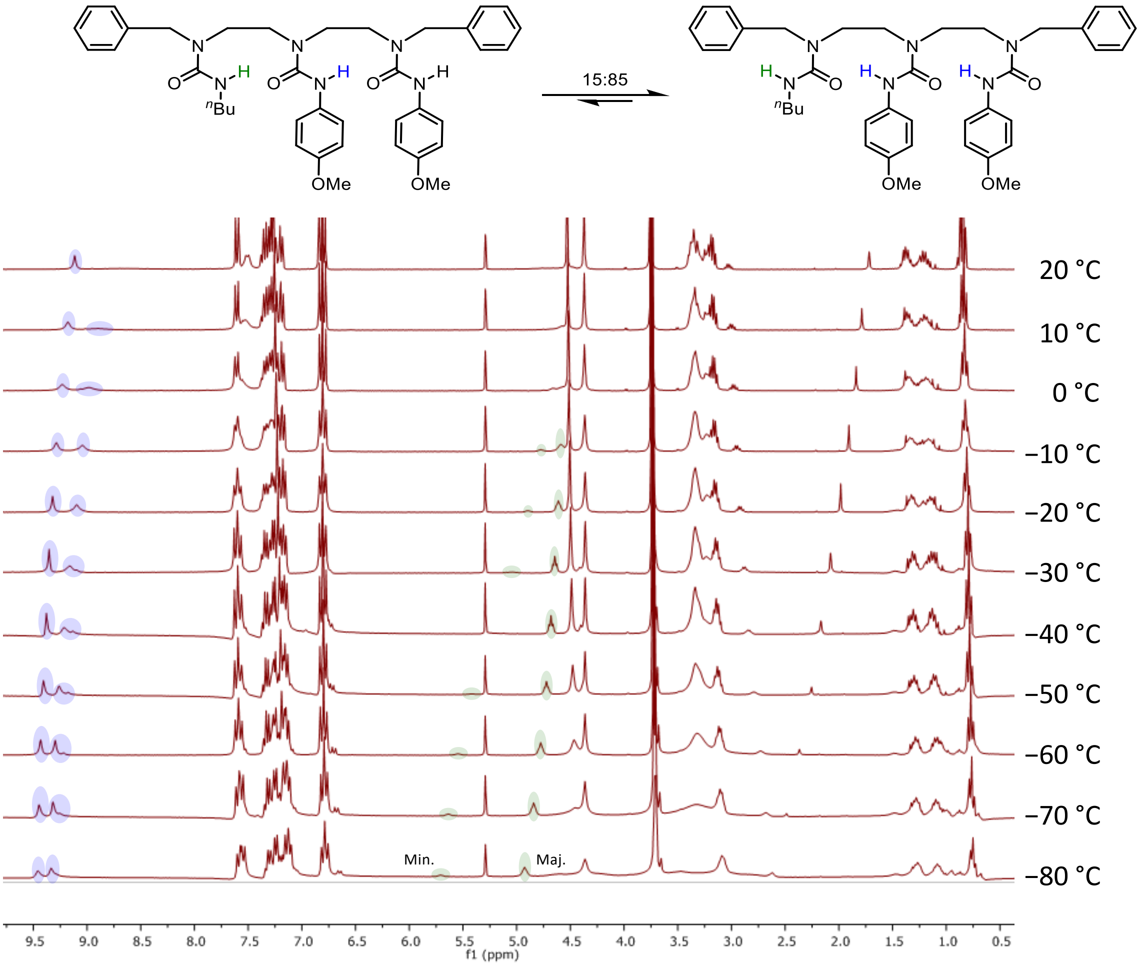

Figure S23 – VT NMR data for 3e (300 MHz, CD_2Cl_2, 15 mM). Signals at 20 °C: 4.37 (alkyl urea benzylic methylene), 4.53 (aryl urea benzylic methylene), 8.65 (peripheral aryl urea ureido proton) and 9.11 ppm (central ureido proton) at 20 °C. Upon cooling, the signal at 4.37 ppm stays approximately constant until it broadens at −20 °C and decoalesces into two singlets (0.30 H and 1.70 H) at −30 °C. The signal at 4.53 ppm broadens until it cannot be observed at −80 °C. The signals at 8.65 (very broad) and 9.11 ppm sharpen and shift downfield with decreasing temperature. At −10 °C, signals appear at 4.59 (0.85 H) and 4.77 ppm (0.15 H), which sharpen and shift downfield upon further decreasing the temperature. Another signal appears at 9.10 ppm (0.15 H) at −30 °C, which shifts downfield with further cooling. These data indicate that **3e** exists as two conformations populated in a 85:15 ratio at −60 °C. These conformations are the two directionalities, and the major conformer is the directionality where the *n*-butyl urea is at the N-terminus. This is evidenced by the observable decoalescence of the alkyl urea benzylic methylene into two signals in a 85:15 ratio. At −60 °C, the two conformers are in slow exchange on the NMR timescale, and so three ureido protons can be observed for each. For the major conformer, there is a non-hydrogen bonded alkyl ureido proton (4.78 ppm, 0.85 H), and two aryl ureido protons that are (9.30 and 9.43 ppm, 0.85 H each). For the minor conformer, there is an alkyl ureido proton in hydrogen bonding (5.55 ppm, 0.15 H) and an aryl ureido proton in hydrogen bonding (9.21 ppm, 0.15 H).

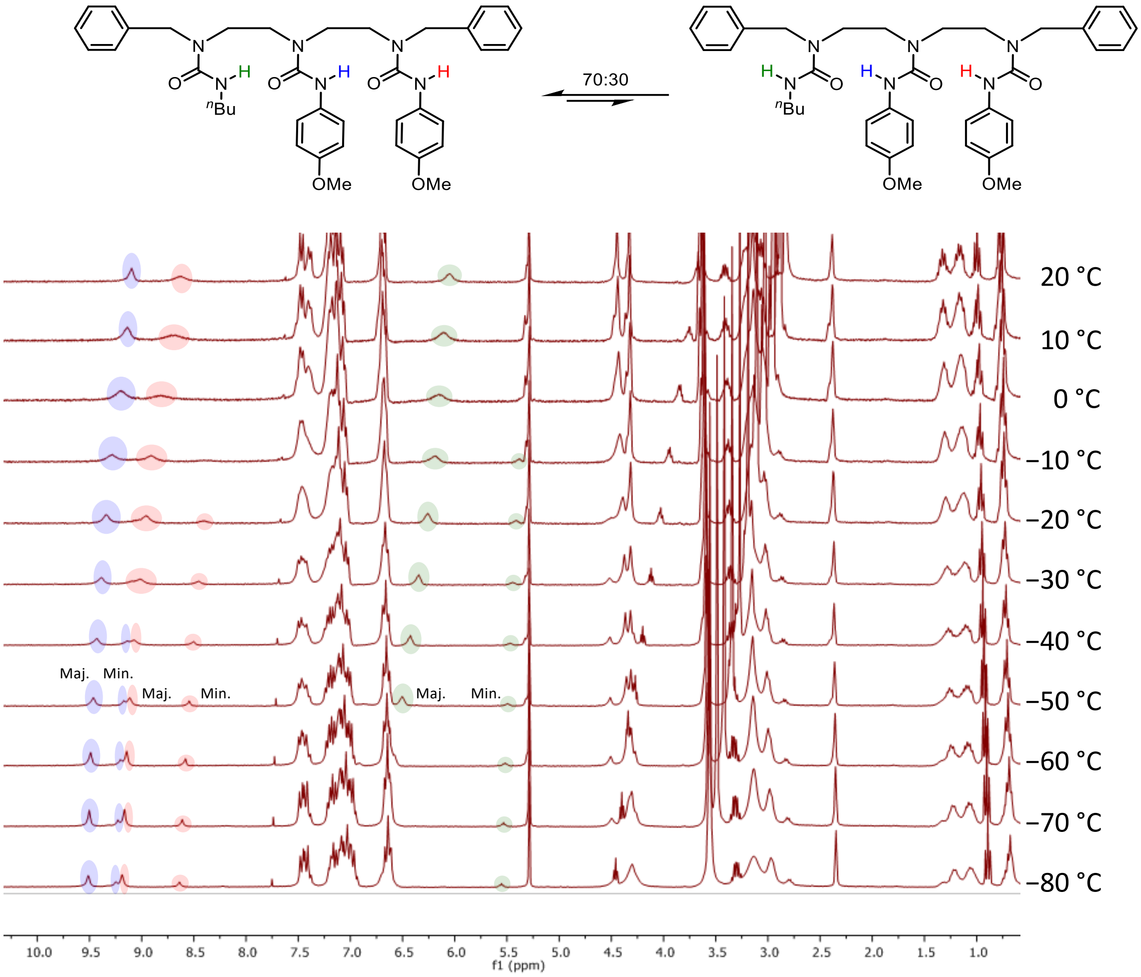

Figure S24 – VT NMR data for 3e (300 MHz, 10% $(CD_3)_2SO$ in CD_2Cl_2, 15 mM). Signals at 20 °C: 4.33 (alkyl urea benzylic methylene), 4.44 (aryl urea benzylic methylene), 6.05 (alkyl urea ureido proton), 8.62 (peripheral aryl urea ureido proton) and 9.09 ppm (central ureido proton) at 20 °C. Upon cooling, the signal at 4.33 ppm broadens throughout the experiment. The same is observed for the signal at 4.44 ppm but it also moved upfield. The signals at 6.05, 8.62 and 9.09 ppm shift downfield with decreasing temperature. Additional signals appear at 5.38 (0.30 H), 8.38 (0.30 H) and 9.07 ppm (0.30 H) at −10 °C, which sharpen and shift downfield upon further cooling. These observations can be explained by the population of two conformers in a 70:30 ratio. This is not elucidated by an observable decoalescence of the benzylic methylene signals but can be inferred from the chemical shifts and relative integrations of the ureido protons. The two conformers correspond to the two directionalities and in each case, d_6-DMSO hydrogen bonds to the exposed ureido proton. At slow exchange, each of the two conformers contain three ureido protons. In one directionality, the *n*-butyl urea acts as an intramolecular HBA, forcing the other two aryl ureido protons into intramolecular hydrogen bonding and leaving its own ureido proton to hydrogen bond to d_6-DMSO. This is shown by the signals at 5.52 (alkyl urea ureido proton, 0.30 H), 8.58 (peripheral aryl urea ureido proton, 0.30 H) and 9.20 ppm (central ureido proton, 0.30 H) at −60 °C. This also identifies this directionality as the minor conformer. In the other directionality (the major conformer), where the *n*-butyl urea acts as an intramolecular HBD, one of the aryl ureas is in an intramolecular hydrogen bond and the other an intermolecular hydrogen bond with d_6-DMSO. This shows signals at 6.58 (alkyl urea ureido proton, 0.70 H), 9.18 (peripheral aryl urea ureido proton) and 9.49 ppm (central ureido proton) at −60 °C.

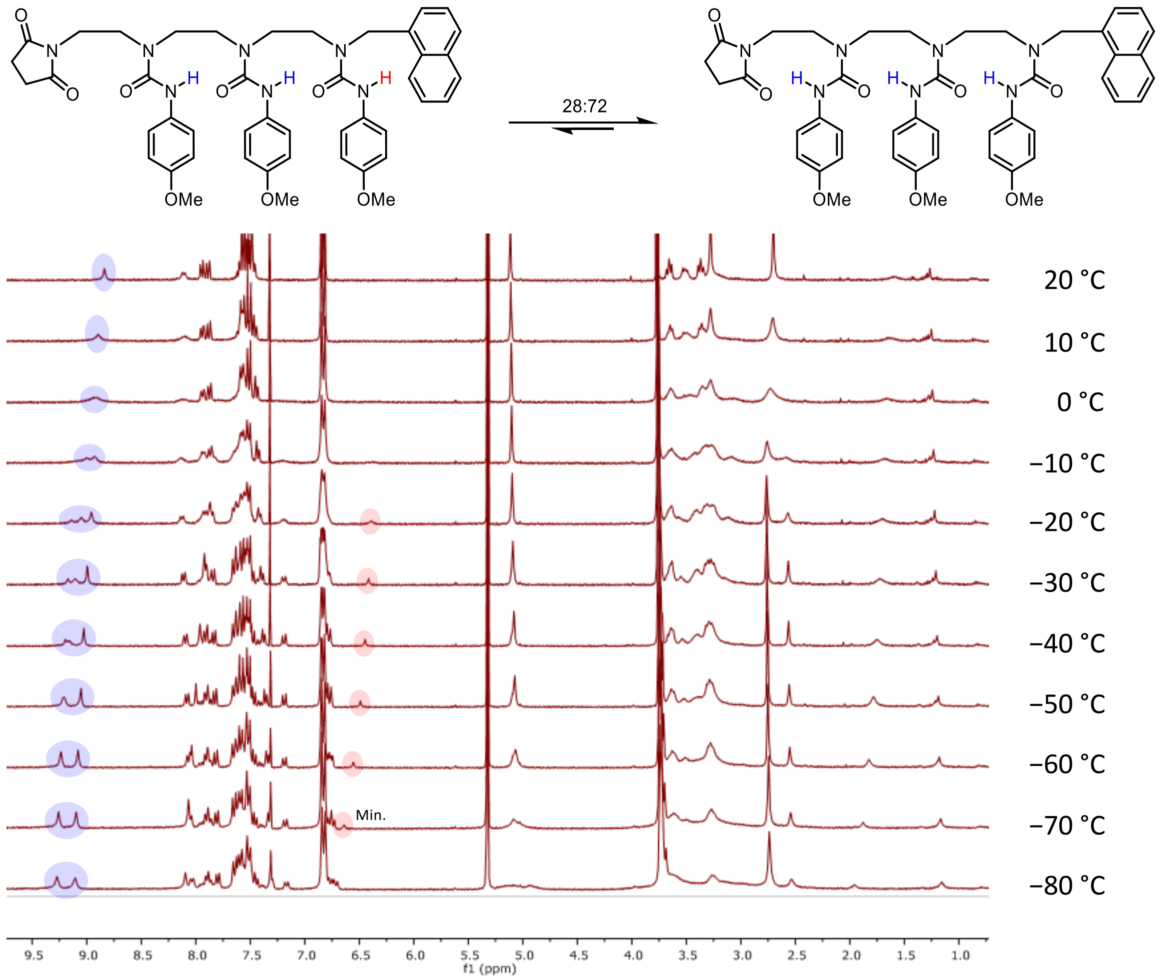

Figure S25 – VT NMR data for 3f (300 MHz, CD_2Cl_2, 12 mM). Signals at 20 °C: 2.70 (succinimidyl methylenes), 5.11 (benzylic methylene) and 8.84 ppm (one ureido proton) at 20 °C. Upon cooling, the signal at 2.70 ppm broadens and decoalesces at −10 °C into two singlets, which then sharpen upon further decreasing the temperature (2.87 H and 1.13 H at −60 °C). The signal at 5.11 ppm also broadens and decoalesces into two closely overlapping singlets at −40 °C. The signal at 8.84 ppm moves downfield with decreasing temperature before decoalescing into three signals at −20 °C, which then shift further downfield. Additionally, a new signal appears at 6.39 ppm (0.28 H) at −10 °C, which sharpens and moves downfield upon further decreasing the temperature. These data are consistent with the presence of two conformations populated in a 28:72 ratio at −60 °C, which are the two different directionalities. This is indicated by the two different environments of the succinimide at slow exchange – one where it is hydrogen bonding to the adjacent urea, and one where it is not. Finally, the two different conformations give rise to six different environments for ureido protons. Five of these ureido protons hydrogen bond either to other ureas or to the succinimide, but the sixth ureido proton is not hydrogen bonded, giving rise to a signal at 6.39 ppm at −10 °C. This signal also integrates for 0.28 H at −60 °C, and the other ureido proton integrations account for the remaining 2.72 H, indicating that the conformer where the succinimide is at the C-terminus is the minor conformer.

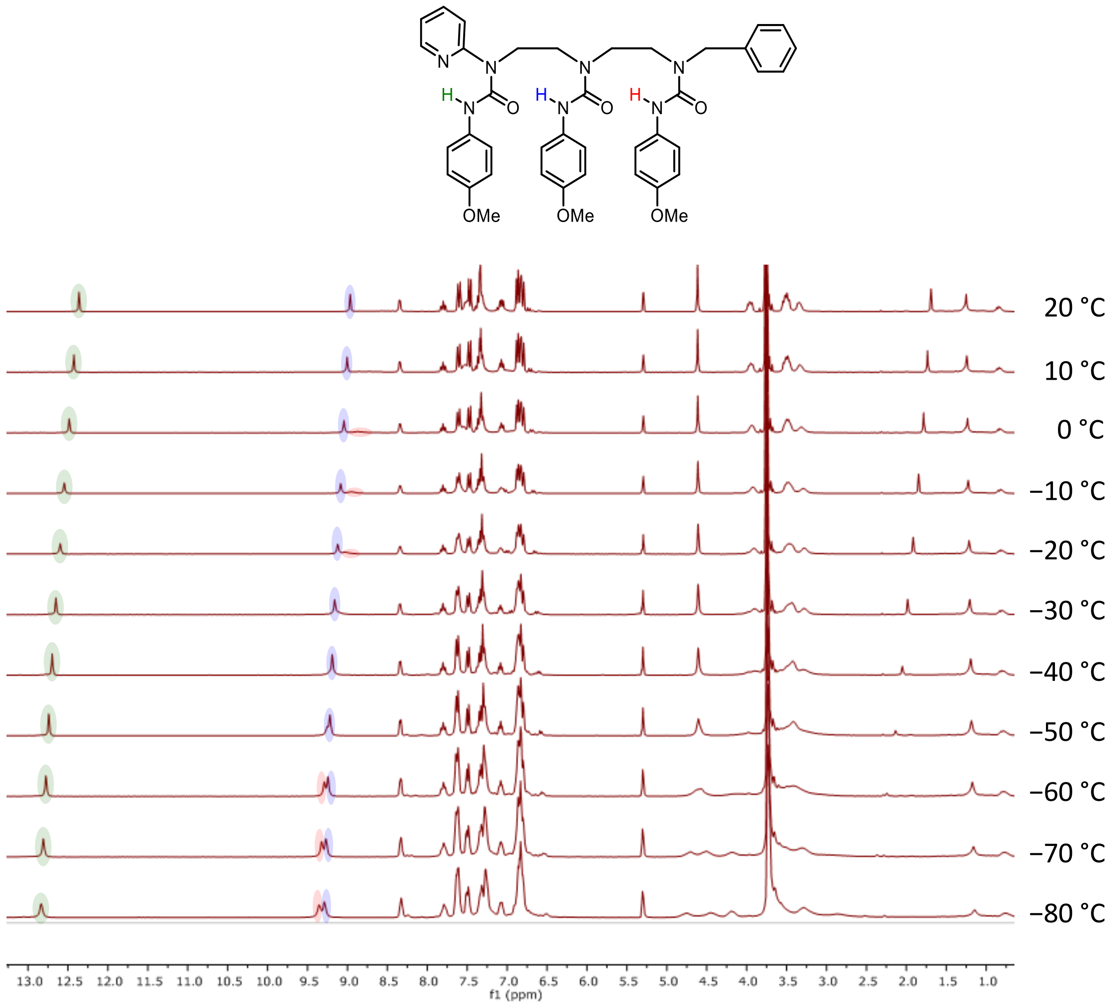

Figure S26 – VT NMR data for 3g (300 MHz, CD_2Cl_2, 14 mM).

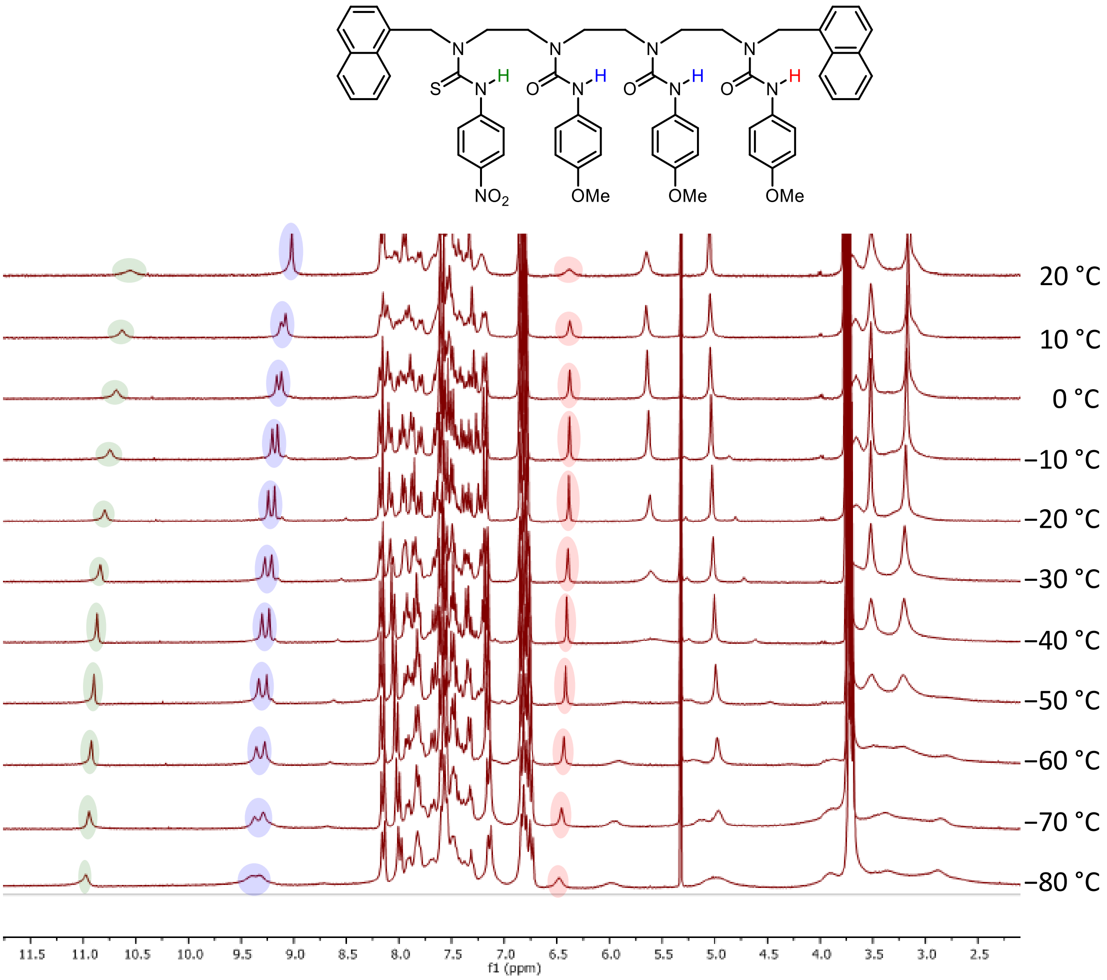

Figure S27 – VT NMR data for 5a (300 MHz, CD_2Cl_2, 19 mM).

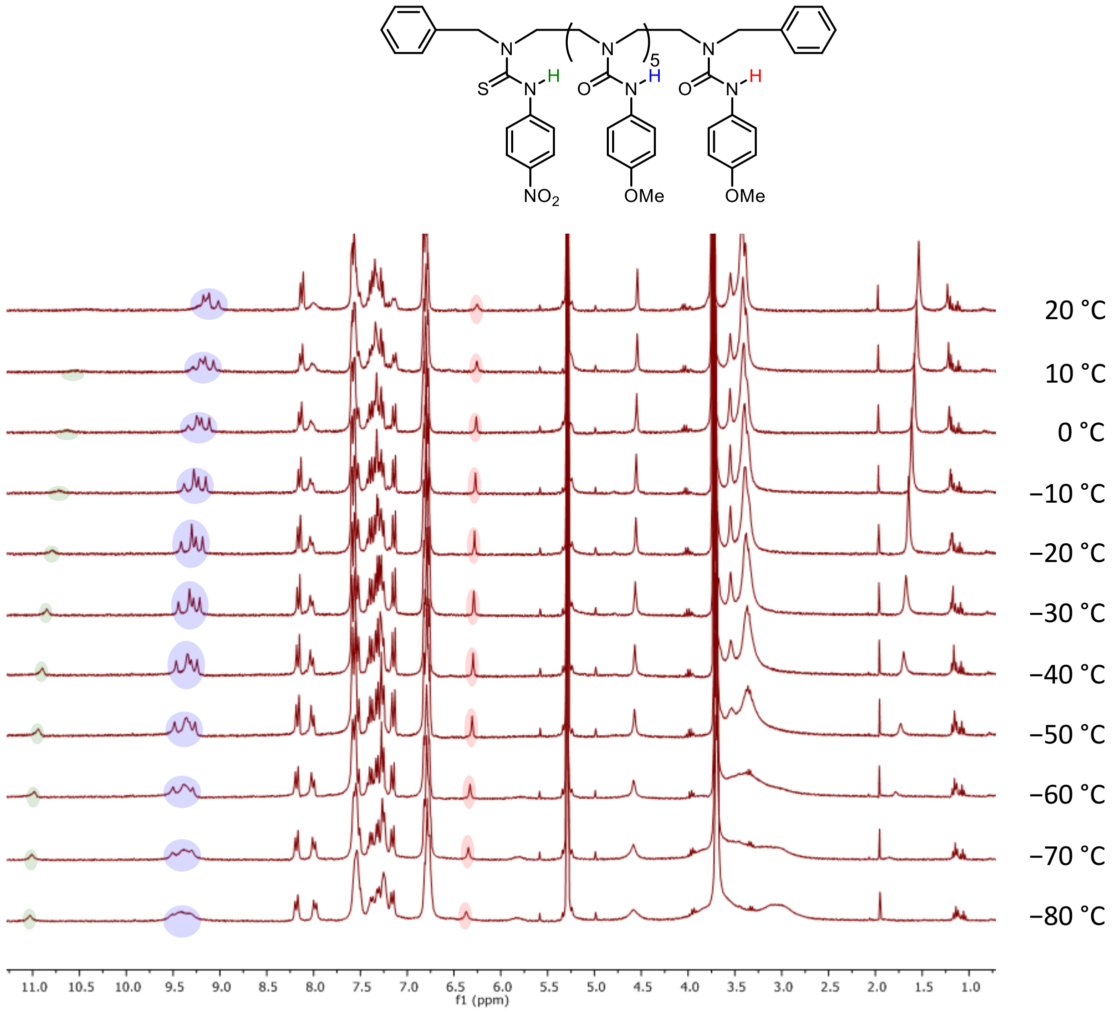

Figure S28 – VT NMR data for 5b (300 MHz, CD_2Cl_2, 61 mM).

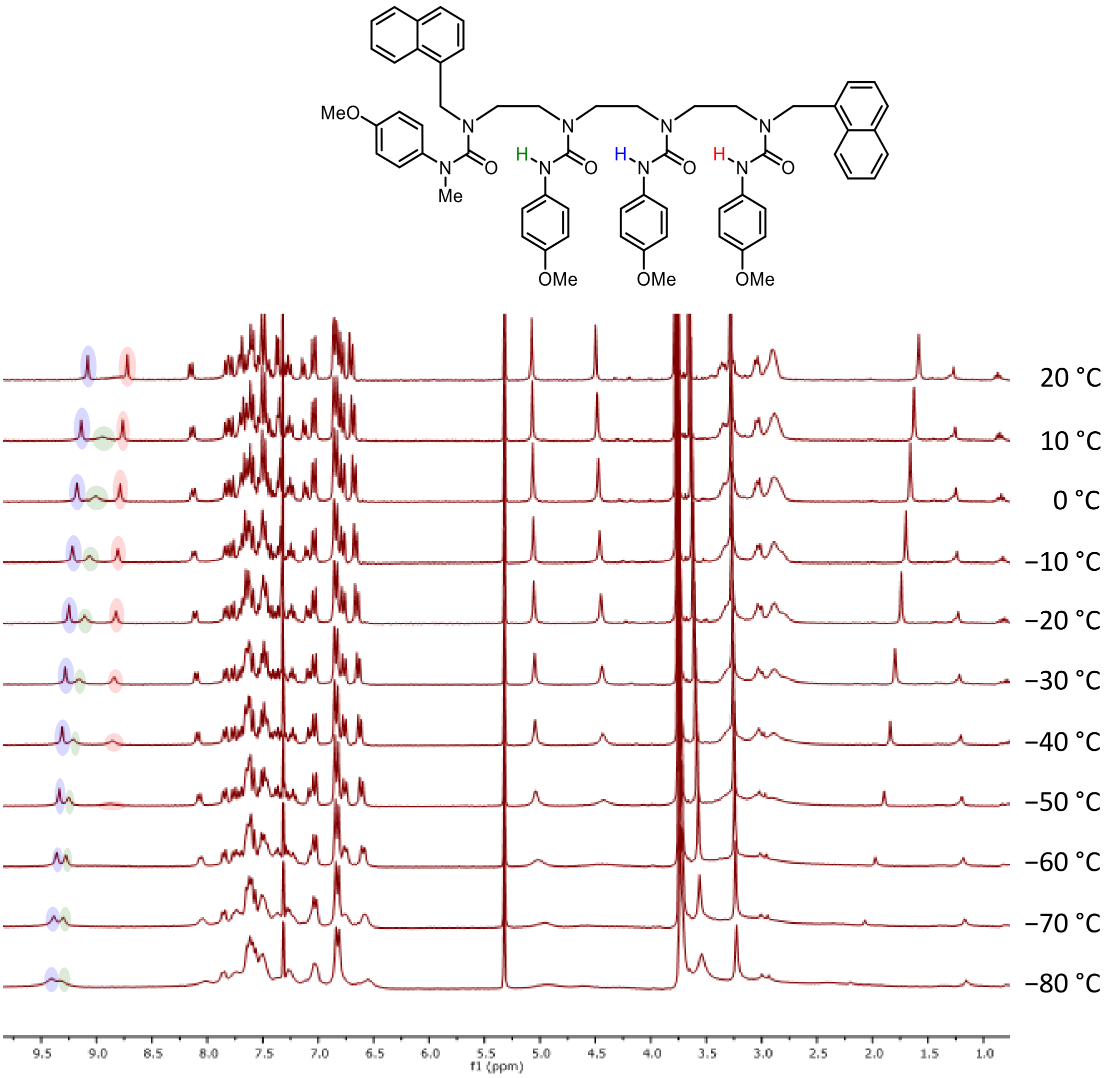

Figure S29 – VT NMR data for 6a (300 MHz, CD_2Cl_2, 19 mM).

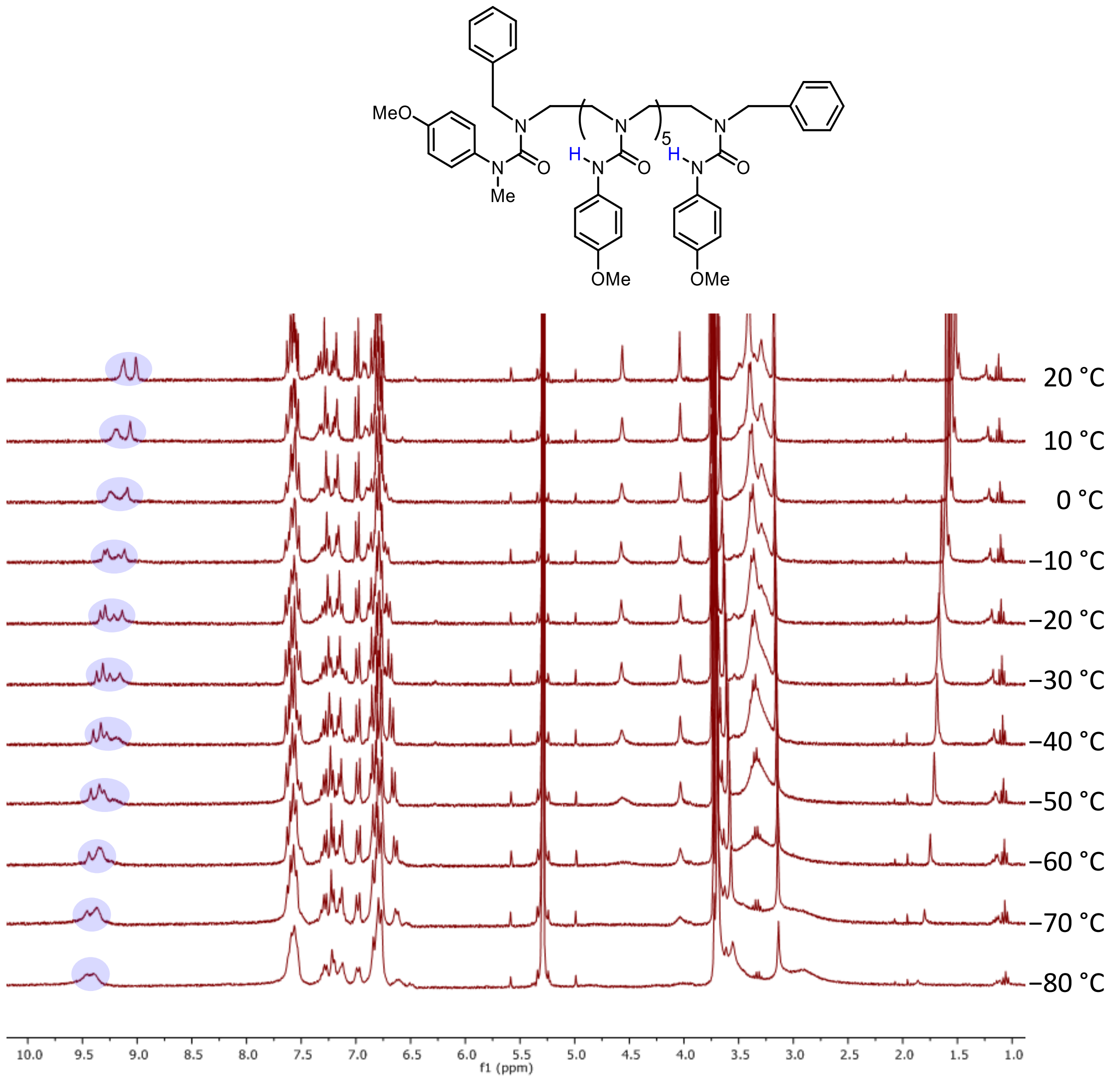

Figure S30 – VT NMR data for 6b (300 MHz, CD_2Cl_2, 49 mM).

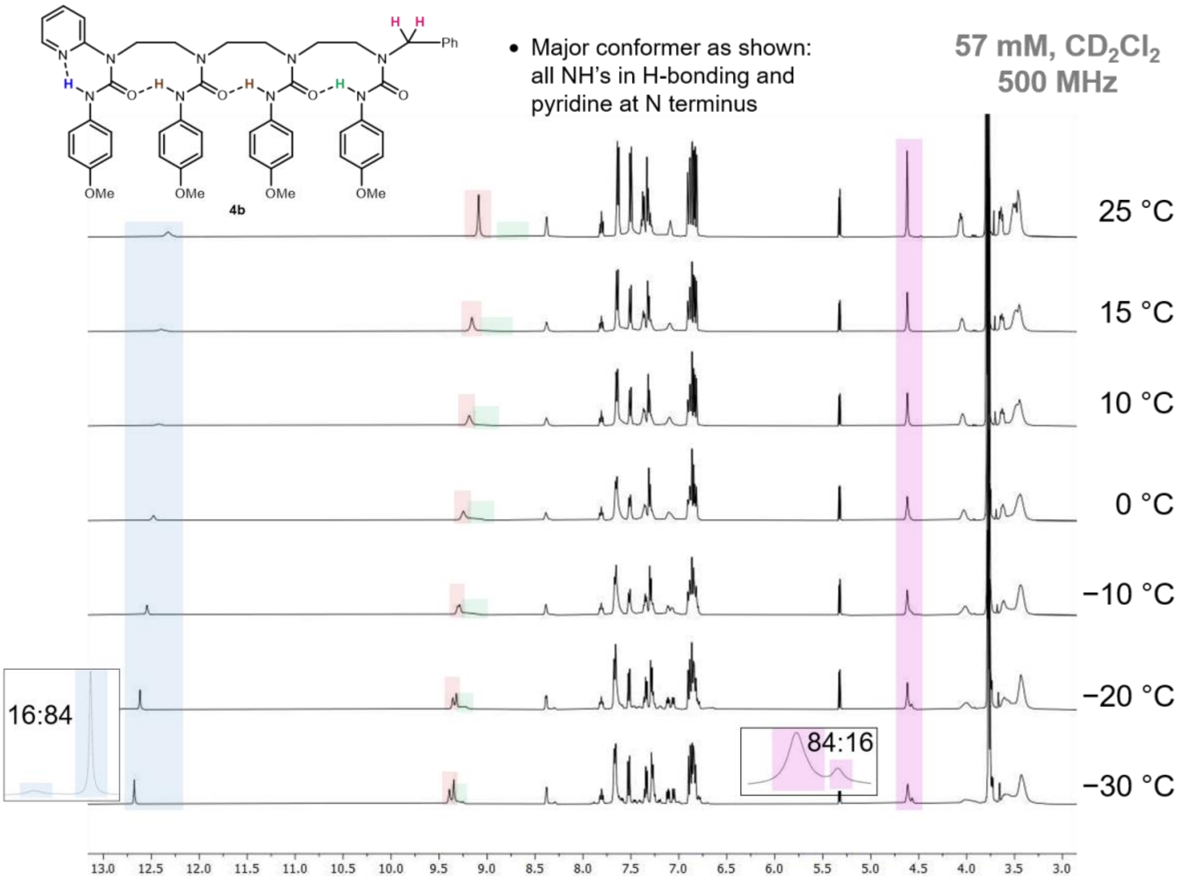

Figure S31 – VT NMR data for 4b (500 MHz, CD_2Cl_2, 57 mM).

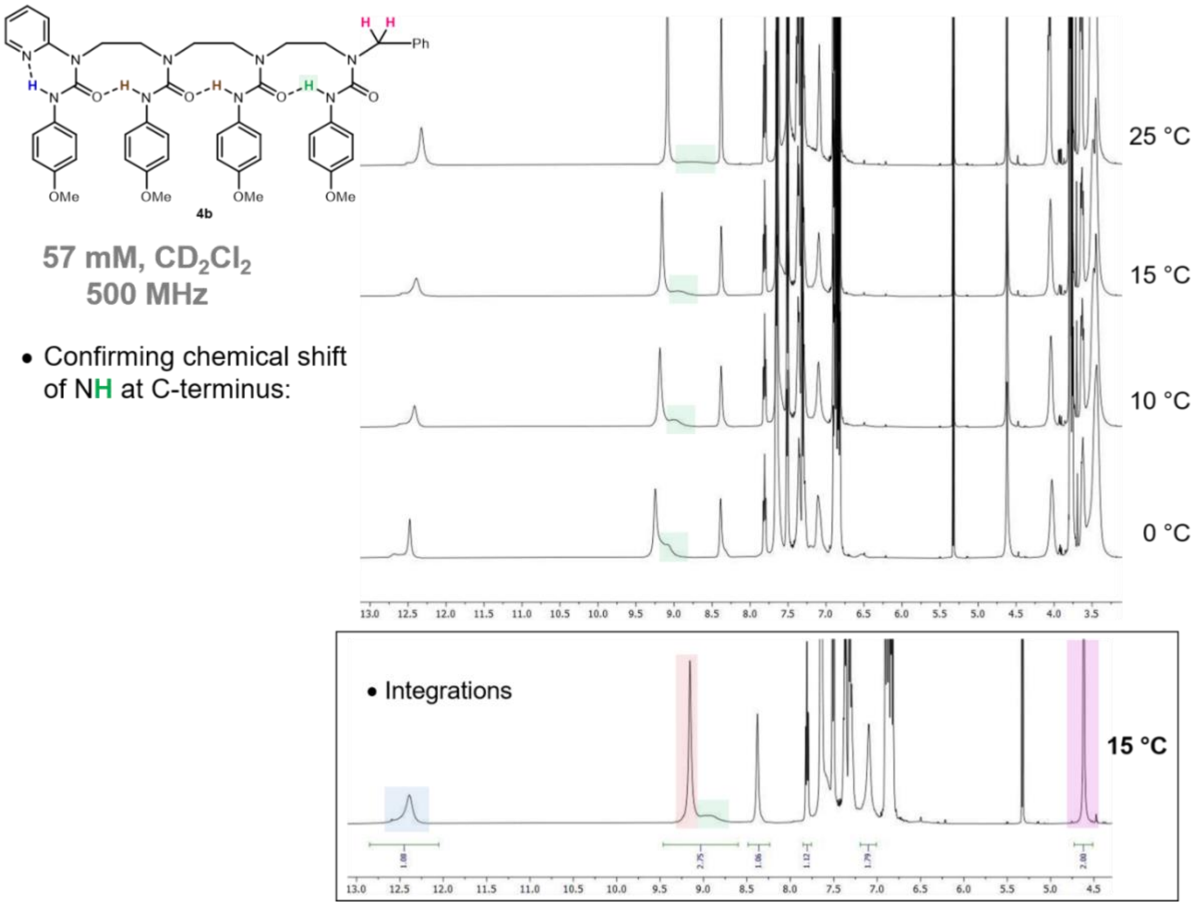

Figure S32 – VT NMR data for monitoring the C-terminal N–H of 4b (500 MHz, CD_2Cl_2, 57 mM).

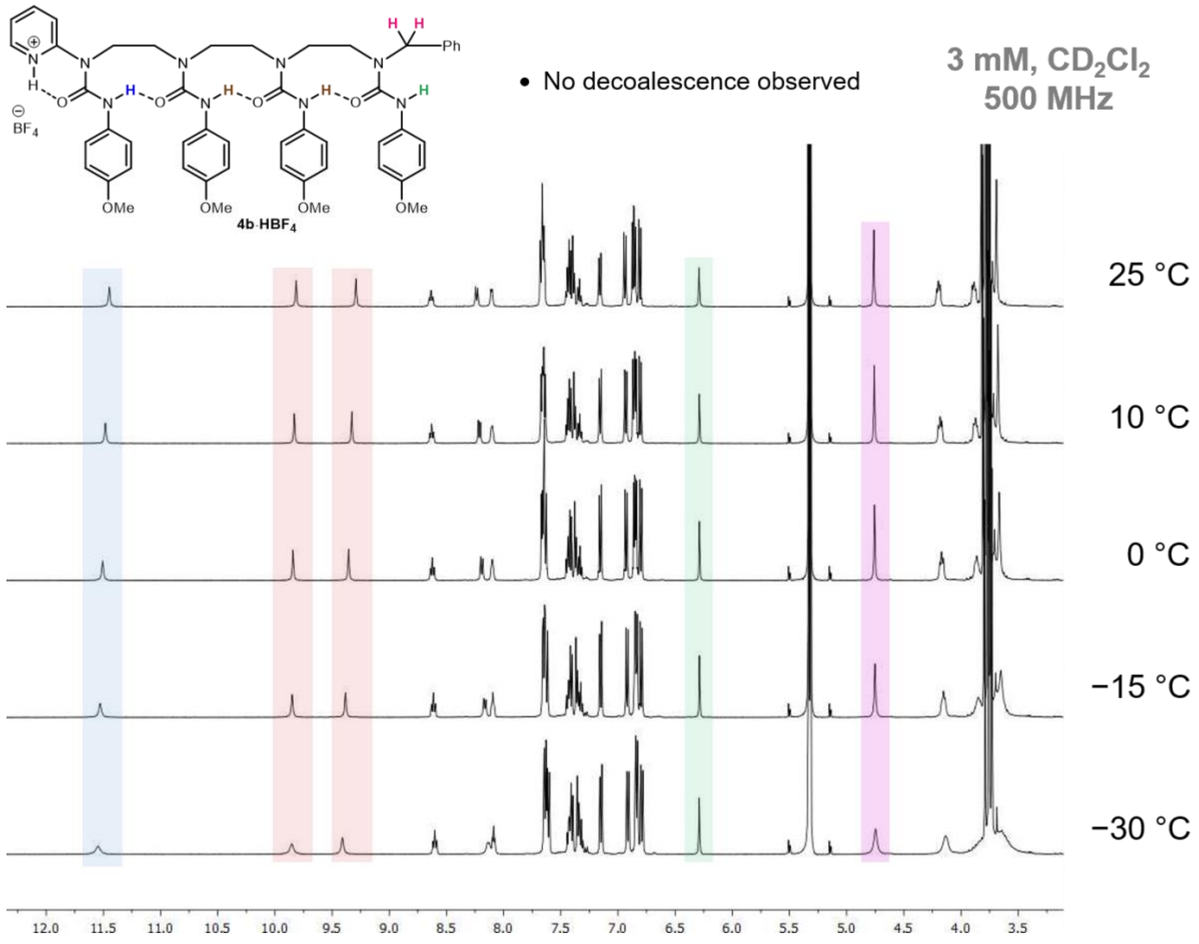

Figure S33 – VT NMR data for 4b.HBF_4 (500 MHz, CD_2Cl_2, 3 mM).

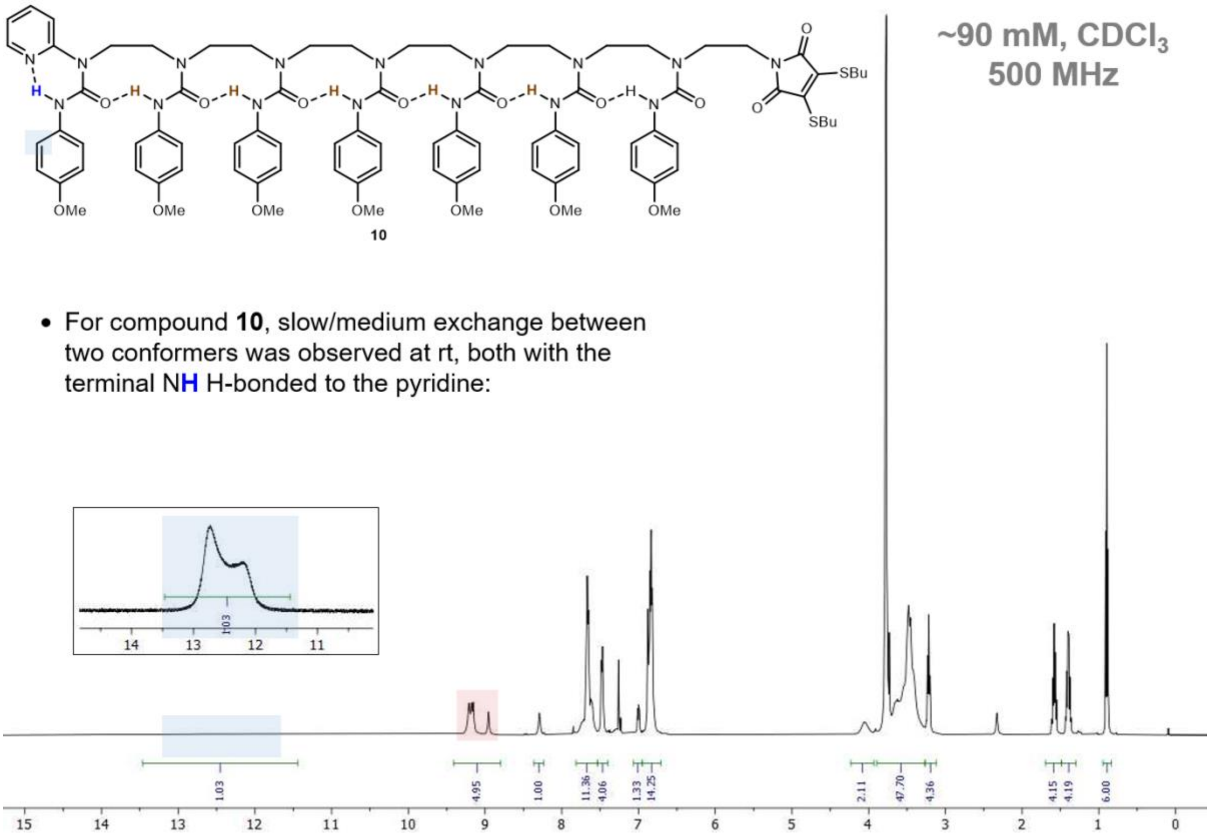

Figure S34 – ^{1}H NMR spectrum of 10 (500 MHz, CDCl$_3$, 90 mM).

- Coalescence was observed upon heating:

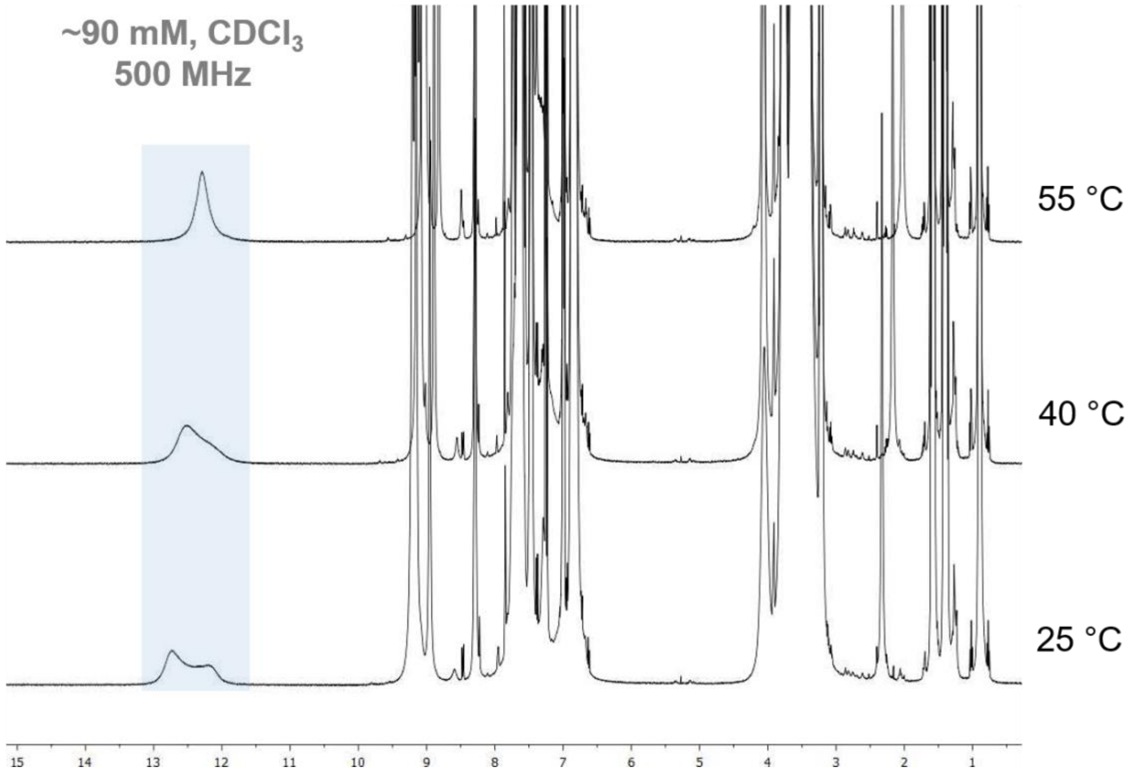

Figure S35 – VT NMR data for 10 (500 MHz, CDCl$_3$, 90 mM).

Eyring Analyses

All Eyring plots were constructed using rate constants that were attained by line shape fitting of experimental spectra at different temperatures. Spectra were simulated using SpinWorks 4 NMR processing software (available at ftp://davinci.chem.umanitoba.ca/pub/marat/SpinWorks/) with appropriate exchange vectors. For each temperature, the corresponding rate of hydrogen-bond-directionality reversal was estimated using the dynamic NMR simulation module DNMR3. The Eyring-Polanyi equation was rearranged to give an equation of the form y = mx + c such that a plot of *1/T* against *ln(k/T)* gives a straight line of gradient $\frac{-\Delta H^{\ddagger}}{R}$ and intercept $ln\frac{k_B}{h}+\frac{\Delta S^{\ddagger}}{R}$. These data were used with the Gibbs free energy equation to extrapolate $\Delta G^{\ddagger}$ at 298 K. Examples of simulation of systems in mutual exchange and non-mutual exchange are shown below.

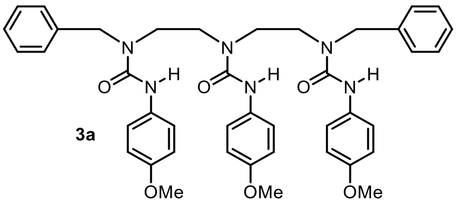

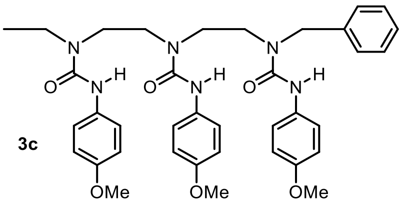

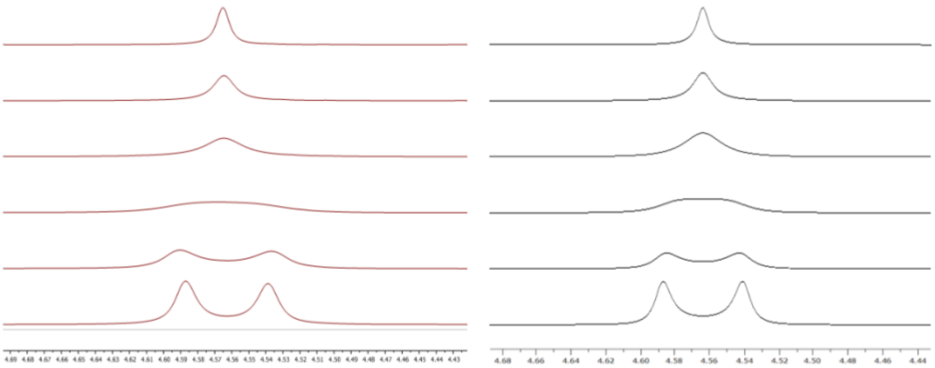

T / K	*k* / s^{-1}
298	250
288	150
278	90
268	51
258	27
248	17

Figure S36 – Experimental (left) and simulated (right) spectra for 3a.

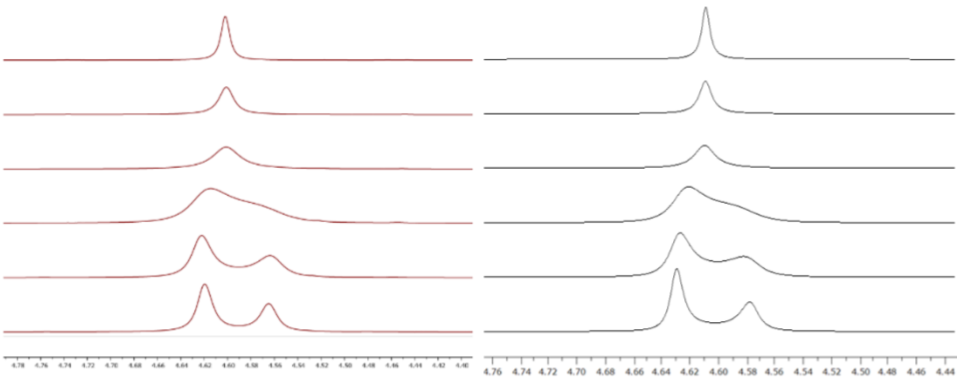

T / K	*k* / s^{-1}
298	330
288	200
278	120
268	44
258	27
248	15

Figure S37 – Experimental (left) and simulated (right) spectra for 3c.

Table S1 – Eyring plot for 1.

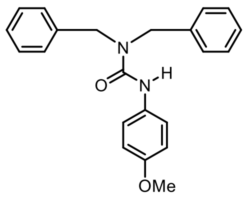

$\Delta G^{\ddagger}_{298\ K}$ = 48.9 kJ mol^{-1}

(CD_2Cl_2)

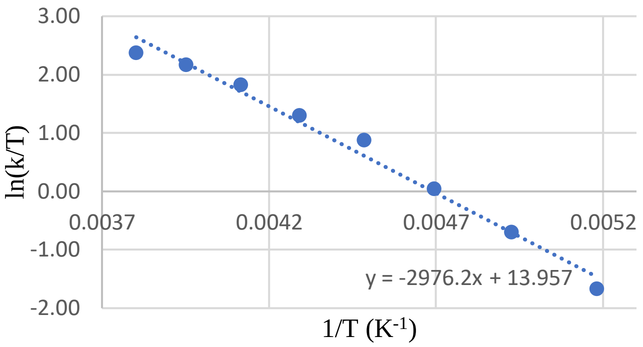

Temperature (K)	Rate Constant (s^{-1})	1/T (K^{-1})	ln(k/T)	ΔG‡ (kJ mol^{-1})	$t_{½}$ (s)
193	36	0.0052	-1.6792	40.4763	0.0096
203	100	0.0049	-0.7080	41.2913	0.0035
213	220	0.0047	0.0323	42.1064	0.0016
223	530	0.0045	0.8657	42.9215	0.0007
233	850	0.0043	1.2942	43.7365	0.0004
243	1500	0.0041	1.8202	44.5516	0.0002
253	2200	0.0040	2.1628	45.3667	0.0002
263	2800	0.0038	2.3652	46.1817	0.0001

Table S2 – Eyring plot for 1 (40% CD_3OH in CD_2Cl_2).

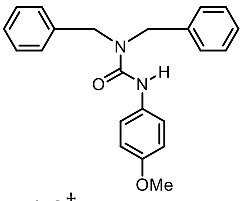

$\Delta G^{\ddagger}_{298\ K}$ = 59.9 kJ mol^{-1}

(40% CD_3OD in CD_2Cl_2)

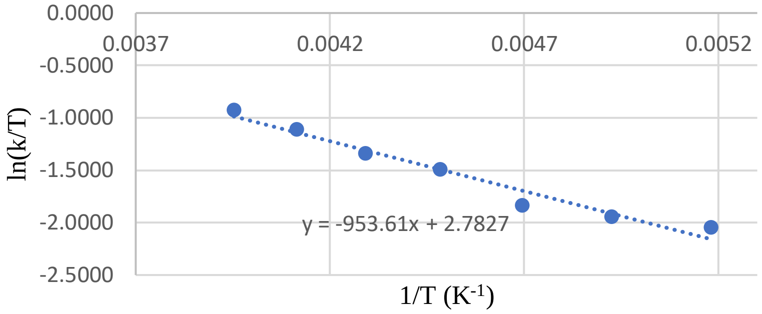

Temperature (K)	Rate Constant (s^{-1})	1/T (K^{-1})	ln(k/T)	ΔG‡ (kJ mol^{-1})	$t_{½}$ (s)
193	25	0.0052	-2.0438	41.5908	0.0139
203	29	0.0049	-1.9459	43.3350	0.0120
213	34	0.0047	-1.8349	45.0791	0.0102
223	50	0.0045	-1.4951	46.8233	0.0069
233	61	0.0043	-1.3402	48.5674	0.0057
243	80	0.0041	-1.1110	50.3116	0.0043
253	100	0.0040	-0.9282	52.0557	0.0035

Table S3 – Eyring plot for 1 (10% $(CD_3)_2SO$ in CD_2Cl_2).

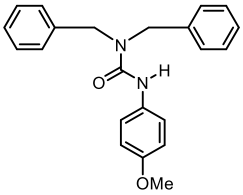

$\Delta G^{\ddagger}_{298\ K}$ = 54.5 kJ mol^{-1}

(10% $(CD_3)_2SO$ in CD_2Cl_2)

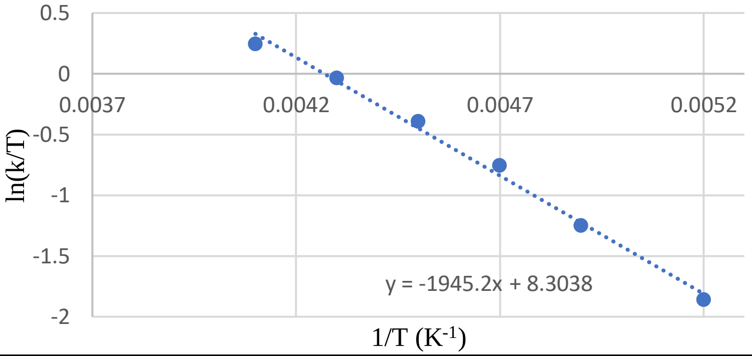

Temperature (K)	Rate Constant (s^{-1})	1/T (K^{-1})	ln(k/T)	ΔG‡ (kJ mol^{-1})	$t_{½}$ (s)
193	30	0.0052	-1.8615	40.9945	0.0116
203	58	0.0049	-1.2528	42.2732	0.0060
213	100	0.0047	-0.7561	43.5518	0.0035
223	150	0.0045	-0.3965	44.8305	0.0023
233	225	0.0043	-0.0349	46.1091	0.0015
243	310	0.0041	0.2435	47.3878	0.0011

Table S4 – Eyring plot for 2a.

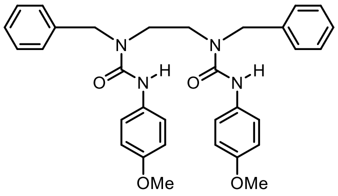

$\Delta G^{\ddagger}_{298\,K}$ = 59.4 kJ mol^{-1}

(CD_2Cl_2)

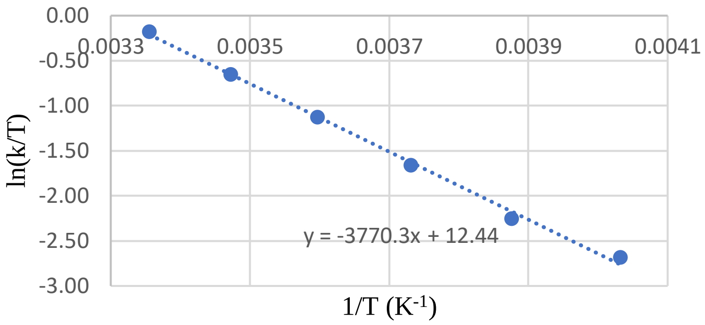

Temperature (K)	Rate Constant (s^{-1})	1/T (K^{-1})	ln(k/T)	ΔG‡ (kJ mol^{-1})	$t_{1/2}$ (s)
248	17	0.0040	-2.6802	54.6897	0.0204
258	27	0.0039	-2.2571	55.6309	0.0128
268	51	0.0037	-1.6592	56.5721	0.0068
278	90	0.0036	-1.1278	57.5133	0.0039
288	150	0.0035	-0.6523	58.4544	0.0023
298	250	0.0034	-0.1756	59.3956	0.0014

Table S5 – Eyring plot for 2a (40% CD_3OH in CD_2Cl_2).

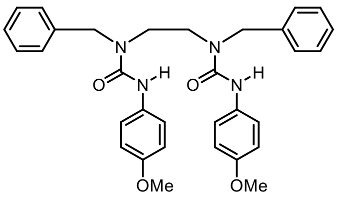

$\Delta G^{\ddagger}_{298\,K}$ = 55.3 kJ mol^{-1}

(40% CD_3OD in CD_2Cl_2)

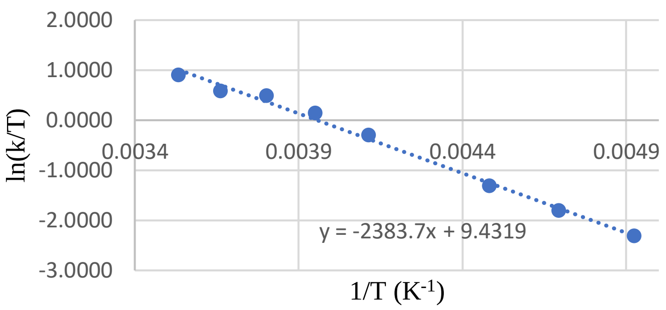

Temperature (K)	Rate Constant (s^{-1})	1/T (K^{-1})	ln(k/T)	ΔG‡ (kJ mol^{-1})	$t_{1/2}$ (s)
203	20	0.0049	-2.3175	44.0026	0.0173
213	35	0.0047	-1.8059	45.1939	0.0099
223	60	0.0045	-1.3128	46.3852	0.0058
243	180	0.0041	-0.3001	48.7678	0.0019
253	290	0.0040	0.1365	49.9591	0.0012
263	430	0.0038	0.4916	51.1504	0.0008
273	490	0.0037	0.5849	52.3418	0.0007
283	700	0.0035	0.9056	53.5331	0.0005

Table S6 – Eyring plot for 2a (10% $(CD_3)_2SO$ in CD_2Cl_2).

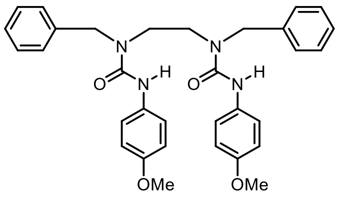

$\Delta G^{\ddagger}_{298\,K}$ = 52.3 kJ mol^{-1}

(10% $(CD_3)_2SO$ in CD_2Cl_2)

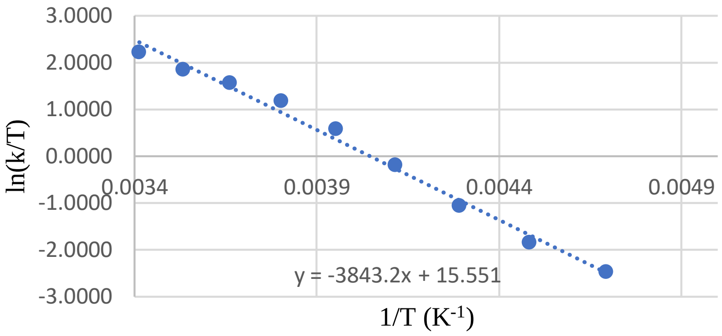

**Temperature (K)**	**Rate Constant (s^{-1})**	**1/T (K^{-1})**	**ln(k/T)**	**ΔG‡ (kJ mol^{-1})**	**$t_{½}$ (s)**
213	18	0.0047	-2.4709	46.4921	0.0193
223	35	0.0045	-1.8518	47.1746	0.0099
233	80	0.0043	-1.0690	47.8572	0.0043
243	200	0.0041	-0.1947	48.5397	0.0017
253	450	0.0040	0.5759	49.2222	0.0008
263	850	0.0038	1.1731	49.9048	0.0004
273	1300	0.0037	1.5606	50.5873	0.0003
283	1800	0.0035	1.8501	51.2698	0.0002

Table S7 – Eyring plot for 2b.

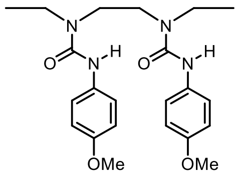

$\Delta G^{\ddagger}_{298\ K}$ = 51.5 kJ mol^{-1}

(CD_2Cl_2)

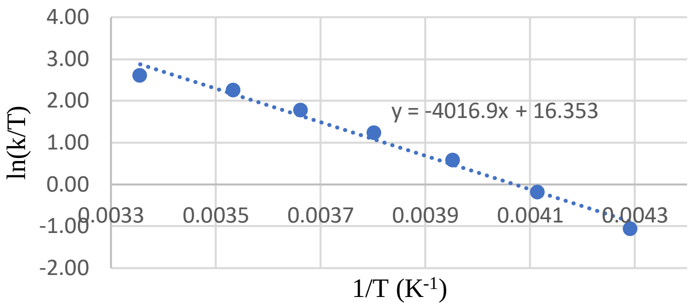

Temperature (K)	Rate Constant (s^{-1})	1/T (K^{-1})	ln(k/T)	ΔG‡ (kJ mol^{-1})	t½ (s)
193	55	0.0052	-1.2554	45.5790	0.0063
233	80	0.0043	-1.0690	47.8309	0.0043
243	200	0.0041	-0.1947	48.3939	0.0017
253	450	0.0040	0.5759	48.9569	0.0008
263	900	0.0038	1.2302	49.5198	0.0004
273	1600	0.0037	1.7683	50.0828	0.0002
283	2700	0.0035	2.2556	50.6458	0.0001
298	4000	0.0034	2.5970	51.4902	0.0001

Table S8 – Eyring plot for 3a.

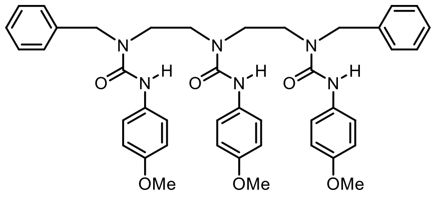

$\Delta G^{\ddagger}_{298\ K}$ = 59.0 kJ mol^{-1}

(CD_2Cl_2)

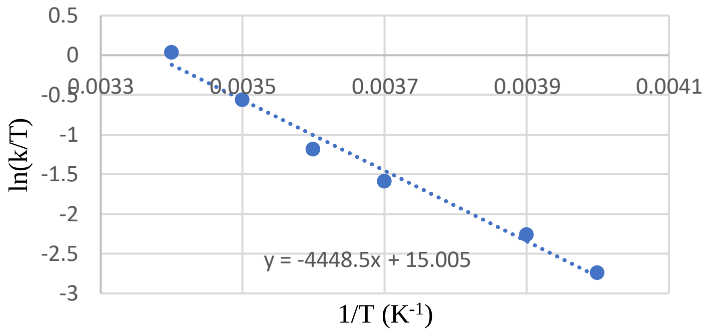

Temperature (K)	Rate Constant (s^{-1})	1/T (K^{-1})	ln(k/T)	ΔG‡ (kJ mol^{-1})	t½ (s)
248	16	0.0040	-2.7408	54.8299	0.0217
258	27	0.0039	-2.2571	55.6705	0.0128
268	55	0.0037	-1.5837	56.5110	0.0063
278	85	0.0036	-1.1850	57.3515	0.0041
288	165	0.0035	-0.5570	58.1920	0.0021
298	310	0.0034	0.0395	59.0325	0.0011

Table S9 – Eyring plot for 3a (40% CD_3OH in CD_2Cl_2).

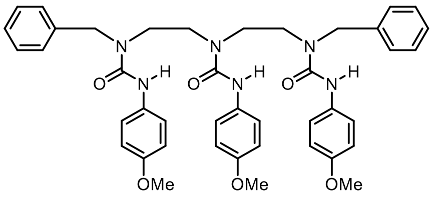

$\Delta G^{\ddagger}_{298\ K}$ = 58.6 kJ mol^{-1}

(40% CD_3OD in CD_2Cl_2)

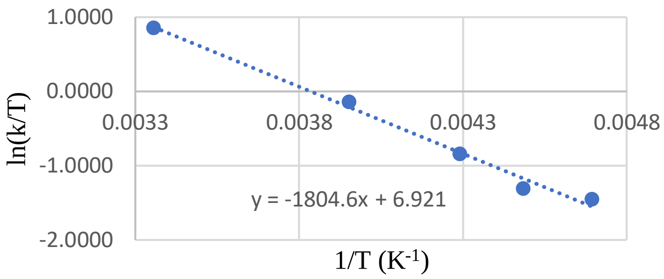

Temperature (K)	Rate Constant (s^{-1})	1/T (K^{-1})	ln(k/T)	ΔG‡ (kJ mol^{-1})	$t_{½}$ (s)
213	50	0.0047	-1.4493	52.4842	0.0069
223	60	0.0045	-1.3128	53.2037	0.0058
233	100	0.0043	-0.8459	53.9233	0.0035
253	220	0.0040	-0.1398	55.3623	0.0016
298	700	0.0034	0.8540	58.6002	0.0005

Table S10 – Eyring plot for 3a (10% $(CD_3)_2SO$ in CD_2Cl_2).

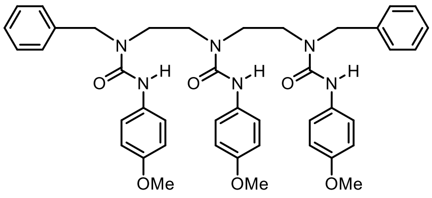

$\Delta G^{\ddagger}_{298\ K}$ = 54.9 kJ mol^{-1}

(10% $(CD_3)_2SO$ in CD_2Cl_2)

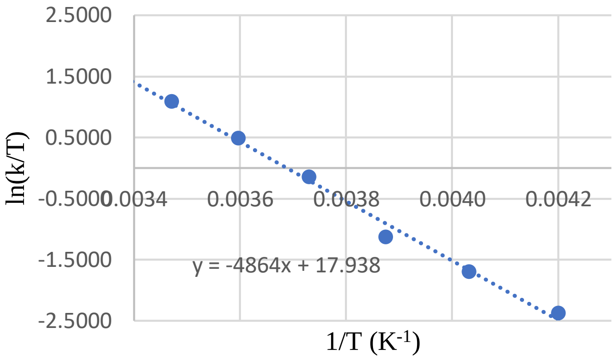

Temperature (K)	Rate Constant (s^{-1})	1/T (K^{-1})	ln(k/T)	ΔG‡ (kJ mol^{-1})	$t_{½}$ (s)
238	22	0.0042	-2.3812	51.9623	0.0158
248	45	0.0040	-1.7068	52.4464	0.0077
258	83	0.0039	-1.1341	52.9305	0.0042
268	230	0.0037	-0.1529	53.4145	0.0015
278	450	0.0036	0.4816	53.8986	0.0008
288	850	0.0035	1.0823	54.3827	0.0004
298	1500	0.0034	1.6161	54.8667	0.0002

Table S11 – Eyring plot for 3b.

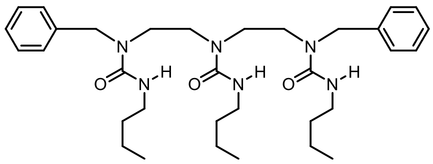

$\Delta G^{\ddagger}_{298\ K}$ = 57.9 kJ mol^{-1}

(CD_2Cl_2)

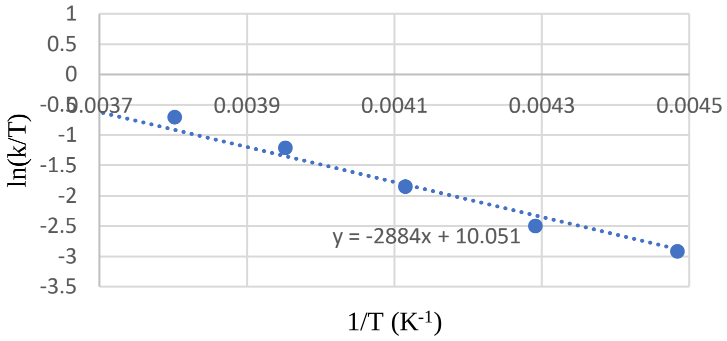

Temperature (K)	Rate Constant (s^{-1})	1/T (K^{-1})	ln(k/T)	ΔG‡ (kJ mol^{-1})	t½ (s)
223	12	0.0045	-2.9223	49.3977	0.0289
233	19	0.0043	-2.5066	50.5376	0.0182
243	38	0.0041	-1.8555	51.6775	0.0091
253	75	0.0040	-1.2159	52.8173	0.0046
263	130	0.0038	-0.7046	53.9572	0.0027
273	198	0.0037	-0.3212	55.0970	0.0018
283	260	0.0035	-0.0848	56.2369	0.0013
298	310	0.0034	0.0395	57.9467	0.0011

Table S12 – Eyring plot for 4a.

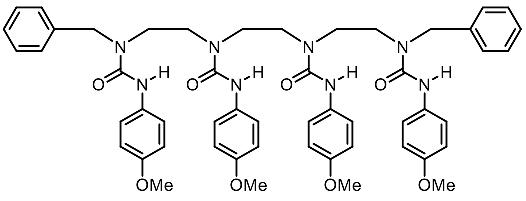

$\Delta G^{\ddagger}_{298\ K}$ = 60.9 kJ mol^{-1}

(CD_2Cl_2)

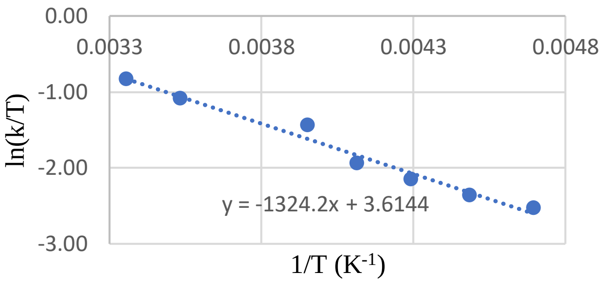

Temperature (K)	Rate Constant (s^{-1})	1/T (K^{-1})	ln(k/T)	ΔG‡ (kJ mol^{-1})	t½ (s)
213	17	0.0047	-2.5281	46.6874	0.0204
223	21	0.0045	-2.3626	48.3624	0.0165
233	27	0.0043	-2.1552	50.0374	0.0128
243	35	0.0041	-1.9377	51.7124	0.0099
253	60	0.0040	-1.4390	53.3874	0.0058
283	95	0.0035	-1.0916	58.4124	0.0036
298	130	0.0034	-0.8296	60.9249	0.0027

Table S13 – Eyring plot for 4a.

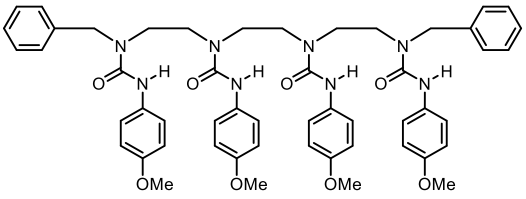

$\Delta G^{\ddagger}_{298\,K}$ = 58.0 kJ mol^{-1}

(40% CD_3OH in CD_2Cl_2)

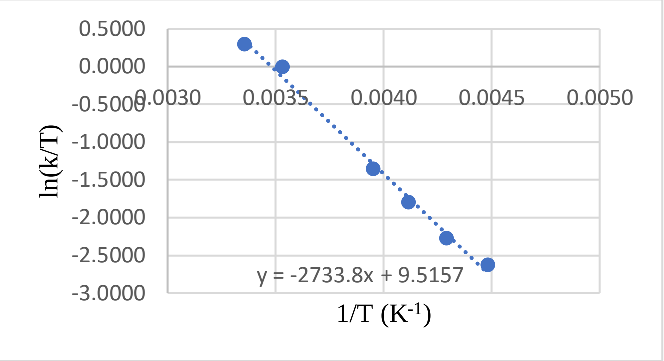

Temperature (K)	Rate Constant (s^{-1})	1/T (K^{-1})	ln(k/T)	ΔG‡ (kJ mol^{-1})	t$_{½}$ (s)
223	16	0.0045	-2.6346	49.1408	0.0217
233	24	0.0043	-2.2730	50.3251	0.0144
243	40	0.0041	-1.8042	51.5094	0.0087
253	65	0.0040	-1.3590	52.6938	0.0053
283	280	0.0035	-0.0107	56.2468	0.0012
298	400	0.0034	0.2944	58.0233	0.0009

Table S14 – Eyring plot for 4a (10% $(CD_3)_2SO$ in CD_2Cl_2).

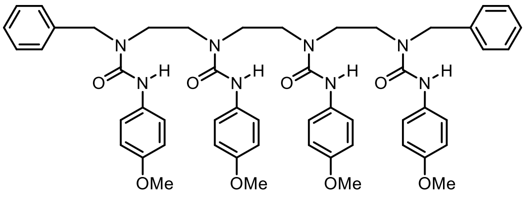

$\Delta G^{\ddagger}_{298\,K}$ = 53.7 kJ mol^{-1}

(10% $(CD_3)_2SO$ in CD_2Cl_2)

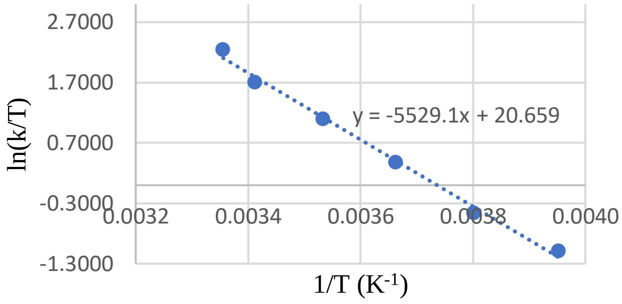

Temperature (K)	Rate Constant (s^{-1})	1/T (K^{-1})	ln(k/T)	ΔG‡ (kJ mol^{-1})	t$_{½}$ (s)
253	85	0.0040	-1.0907	52.4946	0.0041
263	165	0.0038	-0.4662	52.7524	0.0021
273	400	0.0037	0.3820	53.0102	0.0009
283	850	0.0035	1.0998	53.2681	0.0004
293	1600	0.0034	1.6976	53.5259	0.0002
298	2800	0.0034	2.2403	53.6548	0.0001

Table S15 – Eyring plot for 3c (minor conformer to major conformer).

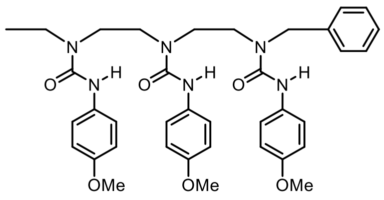

$\Delta G^{\ddagger}_{298\,K}$ = 58.1 kJ mol^{-1}
(minor conformer to major conformer)

$\Delta G^{\ddagger}_{298\,K}$ = 59.2 kJ mol^{-1}

(major conformer to minor conformer)

(CD_2Cl_2)

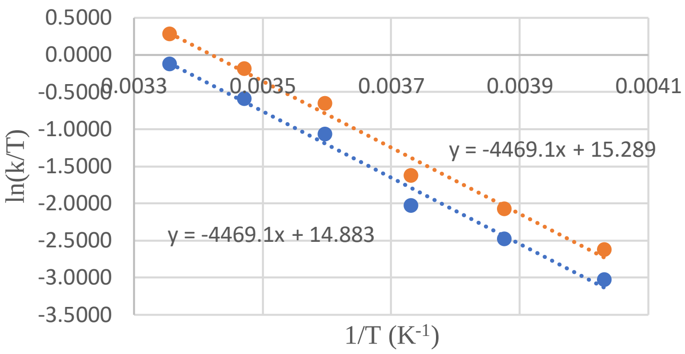

Temperature (K)	Rate Constant (s^{-1})	1/T (K^{-1})	ln(k/T)	ΔG‡ (kJ mol^{-1})	$t_{1/2}$ (s)
248	18	0.0040	-2.6231	54.6252	0.0193
258	32	0.0039	-2.0748	55.3295	0.0107
268	52	0.0037	-1.6245	56.0338	0.0066
278	144	0.0036	-0.6578	56.7382	0.0024
288	240	0.0035	-0.1823	57.4425	0.0014
298	396	0.0034	0.2843	58.1468	0.0009

Table S16 – Eyring plot for 3c (major conformer to minor conformer).

Temperature (K)	Rate Constant (s^{-1})	1/T (K^{-1})	ln(k/T)	ΔG‡ (kJ mol^{-1})	$t_{1/2}$ (s)
248	12	0.0040	-3.0285	55.4624	0.0289
258	22	0.0039	-2.4803	56.2005	0.0160
268	35	0.0037	-2.0299	56.9385	0.0098
278	96	0.0036	-1.0633	57.6766	0.0036
288	160	0.0035	-0.5878	58.4147	0.0022
298	264	0.0034	-0.1211	59.1527	0.0013

nOe Experiments

All 1D nOe studies were conducted on a Bruker AVANCE III HD 500 MHz NMR Spectrometer with 5 mm DCH ^{13}C–^{1}H/D Cryo Probe (500 MHz) at 0 °C in CD_2Cl_2.

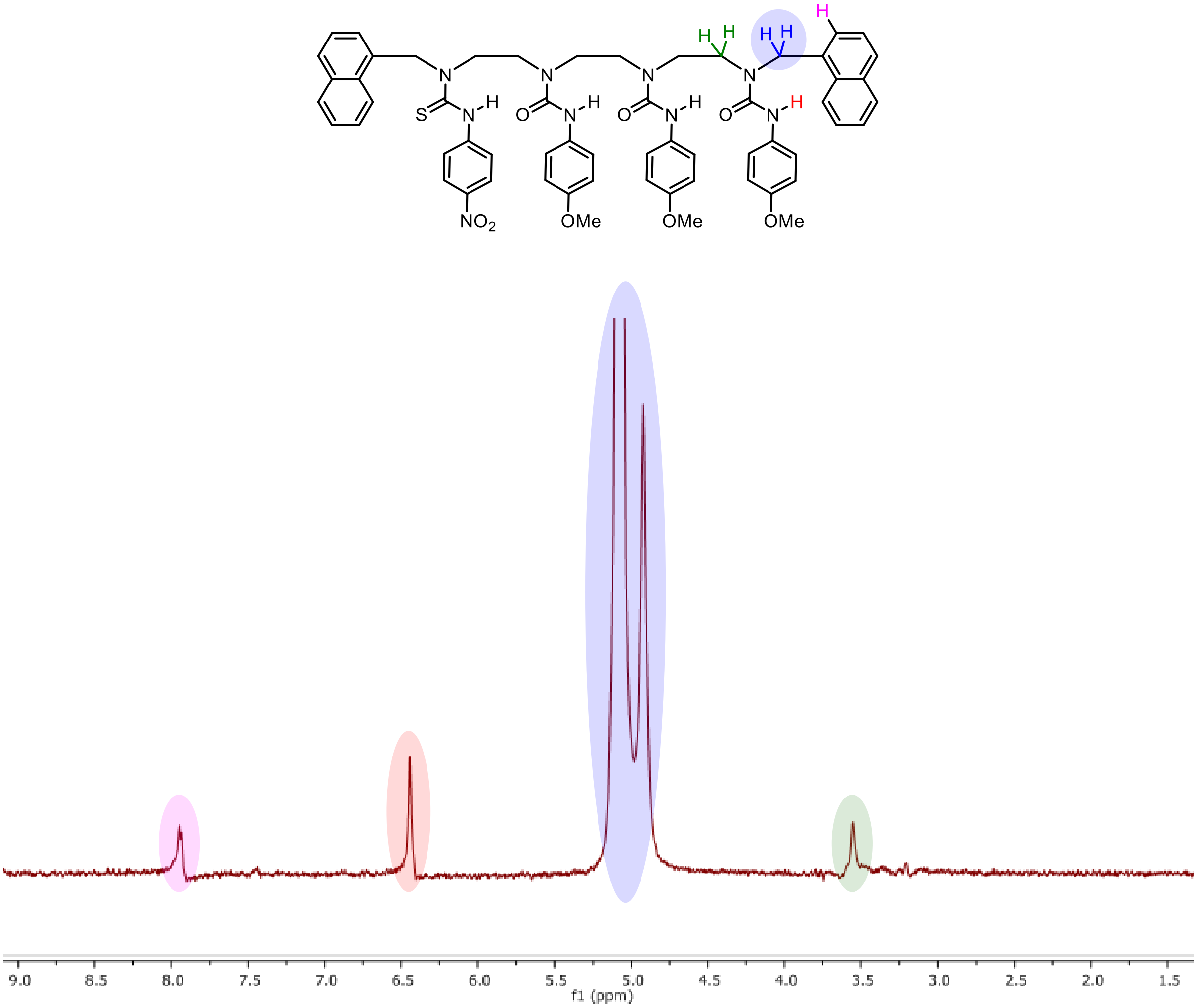

Figure S38 – nOe studies of 5a (irradiation of blue).

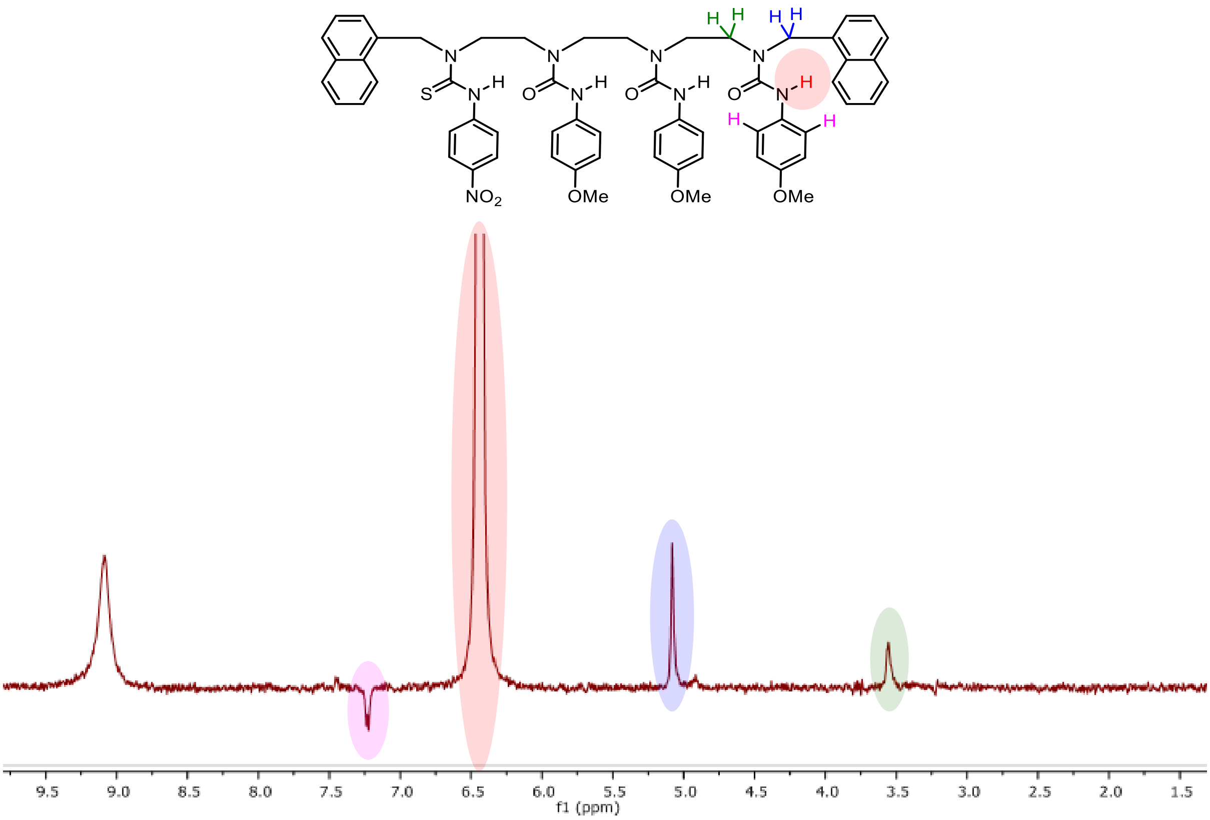

Figure S39 – nOe studies of 5a (irradiation of red).

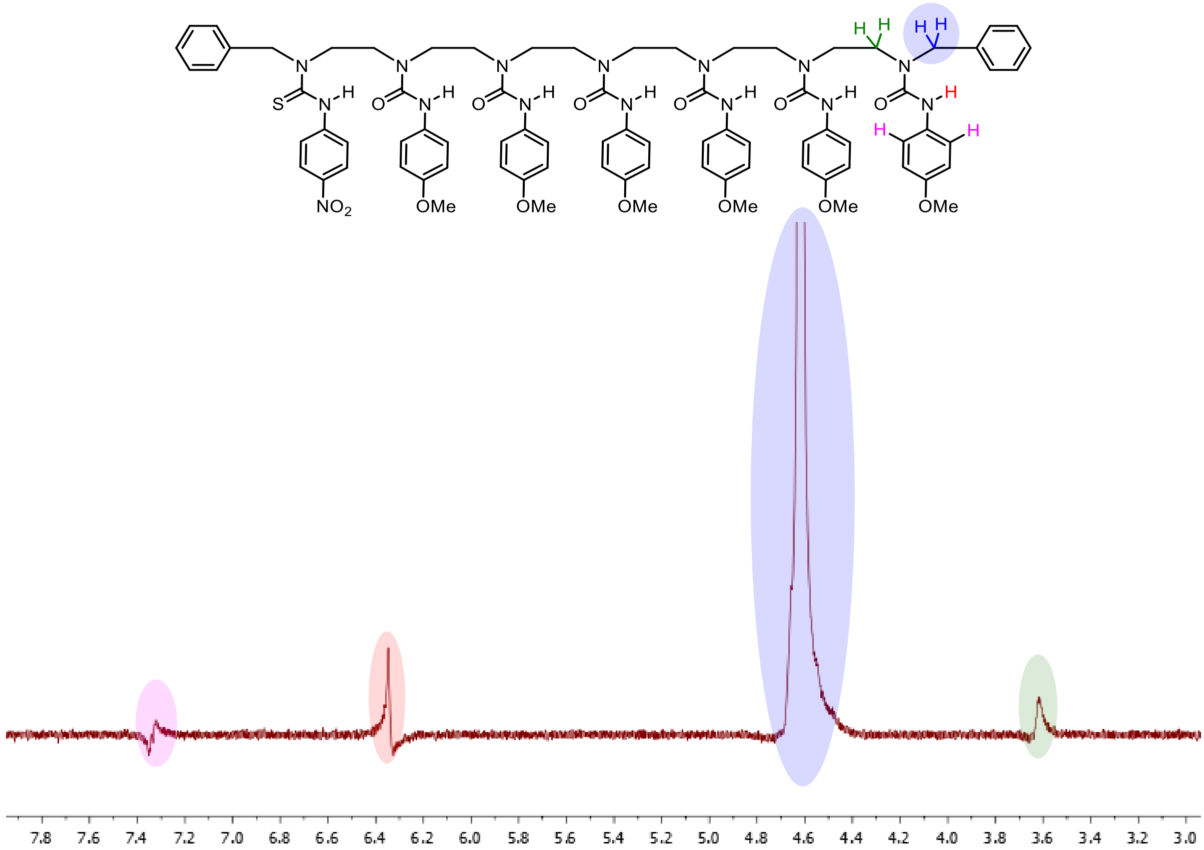

Figure S40 – nOe studies of 5b (irradiation of blue).

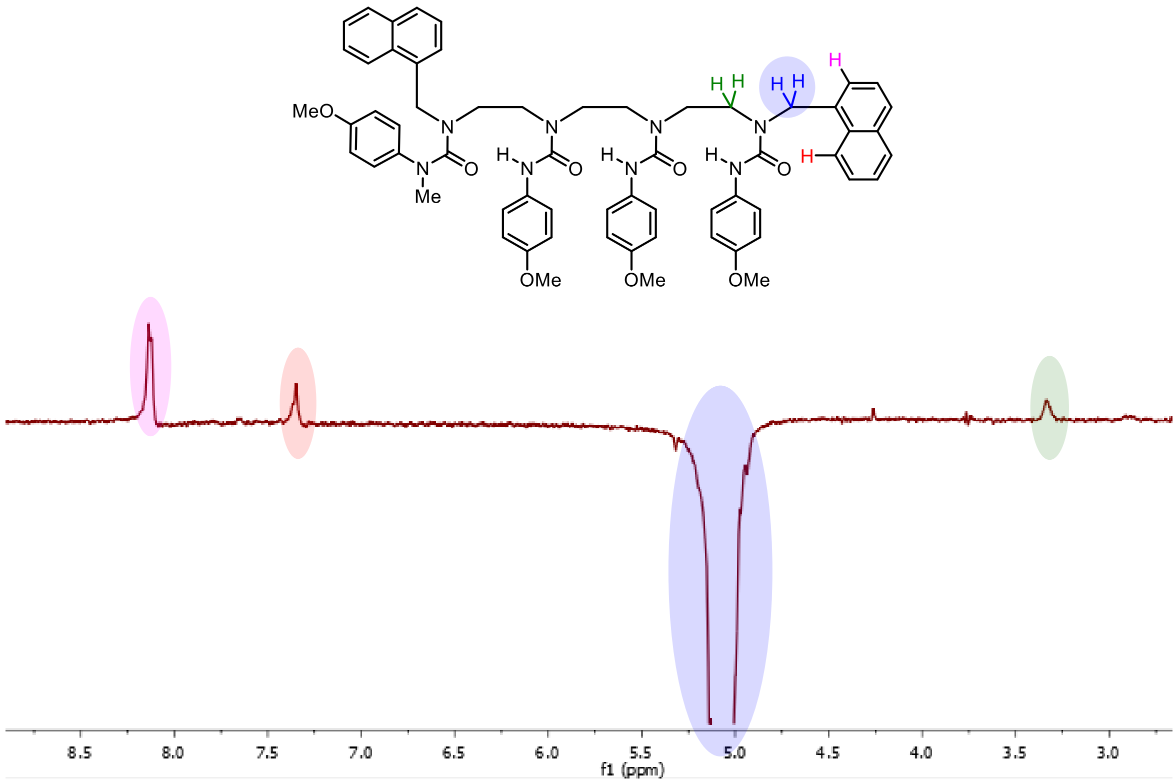

Figure S41 – nOe studies of 6a (irradiation of blue).

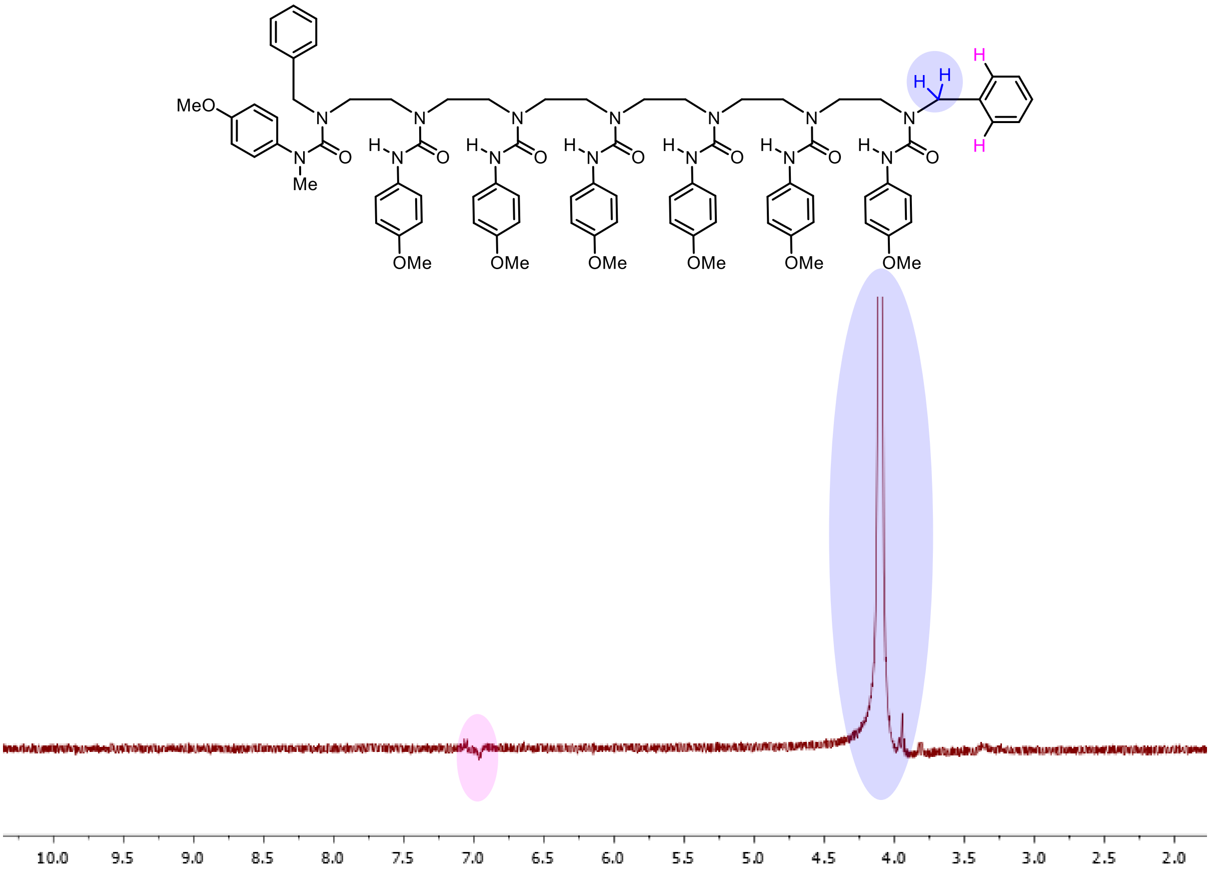

Figure S42 – nOe studies of 6b (irradiation of blue).

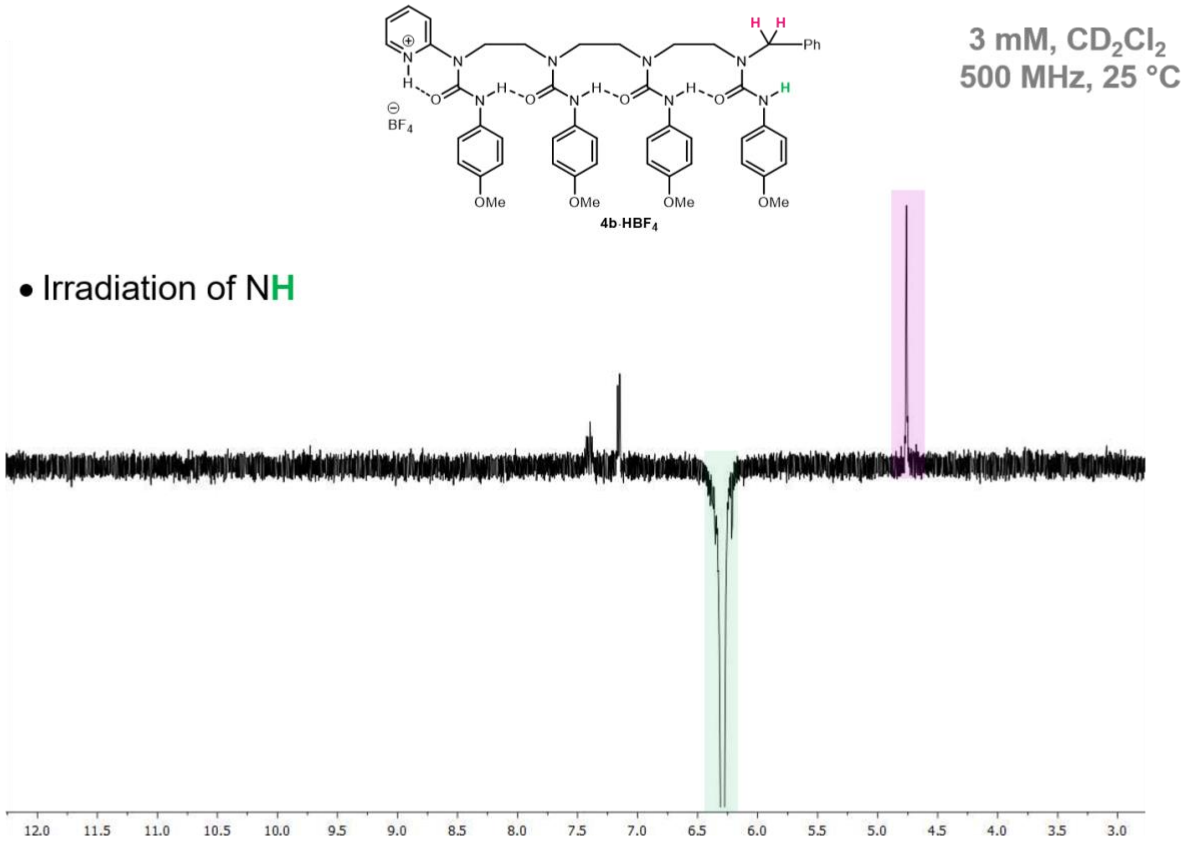

Figure S43 – nOe studies of 4b.HBF$_4$ (irradiation of green).

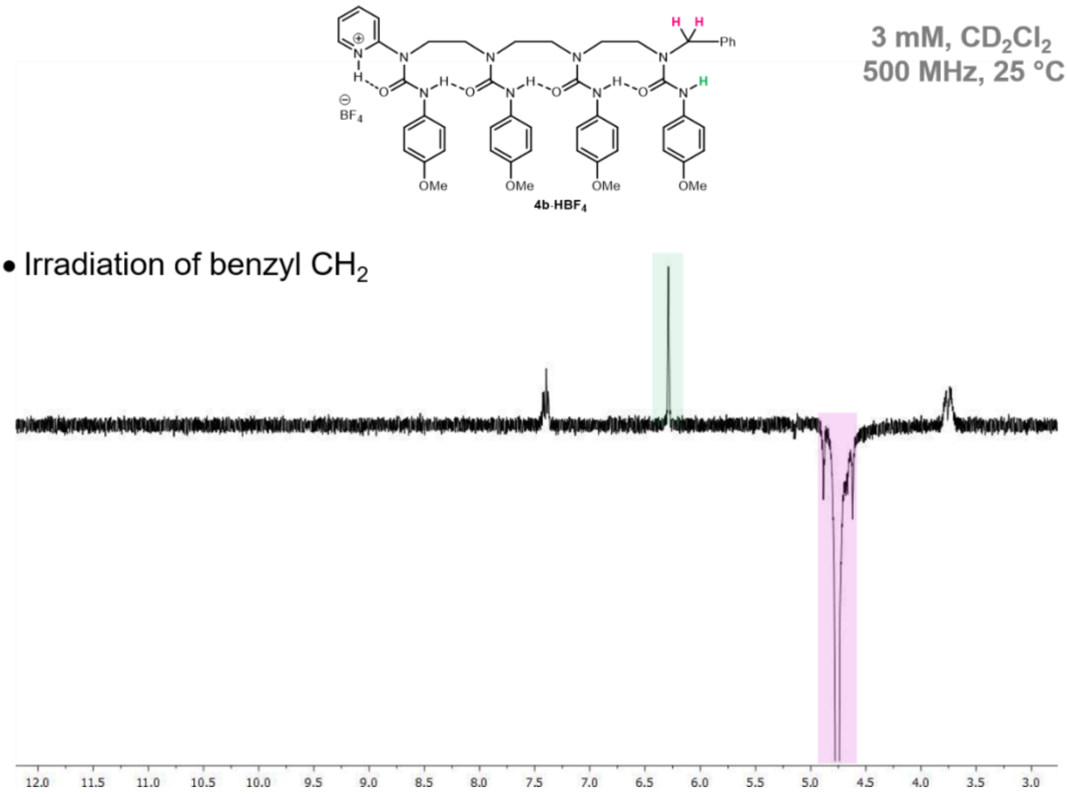

Figure S44 – nOe studies of 4b.HBF$_4$ (irradiation of pink).

Fluorimetry

Supplemental Experimental Procedures

Summary of Fluorescence Wavelength Maxima

Table S17 – Fluorescence wavelength maxima for compounds 7-10.

**Compound**	**CH_2Cl_2 at 1 mM**	
	**Excitation λ_{max} ($S_0 \rightarrow S_2$)**	**Emission λ_{max}**
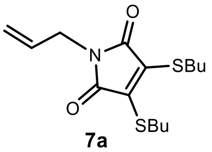 **7a**	339 nm	522 nm
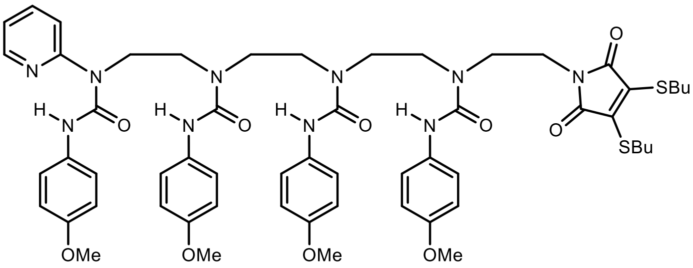 **8a**	356 nm	523 nm
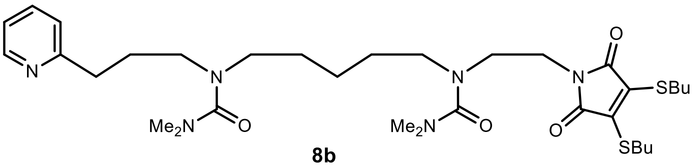 **8b**	344 nm	523 nm
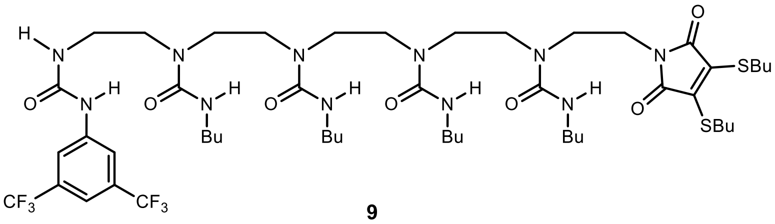 **9**	352 nm	530 nm
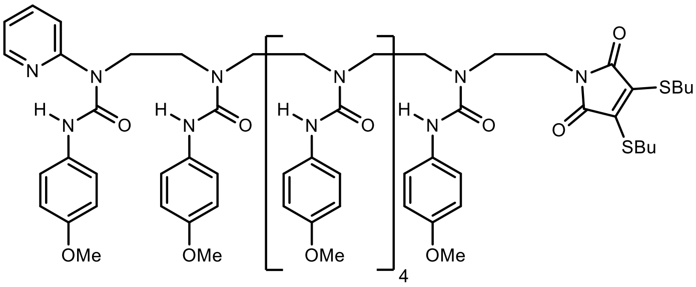 **10**	364 nm	525 nm

General Points

• All fluorescence experiments were carried out on a PerkinElmer LS45 fluorimeter at room temperature at a concentration of 1.0 mM in lab grade dichloromethane. Excitation spectra were recorded at the maximum emission wavelength for each substrate (see Table S17 above)

• All samples were prepared by directly weighing 2.0 μmol of the substrate into a glass fluorescence cuvette followed by the addition of 2.0 mL of dichloromethane

• All fluorescence spectral measurements represent the average of 2 accumulation scans

• Magnetic stirring of all samples was performed (using a micro stir bar inside the cuvette) with the stirring power set to 'high'. In all titrations, a homogenous solution was maintained throughout the entire experiments.

• The photomultiplier voltage used was dependent on the substrate: experiments with **7a**, **8b** and **9** were performed at 700 V; experiments with **8a** and **10** were performed at 900 V. The requirement for an increased photomultiplier voltage for compounds **8a** and **10** was due to their fluorescence intensities being approximately two orders of magnitude lower than the other compounds (at constant concentration and voltage) - see excitation spectra below:

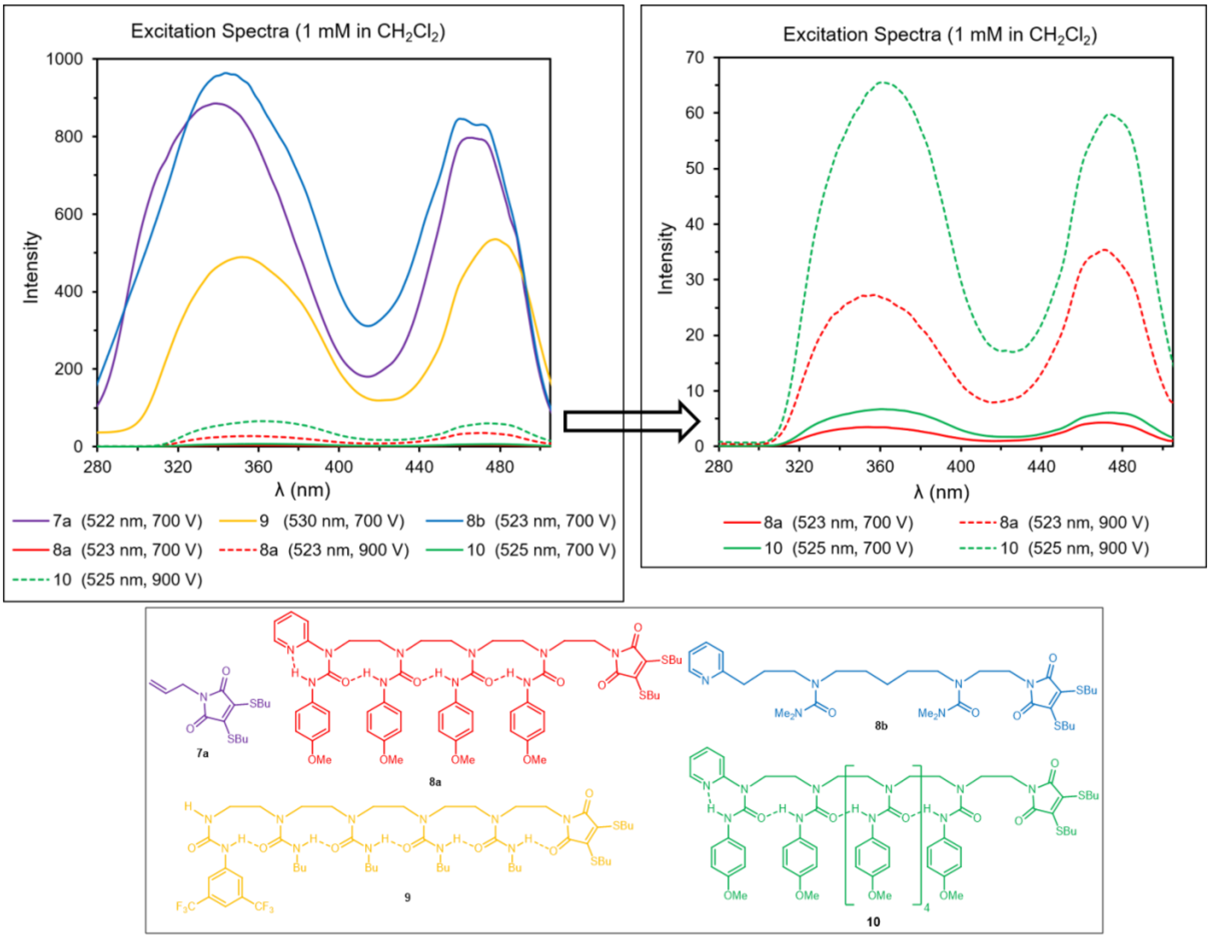

Figure S45 – Fluorescence traces for compounds 7-10.

Fluorescence Titrations with Acid

• ***General titration procedure with acid***: To a solution of the substrate (2.0 µmol) in lab grade dichloromethane (2.0 mL) in a glass cuvette at room temperature with magnetic stirring was sequentially added aliquots of a solution of $HBF_4{\cdot}Et_2O$ (0.0125 M in CH_2Cl_2, 20 µL each time = 0.25 µmol, 0.125 equiv) until 1.5 equiv of acid had been added (12 aliquots in total). A fluorescence spectrum was recorded after the addition of each aliquot.

• Stock solutions of HBF_4 for the titration experiments were prepared using the commercially available 1:1 complex with diethyl ether ($HBF_4{\cdot}Et_2O$: Aldrich 400068). Stock solutions were prepared in plastic bottles to prevent glass etching which was otherwise observed. $HBF_4{\cdot}Et_2O$ is an oil that is miscible with dichloromethane. Note, however, that over several hours/days, some droplets of $HBF_4{\cdot}Et_2O$ may be observed to precipitate out. Gentle shaking quickly restores the homogeneity. [During titrations, gentle shaking of the stock solution was always carried out before each new aliquot was added].

• Fluorescence intensities shown in acid titration spectra in the manuscript and below are not corrected for changes in concentration - the substrate concentrations decreased from 1.0 to 0.9 mM at the completion of the titrations.

• After completion of the fluorescence titration of foldamer **8a** with acid (see Fig 3b in manuscript), further evidence of protonation of the pyridine nitrogen and directionality switching was provided by ^{1}H NMR spectroscopy:

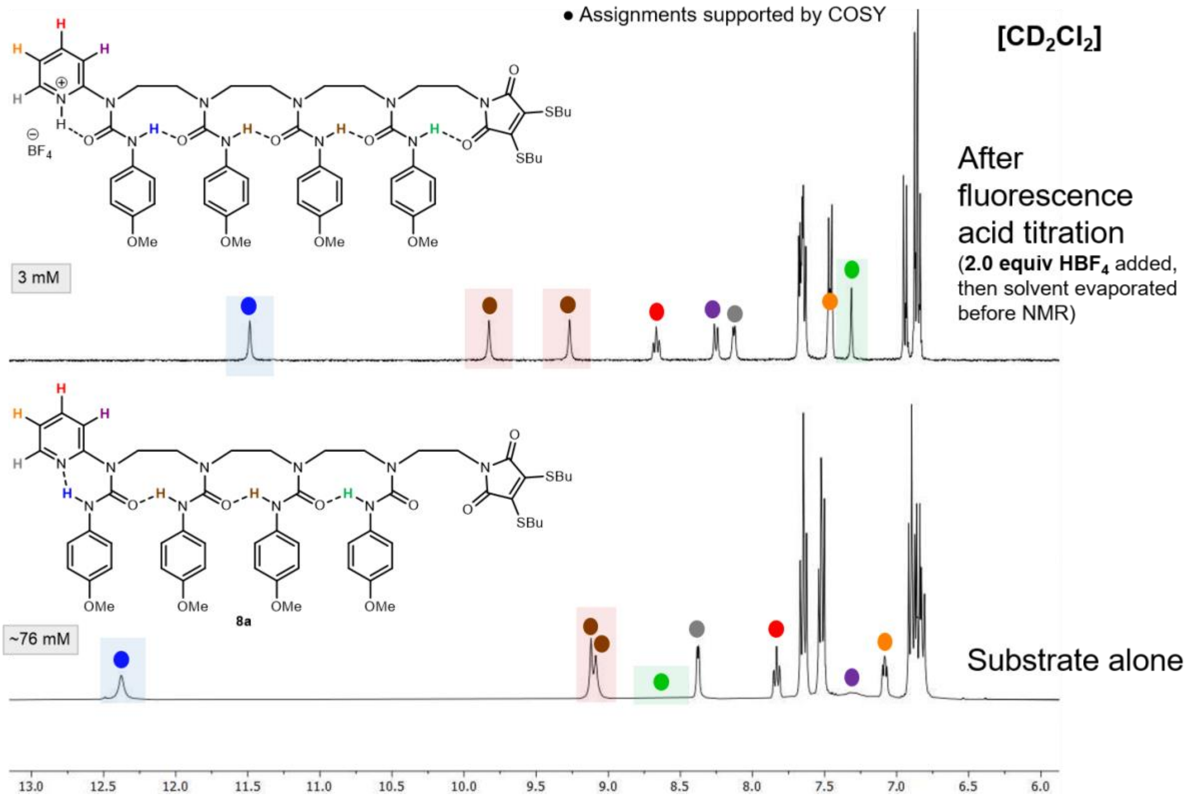

Figure S46 – NMR studies of 8a upon treatment with HBF_4.

• <u>Additional titrations with HBF_4</u>: The fluorescence spectra below show the effect of HBF_4 (0–1.5 equiv) in CH_2Cl_2 on the excitation spectrum (at 1 mM) of longer foldamer **10** (emission at 525 nm) and control compound **8b** (emission at 523 nm). As substoichiometric amounts of acid were sequentially added to **10**, the S_0–S_2 excitation maximum (λ_{max}) at 364 nm gradually red-shifted to 385 nm after 1 equiv. Minimal spectral changes were observed beyond 1 equiv of acid. Under the same conditions, **8b**

(without a linking chain of urea hydrogen bonds between the pyridine and the fluorophore) did not undergo any significant λ_{max} shifting during the titration.

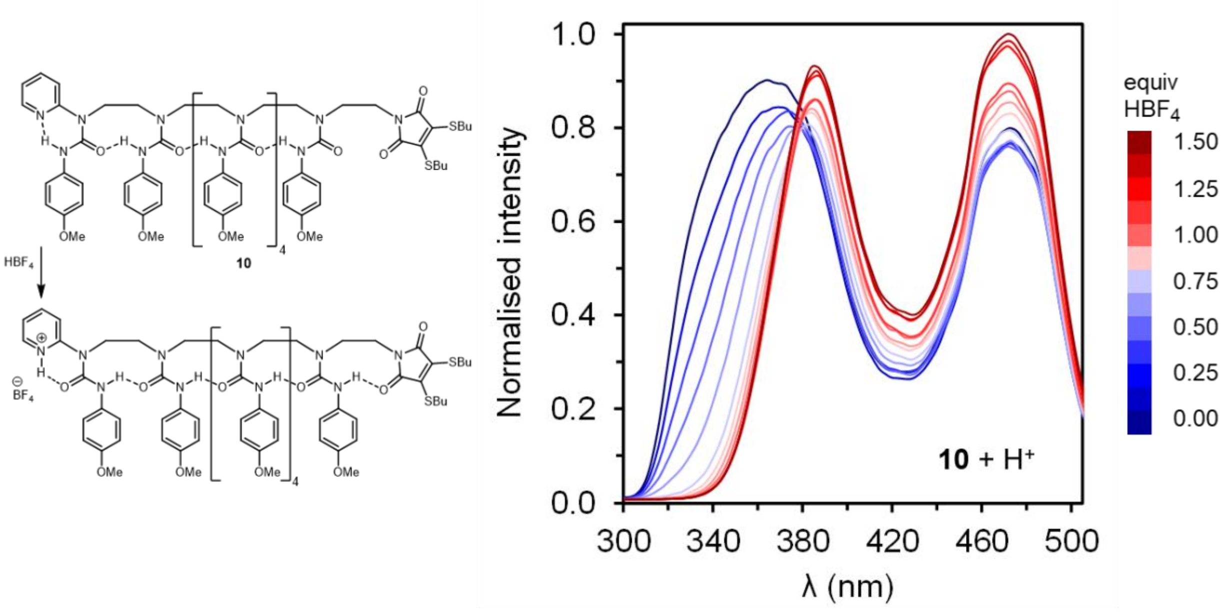

Figure S47 – Fluorescence titrations for compound 10.

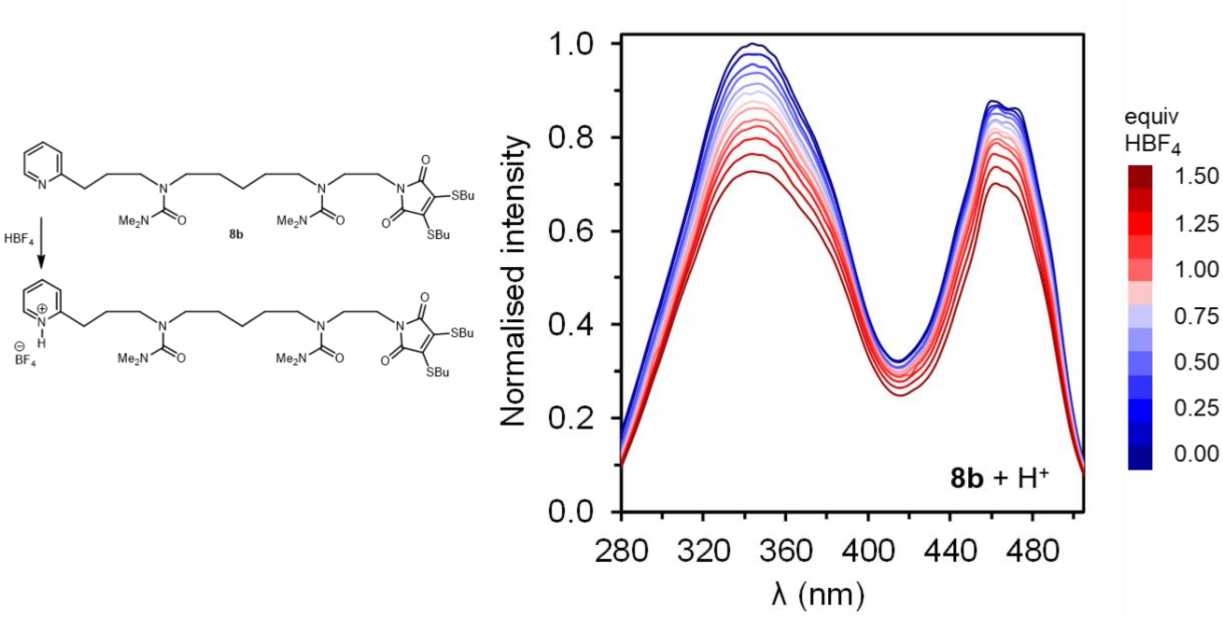

Figure S48 – Fluorescence titrations for compound 8b.

• After completion of the fluorescence titration of control compound **8b** with acid, evidence of pyridine N-protonation was provided by ^{1}H NMR spectroscopy:

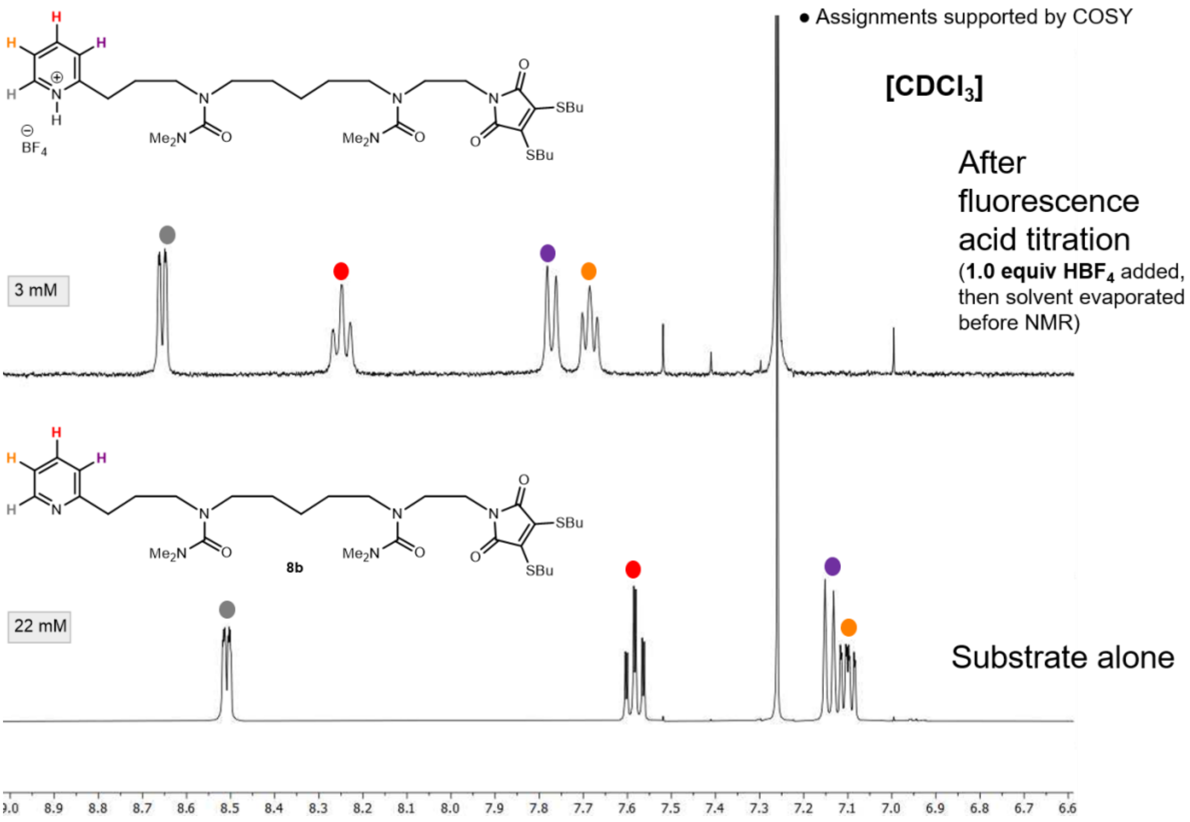

Figure S49 – NMR studies of 8b upon treatment with HBF_4.

• The plots below show the change in the fluorescence excitation wavelength maxima (relative to the initial wavelength maxima) for the S_0–S_2 transition band during the titrations with HBF_4. Only compounds **8a** and **10** containing a tethered pyridyl group and a linking chain of urea H-bonds to the fluorophore underwent significant λ_{max} changes (red shifting).

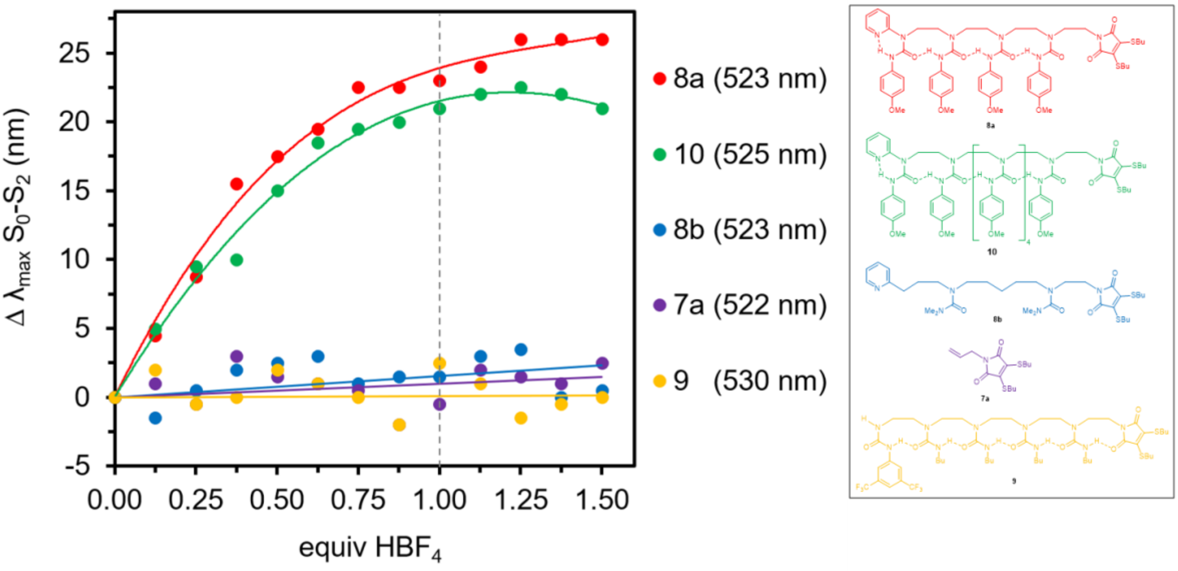

Figure S50 – Fluorescence excitation plots upon titration of HBF_4 into compounds 7-10.

Further Evidence of the Maleimide Fluorophore as a Hydrogen-Bond Acceptor

• Further evidence of the proposed fluorophore hydrogen-bonding interaction was provided by comparing the ^{1}H NMR spectra of **4b**·HBF_4 and **8a**·HBF_4 (obtained at constant concentration) in which sharp signals for all NH resonances were observed; these pyridinium salts differ only by the identity of their terminal group (benzyl versus *N*-ethylmaleimide). The chemical shift of the urea NH proximal to the maleimide in **8a**·HBF_4 was 7.3 ppm (CD_2Cl_2) which is significantly more downfield than that of the NH proximal to the benzyl group in **4b**·HBF_4 (6.3 ppm, CD_2Cl_2), indicative of the maleimide acting as a hydrogen-bond acceptor; all other pairs of analogous NH protons in **4b**·HBF_4 and 8**a**·HBF_4 had essentially identical chemical shifts (≤0.04 ppm differences).

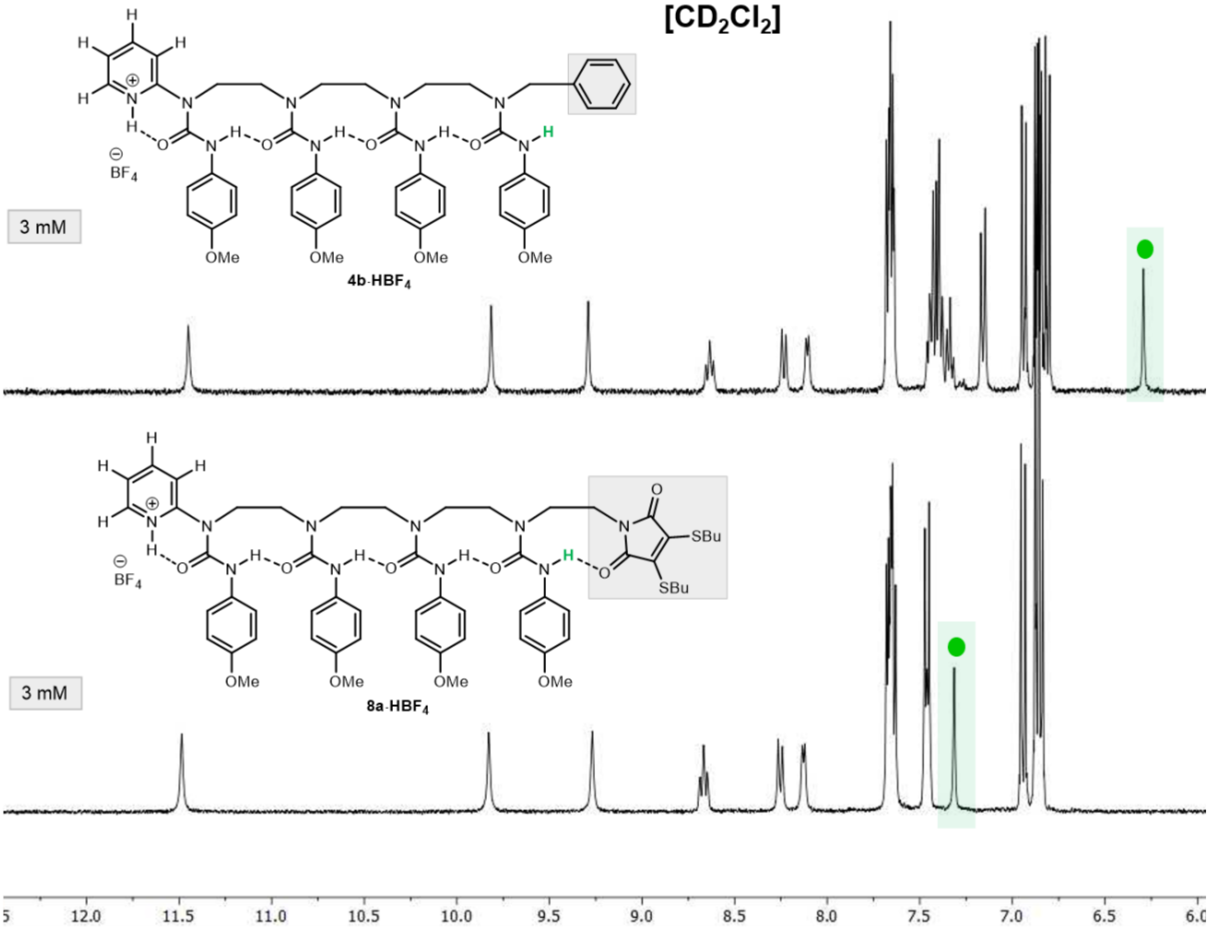

Figure S51 – Comparison of NMR spectra of 4b and 8a upon addition of HBF_4.

Fluorescence Titrations with Acetate

• ***General titration procedure with acetate:*** To a solution of the substrate (2.0 μmol) in lab grade dichloromethane (2.0 mL) in a glass cuvette at room temperature with magnetic stirring was sequentially added aliquots of a solution of $Bu_4N{\cdot}OAc$ (0.0125 M in CH_2Cl_2, 20 μL each time = 0.25 μmol, 0.125 equiv) until 1.5 equiv of acetate had been added (12 aliquots in total). A fluorescence spectrum was recorded after the addition of each aliquot.

• Fluorescence intensities shown in acetate titration spectra in the manuscript are not corrected for changes in concentration - the substrate concentrations decreased from 1.0 to 0.9 mM at the completion of the titrations.

• After completion of the fluorescence titration of foldamer **9** with acetate (see Fig 4f in manuscript), further evidence of acetate binding was provided by ^{1}H NMR and NOE spectroscopy:

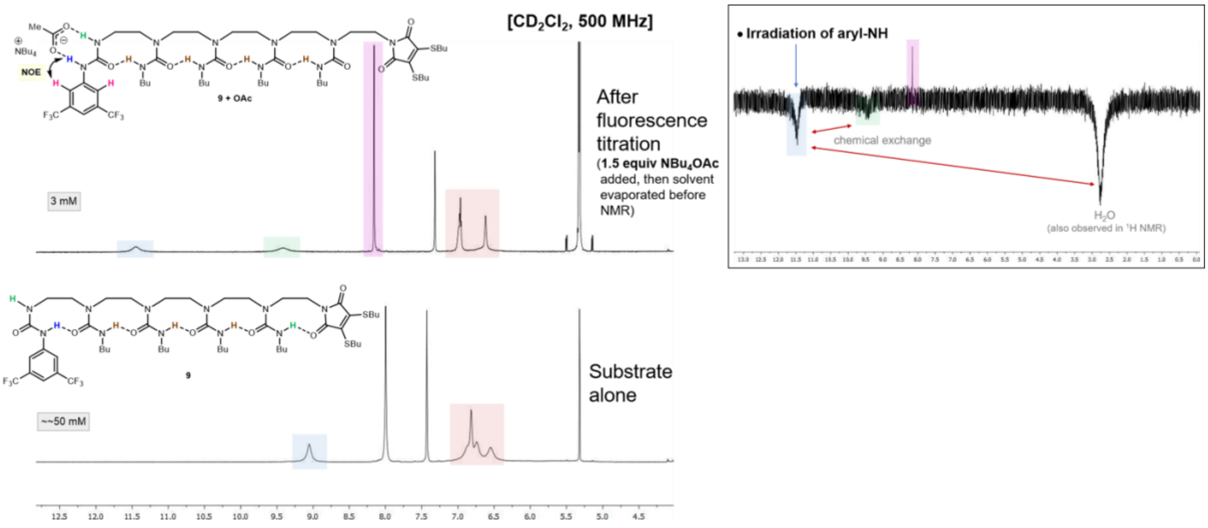

Figure S52 – NMR studies of 9 upon treatment with NBu_4OAc.

• ^{1}H NMR spectroscopy was also used to confirm a lack of binding interaction/s of **8a** with acetate (see Fig 4g in manuscript), as indicated by minimal/no changes in chemical shifts:

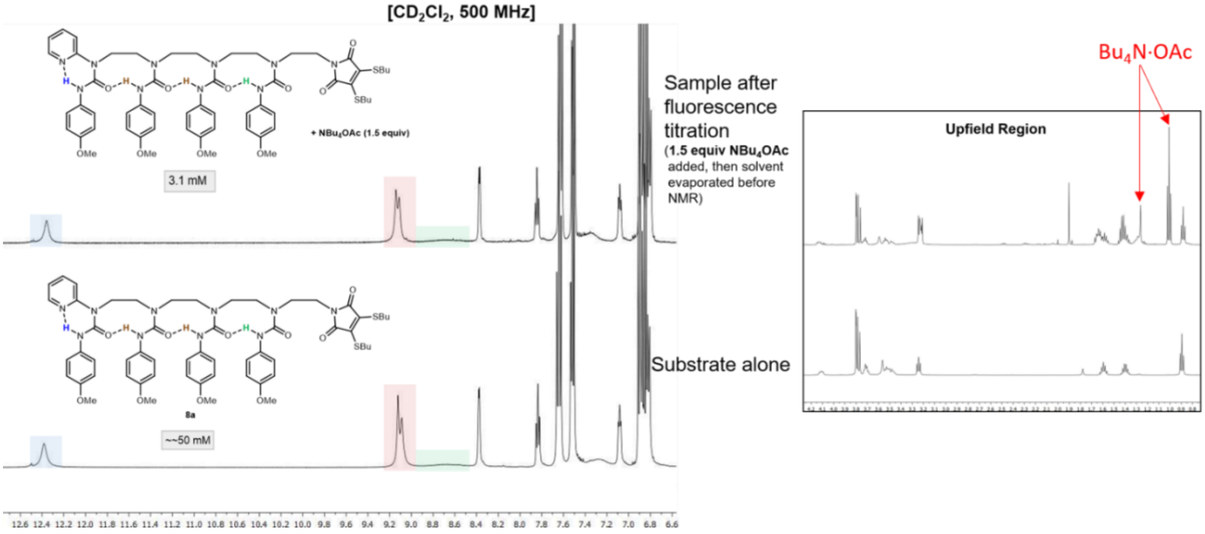

Figure S53 – NMR studies of 8a upon treatment with NBu_4OAc.

• The plots below show the change in the fluorescence excitation wavelength maxima (relative to the initial wavelength maxima) for the S_0–S_1 transition band during the titrations with $Bu_4N{\cdot}OAc$. Only compound **9** containing an anion binding site (electron-deficient disubstituted urea) and a linking chain of urea H-bonds to the fluorophore underwent a significant λ_{max} change with 1 equiv of acetate (blue shift).

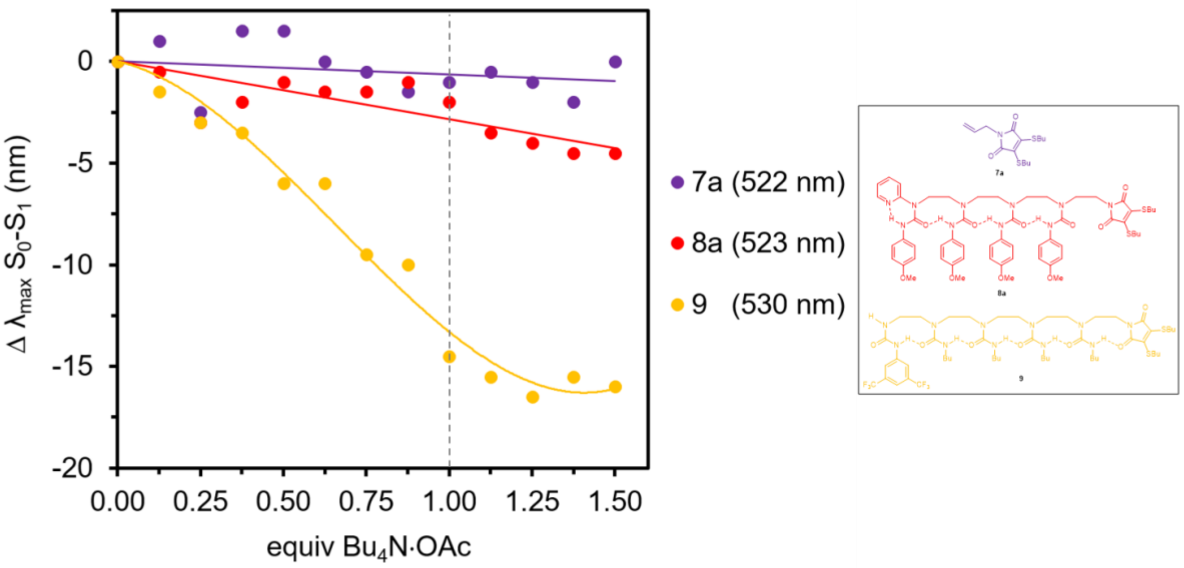

Figure S54 – Fluorescence excitation plots upon titration of NBu_4OAc into compounds 7-9.

Fluorescence Experiments with Acid/Base Switching

• ***General titration procedure for acid/base switching cycles:*** To a solution of the substrate (2.0 µmol) in lab grade dichloromethane (2.0 mL) in a glass cuvette at room temperature with magnetic stirring was sequentially added 20–50 µL aliquots (specified below) of a solution of $HBF_4{\cdot}Et_2O$ (0.10 M in CH_2Cl_2) and a solution of Et_3N (0.10 M in CH_2Cl_2). A fluorescence spectrum was recorded at each of the following titration points:

Table S18 – Acid and base cycles for remote fluorescence switching of 8a.

Titration Point	Additive	Total equiv of HBF_4 added	Total equiv of Et_3N added
'Neutral'	-	0.0	0.0
'Acidic'	20 µL of HBF_4 solution	1.0	0.0
'Basic'	30 µL of Et_3N solution	1.0	1.5
'Acidic'	40 µL of HBF_4 solution	3.0	1.5
'Basic'	50 µL of Et_3N solution	3.0	4.0
'Acidic'	50 µL of HBF_4 solution	5.5	4.0
'Basic'	50 µL of Et_3N solution	5.5	6.5
'Acidic'	50 µL of HBF_4 solution	8.0	6.5
'Basic'	50 µL of Et_3N solution	8.0	9.0

• Comments regarding the preparation of stock solutions of $HBF_4{\cdot}Et_2O$ are given previously in the 'fluorescence titrations with acid' section.

• Fluorescence intensities shown in acid/base switching titration spectra in the manuscript and below are not corrected for changes in concentration - the substrate concentrations decreased from 1.0 to 0.85 mM at the completion of the titrations.

• <u>Additional results with acid/base switching cycles</u>: Reversible pH-controlled switching between the two polarity states was also demonstrated with the shorter foldamer **8a**. Starting in the absence of additives, followed by the repeated sequential addition of acid (HBF_4) and base (Et_3N), four cycles of the fluorophore excitation response between the blue-shifted, non-hydrogen bonded 'neutral' state and the red-shifted, hydrogen bonded 'protonated' state were successfully induced.

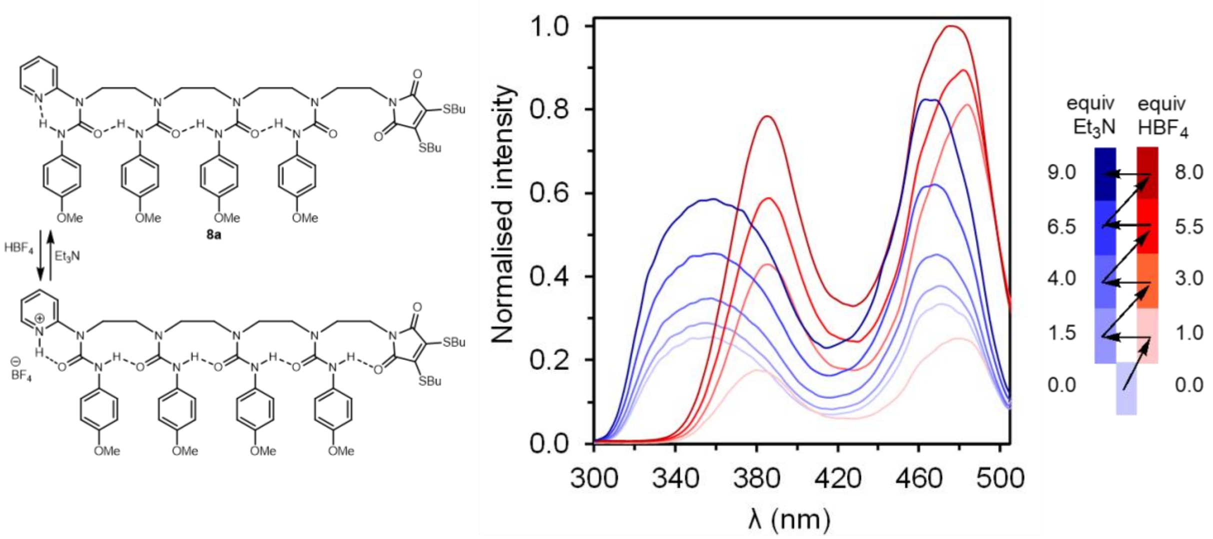

Figure S55 – Acid/base switching by sequential addition of HBF_4 and Et_3N to 8a.

NMR Characterisation

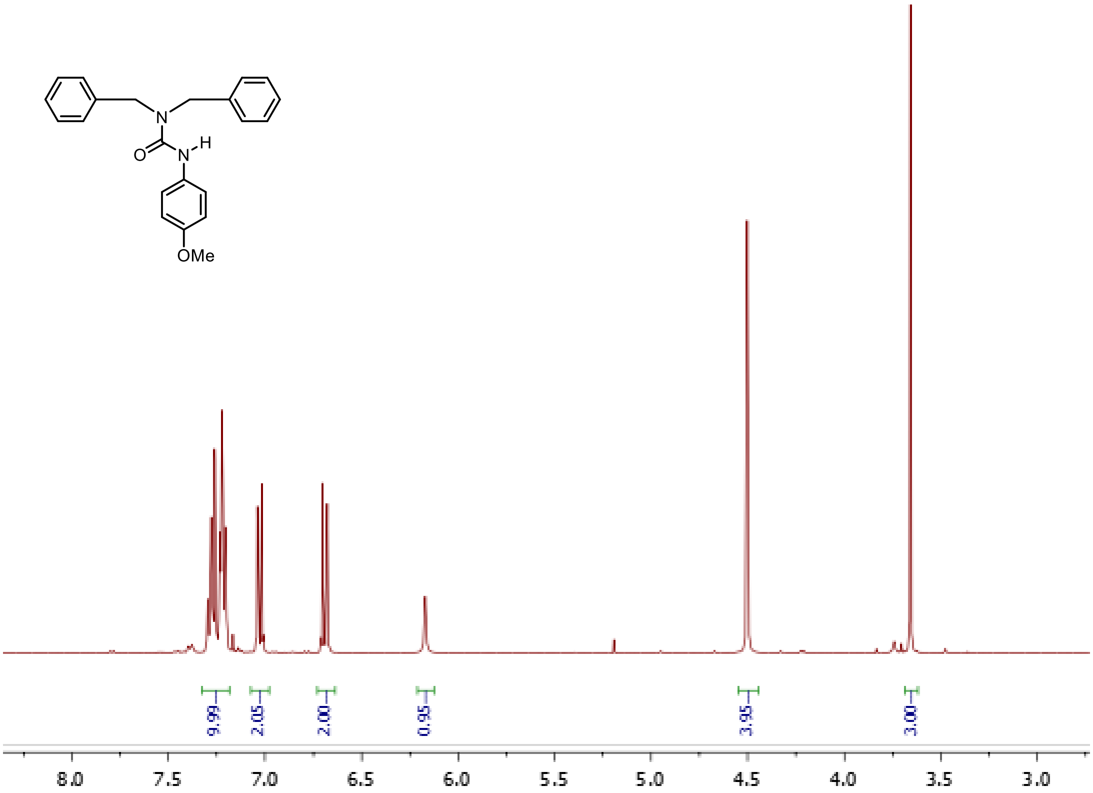

Figure S56 – ^{1}H NMR spectrum of 1 (400 MHz, $CDCl_3$).

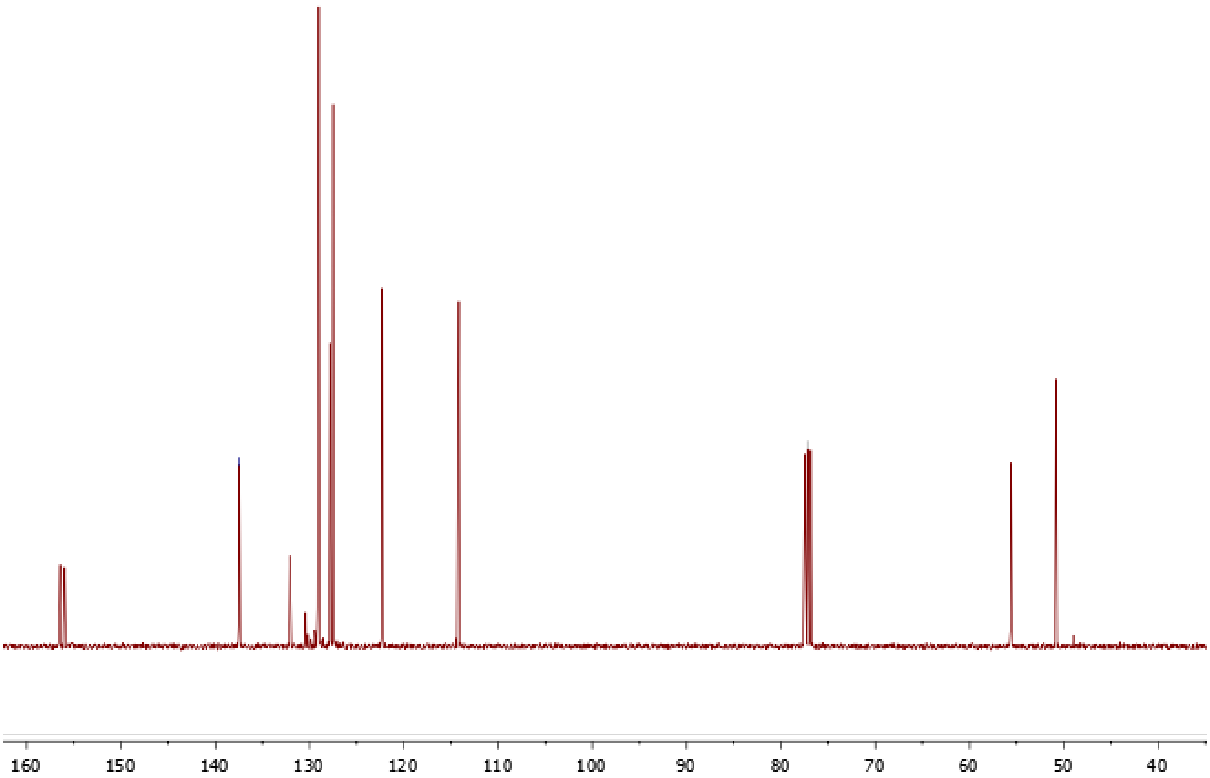

Figure S57 – ^{13}C NMR spectrum of 1 (101 MHz, $CDCl_3$).

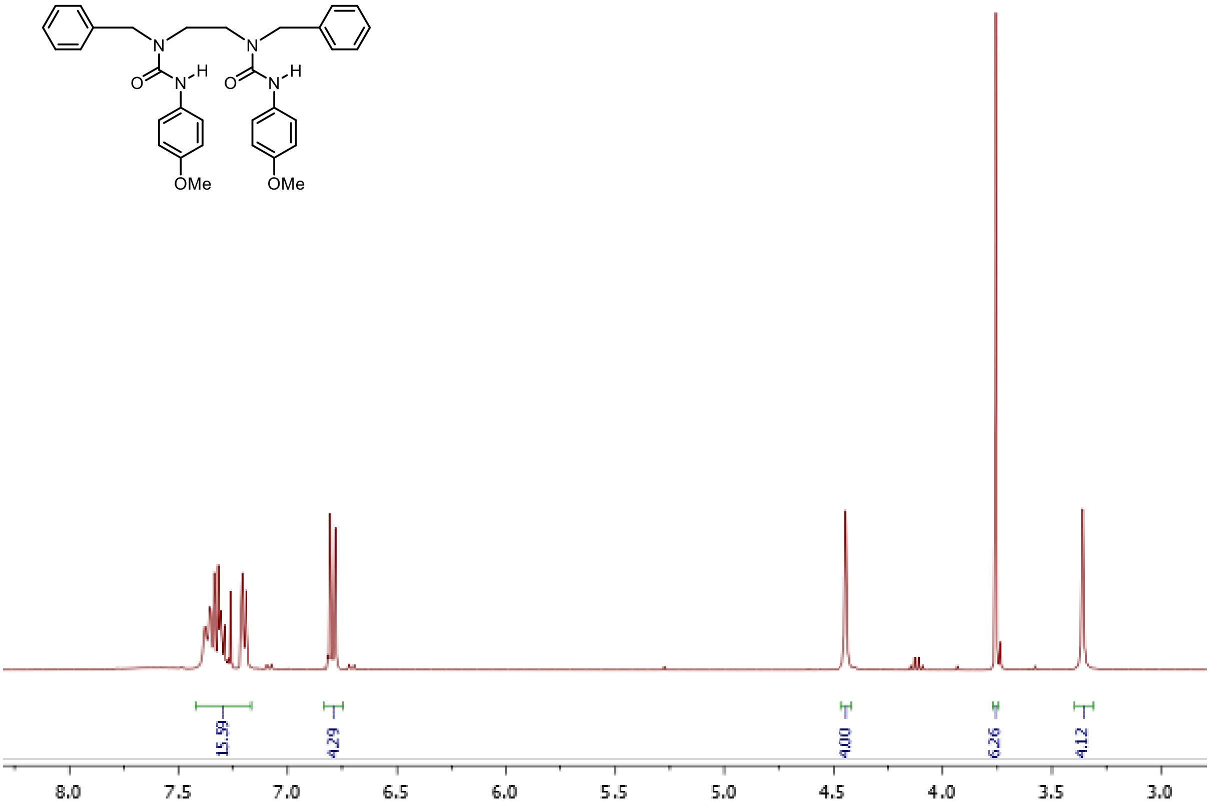

Figure S58 – ^{1}H NMR spectrum of 2a (400 MHz, $CDCl_3$).

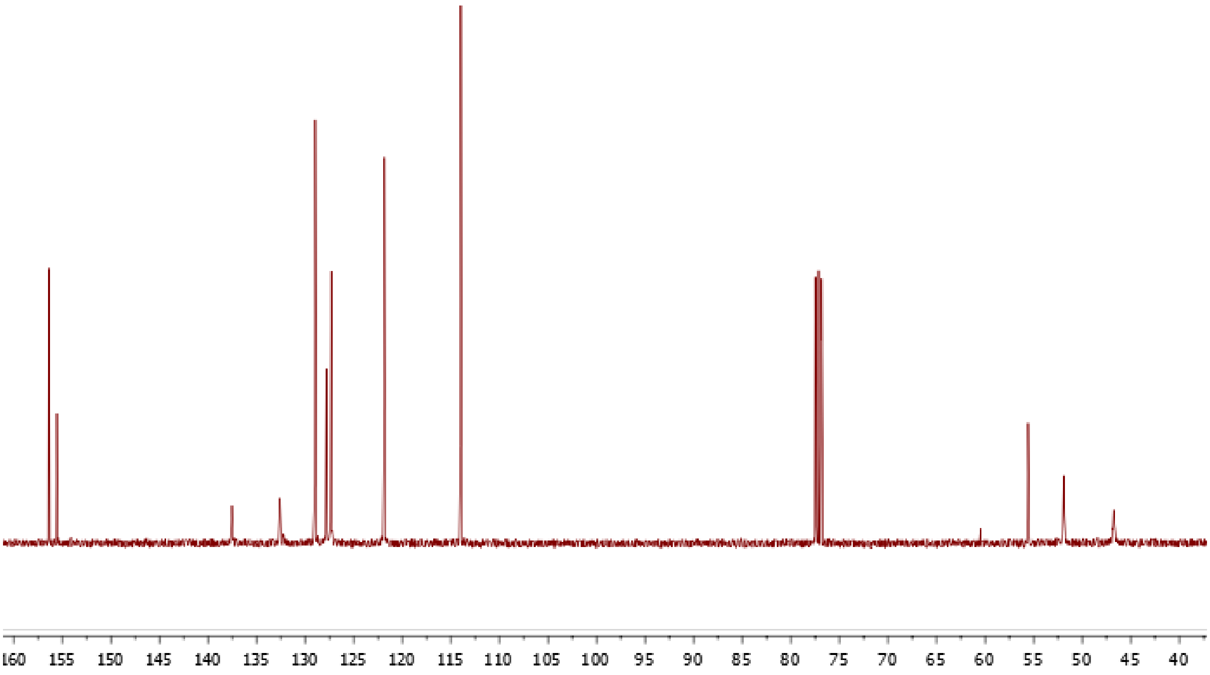

Figure S59 – ^{13}C NMR spectrum of 2a (101 MHz, $CDCl_3$).

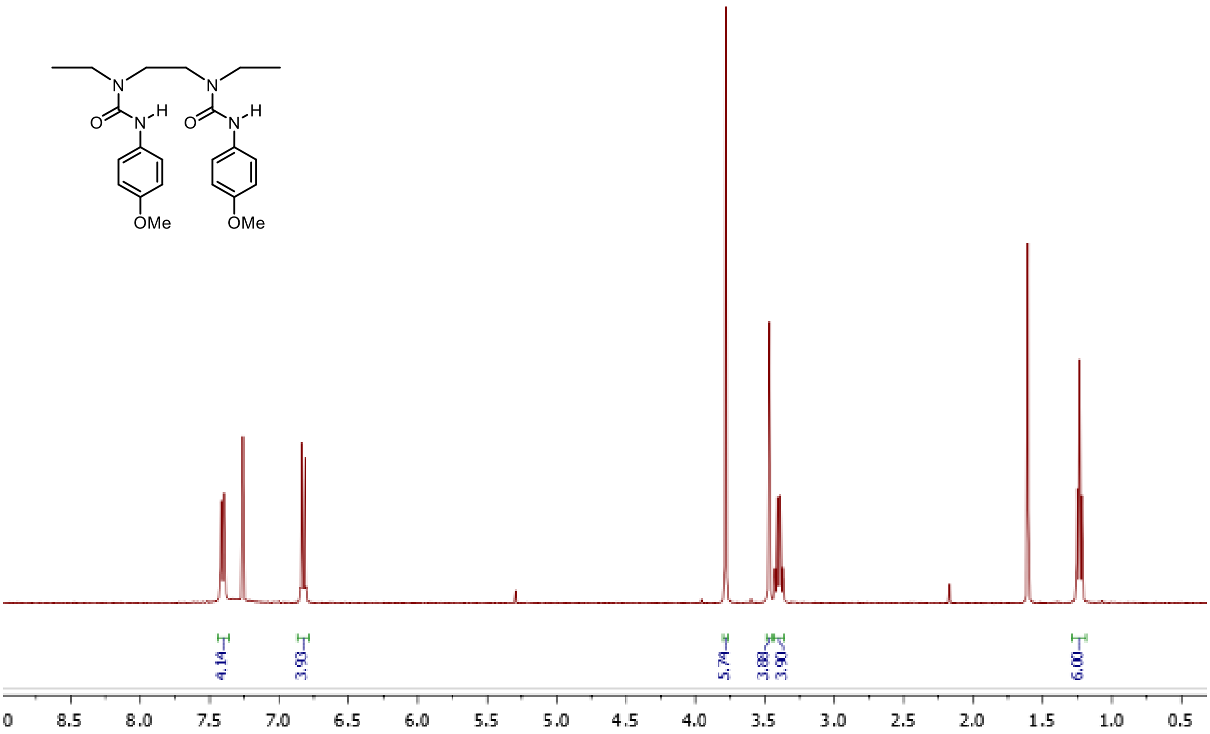

Figure S60 – ^{1}H NMR spectrum of 2b (400 MHz, $CDCl_3$).

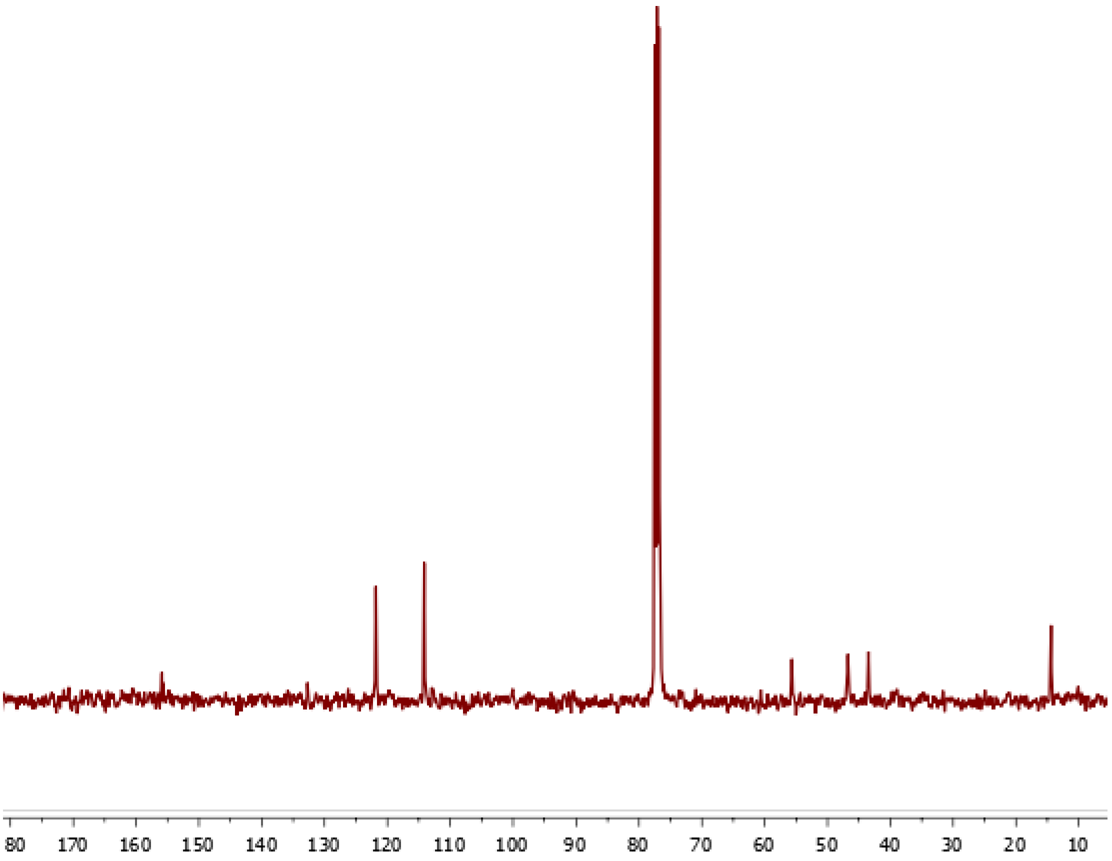

Figure S61 – ^{13}C NMR spectrum of 2b (101 MHz, $CDCl_3$).

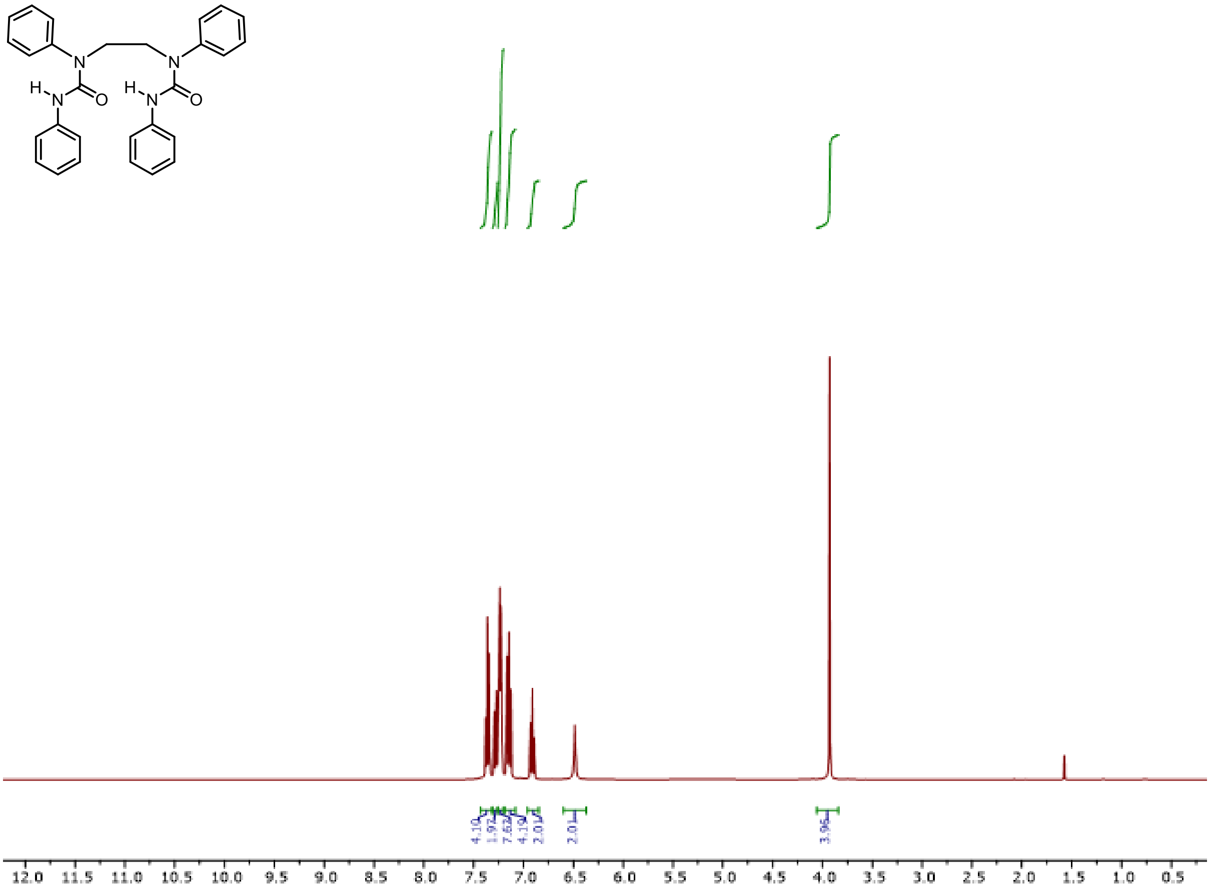

Figure S62 – ^{1}H NMR spectrum of 2c (400 MHz, $CDCl_3$).

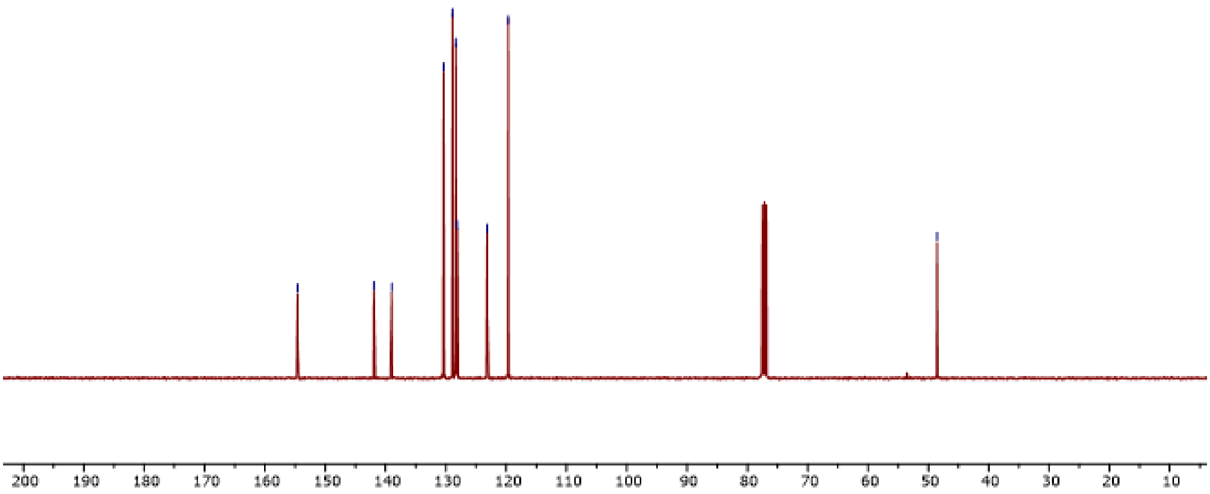

Figure S63 – ^{13}C NMR spectrum of 2c (101 MHz, $CDCl_3$).

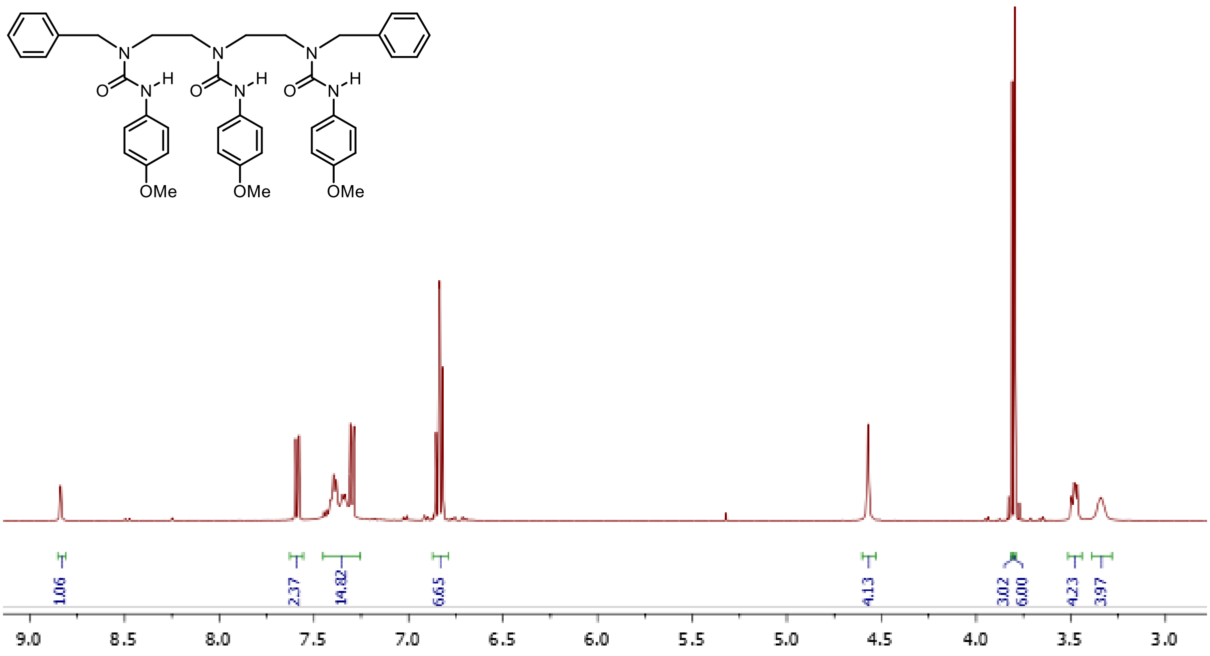

Figure S64 – ^{1}H NMR spectrum of 3a (400 MHz, $CDCl_3$).

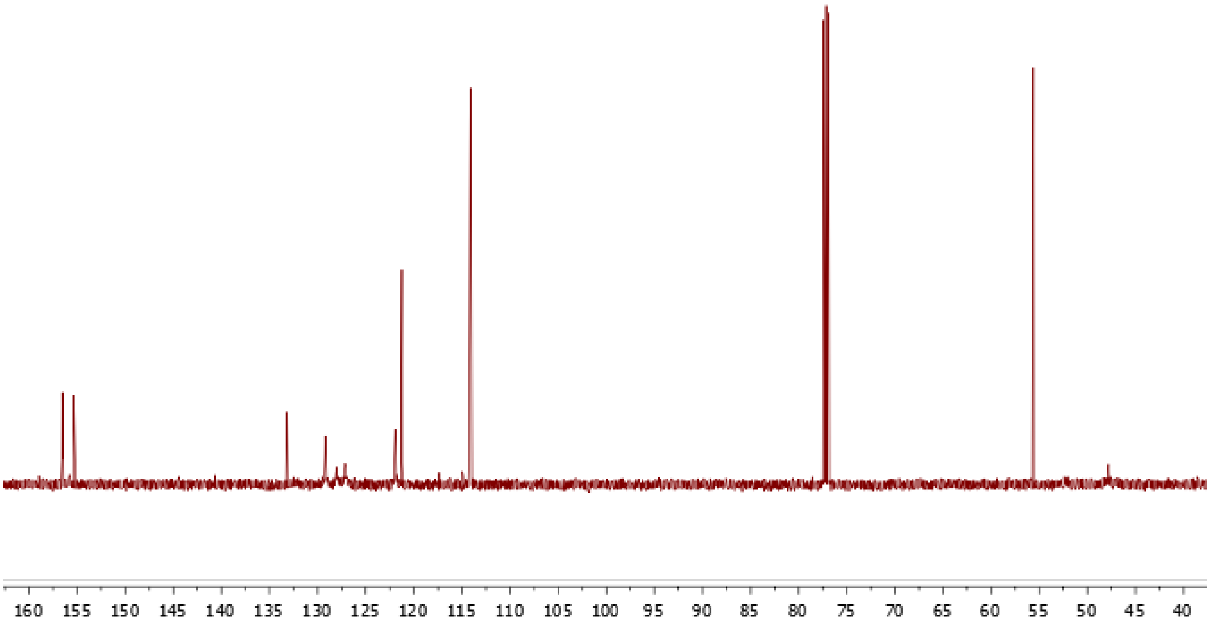

Figure S65 – ^{13}C NMR spectrum of 3a (101 MHz, $CDCl_3$).

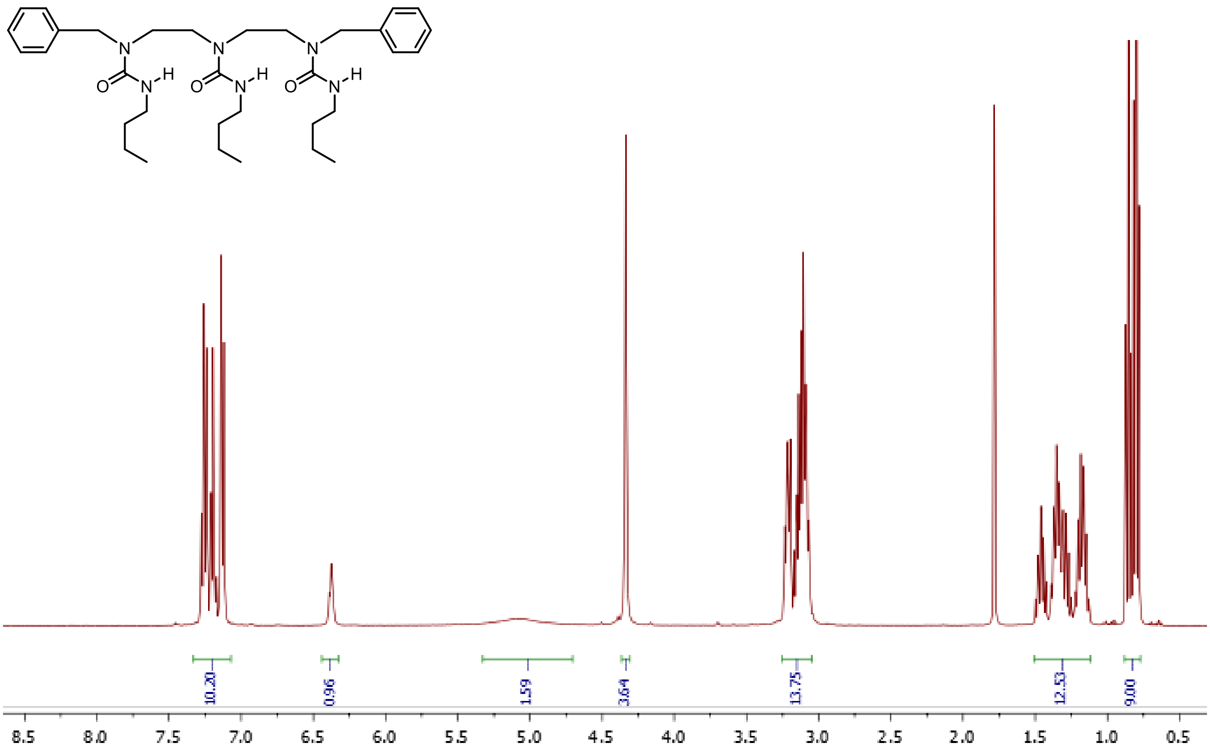

Figure S66 – ^{1}H NMR spectrum of 3b (400 MHz, $CDCl_3$).

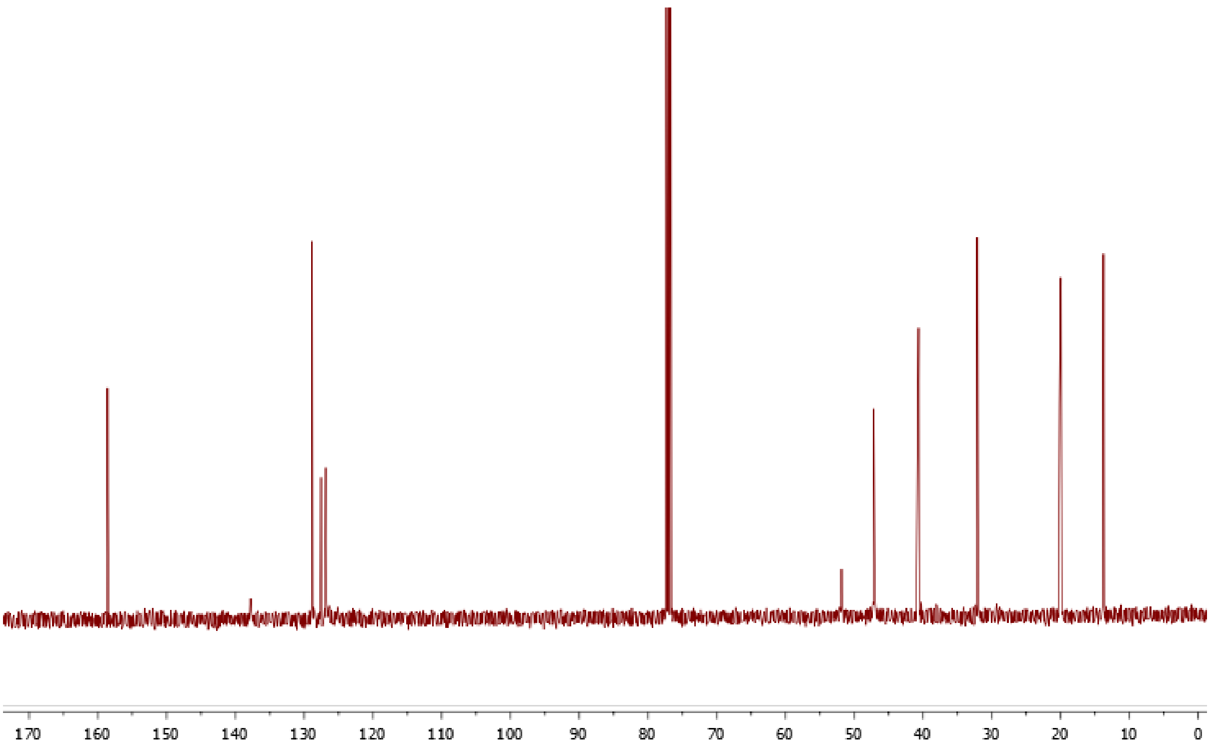

Figure S67 – ^{13}C NMR spectrum of 3b (101 MHz, $CDCl_3$).

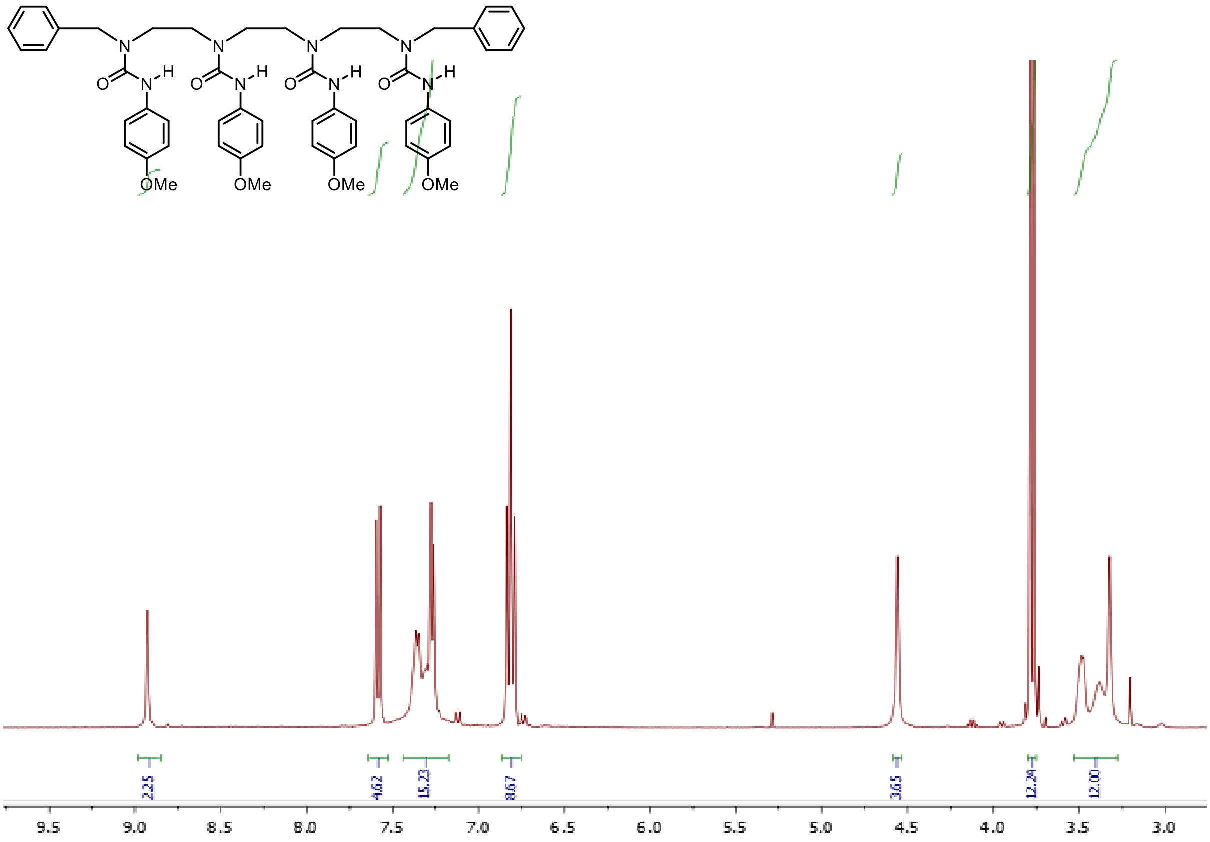

Figure S68 – ^{1}H NMR spectrum of 4a (400 MHz, $CDCl_3$).

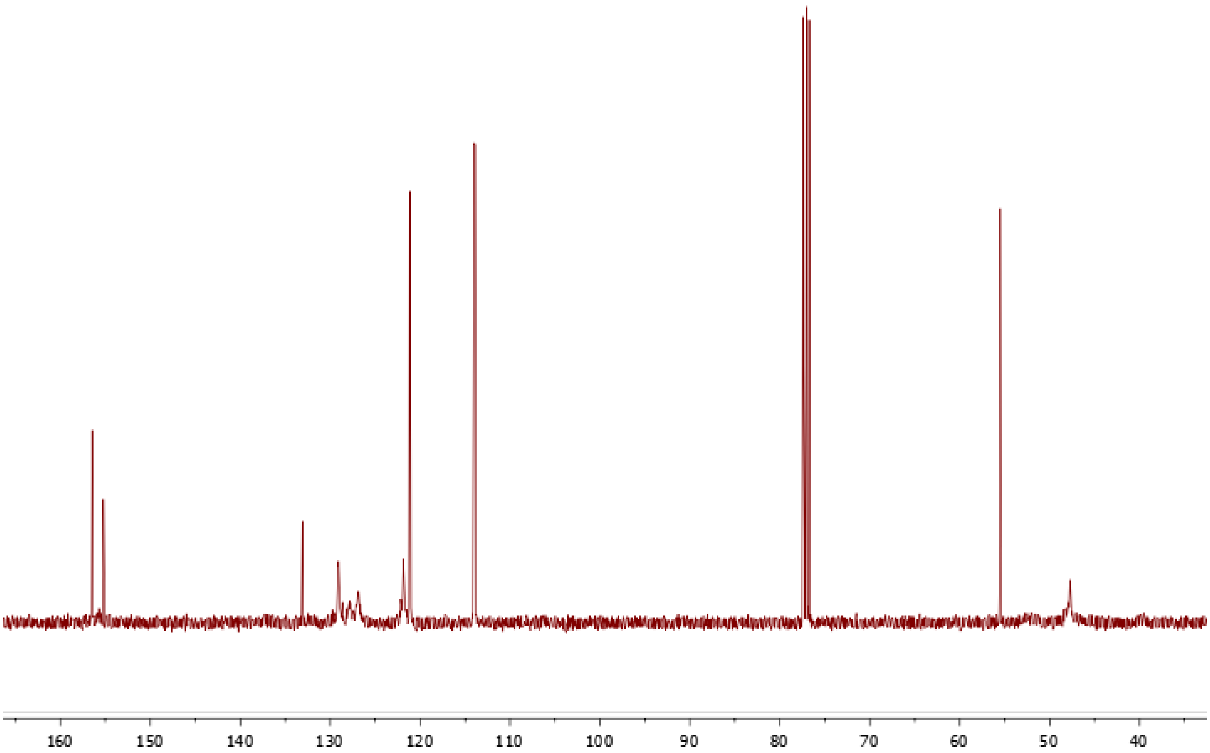

Figure S69 – ^{13}C NMR spectrum of 4a (101 MHz, $CDCl_3$).

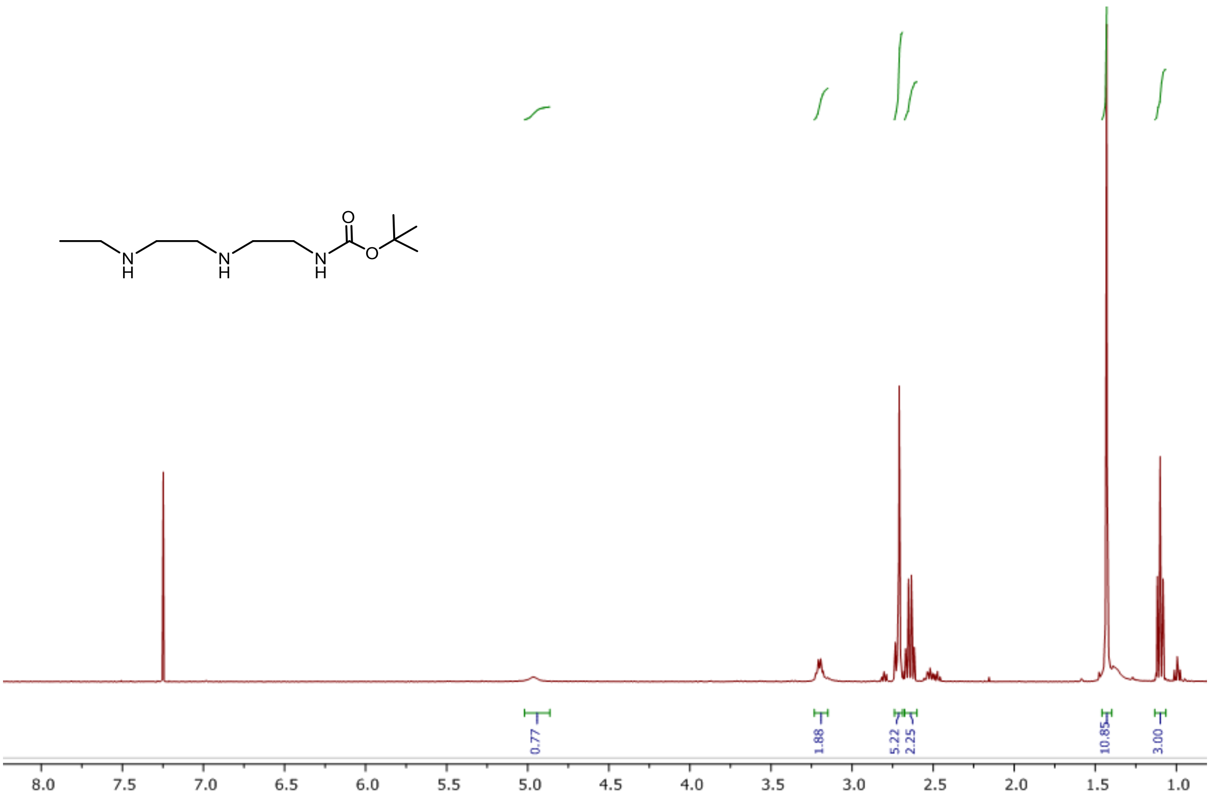

Figure S70 – ^{1}H NMR spectrum of 3c-1 (400 MHz, $CDCl_3$).

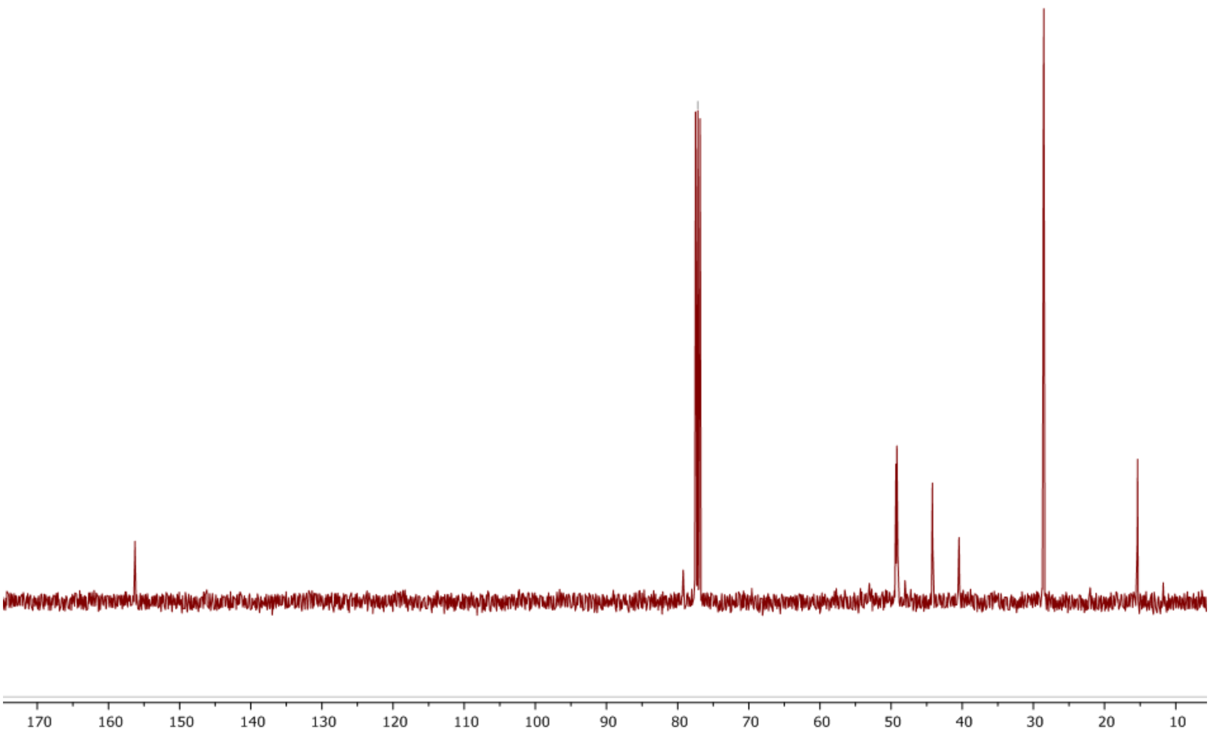

Figure S71 – ^{13}C NMR spectrum of 3c-1 (101 MHz, $CDCl_3$).

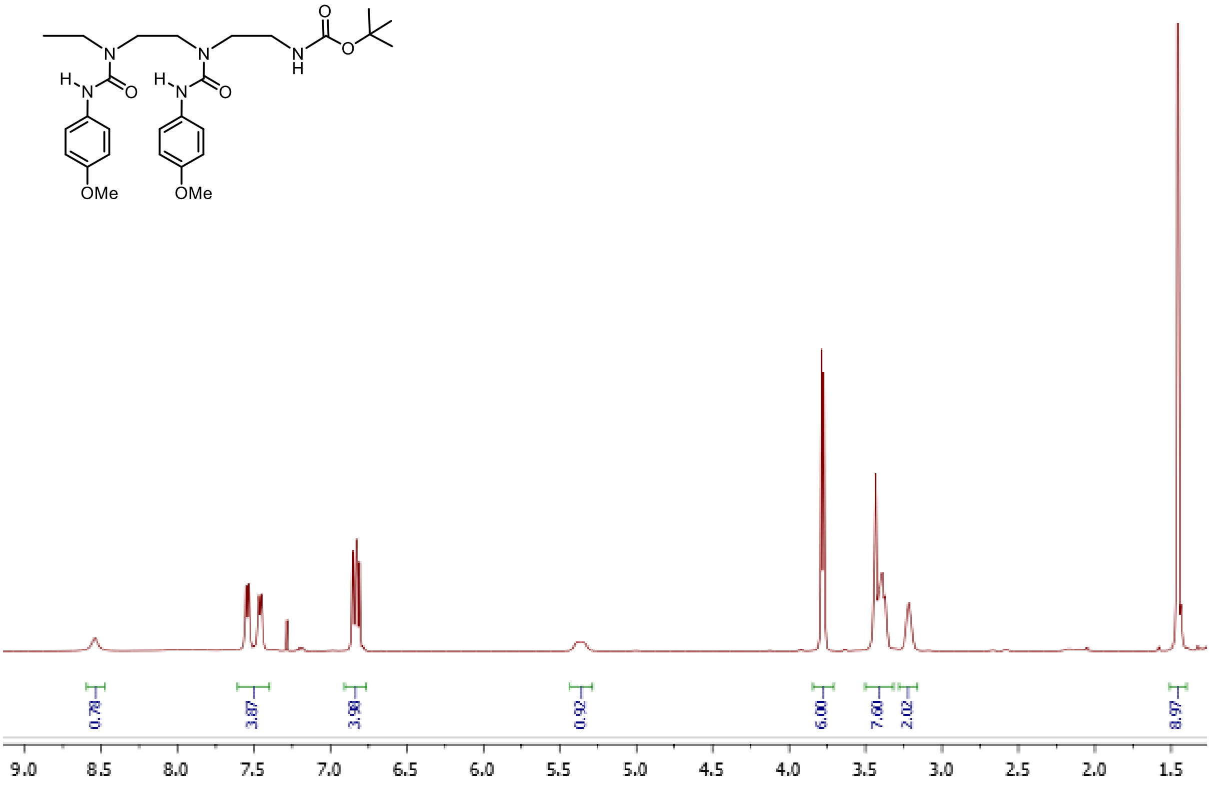

Figure S72 – ^{1}H NMR spectrum of 3c-2 (500 MHz, CDCl3).

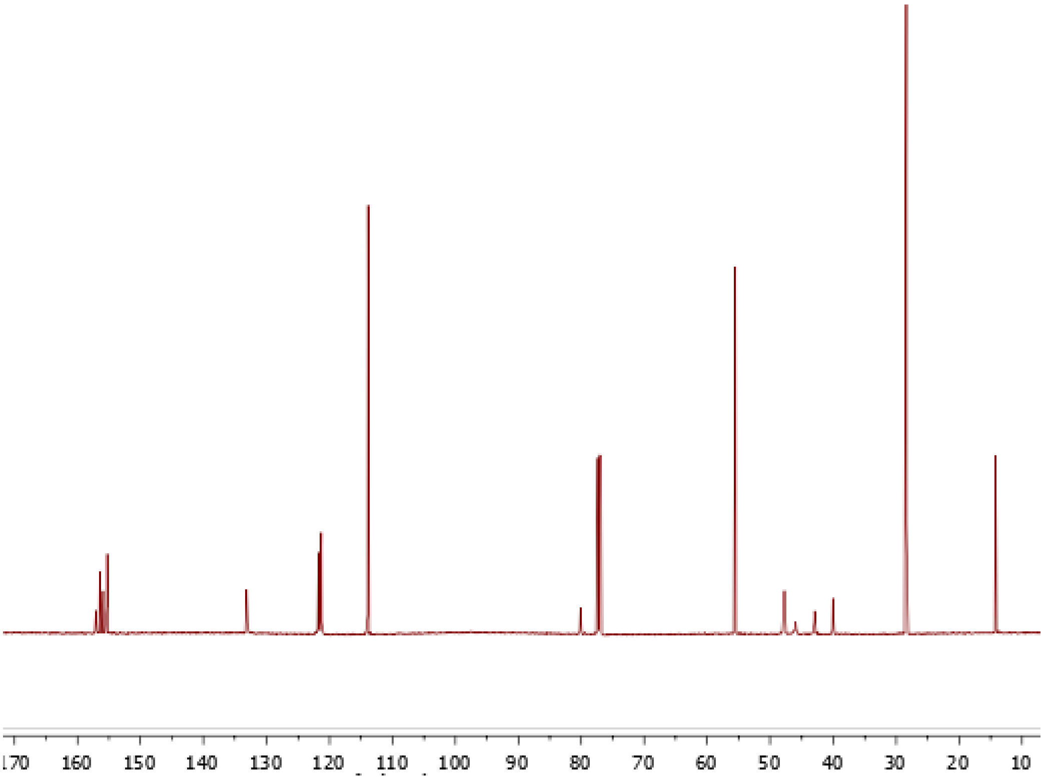

Figure S73 – ^{13}C NMR spectrum of 3c-2 (126 MHz, CDCl3).

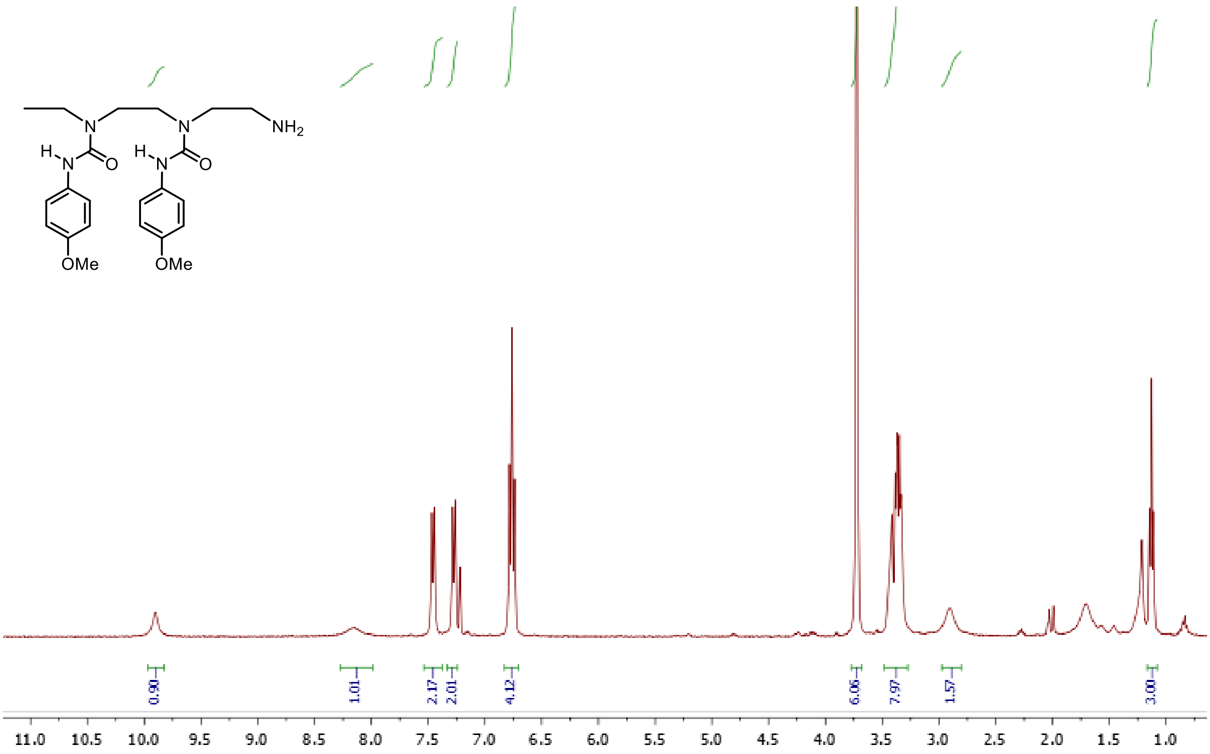

Figure S74 – ^{1}H NMR spectrum of 3c-3 (400 MHz, CDCl$_3$).

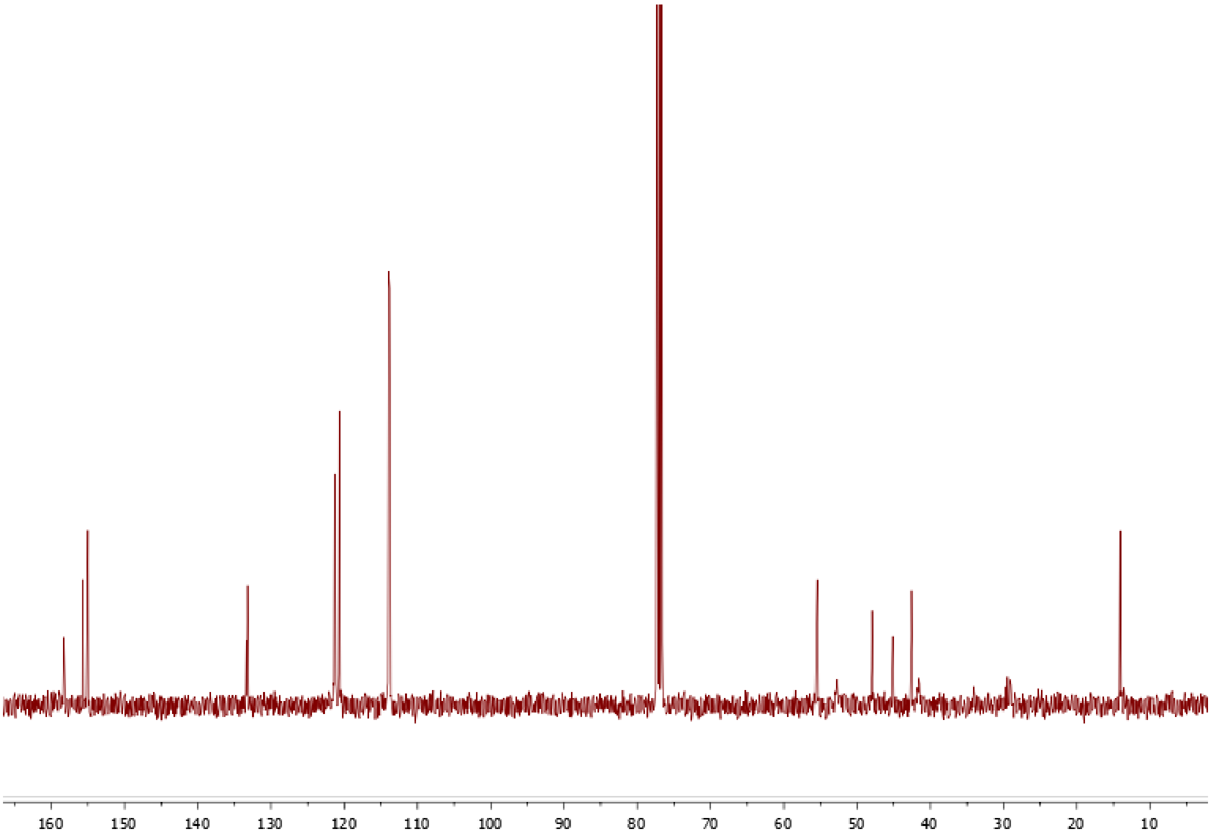

Figure S75 – ^{13}C NMR spectrum of 3c-3 (101 MHz, CDCl$_3$).

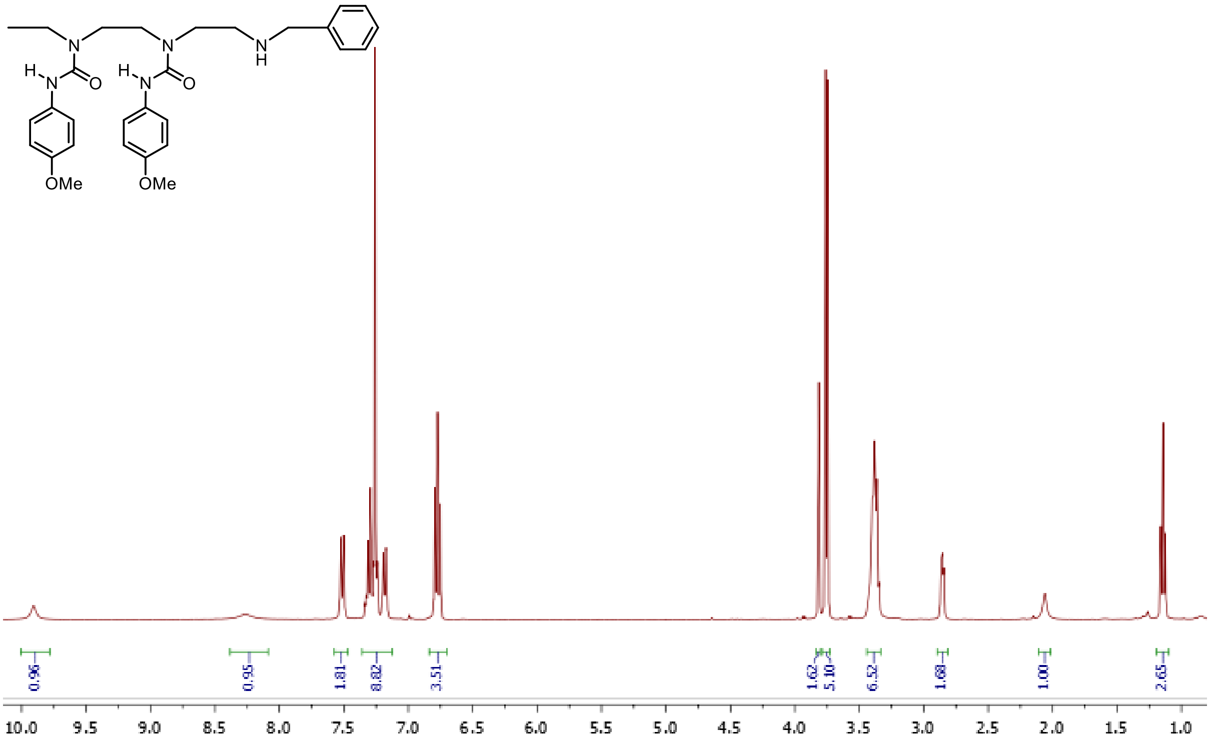

Figure S76 – ^{1}H NMR spectrum of 3c-4 (400 MHz, $CDCl_3$).

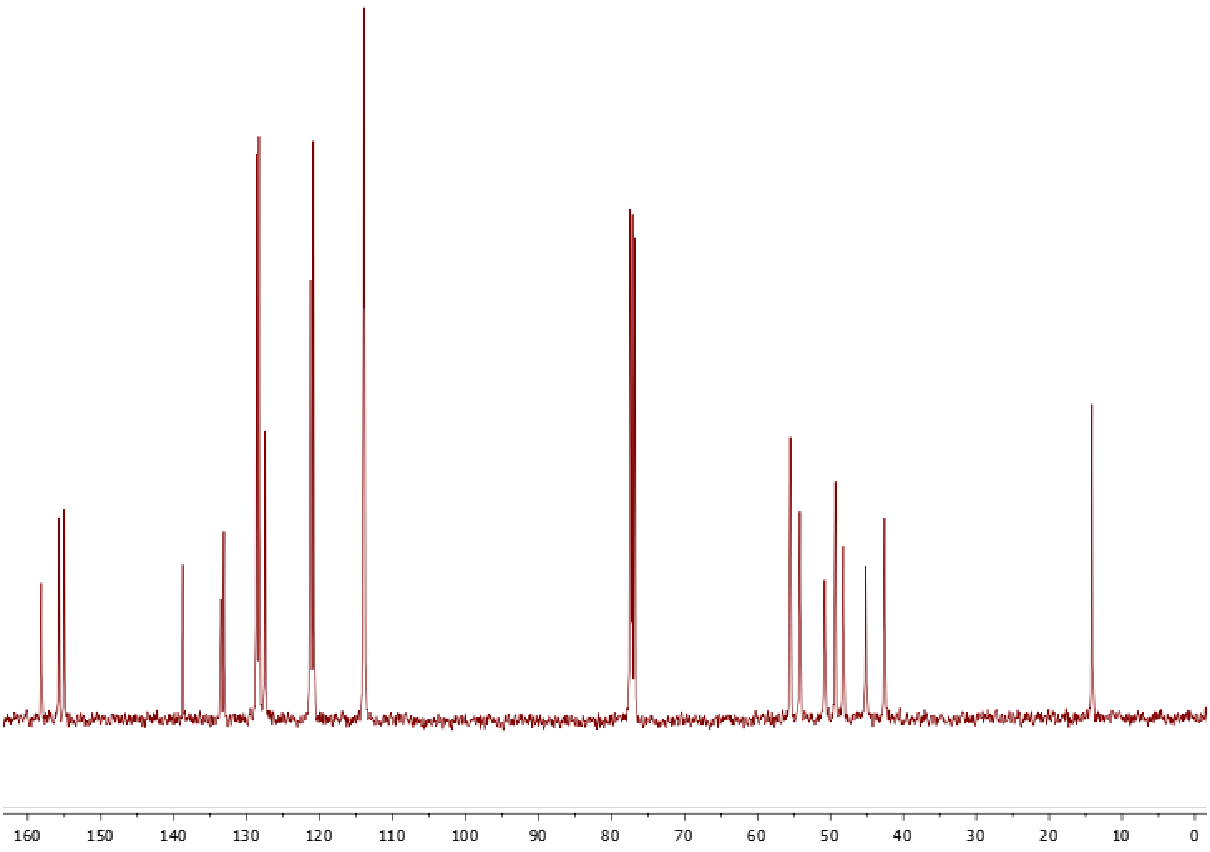

Figure S77 – ^{13}C NMR spectrum of 3c-4 (101 MHz, $CDCl_3$).

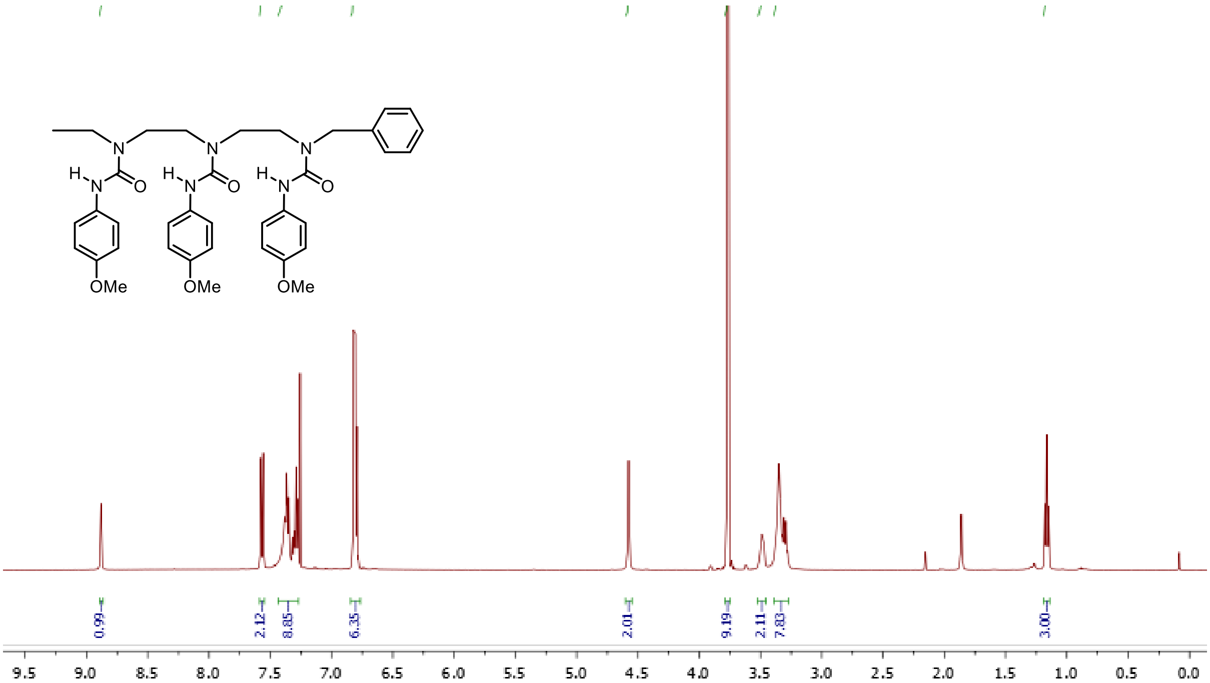

Figure S78 – ^{1}H NMR spectrum of 3c (500 MHz, $CDCl_3$).

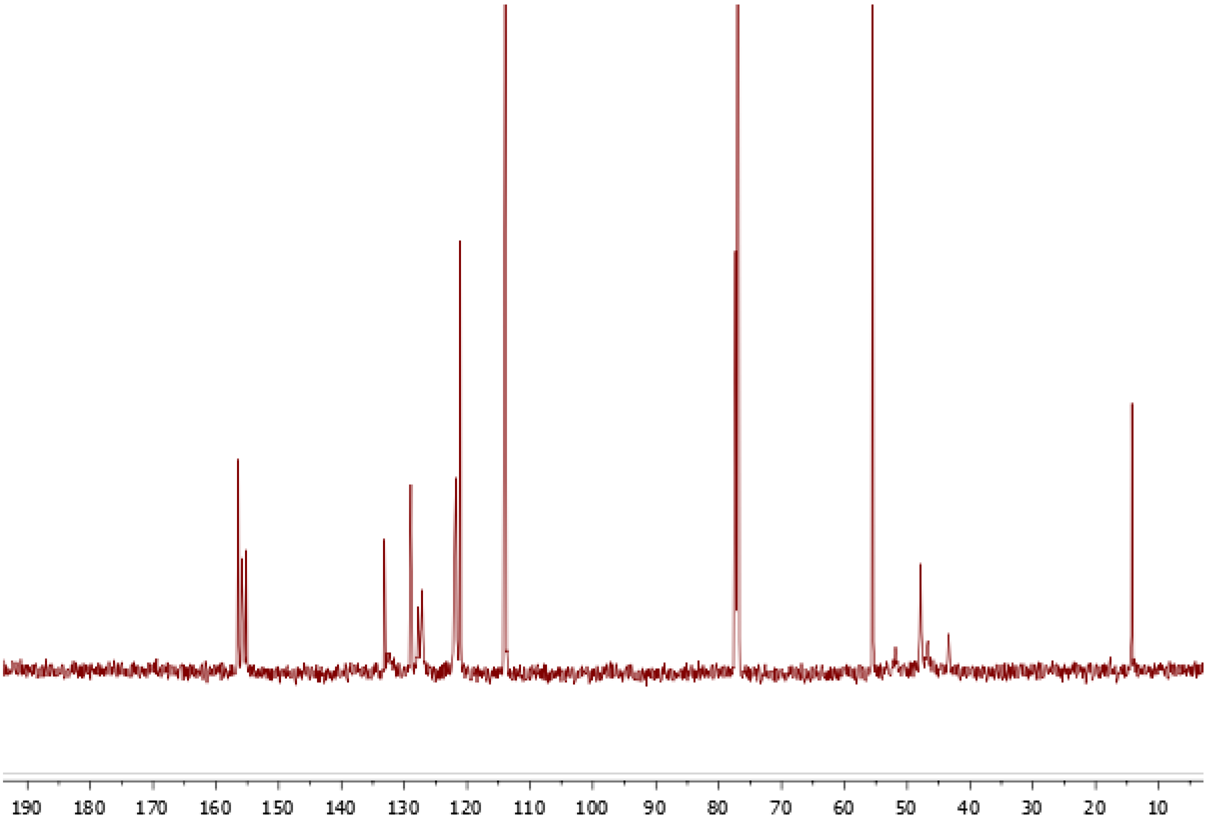

Figure S79 – ^{13}C NMR spectrum of 3c (126 MHz, $CDCl_3$).

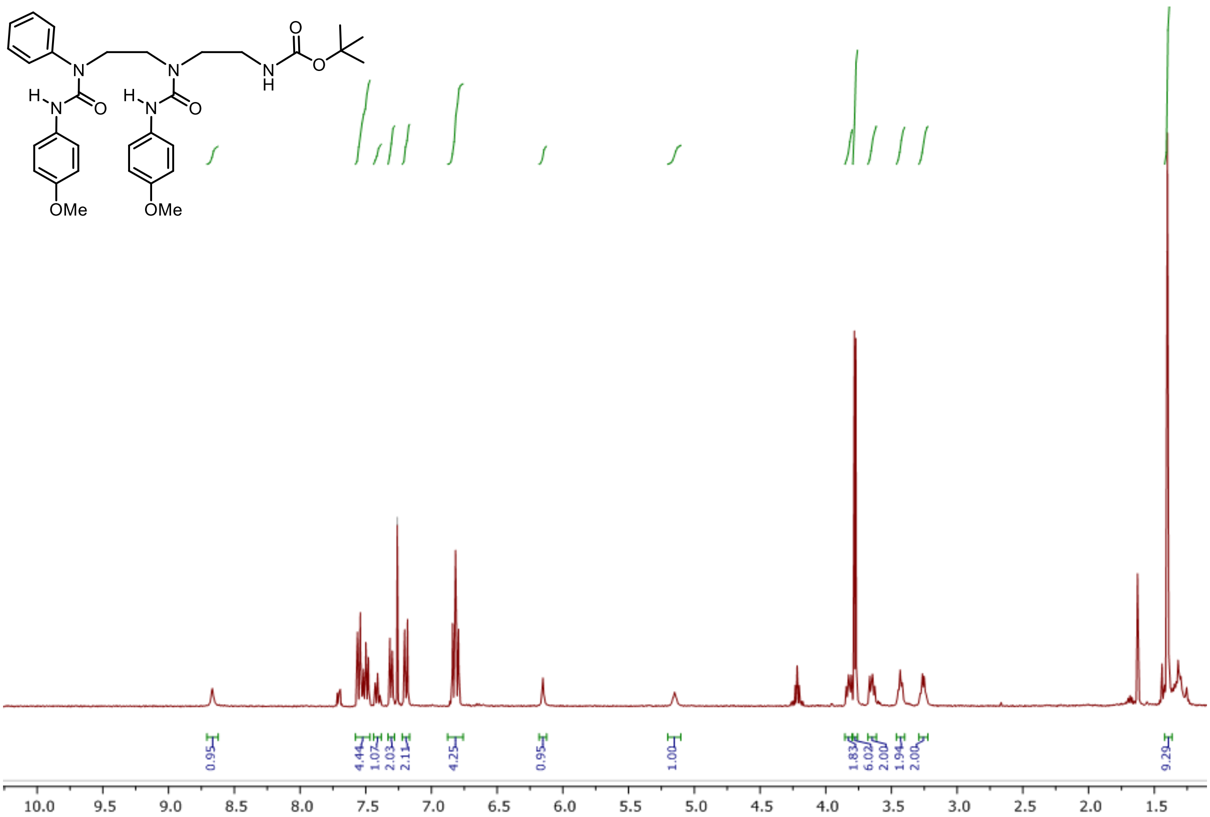

Figure S80 – ^{1}H NMR spectrum of 3d-2 (400 MHz, $CDCl_3$).

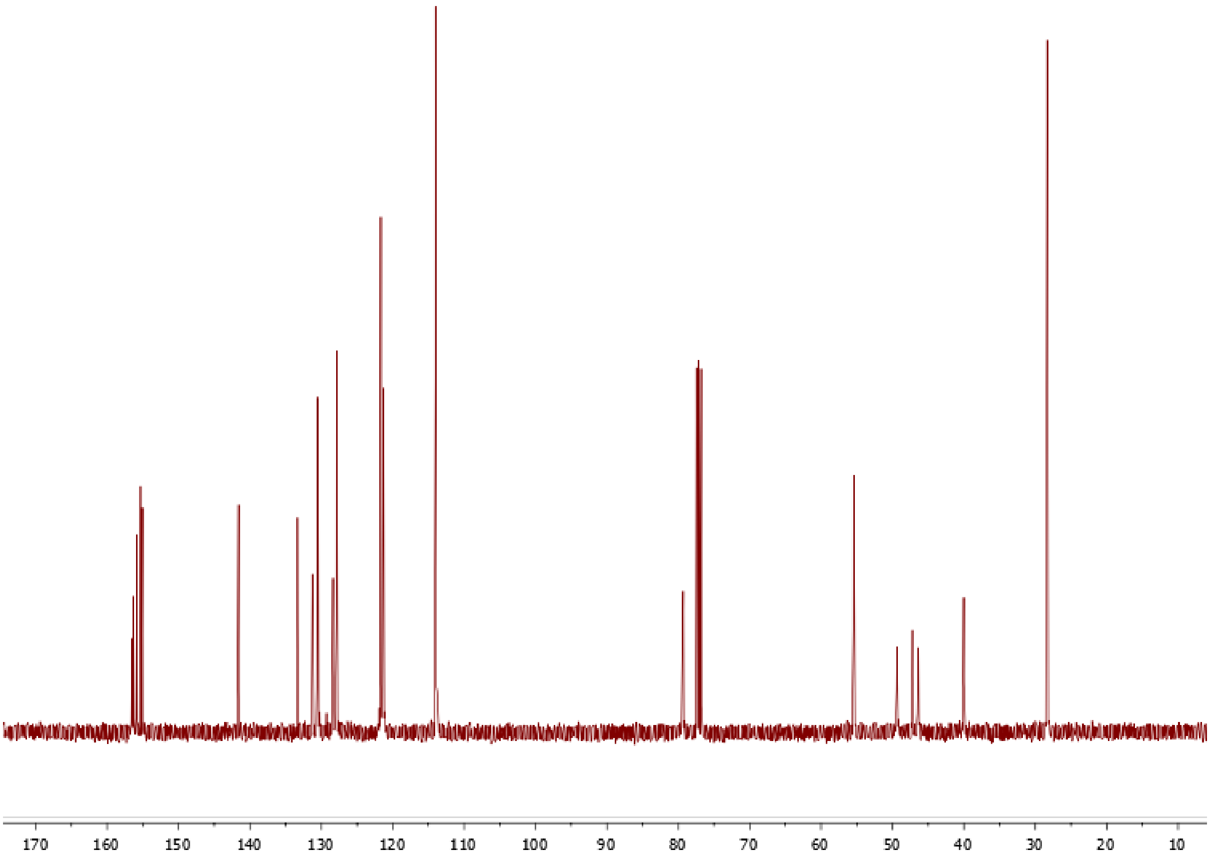

Figure S81 – ^{13}C NMR spectrum of 3d-2 (101 MHz, $CDCl_3$).

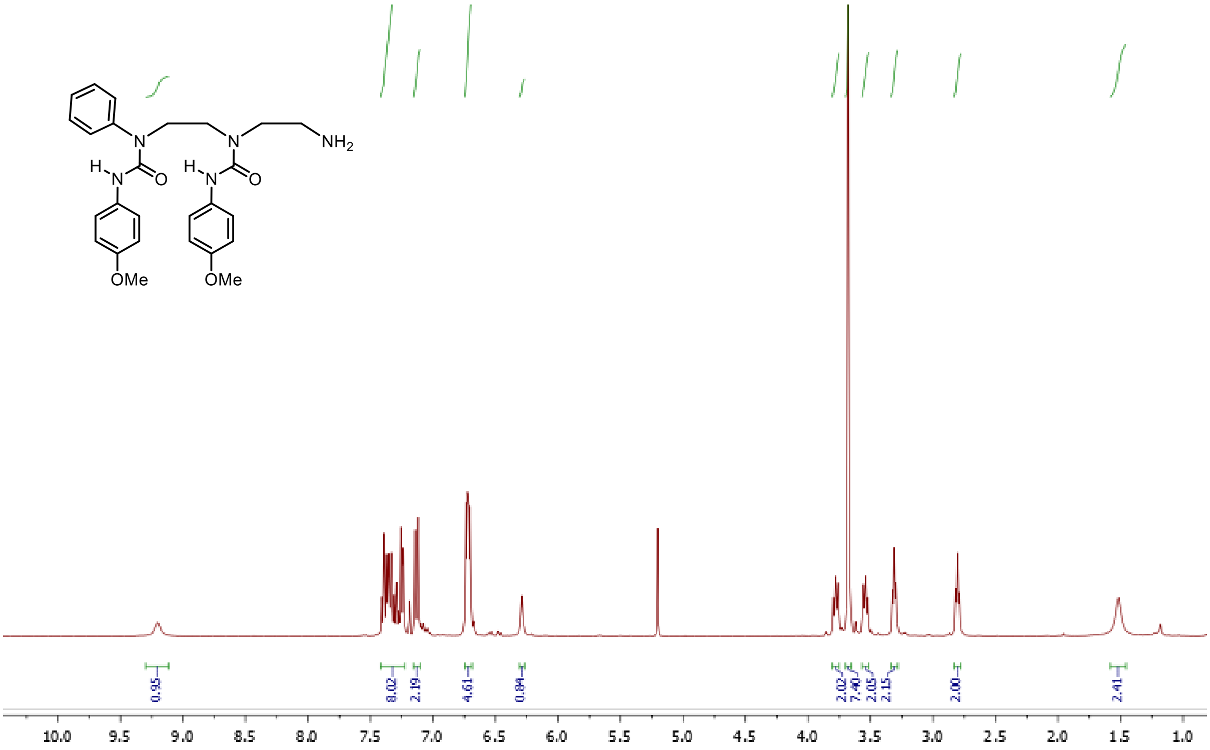

Figure S82 – ^{1}H NMR spectrum of 3d-3 (400 MHz, $CDCl_3$).

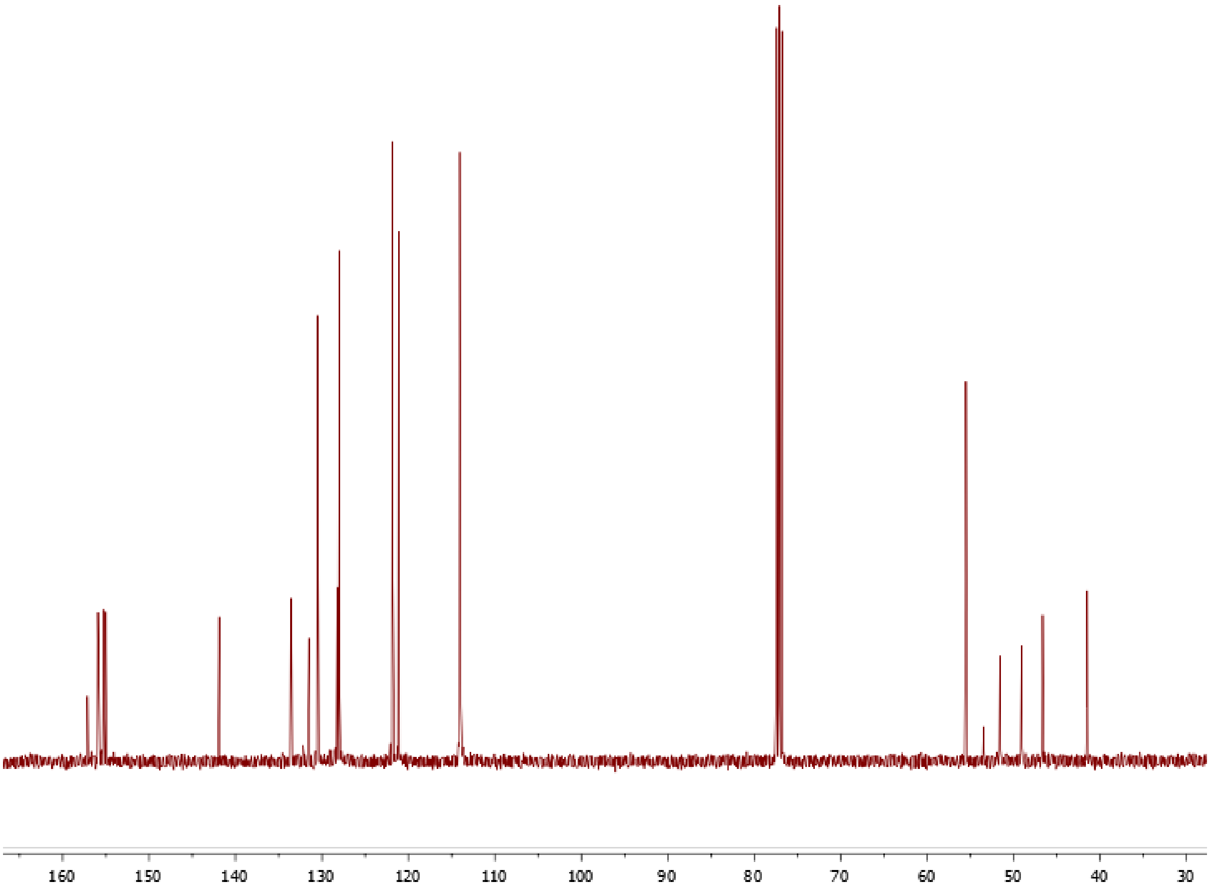

Figure S83 – ^{13}C NMR spectrum of 3d-3 (101 MHz, $CDCl_3$).

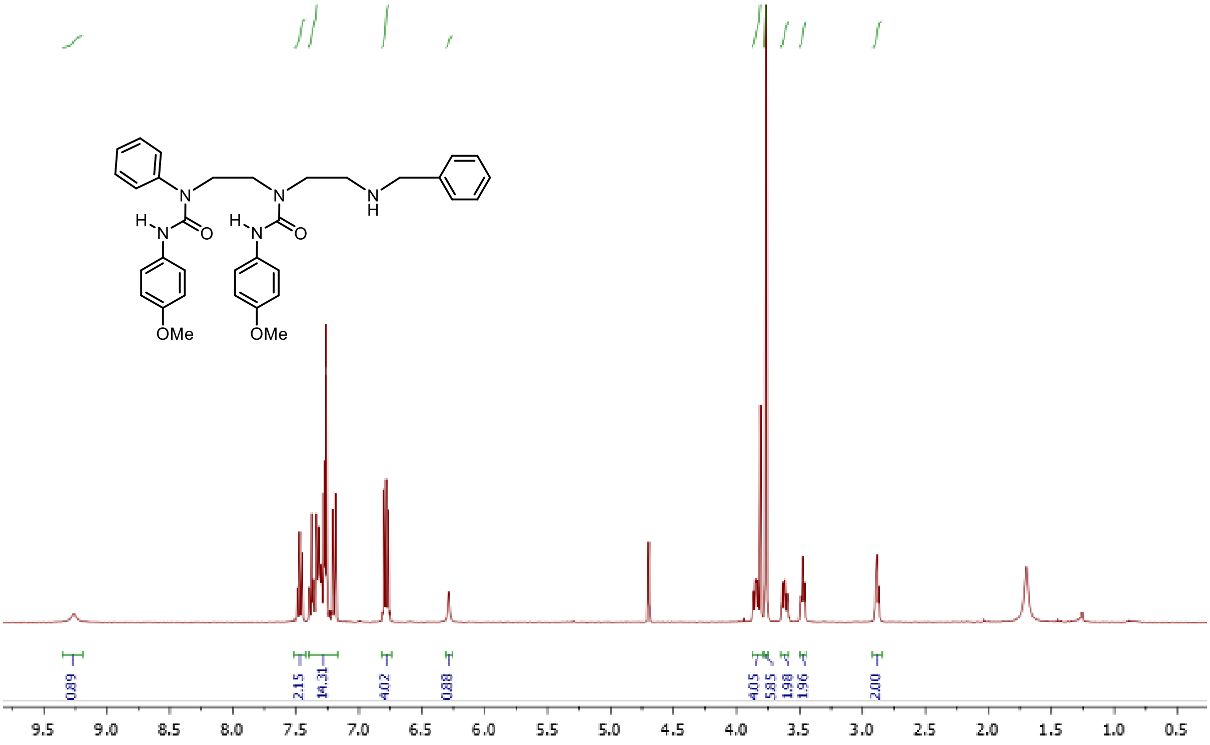

Figure S84 – ^{1}H NMR spectrum of 3d-4 (400 MHz, $CDCl_3$).

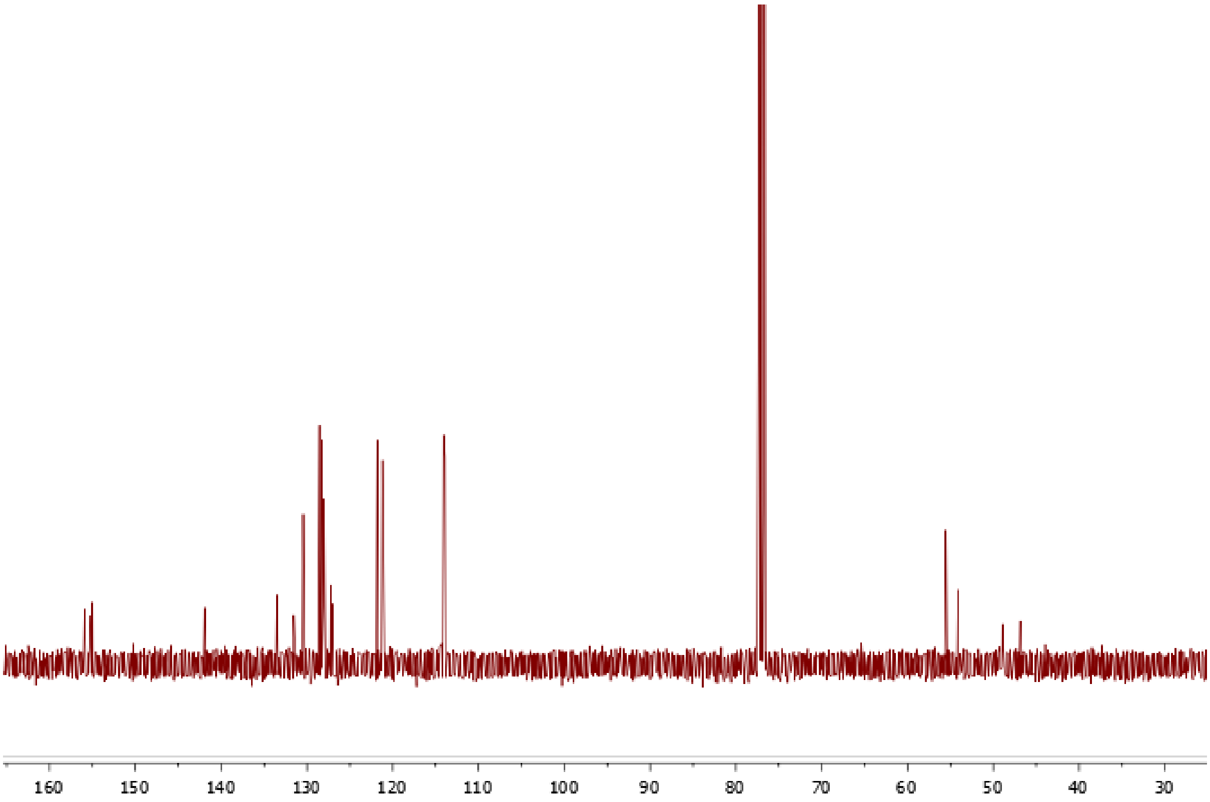

Figure S85 – ^{13}C NMR spectrum of 3d-4 (101 MHz, $CDCl_3$).

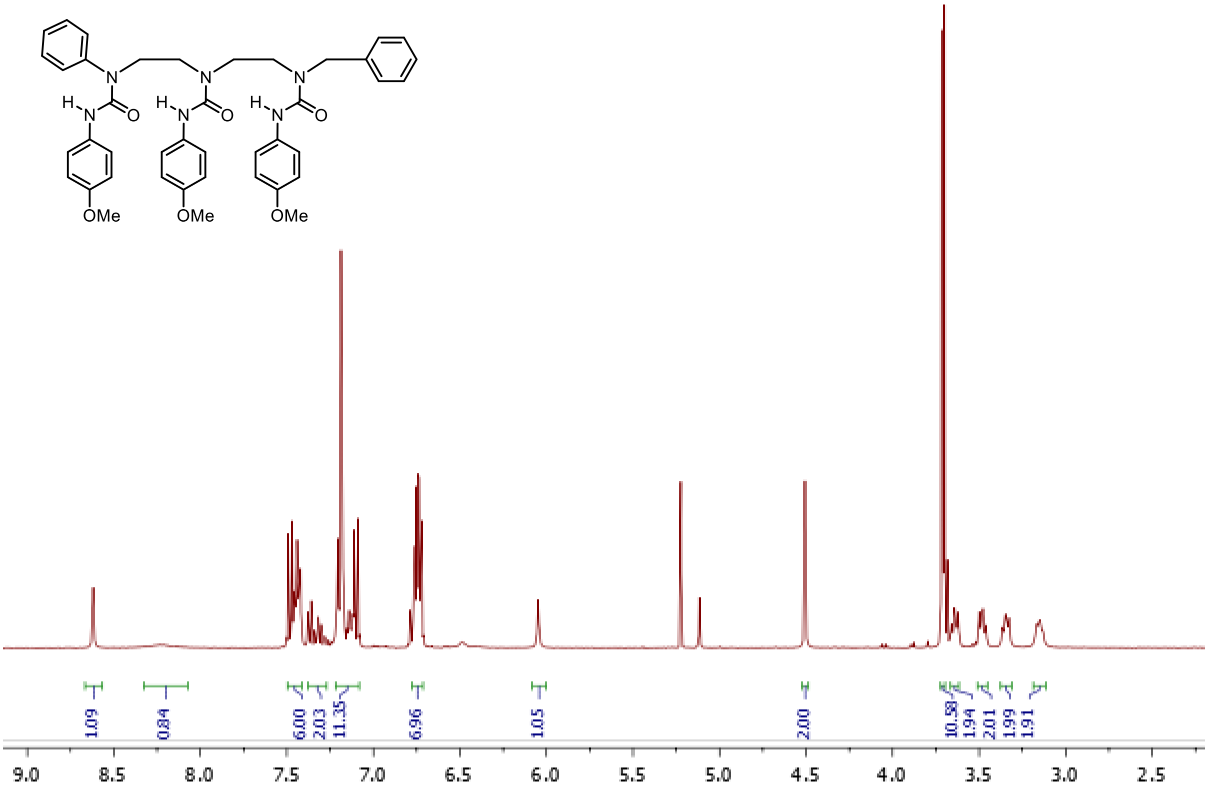

Figure S86 – ^{1}H NMR spectrum of 3d (400 MHz, $CDCl_3$).

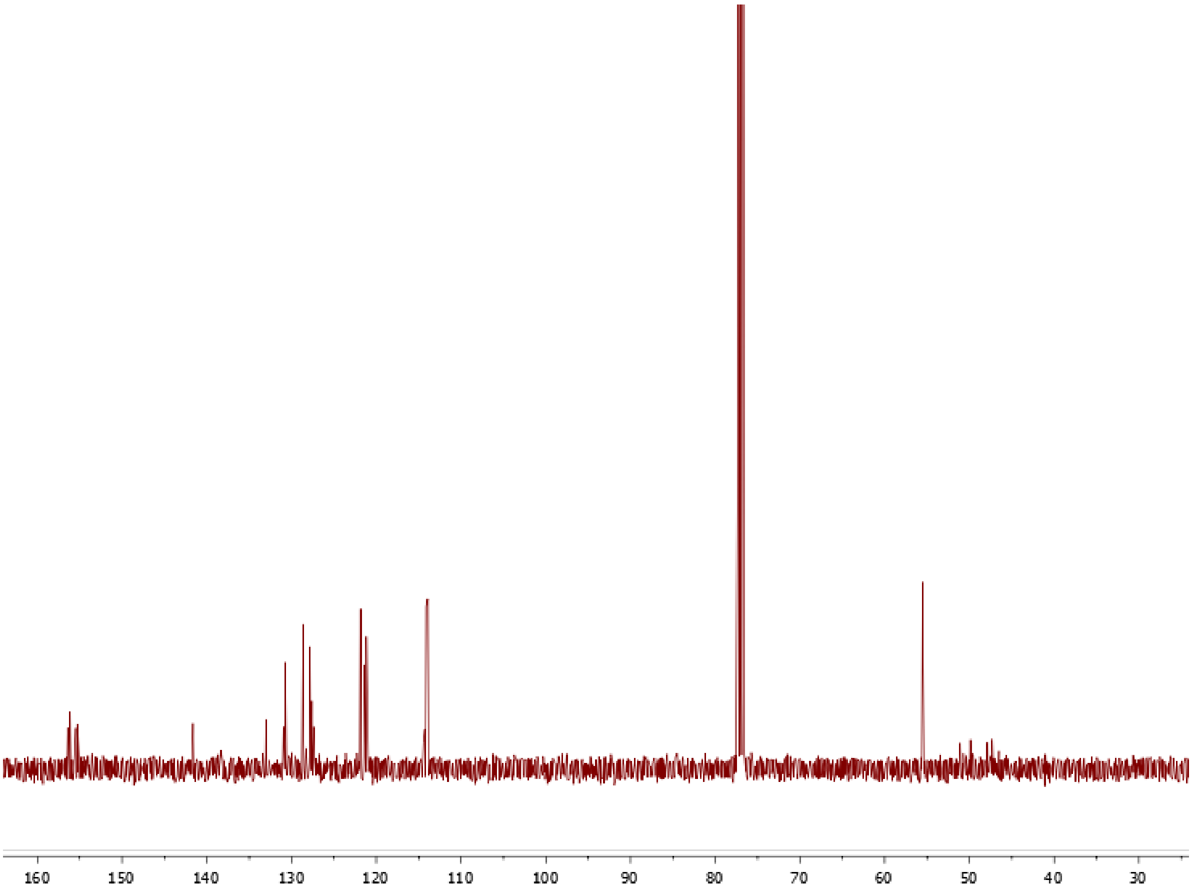

Figure S87 – ^{13}C NMR spectrum of 3d (101 MHz, $CDCl_3$).

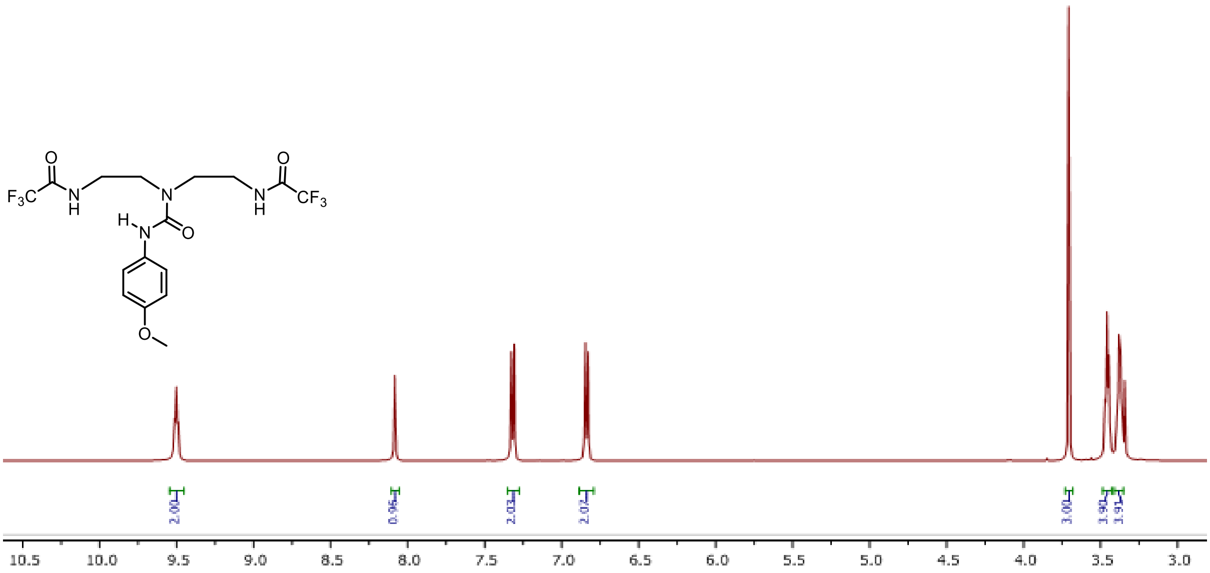

Figure S88 – ^{1}H NMR spectrum of 3e-1 (500 MHz, $(CD_3)_2SO$).

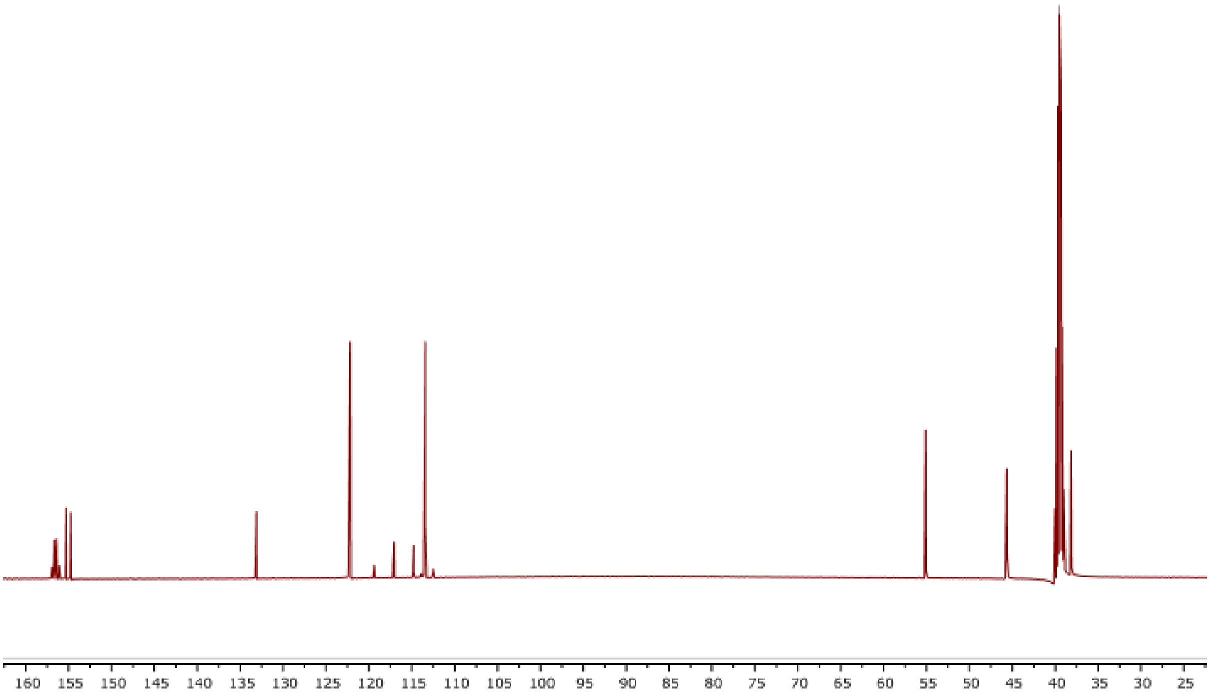

Figure S89 – ^{13}C NMR spectrum of 3e-1 (126 MHz, $(CD_3)_2SO$).

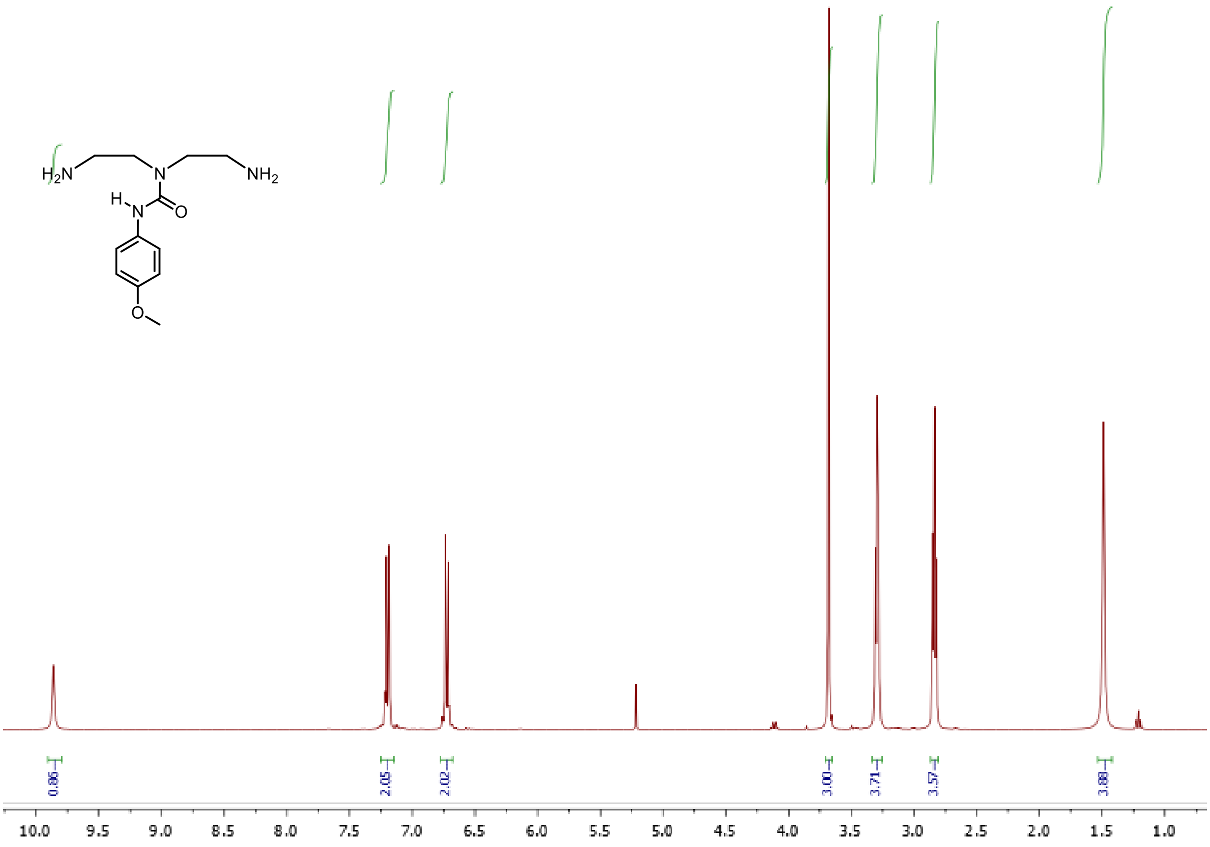

Figure S90 – ^{1}H NMR spectrum of 3e-2 (400 MHz, $CDCl_3$).

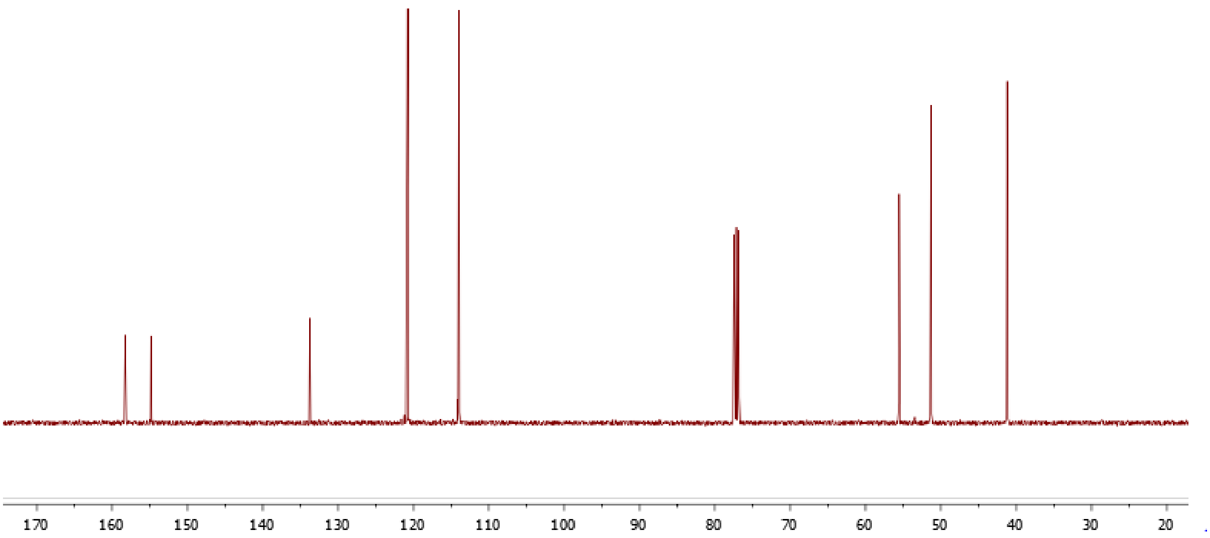

Figure S91 – ^{13}C NMR spectrum of 3e-2 (101 MHz, $CDCl_3$).

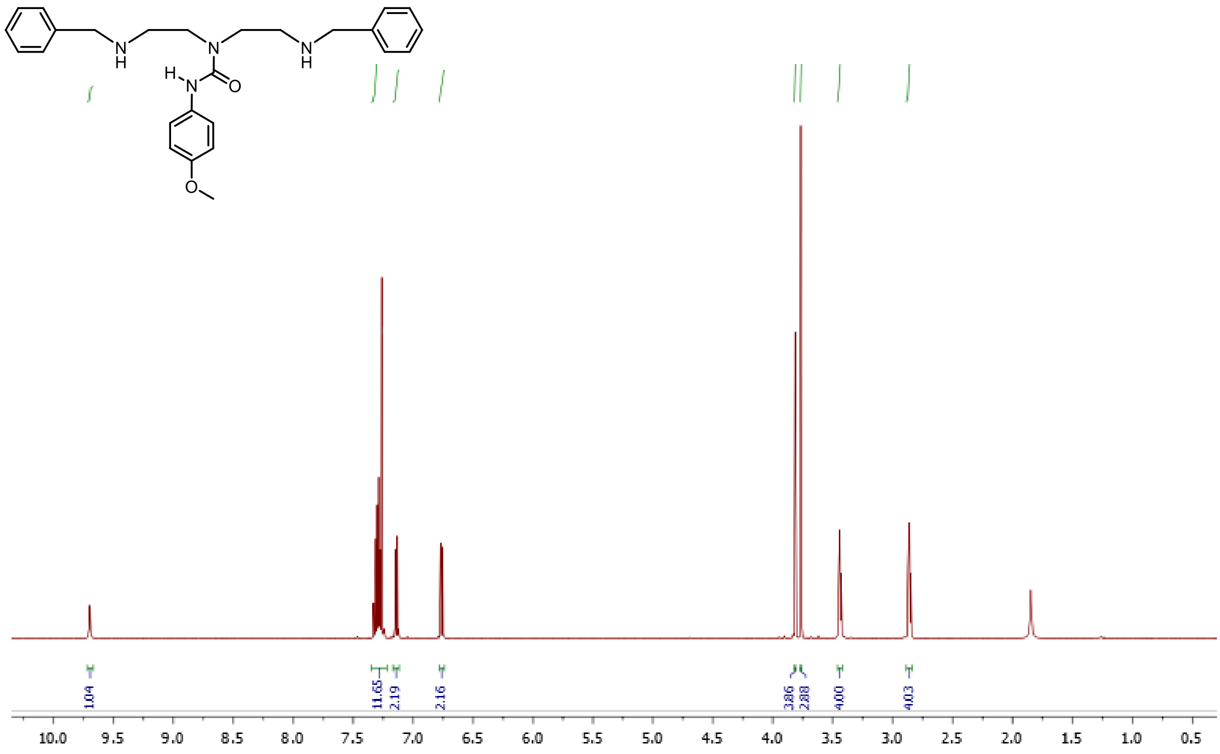

Figure S92 – ^{1}H NMR spectrum of 3e-3 (500 MHz, $CDCl_3$).

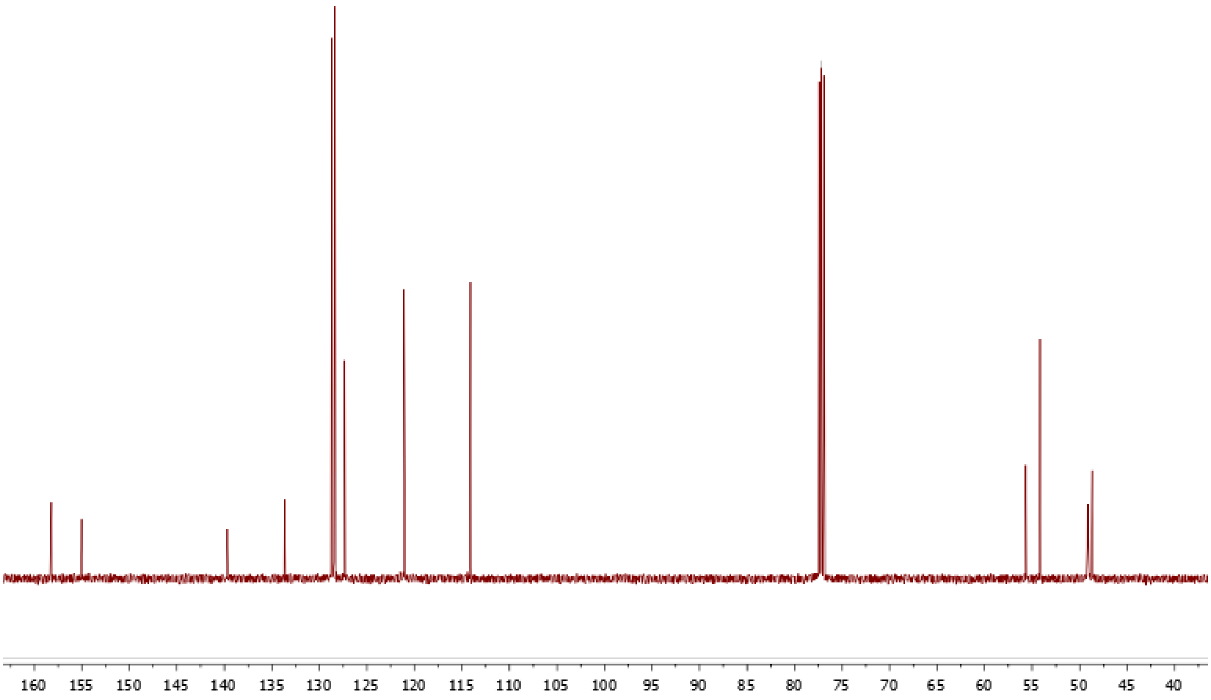

Figure S93 – ^{13}C NMR spectrum of 3e-3 (126 MHz, $CDCl_3$).

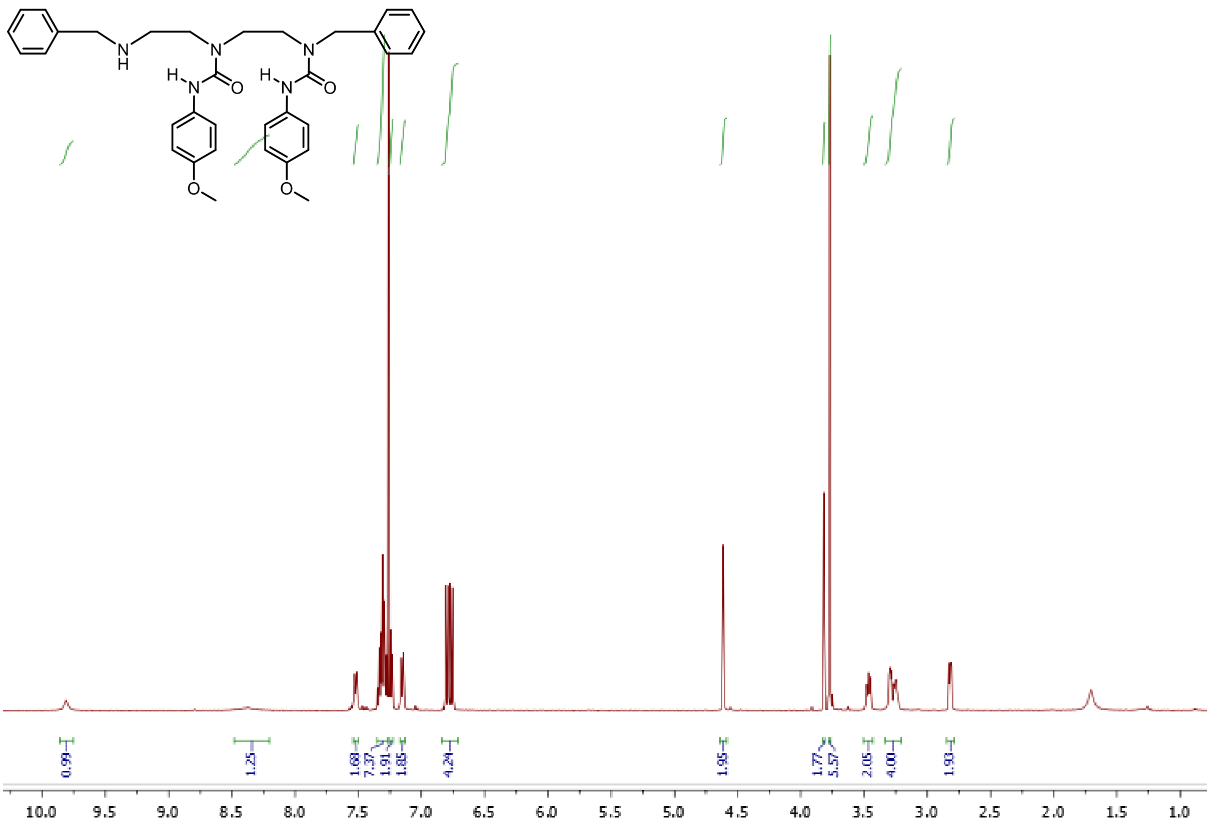

Figure S94 – ^{1}H NMR spectrum of 3e-4 (500 MHz, $CDCl_3$).

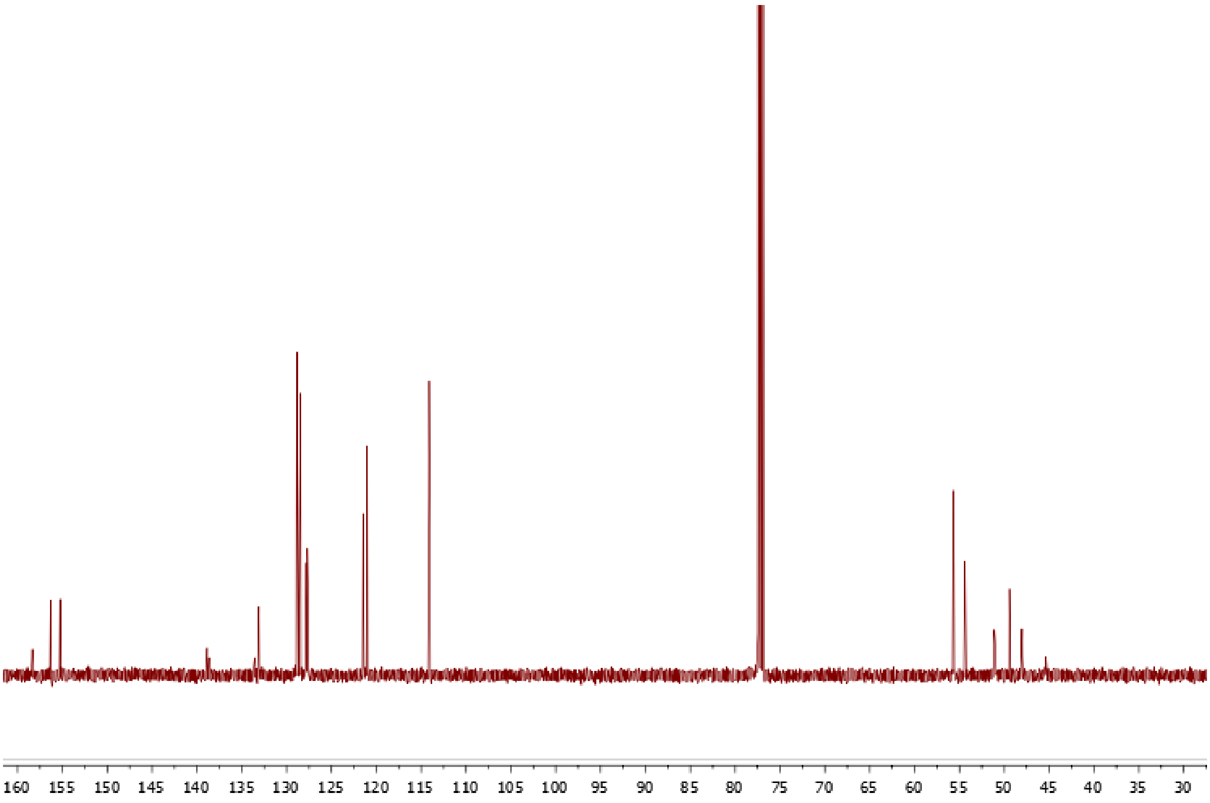

Figure S95 – ^{13}C NMR spectrum of 3e-4 (126 MHz, $CDCl_3$).

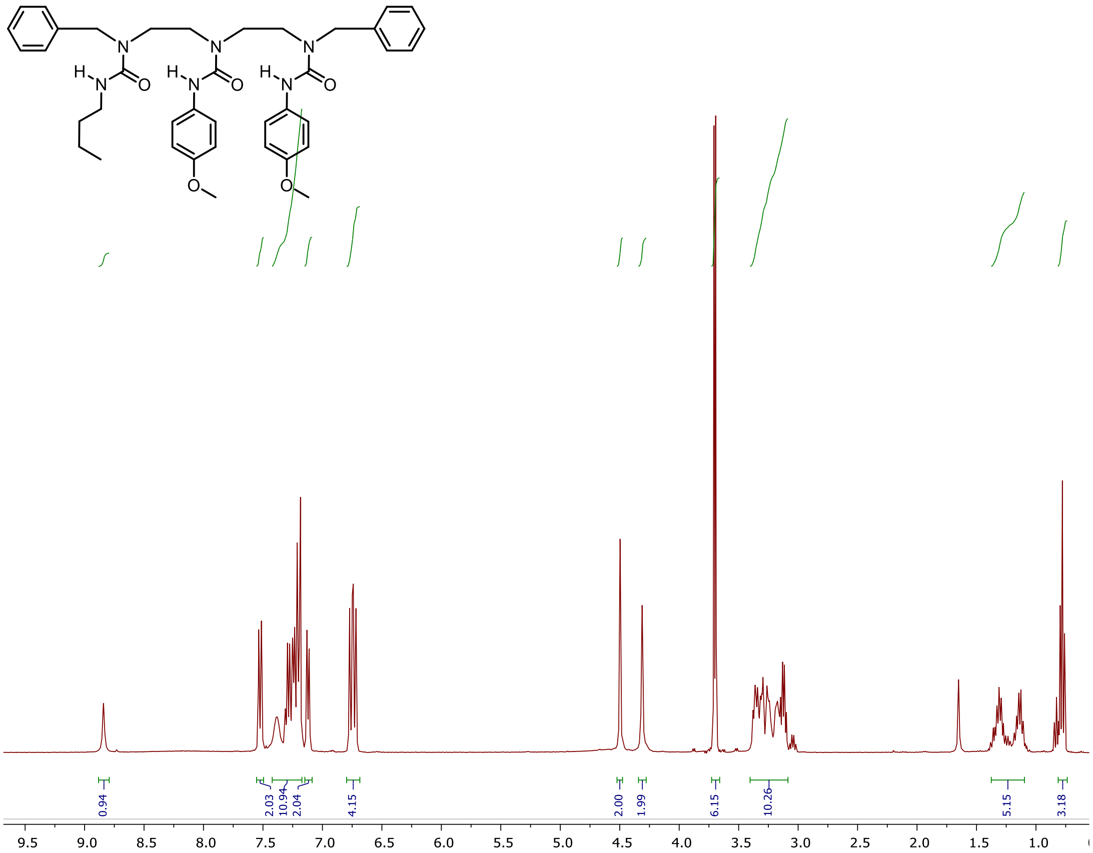

Figure S96 – ^{1}H NMR spectrum of 3e (400 MHz, $CDCl_3$).

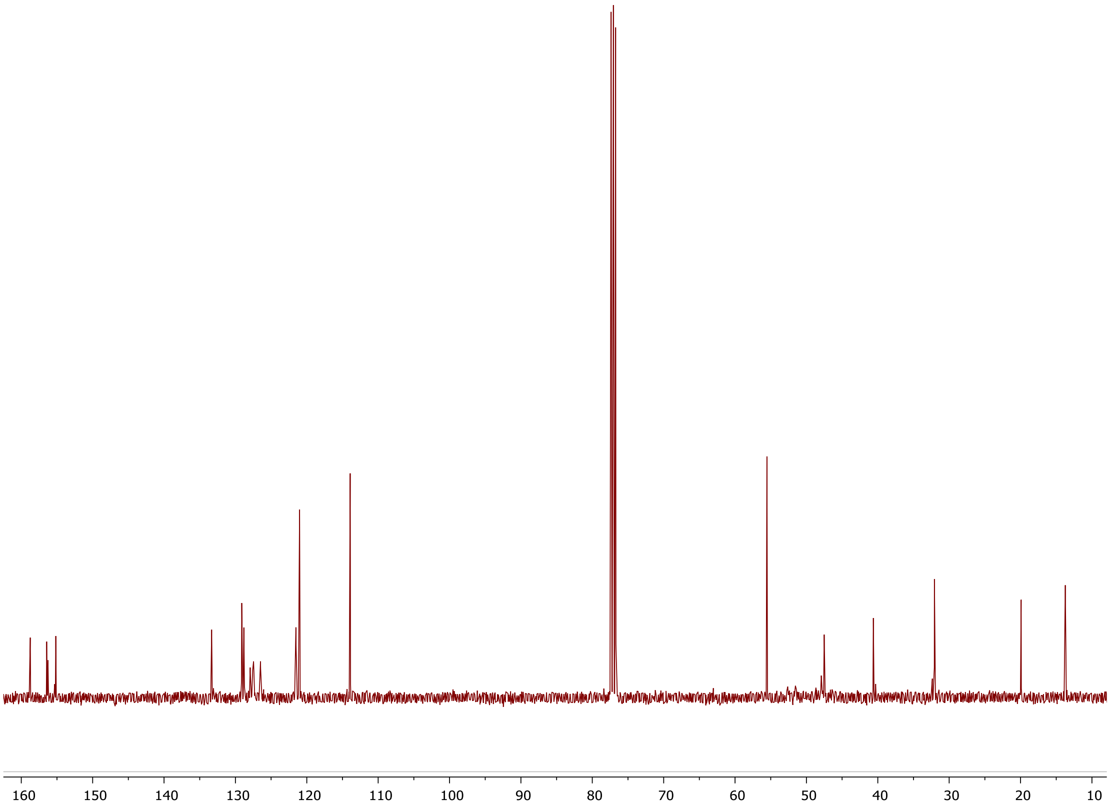

Figure S97 – ^{13}C NMR spectrum of 3e (101 MHz, $CDCl_3$).

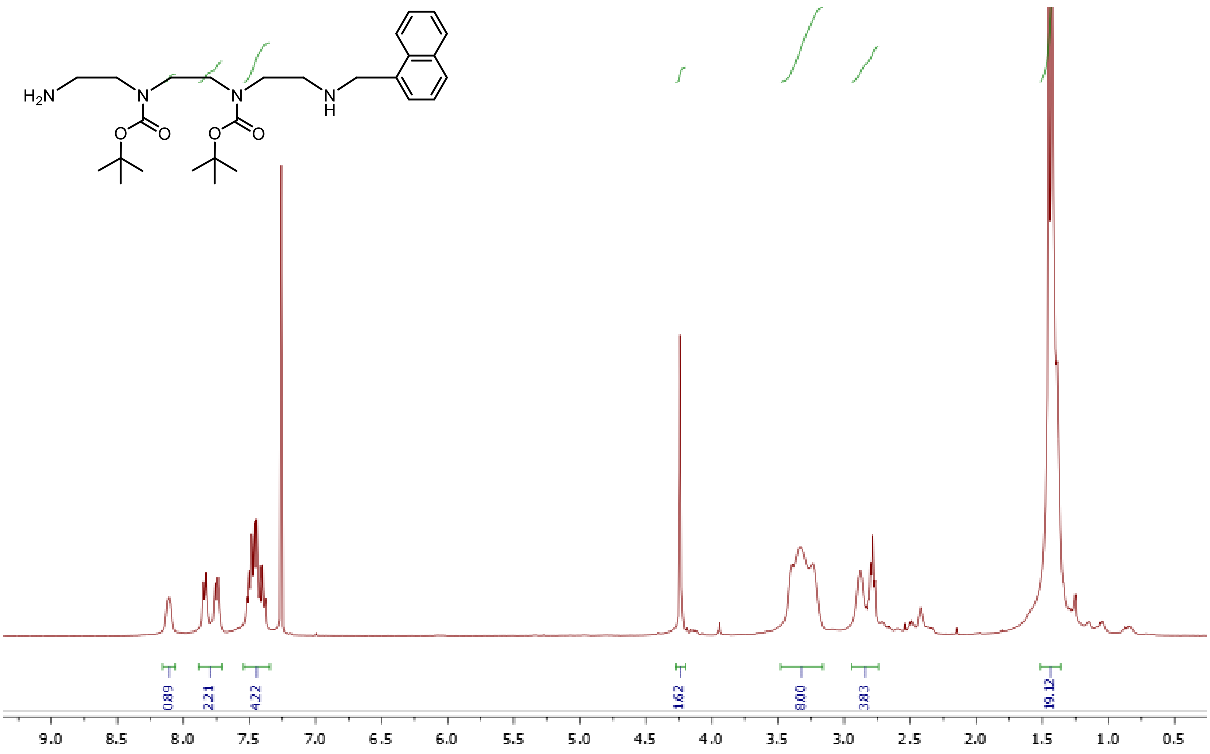

Figure S98 – ^{1}H NMR spectrum of 3f-2 (400 MHz, $CDCl_3$).

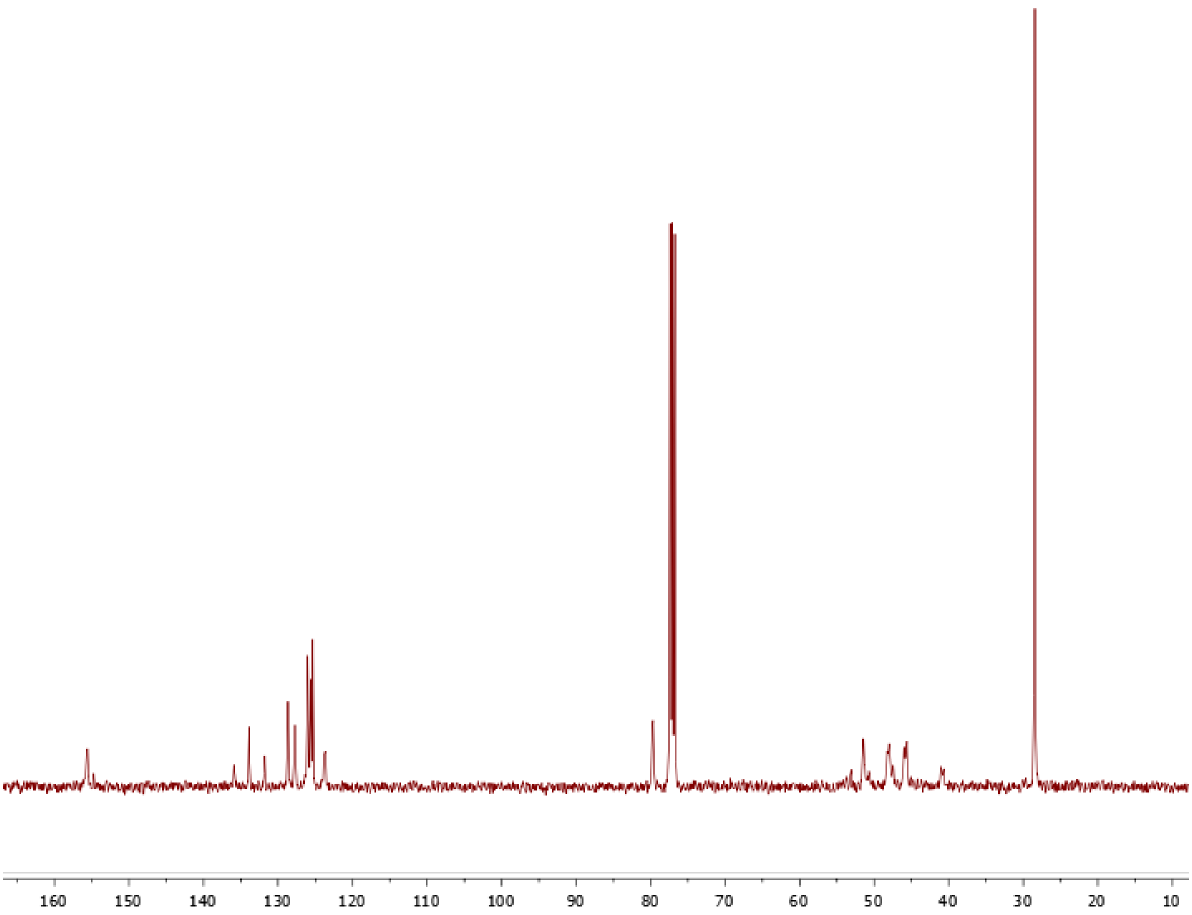

Figure S99 – ^{13}C NMR spectrum of 3f-2 (101 MHz, $CDCl_3$).

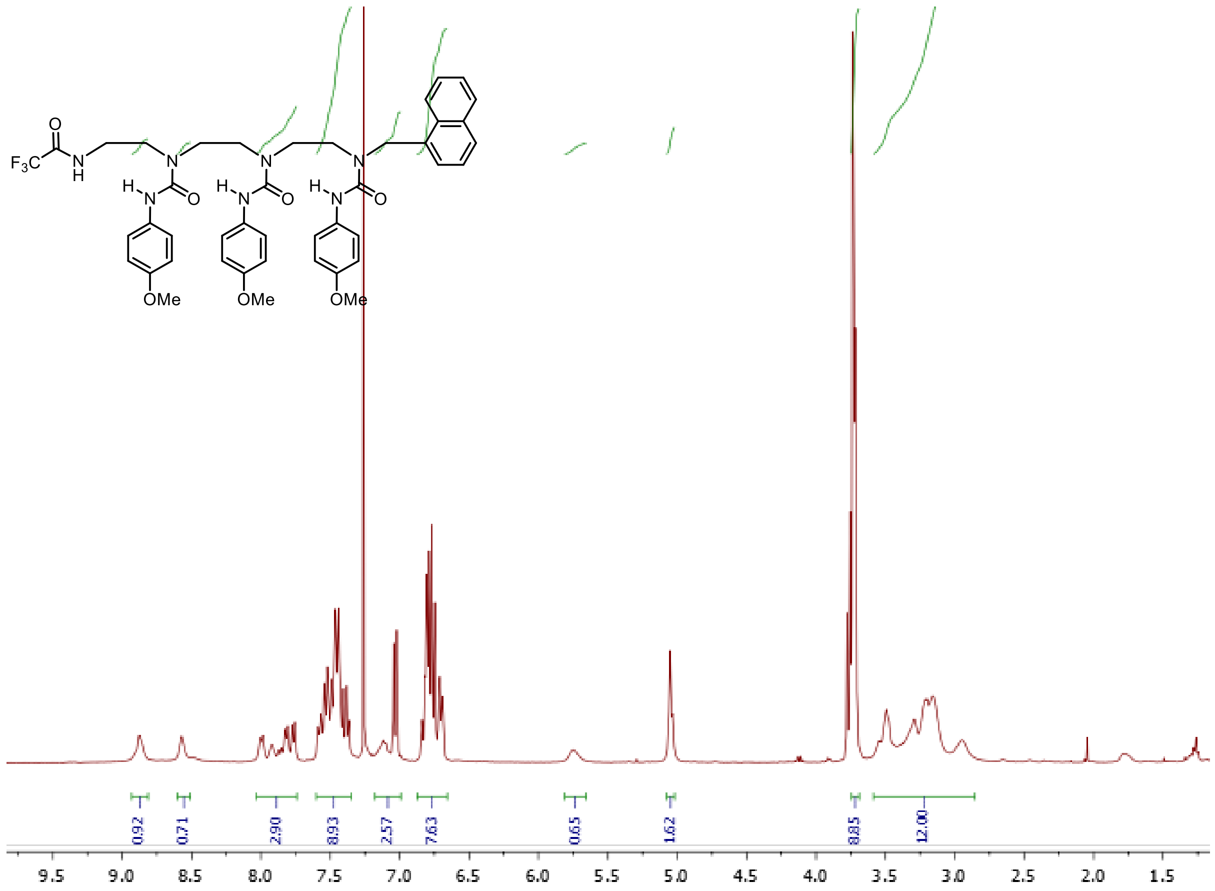

Figure S100 – ^{1}H NMR spectrum of 3f-3 (400 MHz, $CDCl_3$).

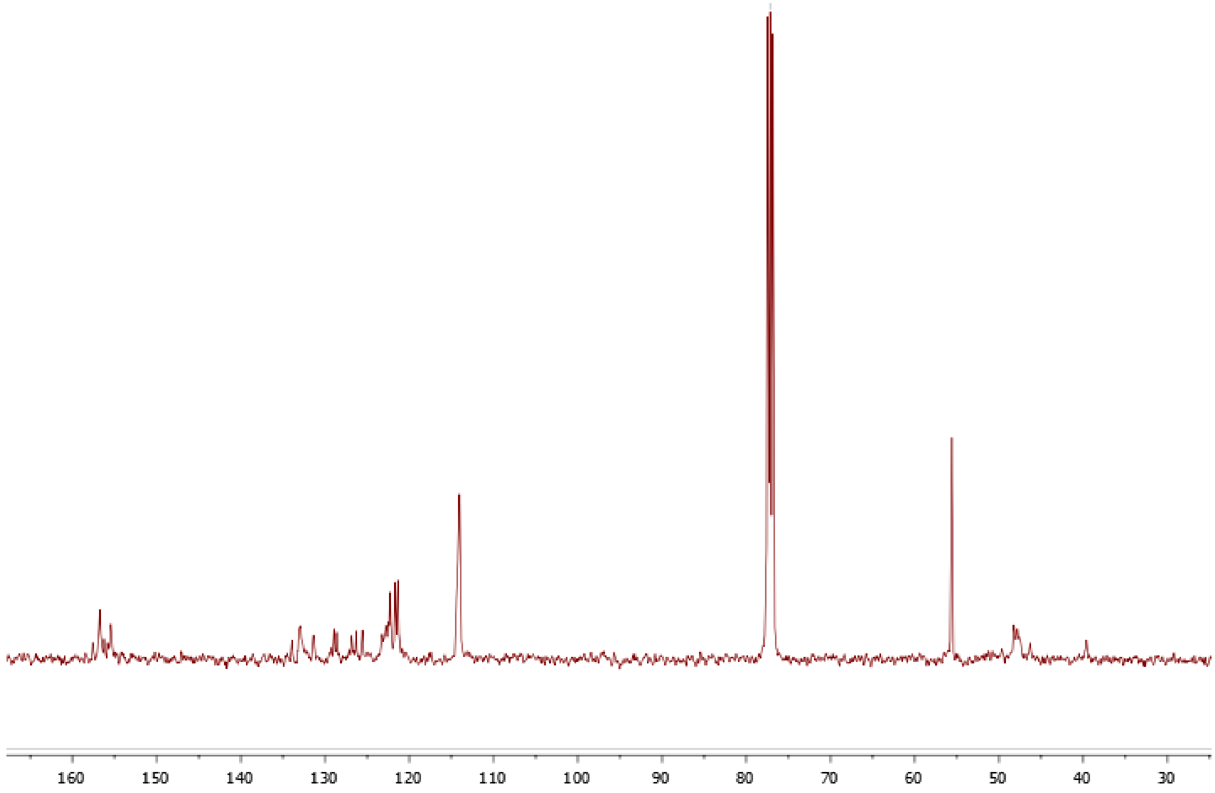

Figure S101 – ^{13}C NMR spectrum of 3f-3 (101 MHz, $CDCl_3$).

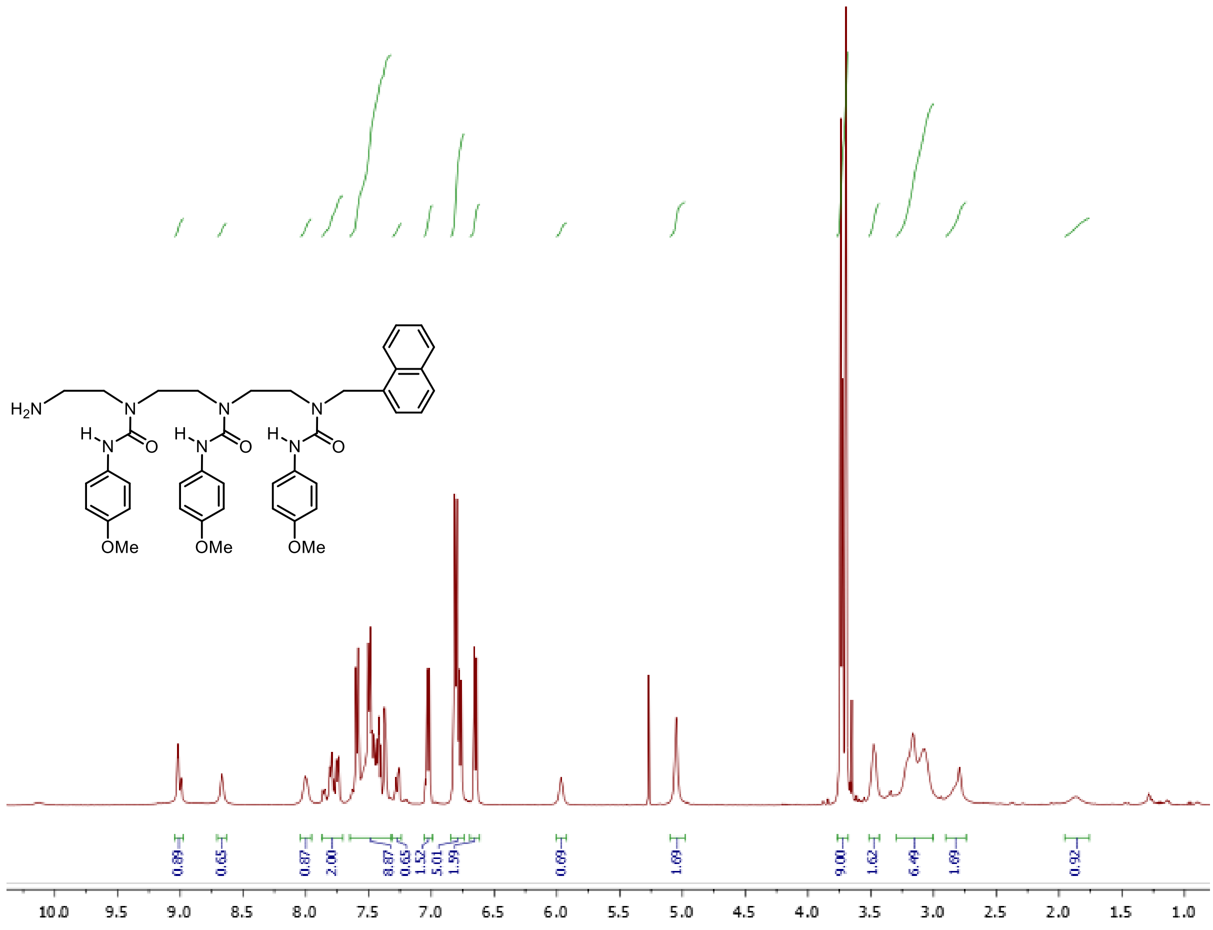

Figure S102 – ^{1}H NMR spectrum of 3f-4 (500 MHz, $CDCl_3$).

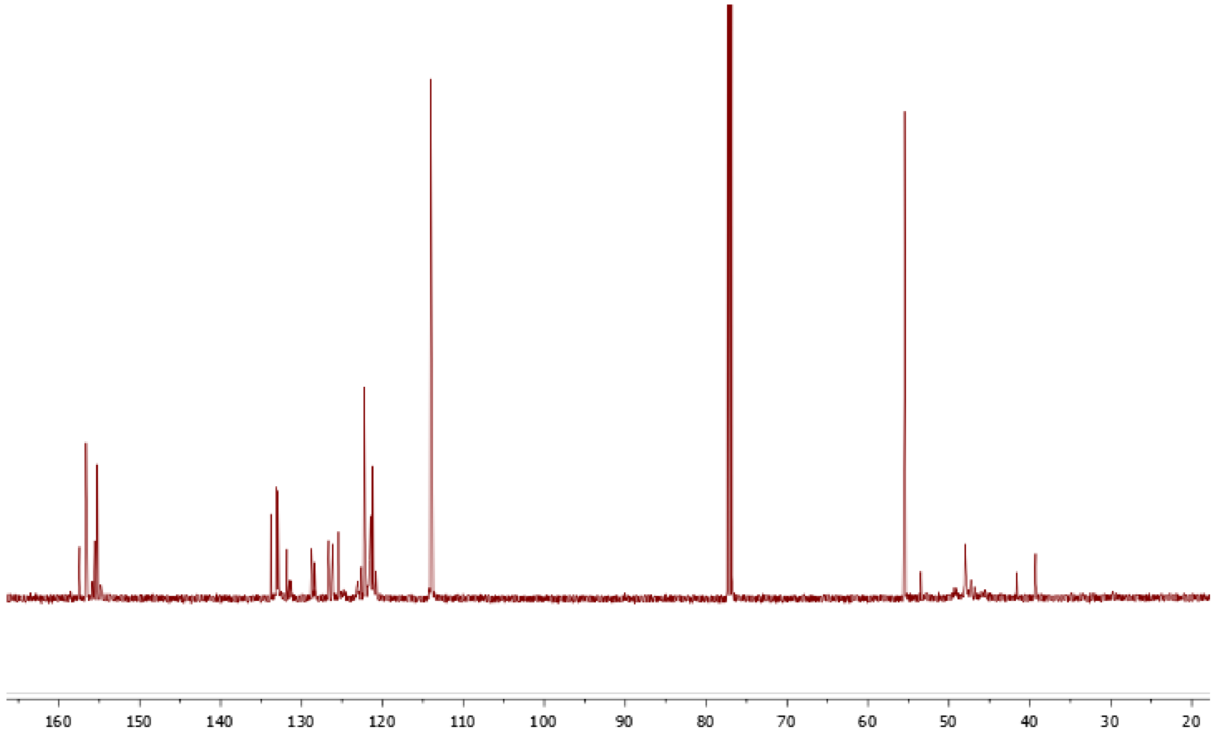

Figure S103 – ^{13}C NMR spectrum of 3f-4 (126 MHz, $CDCl_3$).

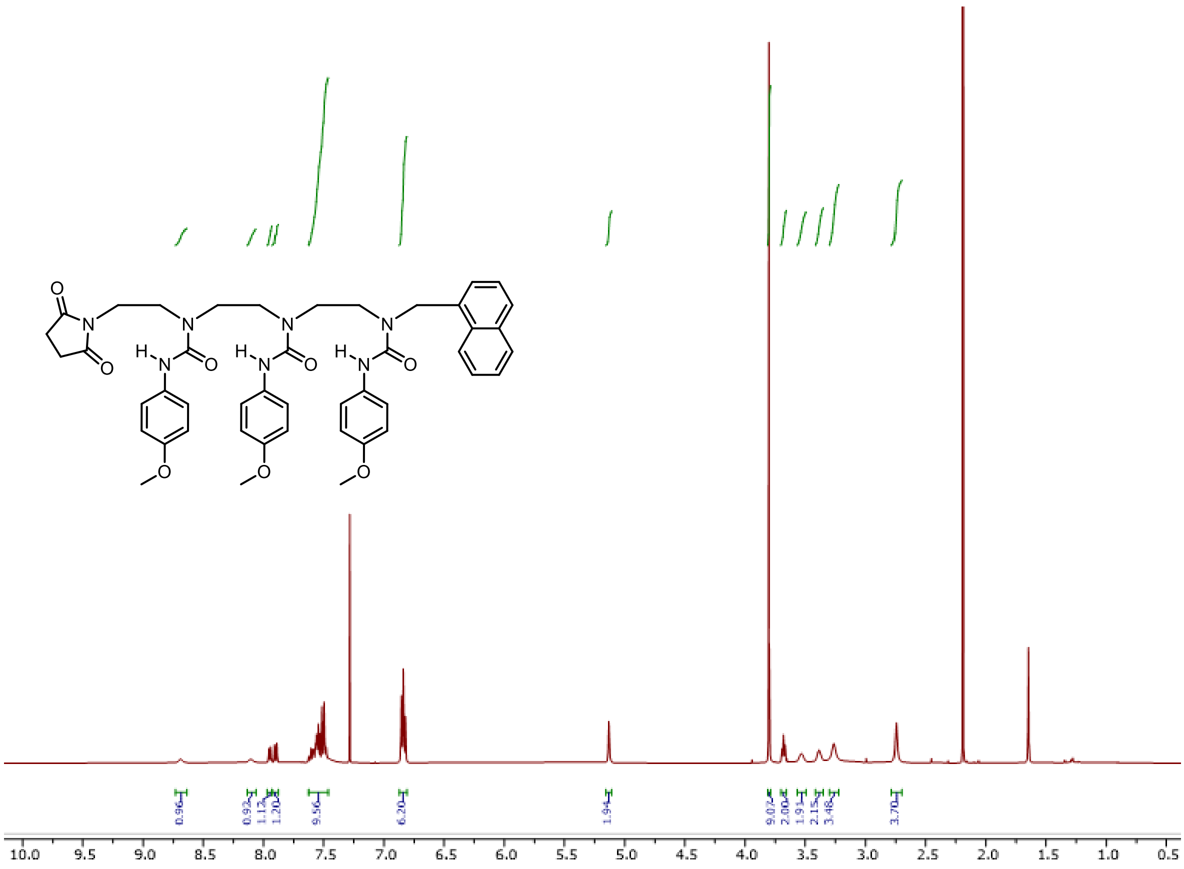

Figure S104 – ^{1}H NMR spectrum of 3f (500 MHz, $CDCl_3$).

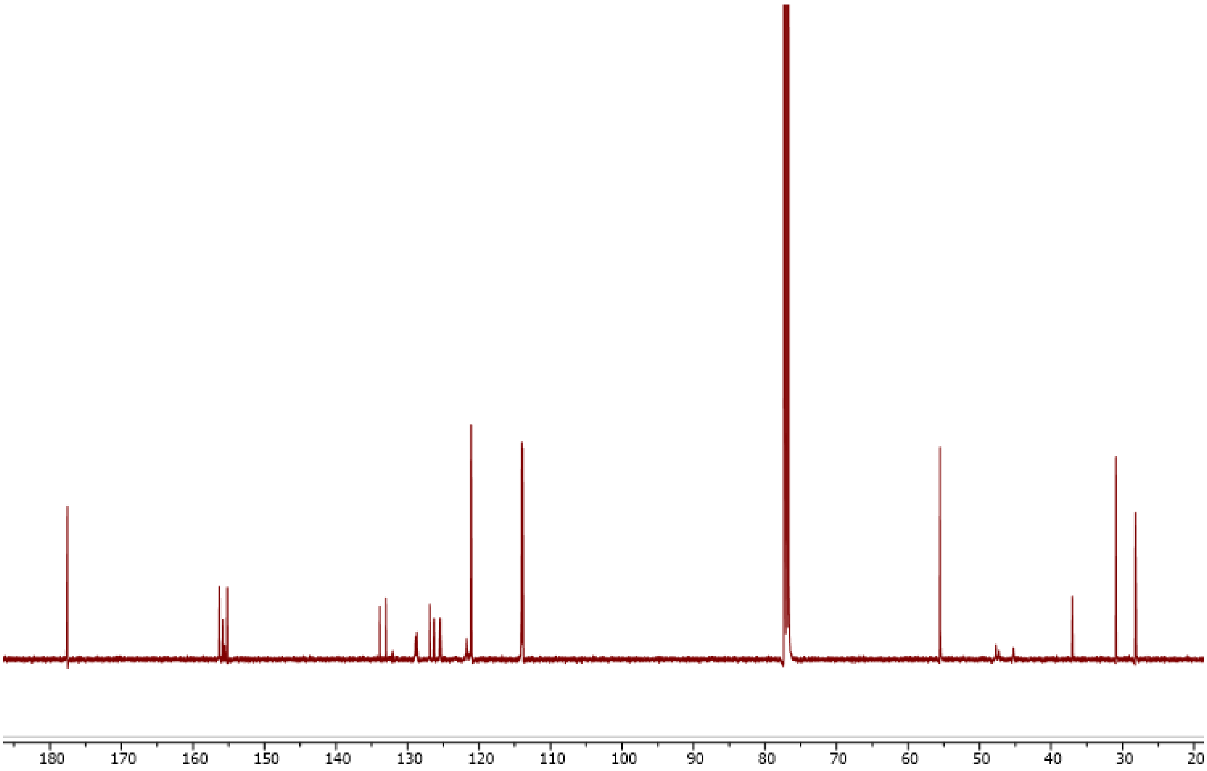

Figure S105 – ^{13}C NMR spectrum of 3f (126 MHz, $CDCl_3$).

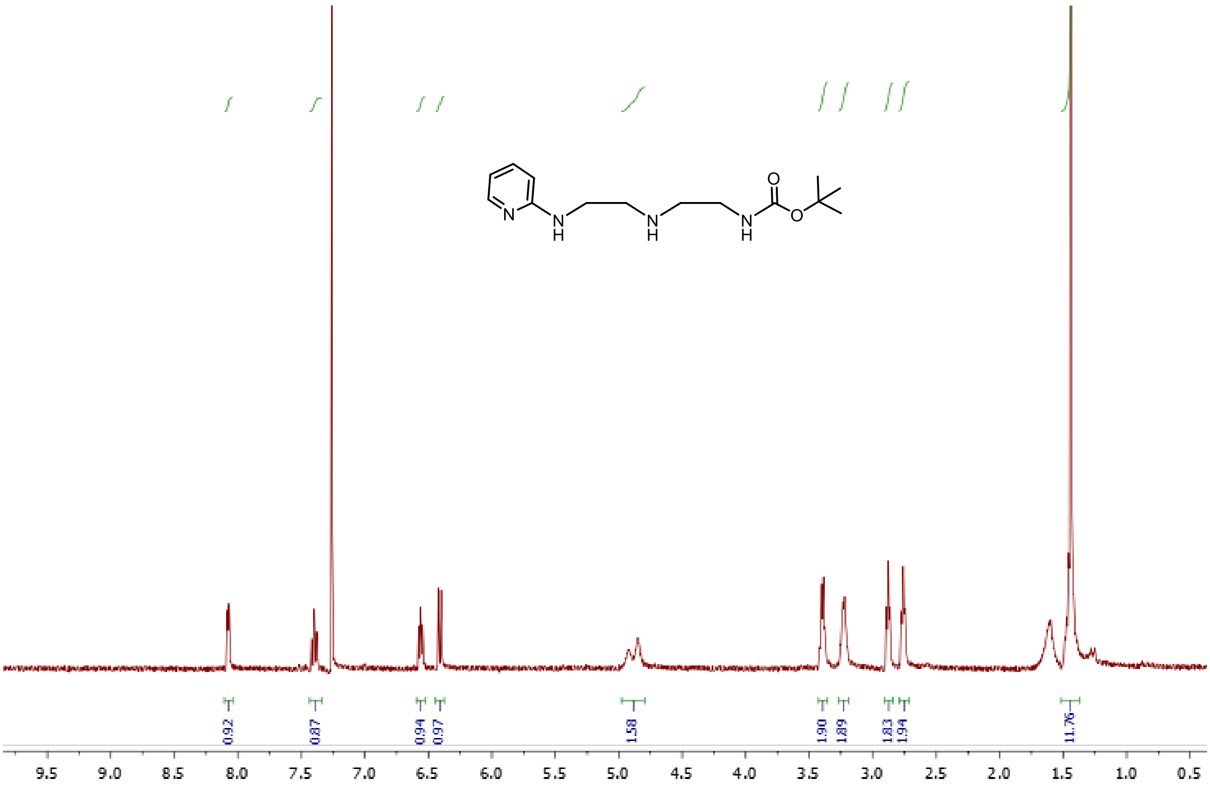

Figure S106 – ^{1}H NMR spectrum of 3g-2 (400 MHz, $CDCl_3$).

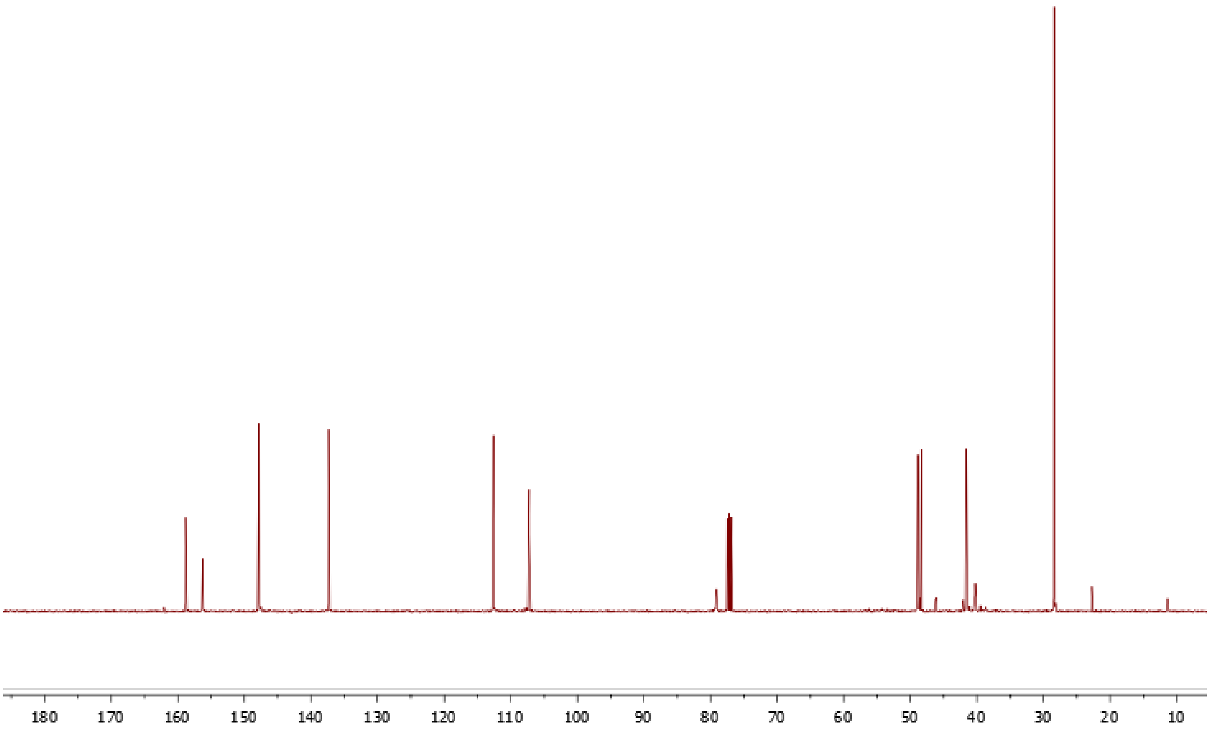

Figure S107 – ^{13}C NMR spectrum of 3g-2 (101 MHz, $CDCl_3$).

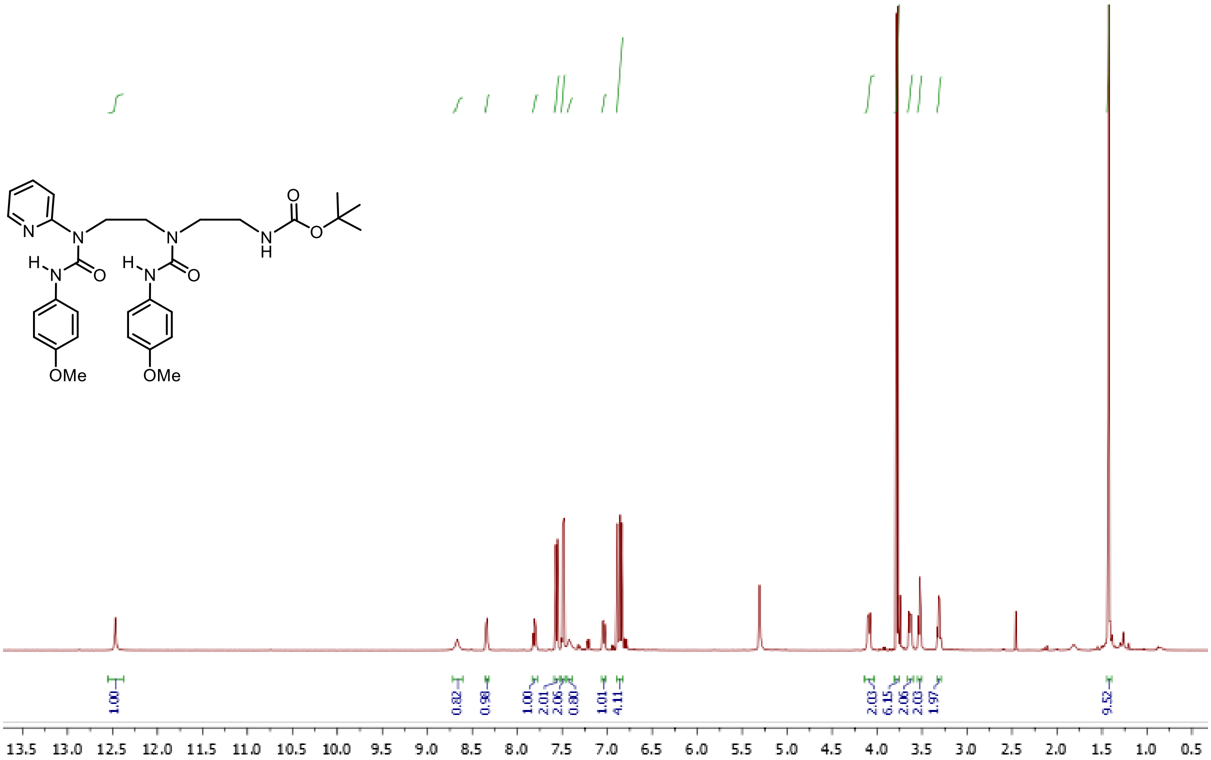

Figure S108 – ^{1}H NMR spectrum of 3g-3 (400 MHz, CD_2Cl_2).

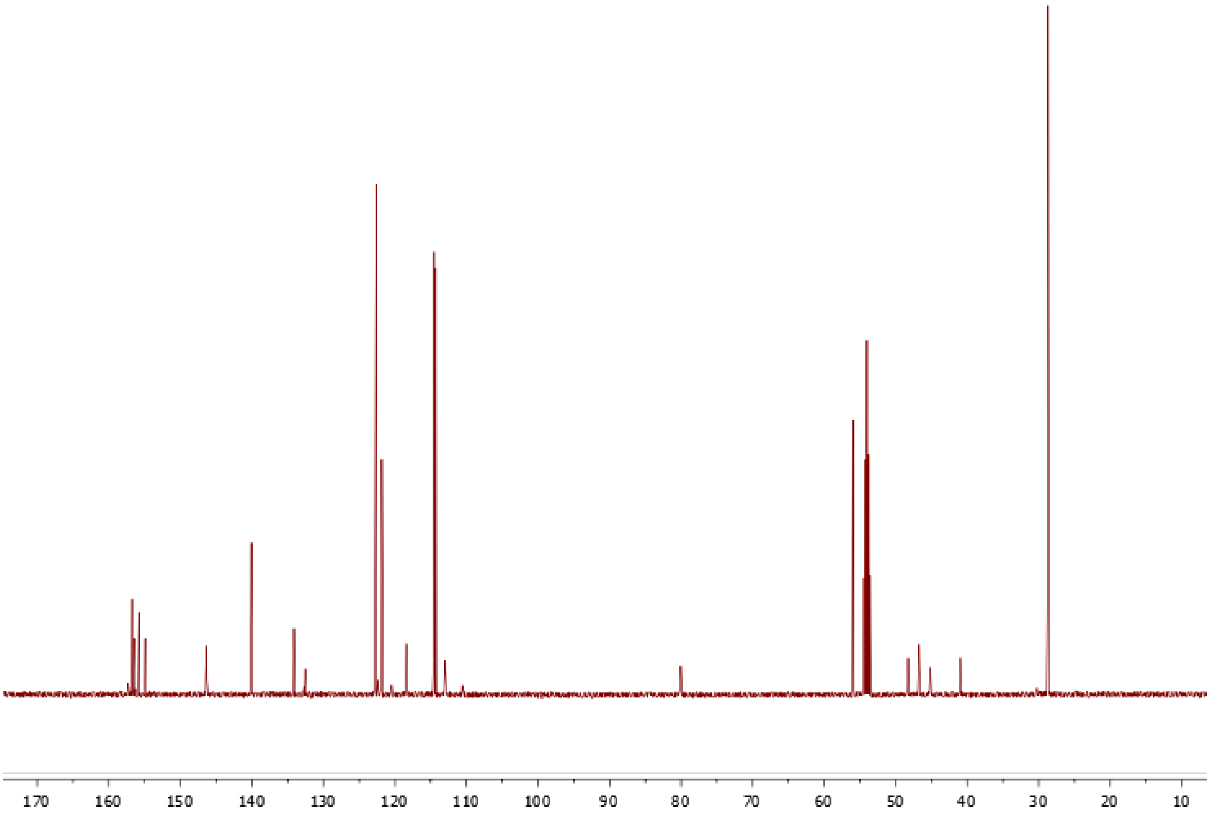

Figure S109 – ^{13}C NMR spectrum of 3g-3 (101 MHz, CD_2Cl_2).

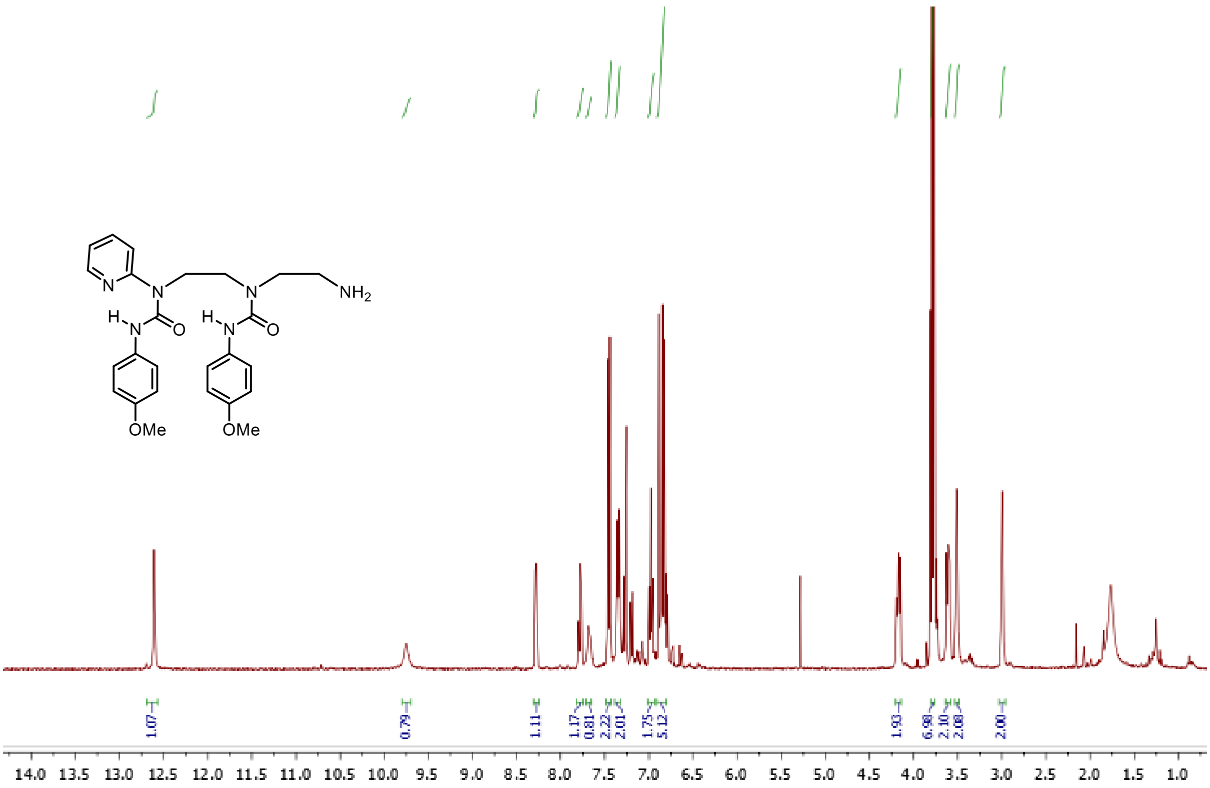

Figure S110 – ^{1}H NMR spectrum of 3g-4 (400 MHz, CD_2Cl_2).

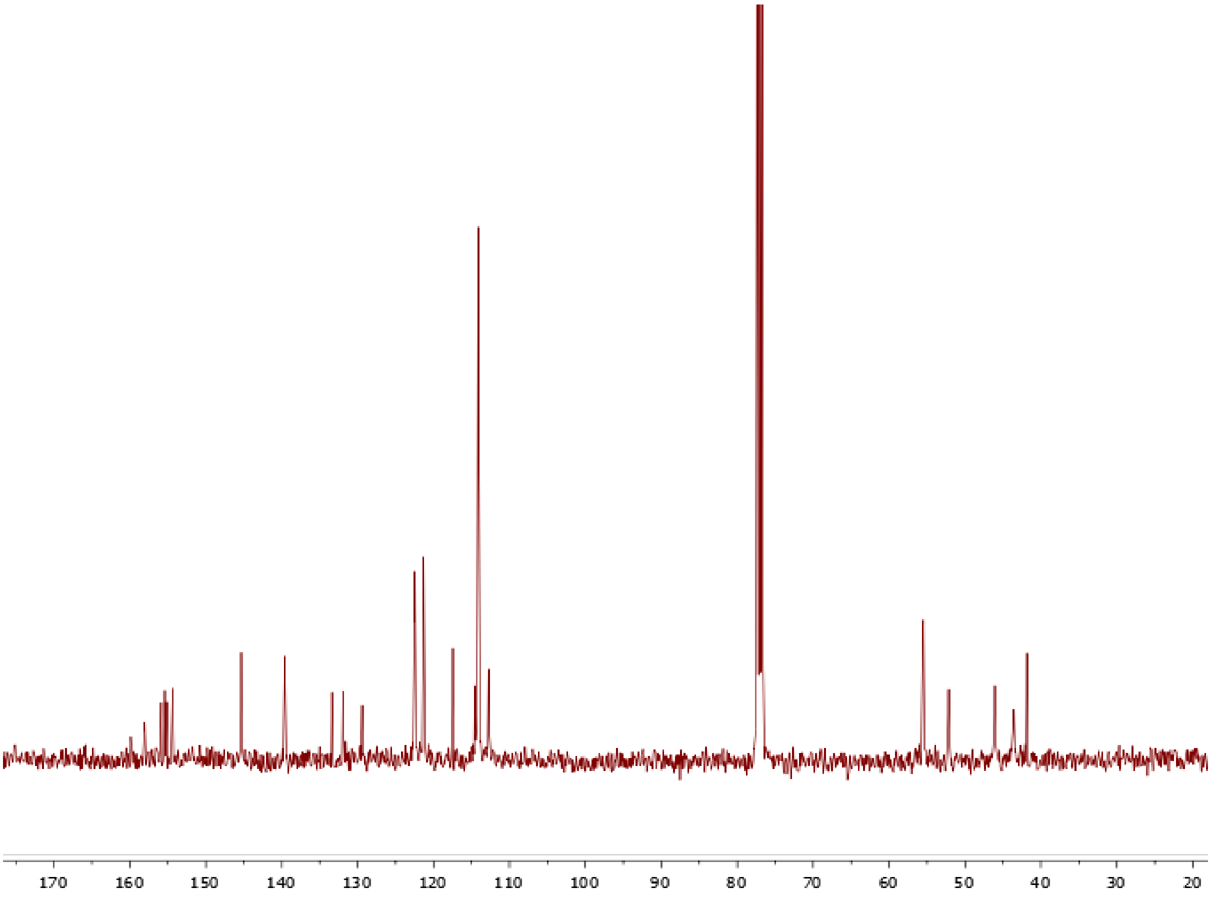

Figure S111 – ^{13}C NMR spectrum of 3g-4 (101 MHz, $CDCl_3$).

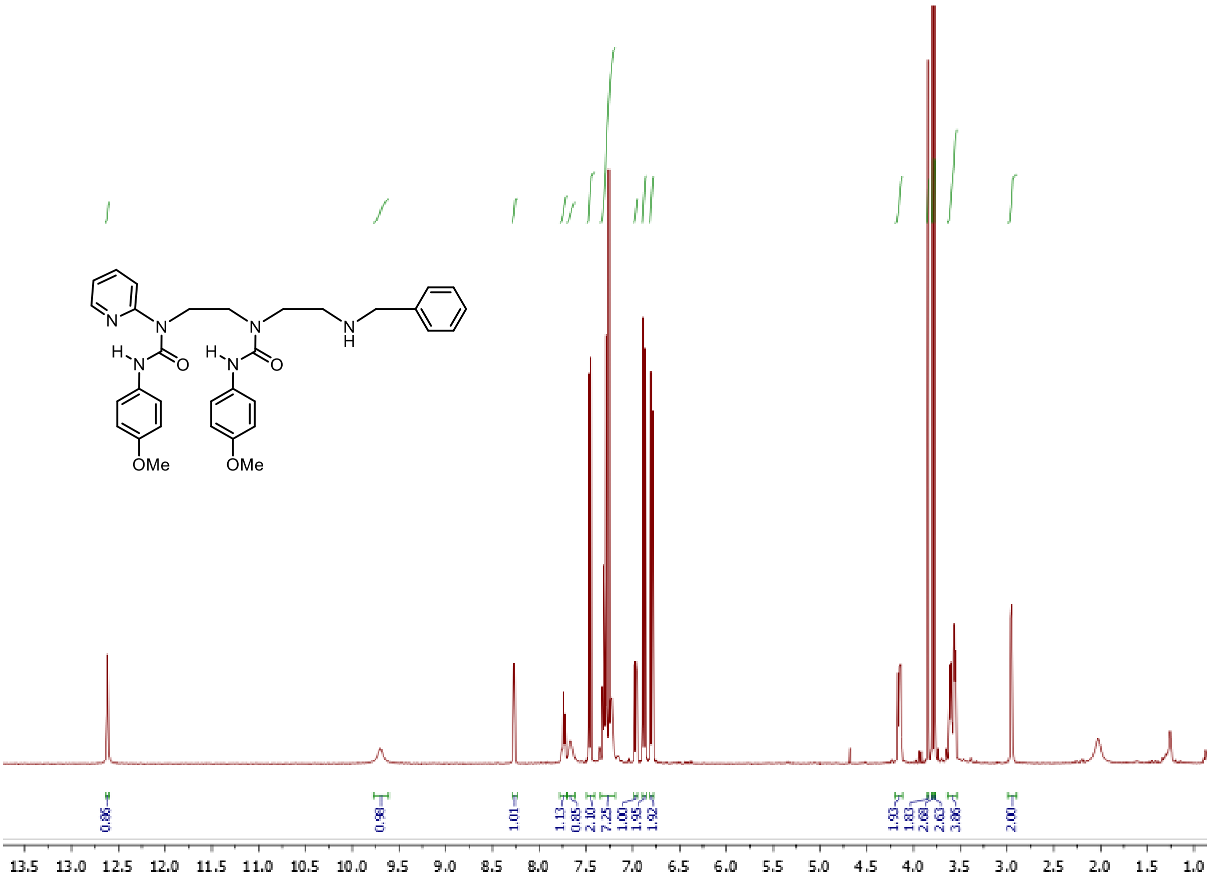

Figure S112 – ¹H NMR spectrum of 3g-5 (500 MHz, $CDCl_3$).

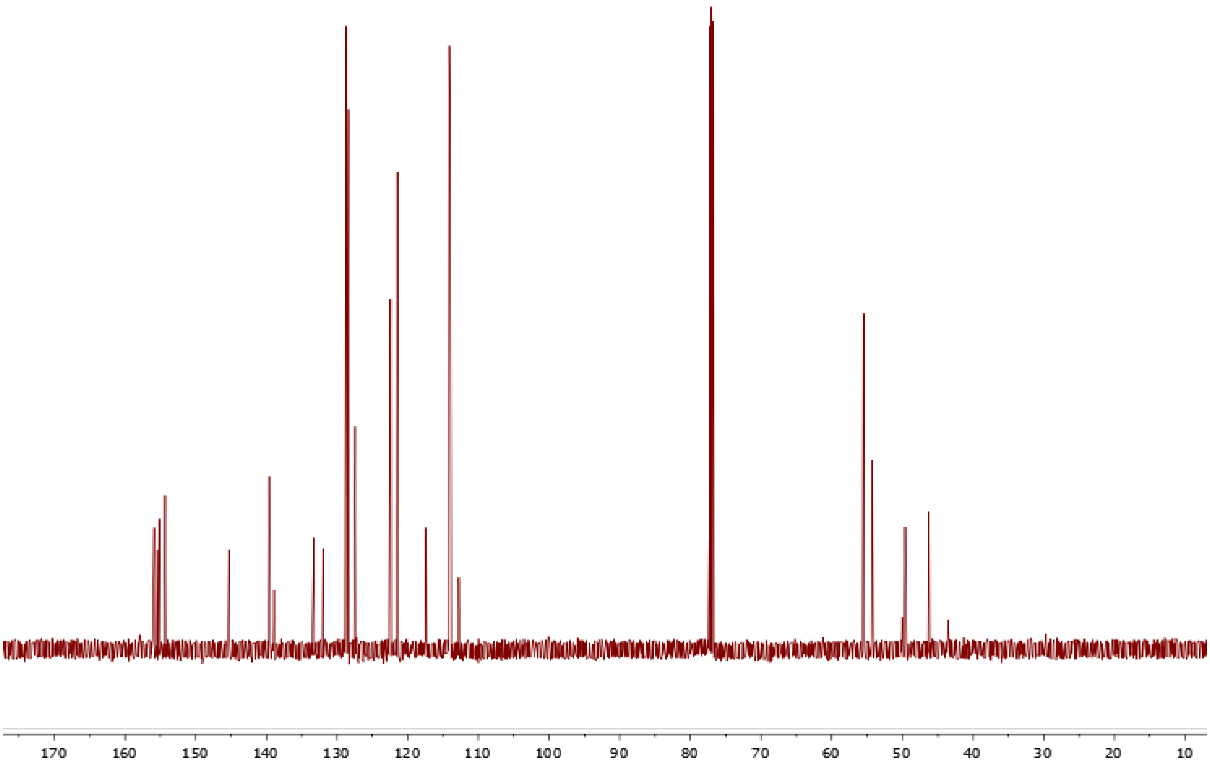

Figure S113 – ¹³C NMR spectrum of 3g-5 (126 MHz, $CDCl_3$).

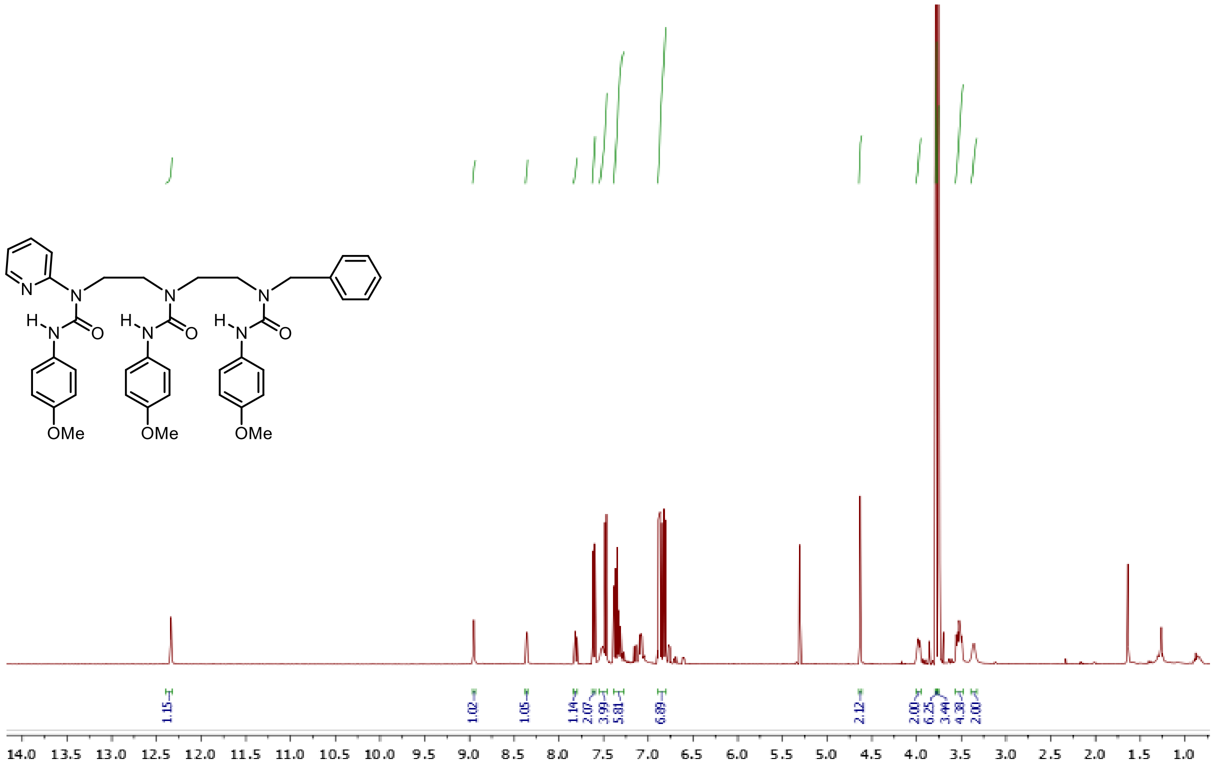

Figure S114 – ^{1}H NMR spectrum of 3g (500 MHz, CD_2Cl_2).

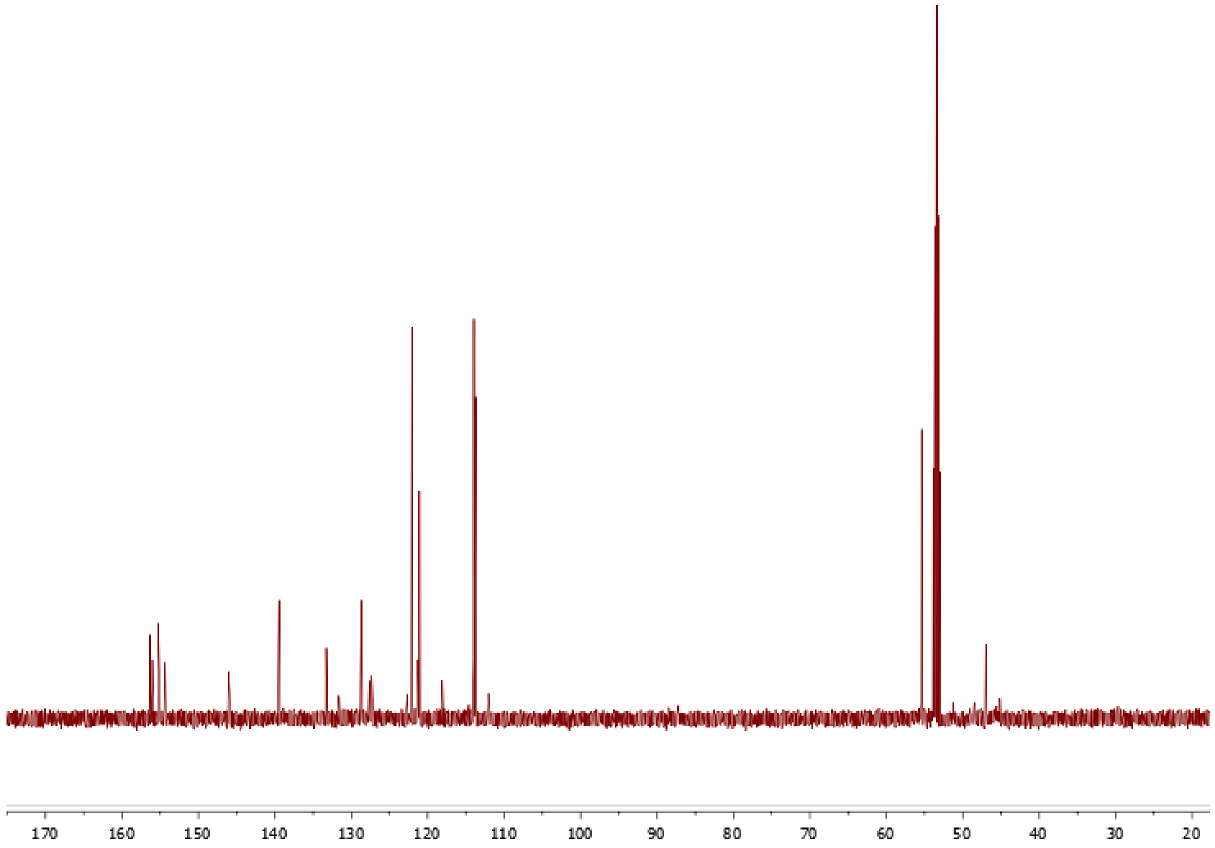

Figure S115 – ^{13}C NMR spectrum of 3g (126 MHz, CD_2Cl_2).

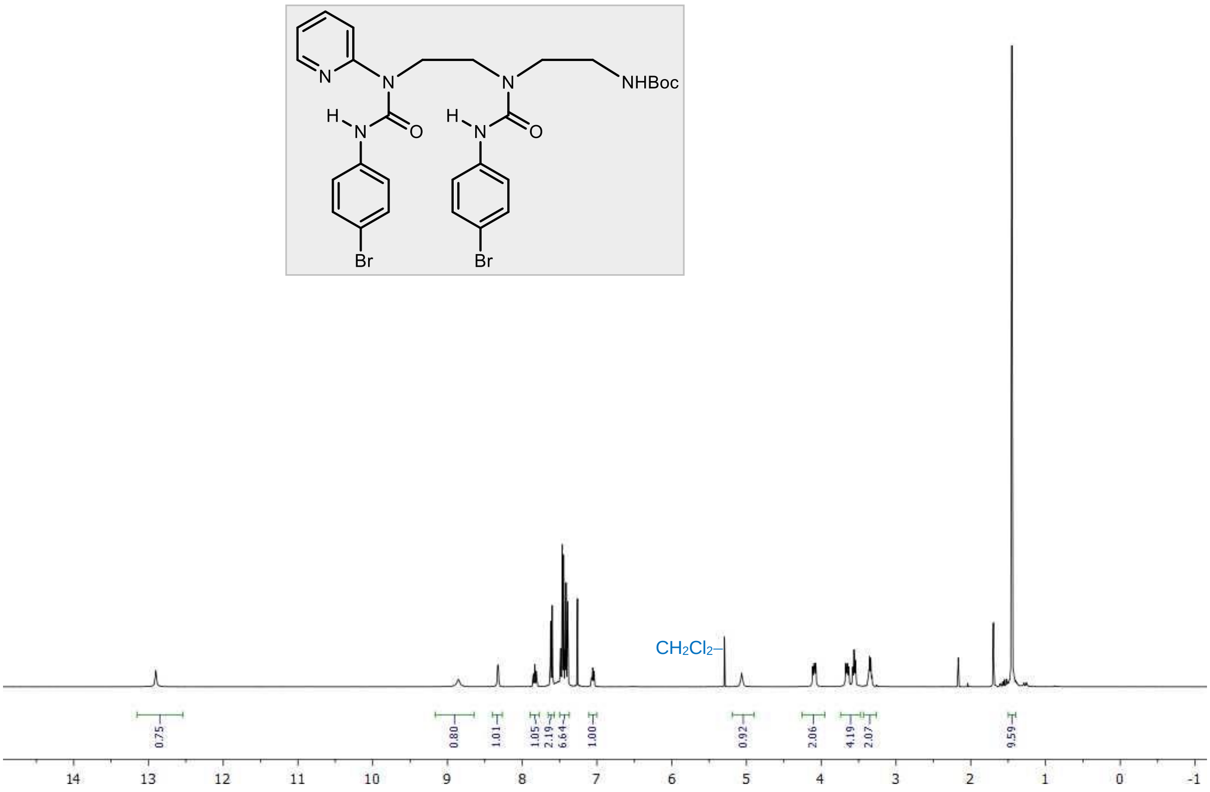

Figure S116 – ^{1}H NMR spectrum of 3h-1 (400 MHz, $CDCl_3$).

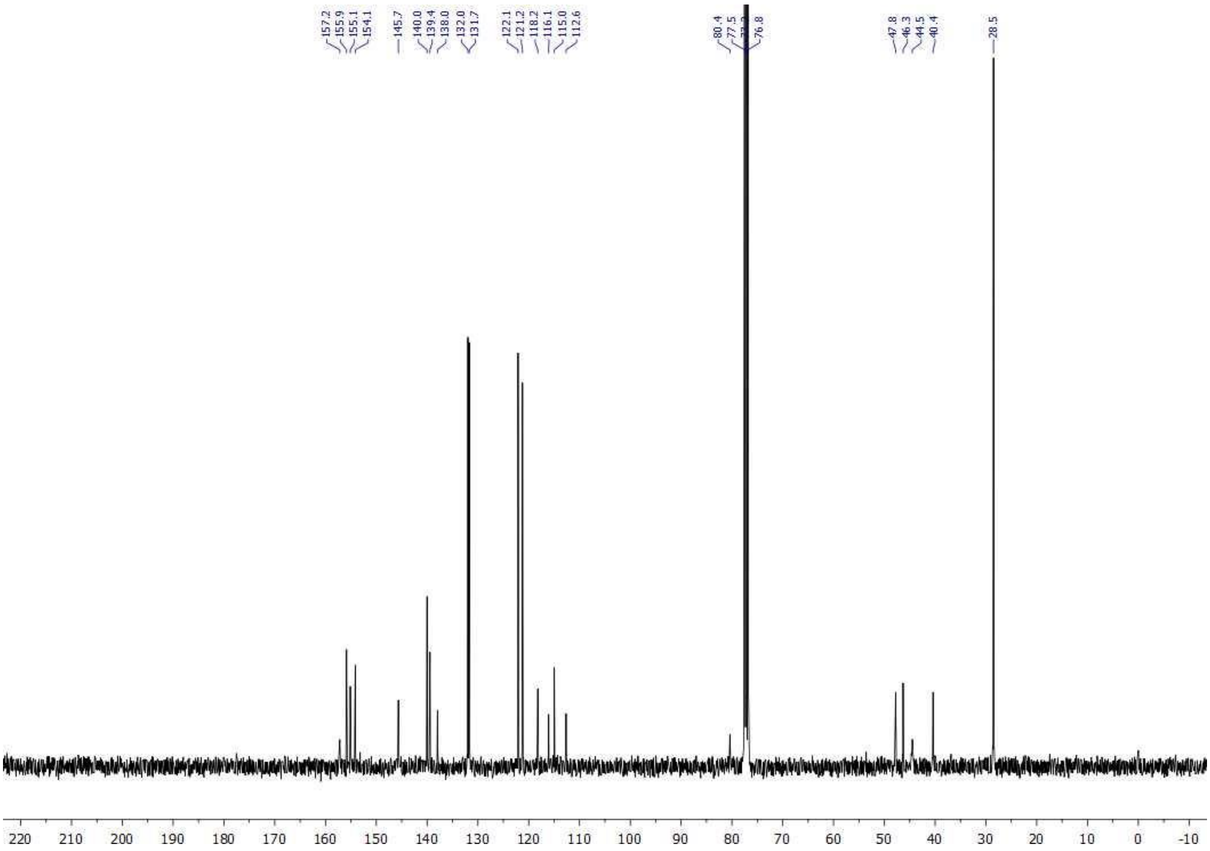

Figure S117 – ^{13}C NMR spectrum of 3h-1 (101 MHz, $CDCl_3$).

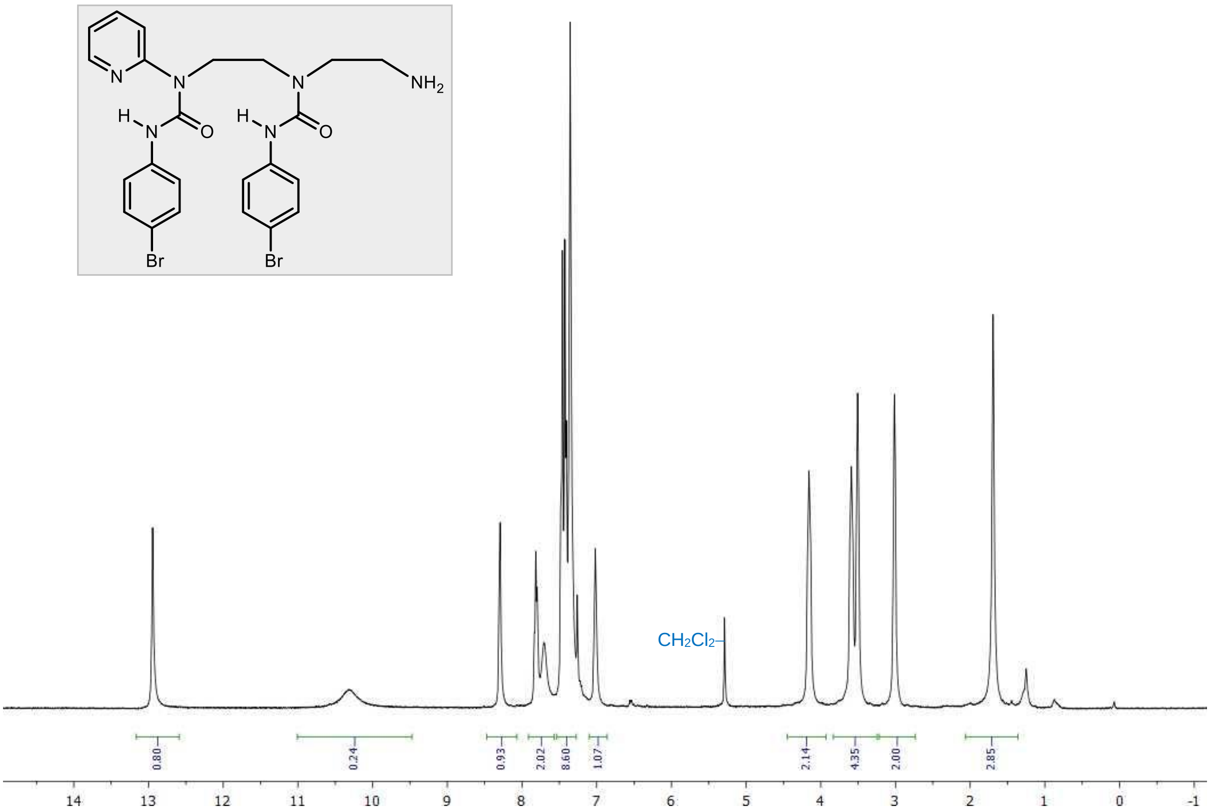

Figure S118 – ^{1}H NMR spectrum of 3h-2 (400 MHz, $CDCl_3$).

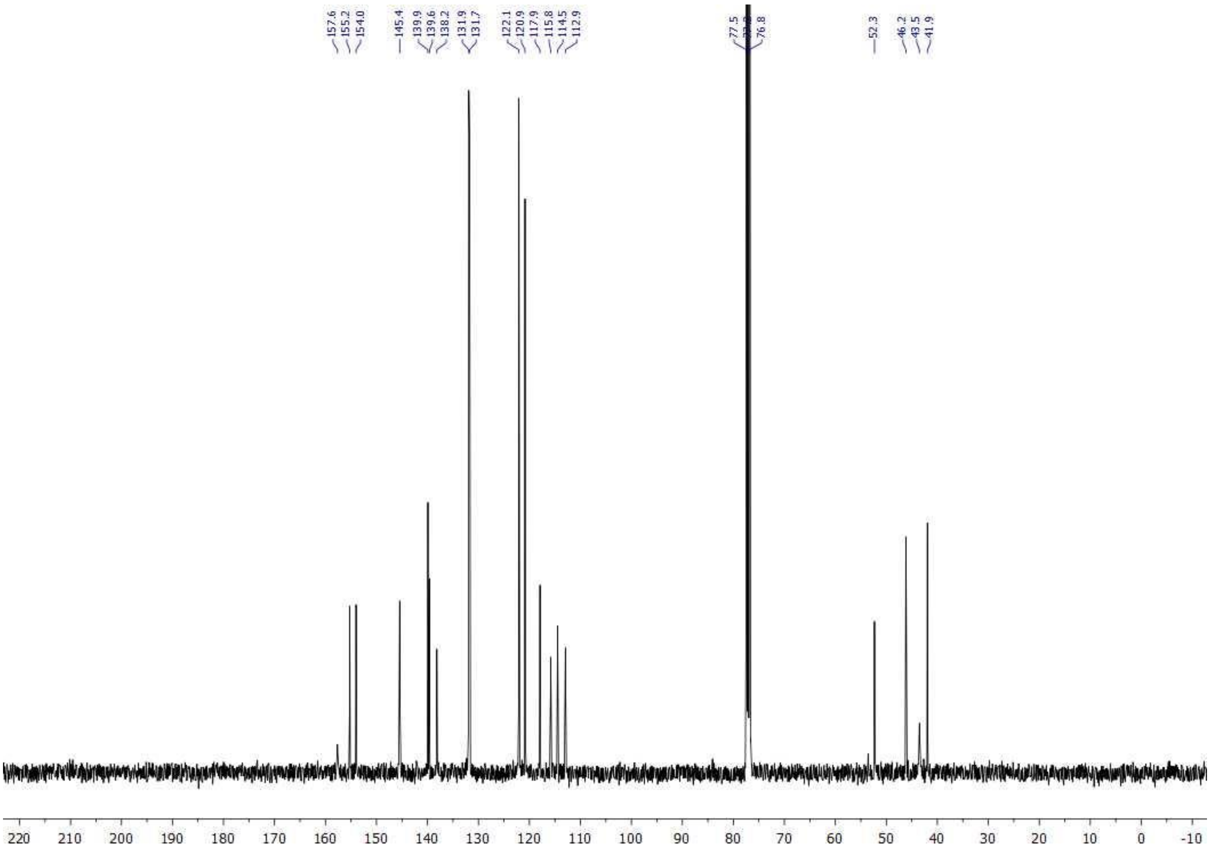

Figure S119 – ^{13}C NMR spectrum of 3h-2 (101 MHz, $CDCl_3$).

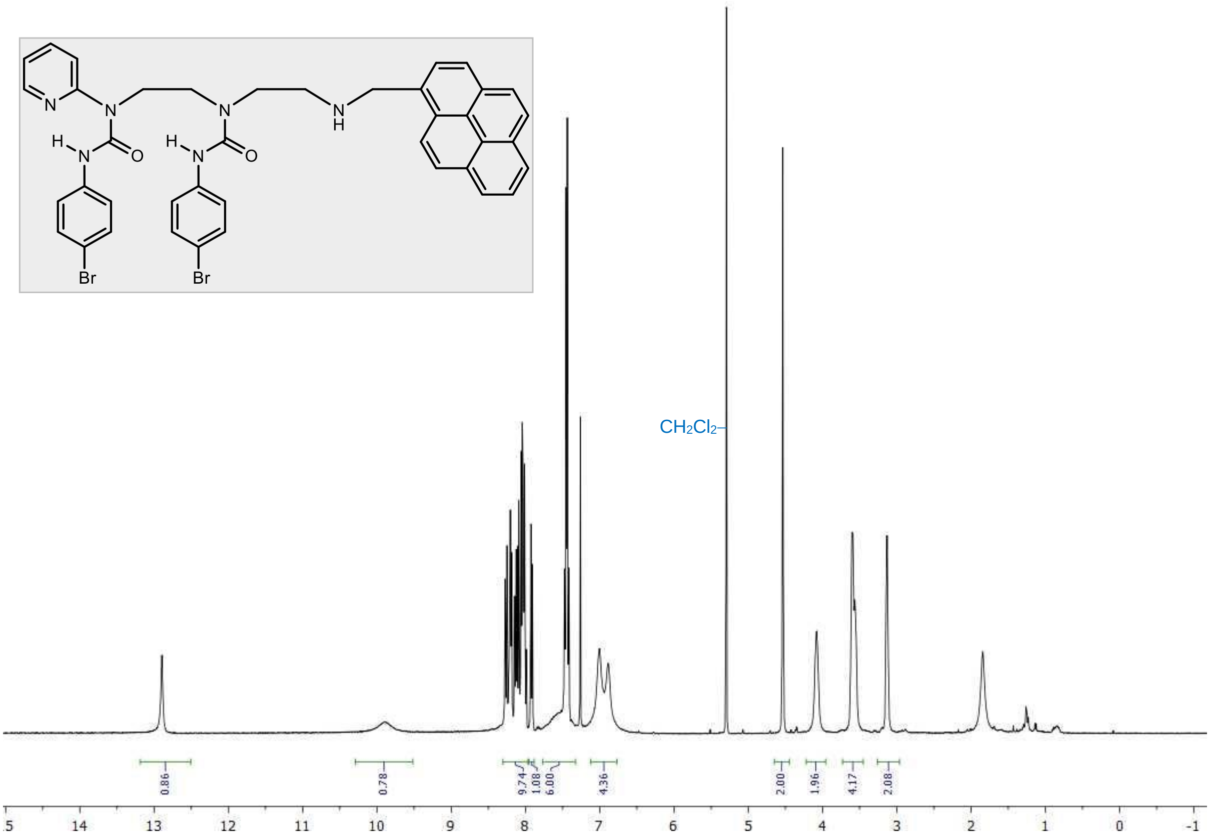

Figure S120 – ^{1}H NMR spectrum of 3h-3 (400 MHz, $CDCl_3$).

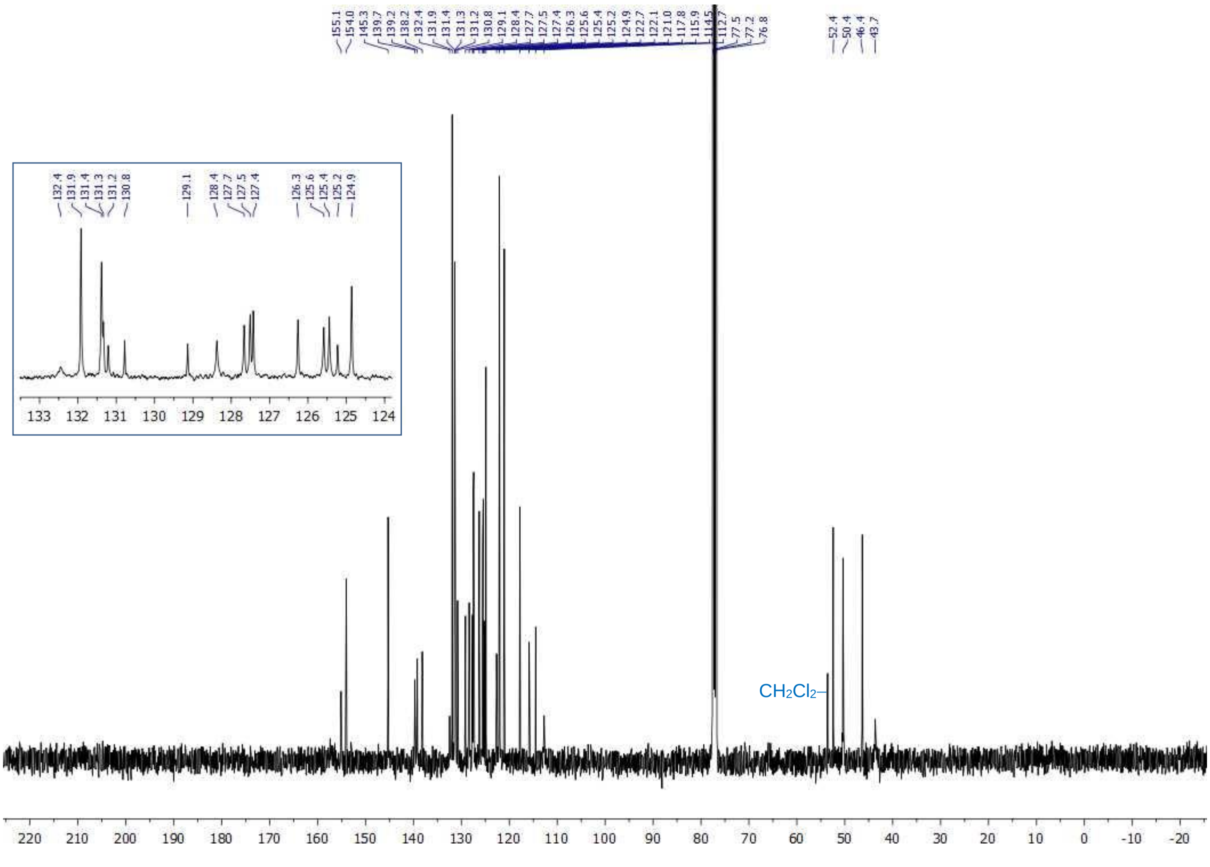

Figure S121 – ^{13}C NMR spectrum of 3h-3 (101 MHz, $CDCl_3$).

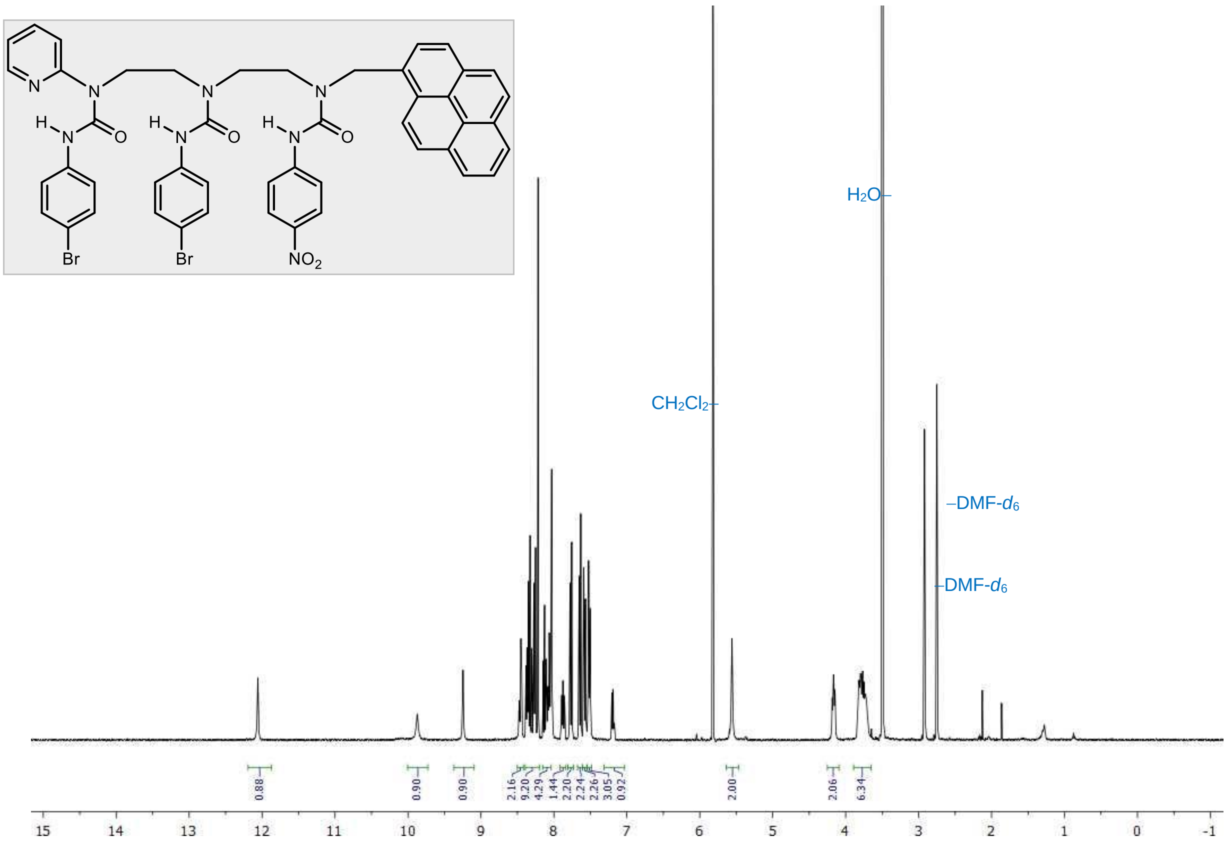

Figure S122 – ^{1}H NMR spectrum of 3h (400 MHz, DMF-d_7).

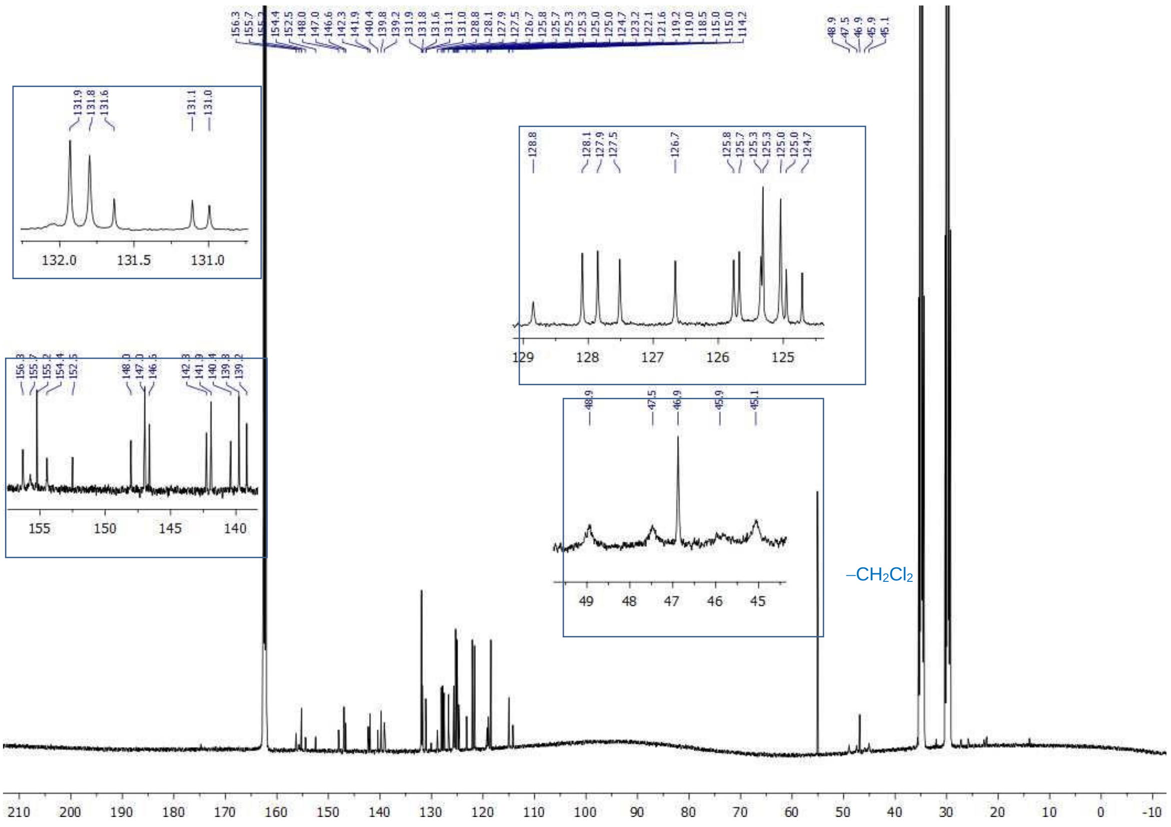

Figure S123 – ^{13}C NMR spectrum of 3h (101 MHz, DMF-d_7).

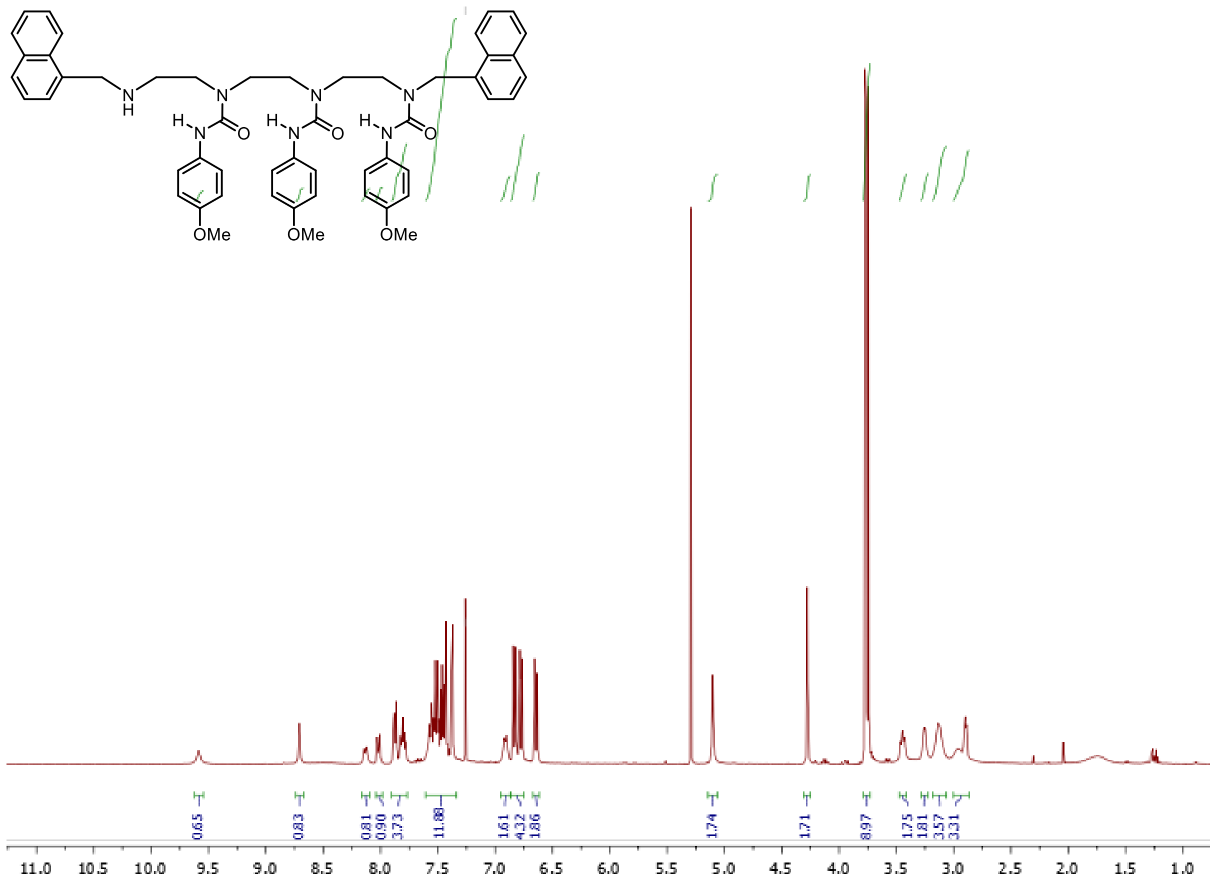

Figure S124 – ^{1}H NMR spectrum of 5a-1 (400 MHz, $CDCl_3$).

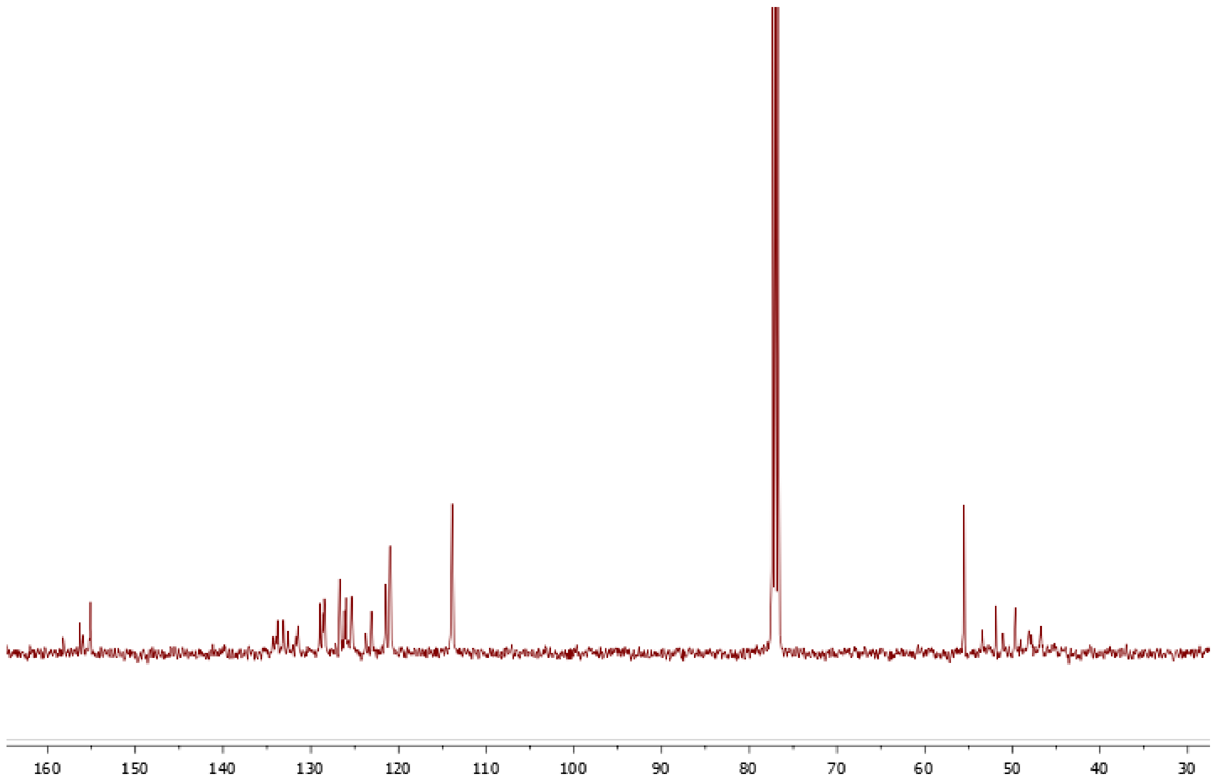

Figure S125 – ^{13}C NMR spectrum of 5a-1 (101 MHz, $CDCl_3$).

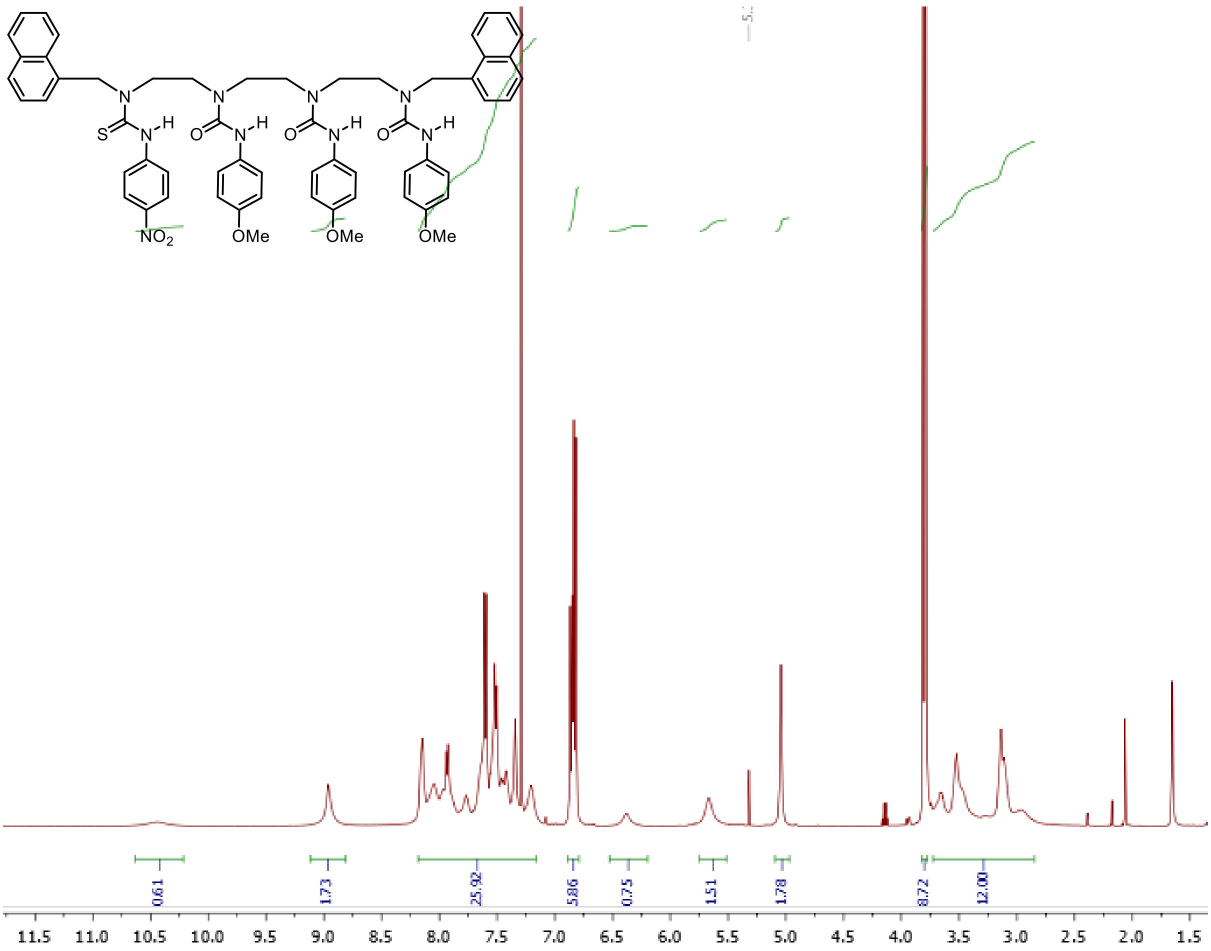

Figure S126 – ^{1}H NMR spectrum of 5a (500 MHz, $CDCl_3$).

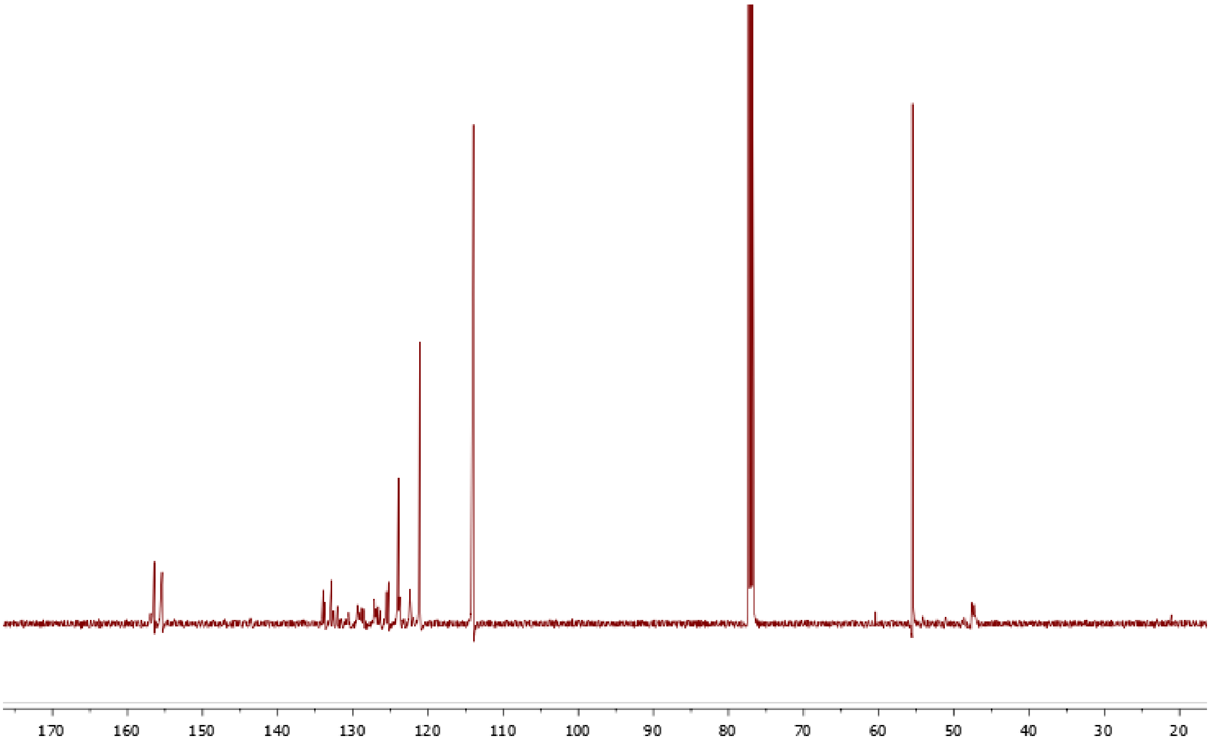

Figure S127 – ^{13}C NMR spectrum of 5a (126 MHz, $CDCl_3$).

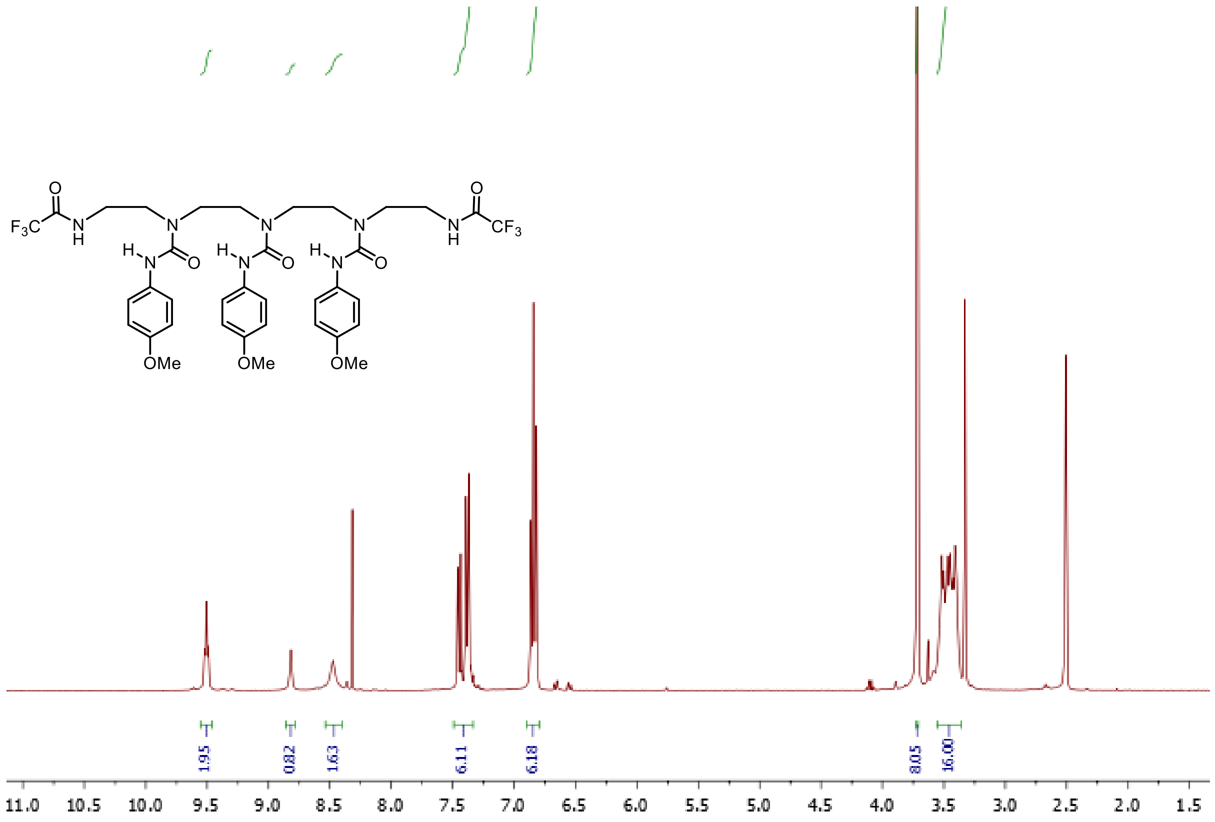

Figure S128 – ^{1}H NMR spectrum of 5b-1 (400 MHz, $CDCl_3$).

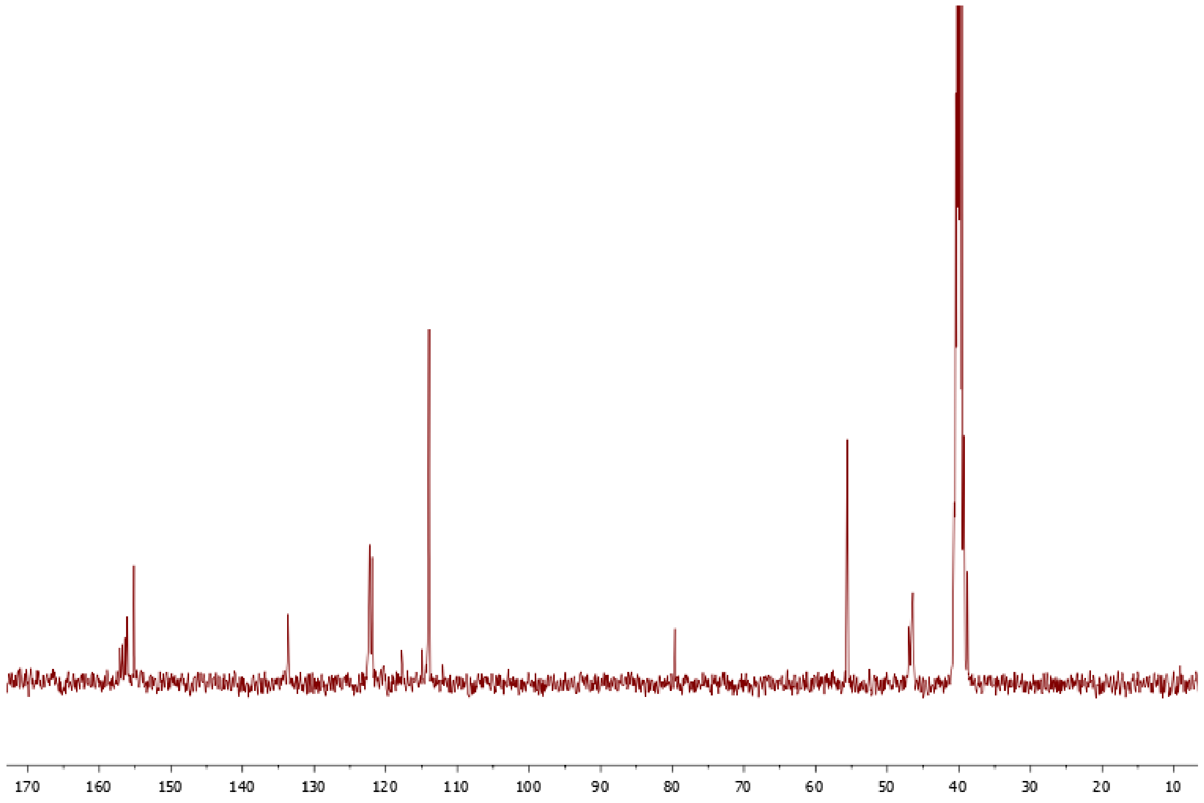

Figure S129 – ^{13}C NMR spectrum of 5b-1 (101 MHz, $CDCl_3$).

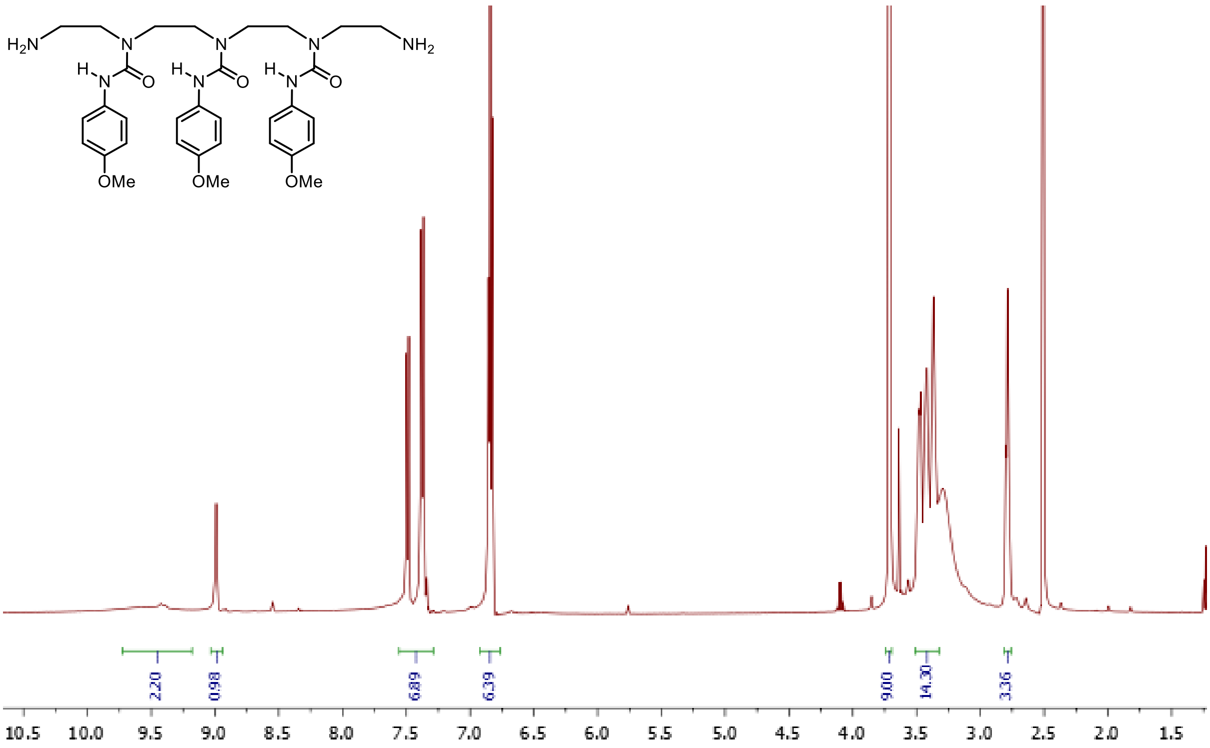

Figure S130 – ^{1}H NMR spectrum of 5b-2 (500 MHz, $(CD_3)_2SO$).

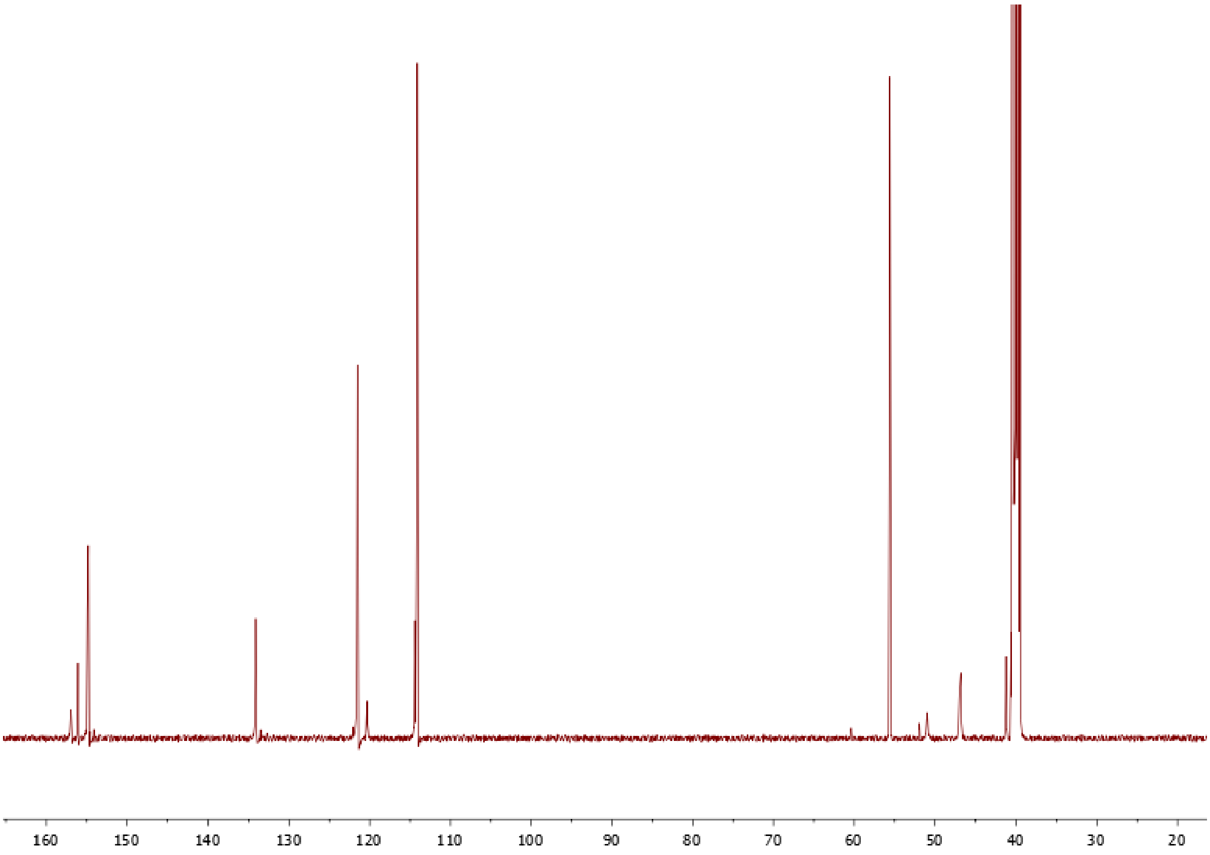

Figure S131 – ^{13}C NMR spectrum of 5b-2 (126 MHz, $(CD_3)_2SO$).

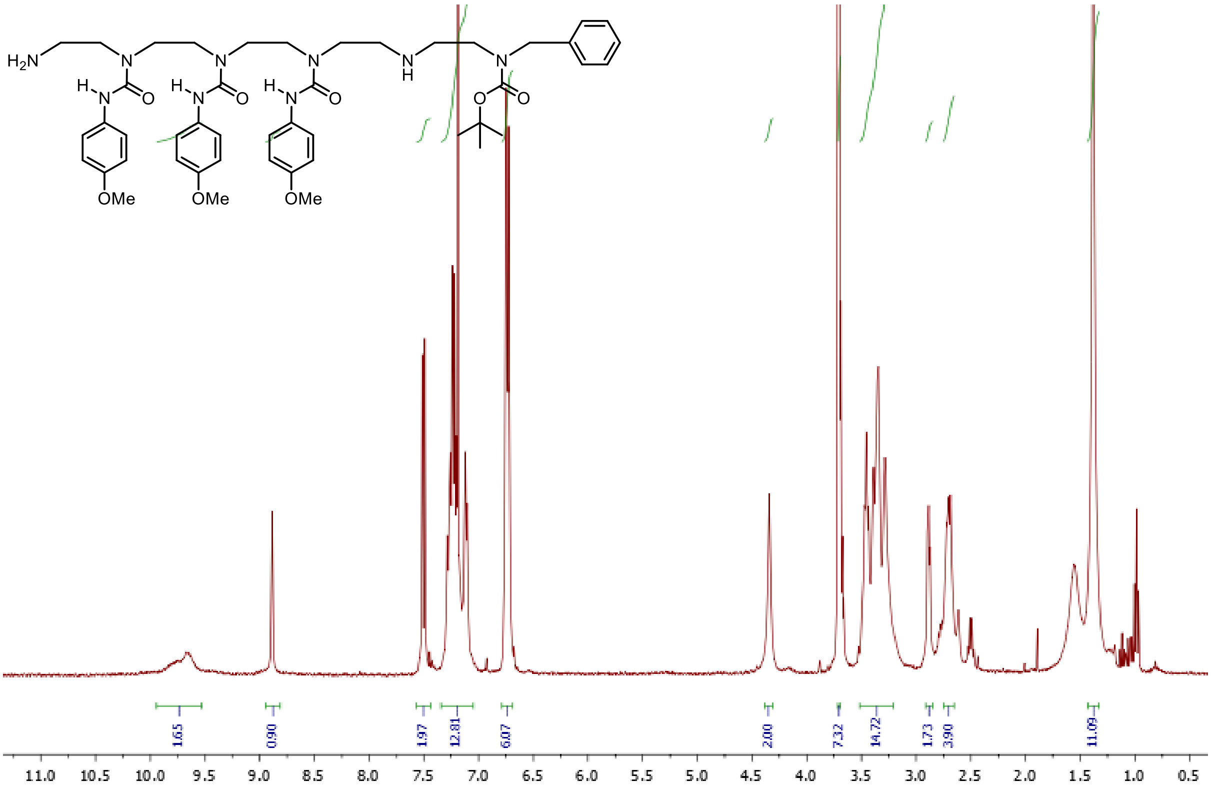

Figure S132 – ^{1}H NMR spectrum of 5b-5 (400 MHz, CDCl$_3$).

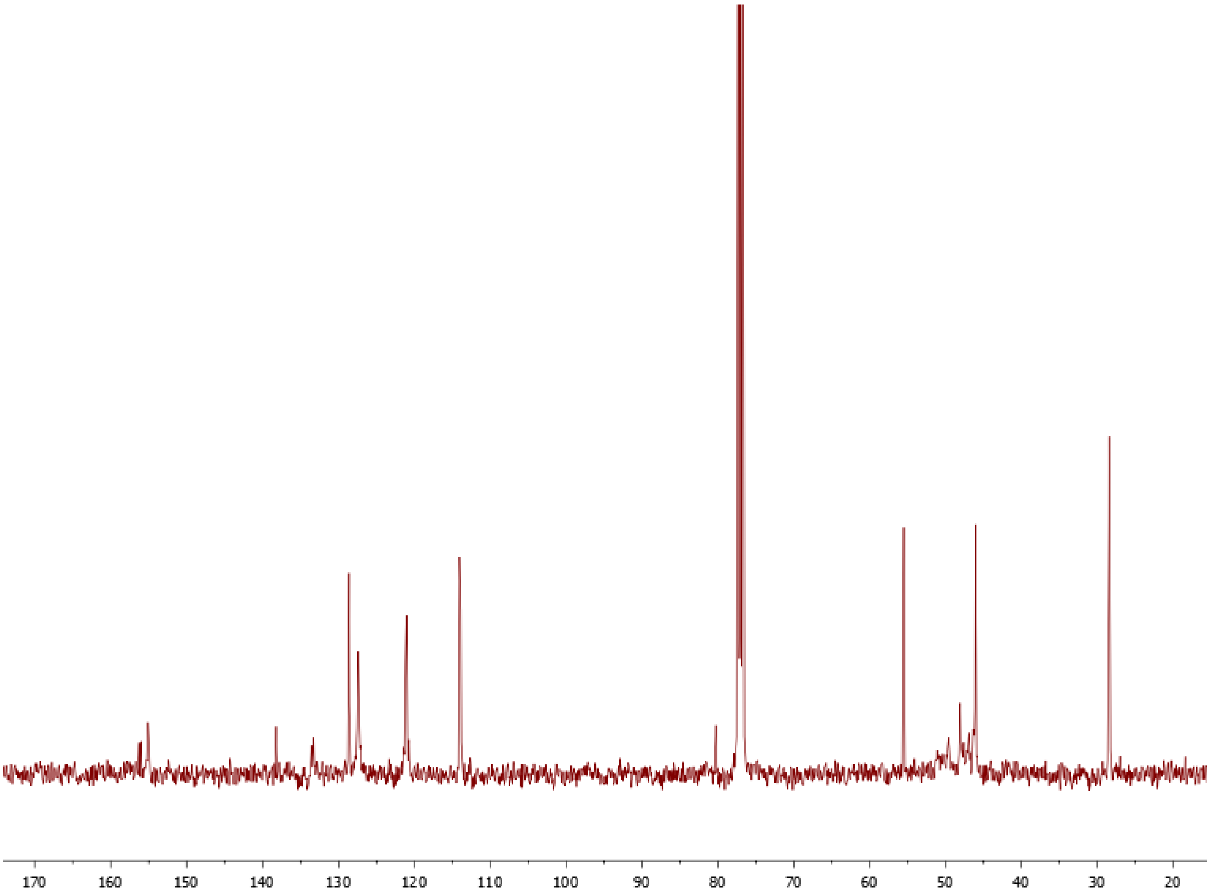

Figure S133 – ^{13}C NMR spectrum of 5b-5 (101 MHz, CDCl$_3$).

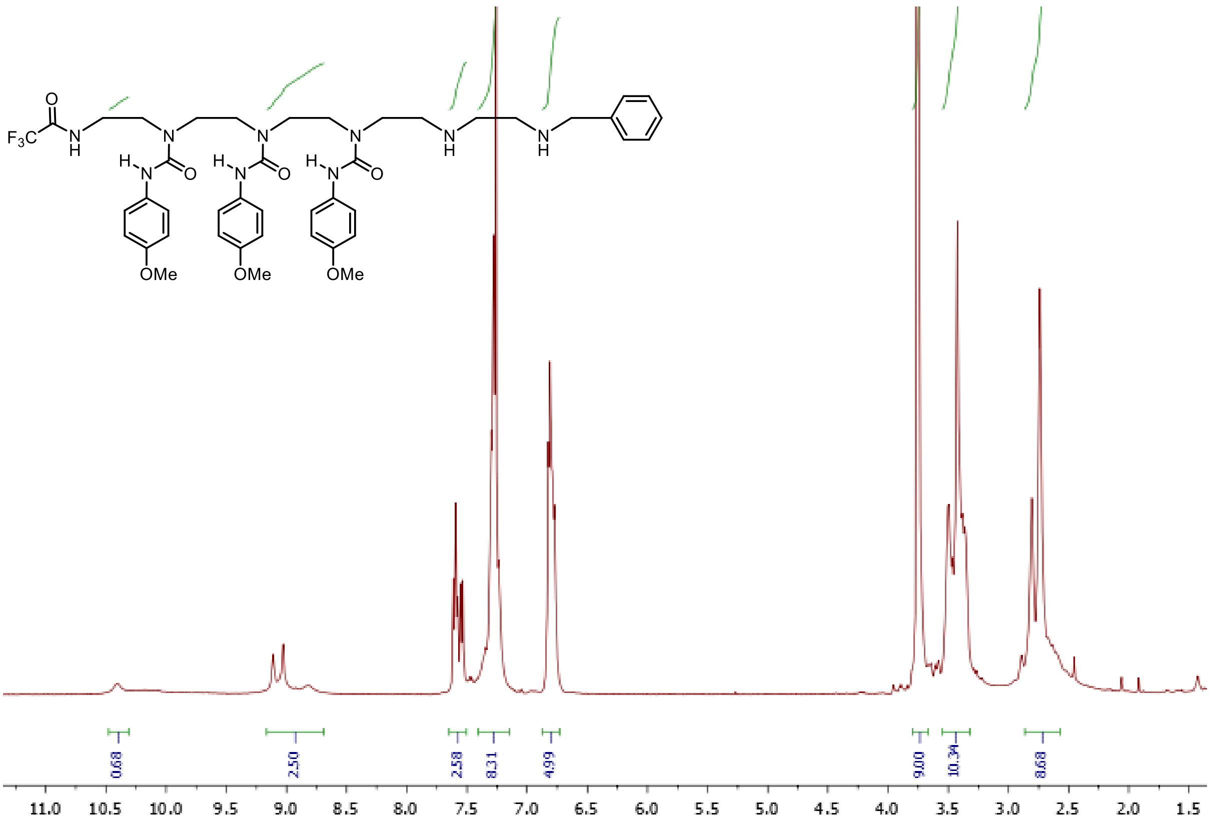

Figure S134 – ^{1}H NMR spectrum of 5b-6 (400 MHz, $CDCl_3$).

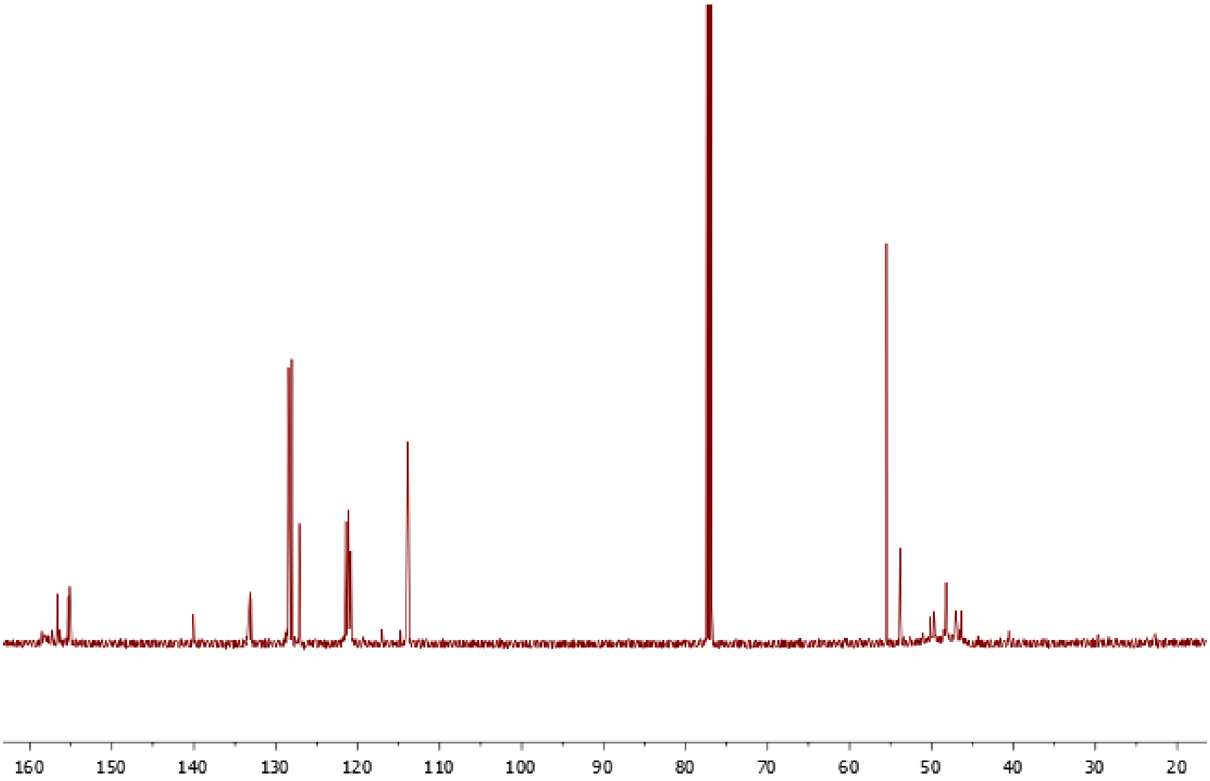

Figure S135 – ^{13}C NMR spectrum of 5b-6 (101 MHz, $CDCl_3$).

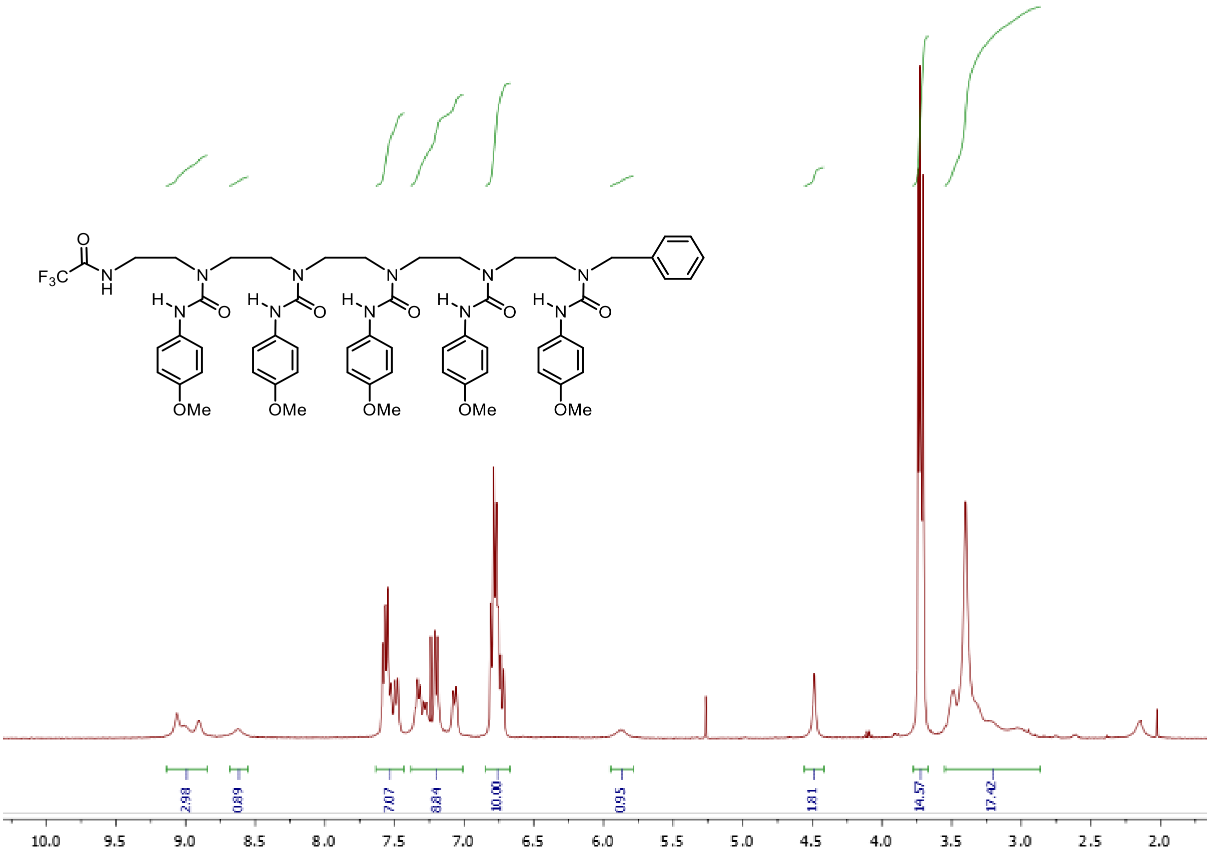

Figure S136 – ^{1}H NMR spectrum of 5b-7 (400 MHz, $CDCl_3$).

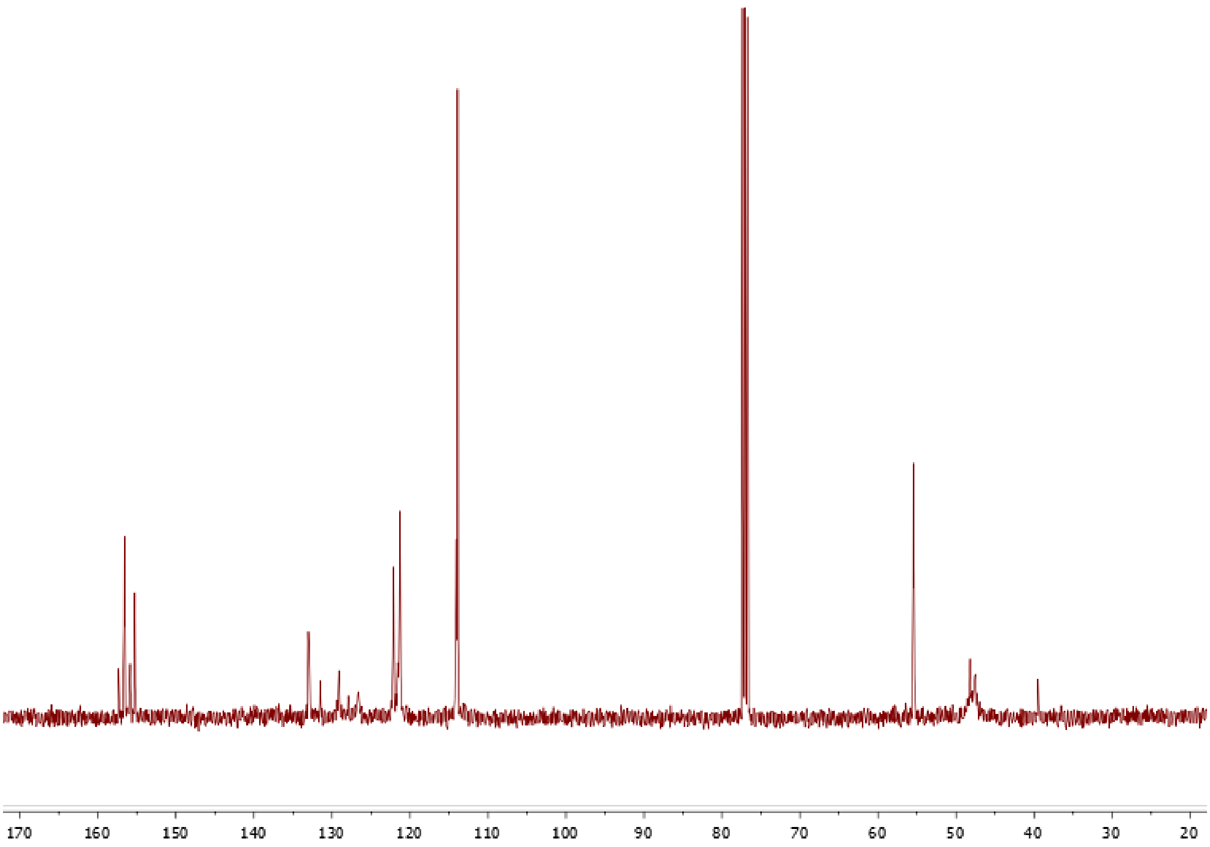

Figure S137 – ^{13}C NMR spectrum of 5b-7 (101 MHz, $CDCl_3$).

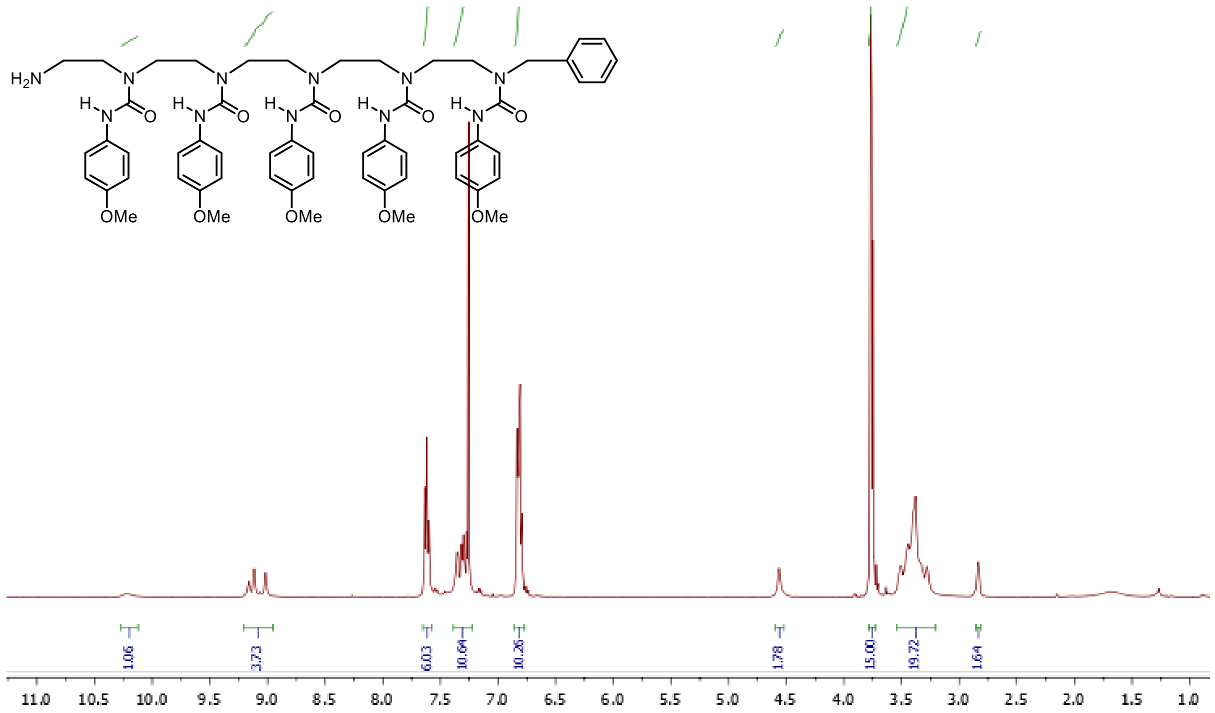

Figure S138 – ^{1}H NMR spectrum of 5b-8 (500 MHz, $CDCl_3$).

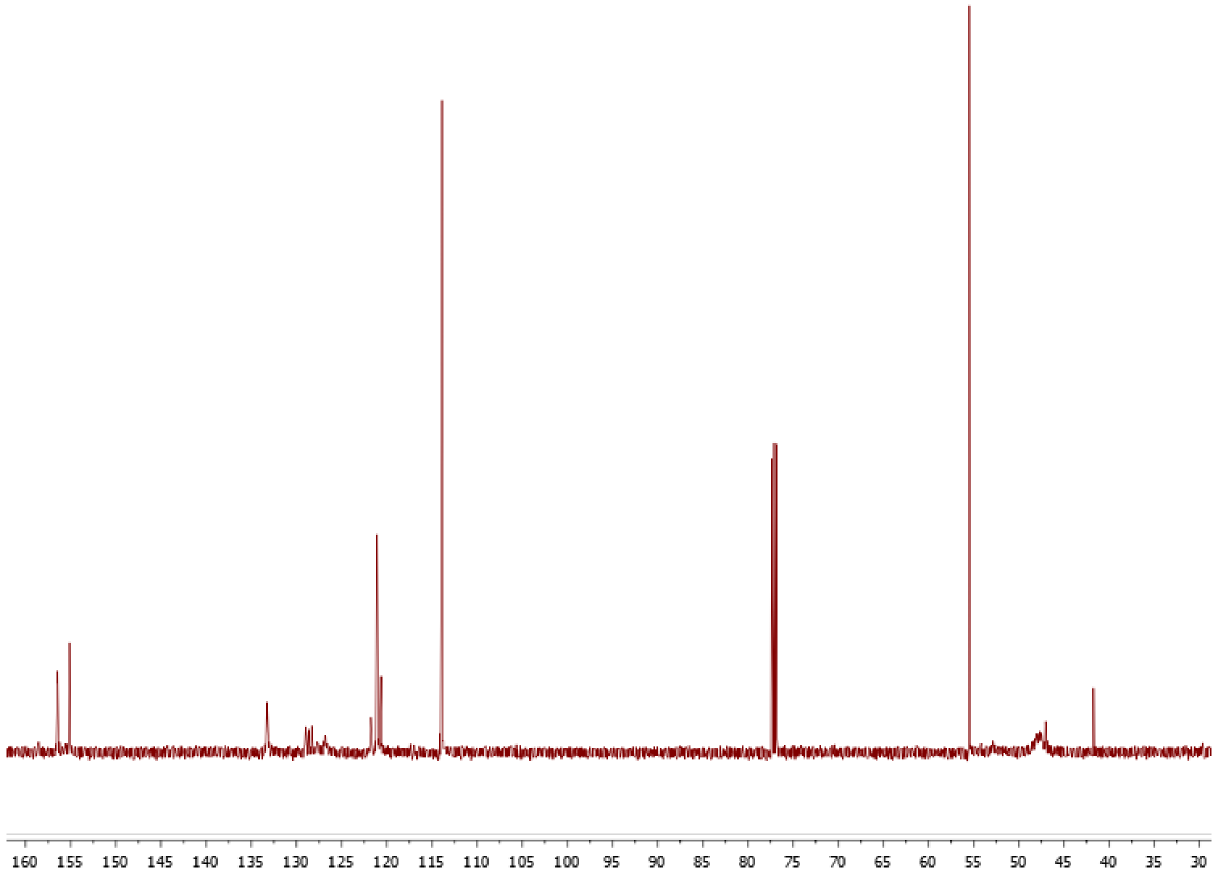

Figure S139 – ^{13}C NMR spectrum of 5b-8 (126 MHz, $CDCl_3$).

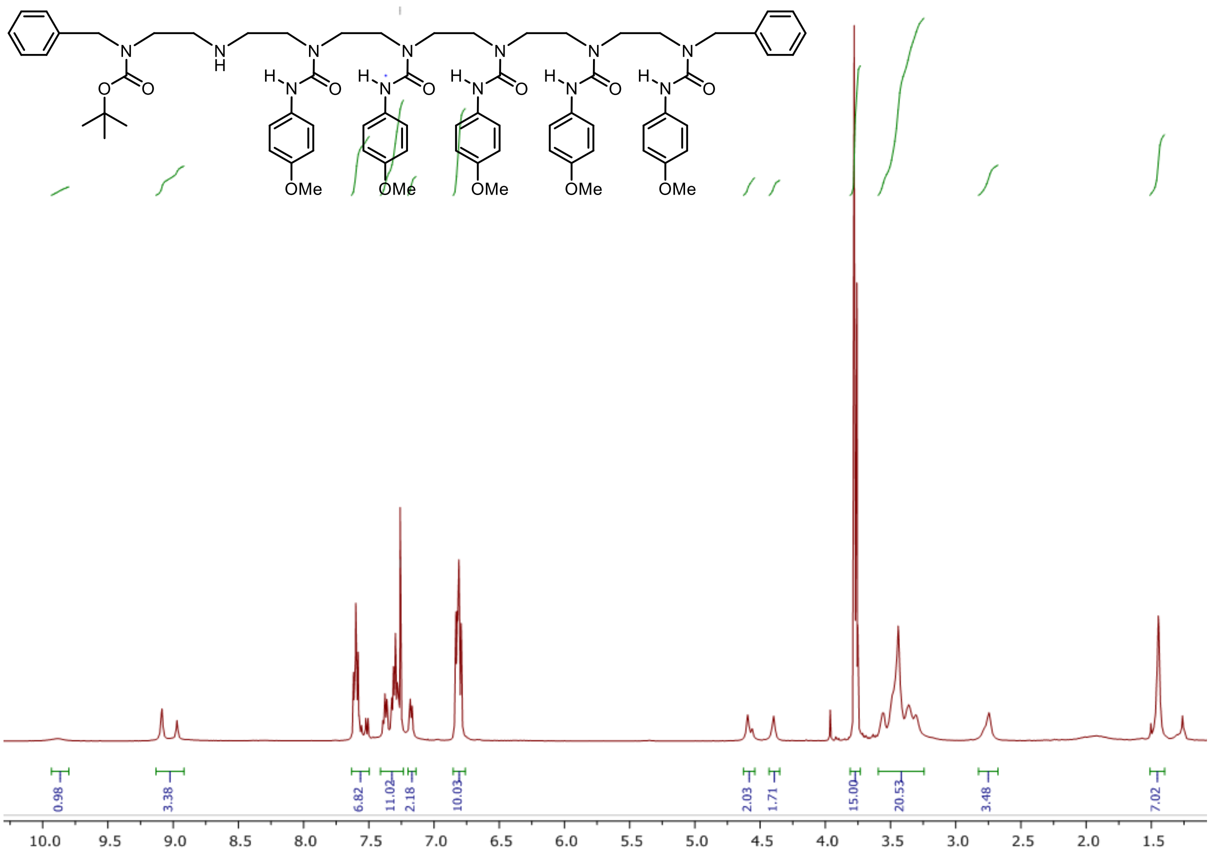

Figure S140 – ^{1}H NMR spectrum of 5b-9 (500 MHz, $CDCl_3$).

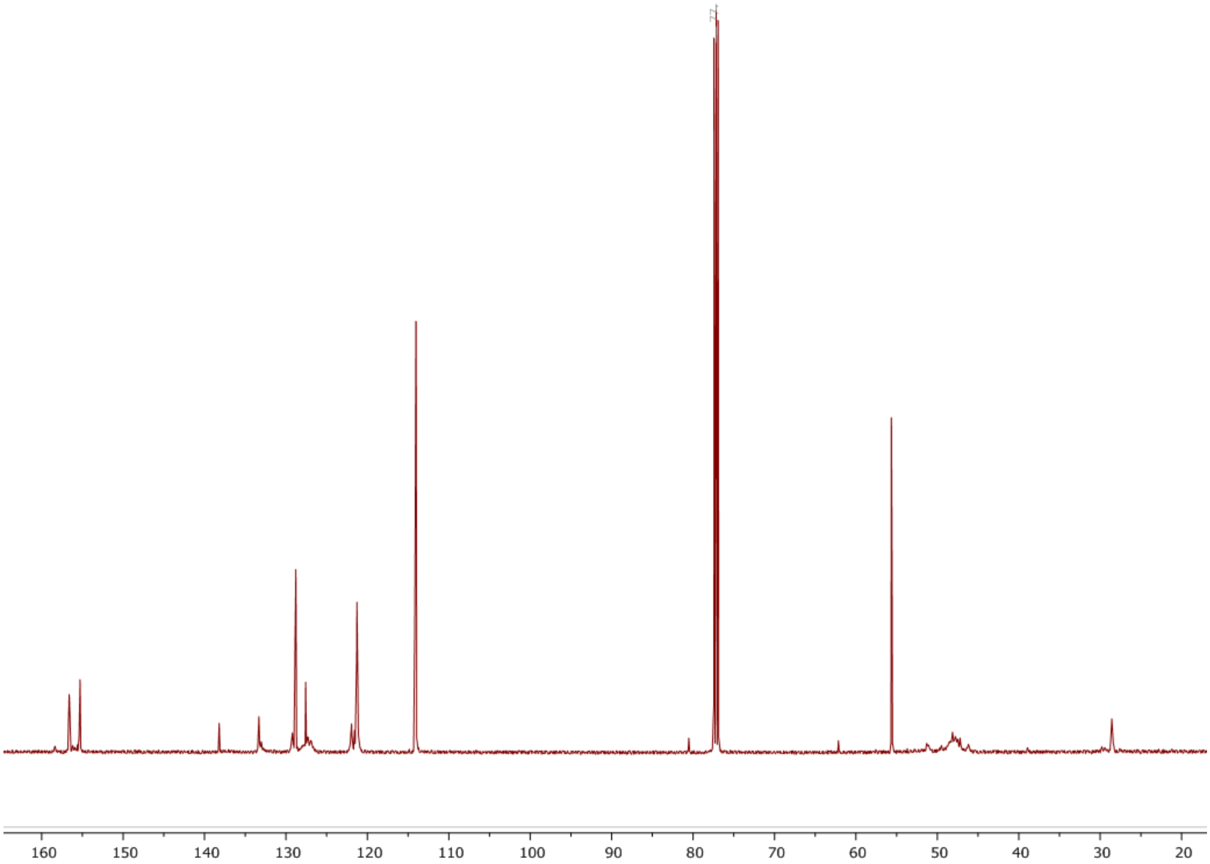

Figure S141 – ^{13}C NMR spectrum of 5b-9 (126 MHz, $CDCl_3$).

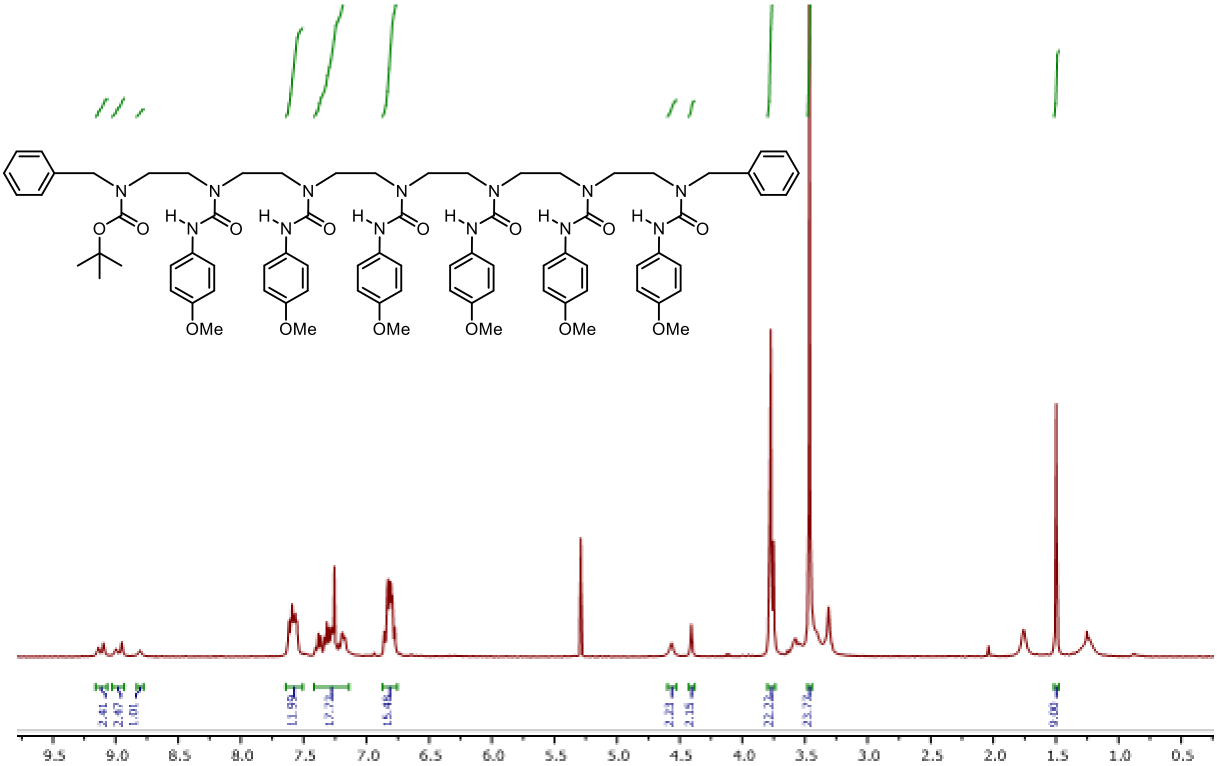

Figure S142 – ¹H NMR spectrum of 5b-10 (500 MHz, $CDCl_3$).

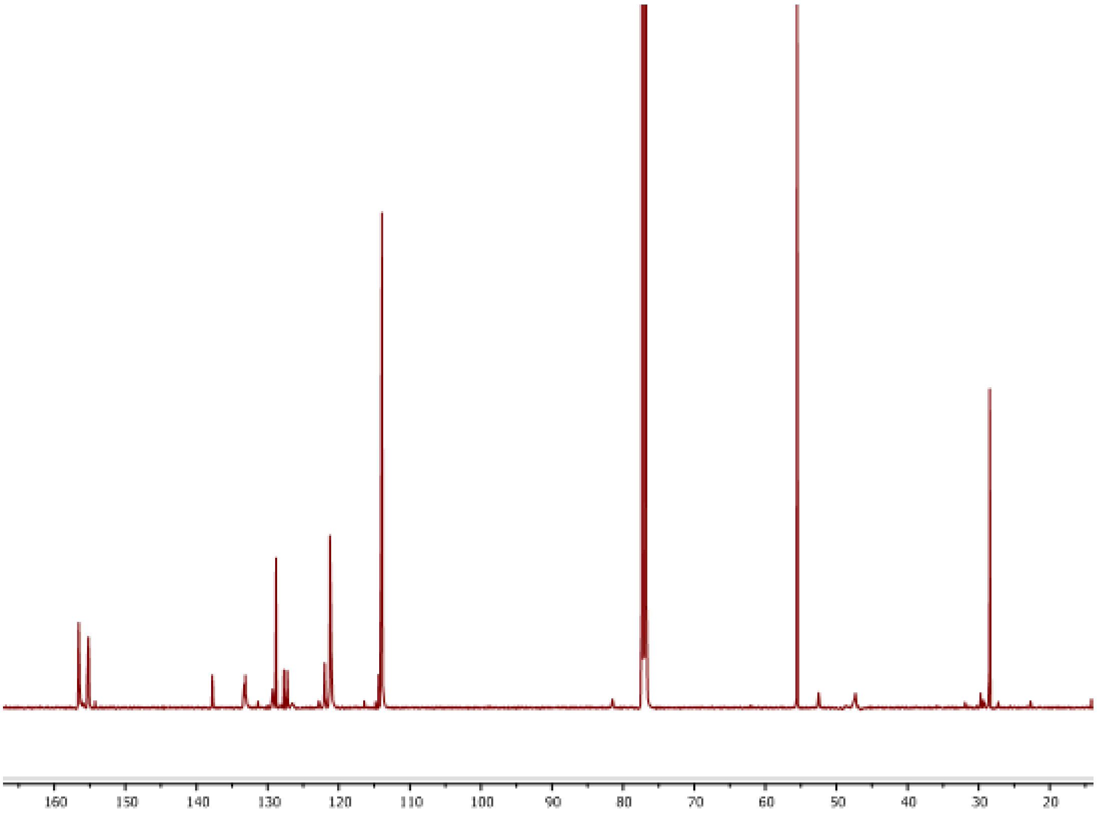

Figure S143 – ¹³C NMR spectrum of 5b-10 (126 MHz, $CDCl_3$).

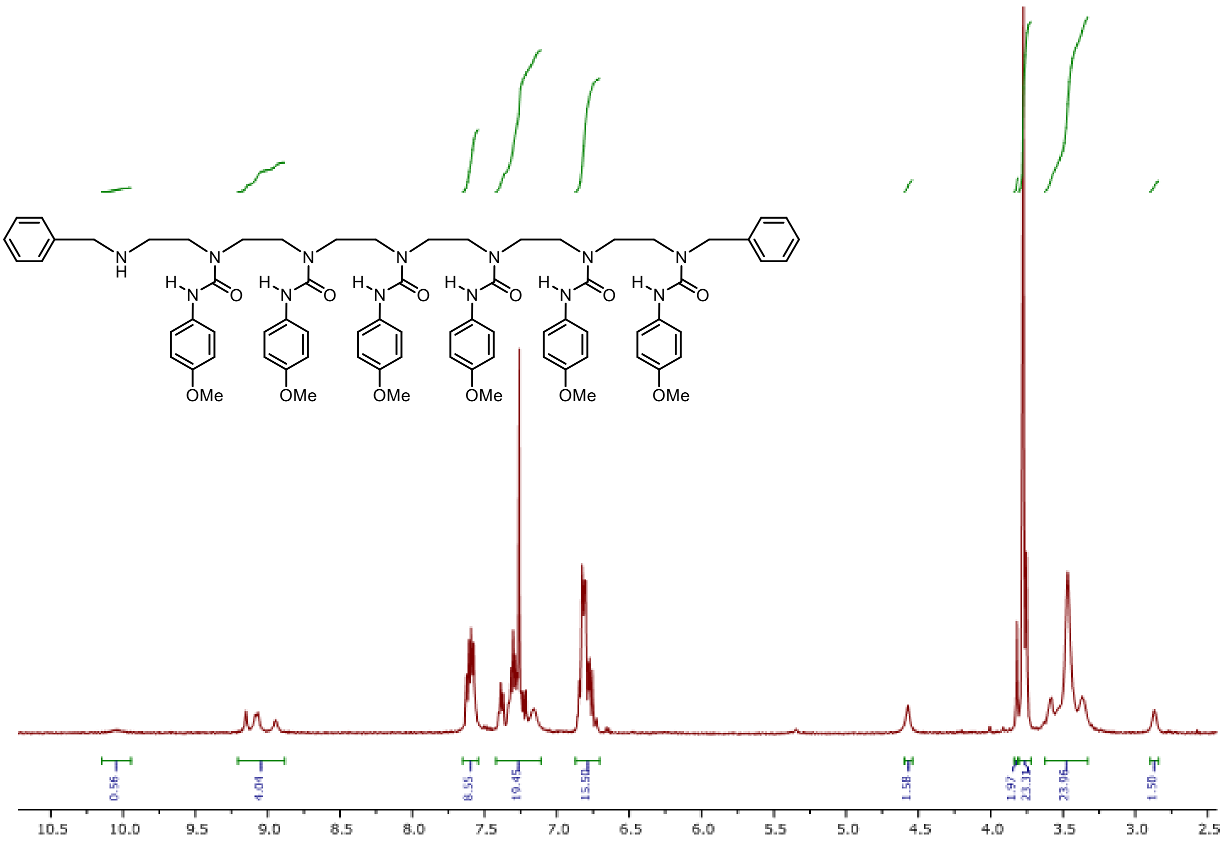

Figure S144 – ^{1}H NMR spectrum of 5b-11 (400 MHz, $CDCl_3$).

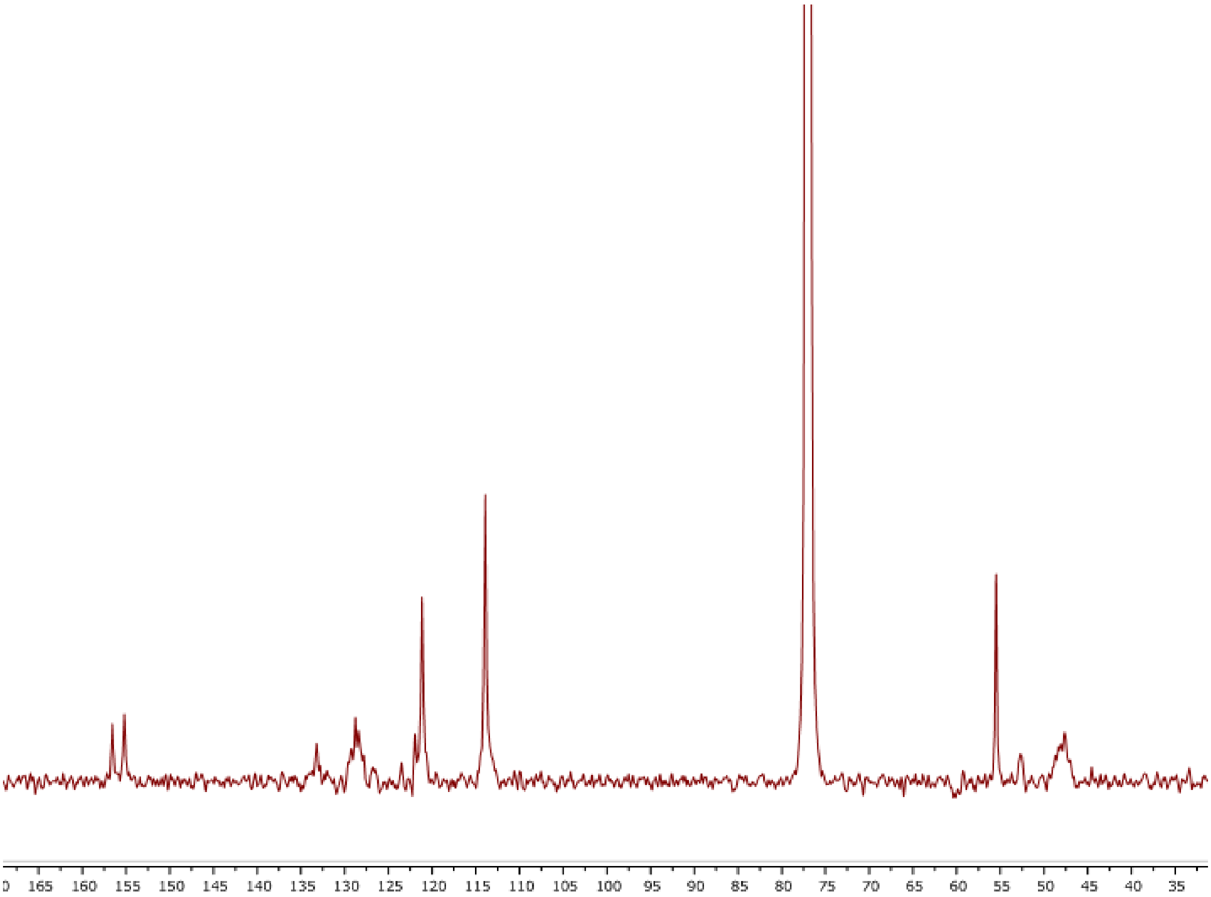

Figure S145 – ^{13}C NMR spectrum of 5b-11 (101 MHz, $CDCl_3$).

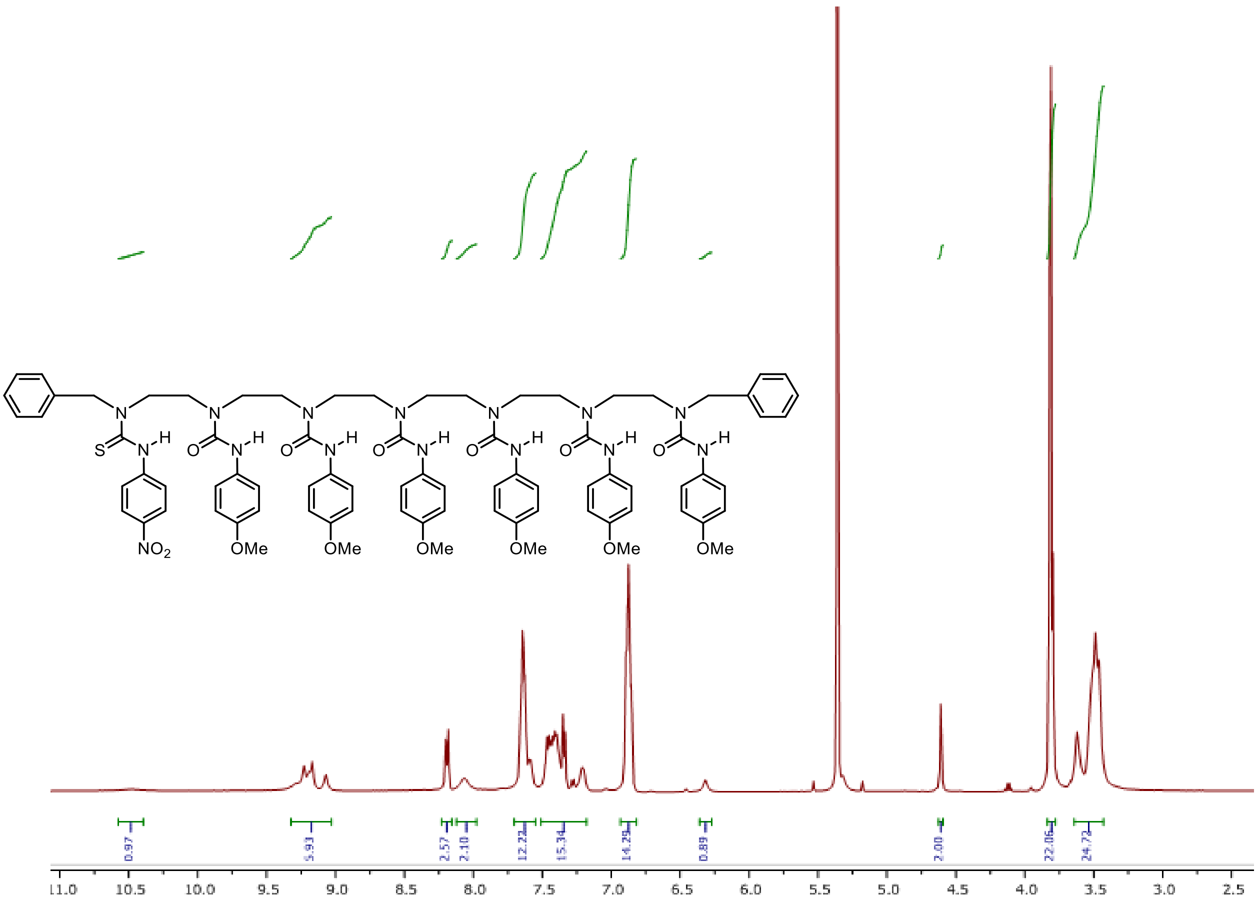

Figure S146 – ^1H NMR spectrum of 5b (500 MHz, CD_2Cl_2).

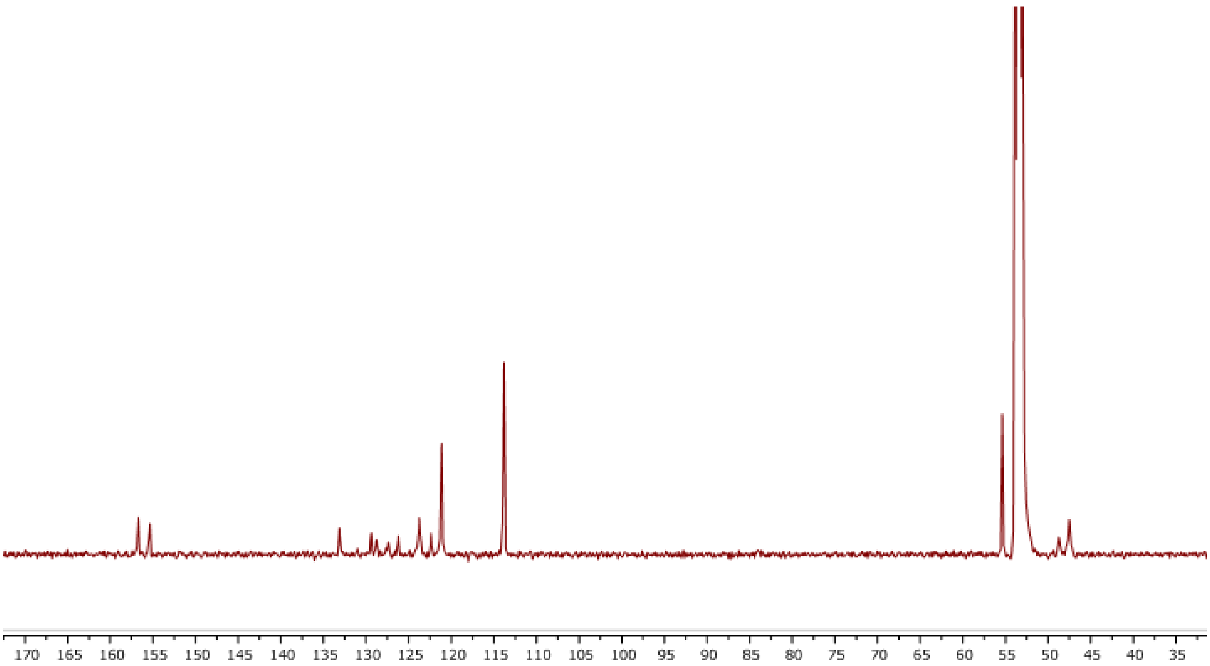

Figure S147 – ^{13}C NMR spectrum of 5b (126 MHz, CD_2Cl_2).

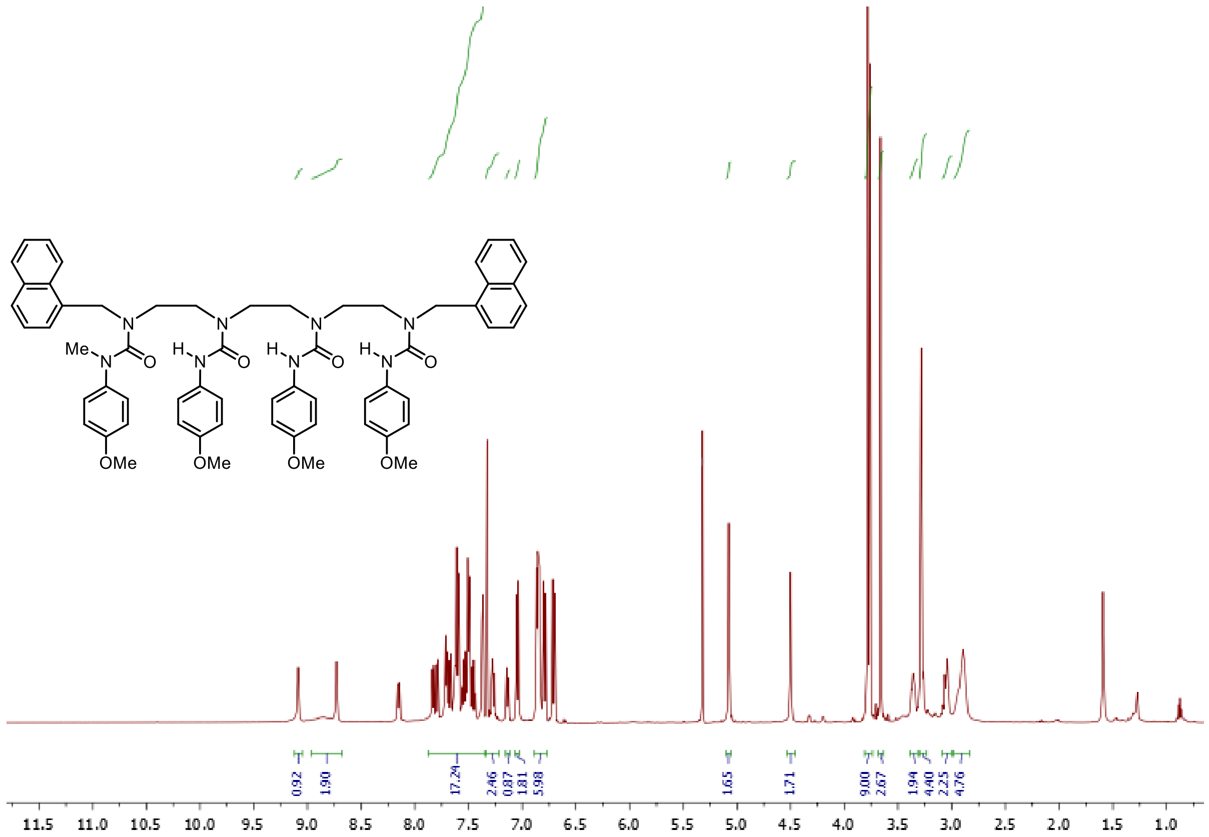

Figure S148 – ^{1}H NMR spectrum of 6a (500 MHz, CD_2Cl_2).

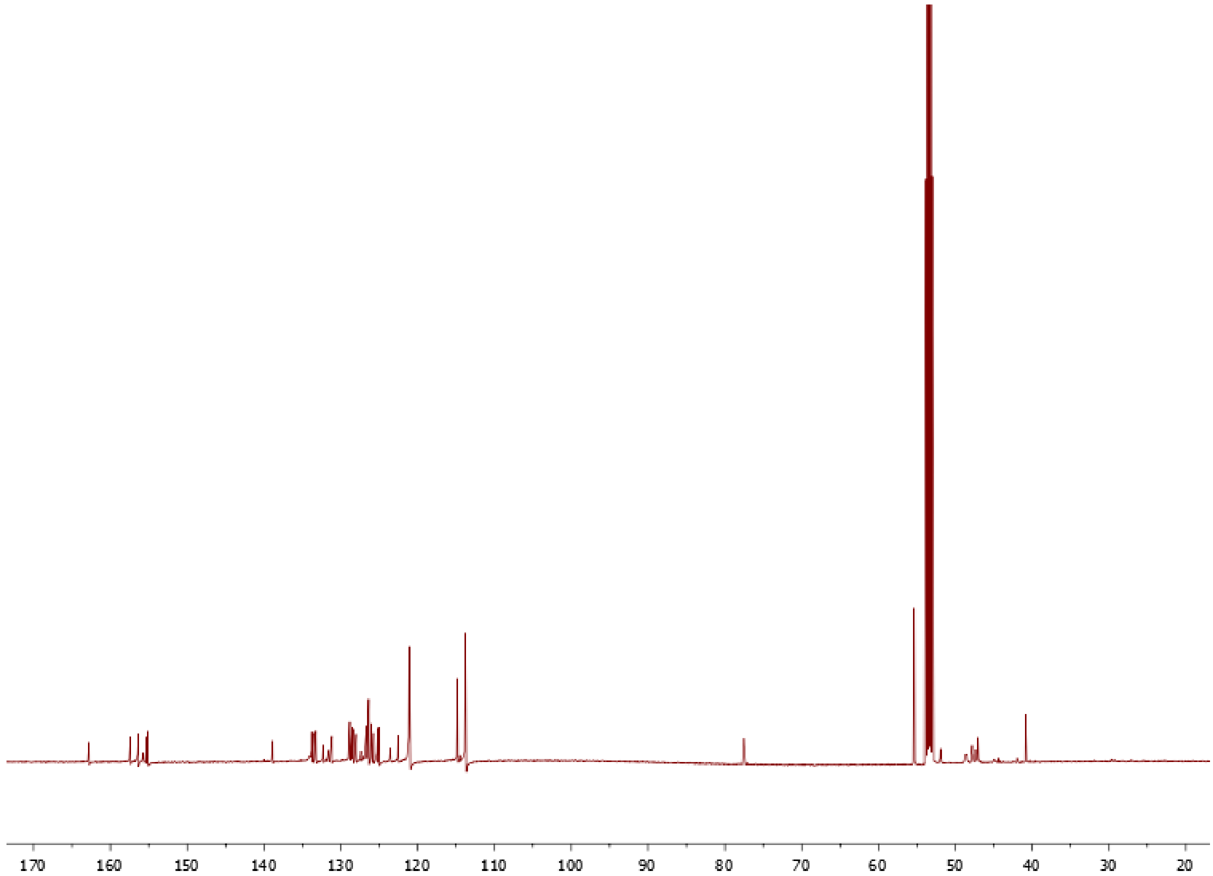

Figure S149 – ^{13}C NMR spectrum of 6a (126 MHz, CD_2Cl_2).

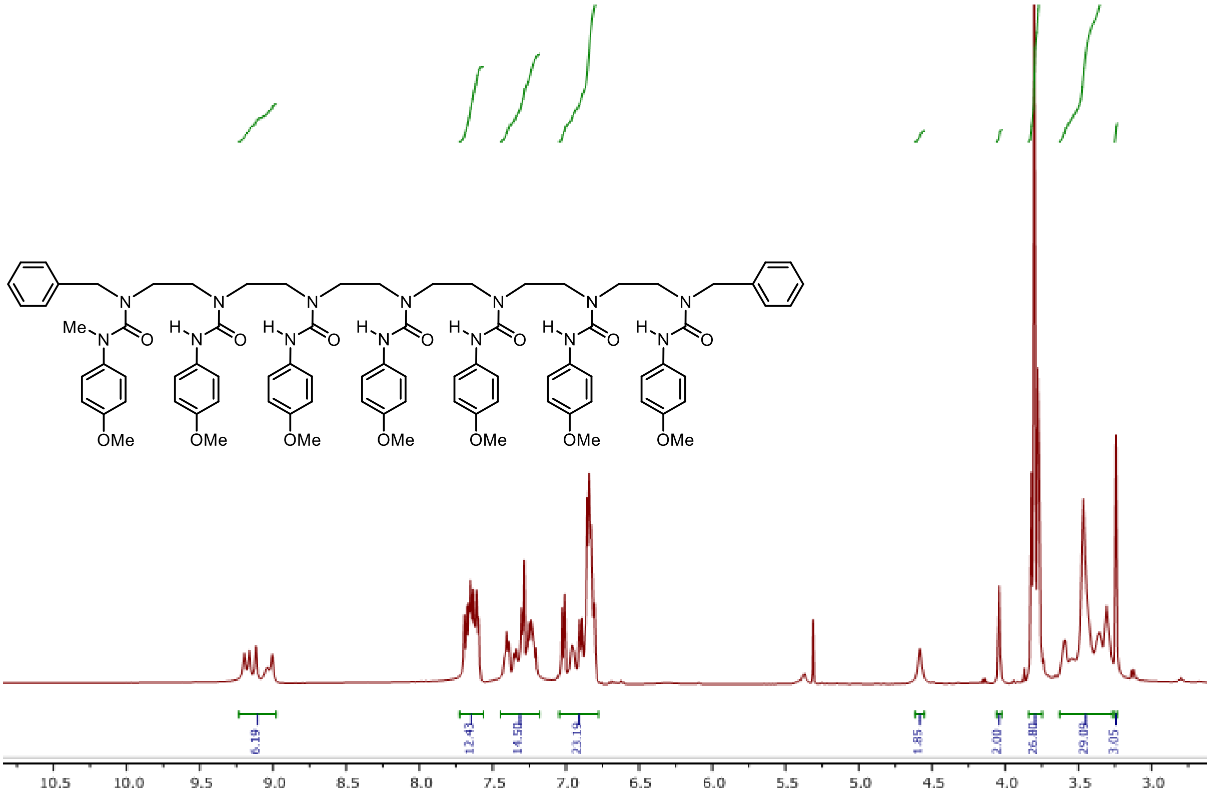

Figure S150 – ^{1}H NMR spectrum of 6b (500 MHz, $CDCl_3$).

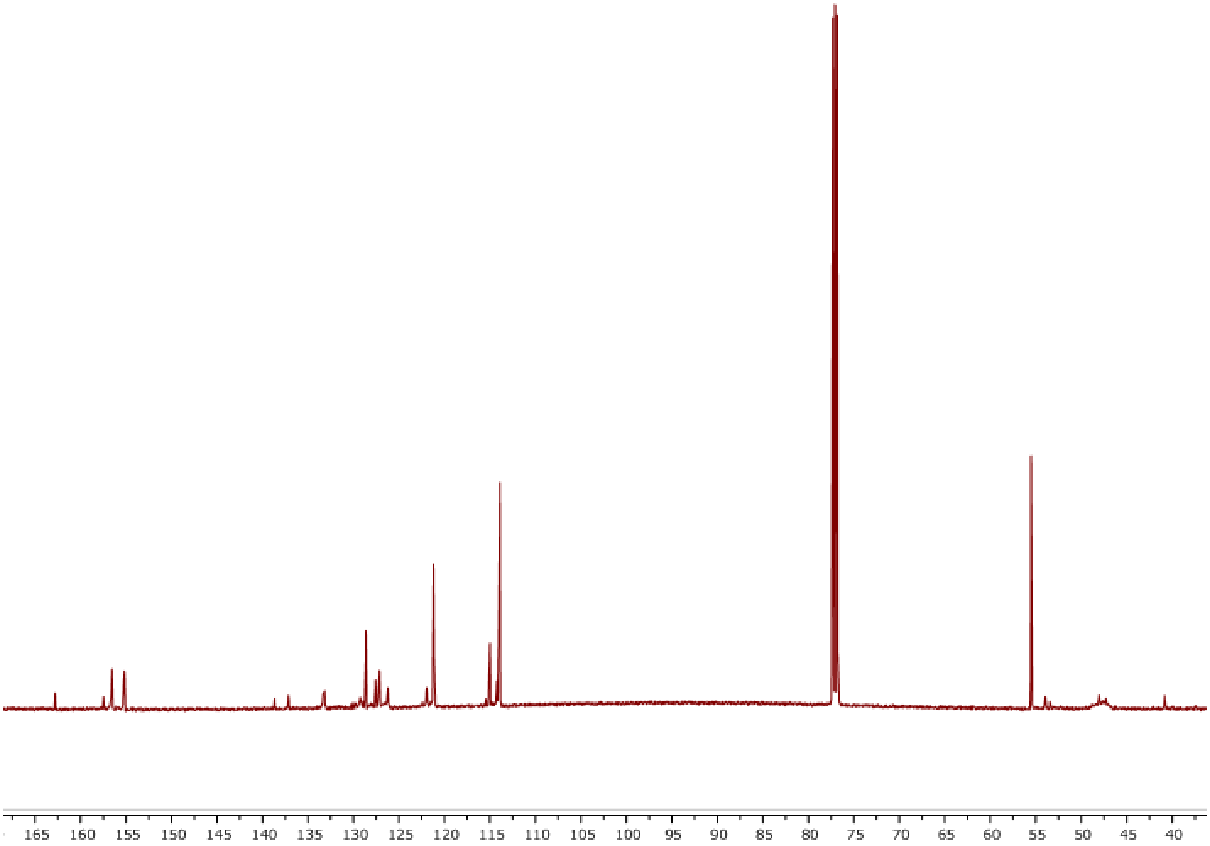

Figure S151 – ^{13}C NMR spectrum of 6b (126 MHz, $CDCl_3$).

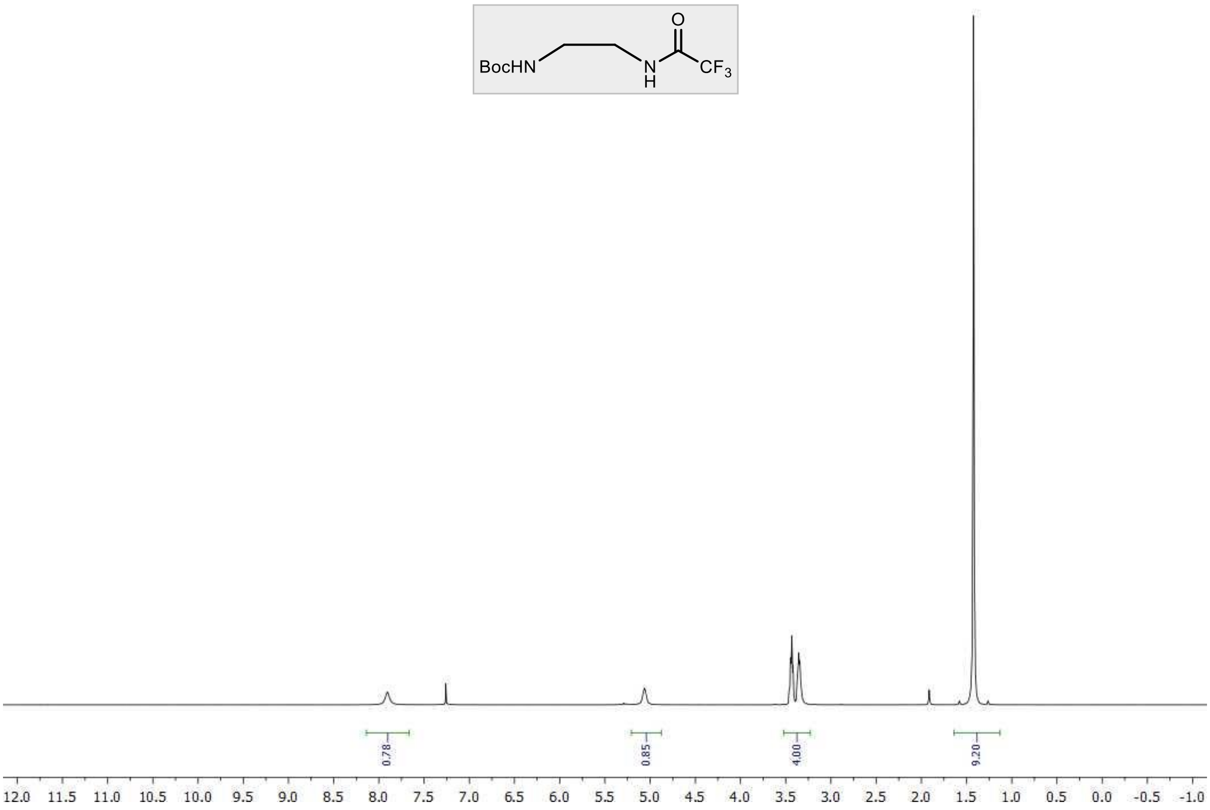

Figure S152 – ^{1}H NMR spectrum of 4b-1 (400 MHz, CDCl$_3$).

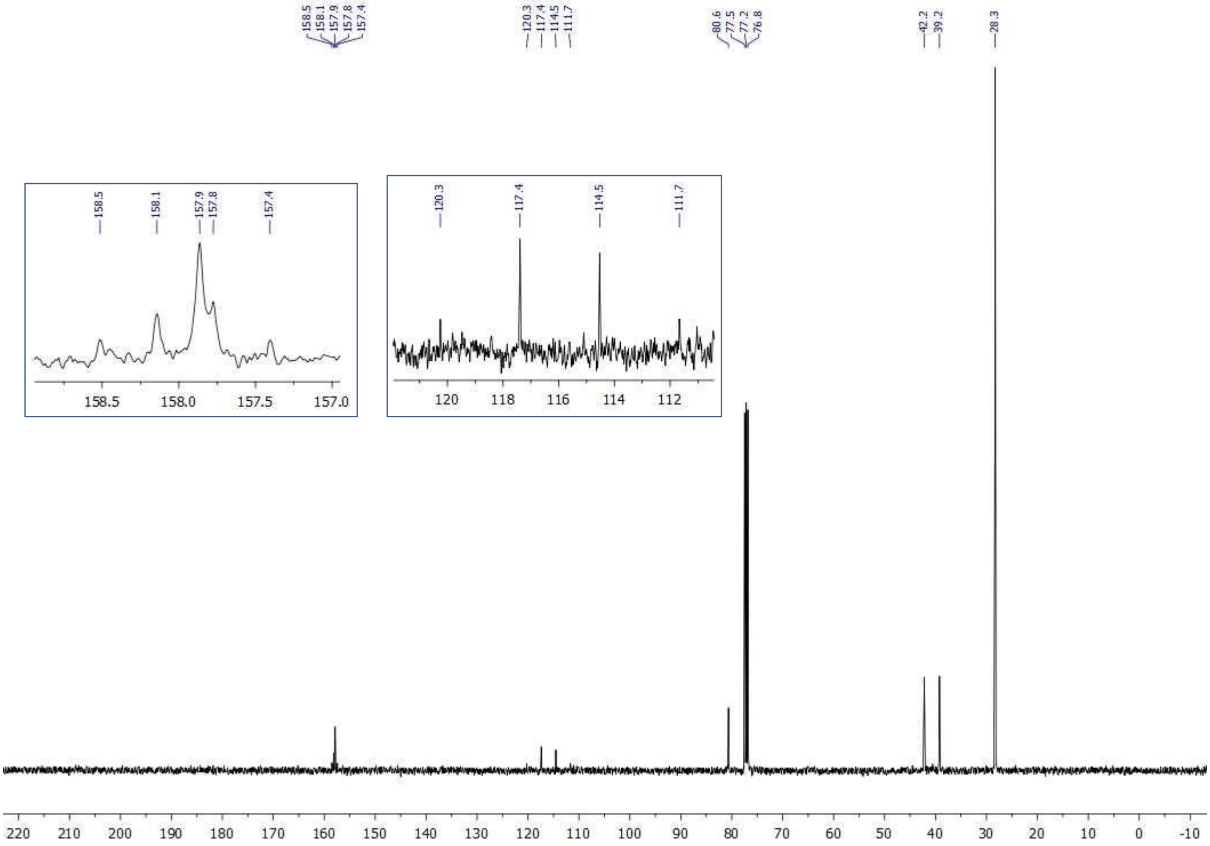

Figure S153 – ^{13}C NMR spectrum of 4b-1 (101 MHz, CDCl$_3$).

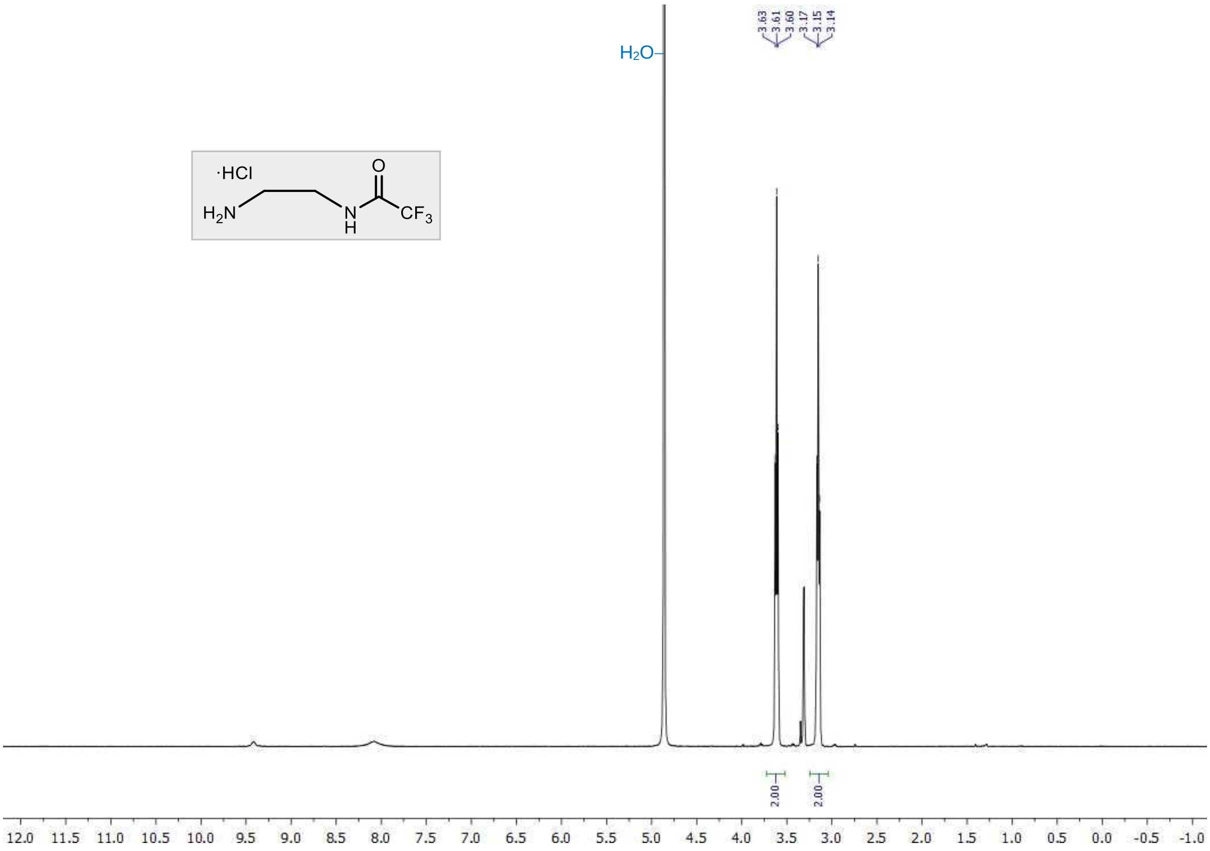

Figure S154 – ^{1}H NMR spectrum of 4b-2 (400 MHz, CD_3OD).

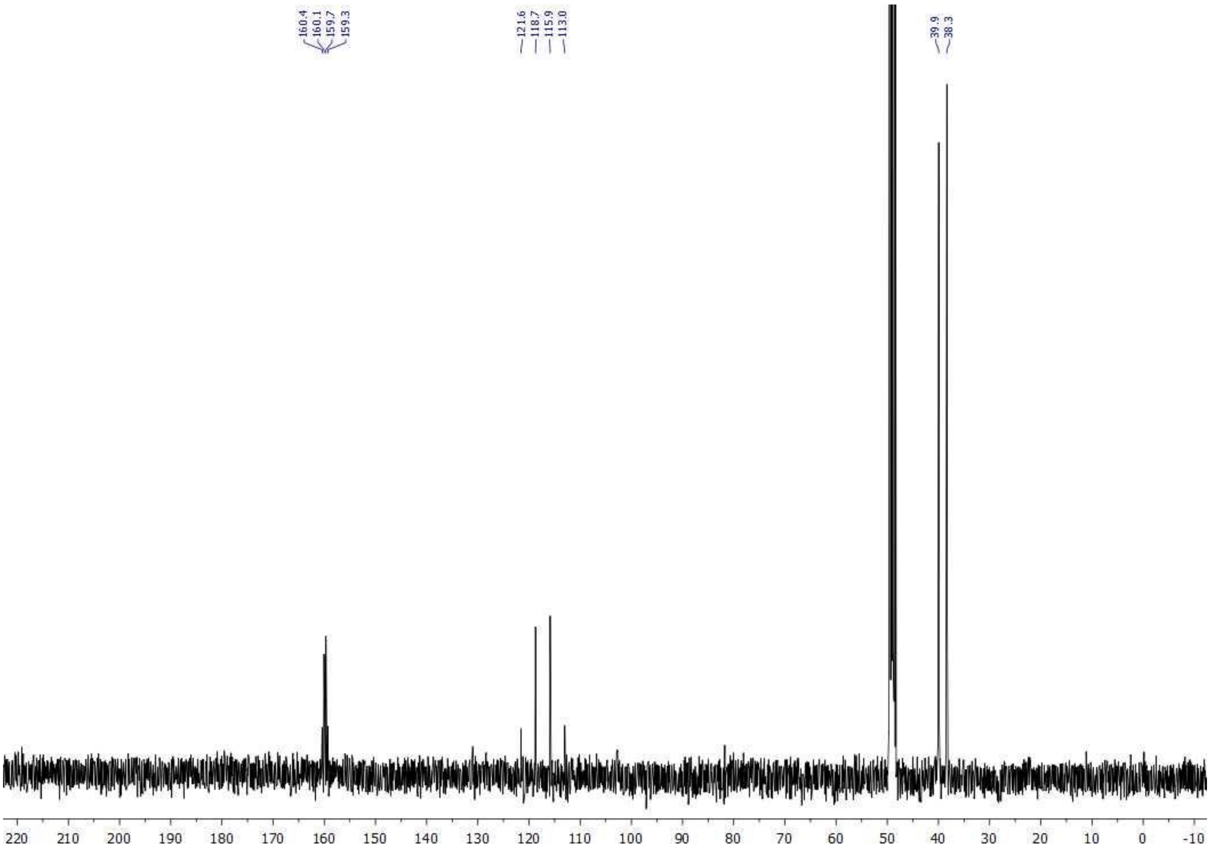

Figure S155 – ^{13}C NMR spectrum of 4b-2 (101 MHz, CD_3OD).

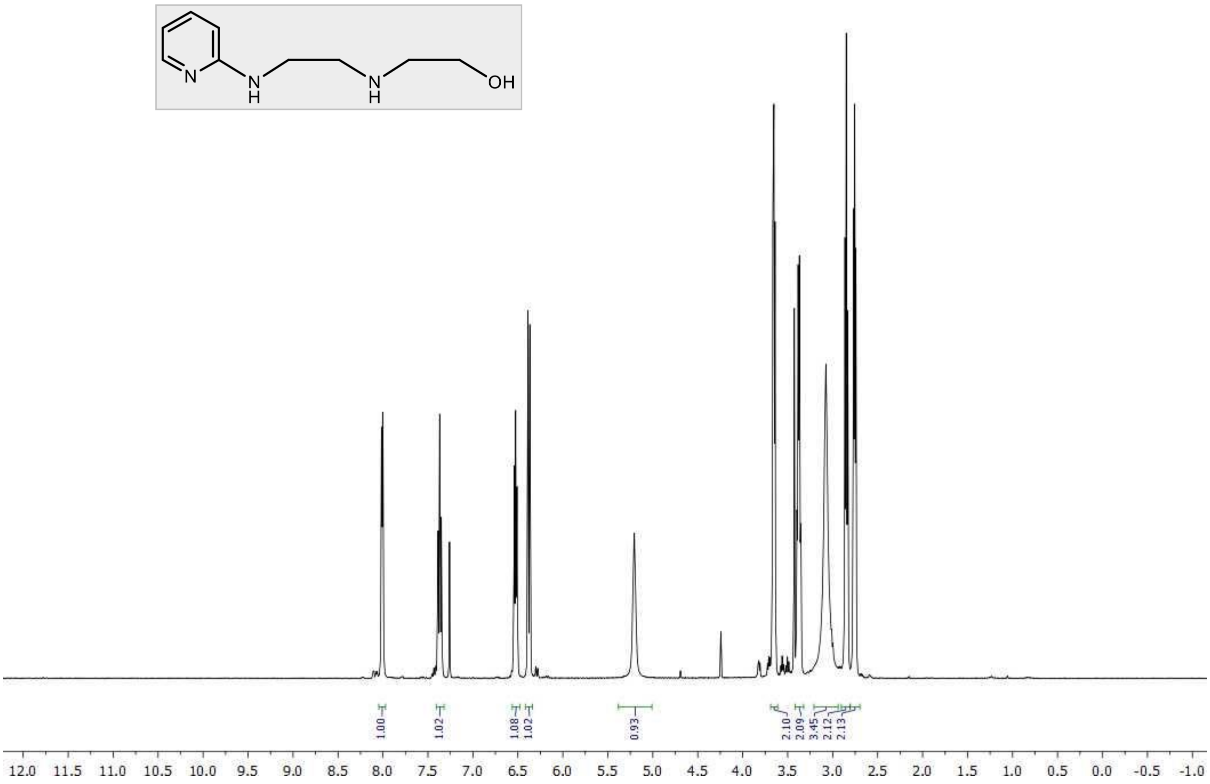

Figure S156 – ^{1}H NMR spectrum of 4b-3 (400 MHz, $CDCl_3$).

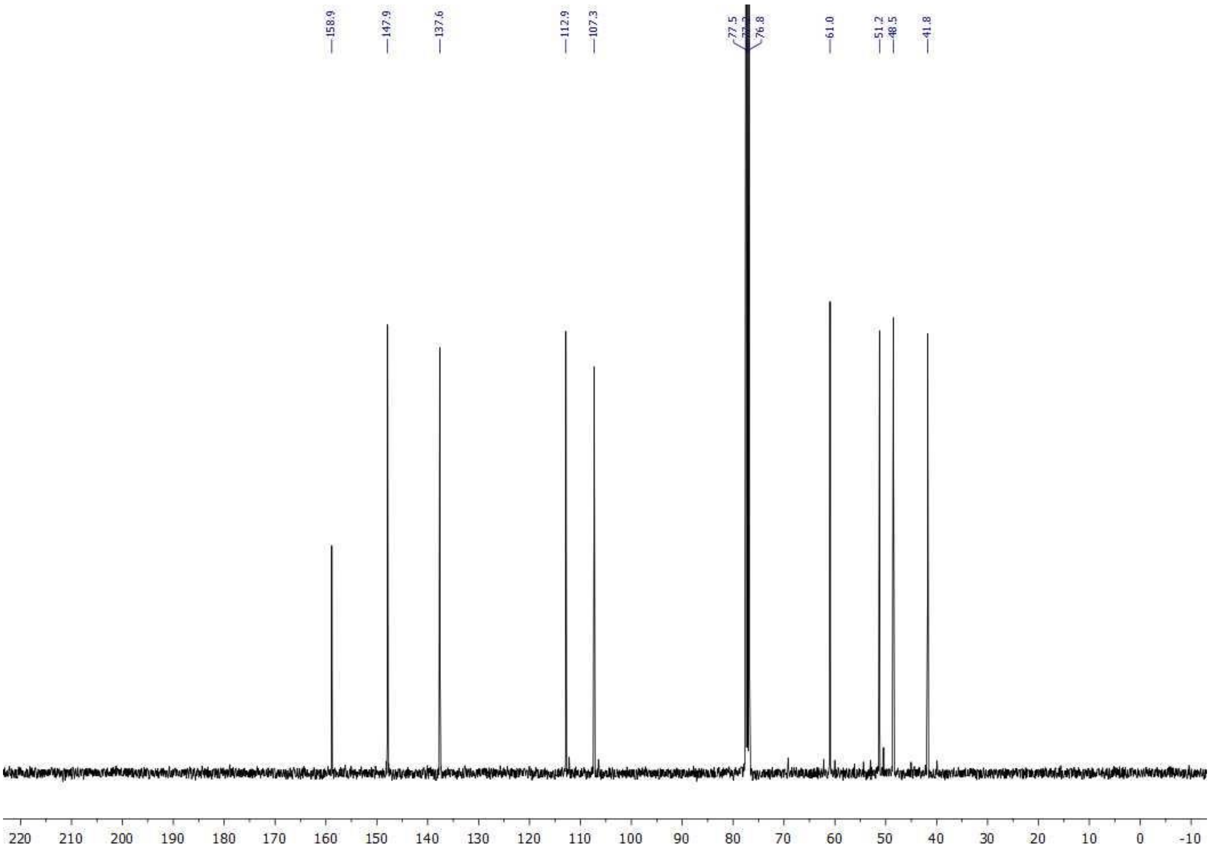

Figure S157 – ^{13}C NMR spectrum of 4b-3 (101 MHz, $CDCl_3$).

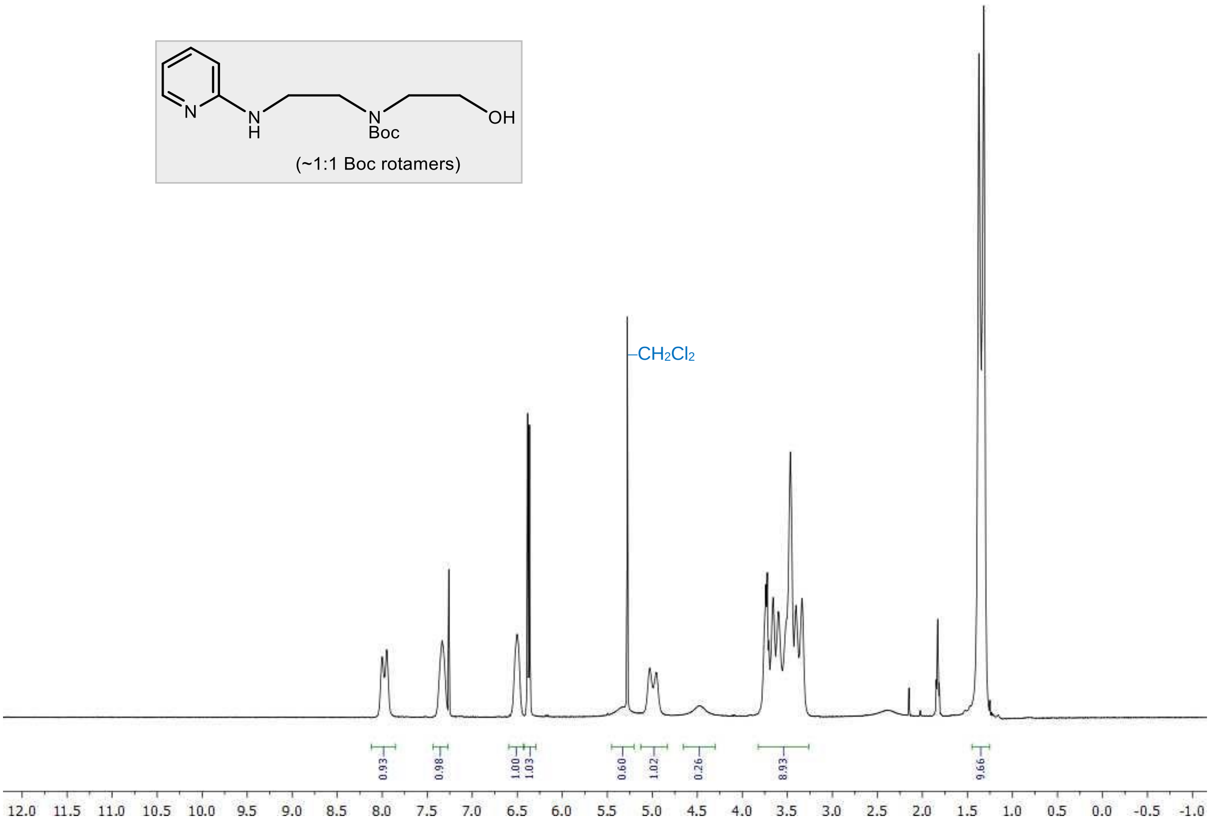

Figure S158 – ^{1}H NMR spectrum of 4b-4 (400 MHz, $CDCl_3$).

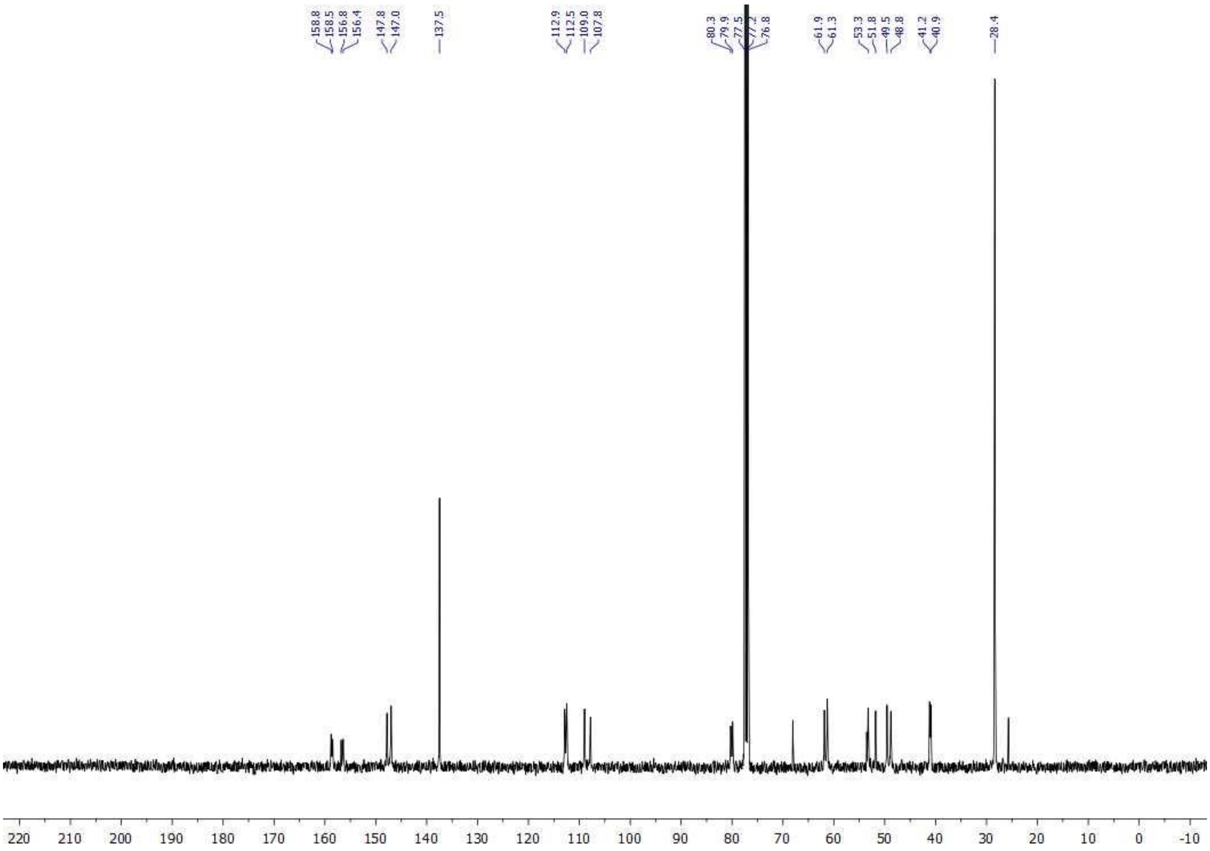

Figure S159 – ^{13}C NMR spectrum of 4b-4 (101 MHz, $CDCl_3$).

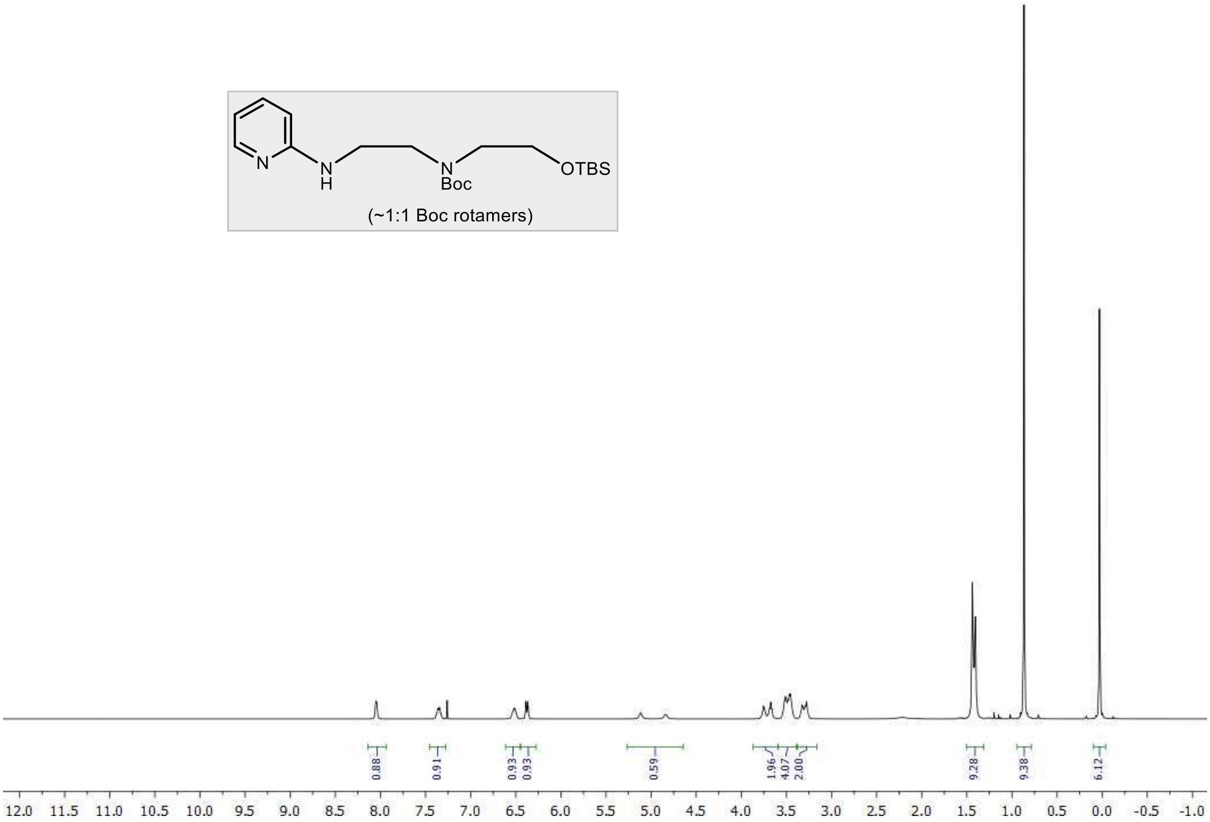

Figure S160 – ^{1}H NMR spectrum of 4b-5 (400 MHz, $CDCl_3$).

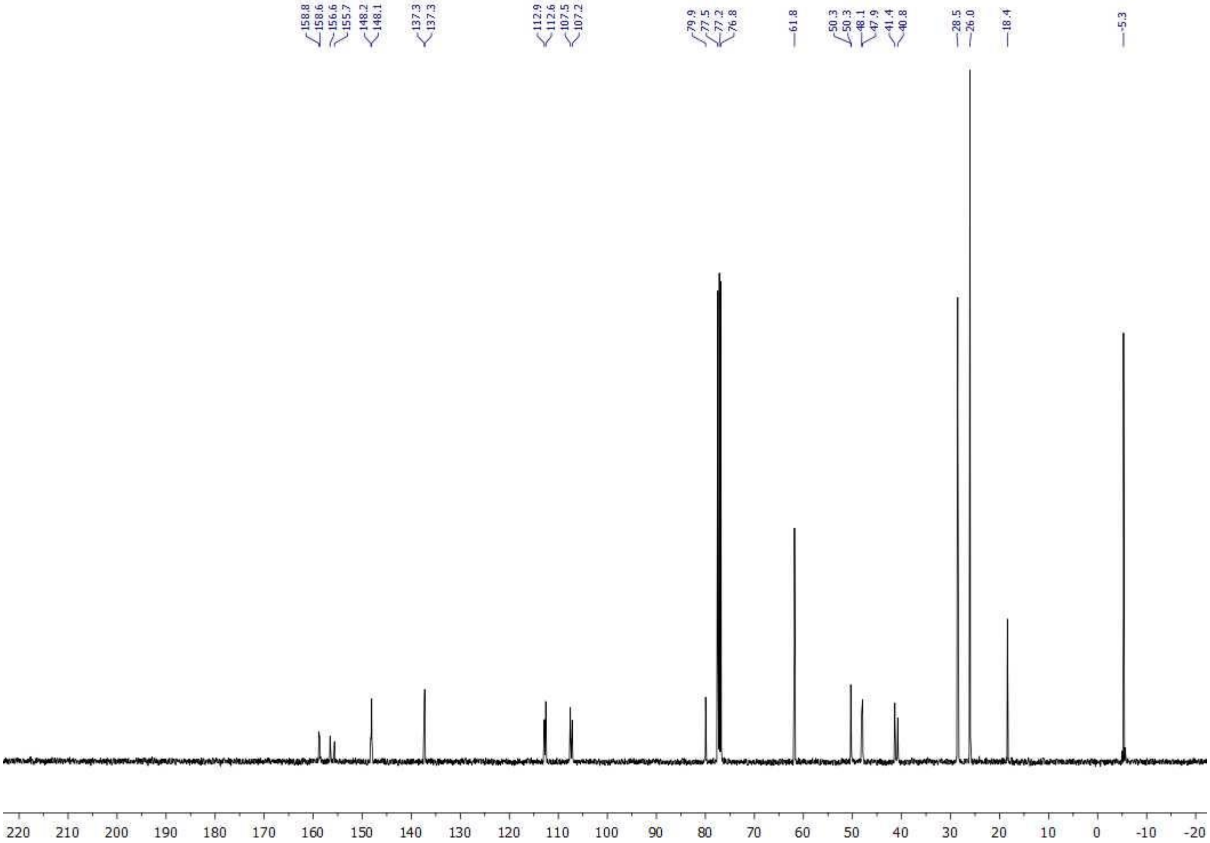

Figure S161 – ^{13}C NMR spectrum of 4b-5 (101 MHz, $CDCl_3$).

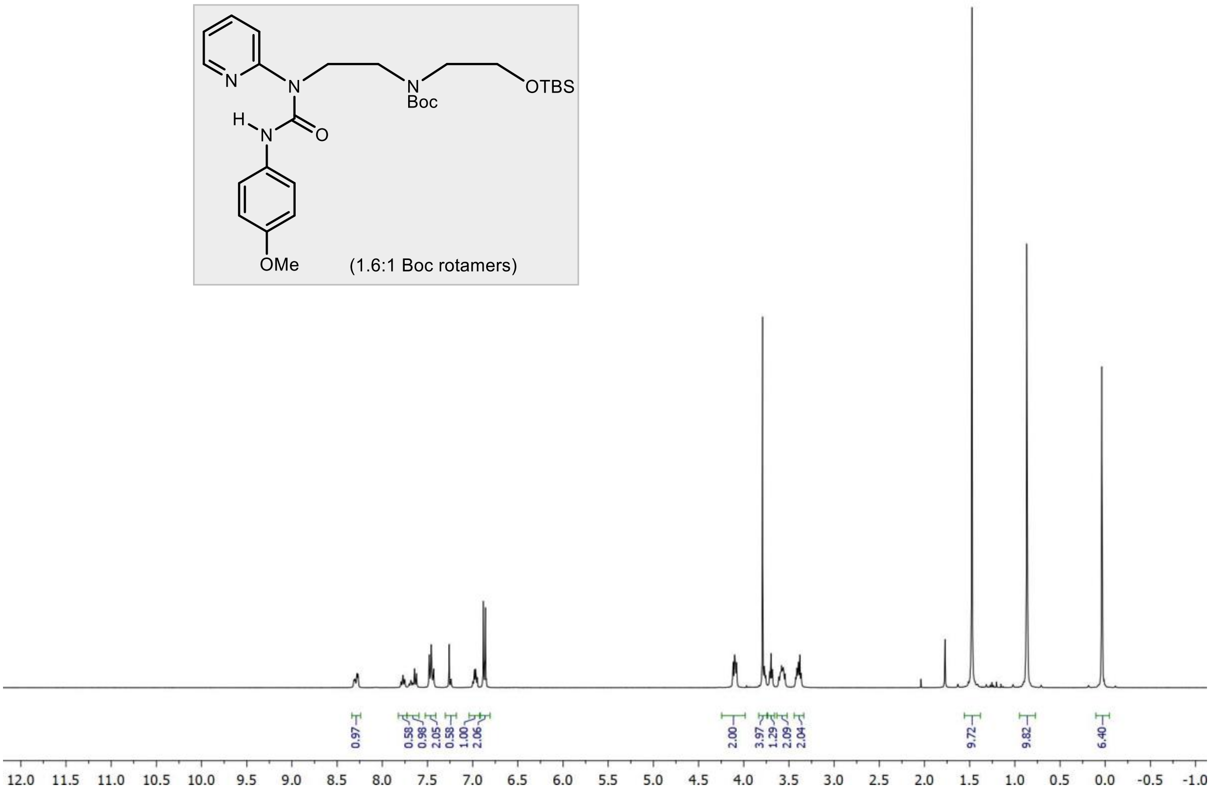

Figure S162 – ^{1}H NMR spectrum of 4b-6 (400 MHz, $CDCl_3$).

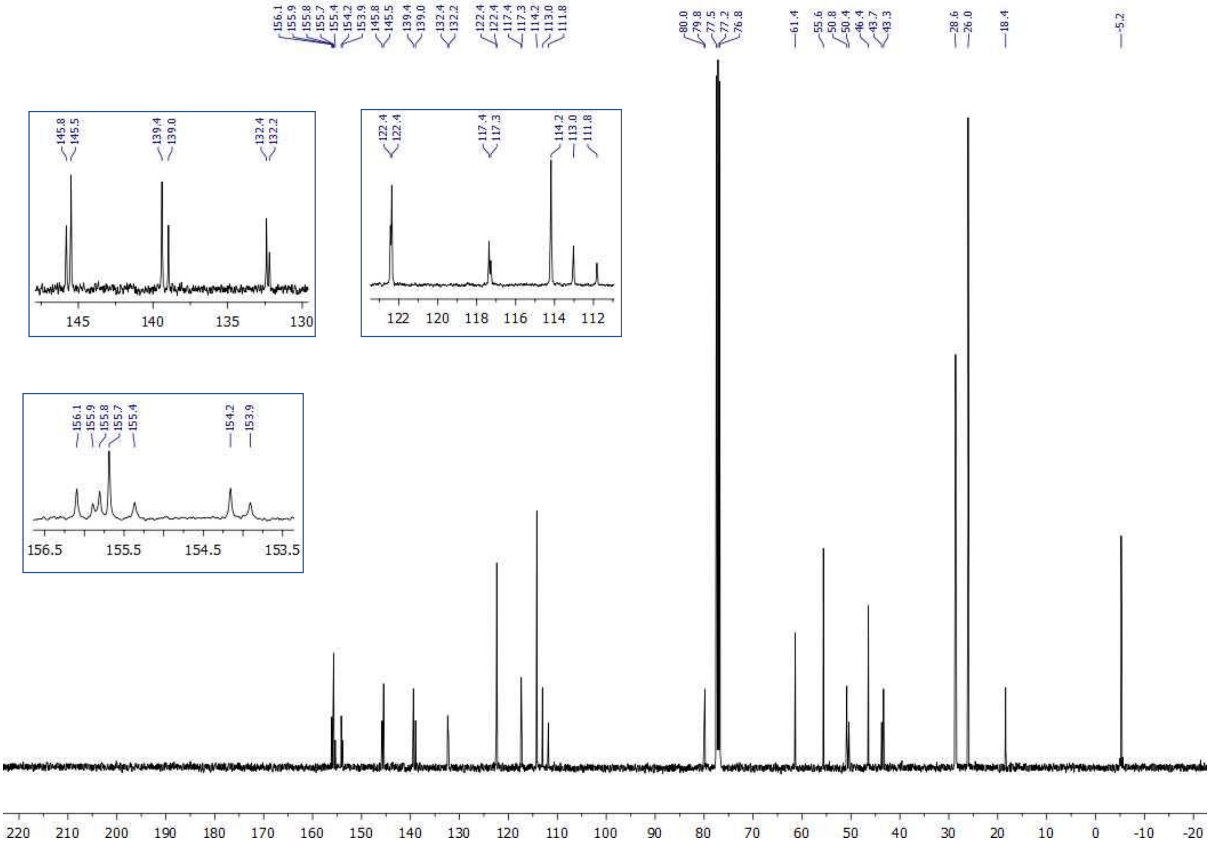

Figure S163 – ^{13}C NMR spectrum of 4b-6 (101 MHz, $CDCl_3$).

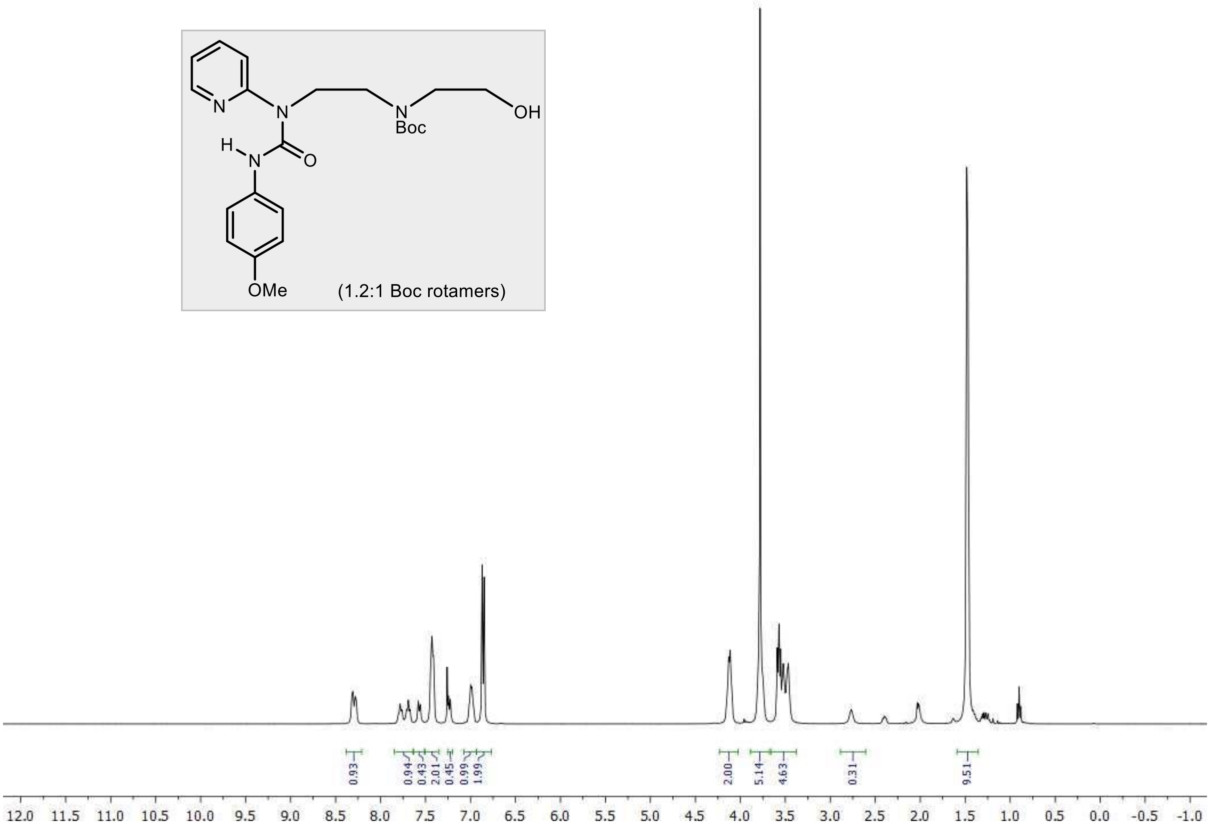

Figure S164 – ^{1}H NMR spectrum of 4b-7 (400 MHz, $CDCl_3$).

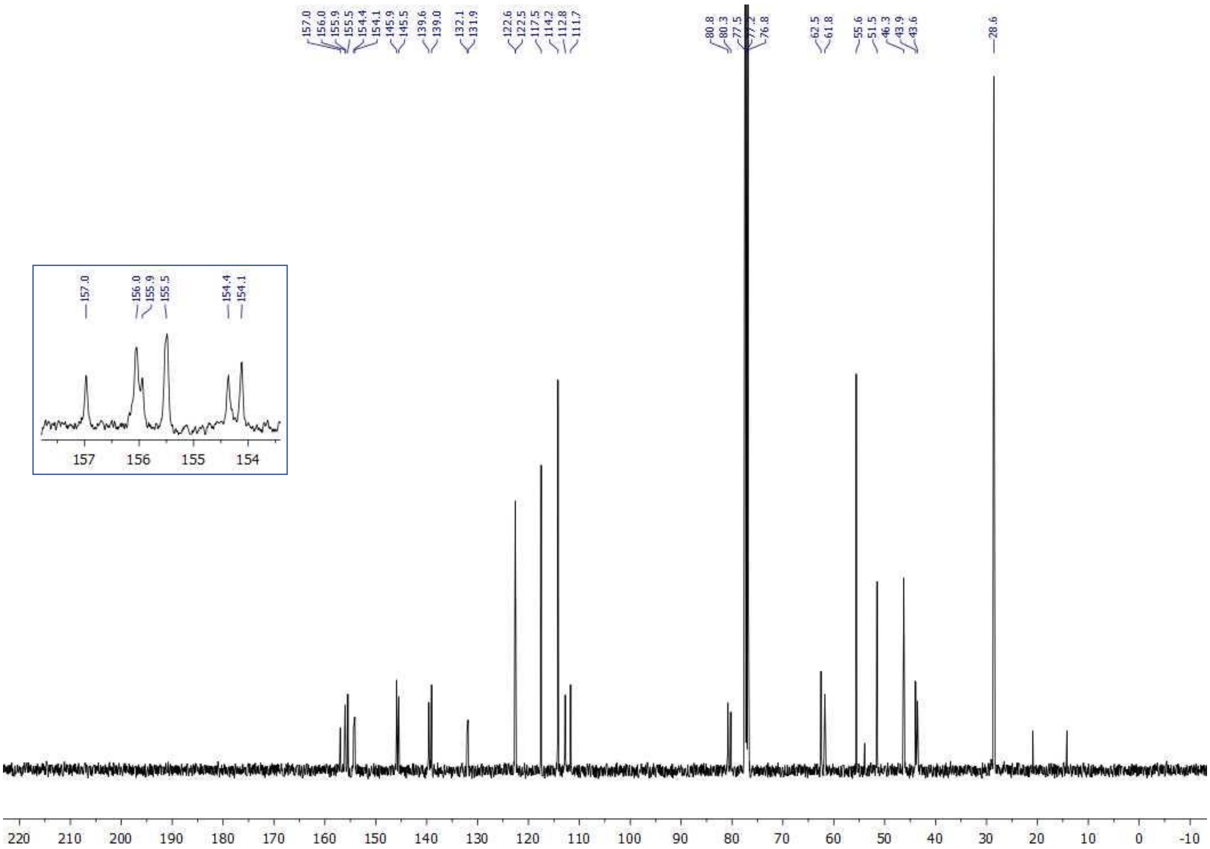

Figure S165 – ^{13}C NMR spectrum of 4b-7 (101 MHz, $CDCl_3$).

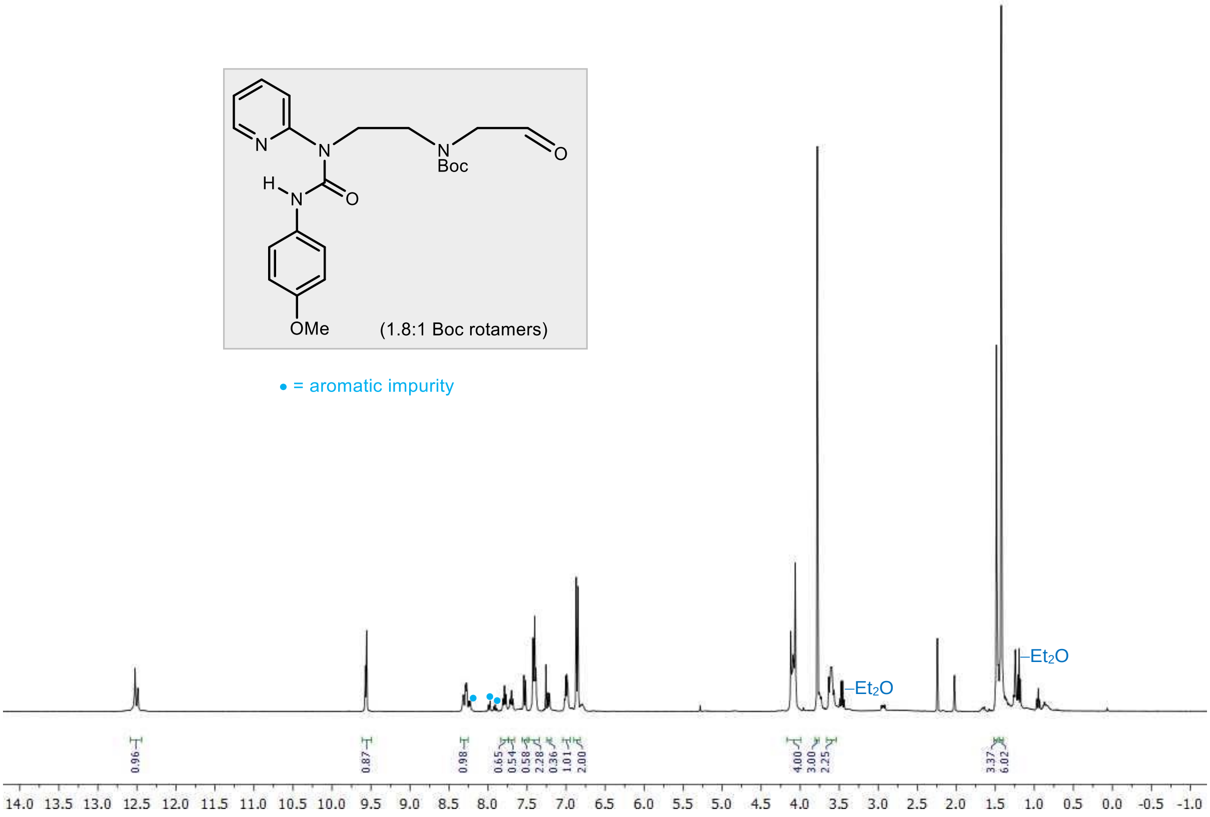

Figure S166 – ^{1}H NMR spectrum of 4b-8 (400 MHz, $CDCl_3$).

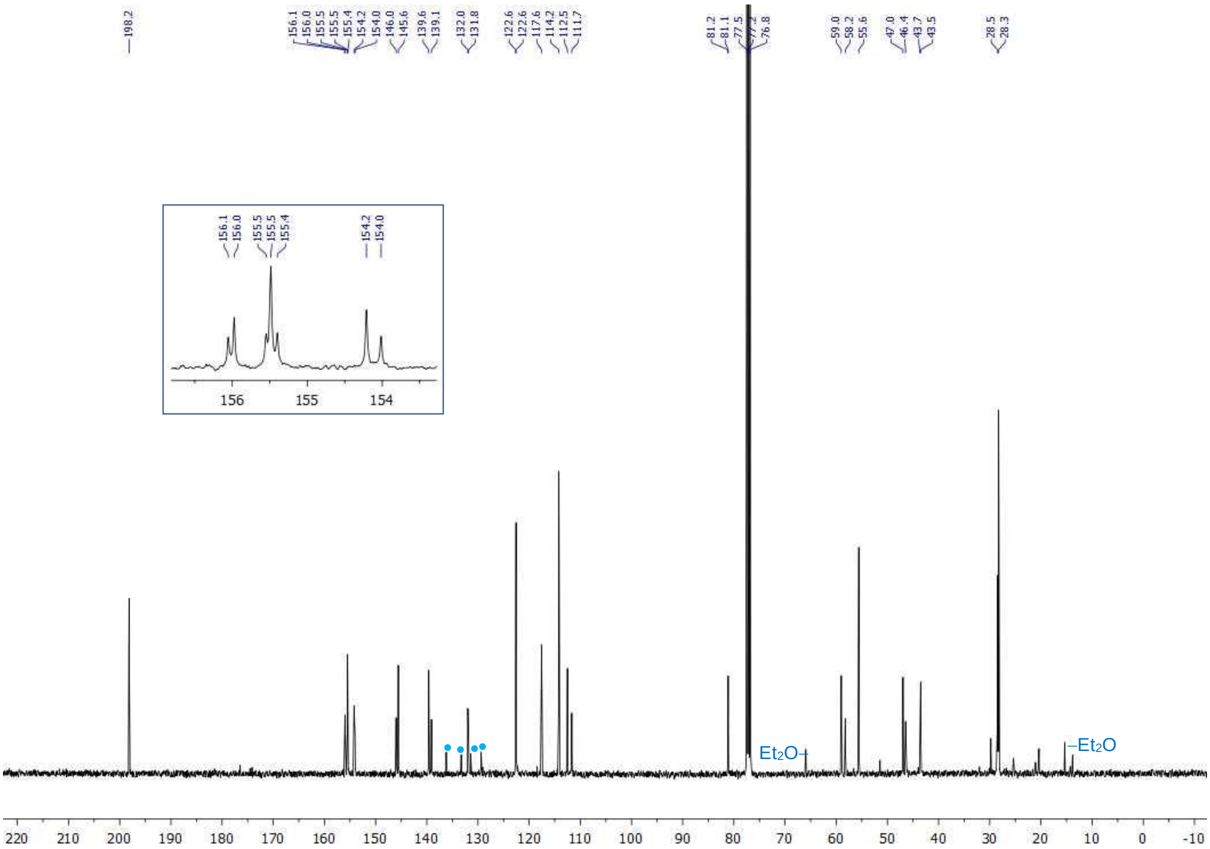

Figure S167 – ^{13}C NMR spectrum of 4b-8 (101 MHz, $CDCl_3$).

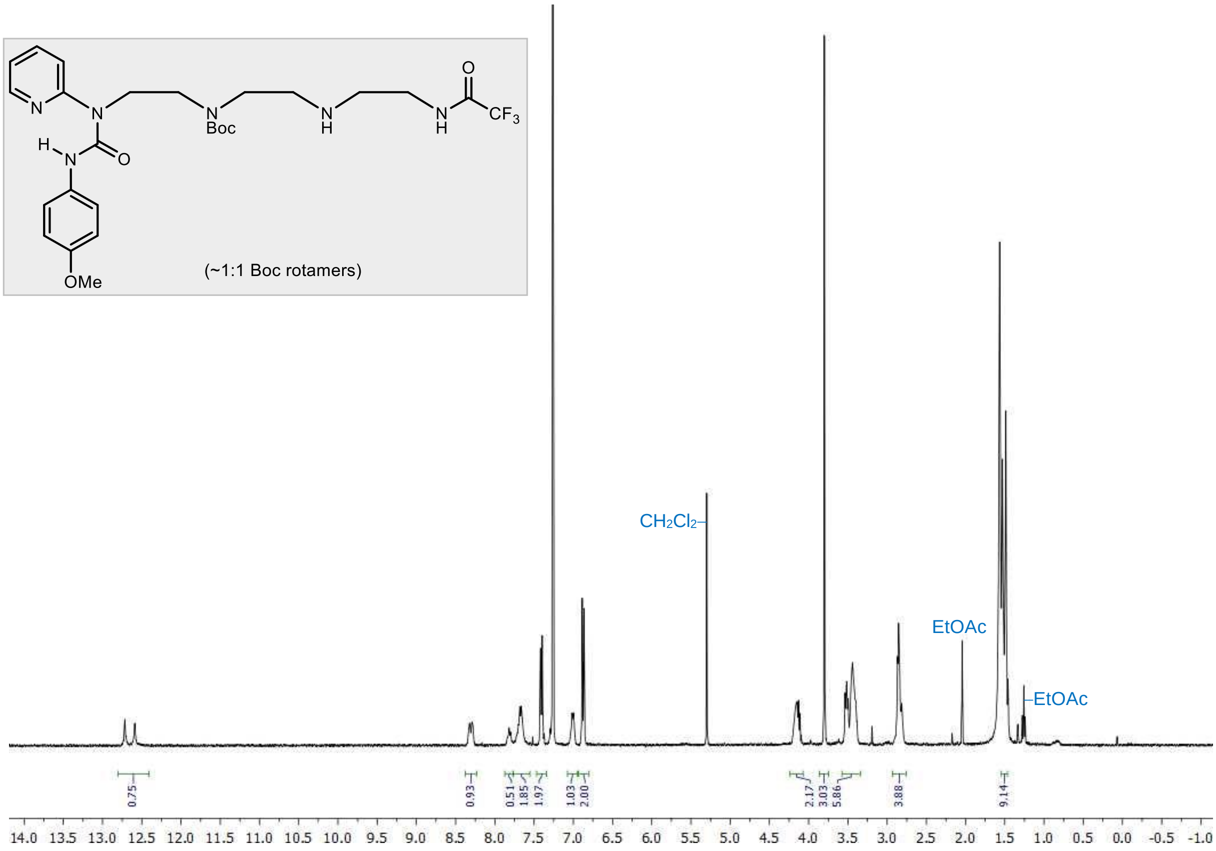

Figure S168 – ^{1}H NMR spectrum of 4b-9 (400 MHz, $CDCl_3$).

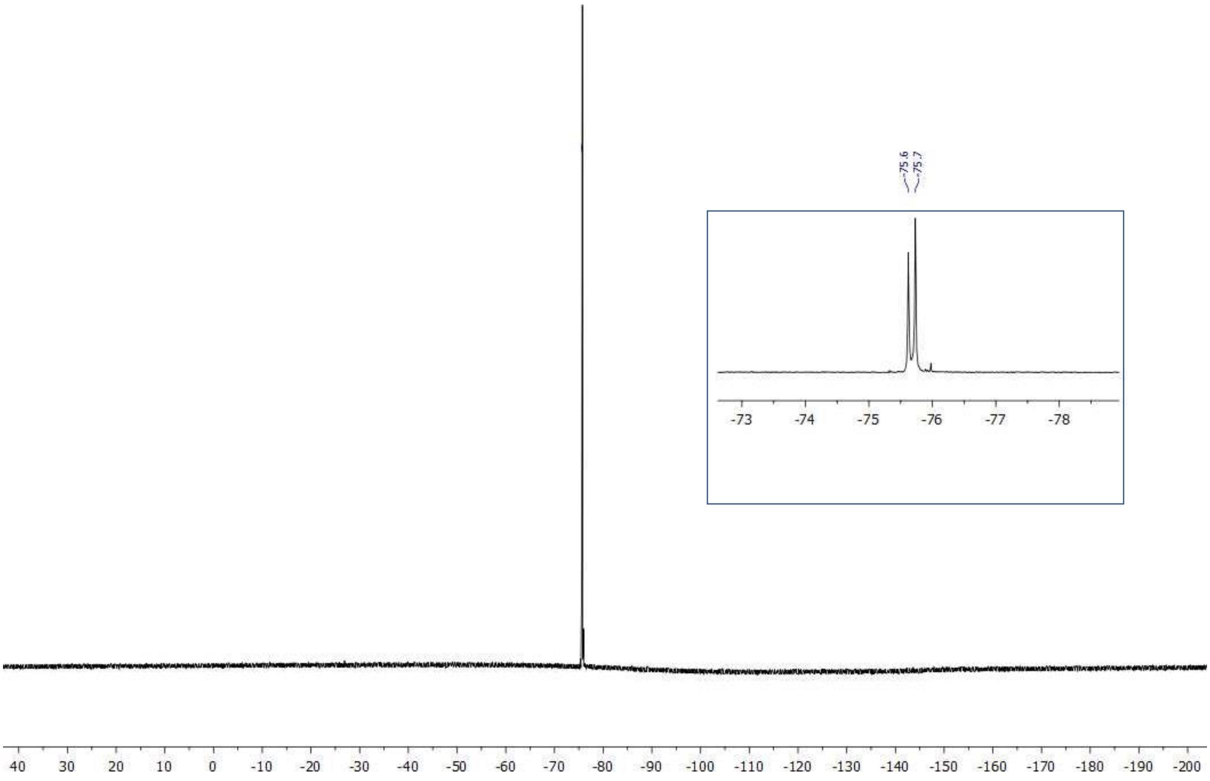

Figure S169 – ^{19}F NMR spectrum of 4b-9 (376 MHz, $CDCl_3$).

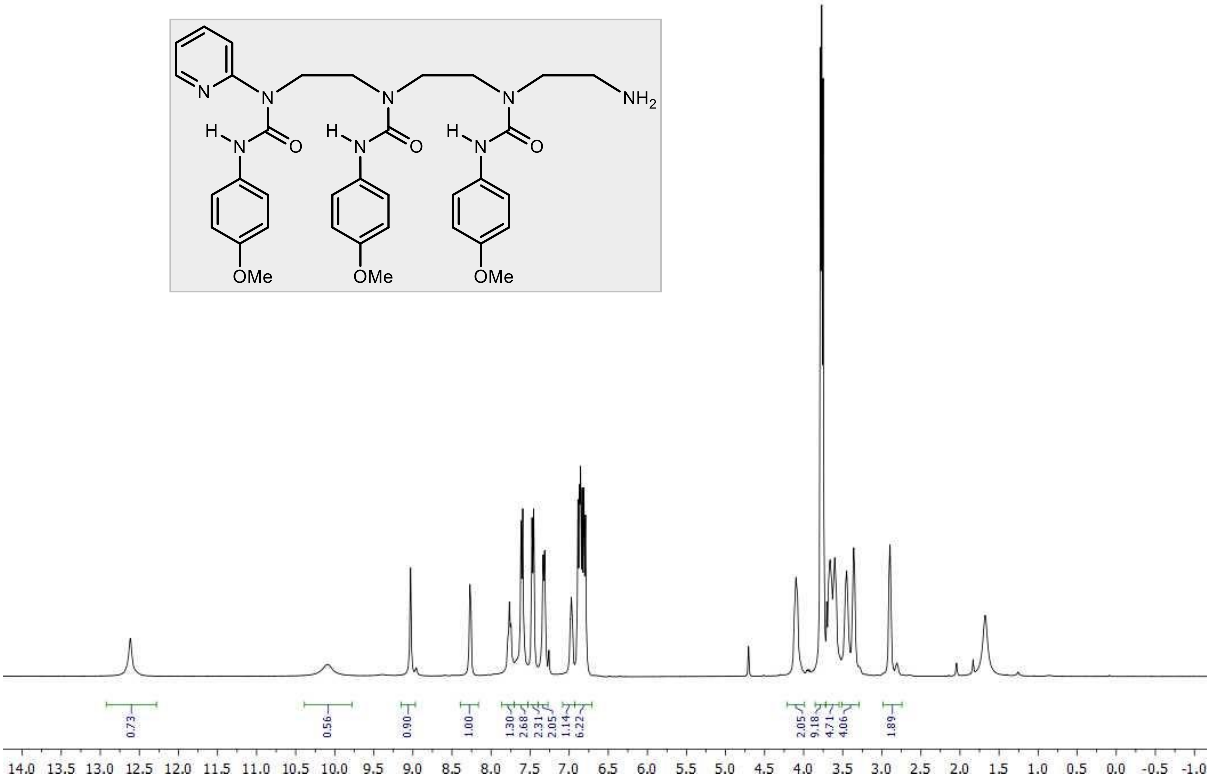

Figure S170 – ^{1}H NMR spectrum of 4b-10 (400 MHz, $CDCl_3$).

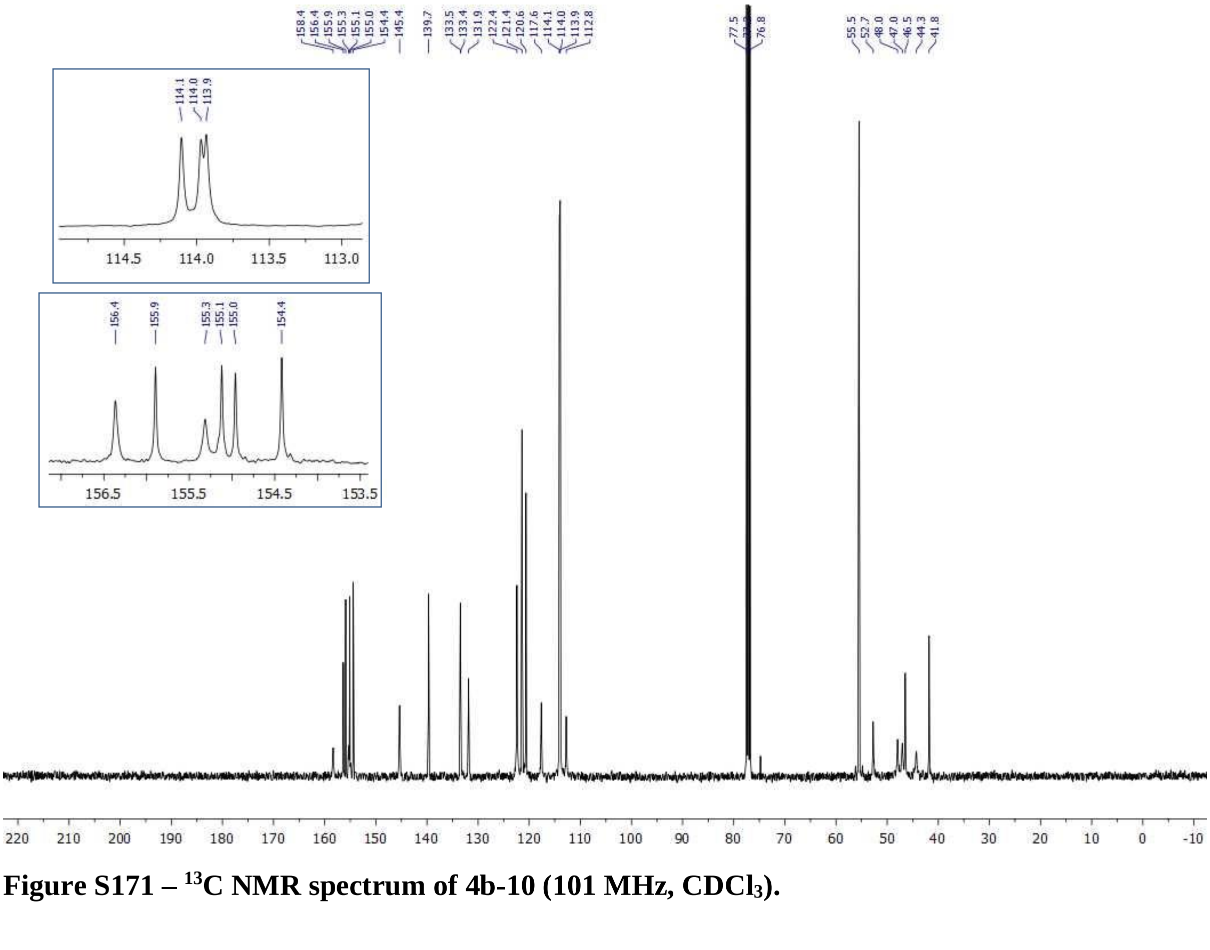

Figure S171 – ^{13}C NMR spectrum of 4b-10 (101 MHz, $CDCl_3$).

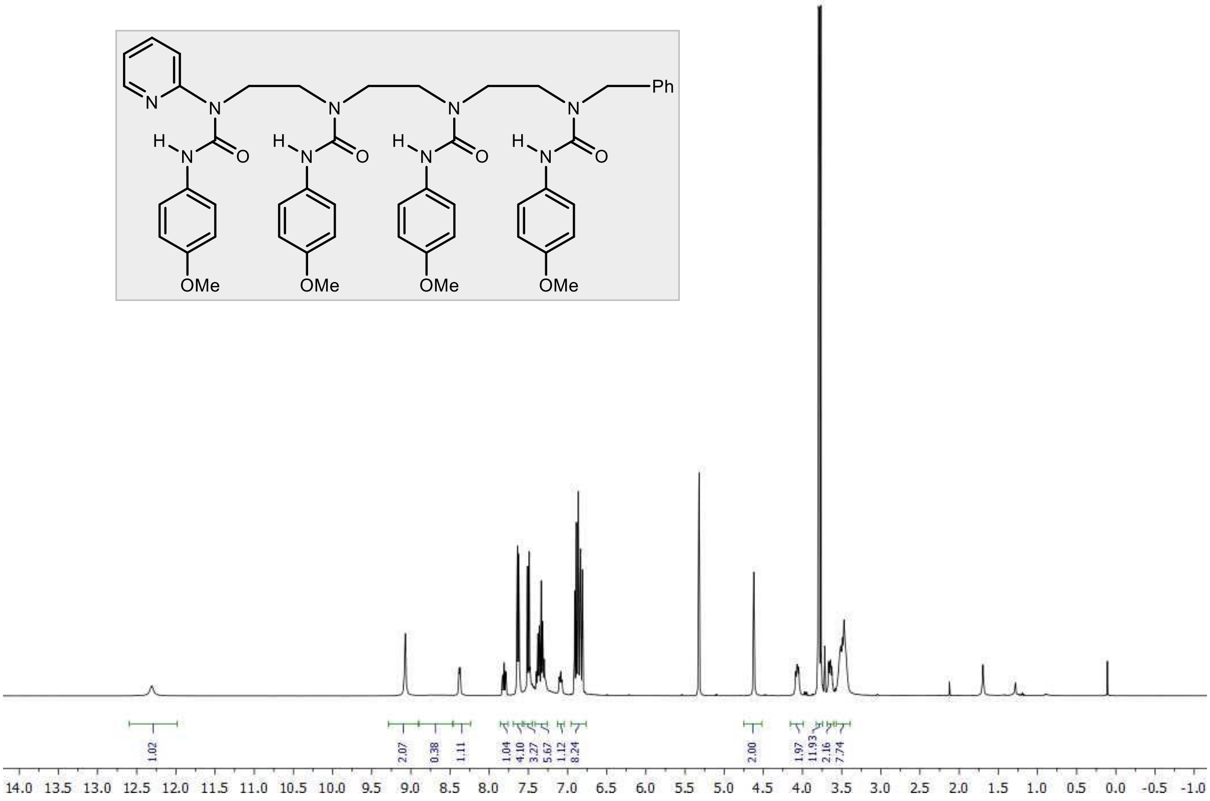

Figure S172 – ^{1}H NMR spectrum of 4b (400 MHz, CD_2Cl_2).

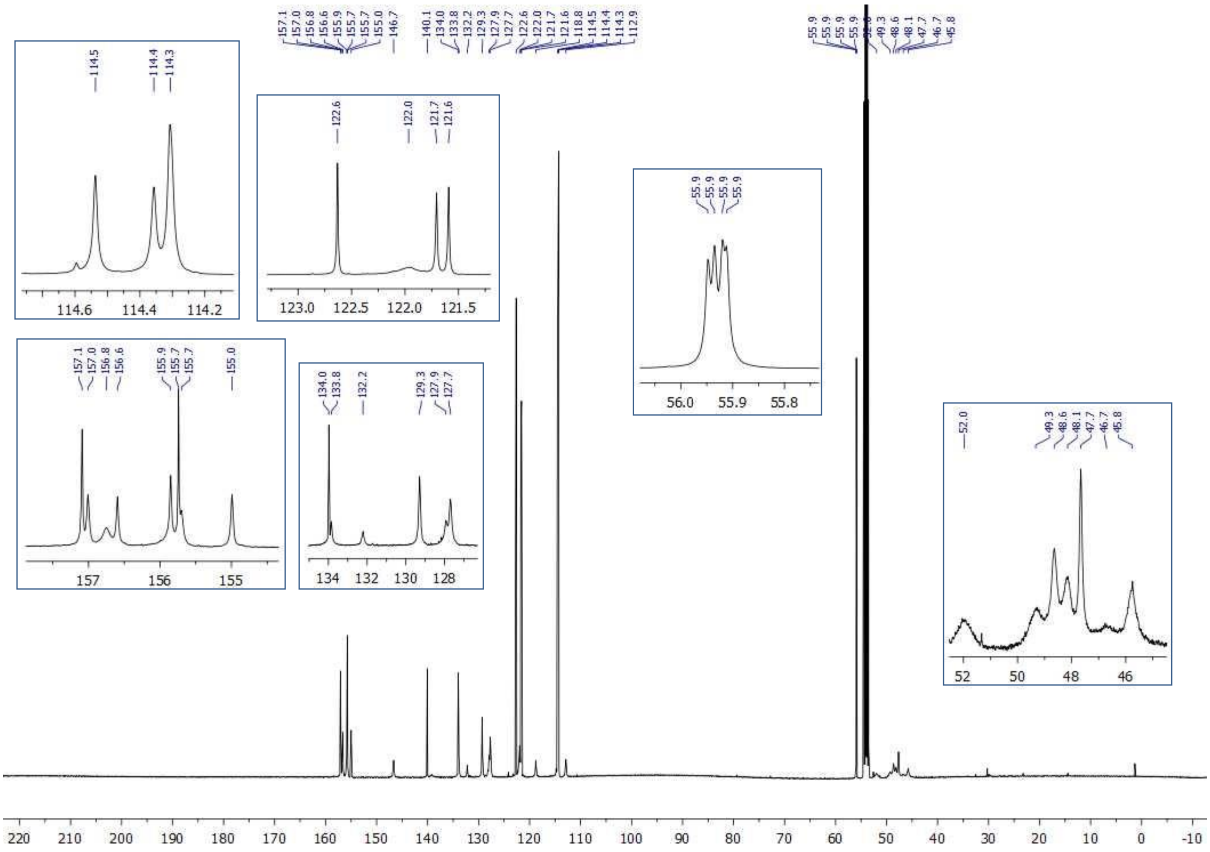

Figure S173 – ^{13}C NMR spectrum of 4b (101 MHz, CD_2Cl_2).

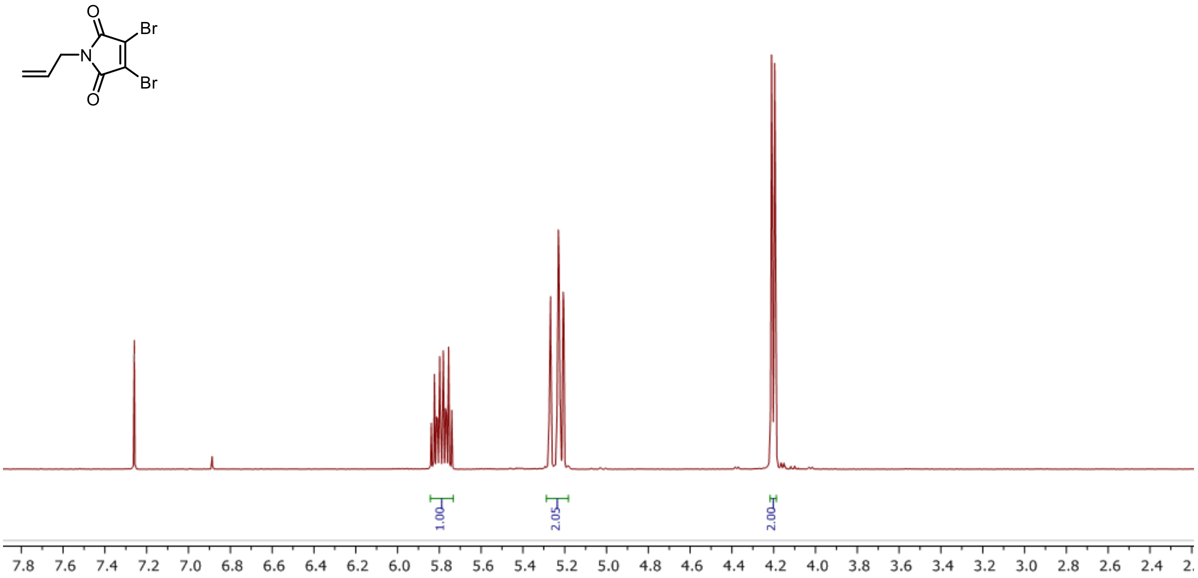

Figure S174 – ^{1}H NMR spectrum of 7a-2 (400 MHz, $CDCl_3$).

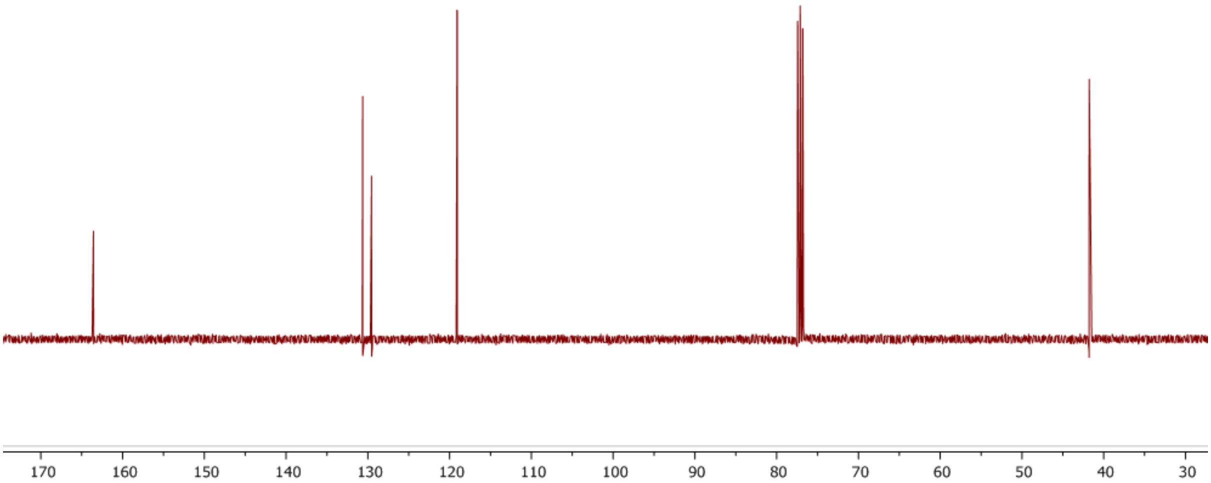

Figure S175 – ^{13}C NMR spectrum of 7a-2 (101 MHz, $CDCl_3$).

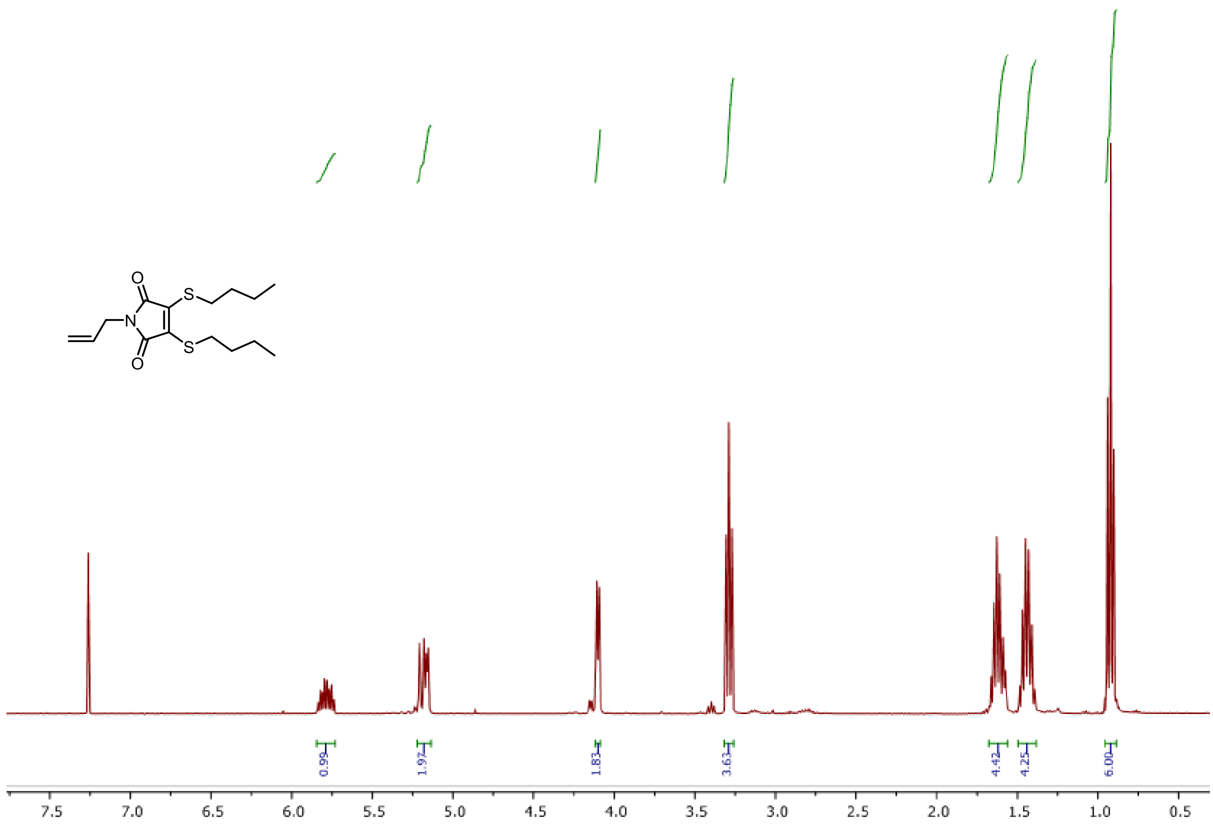

Figure S176 – ^{1}H NMR spectrum of 7a (400 MHz, $CDCl_3$).

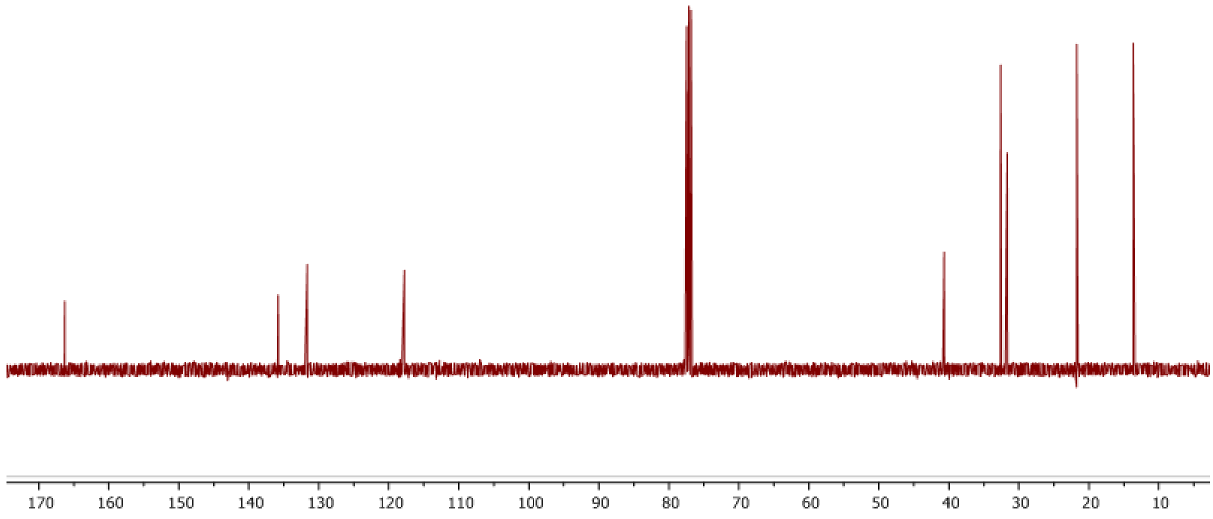

Figure S177 – ^{13}C NMR spectrum of 7a (101 MHz, $CDCl_3$).

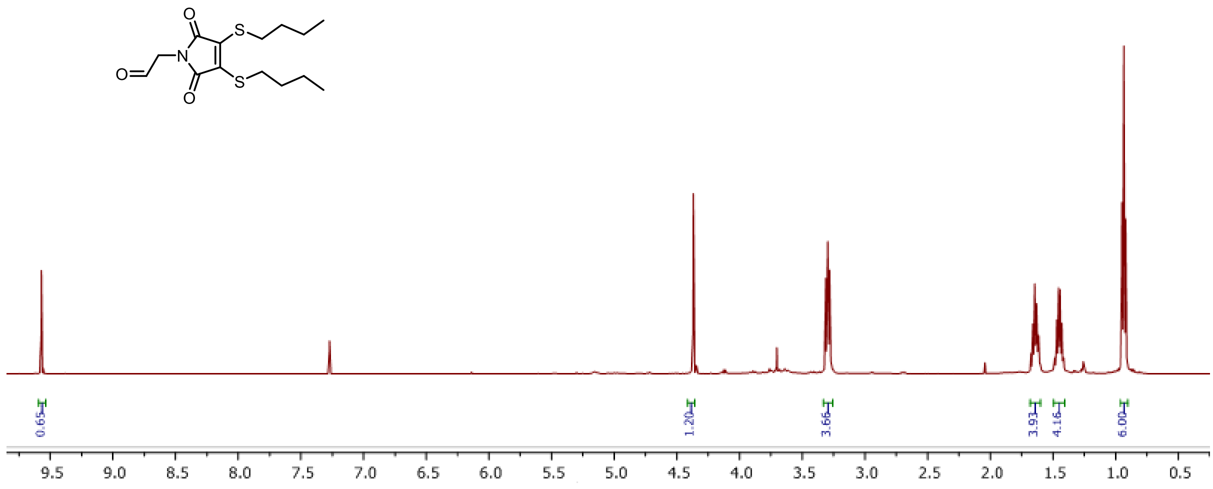

Figure S178 – ^{1}H NMR spectrum of 7a-3 (400 MHz, $CDCl_3$).

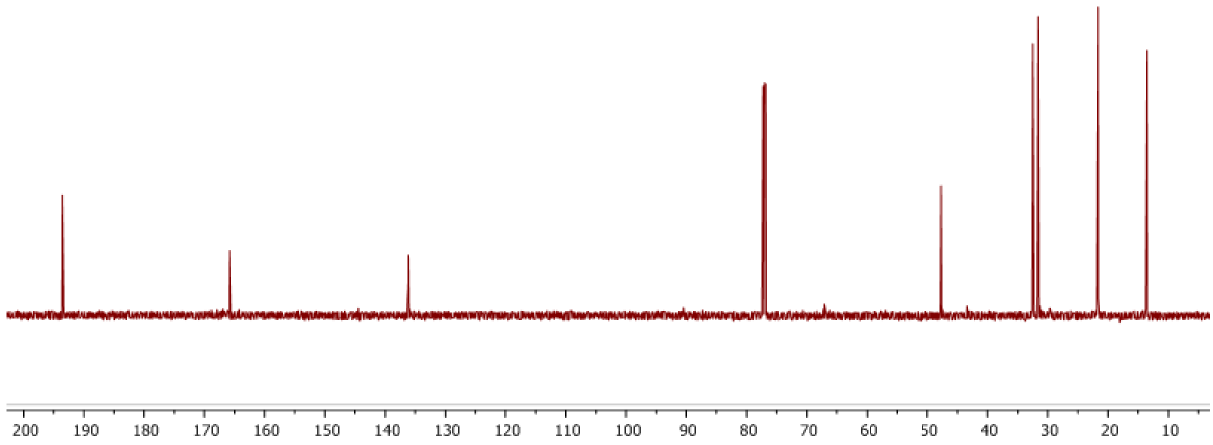

Figure S179 – ^{13}C NMR spectrum of 7a-3 (101 MHz, $CDCl_3$).

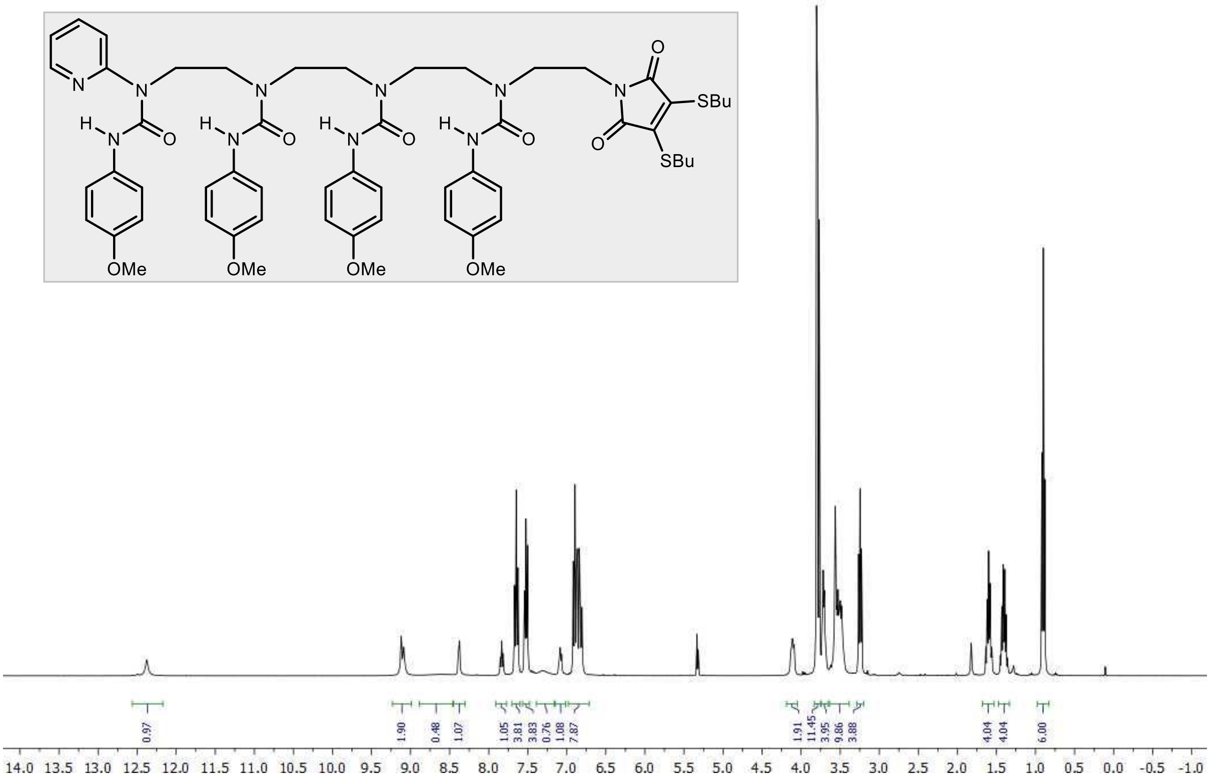

Figure S180 – ^{1}H NMR spectrum of 8a (400 MHz, CD_2Cl_2).

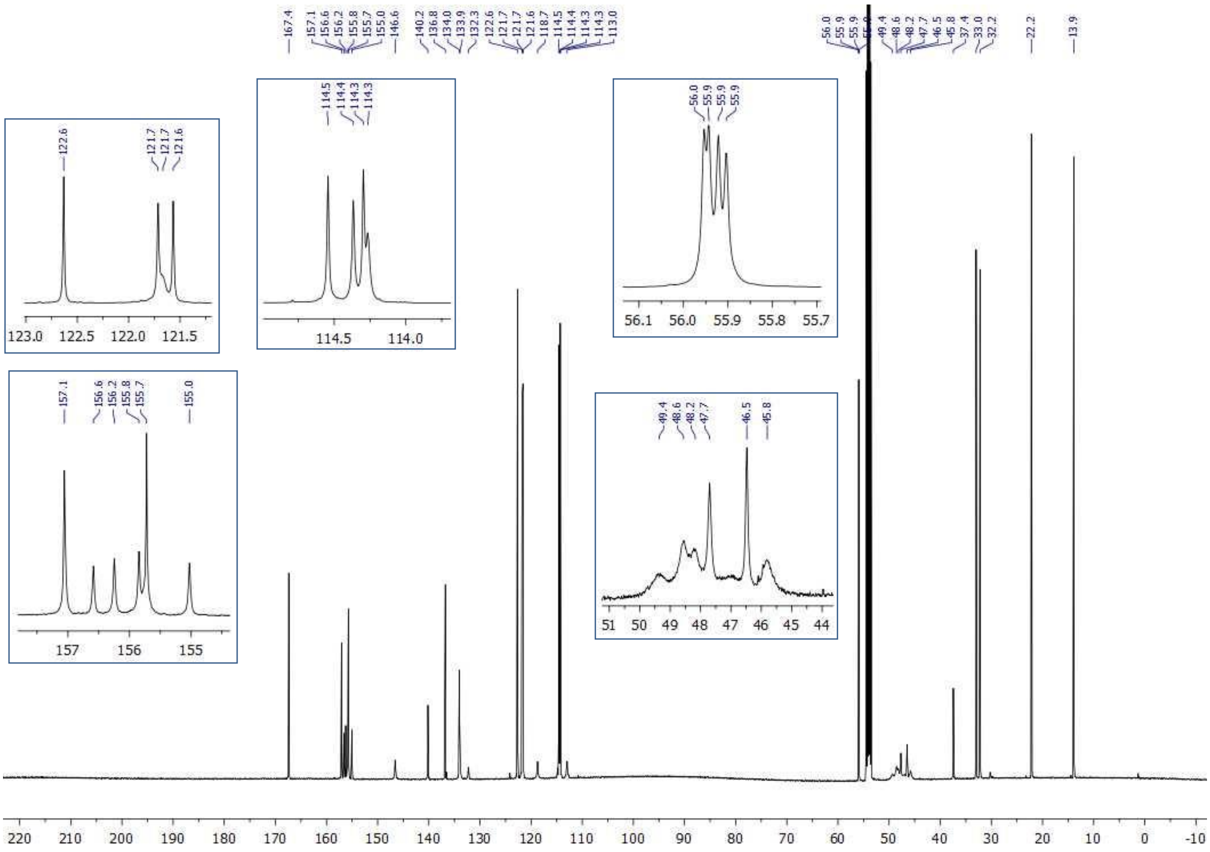

Figure S181 – ^{13}C NMR spectrum of 8a (126 MHz, CD_2Cl_2).

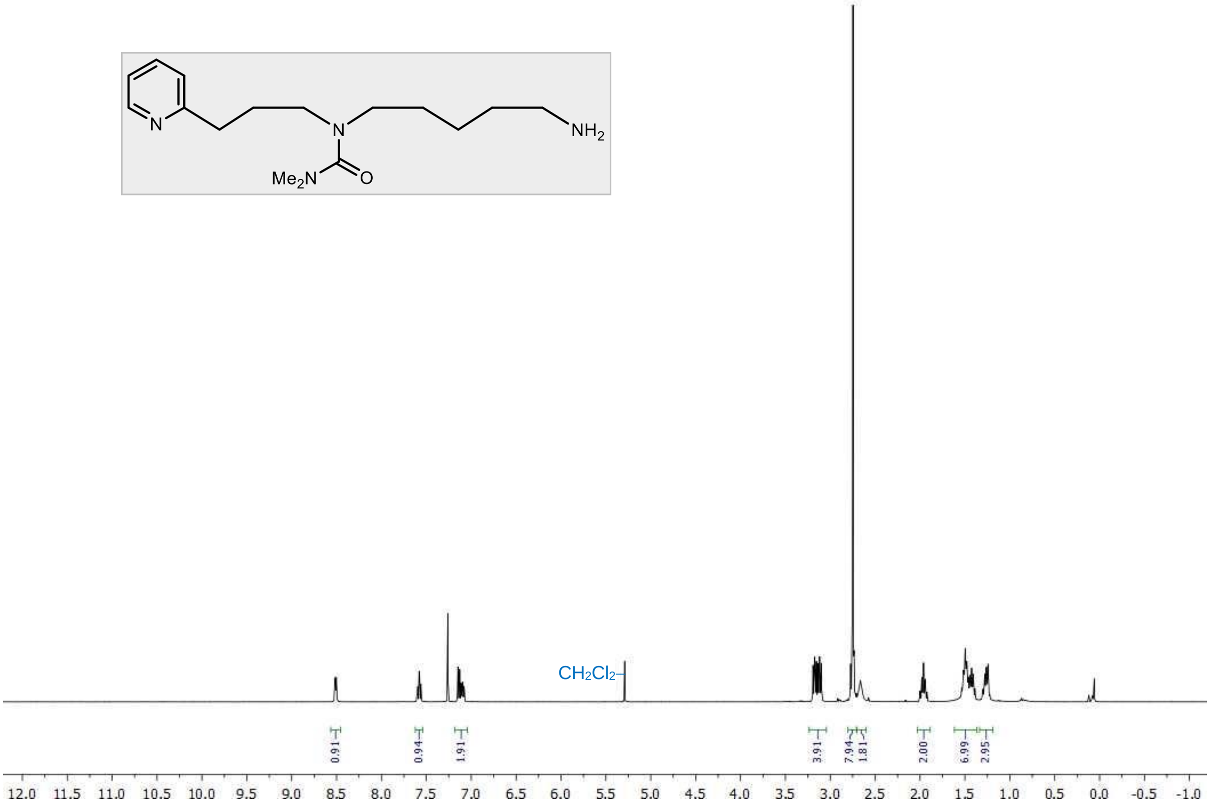

Figure S182 – ^{1}H NMR spectrum of 8b-1 (400 MHz, $CDCl_3$).

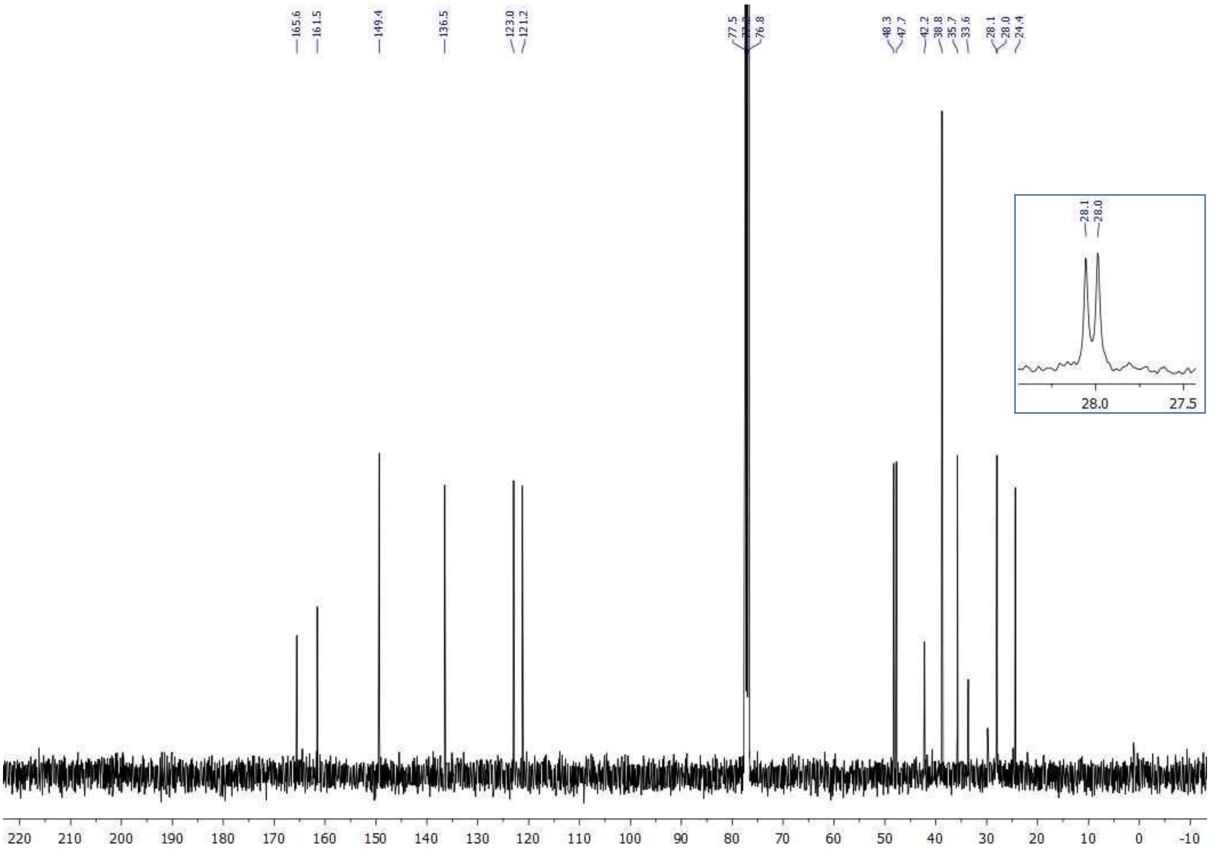

Figure S183 – ^{13}C NMR spectrum of 8b-1 (101 MHz, $CDCl_3$).

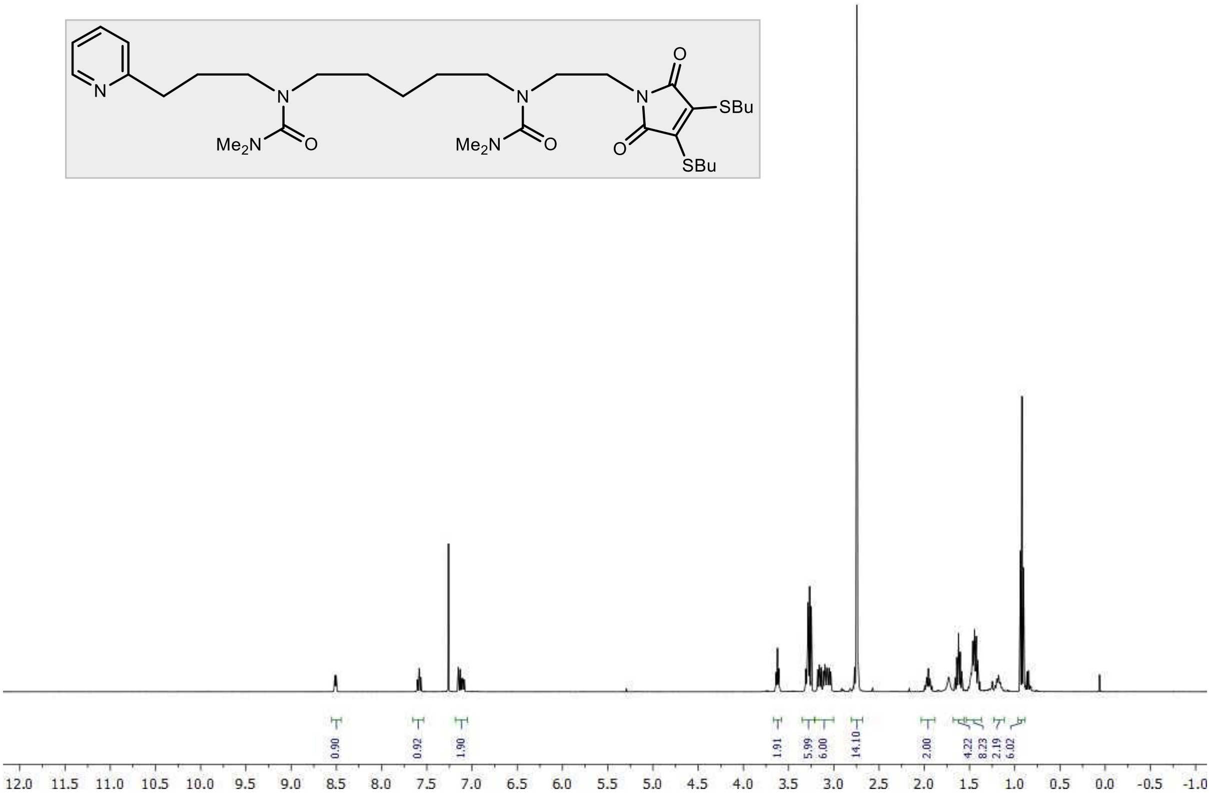

Figure S184 – ^{1}H NMR spectrum of 8b (400 MHz, CDCl$_3$).

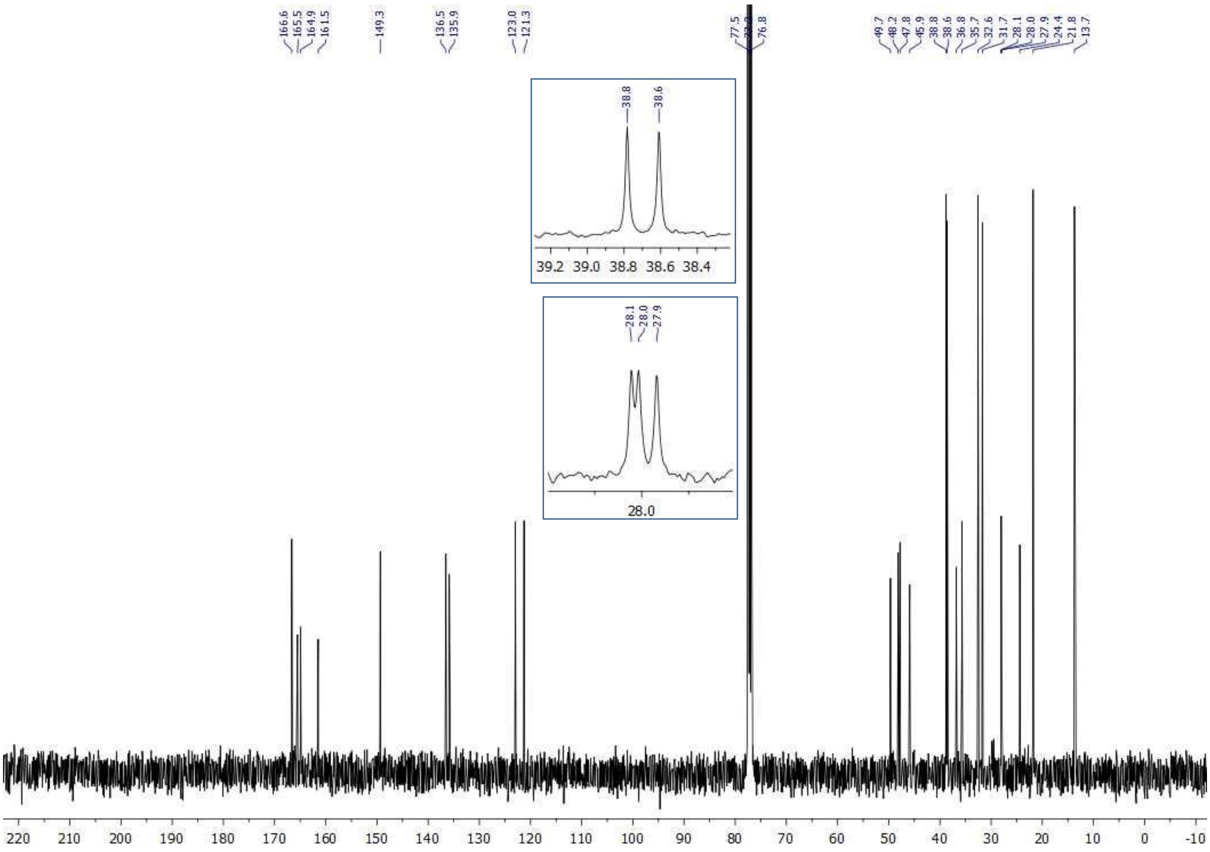

Figure S185 – ^{13}C NMR spectrum of 8b (101 MHz, CDCl$_3$).

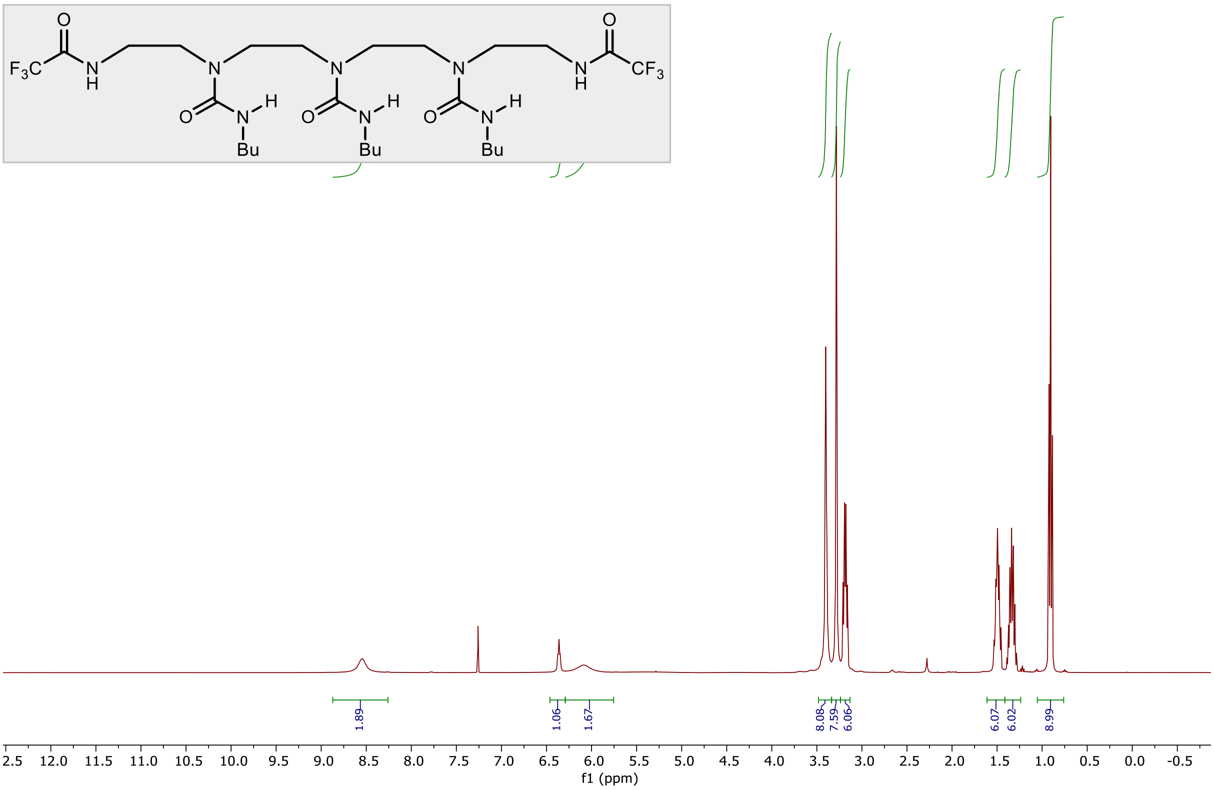

Figure S186 – ^{1}H NMR spectrum of 9-2 (400 MHz, CDCl$_3$).

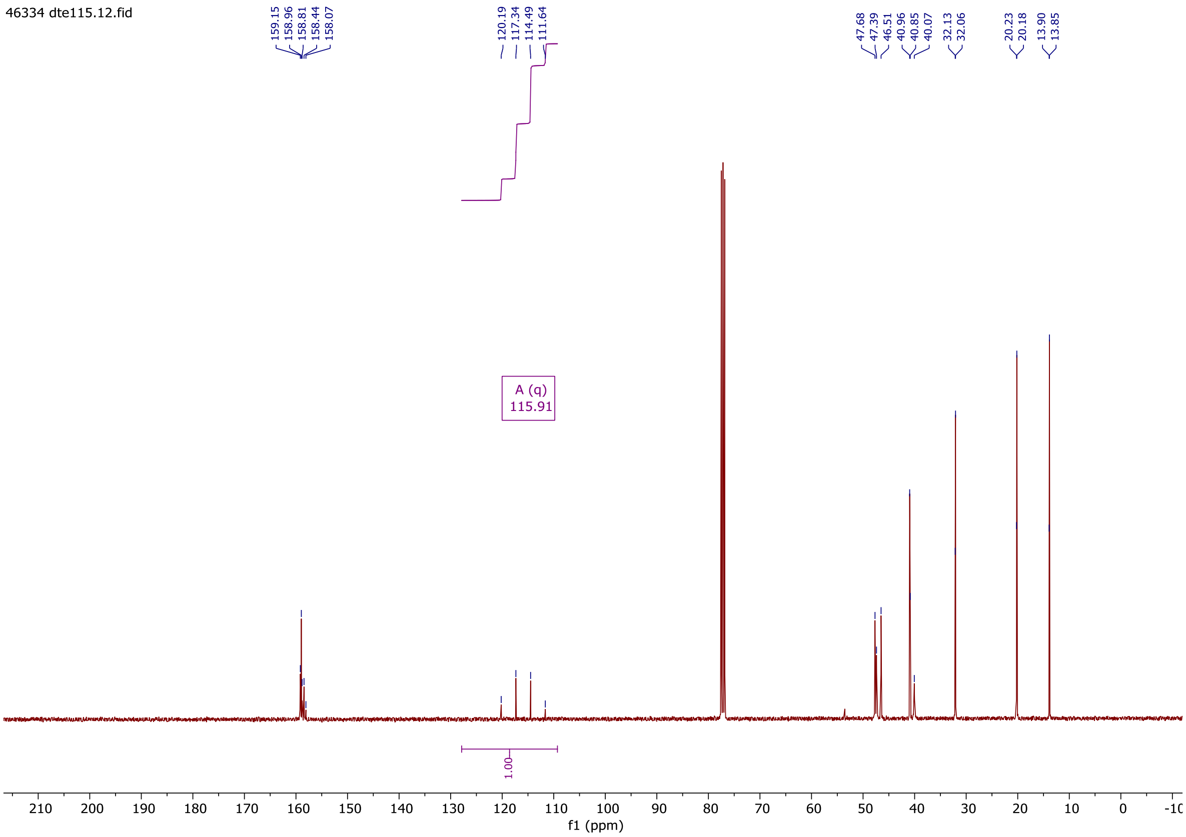

Figure S187 – ^{13}C NMR spectrum of 9-2 (101 MHz, CDCl$_3$).

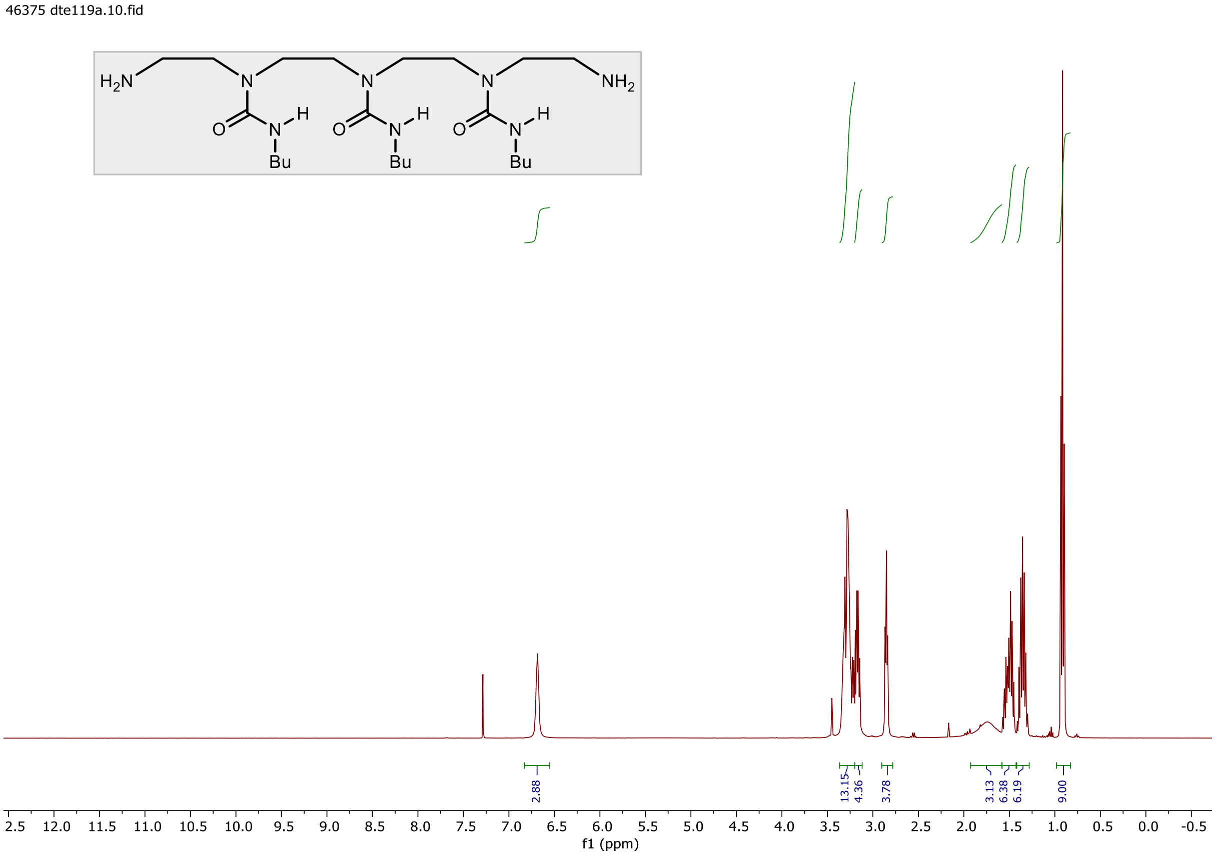

Figure S188 – ^{1}H NMR spectrum of 9-3 (400 MHz, CDCl₃).

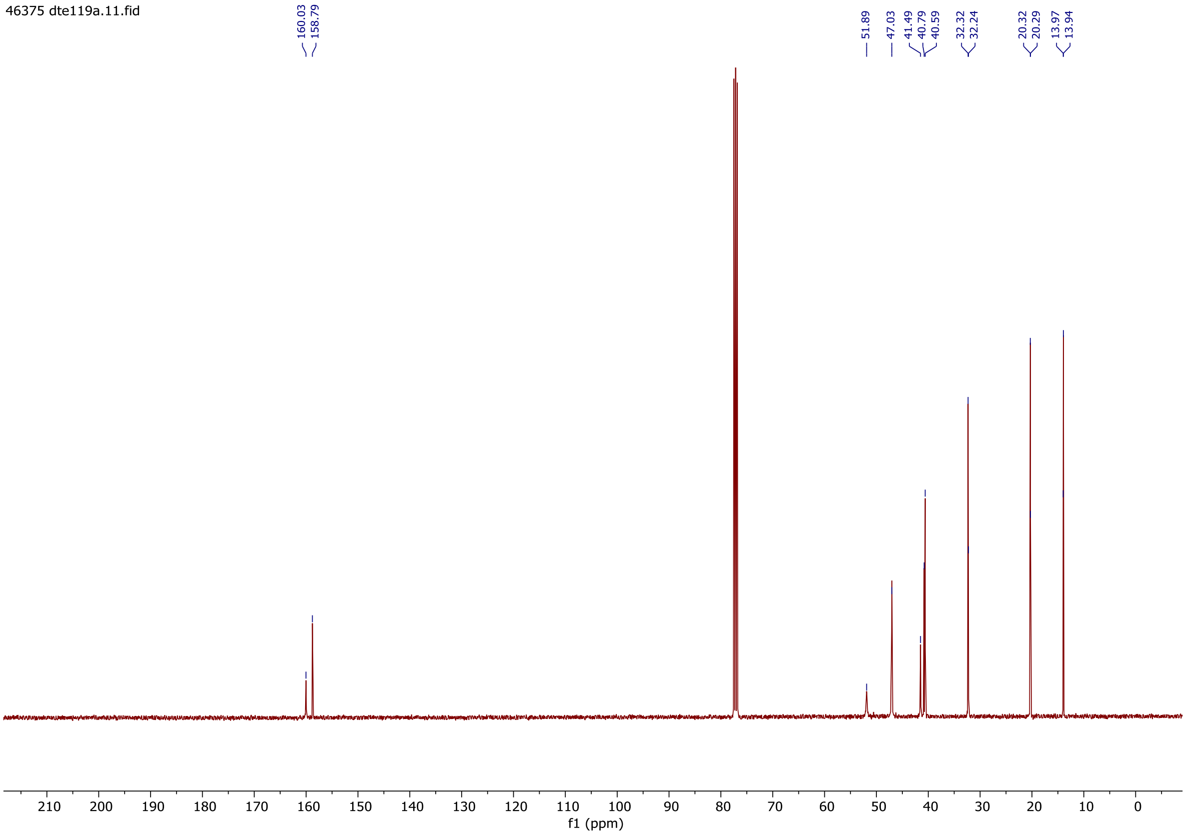

Figure S189 – ^{13}C NMR spectrum of 9-3 (101 MHz, CDCl₃).

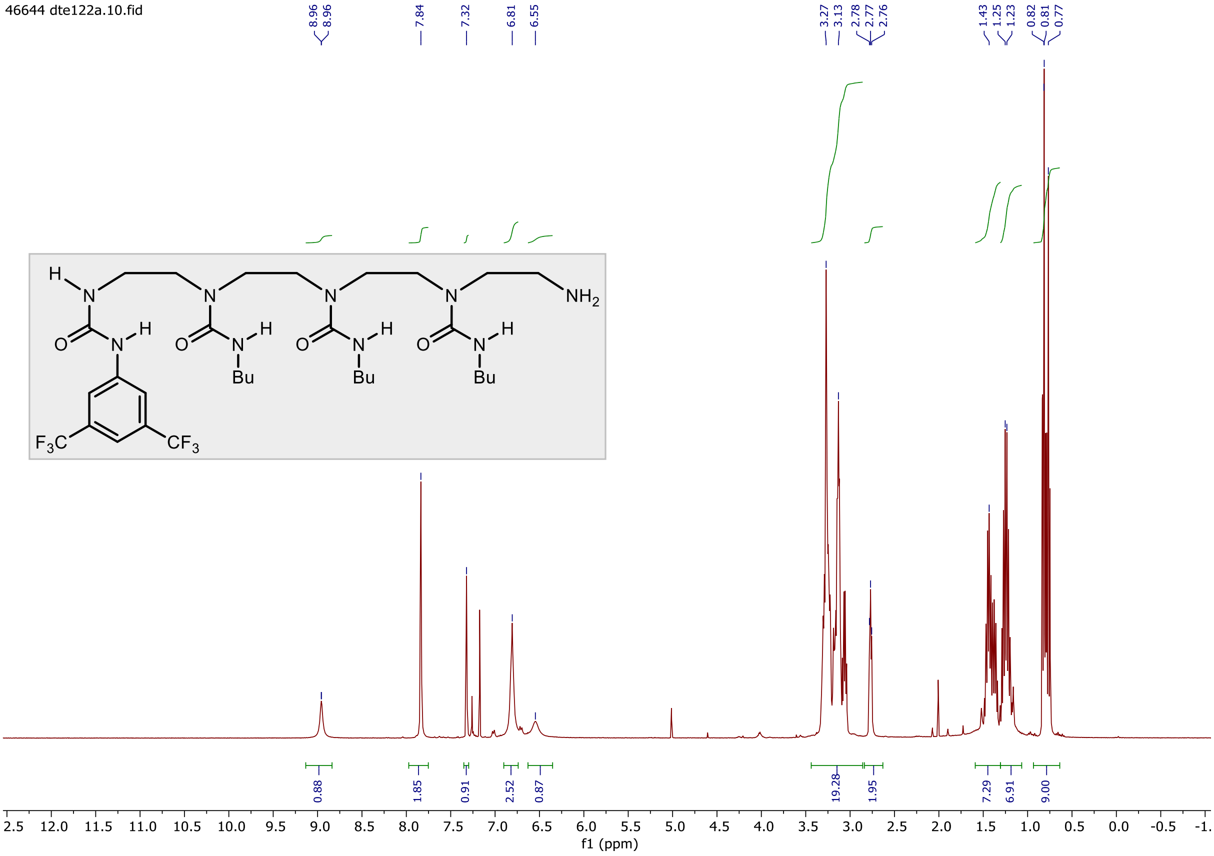

Figure S190 – ^{1}H NMR spectrum of 9-4 (400 MHz, $CDCl_3$).

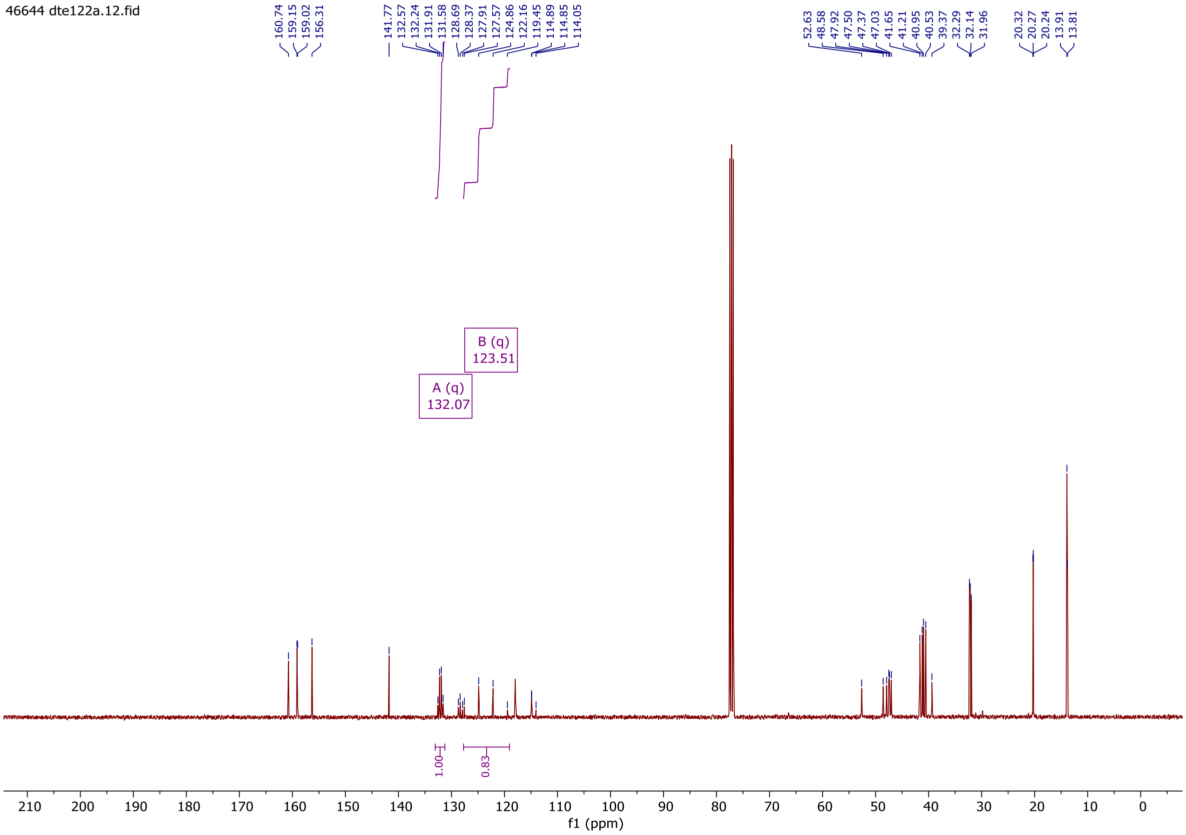

Figure S191 – ^{13}C NMR spectrum of 9-4 (101 MHz, $CDCl_3$).

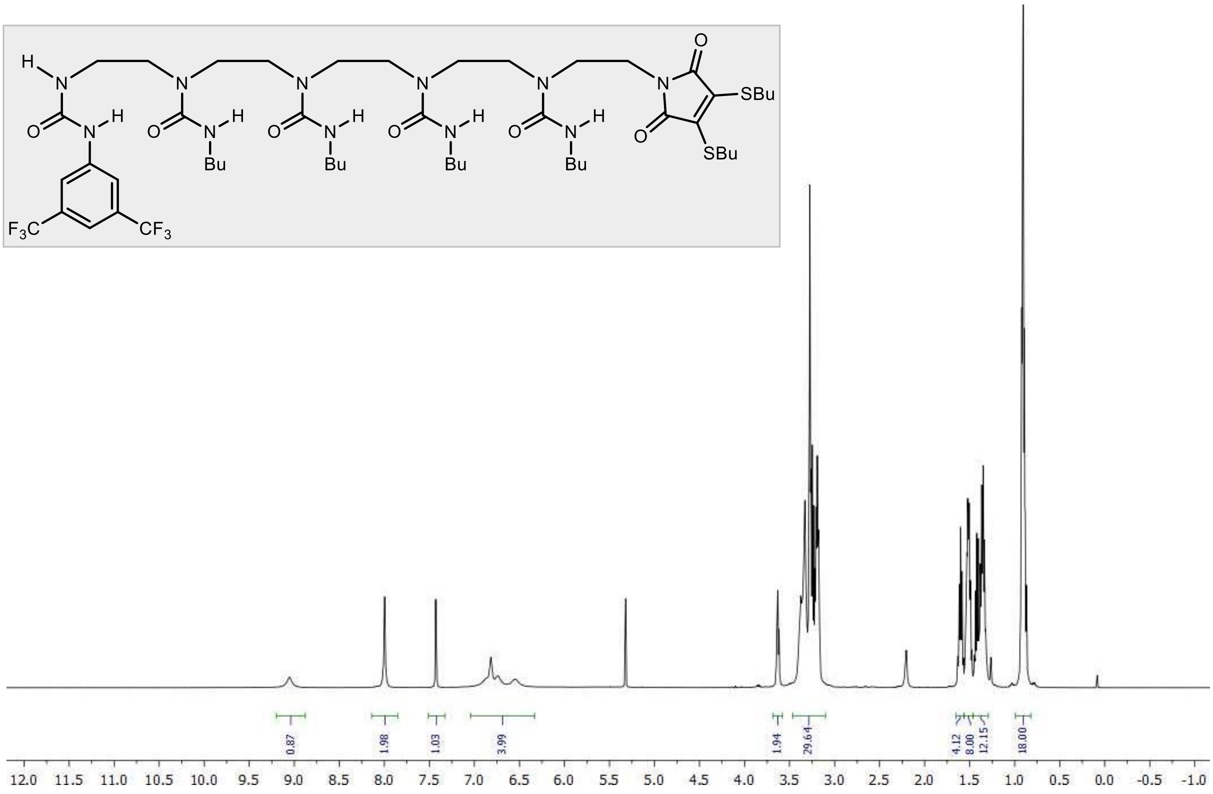

Figure S192 – ^{1}H NMR spectrum of 9 (500 MHz, CD_2Cl_2).

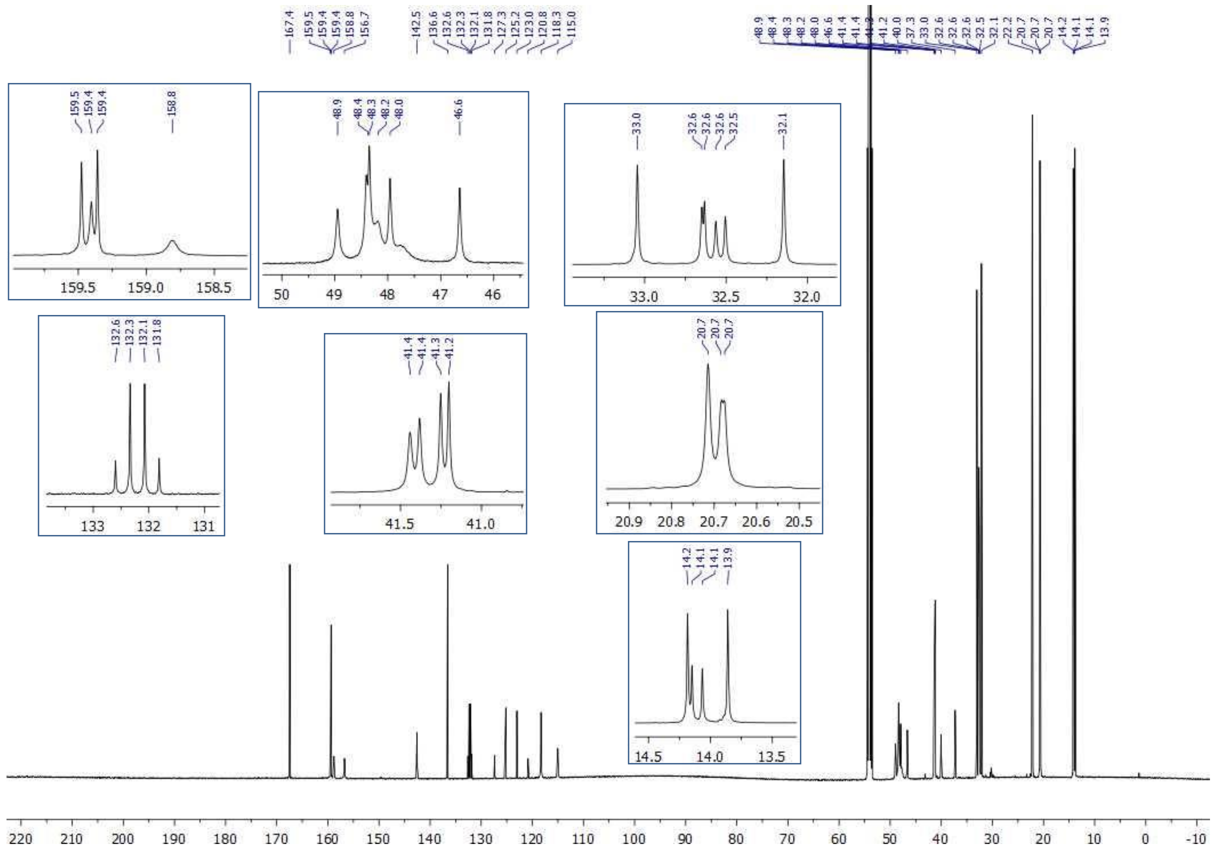

Figure S193 – ^{13}C NMR spectrum of 9 (126 MHz, CD_2Cl_2).

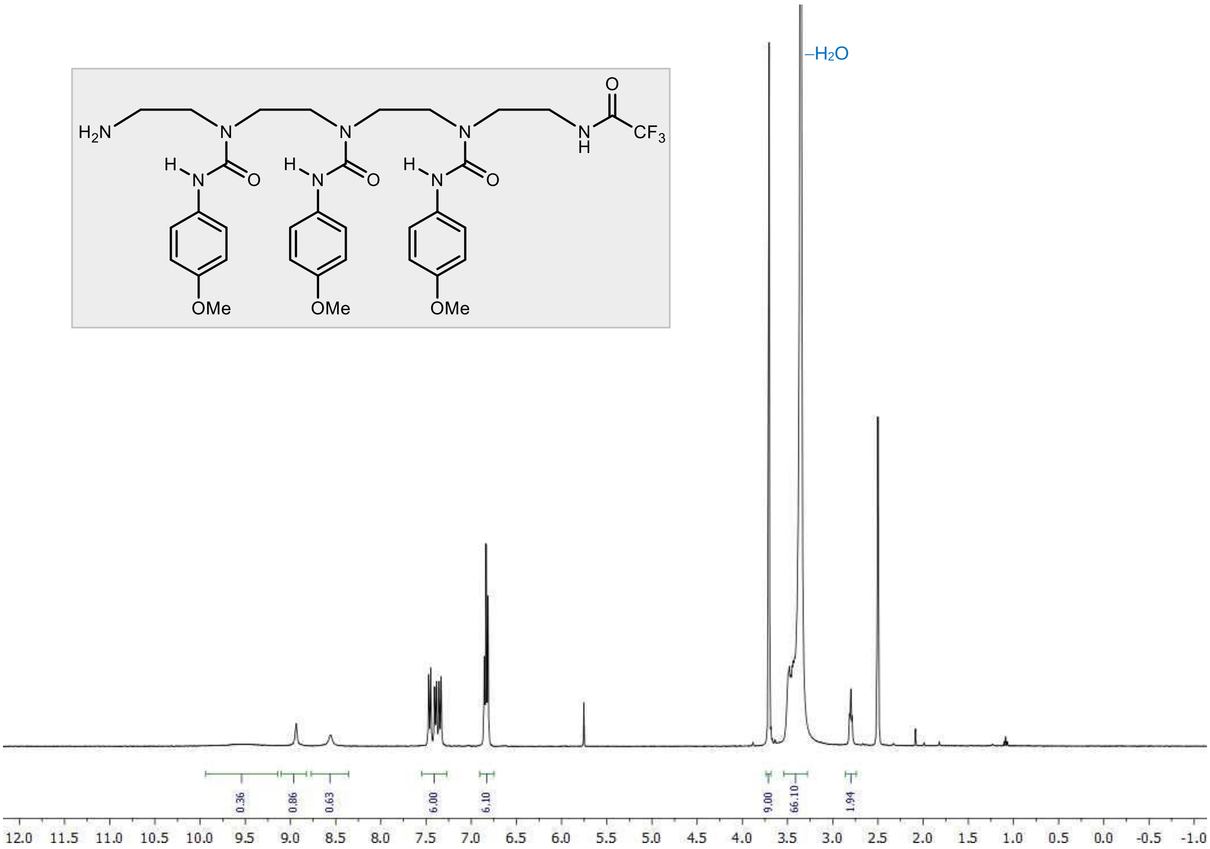

Figure S194 – ^{1}H NMR spectrum of 10-1 (400 MHz, $(CD_3)_2SO$).

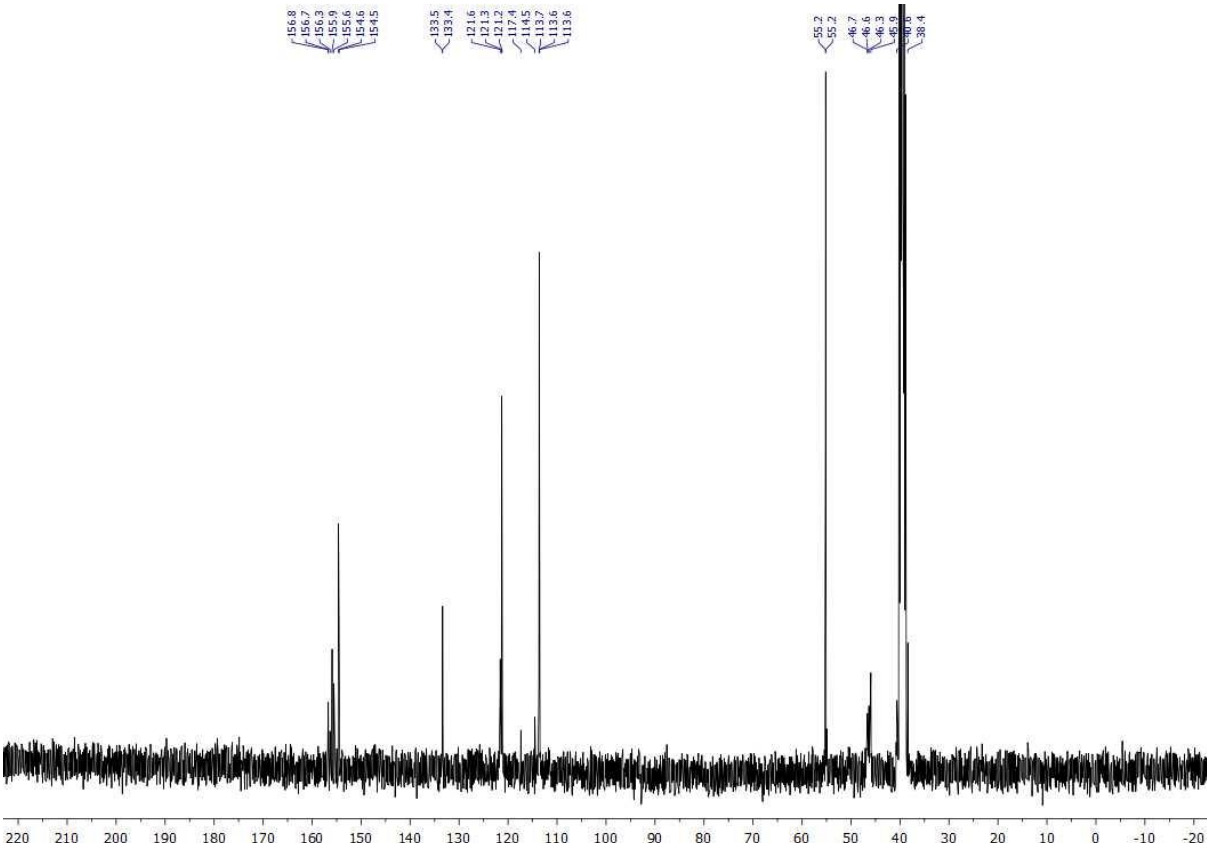

Figure S195 – ^{13}C NMR spectrum of 10-1 (101 MHz, $(CD_3)_2SO$).

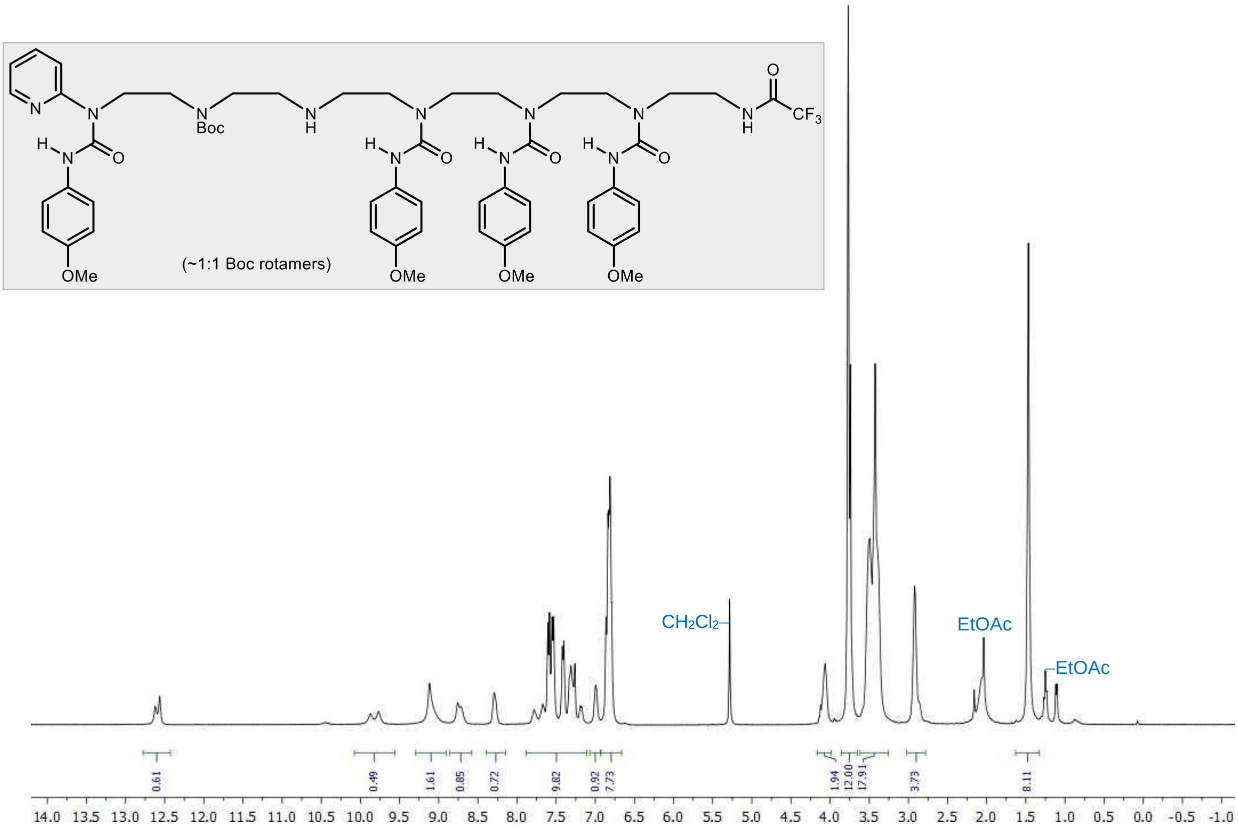

Figure S196 – ^{1}H NMR spectrum of 10-2 (400 MHz, $CDCl_3$).

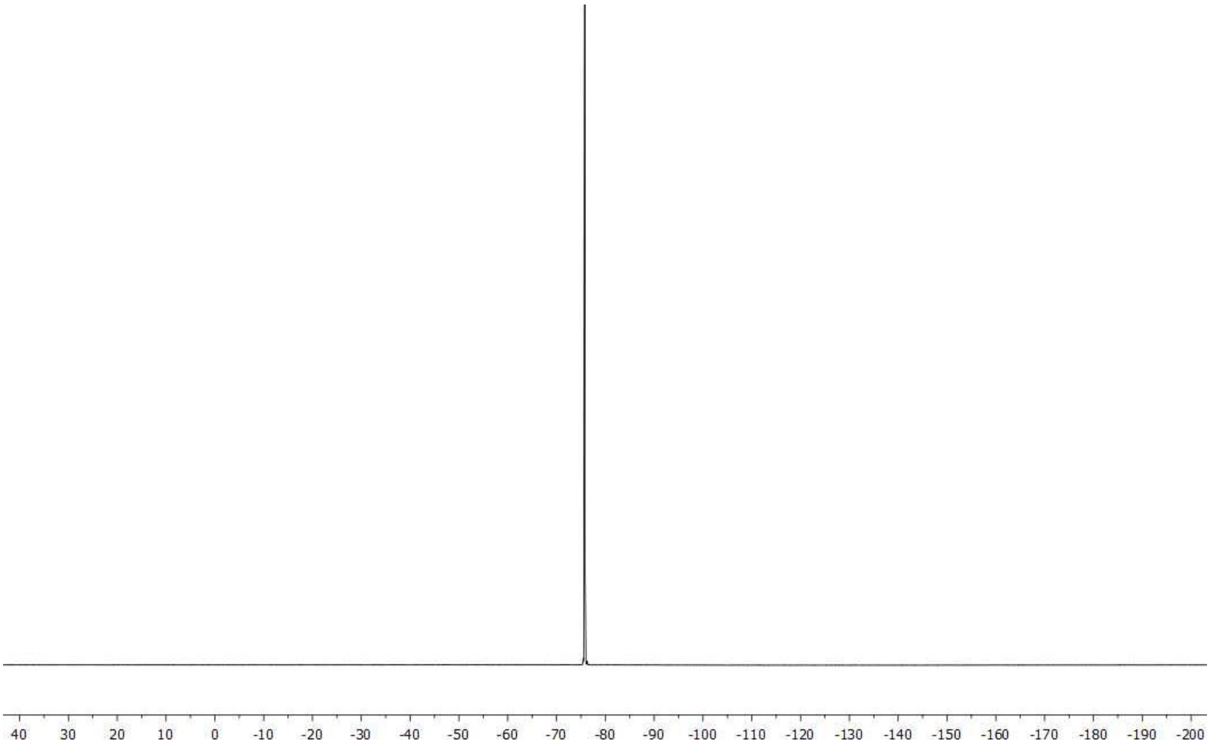

Figure S197 – ^{19}F NMR spectrum of 10-2 (376 MHz, $CDCl_3$).

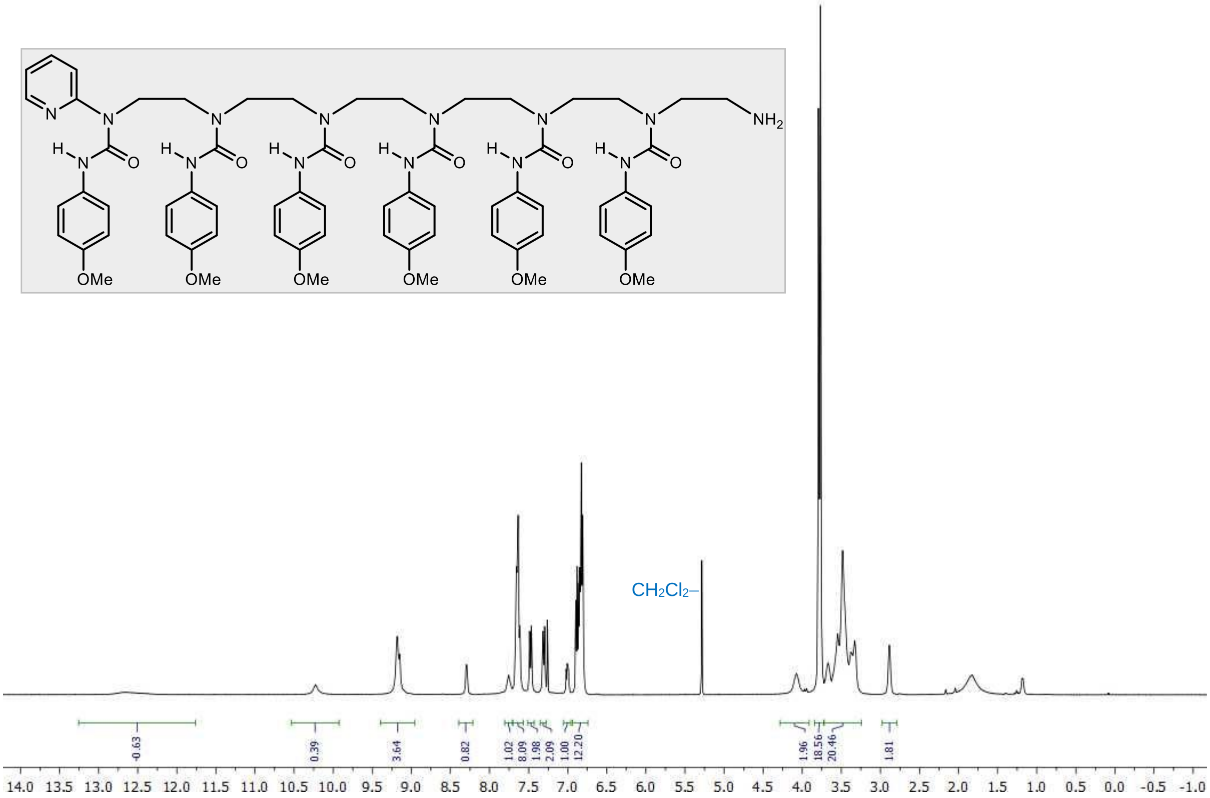

Figure S198 – ^{1}H NMR spectrum of 10-3 (400 MHz, $CDCl_3$).

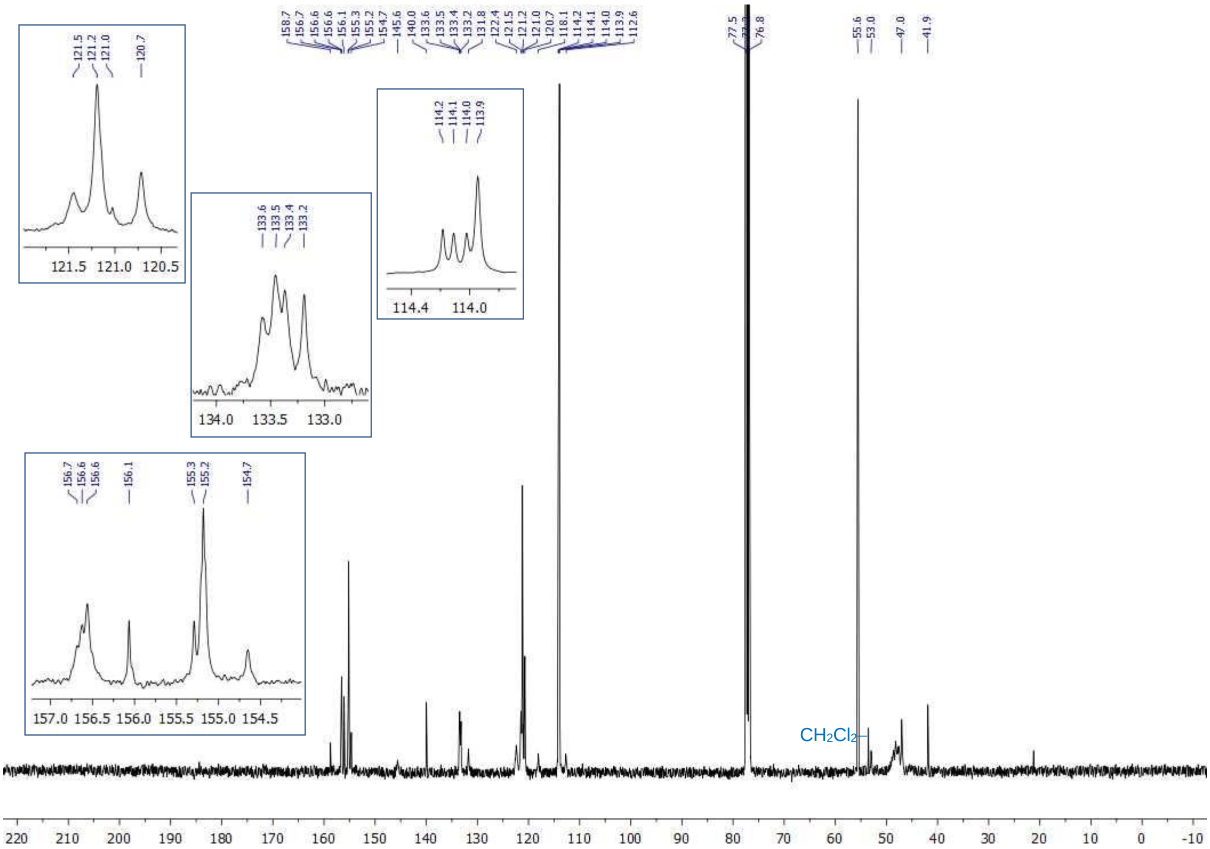

Figure S199 – ^{13}C NMR spectrum of 10-3 (101 MHz, $CDCl_3$).

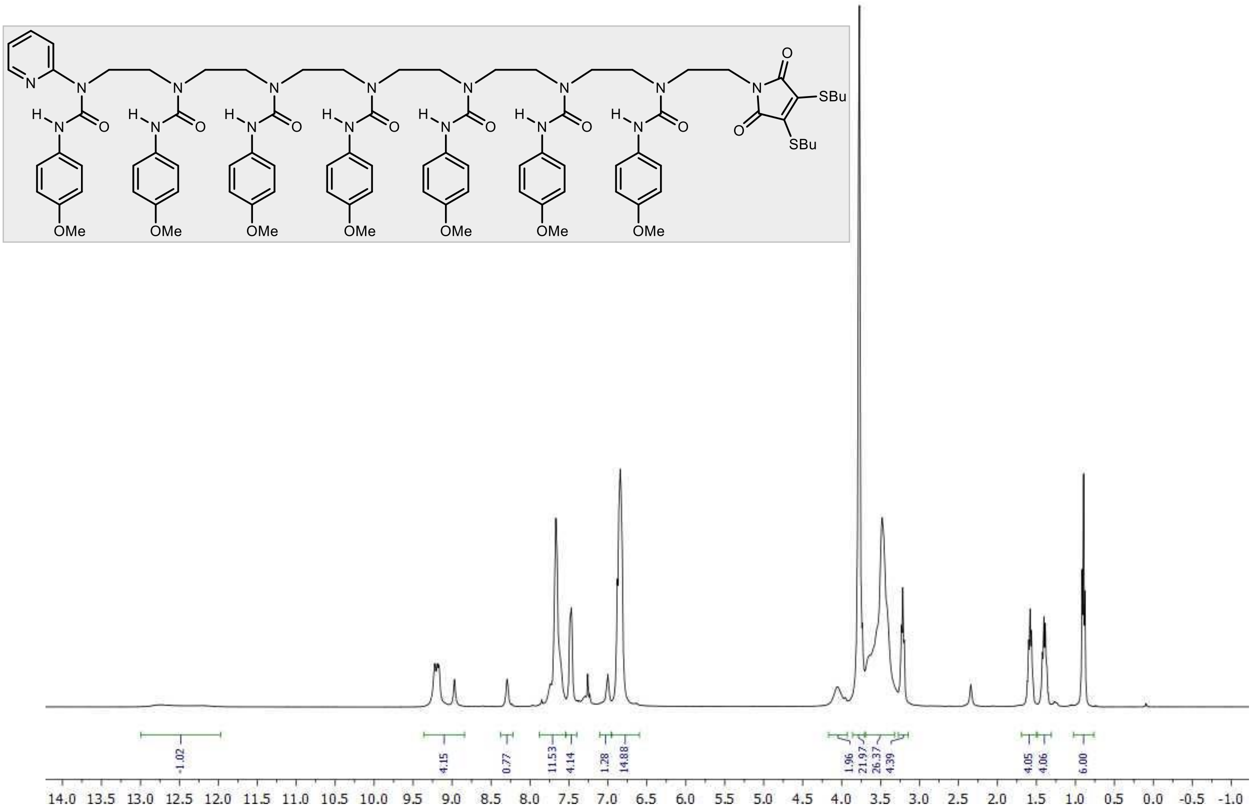

Figure S200 – ^{1}H NMR spectrum of 10 (400 MHz, $CDCl_3$).

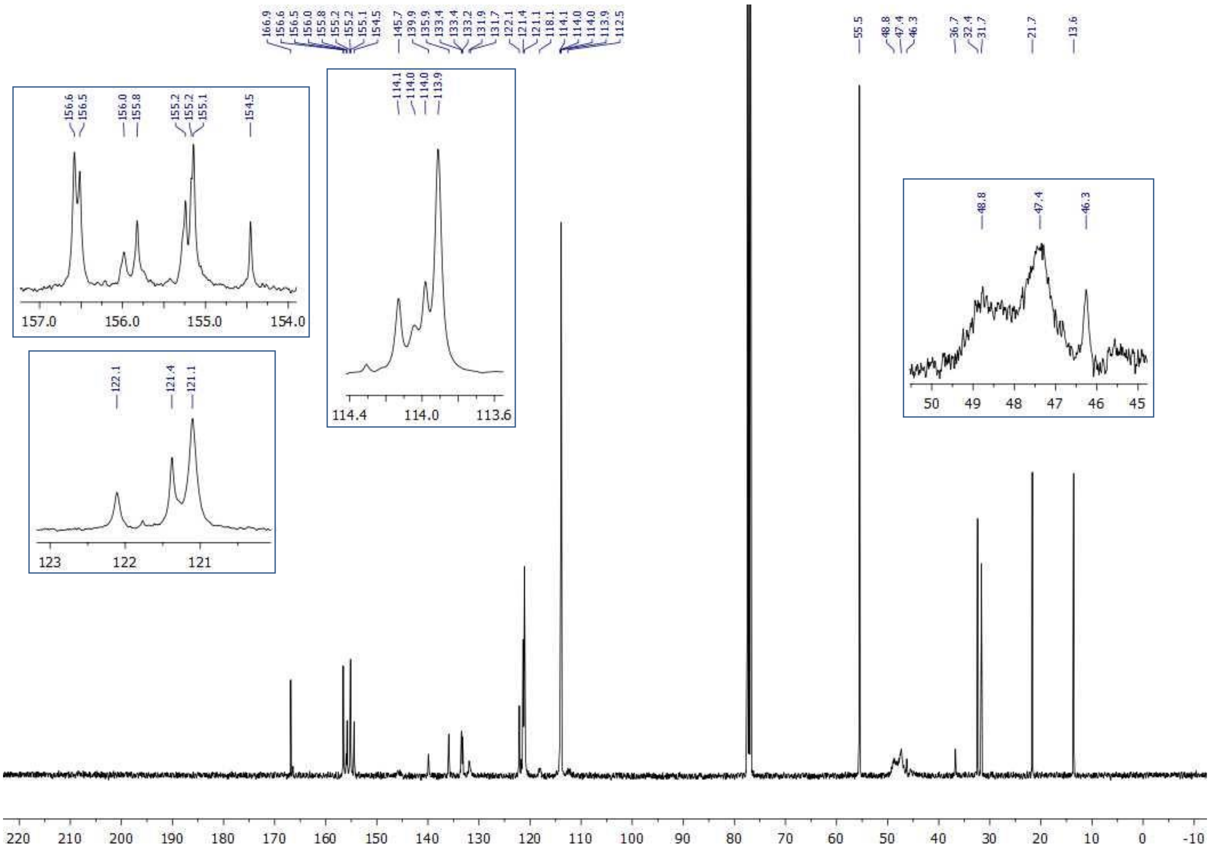

Figure S201 – ^{13}C NMR spectrum of 10 (101 MHz, $CDCl_3$).

Computational Details

Conformational searches were carried out for all computed structures using the conformational search tool within Schrödinger's MacroModel (version 11.3)[11,12] with the OPLS3e force field.[13] A Monte Carlo Multiple Minimum (MCMM)[14] / low-mode sampling approach[15] was used to explore the possible conformations of each species. Conformations provided by these searches were subsequently optimised by DFT calculations carried out using Gaussian16 (Revision A.03)[16] with the B3LYP density functional,[17,18] dispersion-corrected with the D3 version of Grimme's dispersion[19] with Becke-Johnson damping (D3(BJ)),[20-22] and a split-valence polarised 6-31G(d,p) basis set.[23]

Single point energy (SPE) calculations were used to correct the Gibbs free energy derived from the original B3LYP calculations.[24] These were performed with an ultrafine integration grid using the B3LYP-D3(BJ) density functional and the larger split-valence double polarised 6-311G(d,p) basis set.[25] The integral equation formalism version of the polarisable continuum model (IEF-PCM)[26] (dichloromethane (DCM)) was used to incorporate the effect of solvent. All temperature (298.15 K, except where specified) and concentration-corrected (1 mol/l) quasiharmonic (Grimme approximation[27]) free energies were calculated with GoodVibes[28] with a vibrational scaling factor of 0.977.[29]

Similar methods have previously been used for the successful modelling of ureas.[30,31]

Anti vs. gauche 9-membered ring conformation

A simple system **S** (Figure S202a) containing two truncated urea units was modelled in order to compare the relative free energies of the gauche and anti conformations of the 9-membered hydrogen bonded ring. The gauche conformation was found to be 2.1 kJ mol^{-1} lower in energy than the anti conformation (Figure S202b).

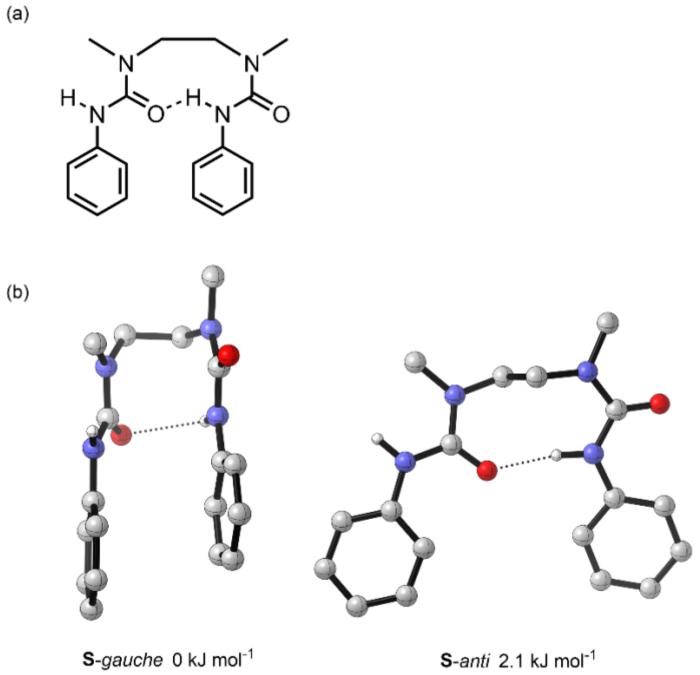

Figure S202 – Representation and simulations of diurea **S**. (a) Two-urea system **S**; (b) Computed anti and gauche conformations of **S** (B3LYP-D3(BJ)/6-311G(d,p)/IEF-PCM(DCM)//B3LYP-D3(BJ)/6-31G(d,p)).

Conformations of 4’

Modelling of **4’** (a truncated version of **4**, with Ar = Ph and benzyl substituents truncated to methyl, Figure S203) revealed a slightly greater prevalence of the gauche backbone conformation, as observed for **7c**.

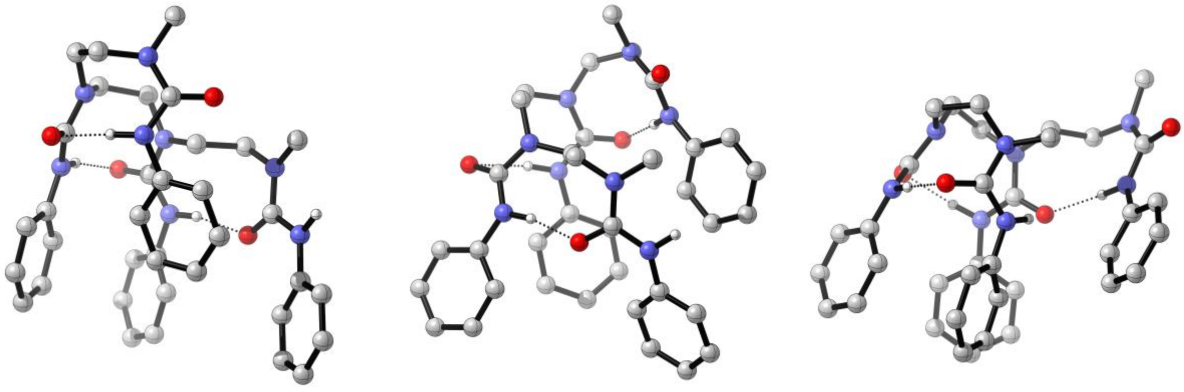

4'-*gag* 0 kJ mol^{-1} **4'**-*agg* 1.6 kJ mol^{-1} **4'**-*gga* 5.7 kJ mol^{-1}

Figure S203 – Three lowest energy conformers of **4’** with corresponding backbone conformations (*g* = gauche, *a* = anti) starting from N terminus (B3LYP-D3(BJ)/6-311G(d,p)/IEF-PCM(DCM)//B3LYP-D3(BJ)/6-31G(d,p)).

Energies and molecular geometries of included computed structures

All energies in Hartrees, coordinates in Å. Cartesian coordinates generated by ESIgen software.[32] All quasiharmonic energies were temperature-corrected at 298.15 K.

The energies and molecular geometries for all other computed structures are available upon request.

<u>S-anti</u>

B3LYP-D3(BJ)/6-31G(d,p) Energy = -1068.796201
B3LYP-D3(BJ)/6-311G(d,p)/IEF-PCM(DCM) Energy = -1069.057396
B3LYP-D3(BJ)/6-311G(d,p)/IEF-PCM(DCM)//B3LYP-D3(BJ)/6-31G(d,p) Quasiharmonic Free Energy = -1068.728710

Frequencies (Top 3 out of 132)

1. 14.4753 cm^{-1}
2. 19.0664 cm^{-1}
3. 28.3748 cm^{-1}

B3LYP-D3(BJ)/6-31G(d,p) Molecular Geometry in Cartesian Coordinates

C	3.58070	-3.28547	-0.47419
H	3.34865	-4.00632	0.32071
H	3.53984	-3.80885	-1.43783
N	2.65827	-2.16343	-0.45736
C	3.17398	-0.88736	-0.23495
O	4.38585	-0.69493	-0.16247

N	2.22601	0.10938	-0.10866
H	1.24448	-0.13691	-0.02261
C	1.24951	-2.44157	-0.64320
H	1.15532	-3.35677	-1.23736
H	0.78206	-1.64327	-1.22466
C	0.48613	-2.59768	0.68993
H	0.80600	-1.81155	1.37654
H	0.71513	-3.55671	1.16054
N	-0.96235	-2.50461	0.51636
C	-1.77169	-3.70708	0.39525
H	-1.11239	-4.57499	0.37398
H	-2.45688	-3.83705	1.24508
H	-2.35511	-3.70905	-0.53274
C	-1.46181	-1.23895	0.31568
O	-0.71347	-0.25591	0.24856
N	-2.83091	-1.15187	0.19344
H	-3.37120	-1.96511	0.44333
C	2.47399	1.48659	0.06310
C	3.72998	2.08565	-0.11583
C	1.37151	2.28793	0.40787
C	3.86229	3.46375	0.05021
H	4.57949	1.46938	-0.36818
C	1.52397	3.66151	0.56660
H	0.40240	1.82299	0.55583
C	2.77087	4.26263	0.38904
H	4.84059	3.91487	-0.08993
H	0.66000	4.26309	0.83453
C	-3.59658	0.01224	-0.03908
C	-4.97254	-0.07104	0.21596
C	-3.05662	1.20536	-0.53853
C	-5.80112	1.02141	-0.02082
H	-5.39261	-0.99566	0.60546
C	-3.89942	2.29220	-0.76511
H	-1.99758	1.27363	-0.73585
C	-5.26851	2.21355	-0.51140
H	-6.86409	0.93824	0.18325
H	-3.47141	3.21293	-1.14957
H	-5.91160	3.06836	-0.69283
H	2.88948	5.33426	0.51503
H	4.58561	-2.89711	-0.32113

<u>S-gauche</u>

B3LYP-D3(BJ)/6-31G(d,p) Energy = -1068.804266
B3LYP-D3(BJ)/6-311G(d,p)/IEF-PCM(DCM) Energy = -1069.060786
B3LYP-D3(BJ)/6-311G(d,p)/IEF-PCM(DCM)//B3LYP-D3(BJ)/6-31G(d,p) Quasiharmonic Free Energy = -1068.729494

Frequencies (Top 3 out of 132)

1. 27.1193 cm^{-1}
2. 38.6956 cm^{-1}
3. 43.9169 cm^{-1}

B3LYP-D3(BJ)/6-31G(d,p) Molecular Geometry in Cartesian Coordinates

C	-4.57791	0.83436	0.23840
H	-5.43496	0.62352	-0.40730
H	-4.64988	0.19988	1.13283
N	-3.35429	0.61070	-0.52023
C	-2.23176	1.30606	-0.07587
O	-2.30204	2.07182	0.88643
N	-1.07831	1.10932	-0.80558
H	-1.07780	0.38356	-1.50574
C	-3.28184	-0.63813	-1.26369
H	-2.51311	-0.58863	-2.03473
H	-4.23102	-0.77632	-1.79307
C	-3.02336	-1.87433	-0.37783
H	-2.83182	-2.73143	-1.02829
H	-3.90751	-2.09547	0.22465
N	-1.90213	-1.71357	0.54340
C	-2.15179	-1.32957	1.92781
H	-3.18877	-1.56008	2.17362
H	-1.52327	-1.91179	2.61024
H	-1.99750	-0.25683	2.10505
C	-0.64249	-1.71348	-0.01784
O	-0.47989	-1.93438	-1.22238
N	0.39062	-1.45166	0.85933
H	0.14616	-1.03546	1.74370
C	0.20156	1.64595	-0.55793
C	0.48420	2.57856	0.44984
C	1.23694	1.19799	-1.39391
C	1.79096	3.04526	0.59945
H	-0.31314	2.92543	1.08947
C	2.53076	1.67664	-1.23270
H	1.02553	0.44616	-2.14810
C	2.81851	2.60695	-0.23360
H	1.99967	3.76872	1.38251
H	3.31960	1.29839	-1.87398
C	1.76043	-1.35634	0.53774
C	2.57014	-0.60380	1.39694
C	2.33206	-2.00251	-0.56591
C	3.93372	-0.48613	1.15331
H	2.12100	-0.07913	2.23581
C	3.69910	-1.86958	-0.80100
H	1.70343	-2.57495	-1.23189
C	4.50859	-1.11653	0.04976
H	4.54347	0.11400	1.82123
H	4.13414	-2.36795	-1.66230
H	5.57187	-1.02057	-0.14509
H	3.83049	2.97605	-0.10209
H	-4.60930	1.87166	0.56483

4’ Conformation 1

B3LYP-D3(BJ)/6-31G(d,p) Energy = -2136.447874
B3LYP-D3(BJ)/6-311G(d,p)/IEF-PCM(DCM) Energy = -2136.947469

B3LYP-D3(BJ)/6-311G(d,p)/IEF-PCM(DCM)//B3LYP-D3(BJ)/6-31G(d,p) Quasiharmonic Free Energy = -2136.275887

Frequencies (Top 3 out of 264)

1. 12.0903 cm^{-1}
2. 16.4682 cm^{-1}
3. 25.5427 cm^{-1}

B3LYP-D3(BJ)/6-31G(d,p) Molecular Geometry in Cartesian Coordinates

C	2.970479	-0.319753	-2.452636
H	3.034539	-1.406484	-2.551550
H	3.146801	0.119826	-3.440884
C	4.100294	0.169544	-1.529381
H	5.046061	-0.189462	-1.947254
H	4.149588	1.257036	-1.571766
N	1.611738	-0.018525	-2.011201
N	4.007042	-0.294713	-0.144687
C	1.201330	1.298923	-2.045095
O	2.033997	2.203176	-2.252113
C	3.456770	0.471813	0.870578
O	3.468268	0.039646	2.037122
N	-0.123017	1.527191	-1.803277
N	2.905343	1.674748	0.529944
H	-0.770452	0.745365	-1.684997
H	2.842335	1.928466	-0.452657
C	0.742911	-1.103004	-1.573830
H	0.165033	-0.776415	-0.707615
H	1.368500	-1.922240	-1.234220
C	4.921349	-1.365121	0.280343
H	5.381398	-1.753889	-0.630801
H	5.724137	-0.946127	0.901375
C	-0.223276	-1.617299	-2.661504
H	0.286770	-2.306047	-3.339776
H	-0.592318	-0.780272	-3.256846
N	-1.370206	-2.314188	-2.086084
C	-1.300608	-3.748290	-1.824472
H	-1.223464	-3.962111	-0.754915
H	-2.158034	-4.270769	-2.267475
H	-0.394712	-4.142327	-2.281369
C	-2.348569	-1.512713	-1.556607
O	-2.280211	-0.276091	-1.625367
N	-3.423631	-2.163474	-0.993826
H	-3.341801	-3.160963	-0.879049
C	2.166040	2.540890	1.373474
C	1.511869	3.604634	0.735205
C	2.044210	2.388269	2.762417
C	0.709090	4.476423	1.461026
H	1.614321	3.724700	-0.335958
C	1.237986	3.275758	3.476791
H	2.560110	1.582536	3.260730
C	0.560649	4.314223	2.839383
H	0.187158	5.269027	0.935625
H	1.141773	3.143021	4.550406

C	-0.728593	2.785977	-1.595731
C	-0.270515	3.968988	-2.188761
C	-1.858613	2.814617	-0.765189
C	-0.929757	5.168985	-1.922784
H	0.600271	3.939896	-2.828216
C	-2.513709	4.017212	-0.521110
H	-2.204255	1.891377	-0.313361
C	-2.050464	5.204142	-1.092156
H	-0.564328	6.083931	-2.379856
H	-3.384029	4.026856	0.128345
C	-4.483133	-1.578136	-0.274186
C	-5.243530	-2.428558	0.541830
C	-4.809839	-0.216228	-0.341124
C	-6.294012	-1.926465	1.303055
H	-4.998121	-3.486896	0.589009
C	-5.862703	0.272393	0.430955
H	-4.234365	0.439184	-0.976217
C	-6.606999	-0.567271	1.259446
H	-6.864325	-2.600665	1.934512
H	-6.101306	1.330442	0.377240
H	-0.071006	4.989279	3.408154
H	-2.558166	6.143159	-0.895291
H	-7.421732	-0.171671	1.856894
C	4.319470	-2.543244	1.064831
H	5.141808	-3.248725	1.245990
H	3.983846	-2.201795	2.039533
N	3.258315	-3.261661	0.383056
C	3.668535	-4.248252	-0.601132
H	4.322524	-5.003763	-0.147113
H	2.777717	-4.734390	-0.992432
H	4.215803	-3.775373	-1.426400
C	1.911884	-3.161219	0.686360
N	1.565935	-2.169521	1.580836
H	2.254960	-1.455536	1.810648
O	1.079598	-3.900933	0.149245
C	0.228125	-1.762774	1.796698
C	-0.026958	-0.389905	1.914952
C	-0.831404	-2.672549	1.906401
C	-1.325031	0.071925	2.120501
H	0.790027	0.316075	1.846793
C	-2.125822	-2.196584	2.108651
H	-0.631949	-3.732272	1.828705
C	-2.386248	-0.828857	2.210439
H	-1.494339	1.141354	2.200689
H	-2.945670	-2.902973	2.191638
H	-3.402182	-0.476708	2.349471

<u>**4’** Conformation 2</u>

B3LYP-D3(BJ)/6-31G(d,p) Energy = -2136.448287
B3LYP-D3(BJ)/6-311G(d,p)/IEF-PCM(DCM) Energy = -2136.947461
B3LYP-D3(BJ)/6-311G(d,p)/IEF-PCM(DCM)//B3LYP-D3(BJ)/6-31G(d,p) Quasiharmonic Free Energy = -2136.275264

Frequencies (Top 3 out of 264)

1. 16.7922 cm^{-1}
2. 21.4112 cm^{-1}
3. 24.8879 cm^{-1}

B3LYP-D3(BJ)/6-31G(d,p) Molecular Geometry in Cartesian Coordinates

C	2.232213	-2.825778	-1.311286
H	2.165400	-3.679448	-1.992279
H	2.980672	-2.132363	-1.702252
C	2.703366	-3.370567	0.047873
H	1.970772	-4.093927	0.412077
H	3.620824	-3.939176	-0.123768
N	0.951970	-2.125953	-1.312459
N	3.005968	-2.350626	1.055597
C	-0.185237	-2.906449	-1.163355
O	-0.073675	-4.104089	-0.857006
C	2.046433	-1.720128	1.819034
O	2.349066	-0.734907	2.515593
N	-1.382092	-2.271695	-1.364609
N	0.778739	-2.236888	1.770944
H	-1.386630	-1.264509	-1.536660
H	0.624590	-3.056867	1.199470
C	0.928082	-0.671408	-1.227326
H	1.887003	-0.341386	-0.837819
H	0.172987	-0.364644	-0.501689
C	4.404880	-2.105276	1.419230
H	4.547938	-2.298673	2.488931
H	4.997186	-2.843274	0.874803
C	0.650340	0.030421	-2.573568
H	1.559289	0.068114	-3.175853
H	-0.101560	-0.529746	-3.136015
N	0.193333	1.402094	-2.359007
C	1.064076	2.520685	-2.702735
H	0.997180	3.311561	-1.954174
H	2.100379	2.185221	-2.673137
H	0.829275	2.929257	-3.694468
C	-1.058402	1.532244	-1.826889
O	-1.760283	0.535092	-1.585569
N	-1.502160	2.807992	-1.570181
H	-0.883756	3.567525	-1.806339
C	-0.417137	-1.619720	2.205528
C	-0.469946	-0.422704	2.934642
C	-1.609384	-2.264385	1.848645
C	-1.712521	0.135636	3.238618
H	0.446191	0.046997	3.257634
C	-2.839873	-1.699623	2.164162
H	-1.571802	-3.198654	1.299597
C	-2.899415	-0.487246	2.853775
H	-1.744006	1.070583	3.790022
H	-3.746113	-2.205690	1.849832
C	-2.669837	-2.808400	-1.157109
C	-2.925516	-4.166789	-0.915859
C	-3.742327	-1.899869	-1.189670

C	-4.235534	-4.589207	-0.686812
H	-2.103959	-4.866630	-0.901083
C	-5.041823	-2.342752	-0.968719
H	-3.543649	-0.850701	-1.375189
C	-5.300458	-3.689941	-0.707343
H	-4.418787	-5.642338	-0.493932
H	-5.856300	-1.624739	-0.994680
C	-2.545219	3.161637	-0.686873
C	-3.135425	2.265779	0.216799
C	-2.937446	4.507365	-0.679233
C	-4.103535	2.730858	1.104842
H	-2.830979	1.230089	0.231521
C	-3.901020	4.957395	0.218755
H	-2.478508	5.200465	-1.379407
C	-4.494082	4.070060	1.117242
H	-4.546937	2.027635	1.802934
H	-4.188489	6.004346	0.211934
H	-3.858055	-0.042407	3.100569
H	-6.314835	-4.032398	-0.528777
H	-5.247474	4.417921	1.816445
C	4.962034	-0.697708	1.138055
H	4.587784	-0.003909	1.884694
H	6.052849	-0.750564	1.268724
N	4.676921	-0.185567	-0.190731
C	5.499174	-0.695233	-1.274840
H	5.170373	-0.238125	-2.205789
H	6.558886	-0.453545	-1.117269
H	5.403748	-1.784398	-1.347156
C	3.911870	0.942928	-0.431095
N	3.107462	1.340566	0.621024
H	2.856861	0.650853	1.331518
O	3.932371	1.519271	-1.522008
C	2.283376	2.484292	0.578770
C	0.993438	2.395687	1.117506
C	2.736051	3.706678	0.064972
C	0.166639	3.515702	1.149263
H	0.639810	1.447392	1.504784
C	1.894197	4.817552	0.085163
H	3.728995	3.770630	-0.360643
C	0.607424	4.731643	0.624461
H	-0.832592	3.427237	1.561139
H	2.252277	5.760257	-0.318374
H	-0.044387	5.599359	0.640268

4’ Conformation 3

B3LYP-D3(BJ)/6-31G(d,p) Energy = -2136.447515
B3LYP-D3(BJ)/6-311G(d,p)/IEF-PCM(DCM) Energy = -2136.946462
B3LYP-D3(BJ)/6-311G(d,p)/IEF-PCM(DCM)//B3LYP-D3(BJ)/6-31G(d,p) Quasiharmonic Free Energy = -2136.273715

Frequencies (Top 3 out of 264)

1. 16.1269 cm^{-1}

2. 22.8771 cm^{-1}
3. 33.5560 cm^{-1}

B3LYP-D3(BJ)/6-31G(d,p) Molecular Geometry in Cartesian Coordinates

C	0.074350	-4.054180	-0.448270
H	-0.522280	-4.974650	-0.412300
H	0.993810	-4.278060	-0.994530
C	0.437330	-3.679760	1.003690
H	-0.448890	-3.719550	1.629870
H	1.126050	-4.446420	1.378290
N	-0.615710	-3.019280	-1.219950
N	1.078940	-2.383010	1.140110
C	-1.874040	-2.661040	-0.762770
O	-2.312390	-3.159750	0.288860
C	0.447860	-1.221040	1.509680
O	1.078140	-0.140780	1.499300
N	-2.558360	-1.721140	-1.486780
N	-0.855500	-1.301330	1.922680
H	-2.018230	-1.136020	-2.125610
H	-1.434230	-2.055480	1.553490
C	-0.191570	-2.860590	-2.613860
H	-0.995070	-2.400940	-3.178940
H	-0.034920	-3.857570	-3.048110
C	2.444400	-2.239320	0.648700
H	2.715770	-3.157571	0.122740
H	2.471780	-1.427090	-0.073420
C	1.112630	-2.075820	-2.842510
H	1.215040	-1.944250	-3.931220
H	1.964750	-2.684110	-2.518100
N	1.248910	-0.804940	-2.135340
C	2.578130	-0.196371	-2.297350
H	2.620811	0.766609	-1.793690
H	3.347390	-0.843111	-1.868470
H	2.811740	-0.044251	-3.360830
C	0.149440	0.055350	-2.167270
O	-0.787100	-0.095260	-2.965770
N	0.180961	1.027060	-1.209510
H	0.747931	0.835230	-0.388130
C	-1.575560	-0.218320	2.481250
C	-0.972169	0.728200	3.320420
C	-2.946350	-0.136140	2.214270
C	-1.737669	1.766490	3.847870
H	0.085061	0.660200	3.536660
C	-3.701129	0.902230	2.752480
H	-3.414410	-0.874680	1.575690
C	-3.101669	1.864390	3.565370
H	-1.260719	2.500930	4.490000
H	-4.757959	0.958270	2.514530
C	-3.763550	-1.121110	-1.046180
C	-4.779490	-1.845940	-0.411360
C	-3.922750	0.251030	-1.275580
C	-5.925410	-1.180600	0.023390
H	-4.654280	-2.905260	-0.235910
C	-5.074279	0.900910	-0.842710

H	-3.134069	0.806970	-1.767810
C	-6.079160	0.191600	-0.182990
H	-6.705170	-1.744920	0.526050
H	-5.167909	1.969250	-1.008360
C	-0.779939	2.054860	-1.081080
C	-1.157659	2.451190	0.205360
C	-1.339769	2.683150	-2.199880
C	-2.106919	3.455810	0.373120
H	-0.730819	1.952920	1.065960
C	-2.296499	3.680070	-2.019220
H	-1.045039	2.375320	-3.194320
C	-2.687499	4.070530	-0.736740
H	-2.402639	3.734680	1.378510
H	-2.734339	4.157620	-2.890530
H	-3.689409	2.677740	3.979300
H	-6.974219	0.699730	0.162120
H	-3.433919	4.847610	-0.605580
C	3.465580	-1.956161	1.769490
H	3.039240	-1.240481	2.475580
H	3.668480	-2.866701	2.339770
N	4.728430	-1.465321	1.241830
C	5.752600	-2.453431	0.938410
H	5.443720	-3.141411	0.138020
H	6.648580	-1.929411	0.612880
H	5.974380	-3.041991	1.834350
C	4.863960	-0.206691	0.658310
N	3.808971	0.655829	0.884110
H	2.967800	0.299059	1.327880
O	5.865720	0.092629	0.009870
C	3.629461	1.936899	0.325600
C	2.452591	2.617960	0.686500
C	4.516961	2.538099	-0.581020
C	2.151191	3.856120	0.128840
H	1.771581	2.160800	1.395940
C	4.202221	3.785029	-1.121920
H	5.427621	2.023719	-0.847880
C	3.023931	4.449719	-0.783610
H	1.223061	4.346700	0.404000
H	4.895561	4.237499	-1.825190
H	2.789551	5.414549	-1.221570

6c Conformation 1

B3LYP-D3(BJ)/6-31G(d,p) Energy = -3243.412998
B3LYP-D3(BJ)/6-311G(d,p)/IEF-PCM(DCM) Energy = -3244.160209
B3LYP-D3(BJ)/6-311G(d,p)/IEF-PCM(DCM)//B3LYP-D3(BJ)/6-31G(d,p) Quasiharmonic Free Energy = -3243.117092

Frequencies (Top 3 out of 405)

1. 21.0484 cm^{-1}
2. 24.1099 cm^{-1}

3. 27.5837 cm^{-1}

B3LYP-D3(BJ)/6-31G(d,p) Molecular Geometry in Cartesian Coordinates

C	-0.279648	-2.355445	-3.451955
H	-0.740615	-2.976082	-4.226443
H	0.311125	-1.583836	-3.954094
C	0.649272	-3.255118	-2.616084
H	0.110688	-4.154345	-2.322762
H	1.480565	-3.579274	-3.254206
N	-1.315836	-1.663909	-2.694587
N	1.198147	-2.602278	-1.435774
C	-2.386345	-2.407254	-2.238084
O	-2.316207	-3.651504	-2.234387
C	0.752530	-2.807076	-0.145832
O	1.397860	-2.344677	0.812009
N	-3.465857	-1.700699	-1.790991
N	-0.393835	-3.534471	0.011903
H	-3.484187	-0.683852	-1.904236
H	-0.992339	-3.696350	-0.796931
C	-1.114210	-0.263510	-2.348175
H	-0.042623	-0.100049	-2.235577
H	-1.557994	-0.072228	-1.370064
C	2.439164	-1.848250	-1.565936
H	2.510077	-1.463632	-2.584372
H	2.404541	-0.984522	-0.903819
C	-1.702273	0.723900	-3.380868
H	-2.627662	0.311646	-3.786931
H	-1.008196	0.871368	-4.212817
N	-1.984362	2.030394	-2.798292
C	-0.966541	3.066553	-2.891380
H	-0.650750	3.138309	-3.938895
H	-1.407424	4.027983	-2.639504
C	-3.084417	2.086252	-1.977512
O	-3.851619	1.108813	-1.892900
N	-3.297204	3.252417	-1.285352
H	-2.496955	3.864389	-1.140821
C	-0.988165	-3.870358	1.249658
C	-0.271193	-3.986685	2.447933
C	-2.364668	-4.129957	1.236640
C	-0.945925	-4.339853	3.615853
H	0.788238	-3.779079	2.464407
C	-3.025436	-4.478501	2.410073
H	-2.915513	-4.046364	0.308230
C	-2.320784	-4.582473	3.611105
H	-0.384197	-4.422131	4.541665
H	-4.096076	-4.655481	2.376973
C	-4.583865	-2.217394	-1.099023
C	-4.953188	-3.570160	-1.111170
C	-5.356155	-1.295734	-0.372257
C	-6.055029	-3.987533	-0.363517
H	-4.373948	-4.273936	-1.690231
C	-6.457803	-1.728898	0.358716
H	-5.083731	-0.246405	-0.387947
C	-6.809824	-3.079530	0.378413

H	-6.326762	-5.039030	-0.373487
H	-7.037303	-1.002864	0.920953
C	-4.345292	3.485935	-0.367630
C	-4.133650	4.504286	0.574973
C	-5.562695	2.790897	-0.373799
C	-5.116101	4.806482	1.512410
H	-3.190498	5.040944	0.565328
C	-6.534083	3.103970	0.576934
H	-5.737285	2.020407	-1.109181
C	-6.321931	4.103350	1.525523
H	-4.934916	5.595489	2.236232
H	-7.472226	2.556827	0.565935
H	-2.834415	-4.848192	4.529477
H	-7.664874	-3.416975	0.955377
H	-7.085913	4.335475	2.260412
C	3.667175	-2.713575	-1.210203
H	3.416157	-3.336710	-0.347227
H	3.897212	-3.397411	-2.030708
N	4.861225	-1.930480	-0.922973
C	5.960662	-1.880583	-1.884387
H	5.734411	-2.618386	-2.657517
H	6.893637	-2.180760	-1.396874
C	5.063519	-1.296993	0.288644
N	4.077990	-1.435994	1.230437
H	3.174528	-1.837347	0.970435
O	6.095882	-0.625397	0.462295
C	4.083065	-0.845353	2.514840
C	5.258842	-0.495846	3.193928
C	2.837742	-0.629062	3.124575
C	5.175656	0.094475	4.454380
H	6.216676	-0.662475	2.723705
C	2.776072	-0.053558	4.390453
H	1.930392	-0.908541	2.601237
C	3.940835	0.322188	5.061480
H	6.092290	0.371895	4.966297
H	1.808255	0.107262	4.855109
H	3.886807	0.778089	6.044939
C	6.192756	-0.502914	-2.539053
N	4.982134	0.117174	-3.044084
C	4.463574	-0.396453	-4.299357
H	4.134723	-1.441706	-4.212342
H	3.615101	0.215230	-4.597880
H	5.240618	-0.350437	-5.070527
C	4.221270	1.044029	-2.352559
O	3.144112	1.447845	-2.807949
N	4.753033	1.494602	-1.160074
H	5.432088	0.906402	-0.686415
C	4.105822	2.407564	-0.293858
C	4.276644	2.223806	1.083975
C	3.331357	3.477115	-0.757919
C	3.621276	3.053746	1.989390
H	4.905756	1.418585	1.440635
C	2.674326	4.299151	0.156970
H	3.218698	3.629883	-1.821572
C	2.799050	4.084930	1.530724

H	3.745454	2.871601	3.052233
H	2.037039	5.098239	-0.208571
H	2.268730	4.720529	2.232558
C	0.293983	2.817108	-2.046636
N	0.055237	2.375199	-0.668526
C	0.916894	1.304061	-0.143872
H	0.356309	0.397016	0.091272
H	1.431362	1.636653	0.757207
H	1.670421	1.086834	-0.897235
C	-0.514370	3.301572	0.168723
O	-0.835715	4.431650	-0.252395
N	-0.704123	2.941810	1.490060
C	-1.079937	1.616644	1.888527
C	-2.161325	0.970704	1.281929
C	-0.382903	0.987740	2.923107
C	-2.525684	-0.310806	1.696203
H	-2.720613	1.469770	0.502493
C	-0.767712	-0.282426	3.346997
H	0.475023	1.486498	3.361436
C	-1.838119	-0.935780	2.735337
H	-3.361428	-0.813870	1.222952
H	-0.220562	-0.778412	4.142420
H	-2.132468	-1.923912	3.063684
H	0.915641	2.058950	-2.518707
H	0.872043	3.746339	-2.053907
H	6.689988	0.158459	-1.833458
H	6.886083	-0.639899	-3.378300
C	-1.053078	4.008121	2.430342
H	-0.625160	3.770340	3.406652
H	-0.640563	4.947565	2.066385
H	-2.138650	4.110321	2.535193

6c Conformation 2

B3LYP-D3(BJ)/6-31G(d,p) Energy = -3243.411078
B3LYP-D3(BJ)/6-311G(d,p)/IEF-PCM(DCM) Energy = -3244.156867
B3LYP-D3(BJ)/6-311G(d,p)/IEF-PCM(DCM)//B3LYP-D3(BJ)/6-31G(d,p) Quasiharmonic Free Energy = -3243.115423

Frequencies (Top 3 out of 405)

1. 14.9547 cm^{-1}
2. 21.4245 cm^{-1}
3. 22.8086 cm^{-1}

B3LYP-D3(BJ)/6-31G(d,p) Molecular Geometry in Cartesian Coordinates

C	0.293292	-4.270385	-1.384771
H	-0.614429	-3.958620	-1.908593
H	0.594235	-5.246412	-1.792484
C	-0.063526	-4.505460	0.093804
H	-0.791751	-5.326059	0.101728
H	0.806779	-4.859611	0.637150
N	1.310960	-3.260206	-1.703646

N	-0.672483	-3.378301	0.786957
C	2.563952	-3.408476	-1.138853
O	2.744037	-4.237257	-0.226637
C	-0.037426	-2.507756	1.649313
O	-0.708520	-1.660518	2.268913
N	3.566913	-2.595759	-1.599538
N	1.318145	-2.611769	1.748354
H	3.317857	-1.788550	-2.169097
H	1.795867	-3.277641	1.142632
C	1.083057	-2.483706	-2.925565
H	0.482976	-3.086771	-3.615918
H	2.032197	-2.302350	-3.425476
C	-2.078048	-3.113587	0.530450
H	-2.354828	-3.647641	-0.374287
H	-2.222535	-2.052907	0.318415
C	0.353005	-1.146481	-2.700460
H	-0.707675	-1.330092	-2.514909
H	0.432161	-0.566690	-3.626349
N	0.863387	-0.374957	-1.570719
C	-0.037161	-0.143909	-0.446997
H	-0.709473	-0.997482	-0.399556
H	0.508390	-0.132381	0.493766
C	2.108456	0.183373	-1.677431
O	2.800398	-0.013096	-2.697307
N	2.551549	0.905494	-0.600379
H	1.956789	0.989965	0.219028
C	2.198125	-1.727744	2.416859
C	3.567309	-1.952888	2.202938
C	1.791555	-0.664752	3.235192
C	4.517860	-1.126195	2.788510
H	3.880196	-2.771368	1.566552
C	2.759945	0.150556	3.823721
H	0.742537	-0.485988	3.406697
C	4.120090	-0.069406	3.610629
H	5.567757	-1.309061	2.584998
H	2.432863	0.974196	4.450978
C	4.849826	-2.479869	-1.008500
C	5.486692	-1.237898	-1.128939
C	5.496169	-3.527613	-0.339206
C	6.729278	-1.024204	-0.542961
H	4.992875	-0.439723	-1.667169
C	6.743175	-3.299332	0.243629
H	5.013837	-4.490027	-0.254292
C	7.363400	-2.052072	0.157145
H	7.186581	-0.043096	-0.625562
H	7.232317	-4.112839	0.771400
C	3.740893	1.652826	-0.516720
C	4.456347	2.104902	-1.634289
C	4.192639	1.971431	0.771991
C	5.625791	2.841555	-1.446727
H	4.107957	1.855849	-2.626406
C	5.355095	2.716467	0.941205
H	3.639648	1.609371	1.630643
C	6.085169	3.152306	-0.166336
H	6.179166	3.179136	-2.318196

H	5.696825	2.943472	1.946759
H	4.860432	0.578956	4.068937
H	8.330387	-1.887119	0.622079
H	6.997292	3.725403	-0.033647
C	-3.012812	-3.504891	1.688328
H	-3.147080	-4.588342	1.727717
H	-2.562864	-3.209913	2.638707
N	-4.325976	-2.887405	1.536270
C	-5.441356	-3.666024	1.003695
H	-6.331945	-3.487836	1.613710
H	-5.167177	-4.717940	1.113915
C	-4.539950	-1.538619	1.742285
N	-3.502121	-0.823944	2.285122
H	-2.579172	-1.254956	2.355515
O	-5.629715	-1.033605	1.416108
C	-3.489258	0.570552	2.519820
C	-2.231253	1.180037	2.648321
C	-4.648791	1.351558	2.641006
C	-2.132994	2.552761	2.857902
H	-1.331259	0.583073	2.560940
C	-4.532443	2.727103	2.841805
H	-5.618609	0.884488	2.556445
C	-3.282686	3.339562	2.945609
H	-1.143829	2.991645	2.930075
H	-5.437363	3.322246	2.923648
H	-3.205420	4.411414	3.099109
C	-5.822874	-3.380944	-0.465639
N	-4.704864	-3.390682	-1.394553
C	-4.241499	-4.691567	-1.845382
H	-5.078106	-5.263404	-2.262452
H	-3.486426	-4.542222	-2.614265
H	-3.805844	-5.278220	-1.024617
C	-4.037826	-2.262360	-1.843917
O	-3.073462	-2.347283	-2.614049
N	-4.526376	-1.061887	-1.373458
H	-5.105341	-1.059159	-0.539124
C	-4.116906	0.222797	-1.780090
C	-3.519965	0.493338	-3.020245
C	-4.387326	1.278582	-0.896550
C	-3.199050	1.810948	-3.350890
H	-3.307339	-0.319532	-3.699784
C	-4.052258	2.583704	-1.238054
H	-4.849889	1.066609	0.060063
C	-3.456707	2.862287	-2.469193
H	-2.736907	2.012777	-4.312813
H	-4.253761	3.379385	-0.528177
H	-3.193270	3.881615	-2.730623
C	-0.888306	1.117737	-0.587994
N	-0.140305	2.367843	-0.710048
C	0.061558	2.931704	-2.040222
H	0.998534	3.489074	-2.061899
H	0.133015	2.110989	-2.756988
H	-0.754927	3.592635	-2.344311
C	0.457255	2.875456	0.410795
O	0.758984	2.158893	1.380728

N	0.762594	4.229001	0.392630
C	-0.153600	5.172734	-0.165629
C	-1.533812	5.018978	0.011148
C	0.333311	6.264508	-0.891541
C	-2.413208	5.940655	-0.554061
H	-1.914108	4.177640	0.578588
C	-0.550983	7.193412	-1.436577
H	1.403348	6.367910	-1.041189
C	-1.928216	7.032067	-1.276433
H	-3.482350	5.811235	-0.414841
H	-0.162910	8.035771	-2.000856
H	-2.616491	7.750857	-1.709436
H	-1.515967	1.036766	-1.473518
H	-1.552759	1.182405	0.274141
H	-6.535045	-4.153846	-0.780571
H	-6.352857	-2.433005	-0.525029
C	1.660860	4.700487	1.450609
H	1.150757	4.752827	2.419645
H	2.507499	4.020126	1.533227
H	2.021711	5.694430	1.184991

6c Conformation 3

B3LYP-D3(BJ)/6-31G(d,p) Energy = -3243.410674
B3LYP-D3(BJ)/6-311G(d,p)/IEF-PCM(DCM) Energy = -3244.156034
B3LYP-D3(BJ)/6-311G(d,p)/IEF-PCM(DCM)//B3LYP-D3(BJ)/6-31G(d,p) Quasiharmonic Free Energy = -3243.114514

Frequencies (Top 3 out of 405)

1. 15.8495 cm^{-1}
2. 19.2611 cm^{-1}
3. 20.6169 cm^{-1}

B3LYP-D3(BJ)/6-31G(d,p) Molecular Geometry in Cartesian Coordinates

C	-0.059102	-4.322943	-1.304323
H	-0.313361	-5.321104	-1.689716
H	0.836521	-3.983265	-1.831490
C	0.300404	-4.508113	0.181105
H	-0.555916	-4.888108	0.729101
H	1.063563	-5.295954	0.209366
N	-1.119131	-3.367753	-1.654040
N	0.856710	-3.341239	0.852601
C	-2.366933	-3.552858	-1.089172
O	-2.516568	-4.361100	-0.153472
C	0.181364	-2.482638	1.696500
O	0.812176	-1.592430	2.297574
N	-3.401994	-2.798144	-1.578151
N	-1.168308	-2.647463	1.797633
H	-3.185472	-2.001567	-2.174195
H	-1.614544	-3.342722	1.200776

C	-0.919414	-2.614116	-2.895422
H	-1.872517	-2.492846	-3.406342
H	-0.286314	-3.206016	-3.565422
C	2.246500	-3.012412	0.584882
H	2.548081	-3.555609	-0.306349
H	2.336189	-1.951631	0.345052
C	-0.255748	-1.237954	-2.700489
H	-0.364624	-0.680986	-3.637008
H	0.812391	-1.365200	-2.512522
N	-0.805268	-0.471936	-1.585533
C	0.076179	-0.182311	-0.460158
H	-0.466123	-0.214320	0.481566
H	0.808481	-0.985101	-0.419687
C	-2.075528	0.021742	-1.703970
O	-2.743998	-0.201024	-2.733815
N	-2.568891	0.709868	-0.625734
H	-1.996619	0.796906	0.209235
C	-2.088711	-1.791348	2.448253
C	-1.731374	-0.703408	3.256136
C	-3.446204	-2.073637	2.226903
C	-2.735799	0.080987	3.825574
H	-0.691807	-0.478725	3.430221
C	-4.433508	-1.278023	2.794492
H	-3.721204	-2.911766	1.598704
C	-4.084460	-0.195190	3.605040
H	-2.446263	0.924823	4.444449
H	-5.473683	-1.505389	2.585989
C	-4.688642	-2.711411	-0.989831
C	-5.296502	-3.763107	-0.291567
C	-5.370001	-1.497018	-1.144506
C	-6.551206	-3.564559	0.285567
H	-4.779190	-4.704544	-0.180641
C	-6.619829	-1.313000	-0.563812
H	-4.905454	-0.696804	-1.705739
C	-7.216351	-2.343575	0.164768
H	-7.010663	-4.380415	0.835874
H	-7.113211	-0.352180	-0.673179
C	-3.758485	1.459921	-0.577316
C	-4.440585	1.910765	-1.716540
C	-4.242502	1.788696	0.696561
C	-5.605914	2.661759	-1.564713
H	-4.069151	1.651479	-2.697643
C	-5.400939	2.546592	0.830248
H	-3.714050	1.429891	1.571746
C	-6.095525	2.985694	-0.298776
H	-6.132240	2.999689	-2.452624
H	-5.766605	2.783646	1.825056
H	-4.853629	0.428989	4.049349
H	-8.188998	-2.201155	0.625306
H	-7.004386	3.569625	-0.193747
C	3.206168	-3.321958	1.747039
H	2.747103	-3.024081	2.691957
H	3.398559	-4.395147	1.815938
N	4.483600	-2.639795	1.566906
C	5.631703	-3.370333	1.035388

H	5.413012	-4.432594	1.168021
H	6.518562	-3.135562	1.631612
C	4.628217	-1.276464	1.735594
N	3.561921	-0.602256	2.274731
H	2.663521	-1.078368	2.366990
O	5.685976	-0.724220	1.381386
C	3.479121	0.796472	2.469939
C	4.596932	1.641751	2.541445
C	2.192940	1.341981	2.607464
C	4.410480	3.015703	2.696095
H	5.588814	1.224533	2.453193
C	2.025324	2.714481	2.770256
H	1.324109	0.696290	2.559066
C	3.131928	3.564396	2.803101
H	5.283540	3.660556	2.737584
H	1.016685	3.104226	2.851491
H	2.999138	4.635661	2.916469
C	5.983804	-3.094360	-0.442972
N	4.860664	-3.186310	-1.361398
C	4.469090	-4.520792	-1.781530
H	4.071545	-5.113196	-0.945733
H	3.702981	-4.431328	-2.548738
H	5.334744	-5.053158	-2.191407
C	4.128189	-2.107800	-1.829794
O	3.164278	-2.264814	-2.589525
N	4.550532	-0.870997	-1.390525
H	5.137847	-0.816482	-0.563696
C	4.071465	0.379495	-1.826617
C	3.440519	0.584474	-3.062662
C	4.304467	1.472959	-0.978663
C	3.048897	1.874498	-3.424465
H	3.257297	-0.257066	-3.715236
C	3.900367	2.749938	-1.351048
H	4.792500	1.311965	-0.024766
C	3.270186	2.962749	-2.578336
H	2.560431	2.025008	-4.382727
H	4.076776	3.575118	-0.668145
H	2.952649	3.959647	-2.865249
C	0.836171	1.136725	-0.591174
N	0.007686	2.340407	-0.664600
C	-0.262197	2.907217	-1.982015
H	0.540650	3.566689	-2.322868
H	-1.197105	3.466631	-1.957414
H	-0.373044	2.088516	-2.696732
C	-0.607774	2.770679	0.478606
O	-0.856795	1.994273	1.417019
N	-0.973947	4.109914	0.527990
C	-0.205832	5.135511	-0.082525
C	-0.842693	6.263811	-0.612112
C	1.193983	5.051392	-0.126566
C	-0.091352	7.286249	-1.190735
H	-1.924758	6.332554	-0.582263
C	1.933128	6.068306	-0.723625
H	1.694989	4.192704	0.302887
C	1.298147	7.191727	-1.258291

H	-0.599187	8.154406	-1.599641
H	3.015956	5.989221	-0.752666
H	1.880237	7.985311	-1.715425
H	1.515212	1.225788	0.257030
H	1.445364	1.118244	-1.492779
H	6.459104	-2.119675	-0.526031
H	6.735646	-3.832511	-0.748917
C	-2.046140	4.492491	1.449168
H	-2.984475	4.679622	0.916853
H	-2.196968	3.671257	2.146802
H	-1.754727	5.390999	1.998169

Full Gaussian Reference

Frisch, M. J.; Trucks, G. W.; Schlegel, H. B.; Scuseria, G. E.; Robb, M. A.; Cheeseman, J. R.; Scalmani, G.; Barone, V.; Mennucci, B.; Petersson, G. A.; Nakatsuji, H.; Caricato, M.; Li, X.; Hratchian, H. P.; Izmaylov, A. F.; Bloino, J.; Zheng, J.; Sonnenberg, J. L.; Hada, M.; Ehara, M.; Toyota, K.; Fukuda, R.; Hasegawa, J.; Ishida, M.; Nakajima, T.; Honda, Y.; Kitao, O.; Nakai, H.; Vreven, T.; Montgomery, J. A.; Peralta, J. E.; Ogliaro, F.; Bearpark, M.; Heyd, J. J.; Brothers, E.; Kudin, K. N.; Staroverov, V. N.; Kobayashi, R.; Normand, J.; Raghavachari, K.; A. Rendell, J. C.; Burant, S.; Iyengar, S.; Tomasi, J.; Cossi, M.; Rega, N.; Millam, J. M.; Klene, M.; Knox, J. E.; Cross, J. B.; Bakken, V.; Adamo, C.; Jaramillo, J.; Gomperts, R.; Stratmann, R. E.; Yazyev, O.; Austin, A. J.; Cammi, R.; Pomelli, C.; Ochterski, J. W.; Martin, R. L.; Morokuma, K.; Zakrzewski, V. G.; Voth, G. A.; Salvador, P.; Dannenberg, J. J.; Dapprich, S.; Daniels, A. D.; Farkas, O.; Foresman, J. B.; Ortiz, J. V.; Cioslowski, J.; Fox, D. J. Gaussian 16, Revision A.03. Gaussian, Inc.: Wallingford CT 2016.

References

1. Galaup, C., Couchet, J.-M., Bedel, S., Tisnès, P., and Picard, C. (2005). Direct Access to Terpyridine-Containing Polyazamacrocycles as Photosensitizing Ligands for Eu(III) Luminescence in Aqueous Media. J. Org. Chem. *70*, 2274–2284.

2. Nowick, J.S., Mahrus, S., Smith, E.M., and Ziller, J.W. (1996). Triurea Derivatives of Diethylenetriamine as Potential Templates for the Formation of Artificial β-Sheets1. J. Am. Chem. Soc. *118*, 1066–1072.

3. Campaña, A.G., Leigh, D.A., and Lewandowska, U. (2013). One-Dimensional Random Walk of a Synthetic Small Molecule Toward a Thermodynamic Sink. J. Am. Chem. Soc. *135*, 8639–8645.

4. Robinson, A., Thomas, G.L., Spandl, R.J., Welch, M., and Spring, D.R. (2008). Gemmacin B: bringing diversity back into focus. Org. Biomol. Chem. *6*, 2978–2981.

5. Si, C., Fales, K.R., Torrado, A., Frimpong, K., Kaoudi, T., Vandeveer, H.G., and Njoroge, F.G. (2016). Enantioselective Synthesis of 3,3-Difluoropyrrolidin-4-ol, a Valuable Building Block in Medicinal Chemistry. J. Org. Chem. *81*, 4359–4363.

6. Fujihara, T., Katafuchi, Y., Iwai, T., Terao, J., and Tsuji, Y. (2010). Palladium-Catalyzed Intermolecular Addition of Formamides to Alkynes. J. Am. Chem. Soc. *132*, 2094–2098.

7. Hamilton, S.K., and Harth, E. (2009). Molecular Dendritic Transporter Nanoparticle Vectors Provide Efficient Intracellular Delivery of Peptides. ACS Nano *3*, 402–410.

8. Zhang, G.-D., Nishiyama, N., Harada, A., Jiang, D.-L., Aida, T., and Kataoka, K. (2003). pH-

sensitive Assembly of Light-Harvesting Dendrimer Zinc Porphyrin Bearing Peripheral Groups of Primary Amine with Poly(ethylene glycol)-b-poly(aspartic acid) in Aqueous Solution. Macromolecules *36*, 1304–1309.

9. Castañeda, L., Maruani, A., Schumacher, F.F., Miranda, E., Chudasama, V., Chester, K.A., Baker, J.R., Smith, M.E.B., and Caddick, S. (2013). Acid-cleavable thiomaleamic acid linker for homogeneous antibody–drug conjugation. Chem. Commun. *49*, 8187–8189.

10. Huang, H., Yu, C., Zhang, Y., Zhang, Y., Mariano, P.S., and Wang, W. (2017). Chemo- and Regioselective Organo-Photoredox Catalyzed Hydroformylation of Styrenes via a Radical Pathway. J. Am. Chem. Soc. *139*, 9799–9802.

11. Mohamadi, F., Richards, N.G.J., Guida, W.C., Liskamp, R., Lipton, M., Caufield, C., Chang, G., Hendrickson, T., and Still, W.C. (1990). Macromodel—an integrated software system for modeling organic and bioorganic molecules using molecular mechanics. J. Comput. Chem. *11*, 440–467.

12. Macromodel, version 11.3. (2016).

13. Harder, E., Damm, W., Maple, J., Wu, C., Reboul, M., Xiang, J.Y., Wang, L., Lupyan, D., Dahlgren, M.K., Knight, J.L., et al. (2016). OPLS3: A Force Field Providing Broad Coverage of Drug-like Small Molecules and Proteins. J. Chem. Theory Comput. *12*, 281–296.

14. Chang, G., Guida, W.C., and Still, W.C. (1989). An internal-coordinate Monte Carlo method for searching conformational space. J. Am. Chem. Soc. *111*, 4379–4386.

15. Kolossváry, I., and Guida, W.C. (1999). Low-mode conformational search elucidated: Application to C39H80 and flexible docking of 9-deazaguanine inhibitors into PNP. J. Comput. Chem. *20*, 1671–1684.

16. Frisch, M.J., Trucks, G.W., Schlegel, H.B., and Scuseria, G. (2016). Gaussian16 Revision A. 03 (Wallingford, CT: Gaussian Inc.).

17. Becke, A.D. (1993). Density-functional thermochemistry. III. The role of exact exchange. J. Chem. Phys. *98*, 5648–5652.

18. Stephens, P.J., Devlin, F.J., Chabalowski, C.F., and Frisch, M.J. (1994). Ab Initio Calculation of Vibrational Absorption and Circular Dichroism Spectra Using Density Functional Force Fields. J. Phys. Chem. *98*, 11623–11627.

19. Grimme, S., Antony, J., Ehrlich, S., and Krieg, H. (2010). A consistent and accurate ab initio parametrization of density functional dispersion correction (DFT-D) for the 94 elements H-Pu. J. Chem. Phys. *132*, 154104.

20. Becke, A.D., and Johnson, E.R. (2005). A density-functional model of the dispersion interaction. J. Chem. Phys. *123*, 154101.

21. Johnson, E.R., and Becke, A.D. (2006). A post-Hartree-Fock model of intermolecular interactions: Inclusion of higher-order corrections. J. Chem. Phys. *124*, 174104.

22. Grimme, S., Ehrlich, S., and Goerigk, L. (2011). Effect of the damping function in dispersion corrected density functional theory. J. Comput. Chem. *32*, 1456–1465.

23. Hehre, W.J., Ditchfield, R., and Pople, J.A. (1972). Self—Consistent Molecular Orbital Methods. XII. Further Extensions of Gaussian—Type Basis Sets for Use in Molecular Orbital Studies of Organic Molecules. J. Chem. Phys. *56*, 2257–2261.

24. Simón, L., and Goodman, J.M. (2011). How reliable are DFT transition structures? Comparison of GGA, hybrid-meta-GGA and meta-GGA functionals. Org. Biomol. Chem. *9*, 689–700.

25. Krishnan, R., Binkley, J.S., Seeger, R., and Pople, J.A. (1980). Self-consistent molecular orbital methods. XX. A basis set for correlated wave functions. J. Chem. Phys. *72*, 650–654.

26. Mennucci, B., Cammi, R., and Tomasi, J. (1998). Excited states and solvatochromic shifts within a nonequilibrium solvation approach: A new formulation of the integral equation formalism method at the self-consistent field, configuration interaction, and multiconfiguration self-consistent field level. J. Chem. Phys. *109*, 2798–2807.

27. Grimme, S. (2012). Supramolecular Binding Thermodynamics by Dispersion-Corrected Density Functional Theory. Chem. – A Eur. J. *18*, 9955–9964.

28. Funes-Ardoiz, I., Paton, R.S. (2018) Goodvibes: version 2.0.3. doi:10.5281/zenodo.595246.

29. Alecu, I.M., Zheng, J., Zhao, Y., and Truhlar, D.G. (2010). Computational Thermochemistry: Scale Factor Databases and Scale Factors for Vibrational Frequencies Obtained from Electronic Model Chemistries. J. Chem. Theory Comput. *6*, 2872–2887.

30. Grayson, M.N., and Houk, K.N. (2016). Cinchona Urea-Catalyzed Asymmetric Sulfa-Michael Reactions: The Brønsted Acid−Hydrogen Bonding Model. J. Am. Chem. Soc. *138*, 9041–9044.

31. Grayson, M.N. (2017). Mechanism and Origins of Stereoselectivity in the Cinchona Thiourea- and Squaramide-Catalyzed Asymmetric Michael Addition of Nitroalkanes to Enones. J. Org. Chem. *82*, 4396–4401.

32. Rodríguez-Guerra Pedregal, J., Gómez-Orellana, P., and Maréchal, J.-D. (2018). ESIgen: Electronic Supporting Information Generator for Computational Chemistry Publications. J. Chem. Inf. Model. *58*, 561–564.
